# Multi-tissue coexpression networks reveal unexpected subnetworks associated with disease

**DOI:** 10.1186/gb-2009-10-5-r55

**Published:** 2009-05-22

**Authors:** Radu Dobrin, Jun Zhu, Cliona Molony, Carmen Argman, Mark L Parrish, Sonia Carlson, Mark F Allan, Daniel Pomp, Eric E Schadt

**Affiliations:** 1 Rosetta Inpharmatics, LLC, Merck & Co., Inc., Terry Avenue North, Seattle, Washington 98109, USA; 2 Department of Animal Science, University of Nebraska, Lincoln, NE 68508, USA; 3 Department of Nutrition, Cell and Molecular Physiology, Carolina Center for Genome Science, University of North Carolina, Chapel Hill, NC 27599, USA; 4 Current address: Pfizer Animal Health, Animal Genetics Business Unit, East 42nd Street, New York, NY 10017, USA; 5 Current address: Pacific Biosciences, 1505 Adams Dr, Menlo Park, CA 94025, USA

## Abstract

**Background:**

Obesity is a particularly complex disease that at least partially involves genetic and environmental perturbations to gene-networks connecting the hypothalamus and several metabolic tissues, resulting in an energy imbalance at the systems level.

**Results:**

To provide an inter-tissue view of obesity with respect to molecular states that are associated with physiological states, we developed a framework for constructing tissue-to-tissue coexpression networks between genes in the hypothalamus, liver or adipose tissue. These networks have a scale-free architecture and are strikingly independent of gene-gene coexpression networks that are constructed from more standard analyses of single tissues. This is the first systematic effort to study inter-tissue relationships and highlights genes in the hypothalamus that act as information relays in the control of peripheral tissues in obese mice. The subnetworks identified as specific to tissue-to-tissue interactions are enriched in genes that have obesity-relevant biological functions such as circadian rhythm, energy balance, stress response, or immune response.

**Conclusions:**

Tissue-to-tissue networks enable the identification of disease-specific genes that respond to changes induced by different tissues and they also provide unique details regarding candidate genes for obesity that are identified in genome-wide association studies. Identifying such genes from single tissue analyses would be difficult or impossible.

## Abstract 2

Tissue-to-tissue coexpression networks between genes in hypothalamus, liver or adipose tissue enable identification of obesity-specific genes.

## Background

Significant successes identifying susceptibility genes for common human diseases have been obtained from a plethora of genome-wide association studies in a diversity of disease areas, including asthma [1,2], type 1 and 2 diabetes [3,4], obesity [5-8], and cardiovascular disease [9-11]. To inform how variations in DNA can affect disease risk and progression, studies that integrate clinical measures with molecular profiling data like gene expression and single nucleotide polymorphism genotypes have been carried out to elucidate the network of intermediate, molecular phenotypes that define disease states [12,13]. However, in almost all cases the focus has been on single tissue analyses that largely ignore the fact that complex phenotypes manifested in mammalian systems are the result of a complex array of networks operating within and between tissues. Nowhere is this complexity more apparent than in studies of obesity.

Obesity is a particularly complex disease involving genetic and environmental perturbations to networks connecting peripheral tissues such as adipose, muscle, stomach, intestine, liver, and pancreas with the hypothalamus, resulting in an energy imbalance that affects the system as a whole. With more than 30% of adults in the US overweight or obese (body mass index >30) [14], a dramatic increase in the progression of obesity rates in children aged 2 to 19 years [15], and the fact that obesity is a principal cause of type 2 diabetes [16] and results in an increased risk of asthma, certain forms of cancer, cardiovascular disease and stroke, obesity is truly a disease of significant public health concern. Because of this, significant effort has been undertaken to understand the underlying mechanisms critical to the development of obesity. While many of these efforts have shown great promise, they are also revealing a more complex picture of obesity than was previously thought, consisting of highly integrative, interactive and multi-tissue physiological control.

Energy storage is a complex event in any organism. In higher organisms like mammals, multiple tissues interact to ensure adequate energy storage. A key to understanding obesity is deciphering the paths along which molecules move as well as the signals that control these processes. While white adipose tissue is the primary organ for longer-term storage of energy in the form of triglycerides, it is also a very dynamic compartment within the body. In fact, white adipose tissue can be considered among the most active endocrine organs, secreting hormones like leptin, adiponectin, tumor necrosis factor-α, interleukin-6, estradiol, resistin, angiotensin, and plasminogen activator inhibitor-1. The active state of this organ is evidence enough that it does not act in isolation. In fact, it is already well established that the brain receives signals through small molecules like leptin and insulin circulating in the blood, and through sympathetic and parasympathetic systems. The central nervous system has proven to be a primary player in maintaining energy homeostasis, where it is believed that the brain acts as an 'energy-on-request' system, with a hierarchical organization in which the hypothalamus plays a central role [17,18]. Using the neuronal tracer cholera toxin B and the retrograde neuronal tracer pseudorabies virus, Kreier *et al*. [19] showed that the autonomic nervous system exhibited a distinct organization through sympathetic and parasympathetic innervations. In addition, inactivation of the insulin receptor in brain has been shown to induce hyperphagia and obesity [20]. Further, leptin plays a fundamental role in regulating food intake and long-term energy homeostasis [21]. The inhibition of hypothalamic arcuate nucleus neurons that co-express the agouti-related protein (*Agrp*) and neuropeptide Y (*Npy*) by activating the phosphatidylinositol 3-kinase pathway, is achieved in a manner that is independent of the STAT3 pathway [22]. Alternatively, leptin activates the JAK/STAT3 pathway in pro-pomelacortin neurons [23].

The regulatory processes that ensure intra-tissue coherence (for example, transcription factors) may differ from those that drive biological coherence between tissues. We hypothesize that if genes have correlated expression patterns across tissues, they are more likely to react to the information exchanged between them rather than to be driven by regulatory events specific to each tissue. Therefore, in a disease like obesity, where the hypothalamus receives and integrates signals from peripheral tissues (for example, adipose and liver) and actively sends signals to manage energy balance, tissue-to-tissue coexpression (TTC) networks may highlight communication between tissues and elucidate genes or sets of genes active in one tissue that are able to induce gene activity changes in other tissues.

## Results

Given the complex array of processes driving obesity in multiple organs, we profiled gene expression in adipose, liver and hypothalamus from F_2 _progeny from a cross between the outbred M16 (selectively bred for rapid weight gain) and ICR (control) mouse strains (referred to here as the MXI cross) [24,25]. After constructing coexpression networks for each tissue independently, we identified subnetworks (modules) of highly interconnected sets of genes enriched for common functional categories in the Gene Ontology (GO). Tissue-specific coexpression networks, especially when integrated with DNA variation and clinical data, have led to a number of important discoveries and have for some time now represented the state of the art in elucidating molecular networks underlying complex phenotypes [26-29]. Topologically, coexpression networks are part of a larger class of scale-free networks [30] that include the majority of known biological networks such as metabolic, transcriptional regulatory and protein-protein interactions [13], as well as the class of uncharacterized, TTC networks. Therefore, we constructed TTC networks from adipose, liver and hypothalamus profiles. A comprehensive analysis of these networks revealed a scale-free topology, with single gene expression traits in one tissue correlating with larger numbers of expression traits in other tissues (that is, hub nodes operating across tissues), suggesting that information is passed between tissues in an asymmetric fashion. The asymmetric information relay is observed to be much more common for hypothalamus than for either adipose or liver, suggesting that hypothalamus is the controlling tissue. We demonstrate how these TTC networks complement our knowledge stemming from single tissue analyses, revealing a new dimension in expression networks: cross-tissue specific subnetworks.

We generated high-quality TTC networks from each possible pair of tissues by identifying significantly correlated expression traits from matched adipose, hypothalamus and liver samples collected from F_2 _mice, resulting in three cross-tissue specific networks that were constructed using 308 mice for adipose-hypothalamus (AH; Table T7 in Additional data file 1), 298 for hypothalamus-liver (HL; Table T8 in Additional data file 1) and 302 for adipose-liver (AL; Table T9 in Additional data file 1). Nodes in the TTC networks represent gene expression traits from each tissue in the TTC network; thus, by adipose gene we mean expression levels corresponding to the gene in adipose tissue, and similarly for hypothalamus and liver genes. Two nodes in a TTC network are connected if the gene expression traits are significantly correlated across the two tissues with respect to a predefined significance threshold. Therefore, unlike classical tissue-specific coexpression networks, TTC networks are bipartite graphs with respect to the corresponding tissues (there are no links between genes in the same tissue). To test for correlation between gene expression traits, we used the non-parametric, rank-based Spearman correlation, given this measure makes fewer underlying assumptions on the distribution of the correlation under the null hypothesis and is more robust to outliers compared to parametric correlation measures. The appropriate significance level was determined by assessing the network-specific false discovery rate (FDR) for these correlations where we estimated empirically the null distribution using permutation methods (see Materials and methods). For all the TTC networks, we used a fixed *P*-value threshold of 10^-8^, which corresponds to an FDR <0.1% in all three networks.

**Figure 1:**
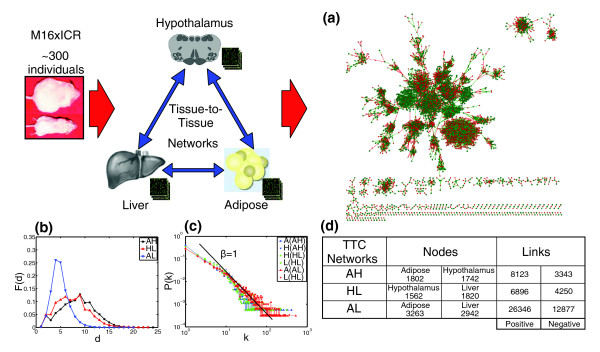
Tissue-to-tissue networks summary. **(a) **Display of the adipose-hypothalamus (AH) TTC network at a *P*-value threshold of 10^-8^. Red and green edges denote negative and positive correlations, respectively. Adipose nodes in the network are marked as green circles while hypothalamus nodes are marked as red diamonds. The networks display a high degree of modularity, as can be seen visually. The largest connected component of the network contains roughly 70% of all of the nodes in the network. **(b) **The all-pairs shortest path distributions F(d) (d is the shortest path between a pair of nodes in the network) for the TTC networks: AH in black, hypothalamus-liver (HL) in red, and adipose-liver (AL) in blue. The diameter of the networks (d_AH _= 8,728, d_HL _= 7.420, d_AL _= 4.926) are dependent on whether hypothalamus is part of the network or not. **(c) **Connectivity distributions P(k) (connectivity k is the number of edges connecting a gene) for adipose, hypothalamus and liver nodes in each of the three TTC networks exhibit scale-free behavior P(k)~k^-γ ^with *γ *= 1. **(d) **TTC networks summary. All the values reported are for TTC networks generated at a *P*-value threshold of 10^-8^. The number of positive correlations in the TTC networks is twice that of the negative correlations.

The TTC networks for the three tissue pairs are very similar with respect to their global topological properties (Figure 1). The connectivity distributions depicted in Figure 1c follow a power-law distribution for genes in either tissue, which is indicative of a scale-free network in which a small proportion of genes serve as hub nodes (that is, a gene connected to a very large number of other genes). The scale-free nature of these networks increases the likelihood that correlations between tissues are highly asymmetric in this population. For example, in the AH network the top 1% connected genes in either tissue are unique with no overlap. At the same time, from the 143 genes that are symmetric (that is, the number of correlations for these genes in both tissues is the same), the maximum connectivity is only 21, with 79 genes in this set singly connected. The most connected hypothalamus gene in this network is *Aqp5*, which is linked to 169 adipose genes, while the adipose gene *Aqp5 *is only linked to 2 genes in hypothalamus. Similar examples can be found in the other two TTC networks. Asymmetric connectivity is an indicator of information exchange between tissues. In the above example, *Aqp5 *in the hypothalamus is either 'sending' information to the 169 adipose genes (that is, regulating expression of the 169 adipose genes) or 'integrating' (responding to) their signals. Only a genetically engineered mouse model in which *Aqp5 *is specifically perturbed in the hypothalamus could provide the detailed information needed in order to determine the direction of the information flow between tissues and rule out alternative explanations, such as the asymmetric connectivity obtaining via a 'hidden' third factor. It is plausible that the exchange of information between tissues is mediated through other clinical traits, such as plasma insulin, glucose, hormone levels, ion concentrations, metabolite concentrations and so on. If we were able to collect all possible 'intermediate' traits, then we could apply our standard causality procedure [29] to test whether gene expression traits in one tissue are supported as causal for such clinical traits, and then construct a new causality model that will test whether these gene expression traits in a different tissue were supported as reactive to the clinical trait; or whether gene expression traits in both tissues were supported as reactive to the clinical traits. In such a case we could begin to differentiate whether a given tissue was supported as causal for, or was associated with, gene expression changes in a different tissue.

While the topological properties of the TTC networks are largely the same, the diameters of these networks, defined as the mean shortest distance between nodes in the network, are significantly different. The AH and HL networks have similar diameters almost twice as large as the AL network diameter. Similarly, the distributions of distances between genes (Figure 1b) are similar for the AH and HL networks, with the AL network exhibiting a much narrower distribution. If we consider the hypothalamus as a primary controlling organ in the body, the TTC networks confirms that the network diameter is representative of the relationship between the tissues within the organism, with the network between metabolic tissues (AL) being more compact (thus having a small diameter) than networks that involve a controlling organ (HL and AH).

**Figure 2:**
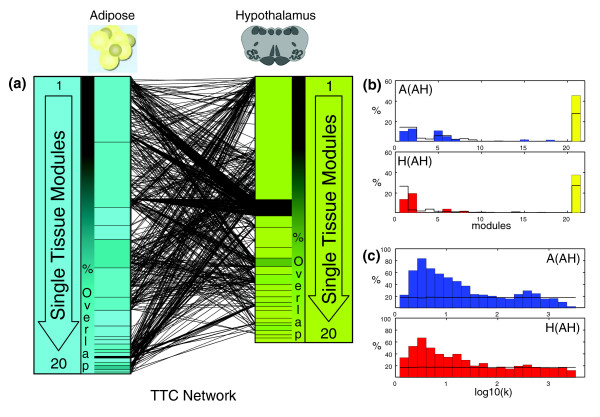
Single tissue projections to the adipose-hypothalamus TTC network. **(a) **Adipose and hypothalamus modules (color shaded rectangles) derived from independent analysis of each tissue's GGC network and their overlap with the AH network. Each tissue-specific module is shaded based on the percentage overlap relative to the module size (the shading scale shown next to the modules). The black lines between modules represent edges identified in the AH network. **(b) **Percentage overlap of GGC modules relative to the TTC network: top adipose modules and bottom hypothalamus modules. The black stairs show the percentage overlap that is observed by chance. The yellow bar represents genes that were not placed in single tissue modules and contains approximately 40% of all genes found in the TTC network. **(c) **Percentage overlaps between the subset of adipose and hypothalamus genes from the AH network and the adipose genes (top panel) and hypothalamus genes (bottom panel). Each bar represents the percentage of genes with connectivity k in the corresponding single tissue (the x-axis) that are part of the TTC network. We can see that expression traits that do not correlate with many other traits in a single tissue are more likely to be found in the TTC network.

To understand whether TTC networks provide additional insights into the system under study, we examined whether these networks overlapped significantly with tissue-specific coexpression networks. Similar to TTC networks, we generated gene-gene coexpression (GGC) networks for each tissue using the Spearman correlation measure. The 9,967 genes (Table T3 in Additional data file 1) included in the construction of the tissue-specific coexpression networks were those genes that were either present in at least one of the TTC networks or that were significantly differentially regulated (compared to the reference pool) in at least 10% of the samples. Because expression traits identified with synergies between the tissues were not necessarily the most correlated traits in the single tissue analyses, the overlap between the expression traits in the TTC networks and those in the tissue-specific coexpression modules is not 100%. Interestingly, about 40% of expression traits in the TTC networks fell outside of the tissue-specific network modules defined by each tissue, with the exception of a few highly overlapping modules in each tissue (Figure 2). This finding was unexpected and reveals a new facet of coexpression networks that complements single tissue analyses.

To assess whether this result was caused by our choice of *P*-value thresholds, we examined the connectivity distributions for each tissue at the same *P*-value threshold used to construct the TTC networks (10^-8^), while simultaneously generating the connectivity distribution for all genes in the TTC network originating from a given tissue. From the connectivity distribution plots in Figures 2c we note a clear trend for nodes in the TTC networks having reduced connectivity in the hypothalamus and adipose coexpression networks, without any apparent peak at any of the connectivity values. This demonstrates that expression traits in the TTC network are enriched for genes that could not be placed into any of the tissue-specific coexpression network modules. That is, expression traits in the TTC network demonstrate a high degree of correlation with expression traits between tissues, but not within tissues. Therefore, via the TTC networks, we have identified entire classes of genes that are systematically ignored in single tissue analyses because they form, on average, no meaningful connections with other genes within a given tissue, but instead are enriched for genes in one tissue that are strongly connected with genes in a different tissue.

**Figure 3:**
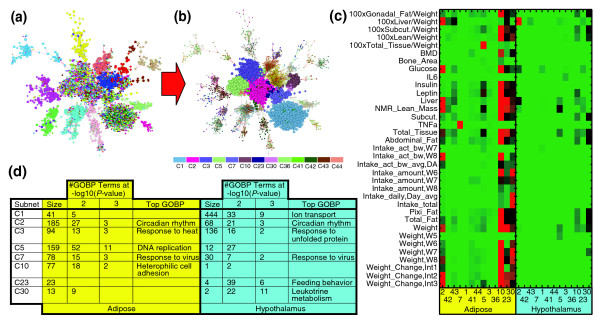
Adipose-hypothalamus network partitioning and analysis. **(a) **Network highlight based on chromosomal location and cis expression quantitative trait loci (eQTL) status. Each node is colored according to chromosomal location with different colors for different chromosomes. Large nodes correspond to genes that have cis-eQTL. Two types of subnetworks are observed in the network: type 1 subnetworks that contain genes located on the same chromosome that also have cis-eQTLs; and type 2 subnetworks with genes that are neither located on the same chromosome nor have cis-eQTLs. **(b) **Highlighted are all the type 2 subnetworks, as identified by the partitioning algorithm. **(c) ***P*-value heatmap for the association between clinical traits and gene expression traits for the type 2 subnetworks. The heatmap scale ranges from 1 (green) to 10^-10 ^(red). All *P*-values smaller than 10^-10 ^are set to 10^-10^. For a detailed description of clinical traits see Materials and methods. **(d) **Gene Ontology enrichments for type 2 subnetworks of size greater than 10. To validate the robustness of the overlap, we recorded the number of GO biological process (GOBP) terms when the FDR corrected *P*-values resulting from the Fischer's exact test, -log10(*P*-value), exceeded 2 and 3. The 'Top GOBP' column lists the GOBP terms that have the lowest Fischer's exact test *P*-value.

To understand more fully how TTC networks differ from tissue-specific coexpression networks, we identified coherent subnetworks (commonly referred to as modules or clusters) that reflect different biological functions associated with these parts of the network. The algorithm we employed partitions the network by removing edges with high betweenness scores as previously described [31], segregating the TTC networks into robust subnetworks (details in Materials and methods; Figure S11 in Additional data file 1). Several other methods [32,33] were tested and led to only minor differences in the subnetworks identified, reflecting the strong modular structure that is apparent by visual inspection of the TTC networks (Figure 3). In the AH network we identified 45 subnetworks (Table T10 in Additional data file 1) labeled based on their size, with C1 being the largest subnetwork, containing 485 nodes, and C45 being the smallest with only 7 nodes.

In order to see whether the correlations between genes across tissues could be driven by common genetic effects, we examined the extent to which genes in a given subnetwork were clustered in common chromosomal regions. Using *P*-values obtained from the Fischer's exact test (FET) [34] to estimate the degree of overlaps between the TTC subnetworks and genes in a given chromosomal region, we found two types of subnetworks. Type 1 subnetworks were composed of genes enriched in common chromosomal regions, while type 2 subnetworks exhibited no apparent enrichment. Figure 3a highlights this segregation in the AH network.

To assess whether the type 1 subnetworks were the result of common genetic control, we carried out genome-wide linkage analysis on each expression trait to map expression quantitative trait loci (eQTL). For a given expression trait we considered an eQTL proximal if the eQTL position was coincident with the location of the corresponding structural gene (referred to here as a cis-eQTL). Otherwise, we considered the eQTL distal (referred to here as a trans-eQTL). Interestingly, nearly all of the genes in the type 1 subnetworks gave rise to cis-eQTL (Figure S12 in Additional data file 1). The magnitude of the effects and proximity of the cis-eQTL in a given type 1 subnetwork suggest that the chromosome-specific correlation patterns are artifacts of gene expression traits controlled by closely linked genetic loci, as we have previously shown [29,35]. At the very least, whether the correlations among gene expression traits in type 1 subnetworks can be attributed to common upstream regulators is confounded by the correlation structure induced by closely linked cis-eQTL. On the other hand, type 2 subnetworks in the TTC networks contained only genes that do not have a detectable cis-eQTL, indicating these genes were more likely to be correlated because of biologically relevant covariation in their expression levels. Therefore, for all further analyses we restricted attention to those TTC subnetworks that were not enriched for genes with cis-eQTL in common chromosomal regions (that is, FET *P *> 0.05 for the overlap between genes with cis-eQTL and genes in a given type 1 subnetwork), as depicted in Figure 3b for the AH network.

One way to establish the biological coherence of a given gene subnetwork is to test whether genes in a given subnetwork are enriched for genes involved in known biological pathways or genes associated with clinical traits [12,28]. Therefore, we tested whether type 2 subnetworks in the TTC networks were enriched for GO biological process (GOBP) terms containing no more than 1,000 genes and for genes correlated with any of the 64 obesity-associated traits scored in the MXI cross. When calculating enrichments for the TTC subnetworks, it is important to remember that unlike tissue-specific coexpression networks, the TTC subnetworks contain two species of nodes corresponding to each tissue.

For the AH network we found several subnetworks enriched in GOBP categories for either adipose or hypothalamus genes. Figure 3d highlights the GOBP terms that exceed the *P*-value threshold in the AH network. We observed the same pattern of enrichment for genes associated with the obesity traits (Figure 3c). The clinical trait-gene correlations were calculated using the Spearman correlation measure. Genes identified as correlated to a specific obesity trait had corresponding *P*-values significant at an FDR level of 5% using Benjamini-Hochberg correction [36]. Regardless of the FDR level there were far fewer hypothalamus genes whose expression was correlated with obesity traits compared to adipose genes. When looking globally at all expression profiles at a 10% Benjamini-Hochberg FDR level we found liver weight to be the trait most correlated with hypothalamic gene expression, with 34 hypothalamus genes associated with this trait. On the other hand, epididymal (males) or perimetrial (females) fat mass was the trait most significantly associated with adipose mRNA levels, with 977 genes significantly correlated with these traits. We thus expect that subnetwork enrichments for the hypothalamus genes associated with clinical traits will be harder to detect than for adipose genes associated with clinical traits.

**Figure 4:**
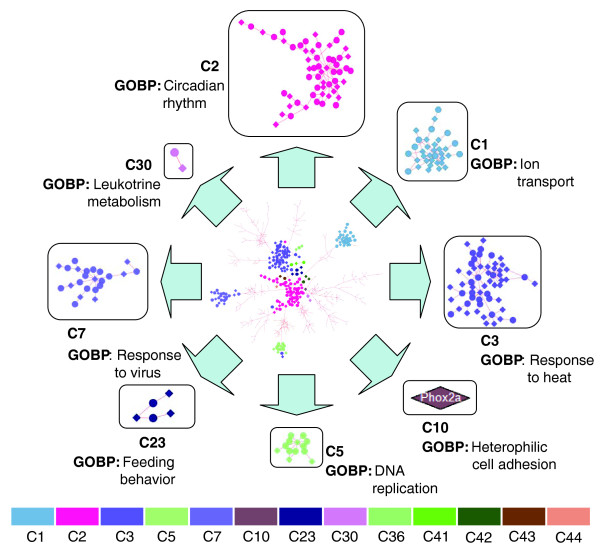
Adipose-hypothalamus network backbone. We define the network backbone as the bonds most visited by the all-pair shortest paths algorithm on the TTC network. In order to generate a robust backbone, we assigned *P*-values of Spearman correlations as bond weights. The subnetworks selected for further analysis are represented by a small number of representative genes on the backbone. Perturbing these genes most likely triggers responses in the complementary tissue.

Networks offer a plethora of information that is often hard to interpret given the density of the different subnetwork components. To extract the most reliable information from the TTC networks, we defined the network backbone (see Materials and methods) to be composed of a limited number of highly correlated genes. As seen in Figure 4 for the AH network, the backbone contains only 613 nodes and 725 edges representing 21.78% and 6.32% of the nodes and edges, respectively, from the original network (Table T13 in Additional data file 1). Each subnetwork contributes to the backbone with its most representative genes, which helps to identify the core relationships from the network (Figure 4).

## Discussion

Combining the TTC subnetwork enrichment analysis with information gathered from the network backbone, the picture emerging for obesity is that of a complex network composed of genes that have been intensively studied as well as genes that have never before been considered as molecular components of biologically relevant pathways. Between adipose and hypothalamus we find several TTC subnetworks that are associated with precise biological functions. As highlighted by the AH network backbone in Figure 4, the C2 subnetwork is at the center of the AH network. This subnetwork is enriched for genes associated with obesity and for genes involved in circadian rhythm. Some genes in this subnetwork, such as *Arntl*, *Dbp*, *Per1*, and *Per2*, are known to associate with obesity traits, while other genes, such as *Map3k6 *and *Tsc22d3*, represent novel factors.

In addition to the clock regulators mentioned above, the C2 subnetwork includes three other genes that are also part of the backbone and that are essential for cellular response to starvation: *Sgk*, *Pdk4 *and *Acot1*. Subnetwork C3 contains hypothalamus genes that are linked to adipose heat shock genes *Hsp110 *and *Dnajb1*. Another important hypothalamus gene from C3 that correlates with adipose *Hsp110 *is *Fem1b*, a gene required for normal glucose homeostasis and pancreatic islet cell function [37]. C3 also contains several highly linked genes like *Dnajb1 *and *Chordc1 *that are known to be down-regulated in the sleep phase [38]. Both C2 and C3 appear to be separated based on circadian patterns, with C2 containing genes up-regulated in mice during sleep and C3 containing several heat shock protein genes that are up-regulated while mice are awake. These subnetworks are very close to each other, with C2 appearing to play a more central role (Figure 4). Two other highly asymmetric subnetworks emerge from the AH analysis: C5, containing the hypothalamus water channel gene *Aquaporin 5 *(*Aqp5*), the most highly connected hypothalamus gene, and C10, containing the hypothalamus gene *Phox2a*, which correlates with 84 adipose genes, the third most highly connected hypothalamus gene. *Aqp5 *is a gene that belongs to the AQP family of major intrinsic membrane proteins, which function as molecular water channels to allow water to flow rapidly across plasma membranes in the direction of osmotic gradients. *Phox2a *is a paired-like homeodomain transcription factor that participates in specifying the autonomic nervous system by controlling the differentiation of sympatho-adrenal precursor cells [39,40]. The AH subnetwork C23 is enriched for adult feeding behavior and energy balance and contains well known genes such as those encoding agouti related protein (*Agrp*) and neuropeptide Y (*Npy*), and also *Ptx3*, a gene recently reported to associate with obesity that is involved in immune system response and modification in feeding behavior [41,42]. C7 is enriched for immune response signaling through the interferon family of genes. The most highly connected nodes in C7 are hypothalamus genes *Ifi44*, *Irf7*, *Tgtp*, *Sp100 *and *Trim30*.

**Figure 5:**
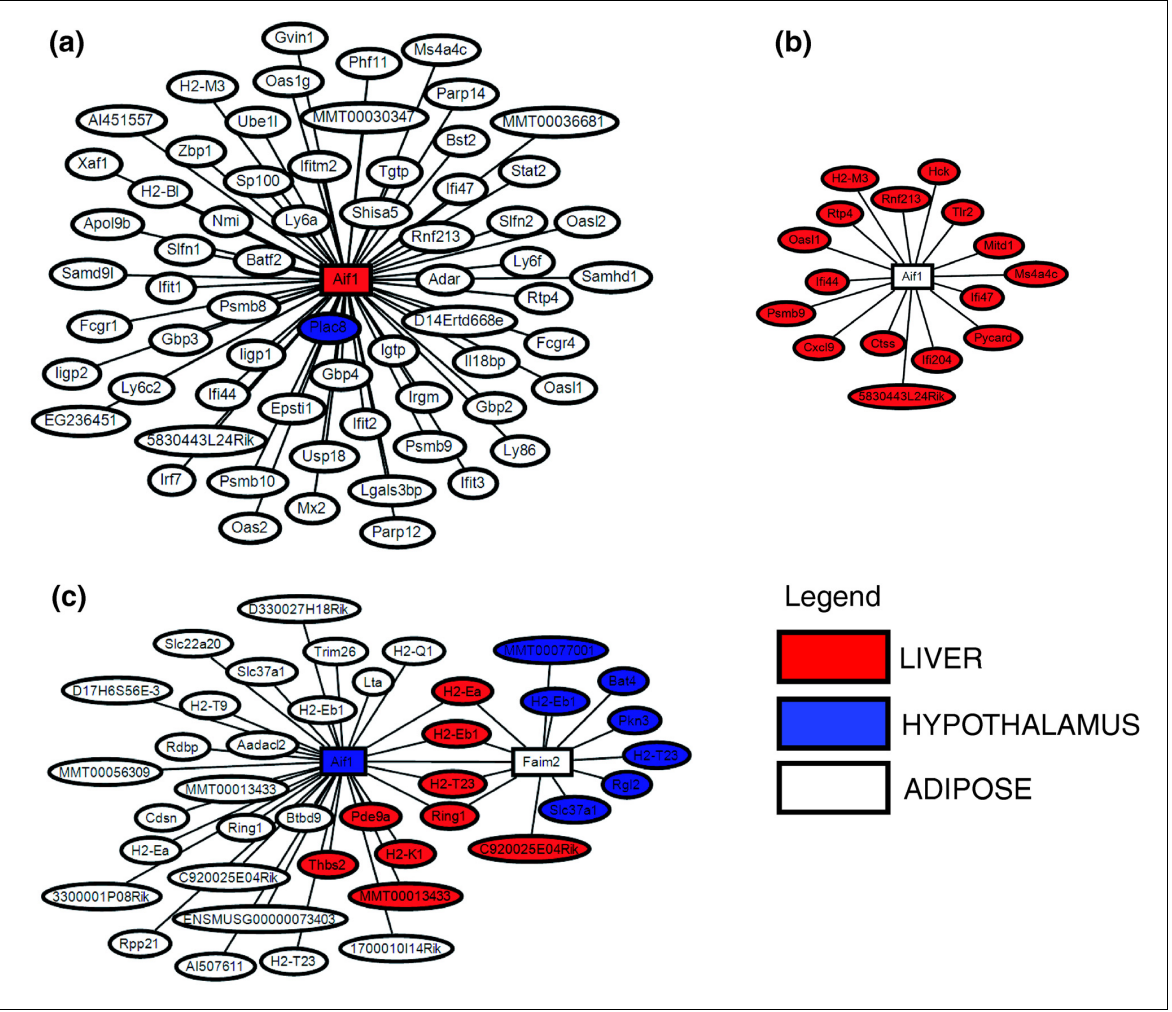
Genome-wide association obesity gene *Aif1 *in TTC networks. Detailed view of TTC network connections for *Aif1 *identified in genome-wide association studies as associated with obesity. Nodes are colored based on the tissue of origin for the mRNA profile, such that white, blue and red are gene expressions in adipose, liver and hypothalamus, respectively. Rectangle nodes denote genome-wide association candidate genes for obesity. **(a) **Liver *Aif1 *and its connection to hypothalamus and adipose tissue. **(b **Adipose *Aif1 *and its connections to liver. **(c) **Hypothalamus *Aif1 *and its connections to liver and adipose.

Two recent papers describing genome-wide association studies [43,44] found a number of novel loci associated with obesity (weight or body mass index) in human populations, raising the total number of loci validated to influence obesity in humans to 24. While genome-wide association studies are incredibly powerful for identifying the ultimate causal changes in DNA that associate with diseases like obesity, they often do not directly indicate the gene or genes that are affected by the DNA change, and they do not provide a context within which to interpret action of the causal genes and how they lead to variations in the disease of interest. Therefore, the next challenge is to understand the mechanisms through which these candidate genes act on energy storage and balance. The suggestion from these previous studies is that neural development plays an important role in obesity. We used the TTC networks described above to elucidate possible mechanisms of how these genes affect obesity phenotypes. When compared with clinical QTLs of fat and weight, only 3 of the 24 published human genes (*Aif1, Bat2 *and *Ncr3 *ortholog) are within 5 cM of clinical QTL peaks. *Bat2 *and *Ncr3 *ortholog do not have cis-eQTL in any tissues. *Aif1 *(allograft inflammatory factor 1), which has a cis-eQTL in hypothalamus, was reported to be associated with weight [43]; itcontributes to anti-inflammatory response to vessel wall trauma. When looking at single tissue networks, we find *Aif1 *in adipose module 5 and liver module 6, both of which are enriched for GOBP inflammatory response. Although *Aif1 *has a cis-eQTL in hypothalamus, it does not belong to any module in the hypothalamus network. When we looked at the TTC networks we observed that *Aif1 *was a hub node in all three, as shown in Figure 5. In the AL network, liver *Aif1 *is linked to 63 adipose genes (Figure 5a), while adipose *Aif1 *is linked to 16 liver genes (Figure 5b). Both gene sets are enriched for interferon-mediated immune response genes. Remarkably, we found *Aif1 *in the HL and AH networks, where hypothalamus *Aif1 *is linked to immune response genes like *H2-Eb1 *and *H2-Ea *(Figure 5c) in both adipose and liver. Hypothalamus *Aif1 *is also linked to *Lta *and *Faim2*, genes that regulate apoptosis and also reported as associated with obesity [43]. The TTC network findings suggest that hypothalamus *Aif1 *is associated with both obesity and diabetes.

## Conclusions

By constructing cross-tissue networks we provided a global view of the gene expression patterns across hypothalamus, liver and adipose tissue in mice confronted with an abnormal state such as obesity. The TTC networks constructed between tissue pairs reflect subnetworks that are not represented in tissue-specific networks, highlighting the importance of considering interactions among molecular states in entire systems to fully characterize complex traits like obesity. The subnetworks we identified as specific to the TTC networks are composed of genes already known to associate with obesity as well as new molecular components that are not well described in the current literature. The asymmetry reflected in the TTC networks provides direct support that these networks represent cross-tissue communication. A central characteristic of all the TTC networks is that the circadian subnetwork is at the center of the TTC networks and connects to all other subnetworks in the network (see Figure 4 for the AH network). It is well established that disregulation of several genes in the circadian subnetwork lead to obesity by disrupting energy balance and glucose homeostasis [45-47]. In a recent paper Lamia *et al*. [48] used a liver-specific *Bmal1*-/- mouse model to show that deletion of the circadian gene *Bmal1 *(*Arntl*) in a peripheral tissue such as liver leads to systemic glucose homeostasis disruptions, although they had normal body fat content compared to the controls. This finding is supported by the TTC networks where *Arntl *and several other circadian genes are central components and also emphasizes that key regulators in each tissue are required to work in synchrony. The fact that liver *Arntl *did not have a global effect on body weight is reflected by the structure of the AL network where adipose *Arntl *has 521 connections, outranking liver *Arntl *with only 83 connections.

Only by looking at the system as a whole can we begin to isolate key molecular networks that are associated with the disease and are not reflected in single tissue networks or in studies of *in vitro *cell systems. TTC networks identify genes related to communication between tissues and provide a first step toward understanding complex diseases like obesity in terms of the hierarchy of interacting molecular networks that define physiological states in mammalian systems.

## Materials and methods

### Resource population

Selection leading to the present M16 line was originally conducted in two replicate lines (M16-1 and M16-2 [49]). The two replicates were subsequently crossed to form the present M16 line, which was maintained (along with the control line ICR) by within-family random selection for approximately 100 generations prior to establishment of the QTL mapping population used in this study.

A large F_2 _population (*n *= 1,181) was established by intercrossing the M16 and ICR lines, whose phenotypes were recently described [24]. Twelve F_1 _families resulted from six pair matings of M16 males × ICR females and six pair matings of the reciprocal cross. A total of 55 F_1 _dams were mated to 11 F_1 _sires in sets of five F_1 _full sisters mated to the same F_1 _sire. These same specific matings were repeated in three consecutive replicates. Thus, the F_2 _population consisted of approximately 55 full-sib families of up to 24 individuals each and 11 three-quarter-sib families of up to 120 individuals each. All litters were standardized at birth to eight pups, with approximately equal representation of males and females, and were weaned at 3 weeks of age with mice provided *ad libitum *access to water and pellet feed (Teklad 8604 rodent chow). Mice were then caged individually from 4 to 8 weeks of age. The University of Nebraska Institutional Animal Care and Use Committee approved all procedures and protocols.

### Phenotypic data collection

Body weights were measured at weekly intervals from 3 to 8 weeks of age. From 4 to 8 weeks of age, feed intake was recorded for all F_2 _mice at weekly intervals. At 8 weeks of age, following a period of 1.5 h where feed was removed but access to water remained, mice were decapitated after brief exposure to CO_2_. Blood was collected from the trunk, and blood glucose was measured using the SureStep Blood Glucose Monitoring System (LifeScan Canada, Burnaby, British Columbia, Canada). The subcranial region was scanned in a consistent, dorsal position using a dual-energy X-ray absorption (DEXA) densitometer (PIXImus, Lunar, Madison, WI, USA). The DEXA measurements estimated two primary body composition characters in each mouse: total subcranial tissue mass (TTM, in grams) and total subcranial fat (FAT, in grams). After scanning, each carcass was dissected and weights of the liver, right hind limb subcutaneous adipose depot, and right epididymal (males) or perimetrial (females) adipose depot were recorded. These and other tissues, including hypothalamus, pituitary, gastrocnemius muscle, heart, spleen, kidney (with adrenal) and tails, were collected and snap frozen in liquid nitrogen.

### Analysis of plasma proteins

All F_2 _males were measured for plasma levels of insulin, leptin, tumor necrosis factor-α, and interleukin 6 using a single multiplex reaction (run in duplicate) based on microsphere bead technology (Linco, St. Louis, MO, USA) using a Luminex^100 ^system (Luminex, Austin, TX, USA). Raw data were processed using Masterplex QT (Miraibio, Alameda, CA, USA); plate-to-plate variation was normalized using a standard sample on all plates.

### RNA sample preparation and hybridization

Global expression analysis was determined using the 23,574-feature mouse Rosetta/Merck Mouse TOE 75k Array 1 (Gene Expression Omnibus (GEO) Platform: GPL 3562; Agilent Technology, Palo Alto, CA, USA). Total RNA from hypothalamus samples (*n *= 308) where isolated and hybridized using the protocol described in Brandish *et al*. [50]. This method utilizes a Moloney murine leukemia virus reverse transcriptase-mediated reverse transcription and double-stranded cDNA production, followed by T7 RNA polymerase transcription. The resultant RNA is further amplified with a second round of reverse transcription and *in vitro *transcription incorporating amino-allyl UTP. Total RNA from liver samples (*n *= 302) and adipose samples (*n *= 308) was isolated from frozen tissue. For liver and adipose, 5 μg of total RNA was used for each amplification reaction. The method used a custom automated version of the Reverse Transcription/In Vitro Transcription (RT/IVT) method referenced in Hughes *et al*. [51]. Labeled cRNA from each F_2 _animal was hybridized against a pool of labeled cRNAs constructed from equal aliquots of RNA from 160 F_2 _animals for each of the three tissues in the cross that was balanced for sex and litter. Samples failing amplification were excluded from the pools. Sample hybridization and array scanning for all three tissues were performed as described [51]. Microarrays were scanned, and individual feature intensities were pre-processed in a series of steps, consisting of background subtraction, normalization to mean intensities of the Cy3 and Cy5 channels, and detrending to fit a linear relationship between channels [52]. Normalized intensities were used to derive expression ratios using the Rosetta error model [52,53]. Expression ratios obtained in this study are available for query or download from the GEO website at the National Center for Biotechnology Information [54] as the following series: [GEO:GSE13745] (hypothalamus), [GEO:GSE13746] (adipose) and [GEO:GSE13752] (liver).

### Single tissue co-expression network construction and module detection

#### Constructing coexpression networks

Coexpression networks were constructed by defining gene-gene relations based on a similarity measure. For gene expression data measured in a large number of individuals the most natural similarity measure between two expression traits is the correlation coefficient. The Spearman correlation measure was used in this case. Only genes identified in the TTC networks together with genes that were differentially expressed (relative to the reference pool) in at least 5% of the samples in each of the tissues were used for creating the tissue-specific co-expression networks. The *P*-value threshold was set to 10^-8^, identical to the threshold used for the TTC networks.

#### Identifying gene modules

GGC networks are highly connected. The clustering results highlighted in Figure 3 and Supplementary Figure S3 in Additional data file 1 reflect that there are modules arranged hierarchically within these networks. Ravasz *et al*. [55] used manually selected height cutoff to separate tree branches after hierarchical clustering, in contrast to Lee *et al*. [56], who formed maximally coherent gene modules with respect to GO functional categories. We employed a measure we previously developed and validated [57] that is similar to that used by Lee *et al*. [56], but without the dependence on the GO functional annotations. Briefly, a gene module in the co-expression network was defined as a maximum set of inter-connected genes. We defined the coherence of a gene module as:



where *GP*_*obs *_is the number of gene pairs that are connected, and *GP*_*tot *_is the total number of possible gene pairs in the module. The efficiency of a gene module was defined as:



where *G*_mod _is the number of genes in the module, and *G*_*net *_is the number of genes in the network. Given these definitions, the process employed to iteratively construct gene modules consisted of the following steps: step 1, order genes in the gene-gene connectivity matrix according to an agglomerative hierarchical clustering algorithm as previously described [51]; step 2, calculate the efficiency *e*_*i*, *j *_for every possible module, including genes from *i *to *j *as given in the ordered connectivity matrix, where *j *≥ *i *+ 9 (that is, minimum module size is 10), using a dynamic programming algorithm; step 3, determine the maximum *e*_*i*, *j*_:



step 4, go to step 3 until no additional modules can be found. The program for identifying the network modules was implemented in MATLAB 7.0.1 (MathWorks, Natick, Massachusetts, USA).

### Tissue-to-tissue coexpression network construction and subnetwork partitioning

#### Network construction

We constructed the TTC networks from gene expression data of individuals that had both tissues relevant to the network profiled. As a consequence, the number of samples varied from network to network. For the AH TTC network we had 308 samples, for HL 298, and 302 samples for the AL TTC network. The correlation between two expression traits from different tissues was computed using the Spearman correlation measure. A *P*-value threshold of 10^-8 ^corresponding to an FDR <0.1% was used for each of the TTC networks. The FDR was assessed using permutations.

#### Identifying tissue-to-tissue coexpression subnetworks

The method chosen to partition the network is based on the betweenness centrality measure as previously defined [31]. On the TTC network, the algorithm finds the edge most used by shortest paths between all possible pairs of nodes in the networks, removes it and repeats these two steps until no edge is left in the network. In order to increase the running time of the algorithm at each step, we removed closed subnetworks, defined as a subnetwork for which the maximum distance between any pair of nodes is 2, or if the number of nodes in a subnetwork is 2. All other subnetworks we call viable subnetworks. To find the best network partition, we selected the partition occurring at the point where the total number of viable subnetworks is the maximum. After this maximum is reached we observe a decrease in the number of viable clusters as the algorithm removes edges between nodes.

### Backbone detection for tissue-to-tissue coexpression networks

To construct the network backbone, we computed the weighted edge betweenness [31] for all edges in the network. An edge connected weight is equal to the *P*-value of the correlation it denotes. Thus, a shortest path is defined as the path between two genes along which the sum of edge weights is minimum. An edge is part of the backbone if its scaled edge-betweenness (defined as the total number of shortest paths that contain that edge divided to N - 1, where N is the total number of nodes in the network) is larger that 1. By weighting the edges that connect genes in this manner we guaranteed that the shortest paths would include the most highly correlated genes in the network.

## Abbreviations

AH: adipose-hypothalamus; AL: adipose-liver; eQTL: expression quantitative trait loci; FDR: false discovery rate; FET: Fischer's exact test; GEO: Gene Expression Omnibus; GGC: gene-gene coexpression; GO: Gene Ontology; GOBP: Gene Ontology biological process; HL: hypothalamus-liver; TTC: tissue-to-tissue coexpression.

## Additional data files

The following additional data are available with the online version of this paper: supporting text, Figures S1 to S13 and Tables T1 to T18 (Additional data file 1).

## Authors' contributions

RD designed the study, developed the method, analyzed the results and drafted the manuscript. JZ designed the study, assisted in developing the method and interpreting the results, and helped draft the manuscript. CM carried out the genetics studies and eQTL detection. CA assisted with the biological interpretation. MFA helped design the mouse cross and led the team that collected all of the mouse phenotypic data under the guidance of DP. MLP and SC supervised the sample extraction and microarray hybridization. DP provided the animals, and helped in interpreting the results and drafting the manuscript. EES designed the study, participated in data analysis and drafting the manuscript. All authors read and approved the final version of the manuscript.

## Supplementary Material

Additional data file 1 Figure S1 shows the connectivity distribution P(k) for GGC networks. Figure S2 shows FDR curves for each tissue in the analysis. Figure S3 shows modules in single tissue GGC networks as detected by the algorithm. Each cluster is marked by the yellow rectangles. Figure S4 shows single tissue module enrichment. Each panel has the following structure: top, *P*-values from FET for cis-eQTL blue bars over- and red bars under-enriched; middle, percentage overlap between module and genes with cis-eQTL; bottom, percentage overlap between each module and genes on each chromosome. The scale is between green and black where green represents 0% overlap and black 100% overlap. Figure S5 shows connectivity distribution for TTC networks. In each panel we show connectivity distribution for both types of genes in the TTC networks as follows: **(a) **blue for adipose, red for hypothalamus; **(b) **blue for hypothalamus, red for liver; **(c) **blue for adipose, red for liver. Figure S6 shows FDR curves for the TTC networks. Figure S7 shows a representation for the AH TTC network. Figure S8 shows a representation for the HL TTC Network. Figure S9 shows a representation for the AL TTC network. Figure S10 shows the number of network partition versus edge removal time. In black we show total number of subnetworks at each edge removal step; in blue we show number of 'open' subnetworks from where we can potentially remove edges. The number is obtained by subtracting from the total number of subnetworks in the partition the subnetworks defined as 'closed'. Figure S11 shows TTC network partitioning. For each of the TTC networks we have highlighted the subnetworks obtained through partitioning. Each color represents a subnetwork. The AH and HL networks are much more modular than the AL network. Figure S12 shows TTC network enrichment. Each panel has the following structure: top, *P*-values from FET for cis-eQTL over- (blue bars) and under-enriched (red bars); middle, percentage overlap between module and genes with cis-eQTLs; bottom, percentage overlap between each module and genes on each chromosome. The scale is between green and black where green represents 0% overlap and black 100% overlap. Figure S13 shows the TTC network backbones. Node color and symbols match the description from Figures 6, 7 and 8 in the main section of the paper. Each backbone contains the most robust links from the TTC network. Table T1 lists clinical trait descriptions. Table T2 lists microarray probe annotations. Table T3 lists the probes selected for single tissue analysis. Table T4 lists the adipose single tissue modules. Table T5 lists the hypothalamus single tissue modules. Table T6 lists the liver single tissue modules. Table T7 lists the AH TTC network. Table T8 lists the HL TTC network. Table T9 lists the AL TTC network. Table T10 lists the AH subnetworks. Table T11 lists the HL subnetworks. Table T12 lists the AL subnetworks. Table T13 provides the AH network backbone. Table T14 provides the HL network backbone. Table T15 provides the AL network backbone. Table T16 lists the adipose cis-eQTL genes. Table T17 lists the hypothalamus cis-eQTL genes. Table T18 lists the liver cis-eQTL genes. Click here for file
