# Supplementary material for: Multi-tissue coexpression networks reveal unexpected subnetworks associated with disease
Source: Genome Biol. 2009 May 22;10(5):R55. doi: 10.1186/gb-2009-10-5-r55 (PMC2718521; doi:10.1186/gb-2009-10-5-r55)
Supplement: Additional data file 1 — Figure S1 shows the connectivity distribution P(k) for GGC networks. Figure S2 shows FDR curves for each tissue in the analysis. Figure S3 shows modules in single tissue GGC networks as detected by the algorithm. Each cluster is marked by the yellow rectangles. Figure S4 shows single tissue module enrichment. Each panel has the following structure: top, P-values from FET for cis-eQTL blue bars over- and red bars under-enriched; middle, percentage overlap between module and genes with cis-eQTL; bottom, percentage overlap between each module and genes on each chromosome. The scale is between green and black where green represents 0% overlap and black 100% overlap. Figure S5 shows connectivity distribution for TTC networks. In each panel we show connectivity distribution for both types of genes in the TTC networks as follows: (a) blue for adipose, red for hypothalamus; (b) blue for hypothalamus, red for liver; (c) blue for adipose, red for liver. Figure S6 shows FDR curves for the TTC networks. Figure S7 shows a representation for the AH TTC network. Figure S8 shows a representation for the HL TTC Network. Figure S9 shows a representation for the AL TTC network. Figure S10 shows the number of network partition versus edge removal time. In black we show total number of subnetworks at each edge removal step; in blue we show number of 'open' subnetworks from where we can potentially remove edges. The number is obtained by subtracting from the total number of subnetworks in the partition the subnetworks defined as 'closed'. Figure S11 shows TTC network partitioning. For each of the TTC networks we have highlighted the subnetworks obtained through partitioning. Each color represents a subnetwork. The AH and HL networks are much more modular than the AL network. Figure S12 shows TTC network enrichment. Each panel has the following structure: top, P-values from FET for cis-eQTL over- (blue bars) and under-enriched (red bars); middle, percentage overlap between module and gene [file gb-2009-10-5-r55-S1.pdf]

Supplementary Material

Supporting Text

Single Tissue Analysis

Gene selection

To construct single tissue gene-gene correlation (GGC) networks we have used Spearman correlation. The subset of genes used in this type analysis included all genes that participate an any TTC network as well as genes that have a fold change (FC) higher that 1.5 in at least 10% of samples and have at least 75% real values (i.e. the microarray spot was not flagged as undesirable). The total number of genes on the microarray is 23698 and they are summarized in Table T2 while the 9967 probes included in single tissue analysis are presented in table T3. We will call this set the "single tissue" (ST) gene set. The connectivity distribution for each GGC network is presented in Figure S1.

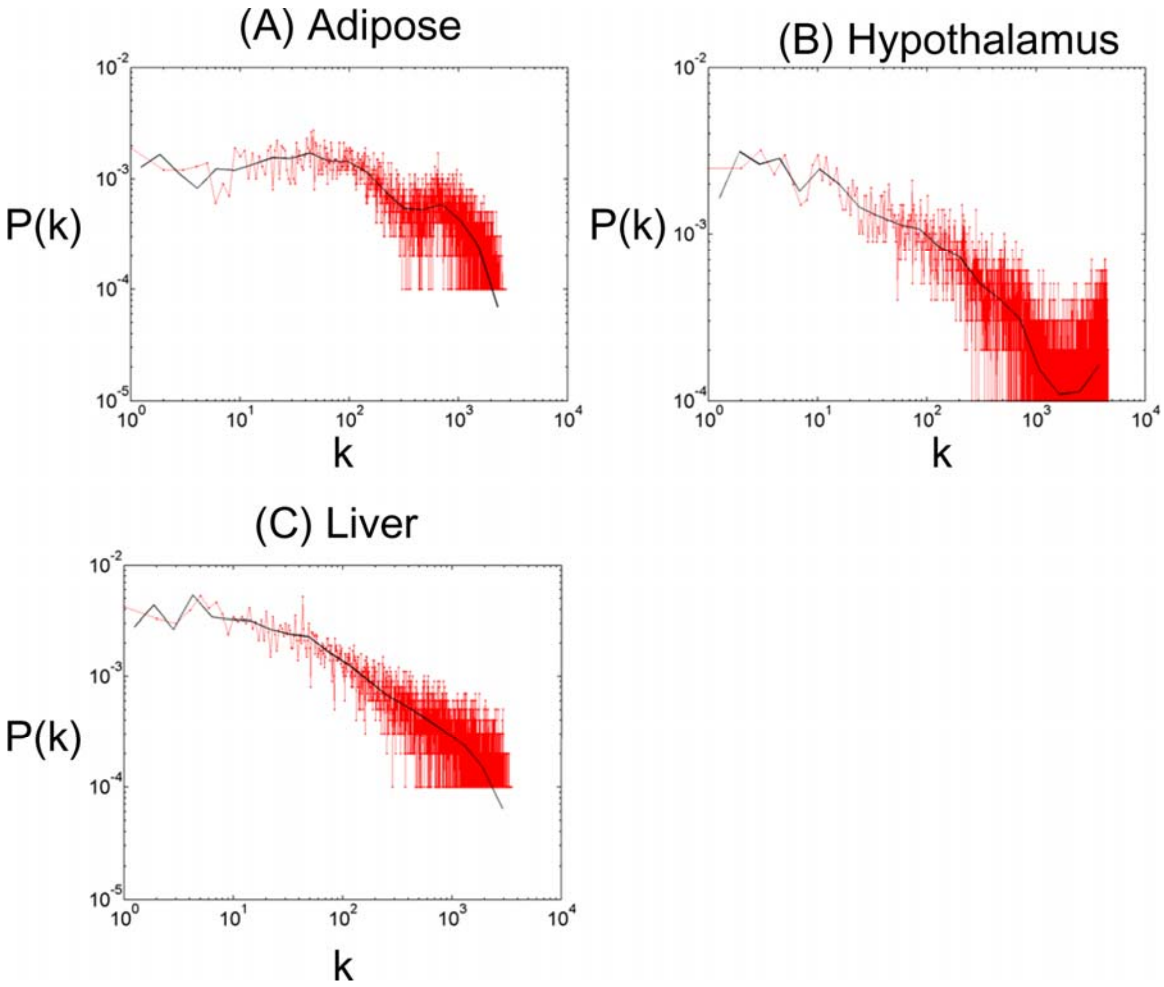

Figure S1. Connectivity distribution P(k) for GGC networks.

False Discovery Rate (FDR)

To obtain the FDR we used permutations to generate the null distribution. Figure S2 shows the FDR curves obtained for adipose, hypothalamus and liver tissues.

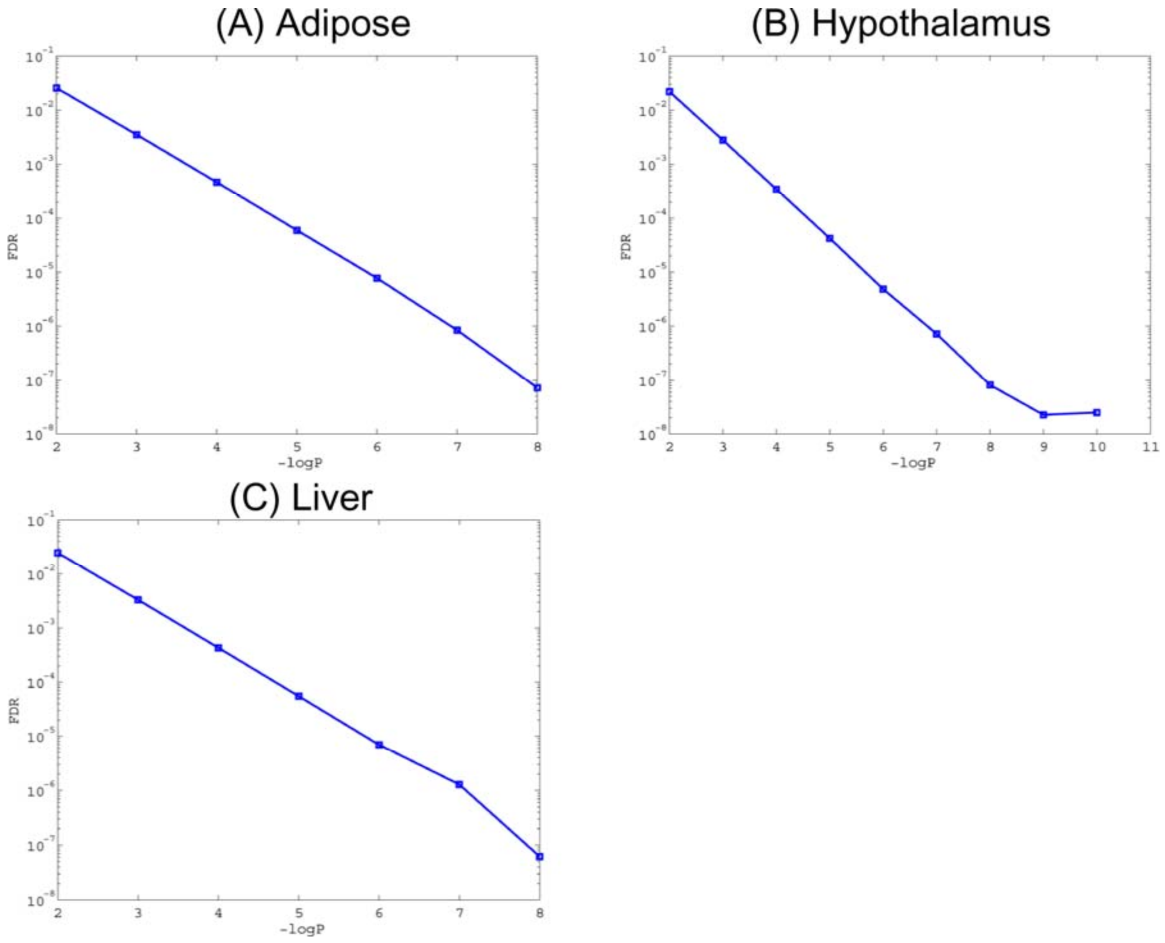

Figure S2. FDR curves for each tissue in the analysis.

Modules enrichment

Modules were constructed using the algorithm described above. The heatmaps in Figure S3 show the modules detected in each tissue. Each module is marked on each heatmap by a yellow square. The heatmap colors represent the GGC coexpression network where a red dot represents a link between 2 genes while a green dot indicates that there is no link between the genes. Both *x* and *y* axis represent the genes in the ST set.

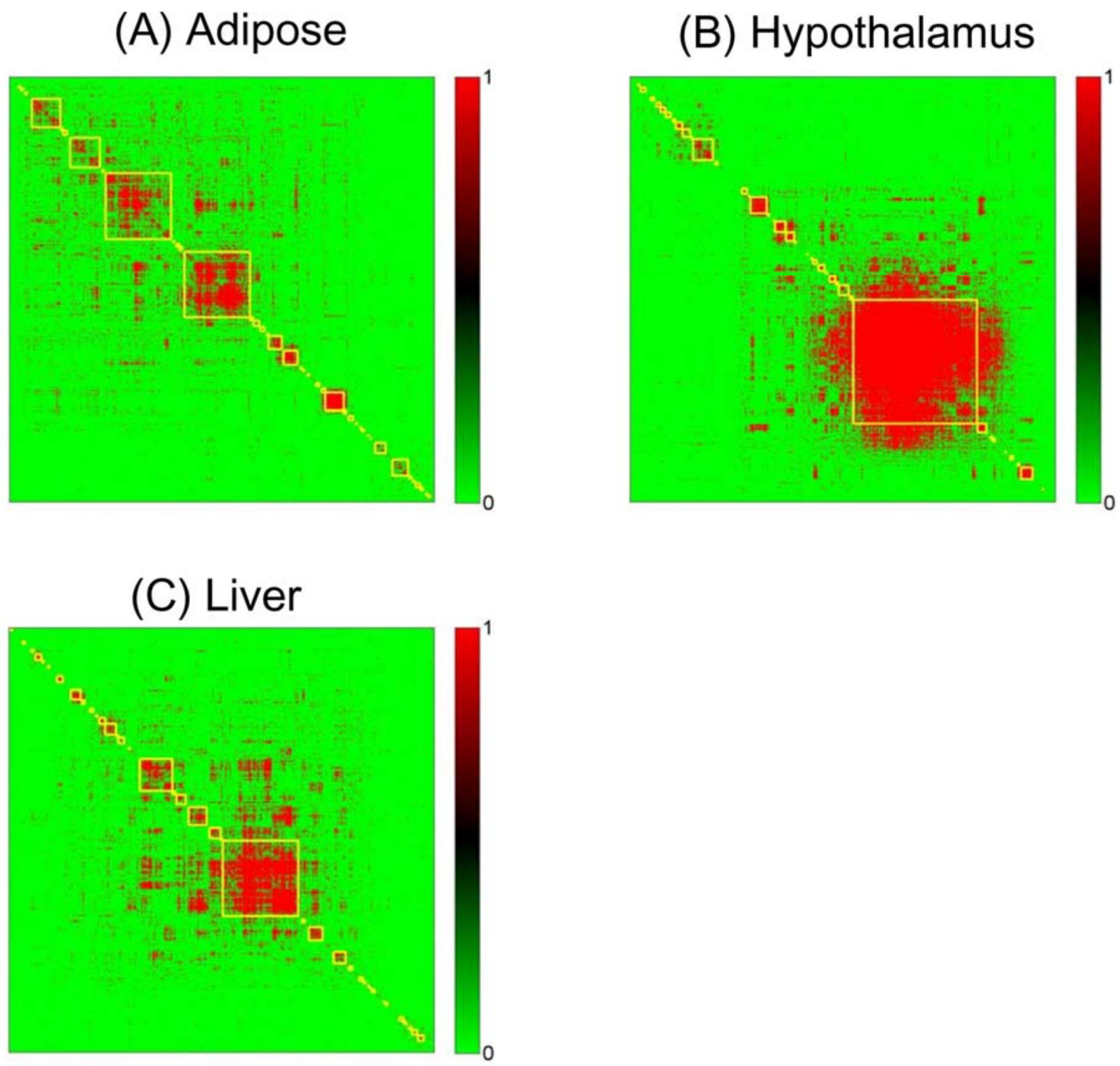

Figure S3. Modules in single tissue GGC networks as detected by the algorithm. Each cluster is marked by the yellow rectangles.

For each network we identified 20 topological clusters for that we tested chromosomal, cis eQTL, Gene Ontology (Biological Processes) and enrichment in genes associated with clinical traits. P values for all the overlaps are calculated

using Fischer's Exact Test (FET). To adjust for FDR dues to the fact that we are testing multiple hypotheses simultaneously we have used Benjamini-Hochberg (BH) method. To determine for each tissue the genes correlated with the 64 clinical traits we computed the Spearman correlation between expression traits in each tissue and the clinical traits. The p values were BH corrected. We selected only genes at10% FDR rate.

The cis eQTL end chromosomal enrichments are summarized in the Figure S4 and show that single tissue modules are not enriched for genes on a specific chromosome nor are they enriched in cis eQTLs.

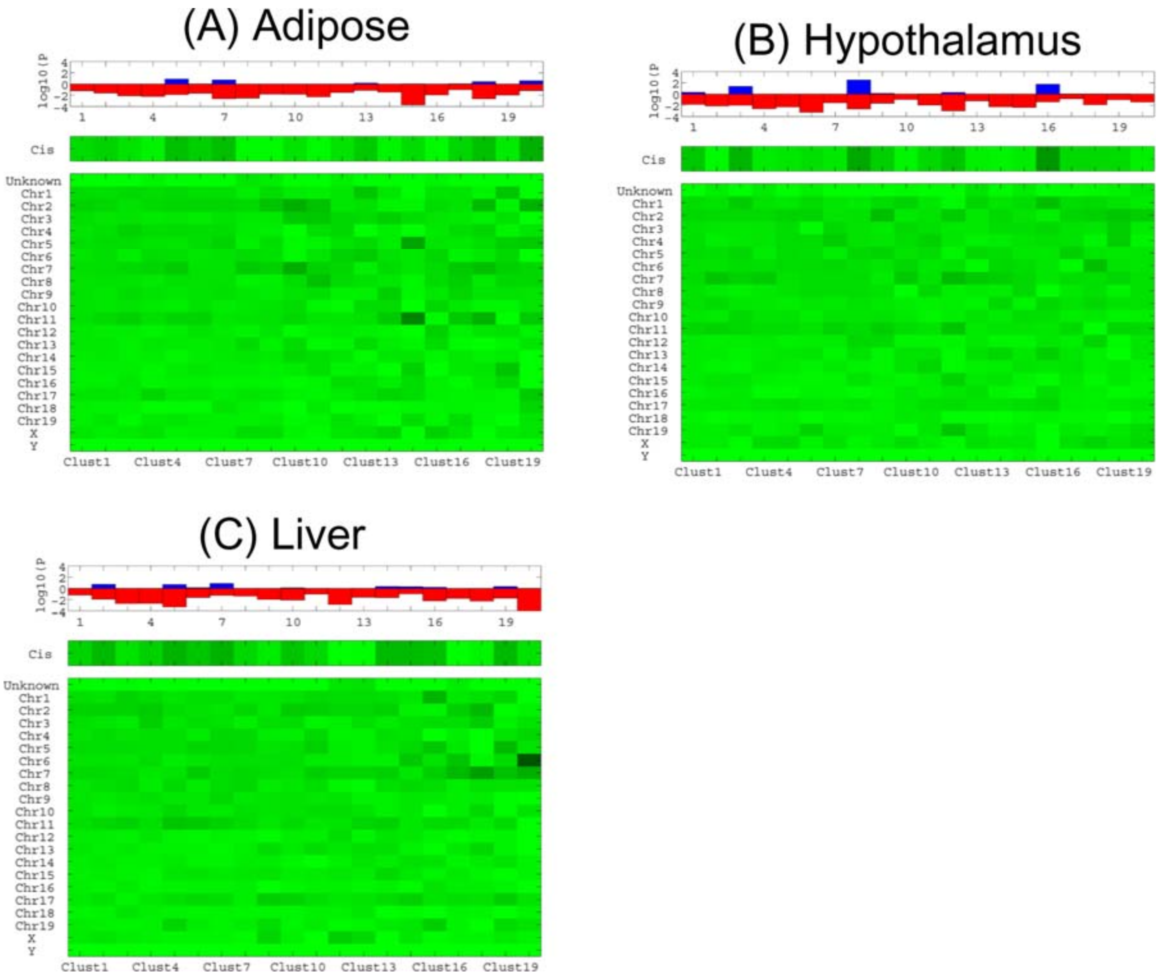

Figure S4. Single tissue module enrichment. Each panel has the following structure: top, p values from FET for cis-EQTLs blue bars over- and red bars under-enriched; middle, percentage overlap between module and genes with cis eQTLs; bottom percentage overlap between each module and genes on each chromosome. The scale is between green and black where green represents 0% overlap and black 100% overlap.

Supplementary results for Tissue-to-tissue (TTC) networks

TTC networks were constructed using Spearman correlation, where we kept only pair of genes that had p values less than 10^{-8}. A summary of each network size can be found in the main section of the paper.

TTC networks connectivity distribution in Figure S5 show scale-free behavior.

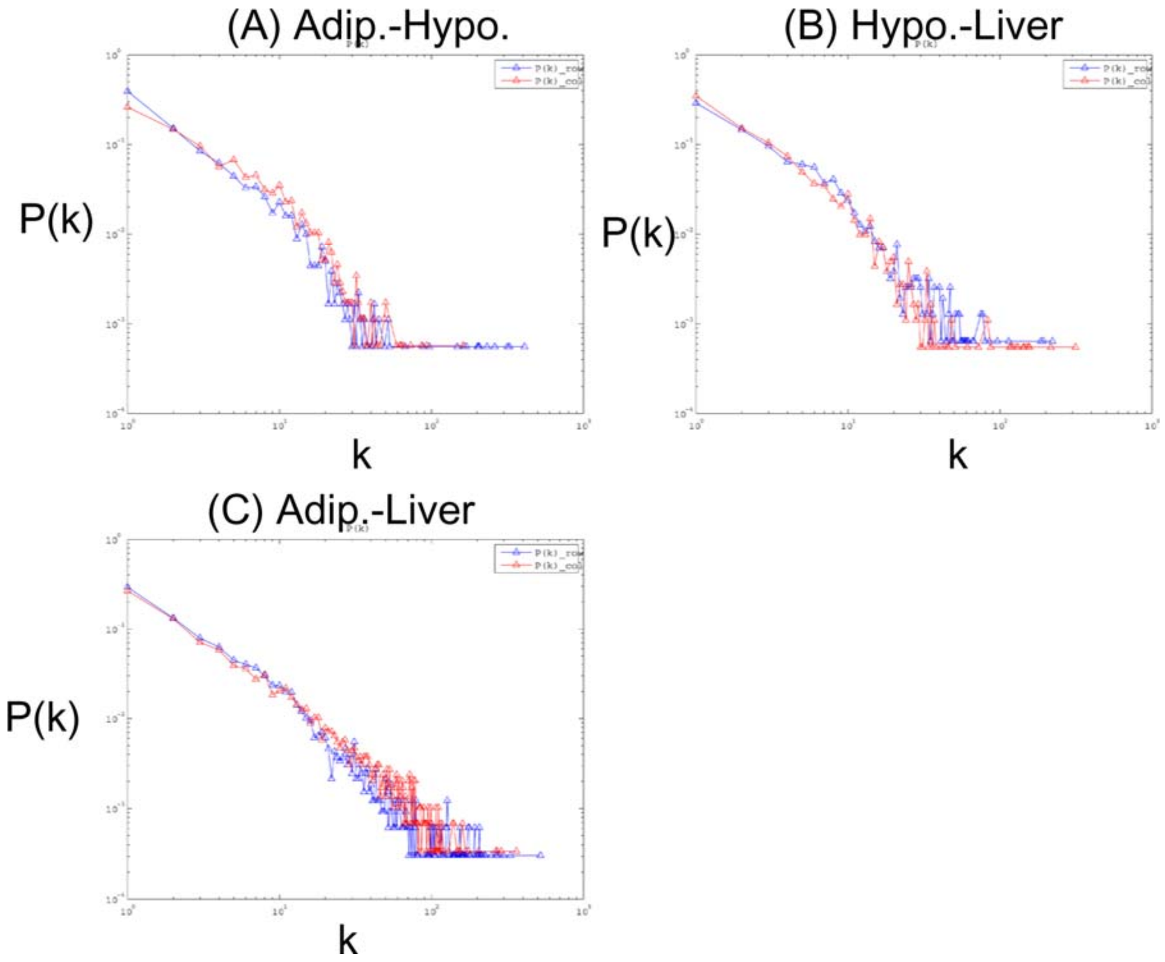

Figure S5. Connectivity distribution for TTC networks. In each panel we show connectivity distribution for both types of genes in the TTC networks as follows: (A) blue for adipose, red for hypothalamus; (B) blue for hypothalamus, red for liver; (C) blue for adipose, red for liver.

TTC Networks False Discovery Rate

Using permutation we computed the FDR for each TTC network as presented in Figure S6. All 3 FDR curves are similar and for p values $<10^{-9}$ FDR is zero. Thus we selected the p value threshold for all TTC networks to be 10^{-8} which corresponds to FDR$<10^{-3}$ for all 3 TTC networks.

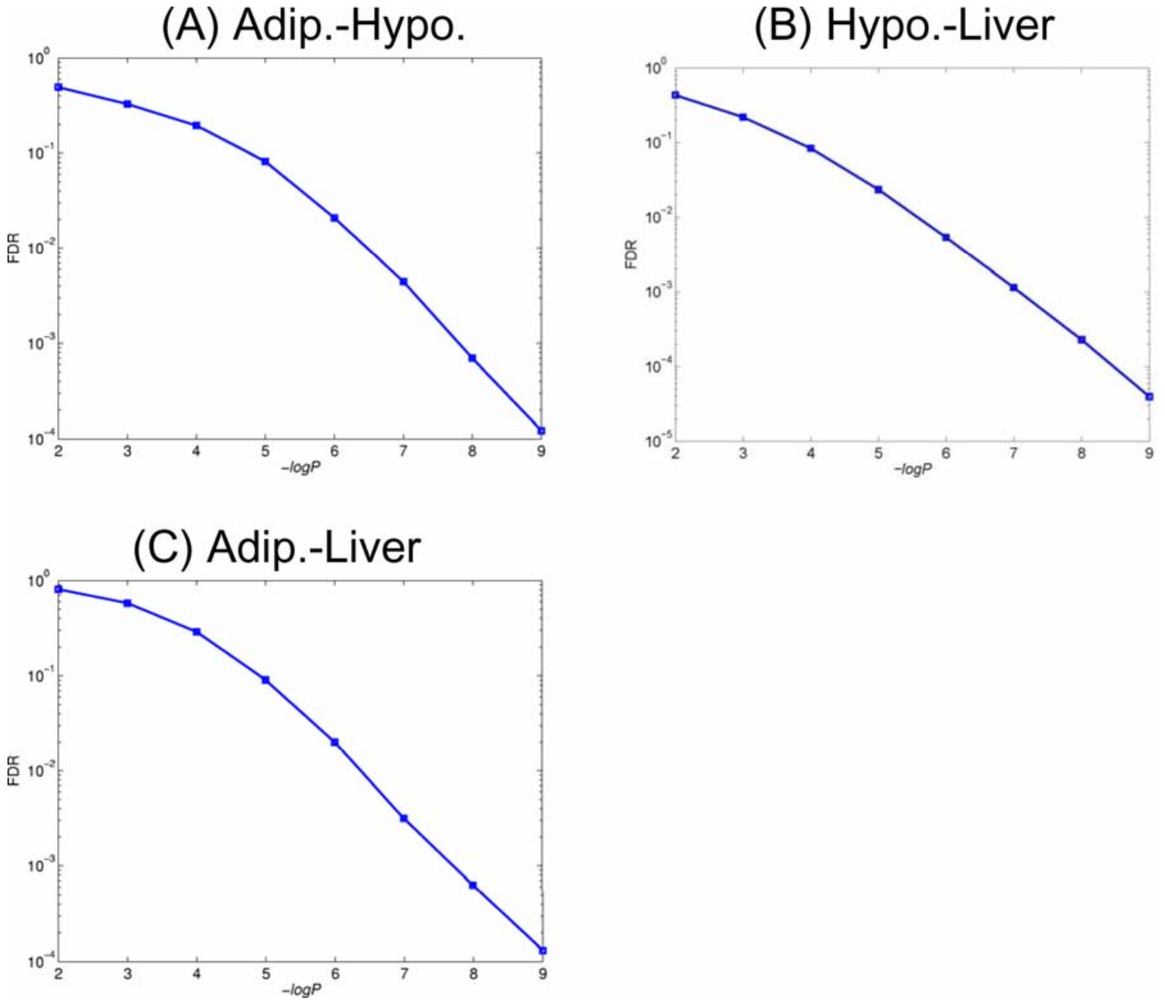

Figure S6. FDR curves for the TTC networks

TTC Networks Summary

We present in Figures S7-9 a summary of each TTC network.

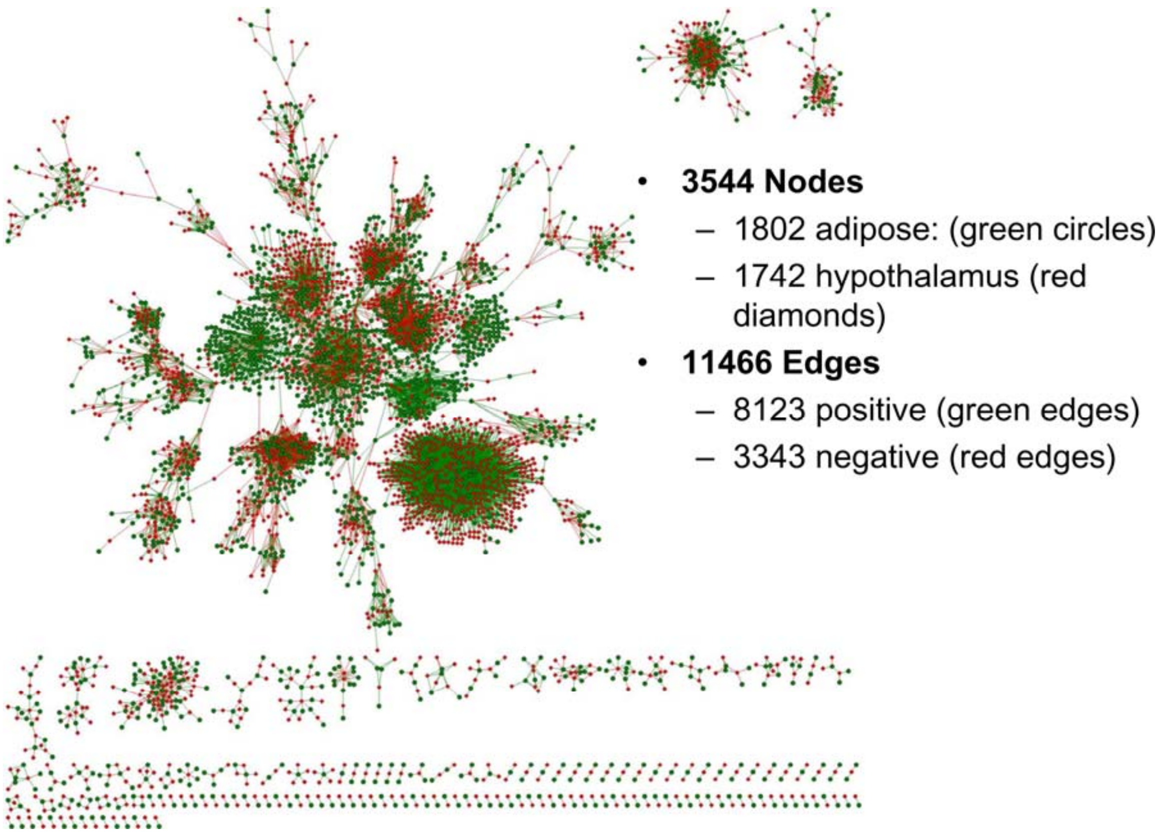

Figure S7. Adipose-Hypothalamus (AH) TTC network

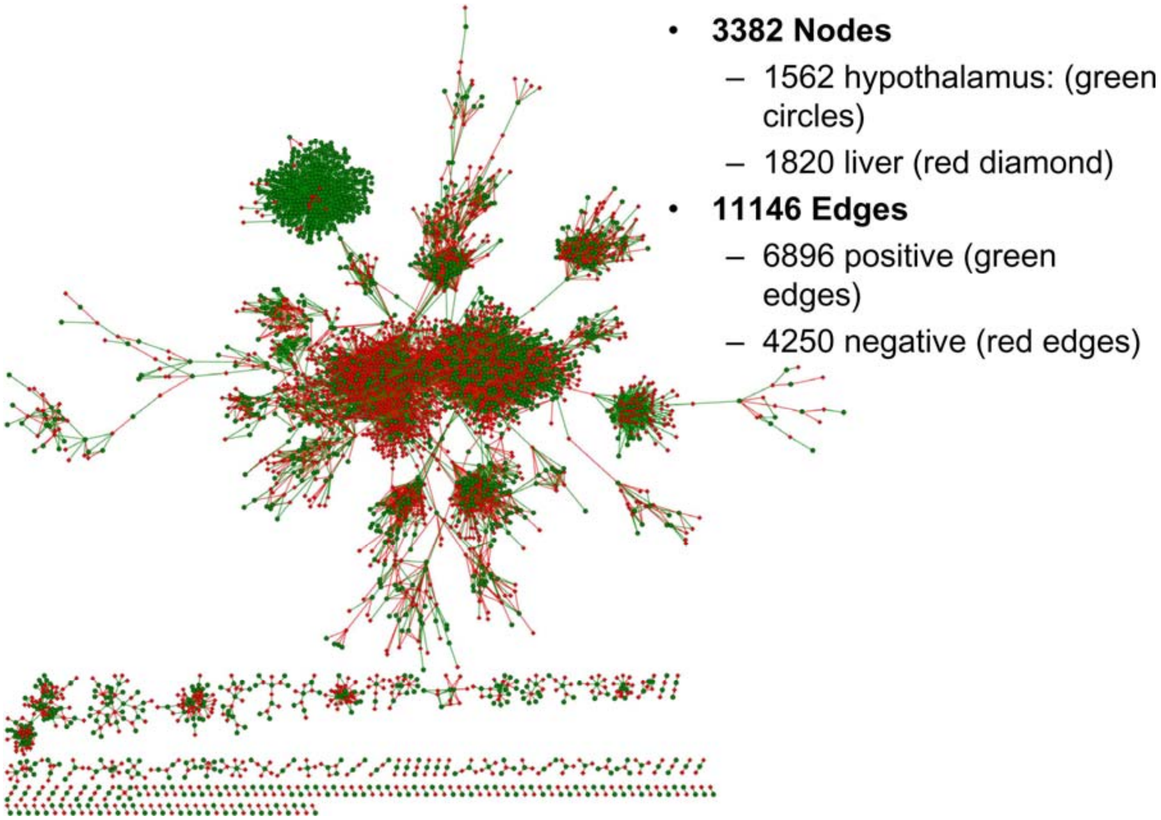

Figure S8. Hypothalamus-Liver (HL) TTC Network

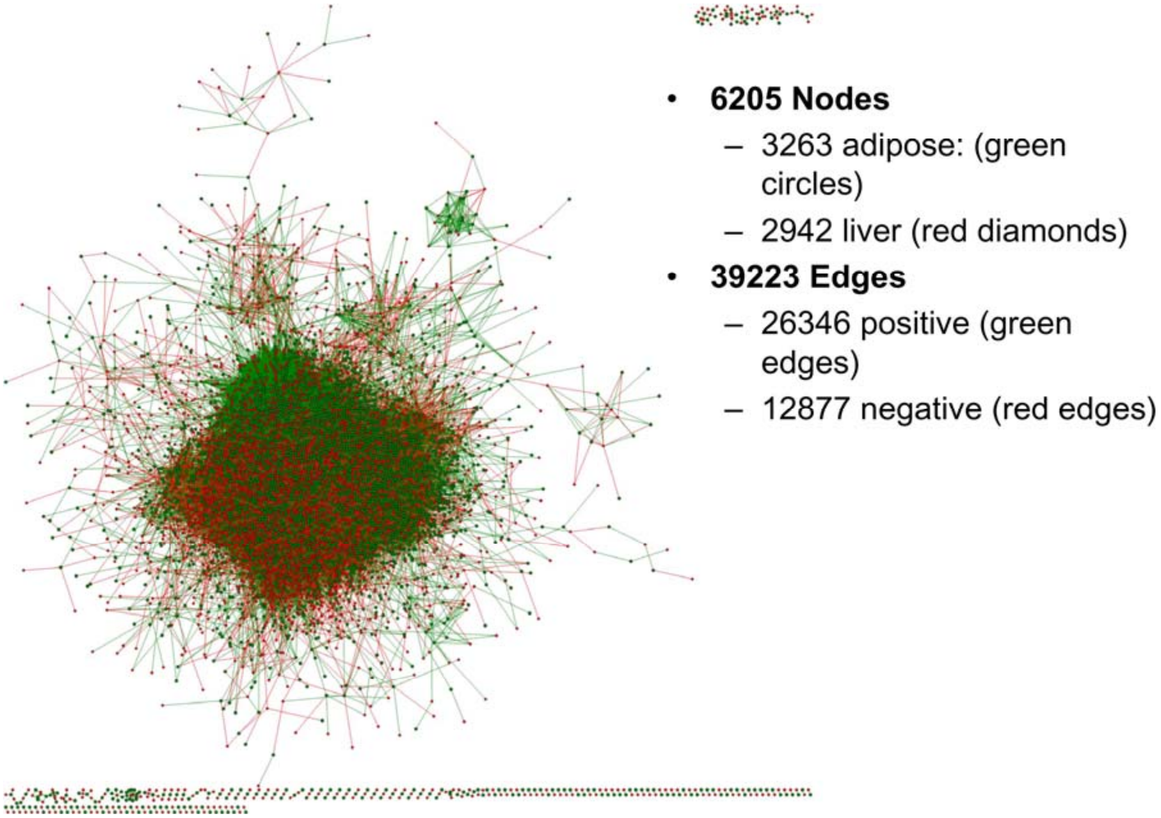

Figure S9. Adipose-Liver (AL) TTC Network

TTC Network Partitioning Algorithm

To partition the AH TTC network we have used an algorithm based on edge betweenness (eb) as previously described(*1*). The algorithm removes the edge with the largest edge betweenness until no edge is left to remove in the network. To speed up the algorithm we have identified 2 types of "closed" subnetworks from which there is no need to further remove edges (otherwise the subnetwork is considered "open" for removal): 1) when the subnetwork contains only 2 nodes and 2) when the maximum path length in 2. Each time when the algorithm detects "closed" subnetworks it completely removes them for further

consideration. Each time we remove an edge we don't necessarily have to obtain 2 disconnected subnetworks thus the number of subnetworks at each removal event can either increase or stay the same. In Figure S10 we show that the total number of subnetworks (black) and "open" subnetworks at every edge removal event.

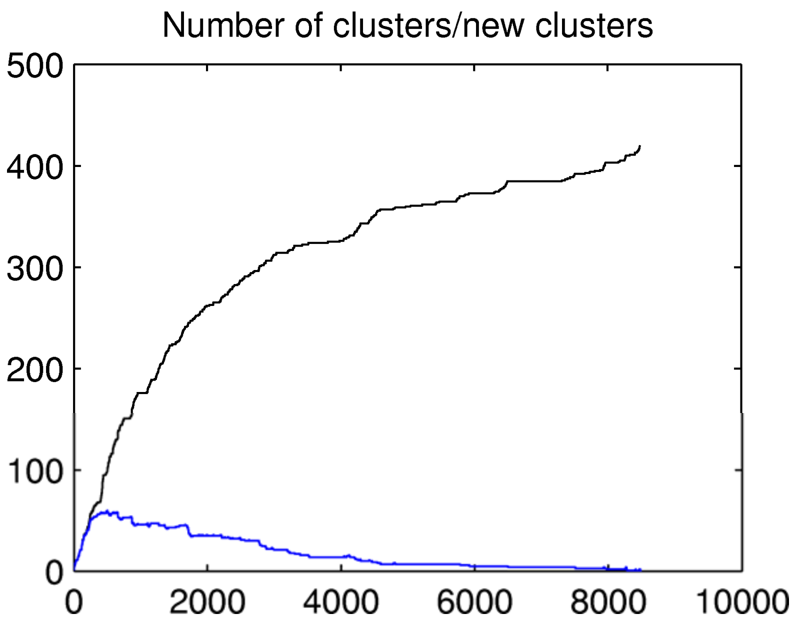

Figure S10. Number of network partition versus edge removal time. In black we show total number of subnetworks at each edge removal step. With blue we show number of "open" subnetworks from where we can potentially remove edges. The number is obtained by subtracting from the total number of subnetwork in the partition the subnetworks defined as "closed".

From Figure S10 we can identify the time interval during which the number of "open" subnetworks is not equal the total number of clusters. For the AH network we used the subnetworks after removing 221 edges from the network. Similar

plots can be generated for any network. We have tried several other partitioning algorithms (*2, 3*) that led to similar results. The subnetworks for each TTC network are highlighted in Figure S11.

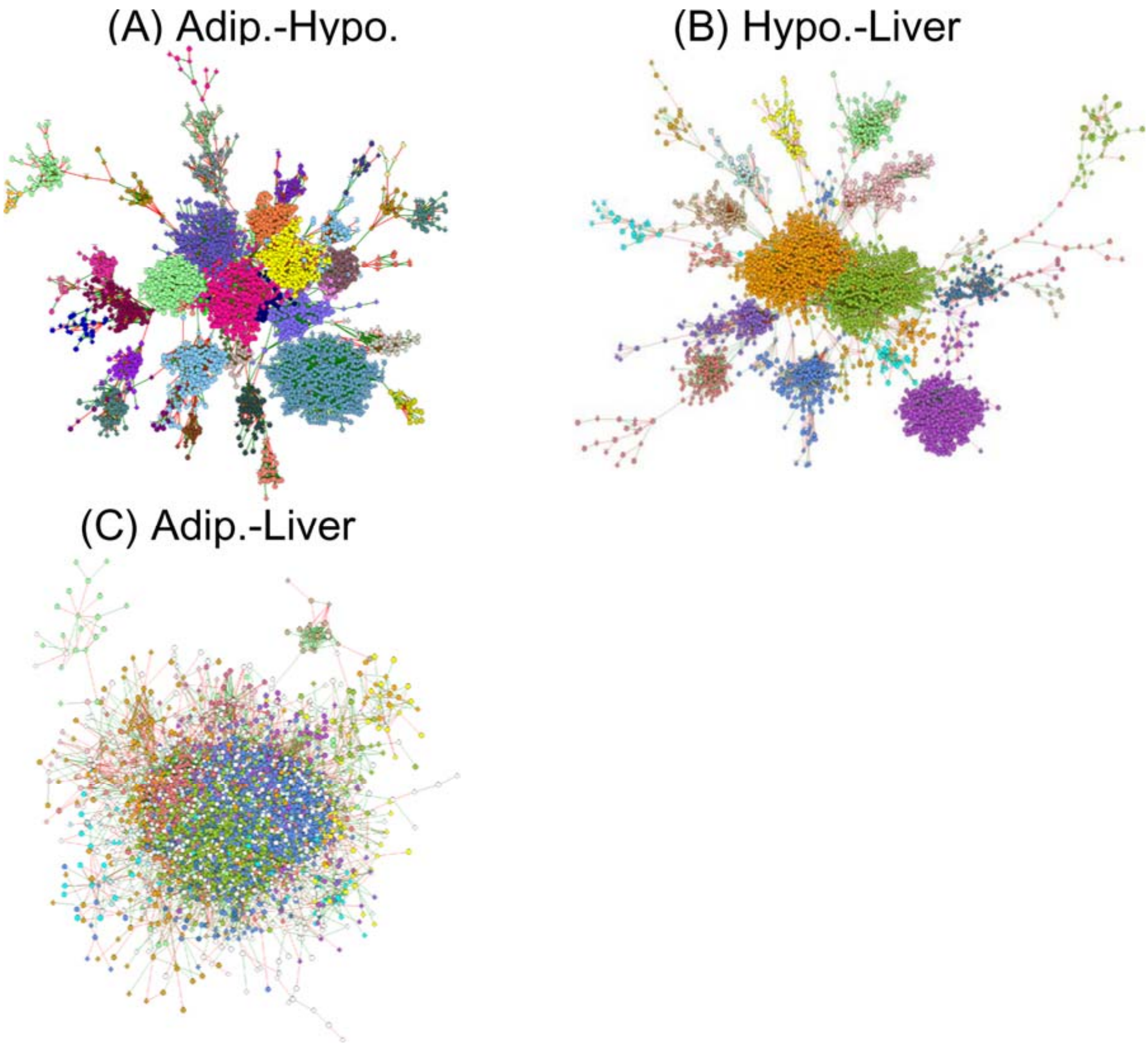

Figure S11. TTC networks partitioning. For each of the TTC networks we have highlighted the subnetworks obtained through partitioning. Each color represents a subnetwork. The AH and HL networks are much more modular than the AL network.

Subnetwork Enrichment

Once we generated the subnetworks we computed enrichment p values using FET for each subnetwork. We separated each type of nodes in the corresponding subnetworks and calculated enrichment for each node category (i.e. in the AH TTC network for adipose and hypothalamus nodes) and in the case of chromosomal location and cis eQTLs for all genes in the subnetwork taken together. Similar to Figure S4, in Figure S11 we show cis eQTL and chromosomal location enrichments for all 3 TTC networks. The 2 types of subnetworks described in the paper –type 1 that are enriched for cis eQTLs and chromosomal location and type 2 where there is no such enrichment - can be observed for all 3 TTC networks,

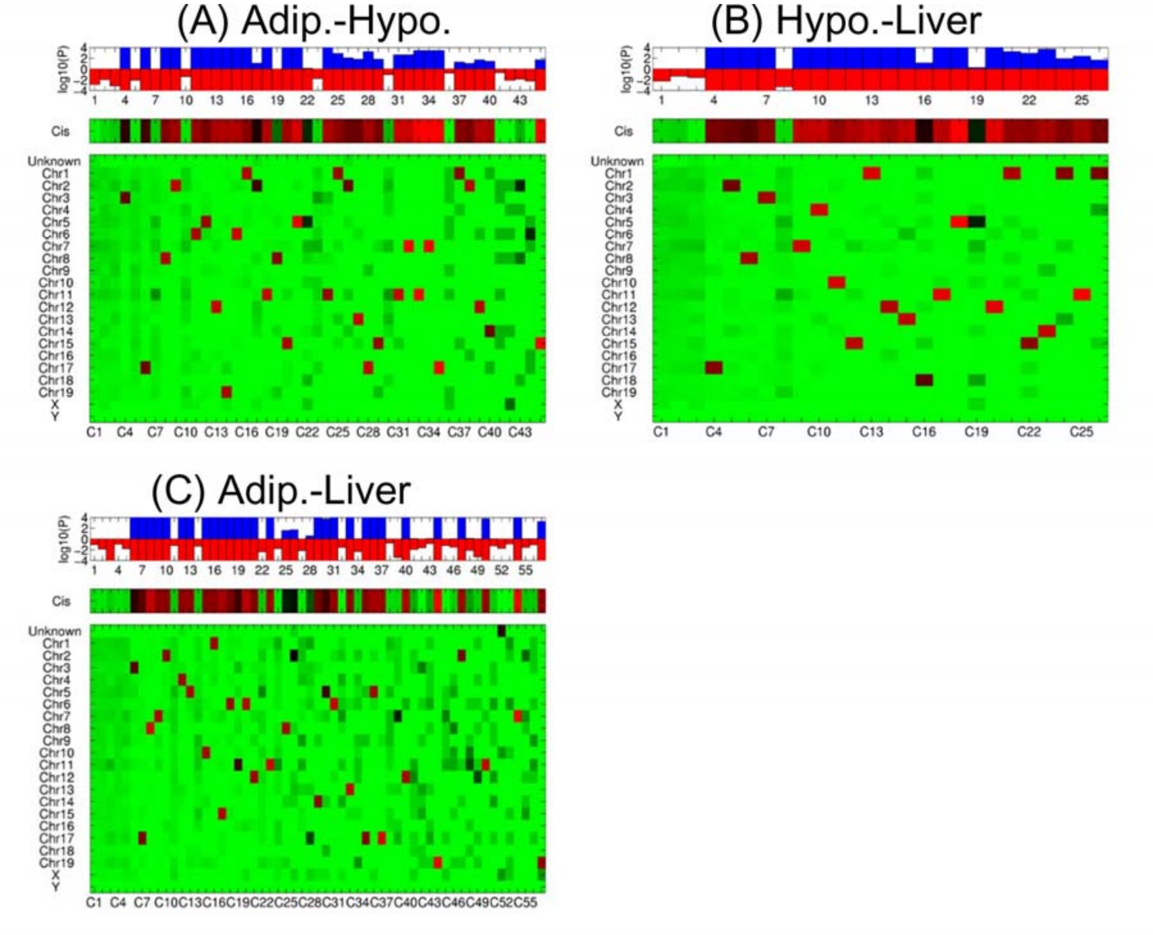

Figure S12. TTC Networks Enrichment. Each panel has the following structure: top, p values from FET for cis-EQTLs blue bars over- and red bars under-enriched; middle, percentage overlap between module and genes with cis eQTLs; bottom percentage overlap between each module and genes on each chromosome. The scale is between green and black where green represents 0% overlap and black 100% overlap.

TTC Network Backbone

Network backbone has to be by definition the most reliable part of the network. In order to construct it we have computed the weighted edge betweens(*1*) for all edges in the network. An edge connected weight is equal to the p value of the

correlation it denotes. Thus a shortest path is defined as the path between 2 genes along which the sum of edge weights is minimum. An edge is part of the backbone if its scaled edge-betweenness (defined as the total number of shortest paths that contain that edge divided to N-1, where N is the total number of nodes in the network) is larger that 1. Figure S12 depicts the backbones for the 3 TTC networks. The genes included in the backbone are thus the ones with the most robust correlations.

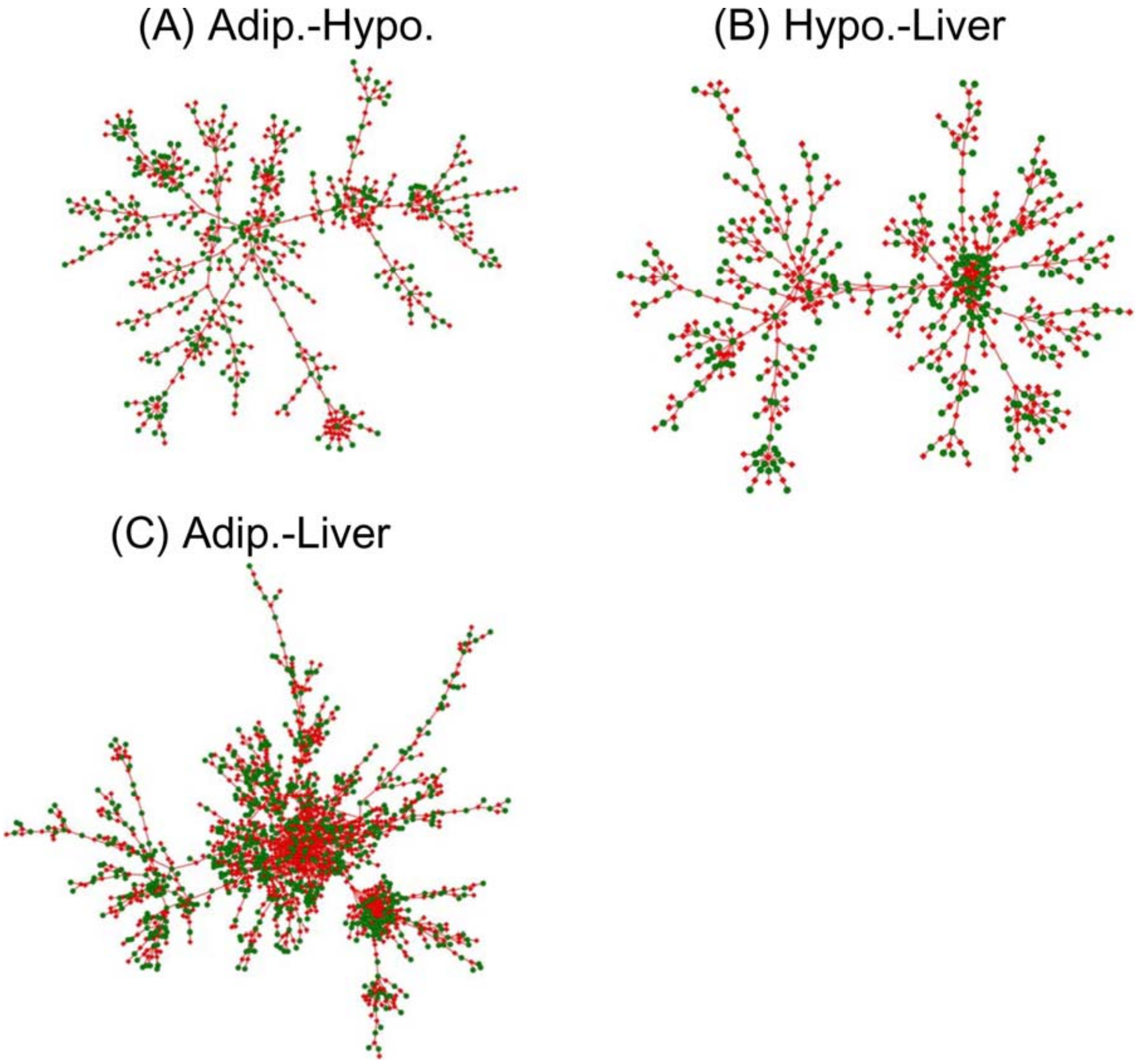

Figure S13. TTC network backbones. **Node color and symbols match the description from Figures 6-8 in the main section of the paper. Each backbone contains the most robust links from the TTC network.**

Supporting Tables

Table T1. Clinical traits description.

Clinical Trait	Additional Description
100xGonadal_Fat/Weight	
100xLiver/Weight	
100xSUBCUT./Weight	
100xlean/Weight	
100xtotal_tissue/Weight	
BMC	Bone mineral circumference
BMD	Bone mineral density
Bone_area	
Glucose	
IL6	
Insulin	
Leptin	
Liver	Whole liver
NMR_LEAN_MASS	
SUBCUTANEOUS	Subcutaneous fat
TNFa	
Total_tissue	
abdominal_fat	
intake_act_bw,W5	Daily intake week5 / body weight end week 5
intake_act_bw,W6	Daily intake week6 / body weight end week 6
intake_act_bw,W7	Daily intake week7 / body weight end week 7
intake_act_bw,W8	Daily intake week8 / body weight end week 8
intake_act_bw_avg,Day_avg	Average daily intake/ body weight end week 8
intake_amount,W5	Total intake amount week 5
intake_amount,W6	Total intake amount week 6
Intake_amount,W7	Total intake amount week 7
Intake_amount,W8	Total intake amount week 8
Intake_daily,Day_avg	Average daily intake
Intake_metab,Day_avg	Average daily intake/(75% weight at end of week 8)
Intake_total	Total intake
Intake_total_act_bw	Sum of intake_act_bw,W5, W6, W7, W8
Pixi_fat	Weight of pixi fat
Total_fat	Weight of total fat
Weight	body weight end week 8
Weight,W3	body weight end week 3
Weight,W4	body weight end week 4
Weight,W5	body weight end week 5
Weight,W6	body weight end week 6

Weight,W7	body weight end week 7
Weight_change,Int1	weight,W6- Weight,W3
Weight_change,Int2	Weight- Weight,W3
Weight_change,Int3	Weight- Weight,W4

Table T2: Microarray probe annotation

Probe_ID	Gene_Symbol	Entrez_ID	Chromosomal_Location
1	NAN		
2	NAN		
3	NAN		
4	NAN		
5	NAN		
6	NAN		
7	NAN		
8	NAN		
9	NAN		
10	NAN		
11	NAN		
12	NAN		
13	NAN		
14	NAN		
15	NAN		
16	NAN		
17	NAN		
18	NAN		
19	NAN		
20	NAN		
21	NAN		
22	NAN		
23	NAN		
24	NAN		
25	NAN		
26	NAN		
27	NAN		
28	NAN		
29	NAN		
30	NAN		
31	NAN		
32	NAN		
33	NAN		
34	NAN		
35	NAN		
36	NAN		
37	NAN		
38	NAN		
39	NAN		
40	NAN		
41	NAN		
42	NAN		
43	NAN		
44	NAN		
45	NAN		
46	NAN		
47	NAN		
48	NAN		
49	NAN		

50	NAN		
51	NAN		
52	NAN		
53	NAN		
54	NAN		
55	NAN		
56	NAN		
57	NAN		
58	NAN		
59	NAN		
60	NAN		
61	NAN		
62	NAN		
63	NAN		
64	NAN		
65	NAN		
66	NAN		
67	NAN		
68	NAN		
69	NAN		
70	NAN		
71	NAN		
72	NAN		
73	NAN		
74	NAN		
75	NAN		
76	NAN		
77	NAN		
78	NAN		
79	NAN		
80	NAN		
81	NAN		
82	NAN		
83	NAN		
84	NAN		
85	NAN		
86	NAN		
87	NAN		
88	NAN		
89	NAN		
90	NAN		
91	NAN		
92	NAN		
93	NAN		
94	NAN		
95	NAN		
96	NAN		
97	NAN		
98	NAN		
99	NAN		
100	NAN		

101	NAN		
102	NAN		
103	NAN		
104	NAN		
105	NAN		
106	NAN		
107	NAN		
108	NAN		
109	NAN		
110	NAN		
111	NAN		
112	NAN		
113	NAN		
114	NAN		
115	NAN		
116	NAN		
117	NAN		
118	NAN		
119	NAN		
120	NAN		
121	NAN		
122	NAN		
123	NAN		
124	NAN		
125	Zfp53	24132	17+:21616661-21646744
126	0610012D14Rik	68352	7+:51720804-51723121
127	Clic1	114584	17+:35187187-35195664
128	MMT00005933		6-:6175246-6239961
129	Podn	242608	4-:107687402-107704561
130	Ywhaq	22630	12-:21397075-21422989
131	Irx3	16373	8-:94322425-94325139
132	Zic2	22772	14+:122874656-122879074
133	Olfr313	258529	11+:58630511-58631437
134	Peli1	67245	11+:21035471-21050325
135	MMT00018153		
136	1700007N14Rik	74215	14+:64713759-64716509
137	Sorcs2	81840	5-:36359879-36740757
138	Bcdin3d	75284	15-:99300514-99305160
139	Mpst	246221	15+:78240468-78244435
140	Lemd2	224640	17-:27326702-27340693
141	Jag1	16449	2-:136908713-136942256
142	Gata6	14465	18+:11052499-11085684
143	Gvin1	74558	7-:113043647-113047500
144	Krt12	268482	11-:99276979-99283570
145	Tmem121	69195	12+:114425891-114427759
146	Rlbp1l2	215890	10-:33232090-33344002
147	Uox	22262	3+:146260130-146294369
148	Trps1	83925	15-:50491996-50721587
149	2310007L24Rik	75573	11+:106226784-106238428
150	Rpp38	227522	2-:3246216-3249873
151	Zfp697	242109	3+:98231389-98232553

152	Mark4	232944	7-:20010443-20043843
153	2010011I20Rik	67017	2+:172171136-172179397
154	Lmf2	105847	15-:89181435-89186047
155	Vmn2r7	319217	3-:64351163-64528955
156	MMT00002772		18+:51162686-51163443
157	MMT00042458		4-:106999987-107001135
158	Nppc	18159	1-:88566228-88567148
159	Gen1	209334	12-:11263643-11272572
160	MMT00078580		X-:147359162-147360639
161	MMT00011761		1-:110273614-110276016
162	Coro2a	107684	4-:46551945-46614720
163	Apoo	68316	X+:91625258-91657834
164	MMT00037331		6+:85105076-85106217
165	Scn5a	20271	9-:119394347-119471790
166	Chmp7	105513	14-:70116807-70132341
167	Gcn5l2	14534	11-:100566551-100573718
168	MMT00003638		14+:81374099-81374467
169	Nkx6-2	14912	7-:146764672-146768696
170	MMT00044003		2-:21364637-21365202
171	MMT00025388		18-:85885324-85886049
172	Tcra	21473	14+:54426200-54426876
173	Vps39	269338	2-:120143770-120178723
174	Mapkapk2	17164	1-:132950279-132955357
175	Psmb2	26445	4+:126354998-126386942
176	Sesn1	140742	10+:41530366-41628229
177	Appl2	216190	10-:83062780-83111409
178	MMT00018265	636126	12-:117106960-117248176
179	Pcgf2	22658	11-:97551138-97561660
180	Rbm39	170791	2-:155973371-156005876
181	Glmn	170823	5-:107978042-108026594
182	5830428H23Rik	414758	19-:61194718-61196414
183	Bmp4	12159	14-:47003534-47006863
184	Sorbs1	20411	19-:40369260-40588240
185	Ap2b1	71770	11+:83116275-83216514
186	Dap3	65111	3-:88727452-88753948
187	Wdr62	233064	7-:31025159-31034896
188	MMT00061078		1-:193098079-193129437
189	MMT00059108		X+:47726478-47727129
190	Tal2	21350	4+:53798692-53799017
191	Fbp2	14120	13-:62938243-62959676
192	Eif3d	55944	15-:77789430-77801190
193	C630028N24Rik	235386	9+:54768279-54795128
194	Nav3	260315	10-:109253309-109270103
195	BC018242	235044	9+:21742717-21753349
196	Nhlh2	18072	3+:101778944-101816984
197	MMT00002471		18+:9632405-9672593
198	Zranb2	53861	3-:157207977-157217790
199	Gabrb3	14402	7+:64846561-65080216
200	D0HXS9928E	108160	X+:71558371-71565472
201	B4galt4	56375	16+:38742394-38768950
202	Naif1	71254	2+:32307958-32312474

203	Eif4e	13684	3+:138190241-138219424
204	EG432649	432649	12+:36808292-36817625
205	Cnksr1	194231	4-:133783960-133794223
206	Dlgap2	244310	8+:14726928-14846707
207	2210408I21Rik	72371	13+:77274810-77407113
208	MMT00031901	433204	18+:80130659-80132771
209	Trim14	74735	4-:46517388-46548988
210	Scaper	244891	9-:55397686-55785917
211	AW125753	105005	12-:14155645-14158860
212	1700026J04Rik	71864	13-:35001832-35011798
213	Rgs16	19734	1+:155587478-155592599
214	Lrp1	16971	10-:126975244-127058182
215	Bbs10	71769	10+:110735778-110741521
216	Ighg	380794	12-:115910110-116148678
217	Ubxd2	67812	1+:130140470-130175951
218	4930511H11Rik	78016	1+:54386983-54425580
219	MMT00058440		12-:65977816-65978615
220	Smr2	20600	5+:88515580-88538078
221	1700009P17Rik	75472	1+:173051791-173057081
222	EG210155	210155	7-:25507928-25511931
223	Trp53inp2	68728	2+:155207575-155215575
224	Zhx2	387609	15+:57652791-57655302
225	MMT00073704	635169	4-:127605246-127628693
226	A730082K24Rik	101786	7+:122211497-122315904
227	B4galt1	14595	4-:40751636-40801009
228	MMT00046353		3-:11008847-11009612
229	Eif4b	75705	15+:101912416-101927603
230	MMT00000744		3+:65417429-65419248
231	Arl3	56350	19-:46605830-46632872
232	Pcdh9	211712	14-:94268644-94290147
233	Slfn10	237887	11-:82843292-82849677
234	4930470P17Rik	67637	2-:170404917-170427517
235	Hcrtr1	230777	4-:129807808-129816631
236	Bbs9	319845	9+:22280165-22483508
237	Cyp2c37	13096	19+:40066929-40086733
238	Rab12	19328	17-:66843852-66869024
239	2900054J07Rik	72958	13+:67880690-67885428
240	Tox4	268741	14+:52899493-52914184
241	BC030867	217216	11+:102110213-102126492
242	4732471D19Rik	319719	13+:54638439-54653391
243	Spg3a	73991	12+:70993822-71035908
244	Olfr167	258937	16-:19514788-19515727
245	Sqrdl	59010	2+:122610696-122635072
246	MMT00053742		13-:112672463-112673919
247	Clns1a	12729	7+:104845200-104867388
248	MMT00059950		
249	Mapk6	50772	9-:75235845-75246447
250	Tubb2a-ps2	627110	12-:11888133-11889704
251	Pkhd1	241035	1-:20107008-20418985
252	C730027P07Rik	245050	9+:121874316-121887805
253	Slc5a8	216225	10+:88381107-88382303

254	Olfr893	258333	9+:38016639-38017577
255	Twf2	23999	9+:106105573-106117694
256	Zbtb7a	16969	10+:80600778-80611198
257	Sftpc	20389	14-:70920746-70923877
258	Narg2	93697	9+:69254922-69258056
259	Bin3	57784	14+:70499919-70537764
260	Serpinb3d	394252	1-:108974771-108980057
261	Pgls	66171	8+:74116082-74120164
262	Icos	54167	1+:61034770-61055963
263	Akr1b3	11677	6-:34253935-34267421
264	Dcakd	68087	11-:102855362-102858862
265	Taf9	108143	13+:101420926-101436363
266	Nr2f2	11819	7-:77498327-77505249
267	Dync1h1	13424	12+:111839661-111905134
268	Sys1	66460	2+:164286495-164291012
269	Tuba1b	22143	15-:98762050-98763635
270	Mett11d1	52535	14+:52504484-52511542
271	4932417H02Rik	74370	11+:119465123-119760720
272	Tgm1	21816	14-:56318872-56332312
273	Ulk2	29869	11-:61591002-61668487
274	Trit1	66966	4+:122693839-122732177
275	Adam19	11492	11+:45869580-45959785
276	Olfr1019	259017	2-:85681013-85681946
277	MMT00073451		10-:21766000-21766840
278	Akap1	11640	11-:88692985-88712813
279	Fgf10	14165	13+:119504190-119578123
280	Snai2	20583	16+:14706026-14709467
281	MMT00063621		X-:53531300-53535766
282	Igh-VJ558	16061	12-:116026559-116027017
283	Gmcl1l	71847	X+:30732653-30734582
284	Osr2	107587	15+:35225866-35233059
285	Catsper1	225865	19+:5335740-5344152
286	MMT00070509	100037278	8+:74127597-74132047
287	MMT00023085		4+:32339197-32352800
288	Col6a2	12834	10-:76058506-76086059
289	Crb1	170788	1-:141094920-141273618
290	B230118H07Rik	68170	2-:101402262-101469143
291	Sbp	20234	17+:24078962-24082567
292	MMT00020389		1+:3044371-3045135
293	MMT00075127		
294	Ehhadh	74147	16-:21761365-21763011
295	Pdlim1	54132	19-:40296730-40346025
296	MMT00006951		5-:6426255-6429114
297	MMT00028756		11+:57262583-57263369
298	9630013A20Rik	319903	14-:84859476-84876230
299	MMT00061667		1-:53641301-53693021
300	Kcnk13	217826	12+:101204117-101300884
301	AU014645	433702	4+:46151508-46185276
302	Uprt	331487	X+:101678261-101697244
303	Casp8	12370	1+:58852269-58903856
304	Pus7l	78895	15-:94353071-94373932

305	Cma2	545055	14+:56569816-56592866
306	MMT00067578		X+:16829153-16831523
307	MMT00013484		5+:94167091-94170797
308	Meig1	104362	2-:3326318-3336313
309	Slc25a11	67863	11-:70457826-70460517
310	Olfr288	545140	15-:98017227-98025973
311	Dock3	208869	9-:106810842-106817008
312	Amfr	23802	8-:96495825-96536735
313	Ppp5c	19060	7-:17589996-17613248
314	Samd12	320679	15-:53489903-53551239
315	OTTMUSG000000	433287	1-:33892732-33895906
316	4930471G03Rik	74966	18+:36265806-36269607
317	Ceacam13	69785	7+:18595246-18604569
318	9130208D14Rik	77700	7-:112972738-112980029
319	MMT00032241		
320	MMT00043952		
321	Crbn	58799	6-:106729923-106750031
322	Abcb5	77706	12-:120106320-120204895
323	Tex13	83555	X-:137344599-137347629
324	9830134C10Rik	442827	17+:29282178-29285917
325	MMT00012327		10-:21155743-21156941
326	4931409K22Rik	231045	5-:24049250-24061227
327	H2-Ea	14968	17-:34479878-34481590
328	Map4k1	26411	7+:29767868-29788299
329	Lmod1	93689	1+:137221407-137263405
330	MMT00063866		5-:110508280-110528879
331	Rnf141	67150	7-:117959858-117987873
332	Accn4	241118	1+:75447024-75469922
333	Ciapin1	109006	8-:97346460-97356595
334	Ppp1r3f	54646	X-:7136078-7151246
335	V1ra8	113850	6+:90152810-90153650
336	Arhgef12	69632	9-:42773841-42914463
337	Rasgef1b	320292	5-:99646445-99681873
338	B930006L02Rik	319604	7+:107855198-107983967
339	Hmgcs1	208715	13_random-:114708-127798
340	Olfr610	259085	7-:110654511-110655458
341	1700007B14Rik	71831	8+:78078740-78287008
342	Nrbf2	641340	10-:66729439-66748031
343	1700123I01Rik	76663	19+:6184400-6224217
344	MMT00036566		1+:29794139-29881997
345	MMT00078552		6+:117758315-117759614
346	Olfr1484	258288	19+:13659795-13660741
347	Pdcl	67466	2-:37207351-37212895
348	Ccdc52	212514	16+:44347233-44387344
349	BC060632	244654	8+:113250085-113264785
350	St6galnac6	50935	2+:32462545-32475051
351	MMT00070461		10-:41082639-41083974
352	4732415M23Rik	320869	8+:125736748-125745944
353	Olfr447	258990	6+:42861523-42862455
354	MMT00007996		6-:67583076-67584137
355	Rasef	242505	4-:73360482-73380457

356	Sart1	20227	19-:5380059-5388661
357	Ftsj3	56095	11-:106110457-106117112
358	Ercc2	13871	7+:19967627-19979792
359	Ciita	12265	16+:10488371-10527656
360	Igh-VJ558	16061	12-:115984439-115984952
361	Fign	60344	2-:63815896-63936045
362	Gng13	64337	17+:25855645-25855976
363	Adora2a	11540	10+:74787665-74797535
364	0610040B09Rik	75395	11-:96152349-96154868
365	1700019N12Rik	67077	19-:6996915-6999870
366	Adcy6	11512	15-:98420421-98435257
367	Hoxc13	15422	15+:102751780-102757855
368	Ccdc97	52132	7-:26496135-26504072
369	Lcn6	620709	2+:25532303-25532741
370	MMT00012017		19-:12407384-12410207
371	MMT00058786		8-:109560987-109562129
372	Txndc1	72736	12+:71554101-71569008
373	BC066135	271887	2-:180013806-180015691
374	Slc25a40	319653	5+:8422281-8452820
375	MMT00077729		19+:23680345-23734472
376	Tbc1d2b	67016	9-:90099151-90114000
377	MMT00004099		14+:9241367-9243187
378	Hrh4	225192	18+:13165543-13181089
379	Nmnat1	66454	4-:148842745-148848706
380	Fry	320365	5+:151062843-151300326
381	Rbmxrt	19656	8-:81029169-81032740
382	Nsf	18195	11-:103684371-103815328
383	Thoc2	331401	X-:39150337-39160580
384	Olfr668	259061	7-:112073321-112074276
385	Cog3	338337	14-:76102158-76154245
386	Deaf1	54006	7-:148483083-148513477
387	Rgs10	67865	7-:135517138-135561686
388	Chst5	56773	8-:114413035-114434099
389	MMT00057495		X+:73442850-73444156
390	MMT00047307	639774	4+:111591992-111609723
391	4933416I08Rik	71159	X+:50989700-50994509
392	Sec22b	20333	3+:97705203-97725429
393	Ucp1	22227	8+:85814481-85821868
394	Ccdc85a	216613	11-:28288819-28334158
395	Cep27	66296	2+:120435171-120445549
396	Sepsecs	211006	5-:53031331-53060947
397	Ak7	78801	12+:106944326-107020654
398	BC025446	223631	15+:75047025-75053089
399	Cr1l	12946	1-:196930077-196957759
400	EG434402	434402	9+:48302955-48304076
401	MMT00061194		4+:33051752-33052247
402	Timp3	21859	10+:85763281-85812250
403	Leprotl1	68192	8-:35200370-35209862
404	Fcrlb	435653	1-:172837403-172842410
405	Emr4	52614	17+:55889297-55992735
406	Wbp2	22378	11-:115939887-115948278

407	Serpina6	12401	12-:104884842-104895281
408	Fpr3	14294	17+:18024815-18108641
409	Lxn	17035	3-:67261918-67267735
410	MMT00048420		17+:85248800-85273194
411	Ccdc68	381175	18+:70098496-70129265
412	Cib4	73259	5-:30787955-30848209
413	Acy1	109652	9-:106335326-106340567
414	Prpf40a	56194	2-:52997511-53050221
415	Ypel2	77864	11+:86749926-86750408
416	Xlr5b	627081	X+:70397218-70401916
417	E030010N08Rik	381284	1+:95109362-95112311
418	MMT00068225		10+:68787954-68788417
419	Foxk2	68837	11+:121121570-121171208
420	Ear11	93726	14-:51875113-51875581
421	V1rb2	24112	6-:90022080-90023012
422	Vmn2r69	330581	7-:92555098-92561077
423	Klhl5	71778	5+:65499025-65559424
424	Olfr1240	258804	2-:89279490-89280434
425	MMT00064181		X-:53144982-53147353
426	Mobkl3	19070	1+:55188133-55210100
427	Smg6	103677	11+:74802858-74805687
428	Klhl29	208439	12-:5087957-5298259
429	2410012H22Rik	69747	11-:62080721-62082322
430	Becn1	56208	11-:101150126-101163168
431	MMT00010015		10-:120202829-120215942
432	EG215974	215974	10+:44723195-44726921
433	Pla2g12b	69836	10+:58866410-58884481
434	Psmd1	70247	1+:87961212-88035865
435	Arpc1a	56443	5+:145844737-145869625
436	Prnp	19122	2+:131735692-131764163
437	Ndrg1	17988	15-:66760883-66801201
438	MMT00045243		X-:23431938-23433160
439	Ugt2b35	243085	5+:87429916-87437385
440	Psip1	101739	4-:83101585-83131696
441	MMT00000745		6+:41101789-41138740
442	Ngb	64242	12-:88439267-88443286
443	Xpo5	72322	17+:46339694-46380544
444	Uba52	22186	8-:73032172-73033534
445	2610029I01Rik	77032	4-:21684530-21694363
446	MMT00020922		
447	Cpa4	71791	6+:30523862-30542417
448	Sh3glb2	227700	2-:30200330-30214751
449	1700019P21Rik	75546	1-:140772851-140773554
450	Gpr162	14788	6-:124808464-124813792
451	Mllt11	56772	3-:95023058-95032166
452	Capzb	12345	4+:138748922-138847181
453	Uqcr	66594	10-:79865743-79869575
454	Klrg1	50928	6-:122220621-122232851
455	MMT00052147		
456	Frmd6	319710	12+:72001195-72003219
457	Cacng5	140723	11-:107738666-107745855

458	Olfr876	258883	9+:37611497-37612429
459	Tcfap2a	21418	13-:40812269-40829150
460	1700011A15Rik	66322	15+:101278204-101284335
461	Zfp664	269704	5+:125343059-125369006
462	Vdac2	22334	14+:22650782-22665099
463	Olfr638	259124	7+:111151772-111152736
464	2310038E17Rik	71903	8+:107482964-107483897
465	6430571L13Rik	235599	9+:107244680-107250961
466	Ampd3	11717	7+:117921317-117953376
467	Ube2b	22210	11+:51798649-51799734
468	2610020C07Rik	69918	16+:11203489-11220817
469	Drg1	13494	11-:3149924-3166389
470	MMT00076063		7-:14168976-14170985
471	MMT00012978		11-:6425473-6428703
472	EG434782	434782	X-:57298120-57299423
473	MMT00063585		X-:53407721-53409696
474	Il20ra	237313	10+:19432400-19479459
475	Matr3	17184	18+:35731677-35732596
476	Lyrm1	73919	7+:127039805-127060264
477	Vmn2r3	637004	3-:64062882-64091417
478	Sfrs9	108014	5+:115777225-115783086
479	Best1	24115	19-:10059660-10067811
480	Zdhhc6	66980	19-:55372790-55390516
481	MMT00015583		3-:69370441-69371448
482	Rnf43	207742	11+:87477636-87549040
483	Atp5j	11957	16-:84828116-84831744
484	Atp4a	11944	7+:31497370-31510203
485	MMT00042797		2-:77498710-77499405
486	Josd2	66124	7+:51723425-51727021
487	Slc6a15	103098	10+:102830476-102882011
488	Igh-VJ558	16061	12-:116396590-116397077
489	Itga4	16401	2+:79095745-79166935
490	Klf5	12224	14+:99698047-99712632
491	MMT00030239		2+:11341818-11343062
492	Cd37	12493	7-:52489270-52494098
493	Acvrl1	11482	15+:100958952-100974069
494	Tspan7	21912	X+:10062293-10173728
495	Ddx10	77591	9-:52906745-53056196
496	Fgf1	14164	18-:38999700-39078312
497	Myrip	245049	9+:120244714-120381985
498	MMT00055322		10+:84776247-84777188
499	2310057J16Rik	69697	8+:3587811-3602281
500	OTTMUSG00000(	791364	6+:52212761-52216083
501	MMT00051491		8-:32390340-32391270
502	Iars	105148	13+:49777445-49829628
503	Gsto1	14873	19+:47929478-47939276
504	Jmjd2a	230674	4-:117809563-117850181
505	Dennd2c	329727	3+:102956187-102970422
506	Gm207	240879	1-:165633186-165647389
507	Leprel2	14789	6-:124791112-124807705
508	MMT00028550		2+:83985036-83985869

509	MMT00046302		13-:20796215-20797275
510	Neu1	18010	17+:35068318-35071775
511	Itpa	16434	2+:130493550-130507349
512	Asna1	56495	8-:87541830-87549177
513	Krtap16-8	68484	16-:89047544-89048126
514	Abca6	76184	11-:110038137-110113042
515	Ica1l	70375	1-:60039332-60085073
516	Olfr1115	258294	2+:87092095-87093075
517	Slc17a7	72961	7+:52419167-52431507
518	B3gnt4	231727	5+:123960582-123961635
519	EG627299	627299	X+:59083639-59085712
520	H2-T3	15043	17-:36323516-36327073
521	Dnajb12	56709	10+:59342365-59359523
522	Cntln	338349	4+:84530307-84777821
523	1700040L02Rik	73287	10-:67893701-67996666
524	Hsd3b2	15493	3-:98515030-98528478
525	Lyl1	17095	8+:87225442-87228591
526	Car8	12319	4-:8070511-8166167
527	Olfr1279	258388	2+:111146693-111147955
528	Mrps24	64660	11-:5603985-5607688
529	Tbx22	245572	X+:104863302-104884314
530	Mest	17294	6+:30688062-30698457
531	E130201H02Rik	78552	7+:127741134-127741791
532	R3hdm2	71750	10+:126881331-126936427
533	MMT00040762		2-:150222864-150225421
534	Plrg1	53317	3+:82860692-82876092
535	Fbxl20	72194	11+:97943867-97947256
536	Got2	14719	8-:98388036-98412265
537	Dmbt1	12945	7+:138175590-138265140
538	Mpp2	50997	11-:101918332-101949816
539	MMT00024269		
540	Chd9	109151	8+:93456138-93507447
541	Olfr570	259114	7+:110048882-110049819
542	RP23-451D4.3	279185	4+:143204962-143207651
543	Braf	109880	6-:39564111-39593219
544	Il16	16170	7-:90791575-90803906
545	4930524O08Rik	74700	9+:89721353-89758866
546	Gltscr1	243842	7-:16556611-16584843
547	Mknk2	17347	10-:80128787-80134754
548	Gpr109a	80885	5-:124313585-124315505
549	9330101J02Rik	664857	7-:146866720-146869369
550	Hars2	70791	18+:36942933-36952207
551	Vill	22351	9+:118961915-118980455
552	MMT00013481		5-:94124018-94126763
553	Plekhb2	226971	1+:34906843-34936413
554	Eml5	319670	12-:100098164-100099522
555	MMT00074502		1-:184057951-184058631
556	Spint2	20733	7-:30041350-30066997
557	0610010D20Rik	67432	19+:42120340-42145429
558	Crebl1	12915	17+:34784179-34791641
559	Tyro3	22174	2+:119625305-119643217

560	Sprr2d	20758	3+:92143079-92144793
561	Smarcd1	83797	15+:99532760-99544421
562	Cabin1	104248	10-:75108855-75226916
563	D4Bwg0951e	52829	4+:80557282-80600207
564	Endogl1	208194	9+:119354069-119374623
565	MMT00048441		17+:85917619-85934316
566	Ccdc132	73288	6+:3505430-3553313
567	Gpc5	103978	14+:115491542-115532530
568	A330049M08Rik	230822	4-:134925824-134954136
569	1700125D06Rik	68233	4+:129168442-129169450
570	Zfp371	170734	7+:6181780-6185570
571	Cd209c	170776	8-:3940280-3945908
572	MMT00014316		4-:84569446-84572305
573	En1	13798	1+:122499257-122504545
574	Zfp57	22715	17+:37139982-37147580
575	Edn1	13614	13+:42397233-42403356
576	MMT00057952		X+:73337281-73408402
577	Rrp12	107094	19-:41937331-41970690
578	Gpr35	64095	1+:94869782-94881620
579	Pcdhb7	93878	18+:37501559-37504457
580	MMT00003547		4-:27923780-27923977
581	Zzz3	108946	3+:152091576-152123216
582	Pcgf1	69837	6+:83028184-83030848
583	Taf15	70439	11+:83286586-83320246
584	Tmem207	224058	16-:26504592-26526809
585	Tas2r119	57254	15+:32107043-32108047
586	Glb1l	74577	1-:75193552-75206077
587	Sco1	52892	11+:66866168-66880942
588	Ssxb2	387132	X-:8031470-8038853
589	Epb4.1l4b	54357	4-:57074610-57156520
590	MMT00078555	238771	13+:93614741-93619030
591	Slco6c1	74441	1-:98955615-99024881
592	MMT00075676		2-:153912614-153920539
593	Zcchc3	67917	2-:152239311-152240513
594	Evc2	68525	5+:37729786-37815914
595	Cdh8	12564	8-:101554282-101940011
596	1110019K23Rik	665563	5+:91375815-91450389
597	Xcr1	23832	9-:123764800-123765768
598	V1rb9	113857	6+:89696417-89697407
599	Prm1	19118	16-:10796576-10796824
600	Pfpl	56093	19+:12502418-12505682
601	Sytl3	83672	17+:6877959-6942184
602	9630041N07Rik	214779	11-:50645537-50652056
603	Cckbr	12426	7+:112574538-112583935
604	Ppp1r15b	108954	1+:135027863-135036364
605	Unc119b	106840	5-:115572577-115584936
606	Vmn2r96	433070	17+:18719501-18723401
607	Me1	17436	9-:86474982-86589830
608	Olfr1360	258536	13-:21765871-21766811
609	MMT00060868		13-:63068834-63070096
610	MMT00002170		3-:92121379-92212836

611	Tac2	21334	10+:127161533-127168823
612	Ccr1	12768	9-:123878257-123879324
613	Calcoco2	76815	11-:95960229-95973274
614	C8g	69379	2-:25354166-25357244
615	Fbxl10	30841	5-:123320689-123433155
616	MMT00037897		11+:6142380-6142673
617	MMT00077405		15+:62812180-62813611
618	Trap1a	22037	X+:135868221-135872704
619	Fmnl2	71409	2+:52873836-52992905
620	Prpf31	68988	7+:3581614-3592829
621	Cyp2b13	13089	7+:26846513-26881215
622	Serping1	12258	2-:84605543-84615561
623	Lmf1	76483	17+:25716148-25799668
624	4931406I20Rik	66743	4-:116944531-117048062
625	Hand2	15111	8+:59800703-59802656
626	D19Ertd386e	52013	19+:42636995-42665857
627	Eif1a	13664	18+:46767353-46767788
628	Ccdc55	237859	11-:76858644-76951747
629	Glipr1l3	544736	10-:111595623-111597283
630	C1s	50908	6-:124480362-124492299
631	Arrdc5	76920	17-:56433532-56439666
632	Eif1ay	66235	X+:155810139-155823643
633	Ap1b1	11764	11+:4886971-4942732
634	Usp16	74112	16+:87455300-87483756
635	MMT00008587		2+:147493729-147494129
636	2310006M14Rik	76422	1-:138078229-138083385
637	Cdh23	22295	10-:59766122-60120081
638	MMT00061457		4-:144136897-144138532
639	Hpcal1	53602	12+:17697631-17798732
640	MMT00065211		14+:14734306-14735766
641	Chmp4c	66371	3+:10366972-10391003
642	Bcas3	192197	11+:85615032-85639559
643	Spata2	263876	2-:167306893-167311025
644	Cd160	54215	3-:96604163-96618415
645	MMT00040509		X-:14532347-14533004
646	Zfp346	26919	13+:55206671-55236177
647	Rab13	68328	3+:90024738-90029885
648	Map2k7	26400	8+:4250465-4251310
649	Pop7	74097	5-:137942666-137943657
650	MMT00061138		X+:99850965-99852402
651	MMT00056891		X-:71749544-71750596
652	Veph1	72789	3-:65860558-65940360
653	Nipsnap3a	66536	4+:53024795-53034929
654	Rassf5	54354	1-:133073356-133141736
655	Tssk4	71099	14+:56269020-56271368
656	Eif2c5	286951	X+:137332907-137334477
657	Fyn	14360	10+:39089891-39285178
658	Vmn2r-ps88	209786	8+:3093456-3104954
659	Bace1	23821	9+:45646681-45670302
660	Slit2	20563	5+:48561392-48653904
661	Siglec1	20612	2-:130894478-130912501

662	Mid1ip1	68041	X+:10294715-10296811
663	Muc19	239611	15+:91702790-91736981
664	Mrpl23	19935	7+:149719021-149726647
665	Slc26a1	231583	5-:109098950-109110090
666	Lzts1	211134	8-:71658739-71664852
667	Olfr399	259006	11-:73867323-73868259
668	Olfr802	258934	10-:129118855-129119793
669	MMT00014769		19+:32146396-32149418
670	MMT00049472		2+:104377593-104378614
671	Coro7	78885	16-:4626882-4679687
672	Calcrl	54598	2-:84170790-84217144
673	Tchh	99681	3+:93246251-93253000
674	Ptgdr	19214	14-:45472900-45478928
675	Xrn2	24128	2+:146838857-146900814
676	Ms4a4d	66607	19+:11611290-11632952
677	Ace2	70008	X+:160578419-160626353
678	Ncaph	215387	2-:126929566-126959641
679	Lrpprc	72416	17-:85104590-85189855
680	Loxl4	67573	19-:42669824-42683021
681	Hcn1	15165	13+:118391506-118765609
682	Klrb1c	17059	6-:128728504-128738598
683	Olfr1450	258368	19+:13028080-13029056
684	1700102P08Rik	112418	9+:108295164-108300094
685	Pex7	18634	10-:19580330-19627455
686	Lalba	16770	15-:98310831-98313114
687	MMT00052624		3-:77366866-77367792
688	MMT00020896		
689	2010004A03Rik	76527	8-:113264997-113329776
690	Lrrc9	78257	12+:73600566-73611545
691	Vpreb2	22363	16+:17980657-17981173
692	Pelo	105083	13-:115878571-115881033
693	Vps13d	230895	4-:144564695-144752031
694	Fgd1	14163	X+:147482113-147523544
695	EG232930	232930	7-:19040829-19042079
696	MMT00037949		16+:38927463-38927965
697	Ldb2	16826	5-:44863931-45190858
698	MMT00071792		7+:50410632-50410682
699	Nsl1	381318	1+:192886892-192906324
700	4930529C04Rik	619677	3+:90875583-90880395
701	Polr3g	67486	13-:81812841-81849966
702	MMT00046441		8-:76273392-76274717
703	Spryd4	66701	10-:127646969-127648855
704	Elac1	114615	18-:73898191-73914133
705	MMT00059766		
706	Rad9	19367	19-:4195197-4201603
707	Bdnf	12064	2+:109532875-109564409
708	Ophn1	94190	X-:95753590-96086120
709	Pparg	19016	6+:115372090-115440418
710	MMT00001859	329584	2+:181120264-181121750
711	Cacnb3	12297	15+:98465566-98474960
712	Foxp4	74123	17-:48004081-48061392

713	Psap	19156	10+:59740465-59764739
714	Igk-V8	384422	6+:68071041-68387930
715	Padi3	18601	4-:140341283-140366563
716	Dbpht1	20749	15+:64648686-64649468
717	Xpo1	103573	11+:23184334-23196903
718	Pnkd	56695	1+:74331500-74334030
719	Usp2	53376	9+:43897214-43901997
720	Cryba1	12957	11-:77532233-77538795
721	Cenpj	219103	14-:57145272-57190653
722	MMT00013072		5-:87830341-87831925
723	4931433A01Rik	78933	4+:111199188-111239615
724	MMT00024988		4-:143048875-143050393
725	BC005561	243171	5+:104946438-104951890
726	Phkb	102093	8+:88366929-88420438
727	Ywhae	22627	11+:75546501-75579305
728	Tnfrsf1a	21937	6+:125300041-125312010
729	1700011I03Rik	75444	18+:57693431-57890719
730	Olfr894	258868	9+:38026409-38027350
731	Lin37	75660	7-:31340459-31344665
732	Gja8	14616	3-:96722945-96724267
733	4930404H21Rik	73808	7-:140778705-140793842
734	Lrrc50	68270	8+:122099134-122122354
735	Wnt3	22415	11+:103635537-103679271
736	Gm996	381353	2-:25433591-25435782
737	MMT00023716		
738	Rbp4	19662	19-:38191112-38199804
739	Ric8b	237422	10+:84432607-84479185
740	Txnrd3	232223	6+:89593988-89625522
741	OTTMUSG000000	665998	11-:99647104-99647344
742	1110006O17Rik	68545	18-:35873293-35882009
743	Ttc24	214191	3-:87876878-87878576
744	C230081A13Rik	244895	9-:56054256-56075197
745	Abca17	381072	17-:24415992-24465733
746	1700028J19Rik	70004	7-:51485301-51491494
747	7420416P09Rik	432677	12+:85989427-85991649
748	Hs3st3a1	15478	11+:64249485-64334321
749	Mex3c	240396	18+:73733247-73752224
750	Det1	76375	7-:85978992-85992125
751	Vmn2r58	628422	7-:49092475-49120704
752	Sema7a	20361	9+:57787952-57810013
753	9330198N18Rik	77516	2+:30496891-30543865
754	Axin1	12005	17+:26279560-26332754
755	Ppp1r13l	333654	7+:19946374-19963890
756	Pou1f1	18736	16+:65520846-65535866
757	Trip12	14897	1-:84720878-84755603
758	V1rh12	171271	13-:23275497-23276393
759	Bbx	70508	16-:50197274-50432494
760	Akr1c19	432720	13+:4227945-4247604
761	Amigo1	229715	3+:107989267-107994694
762	Brms1	107392	19+:5041403-5049917
763	4933406E20Rik	74443	9-:108481213-108499902

764	A830035A12Rik	442842	11-:107393070-107409448
765	Olfr1134	259032	2-:87496138-87497076
766	Tcf12	21406	9-:71693559-71959426
767	Ccdc93	70829	1+:123327671-123346328
768	Samd1	666704	8+:86522443-86524316
769	5430437P03Rik	68251	8+:73920759-73928517
770	Ndufs8	225887	19-:3908867-3912735
771	Zbtb45	232879	7-:13591013-13593679
772	Hnrnpr	74326	4+:135866911-135896587
773	Gm514	208080	9+:65208859-65228182
774	Bcl10	12042	3+:145587341-145597247
775	Rps27l	67941	9+:66793924-66797316
776	Irx6	64379	8+:95198923-95203963
777	EG546052	546052	8-:33583843-33587801
778	Galnt3	14425	2-:65921491-65962686
779	Acbd3	170760	1+:182655890-182684334
780	Cish	12700	9+:107199182-107204295
781	Mvk	17855	5+:114895392-114910220
782	X76971	634322	14+:53698137-53699995
783	Ccdc66	320234	14-:28310524-28319907
784	Actg1	11465	11-:120208395-120209809
785	MMT00034119		5+:78903282-78911968
786	Macrod1	107227	19+:7131532-7272547
787	Zfp532	328977	18+:65662270-65849088
788	2310042D19Rik	74183	4+:155587425-155589167
789	MMT00023104		12-:99337184-99338413
790	MMT00026414	83701	5-:137737059-137748395
791	Il18r1	16182	1+:40522850-40557678
792	Gyg	27357	3-:20021969-20054937
793	Morc3	338467	16+:93832534-93876317
794	Mrps14	64659	1+:162125378-162130567
795	Snx27	76742	3-:94301464-94386471
796	Olfr830	258559	9+:18679772-18680719
797	Gpi1	14751	7-:34987176-35015281
798	Tbccd1	70573	16-:22813292-22844715
799	Pigp	56176	16-:94586059-94591801
800	Nlrp9b	243874	7+:20593370-20648290
801	Olfr295	258850	7+:93733786-93734714
802	Src	20779	2+:157282820-157295697
803	Lhb	16866	7+:52676322-52677147
804	2210407C18Rik	78354	11-:58421710-58426972
805	Reep1	52250	6+:71657831-71760532
806	Ly96	17087	1+:16678536-16699686
807	MMT00002637		6-:46728598-46789042
808	Ctsc	13032	7+:95426644-95458806
809	Olfr1416	259040	1-:94376257-94377196
810	Zzef1	195018	11+:72666660-72740612
811	Pik3r5	320207	11+:68287309-68309522
812	En2	13799	5+:28493019-28498710
813	Nkd2	72293	13-:73958024-73985029
814	Gpr64	237175	X+:156828597-156935996

815	Hk1	15275	10-:61732609-61842631
816	MMT00081658		X-:43889040-43889672
817	C130039O16Rik	238317	12-:85490239-85500005
818	Ppp2r3a	19054	9_random-:49190-55020
819	Vamp2	22318	11+:68902067-68904203
820	3200002M19Rik	75430	7+:109044930-109048057
821	Dcun1d5	76863	9+:7184515-7207045
822	Ddah1	69219	3+:145421689-145557242
823	Mnd1	76915	3-:83891856-83945694
824	Esco1	77805	18-:10566600-10594053
825	Ccl25	20300	8+:4349614-4360019
826	Fezf1	73191	6-:23195050-23198148
827	Flcn	216805	11-:59605254-59623275
828	Flnc	68794	6+:29409404-29411881
829	Prodh2	56189	7+:31278702-31297999
830	Socs1	12703	16-:10783903-10785602
831	Odz1	23963	X-:39885576-40627963
832	Vsig2	57276	9+:37346959-37351726
833	9930013L23Rik	80982	7-:91083298-91092849
834	Zfp472	224691	17+:33102786-33242747
835	Dhh	13363	15-:98724366-98728703
836	Rai12	54351	11-:69781729-69794012
837	Rabl5	67286	5+:137384063-137389113
838	MMT00011949		19-:12219977-12241545
839	Gm766	330440	6-:142734827-142762438
840	Olfr49	18348	14-:54901630-54902568
841	Gm53	193022	11+:96113002-96126108
842	Slc29a2	13340	19+:5026105-5031964
843	Epyc	13516	10+:97110069-97144317
844	Rad23a	19358	8-:87359459-87364464
845	Myo5a	17918	9+:74919406-75067280
846	Hes1	15205	16+:30065676-30067513
847	S100a7a	381493	3+:90458166-90461748
848	MMT00008599		8+:53859486-53860062
849	Pxmp4	59038	2-:154413323-154429398
850	Mrps30	59054	13-:119168630-119176062
851	MMT00024065		7-:70955713-70956239
852	Hsf2bp	74377	17-:32081714-32171439
853	Crct1	74175	3-:92818128-92819608
854	EG432448	432448	10+:30967936-30970683
855	Serpinb13	241196	1+:108878731-108897772
856	MMT00026922		
857	Sfrs12	218543	13-:104533165-104564597
858	MMT00038787		X+:138414953-138416864
859	AA467197	433470	2+:122463557-122466925
860	Psmb10	19171	8-:108459663-108462005
861	Hif3a	53417	7-:17621082-17642028
862	Ms4a4b	60361	19+:11518090-11538036
863	Dnajc14	74330	10+:128242731-128254559
864	MMT00031865	329122	1+:37992371-38006685
865	Npc1	18145	18-:12348370-12394867

866	Zfp369	170936	13+:65380235-65398890
867	Pag1	94212	3-:9693766-9706213
868	Lrrc9	78257	12+:73542919-73598226
869	C330007P06Rik	77644	X-:34391096-34404197
870	Rint1	72772	5+:23293529-23327653
871	Mrps25	64658	6-:92124115-92133977
872	MMT00004441		4+:76573197-76573933
873	Slc22a3	20519	17-:12612841-12700570
874	Aldh1a7	26358	19-:20767442-20802046
875	Gm50	215090	4-:124531960-124537692
876	Purg	75029	8+:34496915-34545314
877	MMT00015473		19+:43618672-43619815
878	MMT00021302		12+:88860673-88861267
879	Plekhn1	231002	4-:155595564-155597700
880	Atp6v0a2	21871	5+:125079736-125202515
881	Adi1	104923	12+:29360071-29366504
882	Olfr381	259024	11-:73299390-73300324
883	MMT00033758		15+:13353850-13382866
884	Kif26b	269152	1+:180751613-180862984
885	2210010L05Rik	98682	1-:52714300-52766004
886	Slc35a5	74102	16-:45140924-45158713
887	MMT00078651		2+:181631355-181633754
888	Col25a1	77018	3+:129884134-130298050
889	Ogdh	18293	11+:6191570-6256788
890	Tmem61	329909	4-:106171420-106175696
891	Lmbrd2	320506	15+:9070303-9127238
892	Nfix	18032	8-:87232512-87324039
893	Cdc42bpg	240505	19+:6322553-6325656
894	Ddit4	74747	10-:59412422-59414518
895	Ccdc57	71276	11-:120780652-120783276
896	Prpf39	328110	12+:66137349-66164372
897	Mut	17850	17+:41073992-41098598
898	Txndc3	73412	13-:19752366-19767699
899	Gramd2	546134	9+:59559877-59564231
900	Top1	21969	2+:160471965-160548447
901	Ndufs3	68349	2-:90734784-90744984
902	Slc30a1	22782	1+:193730738-193733694
903	Gm106	226866	1-:15845775-15882757
904	MMT00003579		14-:88179628-88179937
905	Fyco1	17281	9-:123698627-123761010
906	Olfr556	258749	7+:109818435-109819409
907	Wbscr17	212996	5-:131352019-131782679
908	Lypd2	68311	15-:74562676-74564745
909	Lphn1	330814	8+:86439298-86454279
910	Sned1	208777	1+:95192288-95194148
911	Ngfr	18053	11-:95432077-95448870
912	Slc30a5	69048	13+:101603172-101604342
913	MMT00019999		11+:21853142-21853492
914	Itga3	16400	11-:94907794-94937650
915	9130206N08Rik	103406	10+:80702678-80713479
916	Socs4	67296	14+:47909284-47910594

917	Kal1	14038	11+:83522607-83524842
918	Rpa2	19891	4+:132324302-132334356
919	MMT00041659		10+:77946936-77961004
920	A230107C01Rik	320225	7-:29966548-29969768
921	Rspry1	67610	8+:97163546-97183981
922	MMT00059556		5+:26681977-26683672
923	MMT00033681		14-:120043166-120048850
924	Olfr809	258321	10+:129212926-129213912
925	6030458C11Rik	77877	15-:12739800-12754403
926	MMT00005746		18-:13265801-13392930
927	Olfr113	258286	17-:37711427-37712366
928	Ccr4	12773	9-:114400742-114405662
929	Dusp4	319520	8+:35870424-35885009
930	Cd55	13136	1-:132336790-132359261
931	Ccdc147	381229	19+:48011400-48129383
932	Chad	12643	11+:94426362-94430433
933	Stra6	20897	9+:57977155-58001760
934	Rara	19401	11+:98799173-98833984
935	MMT00060809		13+:62880141-62880565
936	Dnajc21	78244	15-:10376516-10400116
937	Pi4ka	224020	16-:17280441-17405919
938	Sdcbp	53378	4+:6292867-6322797
939	Daxx	13163	17+:34047373-34052463
940	Pramel3	83565	X+:131836850-131847175
941	MMT00010305		17+:57554875-57555404
942	Kcna5	16493	6-:126482570-126485455
943	1700106J16Rik	74297	11+:88107597-88108055
944	Sla2	77799	2-:156699887-156712913
945	Shprh	268281	10+:10869301-10877121
946	Dnajc5	13002	2+:181281086-181284079
947	Bud31	231889	5+:145901273-145908753
948	Twf1	19230	15-:94410159-94420259
949	Plekha6	240753	1+:135155585-135200013
950	Ulk3	71742	9+:57437250-57442656
951	Prkcb1	18751	7+:129432810-129772169
952	1810008K16Rik	67366	5-:109206253-109224382
953	Wnt16	93735	6+:22238506-22248229
954	Adam25	23793	8+:41837561-41841529
955	Pigq	14755	17-:26063372-26078889
956	EG547447	547447	X+:67766158-67773531
957	Hras1	15461	7-:148377247-148378839
958	Ppp2r5e	26932	12-:76550450-76694320
959	Cckar	12425	5-:54089726-54099341
960	2410012M07Rik	71979	9+:98756998-98766998
961	Rwdd4a	192174	8+:48619008-48638304
962	Klhl10	66720	11+:100303237-100318336
963	Lrrfip2	71268	9+:111063805-111128172
964	Rpl23	65019	11-:97639434-97643681
965	Vmn2r2	100125586	3-:63920353-63944465
966	Wif1	24117	10+:120471285-120537698
967	Matr3	17184	18+:35747218-35751870

968	Atp13a1	170759	8+:72315132-72331643
969	MMT00054728		10+:78006018-78006876
970	1700021O21Rik	75536	5+:39127219-39139136
971	Prdm14	383491	1-:13104340-13115916
972	Sri	109552	5+:8046077-8067854
973	Cilp2	68709	8-:72408185-72411250
974	Tgm4	331046	9+:122949489-122950710
975	Nt5c	50773	11-:115351748-115353128
976	Cdcp1	109332	9-:123082515-123125148
977	Npal2	223473	15-:34504691-34608474
978	Cd244	18106	1+:173503811-173507478
979	Cep164	214552	9-:45600266-45602672
980	BC067074	408066	13+:114107291-114159738
981	1110015M06Rik	68511	12-:29209539-29215352
982	Ccl7	20306	11+:81859233-81861023
983	MMT00078030		13-:91400536-91402025
984	Derl3	70377	10+:75356142-75358685
985	Hsd3b6	15497	3-:98609427-98615031
986	Plekhg3	263406	12+:77634569-77679454
987	Zfp202	80902	9+:39999910-40020773
988	Olfr491	258731	7+:115460409-115461342
989	Phyhd1	227696	2+:30121720-30137667
990	Fras1	231470	5+:97164216-97169877
991	MMT00073000		8+:98101360-98102813
992	8430410A17Rik	232210	6+:87863943-87886618
993	Rbm22	66810	18+:60719976-60732464
994	Arfrp1	76688	2-:181094040-181100056
995	MMT00082379		10+:24773293-24774049
996	Ppbp	57349	5+:91197543-91199086
997	Olfr508	258769	7+:115773507-115774438
998	MMT00075117		12+:37265916-37266514
999	Gm527	217648	12+:66019160-66025579
1000	2900024O10Rik	72852	13+:81850045-81890207
1001	MMT00054937	194960	11+:101800814-101801213
1002	Chrm1	12669	19+:8752422-8753803
1003	Ext1	14042	15-:52899814-52939422
1004	MMT00042857		7+:87574990-87575805
1005	Tigd2	68140	6+:59160143-59161719
1006	2600011E07Rik	72125	14+:60997188-60999841
1007	Slc16a12	240638	19-:34746925-34821778
1008	MMT00069490		X+:71732110-71732758
1009	Rps2	16898	17+:24857053-24858868
1010	Prr14	233895	7+:134618657-134620271
1011	MMT00007038		15+:52090530-52091480
1012	Il21r	60504	7+:132746990-132777081
1013	Smad6	17130	9-:63801330-63868831
1014	Rtcd1	66368	3-:116191883-116211028
1015	Nuak2	74137	1+:134212715-134230043
1016	Adra1d	11550	2-:131317303-131388063
1017	Dync2li1	213575	17+:85025814-85054927
1018	MMT00071883		

1019	Slc26a3	13487	12+:32123165-32158584
1020	Olfr195	259000	16+:59148677-59149602
1021	Rhpn2	52428	7+:36119332-36177300
1022	6330577E15Rik	67788	19+:47730282-47810077
1023	Ccdc64b	212733	17+:23798726-23805584
1024	Nes	18008	3+:87775050-87784369
1025	Slc26a11	268512	11+:119216905-119238223
1026	Ftsj2	68017	5-:140803627-140807852
1027	Accs	329470	2-:93675574-93690099
1028	Pgam1	18648	19+:41986453-41993149
1029	MMT00043362	669021	1+:60957702-60958272
1030	Fuz	70300	7+:52151461-52155982
1031	Uros	22276	7-:140878038-140900716
1032	MMT00060488		8+:127289089-127290039
1033	Ube2c	68612	2+:164595428-164598402
1034	Olfr1364	258533	13-:21665380-21666323
1035	Rabep1	54189	11+:70658426-70754442
1036	Dusp19	68082	2+:80457201-80471351
1037	Kif26a	668303	12+:113410781-113419957
1038	Ndrg2	29811	14-:52524957-52533026
1039	Zfp207	22680	11+:80196847-80209639
1040	Dnhd1	77505	7+:112826403-112870312
1041	3110049J23Rik	67307	10+:62487259-62521560
1042	Tm7sf4	75766	15+:39577475-39591168
1043	Tbx10	109575	19+:3996644-3999424
1044	Adamts19	240322	18+:59170656-59212295
1045	MMT00053214		9-:100525392-100543661
1046	MMT00005405		7+:12029339-12030685
1047	4932413O14Rik	320696	5-:93082527-93091278
1048	MMT00026905		
1049	Myst1	67773	7+:135056030-135069276
1050	MMT00044489		4-:59707420-59707804
1051	MMT00065456	100041868	1+:100272681-100356563
1052	Cog4	102339	8+:113370946-113406124
1053	Wdsof1	223499	15+:38944455-38978406
1054	Tm4sf5	75604	11+:70318778-70324674
1055	Gm525	217071	11+:88934958-88954377
1056	Slc17a4	319848	13-:23989637-24006874
1057	Ap3m2	64933	8-:23897826-23916099
1058	Olfr868	258552	9+:19905204-19906133
1059	P2rx6	18440	16+:17562092-17571358
1060	6030419C18Rik	319477	9+:58337474-58347473
1061	Krt20	66809	11-:99289716-99299467
1062	Npnt	114249	3-:132547393-132613007
1063	Ntf5	78405	7+:52669032-52672023
1064	MMT00058736	76652	3-:30496756-30497631
1065	Hmgcr	15357	13-:97418921-97440804
1066	MMT00081797		1+:12106050-12111666
1067	Tmem50b	77975	16-:91575938-91597930
1068	1700129I15Rik	78478	X-:132253204-132255213
1069	Cic	71722	7+:26056738-26058720

1070	Cdr2l	237988	11+:115243231-115255778
1071	Smcr7l	239555	15+:80064534-80082835
1072	EG624855	624855	8+:87695403-87696165
1073	Ppp1r7	66385	1+:95240221-95264195
1074	Acsm3	20216	7+:126904456-126928403
1075	AI894139	101197	6+:47893204-47927924
1076	Megf11	214058	9+:64345146-64554489
1077	Ap1g1	11765	8+:112326849-112384094
1078	Ccdc14	239839	16+:34709608-34725287
1079	Hr	15460	14+:70954578-70972201
1080	Commd8	27784	5-:72549959-72559419
1081	Gm608	207806	16+:44199014-44222174
1082	H2afj	232440	6+:136755603-136757249
1083	Ddx3y	26900	Y-:597157-623024
1084	Pros1	19128	16+:62854153-62929179
1085	Col5a2	12832	1-:45432930-45559759
1086	2610205E22Rik	66617	2+:30663460-30679260
1087	Dusp12	80915	1-:172804319-172815593
1088	Lef1	16842	3+:130813250-130927276
1089	1700010D01Rik	76386	X-:92927928-92928600
1090	5730409G15Rik	70511	16-:5244523-5256050
1091	Acsl5	433256	19+:55328144-55371117
1092	MMT00024026	554292	15+:100183640-100214386
1093	MMT00012906		3-:78695787-78696346
1094	Cul5	75717	9-:53425801-53475204
1095	Olfr798	258549	10-:129062181-129063115
1096	Rhd	19746	4+:134420515-134451893
1097	Dctn6	22428	8-:35153477-35171497
1098	2810408M09Rik	381406	2-:165316542-165318823
1099	Ap1m1	11767	8+:74764030-74781278
1100	Utrn	22288	10-:12101613-12588943
1101	Sec11a	56529	7-:88060264-88092436
1102	Abhd14b	76491	9+:106351016-106355246
1103	Lix1l	280411	3+:96405035-96428737
1104	Yars2	70120	16+:16303055-16309720
1105	Crls1	66586	2+:132672401-132692502
1106	Hira	15260	16+:18877362-18935165
1107	Tspyl5	239364	15-:33523824-33617552
1108	MMT00067207		14+:56868857-56870107
1109	Snai1	20613	2+:167363775-167368309
1110	MMT00014191		19+:25568325-25569189
1111	Mll5	69188	5+:22938111-23009638
1112	Ccdc56	52469	11-:101139283-101140262
1113	Higd1b	75689	11+:102697609-102699351
1114	Cdca7l	217946	12+:119082333-119117178
1115	Rbbp7	245688	X+:159206779-159216559
1116	MMT00007297		8+:78817822-78819090
1117	MMT00068790		15-:34087703-34087843
1118	Diras1	208666	10-:80484564-80485160
1119	Exosc6	72544	8+:113580270-113580843
1120	Rbpms2	71973	9+:65477454-65508333

1121	Fusip1	14105	4+:135412073-135425812
1122	Pax3	18505	1-:78099278-78193621
1123	Ahsa2	268390	11-:23373213-23397943
1124	Strbp	20744	2-:37338752-37502739
1125	Atrx	22589	X-:102995491-103124681
1126	Slc25a14	20523	X+:45976754-46015469
1127	Pomt2	217734	12-:88488120-88488849
1128	Amtn	71421	5+:88805132-88814941
1129	Gsta4	14860	9+:78039838-78057155
1130	Ly75	17076	2-:60130160-60221315
1131	Itgb7	16421	15-:102046428-102062317
1132	Mutyh	70603	4+:116486929-116491984
1133	MMT00012299		13+:18051600-18053324
1134	Vmn2r19	232358	6+:123259676-123280222
1135	Akap12	83397	10-:5987069-6080163
1136	Kdelr3	105785	15+:79346897-79358168
1137	Mlxip	208104	5+:123893038-123903952
1138	Gabrg2	14406	11-:41725358-41814180
1139	MMT00053597		12-:11767222-11767700
1140	Rmnd5a	68477	6-:71338631-71390133
1141	Oxr1	170719	15+:41621060-41692591
1142	MMT00065065	214854	1-:36323205-36330256
1143	Olfr1049	259018	2-:86094921-86095848
1144	MMT00060803		11+:32084994-32085702
1145	Arhgap6	11856	X+:165233904-165742367
1146	Akap4	11643	X+:6645368-6655543
1147	Add3	27360	19+:53291288-53319906
1148	Pak1	18479	7+:104991479-105059842
1149	MMT00052327		11+:54542178-54547974
1150	Accn1	11418	11-:81777432-81781686
1151	Egln1	112405	8-:127452359-127473724
1152	Ralbp1	19765	17-:66197782-66234165
1153	Abcc10	224814	17-:46440369-46462715
1154	Atp7b	11979	8-:23104819-23170548
1155	MMT00015484		X+:124796305-124797382
1156	Rbj	217378	12+:4082556-4110603
1157	Dpt	56429	1+:166726862-166754397
1158	Olfr206	258993	16-:59344606-59345525
1159	Tmem104	320534	11+:115048841-115108336
1160	Obscn	380698	11-:58876966-58904285
1161	4930419G24Rik	73874	3+:32954619-32987529
1162	Cpsf4	54188	5+:145928113-145942911
1163	Olfr52	18352	2-:86021307-86022266
1164	A930011O12Rik	268755	14+:65209109-65212802
1165	Pik3cd	18707	4-:149024748-149037393
1166	Il21	60505	3-:37121684-37131540
1167	Atg2a	329015	19+:6241637-6262304
1168	Ulk1	22241	5-:111214747-111238794
1169	Centg3	213990	5+:23957994-24007865
1170	Faim3	69169	1+:132762337-132777421
1171	Tmem106a	217203	11+:101443814-101452227

1172	MMT00014144		13+:39852661-39853335
1173	Rbpj	19664	5+:54033326-54044857
1174	Sgce	20392	6-:4624361-4697060
1175	Tktl1	83553	X+:71422622-71453828
1176	MMT00046049		1+:169070214-169071790
1177	EG545391	545391	1+:180034087-180086933
1178	Atp5h	71679	11-:115277062-115281254
1179	Ncdn	26562	4-:126422079-126431010
1180	Clec4a3	73149	6+:122902683-122919897
1181	Pkia	18767	3+:7437335-7444106
1182	MMT00021420		2-:178755194-178755764
1183	MMT00009390		
1184	Pqlc3	217430	12-:17004421-17006862
1185	Atxn2	20239	5+:122161617-122264717
1186	Hadhb	231086	5+:30481861-30511122
1187	MMT00075773		19+:24930589-24930857
1188	MMT00077843		12+:52398473-52398948
1189	AI314180	230249	4-:58811784-58924796
1190	Sema3g	218877	14+:32033744-32041392
1191	MMT00009498		1+:64196744-64197237
1192	Btk	12229	X-:131076922-131117655
1193	MMT00014243		3-:13838740-13839769
1194	Per3	18628	4-:150378820-150418698
1195	Gip	14607	11+:95886684-95892124
1196	MMT00014158	100043240	1+:148573286-148574672
1197	Flvcr1	226844	1-:192835066-192850004
1198	4933429F08Rik	328967	18-:61659358-61677796
1199	Foxb1	64290	9-:69607076-69608053
1200	Grm3	108069	5-:9485239-9589959
1201	Ptger3	19218	3+:157229863-157307720
1202	Apbb2	11787	5-:66782860-67010042
1203	Enah	13800	1-:183835645-183949792
1204	Pacrg	69310	17-:10783579-10823998
1205	Rhox4b	57737	X+:34847020-34851785
1206	BC011487	232748	6-:42573041-42595040
1207	4930444A02Rik	74653	8-:27092790-27104585
1208	Pnrc2	52830	4-:135426843-135429742
1209	MMT00047482		1-:133529084-133550537
1210	MMT00011015		6+:133682134-133682248
1211	Dpp6	13483	5-:28053379-28054040
1212	Adora3	11542	3+:105707340-105711846
1213	Stoml3	229277	3+:53302045-53311573
1214	Trip13	69716	13-:74049907-74075187
1215	Zfp280c	208968	X-:45895791-45945315
1216	Btnl7	195349	17-:34670342-34684596
1217	MMT00008964		6+:131830700-131830893
1218	Ms4a5	269063	19-:11351097-11358267
1219	EG665503	665503	13-:62629440-62630551
1220	D12Ertd553e	76820	12+:12283107-12380920
1221	Matk	17179	10+:80720289-80725726
1222	2310061F22Rik	66965	8+:124992597-125006021

1223	Slc30a9	109108	5+:67698199-67747022
1224	Nup210	54563	6-:90963063-91066788
1225	Rpl10a	19896	17+:28465543-28467886
1226	Ccdc14	239839	16+:34697352-34706687
1227	4933421I07Rik	71162	7-:49700831-49703450
1228	Rabgap1	227800	2+:37396654-37421970
1229	MMT00072551		X+:12879365-12881348
1230	MMT00057236	630663	13-:13413093-13415645
1231	Dbn1	56320	13-:55575376-55589427
1232	Abhd1	57742	5+:31252444-31257456
1233	Sidt1	320007	16-:44240015-44333081
1234	Fbxl2	72179	9-:113887057-113927169
1235	Cpne9	211232	6+:113232298-113254819
1236	Ccdc113	244608	8+:98060363-98082788
1237	Irf3	54131	7+:52253043-52257759
1238	Habp2	226243	19+:56362427-56394578
1239	Cerkl	228094	2-:79208838-79269162
1240	Ddx24	27225	12-:104646190-104664057
1241	Meox1	17285	11-:101738837-101755623
1242	Grin2b	14812	6-:135682116-135994319
1243	Phf3	213109	1-:30859185-30930877
1244	Tmem150	232086	6+:72305486-72309756
1245	MMT00029756		16-:40400621-40400995
1246	EG434121	434121	7-:14495025-14574937
1247	Thsd7b	210417	1+:131492038-131848145
1248	Usp24	329908	4+:106032818-106046647
1249	Ncapd3	78658	9+:26837760-26903085
1250	Nol6	230082	4-:41061460-41071321
1251	Lgi1	56839	19+:38339276-38381298
1252	Tacc2	57752	7+:137765094-137815172
1253	Scel	64929	14+:103912558-104012389
1254	Lum	17022	10+:97028463-97035337
1255	Grhpr	76238	4+:44994282-45003567
1256	Baiap2l2	207495	15-:79088628-79115110
1257	Slc25a45	107375	19+:5877841-5885763
1258	Hba-a1	15122	11+:32183673-32184492
1259	Zdhhc14	224454	17+:5731540-5753810
1260	Islr2	320563	9-:58045540-58052127
1261	4932418E24Rik	329366	2-:26127166-26131627
1262	Gnpda1	26384	18-:38487211-38498605
1263	Folh1	53320	7-:93867906-93924342
1264	1110004E09Rik	68001	16-:90926056-90935185
1265	Spns2	216892	11-:72264692-72303418
1266	Olfr1246	258788	2-:89430326-89431270
1267	Olfr1404	258881	1+:175145783-175146723
1268	Cyb5r1	72017	1+:136302566-136308315
1269	Paqr8	74229	1+:20912332-20927188
1270	1700029I01Rik	70005	4+:146506142-146554953
1271	2810403A07Rik	74200	3+:88489739-88516844
1272	1110008P14Rik	73737	2-:32234623-32237433
1273	MMT00067831		9+:14136348-14137544

1274	Kbtbd8	243574	6+:95068516-95076932
1275	Olfr1054	259021	2-:86172574-86173511
1276	Col4a1	12826	8-:11198421-11312621
1277	Brsk1	381979	7+:4644481-4667091
1278	4930430D24Rik	194735	X-:35367086-35414160
1279	Ctps2	55936	X+:159340115-159469249
1280	Mesdc2	67943	7+:91040533-91047690
1281	Alkbh3	69113	2-:93820708-93850369
1282	Gm628	268816	15-:73591839-73594313
1283	Tmem161a	234371	8+:72696317-72706504
1284	Serpina3h	546546	12+:105452765-105492619
1285	MMT00008393		5-:10236233-10236975
1286	Rad51c	114714	11-:87192888-87217923
1287	Acss3	380660	10-:106441719-106560639
1288	3110057O12Rik	269423	3+:40698181-40738951
1289	Gm94	225443	18-:43936871-43952534
1290	Zscan12	22758	13+:21455153-21461693
1291	Sft2d1	106489	17+:8504012-8520305
1292	Plekhg2	101497	7-:29144622-29157596
1293	Safb	224903	17+:56724245-56745715
1294	MMT00016640		7+:12194645-12196115
1295	MMT00014481		15+:60299620-60301359
1296	5330401P04Rik	227094	1+:52694208-52708763
1297	Phox2a	11859	7+:108967003-108971238
1298	Trim41	211007	11-:48619905-48622632
1299	Chst9	71367	18-:15610432-15656679
1300	Eef1d	66656	15-:75725921-75739736
1301	A130092J06Rik	241303	2-:31924655-31938797
1302	A530098C11Rik	433294	1-:44066253-44076852
1303	Nup43	69912	10+:7387313-7398675
1304	H2afy	26914	13-:56174981-56236881
1305	4930485B16Rik	380654	10-:92507829-92510823
1306	Degs2	70059	12-:109928437-109940516
1307	Tmem116	77462	5+:121913205-121945429
1308	Cwf19l2	244672	9+:3430834-3479305
1309	Defb20	319579	2+:152293129-152305669
1310	Cabc1	67426	1-:182096745-182123597
1311	Mpp4	227157	1-:59177778-59215512
1312	MMT00051556		X-:137356725-137392567
1313	BC063749	414801	19-:47861069-47993777
1314	Tas2r137	574417	6+:40441357-40442226
1315	Tmem16j	71345	7-:148287124-148303661
1316	Cd28	12487	1+:60803262-60827406
1317	Fcgrt	14132	7-:52348433-52358462
1318	Alox12	11684	11-:70055887-70068774
1319	Vmn2r115	638102	17+:23481014-23483439
1320	Erh	13877	12-:81735023-81744777
1321	AW548124	106522	17+:83615139-83624400
1322	Cep290	216274	10+:100005050-100036288
1323	Orc2l	18393	1-:58520039-58561763
1324	Rfc5	72151	5-:117828846-117839045

1325	Pigu	228812	2-:155103980-155127101
1326	Tox	252838	4-:6615494-6917657
1327	Pitpnc1	71795	11-:107073419-107332013
1328	4922501L14Rik	71845	3+:154383062-154396500
1329	5730494N06Rik	70612	2-:132065229-132073518
1330	Fgf21	56636	7-:52869390-52870676
1331	Map2k6	26399	11+:110260433-110375009
1332	Esrrb	26380	12+:87811066-87860014
1333	Ubqln2	54609	X+:149932773-149935068
1334	Amacr	17117	15+:10911604-10925359
1335	Gria4	14802	9-:4420119-4795519
1336	MMT00069537		14-:61214340-61215729
1337	Pigf	18701	17-:87396598-87424741
1338	Ebag9	55960	15+:44456003-44471966
1339	MMT00077775		19-:26308024-26629706
1340	Tap2	21355	17+:34342239-34353037
1341	MMT00009551		1+:71068738-71074455
1342	Pik3ca	18706	3+:32335127-32361752
1343	Ankhd1	108857	18+:36734672-36749235
1344	Marcks	17118	10-:36854626-36858720
1345	MMT00024601		
1346	MMT00068311		10+:59552212-59552735
1347	Espnl	227357	1+:93218709-93242311
1348	Prl3a1	67000	13+:27351357-27368529
1349	Defb13	246083	8+:23057233-23059093
1350	MMT00064364		1+:166392507-166394121
1351	Whsc1l1	234135	8+:26712042-26825166
1352	Olfr1269	258339	2-:89958823-89959753
1353	Plcg2	234779	8+:120022087-120159043
1354	Lsmd1	78304	11+:69209260-69210703
1355	MMT00047341		1-:28858362-28859394
1356	MMT00045517		Y-:1317414-1317910
1357	Pabpn1	54196	14+:55512992-55516891
1358	Pappa2	23850	1-:160644843-160887569
1359	4931406C07Rik	70984	9-:15087782-15106000
1360	MMT00044338		17+:19290585-19313713
1361	2010001M06Rik	69812	5-:135487979-135489124
1362	Avpr1a	54140	10+:121885554-121890509
1363	BC021767	545551	3-:94464511-94474619
1364	Prl8a9	67310	13-:27650021-27656415
1365	Cuta	67675	17-:27074917-27076413
1366	Tcf23	69852	5+:31271049-31279390
1367	MMT00079643		13+:78218648-78239953
1368	Pctp	18559	11-:89845905-89864179
1369	Zswim2	71861	2-:83755235-83781383
1370	MMT00051564		13-:99259316-99259787
1371	1200009I06Rik	74190	12+:112659771-112669393
1372	Wscd1	216881	11+:71564874-71603145
1373	Lin7b	22342	7-:52623261-52625934
1374	Mdn1	100019	4+:32771613-32780612
1375	Strap	20901	6+:137683646-137699610

1376	Mdm2	17246	10-:117127265-117146840
1377	MMT00021279		12+:88820932-88823764
1378	Tcra-V13.1	21507	14+:53621386-53622033
1379	A930041C12Rik	78443	5-:108059270-108062871
1380	Sycp3	20962	10+:87922331-87935980
1381	Txndc9	98258	1-:38044506-38054063
1382	Vmn2r-ps57	110931	7-:49609077-49613364
1383	Bhlhb9	70237	X+:132420530-132425617
1384	MMT00077464	433217	19+:7742776-7743282
1385	Apba1	319924	19+:23967295-24019516
1386	Ppid	67738	3+:79395353-79407570
1387	Adrm1	56436	2+:179906735-179910988
1388	Plcxd3	239318	15+:4466618-4524876
1389	Chrna9	231252	5+:66325995-66368563
1390	AU015836	385493	X+:91214066-91214923
1391	2410089E03Rik	73692	15+:8119104-8132192
1392	Gm1040	433864	5+:29761265-29778014
1393	Klhl2	77113	8-:67218481-67373628
1394	MMT00051501		17+:8005628-8006677
1395	V1rh11	171270	13+:22615919-22616854
1396	Rassf7	66985	7+:148401731-148404555
1397	2900009I07Rik	68034	19-:24550939-24551933
1398	MMT00060205		14+:31215187-31215917
1399	Casc1	320662	6-:145123202-145140214
1400	Fxyd6	59095	9+:45198075-45200786
1401	Klk8	259277	7+:51052946-51059192
1402	Cops4	26891	5+:100947477-100976792
1403	Dak	225913	19-:10666687-10678748
1404	Rp2h	19889	X+:19941782-19975303
1405	MMT00078938		7+:110292103-110293045
1406	Clic3	69454	2+:25312359-25314298
1407	Spata18	73472	5+:74042658-74070728
1408	Hdgfrp2	15193	17+:56219079-56240019
1409	Pbp2	76400	6-:135259146-135260423
1410	Bicc1	83675	10-:70387849-70622371
1411	Tada3l	101206	6-:113316656-113327514
1412	Phf17	269424	3+:41367382-41420773
1413	Il10rb	16155	16+:91406570-91425993
1414	Tsta3	22122	15-:75755113-75760160
1415	Timm9	30056	12-:72224282-72237621
1416	6720460F02Rik	109212	11+:71856011-71860873
1417	1110006O24Rik	66123	5-:116081081-116081825
1418	Aldh3a1	11670	11+:61022246-61031916
1419	Olfr1121	258345	2+:87211690-87212635
1420	4930548H24Rik	67656	5+:31788231-31790633
1421	2310004I24Rik	66358	11-:66851406-66866096
1422	Acadvl	11370	11-:69823686-69828935
1423	Spdya	70891	17+:71876198-71938872
1424	Dapk1	69635	13+:60703790-60864543
1425	ENSMUSG000000	100036526	12-:70471471-70473508
1426	OTTMUSG000000	544810	11-:96183694-96203518

1427	Ahrr	11624	13-:74348565-74429757
1428	Spty2d1	101685	7-:54245769-54263815
1429	Strn4	97387	7+:17401704-17426284
1430	MMT00038864		8-:48717625-48718172
1431	Eif2s3x	26905	X-:91435837-91457980
1432	MMT00040339	632654	12-:115460975-115461465
1433	Deb1	26901	9+:121619503-121622037
1434	Isg20l2	229504	3+:87735517-87743639
1435	Ptges3	56351	10+:127505742-127514045
1436	Nfu1	56748	6+:86959897-86978409
1437	Evc	59056	5-:37690411-37728100
1438	Dclre1b	140917	3-:103606679-103612107
1439	Clcn3	12725	8-:63391781-63461935
1440	Gpr123	52389	7+:147031483-147062962
1441	MMT00001620		X+:9588913-9590834
1442	Slc45a1	242773	4-:149955148-150026242
1443	Znhit2	29805	19+:6061196-6062470
1444	C1r	50909	6+:124469358-124473458
1445	Ssbp2	66970	13+:91600701-91840840
1446	Eno3	13808	11+:70470705-70476012
1447	Naaa	67111	5-:92687286-92707207
1448	Zwilch	68014	9-:63985482-64020715
1449	Chaf1b	110749	16+:93884145-93906351
1450	4930565N06Rik	68306	16-:36880604-36897225
1451	2010300C02Rik	72097	1-:37668520-37694874
1452	Gm1027	381538	4-:106353243-106382321
1453	Gfod2	70575	8-:108240012-108282507
1454	MMT00027959		16-:21841080-21841617
1455	Smek1	68734	12-:102277619-102321144
1456	Cnih3	72978	1+:183282758-183390040
1457	Efr3b	668212	12-:3967102-4038729
1458	Cpsf2	51786	12+:103214197-103241820
1459	AU042671	269700	5+:121770019-121781922
1460	Chaf1a	27221	17+:56179934-56207041
1461	Bach2	12014	4+:32325924-32668174
1462	Rpusd4	71989	9+:35075446-35083543
1463	Rbbp5	213464	1+:134373920-134402243
1464	Taf13	99730	3+:108374616-108384986
1465	Lcn4	16821	2-:26523193-26526802
1466	Ubap2l	74383	3-:89803505-89856403
1467	Cnot1	234594	8-:98244306-98249987
1468	Caprin2	232560	6-:148791006-148843713
1469	Zfp688	69234	7-:134562478-134565536
1470	Kcns3	238076	12-:11098027-11099502
1471	Igfbp7	29817	5-:77778270-77837070
1472	MMT00078790		1+:33157660-33159540
1473	MMT00007831		8+:40369078-40370255
1474	MMT00012859		13-:16061879-16088923
1475	Nip7	66164	8+:109580776-109583145
1476	2610020H08Rik	434234	7+:126937638-126992452
1477	Nfyb	18045	10-:82212768-82226882

1478	Ptpn12	19248	5-:20493251-20561565
1479	Yipf2	74766	9-:21393126-21397190
1480	Plekhh3	217198	11-:101023995-101032616
1481	EG329541	329541	2+:153618437-153620916
1482	Nme5	75533	18-:34722376-34738340
1483	V1rc19	171192	6+:56935335-56936244
1484	Aasdhppt	67618	9-:4296039-4309451
1485	Plek	56193	11-:16871452-16908705
1486	Pla2g2a	18780	4+:138388576-138390946
1487	MMT00019522		6+:147220566-147221162
1488	MMT00065132		7+:27030918-27032915
1489	MMT00081278		12-:62600661-62622236
1490	Mapk1	26413	16+:16983659-17039133
1491	Dpf3	70127	12-:84370184-84828658
1492	Cda	72269	4-:137894337-137923895
1493	MMT00047335		12-:10731050-10731395
1494	Cyp2c69	100043108	19-:39917150-39961217
1495	Zfp445	235682	9-:122758064-122771246
1496	MMT00061267		2-:84997574-84998454
1497	Dnaja4	58233	9+:54547190-54562571
1498	MMT00059339		2+:120454884-120460025
1499	MMT00018202		4+:116817861-116820827
1500	Leprel1	210530	16-:25959375-25997389
1501	Rnf145	74315	11+:44332196-44379023
1502	Prelid2	77619	18-:42035254-42072135
1503	Lingo1	235402	9-:56466864-56483435
1504	Xpo7	65246	14-:71064653-71166341
1505	Dusp9	75590	X+:70884779-70888449
1506	OTTMUSG00000C	791264	X+:57736750-57802022
1507	Tcof1	21453	18-:60974196-61008562
1508	Tlr4	21898	4+:66488844-66503828
1509	6330514A18Rik	216166	10+:79819233-79825910
1510	A530058N18Rik	320846	2+:113839131-113858021
1511	Gprc2a-rs5	75835	7+:48923348-48943029
1512	MMT00038113		17+:74886579-74887403
1513	Gzmb	14939	14-:56877696-56881097
1514	Dom3z	112403	17+:34974027-34976177
1515	Med20	56771	17+:47748579-47761199
1516	5830418K08Rik	319675	9-:15156224-15159180
1517	Atxn2l	233871	7-:133635098-133647162
1518	Amigo2	105827	15-:97075410-97076880
1519	Drd1a	13488	13-:54148222-54149541
1520	MMT00069758		6+:4308509-4309482
1521	Copg	54161	6+:87837933-87863586
1522	Nfkbib	18036	7-:29544062-29551512
1523	Prl3d1	18775	13+:27186080-27192118
1524	Krt82	114566	15-:101372139-101381034
1525	MMT00024951		1-:121138548-121139323
1526	Krtap16-9	170657	16-:88869379-88869642
1527	MMT00008776		12-:73720252-73729202
1528	Olfr67	18368	7-:110935737-110940344

1529	Olfr139	259005	11-:73857827-73858774
1530	Cideb	12684	14-:56372896-56377295
1531	Uqcrc2	67003	7+:127778720-127802784
1532	Rab20	19332	8-:11453391-11478681
1533	BC013712	230787	4-:132337754-132352268
1534	Fabp3	14077	4+:129986021-129992674
1535	Vmn2r17	384221	5+:109857438-109882405
1536	Hopx	74318	5-:77470227-77524282
1537	1700052K11Rik	73431	11-:105041137-105042747
1538	Olfr437	258293	6+:43117058-43117990
1539	RP23-233B9.8	622665	4+:116270778-116273177
1540	MMT00003107		18-:3705023-3711635
1541	Olfr13	18310	6+:43123986-43124918
1542	Upp1	22271	11+:9018105-9036167
1543	Acta2	11475	19-:34316006-34329805
1544	MMT00046046		17-:24029863-24030559
1545	Polr2a	20020	11-:69547912-69571725
1546	Ugcg	22234	4+:59202330-59233418
1547	Eid1	58521	2+:125498830-125500519
1548	Rwdd1	66521	10-:33716360-33739422
1549	Gmfg	63986	7+:29226451-29231560
1550	Chst12	59031	5+:140999493-141001191
1551	MMT00011137		8+:22571492-22571863
1552	Ppil2	66053	16-:17085628-17111350
1553	E130112L23Rik	268739	14+:52604579-52614920
1554	Rph3al	380714	11-:75646902-75667983
1555	D430041B17Rik	232813	7-:4781237-4782699
1556	Olfr514	258721	7-:115968578-115969511
1557	Ikzf4	22781	10-:128070769-128081704
1558	Adam15	11490	3-:89143567-89153893
1559	MMT00004662		6-:24293522-24377431
1560	Samm50	68653	15+:84022669-84045284
1561	Aurka	20878	2-:172181690-172196042
1562	Btg2	12227	1-:135971905-135975696
1563	Galp	232836	7+:6154402-6165516
1564	Lbr	98386	1-:183745473-183772463
1565	Gas2l2	237891	11-:83240756-83242930
1566	Kctd15	233107	7-:35424037-35437860
1567	MMT00065763		2+:125423373-125423729
1568	Frmd4b	232288	6-:97236861-97567576
1569	Tmco4	77056	4+:138528806-138615079
1570	Olfr522	258954	7-:147347909-147348847
1571	Adcyap1	11516	17+:93599353-93603467
1572	Saa3	20210	7-:53967496-53970470
1573	Ttc1	66827	11-:43543508-43561475
1574	Nebl	74103	2-:17268360-17652695
1575	AW554918	225289	18+:25333847-25623232
1576	MMT00022934	433745	4-:112627609-112630245
1577	2900011O08Rik	67254	16+:13986750-14101067
1578	4921505C17Rik	78757	15+:6727929-6729266
1579	Clca1	12722	3-:144392881-144423733

1580	Mrs2	380836	13-:25084440-25112248
1581	Tgif2	228839	2+:156665741-156680060
1582	Srr	27364	11-:74721776-74722708
1583	Specc1l	74392	10+:74674815-74775479
1584	Dub2	13532	7+:110565231-110567651
1585	Mif4gd	69674	11-:115469234-115474236
1586	Sfi1	78887	11-:3064552-3077624
1587	MMT00044543		4+:52913468-52915778
1588	Clic5	224796	17+:44325528-44412709
1589	Napg	108123	18+:63137516-63159102
1590	Chst2	54371	9-:95304918-95307141
1591	Bcl2l13	94044	6+:120786233-120838986
1592	Amy1	11722	3-:113258871-113277680
1593	D3Ertd300e	56790	3+:54497026-54520758
1594	Mrpl24	67707	3+:87723465-87727586
1595	Exosc8	69639	3-:54520866-54538960
1596	Garnl3	99326	2-:32841742-32986128
1597	Rbbp7	245688	X+:159198364-159216559
1598	Nhs	195727	X-:158282214-158313324
1599	Lats2	50523	14-:58309954-58353186
1600	Csrp1	13007	1+:137635972-137647923
1601	AA881470	231841	5+:141180979-141195330
1602	Mina	67014	16+:59471632-59492113
1603	Lrwd1	71735	5-:136598936-136611871
1604	Ssbp4	76900	8-:73121389-73132172
1605	Egfr	13649	11+:16652270-16787642
1606	Srp14	20813	2-:118301586-118305357
1607	Memo1	76890	17-:74600045-74694323
1608	Rhbdd1	76867	1+:82335683-82439640
1609	AW146154	101835	7-:48734245-48736869
1610	Ppm1b	19043	17+:85357676-85414115
1611	MMT00014804	634967	17+:40252300-40253014
1612	Clec1b	56760	6+:129347603-129355294
1613	Trim26	22670	17+:36974128-36996321
1614	4933402P03Rik	108803	11-:69630066-69631970
1615	Timm10	30059	2+:84667199-84670368
1616	Dusp13	27389	14-:22552781-22559498
1617	Trp53bp2	209456	1+:184339451-184392564
1618	Mfap1a	67532	2-:121319443-121332385
1619	Fbxw17	109082	13+:50513253-50527925
1620	Htra1	56213	7+:138079748-138129167
1621	Itln1	16429	1-:173448372-173463537
1622	Emd	13726	X+:71500177-71503085
1623	MMT00003407		15+:80306627-80308390
1624	Dzip3	224170	16-:48925921-48994254
1625	AW146154	101835	7+:48745783-48746625
1626	Fmo6	226565	1-:164846855-164867257
1627	2310030N02Rik	76947	4-:10978197-11003334
1628	Cdc25c	12532	18-:34892650-34911162
1629	Gcsh	68133	8-:119505871-119517351
1630	Antxrl	239029	14+:34866851-34868169

1631	Aldob	230163	4-:49549296-49562357
1632	Rnf185	193670	11-:3315985-3352290
1633	Kars	85305	8-:114517507-114535168
1634	MMT00022209		6-:149381664-149383119
1635	8430427H17Rik	329540	2-:153233197-153262098
1636	Zfp777	72306	6-:47974186-48033604
1637	MMT00071316		1-:71760373-71761572
1638	MMT00001787		3+:109710332-109710788
1639	Atp10b	319767	11+:42968991-43067891
1640	Reps1	19707	10+:17809256-17844938
1641	Hoxc11	109663	15+:102784937-102787130
1642	Mapk4	225724	18-:74090055-74130090
1643	MMT00013277		3-:150178180-150179116
1644	Loxhd1	240411	18+:77643537-77652988
1645	Vps13b	666173	15+:35724059-35860983
1646	Slc15a4	100561	5-:128076036-128097762
1647	Olfr1216	258895	2-:88853283-88854219
1648	Gnpnat1	54342	14-:45996095-46008471
1649	Pcolce	18542	5-:138046337-138052587
1650	Ctdsp2	52468	10+:126432666-126437025
1651	V1rc29	171202	6-:57272719-57273629
1652	MMT00027298		9-:72278992-72279648
1653	Anxa5	11747	3-:36347847-36374620
1654	Dhx58	80861	11-:100564073-100565585
1655	Mgrn1	17237	16+:4886343-4938284
1656	Ahr	11622	12-:36182693-36219576
1657	Olfr912	258806	9+:38388863-38389796
1658	Gp49a	14727	10+:51200484-51206025
1659	Hoxb3	15410	11+:96205082-96207821
1660	Zfp33b	69623	5+:105012010-105013638
1661	Duoxa1	213696	2-:122129288-122139429
1662	Nos1ap	70729	1-:172248602-172320956
1663	Fkbp4	14228	6-:128380127-128388545
1664	Gspt1	14852	16-:11220493-11254089
1665	Rpusd1	106707	17+:25864672-25868400
1666	Gp1ba	14723	11+:70452623-70455555
1667	Tbr1	21375	2+:61642669-61652168
1668	Lhx1	16869	11-:84332880-84339036
1669	Lhfpl4	269788	6-:113121776-113145339
1670	Ssu72	68991	4+:155079232-155107891
1671	2210412E05Rik	75690	7+:50719338-50727385
1672	Mlh1	17350	9-:111130758-111174066
1673	Cldn16	114141	16+:26463430-26482827
1674	MMT00081013		X-:141699601-141700072
1675	Rest	19712	5+:77714870-77715430
1676	Tmem170	66817	8-:114388797-114400575
1677	Fabp1	14080	6+:71149881-71155016
1678	Tcf15	21407	2+:151969307-151974830
1679	MMT00009278		3+:95786885-95787446
1680	Atp6v1f	66144	6+:29417782-29420509
1681	Kng2	385643	16-:22987406-23029166

1682	MMT00072239		9+:31755961-31756616
1683	Igh-VJ558	16061	12-:115034367-115034778
1684	Scnn1b	20277	7+:129008624-129062029
1685	Zcchc7	319885	4+:44773314-44775353
1686	MMT00064576		
1687	Gp1bb	14724	16-:18620816-18621673
1688	Camp	12796	9-:109749900-109751963
1689	Trim72	434246	7+:135147997-135153974
1690	Nlrp5	23968	7+:24189120-24226935
1691	Maml1	103806	11-:50071351-50105845
1692	Fancm	104806	12+:66176788-66232982
1693	Ufsp1	70240	5+:137735896-137736892
1694	Ndufb5	66046	3+:32635983-32650486
1695	MMT00006993		1+:31331746-31344343
1696	V1re9	252904	7-:11165193-11166151
1697	Sf4	70616	8+:72566711-72596244
1698	Olfr1428	258673	19-:12183090-12184034
1699	Chrac1	93696	15+:72920866-72924069
1700	Ccdc64	75665	5-:116098188-116181568
1701	Tesk1	21754	4+:43456306-43460369
1702	Galc	14420	12-:99442005-99497617
1703	MMT00009122		9-:71446705-71447587
1704	Obox5	252829	7+:16343257-16344345
1705	Gltpd1	79554	4-:155239391-155241412
1706	C2cd2l	71764	9-:44117963-44128368
1707	Dock3	208869	9-:106797471-107081497
1708	Tmem111	66087	6-:113464877-113481633
1709	Mt2	17750	8+:96696346-96697461
1710	2300005B03Rik	69462	15-:74573194-74573919
1711	Grhl1	195733	12+:25265583-25302252
1712	Rnf2	19821	1-:153316535-153348027
1713	Nsun6	74455	2-:14917926-14976511
1714	Ampd1	229665	3+:102877953-102903646
1715	Spem1	74288	11-:69634389-69635682
1716	Git1	216963	11+:77307063-77321269
1717	Ndufs2	226646	1-:173176219-173177245
1718	Metrn	70083	17-:25931157-25934027
1719	Trak2	70827	1-:58957293-59030314
1720	D630042P16Rik	243612	6-:112319449-112334459
1721	MMT00001668		5+:17849268-17857976
1722	Iqgap1	29875	7-:87857671-87948180
1723	Usp42	76800	5-:144474191-144488886
1724	MMT00004378		X-:92406801-92407116
1725	MMT00045736		16-:25441098-25443404
1726	Avp	11998	2-:130406413-130408277
1727	Gng2	14702	14-:20695030-20710625
1728	Car6	12353	4-:149561126-149572579
1729	Psmc5	19184	11+:106117498-106124426
1730	Pcnxl2	270109	8-:128386985-128389958
1731	Rnf20	109331	4+:49644958-49670124
1732	Hspa4	15525	11-:53075072-53113683

1733	Dopey2	70028	16+:93712222-93740523
1734	Arrdc2	70807	8-:73360045-73363618
1735	Prss16	54373	13-:22094044-22101610
1736	Elovl3	12686	19+:46206388-46210184
1737	Gtpbp4	69237	13-:8972368-8995242
1738	Mfap5	50530	6+:122463693-122479108
1739	Lrrc17	74511	5+:21049376-21081718
1740	RP23-244H7.9	378700	2+:153745371-153758732
1741	MMT00066806		X-:118290617-118291554
1742	4930415F15Rik	73862	11+:11388510-11415191
1743	Cisd1	52637	10-:70793466-70807623
1744	Clec3b	21922	9+:123059966-123066545
1745	Gas7	14457	11+:67416606-67498461
1746	MMT00010791		6+:58411063-58411603
1747	RP23-162H3.1	666581	4+:146753337-146753561
1748	Rbp1	19659	9+:98323379-98346967
1749	Hnrnpu	51810	1-:180259449-180267843
1750	Fcer1a	14125	1-:175151414-175157345
1751	Xdh	22436	17-:74233251-74299493
1752	MMT00035634		19-:53842240-53842847
1753	Slc36a4	234967	9+:15487345-15516132
1754	Bccip	66165	7+:140901019-140912827
1755	Ocel1	77090	8+:73895224-73897056
1756	Usp43	216835	11-:67668032-67735384
1757	Olfr629	258818	7-:110888808-110889752
1758	Cadps2	320405	6-:23446750-23638963
1759	Rgs3	50780	4+:62220880-62320868
1760	Nek8	140859	11-:77979607-77990118
1761	2010110K16Rik	67899	9-:117973646-118059210
1762	EG620986	620986	1-:9792275-9792622
1763	Mcam	84004	9+:43942753-43950800
1764	Tifab	212937	13-:56275845-56280014
1765	Hist1h2af	319173	13+:23622983-23626528
1766	Srpx2	68792	X+:130443234-130466352
1767	BC016495	225994	19+:18706505-18726505
1768	Fmo9	240894	1-:168593079-168611977
1769	Nr5a1	26423	2-:38548146-38570020
1770	BC054059	246747	2+:158328347-158333934
1771	Park2	50873	17+:11494485-11696865
1772	Stambp	70527	6-:83493200-83522430
1773	MMT00025876	100042378	4+:155610026-155610434
1774	Olfr916	258780	9-:38465042-38465975
1775	MMT00019356		13-:114270104-114270513
1776	4921511C04Rik	70853	1+:37210690-37244460
1777	Slc5a4a	64452	10+:75610195-75652010
1778	Gtf2e1	74197	16-:37509881-37539855
1779	Myo1c	17913	11+:75471014-75486146
1780	Ensa	56205	3+:95429041-95432629
1781	E030010A14Rik	226040	19+:24748501-24753430
1782	Zfp650	68795	2+:69735538-69790926
1783	Arcn1	213827	9-:44550229-44575876

1784	Lipi	320355	16-:75560971-75574403
1785	Slc22a8	19879	19+:8667730-8685127
1786	Tnfsf10	22035	3+:27216022-27235019
1787	MMT00004455		14-:63133622-63134211
1788	Gadd45gip1	102060	8+:87356182-87358373
1789	Pcbp2	18521	15+:102303688-102329310
1790	Hic1	15248	11-:74978070-74983021
1791	Ints4	101861	7+:104629519-104689907
1792	Zmat4	320158	8+:24858871-25125688
1793	D3Ucla1	28146	3-:58325899-58329676
1794	EG633692	633692	14-:53158326-53160552
1795	Rabgap1l	29809	1-:162237110-162402248
1796	5033428B15Rik	76002	19-:11071514-11092520
1797	Batf2	74481	19+:6140980-6172472
1798	Hoxc5	15424	15+:102844512-102845873
1799	Khdrbs2	170771	1+:32229650-32714583
1800	Retn	57264	8+:3655788-3658093
1801	4930474N05Rik	218921	14+:36908101-36910039
1802	Ipo4	75751	14-:56244749-56254435
1803	Ngdn	68966	14+:55639889-55643072
1804	Tgm2	21817	2-:157943535-157972042
1805	Tmem204	407831	17-:25194652-25218124
1806	Nola3	66181	2+:112102125-112103055
1807	MMT00033277		6+:50558635-50560695
1808	Rrp1	18114	10-:77863131-77875740
1809	Odz3	23965	8+:49311026-49311684
1810	Pde7b	29863	10-:20119357-20444655
1811	Rnaseh1	19819	12+:29334512-29344454
1812	Npm2	328440	14-:71048070-71051968
1813	Commd3	12238	2+:18594091-18597551
1814	MMT00042573		14+:19961424-19963751
1815	1200015F23Rik	67809	2-:118962555-118982699
1816	Gjb2	14619	14-:57718907-57719586
1817	Lrrc44	74435	3+:154756502-154857113
1818	Zfp362	230761	4-:128450330-128467559
1819	Rnf17	30054	14+:57021419-57057480
1820	Mat2b	108645	11-:40492815-40506115
1821	MMT00010266		4-:109076399-109078852
1822	Dpf1	29861	7+:30089023-30102605
1823	MMT00080166		2+:161112955-161113226
1824	Zfp385b	241494	2-:77288196-77357669
1825	Gga1	106039	15+:78707619-78725014
1826	Acaa2	52538	18+:74938568-74965868
1827	Gnb1l	13972	16+:18498912-18564614
1828	Elmo3	234683	8+:107821623-107833891
1829	MMT00030026		1+:193816705-193817410
1830	Kctd10	330171	5-:114813581-114830495
1831	Ager	11596	17+:34734833-34737878
1832	Plekha1	101476	7+:138009423-138056815
1833	Stard6	170461	18+:70635757-70660279
1834	Ermap	27028	4-:118848195-118862579

1835	MMT00028430		5-:136119256-136120393
1836	Igh-VJ558	16061	12-:115920819-116089872
1837	MMT00014156		19+:24228659-24234446
1838	Kif9	16578	9+:110412883-110413959
1839	Igf2	16002	7-:149836673-149847765
1840	Fpr1	14293	17-:18013596-18014689
1841	MMT00054498		2+:31841028-31842169
1842	V1rc15	171188	6-:66502876-66503784
1843	Tsc2	22084	17-:24733465-24769541
1844	Rrm2b	382985	15-:37856984-37890716
1845	Olfr1465	258121	19-:13356623-13357546
1846	MMT00064320		
1847	Naga	17939	15-:82160491-82168683
1848	Tspan32	27027	7+:150191823-150204644
1849	MMT00063181		1-:151869353-151870959
1850	MMT00075142		5+:7856961-7857846
1851	Tmem41a	66664	16-:21934399-21947617
1852	MMT00041847		
1853	Stat5a	20850	11+:100720969-100746470
1854	Vgf	381677	5+:137506965-137509195
1855	Prss12	19142	3+:123149830-123209512
1856	MMT00000408		9-:108260488-108261433
1857	Nfatc3	18021	8+:108583747-108655624
1858	2810432L12Rik	67063	4-:49598286-49610670
1859	Idua	15932	5+:109098394-109113573
1860	Stau2	29819	1-:16334278-16365476
1861	Cetn2	26370	X-:70159044-70163683
1862	Vdac1	22333	11+:52174616-52202898
1863	MMT00052951		5-:109445281-109445937
1864	MMT00020890	100041434	8+:73242440-73246532
1865	Polr2f	69833	15+:78971433-78982281
1866	2010012O05Rik	66439	19+:46764403-46776385
1867	Bcas2	68183	3+:102975667-102982750
1868	Atp6v0e2	76252	6+:48487613-48491793
1869	Dkc1	245474	X+:72341216-72355336
1870	EG627821	627821	7+:6224575-6233457
1871	Tspyl2	52808	X-:148771400-148774899
1872	MMT00039686		6+:67674907-67675754
1873	Itgb3bp	67733	4-:99432896-99495833
1874	Slc19a2	116914	1+:166179185-166195497
1875	Cd19	12478	7-:133551964-133558350
1876	Amdhd1	71761	10-:92986449-93002732
1877	Amica1	270152	9+:44896695-44916591
1878	MMT00047735		2+:26890199-26890933
1879	MMT00054521	666814	1+:45658897-45659599
1880	Vps18	228545	2+:119114463-119124188
1881	Slc16a14	71781	1-:84908769-84931804
1882	Kcnip4	80334	5-:48782151-48901000
1883	MMT00048636		14+:12007493-12012887
1884	Ppt1	19063	4+:122513497-122535625
1885	Smr3a	20599	5+:88431573-88437558

1886	Tcte1	21645	17+:45670001-45679629
1887	Mapkapk3	102626	9-:107157262-107191727
1888	Olfr1396	258334	11-:48926279-48927259
1889	Dcun1d3	233805	7-:127000760-127039194
1890	Nanp	67311	2-:150855452-150865091
1891	Sv2a	64051	3+:95988908-95997838
1892	A1bg	117586	15-:60749156-60752829
1893	MMT00072326	330914	9+:31995218-32068816
1894	Lrp4	228357	2+:91297664-91351916
1895	Depdc1a	76131	3+:159158482-159192052
1896	Vars	22321	17+:35138388-35153199
1897	1110034A24Rik	109065	12-:70290077-70299272
1898	Aadat	23923	8+:62984920-63024474
1899	1110031B06Rik	67826	11-:59220652-59263458
1900	Hlf	217082	11-:90249097-90252154
1901	Tpp1	12751	7-:112894592-112900729
1902	Eml2	72205	7+:19761962-19791828
1903	Igh	111507	12-:114954877-115045958
1904	Aanat	11298	11+:116455000-116458858
1905	B4galnt1	14421	10+:126602932-126609276
1906	Ssr3	67437	3-:65183578-65196475
1907	D230010M03Rik	241547	2+:91551082-91553022
1908	Rnf213	629974	11+:119308548-119311188
1909	Tcap	21393	11+:98245124-98246526
1910	Krtap5-2	71623	7+:149442728-149444277
1911	1300007F04Rik	67477	11+:77328621-77334117
1912	MMT00048357		5-:16730336-16732695
1913	Polr2j	20022	5+:136592606-136598809
1914	EG384325	384325	5-:30547413-30547756
1915	Trim47	217333	11-:115967072-115971552
1916	Espn	56226	4-:151495231-151502918
1917	Sh2d3c	27387	2+:32576639-32610427
1918	Usp39	28035	6-:72268671-72295169
1919	MMT00027062		
1920	Oas1e	231699	5-:121236330-121245513
1921	4930431L04Rik	270049	8-:60980148-61179845
1922	Rell1	100532	5-:64300775-64360105
1923	MMT00015352		9-:57981776-58003600
1924	Pex3	56535	10-:13243926-13272871
1925	6430531B16Rik	381933	7-:147158198-147161888
1926	RP23-12I24.6	272009	4+:33296280-33318134
1927	Gabrr3	328699	16+:59415094-59447946
1928	Triml2	622117	8+:44285339-44285697
1929	Leng8	232798	7+:4088637-4099773
1930	Ccdc109b	66815	3-:129617878-129672997
1931	2310047B19Rik	66962	9+:21760203-21762707
1932	Racgap1	26934	15-:99450955-99482031
1933	MMT00002546		18-:7842980-7845679
1934	Ttc24	214191	3-:87873332-87875975
1935	2810439F02Rik	72747	18+:12802308-12895558
1936	Cbwd1	226043	19-:24994920-25036048

1937	MMT00075644		3-:26945708-26946550
1938	Casz1	69743	4+:148178585-148264493
1939	MMT00052169		
1940	MMT00002367		11+:41312177-41313958
1941	MMT00079618		6+:78998315-78998815
1942	Hoxd1	15429	2+:74601036-74603200
1943	Slc27a4	26569	2+:29658212-29672138
1944	Sprr2h	20762	3+:92189634-92191241
1945	Fut10	171167	8+:32297804-32346953
1946	Phospho1	237928	11+:95685808-95693272
1947	Myo9b	17925	8+:73796632-73884612
1948	Stard7	99138	2+:127095974-127124668
1949	BC048609	433215	19+:6080030-6080786
1950	Bysl	53414	17-:47738502-47741320
1951	Sema4g	26456	19+:45063833-45077884
1952	Cntn6	53870	6+:104519032-104813220
1953	Bard1	12021	1-:71076940-71149492
1954	Bank1	242248	3-:135876793-135988939
1955	Wdr48	67561	9+:119803960-119835697
1956	Usp50	75083	2-:126587157-126609180
1957	Mansc1	67729	6-:134559227-134582420
1958	Bai1	107831	15+:74359586-74419886
1959	AY074887	246735	9-:54798065-54798805
1960	Ubr2	224826	17-:47067196-47107076
1961	Mlf1	17349	3+:67178112-67203884
1962	Rpgrip1	77945	14-:52762983-52763826
1963	MMT00039783		10+:34618039-34618914
1964	C730048C13Rik	319800	19-:8145456-8479572
1965	Ncoa2	17978	1-:13132555-13364145
1966	OTTMUSG00000(	433520	2+:177282474-177294210
1967	Brwd1	93871	16-:96214055-96304033
1968	Nudc	18221	4-:133088458-133101864
1969	Cep72	74470	13-:74175282-74199722
1970	Gpm6a	234267	8+:56122674-56146391
1971	Kcnh5	238271	12-:76066111-76255268
1972	Ly6g6e	70274	17+:35213711-35215749
1973	Asb8	78541	15-:97965817-97976057
1974	MMT00038542		10-:92005149-92006415
1975	Dnahc9	237806	11-:65644827-65794849
1976	Rpl31	114641	1+:39424723-39428176
1977	Timm17b	21855	X+:7476512-7484063
1978	Ublcp1	79560	11-:44268078-44274332
1979	Tubgcp4	51885	2+:120996903-121024358
1980	Ahcyl1	229709	3-:107467961-107499295
1981	Basp1	70350	15+:25293027-25307123
1982	EG668192	668192	10+:43541036-43542042
1983	MMT00052857		10-:38872953-38878239
1984	Ubqln3	244178	7-:111289418-111291464
1985	Pepd	18624	7+:35697425-35829725
1986	Crebl2	232430	6+:134799120-134808700
1987	BC066135	271887	2-:180016078-180024587

1988	EG545987	545987	7-:112097690-112098949
1989	Sp4	20688	12-:119476449-119539841
1990	Prkg1	19091	19-:30643036-31738860
1991	Ttc17	74569	2-:94140923-94246822
1992	Arl8b	67166	6+:108733089-108772404
1993	EG632649	632649	5-:152315709-152351823
1994	Tram1l1	229801	3+:124024026-124025770
1995	Rab6b	270192	9+:103014278-103085542
1996	Gsdma3	450219	11+:98460132-98499247
1997	Vamp1	22317	6+:125168591-125170083
1998	MMT00061243		9-:22284377-22284926
1999	Tor3a	30935	1-:158584715-158604438
2000	Bola1	69168	3-:96000163-96001660
2001	BC052688	432812	13+:61887326-61887574
2002	Hapln3	67666	7-:86261914-86275906
2003	Ppapdc1	71910	8+:26830513-26835364
2004	1810024B03Rik	329509	2+:127030406-127030783
2005	Rheb	19744	5-:24309068-24348231
2006	Agbl1	244071	7+:83843594-83911353
2007	4933425L03Rik	71169	12+:13316980-13590610
2008	Aff3	16764	1-:38591750-38683994
2009	Ear4	53877	14+:51823364-51823831
2010	Fer1l6	631797	15+:58389803-58494338
2011	Slc2a5	56485	4+:149494830-149517864
2012	Cetn4	207175	3-:37207549-37211368
2013	Il15ra	16169	2+:11627162-11655588
2014	Atp6v0a4	140494	6-:37998478-38044056
2015	Aga	11593	8+:54597057-54608774
2016	Wdr78	242584	4-:102710674-102786829
2017	Dock2	94176	11-:34127668-34597325
2018	Atp7a	11977	X+:103265113-103320039
2019	Sf3a1	67465	11+:4060374-4080626
2020	MMT00032099		19+:33062444-33073597
2021	Atf7ip	54343	6+:136467378-136555589
2022	Phc2	54383	4+:128382239-128430118
2023	MMT00072215		1+:77046869-77047310
2024	Tdgf1	21667	9-:110842106-110848571
2025	Prssl1	73106	10-:79245663-79251669
2026	Nrg2	381149	18-:36177313-36228471
2027	Wnt5b	22419	6-:119383007-119398272
2028	Isca2	74316	12+:86114218-86116035
2029	MMT00065054		14-:80741501-80742340
2030	Zc3h15	69082	2+:83484637-83498459
2031	Il6	16193	5+:30339700-30346508
2032	1700007K13Rik	69327	2-:28317375-28321853
2033	2010106E10Rik	67715	X+:109608883-109671942
2034	Ptprk	19272	10+:27794625-28316026
2035	Slc44a3	213603	3-:121162446-121235249
2036	Rnf150	330812	8+:85514245-85566680
2037	Ankrd16	320816	2+:11700151-11711955
2038	Cacna1c	12288	6-:118542481-119058572

2039	9930105H17Rik	100669	14+:41866897-41867955
2040	MMT00006448		6-:4341029-4355205
2041	Cct7	12468	6+:85409107-85418368
2042	Schip1	30953	3+:68376482-68429779
2043	Arl2bp	107566	8+:97190654-97198320
2044	Hsd17b7	15490	1-:171882491-171899326
2045	Olfr1457	258568	19-:13169184-13170136
2046	Tmem80	71448	7+:148514028-148522710
2047	Aig1	66253	10-:13372514-13588636
2048	Uty	22290	Y-:439205-582063
2049	Gsta3	14859	1+:21239829-21255300
2050	Lpl	16956	8+:71404452-71430830
2051	Ddx27	228889	2+:166840809-166891360
2052	Slc38a9	268706	13+:113459675-113523259
2053	Olfr5	18349	7-:6432927-6433865
2054	Acaa1a	113868	9+:119250595-119259412
2055	MMT00012951		5+:84157713-84158184
2056	Ttn	22138	2-:76705214-76709945
2057	Pawr	114774	10+:107769727-107851885
2058	Hk3	212032	13-:55106856-55122559
2059	5630401D24Rik	71449	1-:164463804-164478476
2060	Olfr1008	258866	2+:85529587-85530527
2061	Lgi3	213469	14+:70930645-70937817
2062	EG668830	668830	17-:81337261-81338134
2063	EG546335	546335	X+:75371334-75372453
2064	Eps8l1	67425	7+:4416997-4430528
2065	Aph1a	226548	3+:95697915-95701385
2066	Olfr1256	258985	2-:89675179-89676100
2067	Myo1a	432516	10+:127142870-127157997
2068	EG623133	623133	8+:4652053-4653615
2069	Rps18	20084	17-:34088944-34092586
2070	Gnat1	14685	9-:107578218-107581879
2071	Pih1d2	72614	9+:50425438-50429992
2072	Olfr980	259110	9-:39813599-39814532
2073	Sirpb1	320832	3-:15416834-15426482
2074	Sfxn1	14057	13+:54180828-54201971
2075	Atp6v1g1	66290	4+:63205870-63211734
2076	Rin2	74030	2+:145686398-145712905
2077	Olfr181	259001	16-:58925443-58926395
2078	MMT00057419		12-:115850430-115854494
2079	Mthfs	107885	9+:89106027-89135063
2080	Dnahc12	110083	14+:27618049-27620521
2081	Dlat	235339	9-:50444459-50467622
2082	AU018778	234564	8-:95780140-95803610
2083	1110007C09Rik	68480	13-:49298319-49311395
2084	Pftk1	18647	5-:5373457-5380364
2085	MMT00007546		4-:114811245-114828986
2086	Prpf8	192159	11+:75300591-75322885
2087	MMT00011735		17-:37106151-37108058
2088	Ppan	235036	9+:20692700-20696622
2089	Znrf4	20834	17-:56650670-56651839

2090	Rpo1-2	20017	2+:128926776-128952330
2091	Wfdc1	67866	8+:122190395-122211892
2092	Bcat1	12035	6-:144948782-144995886
2093	Mbp	17196	18+:82644554-82755019
2094	RP23-360A2.8	100043403	2-:177715240-177718683
2095	Siglech	233274	7+:63023596-63027953
2096	MMT00072212	380822	12-:116446282-116746212
2097	C8a	230558	4-:104488290-104548988
2098	2810428J06Rik	67058	3-:135088200-135089958
2099	Ift74	67694	4+:94321517-94359920
2100	Cep68	216543	11-:20128514-20149410
2101	Ube2r2	67615	4+:41082921-41138282
2102	Ceacam14	67084	7+:18398030-18401012
2103	Svopl	320590	6-:37972996-37996998
2104	Olfr646	259058	7+:111254794-111255732
2105	Pear1	73182	3-:87554022-87572878
2106	4931406H21Rik	77592	14-:26406032-26413423
2107	Mrpl4	66163	9+:20807429-20813275
2108	Fzd3	14365	14-:65821243-65872267
2109	Aqp2	11827	15+:99409583-99414472
2110	Gtf2ird1	57080	5-:134833538-134932560
2111	MMT00003004		14+:51931170-51936428
2112	MMT00061406		7+:11396128-11398849
2113	Rasd1	19416	11-:59776686-59778437
2114	Coro6	216961	11+:77276083-77283293
2115	Gpr12	14738	5-:147394327-147396408
2116	Hyls1	76832	9-:35368410-35378190
2117	Fibp	58249	19+:5460693-5464992
2118	Kptn	70394	7+:16705256-16712863
2119	Lamc3	23928	2+:31742809-31802640
2120	Zfp791	244556	8-:87633546-87678846
2121	Brms1l	52592	12+:56937603-56970720
2122	Olfr954	258328	9+:39269017-39269960
2123	EG384525	384525	7+:5970606-5971109
2124	MMT00002232		X+:10258089-10258566
2125	MMT00008580		8-:53639725-54014209
2126	Dgat2	67800	7-:106302951-106331081
2127	Ldha	16828	7+:54102582-54110996
2128	4930447K03Rik	432745	13-:34176023-34205141
2129	MMT00046536		18-:20841057-20841503
2130	Smcr7	237781	11+:60541924-60546442
2131	Zdbf2	73884	1+:63319840-63350080
2132	MMT00030693	100047070	6-:70093482-70167167
2133	MMT00013038		11-:90716203-90731816
2134	Pes1	64934	11+:3864039-3870699
2135	Taf4b	72504	18+:14941751-15058151
2136	Cnksr2	245684	X-:154261433-154434622
2137	Nexn	68810	3-:151899946-151916671
2138	A930008G19Rik	77938	7-:139906629-140028890
2139	Zfp825	235956	13-:74617505-74631398
2140	Myo16	244281	8+:10569452-10633649

2141	Chmp1a	234852	8-:125728163-125736638
2142	Phldb1	102693	9-:44494391-44535257
2143	MMT00066022		15+:82294587-82295230
2144	MMT00060164		18+:80155666-80156692
2145	MMT00075271		7+:13328413-13329074
2146	MMT00044046		18-:51277050-51414788
2147	MMT00060230		13-:62488652-62489382
2148	Tas2r105	57252	6-:131636578-131637481
2149	Mtch1	56462	17-:29469653-29484732
2150	Rragc	54170	4+:123594755-123614233
2151	Cgnl1	68178	9-:71476623-71478903
2152	Srfbp1	67222	18+:52625344-52643367
2153	Ssbp1	381760	6+:40421293-40430999
2154	Rgs9	19739	11-:109086668-109159443
2155	Olfr582	259055	7+:110189994-110190953
2156	Tnfaip8	106869	18+:50213173-50251431
2157	0610007C21Rik	381629	5+:31350592-31356994
2158	Slc10a3	214601	X-:71614567-71618595
2159	Ceacam9	26368	7+:17307277-17311459
2160	Slc6a17	229706	3-:107274099-107320936
2161	Cd177	68891	7-:25529003-25531220
2162	2410042D21Rik	72425	2+:112219411-112253491
2163	4930503E14Rik	74954	14-:44740833-44749036
2164	Olfr796	258933	10-:129044604-129045535
2165	Tmem128	66309	5+:38651613-38660857
2166	Amd1	11702	10-:40007283-40021921
2167	Pif1	208084	9+:65435008-65443769
2168	Cox15	226139	19-:43809623-43827450
2169	Lrrc56	70552	7+:148381805-148396059
2170	Asphd1	233879	7-:134089499-134092250
2171	Sytl4	27359	X-:130470923-130516322
2172	EG544954	544954	13+:75001727-75002729
2173	C030048B08Rik	269623	5-:3583724-3596547
2174	MMT00053383		7+:24142108-24143830
2175	Lin52	217708	12+:85792456-85879602
2176	Trim59	66949	3-:68847478-68848667
2177	Htatip2	53415	7+:57014968-57028895
2178	Slc8a1	20541	17-:81784942-82137709
2179	Trim3	55992	7-:112759161-112782045
2180	1700081D17Rik	73523	14+:70240212-70251766
2181	Slc35f1	215085	10+:52652897-52828163
2182	Slc9a10	208169	16+:45601995-45607112
2183	1810035L17Rik	380773	12+:88784780-88791117
2184	MMT00043920		
2185	Pef1	67898	4+:129784858-129805378
2186	2900026A02Rik	243219	5-:113592213-113621205
2187	Kif5b	16573	18-:6203086-6241342
2188	Prpf38a	230596	4-:108237313-108251934
2189	Mov10	17454	3-:104597753-104621160
2190	Heyl	56198	4+:122910798-122925962
2191	2900075B16Rik	78506	8+:41392783-41466183

2192	Casc3	192160	11+:98666218-98695120
2193	Oca2	18431	7+:63495140-63791887
2194	Hectd2	226098	19+:36679657-36693329
2195	Lgals7	16858	7+:29649849-29651210
2196	Foxq1	15220	13+:31650785-31651987
2197	Snap23	20619	2+:120410003-120425183
2198	Epb4.1l5	226352	1-:121444088-121451728
2199	Aff1	17355	5+:104283521-104284335
2200	BC006779	229003	2-:180962318-180975622
2201	Tssk5	73542	15-:76202383-76205370
2202	Lcor	212391	19+:41620364-41661025
2203	Cts3	117066	13-:61589372-61591397
2204	Olfr284	258278	15-:98170452-98171370
2205	Olfr1243	258971	2-:89367647-89368565
2206	MMT00046613		
2207	Ogn	18295	13+:49704513-49718684
2208	Klhl32	212390	4-:24671004-24719944
2209	Il1rapl1	331461	X-:83992581-84033187
2210	Chn1	108699	2-:73450944-73463797
2211	Ttpa	50500	4+:19935586-19957932
2212	Vps53	68299	11-:75859801-75993132
2213	Cox5a	12858	9+:57369115-57380231
2214	Larp6	67557	9+:60560923-60586337
2215	Zfp71-rs1	235907	13-:67806245-67830019
2216	Fabp3	14077	4+:129986021-129992706
2217	Dydc1	69496	14+:41886198-41905478
2218	D15Mgi27	106073	15+:102110526-102112184
2219	Rdh10	98711	1+:16095815-16123813
2220	Cntnap1	53321	11+:101037430-101051160
2221	Elp3	74195	14-:66149291-66211926
2222	Tagln	21345	9-:45738056-45743980
2223	Slc28a2	269346	2+:122267294-122286740
2224	Oasl2	23962	5+:115346944-115362235
2225	Ceacam11	66996	7+:18557472-18563898
2226	Amt	434437	9+:108199212-108204964
2227	Cenpa	12615	5+:30969274-30977197
2228	Nox1	237038	X-:130621685-130642380
2229	Gjc1	14615	11-:102660894-102665955
2230	Slc35d1	242585	4-:102846085-102887314
2231	Piwil4	330890	9-:14506220-14539101
2232	Strn	268980	17-:79054572-79135827
2233	Eltd1	170757	3+:151100850-151208044
2234	MMT00046581		16-:55940431-55940830
2235	Ndufv1	17995	19-:4007506-4012725
2236	Adnp2	240442	18-:80286084-80348214
2237	Gpr110	77596	17+:43407277-43461686
2238	Ceacam15	101434	7-:17234660-17260961
2239	4930503E24Rik	75030	10+:123906127-123961543
2240	MMT00045791		16-:27343196-27343655
2241	Trim7	94089	11+:48650972-48654917
2242	Ptn	19242	6-:36665662-36761361

2243	Gcsh	68133	8-:119505729-119517349
2244	Klre1	243655	6+:129528324-129535842
2245	Sh2d4b	328381	14-:41673671-41706556
2246	Syne1	64009	10+:4978236-5013130
2247	Olig1	50914	16+:91270169-91270903
2248	Ly6g6d	114654	17-:35208489-35211409
2249	BB014433	434285	8-:15041449-15046069
2250	Tdrd12	71981	7-:36278630-36322701
2251	Ankrd1	107765	19-:36187146-36194285
2252	MMT00020839	100040997	16+:96108180-96123331
2253	Lysmd1	217779	3+:94938016-94943440
2254	D0H4S114	27528	18-:33597766-33623542
2255	Trim56	384309	5-:137588327-137592080
2256	Serpina9	71907	12-:105234831-105251862
2257	2610044O15Rik	72139	17-:95213644-95233933
2258	2510002D24Rik	72307	16+:18836658-18840513
2259	Ddx3x	13205	X+:12858163-12869740
2260	Spink10	328971	18+:62810503-62821020
2261	MMT00028230		2-:161080144-161081236
2262	Papss2	23972	19+:32693437-32740227
2263	Dpy19l2	320752	9-:24361496-24500734
2264	Alb	11657	5+:90889920-90905775
2265	Rasgrf1	19417	9+:89804614-89921764
2266	Angptl2	26360	2+:33083714-33101821
2267	2210417D09Rik	66532	6+:146981058-146982007
2268	4732479N06Rik	215201	X-:130757494-130811522
2269	Gucy1b2	239134	14-:63023323-63067105
2270	Pter	19212	2+:12845756-12923240
2271	Pkdrej	18766	15-:85645783-85652163
2272	MMT00043698		13+:19431717-19444631
2273	Haghl	68977	17-:25919769-25922278
2274	Tnnc2	21925	2-:164602663-164605229
2275	Cyp4b1	13120	4-:115297332-115320310
2276	Nelf	56876	2+:24909932-24918400
2277	Igh	111507	12-:114954754-115034779
2278	Gtf3c1	233863	7-:132784466-132851279
2279	MMT00043269		4-:155628563-155629374
2280	4930555I21Rik	78806	4+:135051901-135093718
2281	1700013F07Rik	75504	3+:108337061-108348195
2282	Cbx8	30951	11-:118899751-118902227
2283	MMT00029554		10+:81893449-81894588
2284	Syt7	54525	19+:10463972-10522933
2285	Lipf	67717	19+:34035737-34051303
2286	MMT00034424		1-:44520616-44521136
2287	Olfr351	258944	2-:36714935-36715866
2288	Lyzl1	67328	18+:4165829-4182234
2289	MMT00078995		1-:184322675-184323666
2290	Il1rapl2	60367	X+:134106087-135381494
2291	MMT00041633		13-:61788625-61789350
2292	Limk2	16886	11-:3244259-3309177
2293	Adam17	11491	12-:21331136-21379293

2294	AU040829	216831	11-:64816675-64976461
2295	MMT00006781		11-:21089046-21090569
2296	Oprl1	18389	2+:181449807-181454108
2297	Syce2	71846	8+:87396009-87411294
2298	Mtmr12	268783	15+:12160027-12201987
2299	Ccl19	24047	4+:42248503-42250497
2300	C330023M02Rik	231713	5+:121847982-121889265
2301	Pcyox1	66881	6-:86336965-86347100
2302	Prmt5	27374	14-:55126019-55136244
2303	AI837181	107242	19+:5425156-5427312
2304	Jund	16478	8+:73222931-73224514
2305	2610002M06Rik	67028	X-:104982682-105011620
2306	Gtdc1	227835	2-:44419938-44490836
2307	MMT00001230		18-:29816873-29817413
2308	A930005I04Rik	403174	5+:35260190-35266487
2309	Tet2	214133	3-:133128829-133134781
2310	Pdia3	14827	2+:121239695-121263715
2311	RP23-336F11.32	213765	2-:112090715-112095980
2312	MMT00035795		4-:129285121-129287721
2313	Olfr641	259075	7+:111188311-111189249
2314	Zswim6	67263	13-:108516172-108523916
2315	Abcb1a	18671	5+:8660091-8748519
2316	MMT00036506		
2317	Ankar	319695	1-:72696779-72705377
2318	Igh-VJ558	16061	12-:117150531-117151034
2319	E2f1	13555	2-:154385373-154395588
2320	Spc24	67629	9-:21560437-21564747
2321	ENSMUSG00000(	677044	9-:62689305-62690180
2322	Tuba4a	22145	1-:75212130-75215799
2323	Itgav	16410	2+:83564671-83643963
2324	Golga4	54214	9+:118415435-118491636
2325	H2-K1	14972	17-:34133342-34137230
2326	zfp507	668501	7-:36560979-36662496
2327	Mrc2	17534	11+:105153996-105211283
2328	Traf3ip1	74019	1+:93391254-93425949
2329	Wdr6	83669	9-:108474648-108480997
2330	Ccdc28b	66264	4-:129296518-129301144
2331	Vezt	215008	10-:93432018-93498556
2332	Nox3	224480	17-:3646475-3695583
2333	MMT00064554		
2334	Gch1	14528	14-:47775327-47809064
2335	Gsdma2	76758	11+:98508076-98519267
2336	Lpar3	65086	3+:145883959-145948924
2337	Necap2	66147	4-:140623171-140634226
2338	Olfr620	258808	7-:110759924-110760865
2339	5730449L18Rik	66637	1-:154217868-154233812
2340	Unc84a	77053	5+:139676594-139725794
2341	Pcdhb20	93891	18+:37663942-37667312
2342	MMT00009948		7-:115671545-115672576
2343	Reck	53614	4+:43953524-43955301
2344	Ogt	108155	X+:98835624-98877668

2345	Olfr667	259062	7-:112064876-112065808
2346	C630035N08Rik	109349	2-:26967521-26997995
2347	Zmynd17	74843	14-:21302091-21315905
2348	Irak1	16179	X-:71260321-71269254
2349	Ccdc73	211936	2+:104792062-104831938
2350	Ruvbl1	56505	6+:88415458-88447559
2351	1110008F13Rik	67388	2+:156688863-156699295
2352	MMT00059848		17+:3097104-3098132
2353	Snx17	266781	5+:31493980-31501341
2354	Lhx1	16869	11-:84331478-84332032
2355	1700065I16Rik	78462	15+:63648738-63653257
2356	Dnajc13	235567	9-:104053611-104140870
2357	Snta1	20648	2-:154202063-154233817
2358	5830411N06Rik	244234	7+:147408055-147486635
2359	Cc2d2a	231214	5+:44053618-44130714
2360	6330407G11Rik	68050	4-:123412602-123427504
2361	Kcnb2	98741	1+:15669745-15772717
2362	Mkks	59030	2-:136699800-136716914
2363	Stx1b	56216	7-:134950355-134968045
2364	Sprr3	20766	3-:92260740-92261457
2365	BC031181	407819	18+:75165582-75169494
2366	MMT00034174		16+:93628337-93630443
2367	Olfr342	258950	2+:36382933-36383871
2368	Heatr5b	320473	17-:79148362-79150134
2369	MMT00026815		11-:121767077-121831952
2370	8430408G22Rik	213393	6+:116600716-116602811
2371	Ptch2	19207	4+:116768960-116787436
2372	Stx12	100226	4-:132409978-132440316
2373	Ror1	26563	4+:99768823-100114848
2374	Vmn2r91	665210	17+:18242881-18273607
2375	Tnpo1	238799	13-:99614334-99619209
2376	Rnase13	497071	14-:52541893-52542355
2377	Mmd	67468	11+:90110836-90139891
2378	Bzw1	66882	1+:58449979-58463392
2379	Lyst	17101	13+:13682675-13869707
2380	MMT00065765		2-:89021616-89022284
2381	Tegt	110213	15+:99232016-99240471
2382	Usp24	329908	4+:106050113-106053588
2383	1500031H01Rik	207740	16-:4874785-4879797
2384	Micalcl	70877	7+:119517848-119556616
2385	Ctsd	13033	7-:149561821-149573775
2386	Zfp42	22702	8-:44380424-44392355
2387	Dscc1	72107	15-:54907654-54922049
2388	Dot1l	208266	10+:80217707-80256188
2389	Ifna5	15968	4+:88481428-88481997
2390	Lcn2	16819	2-:32240172-32243290
2391	Mov10l1	83456	15+:88815989-88885578
2392	Olfr551	258750	7-:109736307-109737255
2393	Centd3	106952	18-:38132278-38156760
2394	Tnc	21923	4-:63620821-63681760
2395	Csn1s2b	12992	5+:88237179-88253442

2396	Tbc1d16	207592	11-:119070431-119071996
2397	Rb1cc1	12421	1+:6220083-6264356
2398	MMT00028038		2+:3604054-3604701
2399	MMT00060167		1-:173609465-173609784
2400	Gck	103988	11-:5801673-5815014
2401	D630037F22Rik	544696	10-:55808126-55871647
2402	Cdc25b	12531	2+:131012814-131024201
2403	Cfl1	12631	19+:5490505-5494032
2404	MMT00049822		1-:113701733-113704372
2405	Adk	11534	14+:21871854-22267766
2406	Pfkm	18642	15+:97942550-97962878
2407	Pgap1	241062	1-:54543313-54550250
2408	Txndc15	69672	13+:55815998-55827591
2409	Vmn2r4	637053	3-:64192239-64219026
2410	Pnck	93843	X-:70902016-70904586
2411	MMT00039404		
2412	Rab11b	19326	17-:33883542-33897242
2413	MMT00041293		X-:91917407-91935041
2414	Prpf4b	19134	13+:34967324-34994903
2415	Rho	212541	6+:115881944-115888847
2416	EG436081	436081	9-:73023234-73025634
2417	Tex261	21766	6-:83720409-83725753
2418	9430008C03Rik	68108	2-:158181042-158187266
2419	MMT00047859		12+:68758197-68759283
2420	2010016I18Rik	69206	3-:106284902-106286465
2421	Itgb5	16419	16+:33829844-33949231
2422	Galk2	69976	2+:125691961-125810033
2423	Polr3a	218832	14-:25267780-25306231
2424	Unc5cl	76589	17+:48594246-48608007
2425	Vmn2r56	629079	7-:13298281-13318461
2426	MMT00013085		11+:92590580-92636328
2427	Anks6	75691	4-:47030394-47069992
2428	Car1	12346	3-:14766229-14808322
2429	Fjx1	14221	2-:102290437-102291745
2430	Fbxl17	50758	17-:63408817-63429912
2431	1700020L24Rik	66330	11+:83251197-83254718
2432	MMT00041434		2-:16900449-16901458
2433	Armc10	67211	5+:21151801-21168410
2434	Prr8	381626	5+:28643735-28721210
2435	MMT00017607		11+:48408777-48409310
2436	Fblim1	74202	4-:141132915-141151866
2437	Lrrc23	16977	6-:124719880-124729736
2438	Zfp759	268670	13+:67229163-67309438
2439	Pitpnm2	19679	5-:124568699-124666427
2440	1500003O22Rik	101867	7-:112880722-112885014
2441	Slc10a7	76775	8+:81033276-81257898
2442	Rab2b	76338	14-:52881440-52899222
2443	5033411D12Rik	192136	13-:16949313-17786589
2444	Nagpa	27426	16-:5195372-5204075
2445	Spopl	76857	2-:23365578-23427574
2446	Nphp3	74025	9+:103932336-103946060

2447	Ubac1	98766	2-:25854067-25877231
2448	Ltk	17005	2-:119577063-119584250
2449	Mybpc2	233199	7-:51757087-51780009
2450	Phf20l1	239510	15+:66409125-66476784
2451	MMT00029755		
2452	Obscn	380698	11-:58874345-58875740
2453	MMT00002725		7+:90904381-90906302
2454	MMT00045890		3-:108042224-108042877
2455	MMT00037845		
2456	Mrpl10	107732	11+:96902899-96910527
2457	1110032E23Rik	68659	3+:79689909-79747306
2458	Hhipl2	78772	1_random+:392595-410156
2459	9530096D07Rik	242681	4-:131858177-131859208
2460	Mctp2	244049	7-:79225674-79374356
2461	Appbp2	66884	11-:85004860-85048327
2462	2610528J11Rik	66451	4+:118199886-118202822
2463	Htr3b	57014	9-:48743982-48772927
2464	A130022J15Rik	101351	6-:97059922-97085264
2465	MMT00048312	632654	12-:115460995-115461531
2466	Alox12b	11686	11+:68970573-68983293
2467	MMT00074770		16+:32408620-32409281
2468	Slc6a14	56774	X+:21292027-21318388
2469	Coasy	71743	11+:100943919-100947931
2470	Grb7	14786	11+:98308204-98316686
2471	Ccdc134	76457	15+:81958392-81972630
2472	MMT00014056		14+:114879627-114879658
2473	4930504H06Rik	75040	12+:34079717-34086135
2474	Pot1b	72836	17-:55805709-55851928
2475	Cyp4f13	170716	17-:33061738-33102711
2476	Slc4a2	20535	5+:23931136-23946763
2477	Cldn11	18417	3+:31048912-31063241
2478	Timm50	66525	7-:29090844-29097065
2479	Cenpo	52504	12-:4213481-4233937
2480	MMT00000773		
2481	EG383341	383341	18+:35676924-35696603
2482	Mtag2	50994	7+:52622602-52623318
2483	Gmeb1	56809	4-:131907849-131909549
2484	Olfr1024	257900	2-:85739877-85740589
2485	Adck1	72113	12+:89598997-89700161
2486	Ephb1	270190	9-:101824459-101969363
2487	MMT00068537		7-:107346074-107346858
2488	Gcc2	70297	10+:57718249-57768345
2489	Npr3	18162	15-:11774933-11835394
2490	MMT00070880		17+:6806524-6812615
2491	Nhlrc3	212114	3-:53255919-53266350
2492	9930012K11Rik	268759	14-:70556465-70559238
2493	Apoh	11818	11+:108256626-108275705
2494	4933414I15Rik	74408	11-:50754321-50757259
2495	Insm2	56856	12+:56700459-56701940
2496	2410127L17Rik	67383	19+:18745384-18778697
2497	Slc43a2	215113	11+:75345211-75385995

2498	Enpp6	320981	8+:48115767-48178782
2499	Asf1a	66403	10+:53316757-53328159
2500	Vmn2r65	100009609	7-:92091650-92096161
2501	Isx	71597	8+:77396613-77417405
2502	Anapc1	17222	2-:128438442-128513116
2503	Lmbrd1	68421	1+:24685471-24821030
2504	MMT00007032	100038875	13+:91062780-91064329
2505	Morf4l2	56397	X-:133267491-133270156
2506	Gm672	269037	18-:75594582-75796966
2507	Oaf	102644	9-:43030009-43047993
2508	Adam22	11496	5-:8082097-8368076
2509	Esam1	69524	9+:37335712-37345898
2510	Olfr136	258803	17+:38472103-38473041
2511	Ltv1	353258	10-:12897947-12912946
2512	Gan	209239	8+:119682034-119729083
2513	RP23-225D24.3	383678	2+:25592576-25593314
2514	Zswim4	212168	8-:86734576-86760942
2515	Gjc2	118454	11-:58989834-58991147
2516	2210010B09Rik	244721	9+:20385935-20420351
2517	MMT00004296		4-:34335063-34337141
2518	Apold1	381823	6+:134889809-134934341
2519	Rhobtb2	246710	14-:70187010-70200626
2520	Rgn	19733	X+:20126943-20139213
2521	Olfml3	99543	3-:103539321-103541871
2522	Olfr1442	258692	19+:12748696-12749633
2523	Pgs1	74451	11+:117848193-117885324
2524	B930006L02Rik	319604	7+:107983839-107990141
2525	9230110F15Rik	77080	9-:35645912-35651736
2526	Epc1	13831	18-:6435950-6516085
2527	1600012F09Rik	67008	13-:18080036-18085171
2528	MMT00074751		10-:7528018-7528584
2529	Hmcn2	665700	2+:31235722-31316253
2530	Pphln1	223828	15+:93228827-93321929
2531	Hspbap1	66667	16+:35770503-35825649
2532	Adrbk1	110355	19-:4285999-4306035
2533	Arhgef19	213649	4+:140803761-140813467
2534	4921517D22Rik	70900	13-:59790687-59795463
2535	Tpk1	29807	6-:43295005-43616174
2536	Timp4	110595	6-:115196040-115201798
2537	MMT00072229		12-:116532265-116757993
2538	Clec4g	75863	8-:3716071-3720645
2539	0610031J06Rik	56700	3+:88128944-88132552
2540	Tmem63a	208795	1+:182872648-182905234
2541	Ppa2	74776	3+:132973079-133041031
2542	Dck	13178	5+:89194268-89212264
2543	MMT00001648	83409	3-:88353828-88356819
2544	Hnf1a	21405	5-:115398370-115421047
2545	2210023G05Rik	72361	8+:107471253-107492564
2546	Gprin3	243385	6-:59303024-59305314
2547	Prdm15	114604	16-:98015711-98021407
2548	Tead2	21677	7+:52472588-52488297

2549	MMT00038378	80795	14+:30781602-30788260
2550	MMT00036681		15-:77234219-77236051
2551	Iscu	66383	5+:114222820-114228289
2552	Stac2	217154	11-:97897937-97914682
2553	1700025K23Rik	66337	10-:38838611-38853767
2554	Sphk1	20698	11+:116396053-116397982
2555	MMT00033243		X+:73160819-73161742
2556	Sars2	71984	7+:29527014-29538873
2557	Casq1	12372	1-:174140470-174149972
2558	Tmem132b	208151	5+:126229604-126269041
2559	Ccdc18	73254	5+:108561895-108661897
2560	MMT00035872		5-:109302232-109320531
2561	RP23-24J10.7	242285	4-:3923088-3943586
2562	Nhedc2	97086	3+:134981255-135005500
2563	Olfr122	258285	17+:37908572-37909563
2564	MMT00052902		12+:50241242-50241986
2565	Zbtb41	226470	1+:141319727-141344171
2566	Pigp	56176	16-:94586059-94592319
2567	Ppef2	19023	5-:92656000-92682185
2568	AU040320	100317	4+:126430796-126531581
2569	Dsc3	13507	18-:20121878-20160598
2570	Tmod4	50874	3+:94929452-94933130
2571	Rmnd1	66084	10+:5914151-5943319
2572	Htf9c	15547	16+:18248771-18253594
2573	Speer4b	73526	5-:27822346-27827978
2574	Rexo4	227656	2-:26809077-26819841
2575	Elavl3	15571	9-:21822471-21856446
2576	Rnf19a	30945	15-:36169688-36195771
2577	MMT00076461		5-:151404492-151409458
2578	Csf2rb2	12984	15-:78112939-78136033
2579	MMT00046493		16+:50143730-50193938
2580	Ucn3	83428	13-:3940403-3940896
2581	Ufsp2	192169	8+:47060892-47082211
2582	Trappc9	76510	15-:72733986-72888977
2583	Runx1	12394	16-:92604349-92697573
2584	Serpind1	15160	16+:17336313-17343665
2585	2810474O19Rik	67246	6+:149257947-149278315
2586	Als2cl	235633	9+:110785266-110801114
2587	Speer2	224318	16-:69857117-69861969
2588	MMT00066526		18+:24102397-24102676
2589	Rxfp2	140498	5+:150821249-150883911
2590	Atp5k	11958	5-:108862313-108863408
2591	2310005N01Rik	70088	4+:124762394-124787133
2592	Slco4a1	108115	2+:180195733-180209570
2593	Tbc1d1	57915	5+:64551546-64741426
2594	Hspg2	15530	4+:137063715-137063986
2595	Acbd4	67131	11+:102964825-102973513
2596	Vsig4	278180	X-:93464386-93464955
2597	10-Sep	103080	10-:58629250-58684597
2598	Naglu	27419	11+:100931438-100938965
2599	Lsm14a	67070	7-:35129663-35174628

2600	A430093A21Rik	227195	1-:63104814-63160890
2601	Dnajc18	76594	18-:35834684-35862745
2602	MMT00008812		Y-:201176-210147
2603	Ipp	16351	4+:116183176-116193421
2604	Kremen1	84035	11-:5094291-5161561
2605	N4bp2l2	381695	5-:151449573-151465088
2606	MMT00077133		
2607	Olfr1467	258686	19+:13439119-13440045
2608	Clcn2	12724	16-:20703040-20716187
2609	Hyal4	77042	6+:24698344-24717663
2610	EG623459	623459	9-:99129887-99136776
2611	MMT00039586		6-:60411890-60412108
2612	Sf3b2	319322	19-:5273934-5295425
2613	Ubxd7	224111	16+:32332372-32387083
2614	EG546723	546723	19+:30613417-30614035
2615	EG624862	624862	14+:44842695-44843903
2616	Ptges2	96979	2+:32251777-32258259
2617	Rqcd1	58184	1+:74553011-74575427
2618	Map3k7ip3	66724	X+:82877615-82879647
2619	Zfp622	52521	15+:25914214-25927182
2620	2810410C14Rik	230582	4+:106739614-106756998
2621	Olfr30	18329	11-:58268502-58269449
2622	Timd2	171284	11-:46484397-46512067
2623	S100a6	20200	3+:90417105-90418337
2624	MMT00056820		13+:116561568-116561707
2625	MMT00077446		15-:73237740-73238725
2626	MMT00013951		4+:89940729-89940760
2627	Ccl12	20293	11+:81915379-81916793
2628	Figla	26910	6+:85967184-85970989
2629	1300018J18Rik	223776	15+:88919431-88930770
2630	Il17re	57890	6+:113408827-113420368
2631	Aldh1l1	107747	6+:90500807-90550196
2632	Aph1a	226548	3+:95697918-95702211
2633	MMT00063659		5-:110185266-110187176
2634	Sgol2	68549	1+:58052862-58082741
2635	Tmem126a	66271	7-:97599221-97605718
2636	Tars	110960	15-:11313417-11329413
2637	MMT00067068		10+:29731106-29733196
2638	Cyp3a41a	53973	5-:146505625-146531712
2639	Abhd2	54608	7+:86418161-86505287
2640	EG383462	383462	19-:34130631-34135718
2641	Bfar	67118	16+:13672086-13702794
2642	Neurod2	18013	11-:98187852-98190775
2643	Inhbe	16326	10-:126786457-126788828
2644	Srrm2	75956	17+:23955853-23996744
2645	Gramd1c	207798	16-:43983239-44030484
2646	Ugt2b37	112417	5-:87669517-87683829
2647	Lmln	239833	16+:33062529-33117325
2648	MMT00005098		6+:22683708-22684595
2649	Nfs1	18041	2-:155967764-155969915
2650	Spo11	26972	2+:172805345-172819059

2651	Ttc7	225049	17+:87681989-87781713
2652	Immp2l	93757	12+:41751159-43056229
2653	EG434394	434394	9-:35089103-35089420
2654	2310066E14Rik	75687	8+:108117878-108140924
2655	Adamdec1	58860	14-:69181444-69200129
2656	Polrmt	216151	10-:79198869-79209308
2657	Ints6	18130	14-:63311853-63363269
2658	Fzd2	57265	11+:102465848-102467886
2659	Kcng2	240444	18-:80492127-80519384
2660	4930558K02Rik	75368	1-:163872221-163899285
2661	Eif4g2	13690	7-:118215446-118226486
2662	Sgpl1	20397	10-:60563471-60610346
2663	Dlg7	218977	14-:48007454-48038082
2664	Frem2	242022	3-:53320638-53461446
2665	Serf1	20365	13+:100878002-100884186
2666	Thoc7	66231	14-:14781529-14793752
2667	Pgam1	18648	19+:41986508-41993149
2668	C3ar1	12267	6-:122797991-122806021
2669	Ier2	15936	8-:87185236-87186752
2670	Igsf1	209268	X-:47135720-47147957
2671	Sh3rf1	59009	8+:63703068-63872992
2672	Soat1	20652	1-:158359923-158396931
2673	Zfp341	228807	2+:154447488-154472547
2674	1810005K13Rik	66255	18-:80426804-80433600
2675	Nfyc	18046	4-:120430782-120463086
2676	Cables2	252966	2-:179994465-180008170
2677	2310057J18Rik	67719	10-:28692170-28706109
2678	Rbm9	93686	15-:76914592-76983986
2679	BC067047	277360	2-:166392240-166539218
2680	4930485B16Rik	380654	10-:92479179-92488296
2681	Hectd1	207304	12-:52844708-52847097
2682	Gjb4	14621	4-:127028590-127029390
2683	Tpst2	22022	5+:112732919-112744375
2684	MMT00053809		
2685	Olfr535	258956	7+:147678538-147679475
2686	Sod1	20655	16+:90220990-90246462
2687	Hdac3	15183	18-:38096632-38114582
2688	V1re10	171263	7+:11032159-11033075
2689	Olfr611	258722	7-:110665926-110666896
2690	Prokr1	58182	6-:87531213-87538855
2691	Ube2d3	66105	3+:135101722-135129022
2692	MMT00020697		1-:13776760-13777028
2693	Asprv1	67855	6+:86578720-86579694
2694	Siah2	20439	3-:58478877-58496025
2695	1700008I05Rik	71841	X-:132189243-132228330
2696	4930422I07Rik	71640	9+:88376847-88465328
2697	MMT00043090		13+:19302211-19308885
2698	Defcr22	382059	8+:22301998-22302175
2699	Hax1	23897	3-:89799378-89802574
2700	MMT00001689		5-:17034487-17034911
2701	Mllt6	246198	11+:97524727-97543842

2702	MMT00070915		13-:76375711-76389252
2703	MMT00073763		1+:163676198-163677877
2704	Lrrc49	102747	9-:60435045-60535708
2705	Hrbl	231801	5-:138104053-138125921
2706	9030625A04Rik	210808	14-:77433131-77436339
2707	Defb39	360214	8-:19037369-19064810
2708	Sgk1	20393	10+:21714500-21719695
2709	BC042720	329178	1+:66514919-66556142
2710	EG666451	666451	14+:44503031-44504250
2711	5033411D12Rik	192136	13-:17344318-17415126
2712	MMT00007399		8-:64965610-64967124
2713	Six1	20471	12-:74142817-74147687
2714	1700001K19Rik	66323	12-:111905926-111920822
2715	Derl1	67819	15-:57703082-57723998
2716	BC014805	236149	19-:7856474-7877157
2717	MMT00076977		5+:144217636-144218441
2718	Scrn3	74616	2+:73150627-73173922
2719	St6galnac2	20446	11-:116538018-116555939
2720	BC053749	333193	7-:31328766-31337287
2721	Inmt	21743	6-:55120620-55124984
2722	Abca5	217265	11-:110133563-110198989
2723	2410091C18Rik	73694	17+:79344234-79346568
2724	Tpsab1	17230	17-:25480190-25482338
2725	Gm1614	381148	18-:35811820-35814853
2726	Chd9	109151	8+:93513353-93578409
2727	Stc2	20856	11-:31260080-31269908
2728	Exoc4	20336	6+:33199123-33922332
2729	Dcun1d4	100737	5+:73875636-73951753
2730	Bcor	71458	X-:11613868-11638772
2731	EG434080	434080	6+:116501152-116502599
2732	MMT00027806		18-:31508540-31510049
2733	Tbc1d12	209478	19+:38988469-38993095
2734	Hfm1	330149	5-:107324214-107332092
2735	Olfr1006	258563	2-:85514368-85515330
2736	Thoc3	73666	13-:54560878-54570180
2737	Mllt10	17354	2+:17986313-18132597
2738	2510039O18Rik	77034	4+:147315003-147321423
2739	MMT00046037		17-:23404485-23412778
2740	Btnl4	632126	17-:34606513-34631428
2741	3230401D17Rik	66680	2-:163231672-163245225
2742	Zbed3	72114	13+:96095191-96107796
2743	Tcf3	21415	6-:72577163-72738950
2744	Mtus1	102103	8-:42133392-42169875
2745	Pycrl	66194	15-:75746906-75751986
2746	MMT00013076		3-:112281372-112281883
2747	Enc1	13803	13+:98011206-98020697
2748	Syt16	238266	12+:75335795-75368901
2749	Naf1	234344	8+:69384156-69414459
2750	MMT00062940		8-:53363981-53364825
2751	Lrriq1	74978	10-:102633054-102698956
2752	Fcgr1	14129	3-:96088140-96097886

2753	Myo6	17920	9+:80012911-80157198
2754	Zc3hav1l	209032	6-:38245057-38249315
2755	EG382421	382421	10+:110937798-110938837
2756	Pmvk	68603	3+:89263237-89272928
2757	EG435927	435927	6-:141684625-141693021
2758	Zscan29	99334	2-:120919325-120996844
2759	V1rj2	171260	7+:13804687-13805637
2760	Dleu2	328425	14-:62221727-62301186
2761	P2rx3	228139	2-:84838741-84875644
2762	Obscn	380698	11-:58919796-58943347
2763	MMT00046393		8+:131157882-131158791
2764	Ffar2	233079	7-:31604049-31605344
2765	Zfp36l3	333473	X-:51077245-51079422
2766	Elp4	77766	2-:105542599-105744652
2767	MMT00031206		6+:57138500-57139337
2768	Oprd1	18386	4-:131668286-131700312
2769	AI606181	226118	19+:41668209-41670648
2770	Fbl	14113	7+:28954778-28964268
2771	Keg1	64697	19+:12770282-12794384
2772	Crtc2	74343	3+:90058209-90068048
2773	Cdk5r1	12569	11+:80290867-80292181
2774	Tmem171	380863	13-:99456193-99462746
2775	Sec61a2	57743	2-:5792855-5816331
2776	Magel2	27385	7+:69524349-69526524
2777	Cntnap5c	620292	17+:58453099-58498744
2778	Coro1a	12721	7-:133843394-133846687
2779	MMT00061842		1+:118643188-118719334
2780	Fes	14159	7-:87522641-87532846
2781	Lamb2	16779	9+:108382233-108392792
2782	Pou3f2	18992	4-:22413941-22415278
2783	Pdzrn4	239618	15+:92427591-92511021
2784	4933415F23Rik	66755	1-:23107345-23109092
2785	Mtf2	17765	5+:108516037-108536004
2786	Mrps7	50529	11+:115465332-115469458
2787	2310011J03Rik	66374	10-:79781454-79783276
2788	Galnt10	171212	11+:57583010-57599560
2789	Sirt5	68346	13+:43460868-43490571
2790	MMT00004640	328362	14-:25883679-25909807
2791	4933421O10Rik	71073	4-:33115795-33119205
2792	Trim11	94091	11+:58791617-58804959
2793	MMT00005644	497266	
2794	Map3k6	53608	4+:132796732-132808843
2795	Olfr478	258729	7-:115174910-115175855
2796	Tmem19	67226	10-:114777797-114799301
2797	AB041806	58213	4+:137951112-137953615
2798	Arhgef11	213498	3+:87487407-87542077
2799	Dsc1	13505	18-:20243946-20273194
2800	Dusp11	72102	6-:85897507-85900556
2801	H2-Bl	14963	17-:36216993-36221404
2802	Lin7c	22343	2+:109731034-109737862
2803	Faah	14073	4-:115671092-115690262

2804	Atpbd3	233189	7+:50927400-50933665
2805	Rhog	56212	7-:109388183-109388758
2806	Mtap7d2	78283	X+:155881854-155934215
2807	Cdh4	12561	2+:179177271-179631110
2808	Spesp1	66712	9-:62118535-62129986
2809	Hoxd11	15431	2+:74520449-74522194
2810	Olfr1427	258674	19-:12166567-12173992
2811	MMT00062390		7+:144002509-144004197
2812	MMT00031955		18+:80359546-80360255
2813	Mrpl55	67212	11+:59016018-59019504
2814	Olfr410	258702	11-:74147784-74148731
2815	Eif2ak3	13666	6+:70794528-70855231
2816	Klhdc3	71765	17-:46811502-46817853
2817	MMT00079184		5-:89928184-89929004
2818	Egr1	13653	18+:35020859-35024635
2819	Kcnk5	16529	14-:20960805-21000674
2820	Plekha7	233765	7-:123307545-123314351
2821	Bub1b	12236	2+:118423991-118467326
2822	Gckr	231103	5+:31599953-31629673
2823	Sipa1l2	244668	8-:127941972-128016496
2824	Btnl6	624681	17-:34645826-34654308
2825	Syne1	64009	10+:5081309-5085648
2826	Pdcd1	18566	1-:95934883-95949130
2827	Raet1d	56554	10+:22082079-22093806
2828	Fndc8	78919	11+:82705623-82714301
2829	Lrfn3	233067	7-:31140503-31145817
2830	4632417K18Rik	107373	19+:12648014-12664186
2831	Hrh2	15466	13+:54309375-54310450
2832	2810021J22Rik	69944	11+:58680716-58693934
2833	Rplp2	67186	7+:148633554-148637484
2834	Crim1	50766	17+:78743688-78775925
2835	Gpr120	107221	19+:38171568-38188749
2836	Wdfy4	545030	14-:33779543-33960284
2837	H2afz	51788	3+:137528090-137529884
2838	Stk25	59041	1-:95518484-95531962
2839	Thoc2	331401	X-:39165224-39265048
2840	Adh6-ps1	71749	3+:138044791-138051255
2841	Kcne4	57814	1+:78813523-78816599
2842	Cox6b2	333182	7-:4691673-4704630
2843	MMT00065823		7-:26789618-26800164
2844	Slc25a12	78830	2-:71112325-71205785
2845	Lce1d	69611	3-:92489420-92491132
2846	MMT00002427		18+:3986542-4011157
2847	Pscd4	72318	15+:78449725-78452443
2848	Serpine2	20720	1-:79790897-79855211
2849	EG227054	227054	1+:46038392-46038858
2850	Slc16a6	104681	11-:109314053-109334523
2851	Inpp5e	64436	2-:26252027-26264701
2852	Nab1	17936	1-:52515863-52557170
2853	Glipr2	384009	4+:43970561-43991988
2854	Lingo2	242384	4-:35950865-36150286

2855	D330050I23Rik	399603	15-:60654517-60655450
2856	Tpd52	21985	3-:8929597-9004444
2857	MMT00046819		5-:100942162-100942648
2858	Arhgap20	244867	9+:51593840-51661960
2859	Ccdc63	330188	5-:122558047-122572757
2860	Pfn3	75477	13-:55516049-55516577
2861	Gucy2d	14918	7+:105592117-105625801
2862	MMT00068885		14-:79365696-79366375
2863	MMT00007006		12-:119996497-119999465
2864	Pmepa1	65112	2-:173053322-173101642
2865	Sycp2	320558	2-:178080001-178142390
2866	Clk1	12747	1-:58468844-58480848
2867	MMT00001941		6-:39792201-39794334
2868	Hbp1	73389	12-:32611342-32635100
2869	Copa	12847	1+:174012669-174052460
2870	D5Ertd577e	320549	5+:95947713-95951732
2871	Mageb1	17145	X-:89260529-89261672
2872	EG383032	383032	15-:54143476-54143920
2873	Usp6nl	98910	2+:6265924-6365582
2874	Nol3	78688	8+:107800401-107803949
2875	Wfdc2	67701	2+:164388215-164394006
2876	4930402E16Rik	319518	8+:113618555-113663677
2877	Pcyt2	68671	11-:120471401-120479198
2878	MMT00006672	100046475	13+:22909126-22909829
2879	Ptprv	13924	1-:137005090-137023649
2880	Cd93	17064	2-:148262387-148269280
2881	Hlf	217082	11-:90197850-90207295
2882	MMT00052707		
2883	MMT00048540		17-:90309276-90379134
2884	Shc3	20418	13-:51526409-51662453
2885	Hebp2	56016	10-:18260571-18265814
2886	Rps15a	267019	7-:125252472-125273136
2887	Tpo	22018	12-:30739526-30816759
2888	Guca2a	14915	4+:119310336-119312070
2889	Pqlc2	212555	4-:138854445-138866582
2890	Ccdc62	208908	5+:124380707-124386564
2891	MMT00069002		8-:74227847-74228867
2892	MMT00046974	668030	2-:175248476-177463046
2893	Lias	79464	5+:65782803-65800192
2894	Gm1499	385109	6-:69176840-69193456
2895	2410076I21Rik	73673	9-:58500635-58589403
2896	Mbtps2	270669	X-:153988863-154014120
2897	MMT00019125		X-:89133227-89134904
2898	Sema4b	20352	7+:87343538-87370309
2899	Psmb7	19177	2-:38443565-38499426
2900	6330416L07Rik	319615	17-:22475663-22498377
2901	Tmem132d	243274	5-:128263790-128276473
2902	D4Ertd196e	52174	4-:132821961-132833702
2903	Pacrg	69310	17-:10969999-11033054
2904	Gzme	14942	14-:56736460-56739393
2905	4833413D08Rik	71425	2+:154093588-154095246

2906	EG545886	545886	6-:133714015-133717433
2907	MMT00010854		3+:100217482-100218699
2908	Zfy2	22768	Y-:1362123-1426318
2909	Cfdp1	23837	8-:114292372-114378210
2910	MMT00024586		13-:25772646-25868662
2911	Lphn1	330814	8+:86455019-86465844
2912	Wtip	101543	7-:34894583-34918078
2913	Actn1	109711	12-:81291564-81295430
2914	Slc13a2	20500	11-:78210777-78235687
2915	Nfs1	18041	2-:155949385-155969921
2916	4931423N10Rik	70981	2+:23062993-23122638
2917	Scly	50880	1+:93194962-93217639
2918	Mtmr10	233315	7+:71432585-71485693
2919	Ptprn2	19276	12+:118062776-118139520
2920	4922501C03Rik	382090	9+:87084408-87085910
2921	Kctd8	243043	5-:69501575-69559830
2922	Scamp4	56214	10+:80072070-80077528
2923	Atg4a	666468	X+:137593268-137598807
2924	Actr1a	54130	19-:46451303-46470225
2925	Prkd3	75292	17-:79371615-79408028
2926	Eif3i	54709	4-:129269226-129277861
2927	AY616753	433899	5+:68501619-68559057
2928	Igf1	16000	10+:87324073-87393619
2929	2510009E07Rik	72190	16-:21653274-21694507
2930	Rasef	242505	4-:73386095-73408866
2931	Vwc2	319922	11+:11014745-11054295
2932	Slco1a1	28248	6-:141856373-141895315
2933	Ptrf	19285	11-:100819825-100831886
2934	MMT00080840	57316	11+:17157593-17167233
2935	Slc28a1	434203	7+:88269753-88313948
2936	MMT00059870		16+:74961436-74962424
2937	Robo2	268902	16-:73895320-74047182
2938	H2-T23	15040	17-:36166923-36169646
2939	Dnahc17	69926	11-:117884264-117990533
2940	Gramd1b	235283	9-:40105460-40107681
2941	Olfr957	258740	9-:39318367-39319303
2942	Olfr69	18370	7-:110915959-110916909
2943	Capn10	23830	1+:94831430-94844517
2944	Zyx	22793	6+:42300255-42308393
2945	MMT00005190		X-:131362702-131363503
2946	Tmem199	195040	11-:78320525-78325631
2947	Bcl2a1b	12045	9+:89094066-89102664
2948	Srcrb4d	109267	5-:136436093-136450347
2949	MMT00076941		14+:45140310-45141329
2950	Micall1	27008	15+:78945434-78967328
2951	Anxa10	26359	8-:64536318-64601890
2952	Adamts17	233332	7+:74215474-74223274
2953	Zfp770	228491	2-:114021204-114027169
2954	AI317395	215929	10-:39722405-39744978
2955	Cdc20b	238896	13+:113868314-113873623
2956	Wbscr16	94254	5-:134623945-134652637

2957	Slc24a3	94249	2+:145274241-145442449
2958	Grik4	110637	9-:42328494-42496180
2959	Rgs3	50780	4+:62350531-62363279
2960	Map3k7	26409	4+:32051091-32108278
2961	Cenpi	102920	X+:130842715-130897178
2962	AW146154	101835	7-:48722734-48754789
2963	Tmem64	100201	4+:15193310-15213871
2964	Hn1	15374	11-:115358668-115375684
2965	Fgf15	14170	7+:152082436-152086854
2966	Mobkl1b	232157	6+:83276044-83292799
2967	Igh	111507	12-:116081854-116170993
2968	Rab23	19335	1+:33780675-33796243
2969	Ptpro	19277	6+:137359257-137411751
2970	MMT00022996		10+:18821380-18822122
2971	Slc17a2	218103	13+:23902623-23914627
2972	Zfp3	193043	11+:70577859-70586432
2973	4-Sep	18952	11+:87394632-87404042
2974	Trim28	21849	7+:13609509-13616378
2975	Gfi1b	14582	2-:28464977-28477461
2976	Zfp606	67370	7+:13063608-13080894
2977	MMT00013814		
2978	St6galnac1	20445	11-:116626339-116627586
2979	MMT00007569		4-:114911355-114911940
2980	Slc37a2	56857	9-:37037898-37063215
2981	Khdrbs1	20218	4-:129393055-129419504
2982	Mdfic	16543	6+:15670667-15720492
2983	MMT00061530		X-:149343315-149344844
2984	MMT00029895		13-:50710320-50711479
2985	Map2k1	26395	9-:64033594-64101139
2986	Pkig	18769	2+:163519773-163551888
2987	Ppapdc3	227721	2+:31951239-31966333
2988	MMT00004204		4+:40016268-40018020
2989	Zfp36l1	12192	12-:81208994-81213931
2990	EG384179	384179	5-:44335092-44335761
2991	Brcc3	210766	X+:72662042-72696482
2992	Msi1	17690	5+:115879693-115904211
2993	Gm1752	385658	16-:55843302-55895391
2994	Cldn7	53624	11+:69779428-69781218
2995	MMT00058834		3-:43567634-43568688
2996	Pik3r1	18708	13-:102450519-102538126
2997	Tnrc6b	213988	15+:80753493-80760448
2998	Lzic	69151	4+:148859401-148870773
2999	BC061194	381350	2+:18620696-18671374
3000	5330431N19Rik	226162	19+:45646372-45652771
3001	Tsga8	60600	X-:80732016-80733263
3002	Abhd12	76192	2-:150658231-150668306
3003	2310008H04Rik	224008	16-:15889319-15936787
3004	Calcr	12311	6-:3635733-3714602
3005	MMT00048199		1+:90945548-90945850
3006	Alas2	11656	X+:146981966-147005166
3007	Ints12	71793	3+:132754898-132772507

3008	Nxt1	56488	2+:148498409-148501499
3009	Serpina1c	20702	12-:105133137-105137037
3010	AU042671	269700	5+:121756950-121765157
3011	EG13909	13909	8+:107607669-107617467
3012	Actl6a	56456	3+:32607647-32625880
3013	4930434E21Rik	381693	5+:150332263-150402484
3014	Rfwd3	234736	8-:113794844-113812032
3015	Arhgef6	73341	X-:54484663-54591865
3016	Bsdc1	100383	4+:129138915-129165740
3017	Fas	14102	19+:34365155-34402260
3018	Sap30l	50724	11+:57615633-57623720
3019	Frmpd1	666060	4+:45242706-45298809
3020	C030037D09Rik	193280	11+:88580108-88588954
3021	Gdf9	14566	11+:53246879-53251402
3022	Als2cr12	108812	1-:58714959-58752806
3023	EG382723	382723	13-:8939578-8940223
3024	Ptger1	19216	8+:86190538-86194002
3025	Dmrt1	50796	19+:25580214-25677839
3026	D10Ertd610e	52666	10-:126619581-126627091
3027	MMT00030402		8-:4812818-4813104
3028	Uhrf1	18140	17+:56442848-56462908
3029	Ybx1	22608	4-:118950838-118966413
3030	Dppa2	73703	16+:48310534-48319717
3031	Rab10	19325	12-:3249734-3309662
3032	Ccnj	240665	19+:40906509-40920587
3033	Kctd16	383348	18+:40390578-40484222
3034	D11Wsu99e	28081	11-:113522633-113545346
3035	Cd99l2	171486	X-:68673246-68745820
3036	Slamf9	98365	1+:174405535-174408538
3037	MMT00017312		17-:15712487-15714358
3038	Serpina12	68054	12-:105266978-105282653
3039	S100a11	20195	3+:93324410-93330219
3040	Bcl3	12051	7-:20393811-20408064
3041	2410004A20Rik	66991	9+:72950266-72952250
3042	Xrra1	446101	7+:107023664-107066330
3043	Otop1	21906	5+:38668654-38685787
3044	Rnf219	72486	14-:104901605-104921862
3045	Alx3	11694	3+:107397948-107408687
3046	6430573F11Rik	319582	8+:37521311-37576010
3047	Cyp2d26	76279	15-:82620536-82624675
3048	Atxn1l	52335	8-:112254993-112261594
3049	MMT00005322		6-:14031328-14089851
3050	Olfr900	258874	9+:38185467-38186417
3051	Ywhah	22629	5+:33361539-33370614
3052	9130404D08Rik	74549	8-:72542479-72566599
3053	Ube2w	66799	1-:16558759-16609419
3054	Hnrnpa1	15382	15+:103070935-103075386
3055	Pde4c	110385	8+:73247962-73274318
3056	Dbil5	13168	11+:76031129-76032832
3057	2610209M04Rik	66618	6-:86625145-86634511
3058	Ccdc123	72140	7+:36182074-36188189

3059	Tctex1d2	66061	16+:32419829-32429184
3060	Slc12a6	107723	2+:112124844-112203312
3061	Scarf2	224024	16+:17802608-17834411
3062	Ubr1	22222	2-:120687381-120796451
3063	MMT00069003		8+:74219425-74220254
3064	Rnd2	11858	11+:101329651-101332619
3065	Prmt8	381813	6-:127639699-127718838
3066	MMT00035562	545618	4-:44765021-44766010
3067	Ucp3	22229	7+:107627975-107633616
3068	Med26	70625	8-:75018157-75073665
3069	Slitrk2	245450	X+:63906180-63909619
3070	Scgb3a2	117158	18+:43923980-43927047
3071	Tmed10	68581	12-:86684378-86715615
3072	MMT00002308		1-:13397087-13397792
3073	Ptgfrn	19221	3-:100847215-100914089
3074	EG545936	545936	7+:25623316-25624096
3075	EG629203	629203	7-:14652905-14708339
3076	MMT00078762		14+:53186873-53187733
3077	Ppfibp1	67533	6+:146837182-146980547
3078	9630033F20Rik	319801	6-:127037487-127059512
3079	Gstt3	103140	10-:75236865-75243787
3080	MMT00014480		15+:60575413-60578213
3081	Dhx38	64340	8-:112071909-112089761
3082	Olfr1044	259013	2-:86011028-86011972
3083	Cpne7	102278	8+:125641281-125659084
3084	Ly6d	17068	15-:74592485-74593997
3085	1110014N23Rik	68505	19-:6067988-6077203
3086	Tubg2	103768	11+:101017226-101023100
3087	Tmem30c	71027	16-:57266330-57292978
3088	Ftl2	14337	4+:124787922-125182221
3089	Eif2s3y	26908	Y+:347054-365035
3090	Gna14	14675	19+:16511021-16684297
3091	MMT00035674		19-:11951133-11953157
3092	Crygd	12967	1-:65108416-65109983
3093	Egflam	268780	15-:7156120-7348390
3094	Clec5a	23845	6-:40527770-40535771
3095	4833426J09Rik	382051	8+:107033260-107119017
3096	Ugt1a6a	94284	1+:90031383-90115578
3097	Ppp1r3g	76487	13+:36035371-36061510
3098	MMT00081523		6-:128926735-128927990
3099	MMT00022741		3+:122564795-122565292
3100	MMT00082603		2+:38478445-38478784
3101	MMT00080431		7-:7013153-7015975
3102	2610510H03Rik	68215	2+:117075374-117093683
3103	MMT00062748	625360	Un_random-:688-21009
3104	4933411G11Rik	330228	5+:143953957-143966747
3105	Trib1	211770	15-:59478736-59479702
3106	Ecel1	13599	1-:89044492-89051600
3107	Hspb3	56534	13-:114453103-114453867
3108	Dhx36	72162	3-:62271790-62310902
3109	Tert	21752	13+:73764448-73786489

3110	MMT00024045		3+:13429560-13466794
3111	Ddefl1	230837	4+:135762433-135801229
3112	Sgca	20391	11-:94824521-94837641
3113	Vti1b	53612	12-:80257262-80273332
3114	Xiap	11798	X+:39446904-39458678
3115	1700013H16Rik	75514	X-:51046077-51060991
3116	Gm1574	380842	13+:46380746-46394099
3117	Atxn1	20238	13-:45651834-46051866
3118	9130404H23Rik	74556	17-:66904593-66943962
3119	Mapk10	26414	5-:103341387-103640096
3120	D330038O06Rik	330788	8-:72268358-72268582
3121	Ptgds2	54486	6-:65069812-65094902
3122	Chia	81600	3+:105918209-105933587
3123	MMT00012256		13+:19504663-19505409
3124	Olfr1128	258346	2-:87384764-87385699
3125	Olfr837	258558	9+:18941438-18942376
3126	Grm4	268934	17-:27563728-27640110
3127	Plcd1	18799	9-:118980647-119002614
3128	Clec12b	71183	6-:129325531-129335521
3129	MMT00078028		19+:33897397-33899560
3130	Mrpl20	66448	4+:155177726-155182938
3131	Pkd1l2	76645	8-:119589421-119592163
3132	5730446D14Rik	66640	6+:52116563-52118757
3133	Olfr1046	258575	2-:86056916-86057865
3134	Usp11	236733	X+:20289265-20297663
3135	Gcdh	270076	8-:87410489-87417601
3136	Itfg1	71927	8-:88242438-88364671
3137	Magef1	76222	16-:21332668-21333430
3138	Espl1	105988	15+:102126691-102154789
3139	Gba2	230101	4-:43579792-43591409
3140	Ptpn20	19256	14+:34416291-34453940
3141	Tmf1	232286	6-:97105257-97129094
3142	Rdh12	77974	12+:80309902-80323649
3143	Slc17a6	140919	7+:58877375-58924852
3144	Ube2j2	140499	4+:155323115-155331473
3145	Idi1	319554	13+:8885230-8891614
3146	Hace1	209462	10+:45369686-45429800
3147	Higd2a	67044	13+:54691591-54692493
3148	Ankrd49	56503	9-:14584652-14587408
3149	1190005I06Rik	68918	8-:123132502-123152417
3150	Syne2	319565	12+:77167499-77211887
3151	Zhx2	387609	15+:57526241-57594276
3152	Ccdc92	215707	5-:125315348-125342524
3153	Zdhhc15	108672	X-:101735455-101793528
3154	Mcm5	17218	8+:77633522-77651587
3155	MMT00039886		3+:101878553-101878995
3156	Usp13	72607	3+:32716612-32834476
3157	BC003940	192173	11-:120404200-120412448
3158	Smc2	14211	4+:52482785-52501129
3159	Pld1	18805	3+:27837544-28032284
3160	Ap1gbp1	217030	11+:83777855-83855578

3161	Gtf2h4	14885	17-:35804683-35810625
3162	Lep	16846	6+:29018951-29021181
3163	MMT00039919		13-:8028796-8030700
3164	Hsd11b1	15483	1-:195047886-195090214
3165	Cntn3	18488	6-:102115143-102414660
3166	Vmn2r57	269902	7-:48655329-48683582
3167	Dym	69190	18+:75216372-75446618
3168	Camk2a	12322	18+:61123207-61144517
3169	Cdgap	12549	16-:38603353-38712789
3170	Mcart1	230125	4-:45411210-45421633
3171	Smpd1	20597	7+:112702928-112706895
3172	1810007E14Rik	53906	2+:118598509-118603900
3173	Glycam1	14663	15-:103393188-103395512
3174	Acyp1	66204	12-:86613355-86621385
3175	Sulf2	72043	2-:165899649-165981105
3176	MMT00004863		1-:154575960-154577890
3177	Setd8	67956	5+:124889948-124901388
3178	Kcnb1	16500	2-:166929853-167014123
3179	Rbm24	666794	13+:46466592-46538402
3180	Tmprss9	432478	10+:80350128-80362084
3181	MMT00050022		5-:123147199-123147637
3182	Stau1	20853	2-:166773772-166821799
3183	Eraf	170812	
3184	Nkx2-9	18094	12-:57712385-57714261
3185	MMT00018567		2-:22874724-22875187
3186	Kcnj16	16517	11+:110829345-110888155
3187	Syne2	319565	12+:76919320-76994231
3188	Plp1	18823	X+:133357289-133372867
3189	Ndst2	17423	14-:21542962-21549549
3190	Anxa4	11746	6-:86686840-86743550
3191	Lamc2	16782	1-:154969888-155033148
3192	2310007H09Rik	76429	7+:139802326-139898336
3193	Shc1	20416	3+:89222582-89232366
3194	Dnahc8	13417	17+:30807641-30814219
3195	Mypn	68802	10-:62578538-62656030
3196	Tat	234724	8+:112514356-112523700
3197	Olfr323	258373	11-:58438574-58439546
3198	Fbxw4	30838	19-:45652746-45734683
3199	Olfr259	258766	2-:86947604-86948542
3200	Dhodh	56749	8-:112117378-112132278
3201	Ptcd2	68927	13-:100089605-100114651
3202	Boc	117606	16-:44485154-44520616
3203	Dnahc14	240960	1+:183571355-183744322
3204	Gm24	195236	13+:22073068-22076348
3205	Ankib1	70797	5-:3692264-3803125
3206	Gpr160	71862	3+:30754883-30796089
3207	MMT00020721		16+:95324010-95324804
3208	4930511I11Rik	67645	17+:28667805-28676052
3209	Lmna	16905	3-:88287127-88297234
3210	Gm239	237558	10-:116213587-116214511
3211	MMT00046813		1+:144651732-144653209

3212	Lrrtm1	74342	6+:77192711-77195505
3213	Prdx6	11758	1-:163171140-163181296
3214	Dnmt3b	13436	2+:153475338-153513462
3215	Efcab7	230500	4+:99496190-99585386
3216	Neurog3	11925	10+:61596210-61596854
3217	Setx	269254	2+:28981401-28994959
3218	Grap2	17444	15+:80453912-80480727
3219	Vps13a	271564	19-:16754499-16855407
3220	Ap2s1	232910	7+:17328567-17334361
3221	Mill1	266815	7+:18847759-18850227
3222	Zbtb10	229055	3+:9251357-9285327
3223	Ak2	11637	4+:128670545-128688054
3224	Trpc7	26946	13-:56874468-56996949
3225	Tmem178	68027	17+:81343971-81401150
3226	Tnfrsf17	21935	16+:11313901-11320161
3227	Zfp462	242466	4+:55024171-55036479
3228	Saa1	20208	7-:53996074-53997892
3229	Tdo2	56720	3-:81762706-81779637
3230	Sh3bgrl	56726	X+:106290892-106356950
3231	1700022A21Rik	72252	5+:24151271-24154678
3232	Rrh	20132	3-:129511718-129525421
3233	Skp1a	21402	11+:52045572-52059873
3234	Scn4a	110880	11-:106180617-106210704
3235	BC029214	227622	2-:25310643-25316613
3236	Tcfap4	83383	16-:4544661-4559720
3237	Kcnj1	56379	9+:32180181-32205140
3238	Ppp3ca	19055	3+:136460678-136600692
3239	Zfp294	78913	16-:87376923-87432780
3240	Mex3a	72640	3+:88339590-88341488
3241	Gm765	330390	6-:98188084-98198226
3242	2700049A03Rik	76967	12+:72238207-72344284
3243	Ambn	11698	5+:88885035-88897552
3244	Hist1h4a	326619	13-:23852716-23853026
3245	Zfp92	22754	X+:70656434-70669490
3246	Prei4	74182	2-:132355135-132376184
3247	MMT00011992	100045918	19-:12303010-12303680
3248	MMT00058895		3-:49396836-49436888
3249	Pnpla8	67452	12+:45370172-45413274
3250	Olfm1	56177	2+:28061195-28085980
3251	Thg1l	66628	11-:45760347-45768989
3252	Kbtbd4	67136	2+:90744942-90750717
3253	Tbc1d20	67231	2+:152119604-152139732
3254	Grifin	77998	5-:141039077-141041023
3255	Fgf11	14166	11-:69611704-69615127
3256	Phox2b	18935	5-:67486914-67490394
3257	Map1lc3a	66734	2+:155102079-155103807
3258	Ascl1	17172	10-:86954651-86955910
3259	Chrna6	11440	8-:28514848-28524254
3260	2900083I11Rik	58212	5+:136282759-136317836
3261	Rpap2	231571	5+:108026753-108090853
3262	Mpa2l	100702	5-:105601034-105611307

3263	MMT00013139		
3264	1810009A15Rik	66276	19+:8963391-8965230
3265	Dntt	21673	19+:41103877-41134004
3266	Olfr347	258945	2+:36589842-36590779
3267	Vps8	209018	16+:21495949-21644748
3268	Olfr1366	258280	13-:21628886-21629827
3269	Ylpm1	56531	12+:86337268-86411543
3270	Ttc12	235330	9-:49252771-49253987
3271	Dis3	72662	14-:99475442-99498979
3272	Akap8	56399	17-:32441600-32458086
3273	MMT00081689		12-:104982240-104983199
3274	Evx1	14028	6+:52263491-52268371
3275	OTTMUSG00000(	627585	4+:145222406-146035579
3276	MMT00075816		1+:5053614-5054041
3277	MMT00038655		
3278	Ggt7	207182	2-:155316122-155340563
3279	Slc46a1	52466	11+:78279176-78285445
3280	Tlr1	21897	5-:65316072-65323938
3281	Slc12a3	20497	8+:96853125-96889863
3282	Ndufs6	407785	13-:73457318-73465924
3283	Mmp9	17395	2+:164773756-164780405
3284	Plekha7	233765	7-:123298142-123301893
3285	MMT00023436		1-:49456218-49456550
3286	Alox5ap	11690	5+:150076637-150099722
3287	MMT00036232		5+:109588434-109597282
3288	Slc26a6	171429	9+:108757068-108815373
3289	Adamtsl4	229595	3-:95480126-95483599
3290	2900042B11Rik	72938	4+:106926498-106952537
3291	Ptk2	14083	15-:73035535-73225154
3292	MMT00014416		4-:63323392-63323872
3293	Nup153	218210	13-:46775254-46823638
3294	Prss23	76453	7-:96657618-96666054
3295	4930534B04Rik	75216	12-:92452004-92505454
3296	Olfr1143	258290	2+:87642547-87643490
3297	Trappc4	60409	9-:44211851-44215590
3298	Man2a1	17158	17+:64950988-65104450
3299	EG243431	243431	6-:67892044-67892534
3300	Commd4	66199	9-:57002847-57006106
3301	Zcchc14	142682	8-:124123367-124175833
3302	Dock1	330662	7+:141862388-141996340
3303	B430119L13Rik	320974	2-:132695451-132702944
3304	Tle2	21886	10+:81038276-81053590
3305	Gdnf	14573	15+:7761010-7787574
3306	Rps6kb2	58988	19-:4156976-4163245
3307	Cd8b1	12526	6+:71272805-71287445
3308	Satb2	212712	1-:56853110-57028172
3309	Grpel2	17714	18-:61874372-61885977
3310	Tdrd5	214575	1-:158230724-158232736
3311	MMT00031321		7+:127648843-127657879
3312	6430704M03Rik	230235	4-:56975738-57003009
3313	Gpi1	14751	7-:35000227-35015318

3314	Wars2	70560	3+:98945006-99021178
3315	Serf2	378702	2+:121275152-121276793
3316	Fah	14085	7-:91733682-91754260
3317	Ak3	56248	19-:29095320-29123218
3318	2310003F16Rik	67693	2+:121282836-121284175
3319	AI662250	106639	17-:56853360-56856247
3320	Cox18	231430	5-:90643752-90653013
3321	Pla2g1b	18778	5+:115916274-115924725
3322	Ofd1	237222	X-:162828874-162878489
3323	Idh3b	170718	2-:130105048-130110164
3324	4930550L24Rik	75352	X+:56172059-56173020
3325	Plekhj1	78670	10-:80258842-80261303
3326	Lysmd2	70082	9+:75473547-75485578
3327	Trappc2	66226	X+:162878732-162891070
3328	Acsm2	233799	7+:126705245-126739690
3329	Car5a	12352	8-:124440045-124468804
3330	MMT00020226		10+:98907117-98908450
3331	A730055C05Rik	338369	11+:66838680-66845740
3332	Lrrc59	98238	11+:94491137-94506530
3333	Npm3	18150	19-:45822225-45824053
3334	Etl4	208618	2+:20704808-20705933
3335	Vps29	56433	5+:122804421-122813295
3336	Gart	14450	16-:91621653-91647198
3337	Klra7	16638	6-:130168623-130181705
3338	Olfr1250	258967	2-:89496652-89497596
3339	Dip2a	64451	10-:75780611-75807996
3340	MMT00066654		3-:159554432-159555159
3341	Ttc9	69480	12+:82732274-82765508
3342	Ik	24010	18+:36904353-36917289
3343	MMT00080053		18+:4484584-4485357
3344	Rad1	19355	15+:10415913-10423430
3345	EG620934	620934	7-:68694130-68694587
3346	Sema6d	214968	2+:124436334-124479479
3347	Mfsd1	66868	3+:67386691-67408153
3348	Cbl	12402	9-:43959507-44042067
3349	MMT00043953		
3350	MMT00044519	670211	4+:55267553-55267908
3351	Rad23b	19359	4+:55363233-55402868
3352	Ppifos	68173	14-:26516459-26520768
3353	Defb9	246079	8-:22992210-22995893
3354	Ropn1l	252967	15-:31370967-31383402
3355	Arhgap30	226652	1+:173319322-173337521
3356	Adhfe1	76187	1+:9538150-9566987
3357	Rev1	56210	1-:38109862-38165302
3358	MMT00004886		4+:105250899-105251603
3359	Pp11r	19011	15-:97541449-97551581
3360	Foxo3a	56484	10-:41905592-41996548
3361	Pot1a	101185	6-:25693945-25759188
3362	Fgf8	14179	19-:45811308-45817363
3363	MMT00033159		8-:58017542-58019101
3364	Cyp21a1	13079	17-:34938725-34941344

3365	Tiam2	24001	17+:3397206-3519396
3366	Olfr272	258836	4-:52923706-52924664
3367	Cox4i1	12857	8+:123192205-123198107
3368	MMT00038343		5+:107670641-107671378
3369	MMT00008524	100039801	8+:48907496-48908157
3370	Glipr1	73690	10-:111422512-111434251
3371	Snd1	56463	6+:28430397-28838820
3372	Prok1	246691	3-:107038048-107041576
3373	MMT00033504		13-:84829822-84830594
3374	Amot	27494	X-:141884974-141939558
3375	MMT00043922		4-:155522207-155522933
3376	Trip4	56404	9-:65681156-65732835
3377	Cs	12974	10+:127774857-127798355
3378	Cnfn	72383	7-:26152635-26154745
3379	Cdo1	12583	18-:46873158-46888009
3380	MMT00067570		X-:17457996-17494616
3381	Ctdspl	69274	9+:118835589-118953116
3382	Fgf4	14175	7+:152047409-152048845
3383	Cd99	673094	
3384	Ints5	109077	19+:8969016-8972224
3385	Wdr66	269701	5+:123738651-123776804
3386	Clec4n	56620	6+:123179891-123197037
3387	Llgl2	217325	11+:115685421-115717092
3388	a	50518	2+:154839266-154876747
3389	MMT00013165		
3390	Cbx7	52609	15-:79747712-79763057
3391	Mfap3	216760	11+:57332208-57344384
3392	Rnf141	67150	7-:117959026-117987883
3393	Snhg8	66487	14-:31937673-31942116
3394	Als2cr13	72750	1+:60005896-60042189
3395	MMT00060664		11+:76963128-76964664
3396	Ctage5	217615	12+:60230780-60291160
3397	EG636127	636127	14+:53705279-53711603
3398	Lce1a1	67127	3-:92450456-92452228
3399	4930550C14Rik	75311	9+:53213390-53240908
3400	Heatr6	217026	11+:83579626-83595487
3401	BB146404	103511	10-:33811230-33816278
3402	A930018P22Rik	68243	2+:103962925-103964903
3403	Nrxn1	18189	17-:90960080-91103707
3404	Arhgap28	268970	17-:68199343-68245606
3405	Map3k3	26406	11+:105869516-106017968
3406	Slc22a9	207151	19-:7747564-7785743
3407	Olfr653	57250	7+:111728161-111729114
3408	Cyp2c40	13099	19-:39841563-39887218
3409	Arhgap5	11855	12+:53617234-53668323
3410	Paqr9	75552	9+:95460377-95461504
3411	Rabl2a	68708	15-:89412967-89422296
3412	Olfr78	170639	7-:109890553-109891515
3413	D030013I16Rik	328734	17-:3076162-3084584
3414	MMT00059380		3+:57166891-57167200
3415	MMT00082878		3+:129771530-129771938

3416	Tmprss13	214531	9+:45136499-45154305
3417	Igh-VJ558	16061	12-:117248057-117248329
3418	MMT00009531		11+:114525933-114526409
3419	MMT00056938		X+:71252568-71252806
3420	Ldlr	16835	9+:21528221-21552860
3421	2010106G01Rik	66552	2-:126719868-126758971
3422	Uchl4	93841	9+:64083007-64084169
3423	MMT00070071		7+:12452031-12452949
3424	Mrpl43	94067	19-:45079909-45080912
3425	MMT00032998		7-:3284595-3285475
3426	Hap1	15114	11-:100208643-100217391
3427	Olfr918	258372	9-:38480089-38481066
3428	Il1r2	16178	1+:40141612-40182068
3429	Gzmf	14943	14-:56824105-56830265
3430	Rhof	23912	5-:123569439-123582428
3431	Lrfn5	238205	12+:62940414-62959327
3432	EG330963	330963	9-:65349991-65362552
3433	Naaladl2	635702	3-:24312271-24332132
3434	Capn1	12333	19-:5988614-6015126
3435	MMT00006420		X+:35533132-35536354
3436	Rfx5	53970	3+:94758955-94763289
3437	MMT00047857		5-:15004162-15004577
3438	Plac8l1	69401	18+:42351518-42352400
3439	Ebna1bp2	69072	4+:118293434-118298583
3440	Prrt3	210673	6-:113443632-113451879
3441	OTTMUSG00000(	547428	14+:54464909-54465512
3442	Refbp2	56009	1+:173433787-173434442
3443	Mtmr7	54384	8-:41633074-41634766
3444	MMT00077174	627268	X-:163304166-163542190
3445	Rnf25	57751	1-:74640326-74647951
3446	Eno1	13806	4+:149611363-149622981
3447	Fmr1	14265	X+:65931874-65971134
3448	Apobec3	80287	15+:79722855-79738344
3449	Hes6	55927	1-:93308080-93309767
3450	Itgb2l	16415	16-:96643897-96665206
3451	Spint1	20732	2+:119063153-119075247
3452	Ccrn4l	12457	3+:51028381-51055565
3453	V1rg7	171242	7+:12737812-12738753
3454	MMT00030912	545291	19-:43368684-43462771
3455	Pou2af1	18985	9+:51021794-51048183
3456	MMT00063288		
3457	Olfr1258	258980	2+:89769967-89770901
3458	MMT00001194		4-:122952126-122952715
3459	Nags	217214	11+:102006900-102010384
3460	Ddb2	107986	2-:91051744-91077064
3461	Stard4	170459	18-:33363241-33373325
3462	Nmnat3	74080	9+:98197001-98311845
3463	MMT00029730		10-:53018944-53020003
3464	1700048O20Rik	69430	9+:121847232-121851112
3465	MMT00064177		X-:53098936-53099743
3466	Darc	13349	1-:175261941-175263575

3467	1700061I17Rik	433646	3-:116763439-116780686
3468	MMT00009124		9+:71756669-71757359
3469	MMT00053608		10-:39381987-39383025
3470	Zmiz2	52915	11+:6295171-6306422
3471	Stmn3	20262	2-:181041163-181048985
3472	Steap3	68428	1-:122124000-122161835
3473	Pitpna	18738	11-:75401455-75442295
3474	Slfn4	20558	11+:83000066-83003709
3475	2610305D13Rik	112422	4-:146986047-146989538
3476	D6Mm5e	110958	6+:82906402-82980158
3477	Dhrs3	20148	4+:144482998-144517543
3478	Lonp1	74142	17-:56753718-56859205
3479	A730011L01Rik	338371	11+:119352694-119367452
3480	Pnpo	103711	11-:96799141-96805260
3481	Zfp536	243937	7-:38263614-38355010
3482	MMT00069653		14-:54943752-54944517
3483	Patl1	225929	19+:11988836-12019582
3484	Tmem176b	65963	6-:48783832-48790547
3485	Six3os1	100043902	17-:86011912-86018156
3486	Chmp5	76959	4+:40895585-40912335
3487	Grm7	108073	6+:111445646-111516059
3488	C1qtnf6	72709	15-:78353777-78360022
3489	Atg9b	213948	5-:23889934-23894261
3490	Tspan18	241556	2-:93044399-93174664
3491	Btn1a1	12231	13-:23548877-23557361
3492	Olfr549	259105	7+:109702799-109703749
3493	Wbp4	22380	14-:79861555-79880917
3494	Pcdh21	170677	14-:37891043-37911384
3495	EG633752	633752	15+:68707192-68708287
3496	Slc41a2	338365	10-:82696146-82779298
3497	Usp47	74996	7+:119167097-119254897
3498	Ccdc137	67291	11+:120319438-120326012
3499	MMT00056807		
3500	MMT00024189		2-:154594990-154595766
3501	Raph1	77300	1-:60540030-60623608
3502	Gde1	56209	7-:125832516-125849233
3503	Zfp13	22654	17-:23713023-23717541
3504	Anpep	16790	7-:86966690-86987170
3505	MMT00031335		8+:86124188-86124576
3506	Med13l	76199	5+:119130896-119215444
3507	Cacna1e	12290	1-:156243081-156573486
3508	MMT00015970		19+:60155334-60164136
3509	Olfr584	259056	7+:110234033-110234992
3510	MMT00034367		17+:37267832-37268733
3511	Rnf182	328234	13+:43763343-43816877
3512	6530401N04Rik	328092	12-:53089296-53107491
3513	Aloxe3	23801	11+:68939878-68962616
3514	MMT00027508	100046710	12-:115821756-115822263
3515	EG665467	665467	2+:181022665-181028547
3516	Elovl7	74559	13+:109004624-109073932
3517	Pcdhga1	93709	18+:37821598-38001524

3518	Smad5	17129	13+:56804412-56841318
3519	Trp53	22059	11+:69393482-69405373
3520	Lat	16797	7-:133507717-133512764
3521	Tmprss7	208171	16-:45656429-45667722
3522	Prpsap2	212627	11-:61543158-61575564
3523	Trim34	94094	7+:111393077-111485295
3524	Erc1	111173	6-:119520813-119521152
3525	Irx5	54352	8+:94881647-94884993
3526	Rccd1	269955	7-:87461398-87465354
3527	6330576A10Rik	76198	14+:100858640-100878748
3528	Il23a	83430	10-:127733195-127735140
3529	Cyp8b1	13124	9-:121823880-121825382
3530	Yipf5	67180	18-:40365892-40378509
3531	Tslp	53603	18+:32975036-32979446
3532	S100a5	20199	3+:90412443-90415698
3533	Cacna1a	12286	8+:86939687-87162764
3534	Slamf7	75345	1-:173564624-173579070
3535	S100a9	20202	3-:90496554-90499613
3536	C79127	232941	7-:19862154-19865385
3537	F9	14071	X+:57252640-57283936
3538	4930571C24Rik	78217	2+:25143662-25144701
3539	Itm2b	16432	14-:73762040-73785078
3540	MMT00015118		4-:100034062-100055849
3541	Ache	11423	5+:137731261-137735465
3542	Atp5j2	57423	5-:145944575-145952461
3543	Acsm1	117147	7+:126761341-126806028
3544	Tomm40l	641376	1-:173149165-173152625
3545	Mtrf1l	108853	10+:4522321-4533475
3546	Dhrs13	70451	11+:77845691-77851400
3547	Med25	75613	7-:52134757-52147737
3548	Olfr29-ps1	29848	4-:43794360-43809977
3549	Tbkbp1	73174	11-:96997483-97011363
3550	Bmyc	107771	2+:25562351-25563414
3551	Inpp4b	234515	8+:84265308-84426772
3552	1110017I16Rik	68527	2+:4897203-4906790
3553	MMT00015497		19+:44063343-44064044
3554	Npas3	27386	12+:54349663-55170060
3555	Sbds	66711	5-:130721604-130731385
3556	AI661453	224833	17+:47573587-47607092
3557	Dcp1a	75901	14+:31292750-31336495
3558	C85627	546161	9-:109592950-109594173
3559	4933433P14Rik	66787	12+:106923037-106940153
3560	Usp5	22225	6-:124765036-124779465
3561	MMT00020644		4-:3646817-3648177
3562	MMT00011909		10+:66467767-66503648
3563	MMT00045357		14-:118580620-118581352
3564	Spcs2	66624	7-:106987418-106997571
3565	MMT00045908		5-:117442097-117452943
3566	Rpp25	102614	9+:57351908-57353253
3567	Pfkfb4	270198	9+:108894060-108933108
3568	MMT00067704		10-:16144068-16144830

3569	ENSMUSG00000(	100038417	18-:62767196-62774658
3570	Olfr821	258772	10+:129470683-129471615
3571	Oxnad1	218885	14+:32899135-32916388
3572	Ebi3	50498	17+:56092062-56096442
3573	Tcp11	21463	17-:28203696-28217510
3574	Hcn2	15166	10+:79179378-79198853
3575	BC023179	232855	7-:7155730-7162665
3576	Ythdc2	240255	18+:44994112-45046077
3577	Ptplad2	66775	4-:88059863-88084813
3578	A130090K04Rik	320495	10+:3366096-3455988
3579	Ncbp2	68092	16+:31948712-31957682
3580	MMT00008557	668917	2+:144291073-144292697
3581	Txndc17	52700	11+:72021052-72023513
3582	Mmp21	214766	7-:140866088-140870760
3583	Paqr5	74090	9-:61803147-61874611
3584	Foxp1	108655	6-:99102052-99216516
3585	Hint1	15254	11+:54679941-54683999
3586	MMT00019611		3-:98483339-98492169
3587	MMT00041940		3-:41779988-41781970
3588	Ube4b	63958	4-:148702525-148800858
3589	Cdc6	23834	11+:98769464-98783207
3590	Pdzrn4	239618	15+:92574001-92577131
3591	BC018507	218333	13-:70727575-70740033
3592	Twistnb	28071	12+:34114409-34125424
3593	MMT00031732		4+:110962686-110963670
3594	Rasa4	54153	5+:136559867-136587725
3595	Vps37a	52348	8+:41633073-41634766
3596	Bbs5	72569	2+:69485311-69505626
3597	6820408C15Rik	228778	2+:152257979-152270063
3598	Rpo1-3	20018	5+:147889116-147890656
3599	MMT00035426		8+:35570238-35570761
3600	MMT00046966		7-:14813029-14814183
3601	Arfgef1	211673	1-:10127642-10223151
3602	Zfp526	210172	7+:26009320-26035228
3603	BC027231	212547	16+:44724416-44737241
3604	2610029G23Rik	67683	X+:102275133-102278823
3605	Slc7a14	241919	3-:31107999-31156791
3606	S100a14	66166	3+:90330791-90332755
3607	Car15	80733	16-:17835369-17838231
3608	Igbp1	18518	X+:97689688-97711464
3609	Plvap	84094	8-:74021665-74035630
3610	Rnf38	73469	4-:44139090-44200391
3611	Agxt2l2	72947	11+:51398285-51416765
3612	Cd2	12481	3-:101079832-101091862
3613	1110018J18Rik	66129	13-:64393143-64414018
3614	2610030H06Rik	67048	X+:69061263-69070044
3615	Ostm1	14628	10+:42398743-42422254
3616	Dnajc16	214063	4-:141319050-141344970
3617	Rasl10b	276952	11+:83232799-83234515
3618	EG234097	234097	8+:15431223-15438175
3619	Cdan1	68968	2-:120543922-120546814

3620	Bet1	12068	6-:4027877-4036899
3621	Hoxd13	15433	2+:74506366-74508192
3622	Trim21	20821	7-:109707349-109713948
3623	Olfr1324	258289	X-:47778673-47779671
3624	Pygb	110078	2+:150612521-150657491
3625	4930563D23Rik	75328	16-:92318517-92321643
3626	Ralgps1	241308	2-:32989395-33140437
3627	MMT00048939		16-:18944350-18944684
3628	Scap	235623	9+:110235924-110287451
3629	Ankrd60	70065	2-:173394105-173403754
3630	Lgr6	329252	1-:136882644-136960275
3631	Vps37b	330192	5-:124454649-124482254
3632	Lctl	235435	9+:63964953-63985923
3633	MMT00009144		8-:56165074-56167391
3634	Mrps34	79044	17+:25032064-25033216
3635	Ndrg4	234593	8+:98226962-98238855
3636	Tram2	170829	1-:20991459-21069280
3637	D930014E17Rik	57373	7+:116847250-116855695
3638	Mkx	210719	18-:6992775-7002542
3639	Alpi	76768	1-:88994578-88998110
3640	D11Bwg0517e	52897	11-:118352177-118471514
3641	4930422G04Rik	71643	3+:127259131-127266610
3642	Spsb4	211949	9-:96843901-96896732
3643	Nell2	54003	15-:95050363-95358683
3644	Umps	22247	16-:33954879-33967394
3645	Sipa1l1	217692	12+:83412332-83551902
3646	Clcc1	229725	3+:108457164-108480927
3647	Nucks1	98415	1+:133807070-133832744
3648	Odf2l	52184	3+:144781563-144816849
3649	Zfp395	380912	14+:66004224-66015602
3650	4931400O07Rik	70936	X+:59721161-59721986
3651	Es31	382053	8+:107572495-107582310
3652	Adm	11535	7+:117771182-117773333
3653	Olfr1298	258888	2-:111485215-111486152
3654	St13	70356	15-:81195474-81230124
3655	0610009O20Rik	66839	18+:38409928-38415204
3656	Hoxb3	15410	11-:96204379-96211416
3657	Armcx1	78248	X+:131252549-131256443
3658	Heatr5a	320487	12-:52976803-53072331
3659	EG236451	236451	14-:59939802-59946936
3660	Tlk1	228012	2-:70550466-70625056
3661	Dhx15	13204	5-:52541454-52581633
3662	1700081L11Rik	76719	11+:104303802-104353236
3663	Supt4h2	100041294	11+:87551101-87556815
3664	Carhsp1	52502	16-:8661033-8664508
3665	Zfp61	22719	7-:25076064-25084609
3666	Tas1r1	110326	4-:151402022-151412677
3667	MMT00003196		6+:43039921-43049586
3668	Cbfa2t2	12396	2+:154262241-154365090
3669	Kdsr	70750	1-:108619915-108656299
3670	Dusp3	72349	11-:101835472-101846084

3671	1810033B17Rik	69189	8+:3665752-3668915
3672	Stt3b	68292	9-:115151315-115219253
3673	Tro	56191	X-:147080247-147084030
3674	Edil3	13612	13+:89084416-89324469
3675	Pglyrp4	384997	3+:90532548-90544383
3676	Ucp2	22228	7+:107645261-107647959
3677	MMT00052835	633387	10+:38918264-38918972
3678	Fxc1	14356	7+:112788984-112789864
3679	Rpl3	27367	15-:79908225-79913765
3680	Acvr2b	11481	9+:119311618-119342633
3681	Edc4	234699	8+:108404795-108417105
3682	MMT00047887		5+:34644025-34648105
3683	Oas2	246728	5-:121180342-121199797
3684	AI854703	243373	6+:48576763-48580001
3685	Apoc1	11812	7-:20274832-20277988
3686	Psmd9	67151	5+:123696248-123698970
3687	Bcl9	77578	3-:97007591-97009680
3688	Apobec4	71281	1+:154597705-154604674
3689	Adh1	11522	3+:137940608-137953654
3690	EG232974	232974	7-:25713721-25714387
3691	Syne1	64009	10+:5217964-5235717
3692	BC027072	225004	17-:72093259-72099806
3693	Adcy1	432530	11+:7000568-7069510
3694	Ankrd44	329154	1-:54704054-54885676
3695	1700007H22Rik	75484	16-:86139548-86150921
3696	Elovl6	170439	3+:129235356-129341406
3697	MMT00021930		5-:30045974-30046246
3698	1600014C10Rik	72244	7+:38968834-38980115
3699	Dscr3	13185	16-:94719394-94748050
3700	Nrxn1	18189	17-:90436234-90458903
3701	Zfp68	24135	5-:139047347-139060955
3702	Olfr979	259112	9-:39807876-39808810
3703	Ggcx	56316	6+:72364326-72380701
3704	H2-Q7	15018	17+:35576165-35580297
3705	5730590G19Rik	77011	7+:86814405-86820605
3706	2410002O22Rik	66975	13-:104933396-104968500
3707	Etos1	114660	7-:137914151-137916166
3708	Thrap3	230753	4-:125856027-125879856
3709	Mylip	218203	13+:45485109-45507382
3710	Scamp5	56807	9-:57291453-57315828
3711	Rufy1	216724	11-:50194999-50244901
3712	Mrpl32	75398	13-:14702567-14705304
3713	Acot6	217700	12+:85441455-85452294
3714	Nobox	18291	6-:43253672-43259553
3715	Mapk14	26416	17+:28828389-28885350
3716	Olfr160	80706	9-:37518932-37519862
3717	Nlrp1c	627984	11-:70905760-71094536
3718	Brd3	67382	2-:27303349-27331141
3719	MMT00006786		1-:55176947-55177656
3720	Zbtb44	235132	9+:30838107-30877921
3721	Me2	107029	18-:73929695-73975046

3722	MMT00004801		13+:36084134-36085158
3723	Pcdhb12	93883	18+:37595456-37597826
3724	Zfp260	26466	7+:30889695-30890917
3725	OTTMUSG000000	666048	4+:114252888-114283277
3726	MMT00052248		1-:17387369-17390781
3727	Erdr1	170942	
3728	Sfxn4	94281	19-:60914648-60937267
3729	Cldn14	56173	16-:93919482-93920210
3730	Hus1	15574	11-:8893147-8911163
3731	MMT00060364		16+:16853156-16853572
3732	Spata21	329972	4+:140660744-140668078
3733	Wnt2	22413	6-:17939814-17980287
3734	Trim37	68729	11+:86940607-87031832
3735	Rspo2	239405	15-:42909644-43001574
3736	0610037P05Rik	66086	16-:14299988-14317419
3737	Thap7	69009	16-:17528071-17531151
3738	Cadm2	239857	16-:66663339-66815707
3739	Mef2a	17258	7-:74376969-74461806
3740	MMT00079610		12-:47579785-47580402
3741	Casp9	12371	4+:141349769-141369601
3742	Chrd	12667	16+:20733199-20742458
3743	Corin	53419	5-:72691264-72895447
3744	Rmnd1	66084	10+:5918863-5938724
3745	AI463229	100042802	13-:37750568-37759030
3746	Osbpl5	79196	7-:150874667-150927868
3747	4932443I19Rik	403185	8+:13734767-13743066
3748	Rhox8	434768	X-:35231025-35231750
3749	Aebp1	11568	11+:5761949-5772052
3750	Slc36a3	215332	11-:54938361-54950875
3751	Luzp1	269593	4+:136077306-136105230
3752	MMT00078260		12+:88878853-88879607
3753	Setdb1	84505	3-:95127447-95161078
3754	Dock6	319899	9-:21604303-21651716
3755	Ndn	17984	7+:69493207-69494811
3756	Chrne	11448	11-:70428385-70432696
3757	Ctnnd1	12388	2-:84441482-84490669
3758	Rab9b	319642	X-:133395644-133403077
3759	4931406B18Rik	74054	7-:50749967-50761297
3760	Vmn2r90	626942	17+:17849235-17870742
3761	1700026J12Rik	73255	16-:49210753-49255911
3762	2310045A20Rik	231238	5-:53498322-53604446
3763	MMT00052953		5+:108946197-108946551
3764	MMT00009379		10+:125622952-125623497
3765	2310022B05Rik	69551	8-:127159654-127187125
3766	Gria1	14799	11+:56825272-57141293
3767	Pnma2	239157	14+:67530073-67536686
3768	Cyp2c44	226143	19-:44079510-44103732
3769	MMT00048655		
3770	Zbtb9	474156	17+:27110108-27113149
3771	MMT00014874		
3772	MMT00002631		X-:140720262-140722876

3773	MMT00001709		5-:16473671-16501206
3774	Ddx18	66942	1-:123450418-123464480
3775	Hoxa5	15402	6-:52151788-52154586
3776	Btbd16	330660	7+:137923070-137967890
3777	Wdr91	101240	6-:34830279-34860843
3778	Phlppl	244650	8+:112392518-112464807
3779	Ghdc	80860	11-:100627647-100632271
3780	Ryk	20187	9+:102737245-102810635
3781	Arpc2	76709	1+:74283073-74314782
3782	Rgs5	19737	1+:171585677-171622687
3783	MMT00082345		6-:39777684-39778771
3784	Mafg	17134	11-:120490597-120491167
3785	Pten	19211	19-:32832069-32832788
3786	Mta1	116870	12+:114336533-114375384
3787	Myf6	17878	10-:106929915-106931785
3788	Tcam1	75870	11+:106138035-106147071
3789	4833442J19Rik	320204	6+:149090114-149099694
3790	Mtss1	211401	15-:58774802-58913544
3791	Tgfb2	21808	1-:188447523-188529868
3792	Zan	22635	5-:137819870-137918292
3793	Htr1a	15550	13+:106234265-106236638
3794	Ywhab	54401	2+:163820933-163842538
3795	Psmc6	67089	14+:45951608-45969355
3796	D19Bwg1357e	52874	19-:27463189-27504341
3797	Nrip2	60345	6+:128349778-128358946
3798	Isoc2b	67441	7-:4796561-4817781
3799	Serpinh1	12406	7-:106493887-106501606
3800	Ltf	17002	9+:110921795-110944706
3801	Casp8ap2	26885	4+:32702487-32740235
3802	MMT00020288		10-:13610397-13611510
3803	MMT00041842	670880	10+:77273492-77274612
3804	MMT00015534		5+:7179364-7180662
3805	Siglece	83382	7-:50906441-50915515
3806	MMT00063223		11-:102437760-102440602
3807	Vmn2r83	625029	10+:78940711-78943081
3808	2010209O12Rik	100910	5+:24093855-24098306
3809	Slc9a3	105243	13+:74258273-74303510
3810	Fbxo2	230904	4+:147538177-147540527
3811	MMT00052318		2+:23763958-23764363
3812	Zfyve26	211978	12-:80333330-80374663
3813	Nell1	338352	7+:57474942-57535402
3814	MMT00080381		7-:4127964-4128271
3815	Slamf1	27218	1+:173697296-173722842
3816	Gstm5	14866	3+:107698773-107701602
3817	Aire	11634	10-:77492775-77506325
3818	Cep192	70799	18+:67994293-68044828
3819	Pigo	56703	4-:43030509-43038628
3820	MMT00002991		X-:64222392-64223901
3821	Lnp	69605	2-:74357830-74416979
3822	Ly6g6c	68468	17+:35204334-35206478
3823	Slc22a15	242126	3-:101663850-101728370

3824	Tnfrsf23	79201	7-:150854400-150871752
3825	Uaca	72565	9+:60642346-60728237
3826	Ptplad1	57874	9-:64834791-64869516
3827	Mical2	320878	7+:119369396-119498504
3828	BC063749	414801	19-:47862801-47876472
3829	Uba2	50995	7-:34925721-34953541
3830	4933422H20Rik	432613	11+:115302394-115309583
3831	Bmp8b	12164	4+:122782403-122802585
3832	Sdhb	67680	4+:140517319-140535015
3833	Atxn7l1	380753	12+:33832644-33936011
3834	Calb2	12308	8-:112666424-112692105
3835	Btbd14b	66830	8-:87195777-87211743
3836	2200001I15Rik	69134	14-:35165067-35168578
3837	Tradd	71609	8-:107782144-107788511
3838	Trim32	69807	4+:65266054-65277275
3839	Phactr3	74189	2+:177910082-178071196
3840	Gm444	242915	5+:30440465-30444916
3841	MMT00014519		7-:49303224-49305709
3842	Abca12	74591	1-:71408315-71461470
3843	Ubtf	21429	11-:102167305-102177788
3844	Speg	11790	1+:75397624-75401138
3845	MMT00048576		17+:91505594-91508123
3846	Ccdc32	269336	2-:118843537-118855094
3847	Tdrd7	100121	4+:46000333-46047629
3848	Mrpl21	353242	19+:3282918-3291200
3849	MMT00047578		1+:111171746-111732864
3850	Dach1	13134	14-:98188801-98568528
3851	MMT00077752		X-:154050420-154051106
3852	Nfam1	74039	15-:82828165-82831943
3853	Pprc1	226169	19+:46139106-46147393
3854	MMT00008389		5+:10866071-10868776
3855	ENSMUSG00000(	218695	14-:8577304-8624164
3856	Slc30a5	69048	13-:101572917-101584591
3857	1700001C19Rik	75462	17-:47550324-47574327
3858	Gcnt2	14538	13+:40982533-41054685
3859	Fpr2	14289	17+:18029707-18030762
3860	Mlx	21428	11+:100948632-100952829
3861	Osbpl7	71240	11+:96912159-96930208
3862	Gpd1	14555	15+:99548031-99554203
3863	Scube2	56788	7-:116942162-117009168
3864	Gpd1l	333433	9-:114808465-114843018
3865	Acta1	11459	8-:126415661-126418675
3866	Scyl1bp1	98376	1-:165316007-165333769
3867	Sln	66402	9+:53697970-53702367
3868	Ebpl	68177	14-:61959597-61979276
3869	Zadh2	225791	18+:84264344-84266906
3870	Olfr1312	258359	2-:111882235-111883187
3871	MMT00067361		12-:91309251-91382876
3872	Cltb	74325	13-:54693787-54712701
3873	Lefty1	13590	1+:182865152-182868531
3874	Slitrk3	386750	3-:72852375-72860782

3875	MMT00077845		12-:45303417-45303845
3876	Zeb2	24136	2-:44843941-44965649
3877	Gsk3b	56637	16+:38090445-38240746
3878	Rnps1	19826	17+:24555401-24562840
3879	Bmp1	12153	14-:70874366-70919894
3880	Trf	22041	9-:103111206-103132616
3881	Sntb1	20649	15-:55470708-55738502
3882	Itfg3	106581	17-:26349653-26381152
3883	Wdr7	104082	18+:63868378-64147012
3884	Icam5	15898	9+:20836533-20843480
3885	Apol7c	108956	15-:77355282-77363746
3886	MMT00047275	546271	X+:23730763-23731986
3887	Tcrg-V5	21639	13+:19282236-19286398
3888	Ran	19384	5+:129526094-129528692
3889	Wasf3	245880	5+:147196581-147282701
3890	Sdc3	20970	4+:130348560-130378849
3891	Usp34	17847	11+:23206973-23321366
3892	D0H6S2654E	108161	13+:34838320-34839491
3893	Tmem16b	243634	6+:125640433-125989428
3894	EG435366	435366	13-:62470568-62887084
3895	Art5	11875	7-:109245386-109251361
3896	Rab11fip4	268451	11+:79404847-79506340
3897	Papln	170721	12+:85104610-85133300
3898	Zc3h3	223642	15-:75614822-75672335
3899	MMT00029169		X+:5436870-5438658
3900	Olfr166	259071	16+:19486932-19487869
3901	F730047E07Rik	212377	4+:24448387-24530093
3902	Spag16	66722	1+:69873543-70313851
3903	MMT00041825		
3904	Serinc1	56442	10-:57235583-57252255
3905	H2-Q1	15006	17+:35457506-35460984
3906	Ppp6c	67857	2-:39052317-39081859
3907	Dkk3	50781	7-:119260526-119302562
3908	Fundc2	67391	X+:72627760-72640322
3909	MMT00012984		5-:87389544-87390739
3910	Trpm7	58800	2-:126617301-126701947
3911	Cugbp1	13046	2+:90780639-90859649
3912	Zzef1	195018	11+:72645044-72649832
3913	Mrpl15	27395	1-:4764014-4775808
3914	MMT00042063		6-:124901241-124902022
3915	Gpr125	70693	5-:50351190-50450064
3916	Vmn2r12	627569	5-:109514910-109521231
3917	MMT00045955		3+:107145807-107146412
3918	Hrg	94175	16+:22951186-22961718
3919	Ucn	22226	5-:31440362-31441268
3920	Slc25a3	18674	10-:90579535-90586723
3921	2300002D11Rik	69539	4-:133047015-133054050
3922	Clec11a	20256	7-:51559624-51562264
3923	D9Ertd402e	382117	9+:122714739-122744303
3924	Gimap5	317757	6+:48700458-48703596
3925	Akap2	11641	4+:57867030-57906562

3926	Tmcc3	319880	10+:93977620-94052846
3927	MMT00021352		15-:99385921-99386665
3928	Polr3k	67005	2+:181599160-181604295
3929	Sfrs11	69207	3-:157673458-157699601
3930	D730001G18Rik	78725	15-:74601346-74609289
3931	Gatad1	67210	5-:3639964-3647957
3932	Pyy	217212	11-:101967993-101969090
3933	MMT00035461		13+:28021453-28022774
3934	Dfnb59	381375	2+:76488343-76496600
3935	Dnahc7c	100101919	1+:46571397-46703970
3936	Pde1c	18575	6-:56031774-56312513
3937	Pldn	18457	2+:122564340-122572412
3938	Abt1	30946	13-:23513408-23515770
3939	Dazap1	70248	10+:79727841-79750817
3940	Olfr420	258302	1+:176088905-176089869
3941	MMT00049081		X+:82961759-82962076
3942	Padi2	18600	4+:140473480-140482232
3943	Atoh1	11921	6+:64679316-64680372
3944	Nlgn3	245537	X+:98497626-98515601
3945	Cugbp2	14007	2-:6462915-6806015
3946	Klk1	16612	7+:51480848-51484938
3947	MMT00014106	331003	9+:92612292-92613916
3948	V1rb4	113854	6+:89843267-89844199
3949	A630007B06Rik	213993	19-:56873047-56875755
3950	Syne1	64009	10+:5089617-5091148
3951	MMT00048877	623568	16+:17137420-17137680
3952	Bcar1	12927	8-:114234650-114267647
3953	MMT00077470		19+:27930028-27932990
3954	Syne1	64009	10+:5116826-5137085
3955	Neurod1	18012	2-:79292678-79296793
3956	Psat1	107272	19-:15980536-15999413
3957	Dennd1b	329260	1-:141030362-141031320
3958	Kcnc3	16504	7+:51846489-51851933
3959	Bzw2	66912	12-:36818848-36883368
3960	MMT00007654		2+:117066618-117068004
3961	Serpinb9g	93806	13+:33578399-33587146
3962	EG629959	629959	11+:104411100-104436986
3963	Osbp	76303	19+:12040565-12068603
3964	Colq	382864	14-:32337492-32390504
3965	Pwp2	110816	10-:77633654-77647874
3966	9330101J02Rik	664857	7-:146825440-146828512
3967	Tysnd1	71767	10+:61158261-61165516
3968	Gsc2	195333	16-:17913206-17915135
3969	MMT00002495		18-:9498050-9498459
3970	4931414P19Rik	74359	14-:55202500-55224741
3971	Ppp1r16a	73062	15+:76520597-76525347
3972	Ube2o	217342	11+:116398987-116399889
3973	Dmtf1	23857	5-:9119662-9161724
3974	MMT00000792	100044772	7+:37019310-37020770
3975	Zfp711	245595	X+:109717872-109747165
3976	MMT00025735		11+:38032595-38033343

3977	1110002H13Rik	66139	2-:26917192-26927681
3978	Shank2	210274	7+:151238193-151472669
3979	MMT00037549		4+:155109506-155114102
3980	Gkap1	56278	13-:58334711-58365534
3981	MMT00000262		18+:56001432-56002661
3982	Myo15	17910	11+:60282840-60341868
3983	4930542C12Rik	67648	9-:78242762-78243965
3984	Dclre1a	55947	19-:56603992-56621586
3985	Gjb3	14620	4-:127002480-127006405
3986	Ep400	75560	5-:111093125-111199611
3987	MMT00005386		11-:32780362-32780990
3988	4933427G17Rik	74466	7+:128126021-128158300
3989	Wrnip1	78903	13+:32894049-32914477
3990	Trpc6	22068	9+:8544141-8680741
3991	Hoxb9	15417	11+:96132821-96137905
3992	Slc5a1	20537	5+:33447105-33504504
3993	Nlrp9c	330490	7-:27149713-27170826
3994	Crygs	12970	16-:22805276-22811479
3995	MMT00028835		6+:68541373-68630828
3996	MMT00014442		4-:109427053-109427410
3997	Eya3	14050	4+:132194999-132279392
3998	4930528F23Rik	75178	17+:24955221-24976731
3999	Nr3c1	14815	18-:39573930-39646901
4000	Eif2ak1	15467	5+:144578707-144663454
4001	Apoa4	11808	9+:46049236-46051372
4002	Neurog1	18014	13-:56352558-56353293
4003	4930401O10Rik	73816	11-:71741971-71753609
4004	Thbs2	21826	17-:14804160-14831097
4005	Ccdc19	71870	1+:174451267-174476002
4006	Rassf4	213391	6-:116588257-116623808
4007	C030017K20Rik	381581	4+:154829715-154840508
4008	Gnl3l	237107	X-:147419697-147451866
4009	Cotl1	72042	8-:122333124-122364440
4010	Alkbh2	231642	5-:114573944-114578157
4011	Kcnmb3	435726	3-:32371371-32381437
4012	Ctsw	13041	19-:5465341-5468483
4013	Foxa2	15376	2-:147868658-147872705
4014	Dock1	330662	7+:142039811-142067998
4015	Gabrr2	14409	4+:33150228-33182560
4016	Zbtb1	268564	12+:77471558-77489727
4017	Kcna7	16495	7+:52661329-52666751
4018	Slc25a28	246696	19-:43738293-43749371
4019	Tbk1	56480	10-:120983665-121021376
4020	5031439G07Rik	223739	15-:84753565-84818377
4021	4833403I15Rik	74574	18+:47009692-47054394
4022	Gdpd5	233552	7+:106535613-106610356
4023	Angptl3	30924	4+:98697637-98704883
4024	Psp	19194	2+:153834856-153841758
4025	MMT00009397	625963	3-:35555966-35565048
4026	Kif24	109242	4-:41401422-41411920
4027	Exosc2	227715	2+:31526255-31536825

4028	4930589M24Rik	75906	10-:53332071-53470991
4029	Samsn1	67742	16-:75859504-75909469
4030	Prps1	19139	X+:136991211-137010677
4031	MMT00064952		14-:49454090-49454209
4032	MMT00008594		13-:109010657-109011035
4033	Mgst2	211666	3+:51465110-51486596
4034	Slc6a13	14412	6+:121250313-121287735
4035	MMT00027516		9-:63648474-63649604
4036	Gna12	14673	5-:141235897-141306385
4037	Sox1	20664	8+:12396360-12397536
4038	1200009O22Rik	66873	6-:53767162-53770477
4039	Klc3	232943	7-:19979789-19985205
4040	Paqr3	231474	5-:97511347-97540588
4041	MMT00051968		1+:152983499-152984964
4042	OTTMUSG00000(	100038451	2+:20890669-20891647
4043	Dctn2	69654	10+:126703415-126721820
4044	C230052I12Rik	101831	7-:36176629-36181813
4045	MMT00072411	84652	5-:23466847-23536487
4046	Phb	18673	11+:95529348-95541341
4047	Nbeal1	269198	1+:60238432-60292289
4048	BC002230	217827	12-:101363660-101397843
4049	MMT00053760		7-:135159956-135160734
4050	Cttnbp2nl	80281	3-:104806662-104855833
4051	ENSMUSG00000(	100127434	7+:50352745-50355503
4052	Csrp2bp	228714	2+:144206438-144220418
4053	Pcdha4	12936	18+:37112342-37347311
4054	MMT00040937		7-:96910028-96910611
4055	Mobkl2b	214944	4-:34899953-35104715
4056	Tnfaip8l1	66443	17+:56301952-56312440
4057	Slc2a4	20528	11-:69756044-69761636
4058	Mapk11	19094	15-:88974168-88979943
4059	MMT00048362		X+:87308393-87309159
4060	Stard3	59045	11+:98219697-98242410
4061	Apcs	20219	1-:174824092-174825172
4062	MMT00015329	627626	17+:42639346-42640863
4063	Upk2	22269	9-:44260808-44262850
4064	Peg10	170676	6+:4697379-4707164
4065	Igsf5	72058	16+:96583400-96625635
4066	Actg2	11468	6-:83462903-83486219
4067	MMT00016020		4-:64517954-64518804
4068	1110049B09Rik	68764	12-:33727221-33767238
4069	Tet3	194388	6-:83317730-83391267
4070	Chst10	98388	1-:38920721-38954837
4071	Ccbe1	320924	18-:66428679-66462396
4072	Adcy2	210044	13-:68758930-69138158
4073	Eif4e2	26987	1+:89110574-89134459
4074	Ush2a	22283	1+:190686251-190768687
4075	Stk11ip	71728	1+:75518150-75533600
4076	Larp5	217980	13+:9093165-9173650
4077	MMT00022995	106407	16-:32475622-32487776
4078	Gpaa1	14731	15+:76161794-76165329

4079	Emilin3	280635	2-:160731240-160738052
4080	Atp2a1	11937	7-:133589373-133591457
4081	1700030J22Rik	69528	8-:119495008-119502848
4082	Colec11	71693	12-:29279038-29308156
4083	Olfr688	259161	7+:112382715-112437572
4084	Asah3l	230379	4+:86520367-86566778
4085	Cd302	66205	2-:60090049-60122475
4086	Adamts8	30806	9+:30750146-30770441
4087	Olfr632	259123	7+:111085895-111086847
4088	Pdcl2	79455	5-:76741291-76760158
4089	Socs6	54607	18-:89038345-89056679
4090	Orai3	269999	7+:134913355-134918664
4091	Smok2a	27263	17+:13418376-13428942
4092	D17Ertd663e	52042	17+:13011442-13012476
4093	Akp5	11650	1-:88983623-88986338
4094	MMT00018835		16+:36778915-36779686
4095	Trub2	227682	2-:29631630-29643170
4096	Slc22a14	382113	9-:119078574-119090145
4097	MMT00065861		18-:45306180-45306781
4098	Il2ra	16184	2+:11564614-11611409
4099	Abcc4	239273	14-:118881913-119105331
4100	Pla2g3	237625	11+:3388354-3393064
4101	Atf2	11909	2-:73654580-73730651
4102	MMT00059041		1+:133113761-133114665
4103	Glra1	14654	11-:55328315-55421235
4104	Ift80	68259	3-:68696433-68794891
4105	Speer5-ps1	70365	10+:43891440-43939389
4106	MMT00041409		4-:73158398-73159655
4107	MMT00068618		6+:126721939-126724832
4108	Apobec2	11811	17-:48558555-48572044
4109	Abpb	233099	7-:34797102-34798961
4110	Gab2	14389	7+:104230260-104453546
4111	EG238507	238507	13+:12772251-12772704
4112	MMT00062664	100041265	7+:133630961-133634007
4113	Nudt3	56409	17-:27716335-27760397
4114	Parp12	243771	6-:39036411-39068158
4115	MMT00013679		13-:55723727-55724168
4116	Ifi205	226695	1-:175664569-175962188
4117	MMT00007872		2-:127394837-127395484
4118	Tnks	21951	8-:35894526-36029262
4119	Magea7	17143	X+:73592732-73592974
4120	Mrpl3	94062	9+:104955628-104979693
4121	Dhx33	216877	11-:70800043-70817929
4122	Ehbp1l1	114601	19-:5708051-5726270
4123	Ky	16716	9+:102408467-102448273
4124	1110037F02Rik	66185	4+:11413147-11542956
4125	Tmem205	235043	9-:21725236-21731973
4126	Vti1a	53611	19+:55390850-55701007
4127	7-Sep	235072	9+:25058618-25085233
4128	Grcc10	14790	6-:124689202-124691097
4129	Taf6l	225895	19-:8848983-8860753

4130	Itga9	104099	9+:118515826-118808057
4131	Olfr769	257667	10-:128548541-128549479
4132	Hrc	15464	7+:52590770-52594340
4133	Cbr3	109857	16+:93683459-93691231
4134	MMT00054523		1+:45234935-45235440
4135	Pard6g	93737	18+:80243782-80314822
4136	Ttll6	237930	11+:96017858-96020270
4137	Pof1b	69693	X-:109753196-109812262
4138	Cited4	56222	4+:120339205-120340423
4139	Sh2d5	230863	4+:137806379-137815136
4140	Dmc1	13404	15-:79391929-79435514
4141	Pank4	269614	4+:154338254-154355046
4142	Syde1	71709	10-:78047248-78054692
4143	Zfp286	192651	11-:62593107-62597268
4144	Ndufb2	68198	6+:39542581-39549470
4145	MMT00040749		
4146	Cml1	66116	6-:85860150-85865655
4147	Zmpste24	230709	4-:120731843-120770837
4148	1700034J05Rik	73344	6-:146899823-146902943
4149	2810055G20Rik	77994	16+:77556176-77595147
4150	Isg20l1	68048	7+:86040828-86052462
4151	1500009C09Rik	76505	15+:82086520-82091180
4152	Lrrc33	224109	16-:32142908-32165572
4153	Dnahc10	56087	5+:125217172-125314468
4154	Cdh6	12563	15-:12963958-13021632
4155	2700062C07Rik	68046	18+:24629368-24636266
4156	A730017C20Rik	225583	18+:59225484-59235555
4157	MMT00056386		
4158	4933400N17Rik	619289	1-:39722152-39727434
4159	Htr5b	15564	1-:123406351-123425042
4160	Ceacam16	330483	7-:20438920-20440532
4161	Krt31	16660	11-:99907959-99911865
4162	MMT00001543		7-:114342678-114349637
4163	5830415L20Rik	68152	5+:3543835-3561044
4164	Junb	16477	8-:87500831-87502647
4165	Cux1	13047	5-:136748771-136849218
4166	Cblc	80794	7-:20366318-20382131
4167	Rgmb	68799	17-:15957568-15958170
4168	MMT00052774		6+:41091187-41092199
4169	Ceacam16	330483	7-:20441422-20472001
4170	Ttc15	217449	12-:29375494-29435300
4171	1700023F02Rik	69433	10-:65583359-65584736
4172	Armc9	78795	1+:88051366-88172829
4173	MMT00008170		13-:47104637-47106562
4174	Unc13b	22249	4+:43071840-43277742
4175	Ren2	19702	1+:135247242-135256894
4176	MMT00040721		
4177	MMT00076823		15-:96753247-96753838
4178	Ntn1	18208	11-:68026507-68199622
4179	Zfp179	22671	11-:61261945-61267388
4180	2010015L04Rik	544678	4+:154796690-154822053

4181	Gzf1	74533	2+:148506915-148518686
4182	Foxd3	15221	4+:99322989-99325313
4183	Sms	20603	X-:153883582-153929961
4184	Olfr135	258329	17+:38345191-38346129
4185	Hmgb2	97165	8+:59991854-59993062
4186	Car7	12354	8+:107064683-107074245
4187	V1rc13	171186	6-:66726217-66727124
4188	Sult1a1	20887	7-:133816384-133819871
4189	Epb4.1l1	13821	2+:156246836-156366015
4190	Arhgap8	109270	15+:84500049-84534102
4191	Fbln2	14115	6+:91162857-91222533
4192	Zranb1	360216	7+:140141290-140178050
4193	Taf10	24075	7-:112891411-112892857
4194	Dnttip2	99480	3+:121977323-121988194
4195	Csrp2bp	228714	2+:144194758-144233390
4196	Atg16l2	73683	7-:108438734-108450613
4197	A930037G23Rik	320678	6+:125095258-125111794
4198	Ghr	14600	15-:3267776-3533231
4199	B3galt5	93961	16+:96536775-96537701
4200	Hmgcll1	208982	9+:75862881-75981671
4201	Tubb2c	227613	2-:25077681-25080161
4202	Nkrf	77286	X-:34427463-34443529
4203	Fermt2	218952	14-:46078471-46087081
4204	Rapgef5	217944	12+:118772579-118995776
4205	Rgs1	50778	1-:146091799-146096165
4206	Nipsnap1	18082	11+:4774005-4794203
4207	MMT00055140		9-:70701738-70702277
4208	MMT00013996		13+:58320079-58322352
4209	Tcfap2c	21420	2+:172375113-172384109
4210	Cygb	114886	11-:116506910-116515627
4211	Tlx1	21908	19+:45225405-45230605
4212	Slco2b1	101488	7-:106806908-106844138
4213	MMT00051158		7-:17724169-17724860
4214	MMT00014098		13+:34653871-34654238
4215	Olfr704	257902	7+:114008495-114009443
4216	Ccdc89	70054	7+:97575093-97576204
4217	Lynx1	23936	15-:74578286-74583383
4218	Iqca	74918	1-:91938708-92049978
4219	Ccdc120	54648	X-:7308842-7315649
4220	EG629065	629065	17-:53840190-53842391
4221	Zfp12	231866	5+:143996841-144007562
4222	MMT00004340		14-:53900874-53901184
4223	Dnahc7l	207778	14+:27516798-27704888
4224	1110008L16Rik	66132	12+:56403810-56483484
4225	Pick1	18693	15+:79060242-79079890
4226	4930547N16Rik	75317	10-:87555528-87609679
4227	MMT00007944		
4228	Mif	17319	10-:75322101-75322953
4229	Pth	19226	7-:120529091-120532086
4230	Snx9	66616	17+:5902447-5931033
4231	Nrk	27206	X+:135449043-135542590

4232	B020011L13Rik	547097	1+:119637961-120026240
4233	Brpf1	78783	6+:113257220-113274854
4234	Tshz3	243931	7+:37553639-37556852
4235	Pfkfb3	170768	2-:11393452-11423652
4236	Lime1	72699	2+:181115939-181118331
4237	Dnahc5	110082	15+:28133520-28400425
4238	Snx25	102141	8-:47118617-47209500
4239	MMT00070824		X-:75002571-75003485
4240	Ctnna3	216033	10+:63283800-63315574
4241	Klf7	93691	1-:64082288-64167854
4242	Wdr25	212198	12+:110218900-110266668
4243	Fbxo38	107035	18-:62663722-62708370
4244	Abca17	381072	17-:24402148-24404682
4245	Ulk4	209012	9-:120873567-121186321
4246	Fbxw12	434440	9-:109125786-109127028
4247	Sfpq	71514	4+:126697954-126713103
4248	D330022A01Rik	320169	9+:107894826-107895793
4249	Fndc7	320181	3-:108665474-108679533
4250	Papd4	100715	13-:93917355-93962238
4251	MMT00065846		
4252	Ikbkb	16150	8-:23769684-23816679
4253	3830431G21Rik	217682	12+:81793586-81807416
4254	Pkm2	18746	9+:59513013-59527176
4255	Muc4	140474	16+:32755976-32782498
4256	EG436332	436332	16+:4230300-4231097
4257	MMT00041230	677156	17+:32762066-32772164
4258	Rpl5	19983	5+:108329587-108345390
4259	Olfr203	258479	16+:59302980-59303899
4260	Gyltl1b	228366	2-:92205198-92211191
4261	MMT00014970		12+:89573046-89574124
4262	Stx7	53331	10+:23869192-23908031
4263	Otof	83762	5-:30669354-30764275
4264	MMT00054075		
4265	Olfr1412	258274	1+:94484908-94485872
4266	Fn1	14268	1-:71632090-71699746
4267	BC018465	228802	2+:154049515-154066636
4268	Tube1	71924	10+:38853833-38870551
4269	Abca4	11304	3+:121747377-121882977
4270	C77080	97130	4-:128896506-128916340
4271	Rps18	20084	17-:34090402-34091507
4272	Hsd17b6	27400	10-:127428006-127437732
4273	MMT00052322		1+:17937133-17938561
4274	Aof1	218214	13+:47158334-47175957
4275	EG331392	331392	X+:20938065-20940149
4276	Chmp4b	75608	2+:154482975-154520518
4277	MMT00042444		1+:75621414-75622277
4278	Zfp787	67109	7-:6083089-6094832
4279	Vmn2r117	619788	17-:23596643-23616564
4280	Cecr6	94047	6-:120438956-120443825
4281	Fbn2	14119	18-:58169911-58369459
4282	RP23-100C5.8	241638	2-:130460502-130468535

4283	Tom1	21968	8+:77557607-77594010
4284	Irf1	16362	11+:53583579-53590825
4285	Dnajc19	67713	3-:33976206-33980243
4286	Ptp4a1	19243	1-:30998436-31006590
4287	Rnf7	19823	9-:96371357-96379014
4288	MMT00072334		15-:18995921-19295653
4289	MMT00057248		X+:16187190-16188720
4290	Ube2g1	67128	11+:72420849-72497807
4291	Impa1	55980	3-:10315185-10329005
4292	5330431K02Rik	68189	13-:100272431-100274394
4293	MMT00019701		10-:90695465-90723993
4294	MMT00034521		X-:54998947-54999480
4295	Xcl1	16963	1-:166861778-166865641
4296	Ppp2r2c	269643	5+:37314202-37344192
4297	Ptdss1	19210	13+:67034358-67098944
4298	St3gal1	20442	15-:66937849-66945371
4299	Lsm8	76522	6+:18798634-18804052
4300	Lasp1	16796	11+:97661044-97697650
4301	AW049604	106014	15+:87455659-87589792
4302	Vmn2r120	224916	17-:57648206-57665382
4303	Phkg1	18682	5-:130339308-130354960
4304	Tgif1	21815	17-:71193950-71200376
4305	Tmem51	214359	4-:141586907-141593849
4306	Cpeb1	12877	7-:88492329-88599562
4307	Arih2	23807	9-:108507002-108547325
4308	Nphp3	74025	9+:103917654-103918975
4309	MMT00010397		1+:20811386-20812224
4310	B3gnt8	232984	7+:26412656-26414509
4311	MMT00033987		4-:40808262-40860214
4312	MMT00014395		4-:67179411-67179966
4313	Ppap2b	67916	4+:104829964-104903745
4314	2310046A06Rik	69642	9-:76986155-77091538
4315	Rpl34	68436	3-:130429748-130433293
4316	EG627624	627624	19+:43355265-43356647
4317	Spire1	68166	18-:67647866-67712461
4318	MMT00065533		10-:77144089-77153502
4319	1810009O10Rik	69109	11-:78564413-78565164
4320	Madcam1	17123	10+:79127331-79131281
4321	MMT00066182		5+:52972590-52975039
4322	MMT00045154	624701	3+:107782502-107782872
4323	Pcdhb16	93887	18+:37637642-37640049
4324	MMT00010259		2-:111022791-111027563
4325	Slc16a4	229699	3+:107094209-107115026
4326	Gtpbp3	70359	8+:74012001-74017309
4327	Klf10	21847	15-:38224168-38230462
4328	Spag11	78128	8+:19141376-19159577
4329	Pbx1	18514	1-:170050129-170362087
4330	Eif2b5	224045	16+:20498899-20509398
4331	OTTMUSG00000(	433779	4+:143951538-143953781
4332	Sf3b1	81898	1-:55042015-55084323
4333	Sumo3	20610	10+:77069014-77080121

4334	Has3	15118	8+:109394141-109406802
4335	Hsf2	15500	10+:57206203-57232264
4336	Rbbp9	26450	2-:144369557-144376514
4337	Myst3	244349	8+:23971985-24053489
4338	EG328280	328280	13+:67884730-67944975
4339	Gemin6	67242	17+:80623828-80627837
4340	Sdk2	237979	11-:113641489-113731229
4341	Olfr600	259048	7-:110494497-110495440
4342	7530428D23Rik	319506	3-:21652450-21652888
4343	Dnahc6	330355	6-:73110857-73123555
4344	1810073G14Rik	67726	11-:57296501-57332117
4345	MMT00074600		8-:41939180-41940429
4346	Ccdc46	76380	11+:108543937-108721925
4347	Mfn2	170731	4-:147247707-147278813
4348	Nedd9	18003	13-:41406940-41582665
4349	Mrpl27	94064	11+:94515109-94521209
4350	Mllt4	17356	17+:13898611-14036511
4351	Plxnb1	235611	9+:109002591-109022421
4352	Rtn4rl1	237847	11+:75078258-75081263
4353	ENSMUSG00000(	553138	19-:29795136-29880313
4354	9330176C04Rik	109280	9-:119129609-119140810
4355	Pard6a	56513	8+:108225582-108227366
4356	Rtbdn	234542	8+:87474046-87480501
4357	Nup98	269966	7-:109268968-109344402
4358	Apba2	11784	7+:71839929-71898255
4359	Tmem208	66320	8+:107850282-107852955
4360	MMT00020996		7-:79857676-79858507
4361	BC046331	230967	4+:153353052-153382851
4362	Krt34	16672	11-:99898664-99902868
4363	2810002I04Rik	72350	12+:86629575-86638361
4364	MMT00014218		3-:14163492-14164042
4365	Tapbp	21356	17+:34056632-34063651
4366	MMT00009414		10-:129880357-129882065
4367	Atp6v0e	11974	17+:26813340-26836585
4368	Ppp1r13b	21981	12-:113066662-113110644
4369	Acot4	171282	12+:85379460-85384746
4370	Psmd8	57296	7-:29959273-29965550
4371	Wdr55	67936	18+:36919911-36923178
4372	Msl2l1	77853	9+:100978479-101007283
4373	Wdr59	319481	8-:113974794-114045887
4374	MMT00048995		2-:39982438-39983458
4375	Cysltr2	70086	14-:73428934-73448879
4376	2310015A05Rik	72315	16+:17646642-17650830
4377	Mgl2	216864	11+:69943921-69951049
4378	1700026D08Rik	75556	7-:90924128-90943336
4379	Glrx	93692	13+:75977354-75987416
4380	Smarcb1	20587	10-:75359519-75384333
4381	Mfge8	17304	7-:86278654-86293891
4382	Gtlf3a	24082	11-:60734442-60745369
4383	MMT00075628		12-:5596370-5597476
4384	Gamt	14431	10-:79720943-79723757

4385	Sfrp4	20379	13+:19715051-19724690
4386	Sptlc1	268656	13-:53428120-53472729
4387	Frs3	107971	17+:47832155-47841235
4388	MMT00019672		X-:96276741-96277655
4389	MMT00037766	78376	17-:35162921-35164861
4390	Hps5	246694	7-:54015839-54051033
4391	Zfp467	68910	6-:48387584-48395606
4392	1810055G02Rik	72056	19+:3708332-3717881
4393	Otud7a	170711	7+:70589657-70903913
4394	Tcl1b3	27378	12+:106429252-106433820
4395	Tspo	12257	15+:83401816-83404473
4396	MMT00071908		8+:5079224-5080930
4397	MMT00011217		X+:164425451-164426156
4398	Aqp4	11829	18-:15551345-15561930
4399	Olfr2	18317	7-:114144388-114145372
4400	9030425E11Rik	71566	9+:40494129-40591188
4401	Olfr1446	258699	19-:12964138-12965065
4402	Lrrk1	233328	7-:73453546-73475703
4403	MMT00052895		10+:88104110-88104617
4404	Commd5	66398	15+:76730370-76731727
4405	Wdr49	213248	3-:75253797-75255740
4406	MMT00003918		6+:41032339-41042339
4407	4930429B21Rik	67576	3+:32264696-32266358
4408	MMT00080682		9+:61381221-61382055
4409	5430435G22Rik	226421	1+:133585301-133610044
4410	Rpl7a	27176	2+:26766524-26768831
4411	2310039H08Rik	67101	17+:46909760-46910344
4412	Lrrc27	76612	7+:146416101-146428874
4413	MMT00030347		5-:12354694-12355404
4414	5033406O09Rik	77675	12-:113180557-113182419
4415	B230399E16Rik	240479	18+:84889670-84906366
4416	5730446C15Rik	226016	19+:21755125-21760127
4417	Stom	13830	2-:35169510-35192529
4418	H2-Aa	14960	17-:34419804-34424733
4419	Alg14	66789	3+:120994734-121064928
4420	Nedd8	18002	14-:56281101-56290710
4421	Synj1	104015	16-:90936337-91011554
4422	Alx1	216285	10-:102471500-102491654
4423	Cep57	74360	9-:13612632-13631497
4424	9530058B02Rik	68241	17-:26000644-26005683
4425	Olfr1346	258918	7+:6426822-6427763
4426	Iigp2	54396	11+:58032970-58036282
4427	Ccdc55	237859	11-:76861894-76891908
4428	Prl8a8	74188	13-:27598940-27605082
4429	MMT00042276		13+:7307045-7308207
4430	Ankrd36	76389	11+:5469687-5484284
4431	Tmem17	103765	11+:22412082-22419236
4432	Pcdha5	12941	18+:37120093-37347311
4433	Snrpa1	68981	7+:73205454-73219468
4434	Snx14	244962	9-:88271585-88333777
4435	Hat1	107435	2+:71227305-71279574

4436	Wwc1	211652	11-:35653727-35728798
4437	Bat3	224727	17+:35271649-35284265
4438	1700017N19Rik	66605	10+:100057167-100081024
4439	Kcnip1	70357	11-:33529789-33743573
4440	Met	17295	6+:17441240-17521822
4441	Ccdc88b	78317	19-:6920835-6928592
4442	Tmprss6	71753	15-:78270099-78299057
4443	Zfp790	233056	7+:30601137-30615958
4444	Ksr2	333050	5+:117955497-118005945
4445	Mzf1	109889	7-:13628498-13639033
4446	Myo5c	208943	9+:75135637-75153255
4447	Ngrn	83485	7+:87406133-87410260
4448	MMT00002500		18-:9496762-9497112
4449	MMT00064573		
4450	Brs3	12209	X+:54296421-54300883
4451	Lrrfip1	16978	1+:92950083-93025495
4452	Phlda2	22113	7-:150687454-150688415
4453	MMT00050565		3-:135623162-135626780
4454	BC055107	268709	14-:9130066-9150355
4455	Ccdc76	229780	3-:116283647-116297970
4456	Elfn2	207393	15-:78497764-78499260
4457	Cks2	66197	13+:51740714-51746023
4458	1700040I03Rik	73327	6-:85396802-85401261
4459	Crybb3	12962	5-:113504860-113509673
4460	MMT00011069		11+:84591431-84633143
4461	Egln1	112405	8-:127439598-127445679
4462	Cyp1a1	13076	9+:57545419-57551629
4463	Ncoa5	228869	2-:164825857-164860279
4464	BC028471	414071	5+:150171612-150195061
4465	Tmed1	17083	9-:21310001-21314629
4466	MMT00014327	233575	7+:109371587-109386263
4467	Ccdc126	57895	6+:49269214-49290972
4468	MMT00052724		
4469	Cyp3a13	13113	5-:138335222-138362823
4470	Slc5a8	216225	10+:88348781-88398828
4471	Sdccag8	76816	1+:178754869-178951013
4472	MMT00074121	100040305	12-:103213872-103215162
4473	EG628040	628040	X-:8903532-8905467
4474	EG627022	627022	6-:136434167-136435708
4475	Rufy2	70432	10+:62442970-62479280
4476	Rpgr	19893	X-:9735348-9793839
4477	Jph1	57339	1-:16987544-17087879
4478	Exoc6b	75914	6-:84939320-85019468
4479	Cdc42ep3	260409	17-:79733364-79754431
4480	Bahcc1	268515	11+:120135297-120151196
4481	Olfr1100	258587	2-:86817987-86818951
4482	Klhl18	270201	9-:110328433-110379183
4483	Syt1	20979	10-:107937005-108447898
4484	MMT00014015		13+:61767510-61768501
4485	Brd4	57261	17-:32333796-32390131
4486	MMT00064996	100046252	12-:116305763-116757993

4487	Gdpd2	71584	X+:97925339-97934238
4488	Ssx2ip	99167	3+:146067650-146103099
4489	Tusc4	56032	9+:107444558-107448026
4490	Msmb	17695	14+:32955240-32971510
4491	E2f6	50496	12+:16817832-16833549
4492	Suv420h1	225888	19+:3768145-3805463
4493	Ptgir	19222	7+:17491838-17496243
4494	Cplx2	12890	13+:54479948-54481562
4495	Nid1	18073	13+:13529957-13604521
4496	MMT00017973		12+:63270822-63538952
4497	Myo3b	329421	2+:70076813-70152495
4498	Cadps2	320405	6-:23788789-23789244
4499	Enpp2	18606	15-:54670455-54751557
4500	Usp53	99526	3-:122655472-122657473
4501	MMT00033071		
4502	Cdkal1	68916	13-:29417176-29947457
4503	Abca14	67928	7+:127347517-127356540
4504	1700113I22Rik	73635	11-:101280127-101286642
4505	Rpl10	110954	X+:71516198-71518463
4506	Zc3h7b	20286	15+:81598389-81626700
4507	Mcph1	244329	8+:18631515-18803185
4508	Ifit1	15957	19+:34715403-34723970
4509	Kif14	381293	1+:138364534-138386961
4510	Ttf1	22130	2+:28915796-28942613
4511	P2rx1	18436	11+:72812651-72828004
4512	Esrra	26379	19-:6985467-6996256
4513	MMT00030775		13-:32807727-32816753
4514	MMT00045906		5-:117799052-117799379
4515	MMT00028055		2-:4755179-4803523
4516	Rsad2	58185	12-:27130172-27141293
4517	Itih3	16426	14-:31721761-31736728
4518	Gpr65	14744	12+:99506866-99514842
4519	Ubash3b	72828	9-:40821726-40887589
4520	Ppp1r11	76497	17-:37086231-37088629
4521	Helb	117599	10-:119520663-119549946
4522	MMT00003022		10-:6352873-6354170
4523	Ptpru	19273	4-:131324372-131394193
4524	Usp12	22217	5-:147546398-147606583
4525	Zfp26	22688	9-:20238133-20244718
4526	Satb1	20230	17-:51878793-51971958
4527	Gcn1l1	231659	5+:116015278-116072659
4528	Lrguk	74354	6+:33979442-34084035
4529	C130057D23Rik	328505	4+:111652874-111691912
4530	Ly9	17085	1-:173518762-173537405
4531	EG434175	434175	7-:49361153-49362629
4532	BC034090	207792	1-:157059602-157089407
4533	Itgb1	16412	8+:131208845-131256651
4534	Ndel1	83431	11-:68634950-68666555
4535	MMT00025220		7-:94846328-94847249
4536	BC013529	215751	10-:7487753-7500703
4537	Olfr44	18343	9-:39291891-39292653

4538	9430081H08Rik	77430	9-:44182824-44183333
4539	MMT00043977		18+:56805031-56827698
4540	Prl8a2	13529	13+:27437557-27446073
4541	Ccdc37	243538	6-:90365692-90378474
4542	Tchp	77832	5+:115158662-115169773
4543	9230112E08Rik	320800	2+:180741409-180753048
4544	MMT00054707		X-:33753524-33754133
4545	Fgd5	232237	6+:92015141-92018571
4546	Gimap9	317758	6+:48626190-48628787
4547	Vps13a	271564	19-:16689756-16752893
4548	Olfr857	257963	9+:19494811-19495490
4549	9930012K11Rik	268759	14-:70554213-70556326
4550	Stard10	56018	7+:108465632-108494825
4551	Cyp2e1	13106	7+:147949637-147960874
4552	4930441O14Rik	68271	13+:67845140-67857775
4553	Xpnpep3	321003	15+:81230579-81283678
4554	Cer1	12622	4-:82527654-82531055
4555	Cchcr1	240084	17+:35654120-35667950
4556	Mapk6	50772	9-:75236277-75246447
4557	MMT00031263		12-:52234347-52235904
4558	Alg3	208624	16-:20605545-20610817
4559	Freq	14299	2+:31101695-31147907
4560	1700011F14Rik	75645	19+:58586480-58627575
4561	Itgbl1	223272	14+:124059394-124373299
4562	Col11a2	12815	17+:34176381-34203187
4563	Ythdc1	231386	5+:87232799-87265682
4564	Smtnl2	276829	11-:72204658-72225229
4565	MMT00019450		7+:54604843-54605630
4566	MMT00078134	547323	14+:53511660-53512863
4567	MMT00004627		9-:75222413-75223332
4568	MMT00022640		8-:91670027-91671049
4569	MMT00007978	243420	6+:67505614-67560601
4570	Ikzf2	22779	1-:69585252-69732408
4571	3830431G21Rik	217682	12+:81808158-81823368
4572	Mrps26	99045	2+:130389076-130391129
4573	Nrxn1	18189	17-:91391454-91488944
4574	MMT00064324		18+:14583369-14650895
4575	MMT00052635		18-:77457092-77457950
4576	Chchd7	66433	4+:3866034-3870674
4577	Ltbp4	108075	7-:28056959-28104789
4578	Pglyrp2	57757	17-:32550305-32557920
4579	Dpys	64705	15-:39614243-39689003
4580	Impact	16210	18+:13130760-13151457
4581	MMT00064145		6+:112513608-112516303
4582	Armcx4	102910	X+:131221039-131231292
4583	Krtap14	23927	16-:88825535-88826375
4584	MMT00022168		13+:18041731-18042930
4585	V1re2	171225	17+:20824471-20825368
4586	Zfp618	72701	4+:62694927-62794905
4587	Ufm1	67890	3-:53660517-53667692
4588	Tsr1	104662	11+:74711605-74722339

4589	MMT00035806		1+:43842905-43843808
4590	Arhgap21	71435	2-:20769558-20793395
4591	Kif5c	16574	2+:49475181-49614434
4592	Slc25a39	68066	11-:102264460-102268786
4593	Il1b	16176	2-:129190765-129196066
4594	Kcnt1	227632	2+:25719383-25772656
4595	Tsr2	69499	X-:147523730-147531064
4596	MMT00050448		17-:5481575-5483005
4597	MMT00051225		10+:3820035-3820664
4598	Olfr1052	259012	2+:86137974-86138911
4599	MMT00030633	637260	6-:70264776-70291185
4600	Sp2	78912	11-:96815533-96844273
4601	Th	21823	7-:150078680-150085871
4602	Pdzrn3	55983	6-:101099608-101327891
4603	MMT00009680		14+:29538056-29538230
4604	Cyfip2	76884	11-:46009590-46125795
4605	Myo1f	17916	17+:33692734-33744703
4606	Nudt22	68323	19-:7067508-7070527
4607	Acad11	102632	9+:103966014-104029975
4608	Lag3	16768	6-:124854378-124861723
4609	Cdkl4	381113	17-:80942952-80976796
4610	F830016B08Rik	240328	18+:60452634-60460520
4611	Robo2	268902	16-:74348790-74887447
4612	Hspa5	14828	2+:34627749-34631981
4613	MMT00063767		19+:37858747-37860255
4614	Slc6a18	22598	13-:73801484-73815449
4615	Lmx1b	16917	2-:33420156-33496031
4616	Kiss1r	114229	10+:79381418-79384748
4617	Agpat9	231510	5+:101275149-101328115
4618	Mrps9	69527	1+:42952194-42962278
4619	Htra3	78558	5-:35994690-36022214
4620	MMT00008456		8-:44785106-44809305
4621	Slc7a6os	66432	8-:108724507-108735053
4622	Zfp295	114565	16-:98170547-98183788
4623	MMT00026770		9-:35396664-35396935
4624	MMT00055457		3-:103448755-103449946
4625	Cwc15	66070	9+:14305171-14314988
4626	2310046K01Rik	69698	2+:151822363-151834992
4627	Sec31a	69162	5-:100790669-100837131
4628	MMT00040865		2+:150017591-150151534
4629	MMT00007987		17+:80453360-80453903
4630	Akr1c20	116852	13-:4506404-4522579
4631	Itgb2	16414	10+:77004727-77028128
4632	Mapk3	26417	7+:133903145-133909329
4633	Olfr125	258287	17+:37971945-37972906
4634	Prdm6	225518	18+:53633115-53735055
4635	MMT00023332		15+:100127548-100128056
4636	Ebf3	13593	7-:144386923-144505669
4637	Tsga10ip	78306	19-:5390048-5400459
4638	Alkbh4	72041	5+:136612024-136617482
4639	Prf1	18646	10+:60762548-60766814

4640	Ccdc42	276920	11+:68400537-68411451
4641	MMT00012367		11-:87282523-87283310
4642	Dact3	629378	7+:17460773-17472812
4643	Mbl2	17195	19+:30307446-30314171
4644	Gm1964	432628	12-:4872964-4878307
4645	MMT00030322		
4646	Adcy7	11513	8+:90796301-90852929
4647	9630050M13Rik	269233	2+:3095502-3145064
4648	1700007G11Rik	75784	5+:99127420-99231068
4649	A230067G21Rik	241694	2-:146070108-146269747
4650	RP23-438H3.2	69357	X+:90428631-90429280
4651	Itgb6	16420	2-:60438541-60514123
4652	Podxl2	319655	6-:88792551-88815283
4653	Slc25a4	11739	8-:47292037-47296375
4654	Myl9	98932	2+:156601152-156607393
4655	1810029B16Rik	66282	8-:69000244-69010404
4656	4930505A04Rik	75087	11-:30326004-30371831
4657	C230096C10Rik	230866	4+:138918044-138931841
4658	Thada	240174	17-:84591489-84672166
4659	Pramel6	347711	2+:87346733-87351032
4660	MMT00011546		3+:95232484-95234360
4661	Rprml	104582	11+:103511094-103511447
4662	Mpzl2	14012	9+:44850645-44860408
4663	MMT00068443	100047498	X+:51397420-51399464
4664	MMT00079290		13+:108138207-108138595
4665	Dynll1	56455	5-:115748543-115750933
4666	Exosc9	50911	3+:36451564-36464647
4667	Ssh1	231637	5-:114387777-114443733
4668	4921511H13Rik	207932	16-:90751773-90810630
4669	H2-M10.5	224761	17+:36870567-36871698
4670	8430426H19Rik	71508	13-:62504184-62706778
4671	Itga7	16404	10+:128370873-128395338
4672	Mthfr	17769	4+:147415695-147429696
4673	Slit1	20562	19-:41674745-41818069
4674	Cxcl12	20315	6+:117118595-117131384
4675	Sparcl1	13602	5-:104508131-104542705
4676	Cenph	26886	13-:101529640-101545854
4677	BC016423	105203	13-:3565281-3574438
4678	Kank1	107351	19+:25311462-25508987
4679	Cxcl15	20309	5+:91223596-91230911
4680	4930449I24Rik	67410	5+:147337699-147339343
4681	Noxa1	241275	2-:24946011-24950699
4682	Sertad4	214791	1-:194670625-194683097
4683	E330013P04Rik	107376	19+:60220559-60236173
4684	MMT00006152	268859	16+:5885447-7410072
4685	Sema4c	20353	1-:36605484-36615106
4686	BC021785	215928	10-:39666719-39706481
4687	Gtpbp8	66067	16-:44739098-44746472
4688	Kazald1	107250	19+:45150580-45153752
4689	Glp1r	14652	17+:31038821-31073478
4690	Kif19a	286942	11+:114626827-114647960

4691	Ttll6	237930	11+:95996324-96008894
4692	MMT00049231		1+:10858784-10859392
4693	Ifna11	15964	4+:88465796-88467279
4694	MMT00046622		7-:113465907-113471636
4695	D330027H18Rik	101359	6-:29121509-29127222
4696	Tac1	21333	6+:7505070-7512973
4697	Cyp2a12	13085	7+:27814108-27821829
4698	Pibf1	52023	14+:99499176-99513436
4699	Bglap1	12096	3-:88187422-88188355
4700	MMT00015274		9+:37493516-37494309
4701	MMT00062346	236260	12+:17888729-17945713
4702	Tmem39b	230770	4-:129353600-129374040
4703	Nubpl	76826	12+:53198723-53411946
4704	MMT00043860		13+:101162662-101164456
4705	2610528K11Rik	72543	2-:33588690-33730043
4706	Olfr1299	258886	2+:111504397-111505321
4707	Hsd3b5	15496	3-:98383764-98385715
4708	Traf3ip2	103213	10+:39359186-39374629
4709	Prune2	77754	19+:17030605-17129452
4710	Lin7a	108030	10+:106708955-106858155
4711	Ccdc108	241116	1-:74948646-74950020
4712	Sdhc	66052	1-:173059295-173080733
4713	MMT00078464		7+:137110717-137122183
4714	Bax	12028	7-:52717232-52722145
4715	Tmem120a	215210	5-:136173097-136220096
4716	Tmem108	81907	9-:103386764-103402538
4717	Cd177	68891	7-:25539571-25545331
4718	MMT00069301	100044315	X-:103477107-103478363
4719	Col2a1	12824	15-:97806472-97834924
4720	Ckm	12715	7+:19996442-20006932
4721	Cdh13	12554	8+:121198909-121380107
4722	Tmco7	272538	8+:109206994-109375028
4723	Art3	109979	5+:92760842-92843656
4724	MMT00033577		6+:40997339-40998100
4725	Slc10a4	231290	5+:73398171-73404192
4726	MMT00016896		12-:27170766-27171735
4727	Phkg2	68961	7+:134622734-134729915
4728	EG574403	574403	11+:34301959-34327977
4729	Slc7a10	53896	7+:35971489-35985965
4730	MMT00079671		13-:83885718-83901943
4731	Zmynd12	332934	4+:119095435-119126500
4732	Idh3a	67834	9+:54434296-54451596
4733	Snap91	20616	9-:86659238-86775207
4734	Ccbl2	229905	3+:142364039-142407498
4735	Gmip	78816	8+:72332625-72345683
4736	Stk31	77485	6+:49348194-49419500
4737	Olfr449	259067	6+:42787881-42788817
4738	Ppm1a	19042	12+:73884668-73894849
4739	Nat9	66176	11-:115044143-115048583
4740	Rxfp1	381489	3-:79448806-79509575
4741	MMT00070324	100041422	14+:44701632-44704860

4742	Ccne2	12448	4+:11119850-11130244
4743	Rasl10a	75668	11+:4958308-4960067
4744	MMT00027592		12-:116081838-116134753
4745	Lhfp	108927	3+:53035649-53065600
4746	1810007M14Rik	67367	16-:91015187-91034444
4747	Mll1	214162	9-:44611444-44689131
4748	Xylt2	217119	11-:94525166-94531854
4749	9230107M04Rik	77684	7-:14140214-14156419
4750	Gngt1	14699	6+:3943817-3947437
4751	Map3k4	26407	17-:12420488-12511614
4752	Pik3r4	75669	9+:105546566-105589978
4753	Cdk7	12572	13-:101472587-101500928
4754	MMT00027078		
4755	Olfr394	259009	11-:73700941-73701872
4756	Arx	11878	X+:90531859-90542859
4757	Mus81	71711	19-:5482893-5488151
4758	MMT00056158		8+:3994376-3999348
4759	Gnai2	14678	9-:107522028-107522804
4760	MMT00038902	328277	13+:67761331-67800906
4761	Kcnv1	67498	15-:44939367-44946463
4762	MMT00060319		2-:64705955-64706777
4763	Svop	68666	5-:114476977-114541544
4764	MMT00031970		16-:43638534-43641910
4765	6430706D22Rik	381280	1+:90131544-90133306
4766	Kcnj6	16522	16-:94982675-95219255
4767	Ktn1	16709	14+:48283453-48356232
4768	Lsm10	116748	4+:125773995-125775823
4769	Stoml2	66592	4-:43040893-43044275
4770	4933411G06Rik	71090	10-:51475979-51477000
4771	C2cd3	277939	7+:107578650-107592072
4772	2810007J24Rik	76971	7-:14996067-15032119
4773	Il20	58181	1-:132803768-132807873
4774	Ccdc112	240261	18-:46445266-46471548
4775	MMT00038658		
4776	Dclk1	13175	3+:55046549-55340599
4777	2810459M11Rik	72792	1+:87942842-87952030
4778	Cyct	13067	2-:76192086-76198505
4779	Dohh	102115	10+:80847255-80851095
4780	Sfrs5	20384	12+:82047562-82051486
4781	Hisppd1	227399	1-:99602622-99666708
4782	Clock	12753	5-:76645723-76695871
4783	Arhgef17	207212	7-:108020564-108080253
4784	Slc2a2	20526	3+:28596898-28627249
4785	ORF63	224419	16+:87553574-87595580
4786	Psg16	26436	7+:17659431-17683864
4787	Parn	74108	16-:13538056-13668261
4788	Pak3	18481	X+:140100287-140226353
4789	Capns1	12336	7-:30971967-30980043
4790	Nrg3	18183	14-:40278445-40340075
4791	ENSMUSG00000(	208595	5+:4195612-4197650
4792	Neurod6	11922	6-:55627816-55631063

4793	Bbs1	52028	19-:4888746-4906638
4794	Gsdmd	69146	15+:75692768-75697834
4795	Trpa1	277328	1-:14863689-14871860
4796	Rcbtb2	105670	14+:73554161-73582489
4797	Fbxo25	66822	8+:13907805-13940521
4798	C330011K17Rik	238692	13-:67541368-67549852
4799	Stac3	237611	10+:126940144-126945863
4800	Phactr1	218194	13+:42776164-42805219
4801	MMT00057721		18+:23375130-23376062
4802	5430433G21Rik	319909	2+:139504507-139584294
4803	Oog4	242737	4-:143027065-143030105
4804	Sart3	53890	5-:114192454-114221658
4805	Hspb1	15507	5+:136363928-136365307
4806	Slc7a13	74087	4+:19745900-19769082
4807	Fgl2	14190	5+:20878499-20882878
4808	Apcdd1	494504	18+:63112743-63113459
4809	Cacna1b	12287	2-:24461894-24618672
4810	Vsig8	240916	1+:174486202-174493847
4811	Gripap1	54645	X+:7367142-7397687
4812	Myom1	17929	17+:71368860-71476195
4813	Agpat6	102247	8-:24284988-24293313
4814	MMT00050024		16+:3534292-3534809
4815	Upf1	19704	8-:72855428-72877178
4816	Kcnmb1	16533	11+:33863012-33870749
4817	MMT00046203		
4818	Enpep	13809	3-:128972537-129035486
4819	MMT00082285		14+:19363856-19365450
4820	2210409D07Rik	70157	18-:57791732-57798242
4821	Lrrc41	230654	4+:115748062-115769920
4822	BC032265	238024	11+:121282744-121291028
4823	Efhc2	74405	X-:16778168-16896493
4824	Gtsf1	74174	15-:103232891-103260837
4825	Nfkbil1	18038	17-:35357120-35372760
4826	Sypl2	17306	3-:108015183-108029517
4827	Sh3bp4	98402	1+:90967036-91050367
4828	MMT00047886		17-:77761900-77794305
4829	Hspa2	15512	12+:77505520-77507422
4830	Kif27	75050	13-:58388877-58456223
4831	Ccdc57	71276	11-:120763292-120764994
4832	Nxnl2	75124	13+:51266393-51270556
4833	Synpo2	118449	3-:122815319-122817593
4834	4930553D19Rik	75266	12+:72212430-72224210
4835	C1qtnf4	67445	2+:90726016-90730676
4836	Gtf2h3	209357	5+:125029100-125046849
4837	Ear2	13587	14+:44680551-44681022
4838	Secisbp2	75420	13+:51747089-51779422
4839	Cyp4a32	100040843	4+:115236254-115294212
4840	Tmem191c	224019	16+:17276400-17278755
4841	EG382106	382106	9-:109503630-109527466
4842	MMT00012981		4+:105758144-105786199
4843	Oma1	67013	4+:102986450-103039033

4844	Zfp709	236193	8+:74406017-74415600
4845	MMT00071334		8-:11714758-11715284
4846	BC022623	224093	16+:30600566-30603166
4847	Sbpl	638345	17-:24090051-24091883
4848	Flt3	14255	5-:148142321-148211871
4849	Zxdc	80292	6+:90319527-90333086
4850	Fancd2	211651	6+:113481695-113547011
4851	Gpn2	100210	4+:133140294-133147646
4852	Kdelr2	66913	5+:144165504-144182947
4853	Plxnc1	54712	10-:94255931-94407212
4854	Tgfbr1	21812	4+:47366183-47423796
4855	Csrnp3	77771	2+:65683869-65716875
4856	Mlf1ip	71876	8+:47637381-47664937
4857	2210010N04Rik	70381	5-:144871958-144872641
4858	V1re13	252910	7-:11333186-11334108
4859	Mef2d	17261	3+:87946348-87972645
4860	Mapkbp1	26390	2+:119798638-119851074
4861	Pctk1	18555	X+:20265282-20277003
4862	MMT00048585		13-:74155983-74157632
4863	Atad4	217138	11+:96790637-96796959
4864	S100a16	67860	3+:90341297-90347070
4865	Hist1h1b	56702	13-:21871751-21872423
4866	Cnot6	104625	11-:49485008-49516159
4867	5830403F22Rik	74743	7-:141376576-141423815
4868	Mertk	17289	2+:128524732-128627924
4869	4930432H15Rik	73866	X+:50626609-50684362
4870	Olfr550	259108	7+:109727010-109727959
4871	MMT00058874		3-:46854304-46855563
4872	Serpinc1	11905	1+:162917673-162932592
4873	5-Mar	69104	19+:37282057-37295555
4874	Tbc1d9	71310	8+:85689972-85796793
4875	Rapsn	19400	2+:90875797-90885886
4876	Purb	19291	11-:6374791-6375920
4877	Prss8	76560	7-:135069236-135073608
4878	MMT00079752		2-:155284598-155286632
4879	Olfr784	258724	10+:128824690-128825650
4880	Gm505	244666	8+:127422057-127427362
4881	4930434E21Rik	381693	5+:150408863-150414465
4882	MMT00027957		7+:52852292-52854937
4883	Lysmd3	80289	13+:81796804-81810887
4884	Cpne2	234577	8+:97056935-97094430
4885	Fhad1	329977	4-:141484294-141507592
4886	Jarid1a	214899	6+:120314475-120369459
4887	MMT00071942	620515	3+:106100602-106101941
4888	MMT00006903		X-:59064648-59065062
4889	Bphl	68021	13+:34129509-34165943
4890	Pex5	19305	6-:124347922-124364451
4891	Atp6v0b	114143	4-:117556934-117559934
4892	Zfp109	56869	7-:25012590-25022579
4893	V1rg12	171268	7+:12890153-12891066
4894	Cox7a2	12866	9-:79603214-79607492

4895	Pdk3	236900	X-:91009956-91077434
4896	4930405M20Rik	73942	13-:93219577-93254049
4897	Ciao1	26371	2-:127068300-127073496
4898	MMT00072808		14-:114708037-114710025
4899	Nr4a2	18227	2-:56960568-56967666
4900	5031426D15Rik	68144	2-:6845969-6849768
4901	MMT00073557	171504	7+:133728521-133732598
4902	6030498E09Rik	77883	X+:36311239-36315864
4903	MMT00017132		8+:75877056-76176078
4904	Tinf2	28113	14-:56297933-56300645
4905	Urod	22275	4-:116662822-116666965
4906	A830059I20Rik	58238	7+:100228428-100230228
4907	Nup155	170762	15+:8059330-8107827
4908	5530401N06Rik	71386	1+:83057504-83058496
4909	Olfr698	258595	7-:113895964-113896900
4910	5530400C23Rik	232426	6+:133242234-133245807
4911	Nme4	56520	17-:26228689-26232415
4912	Kcnq4	60613	4-:120370499-120419715
4913	Zfp619	70227	7+:46753549-46822953
4914	Acan	11595	7+:86198368-86259984
4915	Asb18	208372	1-:91849252-91911152
4916	Gabpb1	14391	2-:126464756-126501222
4917	MMT00081200		1-:7385719-7640787
4918	Scara5	71145	14+:66285263-66383660
4919	Med12l	329650	3+:58809934-58897619
4920	Rbl1	19650	2-:156971623-157025312
4921	MMT00014712		13-:49074183-49076609
4922	Mmp19	58223	10+:128228028-128236524
4923	BC059842	230676	4-:118035038-118059595
4924	Tmprss3	140765	17-:31316214-31334694
4925	Iah1	67732	12+:21322274-21327219
4926	Vps4a	116733	8+:109555225-109569655
4927	Plcb4	18798	2+:135703118-135733316
4928	MMT00048249		3+:159154379-159154608
4929	Usp8	84092	2+:126543780-126585017
4930	Ankrd13c	433667	3+:157610400-157671296
4931	EG622139	622139	3-:144524608-144552709
4932	Cd70	21948	17-:57285420-57289200
4933	Clec14a	66864	12-:59366922-59369883
4934	Abca13	268379	11+:9235231-9528671
4935	Pcdhb5	93876	18+:37480222-37482599
4936	MMT00031931		18-:80072327-80073065
4937	Krtap26-1	69533	16-:88647068-88648041
4938	Mccc2	78038	13-:100718483-100785567
4939	1700023B02Rik	66935	2-:73121928-73150560
4940	Ss18	268996	18-:14784139-14841423
4941	MMT00043485		3-:46249887-46251871
4942	Kcnj12	16515	11+:60882684-60884302
4943	Thap11	59016	8+:108379042-108380850
4944	MMT00067472		
4945	Grin3a	242443	4-:49676956-49857953

4946	Ipp	16351	4+:116202275-116210847
4947	Erich1	234086	8-:14027908-14090291
4948	Arl2	56327	19-:6134667-6141101
4949	Taok2	381921	7-:134009189-134024277
4950	Dnajb10	56812	1+:75233130-75242265
4951	Lsm3	67678	6+:91466028-91472614
4952	MMT00016217		4-:10365963-10366981
4953	Etv3	27049	3+:87331177-87340618
4954	Puf60	67959	15-:75900619-75911300
4955	Cxcl1	14825	5+:91320335-91322141
4956	OTTMUSG000000	435285	11+:99708285-99712796
4957	Olfr623	259126	7-:110808809-110809762
4958	Ndufa7	66416	17+:33961574-33975133
4959	Optn	71648	2-:4941686-4963566
4960	MMT00067115		17-:18048003-18049853
4961	MMT00058801		3+:37630868-37633894
4962	MMT00017883	232934	7+:19576667-19587112
4963	Hyi	68180	4+:118032600-118035936
4964	MMT00003197		17-:73048798-73049646
4965	Kl	16591	5+:151755273-151796386
4966	Zscan20	269585	4-:128262620-128281844
4967	Plekhm1	353047	11-:103241432-103274027
4968	2510027J23Rik	66568	3-:120873900-120874612
4969	Acvr1b	11479	15+:101004566-101041941
4970	MMT00013473		13+:51020708-51062118
4971	Hist1h4i	319158	13-:22129747-22133192
4972	Sec16b	89867	1+:159456300-159498196
4973	Grik4	110637	9-:42616574-42616709
4974	Ace	11421	11+:105829271-105851275
4975	MMT00036512		
4976	Psmc1	19179	12+:101350539-101361574
4977	MMT00066891	666038	5-:134317440-134318539
4978	Cherp	27967	8-:74983942-74999124
4979	Vmn2r15	211223	5-:109715287-109726575
4980	MMT00006445		6-:68966533-68993274
4981	MMT00008799		3+:10232103-10232459
4982	4930449A18Rik	74672	3+:59642287-59650737
4983	Rabgef1	56715	5+:130647717-130690211
4984	Snap29	67474	16+:17406095-17428573
4985	RP23-36P22.5	100043899	2+:163318052-163325664
4986	Atg3	67841	16+:45158941-45188651
4987	Olfr1218	258815	2-:88894647-88895581
4988	2400001E08Rik	66508	7+:109054442-109060398
4989	4930504B08Rik	75057	9-:74909101-74946994
4990	Mmp27	234911	9+:7571944-7581415
4991	MMT00004329		17+:22871900-22872343
4992	MMT00070169		7+:30870365-30871036
4993	Tsc22d1	21807	14-:76906439-76907570
4994	Olfr1264	258206	2-:89861295-89862221
4995	Plxna4	243743	6-:32446712-32467376
4996	Echdc3	67856	2-:6109865-6134021

4997	Trim24	21848	6+:37820630-37916287
4998	2310002B06Rik	53951	17+:79234797-79244701
4999	BC022960	237246	X+:165806120-165807944
5000	Tmprss11f	243083	5-:86953136-86962861
5001	MMT00048925		16+:17734259-17735348
5002	Ifi202b	26388	1-:175892745-175912845
5003	2410081M15Rik	73680	4-:129030875-129055272
5004	MMT00073757		12+:57290478-57291884
5005	Dnajc15	66148	14-:78226021-78274712
5006	Enoph1	67870	5+:100469032-100497781
5007	Cdkn3	72391	14+:47380253-47387387
5008	Ehd1	13660	19+:6276900-6298898
5009	MMT00054393		1+:66998402-66999410
5010	Gnpda2	67980	5-:69964348-69982030
5011	A930016P21Rik	68073	15+:31531872-31547290
5012	Fbxl13	320118	5-:21086998-21109530
5013	Fancl	67030	11+:26287201-26371876
5014	MMT00017503		1+:7654291-7655057
5015	MMT00027075		
5016	Mfap4	76293	11+:61298948-61302206
5017	Plat	18791	8+:23868239-23893316
5018	Phf21a	192285	2+:92024494-92201050
5019	Rnf44	105239	13-:54780772-54795197
5020	MMT00037480		
5021	Gdap2	14547	3+:99966440-100010904
5022	Wwtr1	97064	3-:57262381-57379733
5023	Spast	50850	17+:74751284-74790540
5024	MMT00020852		10+:128052418-128053090
5025	Atad5	237877	11+:79902903-79937633
5026	MMT00042079		1-:97152539-97153387
5027	Pde4b	18578	4+:101927607-102278428
5028	Spata20	217116	11-:94340220-94341759
5029	1110034G24Rik	73747	2+:132512673-132576943
5030	Hirip3	233876	7+:134005965-134008887
5031	Adipoq	11450	16+:23155297-23157666
5032	Gm872	380653	10-:92238409-92278681
5033	MMT00012520		X-:129422743-129423698
5034	EG621976	621976	13+:41701584-41730291
5035	Ssrp1	20833	2+:84877620-84887267
5036	C030003D03Rik	77220	10-:25712699-25798168
5037	MMT00044669		11+:96731662-96732158
5038	Kif9	16578	9+:110379528-110427678
5039	Hs3st2	195646	7+:128536243-128644549
5040	Fktn	246179	4+:53726869-53775827
5041	Wdr82	77305	9+:106073266-106091506
5042	Tada1l	27878	1+:168309495-168323749
5043	Cxcl13	55985	5+:96385957-96390086
5044	Ifi35	70110	11+:101309794-101319776
5045	MMT00017230		8+:36832015-36859436
5046	Pthlh	19227	6-:147200621-147212723
5047	Rpl5	19983	5+:108330887-108337013

5048	Mgll	23945	6+:88674477-88778351
5049	Emid1	140703	11-:5006268-5052225
5050	Gnal	14680	18+:67248099-67382383
5051	Angpt1	11600	15-:42258288-42508313
5052	Lrrn3	16981	12-:42178781-42216157
5053	BC002199	211556	3-:127510184-127511782
5054	Zfp821	75871	8+:112230251-112248831
5055	Gss	14854	2-:155388918-155418442
5056	Tmed8	382620	12-:88512768-88522422
5057	Prlh	623503	1+:92849684-92850588
5058	Pemt	18618	11-:59784320-59845307
5059	Abhd3	106861	18-:10644410-10706694
5060	MMT00066059		4+:149583796-149602550
5061	Myo9a	270163	9+:59598979-59773196
5062	Dnase1l2	66705	17-:24577509-24580029
5063	MMT00046201		2-:78353035-78353496
5064	Tekt2	24084	4-:125999366-126002443
5065	MMT00069141		14+:85878799-85879352
5066	4833439L19Rik	97820	13-:54652585-54666701
5067	Alkbh8	67667	9+:3335471-3387046
5068	Afap1l2	226250	19-:56986849-57082667
5069	Cdkn2aipnl	52626	11+:51781162-51789731
5070	2610024E20Rik	225358	18-:34602005-34666477
5071	Aff2	14266	X+:66613505-67121211
5072	MMT00052662		15+:48720103-48721563
5073	Chst8	68947	7-:35459659-35597474
5074	Tgm4	331046	9+:122954102-122975991
5075	Saal1	78935	7-:53943182-53966032
5076	Tmem57	66146	4-:134358671-134408862
5077	Prrg1	546336	X-:75698026-75794754
5078	1700020N15Rik	75509	X+:67198474-67199153
5079	Bet1l	54399	7-:148040354-148042138
5080	Pcdh24	268663	13+:54818970-54838029
5081	Onecut1	15379	9+:74709934-74737457
5082	Ibsp	15891	5+:104728315-104740484
5083	Gprc5b	64297	7-:126115043-126128159
5084	Gsn	227753	2+:35137961-35163414
5085	Prdx5	54683	19-:6981308-6984135
5086	Ccnh	66671	13+:85329041-85352512
5087	Ush1g	16470	11-:115179295-115183170
5088	EG229571	229571	3+:92877589-92878554
5089	Dlx5	13395	6-:6827804-6831910
5090	Eda	13607	X+:97170944-97596098
5091	C85492	215494	9-:121891088-122014787
5092	Ydjc	69101	16+:17144711-17148938
5093	Apol10b	328561	15-:77415401-77422580
5094	Yes1	22612	5+:32913605-32989429
5095	Jak1	16451	4-:100825650-100937838
5096	Tecta	21683	9-:42137705-42208012
5097	Srgap3	259302	6-:112672782-112896490
5098	1700029J11Rik	76426	2+:172298044-172299791

5099	MMT00056385		
5100	Pebp1	23980	5-:117732662-117737555
5101	Gm101	226356	1-:121819919-121832748
5102	Cmtm5	67272	14+:55555306-55558114
5103	EG237361	237361	10+:60772577-60773022
5104	1700094C09Rik	78634	8+:12573048-12600738
5105	MMT00008146		7-:88292143-88292283
5106	Usp38	74841	8-:83511469-83539565
5107	Gprc5c	70355	11+:114713368-114730409
5108	2200002D01Rik	72275	7-:30032521-30033492
5109	Cyp3a11	13112	5-:146666541-146691426
5110	1700001O22Rik	73598	2-:30650288-30659177
5111	Olfr380	259027	11-:73266776-73267712
5112	MMT00071941		1-:131693185-131694546
5113	Nxnl1	234404	8-:74084454-74090500
5114	Fbxo33	70611	12-:60301643-60320437
5115	Usp19	71472	9+:108396960-108404028
5116	Otop3	69602	11+:115196091-115201288
5117	Krt83	406219	15-:101261484-101321734
5118	Hmcn1	545370	1-:152409635-152707507
5119	MMT00065554		18-:63703066-63704860
5120	Alkbh7	66400	17+:57136775-57138745
5121	Tmem93	66048	11-:72989034-72990539
5122	Olfr525	258958	7+:147508720-147509528
5123	Zfp191	59057	18-:24172660-24176588
5124	Olfr424	258716	1+:176066876-176067822
5125	Fmod	14264	1+:135934119-135944736
5126	Fcnb	14134	2-:27931897-27940377
5127	Kif20b	240641	19+:34996848-35050241
5128	Rfxap	170767	3-:54608116-54611693
5129	Olfr746	258295	14+:51272913-51273856
5130	Phip	83946	9-:82787637-82869015
5131	Taar1	111174	10+:23640211-23641208
5132	1600016N20Rik	72000	7-:148395760-148396896
5133	6430517E21Rik	240843	1-:160175400-160225908
5134	1190002A17Rik	68870	2+:28555681-28668684
5135	Pisd	320951	5+:33069248-33079766
5136	MMT00023802		1-:6427687-6431345
5137	Cdh18	320865	15+:23296607-23340575
5138	Fau	14109	19+:6057948-6059520
5139	Zfp101	22643	17-:33517637-33531544
5140	Cbln3	56410	14-:56500897-56503069
5141	9330101J02Rik	664857	7-:146828734-146832352
5142	Eppk1	223650	15-:75949949-75950994
5143	Ccdc13	434446	9-:121706746-121723464
5144	MMT00058978		2+:66780612-66781514
5145	Scn3b	235281	9+:40077125-40096767
5146	2810021B07Rik	66308	13+:17787252-17789168
5147	5730455P16Rik	70591	11-:80173994-80191506
5148	MMT00050920		11+:54760936-54761388
5149	Sirt3	64384	7-:148049572-148068208

5150	Syf2	68592	4+:134486894-134493452
5151	Akt2	11652	7+:28376774-28423221
5152	Cdh10	320873	15+:18748679-18943559
5153	MMT00072251		4-:101719173-101722109
5154	Pcid2	234069	8-:13077183-13105453
5155	Nwd1	319555	8+:75186045-75195006
5156	Srm	20810	4+:147965655-147968701
5157	Fgf20	80857	8-:41365111-41372061
5158	Rtn4	68585	11+:29618782-29642086
5159	MMT00020962		7-:80133985-80135027
5160	Cdh24	239096	14-:55250950-55258381
5161	Tbc1d1	57915	5+:64726514-64736671
5162	Uqcrb	67530	13-:67001556-67006286
5163	Slc5a2	246787	7+:135409200-135415713
5164	Hoxc12	15421	15+:102767283-102768948
5165	4933427I04Rik	664620	4+:123515473-123538243
5166	Lix1	66643	17+:17582921-17594587
5167	4930478P22Rik	74429	5+:35704187-35710325
5168	Atp1b3	11933	9-:96233098-96264718
5169	MMT00037010		
5170	MMT00012537		11+:90270092-90381426
5171	Adamtsl5	66548	10-:79805483-79811157
5172	Sumo1	22218	1-:59696287-59727653
5173	Azgp1	12007	5+:138422787-138431458
5174	Slc5a7	63993	17-:54415843-54436461
5175	MMT00032221		
5176	MMT00004357		14+:69350007-69351097
5177	Agtrap	11610	4-:147454592-147462052
5178	Wnt2b	22414	3-:104749673-104764504
5179	Gdpd4	233537	7+:105122890-105152908
5180	E130304I02Rik	78547	7+:36587185-36623093
5181	4930481B07Rik	74937	3+:94819035-94820196
5182	Pcx	18563	19+:4594357-4621697
5183	St5	76954	7-:116667426-116760080
5184	Tac4	93670	11+:95122842-95130573
5185	Dus2l	66369	8+:108535394-108577739
5186	MMT00062958		15-:36559277-36559671
5187	Speer4f	70935	5+:16981939-16986754
5188	Cma1	17228	14-:56560289-56563498
5189	Capns2	69543	8+:95425383-95426126
5190	Phkb	102093	8+:88459294-88584881
5191	MMT00076927	236069	4+:145045535-145427380
5192	MMT00038487		7-:66092018-66093401
5193	Tmem32	236792	X-:53838689-53851111
5194	Nsmce2	68501	15+:59205801-59433236
5195	Prx	19153	7+:28284342-28305233
5196	Zfp97	22759	17+:17258378-17403111
5197	6430710C18Rik	76218	2-:72583901-72651773
5198	1700029N11Rik	69491	13+:44535041-44550695
5199	Ppp2r5d	21770	17-:46819940-46821655
5200	Tm6sf1	107769	7+:89004016-89028952

5201	MMT00076398		11+:105634739-105636097
5202	Bcl11a	14025	11+:23978116-24073193
5203	MMT00001924		6+:39812256-39841710
5204	Lifr	16880	15+:7090540-7141218
5205	MMT00065634		10+:62137891-62165263
5206	Igk-V	16080	6+:68469005-68469494
5207	1700123K08Rik	76658	5-:139003067-139005922
5208	Plod2	26432	9+:92437289-92503258
5209	Hcfc2	67933	10+:82161883-82204138
5210	MMT00036454		10-:15240325-15241131
5211	Fubp3	320267	2+:31428259-31473015
5212	Mos	17451	4-:3797930-3799104
5213	Rhoh	74734	5+:66254217-66284779
5214	Zadh1	77219	12+:85626355-85655031
5215	Col6a1	12833	10-:76171536-76188789
5216	Spnb5	640524	2-:119872115-119911478
5217	Lpcat2	270084	8+:95379247-95414730
5218	ENSMUSG00000(	194612	17+:22825894-22884251
5219	Fbxo22	71999	9+:55056765-55073204
5220	MMT00037083		12+:55817773-55818252
5221	Als2cr4	381259	1-:59157437-59177240
5222	B630005N14Rik	101148	6-:13575937-13627833
5223	Rcan2	53901	17+:44090353-44174101
5224	MMT00071383		10-:68154349-68154847
5225	Maf1	68877	15+:76182432-76184802
5226	Ovgp1	12659	3+:105776720-105790340
5227	2310008M10Rik	66357	3-:130399068-130412335
5228	Actr3b	242894	5+:25265902-25355764
5229	Katna1	23924	10+:7445819-7482948
5230	MMT00077995		6-:117423016-117423443
5231	Dus3l	224907	17+:56904200-56909263
5232	Arhgef16	230972	4-:153652595-153662123
5233	Pcbd1	13180	10+:60552108-60557067
5234	Lhx8	16875	3-:153969261-153993364
5235	Anxa6	11749	11-:54792613-54835083
5236	Cldn4	12740	5-:135420996-135422776
5237	Cpa1	109697	6+:30592297-30595360
5238	Il12b	16160	11+:44213564-44227512
5239	3110009E18Rik	73103	1+:122017761-122084759
5240	Atp6v1c1	66335	15+:38591708-38621638
5241	Dlk2	106565	17+:46439666-46440218
5242	Gata3	14462	2-:9779481-9799424
5243	Slc25a26	67582	6+:94450324-94554141
5244	MMT00022648		X+:75636043-75636562
5245	Cacna1h	58226	17-:25511294-25570113
5246	MMT00039086		13-:99743252-99743775
5247	Irak2	108960	6+:113588493-113645003
5248	Tjp1	21872	7-:72441053-72516125
5249	Olfr615	259084	7+:110708992-110709933
5250	Ifit2	15958	19+:34644804-34651013
5251	A630089N07Rik	320586	16-:98286709-98303473

5252	Mfsd3	69572	15+:76531894-76533998
5253	Fastkd3	69577	13+:68721122-68731155
5254	MMT00015794		10-:41332575-41334698
5255	Zkscan16	100041581	4+:58959022-58970725
5256	Tnfrsf13c	72049	15-:82052173-82054764
5257	Zfp236	329002	18-:82768338-82850148
5258	Mcts2	66405	2+:152512883-152513682
5259	Mat1a	11720	14+:41918669-41937699
5260	Dnalc1	105000	12+:85455318-85479814
5261	Gm75	219026	14+:51734137-51736753
5262	Mecp2	17257	X-:71272168-71330932
5263	Anapc13	69010	9+:102532119-102536468
5264	BC042720	329178	1+:66647628-66742170
5265	A3galt2	215493	4+:128438662-128444916
5266	Gh	14599	11-:106161614-106163124
5267	4933439F18Rik	66771	11+:60230646-60264429
5268	MMT00017380		19-:61042140-61043315
5269	MMT00068783		X+:99403740-99404795
5270	Rnf8	58230	17+:29751806-29778603
5271	Npy	109648	6+:49773596-49779502
5272	Grk4	14772	5+:35003601-35097952
5273	Vmn2r89	22301	14+:52071636-52080967
5274	MMT00065196		2-:134587804-134589386
5275	Pcsk2	18549	2+:143372142-143639765
5276	Coro1b	23789	19+:4148662-4154034
5277	5033414K04Rik	98496	1-:84034606-84065875
5278	2700078K21Rik	101513	7-:149194457-149246939
5279	MMT00079802		17+:36295759-36318102
5280	Htr1f	15557	16-:64925523-65105610
5281	Gpr107	277463	2+:31007845-31072047
5282	1700027A15Rik	69449	1+:73031411-73072072
5283	B3galt1	26877	2+:67955999-67956980
5284	Npffr1	237362	10+:61076703-61089132
5285	Tmc8	217356	11+:117643432-117645506
5286	Thoc6	386612	17-:23805579-23812190
5287	MMT00002272		X-:12521523-12522092
5288	Galnt4	14426	10+:98571048-98572785
5289	Tln1	21894	4-:43544390-43569622
5290	1110028C15Rik	68691	1-:66767384-66848713
5291	Chl1	12661	6+:103460996-103679821
5292	Adam4	11498	12-:82500780-82522832
5293	Zdhhc8	27801	16-:18222319-18235103
5294	C730048C13Rik	319800	19-:8409849-8479572
5295	F2r	14062	13-:96371743-96388388
5296	Rnf181	66510	6-:72309714-72312387
5297	MMT00079371		3-:144288366-144288703
5298	Sbno1	243272	5-:124842977-124875577
5299	Emilin1	100952	5+:31216158-31223639
5300	1300001I01Rik	74148	11+:74462376-74484348
5301	Cd38	12494	5+:44260111-44302281
5302	Exosc4	109075	15+:76157857-76161107

5303	Ndufv2	72900	17-:66428550-66450866
5304	Pdzd2	68070	15-:12291652-12388060
5305	A730076H11Rik	211027	14+:53627284-53992817
5306	Ywhag	22628	5-:136386681-136410446
5307	Fkbp8	14232	8+:73053425-73059221
5308	MMT00008486		6-:50150172-50152566
5309	D19Wsu162e	226178	19+:46673609-46730236
5310	Ppie	56031	4-:122804621-122817175
5311	OTTMUSG00000(	100042922	X+:10876349-10876876
5312	Ccdc91	67015	6+:147424392-147581126
5313	9630058J23Rik	226744	1+:181536593-181557605
5314	Dnajc2	22791	5-:21263094-21290983
5315	Slc5a4b	64454	10-:75521430-75573763
5316	Yipf7	75581	5-:69908276-69932401
5317	MMT00056638		2-:100466613-100468400
5318	Fbxo45	268882	16-:32230195-32247161
5319	Hydin	244653	8+:113110933-113134145
5320	MMT00007643		8+:23517938-23518974
5321	Syngr4	58867	7-:53142209-53152004
5322	Slpi	20568	2-:164179806-164198446
5323	Hsdl1	72552	8-:122085878-122099045
5324	Tsga14	83922	6-:30604391-30643712
5325	C230097I24Rik	328918	18-:24122539-24130290
5326	Frmd6	319710	12+:71965030-72000654
5327	Senp6	215351	9+:79984179-79991975
5328	MMT00020895		
5329	Hpd	15445	5-:123620928-123632734
5330	Cbfa2t3	12398	8-:125153890-125222877
5331	Cdkn2b	12579	4-:88952963-88956706
5332	Pdlim4	30794	11-:53868433-53882470
5333	Ttc26	264134	6+:38331473-38377171
5334	MMT00021505		13-:109098648-109100938
5335	Olfr211	258914	6+:116443628-116444556
5336	Timeless	21853	10+:127675120-127689988
5337	Cyp24a1	13081	2-:170309826-170322576
5338	MMT00031733	100045011	15-:84176768-84177267
5339	4930488E11Rik	399591	X-:133491272-133511389
5340	MMT00048531		X+:144181262-144199155
5341	Gfra4	14588	2-:130865911-130868418
5342	Eps8l3	99662	3+:107680154-107695817
5343	Dqx1	93838	6+:83008483-83016834
5344	MMT00019216		4-:129288154-129291525
5345	MMT00023326	57435	17-:56240016-56249226
5346	Hbb-bh1	15132	7-:110990267-110991624
5347	Zfp371	170734	7+:6190171-6191018
5348	Olfr9	18373	10+:128426969-128427907
5349	Psd3	234353	8-:70220794-70484678
5350	Lipt1	623661	1+:37929071-37933063
5351	Dnahc3	381917	7-:127066352-127202904
5352	Pdcd2l	68079	7-:34969515-34981692
5353	MMT00047295		18-:21651271-21652423

5354	Upk1a	109637	7-:31388099-31397755
5355	Gpsm2	76123	3-:108482007-108525087
5356	D15Wsu169e	223666	15-:76554282-76596543
5357	MMT00021917		2-:174979623-174979793
5358	Arc	11838	15-:74499513-74502982
5359	Psmd13	23997	7+:148068331-148084287
5360	Cep76	225659	18-:67777455-67800854
5361	Gpr88	64378	3-:115952573-115956372
5362	Nans	94181	4+:46502153-46515545
5363	Phf14	75725	6+:11875880-11958012
5364	Kif22	110033	7-:134171245-134185934
5365	C87436	232196	6+:86388681-86420379
5366	MMT00048276	544905	12-:115378879-115538476
5367	Zfp629	320683	7-:134750545-134758917
5368	Slc12a4	20498	8-:108467492-108489939
5369	Rag1	19373	2-:101478438-101489658
5370	Crh	12918	3-:19593437-19595485
5371	MMT00073868		3-:37858514-37858925
5372	Tor1a	30931	2-:30816147-30823404
5373	Ccr1l1	12770	9-:123892172-123893241
5374	Chka	12660	19+:3851913-3894367
5375	Sec23b	27054	2+:144384956-144416109
5376	Ncf1	17969	5-:134697620-134705474
5377	MMT00077700		X+:161986982-162118532
5378	Tex19.1	73679	11+:121007470-121009632
5379	Grina	66168	15+:76077252-76080334
5380	Olfr1403	258645	1+:175200515-175201240
5381	Scube1	64706	15-:83435438-83555336
5382	Fbn1	14118	2-:125126331-125332121
5383	Usmg2	83677	17+:88932377-88932547
5384	A530064D06Rik	328830	17-:48282346-48303639
5385	Tubb2b	73710	13-:34218876-34222223
5386	Ccnl1	56706	3-:65750075-65762103
5387	Olfr170	258959	16-:19605818-19606759
5388	MMT00054957		11-:101985403-101990378
5389	Abce1	24015	8-:82207543-82235514
5390	Otub1	107260	19-:7272695-7280774
5391	Gdf2	12165	14+:34754223-34760383
5392	RP23-268N22.9	546519	11+:117722065-117725672
5393	Olfr1095	258725	2-:86690928-86691853
5394	MMT00015556		5+:10116908-10164483
5395	Dhx9	13211	1-:155302893-155334792
5396	4933425O20Rik	66766	2+:130232267-130232932
5397	Ubl5	66177	9+:20449247-20451233
5398	1700030F18Rik	72277	15-:99758129-99758310
5399	Usp44	327799	10+:93308434-93320831
5400	2610318N02Rik	70458	16-:17113491-17125223
5401	Nlrx1	270151	9-:44061274-44073930
5402	MMT00076103		19+:3527155-3575893
5403	Ccdc25	67179	14+:66456212-66485044
5404	Plekhg1	213783	10-:6379147-6473615

5405	Apbb2	11787	5-:66693061-66756362
5406	Cd207	246278	6-:83621677-83627793
5407	Chd2	244059	7-:80702660-80703136
5408	Habp4	56541	13+:64263227-64287842
5409	MMT00046838		5-:100649459-100784138
5410	Cyp4f15	106648	17+:32822603-32840294
5411	Pank2	74450	2+:131088184-131124862
5412	MMT00020442	544737	10+:112365516-112365796
5413	Flot1	14251	17+:35960301-35969732
5414	Etnk2	214253	1+:135260359-135276200
5415	Ube2t	67196	1+:136859153-136870714
5416	Jhdm1d	338523	6-:39086652-39088048
5417	Ssb	20823	2+:69699689-69709856
5418	Cdh12	215654	15+:20614288-21288936
5419	ENSMUSG00000(	629141	7+:54238978-54243165
5420	Mbnl1	56758	3+:60333070-60433302
5421	Arhgef9	236915	X-:92245446-92391839
5422	4933422A05Rik	71133	9+:35090119-35093008
5423	1110005A23Rik	66118	10+:128258974-128314673
5424	Scpep1	74617	11-:88785576-88816756
5425	Ung	22256	5+:114581208-114589330
5426	Olfm2	244723	9-:20472142-20550683
5427	Olfr1301	258889	2+:111594407-111595344
5428	Col12a1	12816	9-:79449565-79566272
5429	2810417H13Rik	68026	9+:65738169-65751073
5430	Ifna4	15967	4+:88487764-88488324
5431	Fkbp6	94244	5-:135767574-135825861
5432	EG245297	245297	12+:17999921-18072431
5433	2410004L22Rik	76478	8-:73775023-73796275
5434	Inpp5b	16330	4+:124428773-124478680
5435	Lins2	72635	7+:73834840-73859630
5436	Prl7a1	19113	13-:27725247-27734343
5437	MMT00013238		14-:70264425-70264583
5438	Slc25a15	18408	8-:23487758-23508932
5439	Fuca2	66848	10+:13220892-13235422
5440	1810020G14Rik	66272	12-:82572811-82586069
5441	Csdc2	105859	15+:81767370-81781370
5442	Siglecg	243958	7+:50663662-50673717
5443	Nap1l2	17954	X-:100379514-100381979
5444	Stat4	20849	1+:52065089-52164027
5445	1700003E16Rik	71837	6+:83106456-83112936
5446	Atg4a	666468	X+:137491356-137530185
5447	Wdr17	244484	8-:55753703-55759051
5448	Olfr70	56014	4-:43709094-43710043
5449	Got1	14718	19-:43574244-43598999
5450	Nrbp1	192292	5+:31543290-31553933
5451	MMT00073305		4+:52834325-52834950
5452	Mogat2	233549	7-:106367595-106387078
5453	Odf3l1	382075	9-:56696466-56699583
5454	MMT00059595		16+:20663826-20665211
5455	Kcnab3	16499	11+:69143825-69145645

5456	Rabgap1l	29809	1-:162153666-162187675
5457	BC052688	432812	13+:61758492-61759033
5458	Nde1	67203	16+:14169567-14192659
5459	2510049I19Rik	67922	8+:74743643-74747329
5460	Tnrc6c	217351	11+:117575439-117622165
5461	Rasgrp4	233046	7+:29919951-29936634
5462	Pramel5	384077	4-:143860532-143870359
5463	Krt14	16664	11-:100064477-100068824
5464	Tk2	57813	8-:106750600-106772436
5465	Slamf8	74748	1-:174511890-174520620
5466	2310047D13Rik	116972	11+:76014676-76022918
5467	Plxdc1	72324	11-:97785291-97847758
5468	MMT00053630		10+:40268705-40269545
5469	Rnf41	67588	10+:127852245-127876834
5470	Cacna2d2	56808	9+:107302399-107429794
5471	Plxdc2	67448	2+:16278731-16673311
5472	Mppe1	225651	18-:67384702-67405479
5473	Ggps1	14593	13-:14145945-14150160
5474	Zswim1	71971	2+:164648512-164652367
5475	Akr1c18	105349	13-:4131872-4149877
5476	Megf9	230316	4-:70092832-70196035
5477	2210412D01Rik	70178	7-:91257869-91300403
5478	Scn1b	20266	7-:31901707-31911743
5479	Ap3d1	11776	10-:80169723-80204956
5480	Hydin	244653	8+:113095637-113098959
5481	Tmem162	76415	7+:31758843-31774739
5482	Cdsn	386463	17+:35689120-35693858
5483	Gpc5	103978	14+:115768753-115798511
5484	2400003C14Rik	71955	8-:112195228-112217164
5485	Rnmtl1	67390	11+:76057257-76064123
5486	Wbscr28	76629	5-:135378446-135382606
5487	Palmd	114301	3-:116621185-116671667
5488	C920009B18Rik	606736	10+:22026526-22034002
5489	Nek3	23954	8-:23238754-23276872
5490	MMT00071314		1+:72134389-72135081
5491	MMT00037875		11+:3528832-3529639
5492	MMT00039914		13+:4247980-4249023
5493	Dnajc4	57431	19-:7062400-7066762
5494	Gpr101	245424	X-:54753277-54754965
5495	L3mbtl3	237339	10-:25994276-26072166
5496	EG434025	434025	6+:67999975-68033138
5497	Il17f	257630	1-:20767848-20769593
5498	Trpc1	22063	9-:95607049-95650777
5499	MMT00075646	666661	15+:77449676-77457066
5500	4921513D23Rik	223989	16-:14111447-14163682
5501	Dppa2	73703	16+:48310619-48318824
5502	Tmem107	66910	11+:68884312-68886794
5503	Mcrs1	51812	15-:99073288-99082342
5504	Itsn1	16443	16+:91782437-91912254
5505	Thap2	66816	10-:114808894-114821470
5506	D17Wsu92e	224647	17-:27888201-27957304

5507	Olfr16	18313	1+:174886927-174887855
5508	Lrig2	269473	3-:104261050-104315822
5509	Kcnh4	380728	11-:100605437-100620919
5510	Gm1967	381411	2-:93701168-93709235
5511	BC055004	381680	5+:138680464-138693315
5512	Crtc3	70461	7-:87733090-87822341
5513	EG639869	639869	3+:64251881-64264060
5514	MMT00044522	435784	4-:53802397-53804328
5515	Apobec3	80287	15+:79741603-79747835
5516	V1rc14	171187	6-:66561646-66562562
5517	Stx17	67727	4+:48137801-48195989
5518	Jmjd1c	108829	10+:66590005-66719072
5519	1700067K01Rik	73453	8+:86525432-86528667
5520	Gltpd2	216871	11+:70332710-70334237
5521	Perld1	320655	11-:98251110-98261750
5522	Pink1	68943	4-:137869319-137882193
5523	Taok1	216965	11-:77343564-77344872
5524	Klhl7	52323	5+:23606429-23666603
5525	Prkca	18750	11-:107799561-108205242
5526	Ush1c	72088	7-:53450726-53493860
5527	Zfp398	272347	6+:47785665-47818255
5528	Rfx1	19724	8+:86590827-86619942
5529	Setbp1	240427	18-:78952276-79120781
5530	MMT00027303		16+:21896562-21897207
5531	Ptk6	20459	2-:180929828-180937494
5532	Fezf2	54713	14-:13174615-13178342
5533	Baalc	118452	15+:38765454-38780774
5534	2310036O22Rik	68544	8+:87550770-87554184
5535	Olfr446	258292	6+:42877231-42878157
5536	5830415F09Rik	74753	4-:46389379-46402540
5537	Kcnt2	240776	1+:142247784-142506288
5538	Slc7a3	11989	X-:98274560-98280691
5539	Eif2s2	67204	2-:154697153-154718518
5540	Abp1	76507	6+:48845252-48859186
5541	Bcap29	12033	12-:32280260-32319245
5542	Il27	246779	7-:133732808-133738424
5543	Phlpp	98432	1+:108177939-108290821
5544	Camsap1	227634	2-:25782493-25838494
5545	Shroom3	27428	5+:93112460-93393977
5546	Slc5a12	241612	2+:110481878-110488763
5547	Cd3eap	70333	7-:19942219-19944768
5548	Fgf6	14177	6+:126965603-126977447
5549	Zfp217	228913	2-:169935942-169945905
5550	MMT00047096		2-:105377095-105377639
5551	Pim1	18712	17+:29628088-29631692
5552	Rasgrp3	240168	17+:75895692-75927270
5553	MMT00013152		12-:72369920-72371344
5554	Emr1	13733	17+:57498112-57622948
5555	Fbln7	70370	2+:128703086-128722768
5556	Ccl4	20303	11+:83476088-83478182
5557	Tpx2	72119	2+:152673718-152719973

5558	V1ra9	113851	6+:90219099-90220045
5559	Pftk1	18647	5-:4803733-5380197
5560	Sh2b2	23921	5-:136694578-136720741
5561	MMT00054702	100040158	1+:84960243-84961132
5562	Arhgdia	192662	11-:120439418-120442562
5563	Slco1a4	28250	6-:141753960-141804692
5564	Tmprss11e	243084	5-:87136305-87174863
5565	MMT00051106	546543	12+:89460808-89552820
5566	2610002J02Rik	67513	4+:154623908-154630799
5567	Mapk8ip1	19099	2-:92224326-92241310
5568	EG627866	627866	1+:53447249-53449740
5569	Pdgfc	54635	3+:80841161-81017954
5570	MMT00025545	433776	4-:142831926-142833276
5571	MMT00040490		6-:70384922-70385482
5572	Opalin	226115	19-:41134027-41151655
5573	MMT00043445		
5574	Hsd3b7	101502	7+:134944182-134947312
5575	Pign	27392	1-:107417718-107560239
5576	Polr2e	66420	10-:79498695-79502410
5577	MMT00009628		11+:117736358-117825335
5578	Phactr1	218194	13+:43051984-43230728
5579	MMT00004201		4-:40061626-40064353
5580	4933407P14Rik	237958	11+:104047835-104049761
5581	MMT00000722		17-:19559420-19581898
5582	EG433387	433387	1+:186441126-186441607
5583	Igh	111507	12-:115008053-115008508
5584	MMT00007678		2+:118571806-118572308
5585	Map3k14	53859	11-:103082338-103103684
5586	OTTMUSG00000(	433719	4-:72512345-73084353
5587	Rmnd5b	66089	11-:51436993-51449499
5588	Snx20	71607	8-:91150726-91160027
5589	D930028F11Rik	244853	9+:48200799-48207153
5590	Centd2	69710	7+:108496655-108560407
5591	Igsf8	140559	1+:174242069-174249971
5592	Runx3	12399	4+:134708410-134732096
5593	Fmn2	54418	1+:176431976-176752198
5594	Erbb3	13867	10-:128004583-128026677
5595	Kifap3	16579	1+:165709809-165847230
5596	Wnt1	22408	15+:98621280-98624269
5597	Ttll4	67534	1+:74725565-74744545
5598	Trp73	22062	4-:153430356-153516627
5599	Tbc1d10b	68449	7-:134341309-134351337
5600	Zfyve26	211978	12-:80388780-80397266
5601	4933417O08Rik	71146	19+:42322061-42343631
5602	3222402P14Rik	235542	9-:101004710-101115476
5603	BC020535	228788	2+:152891690-152907334
5604	Ptprb	19263	10+:115712576-115820990
5605	Nxph1	18231	6+:9185910-9197845
5606	Atg7	74244	6+:114593071-114812050
5607	Ick	56542	9+:77961215-78015745
5608	Ntrk2	18212	13+:58910089-59034481

5609	Gtf2a2	235459	9+:69860390-69870268
5610	Syngap1	240057	17+:27081577-27107590
5611	Rapgef3	223864	15-:97575386-97598097
5612	Cdv3	321022	9-:103255487-103267828
5613	Mrpl12	56282	11+:120345982-120350067
5614	Cope	59042	8+:72826683-72836889
5615	Sertad2	58172	11+:20443336-20549114
5616	Tmod2	50876	9-:75421721-75459073
5617	Irs4	16370	X-:138156091-138159741
5618	Phxr4	18689	9+:13234580-13235956
5619	Rap1gds1	229877	3-:138588876-138738034
5620	2700038C09Rik	66496	2+:180922054-180922942
5621	Nudt9	74167	5+:104476015-104494386
5622	Zfp655	72611	5+:145992628-146006038
5623	Mc2r	17200	18-:68566983-68567874
5624	Olfr763	258861	10+:128448342-128449272
5625	MMT00035309		5-:102845385-102845885
5626	Kremen2	73016	17-:23878166-23882796
5627	D130051D11Rik	319737	15+:85456126-85476733
5628	MMT00035417	634100	12-:116067451-116067908
5629	Als2	74018	1-:59219775-59294061
5630	Fnta	14272	8-:27109439-27126067
5631	Cited1	12705	X-:99442796-99443961
5632	Reck	53614	4+:43950178-43957675
5633	MMT00006481	100044456	3-:93007274-93023729
5634	Creb1	12912	1+:64579467-64644851
5635	4930519L02Rik	75085	3+:142703460-142703714
5636	Trmu	72026	15+:85709756-85727820
5637	Slc17a8	216227	10-:89038954-89040773
5638	Otud4	73945	8+:82163291-82201646
5639	Gdpd3	68616	7+:133910686-133919157
5640	Gpx4	625249	10+:79516232-79519182
5641	MMT00024854		7-:109716505-109718267
5642	Crebzf	233490	7+:97592081-97593285
5643	Gas2l1	78926	11-:4960472-4964854
5644	Spsb2	14794	6+:124758958-124760632
5645	Tmem8	60455	17+:26250261-26260196
5646	Kifc1	16580	17+:27054159-27069227
5647	MMT00026884		9+:41459975-41463363
5648	Zfp213	449521	17-:23693735-23701168
5649	MMT00045864		16-:33138632-33140656
5650	Arhgap21	71435	2-:20801969-20889034
5651	Olfr541	258964	7+:147890151-147891088
5652	Adam26a	13525	8-:44653631-44662020
5653	BC050777	241850	2+:181196603-181229436
5654	Tln2	70549	9-:67068460-67159701
5655	OTTMUSG00000(	194225	4+:143436442-143443542
5656	4933434I20Rik	67555	8+:85872369-85903301
5657	Arpc1b	11867	5+:145883424-145887638
5658	Mycl1	16918	4+:122673765-122677756
5659	MMT00053254		12+:88055935-88056393

5660	Fat3	270120	9-:16179392-16182698
5661	Lrrc46	69297	11-:96895911-96902658
5662	Cyp27b1	13115	10+:126485307-126490024
5663	Rev3l	19714	10+:39452374-39595013
5664	Fez2	225020	17-:78777222-78817426
5665	Nsun2	28114	13+:69754381-69773211
5666	Vegfb	22340	19-:7056961-7062093
5667	Ranbp3l	223332	15+:8986847-8992882
5668	Tdp1	104884	12+:101147885-101193432
5669	Fndc1	68655	17-:7931447-7966670
5670	EG625253	625253	X+:153978628-153979376
5671	Pxmp2	19301	5-:110703579-110715024
5672	Galntl4	233733	7-:118615168-118759998
5673	1500001A10Rik	68955	5-:116894578-116932555
5674	MMT00073552		3-:121886794-121887878
5675	Slc5a9	230612	4-:111549856-111575348
5676	MMT00039774		4+:90823384-90823888
5677	Cmas	12764	6+:142705292-142724227
5678	Recql5	170472	11-:115753990-115794320
5679	MMT00002281		
5680	Lrg1	76905	17-:56259102-56261312
5681	Mbd1	17190	18+:74427957-74442339
5682	Kcnh7	170738	2-:62541001-62715414
5683	Arhgap1	228359	2+:91490378-91512475
5684	Ranbp10	74334	8-:108292209-108351095
5685	Gbx2	14472	1-:91824474-91827752
5686	Cys1	12879	12-:25350702-25366678
5687	Klf11	194655	12+:25336389-25347640
5688	Cacng3	54376	7+:129815287-129912359
5689	Dsg2	13511	18+:20716573-20763024
5690	Rbm15	229700	3-:107128544-107135866
5691	Csrp3	13009	7-:56085929-56103395
5692	2010005H15Rik	76770	16+:36253090-36257530
5693	Zfp809	235047	9+:22009228-22047796
5694	Sp7	170574	15-:102187040-102196764
5695	Prdm12	381359	2+:31495621-31509812
5696	Tarbp2	21357	15+:102349551-102354105
5697	Naca	17938	10+:127219576-127485561
5698	Nkain1	67149	4+:130125774-130168392
5699	MMT00042766		
5700	Tenc1	209039	15+:101933517-101946833
5701	Tpsg1	26945	17+:25507596-25511380
5702	Akap14	434756	X-:34689906-34704089
5703	4930451C15Rik	74685	16-:17548781-17554525
5704	MMT00077118		14+:43787610-43793723
5705	Bmpr1b	12167	3-:141503331-141832182
5706	Cyp1a2	13077	9-:57525035-57530336
5707	MMT00017539		4-:136165086-136165774
5708	C1ql1	23829	11-:102801047-102807775
5709	Hnrnpa3	229279	2+:75500034-75501775
5710	Centg2	347722	1+:91501172-91640473

5711	Ndufc1	66377	3-:51209400-51212877
5712	Kel	23925	6-:41636352-41654296
5713	1700009J07Rik	75188	10+:77356172-77356908
5714	Zmynd19	67187	2+:24805366-24814933
5715	ENSMUSG00000(	433237	19+:29141789-29143990
5716	Intu	380614	3+:40439714-40503718
5717	2310007B03Rik	71874	1-:95048160-95057448
5718	Tspan2	70747	3+:102538889-102575154
5719	11-Sep	52398	5+:93522464-93604665
5720	Olfr651	258809	7+:111701434-111702375
5721	Mlc1	170790	15-:88786314-88809437
5722	Manba	110173	3+:135148574-135234246
5723	Dtwd2	68857	18-:49857130-49915232
5724	BC039632	330657	7-:135028957-135039133
5725	2-Mar	224703	17-:33828474-33855582
5726	MMT00024129		2-:175439916-175442156
5727	Camk2g	12325	14-:21554097-21611844
5728	MMT00044077		18-:47853154-47895055
5729	2310016C08Rik	69573	6+:29222749-29225322
5730	Tasp1	75812	2-:139659214-139736380
5731	1810010D01Rik	381935	7-:152391729-152393991
5732	Sh3tc2	225608	18+:62137530-62150981
5733	B4galnt2	14422	11-:95727335-95776185
5734	EG619653	619653	8-:128999567-129000755
5735	Nkapl	66707	13-:21558967-21560356
5736	Nlrp4d	384752	7-:10957389-10974325
5737	Nkx2-2	18088	2-:147008887-147012095
5738	St8sia1	20449	6-:142777291-142912596
5739	Adsl	11564	15+:80778943-80799549
5740	Efemp2	58859	19+:5475020-5481852
5741	Zc3h10	103284	10-:127980622-127984800
5742	Olfr782	257985	10+:128787620-128788563
5743	L3mbtl	241764	2+:162773893-162800257
5744	Pxt1	69307	17-:29070930-29079210
5745	Cartpt	27220	13-:100668874-100670589
5746	Ndufs2	226646	1-:173164990-173171276
5747	Fbxl16	214931	17+:25953360-25956727
5748	Brsk2	75770	7+:149136256-149184917
5749	Ube2j2	140499	4+:155323115-155331473
5750	2610101N10Rik	67958	9-:95360816-95412304
5751	MMT00003389		19+:59162039-59163044
5752	Dffa	13347	4+:148478359-148492047
5753	4931440F15Rik	216622	11-:29723491-29725455
5754	MMT00062691		3-:6450592-6451588
5755	BC024978	414069	7+:27982290-27989319
5756	Snx19	102607	9+:30235293-30274729
5757	Arglu1	234023	8-:8666300-8690517
5758	Tpst1	22021	5+:130577459-130611598
5759	Trim23	81003	13+:104968876-104994498
5760	Prl	19109	13+:27149444-27157051
5761	Ralgps2	78255	1-:158738960-158837895

5762	Trpc3	22065	3-:36519494-36570525
5763	Tnni3k	435766	3-:154601714-154756153
5764	Ltb4dh	67103	4-:58979360-58999973
5765	Ifitm2	80876	7-:148140737-148141860
5766	5033414D02Rik	67759	19-:29423166-29436361
5767	Cpne6	12891	14+:56130196-56136267
5768	MMT00077590		18-:90480351-90481596
5769	MMT00050131	100040591	1-:89282859-89291281
5770	2810485I05Rik	72826	9+:13632418-13650981
5771	Ysk4	22625	1-:129713831-129714509
5772	Katnal2	71206	18-:77231938-77286035
5773	1700129C05Rik	67932	14-:59751876-59761730
5774	MMT00040053		X+:100702148-100702927
5775	Jam2	67374	16+:84774672-84823137
5776	Rpa1	68275	11-:75113850-75161806
5777	Dusp16	70686	6-:134665487-134742493
5778	Map4k5	399510	12-:70904745-70993996
5779	Olfr1311	258271	2-:111861072-111862009
5780	Col14a1	12818	15+:55139299-55352357
5781	Rad54l	19366	4-:115769559-115795741
5782	MMT00042046		
5783	4833413O15Rik	242286	4-:3989766-4004072
5784	Tmem48	72787	4+:107040645-107088959
5785	Fbxw10	213980	11+:62660409-62691099
5786	Lta	16992	17-:35340107-35342128
5787	Aff1	17355	5+:104183347-104280640
5788	Smpdl3b	100340	4-:132288880-132313086
5789	Pten	19211	19+:32831986-32894517
5790	Reep3	28193	10-:66474255-66559697
5791	Baiap2l1	66898	5-:145025397-145118981
5792	Tpd52l2	66314	2+:181231905-181250603
5793	Cpeb3	208922	19-:37098242-37249881
5794	Ict1	68572	11+:115265079-115272227
5795	MMT00057992		4+:41115654-41116684
5796	MMT00013344		
5797	MMT00034010		4-:40663118-40664024
5798	Tcfcp2l1	81879	1+:120524530-120581744
5799	Tlr13	279572	X+:103338613-103354972
5800	Mta3	116871	17+:84105548-84203938
5801	MMT00042142		6-:127133424-127174210
5802	Mad2l2	71890	4+:147514598-147519805
5803	Tet2	214133	3-:133151131-133177138
5804	Thrsp	21835	7-:104565560-104566013
5805	Aak1	269774	6+:86799634-86946961
5806	Prrx2	20204	2+:30700800-30736771
5807	Gif	14603	19+:11822136-11837937
5808	Myeov2	66915	1-:94533723-94538545
5809	MMT00030928		X-:79067525-79068260
5810	1700023F06Rik	69441	11-:103061375-103069850
5811	Col4a2	12827	8+:11312890-11449286
5812	Dkk1	13380	19-:30621323-30623891

5813	1700021K10Rik	66331	8-:60255021-60441361
5814	Nrbp2	223649	15-:75916024-75920443
5815	Ehf	13661	2-:103106667-103123917
5816	Pdss1	56075	2+:22757272-22795711
5817	Tbl3	213773	17-:24837590-24844551
5818	Papola	18789	12+:107037860-107075068
5819	Pou6f1	19009	15-:100408673-100416283
5820	MMT00041363		6+:112942114-112942610
5821	Pin4	69713	X+:99314860-99322900
5822	Car2	12349	3+:14886697-14900576
5823	6530418L21Rik	109050	3+:105507591-105521076
5824	V1rh4	171247	13+:22474406-22475508
5825	RP23-434B7.2	68280	4-:91857427-91858446
5826	1700112E06Rik	76633	14+:22838931-22846550
5827	6332401O19Rik	319832	6-:24901146-24906085
5828	MMT00048630		14+:10718704-10718927
5829	2810455K09Rik	72805	12+:112088515-112107287
5830	2410066E13Rik	68235	6+:54631739-54649251
5831	Sh2b3	16923	5-:122267730-122282573
5832	Fbxl19	233902	7+:134891763-134912995
5833	Kcnk7	16530	19+:5704366-5707100
5834	MMT00015240		11-:16543098-16543894
5835	Hrh1	15465	6+:114429753-114431218
5836	Ndufb9	66218	15+:58765357-58771305
5837	Alg8	381903	7+:104520046-104540670
5838	Magi1	14924	6-:93626348-93765821
5839	Olfr1045	259019	2-:86037961-86038907
5840	Tbc1d9b	76795	11+:49944902-49986279
5841	Atg12	67526	18-:46892080-46901211
5842	Cldn6	54419	17+:23818030-23818689
5843	Anks1b	77531	10+:89973340-90143666
5844	Kctd21	622320	7+:104495831-104504545
5845	Il18bp	16068	7-:109163590-109166669
5846	Plscr3	70310	11+:69660396-69664545
5847	Olfr803	258547	10-:129128168-129129095
5848	MMT00064917		15-:37096092-37102192
5849	2310031A18Rik	69627	8-:127264157-127275506
5850	MMT00051577		14+:46587419-46612844
5851	Kctd4	67516	14+:76362278-76363810
5852	2410019A14Rik	229937	3+:145239184-145267650
5853	Trim30	20128	7-:111557541-111613625
5854	Kif11	16551	19+:37450840-37496351
5855	A730020M07Rik	319738	3-:121340072-121349373
5856	Mfsd2	76574	4-:122623711-122638436
5857	Rassf1	56289	9+:107457089-107464591
5858	Itch	16396	2+:154959300-155052587
5859	Ssr4	20832	X+:71032400-71036161
5860	MMT00070014		13-:9595591-9596642
5861	Anxa3	11745	5+:97222433-97274987
5862	MMT00053780		9+:70700765-70701863
5863	Gm606	239789	16-:26960339-26968392

5864	C730036D15Rik	209186	4-:49392792-49396448
5865	H2-DMb2	15000	17+:34282360-34288040
5866	EG432502	432502	10+:110562915-110563781
5867	Stk4	58231	2+:163899900-163977621
5868	Zfp33b	69623	5+:105064924-105068106
5869	MMT00058347		14+:108147699-108148978
5870	Limd1	29806	9+:123387823-123430634
5871	Ndst3	83398	3-:123231726-123375393
5872	Ankle1	234396	8+:73929917-73934441
5873	2810471M01Rik	72818	11+:28581562-28593276
5874	Olfr808	258930	10+:129204553-129205491
5875	Cd86	12524	16-:36606620-36666079
5876	1600029O15Rik	442832	9+:58055194-58056247
5877	Stx8	55943	11+:67779986-68017347
5878	Iqgap2	544963	13-:96397129-96661872
5879	Lmnb1	16906	18+:56867526-56913076
5880	Ppme1	72590	7-:107475245-107503353
5881	Fzd4	14366	7+:96115428-96557763
5882	Tyr	22173	7-:94575914-94641921
5883	Olfr1087	258843	2-:86530190-86531130
5884	Gstm2	14863	3-:107784616-107789315
5885	Dnm1	13429	2-:32165970-32208815
5886	Kcnq2	16536	2-:180843148-180869882
5887	Dctn5	59288	7+:129276596-129292152
5888	Slco4c1	227394	1-:98715153-98768749
5889	Errfi1	74155	4+:150229199-150242988
5890	Zfx	22764	X-:91319970-91368239
5891	Loxhd1	240411	18+:77546488-77568068
5892	MMT00038457		13+:99383173-99384945
5893	MMT00023658		
5894	Wdr21	73828	12+:84867032-84882865
5895	Fcrl6	677296	1-:174528781-174529409
5896	Ccr8	12776	9+:120002924-120004024
5897	MMT00013714		4+:90692200-90781826
5898	MMT00034680		6+:122594283-122594787
5899	Cpa3	12873	3-:20115521-20142076
5900	Igsf21	230868	4-:139582764-139802286
5901	Ap1g2	11766	14-:55717692-55724965
5902	4930563P21Rik	75275	2+:113125896-113137343
5903	Gpr135	238252	12-:73170606-73171978
5904	Chst4	26887	8-:112553715-112554880
5905	Nos2	18126	11+:78734360-78773626
5906	Lmbr1l	74775	15-:98734350-98748461
5907	BC026439	210463	15-:57075348-57095248
5908	MMT00027552		13-:79092343-79094053
5909	Psma6	26443	12+:56499883-56519438
5910	1110014J01Rik	74778	15-:82946278-82953181
5911	Cyp4a12b	13118	4+:115084260-115111200
5912	Ccbe1	320924	18-:66220639-66244526
5913	1700007E06Rik	75459	15-:89220593-89240649
5914	Usp36	72344	11-:118120965-118151557

5915	Npc1l1	237636	11-:6111019-6129360
5916	Triml2	622117	8+:44244116-44269210
5917	Zfp608	269023	18-:55047736-55148182
5918	Ccl9	20308	11-:83388160-83392112
5919	B930041F14Rik	230991	4+:155042736-155069280
5920	Las1l	76130	X-:93147318-93152187
5921	MMT00026738		9-:72454194-72454753
5922	Hyal2	15587	9+:107471456-107475107
5923	Vav1	22324	17+:57418607-57467126
5924	Fras1	231470	5+:97074846-97086491
5925	Tmem59l	67937	8-:73007757-73011236
5926	MMT00011275		
5927	Rshl1	83434	7+:19640042-19659795
5928	Mizf	102423	9-:44105767-44113809
5929	Cpz	242939	5-:35844865-35868184
5930	Mst1r	19882	9+:107809219-107822712
5931	4930567K20Rik	78056	10+:10468460-10477965
5932	Arfgap2	77038	2+:91105462-91117087
5933	Ppp1r1b	19049	11+:98210051-98219106
5934	S100a10	20194	3+:93359085-93368565
5935	Brwd3	382236	X-:105936923-106020834
5936	Pfn1	18643	11-:70465350-70468033
5937	MMT00079008		
5938	Pfn2	18645	3-:57645822-57651410
5939	Morn3	74890	5-:123485797-123496983
5940	Olfr521	258353	7+:106915673-106916637
5941	Ttn	22138	2-:76673993-76674677
5942	2310016E02Rik	67695	5-:31208891-31210098
5943	Rp1l1	271209	14+:64649446-64652341
5944	Sp3	20687	2-:72774485-72819017
5945	MMT00056805		4+:115153572-115153848
5946	MMT00048659		
5947	BC008155	214917	17-:25927451-25929329
5948	MMT00019326		13-:114551590-114552329
5949	Zbp1	58203	2-:173032112-173044391
5950	EG545052	545052	14+:55660560-55661539
5951	Dmbx1	140477	4-:115590556-115596407
5952	Rhoj	80837	12+:76409412-76502442
5953	MMT00008178		5-:28186044-28186980
5954	Lcn13	227627	2+:25555592-25558846
5955	Six2	20472	17-:86083785-86087377
5956	Selp	20344	1+:166053832-166079873
5957	Tsen34	66078	7+:3646175-3652609
5958	Mei1	74369	15+:81900575-81902435
5959	Cdh3	12560	8+:109034779-109080047
5960	EG381065	381065	17+:20707334-20708162
5961	Pla2g4b	211429	2+:119853241-119858329
5962	Olfr1356	258305	10-:78309517-78310658
5963	2900046G09Rik	78408	16+:20693424-20703116
5964	Cxxc4	319478	3+:133903180-133903792
5965	Pbld	68371	10+:62524345-62539576

5966	Cacnb4	12298	2-:52289380-52413917
5967	Nup85	445007	11+:115425773-115430038
5968	MMT00012419		4+:140640935-140641534
5969	Rpn2	20014	2+:157109318-157151786
5970	MMT00008223		2-:89592028-89592983
5971	Ptms	69202	6-:124863703-124865616
5972	2700007P21Rik	212772	2-:106802692-106810599
5973	MMT00070628		14-:52249951-52254309
5974	Arntl	11865	7+:120351092-120457638
5975	Car13	71934	3+:14641776-14661804
5976	Col6a3	12835	1-:92663435-92740506
5977	BC061039	68222	2+:25074264-25077798
5978	MMT00016241		X-:128382520-128384996
5979	Lmo2	16909	2+:103810828-103821251
5980	Gabrb1	14400	5+:72091254-72528483
5981	Manea	242362	4-:26254799-26268038
5982	Rab3gap1	226407	1+:129765370-129840443
5983	Pcolce2	76477	9+:95538046-95595968
5984	Parva	57342	7+:119571451-119732297
5985	Dbx1	13172	7-:56886866-56892218
5986	Map3k15	270672	X+:156478917-156555772
5987	Lrrc14	223664	15+:76541169-76545520
5988	4933404O12Rik	66752	5+:137395075-137409528
5989	2310042D19Rik	74183	4+:155590823-155595417
5990	Rhbg	58176	3-:88047046-88058606
5991	Tsc22d2	72033	3+:58219610-58265759
5992	Znrf2	387524	6+:54766798-54843492
5993	Zcchc5	213436	X-:104032691-104047583
5994	Olfr609	259086	7-:110640431-110641390
5995	Zbtb43	71834	2-:33309218-33410287
5996	Tmod3	50875	9-:75345588-75407373
5997	3110057O12Rik	269423	3+:40650687-40679531
5998	Snrpd3	67332	10+:74982079-74998065
5999	RP23-275N21.2	329575	2-:177567215-177575136
6000	Olfr1111	258586	2-:86989878-86990816
6001	Psmb5	19173	14-:55232956-55236832
6002	Med24	23989	11-:98565914-98590681
6003	MMT00014122		13-:38766153-38766537
6004	Klra9	16640	6-:130128991-130139805
6005	Gucy2f	245650	X-:138523396-138631440
6006	Il5	16191	11+:53534295-53538605
6007	A630038E17Rik	219065	14+:54694891-54765832
6008	Lsg1	224092	16-:30561453-30587674
6009	Jmjd5	77035	7+:132588189-132605773
6010	1110057K04Rik	68832	12+:8214949-8283026
6011	Fbxl13	320118	5-:20989666-21029735
6012	Chid1	68038	7-:148681237-148725703
6013	Epha8	13842	4-:136485334-136512731
6014	Rnf5	54197	17-:34738514-34740418
6015	MMT00008782		8-:83832077-83832995
6016	A530057A03Rik	215456	2+:127259321-127261826

6017	MMT00018015	667716	11-:120388168-120393097
6018	Lrp10	65107	14+:55083081-55088865
6019	Gdap10	14546	12+:33506736-33511764
6020	EG640530	640530	6+:121683047-121734773
6021	MMT00008784		14-:93001678-93003616
6022	Glt25d2	269132	1+:154246995-154355917
6023	Saa4	20211	7-:53984018-53987943
6024	Ddo	70503	10+:40349864-40369234
6025	Il8rb	12765	1+:74200576-74206630
6026	MMT00058578		X-:151405749-151407971
6027	Rtn3	20168	19-:7500390-7557725
6028	Alas1	11655	9-:106137717-106150212
6029	Fmnl1	57778	11+:103032714-103059540
6030	As3mt	57344	19+:46781962-46815562
6031	MMT00035612		19+:11566536-11586198
6032	Rars	104458	11-:35621884-35648008
6033	Psd3	234353	8+:70212986-70214232
6034	Ipo9	226432	1-:137278894-137327055
6035	Nkd1	93960	8+:91045333-91116220
6036	Pram1	378460	17+:33766045-33782650
6037	Ccnl2	56036	4+:155195181-155198405
6038	Acot1	26897	12+:85350454-85358617
6039	Ccndbp1	17151	2+:120834246-120842627
6040	Nfkbie	18037	17+:45692889-45699015
6041	Penk1	18619	4-:4060684-4065921
6042	Oit1	18300	14-:9181462-9211277
6043	Cyp2ab1	224044	16-:20309922-20316132
6044	Sucnr1	84112	3+:59885790-59891481
6045	Podxl	27205	6-:31472572-31513818
6046	Nkain3	269513	4-:20396586-20412177
6047	Snrpg	68011	6+:86321533-86328896
6048	Psmd9	67151	5+:123678254-123698970
6049	Cpeb2	231207	5+:43626124-43628829
6050	Usp20	74270	2+:30837852-30878163
6051	4930511M11Rik	75010	5+:3657003-3679416
6052	Galnt1	14423	18+:24396847-24443356
6053	MMT00017114		13-:112151452-112211476
6054	Olfr1371	276865	11-:52026553-52027499
6055	Arhgap12	75415	18-:6024458-6135954
6056	Ndph	17986	X-:16462649-16488737
6057	Rnf216	108086	5-:143752572-143874662
6058	Myo1e	71602	9+:70055156-70150173
6059	Dsn1	66934	2-:156821000-156832811
6060	Tle6	114606	10-:81053649-81063645
6061	Bloc1s2	73689	19-:44214172-44220906
6062	E130308A19Rik	230259	4+:59750134-59767510
6063	Kif21a	16564	15-:90764813-90880242
6064	EG628119	628119	10-:95135624-95135995
6065	Plce1	74055	19+:38695004-38858367
6066	Ppp1r1a	58200	15-:103361353-103368313
6067	Spam1	20690	6+:24745851-24751048

6068	MMT00046866	191578	5-:101191177-101227555
6069	Banf1	23825	19-:5364747-5366608
6070	D630003M21Rik	228846	2-:158021486-158046331
6071	6720456B07Rik	101314	6+:113554765-113566944
6072	C1qc	12262	4-:136445724-136448788
6073	Mthfd2	17768	6-:83255699-83267598
6074	Noc4l	100608	5-:111077600-111082438
6075	6530404N21Rik	67795	19-:33456855-33467331
6076	Olfr1180	258920	2-:88251884-88252813
6077	Pik3cb	74769	9-:98938820-99022799
6078	Actrt1	73360	X+:43682183-43683522
6079	Tmem169	271711	1+:72330942-72348101
6080	Adam10	11487	9+:70526985-70626105
6081	Stox2	71069	8-:48271309-48373472
6082	Exoc2	66482	13-:30906971-31042167
6083	Cd40lg	21947	X+:54465319-54477219
6084	MMT00049235		1+:180229452-180230499
6085	MMT00007454		12-:64173516-64174101
6086	Fgl1	234199	8-:42276788-42300478
6087	MMT00074734		6+:53727376-53728056
6088	Bambi	68010	18+:3507931-3512756
6089	Tmem176a	66058	6+:48791507-48795363
6090	MMT00045476		
6091	Rbbp4	19646	4-:128986753-129012583
6092	Phlda3	27280	1+:137662709-137665710
6093	Sspo	243369	6+:48399660-48448773
6094	Otud3	73162	4-:138451297-138469949
6095	Cdkl3	213084	11+:51843344-51903279
6096	Acot3	171281	12+:85394401-85399975
6097	Pld5	319455	1-:177893958-177975093
6098	Ncf4	17972	15+:78075263-78093004
6099	Rims3	242662	4+:120527423-120564478
6100	Adprhl1	234072	8-:13235676-13254100
6101	Vmn2r71	233445	7+:92767046-92773055
6102	MMT00031107		2+:48243555-48243901
6103	Cytl1	231162	5+:38126786-38131053
6104	Aff4	93736	11+:53164334-53235332
6105	Cryaa	12954	17+:31814883-31818667
6106	EG627828	627828	8-:114538239-114538908
6107	Ttc30a2	620631	2-:75814228-75816223
6108	Ndufa8	68375	2-:35891853-35904812
6109	Hsd17b2	15486	8+:120225884-120282774
6110	MMT00063881	674274	X-:114997327-115000503
6111	Irgc1	210145	7-:25216945-25220554
6112	Tmem183a	57439	1-:136242673-136258532
6113	Sec61b	66212	4+:47487532-47496105
6114	Aim1l	230806	4+:133628372-133630966
6115	Cd164	53599	10+:41239305-41250847
6116	Pcgf5	76073	19+:36534818-36535452
6117	Atp9a	11981	2-:168459964-168567462
6118	Slc1a4	55963	11-:20202209-20232587

6119	Ankrd24	70615	10+:81103683-81110352
6120	MMT00013604		4-:103215973-103223326
6121	Dand5	23863	8-:87339303-87356198
6122	Tktl2	74419	8+:69035690-69037573
6123	Tmem77	67171	3+:106350797-106377068
6124	1700027L20Rik	69453	1+:89081327-89083008
6125	Grm6	108072	11+:50664643-50665184
6126	MMT00067575		X-:17251959-17253344
6127	MMT00009923		15+:76624254-76628458
6128	2210418O10Rik	76958	2-:175456017-175468166
6129	Msc	17681	1-:14743430-14746047
6130	G430022H21Rik	210529	3-:123071212-123089017
6131	Garnl1	56784	12-:56713628-56796197
6132	Zmat1	215693	X-:131506819-131543751
6133	Agrp	11604	8-:108090655-108091389
6134	Hspb8	80888	5-:116858505-116872873
6135	Nrg1	211323	8-:32928500-33028827
6136	Nrap	18175	19-:56394536-56464501
6137	MMT00039830		15-:8878312-8879219
6138	Tlr8	170744	X-:163680627-163701738
6139	Utp3	65961	5+:88983507-88985108
6140	MMT00034067	100039191	12-:89302466-89303300
6141	MMT00011697		
6142	Cenpp	66336	13-:49649046-49748153
6143	Pacsin2	23970	15-:83206547-83263249
6144	1700055N04Rik	73458	19+:3965901-3970438
6145	MMT00048203		3-:124291508-124292041
6146	Clta	12757	4+:44025555-44045717
6147	Dirc2	224132	16-:35696991-35769403
6148	Sp4	20688	12-:119473510-119474191
6149	Mn1	433938	5+:111846485-111883810
6150	Olfr1497	258736	19-:13869154-13870099
6151	MMT00002582		18+:7512631-7549523
6152	MMT00016029		4+:115024914-115035721
6153	Ankfn1	382543	11-:89283939-89290375
6154	1700030G06Rik	69507	5-:22229852-22231992
6155	AB056442	171405	19-:7938880-8040517
6156	Actr8	56249	14+:30791578-30806399
6157	MMT00041452		19+:40356699-40357187
6158	Msh2	17685	17+:88071898-88123052
6159	MMT00002938		16-:14010955-14011768
6160	MMT00060223		9+:102454352-102455005
6161	Olfr1510	258423	14-:53029588-53030356
6162	Sgsm1	52850	5-:113673165-113717919
6163	Nudt13	67725	14+:21113943-21135981
6164	Plekhh1	211945	12+:80141478-80183365
6165	Hhex	15242	19+:37509359-37514294
6166	Itpk1	217837	12-:103808035-103943081
6167	Pitpnm3	327958	11-:71863895-71949276
6168	AU022870	231874	5-:144748785-144775752
6169	Nap1l5	58243	6-:58855226-58857059

6170	Abcc6	27421	7-:53231749-53285652
6171	Abtb2	99382	2+:103518900-103558581
6172	Kif14	381293	1+:138392432-138424170
6173	Fndc1	68655	17-:7981858-8020167
6174	MMT00000714		5-:15004031-15004577
6175	V1rg1	252905	7-:12254947-12255868
6176	Id1	15901	2+:152562076-152562583
6177	Thpo	21832	16-:20725239-20730599
6178	Olfr1272	258982	2-:90121804-90122730
6179	Cxcl5	20311	5+:91188403-91190650
6180	Slk	20874	19+:47654508-47716944
6181	5430437J10Rik	71432	15+:5446230-5531127
6182	Gpr143	18241	X+:149216585-149242877
6183	Atxn10	54138	15+:85166697-85293394
6184	MMT00000944		7+:114093791-114096155
6185	Icam4	78369	9+:20833897-20834938
6186	Nono	53610	X+:98625053-98643928
6187	4930473A06Rik	320226	4+:83326908-83510420
6188	Myh7b	668940	2+:155437367-155460788
6189	MMT00020987		11+:79710708-79712255
6190	MMT00006494		9+:20359744-20360298
6191	Ddost	13200	4+:137860650-137868147
6192	Wars	22375	12-:110098522-110126696
6193	Lactb2	212442	1-:13615982-13650590
6194	Ube2f	67921	1+:93146887-93182317
6195	Mtx1	17827	3-:89013010-89017827
6196	Tpbpb	116913	13-:61002548-61006207
6197	Lss	16987	10+:75994371-76016671
6198	Lrrn2	16980	1+:134833775-134835966
6199	MMT00046861		2+:84499058-84507896
6200	Rhbdl1	214951	17-:25971410-25974072
6201	Slc2a7	435818	4+:149526236-149542590
6202	Hrk	12123	5+:118619772-118634966
6203	MMT00022643		X+:75869971-75870766
6204	Rbpj	19664	5+:53981583-54047272
6205	Ccl21b	18829	4+:42231058-42231890
6206	MMT00063701		7-:11692356-11693726
6207	Sh3tc2	225608	18+:62167299-62175374
6208	Il6st	16195	13+:113262816-113294806
6209	Dsp	109620	13+:38243178-38290443
6210	Hmgn3	94353	9-:83003554-83040132
6211	Arhgap24	231532	5+:102910571-103326843
6212	E130304F04Rik	245269	13_random+:66570-95207
6213	Ccl2	20296	11+:81849075-81850728
6214	Mmd2	75104	5-:143039439-143084739
6215	MMT00070426		6-:8327580-8329325
6216	Gm944	381126	18-:21286987-21458584
6217	MMT00019564		3-:95754703-95768299
6218	Hnrnpc	15381	14-:52694473-52723657
6219	BC030307	103220	10+:86221002-86226783
6220	Ifltd1	74071	6-:145491810-145562838

6221	Gng4	14706	13+:13895950-13917682
6222	MMT00015481		13-:56419316-56421695
6223	Zfp597	71063	16-:3865684-3872379
6224	Tnfrsf9	21942	4+:150299414-150319313
6225	Trim24	21848	6-:37816220-37816435
6226	Vmn2r77	546983	7+:93949999-93960378
6227	5830418K08Rik	319675	9-:15121358-15122533
6228	Csnk1g2	103236	10+:80085582-80103479
6229	MMT00000233		7+:112305375-112306194
6230	MMT00028343		7+:48591133-48593906
6231	9030622O22Rik	71570	2-:147770247-147855472
6232	BC031748	245622	X+:133584167-133612027
6233	Wnk2	75607	13-:49132968-49242559
6234	P4ha2	18452	11+:53915017-53945150
6235	Cyp2s1	74134	7-:26587494-26601548
6236	MMT00024286		15+:39012214-39013022
6237	Aup1	11993	6+:83004731-83007676
6238	Tmem165	21982	5-:76612471-76613479
6239	Aim1l	230806	4+:133634752-133638613
6240	MMT00074975		15-:59499016-59523317
6241	U2af1	108121	17-:31784159-31795641
6242	Rnf12	19820	X-:101152504-101156067
6243	Gucy2e	14919	11-:69036537-69050147
6244	Ing5	66262	1+:95700551-95716035
6245	Syap1	67043	X-:159294783-159326363
6246	MMT00010299		14+:40242946-40243798
6247	Adam5	11499	8-:25837568-25934513
6248	MMT00022090		3-:154939019-155056148
6249	Cep170	545389	1-:178663794-178737263
6250	Csnk2a1	12995	2+:152052631-152104985
6251	Ptger2	19217	14+:45608494-45622151
6252	Mael	98558	1-:168131503-168168897
6253	Bdkrb1	12061	12+:106842386-106843389
6254	MMT00039993		1+:147030975-147032072
6255	Vmn2r22	546913	6-:123598980-123622883
6256	EG666257	666257	9+:122818108-122827887
6257	Efcab1	66793	16+:14906738-14924615
6258	MMT00052726		
6259	Ms4a3	170813	19-:11703988-11715279
6260	MMT00002928		17+:8453516-8455093
6261	2810026P18Rik	72655	9-:88415892-88418223
6262	Hprt1	15452	X+:50341313-50374836
6263	Slc7a4	224022	16-:17573053-17576299
6264	MMT00078690		13-:61400625-61404287
6265	Trdn	76757	10+:32803315-33052271
6266	Gng10	14700	4+:59048069-59054285
6267	Homez	239099	14-:55475583-55479304
6268	Slc45a3	212980	1+:133867185-133879540
6269	MMT00003834		6-:41506785-41508370
6270	Cysltr1	58861	X-:103773094-103798935
6271	4833409A17Rik	74580	19-:42800347-42827265

6272	MMT00007926		
6273	Ctcfl	664799	2-:172927382-172947226
6274	Olfr695	258591	7-:113857245-113858193
6275	1700016M24Rik	69439	15-:74436458-74466783
6276	Olfm4	380924	14+:80400103-80422946
6277	Prdm1	12142	10-:44156982-44178554
6278	Lrrc47	72946	4+:153385924-153395619
6279	1700034O15Rik	76606	6+:41634429-41635710
6280	Pcf11	74737	7-:99791972-99818447
6281	Matn3	17182	12+:8954771-8977833
6282	Them4	75778	3+:94114087-94136460
6283	Ptpn22	19260	3+:103664214-103716169
6284	Cacnb2	12296	2+:14795279-14909057
6285	1700016C15Rik	69428	1+:179659943-179683436
6286	Rfc1	19687	5-:65653098-65726844
6287	Rabac1	14470	7-:25754594-25757781
6288	Metapl1	66559	2-:71361968-71366010
6289	Dab1	13131	4+:104040194-104352979
6290	2900079G21Rik	331033	9+:112136780-112138521
6291	Josd1	74158	15-:79504684-79518249
6292	Sesn3	75747	9+:14080845-14131435
6293	Pggt1b	225467	18-:46397165-46440650
6294	Wdr20b	70948	12+:66326503-66329441
6295	Parl	381038	16-:20279889-20302458
6296	Slc25a32	69906	15-:38925588-38944192
6297	2310079F23Rik	78890	5-:35899986-35917709
6298	Insm1	53626	2+:146047839-146050149
6299	MMT00063871		5-:110211255-110276791
6300	RP24-87L14.2	100125272	2+:177757942-177761426
6301	MMT00075917		7-:14024941-14025650
6302	C030046I01Rik	109284	10-:79372797-79379676
6303	MMT00055710		12-:116148240-116216332
6304	Mrgprf	211577	7+:152486813-152495462
6305	MMT00012844		17-:87607995-87609589
6306	Dab2	13132	15+:6336823-6389597
6307	Npepl1	228961	2+:173935865-173948199
6308	2410075B13Rik	223648	15-:75811102-75812323
6309	Cd209e	170780	8-:3849084-3854253
6310	Ap2s1	232910	7+:17323785-17334644
6311	MMT00074658		7+:14066063-14066450
6312	1500035H01Rik	76568	9+:44581063-44598976
6313	Sim1	20464	10+:50615456-50708958
6314	Hmcn2	665700	2+:31208128-31209377
6315	Epc2	227867	2+:49307300-49407169
6316	Olfr1475	258298	19-:13553743-13554686
6317	Crip1	12925	12+:114390263-114392088
6318	Cdc37	12539	9-:20943875-20954390
6319	D3Bwg0562e	229791	3-:117024820-117063166
6320	1810055E12Rik	67894	19+:60887513-60912130
6321	EG626841	626841	3+:80770788-80805385
6322	Pknox2	208076	9-:36698573-36776010

6323	Rnasen	14000	15+:12754609-12865045
6324	Zmym5	219105	14-:57410936-57411594
6325	Cpsf3l	71957	4+:155243714-155262977
6326	4930579J09Rik	67752	19-:10548747-10557387
6327	BC046404	192976	11-:62419198-62461802
6328	Maf	17132	8-:118214463-118229968
6329	Cyp2b10	13088	7+:26682694-26711474
6330	Dgkq	110524	5-:109075709-109089714
6331	Tll1	21892	8-:66493728-66684790
6332	Pmp2	18857	3-:10182120-10183819
6333	Iqcg	69707	16-:33016660-33045787
6334	Olfr960	258276	9+:39430709-39431658
6335	Gbp1	14468	3+:142261109-142262087
6336	MMT00013485		3+:59669154-59681097
6337	Trem1	58217	17+:48371947-48384102
6338	Setd3	52690	12-:109344649-109417473
6339	V1rc26	171199	6-:57339661-57340561
6340	Dynlt3	67117	X-:9233073-9240085
6341	MMT00066148		15-:82501708-82509830
6342	Dazap2	23994	15+:100446127-100451176
6343	Slc20a1	20515	2+:129024535-129037344
6344	Pcgf6	71041	19-:47108105-47125356
6345	Tdrd12	71981	7-:36272550-36274374
6346	Ppp2ca	19052	11+:51912550-51935474
6347	Rad18	58186	6-:112570798-112646588
6348	4930556G01Rik	75332	4+:30713971-30782894
6349	MMT00045058		4+:117397760-117399476
6350	Gcnt3	72077	9-:69881351-69884506
6351	3321401G04Rik	77574	6-:42618000-42659527
6352	Alms1	236266	6+:85537524-85652635
6353	Spata13	219140	14+:61325636-61383386
6354	Tlr7	170743	X-:163743146-163768424
6355	2810046L04Rik	212127	3+:53267584-53283715
6356	Dnahc1	110084	14-:32073559-32120346
6357	Ankrd9	74251	12-:112214633-112217206
6358	MMT00016595		7-:12058990-12185321
6359	Asphd2	72898	5-:112813377-112821279
6360	D17H6S56E-3	27762	17+:35153523-35163686
6361	Tmprss7	208171	16-:45680606-45685490
6362	Crispld2	78892	8+:122526690-122575693
6363	9130014G24Rik	215772	10-:10055507-10122805
6364	Cln3	12752	7-:133715599-133726669
6365	Rpl9	20005	5-:65779805-65782626
6366	Arrb1	109689	7+:106684244-106750660
6367	Lhfp	108927	3+:52845468-52847620
6368	4930444G20Rik	114671	10-:21786690-21787855
6369	Sh2b1	20399	7-:133610510-133616525
6370	Aqp8	11833	7+:130605825-130611500
6371	Dedd2	67379	7-:25987856-26004946
6372	1110021L09Rik	76306	10+:23516786-23547773
6373	MMT00020788		10+:129979421-129979507

6374	EG231885	231885	5-:145790471-145793239
6375	MMT00012910		6-:6300745-6302275
6376	Nle1	217011	11-:82714272-82721864
6377	D18Ertd653e	52662	18+:68387669-68415207
6378	EG432950	432950	15-:44803341-44847630
6379	Asb9	69299	X+:160935834-160977684
6380	6330569M22Rik	229707	3-:107415448-107434612
6381	Dmxl2	235380	9-:54212966-54328272
6382	Col23a1	237759	11+:51371951-51396050
6383	Gbp2	14469	3+:142283626-142300970
6384	Rbm12	75710	2-:155920627-155929101
6385	Men1	17283	19+:6335038-6340883
6386	Por	18984	5+:136165083-136211195
6387	MMT00042391		14+:105442696-105443405
6388	Dhtkd1	209692	2-:5831339-5842852
6389	Cstf2	108062	X+:130593751-130618995
6390	MMT00073452		10+:21009077-21010230
6391	Chkb	12651	15-:89256797-89260330
6392	Pdcl3	68833	1+:39044633-39054080
6393	3110043O21Rik	73205	4-:35138535-35173083
6394	Fbxo6	50762	4-:147519896-147525933
6395	Vmn2r81	216144	10+:78729934-78756451
6396	Zfp105	22646	9+:122832215-122840137
6397	Slc2a9	117591	5-:38740559-38893391
6398	Gm973	381260	1+:59605672-59639323
6399	Wnk1	232341	6-:119873993-119988364
6400	MMT00050153		1+:119637961-119883049
6401	MMT00053163	100041639	17-:15132750-15147126
6402	Npm3	18150	19-:45822225-45823640
6403	MMT00039607		14-:26661366-26662576
6404	Dbr1	83703	9+:99476235-99484485
6405	B3galnt1	26879	3-:69378854-69379848
6406	Sh3rf2	269016	18+:42213323-42313030
6407	Tacstd2	56753	6-:67484746-67485700
6408	Entpd8	72090	2+:24935841-24941238
6409	Hgf	15234	5+:16059377-16125258
6410	Psd3	234353	8-:70212983-70214044
6411	Adcy9	11515	16-:4287544-4419587
6412	MMT00012331		11+:84885970-84886514
6413	Fbxo4	106052	15-:3928766-3929707
6414	Yif1b	77254	7+:30023371-30032553
6415	OTTMUSG00000(	433485	2+:149725145-149829067
6416	Sox18	20672	2-:181404547-181406314
6417	Rasgef1c	74563	11+:49715351-49793724
6418	Cc2d2b	668310	19+:40839839-40901847
6419	MMT00028754		14+:74307464-74307769
6420	Mipep	70478	14+:61403394-61524315
6421	C230081A13Rik	244895	9-:56075514-56107846
6422	Grk5	14773	19+:60966242-61167953
6423	MMT00055016		11-:109020502-109054524
6424	Tdrd3	219249	14+:87816390-87945316

6425	MMT00025472		X-:131159558-131160130
6426	Mfsd4	213006	1-:133933357-133964488
6427	C86695	97476	8+:23069200-23074775
6428	MMT00035510		5-:30422633-30424209
6429	Sim2	20465	16+:94085175-94348630
6430	Slc35f5	74150	1+:127457612-127492392
6431	Nudt15	214254	14-:73920660-73925029
6432	2010109K11Rik	72123	12+:33064510-33065324
6433	Arpc4	68089	6+:113328108-113339643
6434	1700028P14Rik	67483	19-:23633249-23727302
6435	Tmem49	75909	11-:86420573-86497338
6436	4933401B06Rik	71011	X+:104737986-104739082
6437	Gnptg	214505	17-:25369169-25377082
6438	Vmn2r121	100038941	X-:121241111-121249525
6439	Samsn1	67742	16-:75945468-75947596
6440	Brunol6	76183	9+:59426184-59454125
6441	MMT00021409		14+:17471842-17556180
6442	Magi3	99470	3-:103819187-104023950
6443	Polr3d	67065	14-:70838602-70842950
6444	Cox7c	12867	13-:86184421-86186351
6445	Mllt3	70122	4-:87419819-87452166
6446	Tead1	21676	7+:119823106-120043504
6447	MMT00070270		X+:20105275-20105960
6448	MMT00065333		19-:8330430-8332076
6449	1190007F08Rik	68859	4-:153397196-153399760
6450	Arhgap29	214137	3+:121676788-121719671
6451	Olfr639	259088	7-:111160264-111161214
6452	MMT00021437		
6453	EG238395	238395	12+:105552779-105558791
6454	MMT00056567		5-:115278442-115279261
6455	Itgb3	16416	11+:104469369-104528689
6456	Tyms	22171	5-:30387351-30400180
6457	EG432951	432951	15-:45809598-45811605
6458	Sycn	68416	7+:29325896-29327224
6459	Cldn2	12738	X+:136343199-136343989
6460	Setd6	66083	8+:98239809-98242903
6461	Mtmr3	74302	11-:4380873-4494789
6462	Brip1	237911	11-:85874104-86014718
6463	Zfp1	22640	8+:114167333-114194914
6464	Pcbp4	59092	9+:106356282-106366340
6465	Zfp474	66758	18+:52775568-52799484
6466	Smarcc1	20588	9+:110034558-110141813
6467	Fndc4	64339	5-:31595264-31596549
6468	Sec61a1	53421	6-:88454849-88468806
6469	2700059D21Rik	433693	4+:34498185-34513819
6470	Arnt	11863	3+:95238458-95299492
6471	MMT00077542		4-:135271637-135272579
6472	MMT00005056		14+:55431701-55467410
6473	Kcnd3	56543	3+:105261733-105472125
6474	MMT00036909		8-:106322472-106324893
6475	Sdc4	20971	2-:164249747-164268686

6476	Sntg2	268534	12-:30859858-31058095
6477	Olfr675	258147	7-:112172538-112173480
6478	H2-Oa	15001	17+:34229376-34232179
6479	Tcerg1	56070	18+:42671176-42734498
6480	Lrfn5	238205	12+:62622416-62696244
6481	Atp1a1	11928	3-:101380147-101408580
6482	Nudt2	66401	4+:41412184-41427945
6483	M6prbp1	66905	17-:56425632-56432296
6484	A130010J15Rik	319266	1+:194999780-195002491
6485	Map3k7ip1	66513	15+:79963592-79992158
6486	OTTMUSG00000(	217066	11-:88826063-88826549
6487	Slc4a4	54403	5+:89316321-89664563
6488	Stat6	20852	10+:127080226-127097459
6489	MMT00008763		5-:121553095-121553514
6490	Il1r1	16177	1+:40281924-40373020
6491	Rpl5	19983	5+:108329620-108336243
6492	Wtap	60532	17-:13167884-13185353
6493	Dppa5a	434423	9-:78214862-78215961
6494	Evi5	14020	5-:108173814-108304126
6495	MMT00069880		9+:11947176-11947306
6496	4932441K18Rik	353170	X-:159219028-159267336
6497	Col4a3	12828	1+:82583550-82717843
6498	MMT00016809	100048067	1+:162965293-162968668
6499	Dtx3	80904	10-:126627434-126631688
6500	MMT00069546		10-:78784301-78785112
6501	Myog	17928	1+:136186589-136189125
6502	Col15a1	12819	4+:47221223-47325161
6503	Cep97	74201	16-:55905064-55934941
6504	Alad	17025	4-:62170201-62175226
6505	9230112E08Rik	320800	2+:180723595-180738188
6506	Scyl2	213326	10-:89102808-89149011
6507	MMT00041681		17+:38151846-38151884
6508	Map3k15	270672	X+:156559577-156561289
6509	EG434219	434219	7-:111530579-111539097
6510	Plcl2	224860	17+:50648932-50665916
6511	Bhlhb3	79362	6-:145811256-145813860
6512	Ryr2	20191	13-:11646782-12198713
6513	Qpctl	67369	7-:19726212-19734592
6514	U46068	228801	2+:154028301-154046079
6515	Eif2a	229317	3+:58329960-58361422
6516	Atf3	11910	1-:192995362-193001349
6517	2410017P09Rik	69709	12+:4234026-4236961
6518	MMT00073501		17+:63920248-63920797
6519	Phldb3	232970	7+:25396363-25414316
6520	Arnt2	11864	7-:91398290-91558424
6521	Gpr149	229357	3-:62334457-62408369
6522	Specc1	432572	11+:61856437-62033763
6523	Hapln1	12950	13+:89679400-89748004
6524	Nol1	110109	6+:125082185-125094769
6525	Uchl5ip	73738	X-:70682834-70704360
6526	Thumpd3	14911	6+:112996326-113018171

6527	MMT00051373		18-:83657546-83663696
6528	Dusp15	252864	2-:152770672-152777141
6529	Apeh	235606	9-:107987743-107996782
6530	MMT00077317		12+:110783607-110798586
6531	EG629557	629557	9-:51810380-51811386
6532	Auh	11992	13-:52930492-53025034
6533	Khsrp	16549	17-:57160473-57179396
6534	Cript	56724	17+:87424935-87435150
6535	Plcxd1	403178	5+:110528987-110532828
6536	MMT00038309		11-:4130756-4131113
6537	Kcna2	16490	3+:106904481-106906619
6538	MMT00075456		17+:88320434-88320760
6539	Npy2r	18167	3-:82342304-82352006
6540	Preb	50907	5-:31257422-31262608
6541	1810019J16Rik	69073	4+:133074949-133086704
6542	Epor	13857	9-:21763341-21767902
6543	Wnt9a	216795	11+:59146527-59147043
6544	AI790298	107239	19-:4164329-4175440
6545	Afm	280662	5+:90947988-90982571
6546	Tsn	22099	1-:120197215-120207709
6547	MMT00017970		13+:48407990-48409575
6548	MMT00047930		17+:82729178-82755264
6549	MMT00004752		
6550	MMT00056589		1+:159526434-159526797
6551	Slc35b4	58246	6-:34105878-34127054
6552	Ebf1	13591	11+:44431635-44818672
6553	MMT00003506		17-:8758802-8759773
6554	MMT00003845	670007	10-:99760711-99859697
6555	Aadacl2	639634	3+:59814418-59829162
6556	Osbpl2	228983	2+:179854077-179897163
6557	Mybl2	17865	2+:162898359-162909405
6558	Zfp395	380912	14-:66017248-66017752
6559	C230078M14Rik	241175	1+:102275627-102381277
6560	Mep1a	17287	17-:43611733-43639721
6561	Best3	382427	10+:116425670-116430409
6562	Dnm2	13430	9+:21229419-21311570
6563	Surf1	20930	2-:26768956-26771851
6564	Cebpd	12609	16+:15887105-15888559
6565	Dis3l	213550	9-:64157971-64189102
6566	Pigu	228812	2-:155140228-155183137
6567	Trpm3	226025	19+:22767088-23062796
6568	MMT00001575		13+:78260330-78262038
6569	Itih1	16424	14-:31742548-31756455
6570	Olfr148	258498	9+:39421153-39422085
6571	3000004C01Rik	70218	11-:102766891-102786403
6572	Foxc1	17300	13+:31898559-31902156
6573	Pecr	111175	1-:72305748-72330794
6574	Ptgis	19223	2-:167028699-167065955
6575	Kcnab1	16497	3+:64913693-65180488
6576	Slfn2	20556	11+:82878613-82884178
6577	Tnp1	21958	1-:73061650-73062446

6578	Pdcd10	56426	3-:75321043-75360677
6579	Gng7	14708	10-:80414140-80477653
6580	Svs3b	329557	2-:164080311-164082363
6581	Cdc42ep1	104445	15+:78673076-78681332
6582	MMT00009037		9-:72140355-72141148
6583	Tspyl1	22110	10+:34002031-34003486
6584	MMT00014057		13+:33858827-33903506
6585	Tacc1	320165	8-:26279666-26367061
6586	Olfr678	258753	7+:112217982-112218923
6587	Irf9	16391	14+:56223039-56228561
6588	Rbmx	19655	X-:54639519-54646158
6589	Mafb	16658	2-:160191441-160192798
6590	Pds5a	71521	5-:66014123-66039244
6591	Cops7a	26894	6-:124908838-124915384
6592	Lgals2	107753	15-:78681291-78685959
6593	Samd10	229011	2-:181329921-181334200
6594	Zfyve27	319740	19+:42245081-42266214
6595	Oas1a	246730	5-:121346267-121357516
6596	Olfr685	258160	7-:112328875-112329624
6597	Slc9a5	277973	8+:107873232-107893244
6598	Tgds	76355	14-:118511695-118531977
6599	1700020C11Rik	67900	11-:3991485-3995400
6600	Pdcd7	50996	9+:65193874-65207449
6601	Ttc30b	72421	2-:75774471-75776464
6602	Gaa	14387	11+:119129339-119146768
6603	Wdr40c	245403	X-:41719777-41721187
6604	Prps2	110639	X-:163786453-163820664
6605	Gpld1	14756	13+:25035080-25082624
6606	V1rf4	171235	17+:21423585-21424588
6607	Reln	19699	5-:21390286-21850508
6608	Mtap7d3	320923	X-:54061020-54064726
6609	Tor1b	30934	2+:30808503-30812761
6610	Rbm16	106583	17+:3145074-3198855
6611	Tbx19	83993	1-:167068600-167090855
6612	MMT00077820		19+:27100470-27227226
6613	Gmcl1	23885	6-:86641764-86683192
6614	Cd300lg	52685	11+:101902823-101916930
6615	1810023F06Rik	217845	12-:104689107-104695433
6616	Prmt3	71974	7+:57033714-57113634
6617	Rabepk	227746	2-:34634737-34655308
6618	Stk32a	269019	18+:43367237-43473173
6619	MMT00065825		1+:37180795-37194732
6620	Gpsm1	67839	2+:26175298-26204303
6621	Zfp521	225207	18-:13846723-14131047
6622	Ddef1	13196	15-:63919996-64040119
6623	Ptbp2	56195	3-:119421914-119486176
6624	Zfp94	22756	7-:25086814-25101620
6625	C1s	50908	6-:124487377-124492316
6626	9530068E07Rik	213673	11+:52209943-52222232
6627	Hmx1	15371	5+:35731765-35735519
6628	MMT00004313		17+:48821671-48822052

6629	Setx	269254	2+:29005539-29037990
6630	Ufc1	66155	1-:173218694-173225113
6631	Tmem30a	69981	9-:79616750-79641079
6632	Dio1	13370	4-:106964078-106979748
6633	Zfp775	243372	6+:48555559-48571530
6634	Ptpla	30963	2-:13948458-13977514
6635	MMT00000020		X+:90773874-90775207
6636	Ptchd2	242748	4-:147614641-147646513
6637	MMT00054486	544905	12-:115378931-115379333
6638	2610018G03Rik	70415	X+:48194612-48245025
6639	Fads3	60527	19+:10116095-10132739
6640	Mt4	17752	8+:96661141-96662766
6641	MMT00045188		
6642	Aldh2	11669	5-:122017711-122043595
6643	Ttll8	239591	15-:88720940-88756097
6644	Klhl9	242521	4-:88364198-88368747
6645	Myh6	17888	14-:55560819-55584297
6646	2900064A13Rik	73024	2+:112295203-112307593
6647	Samd14	217125	11+:94871672-94887397
6648	Cenpl	70454	1+:163001095-163016851
6649	Npvf	60531	6-:50600869-50604392
6650	MMT00050325		
6651	Gpx7	67305	4-:108073018-108079319
6652	MMT00004371		17-:50352073-50353549
6653	Zfand1	66361	3-:10339981-10351313
6654	MMT00043194		2+:147115490-147116396
6655	MMT00043469		1-:93385333-93386329
6656	Pdxdc1	94184	16-:13835684-13903056
6657	Irf6	54139	1+:194979355-194998198
6658	MMT00036759		6+:85888626-85889213
6659	Dpy19l4	381510	4-:11223082-11248777
6660	Mrto4	69902	4-:138903357-138908468
6661	Hdac1	433759	4-:129193347-129219890
6662	MMT00006703		11-:21776923-21777839
6663	MMT00053532		10-:30500660-30500991
6664	MMT00054031		11+:77542445-77580863
6665	AY026312	170939	16-:88932048-88932509
6666	MMT00037681		13-:22175327-22175657
6667	Chat	12647	14-:33221994-33274126
6668	1810022K09Rik	69126	3-:14606290-14611269
6669	MMT00059316		X-:7615684-7616144
6670	Nat10	98956	2-:103561381-103601407
6671	Pbxip1	229534	3+:89240666-89252868
6672	Utx	22289	X+:17740209-17794129
6673	Nasp	50927	4-:116274213-116300546
6674	MMT00016707	625336	4-:116512479-116513030
6675	Sfrs4	57317	4+:131453645-131457615
6676	9530048O09Rik	78611	4-:116792486-116798286
6677	Sestd1	228071	2+:77129084-77130035
6678	Dmxl1	240283	18+:50120711-50124079
6679	MMT00001738		

6680	Olfr393	259010	11-:73660686-73661625
6681	9130409I23Rik	619326	1+:182984887-182989945
6682	Olfr71	56015	4-:43718499-43719438
6683	Cd59a	12509	2+:103936021-103955137
6684	Myo1e	71602	9+:70167867-70247864
6685	Itgam	16409	7+:135206219-135261138
6686	Ldb3	24131	14-:35374453-35401860
6687	Ogfod1	270086	8+:96561094-96588935
6688	Mllt1	64144	17-:57033885-57066595
6689	BC107230	260408	9+:110701629-110767132
6690	MMT00027634	56546	7-:52933821-52934928
6691	Klrb1f	232408	6+:129002474-129003277
6692	MMT00043810		11+:96898300-96898918
6693	MMT00057347		13-:12775382-12776280
6694	Clpb	20480	7+:108812279-108936326
6695	Mrpl45	67036	11+:97177161-97190420
6696	Zfp740	68744	15+:102034999-102046031
6697	Serpini1	20713	3+:75361494-75446276
6698	MMT00063999		X-:57959446-57960326
6699	4930592I03Rik	75853	18-:83089332-83090009
6700	Dleu7	239133	14-:62895069-62912005
6701	Lrrc48	74665	11+:60188572-60191494
6702	H1fx	243529	6-:87930496-87931285
6703	Dbx2	223843	15-:95455200-95485193
6704	Sall4	99377	2-:168573835-168575470
6705	Sorbs2	234214	8+:46713537-46913253
6706	Serpinb3c	381286	1-:109167777-109174950
6707	1300017J02Rik	71775	9-:103152859-103190627
6708	Serpini2	67931	3-:75046293-75074002
6709	MMT00003854		6-:41424683-41431715
6710	MMT00022451		8+:73611709-73613824
6711	MMT00070969		11-:40382711-40384027
6712	Tssk1	22114	16+:17894386-17895744
6713	Lhx6	16874	2-:35939061-35960791
6714	Lima1	65970	15-:99608938-99650503
6715	Usp25	30940	16+:77014167-77116824
6716	Cd180	17079	13+:103483667-103497033
6717	Sh3gl2	20404	4+:84851396-85034605
6718	Rnf168	70238	16+:32277585-32301493
6719	Cacng8	81905	7+:3394458-3415366
6720	Adamts20	223838	15-:94116068-94233981
6721	Fis1	66437	5+:137437957-137442102
6722	Ppat	231327	5-:77342169-77380579
6723	MMT00024870		7-:109525536-109526906
6724	Gsg2	14841	11-:72948986-72951796
6725	Cnot3	232791	7+:3596953-3612701
6726	Meis2	17536	2-:115688829-115890783
6727	Dtd1	66044	2+:144425720-144594455
6728	Olfr75-ps1	258186	11-:73218945-73219881
6729	Hnrnpa1	15382	15+:103070924-103075386
6730	Slc24a2	76376	4-:86872786-86876329

6731	Dennd4c	329877	4+:86448812-86451529
6732	Olfr1038	259015	2+:85962081-85963037
6733	Mfng	17305	15-:78586312-78603875
6734	Ehd4	98878	2-:119915223-119980244
6735	Fus	233908	7+:135111067-135125545
6736	Spata4	69281	8+:55686133-55695451
6737	Vps26b	69091	9-:26815352-26837615
6738	Fxr1	14359	3+:33919029-33968259
6739	Sh2d2a	27371	3+:87650676-87656693
6740	Hp1bp3	15441	4+:137777427-137799132
6741	Rab39b	67790	X-:72819496-72823555
6742	Kctd8	243043	5-:69671796-69732540
6743	Ube2l3	22195	16-:17152108-17201585
6744	Olfr1362	258739	13-:21702910-21703836
6745	Rspo4	228770	2+:151668660-151699212
6746	E2f8	108961	7-:56121797-56136370
6747	Mars	216443	10-:126727867-126748827
6748	Mare	17168	11-:32132275-32167631
6749	Dnase1l3	13421	14-:8800262-8826662
6750	Sstr2	20606	11+:113480794-113487209
6751	MMT00073320		5-:81329653-81330681
6752	Grinl1a	28015	9-:71326249-71333740
6753	Smc6	67241	12+:11272971-11292605
6754	Trpv3	246788	11+:73081121-73110702
6755	Wdr22	320808	12-:81439378-81537574
6756	Sds	231691	5+:120926555-120933912
6757	Lbx2	16815	6+:83036417-83038213
6758	MMT00000302		8+:100287828-100400426
6759	Sqle	20775	15+:59146794-59162747
6760	2810428I15Rik	66462	8-:73028194-73030638
6761	MMT00030685		6-:69942472-70056173
6762	Ifnb1	15977	4-:88167929-88168698
6763	MMT00008804		3+:8214329-8318428
6764	Cald1	109624	6+:34659484-34724091
6765	Ldhb	16832	6-:142438769-142456403
6766	Sspn	16651	6+:145882666-145913740
6767	Erc2	238988	14+:28435693-28466669
6768	Plekhf2	71801	4-:10915811-10934717
6769	Banp	53325	8+:124474479-124549837
6770	Cdh22	104010	2-:164937657-164996417
6771	Insc	233752	7+:121912245-121993886
6772	Lyn	17096	4+:3683437-3685481
6773	Comt	12846	16-:18407636-18426605
6774	Cilp	214425	9+:65113632-65128423
6775	EG380809	380809	12-:115707262-115768307
6776	Slc25a20	57279	9+:108564558-108586224
6777	B3galtl	381694	5+:150511894-150563361
6778	Grm5	108071	7+:94732883-94751711
6779	Gbp6	229900	3+:142193333-142210014
6780	Adamts9	101401	6-:92727386-92746439
6781	Golga3	269682	5+:110610796-110652097

6782	Sh3bp5	24056	14-:32188170-32249047
6783	Zfp828	101994	8+:13869736-13881632
6784	AI182371	98870	2-:34937379-34957037
6785	Dpp6	13483	5+:27709291-28053492
6786	Necab1	69352	4-:14882080-15076111
6787	Fusip1	14105	4+:135412183-135419940
6788	Psen2	19165	1-:182157136-182176032
6789	Trp63	22061	16+:25802027-25892166
6790	Zdhhc24	70605	19+:4878688-4885390
6791	Hectd2	226098	19+:36693710-36695481
6792	4930444P10Rik	75799	1-:16058874-16083445
6793	Tmem16d	320091	10-:88579610-88612189
6794	MMT00071127		X+:19816516-19817413
6795	Cdyl2	75796	8-:119092630-119095285
6796	2810025M15Rik	69953	1+:159342907-159350364
6797	Blvra	109778	2+:126906253-126922775
6798	Tcea3	21401	4+:135803866-135830820
6799	Mon1a	72825	9+:107790512-107805456
6800	C130021I20Rik	329385	2+:33498028-33501851
6801	Zfp513	101023	5-:31501352-31504283
6802	Siah1b	20438	X-:160508634-160513981
6803	MMT00059375		3-:56939554-56940755
6804	Cyp2d22	56448	15-:82201909-82206451
6805	Rrbp1	81910	2-:143811601-143836914
6806	Agrn	11603	4-:155539402-155571448
6807	Trappc1	245828	11+:69137367-69139295
6808	EG433073	433073	17+:20798483-20798976
6809	Rorc	19885	3+:94176707-94202510
6810	Pcdhb1	93872	18+:37424651-37427106
6811	MMT00059520		
6812	MMT00040104		17+:3056713-3057111
6813	Olfr697	258592	7-:113884492-113885446
6814	V1rh7	171250	13-:22711965-22712904
6815	Sort1	20661	3+:108087057-108159607
6816	Slc46a3	71706	5-:148690029-148706391
6817	Lefty2	320202	1+:182823237-182827863
6818	BC030183	230766	4-:129254056-129255782
6819	Cct2	12461	10-:116488054-116500821
6820	MMT00046996		7+:14889997-14894927
6821	Ins2	16334	7-:149864617-149865438
6822	Ldhc	16833	7+:54116624-54133360
6823	Yars	107271	4+:128867107-128896482
6824	Irf7	54123	7-:148449087-148452300
6825	4930528G09Rik	75172	5-:20798784-20930496
6826	2610209A20Rik	67164	7+:107307786-107309875
6827	Tnks2	74493	19+:36909027-36967961
6828	Pigh	110417	12-:80183921-80190636
6829	Pi4k2b	67073	5+:53132849-53160576
6830	Rsc1a1	69994	4-:141239767-141241514
6831	MMT00078868		17-:76683528-76684279
6832	Aldh16a1	69748	7-:52396933-52409893

6833	MMT00056142		10-:96661802-96662943
6834	MMT00048596		17+:91524542-91525858
6835	BC026682	230579	4+:106406519-106420896
6836	MMT00042654	19051	6+:55967523-55982203
6837	Arhgap25	232201	6-:87409383-87446106
6838	Nod2	257632	8+:91176763-91212304
6839	Mrpl46	67308	7-:85920228-85927927
6840	4933424G06Rik	71166	1+:36771346-36814802
6841	Cpa5	74649	6+:30561009-30581521
6842	Amotl2	56332	9+:102624863-102635741
6843	0610038D11Rik	67674	19+:6984642-6985514
6844	Pdzd8	107368	19-:59370579-59420273
6845	Cyb561d1	72023	3-:107998702-108003354
6846	Olfr1402	258272	3-:97214139-97215102
6847	MMT00058311		19-:35126523-35126993
6848	Letm1	56384	5-:34084000-34125353
6849	Pigg	433931	5+:108741943-108777069
6850	EG386506	386506	14+:53394592-53395812
6851	Magohb	66441	6-:131234406-131243262
6852	MMT00007193		Y_random+:10231498-10250577
6853	Hils1	54388	11+:94828974-94829768
6854	Kcnj5	16521	9-:32125165-32130602
6855	Ash1l	192195	3+:88785599-88882514
6856	Tnrc6a	233833	7+:130323303-130337255
6857	Serinc2	230779	4-:129930739-129952831
6858	Gpatch4	66614	3+:87847029-87859909
6859	Ppp1r9a	243725	6+:5084647-5104249
6860	Kif23	71819	9-:61775054-61794429
6861	Krt8	16691	15-:101827141-101834714
6862	Olfr1055	259023	2-:86186982-86187921
6863	Rtdr1	71236	10-:74420223-74495312
6864	Sh3md4	237353	10+:58446668-58598735
6865	Fgf12	14167	16-:28372990-28445823
6866	Gls	14660	1-:52220295-52290240
6867	Gpr171	229323	3-:58900834-58905736
6868	Adcy1	432530	11+:6963584-6979040
6869	Tmem163	72160	1-:129387100-129449382
6870	Xlr4b	27083	X+:70459703-70467795
6871	Neurog2	11924	3+:127336292-127337973
6872	Med1	19014	11-:98013476-98054574
6873	BC023055	226123	19-:42149437-42160836
6874	Pcdh19	279653	X-:130121668-130221803
6875	Slc14a1	108052	18-:78298387-78320015
6876	Bmp15	12155	X-:5892566-5897724
6877	Kcnj10	16513	1+:174271486-174301017
6878	MMT00041953	675689	11+:46375532-46375875
6879	Lrrc16b	268747	14+:56113641-56117805
6880	Dmrtb1	56296	4-:107348899-107356822
6881	5031408O05Rik	331491	X-:103575296-103577519
6882	EG434078	434078	6+:113025843-113027220
6883	MMT00049189		16-:4016871-4017072

6884	Hnrnpf	98758	6+:117856833-117875635
6885	Psmg4	69666	13+:34254836-34270039
6886	4930473A06Rik	320226	4+:83171491-83266751
6887	Mrrf	67871	2+:35992186-36045390
6888	Cxcl9	17329	5-:92752015-92757143
6889	9030624J02Rik	71517	7+:125884245-125927569
6890	Chsy3	78923	18+:59335663-59339203
6891	Wdr57	66585	4+:130037415-130067274
6892	Adamts6	108154	13+:105077919-105269756
6893	Herc3	73998	6+:58781882-58854075
6894	Rtl1	353326	12-:110828379-110833313
6895	3-Sep	24050	15+:82105322-82122489
6896	MMT00078388		12+:39633174-39725375
6897	Galk1	14635	11-:115869839-115873992
6898	Mapk12	29857	15-:88961286-88971067
6899	Pla2g4e	329502	2-:119992149-120002680
6900	BC068281	238037	12+:4850180-4865055
6901	MMT00048285		9-:66653114-66654035
6902	Pigm	67556	1+:174306709-174308709
6903	Acrv1	11451	9+:36500834-36506429
6904	Sp6	83395	11+:96882631-96884720
6905	Pkd1l3	244646	8+:112138563-112145913
6906	2610301B20Rik	67157	4+:10801714-10826398
6907	Usp50	75083	2-:126535090-126539157
6908	Mmp12	17381	9+:7347457-7358683
6909	Ctsm	64139	13-:61637624-61643170
6910	Ly6c2	100093633	15-:74938587-74942237
6911	C130090K23Rik	231293	5+:73797488-73844515
6912	Crym	12971	7-:127329897-127345502
6913	MMT00056400		7-:18270473-18271665
6914	Thrap3	230753	4-:125842644-125844308
6915	5830405M20Rik	74749	1+:182685342-182689075
6916	Wdr5b	69544	16+:36041360-36043058
6917	MMT00070118		7+:13188438-13189997
6918	Cog8	97484	8-:109570428-109572154
6919	Zdhhc19	245308	16+:32499176-32507299
6920	EG629059	629059	14+:63174574-63225261
6921	Olfml1	244198	7+:114710990-114733856
6922	Tmem86b	68255	7-:4579635-4581784
6923	1110017D15Rik	73721	4-:41452048-41454789
6924	Cxxc1	74322	18+:74375867-74381145
6925	Arhgef2	16800	3+:88425059-88443458
6926	BC059841	407813	14+:60261581-60262535
6927	Parp6	67287	9+:59465183-59472825
6928	Klhl8	246293	5-:104291069-104340215
6929	Nfasc	269116	1-:134465113-134638354
6930	Efnb2	13642	8-:8619014-8660641
6931	Hrsp12	15473	15-:34413891-34424945
6932	Ckap4	216197	10-:83989048-83996368
6933	Pqbp1	54633	X-:7471808-7475743
6934	Panx3	208098	9-:37468658-37476776

6935	Arsj	271970	3+:126067769-126143293
6936	Rxra	20181	2+:27532769-27615594
6937	B3gnt6	272411	7-:105342086-105347990
6938	Epha1	13835	6-:42308767-42323209
6939	1110006G06Rik	73833	7-:29937530-29941202
6940	Sec23a	20334	12-:60074014-60108197
6941	Gm459	243451	6-:68860396-68860932
6942	Ubr4	69116	4+:138944222-138952596
6943	MMT00074478		X-:111166697-111167432
6944	EG665536	665536	8+:13887559-13896630
6945	Lmtk3	381983	7+:53040975-53059250
6946	Nol5	55989	1+:59741942-59768352
6947	Lass6	241447	2+:68699506-68946649
6948	Olfr898	258871	9+:38156669-38157619
6949	Usp48	170707	4+:137161663-137169776
6950	Zfp710	209225	7+:87225934-87235824
6951	MMT00075568		2+:153239539-153240068
6952	Ep300	328572	15+:81416575-81482506
6953	Nlrp6	101613	7+:148108308-148114434
6954	Defb4	56519	8+:19198724-19201413
6955	Fut9	14348	4-:25545934-25726754
6956	Gria2	14800	3-:80492368-80606508
6957	BC017647	216971	11+:77929127-77969583
6958	Sval1	71578	6+:41901628-41906097
6959	Slc1a5	20514	7+:17366755-17383591
6960	4930539C22Rik	75208	3-:134067105-134082477
6961	MMT00073650		17+:64542090-64542324
6962	Cyp1b1	13078	17-:80106293-80114381
6963	MMT00033481		13-:85711871-85713200
6964	1200011M11Rik	74133	11-:86891233-86899878
6965	Nsg2	18197	11+:31900458-31959202
6966	Mia3	338366	1_random-:300175-342929
6967	MMT00070157		11+:69764299-69764733
6968	Psca	72373	15+:74545268-74547499
6969	Jub	16475	14-:55186305-55196367
6970	Dagla	269060	19-:10321772-10330723
6971	Gcat	26912	15+:78861345-78867893
6972	Trspap1	71787	4-:131867679-131885431
6973	RP23-281O10.5	195555	4-:143485120-143487828
6974	Tmem65	74868	15-:58613824-58654683
6975	Wdr67	210544	15+:57743754-57801626
6976	Rbm35b	77411	8-:108653857-108692989
6977	Lman1	70361	18-:66143346-66162274
6978	Nat8b	434057	6-:85882726-85883343
6979	ORF9	52793	16-:97692692-97726543
6980	Pla2g4d	78390	2-:120091609-120103314
6981	MMT00027115	544718	10+:89539720-89821960
6982	Rcc1	100088	4-:131887840-131901577
6983	BC003267	233987	8+:4613166-4629548
6984	Gapdh	14433	6-:125112004-125113178
6985	Ntng1	80883	3-:109946353-110040056

6986	Gm1821	624036	14+:46703800-46704073
6987	Hspg2	15530	4+:137024735-137126544
6988	Lims1	110829	10+:57786213-57885404
6989	Klk15	317652	7+:51173842-51194953
6990	Nr1i2	18171	16-:38248436-38294910
6991	Prkar1b	19085	5-:139494582-139605653
6992	Olfr172	259003	16-:58760359-58761287
6993	AI987662	101202	6-:3407096-3437430
6994	Glul	14645	1+:155747097-155756824
6995	Ebp	13595	X-:7762458-7770002
6996	Zbtb16	235320	9-:48465020-48644335
6997	Gm1568	380768	12-:81858445-81861686
6998	EG245651	245651	X-:138967983-138968278
6999	Snx16	74718	3-:10418936-10440090
7000	Map3k12	26404	15-:102329875-102339769
7001	Cox7a2l	20463	17-:83901270-83913641
7002	Lrrn1	16979	6+:107479778-107520204
7003	MMT00008726		11+:104692598-104892629
7004	Pzp	11287	6-:128433591-128476731
7005	Chmp6	208092	11+:119775160-119780866
7006	MMT00030635	672450	6+:70306118-70322028
7007	Rab8a	17274	8+:74685242-74704035
7008	Myo7a	17921	7-:105199838-105255617
7009	Tmprss11c	435845	5-:86660821-86668523
7010	Mlph	171531	1+:92811679-92847718
7011	1810022C23Rik	69123	13-:35038661-35055678
7012	Dennd4a	102442	9+:64686640-64767472
7013	Atp10b	319767	11+:42965251-42966922
7014	Fbxl22	74165	9-:66358971-66362418
7015	Pbrm1	66923	14+:31902737-31933206
7016	Cdc26	66440	4-:62055622-62065585
7017	Rfc4	106344	16-:23114021-23127803
7018	Rbm19	74111	5+:120566523-120648987
7019	Zp3	22788	5+:136455974-136464493
7020	Fbln5	23876	12-:102988149-103057279
7021	Slc16a8	57274	15-:79081443-79085187
7022	Unc5a	107448	13+:55051022-55107371
7023	Agpat4	68262	17+:12312149-12412506
7024	Apln	30878	X-:45381477-45387682
7025	Erc2	238988	14+:28590010-28591003
7026	BC055324	381306	1-:165876123-165924912
7027	5730410I19Rik	66622	12+:103996204-104015916
7028	Olfr974	259111	9+:39749846-39750779
7029	MMT00072012		12+:31569085-31578350
7030	RP23-232H13.5	100043387	2+:176503994-176871927
7031	Wipf3	330319	6+:54431778-54439607
7032	Slc5a3	53881	16+:92077301-92079457
7033	Rfwd3	234736	8-:113796920-113824080
7034	MMT00067631		
7035	4933425L06Rik	66763	13+:105872201-105911860
7036	MMT00012416		11+:88271091-88392059

7037	MMT00041357		15-:93509065-93510432
7038	Abhd5	67469	9+:122260849-122290641
7039	Zmat3	22401	3-:32233719-32264542
7040	4933409G03Rik	227998	2+:68439918-68454435
7041	Sp140	434484	1+:87511062-87516733
7042	C730007P19Rik	20865	7+:14320095-14323700
7043	Cyp11b2	13072	15-:74681468-74686655
7044	Vbp1	22327	X+:72759691-72780249
7045	Olfr1234	258975	2-:89202640-89203584
7046	MMT00044125		18-:45445488-45463340
7047	MMT00019310		12-:13759481-13760503
7048	9830169C18Rik	217705	12-:85686266-85702738
7049	Psmd14	59029	2+:61549825-61638432
7050	1700027J05Rik	69440	15-:89015244-89017852
7051	Zfhx4	80892	3+:5241671-5414508
7052	Arid4a	238247	12+:72116978-72199899
7053	Mmp24	17391	2+:155601120-155642061
7054	Trerf1	224829	17+:47450769-47496407
7055	Eprs	107508	1+:187186957-187193555
7056	Rad54l2	81000	9-:106594571-106691416
7057	Stk10	20868	11+:32433304-32524587
7058	Asns	27053	6-:7625165-7643106
7059	MMT00031310		Y-:2462130-2468679
7060	Xpr1	19775	1-:157127914-157264459
7061	Spsb3	79043	17+:25024425-25029092
7062	ENSMUSG00000(	100041269	15+:76124715-76127804
7063	Cab39l	69008	14+:60059817-60204601
7064	BC037703	242125	3-:101627185-101640073
7065	Vmn2r110	224582	17-:20711311-20721399
7066	Agtr1b	11608	3-:20214282-20302434
7067	EG545758	545758	5-:72973292-72978626
7068	Sec22c	215474	9-:121592137-121614141
7069	Ube1l	74153	9+:107877852-107886389
7070	Rpl11	67025	4-:135605862-135609287
7071	Fgf5	14176	5+:98683267-98704576
7072	Punc	19289	9+:64988995-65033675
7073	Arhgap4	171207	X-:71139697-71156568
7074	Dcbld2	73379	16+:58460843-58465940
7075	Pax7	18509	4-:139294001-139388817
7076	MMT00078003	665032	5-:116251615-116252195
7077	MMT00064540		5-:109956480-109958571
7078	Olfr463	258408	11-:87706489-87707424
7079	Gm884	380730	11-:103403079-103482700
7080	0610038F07Rik	66072	19-:10576934-10599603
7081	Wdr40a	68970	4-:41238333-41261911
7082	MMT00068724		13-:66328085-66963054
7083	MMT00044154		18+:44221143-44222334
7084	Spz1	79401	13-:93344784-93345921
7085	St8sia2	20450	7-:81084007-81158547
7086	Calu	12321	6+:29298180-29326667
7087	Dio2	13371	12-:91962991-91976878

7088	Slc37a1	224674	17+:31432075-31487641
7089	AI646023	192734	10-:75012869-75022834
7090	2010003O02Rik	66434	4+:40216617-40217973
7091	Myf5	17877	10-:106919966-106922987
7092	Rom1	19881	19-:9001883-9003678
7093	Magt1	67075	X-:103165815-103207117
7094	Cyp2c54	404195	19-:40112424-40122137
7095	Timm8a1	30058	X-:131071796-131076168
7096	Npat	244879	9+:53345036-53382448
7097	Dnajb6	23950	5+:30062564-30093628
7098	Pcdhb18	93889	18+:37649272-37651650
7099	Kcnk10	72258	12-:99694928-99812922
7100	Tomm22	223696	15+:79501298-79504002
7101	Gimap6	231931	6-:48651579-48658212
7102	Hnrnpm	76936	17-:33783183-33822348
7103	Zfp524	66056	7+:4967109-4970090
7104	Inpp4b	234515	8+:84476077-84646686
7105	Plac8	231507	5-:100982751-101001224
7106	MMT00077123		14-:43798424-43800938
7107	Olfr663	257914	7+:111852142-111853117
7108	Adamtsl1	77739	4+:86061429-86072048
7109	Bxdc2	67832	15-:10405936-10415579
7110	Pnpla1	433091	17+:28995811-29015520
7111	Pfdn5	56612	15+:102156603-102161905
7112	Apbb3	225372	18-:36830813-36839020
7113	Phf13	230936	4-:151365578-151368201
7114	Schip1	30953	3+:68298495-68299180
7115	Cstl1	228756	2+:148576543-148581127
7116	Katnal1	231912	5-:149687626-149740223
7117	MMT00015170		4-:99131796-99187118
7118	BC051019	57355	7-:116855695-116867392
7119	Dlg3	53310	X+:97963090-98013747
7120	Dusp1	19252	17-:26642536-26645406
7121	Cc2d1a	212139	8-:86656727-86671652
7122	Gigyf2	227331	1+:89329694-89347367
7123	A630050E13Rik	319701	11+:16853292-16854449
7124	Dnajb13	69387	7-:107650250-107661908
7125	5230400G24Rik	75734	1+:82721514-82748291
7126	MMT00002620		2+:73973812-73988889
7127	MMT00007705		2+:120064295-120064626
7128	Gemin8	237221	X+:162614904-162627732
7129	Frat1	14296	19+:41904605-41907073
7130	Vdr	22337	15-:97687803-97738695
7131	1810037I17Rik	67704	3+:122627317-122628654
7132	Sp9	381373	2+:73109987-73112393
7133	Hook2	170833	8+:87514517-87527160
7134	Krba1	77827	6+:48345612-48366884
7135	Ezr	22350	17-:6942812-6987069
7136	Id3	15903	4+:135699736-135701301
7137	Psmc2	19181	5+:21291099-21309606
7138	Cyb561d2	56368	9-:107441890-107444442

7139	Pld3	18807	7-:28317036-28338131
7140	Mtbp	105837	15+:55389041-55457968
7141	MMT00061218	629364	12-:25176464-25178595
7142	Wdr23	28199	14+:56180060-56188901
7143	Cxcr7	12778	1+:92100600-92112182
7144	Zfp169	67911	13-:48584454-48586276
7145	2810021G02Rik	353208	2-:177802399-177813224
7146	0610037D15Rik	68394	4+:116381173-116386756
7147	2610307P16Rik	72518	13-:28552550-28864538
7148	Tln2	70549	9-:67165989-67245454
7149	9530077C05Rik	68283	9+:22215922-22249127
7150	Scg2	20254	1-:79431726-79433579
7151	MMT00007982		6-:67684189-67685598
7152	MMT00010289		14-:36803638-36864433
7153	Glt6d1	71103	2-:25666520-25671357
7154	Fbxw13	211305	9-:109085520-109087904
7155	MMT00017420		14+:53303921-53304140
7156	Tmem1	216131	10-:77649474-77670789
7157	BC038822	239647	15+:97204487-97216120
7158	Nudt16	75686	9-:105032002-105034135
7159	1700031F05Rik	73300	X-:100054526-100061695
7160	Rbm6	19654	9-:107675895-107775029
7161	Malt1	240354	18+:65590647-65637428
7162	Vps72	21427	3+:94914992-94926855
7163	Ptplb	70757	16+:35022513-35106660
7164	Ppp1r3a	140491	6-:14663581-14705246
7165	Nt5m	103850	11+:59661574-59690034
7166	Tmlhe	192289	X_random+:159646-334185
7167	Ear5	54159	14+:51782138-51782606
7168	Cep55	74107	19+:38129497-38149227
7169	Sestd1	228071	2-:77024062-77083145
7170	Magix	54634	X-:7251753-7258173
7171	Nmd3	97112	3+:69525976-69552966
7172	Nme2	18103	11-:93811129-93816939
7173	Trim59	66949	3-:68839215-68841208
7174	Creb3l1	26427	2-:91822810-91864288
7175	MMT00002215		X+:9278279-9331284
7176	MMT00026882		9+:39972217-39972542
7177	4930511M06Rik	75084	18-:57705017-57711535
7178	P4ha3	320452	7+:107465551-107467592
7179	Rfxdc2	319758	9+:72380626-72470624
7180	Marveld1	277010	19+:42221906-42226485
7181	Nedd4	17999	9+:72525155-72595035
7182	Olfr434	258366	6+:43166913-43167879
7183	MMT00040424		X-:109572654-109573976
7184	Klhl26	234378	8-:72973402-73000878
7185	Ostf1	20409	19-:18655244-18706299
7186	Tatdn2	381801	6+:113647419-113660322
7187	Aasdh	231326	5-:77311365-77316555
7188	Atpbd4	66632	2-:114342155-114480619
7189	MMT00064788		13-:45281845-45282781

7190	MMT00009543		11+:114770015-114770770
7191	Fgfr1op	75296	17+:8361897-8389343
7192	Ttc21b	73668	2-:66022384-66090061
7193	Cdx2	12591	5-:148113250-148118788
7194	9430020K01Rik	240185	18+:4673044-4680423
7195	MMT00051203		12+:33394928-33398576
7196	Kcng1	241794	2-:168087489-168107236
7197	MMT00059753		3-:29672396-29792155
7198	Glod4	67201	11-:76035011-76057158
7199	Wee1	22390	7+:117265585-117285647
7200	Rcc2	108911	4+:140256453-140278813
7201	Kcnh7	170738	2-:63019525-63022334
7202	9530003J23Rik	77397	10-:116670816-116675662
7203	Scmh1	29871	4+:120078013-120202560
7204	V1re4	171227	17-:21050245-21051300
7205	Phka1	18679	X-:99711193-99839481
7206	Rgma	244058	7+:80520573-80564784
7207	Olfr1203	258898	2+:88671275-88672197
7208	Trex2	24102	X-:70679268-70679978
7209	Gpa33	59290	1+:168060590-168096640
7210	E130012A19Rik	103551	11-:97489486-97490595
7211	2410014A08Rik	109154	5-:115593029-115608170
7212	MMT00015229		13+:54236301-54237126
7213	MMT00003697		7-:133944395-133952072
7214	Itln1	16429	1-:173459251-173465426
7215	Mip	17339	10+:127662937-127667733
7216	MMT00063554		2+:87233613-87236461
7217	MMT00004295		17+:23010426-23010972
7218	Cylc2	74914	4+:51229548-51242569
7219	Ptpn4	19258	1-:121556321-121699052
7220	1110018M03Rik	67606	2-:110201081-110203150
7221	Mcpt8	17231	14-:56701002-56704034
7222	MMT00063574		
7223	Enam	13801	5+:88916999-88933737
7224	Pafah2	100163	4+:133953385-133983323
7225	Tiam1	21844	16-:89787356-89961072
7226	Defcr3	13237	8+:22427076-22427288
7227	MMT00022763		3+:119155269-119380921
7228	OTTMUSG00000C	328483	14+:54048401-54050831
7229	H2-DMb1	14999	17+:34282252-34297169
7230	Pecam1	18613	11-:106516194-106576465
7231	Setd2	235626	9+:110449709-110521131
7232	MMT00078948		7+:146960610-146960924
7233	5430433E21Rik	74492	9-:65238083-65239459
7234	MMT00005439		7-:12277020-12277559
7235	Psg29	114872	7+:17790215-17797252
7236	L2hgdh	217666	12-:70791439-70825805
7237	Esm1	71690	13+:114000050-114006947
7238	Pycard	66824	7-:135136171-135137239
7239	Zfp819	74400	7+:50862594-50873649
7240	C730007P19Rik	20865	7+:14338705-14341327

7241	Ptpn11	19247	5-:121583799-121641283
7242	2210012G02Rik	66526	4-:106811427-106850925
7243	Slc4a10	94229	2+:61884650-62164782
7244	MMT00034282		3-:120883187-120884060
7245	Ski	20481	4-:154531449-154596629
7246	Xkr5	319581	8-:18933494-18950933
7247	4930452B06Rik	74430	14-:9334934-9498753
7248	4930562C15Rik	78809	16+:4841105-4852127
7249	Smchd1	74355	17-:71791217-71824663
7250	Aldh1l2	216188	10-:82951991-82996805
7251	MMT00018228		16-:16365094-16366304
7252	Nolc1	70769	19+:46150502-46157814
7253	Olfr593	258378	7+:110360375-110361358
7254	Fsd2	244091	7-:88679985-88704863
7255	EG621629	621629	13+:40873408-40947794
7256	MMT00063357		13+:107223869-107266980
7257	Mcfd2	193813	17-:87654006-87665255
7258	Bmp2k	140780	5+:97426707-97520066
7259	4930511H11Rik	78016	1+:54307472-54335805
7260	Uncx	22255	5+:140019891-140024130
7261	Ttc7b	104718	12-:101538998-101758909
7262	MMT00063675		7+:11668231-11674384
7263	Narg1l	66897	14-:79735440-79790484
7264	MMT00041369		6-:116145436-116145876
7265	Dok1	13448	6-:82980951-82983430
7266	Olfr354	258941	2+:36762467-36763419
7267	Clrn3	212070	7-:142705622-142720316
7268	Slco5a1	240726	1-:12861878-12980576
7269	Aip	11632	19-:4114447-4125827
7270	MMT00008590		2-:147896605-147897623
7271	Lsm11	72290	11-:45747017-45758426
7272	Krt1	16678	15-:101675944-101681225
7273	Rnf103	22644	6+:71443887-71460875
7274	Laptm4b	114128	15-:34199401-34199584
7275	Trim35	66854	14+:66915932-66928341
7276	Rbm41	237073	X-:136480231-136533073
7277	Adrb2	11555	18-:62338151-62353069
7278	1810030J14Rik	66289	1+:176260649-176263004
7279	Cldn13	57255	5-:135390571-135391199
7280	Rgs8	67792	1+:155543199-155544554
7281	MMT00048662		1+:3977264-3977944
7282	Ppic	19038	18-:53565994-53577661
7283	Pnmt	18948	11+:98247945-98249410
7284	MMT00008674		5-:121378079-121382183
7285	Tbc1d4	210789	14-:101841584-101910783
7286	Noxo1	71893	17+:24833743-24837519
7287	MMT00006239		
7288	Gcet2	14525	16+:45610552-45621343
7289	L1cam	16728	X-:71100373-71115143
7290	2010111I01Rik	72061	13+:63116526-63169645
7291	EG639576	639576	18+:76778069-76876083

7292	Spna1	20739	1+:176102998-176178198
7293	Zdhhc18	503610	4-:133162627-133183290
7294	Mbd3	17192	10-:79855285-79862224
7295	Olfr54	18354	11+:50840505-50841447
7296	MMT00013662		4+:97803638-97813188
7297	Cbln2	12405	18+:86881456-86887676
7298	Mpp7	75739	18-:7350833-7561872
7299	Pelp1	75273	11-:70216132-70223533
7300	Olfr560	259117	7-:109901496-109902441
7301	Ubtf	21429	11+:102180046-102180517
7302	MMT00021433		9+:21896943-21897351
7303	Tmem182	381339	1+:40862448-40913731
7304	1700020N18Rik	67086	1-:93301453-93302608
7305	Olfr389	259011	11-:73589890-73590827
7306	Zcchc8	70650	5-:124149806-124170949
7307	Agmat	75986	4+:141302582-141315177
7308	Tmem52	69671	4+:154843221-154844966
7309	Mpp5	56217	12+:79850007-79939068
7310	Dagla	269060	19-:10336983-10379365
7311	Lsm6	78651	8-:81330466-81345036
7312	Creg2	263764	1-:39679589-39707778
7313	Tnfsf14	50930	17-:57328914-57333600
7314	Sgpp2	433323	1+:78387049-78390023
7315	EG624219	624219	9+:21639938-21641788
7316	Ifi204	15951	1-:175677426-175843051
7317	Raver2	242570	4+:100741841-100823425
7318	Rfpl4	192658	7-:5061468-5068498
7319	Olfr1490	258098	19+:13728920-13729886
7320	MMT00051732		7+:148592427-148593223
7321	Rdbp	27632	17+:34987863-34993103
7322	D730048I06Rik	68171	9-:35595694-35597668
7323	Txk	22165	5-:73087220-73127841
7324	Mtap	66902	4+:88783020-88826994
7325	Cops3	26572	11-:59631307-59653269
7326	MMT00049214		X+:148089730-148090357
7327	Fscn3	56223	6+:28377788-28388613
7328	Atp5b	11947	10+:127520382-127527328
7329	Tsen2	381802	6+:115497836-115528369
7330	U2af2	22185	7+:5019631-5031534
7331	Slc23a2	54338	2-:131882016-131927295
7332	1700027L20Rik	69453	1+:89083238-89084979
7333	Olfr1339	258851	4+:118407135-118408082
7334	Lor	16939	3-:91884203-91887062
7335	Calm2	12314	17-:87833367-87842067
7336	Cd5l	11801	3+:87161896-87174994
7337	Usp53	99526	3+:122654204-122657473
7338	MMT00070692		19+:8346969-8349204
7339	Tdp1	104884	12+:101122734-101144344
7340	Ascc3l1	320632	2+:127034132-127067416
7341	Olfr1424	258676	19-:12133300-12149054
7342	Anp32e	66471	3+:95733504-95749494

7343	Mcm10	70024	2-:4911213-4933778
7344	Herc2	15204	7+:63305758-63370256
7345	Foxo6	329934	4-:119939866-119959857
7346	MMT00002542		1-:4246317-4250516
7347	1110032A04Rik	66183	3-:69623462-69663796
7348	Lce1h	67718	3-:92567136-92568987
7349	MMT00065780		14+:15270505-15271223
7350	Rrp15	67223	1-:188544970-188573188
7351	Asb6	72323	2-:30678616-30685853
7352	MMT00015446		4-:135312148-135312610
7353	Fras1	231470	5+:96973968-97029820
7354	Fyttd1	69823	16+:32877899-32906805
7355	Procr	19124	2+:155577078-155581194
7356	MMT00005729		12+:116855015-116855234
7357	MMT00006424	545547	3-:92589302-92610901
7358	Slc1a1	20510	19+:28909655-28988449
7359	Evi1	14013	3-:29851638-29896586
7360	Mfi2	30060	16+:31878895-31899103
7361	Ddx50	94213	10-:62078770-62113946
7362	Rrm2	20135	12+:25393147-25398972
7363	Canx	12330	11-:50109784-50125279
7364	Prr16	71373	18+:51446101-51463018
7365	Gpr111	435529	17-:42847008-42851425
7366	BC008163	230789	4-:132456538-132478426
7367	Gchfr	320415	2+:118993513-118998125
7368	Adam8	11501	7-:147164839-147178387
7369	AW549877	106064	15-:3932037-3942078
7370	Spop	20747	11+:95275427-95354327
7371	MMT00063687		5-:110546730-110547947
7372	Fastk	66587	5-:23946857-23950188
7373	MMT00000121		6-:85758479-85759857
7374	Obox6	252830	7-:16418826-16420298
7375	1700036A12Rik	73298	9+:60590923-60618081
7376	3110067C02Rik	73202	X+:146898818-146900060
7377	Suds3	71954	5-:117541690-117566836
7378	1700001J03Rik	69282	5-:146994023-146996876
7379	MMT00078052		19-:37778218-37797762
7380	MMT00077370		7-:72726907-72780800
7381	B9d1	27078	11+:61318673-61326429
7382	RP23-34B24.1	230161	4-:49460165-49464007
7383	Olfr398	258705	11-:73797163-73798108
7384	MMT00079090		11+:48381856-48382528
7385	Timm17a	21854	1-:137197815-137210328
7386	Pex6	224824	17+:46848411-46862489
7387	Pcdh7	54216	5+:58473001-58520590
7388	Amn	93835	12+:112509308-112514469
7389	Sfrs5	20384	12+:82047562-82051486
7390	Ppp2r4	110854	2+:30271653-30303325
7391	Bhlhb8	17341	5+:144951154-144952534
7392	MMT00009784		15+:74285488-74347741
7393	Igk-V28	16114	6+:70143469-70165974

7394	Arid4b	94246	13+:14156971-14291870
7395	Tmprss11d	231382	5-:86731880-86802412
7396	Lao1	100470	4+:118634571-118641489
7397	MMT00020969		3+:144390767-144392049
7398	Rbmxrt	19656	8-:81029168-81032738
7399	1700008A04Rik	69351	11-:32720376-32724575
7400	Cars2	71941	8-:11513978-11550744
7401	Pdzk1ip1	67182	4+:114761327-114766498
7402	Plcd4	18802	1+:74595751-74612554
7403	Zbtb25	109929	12-:77449568-77470501
7404	Prpmp5	381832	6-:132261612-132314135
7405	Snx31	66696	15-:36434159-36485328
7406	Sult2a1	20859	7-:14417914-14422751
7407	Slc9a3r1	26941	11+:115024716-115042493
7408	1700081L11Rik	76719	11-:104194813-104218190
7409	MMT00014691		19-:27836215-27837137
7410	Arfip2	76932	7-:112784135-112788867
7411	MMT00041340		X-:92020117-92031365
7412	Nkg7	72310	7+:50692673-50693466
7413	Olfr1101	258584	2-:86828400-86829331
7414	Clpx	270166	9+:65142115-65178449
7415	Isoc1	66307	18+:58819340-58837894
7416	6430548M08Rik	234797	8+:122638059-122687323
7417	MMT00011799		11+:82784682-82786238
7418	Pkp3	56460	7+:148265676-148276408
7419	Arid5a	214855	1+:36364625-36377372
7420	4833413D08Rik	71425	2+:154083575-154092715
7421	Nlrp4g	235779	9_random+:129260-148047
7422	MMT00067092	637957	7+:127696665-127765631
7423	Wdr74	107071	19+:8810328-8815113
7424	Cdkn1c	12577	7-:150644615-150646883
7425	MMT00033805		15+:3719612-3880561
7426	Sprr2b	20756	3+:92121370-92121666
7427	Flt3l	14256	7-:52387483-52391420
7428	Rnps1	19826	17+:24552037-24562840
7429	Lphn2	99633	3-:148478550-148553592
7430	Gpr137	107173	19-:7012560-7016900
7431	Idh1	15926	1-:65205847-65221943
7432	Abl2	11352	1+:158489179-158648878
7433	Zfp248	72720	6-:118378172-118429270
7434	Slc18a3	20508	14-:33276017-33277610
7435	Fads6	328035	11-:115144834-115146800
7436	1700063K16Rik	76610	16-:76819829-76842029
7437	1700003F12Rik	75480	2+:154374621-154375743
7438	MMT00046942		10-:39278404-39308263
7439	Syt17	110058	7-:125525369-125587066
7440	Fbxo28	67948	1-:184246493-184271788
7441	Ppp1ca	19045	19+:4192176-4195417
7442	Ambra1	228361	2+:91570381-91759004
7443	Upf3a	67031	8+:13785637-13798721
7444	Hspe1	15528	1+:55145175-55148039

7445	Polr2h	245841	16+:20717898-20722337
7446	Shc4	271849	2-:125454806-125549113
7447	MMT00014979		4-:108100953-108123947
7448	Atr	245000	9+:95758143-95852061
7449	Creg1	433375	1+:167693910-167704160
7450	Mfsd9	211798	1-:40830581-40847506
7451	Mast1	56527	8-:87435804-87461235
7452	Abcc9	20928	6-:142537652-142650793
7453	Olfr1104	258763	2-:86861768-86862699
7454	Gnl3	30877	14-:31825259-31832222
7455	MMT00011288		4+:151530158-151531670
7456	Scfd2	212986	5-:74858582-74927745
7457	Mrpl22	216767	11+:57985176-57993080
7458	Cav1	12389	6+:17256449-17289453
7459	2610207O16Rik	66620	1-:42763909-42770328
7460	Krt6a	16687	15-:101520898-101524679
7461	Mrps23	64656	11+:88017943-88023670
7462	Sacs	50720	14+:61799781-61808635
7463	Kcns2	16539	15+:34767512-34770271
7464	MMT00059239		17-:3311765-3314181
7465	MMT00014720		
7466	Insl6	27356	19-:29395844-29399808
7467	Dlx6	13396	6+:6813745-6817331
7468	Hsd3b1	15492	3-:98656117-98662142
7469	Sez6l	56747	5-:112853642-112904607
7470	MMT00057330		13+:13129063-13131202
7471	MMT00030575		7+:81396057-81396384
7472	Klra1	16627	6-:130313935-130332546
7473	Grik5	14809	7-:25794894-25857317
7474	Tbx2	21385	11+:85646579-85654632
7475	Wdr72	546144	9+:73982234-74058594
7476	MMT00059569		13-:66497459-66891917
7477	D630037F22Rik	544696	10-:55921693-55948438
7478	B230209C24Rik	320705	1-:33919497-33964660
7479	Mcf2l	17207	8+:12873825-13020508
7480	4930506M07Rik	71653	19-:59047846-59150114
7481	Trh	22044	6-:92192055-92193882
7482	Tdg	21665	10+:82092625-82113018
7483	Cyp3a25	56388	5-:146788338-146821192
7484	Ccdc77	67200	6-:120274715-120307378
7485	Csf3r	12986	4+:125701979-125721683
7486	1700019N19Rik	67507	19-:58860292-58868904
7487	MMT00067815	545536	3-:88380158-88381112
7488	Ttn	22138	2-:76679873-76686699
7489	Rnuxa	56698	18+:56732693-56746825
7490	Olfr702	258590	7-:113967083-113968038
7491	Ankrd34c	330998	9-:89624688-89633311
7492	Adfp	11520	4-:86302617-86315898
7493	Cngb3	30952	4+:19207996-19437768
7494	EG232930	232930	7-:19094648-19096990
7495	Ugt2b5	22238	5-:87553984-87569343

7496	Arsi	545260	18+:61071649-61078212
7497	Ndrg3	29812	2-:156763062-156763287
7498	MMT00040284		12+:71072008-71073929
7499	Nipa1	233280	7-:63233855-63274944
7500	MMT00011283	433546	2-:156615456-156616113
7501	1700067P10Rik	68224	17+:48226593-48227870
7502	Cd3e	12501	9-:44806825-44817673
7503	Rab11fip1	75767	8-:28251273-28285121
7504	Olfr267	258922	4-:58797651-58798592
7505	Kpna2	16647	11-:106849956-106858626
7506	Tomm34	67145	2-:163879276-163896838
7507	Ccdc136	232664	6+:29349588-29376952
7508	2210021J22Rik	72355	15-:85637403-85642123
7509	MMT00008937		11+:106833514-106834848
7510	Gins3	78833	8+:98157458-98168959
7511	MMT00058829		3-:41289079-41290298
7512	1110004F10Rik	56372	7+:123236893-123248723
7513	Nr5a2	26424	1-:138740852-138857025
7514	Spnb4	80297	7-:28141402-28231610
7515	MMT00069689		16-:51016636-51017020
7516	MMT00018931	100040020	X-:150499736-150504063
7517	T	20997	17+:8627287-8635361
7518	V1rf3	171234	17+:21450980-21451848
7519	Gstm1	14862	3-:107815175-107820891
7520	MMT00070057		X+:65882566-65883524
7521	Gmfg	63986	7+:29222490-29231560
7522	Rb1	19645	14-:73595309-73725547
7523	Daam1	208846	12+:72932055-73016780
7524	Aldh4a1	212647	4+:139178825-139204933
7525	Trim61	260296	8-:67536884-67542428
7526	Aifm1	26926	X-:45828251-45866566
7527	Rnase10	75019	14+:51627649-51630431
7528	Cadm1	54725	9+:47596063-47659115
7529	Fgfr3	14184	5+:34064491-34078479
7530	Thnsl2	232078	6-:71078158-71094432
7531	Lrrc36	270091	8+:107949156-107987980
7532	MMT00011542		13+:24412170-24412614
7533	Pscd2	19158	7-:53063050-53069621
7534	MMT00022070		3+:125980942-125982020
7535	Ttc13	234875	8-:127212756-127245895
7536	MMT00004030	629915	12-:116184137-116229792
7537	Glce	93683	9-:61907817-61918647
7538	Igfn1	226438	1-:137868482-137871424
7539	Eef2	13629	10+:80627326-80645252
7540	Olfr658	259051	7-:111792912-111793884
7541	Eif3h	68135	15-:51618109-51697007
7542	Ppp1r3b	244416	8+:36439708-36447917
7543	Cct5	12465	15-:31520638-31531559
7544	Btbd14a	67991	2-:25915420-25978304
7545	C81431	98077	17-:34012613-34027561
7546	Ddhd1	114874	14-:46214211-46240515

7547	Cd24a	12484	10+:43298988-43304068
7548	Pbk	52033	14+:66424747-66436658
7549	Tmem154	320782	3+:84470178-84505906
7550	Rab11fip5	52055	6-:85284958-85324456
7551	Grik3	14807	4+:125300724-125385592
7552	Olfr77	258336	9+:19724654-19725592
7553	Ints8	72656	4-:11126305-11180104
7554	Tmem146	106757	17+:56766279-56793179
7555	AA960436	101985	8+:97856239-97871407
7556	A530050D06Rik	104816	12+:113345081-113365771
7557	Acbd7	78245	2+:3253437-3258268
7558	Rbm10	236732	X+:20197121-20228014
7559	Fabp6	16204	11-:43409614-43415013
7560	Cyp2r1	244209	7-:121693702-121706471
7561	Mmp17	23948	5+:130090093-130112569
7562	MMT00078102		1+:19321016-19321952
7563	Xkr8	381560	4-:132282890-132288380
7564	P4ha3	320452	7+:107434042-107454553
7565	Il3	16187	11-:54078907-54080779
7566	Olfr1297	258890	2-:111461292-111462229
7567	Olfr1451	258700	19+:13073477-13074408
7568	Lrp6	16974	6-:134404713-134406303
7569	Ntn2l	18209	17-:24343401-24346087
7570	MMT00069130		4-:81101842-81107336
7571	Casz1	69743	4+:148275797-148327420
7572	Ppm1l	242083	3+:69301690-69359313
7573	Magea4	17140	X+:69467355-69468302
7574	MMT00042247		8-:82202798-82204744
7575	Pgpep1	66522	8-:73173782-73183566
7576	Hmox2	15369	16+:4741612-4766248
7577	Cldn8	54420	16-:88562602-88563280
7578	Gfpt2	14584	11+:49607690-49652115
7579	Pip5k1c	18717	10+:80755724-80780650
7580	Slc22a20	381203	19-:5971398-5986140
7581	MMT00034696		1+:34424294-34425704
7582	MMT00065786		14+:14754201-14780995
7583	Zbtb20	56490	16+:43510420-43619182
7584	Myoc	17926	1+:164569394-164579331
7585	Vmn2r76	675969	7-:93377167-93379682
7586	5730469M10Rik	70564	14-:41807028-41827064
7587	Igh-VJ558	16061	12-:114503113-114503244
7588	Sec14l1	74136	11+:116976562-117020535
7589	4933404M02Rik	66748	2+:70346873-70378940
7590	Ankrd40	71452	11+:94199602-94203160
7591	MMT00077934		3-:94408331-94408774
7592	Chrna5	110835	9+:54828678-54854730
7593	Krt28	70843	11-:99226185-99228357
7594	Sned1	208777	1+:95158194-95179565
7595	Ccdc103	73293	11+:102742548-102745670
7596	Tifa	211550	3+:127494298-127501306
7597	4933417G07Rik	71182	3+:55651026-55652321

7598	MMT00013990		19-:14673035-14739140
7599	Olfr1273	258977	2-:90136093-90137016
7600	Zfp382	233060	7+:30915692-30937948
7601	Kcne3	57442	7+:107329675-107333378
7602	Pcca	110821	14+:122933564-123289047
7603	Sod2	20656	17+:13200859-13208205
7604	MMT00003898		6+:41062211-41064132
7605	MMT00047828		2-:136569883-136570094
7606	MMT00037360		10+:120365363-120365944
7607	Theg	21830	10-:79039124-79050077
7608	4931440J10Rik	71000	14+:63379336-63438532
7609	Ctrc	76701	4-:141394566-141402200
7610	Scand1	19018	2-:156137593-156138262
7611	Chrm4	12672	2+:91767405-91768845
7612	MMT00014216		6-:133707411-133713096
7613	MMT00038919		18-:28345733-28347327
7614	Gpkow	209416	X+:7274299-7283887
7615	MMT00045521		7+:31048240-31048684
7616	EG546101	546101	8-:108184234-108184874
7617	Tom1l2	216810	11-:60043518-60084050
7618	Glis2	83396	16+:4608595-4614628
7619	Pik3ip1	216505	11+:3230382-3242971
7620	Foxo3	219038	14+:52538462-52540381
7621	MMT00042139		7-:26577317-26577868
7622	Gpr155	68526	2-:73179564-73220365
7623	9430069I07Rik	77358	15-:34284462-34286190
7624	Mum1l1	245631	X+:135769253-135771297
7625	Krt32	16670	11-:99942170-99949429
7626	Cebpz	12607	17-:79319185-79336377
7627	Ppil6	73075	10+:41210280-41227371
7628	MMT00041592		5+:152083699-152084757
7629	Bnip1	224630	17+:26918026-26929469
7630	Atrnl1	226255	19+:57685843-57871985
7631	Uhrf1bp1	224648	17+:27993567-28033295
7632	Gsdmc3	270328	15-:63689826-63691916
7633	Pik3r6	104709	11+:68316577-68343577
7634	1200013P24Rik	74763	16+:3884768-3904769
7635	Syngr2	20973	11+:117670994-117675595
7636	Brunol4	108013	18-:25837903-25912250
7637	MMT00056309	382035	8-:125144181-125146633
7638	Hs1bp3	58240	12+:8320265-8349568
7639	Tmem149	101883	7+:31351098-31352977
7640	MMT00047329		4-:118345213-118346127
7641	Cyp26a1	13082	19+:37772343-37776026
7642	Igl-V1	16142	16-:19061844-19085553
7643	Mrps28	66230	3-:8802156-8923814
7644	MMT00033296		11+:76327564-76327948
7645	MMT00045828		11+:86892275-86892785
7646	Sfrs18	66625	4+:21775071-21803672
7647	Pcna	18538	2-:132075022-132078916
7648	Olfr63	258939	17+:33405670-33406619

7649	Scyl3	240880	1+:165860157-165884147
7650	Olfr438	258648	6+:43103305-43104237
7651	Tmem192	73067	8+:67471079-67492933
7652	Dnahc7b	227058	1+:46154914-46430417
7653	1700061J05Rik	73376	15-:78208828-78226317
7654	Ptpmt1	66461	2-:90750866-90758602
7655	Tmem30b	238257	12-:74646264-74647326
7656	Lonrf3	74365	X+:33916422-33917017
7657	Olfr731	258360	14-:50857579-50858558
7658	Lce1l	73730	3-:92653870-92655208
7659	Rab30	75985	7+:99968414-99985157
7660	Olfr208	258382	16-:59361125-59362042
7661	Rac1	19353	5-:144266352-144288979
7662	Lyz1	17110	10-:116725639-116729894
7663	4930488L21Rik	75809	8-:96335590-96337455
7664	Mcm3	17215	1-:20804479-20805008
7665	MMT00047694		2-:116353578-116368456
7666	Gcgr	14527	11+:120395988-120400400
7667	5033414K04Rik	98496	1-:84111904-84155958
7668	Prss21	57256	17+:24005038-24010079
7669	Pik3ap1	83490	19-:41348707-41459560
7670	Ccdc57	71276	11-:120687844-120734986
7671	Hoxb1	15407	11+:96227065-96228802
7672	V1rg5	171240	7+:12432123-12433038
7673	Mog	17441	17-:37148449-37160144
7674	Spata7	104871	12+:99866372-99908029
7675	AI413782	104799	12-:88579824-88607159
7676	Jmjd2c	76804	4+:73897851-74051765
7677	BC003885	225215	9+:72961309-72970142
7678	Ltbr	17000	6-:125256593-125263872
7679	Epb4.1l4b	54357	4-:57008606-57053924
7680	EG667232	667232	16-:36569040-36580930
7681	Abpg	110187	7-:34728017-34729983
7682	MMT00018333		
7683	Man1a2	17156	3-:100370339-100489150
7684	Ppp3r1	19058	11+:17092846-17095086
7685	Eif3b	27979	5+:140895279-140919322
7686	Apoa5	66113	9+:46076690-46079492
7687	BC050254	269630	5-:22889961-22939147
7688	MMT00058344		13-:64423876-64424841
7689	Olfr708	258173	7+:114057713-114058635
7690	4632417N05Rik	74032	8-:120185298-120195415
7691	Tnnc1	21924	14+:32021497-32024897
7692	Cd84	12523	
7693	B3galt6	117592	4-:155365330-155366731
7694	Olfr330	258879	11-:58342539-58343486
7695	Cpsf1	94230	15-:76426238-76437984
7696	Pkd2l1	329064	19-:44223642-44266932
7697	Exod1	71151	7-:126930249-126937481
7698	Klrb1a	17057	6-:128559634-128572952
7699	Gm885	380732	11+:106612588-106626605

7700	Iqce	74239	5-:141139461-141178210
7701	Sipa1l3	74206	7-:30105391-30185861
7702	Wdr33	74320	18+:31986938-32066649
7703	Tmprss11f	243083	5-:86967014-87061440
7704	V1rk1	171261	7-:13716821-13717707
7705	Gjb1	14618	X+:98572675-98580961
7706	MMT00031300		Y+:2551480-2631669
7707	Mll3	231051	5-:24777617-24893859
7708	Rxra	20181	2-:27617024-27618471
7709	Rdx	19684	9+:51856431-51896841
7710	V1rc32	171205	6+:56985959-56986869
7711	Slc25a23	66972	17-:57183135-57184155
7712	Ubxd3	212190	4-:138275605-138281113
7713	2610110G12Rik	73242	17-:36036788-36046985
7714	Cdh2	12558	18-:16747385-16967239
7715	Slfn5	327978	11+:82773984-82775014
7716	Sema6c	20360	3+:94968295-94977238
7717	Pskh1	244631	8+:108424328-108455704
7718	Pdia6	71853	12+:17273353-17291577
7719	Myo18b	74376	5-:113121117-113307247
7720	Sec14l3	380683	11+:3964900-3966705
7721	MMT00000356		8-:110214319-110214744
7722	Zfp407	240476	18-:84727718-84758898
7723	Olfr1471	258231	19+:13519635-13535485
7724	Pmpca	66865	2+:26243766-26264701
7725	3230401D17Rik	66680	2-:163231672-163245224
7726	Cyp2d9	13105	15+:82282828-82287257
7727	MMT00065667		14+:121112299-121112967
7728	A530088H08Rik	193003	11+:66725540-66739773
7729	Vmn2r106	224576	17-:20404510-20416095
7730	Taf1b	21340	12+:25183496-25243228
7731	1500001M20Rik	68971	6-:114954374-114987893
7732	Acox1	11430	11-:116033204-116060085
7733	Hsd17b4	15488	18+:50287993-50355840
7734	Cyp2b23	243881	7-:27450574-27471449
7735	Traip	22036	9+:107853299-107873859
7736	2610036L11Rik	66311	10-:29915821-29920415
7737	Bmpr1a	12166	14-:35227673-35273740
7738	Adam21	56622	12-:82659783-82661973
7739	Grm8	14823	6-:27225611-28076195
7740	Gpha2	170458	19+:6226787-6227602
7741	Abcf3	27406	16+:20558629-20561845
7742	Spag9	70834	11+:93857582-93987393
7743	Foxp1	108655	6+:98875334-98877062
7744	Cadps	27062	14-:13205099-13655559
7745	C330016O10Rik	212706	11-:51456591-51464288
7746	Gpr61	229714	3-:107952911-107957802
7747	Olfr17	18314	7+:114240980-114241927
7748	Chek1	12649	9-:36517410-36533464
7749	Mul1	68350	4+:137990605-137995818
7750	Dgcr2	13356	16-:17841402-17891689

7751	Sgk2	27219	2+:162813281-162839862
7752	Ugp2	216558	11-:21221798-21270480
7753	Gfm1	28030	3+:67234045-67278989
7754	Chst3	53374	10-:59648352-59656987
7755	B230317C12Rik	56279	2+:26483991-26492014
7756	Edc3	353190	9+:57587690-57597973
7757	Slc38a8	234788	8-:122009349-122025193
7758	1700039E15Rik	76713	7+:52535253-52543227
7759	Aktip	14339	8-:93647399-93658363
7760	2810012G03Rik	66306	18+:34919209-34933407
7761	Nudt19	110959	7-:36332211-36340852
7762	MMT00037909		12-:4696207-4696758
7763	2010204K13Rik	68355	X-:6989231-7000074
7764	Mtpn	14489	6+:35459066-35461068
7765	Pgbd1	319207	13-:21513735-21526588
7766	MMT00073093		X+:150091149-150091642
7767	Bai3	210933	1-:25124323-25188246
7768	V1rd3	81015	7-:5146518-5148349
7769	Arbp	11837	5+:116009523-116013520
7770	Galt	14430	4+:41702375-41705553
7771	MMT00024336		13-:64558717-64559950
7772	Rffl	67338	11-:82619108-82642532
7773	1110013L07Rik	68521	3+:88987489-88993214
7774	Mc5r	17203	18+:68497256-68499376
7775	Nif3l1	65102	1+:58501994-58518367
7776	Ube2v1	66589	2-:167415839-167480236
7777	MMT00022778		19+:18001042-18003100
7778	Dnase1l1	69537	X-:71518556-71527075
7779	Slit2	20563	5+:48666501-48697803
7780	Ankrd7	75196	6+:18816327-18829582
7781	Pip	18716	6+:41797552-41802063
7782	Gm114	228730	2+:146681598-146716820
7783	Pgm2	72157	4+:99602131-99659609
7784	Msh4	55993	3-:153520112-153569102
7785	MMT00078891		7+:143684648-143849755
7786	Ang3	11730	14+:44534733-44540744
7787	MMT00043009		9-:97127501-97129284
7788	1600021P15Rik	239796	16-:28826263-28929784
7789	Ppt2	54397	17-:34754289-34763932
7790	Traf2	22030	2-:25374384-25402450
7791	Dtx4	207521	19-:12543625-12575707
7792	6720467C03Rik	68099	4-:12080869-12098989
7793	MMT00070312	668699	14+:103409421-103410015
7794	Hbs1l	56422	10+:21015939-21030325
7795	MMT00071427		7-:26204174-26204845
7796	Olfr976	258364	9-:39763573-39764554
7797	Ddx47	67755	6+:134961637-134973789
7798	MMT00077178		19+:11511939-11513093
7799	Myd88	17874	9-:119246474-119249077
7800	Rgr	57811	14-:37850811-37862120
7801	Zcwpw2	74638	9-:117790897-117923278

7802	Adcy4	104110	14-:56387929-56402856
7803	Ttyh1	57776	7+:4071178-4085578
7804	Pgk2	18663	17-:40343971-40345500
7805	Trpm8	171382	1+:90199856-90285426
7806	D1Ertd53e	213056	1-:58584099-58643136
7807	2410004N09Rik	69749	18+:33954572-33956736
7808	Bnc2	242509	4-:83921000-84037038
7809	Pus1	56361	5-:111202690-111208916
7810	Trim45	229644	3+:100726399-100735625
7811	Olfr1226	258969	2-:89033258-89034189
7812	Ipo7	233726	7+:117161785-117200388
7813	Stac	20840	9-:111463937-111592720
7814	Stard3nl	76205	13-:19449546-19487624
7815	Pcdha10	12943	18+:37164973-37347311
7816	Btnl1	100038862	17+:34514099-34522971
7817	Lats1	16798	10+:7411300-7433444
7818	MMT00032604		Y_random-:8866783-8891515
7819	Npas4	225872	19-:4988286-4989970
7820	Ankar	319695	1-:72689556-72696774
7821	MMT00042003		7-:97959541-97960744
7822	Coq10a	210582	10-:127800149-127805773
7823	Pdgfb	18591	15-:79827003-79845407
7824	MMT00001823	100044551	1-:82999078-83001428
7825	Tex16	83556	X+:109207128-109240935
7826	Ephb2	13844	4-:136211031-136391932
7827	1700010A17Rik	75495	2+:35904997-35935236
7828	Limd2	67803	11-:106018401-106021467
7829	Ptprn	19275	1-:75243617-75260727
7830	Heatr5b	320473	17-:79164667-79173057
7831	BC024479	235184	9+:37324863-37331544
7832	MMT00036236		19-:53209519-53209803
7833	Mcts2	66405	2+:152513081-152513683
7834	MMT00032281		
7835	1110020C03Rik	68625	4-:118227155-118242052
7836	Wdr93	626359	7+:86916402-86922191
7837	Pou3f1	18991	4+:124334895-124336413
7838	Prl8a6	19112	13-:27524723-27530466
7839	Asl	109900	5-:130487373-130500201
7840	4931433A01Rik	78933	4+:111287564-111289916
7841	MMT00018824		X-:139618050-139618945
7842	Pdk2	18604	11-:94887572-94902629
7843	V1ri1	252907	13-:22279015-22279917
7844	Btc	12223	5-:91787835-91831845
7845	MMT00002915		13+:34958032-34958257
7846	Pacs1	107975	19-:5133160-5168011
7847	Adra2c	11553	5+:35622534-35623910
7848	Casp12	12364	9+:5345510-5372647
7849	3930401K13Rik	74022	16-:5014029-5049942
7850	C8b	110382	4+:104438926-104477148
7851	Fank1	66930	7+:140968564-141073215
7852	Ing3	71777	6+:21900037-21923699

7853	MMT00033089		12-:105411512-105416134
7854	Igh-VJ558	16061	12-:116831134-116831806
7855	Csrnp2	207785	15-:100312190-100320016
7856	Slc35b3	108652	13-:39024176-39052398
7857	Myo1h	231646	5+:114806823-114814585
7858	1810007P19Rik	230751	4+:125735607-125766578
7859	Eif5b	226982	1+:38054989-38109434
7860	Bmp6	12161	13+:38437584-38591597
7861	Klhdc4	234825	8-:124320233-124353469
7862	Cpsf4l	52670	11-:113559485-113571331
7863	Nova1	664883	12-:47821662-47919751
7864	Ipo11	76582	13-:107665567-107727028
7865	MMT00062659		15-:41861949-41864525
7866	1810054D07Rik	69863	4-:82867854-82869105
7867	Obfc1	108689	19-:47575527-47611607
7868	Dcdc5	329482	2+:106209865-106225780
7869	4930401B11Rik	73823	2-:78685555-78693415
7870	Rsad1	237926	11-:94404194-94410521
7871	MMT00052129		1+:43466263-43468103
7872	Lrrc16a	68732	13-:24104212-24233600
7873	Steap4	117167	5+:7960468-7982212
7874	Prl7a2	19114	13-:27750831-27759893
7875	Crat	12908	2-:30257505-30270980
7876	EG625534	625534	1+:152396446-152396808
7877	Obfc2a	109019	1-:51526333-51535251
7878	MMT00050705		16-:3550447-3550896
7879	Ifitm3	66141	7-:148195488-148196643
7880	Csf2	12981	11-:54061090-54063112
7881	Ccdc114	211535	7+:53195045-53204014
7882	Sprr2e	20759	3+:92156785-92157015
7883	Tyrp1	22178	4+:80480210-80497623
7884	Catsper3	76856	13+:55828729-55910358
7885	MMT00035027		1+:44268596-44269707
7886	Dock7	67299	4-:98603550-98612008
7887	4930418G15Rik	69312	1-:9958707-9999201
7888	Pigz	239827	16+:31934062-31946136
7889	Abcb1b	18669	5+:8798185-8866308
7890	Atp1b4	67821	X+:35669443-35689889
7891	Loxl2	94352	14+:70067073-70075657
7892	Ccr2	12772	9+:124016921-124023289
7893	Akr1c21	77337	13+:4573320-4585787
7894	MMT00029319	100039284	10+:61246511-61247569
7895	Tsc1	64930	2+:28496760-28542873
7896	Polr3e	26939	7+:128061297-128089335
7897	MMT00067427	100041449	5+:146890910-146924395
7898	Ctbp2	13017	7-:140179853-140217453
7899	2310079N02Rik	66566	8+:128258128-128272110
7900	Myo18a	360013	11+:77590766-77679482
7901	Slc41a1	98396	1+:133727198-133745441
7902	Ifng	15978	10+:117878099-117882947
7903	V1rc23	171196	6-:57850077-57850984

7904	Hoxb6	15414	11+:96160484-96162882
7905	Cox4nb	18117	8-:123181732-123192007
7906	Dll3	13389	7-:29078594-29086803
7907	Klk1b8	16624	7+:51206051-51210308
7908	MMT00082889		4-:127085611-127086063
7909	Oas3	246727	5-:121203108-121227668
7910	AK122209	382038	8+:126548955-126572397
7911	Pik3c2g	18705	6+:139792407-139917802
7912	Rnaseh2a	69724	8-:87481949-87491769
7913	MMT00065558		12+:9436283-9437290
7914	Defb2	13215	8+:22950397-22953951
7915	Cdx4	12592	X+:100516736-100525563
7916	MMT00016293		Y_random+:6427127-6429303
7917	Slfn3	20557	11+:83004831-83028655
7918	Rtn4ip1	170728	10+:43621898-43666734
7919	5330430P22Rik	68191	11-:105926395-105928158
7920	Mapk8	26419	14-:34195475-34224187
7921	Txnl4b	234723	8+:112089926-112097123
7922	Casp7	12369	19+:56471618-56516831
7923	MMT00055268		X+:21881404-22016460
7924	Xpc	22591	6-:91439833-91465789
7925	Nit1	27045	1-:173272374-173275757
7926	Kctd1	106931	18-:15127913-15147744
7927	Gata1	14460	X-:7536385-7544941
7928	Klhl34	245683	X+:154255960-154258817
7929	Pole4	66979	6-:82596705-82602857
7930	MMT00010123		7+:4860639-4862139
7931	1810014F10Rik	69064	7-:147285366-147288709
7932	Gkn2	66284	6+:87323358-87329488
7933	Ar	11835	X+:95345120-95512218
7934	MMT00002010		13-:3136659-3139392
7935	Mpv17	17527	5-:31443320-31456602
7936	MMT00061970		1-:12591242-12591281
7937	Efcab3	77627	15-:83697196-83766525
7938	Ppp2r2b	72930	18-:42804924-43219125
7939	Gpr37l1	171469	1-:137043383-137064195
7940	Cyp2b9	13094	7+:26958427-26995679
7941	Tob2	57259	15-:81681159-81682196
7942	Ttc4	72354	4-:106334599-106351512
7943	Rgs12	71729	5+:35292098-35382292
7944	AI132487	104910	12+:110089344-110095023
7945	Gpc2	71951	5-:138714888-138721195
7946	Ly6i	57248	15-:74810331-74813782
7947	Yif1a	68090	19+:5088005-5092855
7948	Sarm1	237868	11-:78288415-78310960
7949	Stub1	56424	17-:25967581-25969853
7950	Efha1	68514	14-:58535098-58618106
7951	Pck2	74551	14+:56159107-56168854
7952	MMT00081715		1-:9385643-9388802
7953	MMT00066281		11+:45643053-45643532
7954	Ccdc12	72654	9+:110559006-110614098

7955	Ambp	11699	4-:62804312-62815176
7956	Pnoc	18155	14-:66020316-66044310
7957	MMT00074126		13+:22374396-22375819
7958	Trim9	94090	12-:71346941-71448846
7959	Tbx5	21388	5+:120246786-120333511
7960	Zfp827	622675	8+:81552585-81714842
7961	Neurl	18011	19+:47253956-47332169
7962	Ajap1	230959	4-:152747328-152856813
7963	Rxrg	20183	1+:169528646-169569752
7964	AI894139	101197	6+:47901319-47912631
7965	Enpp5	83965	17+:44215817-44223508
7966	MMT00004995		14-:56894524-56896278
7967	Acsf2	264895	11-:94419279-94463104
7968	MMT00015383		17-:53441468-53443732
7969	MMT00001293		3+:8327474-8330440
7970	Olfr1294	258887	2-:111377507-111378444
7971	Prrx1	18933	1-:165178320-165242907
7972	Morc2b	240069	17-:33272673-33275741
7973	Lgals9	16859	11-:78776896-78798333
7974	Arhgef7	54126	8+:11782561-11835213
7975	Lancl1	14768	1-:67047090-67085439
7976	Anapc5	59008	5-:123241616-123250569
7977	Cgrrf1	68755	14+:47451893-47460087
7978	Gfpt1	14583	6+:87000913-87038150
7979	Prps1l1	75456	12+:35669559-35670514
7980	4933411K20Rik	66756	8+:47249083-47278200
7981	App	11820	16-:84955470-85173941
7982	MMT00060666		11-:76920772-76921727
7983	Syt13	80976	2+:92755331-92793824
7984	Cbfb	12400	8+:107694643-107741881
7985	MMT00011502		13+:25547971-25548296
7986	Zfp541	666528	7+:16663320-16681681
7987	2810048G17Rik	72691	19-:47206728-47212696
7988	Efcab5	319634	11-:76943354-76945718
7989	4930544D05Rik	114144	11+:70429351-70430391
7990	Nostrin	329416	2+:68973946-69027384
7991	Gpr115	78249	17-:42793837-42829236
7992	Rnf169	108937	7-:107074638-107091488
7993	BC025076	216829	11+:62462947-62478834
7994	Ipo11	76582	13-:107584526-107626382
7995	MMT00037028		12+:55723554-55724221
7996	Wbscr22	66138	5-:135528828-135540829
7997	Pex5l	58869	3-:32849533-32981951
7998	Gpr45	93690	1+:43089043-43090165
7999	Olfr547	259083	7+:109683262-109684195
8000	Ankrd17	81702	5-:90682739-90795590
8001	Efr3a	76740	15+:65618619-65704121
8002	Nfatc2ip	18020	7-:133527829-133540242
8003	Kpna4	16649	3-:68878010-68930722
8004	Fhod1	234686	8-:107813821-107871855
8005	Omg	18377	11-:79314857-79317521

8006	Abi1	11308	2-:22795888-22850121
8007	MMT00040041		8+:27408847-27464610
8008	Paf1	54624	7+:29177997-29184406
8009	Trat1	77647	16-:48733829-48750579
8010	Gpr133	243277	5+:129638568-129685571
8011	Pde11a	241489	2-:75829248-76176664
8012	Mab21l1	17116	3+:55586446-55588192
8013	Nipbl	71175	15-:8239829-8394471
8014	Bmx	12169	X-:160631157-160696111
8015	Tspan15	70423	10-:61650104-61693966
8016	Mrpl35	66223	6-:71765101-71773764
8017	Wdr24	268933	17+:25960598-25965669
8018	Treml1	71326	17+:48499238-48506499
8019	MMT00013162		11-:95038090-95067484
8020	Nope	56741	9+:64949341-64985749
8021	1600002K03Rik	69770	10+:79635687-79637864
8022	MMT00004959		X+:98257488-98258368
8023	MMT00059171		10+:102282010-102282530
8024	Ints9	210925	14+:65569108-65658665
8025	Agpat3	28169	10-:77736355-77758141
8026	MMT00000227	100041325	8-:95214187-95327748
8027	Wscd2	320916	5+:114000752-114038098
8028	MMT00007113		9-:110903868-110904461
8029	Rpap3	71919	15-:97505535-97536253
8030	Fgr	14191	4+:132530071-132556686
8031	Scoc	56367	8-:85958391-85966379
8032	Wap	22373	11-:6535491-6538652
8033	Ldlrad3	241576	2-:101898058-102026516
8034	Gas1	14451	13-:60275765-60278726
8035	Frzb	20378	2-:80252333-80287350
8036	Zfp160	224585	17+:21145937-21165823
8037	MMT00052151		1-:43055955-43077637
8038	MMT00037620		12-:81749624-81750359
8039	H1foo	171506	6+:115894956-115900249
8040	MMT00040904		
8041	Rab6ip1	19347	7-:117037289-117079067
8042	Smg7	226517	1-:154684126-154749777
8043	4930405D01Rik	73795	16+:52849397-52979019
8044	Sftpb	20388	6+:72254618-72264364
8045	Pgam5	72542	5-:110688179-110698898
8046	Scoc	56367	8-:85958391-85966379
8047	4930504E06Rik	75007	3+:95085295-95100089
8048	MMT00033146		X-:72719799-72720887
8049	Pon1	18979	6-:5118104-5143824
8050	Egln1	112405	8-:127432495-127438057
8051	MMT00035998		
8052	Krt17	16667	11-:100117532-100122303
8053	Gal	14419	19-:3410095-3414112
8054	Smarca2	67155	19+:26822948-26852763
8055	Ptcd3	69956	6-:71830634-71839625
8056	EG433923	433923	5+:97693755-97694741

8057	E130102H24Rik	77866	4-:101000723-101044149
8058	Epb4.9	13829	14-:71003509-71018209
8059	MMT00052729		6+:41152745-41178057
8060	EG668158	668158	12-:109512547-109513343
8061	Sfrs2ip	72193	15-:96242131-96277025
8062	Ppp1r12a	17931	10+:107599114-107717039
8063	4930471M23Rik	74919	5+:30950314-30962102
8064	Dusp27	240892	1-:167973486-168057310
8065	Athl1	212974	7+:148128434-148133555
8066	Sec24a	77371	11-:51510145-51569709
8067	Taok3	330177	5+:117583626-117723866
8068	Gm166	233899	7-:134726599-134731714
8069	MMT00015101		4+:100253888-100254336
8070	2010110P09Rik	70261	7+:129362984-129366521
8071	D630002J15Rik	77522	6+:38059245-38065807
8072	Zcchc9	69085	13-:91936137-91945766
8073	Irak1bp1	65099	9+:82723629-82740627
8074	Creb3	12913	4+:43575200-43580499
8075	MMT00011946		5-:73867838-73868525
8076	Rbm20	73713	19+:53925666-53939899
8077	Nr2c2	22026	6+:92042425-92119204
8078	Magea5	17141	X-:151487603-151496096
8079	Pts	19286	9-:50330120-50336746
8080	Lpar1	14745	4-:58447859-58566391
8081	Rhot1	59040	11+:80061973-80080313
8082	Lass4	67260	8+:4493452-4524064
8083	Mrpl14	68463	17+:45823353-45835429
8084	Psmd10	53380	X-:137483679-137491206
8085	MMT00057439		X-:136619365-136619999
8086	Olfr1495	258341	19+:13842833-13843792
8087	Lrrc8a	241296	2+:30093143-30117642
8088	Negr1	320840	3+:156679145-156823707
8089	Olfr390	258344	11+:73600441-73601376
8090	MMT00078696		18+:67722013-67722619
8091	Slc10a1	20493	12-:82056478-82068971
8092	Vat1	26949	11-:101321506-101321933
8093	MMT00023042		8+:19535501-19537741
8094	4930591A17Rik	68175	2+:179149640-179151585
8095	Bex2	12069	X-:132601104-132602649
8096	V1rh17	252912	13+:22179746-22180671
8097	Prss7	19146	16-:78953252-79091337
8098	Olfr1453	258695	19-:13101894-13102817
8099	MMT00052332		19+:58563727-58563925
8100	MMT00004628		6+:26780372-26781059
8101	Lce3b	66344	3+:92736900-92738018
8102	Hivep2	15273	10+:13847381-13870251
8103	Cry2	12953	2-:92245077-92274193
8104	Gja3	14611	14-:57654496-57655750
8105	Smyd4	319822	11+:75162037-75217042
8106	MMT00065672		14-:121214347-121214954
8107	Nlrp4b	210045	7+:11273464-11311322

8108	Cpne4	74020	9+:104775001-104936875
8109	6330545A04Rik	76166	4+:140769916-140775953
8110	Abhd7	384214	5+:107832606-107842646
8111	Matn1	17180	4+:130500338-130511390
8112	Pcm1	18536	8+:42325128-42417695
8113	Agtpbp1	67269	13-:59551261-59658647
8114	Kcnn1	84036	8-:73367056-73379484
8115	Mbnl3	171170	X-:48474134-48517806
8116	Arhgap19	71085	19-:41844074-41876540
8117	Ngp	18054	9+:110322352-110325514
8118	MMT00011146		12+:78920464-78921288
8119	B9d2	232987	7+:26466010-26471579
8120	5730410E15Rik	319613	15-:44503406-44560694
8121	Zfp451	98403	1-:33818388-33871415
8122	Hexim1	192231	11+:102977638-102981036
8123	Taf1	270627	X+:98728160-98796366
8124	Clec16a	74374	16+:10545430-10742556
8125	Taf9	108143	13+:101425005-101425800
8126	MMT00057246		X-:133638428-133638772
8127	Hmg20b	15353	10-:80808796-80813155
8128	Kcnmb2	72413	3+:31801595-32098687
8129	Gtse1	29870	15+:85690405-85707002
8130	MMT00064307		
8131	Btbd3	228662	2+:138104257-138110654
8132	MMT00016884		11-:53716222-53716657
8133	3110003A22Rik	68053	4+:6118254-6143706
8134	AW146020	330361	6+:81889372-81909249
8135	Agbl3	76223	6+:34730450-34764825
8136	Ikzf3	22780	11-:98327555-98380046
8137	Abhd12	76192	2-:150725406-150730573
8138	Epha3	13837	16-:63545797-63653404
8139	Tmem167	66074	13+:90229340-90246927
8140	4931406P16Rik	233103	7-:35021405-35070979
8141	Scnn1a	20276	6+:125271357-125294957
8142	E330011O21Rik	100126450	16+:78251108-78251848
8143	MMT00049078		X+:83516092-83564108
8144	Hoxa9	15405	6-:52174052-52177369
8145	Slc4a8	59033	15+:100592232-100646356
8146	Ankrd46	68839	15-:36407423-36426628
8147	1700029J07Rik	69479	8-:47038961-47060607
8148	Ppp1r9a	243725	6-:4942157-4961002
8149	Nav3	260315	10-:109121128-109207380
8150	Pnn	18949	12+:60167971-60174404
8151	Olfr1480	404339	19+:13587890-13605110
8152	Ccdc110	212392	8+:47020371-47028924
8153	Xirp1	22437	9-:119922873-119932687
8154	Neb	17996	2-:51992182-52194317
8155	Mark3	17169	12+:112813181-112894430
8156	Ttll1	319953	15-:83314201-83341294
8157	MMT00005305		6-:15477154-15588274
8158	Smc5	226026	19-:23281403-23317224

8159	Agpat7	99010	2+:112079411-112087267
8160	Akr1c12	622402	13-:4267422-4278606
8161	MMT00031928		16+:43312605-43313377
8162	Muc16	73732	9-:18337023-18371302
8163	MMT00041082		6-:130528251-130628277
8164	MMT00017950		6-:142919365-142919929
8165	Optc	269120	1-:135793776-135803749
8166	Trpt1	107328	19+:7070620-7073261
8167	Wipf2	68524	11+:98752013-98752926
8168	Slc31a2	20530	4+:61947480-61959445
8169	Akt1s1	67605	7+:52104596-52110778
8170	Snx22	382083	9-:65914914-65917545
8171	Spink8	78709	9+:109719158-109729137
8172	MMT00044495		2+:155881786-155882410
8173	Eif3eip	223691	15+:78905644-78924829
8174	5330426P16Rik	68190	16-:50719411-50732853
8175	Ggh	14590	4+:19969366-19993250
8176	Stx6	58244	1+:157006022-157049145
8177	6330439K17Rik	241688	2+:144297283-144298248
8178	Vtcn1	242122	3+:100629411-100692700
8179	Dnahc9	237806	11-:65803218-65982084
8180	Atp5g2	67942	15-:102493298-102501478
8181	Thap1	73754	8+:27268694-27274622
8182	Tbx1	21380	16-:18581805-18587062
8183	Ap3b2	11775	7-:88619279-88638541
8184	Rbm26	74213	14-:105505998-105576497
8185	Cst7	13011	2+:150396169-150404678
8186	Wsb2	59043	5+:117807469-117827561
8187	Gins1	69270	2+:150735322-150757012
8188	Kcnd1	16506	X+:7400968-7413628
8189	Cflar	12633	1+:58783206-58790070
8190	Rc3h2	319817	2-:37230577-37270266
8191	MMT00064416		5-:110909635-110910925
8192	MMT00082813		
8193	Pi4k2a	84095	19+:42164975-42196520
8194	MMT00077789		X-:153450772-153452073
8195	Pmfbp1	56523	8+:112017926-112066540
8196	Rps6ka5	73086	12-:101786652-101916799
8197	Lhfpl3	269629	5+:22252010-22284972
8198	1810036I24Rik	67510	11+:62693076-62708687
8199	Olfr512	258719	7+:115856868-115857848
8200	Dfna5h	54722	6-:50157402-50211768
8201	Klrg2	74253	6-:38576701-38587248
8202	Krtap3-3	66380	11-:99411445-99412177
8203	Rnf170	77733	8+:27234312-27253102
8204	Dmrtc1c	71083	X+:99998300-100005281
8205	Hdac9	79221	12-:35058652-35213169
8206	Phka2	110094	X+:156940155-157036801
8207	Cd44	12505	2-:102701526-102741641
8208	Trhr	22045	15+:44027778-44067458
8209	MMT00072546		X-:12903091-12904107

8210	Hspa12a	73442	19-:58873309-58936058
8211	4930570C03Rik	67739	15+:97614847-97622236
8212	MMT00052607		3-:77889848-77891186
8213	MMT00081182		7-:146729342-146730175
8214	Lmx1a	110648	1+:169619905-169776891
8215	Olfr140	57272	2-:89891571-89892479
8216	Oxsr1	108737	9-:119147563-119231489
8217	Bhmt	12116	13-:94387430-94407456
8218	Cldn22	75677	8+:48909882-48910543
8219	Nt5e	23959	9+:88222523-88265153
8220	MMT00056600		X-:12196514-12197417
8221	Stxbp3a	20912	3-:108596097-108643420
8222	Etfa	110842	9-:55302316-55359917
8223	Pdlim3	53318	8+:46970814-47004769
8224	Csn3	12994	5+:88354672-88361553
8225	Foxi1	14233	11-:34105509-34107942
8226	Gli1	14632	10-:126766942-126778635
8227	MMT00033394		5-:66302818-66326785
8228	1600029I14Rik	69797	9+:99360108-99371345
8229	Myo1d	338367	11-:80295628-80593505
8230	Olfr56	18356	11+:48943130-48948642
8231	Tcfcp2	21422	15-:100333227-100382378
8232	Olfr707	194433	7-:114034697-114035705
8233	Kif2b	73470	11-:91436763-91438769
8234	Tbpl1	237336	10-:22425181-22451133
8235	Tcfeb	21425	17-:47874043-47888510
8236	Nov	18133	15-:54583815-54936256
8237	Caprin1	53872	2-:103605181-103637211
8238	MMT00004291		4+:35383648-35421149
8239	Gzmn	245839	14-:56784742-56786807
8240	Polr2i	69920	7+:31016818-31018138
8241	MMT00047047		2-:99394281-99564304
8242	Irak3	73914	10-:119579771-119638573
8243	Ccdc78	381077	17+:25924322-25926024
8244	Vamp4	53330	1+:164504431-164525021
8245	Tmprss8	30943	17-:24109094-24112160
8246	MMT00081738		1+:9419117-9441469
8247	Fchsd1	319262	18-:38120896-38129106
8248	Prom2	192212	2-:127354176-127367203
8249	Cyb5r4	266690	9+:86916882-86972599
8250	MMT00007883		8+:41101843-41102200
8251	1700082M22Rik	73534	8+:107182185-107182347
8252	Plcl1	227120	1+:55752583-55808444
8253	1700041B20Rik	73338	2-:126966294-126967936
8254	Bbs2	67378	8-:96591853-96622323
8255	Prkar1a	19084	11+:109510676-109530943
8256	Tjap1	74094	17-:46394824-46419958
8257	Aadac	67758	3+:59835709-59844079
8258	MMT00022030		5-:42244547-42245449
8259	Cd63	12512	10+:128346977-128349872
8260	MMT00035935		1+:19322206-19323172

8261	Hormad1	67981	3+:95363598-95391593
8262	Syp	20977	X+:7215836-7230377
8263	Igk-V28	16114	6-:69714491-69714986
8264	6430598A04Rik	243300	5-:138172910-138180946
8265	Rps11	27207	7-:52377867-52378923
8266	Prosc	114863	8+:28153086-28163885
8267	Csn2	12991	5-:88121648-88128446
8268	Kctd7	212919	5+:130620833-130630984
8269	Ccdc11	74453	18+:74442775-74519638
8270	Clspn	269582	4+:126234236-126271157
8271	Adra2a	11551	19+:54120749-54122101
8272	Ppfia2	327814	10+:106180542-106350848
8273	Olfr1013	258757	2+:85609959-85610875
8274	Zc3h11a	70579	1-:135518092-135550589
8275	Olfr361	258365	2-:36940298-36941266
8276	Taldo1	21351	7+:148578061-148588866
8277	Ccdc53	67282	10+:87663839-87708903
8278	Dlgap1	224997	17+:71158411-71168741
8279	Kif13a	16553	13-:46844458-47025087
8280	Tnpo3	320938	6-:29490888-29559888
8281	Ninj1	18081	13+:49282915-49291620
8282	Ccnt2	72949	1+:129670784-129703477
8283	Ihpk1	27399	9+:107926558-107948303
8284	EG209380	209380	7-:113563185-113566144
8285	Rgs13	246709	1-:145986631-146024499
8286	MMT00075001		15-:60126524-60128520
8287	MMT00061960		3-:33583147-33583747
8288	Pou2f2	18987	7-:25877674-25917479
8289	Cacna1d	12289	14-:30852866-31010049
8290	Galr1	14427	18-:82562793-82576193
8291	Lce1a2	73722	3-:92472534-92474237
8292	Mapkap1	227743	2+:34479448-34480469
8293	Mpi	110119	9-:57392066-57400559
8294	Fv1	14349	4+:147243087-147244398
8295	Hpca	15444	4-:128789672-128795985
8296	Ammecr1l	225339	18+:31921276-31943734
8297	Dhx37	208144	5-:125896235-125977332
8298	Car10	72605	11+:92960629-93047277
8299	MMT00003438		17+:54141661-54142787
8300	MMT00043905		15-:74816454-74817477
8301	Rab34	19376	11+:78002838-78005342
8302	Cryzl1	66609	16-:91689704-91728844
8303	Acn9	71238	6+:6906006-6989662
8304	Fubp1	51886	3+:151873461-151895735
8305	Popdc2	64082	16+:38362337-38378300
8306	Slc13a3	114644	2-:165230795-165298689
8307	Lhx2	16870	2+:38207104-38225108
8308	Tdpoz2	399673	3-:93432586-93456573
8309	Rpl17	319195	18+:75160160-75168482
8310	Atad3a	108888	4-:155115272-155135179
8311	Pla2g5	18784	4-:138356456-138362139

8312	Sebox	18292	11+:78317051-78317906
8313	Frap1	56717	4+:147822715-147931790
8314	Armc6	76813	8-:72744089-72758367
8315	Phactr2	215789	10-:12933209-13043929
8316	Depdc7	211896	2-:104561945-104582958
8317	E130311K13Rik	329659	3-:63724086-63733292
8318	E130308A19Rik	230259	4+:59731495-59733554
8319	MMT00062477		10-:14465758-14467192
8320	Nme3	79059	17+:25033450-25034448
8321	Olfr1120	259031	2+:87197602-87198547
8322	Dpy19l1	244745	9-:24216219-24289576
8323	Cbx6	494448	15-:79654328-79656137
8324	Hemt1	15202	15+:74653494-74654866
8325	Psme2	19188	14-:56206276-56209745
8326	Mageh1	75625	X-:149471224-149471881
8327	4930503B20Rik	75015	3-:146312663-146314276
8328	Klhdc8a	213417	1+:134195637-134202152
8329	Pipox	19193	11-:77694116-77707283
8330	MMT00027613	634100	12-:116067651-116175628
8331	BC052688	432812	13+:61731372-61888643
8332	Tmem206	66950	1+:193149843-193174790
8333	Klhdc2	69554	12+:70397754-70411451
8334	MMT00082897		6-:73478635-73479239
8335	Cftr	12638	6+:18120686-18272767
8336	MMT00041615		5+:152083838-152084048
8337	Lgr4	107515	2+:109810843-109853629
8338	Atg10	66795	13-:91074953-91363573
8339	MMT00046482		14+:60759849-60760245
8340	Pde1a	18573	2-:79677036-79969269
8341	MMT00017270		6-:47926988-47927875
8342	Cwf19l1	72502	19-:44184514-44210366
8343	Sult1b1	56362	5-:87943740-87967190
8344	Cfh	12628	1-:141982455-142079899
8345	Elfn1	243312	5+:140447196-140449682
8346	Hiat1	15247	3-:116334086-116365468
8347	Efhc2	74405	X-:16709180-16740022
8348	Neil2	382913	14-:63802089-63810679
8349	Rhoq	104215	17+:87362641-87399403
8350	D8Ertd738e	101966	8-:86770129-86773749
8351	Zp3r	22789	1-:132473289-132526173
8352	4930512M02Rik	75110	11-:11489225-11524715
8353	Chd5	269610	4+:151715692-151761333
8354	EG547412	547412	X+:72388539-72389809
8355	Pms2	18861	5+:144737915-144746584
8356	Fbxl12	30843	9-:20442232-20447132
8357	Siva1	30954	12+:113883122-113887355
8358	Zpbp2	69376	11+:98412431-98419977
8359	1190002N15Rik	68861	9-:94418281-94438274
8360	Bcl6	12053	16-:23966201-23988685
8361	MMT00025562		9+:18231928-18233469
8362	Fbxw8	231672	5-:118516505-118605412

8363	MMT00006564		19-:28936820-28938254
8364	4930435M08Rik	74651	14+:118444194-118446085
8365	Nfatc4	73181	14+:56443736-56452780
8366	Sema3a	20346	5+:13399847-13600084
8367	Ccdc131	216345	10+:114845930-114869824
8368	Rad51l3	19364	11-:82691886-82704107
8369	Prkaca	18747	8+:86497085-86519316
8370	Sigirr	24058	7-:148277074-148280801
8371	Hipk2	15258	6-:38647904-38825952
8372	Uap1	107652	1-:172072133-172105011
8373	Cdc7	12545	5+:107393340-107413450
8374	Copz1	56447	15+:103103348-103130293
8375	Ankrd2	56642	19+:42110527-42119600
8376	Pdcd2	18567	17-:15658541-15664245
8377	Fgd5	232237	6+:92021180-92026008
8378	Nr2c1	22025	10+:93619512-93658436
8379	2610024G14Rik	56412	5-:77723280-77739083
8380	Gnb2l1	14694	11+:48613869-48619581
8381	Sncg	20618	14-:35183460-35187813
8382	MMT00012541		X-:129628360-129629513
8383	Foxd2	17301	4-:114579897-114581493
8384	Cst9	13013	2+:148660882-148664471
8385	Olfr1061	259022	2-:86253265-86254207
8386	Slc17a8	216227	10-:89040645-89069613
8387	Tcl1b5	27382	12+:106414567-106419353
8388	Zfyve21	68520	12+:113052483-113066560
8389	Pbsn	54192	X-:75083237-75098839
8390	Ttc16	338348	2-:32627626-32630859
8391	Eif1	20918	11+:100181408-100182673
8392	Asf1b	66929	8+:86479592-86494093
8393	Wdr51a	70235	9+:106183440-106252217
8394	Dpy19l3	233115	7-:36473861-36497237
8395	Alg1	208211	16+:5233730-5244989
8396	MMT00040956		18+:10928840-10929732
8397	Arhgef18	102098	8+:3455738-3456600
8398	ND5	17721	M+:0-16297
8399	Herpud1	64209	8+:96910399-96919258
8400	Calm4	80796	13+:3837141-3837576
8401	Slain1	105439	14+:104049416-104104122
8402	Ttll13	269954	7+:87397297-87404074
8403	Rps29	20090	12-:70258711-70260057
8404	Fa2h	338521	8-:113869045-113917720
8405	Slc26a10	216441	10-:126609367-126617701
8406	Ikbkap	230233	4-:56762552-56815207
8407	V1rc17	171190	6-:57958178-57959196
8408	Nr1i3	12355	1+:173144166-173148975
8409	Mid1	17318	X+:166123181-166444500
8410	EG434050	434050	6+:78165871-78167106
8411	Phxr2	18687	10-:98587807-98588959
8412	Tle4	21888	19-:14522561-14524034
8413	Olfr1447	258698	19-:12975340-12976268

8414	Calm3	12315	7-:17502141-17509306
8415	Cyb561	13056	11-:105796687-105805477
8416	Cdc20	107995	4-:118105506-118109854
8417	A430084P05Rik	327957	11-:70604963-70626089
8418	MMT00006138		1-:110894603-110895383
8419	AU042671	269700	5+:121709813-121723808
8420	Nos1	18125	5+:118317127-118403820
8421	Mbd5	109241	2+:49101451-49169245
8422	Adam3	11497	8-:25787704-25836297
8423	Nhedc1	74446	3+:135011021-135056046
8424	Ap2m1	11773	16+:20535576-20543979
8425	0610011L14Rik	68295	2+:156373331-156393892
8426	MMT00068366	100041688	8+:22204744-22204911
8427	Mybpc3	17868	2+:90958326-90976666
8428	Sorbs3	20410	14-:70580275-70607444
8429	Cdyl2	75796	8-:119098389-119256744
8430	Pttg1ip	108705	10+:77044510-77061476
8431	Hdac2	15182	10+:36694389-36721694
8432	Fgf18	14172	11-:33017438-33047400
8433	Gm364	245423	X+:54670835-54692859
8434	Acacb	100705	5+:114615601-114699738
8435	3110047P20Rik	319807	5+:64182682-64201486
8436	Dnajb8	56691	6+:88172261-88173250
8437	Olfr1090	258844	2-:86593951-86594893
8438	Mrpl48	52443	7-:107697222-107756816
8439	Slc25a33	70556	4-:149118583-149148292
8440	MMT00063259		7-:60335153-60945083
8441	Tmem54	66260	4+:128782859-128788829
8442	Med18	67219	4-:132015188-132019836
8443	Pola2	18969	19-:5940547-5964139
8444	Rdh16	19683	10+:127238209-127252895
8445	Tmtc1	387314	6-:148233377-148374506
8446	Cybrd1	73649	2+:70956108-70976909
8447	Nup107	103468	10-:117187558-117229835
8448	Sucla2	20916	14+:73952436-73995949
8449	Vash2	226841	1-:192771628-192803078
8450	Capza1	12340	3-:104627903-104667296
8451	Nr1h2	22260	7-:51805010-51809293
8452	Qrich1	69232	9+:108419952-108462497
8453	MMT00060234		13-:62301099-62301830
8454	Lsm5	66373	6-:56651058-56654687
8455	MMT00008022		14+:110955659-110956508
8456	Ftl2	14337	4+:125180618-125181709
8457	MMT00045470		
8458	MMT00063985		X-:57979510-57980693
8459	Gpatch3	242691	4+:133130674-133136626
8460	Gle1	74412	2+:29790943-29815156
8461	Gpr103	229214	3-:36078346-36121210
8462	Pkd1l1	171395	11-:8726647-8734803
8463	EG640554	640554	9+:54902640-54902891
8464	Snapin	20615	3-:90292300-90294915

8465	BC023814	229503	3-:87727566-87734069
8466	Pkhd1l1	192190	15+:44289098-44361649
8467	Scfd1	76983	12+:52478565-52551094
8468	Hrasls5	66727	19+:7687058-7713728
8469	Sfrs6	67996	2+:162757263-162761082
8470	Psors1c2	57390	17+:35670213-35671590
8471	Tacstd1	17075	17+:88036047-88050467
8472	Txndc16	70561	14-:45754125-45839043
8473	Clint1	216705	11+:45665443-45724128
8474	Tcl1b2	27381	12+:106385242-106393433
8475	Cirh1a	21771	8+:109417548-109446992
8476	Zbtb3	75291	19+:8876982-8879634
8477	MMT00002146	100041835	11-:95934335-95936342
8478	Clec2l	665180	6+:38613235-38630842
8479	MMT00067202		12+:108786602-109114240
8480	MMT00008725		3-:12893012-12893353
8481	Sytl5	236643	X+:9482643-9570223
8482	Trip12	14897	1-:84748272-84750993
8483	Slc25a5	11740	X+:34335725-34338537
8484	Cgref1	68567	5-:31235514-31247859
8485	Lrrk2	66725	15+:91559373-91564538
8486	Plod3	26433	5+:137463486-137472407
8487	Olfr1039	257950	2-:85970858-85971818
8488	Opcml	330908	9+:28620871-28727953
8489	Prdx3	11757	19-:60939989-60950417
8490	Glcci1	170772	6+:8209426-8229555
8491	Elovl4	83603	9-:83672306-83699818
8492	EG114600	114600	6-:132064239-132314153
8493	Gucy1a2	234889	9+:3556726-3643888
8494	MMT00028462		6+:67789786-67864069
8495	Lox	16948	18-:52675720-52689357
8496	1190005P17Rik	66225	10+:119665036-119668428
8497	MMT00069762		
8498	2010317E24Rik	72080	2+:25227856-25233001
8499	C430048L16Rik	77604	4+:12016518-12073876
8500	Ddit3	13198	10+:126730766-126733340
8501	Ndufaf1	69702	2-:119481186-119488534
8502	Olfr866	258551	9-:19831443-19832380
8503	Olfr374	258335	8+:74633466-74634408
8504	Tex10	269536	4-:48443272-48486397
8505	Hif1an	319594	19+:44637367-44644827
8506	MMT00003599		4+:16173206-16177382
8507	1700054F22Rik	73398	4+:53006846-53008858
8508	Myl3	17897	9+:110666184-110672304
8509	E2f5	13559	3+:14578804-14606039
8510	Tbl2	27368	5+:135625593-135636005
8511	Folr4	64931	9-:14704477-14708376
8512	Nr3c2	110784	8+:79398416-79434302
8513	Wbscr27	79565	5+:135408263-135417101
8514	Arntl2	272322	6+:146754950-146782043
8515	Glcci1	170772	6+:8209449-8547480

8516	Plxnb2	140570	15-:88985978-88997478
8517	1-Mar	72925	8+:68910191-68992803
8518	EG236844	236844	X-:65569128-65569492
8519	Gabrd	14403	4-:154759088-154772171
8520	Rdh10	98711	1-:16094708-16095860
8521	Ceacam1	26365	7-:26248486-26262569
8522	Impg2	224224	16+:56205188-56271255
8523	Ddit4l	73284	3+:137286635-137289477
8524	Cbr1	12408	16+:93608083-93610566
8525	2510006D16Rik	76799	4+:129277950-129285114
8526	Cend1	57754	7-:148612347-148615248
8527	Zc3h12a	230738	4-:124796524-124805071
8528	EG545893	545893	6-:147022693-147035521
8529	MMT00070318		14+:44518941-44519459
8530	Snca	20617	6-:60681594-60779849
8531	4930401A09Rik	75777	15-:10429897-10488405
8532	MMT00045840		16+:31377527-31379189
8533	Ptprcap	19265	19+:4154678-4156713
8534	Lhcgr	16867	17-:89140889-89191316
8535	Grm7	108073	6+:111016741-111030476
8536	Mesp2	17293	7+:86955621-86958316
8537	Tmem179	104885	12-:113739953-113749370
8538	Nup62cl	279706	X-:136562676-136573694
8539	Cflar	12633	1+:58783206-58790070
8540	Slc4a1	20533	11-:102210140-102226429
8541	Tubb5	22154	17-:35971751-35975076
8542	Il1rl2	107527	1+:40381474-40422313
8543	Olfr686	259072	7-:112351902-112352855
8544	Znhit3	448850	11-:84724019-84729870
8545	Spink12	78242	18+:44264061-44268195
8546	Vmn2r87	625131	10-:129908893-129934362
8547	Gbp4	17472	5-:105546592-105568563
8548	Tor1aip2	240832	1+:157906683-157912587
8549	Ndufb7	66916	8+:86090656-86095522
8550	Myh13	544791	11+:67140604-67185003
8551	1700113O17Rik	68231	2-:17918048-17918573
8552	Stx19	68159	16+:62814500-62822553
8553	Ftmt	67634	18+:52491189-52492439
8554	Letm2	270035	8-:26688963-26708024
8555	H2-M3	14991	17+:37407206-37411431
8556	MMT00005777	100040391	18-:14465663-14537967
8557	MMT00035973		
8558	Shroom4	208431	X+:6097910-6210833
8559	Rangrf	57785	11-:68785987-68787327
8560	MMT00026471		13+:97146821-97147658
8561	Mds1	17251	3-:30135490-30408409
8562	4921509B22Rik	70859	14-:75484111-75529442
8563	Leng1	69757	7-:3612684-3617072
8564	Pld4	104759	12+:114001601-114007198
8565	MMT00008541		2-:144190097-144191387
8566	MMT00003532		4+:32237002-32262310

8567	MMT00058919	242025	3+:53825085-53825724
8568	Tnik	665113	3+:28308275-28569505
8569	MMT00019147		17+:26038752-26039122
8570	Ppyr1	19065	14-:34959387-34960515
8571	Uchl3	50933	14+:102053273-102095316
8572	Olfr57	18357	10+:78497683-78498136
8573	MMT00003887		6+:41068862-41069443
8574	Top1mt	72960	15-:75487464-75509199
8575	Vmn2r118	383258	17-:55747285-55763906
8576	MMT00002368		8-:63073373-63073526
8577	Stox2	71069	8+:48265405-48266707
8578	Wdr19	213081	5+:65590979-65651651
8579	Rngtt	24018	4+:33397404-33589583
8580	Eps15	13858	4+:108952907-109058299
8581	MMT00031271		2+:30014910-30016998
8582	Igsf10	242050	3-:59121809-59140509
8583	Atp5c1	11949	2-:9977660-10002056
8584	Zmynd11	66505	13-:9685017-9764377
8585	Gnptab	432486	10-:87907830-87913900
8586	MMT00030651		X+:72190268-72195309
8587	Napepld	242864	5-:21170734-21207242
8588	Notch4	18132	17+:34701274-34725441
8589	Sfrs7	225027	17-:80599425-80606625
8590	MMT00078103	244958	9+:87064359-87077665
8591	Il31ra	218624	13-:113313013-113370870
8592	Olfr510	258308	7+:115810931-115811874
8593	1200003C05Rik	104771	12+:73187109-73201827
8594	Myt1	17932	2+:181531011-181562437
8595	MMT00019109		X-:88531214-88531963
8596	Sf3b3	101943	8-:113334391-113370660
8597	Papss1	23971	3+:131227854-131306632
8598	Il24	93672	1-:132778651-132782573
8599	Abl1	11350	2+:31614980-31659058
8600	Scn10a	20264	9-:119517584-119603478
8601	Rps5	20103	7+:13507683-13512034
8602	Scd3	30049	19+:44277788-44316999
8603	Slc6a3	13162	13+:73674271-73716118
8604	V1rh13	171272	13+:23254511-23255448
8605	1700054F22Rik	73398	4+:53010025-53013718
8606	Cntnap5c	620292	17+:58153238-58445139
8607	Elp2	58523	18+:24762495-24797311
8608	Hspg2	15530	4+:137074547-137089650
8609	Zik1	22775	7-:11073719-11080728
8610	Padi4	18602	4-:140301779-140330098
8611	Spred3	101809	7-:29946578-29952992
8612	MMT00020019		11+:23913947-23977306
8613	Pin4	69713	X+:99314771-99323013
8614	Bpil3	228796	2+:153734286-153737258
8615	MMT00010050		10+:123633196-123656241
8616	A730006G06Rik	320638	17-:48202901-48227326
8617	2210412E05Rik	75690	7+:50718602-50719173

8618	EG433865	433865	5-:30120704-30121666
8619	MMT00077170		X+:164193196-164215001
8620	MMT00048670		1+:146800131-146801383
8621	MMT00047655		11-:102520442-102521181
8622	Fuk	234730	8-:113413575-113426357
8623	MMT00015399		17-:53606287-53606659
8624	Bnc1	12173	7-:89111546-89137182
8625	Gsg1	14840	6-:135187349-135204233
8626	Dph4	99349	2-:105806873-105843706
8627	Nxf2	83454	X-:131479069-131499293
8628	MMT00058602		X+:46064705-46065318
8629	Hhat	226861	1-:194376820-194597407
8630	AI413194	100620	5+:137581514-137581949
8631	9930032O22Rik	320454	5-:86916423-86921315
8632	Igfbp1	16006	11+:7097869-7102540
8633	Rfwd3	234736	8-:113817890-113824114
8634	MMT00047654	100041686	8-:73419313-73421275
8635	B4galnt3	330406	6-:120166573-120244577
8636	Cyp2j11	100066	4-:95961214-96015372
8637	Tnpo2	212999	8+:87561130-87579566
8638	Fkbp14	231997	6-:54527600-54543171
8639	Map1lc3b	67443	8+:124114424-124120668
8640	Wfdc6b	433502	2+:164440390-164542664
8641	Slc7a2	11988	8+:41983791-42003073
8642	Serpinb9	20723	13+:33096539-33108356
8643	Mmp1b	83996	9-:7368240-7387993
8644	V1rg11	171267	7-:12947158-12948113
8645	Tcl1b4	27380	12+:106440631-106445201
8646	Plau	18792	14+:21656300-21661671
8647	Gpr137b-ps	664862	13-:12707324-12723020
8648	Flnb	286940	14+:8650664-8784097
8649	MMT00072863	77652	17+:33437886-33441602
8650	Gpi1	14751	7-:34992623-35015310
8651	Ccdc61	232933	7-:19476232-19495813
8652	Krt7	110310	15+:101242855-101258235
8653	AW124722	239570	15+:85662742-85687226
8654	Fbxl13	320118	5-:21119810-21120312
8655	MMT00020731	331000	9+:92100279-92102795
8656	Ankle2	71782	5+:110660255-110684379
8657	MMT00032815		18-:9748691-9749923
8658	Fmo2	55990	1-:164806576-164828517
8659	Psmb8	16913	17+:34335343-34338210
8660	Rragb	245670	X+:149574538-149604845
8661	Grasp	56149	15+:101054674-101063186
8662	Brca2	12190	5+:151325749-151372321
8663	Olfr874	258882	9+:37553720-37554651
8664	MMT00014895		8+:125113580-125114085
8665	EG667977	667977	17-:36274132-36305471
8666	Cerk	223753	15-:85970772-86016078
8667	Plxna3	18846	X+:71574439-71589102
8668	Atxn7	246103	14+:14845586-14936261

8669	3110035E14Rik	76982	1+:9591269-9614975
8670	MMT00051613		1+:46472135-46473775
8671	MMT00028357		8+:13086535-13087234
8672	Tmem26	327766	10+:68213855-68241605
8673	MMT00007012		12-:120305214-120305879
8674	Gclm	14630	3+:121948863-121969397
8675	Cacng1	12299	11-:107564977-107577836
8676	MMT00030667		2+:18680603-18681551
8677	Zbtb8os	67106	4+:129013260-129031791
8678	Myl1	17901	1-:66972786-66978523
8679	Tiparp	99929	3+:65332331-65359440
8680	C1qtnf9	239126	14+:61391133-61399701
8681	Rab9	56382	X-:162895191-162917622
8682	A930003A15Rik	68162	16-:19876662-19884342
8683	3110002H16Rik	76482	18+:12327243-12349336
8684	Il15	16168	8-:84855524-84926444
8685	Klrc1	16641	6-:129624877-129628928
8686	Nkx2-5	18091	17-:26975608-26978554
8687	AB041803	232685	6-:31115524-31168433
8688	Suv39h1	20937	X-:7638429-7651712
8689	Ngdn	68966	14+:55634362-55635984
8690	Cd84	12523	1+:173770734-173816561
8691	Il17c	234836	8+:124945984-124947467
8692	Rhbdd3	279766	11+:4998316-5006094
8693	C130034I18Rik	320701	6-:96793741-96813909
8694	Npdc1	18146	2+:25258765-25265013
8695	Sh3tc2	225608	18+:62112729-62134257
8696	Tmem47	192216	X+:78316049-78341230
8697	Dpysl2	12934	14-:67424007-67487227
8698	1500009L16Rik	69784	10+:83185640-83225505
8699	Gzmm	16904	10+:79152155-79157995
8700	OTTMUSG00000(	620913	11-:48720630-48729864
8701	EG633640	633640	13+:120276876-120284312
8702	Mrpl30	107734	1+:37947397-37955178
8703	Gstp1	14870	19-:4035410-4037912
8704	4933411K16Rik	66765	19+:42126737-42128118
8705	Ahdc1	230793	4+:132621123-132633140
8706	Rab35	77407	5+:116082149-116097568
8707	AI747699	381236	19-:33657536-33664514
8708	Olfr992	258865	2-:85239758-85240688
8709	Trim58	216781	11+:58453969-58459628
8710	Cnnm3	94218	1+:36569651-36582526
8711	5430432M24Rik	73847	2-:151795135-151805865
8712	Olfr726	258313	14-:50703389-50704354
8713	Lrrc43	381741	5+:123939338-123958213
8714	Pcdh20	219257	14-:88867026-88870808
8715	Ntrk1	18211	3-:87582166-87599066
8716	Slc22a17	59049	14-:55525568-55531877
8717	Litaf	56722	16-:10959366-10993182
8718	Socs3	12702	11-:117827402-117829561
8719	Nup214	227720	2+:31830011-31907892

8720	Ssr1	107513	13-:38069961-38086004
8721	Tbc1d10c	108995	19-:4184355-4191049
8722	MMT00022745		X+:109579839-109580241
8723	Oxsm	71147	14-:17073138-17081692
8724	Tgfb3	21809	12-:87397693-87419484
8725	Fance	72775	17+:28450510-28463511
8726	Olfr46	18345	7+:147796066-147797021
8727	Slc27a3	26568	3-:90189153-90193712
8728	4930438A08Rik	73988	11+:58099996-58107798
8729	MMT00006406		6-:69413179-69450640
8730	MMT00008620		8+:54766414-55042434
8731	Tmpo	21917	10-:90624502-90634280
8732	Lce1f	67828	3-:92522606-92524272
8733	Olfr1247	258968	2-:89449313-89450257
8734	Etv5	104156	16-:22383086-22436564
8735	2210408I21Rik	72371	13+:77406949-77473638
8736	Rsbn1	229675	3+:103718307-103770783
8737	Serpina10	217847	12-:104854886-104869631
8738	Reg4	67709	3+:98026078-98040671
8739	MMT00054598		7-:93206490-93211909
8740	Car5b	56078	X-:160417022-160465929
8741	Speer9-ps1	73949	7+:3097628-3150637
8742	Tmem39a	67846	16+:38562953-38591497
8743	Pkp4	227937	2+:58999877-59193260
8744	Bcas1	76960	2-:170172526-170253346
8745	Mcm2	17216	6-:88833468-88848691
8746	MMT00018815		14+:15491999-15492518
8747	Nme6	54369	9+:109735307-109745475
8748	EG625055	625055	3+:86668402-86669324
8749	Wdfy3	72145	5-:102364099-102386564
8750	1700010I14Rik	66931	17+:9181225-9201181
8751	Tex264	21767	9-:106561077-106588258
8752	Stk32c	57740	7-:146289537-146374372
8753	MMT00015209	434186	7+:58425886-58431820
8754	Prl2c3	18812	13-:12884253-12892347
8755	Ppm1k	243382	6-:57463934-57475248
8756	Casq2	12373	3+:101890567-101950172
8757	Igh-VJ558	16061	12-:117190526-117191230
8758	0610007P14Rik	58520	12-:87156407-87165495
8759	F8	14069	X-:72418068-72625380
8760	Tpcn1	252972	5-:120986107-121038658
8761	Sit1	54390	4-:43494954-43496557
8762	Lca5	75782	9-:83284961-83334705
8763	Trpv4	63873	5-:115072163-115108396
8764	Mylk3	213435	8-:87848351-87876829
8765	MMT00037007		
8766	Irs3	16369	5-:138084255-138086955
8767	Rgl2	19732	17+:34066910-34074340
8768	Slc25a2	83885	18-:37734819-37798128
8769	Cenpk	60411	13+:105019467-105039695
8770	Cpxm2	55987	7-:139235576-139346296

8771	Foxr1	382074	9-:44241864-44243735
8772	MMT00042479		X-:65572813-65573103
8773	MMT00027093		14-:93593417-93594940
8774	Klf12	16597	14-:100273195-100548990
8775	Topbp1	235559	9+:103208426-103252755
8776	Aox3	71724	1+:58169979-58257289
8777	1700028K03Rik	76421	5+:107963728-107977396
8778	Rnpepl1	108657	1+:94807645-94817416
8779	Opn5	353344	17-:42713186-42733682
8780	Ndufb3	66495	1+:58643442-58652792
8781	Acpp	56318	9-:104202630-104239998
8782	ENSMUSG00000(	791319	3-:55181377-55195964
8783	Zfyve16	218441	13-:93257085-93300754
8784	Hgd	15233	16+:37580306-37632104
8785	Tbx15	21384	3+:99057793-99158182
8786	Rmnd1	66084	10+:5942553-5943313
8787	Sdf2l1	64136	16-:17130234-17132416
8788	Olfr53	258962	7+:147837879-147838816
8789	MMT00059186		10+:101922990-101923547
8790	Galnt2	108148	8+:126755425-126867361
8791	Sectm1a	209588	11-:120928716-120942420
8792	Bop1	12181	15-:76283425-76307699
8793	EG621100	621100	11+:101304038-101306831
8794	1500011B03Rik	66236	5-:115258209-115263936
8795	EG629966	629966	11-:111474281-111542366
8796	Pgr	18667	9+:8900460-8965153
8797	Snrk	20623	9+:122041019-122078816
8798	Tacr2	21337	10+:61715185-61728035
8799	Ccrl2	54199	9-:110957338-110959703
8800	Rnf213	629974	11+:119313824-119348731
8801	Nlrp10	244202	7-:116067325-116073665
8802	Brd1	223770	15-:88517464-88564662
8803	MMT00080648		10+:56217067-56217903
8804	Usp46	69727	5-:74397825-74464442
8805	Glt6d1	71103	2-:25639458-25651123
8806	Olfr127	258374	17+:38040492-38041462
8807	Ankrd56	78088	5-:93470154-93473980
8808	RP23-136K12.4	19062	11+:75444489-75462373
8809	Tgfbr2	21813	9-:115996815-115998095
8810	ENSMUSG00000(	378466	8-:71894781-71897808
8811	MMT00036449		3-:139779672-139780881
8812	Zfp637	232337	6+:117784463-117795789
8813	5430413K10Rik	433492	2+:154132916-154137239
8814	Kera	16545	10+:97069842-97076321
8815	Mx1	17857	16-:97668643-97684512
8816	Nfkbia	18035	12-:56590847-56593570
8817	Ptpn1	19246	2+:167757865-167802467
8818	MMT00005552		14+:16742939-16746038
8819	2610204K14Rik	67148	7+:88907838-88914376
8820	Cd200	17470	16-:45392358-45400416
8821	Ank1	11733	8+:24168746-24260968

8822	Galntl4	233733	7-:118922826-118923004
8823	Smarcc2	68094	10-:127926395-127927193
8824	Slc25a18	71803	6+:120734387-120743659
8825	4930455F23Rik	74895	1+:166205727-166217975
8826	4933406C10Rik	74076	12-:33609822-33638650
8827	Nudt15	214254	14-:73923099-73948054
8828	Slc30a2	230810	4+:133899201-133908442
8829	Kndc1	76484	7+:147113581-147127435
8830	MMT00081195		1-:7126278-7126658
8831	Ppp1r3e	105651	14-:55494434-55496289
8832	Tspyl3	241732	2-:153050049-153051016
8833	8030451F13Rik	109272	10-:87981029-88067768
8834	Lrrc4b	272381	7+:51712458-51718685
8835	MMT00019528		3-:93961560-93962631
8836	Zmym5	219105	14-:57412164-57416618
8837	MMT00026538	544896	12-:114811134-115097665
8838	1700121N20Rik	76639	12-:107680863-107685878
8839	MMT00002660		6-:44744424-44744757
8840	Tmem18	211986	12+:31269324-31276083
8841	Kif17	16559	4+:137818417-137857341
8842	Snf1lk2	235344	9-:50700904-50817178
8843	Ell2	192657	13+:75899385-75909666
8844	2310037I24Rik	69612	15-:98349240-98364664
8845	9430028L06Rik	225617	18-:63169868-63267784
8846	P2ry2	18442	7-:108145784-108160488
8847	Cpe	12876	8-:67071362-67076365
8848	Tes3-ps	54339	13+:49589776-49590796
8849	Cachd1	320508	4+:100537582-100677150
8850	Olfr157	100040268	4-:43847623-43849389
8851	Pdcd4	18569	19+:53977958-54004340
8852	Ndufa3	66091	7+:3568974-3571763
8853	Ccdc103	73293	11+:102746482-102748045
8854	Stk22s1	22116	7+:52198630-52213403
8855	Psph	100678	5-:130271440-130285077
8856	Npepps	19155	11-:97068246-97141877
8857	Rab3gap2	98732	1+:187028120-187110624
8858	MMT00046222		10-:39609828-39618820
8859	Zdhhc12	66220	2-:29946470-29949142
8860	Prkab2	108097	3+:97462233-97477736
8861	Pkd1l2	76645	8-:119574929-119580355
8862	Dpp10	269109	1-:125230309-125382552
8863	Defcr5	13239	8+:22437117-22437959
8864	MMT00046672		7-:112561228-112562492
8865	Josd3	75316	9+:15110703-15116548
8866	Angptl7	654812	4-:147870264-147874570
8867	Shcbp1	20419	8-:4735979-4779534
8868	Nfkbil2	72749	15-:76456665-76470364
8869	Mtap9	213582	3+:82162117-82182930
8870	Arid3a	13496	10+:79393377-79417448
8871	Gm443	242891	5+:25022106-25023795
8872	MMT00018546		12-:13442893-13444041

8873	MMT00082610		1+:23486377-23487429
8874	MMT00058494		2-:166912685-166913277
8875	Wnt5a	22418	14+:29318741-29336126
8876	Olfr1107	258841	2-:86911351-86912289
8877	1200014M14Rik	67463	13+:97158320-97185524
8878	Kalrn	545156	16-:33975014-34392195
8879	EG433885	433885	5+:49936904-49944507
8880	Sox4	20677	13-:29043491-29045550
8881	Olfr462	258406	11-:87702461-87707424
8882	Adarb1	110532	10-:76757181-76822480
8883	Mtap4	17758	9+:109833963-109986450
8884	Plekho2	102595	9-:65402192-65420938
8885	Gm590	235634	9-:110816885-110819366
8886	Max	17187	12-:78038255-78063172
8887	Dpep1	13479	8+:125710141-125725124
8888	Lama4	16775	10+:38685373-38829988
8889	Dnahc6	330355	6-:72967598-73109292
8890	MMT00013105		
8891	MMT00071061		13-:25673992-25675286
8892	Arid3c	550619	4-:41672777-41677297
8893	Slc22a18	18400	7+:150659693-150685227
8894	Api5	11800	2-:94251890-94278230
8895	Rab4b	19342	7-:27953451-27963902
8896	Fasn	14104	11-:120668388-120685406
8897	BC062115	408062	10+:81523931-81614754
8898	Tekt5	70426	16-:10358044-10395512
8899	Pnlip	69060	19+:58744854-58748574
8900	Olfr307	258610	7-:93469534-93484904
8901	Adamts10	224697	17+:33661165-33690726
8902	Aldh3b1	67689	19-:3913492-3923904
8903	Ssbp3	72475	4+:106584330-106722297
8904	Trerf1	224829	17+:47497941-47498909
8905	Trim67	330863	8+:127317710-127352161
8906	MMT00004929		14-:58668346-58668711
8907	Npr2	230103	4+:43644807-43664301
8908	Wwc2	52357	8-:48912948-49075926
8909	Ryr1	20190	7-:29788362-29805289
8910	Ube4a	140630	9-:44754735-44773595
8911	Ces6	102022	8+:107257927-107265373
8912	1520401A03Rik	320309	17-:23841455-23844695
8913	H2afz	51788	3+:137527565-137529782
8914	Sfrs14	234373	8+:72766517-72787411
8915	Nob1	67619	8-:109936387-109948938
8916	Hps6	20170	19+:46078115-46080533
8917	MMT00078736		7+:130153625-130278539
8918	Prox2	73422	12-:86428858-86436573
8919	BC026374	234677	8+:107655699-107674008
8920	Nol5a	67134	2+:130100160-130105048
8921	Dcp1b	319618	6+:119125310-119170322
8922	Il10ra	16154	9-:45061921-45077219
8923	Gcnt1	14537	19-:17403158-17447334

8924	Fat1	14107	8+:46035567-46038847
8925	MMT00026932		9+:53731827-53732372
8926	MMT00044569		4-:52373697-52375043
8927	Olfr1284	258379	2+:111219158-111220093
8928	4732418C07Rik	230648	4+:115410365-115449931
8929	D14Abb1e	218850	14+:28242017-28296389
8930	Nyx	236690	X+:13045262-13064464
8931	Arf4	11843	14+:27457865-27475992
8932	C430004E15Rik	97031	2+:25118133-25125512
8933	Arpc5	67771	1+:154613782-154622705
8934	Grk6	26385	13+:55546869-55560938
8935	MMT00067043		10+:30093305-30094323
8936	Olfr544	257926	7-:109632628-109633632
8937	Olfr1509	57271	14+:53070089-53071015
8938	Klhdc5	232539	6+:147040053-147056667
8939	MMT00024195		15-:38505007-38506470
8940	MMT00014234	330787	8+:72186839-72188068
8941	Asb4	65255	6+:5340608-5381045
8942	Ncam2	17968	16+:81201059-81596759
8943	Mtl5	17771	19+:3389378-3407784
8944	Cdc14a	229776	3-:115996635-116126896
8945	E030030I06Rik	319887	10-:21868294-21868818
8946	4930463M05Rik	74947	12+:102443927-102444676
8947	MMT00024445		6+:98225937-98227187
8948	Onecut3	246086	10+:79957752-79976890
8949	Prkcd	18753	14-:31408554-31423498
8950	Csf2rb	12983	15+:78156287-78179749
8951	Cd97	26364	8-:86247268-86265068
8952	Marco	17167	1-:122371115-122401661
8953	Notch3	18131	17-:32257837-32303797
8954	Obscn	380698	11-:58807761-58949898
8955	Psenen	66340	7-:31346887-31348168
8956	2310046O06Rik	78323	9+:57385427-57386695
8957	Zfp511	69752	7+:147222272-147226499
8958	Gtsf1l	68236	2-:162912766-162913586
8959	Cd209g	70192	8+:4134775-4137705
8960	Nrip3	78593	7-:116901569-116925059
8961	Zcchc12	72693	X+:33735982-33739145
8962	Nisch	64652	14-:31984115-32004736
8963	Fxyd4	108017	6-:117883577-117887353
8964	Smad2	17126	18+:76422091-76464559
8965	Ahcy	269378	2-:154889942-154900128
8966	Olfr914	258782	9+:38414051-38415002
8967	Acot12	74156	13+:91881125-91925753
8968	EG545790	545790	5-:105417892-105418409
8969	Spata1	70951	3-:146120166-146162717
8970	MMT00038794		4-:12057975-12059122
8971	Slc18a2	214084	19+:59335641-59370495
8972	MMT00014869		19+:37944862-37945603
8973	Slc29a4	243328	5+:143178121-143198443
8974	Golph3l	229593	3+:95392945-95421657

8975	C87017	97184	4-:143184676-143186619
8976	Qser1	99003	2-:104598108-104628258
8977	Atpaf1	230649	4+:115457589-115491486
8978	Khdc1a	368204	1+:21339705-21360925
8979	Ihpk1	27399	9+:107949951-107951111
8980	Plekhf1	72287	7-:39005693-39012978
8981	Cacna1g	12291	11-:94269835-94335039
8982	Lin28b	380669	10-:45100996-45205583
8983	Olfr134	258829	17+:38312030-38312967
8984	Ankrd33	208258	15+:100946185-100950453
8985	Rpap1	68925	2-:119589697-119613264
8986	1300010M03Rik	67998	11-:100957829-100981138
8987	F2	14061	2-:91465477-91476571
8988	1110007A13Rik	210711	7-:135841702-135883950
8989	Gnaq	14682	19+:16207780-16458245
8990	Gm274	218304	13-:65146086-65153089
8991	Itm2c	64294	1+:87791139-87805263
8992	Trfr2	50765	5+:138011947-138028693
8993	Ankk1	244859	9-:49223291-49230066
8994	MMT00015463		X-:129940914-129941649
8995	Pxk	218699	14+:8930756-8997624
8996	Timp1	21857	X+:20448164-20451757
8997	Apol6	71939	15+:76875703-76887537
8998	Btbd7	238386	12-:104023455-104024100
8999	Pspc1	66645	14-:57341285-57397153
9000	Dhrs2	71412	14+:55840843-55860272
9001	Thsd7a	330267	6-:12358804-12505726
9002	Col24a1	71355	3+:145171603-145214975
9003	4933404G15Rik	74066	16-:16707706-16718189
9004	MMT00022623		3-:86826857-86827805
9005	Mapk1ip1l	218975	14+:47917964-47936462
9006	Paip1	218693	13+:120238761-120245886
9007	MMT00066154		
9008	MMT00066203	56304	6-:68581959-68582243
9009	MMT00077097		11-:116393054-116394419
9010	8430419L09Rik	74525	6+:135148061-135184310
9011	Lrrk2	66725	15+:91610821-91646457
9012	MMT00017112		8-:74807637-74808055
9013	Abhd6	66082	14+:8835456-8888423
9014	Dmrt3	240590	19+:25685023-25697711
9015	Lin28	83557	4-:133559244-133574731
9016	Itih2	16425	2-:10016217-10052290
9017	Tnfrsf14	230979	4-:154296191-154300771
9018	Usp15	14479	10-:122550299-122633979
9019	Hrh3	99296	2-:179834177-179838927
9020	Tmem2	83921	19+:21867139-21933212
9021	Cbln4	228942	2-:171861732-171867798
9022	Plek2	27260	12-:79989683-80007937
9023	Fbxw19	235612	9-:109381083-109388598
9024	Myo19	66196	11+:84714425-84724728
9025	C030011O14Rik	215708	3-:151937846-151980701

9026	Cep135	381644	5+:77030375-77075718
9027	EG382884	382884	14-:46177305-46198510
9028	Unc45a	101869	7-:87470180-87485094
9029	MMT00068959		7+:20059659-20066705
9030	OTTMUSG00000(	545693	4+:143089394-143091890
9031	Gpr18	110168	14-:122310652-122315056
9032	Jakmip2	76217	18-:43691062-43750330
9033	1810013L24Rik	69053	16+:8830190-8859008
9034	Trmt12	68260	15+:58704228-58708333
9035	MMT00023699		
9036	Olfr670	384703	7-:112108306-112109046
9037	Olfr368	258371	2+:37187268-37188250
9038	Gnb1	14688	4+:154901522-154931920
9039	MMT00067295		12-:104264325-104266622
9040	Olfr1499	258792	19-:13889135-13890078
9041	Nlrp3	216799	11+:59355086-59380453
9042	Prrg4	228413	2-:104672108-104689928
9043	Nat2	17961	8+:70024922-70026226
9044	MMT00014358		4+:72203729-72204031
9045	Prkar2a	19087	9+:108594676-108652766
9046	4921537P18Rik	70952	8+:28558141-28605422
9047	Defb6	116746	8+:19225477-19228207
9048	Ptpn13	19249	5+:103891151-104027370
9049	1810046J19Rik	103742	11-:98299038-98300284
9050	Msln	56047	17-:25885558-25891272
9051	Kcnj2	16518	11+:110927548-110934943
9052	A730037C10Rik	320604	3+:54943958-54960979
9053	Grm2	108068	9-:106546867-106556619
9054	Rdh7	54150	10-:127321086-127325755
9055	Rnf165	225743	18-:77701622-77743857
9056	Capn9	73647	8+:127100044-127142407
9057	Spp1	20750	5+:104865210-104870062
9058	EG629024	629024	X-:20889337-20911854
9059	Wdr42a	98193	1+:174078210-174126526
9060	Prom1	19126	5-:44384861-44492920
9061	Gdap1l1	228858	2+:163264213-163279687
9062	MMT00032325	620417	10+:9403668-9409352
9063	MMT00010135		1-:108916761-108921902
9064	Ttc12	235330	9-:49256031-49294315
9065	Reep4	72549	14+:70945050-70948740
9066	2210020M01Rik	66528	11+:115761258-115767575
9067	Tra2a	101214	6-:49193925-49214044
9068	Oip5	70645	2-:119435357-119444206
9069	OTTMUSG00000(	628923	X+:75110096-75115370
9070	Zfyve19	72008	2+:119034692-119042785
9071	Plekha3	83435	2+:76513578-76533871
9072	MMT00026795		9-:38294573-38341387
9073	MMT00079700		13-:85757466-86004750
9074	MMT00003693		14+:77730240-77751187
9075	MMT00018252		1-:186434159-186453422
9076	Ttbk2	140810	2-:120576072-120675903

9077	Cnpy4	66455	5+:138628715-138635105
9078	Abpz	233090	7-:34522212-34524322
9079	Gng8	14709	7+:17480292-17480711
9080	MMT00059751		3-:67410193-67411778
9081	EG229746	229746	3+:112105283-112106168
9082	Nrarp	67122	2+:25036542-25038845
9083	MMT00011564		3-:100113318-100114295
9084	MMT00002596		6+:47314860-47354360
9085	MMT00056875		5+:56015371-56016830
9086	Zfp652	268469	11+:95610313-95626488
9087	Wdr63	242253	3-:145703485-145771002
9088	Ppp4c	56420	7-:133929482-133936010
9089	Sh3bgrl2	212531	9+:83441935-83494084
9090	Pm20d1	212933	1+:133693979-133700704
9091	2900024C23Rik	67266	5-:108338221-108416058
9092	Akp3	11648	1+:89021582-89024485
9093	Taf4a	228980	2-:179646857-179710159
9094	MMT00012980		11-:6680585-6683244
9095	Qsox1	104009	1-:157625953-157659937
9096	B3gat1	76898	9+:26559088-26565566
9097	Endod1	71946	9-:14158449-14185414
9098	Rhox9	104384	X-:35252275-35254947
9099	Echs1	93747	7-:147291607-147302353
9100	Ms4a6c	73656	19+:11543888-11555816
9101	MMT00006945		3+:92741602-92741996
9102	Tfpt	69714	7-:3571926-3581486
9103	Igh-VJ558	16061	12-:115920819-116089829
9104	Cobra1	58202	2-:25055236-25066901
9105	Sars	20226	3-:108229343-108248184
9106	4931429I11Rik	70989	9-:40702931-40769108
9107	Lrrc20	216011	10+:60938561-61043655
9108	Cyb5d1	327951	11-:69207139-69209141
9109	BC089597	216454	10+:127303543-127314374
9110	Hoxd12	15432	2+:74513143-74514109
9111	BC038286	227298	1+:75139514-75144481
9112	MMT00022850	629924	11-:99701288-99701692
9113	Ubxd5	67586	4+:133658517-133682691
9114	Strc	140476	2-:121189464-121206676
9115	Dhx40	67487	11-:86583255-86621139
9116	M6prbp1	66905	17-:56418384-56429934
9117	Tmem185b	226351	1+:121422743-121424801
9118	Hspb6	243912	7+:31338337-31340463
9119	Ptprg	19270	14+:12386525-13070441
9120	Atp2c1	235574	9-:105313692-105372478
9121	Mtap7d3	320923	X-:54068701-54072116
9122	Sepw1	20364	7-:16502558-16507675
9123	Cdc42se1	57912	3+:95032669-95040331
9124	BC067068	216292	10-:105263194-105278375
9125	Ppil3	70225	1-:58487840-58502330
9126	Mapk1	26413	16+:17045304-17047542
9127	Mtfmt	69606	9+:65283588-65300209

9128	Nxph1	18231	6+:8859959-8937962
9129	Aox4	71872	1+:58267313-58325340
9130	Centa2	216991	11+:79967616-79992949
9131	Ranbp3l	223332	15+:8917966-8951507
9132	Cyp7a1	13122	4-:6195359-6202718
9133	Scrt1	170729	15-:76348781-76352362
9134	Harbi1	51897	2-:91514782-91550733
9135	Mbd6	110962	10-:126719013-126720699
9136	MMT00009391	544749	10+:128748564-128755512
9137	Hunk	26559	16+:90386641-90499796
9138	Cox10	70383	11-:63807386-63892969
9139	Osbpl9	100273	4-:108734259-108775177
9140	EG623166	623166	9+:98743005-98744062
9141	MMT00028469		
9142	Cacng4	54377	11-:107596093-107655778
9143	Akr1c14	105387	13+:4058836-4089663
9144	Sclt1	67161	3-:41430634-41546433
9145	V1rh14	171273	13-:23205703-23206599
9146	Ergic1	67458	17+:26698503-26793874
9147	Il17rd	171463	14+:27880583-27901592
9148	MMT00042539		11-:119624665-119640686
9149	Ccdc15	245902	9-:37150020-37155974
9150	Rab22a	19334	2+:173485361-173530173
9151	Fhl2	14200	1-:43179922-43220695
9152	Indo	15930	8-:25694614-25707481
9153	Suv39h2	64707	2-:3375895-3392236
9154	Tfg	21787	16-:56690446-56717467
9155	Olfr982	258853	9+:39881881-39882846
9156	Klra23	79410	6-:130253385-130256445
9157	Txnrd1	50493	10+:82321923-82360456
9158	Pde9a	18585	17+:31523223-31613101
9159	MMT00004659		14+:25177580-25177796
9160	1700026L06Rik	69987	2-:28547598-28555257
9161	Mrpl17	27397	7-:112958097-112959581
9162	Fhad1	329977	4-:141558305-141570987
9163	MMT00066243		2+:28332608-28333637
9164	Uba5	66663	9-:103949844-103965449
9165	Nnat	18111	2+:157385858-157388239
9166	2310067B10Rik	71947	11+:115638561-115660408
9167	Vim	22352	2+:13495974-13504403
9168	Eif5	217869	12+:112776303-112784962
9169	Zfp41	22701	15+:75447147-75477861
9170	Cish	12700	9-:107194899-107195627
9171	Il27ra	50931	8-:86554229-86566442
9172	Tmem131	56030	1-:36849038-36996032
9173	Arhgap15	76117	2+:43919268-44243139
9174	Olfr1213	258900	2-:88813112-88814046
9175	MMT00081793		1+:10795325-10796347
9176	Dach2	93837	X+:110411865-110949502
9177	Rpl37	67281	15+:5066644-5069140
9178	Med13	327987	11-:86159360-86169010

9179	Gtl3	14894	8-:97944151-97958692
9180	MMT00014166		19-:24363491-24363741
9181	1700060H10Rik	73390	10+:55826735-55836676
9182	MMT00053423		11-:70870674-70871471
9183	Glrx3	30926	7+:144629332-144659865
9184	Olfr1370	258528	13-:21164219-21165096
9185	MMT00019531		1-:64844975-64865381
9186	EG333563	333563	X-:103501900-103505115
9187	MMT00031477	381346	2+:4863197-4863737
9188	V1rc5	113862	6+:57159851-57160754
9189	Dmgdh	74129	13+:94444392-94522778
9190	Rbmxl2	76572	7+:114352930-114354436
9191	Lbxcor1	207667	9-:62986004-62987935
9192	MMT00042303		7-:17279167-17292589
9193	Ccdc109a	215999	10-:59062612-59063441
9194	Dynlrb2	75465	8+:119031359-119039814
9195	Sub1	20024	15-:11911622-11925746
9196	RP23-330D3.5	664987	2-:174887051-174889489
9197	Slc13a5	237831	11-:72056878-72061327
9198	EG668103	668103	X-:91162043-91167301
9199	Xlr5c	27084	X-:70530535-70535854
9200	Sdc1	20969	12+:8778201-8800491
9201	Zkscan14	67235	5-:145955833-145962707
9202	Fbxo47	72973	11-:97702185-97726481
9203	Slc32a1	22348	2+:158436936-158441398
9204	Olfr1341	258852	4+:118382013-118382939
9205	4921532D01Rik	70961	14-:64472808-64479027
9206	A230083H22Rik	353211	19+:17198260-17282817
9207	Ddx11	320209	17+:66472926-66500068
9208	MMT00038995		6-:102886170-102887552
9209	Sf3a3	75062	4+:124392104-124409659
9210	Lmo3	109593	6-:138314108-138375717
9211	Olfr711	259037	7-:114114912-114115856
9212	Pde6d	18582	1-:88439590-88479035
9213	Osgin1	71839	8+:121961044-121970154
9214	Zyg11b	414872	4-:107905168-107973645
9215	Tnni1	21952	1+:137701629-137706310
9216	Olfr1225	258893	2-:89010433-89011367
9217	Lrrc34	71827	3-:30523190-30546793
9218	Npy5r	18168	8-:69203865-69211993
9219	Mad2l1bp	66591	17-:46284337-46290485
9220	E430004N04Rik	210757	10+:28388162-28583416
9221	Ilf2	67781	3+:90280078-90291653
9222	MMT00005895		6+:7144561-7274017
9223	Tnpo1	238799	13-:99611921-99696341
9224	MMT00013510	227885	2+:50765616-50767367
9225	MMT00001190		15-:82442288-82443404
9226	Aim2	383619	1+:175382956-175395350
9227	5730470L24Rik	66641	3+:102799667-102806904
9228	Mccc1	72039	3-:35882647-35892935
9229	Ggnbp2	217039	11-:84645861-84683653

9230	1700027N10Rik	75564	17-:46266225-46281147
9231	H2-M2	14990	17-:37618416-37620191
9232	4930430A15Rik	67575	2-:111033410-111069755
9233	Acss3	380660	10-:106374199-106425965
9234	Arf1	11840	11-:59025627-59041716
9235	Tnfrsf12a	27279	17-:23812413-23814411
9236	Foxh1	14106	15-:76498738-76500303
9237	Wdr17	244484	8-:55714974-55733780
9238	Zfp786	330301	6-:47769541-47785897
9239	Fiz1	23877	7-:4958661-4965813
9240	Olfr401	258701	11+:73934792-73935739
9241	4930417M19Rik	320571	4+:43318589-43386042
9242	MMT00033212		X-:73216791-73218523
9243	Olfr352	258942	2+:36725087-36726034
9244	Rabif	98710	1+:136391236-136404459
9245	Zc3h3	223642	15-:75587044-75609986
9246	V1re5	171228	17+:20639262-20640157
9247	Dock5	68813	14-:68373452-68454500
9248	Bcl11b	58208	12-:109153432-109241431
9249	Zdhhc3	69035	9-:122987157-123022296
9250	Sec16a	227648	2-:26264952-26296186
9251	P2ry1	18441	3+:60807332-60810924
9252	Slc22a2	20518	17+:12777054-12821353
9253	Metapl1	66559	2+:71291394-71363243
9254	Cyp2j12	242546	4-:95768722-95807733
9255	Emp3	13732	7-:53173433-53176774
9256	Psmd4	19185	3-:94836626-94846467
9257	MMT00021093		11-:83458548-83459123
9258	P4ha1	18451	10+:58799791-58836050
9259	Clcn6	26372	4-:147380592-147412876
9260	Cmtm6	67213	9+:114640320-114658461
9261	MMT00057172		2+:106851082-106851890
9262	MMT00053633		3-:45578522-45580136
9263	Fbxo27	233040	7+:29383177-29484354
9264	MMT00071053		10-:41098780-41100444
9265	Zfp157	72154	5+:138882704-138900293
9266	MMT00023660		
9267	Uqcrh	66576	4-:115739569-115747653
9268	Vps33a	77573	5-:123980755-124023007
9269	Ptprq	237523	10-:106954844-107156535
9270	MMT00072976		
9271	Hspa12b	72630	2+:130953175-130971178
9272	Btbd11	74007	10+:85009186-85123034
9273	MMT00057004		16-:55325603-55325682
9274	1700028E11Rik	70002	13+:62016030-62034220
9275	Aass	30956	6-:23022501-23072733
9276	MMT00008480		8-:44814581-44815307
9277	Ptprh	545902	7-:4500950-4549782
9278	Clec2i	93675	6+:128837631-128846864
9279	MMT00066808		10+:54844229-54844592
9280	Vps13c	320528	9+:67688233-67843934

9281	Rnf122	68867	8+:32222313-32240138
9282	Pcdhb13	93884	18+:37602224-37604613
9283	Dennd1a	227801	2-:37654515-38142904
9284	MMT00028928		12+:97530592-97531244
9285	Mtcp1	17763	X-:72650203-72661882
9286	Zufsp	72580	10-:33638949-33669304
9287	Trpm3	226025	19+:23066780-23067091
9288	4921530L21Rik	66732	14+:96280484-96281994
9289	MMT00068427		18-:90112821-90113394
9290	Cd1d1	12479	3-:86799759-86803259
9291	Usp52	103135	10+:127755857-127758413
9292	4-Mar	381270	1-:72493698-72500173
9293	Pcdhb19	93890	18+:37656807-37659487
9294	BC023829	236848	X-:67713757-67730357
9295	G6pd2	14380	5+:62200122-62201663
9296	Nfic	18029	10-:80863673-80889857
9297	Zeb1	21417	18+:5591888-5774908
9298	MMT00010260		11-:121026773-121028707
9299	MMT00001758		
9300	MMT00021968		18-:15080483-15081679
9301	MMT00050551		3-:62974251-62977961
9302	Ctsr	56835	13-:61260577-61265549
9303	Tesp1	21755	1+:34555274-34559907
9304	AI449175	234362	8-:72134551-72149422
9305	Zfp292	30046	4-:34752099-34758805
9306	Spon1	233744	7+:120909653-121184873
9307	Prpf4	70052	4+:62069832-62085553
9308	MMT00054569		9-:44418453-44418806
9309	MMT00035904		10+:22063211-22065941
9310	Pdrg1	68559	2-:152834624-152841127
9311	Lrrc27	76612	7+:146398908-146414909
9312	Hyal5	74468	6+:24825942-24841959
9313	Samd4	74480	14+:47635951-47723354
9314	Serpina7	331535	X-:135614318-135618240
9315	Gins2	272551	8-:123102895-123112988
9316	9530066K23Rik	214112	11-:45963649-45979863
9317	MMT00063086		3-:14428491-14512010
9318	C79267	212632	4+:139130633-139172985
9319	Uqcrfs1	66694	13-:30632180-30637189
9320	Rnf31	268749	14+:56210683-56222511
9321	Vsx2	12677	12+:85910892-85936408
9322	Relt	320100	7-:107994359-108001637
9323	MMT00059222		17-:36385585-36386060
9324	Egfl6	54156	X-:162960938-163023644
9325	C130060K24Rik	243407	6+:65331097-65407013
9326	Pgm5	226041	19-:24757505-24936279
9327	Eif2s1	13665	12+:79967593-79986306
9328	Gpx4	625249	10+:79516414-79519182
9329	Serpina3m	20717	12+:105625401-105632468
9330	Tapt1	231225	5-:44566401-44617826
9331	Zbtb6	241322	2-:37283376-37286368

9332	Fsd1	240121	17+:56127164-56136303
9333	MMT00074561		1+:183212164-183214022
9334	Syncrip	56403	9-:88350488-88375597
9335	Chac2	68044	11-:30876723-30886334
9336	Pomp	66537	5+:148672203-148687354
9337	1500010J02Rik	68964	11+:68829592-68837709
9338	Zfp313	81018	2+:167340879-167341664
9339	Tnmd	64103	X+:130385600-130400058
9340	Cdc42ep2	104252	19-:5917559-5924803
9341	Sephs1	109079	2+:4802813-4830569
9342	EG628855	628855	X+:18406327-18498338
9343	Ildr1	106347	16+:36694123-36726827
9344	Tmem123	71929	9+:7764127-7794330
9345	D19Ertd652e	70806	19-:47811808-47820093
9346	Zfp112	57745	7+:24897332-24912971
9347	Tcp1	21454	17+:13109379-13117431
9348	Hspd1	15510	1-:55135126-55144638
9349	Il6ra	16194	3-:89680732-89716964
9350	Pcmtd1	319263	1+:7079126-7163706
9351	Zmiz1	328365	14+:26278708-26486233
9352	Hnrpll	72692	17-:80429682-80434896
9353	Emilin2	246707	17-:71601513-71660256
9354	Vps13b	666173	15+:35582009-35700296
9355	Reep1	52250	6-:71759350-71760705
9356	Emid2	140709	5-:137217634-137358977
9357	Morc4	75746	X-:136356173-136406193
9358	Olfr20	258925	11+:73167256-73168200
9359	Dhx34	71723	7-:16782569-16807381
9360	Taf1c	21341	8-:122121877-122129083
9361	Hand1	15110	11-:57642214-57645649
9362	Gast	14459	11+:100197796-100198213
9363	Wisp2	22403	2+:163646634-163658880
9364	Akr1a4	58810	4-:116309115-116324172
9365	4932438H23Rik	74387	16-:91054179-91069390
9366	Ccl27	20301	4+:41720061-41721015
9367	Abp1	76507	6+:48855827-48946996
9368	1500019G21Rik	66245	7-:4612121-4636171
9369	Golph3	66629	15+:12251207-12279658
9370	Rps6	20104	4-:86500567-86503253
9371	Lrrc57	66606	2-:120429975-120435276
9372	Psg19	26439	7-:19375142-19383859
9373	Rpl9	20005	5-:65779603-65782233
9374	Thbs4	21828	13-:93521542-93564713
9375	2210016L21Rik	72357	5+:115392205-115398559
9376	Zscan18	232875	7-:13353442-13380337
9377	Klhdc1	271005	12+:70342803-70359170
9378	Gtf3c5	70239	2-:28421778-28439270
9379	Ypel2	77864	11-:86752611-86807236
9380	Spata5	57815	3+:37319219-37478012
9381	MMT00051394		18+:80222831-80226750
9382	Nwd1	319555	8+:75199002-75212088

9383	MMT00069081		9-:54942182-54942681
9384	4930449I24Rik	67410	5+:147313974-147316759
9385	Hmg20a	66867	9+:56266636-56344688
9386	Bxdc5	70285	3-:146169381-146184382
9387	Tesp2	21756	1-:34609189-34617765
9388	Prr3	75210	17-:36109485-36115916
9389	Rnase6	78416	14+:51748632-51751066
9390	Olfr705	259034	7-:114016806-114017757
9391	MMT00060086		3-:115609181-115610467
9392	4922505G16Rik	629499	2-:157054356-157064177
9393	Cd300a	217303	11+:114761052-114763230
9394	Cfhr1	50702	1-:141443640-141456799
9395	Eif4g3	230861	4+:137601252-137762982
9396	Grlf1	232906	7-:17232551-17233248
9397	AA408865	99980	4+:148334643-148342068
9398	Eif1ad	69860	19+:5366490-5368944
9399	Tcfeb	21425	17+:47922713-47928861
9400	Syt10	54526	15-:89612824-89672200
9401	Fcgr4	246256	1+:172949069-172959880
9402	1200016B10Rik	66875	1-:153214828-153275511
9403	V1rc11	171184	6-:66665967-66666883
9404	Setd7	73251	3-:51325221-51364526
9405	Aldoc	11676	11+:78137559-78141551
9406	Calcoco1	67488	15-:102537209-102552500
9407	Cmah	12763	13-:24417640-24423491
9408	Hmgcs2	15360	3+:98084355-98114656
9409	11-Mar	211147	15+:26238841-26339328
9410	Zmym3	56364	X-:98599723-98616188
9411	Taf7l	74469	X-:130995161-131007506
9412	Vwa3a	233813	7+:127923638-127933758
9413	1190020J12Rik	233038	7-:29328621-29332280
9414	Olfr178	258999	16-:58889086-58890044
9415	MMT00075172		16+:38476685-38477609
9416	MMT00004780		
9417	Cd163	93671	6+:124254673-124280541
9418	MMT00034311		X-:48572007-48573190
9419	Acox2	93732	14-:9058025-9091450
9420	MMT00065589		10+:70781513-70782133
9421	Serpinf2	18816	11-:75245237-75252977
9422	Eif2b1	209354	5-:125020662-125029054
9423	2010107E04Rik	70257	12-:113199586-113205188
9424	Cltc	67300	11-:86507857-86570993
9425	Gusb	110006	5-:130464891-130478698
9426	Sned1	208777	1+:95152841-95155683
9427	Nqo2	18105	13+:34059021-34077508
9428	MMT00061031		1-:194705970-194715458
9429	Dzip1	66573	14-:119278103-119282962
9430	Tmem74	239408	15-:43698273-43699191
9431	Snapc5	330959	9+:64027135-64030457
9432	Mgea5	76055	19-:45826625-45857620
9433	Pou6f2	218030	13-:18216794-18264396

9434	Hnrnpf	98758	6+:117856816-117875022
9435	Rab37	58222	11+:115015512-115022072
9436	Cntnap2	66797	6+:46799032-46858778
9437	MMT00074099		16+:64851023-64912048
9438	Pctk3	18557	1-:134010237-134036261
9439	Dguok	27369	6-:83430212-83456888
9440	Zfp653	319601	9-:21859858-21875966
9441	MMT00052383		
9442	BC031441	240120	17-:56077799-56084682
9443	Cml2	93673	6-:85817593-85819109
9444	Col10a1	12813	10+:34110427-34115881
9445	MMT00024605		13+:24280698-24298158
9446	MMT00017417		16+:4860962-4863120
9447	Tie1	21846	4-:118143795-118162666
9448	Nsun7	70918	5+:66652076-66688297
9449	Sft2d2	108735	1-:167108821-167124443
9450	MMT00033190		9+:78243686-78271394
9451	Gpr180	58245	14+:118536356-118562483
9452	EG668106	668106	19-:33695425-33697158
9453	EG637227	637227	6-:70210379-70210939
9454	2810426N06Rik	67607	7+:48854629-48906895
9455	Gpatch8	237943	11-:102337231-102417438
9456	Zpbp	53604	11-:11182515-11362411
9457	Krt16	16666	11-:100107407-100110216
9458	Twist2	13345	1+:93698053-93744604
9459	Ccdc73	211936	2+:104747693-104791765
9460	Fer1l4	74562	2-:155844875-155877982
9461	Gda	14544	19-:21459479-21547077
9462	Ndst4	64580	3+:125140597-125427692
9463	Zdhhc23	332175	16-:43973580-43979241
9464	MMT00042505		X-:64031616-64138129
9465	AI427515	270097	8+:116754105-116895643
9466	MMT00065267		2+:26505371-26507332
9467	Aoah	27052	13+:20885981-21115571
9468	Gtf2a1l	71828	17+:89068044-89114488
9469	Trim71	636931	9-:114421764-114471675
9470	A230074B11Rik	215413	1+:37092471-37102053
9471	MMT00013158		17+:16863558-16863968
9472	Cstf3	228410	2+:104430679-104505575
9473	Gfra3	14587	18-:34849559-34879919
9474	Cops7b	26895	1+:88483719-88502837
9475	Tmem16h	382014	8-:74005463-74009837
9476	9130005N14Rik	68303	5+:65361352-65432382
9477	Efcab2	68226	1+:180336230-180411764
9478	Trim46	360213	3-:89038106-89049699
9479	MMT00063832		9+:120998128-121156771
9480	A430107O13Rik	214642	6+:21935927-22019450
9481	Ppl	19041	16-:5086823-5132514
9482	Trpm4	68667	7-:52558588-52589110
9483	Wdr13	73447	X-:7700429-7709651
9484	4933434M16Rik	71203	11+:112686492-112693783

9485	2700081O15Rik	108899	19+:7495744-7497596
9486	Coch	12810	12+:52694343-52706748
9487	Kcng4	66733	8-:122147753-122150026
9488	4932413O14Rik	320696	5-:93037279-93052466
9489	Prpf40b	54614	15+:99133711-99147438
9490	Zfp462	242466	4+:55063048-55094476
9491	Bdkrb2	12062	12+:106829789-106830889
9492	MMT00007123		15-:19384382-19386190
9493	MMT00043490		3-:46888982-46889656
9494	Cib2	56506	9-:54393158-54407886
9495	Srms	20811	2-:180940267-180947712
9496	Mtmr9	210376	14-:64142317-64162542
9497	Sgms2	74442	3-:131025655-131047851
9498	Dcst2	329702	3+:89171510-89172170
9499	Gtpbp5	52856	2+:179805291-179820604
9500	EG209589	209589	6-:67338861-67365006
9501	Usp33	170822	3+:152009452-152056205
9502	V1ri7	171258	13-:22593206-22594114
9503	MMT00030769		2+:24448424-24450039
9504	Sfrp1	20377	8+:24522002-24558429
9505	Rps27	57294	3-:90016590-90017579
9506	MMT00048675		1-:146159158-146159590
9507	Trip6	22051	5-:137751127-137755362
9508	AI847670	330050	5+:20930703-20961695
9509	Rsrc1	66880	3+:66789640-67162317
9510	Nt5c2	76952	19-:46963150-47036787
9511	Eif5al3	665733	5+:90561229-90562320
9512	MMT00081874		10+:110593271-110594471
9513	Tardbp	230908	4-:147990975-148000863
9514	Vps37a	52348	8+:41597154-41631914
9515	Lyg2	332427	1-:37966740-37968047
9516	1700020N01Rik	67692	10+:21312962-21342182
9517	Med7	66213	11+:46250483-46255025
9518	Lsm3	67678	6+:91467055-91472612
9519	Fstl5	213262	3+:76125975-76512555
9520	Grem1	23892	2-:113588726-113590380
9521	Ctnnb1	12387	9+:120842695-120868894
9522	Cidea	12683	18+:67503257-67527432
9523	Lcor	212391	19-:41636080-41636736
9524	Pum1	80912	4+:130219303-130337187
9525	MMT00019383		11+:50861610-50862552
9526	5730528L13Rik	66665	4+:48553370-48574791
9527	Pglyrp1	21946	7+:19470038-19475787
9528	Amelx	11704	X-:165617951-165625142
9529	Tmprss2	50528	16-:97787594-97832673
9530	Net1	56349	13-:3882581-3917357
9531	MMT00043554	670496	11+:99676416-99680661
9532	MMT00050594		3+:135994449-135995957
9533	Mdga1	74762	17-:29987436-30025362
9534	MMT00042032		6-:122515894-122517421
9535	MMT00069497		X+:38014636-38015105

9536	Barhl2	104382	5-:106882127-106886860
9537	Sypl	19027	12+:33638832-33660587
9538	MMT00039019	629967	11-:111586054-111586945
9539	Rab14	68365	2-:35037685-35056513
9540	Csnk1e	27373	15-:79249596-79272432
9541	Npal3	74552	4-:135007790-135050455
9542	Tmed10	68581	12-:86683795-86715649
9543	MMT00046366	114679	11+:3414908-3417353
9544	2410018C17Rik	74504	5-:33943000-33972275
9545	Tnfaip3	21929	10-:18722538-18731581
9546	1700023A16Rik	69371	6+:146798623-146804705
9547	MMT00006479		12+:120849421-121115281
9548	Wdr75	73674	1+:45852294-45880468
9549	Gpr22	73010	12-:32393576-32394845
9550	MMT00018088	268885	16+:36093535-36162035
9551	MMT00002569		1+:4512987-4516248
9552	Dnmt3a	13435	12+:3807090-3908996
9553	Alox12e	11685	11-:70129114-70136020
9554	Hmgb2l1	70823	8+:77517580-77555876
9555	Spg3a	73991	12+:71047163-71065073
9556	Akna	100182	4-:63028217-63055868
9557	Cnga3	12790	1+:37289042-37319411
9558	Akr7a5	110198	4+:138866669-138874334
9559	Zfpm2	22762	15+:40486817-40936131
9560	Slc4a1ap	20534	5+:31829382-31856416
9561	MMT00017162		5+:41733361-41734171
9562	MMT00066167		14+:30716149-30717594
9563	Nuak1	77976	10-:83836245-83903255
9564	Cdh20	23836	1+:106665105-106891255
9565	Adamts17	233332	7+:73984657-74054374
9566	MMT00078098	667014	1-:51997510-51998144
9567	Cfl1	12631	19+:5490520-5493563
9568	Fen1	14156	19-:10273635-10278251
9569	Dda1	66498	8+:73993096-74000332
9570	Creld1	171508	6+:113433562-113443332
9571	Parp4	328417	14+:57204393-57278632
9572	Oosp1	170834	19-:11742025-11765546
9573	MMT00015549		4-:46256118-46271626
9574	Mphosph10	67973	7-:71521463-71532406
9575	MMT00011132		8+:71993967-71994296
9576	Polr1e	64424	4+:45031517-45045231
9577	Dusp8	18218	7-:149267402-149276175
9578	Olfr1047	259014	2-:86068166-86069126
9579	MMT00052106		1+:44700765-44702306
9580	Tuba8	53857	6+:121160787-121176115
9581	EG245347	245347	X+:12229428-12229788
9582	MMT00041407		5+:138056838-138057318
9583	Angel1	68737	12-:88041452-88067401
9584	Ybx2	53422	11+:69749476-69755099
9585	Neu3	50877	7-:106959950-106976927
9586	Mrpl53	68499	6+:83059101-83059926

9587	Bri3bp	76809	5+:125921966-125935506
9588	Ghrl	58991	6-:113666245-113669423
9589	Sorcs1	58178	19-:50225469-50752975
9590	Rapgefl1	268480	11+:98698467-98712513
9591	Bai2	230775	4+:129662479-129699886
9592	Pitrm1	69617	13+:6547402-6579385
9593	Olfr545	258837	7-:109642337-109643287
9594	Asb13	142688	13+:3633323-3651018
9595	Rftn1	76438	17-:50131581-50329998
9596	2610301F02Rik	545428	2-:76852941-76880606
9597	Tnfsf13b	24099	8+:10006632-10035997
9598	EG236749	236749	X+:23483390-23484621
9599	Mrpl2	27398	17+:46783204-46787078
9600	Gramd1b	235283	9-:40111080-40220959
9601	Wt1	22431	2+:104966685-105013769
9602	Pogk	71592	1-:168327974-168339463
9603	Bnipl	171388	3-:95045216-95055024
9604	Bscl2	14705	19+:8915702-8923074
9605	Ncan	13004	8-:72618863-72625960
9606	MMT00075599		14+:13700030-13700528
9607	Ccdc114	211535	7+:53179439-53184669
9608	MMT00021413		14+:17938189-17938654
9609	Itln1	16429	1-:173464884-173465418
9610	Narf	67608	11+:121098591-121114836
9611	Ncf2	17970	1+:154655065-154683383
9612	4930570E03Rik	78812	16+:78695277-78709324
9613	Sap18	20220	14+:58417090-58423813
9614	Rufy4	435626	1+:74187492-74194319
9615	MMT00006705		8-:7000589-7002764
9616	AI451557	102084	8+:97047048-97051165
9617	Mrpl34	94065	8+:73988872-73989644
9618	Snap25	20614	2+:136539238-136608152
9619	MMT00032716		9-:25032829-25033463
9620	Sdpr	20324	1+:51345969-51359803
9621	Spink5	72432	18+:44174559-44181803
9622	Rasgef1a	70727	6+:118016462-118041563
9623	Zfp300	245368	X-:20607090-20666655
9624	Sla	20491	15-:66614046-66633071
9625	Vmn2r97	627367	17+:19066488-19085181
9626	4930448C13Rik	73972	12-:15004639-15056241
9627	Cbx6	494448	15-:79656536-79664667
9628	Spg21	27965	9+:65308777-65335260
9629	Spnb3	20743	19+:4718840-4752350
9630	5830417C01Rik	78825	1+:180117878-180179751
9631	Htr4	15562	18+:62500703-62624296
9632	Wfdc12	192200	2-:164015871-164016334
9633	Emp1	13730	6+:135327228-135331156
9634	Atp13a2	74772	4+:140542880-140563244
9635	Rabggtb	19352	3-:153570417-153575023
9636	Syne1	64009	10+:4909718-4941092
9637	Sh3bgr	50795	16+:96422310-96442199

9638	Tpbg	21983	9+:85737586-85738866
9639	MMT00038421		12+:112136317-112136796
9640	Ankrd32	105377	13-:77182354-77223645
9641	MMT00017480		6+:94770523-94771358
9642	Zfp628	232816	7+:4866835-4895205
9643	Doxl2	243376	6+:48925141-48928627
9644	Ankrd32	105377	13-:77243878-77274722
9645	MMT00072449		5+:5749184-5799153
9646	Rpl36al	66483	12-:70283727-70285012
9647	Hip1	215114	5-:135883905-136020985
9648	Csf3	12985	11+:98562626-98564943
9649	Commd6	66200	14-:102039183-102039897
9650	Sh3gl3	20408	7+:89319745-89455919
9651	Cxxc5	67393	18+:36018186-36021341
9652	Igh-2	238447	12-:116055259-116055776
9653	Acox3	80911	5+:35925861-35955256
9654	1810041L15Rik	72301	15-:84202330-84256540
9655	Sult2b1	54200	7-:52985353-53014892
9656	Cenpc1	12617	5-:86441060-86494475
9657	MMT00007450		14+:104880503-104881121
9658	Abr	109934	11-:76230245-76384593
9659	Tmem28	620592	X+:97016638-97041361
9660	Tnni3k	435766	3-:154449264-154519577
9661	MMT00069511		14+:62177612-62178725
9662	Vmn2r27	232367	6-:124095646-124121750
9663	Arl9	384185	5+:77435465-77436432
9664	2700046G09Rik	67188	19+:32463969-32465667
9665	Pou3f4	18994	X+:108009803-108010888
9666	Trim62	67525	4+:128561413-128588578
9667	MMT00024156		14-:88749180-88750210
9668	Astn1	11899	1+:160292842-160621910
9669	Mrpl54	66047	10-:80727466-80729671
9670	Olfr346	258940	2+:36543523-36544452
9671	AK129341	234915	9-:8076460-8134472
9672	Tmem101	76547	11-:102013862-102017675
9673	Sprr2a	20755	3+:92023013-92023264
9674	Pea15a	18611	1-:174128757-174130710
9675	C80435	97813	12+:40601429-40602856
9676	Usp32	237898	11-:84797933-84953270
9677	Rttn	246102	18+:89276977-89299209
9678	AW555464	217882	12+:113962834-113984800
9679	Hivep3	16656	4+:119737539-119808016
9680	Ahsa1	217737	12+:88607677-88614902
9681	Asb12	70392	X-:92665539-92673468
9682	Pvrl4	71740	1+:173300484-173318724
9683	Prdm8	77630	5+:98609919-98616470
9684	Phf21b	271305	15-:84617532-84686558
9685	Bcl9l	80288	9+:44316958-44318493
9686	Nek4	23955	14+:31764673-31800586
9687	Olfr1494	258992	19+:13823597-13824544
9688	MMT00045085		16+:23089518-23094398

9689	4432416J03Rik	78252	9-:48126088-48148880
9690	Tex11	83558	X-:98034002-98254976
9691	Neu4	241159	1+:95919064-95922810
9692	Ankrd26	232339	6-:118456387-118512243
9693	Ccnb1	268697	13-:101548686-101556418
9694	Ifi205	226695	1-:175931686-175945292
9695	MMT00078835		7+:134295864-134299112
9696	Setd1b	208043	5+:123592965-123618638
9697	Mterf	545725	5-:3890581-3893927
9698	MMT00081969		6+:19069995-19070912
9699	Nmur2	216749	11-:55840484-55854385
9700	Prodh	19125	16-:18071818-18089240
9701	Prkcz	18762	4-:154636185-154735352
9702	Stk39	53416	2-:68049808-68310013
9703	MMT00068433		5-:15004162-15004577
9704	Arfgap1	228998	2+:180701975-180716102
9705	Ube2j1	56228	4+:33118477-33137000
9706	EG630579	630579	13+:62231259-62231389
9707	Golt1a	68338	1+:135206391-135218519
9708	Taf2	319944	15-:54846682-54903665
9709	Olfr969	258823	9+:39602961-39603896
9710	Nrp2	18187	1+:62750054-62843547
9711	AU021034	219170	14-:78482551-78488765
9712	Cct3	12462	3+:88101056-88125688
9713	Ykt6	56418	11+:5855888-5866078
9714	S1pr2	14739	9-:20770394-20781216
9715	OTTMUSG00000(	100038514	4+:14865614-14880177
9716	MMT00079592		13+:74577649-74577983
9717	Psmb9	16912	17-:34319043-34324275
9718	Fmo5	14263	3+:97432972-97456516
9719	Cep152	99100	2-:125445396-125450838
9720	Fosb	14282	7-:19888262-19895100
9721	Rrs1	59014	1+:9535522-9537532
9722	Ssfa2	70599	2+:79484767-79513119
9723	Clcn7	26373	17+:25270353-25297926
9724	Oat	18242	7-:139749161-139761813
9725	Dok6	623279	18-:89590366-89768183
9726	Ptchd3	74675	11+:121691557-121703932
9727	Atp9b	50771	18-:80932752-81114580
9728	MMT00031196		X-:147848048-147849090
9729	Got1l1	76615	8-:28307928-28313023
9730	Olfr1501	258626	19-:13912715-13928068
9731	Pik3r3	18710	4+:115894244-115975778
9732	Iapp	15874	6+:142246947-142252337
9733	4921509E07Rik	70897	12+:79792521-79835500
9734	Wwp1	107568	4-:19536781-19605588
9735	Rbm14	56275	19-:4800924-4811585
9736	Shisa2	219134	14+:60244681-60250495
9737	Zswim5	74464	4+:116549958-116659937
9738	Cdk5rap2	214444	4-:69882772-69915753
9739	Rpl18	19899	7+:52974660-52976194

9740	Lyn	17096	4+:3605262-3718751
9741	Ppp1r8	100336	4-:132382844-132399035
9742	Ccdc129	232016	6+:55822484-55847954
9743	Gm189	235952	6-:69525779-69526494
9744	9530002B09Rik	77432	4+:122366609-122379627
9745	Gadd45g	23882	13+:51942063-51943841
9746	Dock1	330662	7+:142182204-142365320
9747	Wnt10b	22410	15-:98602192-98608581
9748	Rxfp3	239336	15-:10965621-10967039
9749	Blvrb	233016	7+:28233056-28251005
9750	Lancl3	236285	X+:8777355-8842809
9751	C1ql3	227580	2-:12923232-12933441
9752	2410002F23Rik	66976	7+:51502156-51507686
9753	Pkhd1	241035	1-:20596410-20608085
9754	2010204N08Rik	69983	3-:72707527-72770788
9755	MMT00066614	667386	5-:149774774-149781471
9756	1500015O10Rik	78896	1+:43787537-43799387
9757	Olfr502	258734	7-:115666517-115667462
9758	Slc39a8	67547	3+:135488242-135550020
9759	Cenpn	72155	8+:119445639-119465403
9760	1700012A16Rik	71836	1+:155272278-155299783
9761	MMT00067282		11+:74136047-74136768
9762	Snx5	69178	2-:144076790-144096607
9763	Tuba3b	22147	6+:145564572-145569938
9764	D330017J20Rik	320609	6+:29867011-29907867
9765	Pcnxl3	104401	19-:5664640-5688149
9766	MMT00050414		4+:119193594-119193965
9767	Vps13b	666173	15+:35384711-35401819
9768	MMT00025372		18-:86410461-86412975
9769	MMT00068055		9-:120664093-120664643
9770	Bcmo1	63857	8+:119619764-119657374
9771	Msra	110265	14-:64741462-65059752
9772	Gmps	229363	3+:63780043-63820699
9773	MMT00068134		8+:107185898-107186994
9774	Mrfap1	67568	5-:37186105-37187943
9775	Trpd52l3	66745	19+:30078279-30080508
9776	EG433184	433184	18+:54143917-54145034
9777	Cdh19	227485	1-:112785786-112826552
9778	MMT00067005		4-:119191741-119192646
9779	Laptm5	16792	4+:130469208-130490946
9780	4930583H14Rik	67749	3-:51192337-51200431
9781	Prune	229589	3-:95057593-95085714
9782	Dnase2a	13423	8+:87432544-87435350
9783	Sfrs15	224432	16-:90229201-90285751
9784	Jakmip1	76071	5+:37419534-37534521
9785	Olfr1259	258338	2-:89783342-89784270
9786	MMT00042592		X-:86005115-86143830
9787	Epb4.1l5	226352	1-:121491653-121545194
9788	Olfr1197	433449	2-:88568832-88586903
9789	Rad51ap1	19362	6-:126874103-126889567
9790	Tspan10	208634	11+:120304006-120308263

9791	Gtf3a	66596	5+:147760150-147767390
9792	Miox	56727	15+:89164904-89167442
9793	Mrpl52	68836	14+:55045691-55048590
9794	Med6	69792	12-:82674551-82695989
9795	Afg3l2	69597	18-:67564420-67608824
9796	MMT00038636	553095	13-:84164902-84204379
9797	MMT00040567		2+:169326047-169330342
9798	MMT00072061		16+:97807935-97808963
9799	MMT00046199		
9800	Phf7	71838	14-:32050881-32064404
9801	MMT00017259		8-:36115184-36116498
9802	Ttc22	230576	4+:106295073-106311803
9803	Abcb8	74610	5+:23900213-23909795
9804	Sdccag1	66244	12-:70412033-70458154
9805	Tagap	72536	17+:8118884-8127761
9806	Klk1b4	18048	7+:51462808-51467114
9807	MMT00073867		11+:66377108-66377936
9808	MMT00015612		4+:116121392-116122314
9809	Tox2	269389	2+:163140046-163148745
9810	AI836003	239650	15-:97998179-98000563
9811	Olfr1145	258317	2+:87649978-87650956
9812	Atf1	11908	15+:100058499-100091674
9813	Cdh18	320865	15+:23103515-23191938
9814	Nbl1	17965	4-:138638206-138648780
9815	1700030K09Rik	72254	8+:74967818-74982668
9816	BC005537	79555	13+:24895151-24905753
9817	Fbxo43	78803	15-:36080958-36094575
9818	1810034K20Rik	67881	14-:56277820-56279211
9819	MMT00005703		12+:117053148-117053784
9820	BC023105	207269	18+:60598948-60603504
9821	Pmaip1	58801	18+:66618257-66625212
9822	Celsr1	12614	15-:85729358-85864207
9823	2210009G21Rik	74243	2+:136717998-136894129
9824	MMT00048830		16-:8623204-8625406
9825	MMT00007741		2+:121544265-121544901
9826	Wdfy1	69368	1-:79722990-79758307
9827	Pam	18484	1-:99717671-99992086
9828	Brwd2	207425	7+:136735449-136779254
9829	Stoml1	69106	9+:58104435-58110325
9830	Rbks	71336	5-:31926808-32000001
9831	Nedd4l	83814	18+:65183547-65372961
9832	Brf1	72308	12-:114198255-114238612
9833	Mfn1	67414	3+:32428401-32477141
9834	Gm1140	382217	X+:64948872-64959272
9835	Fastkd1	320720	2-:69528118-69550456
9836	Oprk1	18387	1+:5578572-5592945
9837	Gpm6b	14758	X+:162676903-162826919
9838	Hpgd	15446	8+:58773394-58799471
9839	Ift20	55978	11+:78345046-78354975
9840	Dmxl1	240283	18+:49992566-50110978
9841	MMT00050244		12+:3222416-3228151

9842	Qdpr	110391	5-:45825293-45841396
9843	Olfr642	258326	7-:111197922-111198866
9844	9230110C19Rik	234912	9-:8021490-8042839
9845	Pde6a	225600	18+:61380152-61448437
9846	Gm1524	385253	6-:69557261-69557795
9847	Ifi27	76933	12-:104680371-104681869
9848	Smad3	17127	9-:63497477-63605725
9849	4930445E18Rik	78115	15-:25225222-25262405
9850	MMT00023282		3-:137307006-137307772
9851	Barhl1	54422	2-:28764556-28771207
9852	Ilkap	67444	1-:93272408-93295311
9853	MMT00028663	100042811	7-:112838553-112839091
9854	Enpp3	209558	10-:24536932-24555942
9855	MMT00079728		2+:58190730-58191342
9856	Tnf	21926	17-:35336337-35338941
9857	Fgfbp3	72514	19-:36992954-36993691
9858	MMT00061598		X+:148867418-148868269
9859	Cx3cl1	20312	8+:97296164-97306280
9860	MMT00060113		9+:38881607-38883427
9861	Serpinb6b	20708	13+:33057381-33070905
9862	AW112010	107350	19-:11122098-11125056
9863	MMT00020611		1+:178765656-178768379
9864	Npy1r	18166	8+:69227746-69230007
9865	Hhatl	74770	9-:121693139-121701502
9866	4930455B06Rik	223825	15+:4848737-4868376
9867	Psrc1	56742	3+:108186760-108191080
9868	Sult4a1	29859	15-:83907820-83936057
9869	Gstt4	75886	10-:75277690-75285265
9870	Mgat2	217664	12+:70285199-70287227
9871	C1qdc2	67389	4+:155336426-155340738
9872	Fgf9	14180	14+:58691754-58728487
9873	Ntrk3	18213	7-:85595661-85722669
9874	MMT00056829		17+:22151966-22155540
9875	Cdk5rap3	80280	11-:96769100-96777795
9876	Tmem20	240660	19+:38474921-38477867
9877	Cdc2a	12534	10-:68801052-68815664
9878	Spaca3	75622	11+:80671863-80681321
9879	RP23-451D4.4	277668	4-:143245211-143250501
9880	Leo1	235497	9+:75293231-75314236
9881	Zcchc2	227449	1+:107887759-107928183
9882	OTTMUSG000000	626870	11+:8952696-8968736
9883	Pou2f3	18988	9-:42935469-43013832
9884	Hp	15439	8-:112099027-112102948
9885	4930401F20Rik	243822	7-:4704830-4709462
9886	Degs1	13244	1-:184206109-184212875
9887	MMT00062394		7+:143144480-143145309
9888	Cd200r2	271375	16+:44909258-44914783
9889	Hecw1	94253	13-:14322916-14470933
9890	Cdk3	69681	11+:116077853-116081599
9891	MMT00001343	74100	9-:111967762-112090230
9892	AU022252	230696	4-:118898347-118905280

9893	Dph3	105638	14-:32893701-32898877
9894	Marveld2	218518	13-:101365915-101387005
9895	Nlgn1	192167	3-:25330763-25338999
9896	Cdh13	12554	8+:120807644-121847354
9897	MMT00002888	233637	7+:111969665-111970861
9898	Traf7	224619	17-:24646475-24664793
9899	D13Wsu177e	28126	13-:54685551-54691435
9900	MMT00062271		14-:118774751-118775110
9901	Edn3	13616	2+:174586119-174607492
9902	Armc5	233912	7+:135381536-135388607
9903	Il22	50929	10+:117641997-117647100
9904	Hbegf	15200	18-:36664586-36675428
9905	MMT00044357		1+:35863811-35867309
9906	Ninj2	29862	6+:120043367-120150356
9907	4932442K08Rik	67544	17+:15539487-15569002
9908	Slc2a13	239606	15-:91102586-91403660
9909	MMT00077883		3+:94257254-94258001
9910	EG666644	666644	1+:43327668-43359076
9911	Inpp4a	269180	1+:37414763-37465103
9912	Ttc27	74196	17+:75117088-75262909
9913	Tbc1d25	209815	X-:7731577-7753049
9914	Sf3b4	107701	3+:95976449-95976966
9915	A630033H20Rik	213438	X+:104344375-104367431
9916	2510048L02Rik	67119	9+:21731914-21740316
9917	Syn3	27204	10-:85518253-85961584
9918	Mki67	17345	7-:142881466-142907822
9919	Gabarapl1	57436	6+:129483464-129491184
9920	Syk	20963	13+:52678541-52744147
9921	Sec24d	69608	3+:122970384-123068560
9922	Inpp5f	101490	7+:135754814-135839949
9923	Tssk6	83984	8+:72426113-72427415
9924	MMT00068388		18-:90187323-90188462
9925	Apoe	11816	7-:20281619-20283579
9926	Snx18	170625	13-:114382392-114408753
9927	Creb3l2	208647	6-:37305642-37392129
9928	Lgmn	19141	12-:103632308-103677907
9929	Mthfsd	234814	8-:123607721-123632284
9930	Phyhip	105653	14+:70857337-70868627
9931	Glt25d1	234407	8+:74134896-74148808
9932	BC059842	230676	4-:118075007-118078156
9933	Katnb1	74187	8+:97605101-97668834
9934	MMT00002304		X-:13504412-13506637
9935	Prnd	26434	2+:131776597-131781865
9936	Speer7-ps1	75858	5+:15218736-15238478
9937	Rtp2	224055	16-:23927293-23930784
9938	MMT00012610		19-:13176870-13177756
9939	Pcnt	18541	10-:75847607-75905657
9940	Tmbim1	69660	1-:74335992-74341973
9941	RP23-394O9.3	58251	11-:118193673-118203814
9942	AK220484	381157	18+:10458222-10557438
9943	Itga10	213119	3+:96451002-96467319

9944	Cntfr	12804	4-:41604529-41644072
9945	Mrpl38	60441	11-:115993135-116000069
9946	Stam2	56324	2-:52550083-52601198
9947	Pramel1	83491	4+:142984330-142990057
9948	MMT00011685		11-:89914224-89924189
9949	Zfp364	67845	3+:96531603-96595075
9950	Mgat4b	103534	11+:50045540-50048605
9951	Rpp21	67676	17-:36392617-36394791
9952	Nrp1	18186	8+:130883139-131027247
9953	Abca16	233810	7+:127567216-127596652
9954	MMT00047280		X-:23931485-23931605
9955	Psmg2	107047	18+:67801252-67813816
9956	Lhx4	16872	1-:157548853-157572091
9957	Ythdf2	213541	4-:131742674-131767972
9958	2310026E23Rik	67621	4+:111087611-111132902
9959	Ecm1	13601	3-:95538071-95543485
9960	Stk16	20872	1+:75207435-75210813
9961	Sox3	20675	X-:58146309-58160279
9962	Krt23	94179	11-:99339287-99354424
9963	Rcn3	52377	7-:52338283-52347529
9964	EG667705	667705	5+:5567458-5579987
9965	MMT00013269		11+:98010375-98011338
9966	Rshl3	212892	10+:33625228-33634371
9967	Dtymk	21915	1-:95697072-95698452
9968	Eif3s10	13669	19-:60842234-60851681
9969	MMT00005446		7+:12210392-12211682
9970	MMT00082622		2+:40411263-40412198
9971	Nqo1	18104	8-:109912124-109927166
9972	Aicda	11628	6+:122503835-122514197
9973	Tmem5	216395	10-:121517989-121534226
9974	MMT00023592		12-:116699302-116842584
9975	Srfbp1	67222	18+:52625346-52650392
9976	Trnt1	70047	6+:106719130-106732466
9977	Zc3h6	78751	2+:128823340-128844729
9978	Lrrc16b	268747	14+:56118294-56120430
9979	Myo1b	17912	1-:51806832-51964160
9980	MMT00008758		1+:98542150-98543132
9981	Snrpn	20646	7-:67128007-67133633
9982	MMT00002004		1+:12555611-12556670
9983	Dnajc8	68598	4+:132091475-132109659
9984	EG215895	215895	10-:33487976-33499645
9985	D16Ertd519e	52645	16+:70618318-70625069
9986	Wdr35	74682	12+:8980816-9035646
9987	Ankrd36	76389	11+:5528730-5545156
9988	Akap6	238161	12+:53896899-54243756
9989	MMT00027411		9-:44471843-44472816
9990	MMT00081303		5-:33773383-33774239
9991	Dph1	116905	11-:74991145-75003976
9992	Paqr4	76498	17-:23873429-23877218
9993	2210010N04Rik	70381	5-:144955316-144984476
9994	Cd209b	69165	8-:3918483-3926757

9995	MMT00046202		X+:91557345-91558125
9996	Rassf9	237504	10+:101974918-102008830
9997	Nr0b2	23957	4+:133109339-133112150
9998	Hfm1	330149	5-:107269612-107274495
9999	Zswim3	67538	2+:164630598-164647626
10000	Ptger4	19219	15-:5183864-5193831
10001	Pygm	19309	19+:6384428-6398458
10002	Krt79	223917	15-:101759764-101770755
10003	B4galt7	218271	13+:55701471-55711314
10004	Nmt1	18107	11+:102889864-102927116
10005	Dhx34	71723	7-:16783903-16784304
10006	Ephx2	13850	14-:66703210-66743289
10007	MMT00033070		2-:97788230-97789852
10008	Etf1	225363	18-:35064135-35091584
10009	MMT00033820		9+:79194550-79195363
10010	Iqcf4	67320	9-:106470651-106473297
10011	Brp44	70456	1+:167391338-167411345
10012	MMT00077406		11+:94603199-94604328
10013	2010005J08Rik	72046	11-:5613427-5641147
10014	MMT00041851		
10015	MMT00065088		12-:74944110-74964607
10016	MMT00067746		15-:53866942-53867404
10017	Wdr73	71968	7-:88035610-88046123
10018	Olfr1417	258938	19-:11902567-11903514
10019	Sprr2k	20765	3+:92237266-92237471
10020	MMT00058547	435962	7+:34188148-34209065
10021	Oog2	381570	4+:143780632-143786149
10022	EG433161	433161	18+:9916922-9917757
10023	Prkcz2	404705	7-:28667425-28669553
10024	MMT00000014		17-:50366966-50367519
10025	A130082M07Rik	100038850	14+:54787766-54843877
10026	Ndufc2	68197	7+:104548601-104556304
10027	Mcm9	71567	10-:53256174-53283263
10028	Chchd6	66098	6-:89333149-89545631
10029	Trak1	67095	9+:121273118-121384034
10030	Gba	14466	3+:89006938-89012594
10031	Zbtb22	81630	17+:34053827-34055743
10032	Sele	20339	1+:165978364-165987808
10033	Ccdc109a	215999	10-:58911228-58930623
10034	2010111I01Rik	72061	13+:63252861-63401173
10035	MMT00041814		8+:38217794-38218856
10036	Nppa	230899	4+:147374756-147376234
10037	Usp51	434872	X+:149442688-149443965
10038	Speer6-ps1	73266	13+:3148720-3188924
10039	V1rh5	171248	13+:23126206-23127308
10040	MMT00080694		4+:60449061-60453064
10041	Ccdc38	237465	10+:93003356-93046117
10042	MMT00028111		8-:100747156-100748701
10043	Shmt1	20425	11-:60610746-60624593
10044	1810074P20Rik	67490	4-:25177296-25208909
10045	Kcnip2	80906	19-:45868159-45890293

10046	MMT00015879		2-:11020493-11021905
10047	Susd5	382111	9+:113966593-114006028
10048	Olfr1347	258383	7-:6440624-6441583
10049	V1rc18	171191	6-:57905636-57906525
10050	Olfr154	27216	2-:85503634-85504589
10051	Mylk2	228785	2+:152737775-152747890
10052	6530404N21Rik	67795	19-:33212236-33277138
10053	MMT00002151		14+:67587971-67588594
10054	Fsip1	71313	2-:118030623-118082690
10055	Tpte	234129	8+:23438115-23476478
10056	Cmtm3	68119	8+:106864586-106871571
10057	Sult6b1	73671	17-:79284235-79306332
10058	Ahsg	11625	16+:22892114-22899511
10059	Olfr1469	258690	19+:13485060-13485990
10060	AI747448	99709	3-:144573884-144595499
10061	Nsd1	18193	13+:55417058-55419683
10062	Pfas	237823	11-:68801073-68817378
10063	Mdn1	100019	4+:32743778-32753548
10064	Mbip	217588	12-:57429306-57446855
10065	9030625A04Rik	210808	14-:77424008-77430769
10066	Krtap8-2	16704	16-:88896212-88896695
10067	Ppapdc1a	381925	7+:136400356-136534568
10068	2310051M13Rik	70202	13-:60899612-60901211
10069	Alkbh1	211064	12-:88768070-88784785
10070	V1rc8	113865	6-:57928387-57929296
10071	MMT00001947		5-:73017832-73019356
10072	Gipc2	54120	3-:151756806-151828864
10073	2010321M09Rik	69882	9+:64808690-64834780
10074	Adat1	30947	8-:114492145-114514296
10075	MMT00075669		15+:77273760-77274850
10076	Cyp2c68	433247	19-:39763326-39815544
10077	Cep63	28135	9-:102488914-102528545
10078	Tbc1d14	100855	5-:36835182-36928876
10079	Abcb4	18670	5+:8893716-8959225
10080	Top2b	21974	14+:17198146-17263301
10081	Fth1	14319	19+:10057192-10059582
10082	Insr	16337	8-:3155669-3279176
10083	Med16	216154	10-:79357455-79371665
10084	Rbm7	67010	9-:48296813-48303410
10085	5730590G19Rik	77011	7+:86822207-86829355
10086	Mfap3l	71306	8+:63134351-63151550
10087	Bmp3	110075	5+:99284131-99309299
10088	Ddc	13195	11-:11714445-11780653
10089	MMT00006552		7-:32255955-32256416
10090	Dpagt1	13478	9+:44135076-44141678
10091	EG114600	114600	6-:132311063-132314138
10092	Cdh7	241201	1+:111880220-112035766
10093	Olfr985	258854	9-:39934609-39935544
10094	MMT00022290		13-:65524617-65833379
10095	Ranbp2	19386	10+:57909727-57956714
10096	Eef1a1	13627	9-:78248867-78329479

10097	Smndc1	76479	19-:53453702-53465067
10098	2610507B11Rik	72503	11+:78075293-78093420
10099	Smyd3	69726	1-:180887389-181448165
10100	Mterfd2	69821	1-:95197806-95201342
10101	Frmpd3	245643	X+:136900194-136969705
10102	Lman2l	214895	1-:36480029-36502105
10103	Asxl3	211961	18+:22610871-22684048
10104	Tcp10a	21460	17+:13254056-13569627
10105	A630054L15Rik	211922	14+:27399063-27447894
10106	Bglap-rs1	12095	3-:88172543-88173478
10107	Pdx1	18609	5+:148081534-148086732
10108	Ctsh	13036	9+:89949346-89970927
10109	Gbp3	55932	3+:142223571-142236171
10110	MMT00044983		17-:70742463-70742806
10111	BC017612	170748	9+:15309938-15349701
10112	Kcns1	16538	2-:163990041-163996825
10113	Lrrk1	233328	7-:73404068-73410332
10114	Rims1	116837	1-:22278447-22812563
10115	Ptk2	14083	15-:73156178-73161446
10116	Mettl7b	71664	10-:128395332-128398044
10117	Vamp7	20955	X_random-:18581-39151
10118	Il3ra	16188	14+:15179008-15188004
10119	MMT00000256		3+:22971516-22973424
10120	C730025P13Rik	227615	2+:25111189-25111600
10121	Esrrg	26381	1+:189821815-190035141
10122	Wdr3	269470	3-:99953787-99966256
10123	Rbm15b	109095	9-:106782816-106788896
10124	Samd3	268288	10+:25964286-25971719
10125	MMT00002345		X+:18689905-18690211
10126	MMT00058622		15-:3167182-3168954
10127	Tpm1	22003	9-:66875702-66896975
10128	MMT00048557		17-:91191787-91193560
10129	MMT00079932		7+:141851959-141853338
10130	MMT00043239		5-:126505516-126506821
10131	Gdap1	14545	1+:17135465-17154094
10132	Ces2	234671	8+:107370985-107378367
10133	MMT00047183		7-:118530306-118530846
10134	Hivep3	16656	4+:119406376-119545933
10135	MMT00002007		1+:28046952-28048558
10136	RP23-149D11.4	381535	4+:101581725-101582688
10137	Sdad1	231452	5-:92727212-92739053
10138	MMT00020352		10-:110037573-110076696
10139	Hdhd1a	67365	18-:50727309-50728324
10140	Cdc2l6	78334	10+:40020193-40203623
10141	Lysmd4	75099	7+:74367428-74371564
10142	5830405N20Rik	67596	10+:129784191-129799697
10143	Uchl1	22223	5+:67067526-67078472
10144	Dgcr6	13353	16+:18066036-18072177
10145	2700038C09Rik	66496	2+:180922047-180923209
10146	Jmjd1b	277250	18+:34952657-34999023
10147	Itgax	16411	7+:135273081-135294170

10148	Cecr5	214932	6-:120459512-120481317
10149	EG631319	631319	X+:92035055-92035686
10150	Oxct1	67041	15+:3976723-4103963
10151	4930538K18Rik	75180	4-:118877660-118890781
10152	Ptpn2	19255	18-:67825166-67884231
10153	Phactr3	74189	2+:177853904-177883498
10154	Nrgn	64011	9-:37352077-37360394
10155	Slc6a5	104245	7+:57167069-57214794
10156	Htr2c	15560	X+:143397188-143630220
10157	MMT00060350	435755	3+:129136789-129137570
10158	Nat1	17960	8+:70014656-70016000
10159	Msh3	17686	13-:93110714-93123989
10160	Olfr23	18321	11+:73753749-73754725
10161	Jtb	23922	3+:90035534-90039771
10162	Pde3a	54611	6+:141197789-141447872
10163	Centg2	347722	1+:91662879-91787524
10164	2310004N24Rik	69535	11+:116060194-116076631
10165	Parp1	11545	1+:182499105-182531384
10166	Rhbdd2	215160	5+:136108591-136120016
10167	Otop3	69602	11+:115206180-115208239
10168	Gm1604	381059	17+:7229892-7233990
10169	4933402J07Rik	330820	8+:90109899-90113324
10170	Trpm2	28240	10-:77372904-77431979
10171	MMT00009402		7-:4902648-4902890
10172	Ntf3	18205	6-:126051744-126052520
10173	Nck1	17973	9-:100395796-100446553
10174	Amz2	13929	11+:109287300-109298071
10175	EG245190	245190	7-:106774004-106774675
10176	Snrpg	68011	6+:86321537-86328896
10177	Osgep	66246	14-:51535358-51544503
10178	Dad1	13135	14-:54855161-54873547
10179	EG333669	333669	10+:75429127-75441087
10180	5730455O13Rik	70567	19-:38215869-38298795
10181	Olfr907	258801	9+:38306255-38307186
10182	Nrxn3	18191	12+:91407359-91572599
10183	Tax1bp1	52440	6+:52663741-52716478
10184	MMT00003597		14-:86889696-86911623
10185	MMT00017571		7-:140600470-140600909
10186	2610003J06Rik	72106	17+:25965991-25968709
10187	Mterfd1	66410	13-:67013061-67033996
10188	MMT00067946		17-:54486047-54486918
10189	Cage1	71213	13-:38097917-38108361
10190	Pcdh7	54216	5+:58109338-58120686
10191	Ilf3	16201	9+:21173026-21209765
10192	Narg2	93697	9+:69263523-69280675
10193	Olfr509	258369	7-:115789123-115790088
10194	Nrxn3	18191	12+:90033628-90041839
10195	Klf1	16596	8+:87425826-87429186
10196	Cebpa	12606	7+:35904437-35905626
10197	Kif16b	16558	2-:142444110-142727165
10198	Ddx25	30959	9-:35349516-35365333

10199	MMT00050019	675135	1-:58918096-58933287
10200	Acot7	70025	4-:151551867-151552533
10201	Slc13a5	237831	11-:72064227-72080104
10202	Klhl32	212390	4-:24544417-24609425
10203	Cyp4f16	70101	17+:32673573-32688741
10204	Gpr126	215798	10-:14125152-14154203
10205	Hagh	14651	17+:24987491-25001393
10206	4931428L18Rik	70988	1-:31197922-31279456
10207	MMT00012324	328192	13+:16122413-16123441
10208	6030446N20Rik	338363	18-:12152072-12280024
10209	Plin	103968	7-:86866051-86877780
10210	Sgms1	208449	19-:32198262-32285470
10211	Pfkl	18641	10-:77450651-77472470
10212	4933430H16Rik	71285	7-:90089039-90115727
10213	Eif3g	53356	9-:20698825-20703024
10214	Sectm1b	58210	11-:120915844-120924852
10215	Nampt	59027	12+:33505339-33536472
10216	Krtap16-7	170656	16-:89403605-89404013
10217	Ccl28	56838	13_random-:365830-393924
10218	Vps41	218035	13+:18815054-18958681
10219	Rpl8	26961	15+:76734528-76736742
10220	Baiap2	108100	11+:119804414-119862782
10221	Thsd4	207596	9-:59820204-59988943
10222	AF366264	231201	8-:13836581-13838059
10223	MMT00013190	236598	
10224	Hes7	84653	11+:68933975-68936495
10225	EG668829	668829	9-:115942871-115964997
10226	MMT00023069		8+:19579894-19580597
10227	Pax5	18507	4-:44544378-44723312
10228	MMT00082629		8-:81010016-81010310
10229	Ripk1	19766	13+:34101477-34124881
10230	Yme1l1	27377	2+:23012055-23052877
10231	Abca1	11303	4-:53045810-53156950
10232	Gja10	14610	4-:32687841-32689357
10233	Abi3bp	320712	16+:56477962-56562602
10234	C230093N12Rik	98952	2+:32390814-32423648
10235	Gm973	381260	1+:59581191-59583821
10236	EG665033	665033	9-:105759273-105791705
10237	Lfng	16848	5+:141083330-141090439
10238	Scml2	107815	X+:157562199-157694816
10239	Syt15	319508	14+:35036029-35040925
10240	Cyp2w1	545817	5+:139828600-139833178
10241	Prc1	233406	7+:87439402-87461144
10242	MMT00031517	628236	19-:33574555-33590216
10243	Rgs19	56470	2-:181423482-181428654
10244	1700040N02Rik	73303	2+:170181859-170205935
10245	Dus1l	68730	11-:120650515-120656813
10246	Tm4sf20	66261	1-:82753223-82765031
10247	V1rc20	171193	6+:56952348-56953258
10248	Cwf19l2	244672	9+:3403830-3430474
10249	Cd200r3	74603	16+:44943854-44966403

10250	Pcsk9	100102	4-:106115335-106126971
10251	Ankrd55	77318	13+:113113259-113174201
10252	MMT00056233		X-:135057334-135058076
10253	Rnf24	51902	2-:131128379-131178649
10254	Sprr2j	20764	3+:92222741-92223069
10255	Muc16	73732	9-:18299897-18323792
10256	Gmds	218138	13-:31911458-32430413
10257	Pcyox1l	240334	18-:61856899-61867310
10258	Cisd2	67006	3-:135069978-135086355
10259	Kcnmb4	58802	10-:115854923-115910579
10260	Zfp36l2	12193	17-:84585332-84586477
10261	Lcp1	18826	14+:75597875-75629249
10262	Nxf7	170722	X-:132114092-132124009
10263	Pip5k3	18711	1+:65233275-65320331
10264	MMT00033776		12-:105157763-105164511
10265	Tbce	70430	13-:14089210-14131968
10266	1500041N16Rik	67836	8-:87598955-87604605
10267	Setdb2	239122	14-:60020844-60059618
10268	MMT00001907		9+:9434591-9435005
10269	Rhot2	214952	17-:25975783-25981449
10270	Setd1b	208043	5+:123598088-123607877
10271	Pvrl3	58998	16-:46395117-46496887
10272	MMT00025452		X-:131216044-131216377
10273	Gpt2	108682	8+:88016483-88051465
10274	MMT00076184		9+:40295245-40295903
10275	Trim12	76681	7-:111452110-111463977
10276	Dcun1d1	114893	3-:35794141-35831888
10277	Exoc3	211446	13-:74308609-74346138
10278	Dusp6	67603	10+:98725864-98730118
10279	Rnf186	66825	4+:138523118-138524274
10280	Asnsd1	70396	1-:53401481-53409416
10281	Slc15a1	56643	14-:121858842-121904475
10282	Dhx57	106794	17-:80637651-80675375
10283	Map2k7	26400	8+:4238985-4246173
10284	Ssh2	237860	11+:77161927-77231033
10285	MMT00048888		5+:16880161-16883486
10286	1700109H08Rik	77036	5+:3571145-3584594
10287	Xkrx	331524	X-:130685090-130692024
10288	Piwil2	57746	14-:70772286-70828901
10289	Zfp313	81018	2+:167328967-167340232
10290	Ssty1	20611	Y_random-:9328876-9331261
10291	Arl13a	74448	X+:130722038-130742568
10292	Cobl	12808	11-:12136680-12364750
10293	Cd5	12507	19-:10792636-10813342
10294	D630002G06Rik	236293	19-:8234704-8293388
10295	Prdm2	110593	4-:142701535-142770894
10296	MMT00076996		5-:143898910-143927802
10297	Lypd1	72585	1-:127768860-127807942
10298	Olfr1015	258564	2+:85625669-85626607
10299	Rad52	19365	6+:119852731-119872840
10300	Igfbp4	16010	11+:98902810-98912892

10301	Fut4	14345	9-:14555139-14556440
10302	Rab18	19330	18+:6765258-6788718
10303	Cyp2a22	233005	7-:27716650-27724409
10304	MMT00040510		6-:71622516-71654758
10305	Gabpa	14390	16+:84835361-84860995
10306	MMT00054080		2-:173872230-173872877
10307	Cnot1	234594	8-:98243530-98331362
10308	Fuk	234730	8-:113406369-113413190
10309	Hdac6	15185	X-:7507255-7524370
10310	V1rg4	171239	7-:12515661-12516634
10311	Cpox	12892	16+:58670404-58680473
10312	MMT00041216		6-:88832626-88833199
10313	Ptpn23	104831	9-:110287593-110310621
10314	Ranbp17	66011	11-:33112419-33413626
10315	6720457D02Rik	77117	13-:62471397-62471713
10316	MMT00078795		1+:32760098-32762064
10317	Nmnat2	226518	1+:154920926-154963144
10318	Cdk8	264064	5+:147043070-147114448
10319	Pkhd1l1	192190	15+:44364402-44367451
10320	Cacnb2	12296	2+:14525940-14535983
10321	Gabra3	14396	X-:69679944-69901488
10322	Mcpt2	17225	14+:56660959-56663471
10323	0610009D07Rik	66055	12+:4824357-4834064
10324	Ncln	103425	10-:80948998-80959472
10325	Prmt7	214572	8+:108734953-108775592
10326	E330009P21Rik	320082	9-:109041970-109050742
10327	Hint2	68917	4-:43667100-43669320
10328	Gpr182	11536	10-:127186677-127188749
10329	MMT00077557		5+:22815623-22845021
10330	Lrch3	70144	16+:32914205-33009522
10331	Slc9a4	110895	1+:40637360-40681146
10332	Gnrhr	14715	5-:86611036-86626895
10333	MMT00004363		14+:54164640-54165341
10334	MMT00020302		7+:34097622-34098118
10335	Arsb	11881	13+:94711475-94712970
10336	Sf1	22668	19+:6364087-6376424
10337	Zmym1	68310	4-:126724836-126726950
10338	D630045J12Rik	330286	6-:38076528-38147092
10339	Tusc3	80286	8+:40068961-40250466
10340	Hc	15139	2-:34838850-34916916
10341	Cdc5l	71702	17-:45544909-45552690
10342	Polq	77782	16+:37011899-37094699
10343	Malat1	72289	19-:5801054-5802672
10344	Timp2	21858	11-:118164194-118216621
10345	Camkk1	55984	11+:72832554-72855565
10346	Rerg	232441	6-:137004017-137118214
10347	Atp12a	192113	14+:56983864-57007387
10348	Fut1	14343	7+:52872657-52874161
10349	Rfxdc1	320995	10+:51397583-51435855
10350	Itpkc	233011	7-:27992193-28013506
10351	Clcn4-2	12727	7-:7236790-7252042

10352	MMT00002544		1-:4677963-4679485
10353	Matn4	17183	2-:164215162-164230037
10354	Stx16	228960	2-:173924479-173925271
10355	Bbs4	102774	9-:59169781-59201261
10356	2810417H13Rik	68026	9+:65738235-65749410
10357	Tm4sf1	17112	3-:57091304-57098795
10358	Fgf3	14174	7+:152024612-152028892
10359	Foxf2	14238	13+:31717647-31723271
10360	Nrxn2	18190	19+:6418943-6533208
10361	MMT00008819		5-:28326713-28327260
10362	S100a3	20197	3+:90404136-90406624
10363	Usp24	329908	4+:106060316-106113935
10364	MMT00077722		X+:156474390-156475366
10365	Aplp1	11803	7-:31220007-31230571
10366	4931403E03Rik	75718	4-:138124983-138179928
10367	Coq7	12850	7-:125668576-125676827
10368	Mphosph8	75339	14+:57287084-57316267
10369	Mier2	70427	10-:79002990-79004705
10370	MMT00017441	623205	11+:53014185-53014559
10371	4930485B16Rik	380654	10-:92395300-92403024
10372	MMT00073733		4-:128545718-128546688
10373	Tmem141	51875	2-:25464153-25477523
10374	Trim15	69097	17-:36997637-37004155
10375	Wdr47	99512	3+:108394353-108448634
10376	C920025E04Rik	667803	17-:36264613-36264812
10377	A430105I19Rik	214239	2-:118583232-118585443
10378	Atg5	11793	10+:43988297-44083108
10379	Olfr577	259113	7-:110121565-110122504
10380	MMT00018157		X-:143033206-143035525
10381	Psma5	26442	3+:108060128-108082891
10382	Mtmr4	170749	11+:87405718-87429798
10383	BC023179	232855	7-:7158965-7231218
10384	Chic2	74277	5-:75402448-75440641
10385	Solh	50817	17-:26096501-26102450
10386	Zfp108	54678	7+:25039838-25046949
10387	4933427D06Rik	232217	6+:89046112-89058002
10388	Ccr7	12775	11-:99005512-99016383
10389	Entpd1	12495	19+:40734322-40814218
10390	Zfp708	432769	13-:67175020-67595664
10391	Lin54	231506	5-:100871061-100929661
10392	Gm2a	14667	11+:54911615-54923557
10393	Slc7a8	50934	14-:55341052-55400378
10394	MMT00028435		7-:48581594-48582685
10395	Abcg2	26357	6+:58605631-58642416
10396	MMT00072312		6+:39156261-39157552
10397	Gtf2e2	68153	8+:34846203-34887452
10398	Blk	12143	14-:63991984-64035954
10399	Myef2	17876	2-:124907224-124949254
10400	Pth2	114640	7+:52436551-52437208
10401	Heph	15203	X+:93650799-93769821
10402	Mia1	12587	7-:27964765-27966169

10403	MMT00012905		13-:13934509-13937001
10404	Dlx3	13393	11+:94981635-94985498
10405	Hs6st3	50787	14+:119537523-120269037
10406	Trip10	106628	17+:57388883-57402845
10407	Gpatch3	242691	4+:133138382-133140157
10408	Per1	18626	11+:68914143-68922861
10409	Usp54	78787	14-:21389469-21410835
10410	Dtx1	14357	5-:121130211-121145412
10411	Plag1	56711	4-:3830781-3832855
10412	Nek10	238944	14+:15697906-15698248
10413	Olfr487	258042	7-:115355096-115394259
10414	Jagn1	67767	6+:113392628-113398221
10415	Dcdc2a	195208	13+:25148072-25294336
10416	Eaf2	106389	16-:36793830-36828334
10417	Socs7	192157	11+:97223864-97254942
10418	Fbxo17	50760	7+:29501891-29523157
10419	Ndufaf2	75597	13-:108842780-108948810
10420	Atoh7	53404	10+:62562903-62563351
10421	MMT00016905		11+:53459332-53459823
10422	Birc6	12211	17+:74927634-75102403
10423	1700020O03Rik	70373	12+:87582822-87630305
10424	Pin1	23988	9+:20456554-20468155
10425	MMT00042994		X+:98983274-98983779
10426	Ly6f	17071	15+:75098850-75102555
10427	Ccdc34	68201	2+:109862124-109884993
10428	V1rg9	171244	7-:12845107-12846028
10429	Olfr1219	258901	2-:88914312-88915246
10430	MMT00002908		X-:36258809-36259567
10431	Uba3	22200	6-:97134021-97155337
10432	Pdcd6ip	18571	9-:113564052-113617225
10433	Mettl5	75422	2-:69709051-69723663
10434	Ppp2r5a	226849	1-:193175864-193180833
10435	Nab2	17937	10-:127097974-127103734
10436	EG240055	240055	17+:26568731-26578740
10437	MMT00062592		X-:51119052-51123209
10438	Mgat4c	67569	10+:101840783-101852606
10439	Rps15	20054	10+:79755201-79756857
10440	Olfr1157	258846	2-:87802109-87803047
10441	Lat2	56743	5-:135075891-135090876
10442	Pla2g4a	18783	1-:151676758-151808362
10443	Pvrl2	19294	7-:20302018-20334651
10444	1810020D17Rik	66273	7-:104698852-104727989
10445	Ube2s	77891	7-:4759617-4763930
10446	Pcdhb3	93874	18+:37460636-37463632
10447	Dynll2	68097	11-:87793034-87794338
10448	Rac3	170758	11+:120582914-120585283
10449	Mycbp	56309	4+:123582293-123589439
10450	C330006K01Rik	231855	5+:142950827-142953758
10451	Olfr608	258751	7+:110618554-110619503
10452	Grn	14824	11+:102294285-102297784
10453	MMT00015425		X-:130588298-130589013

10454	Dgkk	331374	X+:6472630-6507098
10455	MMT00015250		X+:150217937-150218848
10456	Sbf2	319934	7-:117451535-117758429
10457	Olfr853	258908	9-:19341454-19342372
10458	Pabpc3	67543	17-:9859361-9862561
10459	Rab24	19336	13-:55421464-55423015
10460	MMT00033553	664779	19-:12838159-12853443
10461	Mybbp1a	18432	11+:72254870-72265051
10462	MMT00070759		7-:12565016-12569261
10463	1700123L14Rik	78482	6-:96114495-96116091
10464	Zfp367	238673	13-:64235482-64254035
10465	BC023892	212943	9-:85217149-85220900
10466	S1pr5	94226	9-:21047360-21050653
10467	MMT00074572	100038888	5+:143419271-143435261
10468	Tspan33	232670	6+:29644255-29668558
10469	Rogdi	66049	16-:5008842-5013612
10470	Zmym4	67785	4-:126539069-126588529
10471	Igh-VJ558	16061	12-:116930076-116930533
10472	Il11ra1	16157	4+:41714422-41715456
10473	Klra8	16639	6-:130065555-130078178
10474	2410131K14Rik	76792	5+:118695269-118713115
10475	Ccna2	12428	3-:36463787-36470860
10476	4930529M08Rik	78774	2+:145760439-145927787
10477	Trpc4ap	56407	2-:155460044-155518068
10478	Zcchc11	230594	4+:108132030-108230109
10479	Pld2	18806	11+:70353665-70371612
10480	Dcc	13176	18-:71418645-72510232
10481	Tars2	71807	3-:95543898-95558785
10482	Mcat	223722	15-:83377225-83379765
10483	Crot	74114	5-:8966047-8997144
10484	Dnajb11	67838	16+:22869210-22872439
10485	Pitpna	18738	11+:75401609-75442280
10486	1300002K09Rik	74152	4+:45861818-45899880
10487	MMT00016719		3-:93517601-93518479
10488	MMT00042783		12-:108443456-108444324
10489	Aldh1b1	72535	4+:45811998-45817473
10490	Peg3	18616	7-:6660217-6664362
10491	Acpl2	235534	9-:96722838-96789843
10492	1810046K07Rik	69809	9-:51097798-51137024
10493	Hbq1	216635	11+:32200065-32200873
10494	Arhgef10l	72754	4-:140070398-140221778
10495	Grrp1	72690	4-:133807265-133827221
10496	Nsun5	100609	5+:135845829-135852012
10497	Man2b1	17159	8+:87607228-87621415
10498	Ube2z	268470	11-:95908740-95926641
10499	Camkk2	207565	5-:123183212-123214303
10500	H2-M10.5	224761	17+:36860966-36912013
10501	Zfp36	22695	7-:29161802-29164275
10502	Axud1	215418	9-:119880282-119883533
10503	MMT00043055		10+:31358888-31360169
10504	Ripk3	56532	14-:56403833-56407623

10505	MMT00065797		8-:33993977-33995118
10506	Rars2	109093	4+:34562218-34607659
10507	Clec2g	70809	6+:128884398-128934489
10508	MMT00042564		X-:86000484-86002007
10509	Olfr1431	258409	19+:12250062-12284994
10510	Grm7	108073	6+:111157736-111204126
10511	Tmem68	72098	4-:3476389-3501947
10512	MMT00058467	238427	12-:115251355-115645172
10513	Cttnbp2	30785	6-:18317370-18377886
10514	Zp1	22786	19-:10988778-10995122
10515	Klhl4	237010	X+:111588032-111673398
10516	Prr6	73139	11-:62338444-62352764
10517	Gpr75	237716	11+:30785442-30792717
10518	Fntb	110606	12+:77938453-78022399
10519	MMT00061955		5-:66111856-66113448
10520	Xkr6	219149	14+:64413856-64438627
10521	Ttf2	74044	3-:100742784-100773576
10522	Zbtb40	230848	4-:136538970-136551627
10523	Pou4f3	18998	18+:42554296-42555657
10524	MMT00057407		X+:77149783-77150153
10525	MMT00036668		X-:136980119-136980473
10526	MMT00036239		7-:61861136-61866491
10527	Runx1t1	12395	4+:13711928-13818264
10528	Pip5k1b	18719	19-:24369369-24518175
10529	0610025P10Rik	216860	11+:69715378-69727318
10530	Cmklr1	14747	5-:114063832-114105083
10531	Mlana	77836	19+:29772311-29782937
10532	MMT00057790		X-:114777483-114778383
10533	MMT00057494		6-:43716683-43717314
10534	MMT00047748		X-:8232324-8240974
10535	Cby1	73739	15+:79489681-79498092
10536	MMT00054230		
10537	BC066028	407812	7-:147996905-148008079
10538	MMT00051230		10-:7042944-7043492
10539	Unc45b	217012	11+:82725180-82756641
10540	AI851790	268354	10+:123141414-123142884
10541	Ap2a2	11772	7+:148748107-148817541
10542	Hao3	56185	3-:98678630-98697149
10543	BC030307	103220	10+:86193817-86199744
10544	Gdpd1	66569	11-:86847369-86887593
10545	Tmem147	69804	7-:31512734-31514502
10546	Prpf6	68879	2+:181336096-181390365
10547	Maml3	433586	3-:51659420-51660998
10548	MMT00028010		19-:5131907-5132748
10549	C330005M16Rik	101744	7-:29392341-29416033
10550	Rchy1	68098	5-:92377867-92391790
10551	Them5	66198	3+:94146020-94151266
10552	Cd74	16149	18+:60963406-60971580
10553	Dusp23	68440	1-:174560901-174563129
10554	ENSMUSG00000(	100042314	9+:78137728-78153330
10555	A630007B06Rik	213993	19-:56861973-56896634

10556	BC025575	217219	11-:102298615-102308941
10557	2310022M17Rik	69556	11-:31565164-31571863
10558	Cyp2d40	71754	15-:82590264-82594613
10559	Cntrob	216846	11-:69113007-69137364
10560	Nudt14	66174	12-:114172503-114180354
10561	Eln	13717	5-:135179582-135223111
10562	Tusc2	80385	9+:107465700-107468441
10563	MMT00052891		19+:13800713-13801703
10564	Gm804	328121	12+:71264364-71274428
10565	Ccdc67	234964	9-:15365009-15416600
10566	MMT00009174		8-:24068600-24069092
10567	Lass5	71949	15-:99566021-99602873
10568	Rage	26448	12-:112046008-112079149
10569	Zbtb38	245007	9-:96585855-96589499
10570	Ddi1	71829	9-:6265141-6266548
10571	Olfr1228	258973	2-:89088879-89089849
10572	EG384244	384244	5+:139296797-139297521
10573	6230416J20Rik	230376	4-:86228784-86246702
10574	MMT00007972		6+:67741039-67741528
10575	MMT00036467	100041615	5+:109402333-109404896
10576	Xrcc3	74335	12-:113041403-113052052
10577	4930412O13Rik	381347	2+:9802510-9811684
10578	Acot10	64833	15-:20594970-20596505
10579	MMT00029650		6-:131383131-131403560
10580	Bhlhb4	140489	2-:180510673-180511454
10581	Slc2a8	56017	2-:32829083-32837558
10582	Psmg3	66506	5-:140299547-140302781
10583	Mad2l1	56150	6+:66485472-66489946
10584	Krtap5-1	50774	7-:149482282-149482974
10585	MMT00038004		3+:108684744-108685613
10586	Tlr5	53791	1+:184902307-184906558
10587	Thtpa	105663	14+:55713672-55717818
10588	4930578E11Rik	78058	18-:29607479-29611589
10589	Olfr159	29849	4-:43782922-43783881
10590	Hsf1	15499	15+:76307864-76331584
10591	Pmp22	18858	11+:62945013-62973048
10592	Olig3	94222	10+:19076344-19078411
10593	Wdr53	68980	16+:32247617-32257166
10594	Tcrb-V8.3	21608	6+:41066034-41066474
10595	2810422O20Rik	69962	1+:165925045-165927376
10596	Nfrkb	235134	9+:31193719-31228921
10597	Gats	80909	5+:134575580-134614427
10598	MMT00011231		X-:162920153-162921549
10599	Ube1y1	22202	Y+:155155-180667
10600	Olfr1414	259041	1-:94407664-94408603
10601	D330028D13Rik	231946	6+:49317816-49339276
10602	MMT00042056	623374	6+:124201603-124202572
10603	MMT00015989		
10604	Uimc1	20184	13-:55129468-55201429
10605	A430108E01Rik	384382	8-:19994754-20020372
10606	Apbb1ip	54519	2+:22629960-22731172

10607	MMT00008516		2+:142398253-142403592
10608	Mospd1	70380	X-:50698197-50723669
10609	Olfr464	258407	11-:87727464-87759894
10610	Nbeal1	269198	1+:60307878-60388635
10611	MMT00013136		
10612	Slc24a6	170756	5+:120961200-120984028
10613	Armc8	74125	9-:99380022-99469320
10614	Olfr644	259125	7-:111216599-111217543
10615	9430073N08Rik	77296	10-:51305225-51310325
10616	Pkn1	320795	8-:86193564-86222886
10617	Rlbp1	19771	7-:86519756-86529671
10618	Avpr1b	26361	1+:133496317-133506322
10619	MMT00007166		X-:115811627-115812487
10620	Hgs	15239	11+:120328998-120344892
10621	4833420G17Rik	67392	13+:120251565-120258567
10622	Plagl2	54711	2-:153057048-153061565
10623	Rpl28	19943	7+:4744572-4746149
10624	BC021381	212483	13-:55640677-55650821
10625	Ddx49	234374	8-:72786603-72826349
10626	Riok3	66878	18+:12287419-12313836
10627	MMT00010914		13-:33309118-33309544
10628	Bpi	329547	2+:158101655-158110267
10629	BC021614	225884	19-:4057486-4059294
10630	Fbxw17	109082	13-:50528547-50529149
10631	4930455B06Rik	223825	15+:4873609-4875728
10632	Anxa8	11752	14+:34899174-34913752
10633	MMT00021266		17-:74219824-74220690
10634	Kitl	17311	10+:99478457-99563045
10635	Itfg2	101142	6-:128359462-128374928
10636	Mal2	105853	15+:54403040-54434395
10637	Olfr495	258361	7+:115538635-115539627
10638	Gpr97	54672	8+:97564185-97569143
10639	Tgfbr3	21814	5-:107535590-107718704
10640	Nup93	71805	8+:96738492-96841127
10641	Hpdl	242642	4-:116492508-116493928
10642	Ngef	53972	1-:89373412-89406921
10643	Gc	14473	5-:89846537-89886926
10644	Usf2	22282	7-:31731014-31741798
10645	Recql	19691	6-:142310669-142335556
10646	Topors	106021	4-:40206639-40216822
10647	MMT00032809		1+:165659708-165660713
10648	Atp6ap2	70495	X+:12165013-12193866
10649	Nodal	18119	10+:60881047-60887383
10650	Atf7	223922	15-:102367084-102455852
10651	2610044O15Rik	72139	17-:95214567-95233779
10652	Pax4	18506	6-:28392344-28438525
10653	Olfr614	259104	7+:110700300-110701253
10654	Lrrc16b	268747	14+:56120666-56127103
10655	Ccdc62	208908	5+:124388158-124411347
10656	2900092C05Rik	73090	7+:13097865-13141665
10657	Rnf12	19820	X-:101157466-101176587

10658	Macrod2	72899	2+:142035834-142060074
10659	ENSMUSG00000(	100038725	10-:52997850-53099484
10660	Gtf3c4	269252	2-:28680886-28695860
10661	Unc5d	210801	8-:29763239-30001982
10662	Palld	72333	8-:63990051-64069974
10663	Sytl2	83671	7+:97497208-97558948
10664	Txn2	56551	15-:77745477-77759387
10665	Il2	16183	3-:37019992-37024874
10666	Npl	74091	1-:155350145-155396844
10667	H2-Eb1	14969	17+:34442886-34453144
10668	Kars	85305	8-:114517343-114535168
10669	Spry1	24063	3+:37538897-37543519
10670	Col23a1	237759	11+:51103766-51130466
10671	Asz1	74068	6-:18021960-18059036
10672	BC031353	235493	9+:74800803-74880259
10673	MMT00071544		X+:117080065-117081181
10674	Antxr2	71914	5-:98313707-98459568
10675	Mobkl2a	208228	10-:80147456-80164582
10676	Ushbp1	234395	8-:73909205-73919709
10677	Olfr812	258791	10-:129279164-129280096
10678	Odz2	23964	11-:35821163-36978177
10679	MMT00028401		6-:68654503-68654990
10680	Slc12a2	20496	18+:58038331-58106459
10681	Dgkz	104418	2-:91772978-91803812
10682	EG627782	627782	X+:7678553-7683362
10683	Tbrg4	21379	11-:6515601-6521080
10684	Hmgb1	15289	5-:149860314-149861889
10685	EG330503	330503	7+:30959943-30964222
10686	MMT00001732		3-:11658663-11659505
10687	Itpkb	320404	1+:182262242-182352541
10688	Mettl3	56335	14-:52914342-52924758
10689	Pitpnb	56305	5+:111759814-111815537
10690	3110039I08Rik	73144	9+:41184478-41240162
10691	Notum	77583	11-:120515124-120522180
10692	Gcc1	74375	6-:28367828-28378390
10693	Acd	497652	8-:108222055-108224983
10694	MMT00075660		16-:84513293-84514457
10695	MMT00075236		5+:67281488-67282441
10696	Gm1123	382097	9-:98907197-98911555
10697	Nvl	67459	1-:183022932-183074320
10698	MMT00049072		
10699	Pga5	58803	19-:10743561-10752470
10700	Grwd1	101612	7-:53080584-53086157
10701	4933407N01Rik	66753	11-:30830772-30854125
10702	Gm687	272465	8+:13435519-13457094
10703	Sema4d	20354	13-:51797860-51820744
10704	ENSMUSG00000(	408192	1-:32576339-32577718
10705	MMT00033076		X-:77779080-77780619
10706	Pla2g2f	26971	4-:138307980-138313512
10707	Abhd10	213012	16-:45731816-45743030
10708	Msh5	17687	17-:35165551-35183567

10709	Zfp804a	241514	2+:82096371-82100032
10710	Smoc1	64075	12+:82127817-82287387
10711	1700108F19Rik	73272	14+:77077372-77086911
10712	Fchsd2	207278	7+:108257309-108432912
10713	Tnfaip8l3	244882	9-:53874882-53916174
10714	Dtnb	13528	12+:3572522-3781505
10715	Lrrc8b	433926	5+:105908910-105911060
10716	2810021B07Rik	66308	13+:17787251-17789095
10717	Ranbp9	56705	13-:43498750-43576342
10718	Klhl30	70788	1+:93247662-93258984
10719	Zbtb17	22642	4+:141000598-141023144
10720	Mpp1	17524	X-:72355613-72376345
10721	1700058C13Rik	73388	2+:153956079-153964092
10722	MMT00070532	100037278	8+:74123074-74126165
10723	4930556N09Rik	75301	10+:96498493-96523693
10724	Colec10	239447	15+:54242323-54294138
10725	Sfmbt1	54650	14+:31579185-31631149
10726	MMT00015194		4+:98507653-98523417
10727	Klra3	16634	6-:130273303-130287644
10728	Msi2	76626	11-:88157390-88579415
10729	Fgd2	26382	17+:29497916-29516480
10730	MMT00076744		9-:40569329-40570451
10731	Olfr481	258927	7+:115224330-115225247
10732	Iars2	381314	1-:187126586-187153266
10733	Kctd6	71393	14+:9046659-9056081
10734	Limk1	16885	5-:135131922-135164271
10735	Cox6a2	12862	7-:135349127-135349866
10736	Cyba	13057	8-:124948675-124956827
10737	Esr1	13982	10-:5345029-5636365
10738	Llgl1	16897	11+:60513341-60527687
10739	MMT00000423		8-:115461866-115463039
10740	Klk1b16	16615	7+:51392136-51396974
10741	MMT00044006		2+:21989389-21990730
10742	4930433N12Rik	114673	9-:3133846-3199782
10743	Tlr9	81897	9+:106124928-106129207
10744	C1qtnf5	235312	9+:43909924-43917269
10745	Gale	74246	4+:135519666-135524096
10746	MMT00021766		X+:162923611-162924285
10747	Abcf2	27407	5-:24070271-24083143
10748	Papolg	216578	11-:23763824-23795262
10749	V1re12	171265	7-:27396139-27397097
10750	Wdr43	72515	17+:71965534-72008369
10751	Eif5	217869	12+:112776652-112782827
10752	Dnajc5b	66326	3+:19408625-19510857
10753	MMT00015145		4+:99224642-99320761
10754	Styx	56291	14+:45970901-45993451
10755	Yipf6	77929	X+:96133501-96139374
10756	Gng5	14707	3+:146162765-146168533
10757	Cxcr6	80901	9+:123715592-123720770
10758	Gdf1	14559	8+:72839685-72855486
10759	Rbms1	56878	2-:60590796-60800976

10760	MMT00006242		
10761	Ipo13	230673	4-:117567097-117587003
10762	V1rc25	171198	6+:58215167-58216074
10763	Mink1	50932	11+:70376399-70427980
10764	Pdap1	231887	5-:145889918-145901121
10765	Retnla	57262	16+:48842903-48844574
10766	Usp30	100756	5+:114550443-114571796
10767	Plscr2	18828	9+:92170673-92192544
10768	2310008H09Rik	66356	7-:125989276-125999771
10769	Hnrnpd	11991	5-:100390025-100407572
10770	Sell	20343	1+:165992234-166010802
10771	Nus1	52014	10+:52137405-52156571
10772	Aacs	78894	5+:125956242-125997773
10773	AI118078	244886	9+:55174754-55286148
10774	Ehd3	57440	17+:74154211-74181428
10775	Slc16a1	20501	3+:104442606-104461160
10776	Olfr959	258501	9-:39379908-39380842
10777	Mobp	17433	9+:120063828-120082411
10778	Plb1	665270	5+:32628547-32666725
10779	Nipa2	93790	7-:63187937-63217809
10780	Fcgr2b	14130	1-:172890716-172906164
10781	Aof1	218214	13+:47138929-47154183
10782	4933403O08Rik	71030	X+:109276083-109284377
10783	Brca1	12189	11-:101351082-101413157
10784	Gabra2	14395	5-:71352677-71486839
10785	Ctdspl2	329506	2+:121782188-121839391
10786	Mrps36	66128	13-:101505915-101514590
10787	Ndufb10	68342	17-:24859003-24861370
10788	D430019H16Rik	268595	12+:106692172-106726651
10789	Exoc6	107371	19+:37624938-37758545
10790	4930470H14Rik	75815	17-:4044656-4082095
10791	Mettl8	228019	2-:70802620-70893584
10792	EG436193	436193	X+:8700478-8716194
10793	MMT00080658	71780	8+:73118379-73121187
10794	Bcl2l1	12048	2-:152607845-152656237
10795	MMT00006025		5+:61066399-61067270
10796	Spic	20728	10-:88138016-88145752
10797	Mageb2	17146	X+:88489482-88578695
10798	Syne1	64009	10+:5205528-5216531
10799	Fras1	231470	5+:97173659-97182051
10800	MMT00050549		3+:136611545-136611919
10801	Adam26b	382007	8-:44605469-44613492
10802	Kcnn2	140492	18+:45719807-45845536
10803	Wnt5a	22418	14+:29338328-29340631
10804	Scn9a	20274	2-:66321062-66322421
10805	9930021J03Rik	240613	19+:29789802-29790232
10806	Rnf212	671564	5-:109158389-109178310
10807	1100001E04Rik	75404	X+:46850913-46852235
10808	Rab7l1	226422	1+:133763868-133769462
10809	EG232887	232887	7+:13764167-13765543
10810	MMT00076426		11+:58660659-58673495

10811	Baz1a	217578	12-:55995939-56087338
10812	9330101J02Rik	664857	7-:146845410-146847069
10813	Kif5a	16572	10-:126665440-126700106
10814	Vmn2r96	433070	17+:18723277-18735121
10815	Cpe	12876	8-:67071354-67171837
10816	Fcna	14133	2-:25480188-25483386
10817	Ankrd10	102334	8-:11611582-11635689
10818	Tbc1d30	74694	10-:120784158-120788433
10819	Olfr1014	258562	2+:85616742-85617658
10820	Dsel	319901	1-:113755758-113759391
10821	Pkd2l2	53871	18+:34569076-34602443
10822	E330016A19Rik	214763	9-:78283247-78290696
10823	Dyrk4	101320	6-:126826063-126844858
10824	Vegfa	22339	17-:46155491-46168392
10825	Psg23	56868	7-:19191691-19201850
10826	9230113P08Rik	77908	9+:35716004-35717273
10827	MMT00063568		2-:87040084-87041485
10828	Rsl1d1	66409	16-:11193065-11203428
10829	Chga	12652	12+:103793178-103803237
10830	Rgs21	624910	1-:146366821-146384126
10831	Dmn	233335	7-:74877489-74905060
10832	Lamb1-1	16777	12+:31950161-32014752
10833	MMT00002916		16-:13920403-13921492
10834	Smurf2	66313	11-:106681370-106781568
10835	Kcnd2	16508	6+:21166298-21677340
10836	MMT00002878		6-:47753957-47754114
10837	Zfp52	22710	17+:21672601-21699565
10838	Spdef	30051	17-:27851393-27865834
10839	MMT00037665		7+:39285425-39322096
10840	Rpe65	19892	3+:159262196-159287762
10841	MMT00004529		1-:153367445-153367899
10842	Slc25a25	227731	2-:32270605-32279523
10843	Ccar1	67500	10-:62206677-62255110
10844	Erap1	80898	13+:74783616-74828267
10845	Nthl1	18207	17+:24769654-24775782
10846	Nrp2	18187	1+:62862028-62885440
10847	Dyx1c1	67685	9+:72806624-72820873
10848	Gpbp1	73274	13-:112215891-112280324
10849	9330159F19Rik	212448	10+:28931498-28947150
10850	Zap70	22637	1+:36818698-36839503
10851	Tax1bp3	76281	11+:72990793-72995500
10852	Sgol1	72415	17-:53815384-53828637
10853	MMT00077065		14+:41262450-41262749
10854	MMT00046948		15-:82608356-82612612
10855	5730493B19Rik	70630	9-:106640861-106642631
10856	9930021D14Rik	319259	17+:24610490-24612277
10857	Pdha1	18597	X-:156561573-156576288
10858	MMT00014028		19-:16133958-16134518
10859	Nphp3	74025	9+:103920432-103925135
10860	Lrrc8c	100604	5+:105948489-106037970
10861	Stfa2	20862	16-:36405215-36408419

10862	Zfp703	353310	8+:28087804-28091935
10863	Tssc1	380752	12+:29436684-29552361
10864	Gdf3	14562	6-:122556265-122560105
10865	Cd248	70445	19+:5068119-5070637
10866	Car10	72605	11+:93351802-93463016
10867	Asb16	217217	11+:102130193-102178915
10868	A630042L21Rik	106894	18-:61290930-61336704
10869	Hao1	15112	2-:134323097-134380088
10870	E2f7	52679	10+:110200981-110211883
10871	EG434197	434197	7+:75420881-75503087
10872	9430016H08Rik	68115	1+:57463605-57472746
10873	MMT00025861		
10874	MMT00008912		6+:3238569-3267095
10875	MMT00024931		15-:56614190-56614985
10876	Steap1	70358	5-:5736321-5749309
10877	V1re8	171231	17+:20983514-20984465
10878	Tsnax	53424	8+:127536896-127558089
10879	Slc9a6	236794	X+:53863029-53917399
10880	Klra8	16639	6-:130065236-130079897
10881	Uchl5ip	73738	X-:70682655-70704368
10882	Aqp1	11826	6+:55286425-55298549
10883	Pols	210106	13-:69636823-69646016
10884	Add2	11519	6+:86032773-86068819
10885	Bri3	55950	5+:145016176-145025263
10886	Fndc3b	72007	3-:27360597-27541971
10887	MMT00004348		17+:50070453-50076626
10888	Eya2	14049	2+:165455841-165597226
10889	Adamts15	235130	9-:30706738-30729822
10890	Pot1b	72836	17-:55792703-55794170
10891	Atp6v1a	11964	16-:44087536-44116981
10892	Lipc	15450	9-:70646058-70782386
10893	Gprin1	26913	13-:54838033-54841850
10894	Ngfrap1	12070	X+:132804878-132806494
10895	Lipe	16890	7-:26164980-26173652
10896	Cpn2	71756	16-:30258749-30267620
10897	Olfr315	258531	11+:58591630-58592559
10898	MMT00046650		7+:113010295-113011844
10899	Coro2b	235431	9-:62267342-62384586
10900	MMT00008931		11-:104894552-104895058
10901	2810051F02Rik	72704	17+:12934239-13008423
10902	Orc3l	50793	4-:34518044-34562173
10903	Slco2a1	24059	9+:102910949-102989098
10904	Kri1	215194	9-:21077905-21092395
10905	Chd2	244059	7-:80580620-80685847
10906	Ccdc122	108811	14+:77436573-77512011
10907	MMT00047893		18-:20707088-20707860
10908	Hipk4	233020	7+:28308535-28314817
10909	MMT00041265		6+:94367793-94369492
10910	Cdc25a	12530	9+:109778493-109795283
10911	Mcl1	17210	3+:95462730-95467099
10912	Psme3	19192	11+:101177641-101184850

10913	Crlf3	54394	11-:79860001-79894457
10914	Hoxc9	15427	15+:102811735-102814873
10915	2410002I01Rik	78777	11-:119951654-119960016
10916	Gm973	381260	1+:59663659-59686991
10917	Eppk1	223650	15-:75932645-75943109
10918	Catsperb	271036	12+:102826194-102864215
10919	EG624446	624446	3-:28663354-28664314
10920	Mdn1	100019	4+:32758490-32766544
10921	4832428D23Rik	403183	1-:44263194-44275756
10922	MMT00052412		10-:5787807-5833789
10923	Saps2	71474	15+:89041998-89116823
10924	Actr6	67019	10-:89174717-89195040
10925	Cacnb1	12295	11-:97866239-97883952
10926	P2ry13	74191	3-:59011821-59014805
10927	Hccs	15159	X-:165750740-165757523
10928	Arid1a	93760	4-:133234924-133278954
10929	Klk1b9	13648	7+:51231430-51235746
10930	Efcab4b	381812	6+:127553847-127575940
10931	Pkn3	263803	2+:29933255-29946533
10932	Olfr578	259119	7-:110132736-110133676
10933	MMT00054848		1+:85158595-85165839
10934	Ciz1	68379	2+:32218753-32233817
10935	Chpt1	212862	10-:87951126-87966663
10936	Rnmt	67897	18+:68460024-68481556
10937	Rnf19b	75234	4+:128735383-128763127
10938	Zbtb5	230119	4-:45005867-45025240
10939	Gtf3c3	98488	1-:54453825-54495803
10940	Dcun1d1	114893	3-:35794141-35831888
10941	Pcdh18	73173	3-:49547422-49561184
10942	MMT00022130	676542	X-:106584174-106587483
10943	Vmn2r59	628444	7-:49267395-49302477
10944	Raver1	71766	9-:20871956-20896443
10945	5330431N19Rik	226162	19+:45635096-45641977
10946	Ifitm7	74482	16-:13982891-13984714
10947	Acads	11409	5-:115560308-115569279
10948	MMT00080146		2+:145717814-145718316
10949	Smek2	104570	11+:29072995-29119129
10950	Tmhs	328789	17+:28712676-28720448
10951	Alox5	11689	6-:116360088-116411183
10952	Mrpl37	56280	4-:106728487-106739450
10953	Slc3a2	17254	19-:8782057-8788330
10954	Ubp1	22221	9+:113840769-113886120
10955	Cdkn2a	12578	4-:88920540-88940469
10956	Bnip3l	12177	14-:67606223-67627687
10957	Pgc	109820	17+:47863790-47871431
10958	Ddx20	53975	3-:105481194-105490364
10959	RP23-149D11.7	381536	4+:101660693-101662816
10960	Rap2a	76108	14+:120877692-120878962
10961	Pgd	110208	4-:148524102-148540777
10962	Eif2b2	217715	12+:86560430-86567443
10963	4930562D19Rik	216516	11-:4043578-4046381

10964	Cldn23	71908	8-:36887782-36889533
10965	Ppp1cb	19046	5+:32761426-32796085
10966	Ctdsp2	52468	10+:126416113-126433542
10967	Atg4b	66615	1+:95664627-95686102
10968	Ttyh2	117160	11+:114536838-114580517
10969	Panx2	406218	15+:88890503-88903976
10970	MMT00068762		X+:100724363-100725599
10971	Klk11	56538	7+:51031672-51034630
10972	Lrig1	16206	6-:94554527-94650139
10973	Adam2	11495	14-:66646162-66692946
10974	Vmn2r25	545874	6-:123775203-123803157
10975	MMT00009477		4-:109046591-109047553
10976	Mmrn1	70945	6+:60894309-60939366
10977	Olfr1132	258833	2-:87474976-87475902
10978	MMT00078322		6-:93599341-93599899
10979	MMT00007806	673902	7+:21875794-21877639
10980	C230078M14Rik	241175	1+:101837219-102275846
10981	Ppm1g	14208	5-:31505042-31522803
10982	EG244495	244495	8-:66407836-66419976
10983	Olfr1359	258067	13+:21790705-21795811
10984	Ptcra	19208	17-:46892611-46900661
10985	Nefl	18039	14+:68701920-68707144
10986	Pappa	18491	4+:64785865-65013191
10987	Polg	18975	7-:86594267-86611080
10988	Cbara1	216001	10+:59165382-59326868
10989	Ptp4a3	19245	15+:73582137-73587407
10990	Dlg2	23859	7+:98239516-99593125
10991	Lonrf1	244421	8-:37282878-37311805
10992	AU042671	269700	5+:121784347-121818573
10993	Pscdbp	227929	2-:57985179-58012522
10994	EG625253	625253	X-:153978644-153979688
10995	Ap4m1	11781	5+:138613407-138619910
10996	Slc36a1	215335	11+:55027436-55046138
10997	1810013D10Rik	66278	5+:53604148-53669778
10998	2310061C15Rik	66531	8-:119413417-119445354
10999	Fhl5	57756	4-:25127055-25169971
11000	Rps26	27370	10-:128061592-128063296
11001	Pou6f2	218030	13-:18453979-18480108
11002	Smg5	229512	3+:88140232-88166259
11003	Olfr671	257910	7-:112123555-112124497
11004	Rhbdl3	246104	11+:80114413-80167742
11005	MMT00007908		7-:50337498-50338028
11006	1700021A07Rik	76924	10+:21122156-21145455
11007	MMT00010063		1-:109188790-109198931
11008	Gps1	209318	11+:120645828-120650416
11009	MMT00008885	330256	6-:3283189-3291900
11010	MMT00072204		6-:82837510-82838719
11011	Rasgrf2	19418	13-:92024037-92901449
11012	MMT00012665		19+:13449453-13450378
11013	Zfand2b	68818	1+:75165319-75168199
11014	Hibadh	58875	6-:52496224-52590294

11015	Olfr923	258812	9+:38635277-38636230
11016	Pcp2	18545	8-:3623376-3625252
11017	Olfr926	258811	9+:38684762-38685688
11018	MMT00015515		4-:126261754-126262053
11019	Hist1h2ba	319177	13-:24007627-24026025
11020	Cyp2c38	13097	19-:39465162-39537540
11021	MMT00000267		1-:24142519-24146519
11022	MMT00048763		4-:110185653-110186412
11023	Lcn12	77701	2-:25346365-25348950
11024	MMT00049098		10-:126469961-126478497
11025	Cd244	18106	1+:173489499-173507478
11026	6230416C02Rik	545490	2+:174866483-175305255
11027	Prkar2b	19088	12-:32643343-32746105
11028	Slc39a14	213053	14-:70706103-70751225
11029	AK129302	245522	X-:92840645-92853681
11030	Tm9sf4	99237	2+:153027872-153036199
11031	4921520G13Rik	66729	5-:144651611-144655936
11032	MMT00018461		9-:99847331-99847971
11033	Egfl7	353156	2+:26436608-26448203
11034	Kcnh2	16511	5-:23825406-23857422
11035	EG665024	665024	6+:36906832-36908685
11036	Trim6	94088	7+:111367324-111383662
11037	Slc25a24	229731	3+:108926059-108971368
11038	Atf7ip2	75329	16+:10192994-10242047
11039	Smgc	223809	15+:91668696-91691866
11040	4933425B07Rik	74458	14-:47222478-47257099
11041	Vmn2r92	627111	17+:18304214-18308349
11042	Atxn7l3	217218	11-:102149726-102156373
11043	Hsph1	15505	5-:150419419-150438793
11044	Pkd1l3	244646	8+:112159114-112180196
11045	Shank3	58234	15+:89331713-89390689
11046	Dennd1b	329260	1+:140989343-141067133
11047	Retnlb	57263	16+:48817091-48819007
11048	Psd2	74002	18+:36146867-36174366
11049	Pcyt1a	13026	16+:32431045-32471928
11050	2210010C04Rik	67373	6-:40981633-40985477
11051	A130042E20Rik	216119	10-:75930901-75931581
11052	4930422G04Rik	71643	3+:127305315-127316475
11053	MMT00044603		11-:102353567-102353973
11054	Rnase4	58809	14+:51710846-51725817
11055	Atp1a3	232975	7-:25763188-25790914
11056	Surf4	20932	2-:26775558-26788947
11057	MMT00032844	353287	8-:113593397-113604831
11058	Sobp	109205	10-:42740786-42893877
11059	Yaf2	67057	15-:93114709-93167239
11060	Gas2	14453	7+:59134519-59249828
11061	Gpr176	381413	2-:118104997-118199070
11062	Olfr1161	258845	2+:87864880-87865845
11063	Phf8	320595	X+:147955721-148068407
11064	Pols	210106	13-:69648439-69672656
11065	Mccc1	72039	3-:35858760-35899521

11066	MMT00022632		4+:103613563-103614546
11067	Immp1l	66541	2+:105744841-105807343
11068	Ncoa6	56406	2-:155216406-155263862
11069	Bgn	12111	X+:70736128-70740135
11070	Fnbp1l	214459	3-:122241638-122272660
11071	Pcbp1	23983	6-:86474492-86475940
11072	2810407C02Rik	69227	3+:58380616-58395134
11073	MMT00001696		7+:115413377-115420647
11074	Lpgat1	226856	1+:193542327-193608139
11075	Xrcc6bp1	68876	10-:126324304-126338404
11076	Dzip1l	72507	9+:99529994-99543113
11077	Olfr51	18351	11+:50820475-50821399
11078	Olfr725	258314	14-:50654112-50655076
11079	EG544954	544954	13-:74905027-74906331
11080	Tctn2	67978	5+:125048792-125077658
11081	Slc35c1	228368	2-:92294352-92299348
11082	Fbxw13	211305	9-:109081740-109084091
11083	Kctd3	226823	1-:190794977-190831720
11084	AF529169	209743	9-:89485561-89517946
11085	MMT00004952		14+:57836069-57883036
11086	Pnpla5	75772	15-:83942786-83953608
11087	Wnt11	22411	7+:105987354-106002261
11088	Unc84b	223697	15-:79554917-79572963
11089	Tgfbi	21810	13+:56710969-56740698
11090	Lycat	225010	17+:73457353-73589860
11091	Tes	21753	6+:17036194-17055821
11092	Pspn	19197	17-:57138880-57139438
11093	Zdhhc1	70796	8-:107996324-108020802
11094	Slc17a3	105355	13+:23931346-23950219
11095	Bag3	29810	7+:135667363-135689910
11096	Olfr654	258377	7+:111736268-111737293
11097	Sema4f	20355	6-:82863484-82889617
11098	Casr	12374	16-:36493782-36562220
11099	Klhdc8b	78267	9-:108349964-108363912
11100	Lrrc4c	241568	2+:97443644-97471825
11101	MMT00049262	216440	10-:126532704-126558204
11102	Gtf2h2	23894	13-:101238863-101260086
11103	9630028I04Rik	319720	17-:27372019-27374091
11104	MMT00014375		4+:69708524-69709268
11105	Atp11c	320940	X-:57476463-57668102
11106	Stra8	20899	6+:34870959-34889342
11107	Klf8	245671	X+:149794172-149827410
11108	Ltbp1	268977	17+:75404907-75790666
11109	AI931714	102182	8+:80087148-80105245
11110	6620401M08Rik	75744	7-:59252546-59261221
11111	Ubl7	69459	9+:57758792-57777777
11112	Olfr131	258867	17-:38218978-38219921
11113	MMT00006619		12-:63166201-63166660
11114	Ddx19b	234733	8-:113531978-113545000
11115	Cul3	26554	1-:80263633-80336895
11116	Olfr11	218066	13-:21730450-21748040

11117	1700047I17Rik2	100101807	12+:56300533-56306841
11118	Loxl1	16949	9-:58159492-58160996
11119	Frmd4a	209630	2+:4352241-4535076
11120	Elf1	13709	14+:79915486-79982282
11121	Mtmr11	194126	3+:95965934-95975636
11122	Abcb6	74104	1-:75168413-75176937
11123	1600012H06Rik	67912	17+:15080212-15081174
11124	Mboat4	234155	8+:35187193-35187771
11125	Trim39	79263	17-:36395817-36408949
11126	Pnkd	56695	1+:74379183-74400258
11127	Snapc4	227644	2-:26219156-26236172
11128	Aars	234734	8+:113570866-113579468
11129	Ankhd1	108857	18+:36751100-36825562
11130	Slco1c1	58807	6+:141473070-141518237
11131	Olfr516	258720	7-:115988578-115989522
11132	Mbc2	23943	10-:127946802-127962935
11133	MMT00011321		9+:113439298-113440407
11134	Tcf7l2	21416	19+:55816821-56006583
11135	Ascc3	77987	10+:50337582-50571006
11136	Rnf7	19823	9-:96372153-96379001
11137	0910001A06Rik	223601	15-:63762498-63829524
11138	Bbs9	319845	9+:22598193-22692722
11139	MMT00061410		4-:144088304-144088821
11140	Drd3	13490	16+:43762354-43822951
11141	MMT00052073		X+:63190200-63190871
11142	Sez6l2	233878	7+:134094242-134113781
11143	Gm962	381201	19+:5568457-5571183
11144	Abcf3	27406	16+:20548736-20552706
11145	Dctn3	53598	4-:41661981-41670164
11146	Tmc8	217356	11+:117646118-117646627
11147	Gng12	14701	6+:66846416-66967923
11148	Htra4	330723	8-:26135400-26149395
11149	MMT00050488		17-:5110751-5111950
11150	Pcsk9	100102	4-:106131453-106136935
11151	5730472N09Rik	108958	2+:30219777-30241040
11152	MMT00057918		12-:39095259-39095817
11153	1810063B05Rik	67892	8+:128946409-128950654
11154	MMT00037611		5-:92189250-92190710
11155	RP23-191F22.4	100039672	4-:43596135-43597140
11156	MMT00004568		12-:116293410-116293867
11157	Aqp12	208760	1+:94902912-94908848
11158	Kcnk16	74571	14-:21080598-21088384
11159	MMT00065994		10-:111526331-111527619
11160	Pde8a	18584	7+:88358623-88479414
11161	Gpatch2	67769	1+:189039425-189058218
11162	Hkdc1	216019	10-:61845886-61885205
11163	1700011H22Rik	69314	4-:91811029-91813927
11164	Odf4	252868	11-:68735337-68740551
11165	V1rh18	171275	13-:22974958-22976030
11166	Uqcc	56046	2-:155672642-155755767
11167	Dars2	226539	1-:162970732-163000787

11168	1700019H03Rik	228993	2+:180460043-180476982
11169	MMT00078034		11+:107052394-107052843
11170	Cpb1	76703	3-:20148982-20174638
11171	Lnpep	240028	17-:17666454-17761562
11172	1110001A07Rik	66140	11+:86922762-86936476
11173	Dnahc6	330355	6-:73138304-73140444
11174	Zfp2	22678	11-:50712214-51037872
11175	MMT00033256	666185	13+:68051623-68053210
11176	V1rc2	113859	6+:58257290-58258200
11177	Phf6	70998	X+:50265388-50307181
11178	Olfr951	258046	9+:39172371-39202318
11179	Cd244	18106	1+:173489329-173513688
11180	MMT00004147		17-:63034950-63036348
11181	MMT00051455	639628	18+:76998694-77139187
11182	Rab27a	11891	9+:72912049-72945298
11183	Pank3	211347	11+:35582985-35600014
11184	Etfdh	66841	3-:79407733-79432636
11185	Polr3c	74414	3-:96515448-96531733
11186	Prkg1	19091	19-:30638998-30639748
11187	Mettl1	17299	10+:126478987-126482517
11188	MMT00036518		14-:41107406-41108810
11189	D630023F18Rik	98303	1-:65155291-65168012
11190	Cd8a	12525	6+:71323419-71327605
11191	Spert	67926	14-:75982683-75991769
11192	Maged2	80884	X-:147246358-147248858
11193	Tsc22d1	21807	14+:76904337-76907569
11194	Hecw2	329152	1-:53870066-54097375
11195	Fmn1	14260	2+:113167938-113550012
11196	Slco1b2	28253	6+:141578038-141635156
11197	Odc1	18263	12+:17551895-17557883
11198	Gbp1	14468	3+:142163517-142282131
11199	Sirt7	209011	11-:120479680-120486303
11200	MMT00034030		4-:39842197-39882912
11201	Sunc1	194974	11-:8916056-8928120
11202	Olfr672	258755	7-:112144478-112145416
11203	Bag1	12017	4-:40883451-40895270
11204	AI451617	209387	7-:111618529-111656339
11205	Cdh5	12562	8+:106625609-106667033
11206	Itih4	16427	14+:31699698-31715035
11207	MMT00081058		X+:133634025-133634656
11208	Hspb1	15507	5+:136363928-136365307
11209	E130203B14Rik	320736	14+:33670097-33752220
11210	6430514L14Rik	76886	9-:69937115-69989356
11211	Tctex1d4	242646	4+:116799431-116801332
11212	1300018I17Rik	72325	8-:125766256-125778010
11213	Ccr9	12769	9+:123676362-123689893
11214	MMT00049207		1-:10544093-10545078
11215	Arid1b	239985	17+:5040568-5127640
11216	MMT00031221	100039760	X-:147927512-147927829
11217	Pde8b	218461	13-:95803904-95956205
11218	Apc	11789	18+:34420676-34478258

11219	Mrps18a	68565	17+:46247952-46265855
11220	AU021092	239691	16-:5211736-5222387
11221	Isca1	69046	13-:59858216-59871065
11222	Dcpp1	13184	17+:24017842-24019819
11223	Pgr15l	245526	X+:94271253-94273712
11224	Mst1	15235	9+:107982809-107987331
11225	Numbl	18223	7+:28043895-28067160
11226	A530082C11Rik	320541	4+:154975531-154993111
11227	1700123J19Rik	73614	X+:35474856-35483142
11228	MMT00081145		X-:133738139-133739045
11229	Slc6a20b	22599	9-:123504111-123541507
11230	Plac1	56096	X-:50423488-50424008
11231	B2m	12010	2+:121973421-121978607
11232	Ch25h	12642	19-:34548272-34549625
11233	Tef	21685	15+:81641909-81654413
11234	Pygl	110095	12-:71291798-71328604
11235	Itga2b	16399	11-:102314610-102331058
11236	Gabrb2	14401	11+:42234760-42440737
11237	Mycbpap	104601	11-:94362661-94382759
11238	Klk1b24	16617	7+:51443632-51447819
11239	Cspp1	211660	1+:10029872-10058057
11240	Ppm1d	53892	11+:85124974-85159806
11241	Lcn11	227630	2+:25632570-25635798
11242	Obp1a	18249	X-:75330843-75336715
11243	L3mbtl2	214669	15+:81494415-81518709
11244	Atp5a1	11946	18+:78012759-78021605
11245	Sgta	52551	10-:80506817-80522899
11246	Apex1	11792	14+:51544695-51546813
11247	Obscn	380698	11-:58919796-58941838
11248	Glt1d1	319804	5+:128131052-128187392
11249	Rnd1	223881	15-:98493852-98507883
11250	Rsf1	233532	7+:104728420-104835187
11251	F2rl1	14063	13-:96281686-96295154
11252	Apoo	68316	X+:91612492-91662426
11253	MMT00059796		3-:67655294-67655676
11254	Klrb1-ps1	724020	6+:129072981-129079463
11255	Srpx	51795	X-:9616033-9694713
11256	H2-DMa	14998	17+:34272615-34275480
11257	Trem3	58218	17+:48386943-48398207
11258	Ctnna3	216033	10+:62892844-63044811
11259	Ccdc69	52570	11-:54863232-54891630
11260	Frem1	329872	4-:82561985-82586681
11261	4921505C17Rik	78757	15+:6736938-6747411
11262	1700037C18Rik	73261	16-:3905799-3908724
11263	Mib1	225164	18+:10778192-10812206
11264	Zcwpw1	381678	5+:138237799-138263881
11265	Irgm	15944	11-:48678751-48684848
11266	Gtdc1	227835	2-:44606990-44681072
11267	Cd79b	15985	11-:106172655-106175984
11268	Nxf1	53319	19+:8831616-8845173
11269	Pib5pa	170835	11-:3394272-3403146

11270	Gabrp	216643	11-:33450860-33478931
11271	Orc4l	26428	2-:48760726-48804747
11272	MMT00015072		4-:101568568-101569567
11273	Abcc8	20927	7-:53359893-53425360
11274	Srp54a	24067	12+:56181514-56214240
11275	Fanci	208836	7+:86537238-86595150
11276	Zfp148	22661	16+:33380963-33497428
11277	Capn11	268958	17-:45767401-45790682
11278	Rph3al	380714	11-:75692442-75739393
11279	MMT00040653		15+:9353992-9362667
11280	Phpt1	75454	2-:25428948-25430400
11281	Foxj1	15223	11-:116192016-116195998
11282	Tekt1	21689	11-:72158225-72175375
11283	Uroc1	243537	6+:90283299-90314540
11284	Elac2	68626	11+:64792598-64815571
11285	MMT00045951		14+:54580667-54581493
11286	MMT00013106		3+:53257678-53261713
11287	Trappc5	66682	8+:3676509-3679627
11288	Cacna1i	239556	15+:80128755-80226088
11289	MMT00038299		18+:12524307-12524757
11290	MMT00063482		1-:57575368-57575931
11291	1110018H23Rik	68509	17+:25259638-25262158
11292	Nr2f6	13864	8-:73898023-73905283
11293	Bpi	329547	2+:158086802-158100578
11294	Cbr2	12409	11+:120627308-120628679
11295	Rbm25	67039	12+:84973191-85024075
11296	Ccdc33	382077	9-:57876484-57881118
11297	BC017158	233913	7-:135414893-135441615
11298	Rpl27	19942	11+:101303728-101306841
11299	Gemin5	216766	11-:57933913-57982039
11300	Usp9x	22284	X+:12648662-12748912
11301	Csrnp3	77771	2+:65787015-65861515
11302	Bub3	12237	7+:138704223-138714687
11303	Slc5a12	241612	2+:110437461-110481058
11304	Clec3a	403395	8+:116941985-116949726
11305	Galntl2	78754	14+:32842030-32875382
11306	1700016G14Rik	193386	13+:24833101-24838460
11307	MMT00053461		10+:24953010-24977466
11308	Cmbl	69574	15+:31498589-31519874
11309	Ttc30a1	78802	2-:75817799-75819794
11310	Hydin	244653	8+:112790835-112974396
11311	Dusp21	73547	X+:17723074-17723642
11312	2210016H18Rik	70125	18+:11016862-11017825
11313	Vav3	57257	3+:109143930-109487231
11314	6330407J23Rik	67412	10+:28863561-28919474
11315	Dok3	27261	13-:55624595-55629899
11316	Scn3a	20269	2-:65331181-65336653
11317	4632419K20Rik	74349	7-:112529531-112548495
11318	3110001A13Rik	66540	2+:3630764-3697415
11319	Exoc7	53413	11-:116150496-116167987
11320	Sin3b	20467	8+:75247179-75282098

11321	2310014L17Rik	381845	7+:13512767-13515332
11322	EG625347	625347	6-:12049737-12050269
11323	Ndnl2	66647	7-:72016965-72017804
11324	1700012H17Rik	242297	4+:5571345-5727198
11325	MMT00004798		
11326	Mboat1	218121	13+:30228367-30333185
11327	Mks1	380718	11+:87666725-87676500
11328	EG627607	627607	12+:88102600-88104745
11329	Tspan11	68498	6+:127837601-127900061
11330	MMT00024873		2-:19887192-19888317
11331	Ttc28	209683	5+:111308839-111343336
11332	4931432M23Rik	70993	8-:98082962-98099160
11333	Gyk	14933	X-:82948912-83021924
11334	S100pbp	74648	4-:128826586-128866749
11335	MMT00038006		2-:20970257-21041623
11336	Aatf	56321	11-:84236357-84327003
11337	Dhrs1	52585	14-:56357860-56364410
11338	Vamp8	22320	6-:72335372-72340613
11339	Eef1g	67160	19+:9041694-9052666
11340	Syn2	20965	6+:115084919-115225094
11341	Gpr84	80910	15-:103138666-103140869
11342	Maoa	17161	X+:16196825-16263380
11343	Lrp1b	94217	2-:40452292-42509118
11344	Nup37	69736	10+:87609792-87641139
11345	Slc40a1	53945	1-:45964928-45982378
11346	Vcl	22330	14+:21748736-21850956
11347	MMT00076715		1+:168693032-168700418
11348	Med20	56771	17-:47757558-47759119
11349	Cul4b	72584	X-:35886450-35918178
11350	9030420J04Rik	71544	9-:9007965-9048974
11351	Ctnnd2	18163	15+:30102347-30958132
11352	V1re1	171224	17+:20951458-20952377
11353	Ppp3cb	19056	14-:21318536-21365751
11354	Bap1	104416	14+:32064409-32073119
11355	Csta	209294	16-:36122257-36131328
11356	Med27	68975	2+:29202355-29380308
11357	4930558O21Rik	68307	3+:30543428-30571286
11358	2700078E11Rik	78832	19-:60600608-60656902
11359	Clstn2	64085	9-:97345274-97933534
11360	Cbln1	12404	8-:89992767-89994967
11361	Myl2	17906	5+:122550996-122556861
11362	Polr2l	66491	7-:148658602-148661084
11363	MMT00007477	235631	9+:110760484-110767053
11364	2610027L16Rik	67842	14+:56364539-56372831
11365	Bcorl1	320376	X+:45697320-45759881
11366	Smpd4	77626	16+:17619384-17644916
11367	1810009A15Rik	66276	19+:8963392-8965230
11368	Sox8	20681	17-:25702836-25707820
11369	Stx3	20908	19-:11849607-11893761
11370	Prm2	19119	16-:10791473-10792190
11371	Amhr2	110542	15+:102275850-102285061

11372	Cic	71722	7+:26067868-26078553
11373	Maged1	94275	X-:91780814-91787413
11374	Cct6a	12466	5+:130293260-130322231
11375	Derl2	116891	11-:70823427-70832744
11376	Slc7a11	26570	3+:50173828-50174687
11377	Khdc1b	98582	1+:21373664-21376452
11378	Ubl3	24109	5-:149316233-149364209
11379	Dnttip1	76233	2+:164571514-164593717
11380	Tcrb-J	21580	6+:41009441-41009881
11381	Uimc1	20184	13+:55129637-55132382
11382	Cdca3	14793	6+:124780121-124783313
11383	Olfr362	259053	2-:36960215-36961168
11384	Cacng6	54378	7+:3425422-3435658
11385	MMT00081444		12-:3521591-3523555
11386	Vmn2r73	620928	7-:93006177-93020113
11387	MMT00081255		8-:51032460-51032877
11388	Tdrd9	74691	12+:113278635-113290222
11389	Anapc10	68999	8+:82235631-82299302
11390	Kctd16	383348	18+:40690303-40690757
11391	2310016C16Rik	69590	13-:113832970-113836596
11392	AU021838	328099	12+:58331329-58598179
11393	A830007P12Rik	227612	2-:25048485-25052246
11394	Alpk1	71481	3-:127375277-127483433
11395	Tcea1	21399	1+:4847983-4886725
11396	C330043M08Rik	330323	6+:55234739-55268663
11397	Zp2	22787	7-:127275892-127288804
11398	Zfhx3	11906	8+:111238543-111481131
11399	Olfr32	18331	2-:89950615-89951544
11400	MMT00049162		10+:21844382-21845243
11401	Ramp3	56089	11+:6558539-6577477
11402	Pomt1	99011	2+:32092202-32110523
11403	2210016F16Rik	70153	13-:58482608-58486790
11404	Gnpat	14712	8+:127386966-127413803
11405	MMT00031171		6-:70066940-70089088
11406	Ifi203	15950	1-:175787343-175805910
11407	MMT00077704		X+:158790778-158791814
11408	MMT00004171	100039478	4-:8383336-8426186
11409	Phf16	382207	X+:20039237-20094914
11410	EG668804	668804	3-:123212327-123213470
11411	Odf1	18285	15+:38148957-38156393
11412	Abcg5	27409	17-:85057574-85082263
11413	Phyhipl	70911	10-:70020436-70089147
11414	Olfr1330	258331	4+:118565689-118566636
11415	Hnrnpul2	68693	19+:8894758-8906072
11416	Ddr2	18214	1-:171903413-171905635
11417	MMT00018565		17-:85941113-85942045
11418	6330503C03Rik	76156	6-:42267784-42274579
11419	Atrnl1	226255	19+:58116810-58207823
11420	Pou4f1	18996	14-:104864946-104872725
11421	MMT00034261		4+:150221175-150221376
11422	Pltp	18830	2-:164665017-164683113

11423	6720463M24Rik	77744	14+:99445437-99473757
11424	Iqcf3	68265	9-:106445718-106463956
11425	Evx2	14029	2-:74493672-74497476
11426	Sipa1	20469	19-:5651415-5656265
11427	1810053B01Rik	381404	2+:163851216-163875364
11428	4932438A13Rik	229227	3+:36864442-36887237
11429	Lama2	16773	10-:26701086-27187181
11430	MMT00031124		10-:69747193-69747735
11431	Gmpr2	105446	14+:56291136-56297578
11432	Dynll2	68097	11-:87794585-87801001
11433	Aldh1a2	19378	9+:71063650-71143563
11434	MMT00038324		5-:107810464-107811099
11435	Rnaset2a	100037283	17-:8321462-8340653
11436	Mapre1	13589	2+:153567092-153592996
11437	Gatm	67092	2-:122420611-122437013
11438	Cd109	235505	9+:78484255-78564057
11439	Slc28a3	114304	13-:58657047-58712171
11440	Cox8c	75483	12+:104137504-104138746
11441	AA619741	241051	1-:34692385-34735070
11442	MMT00013104		11+:93556395-93556863
11443	Cog2	76332	8+:127044779-127075392
11444	Rnpep	215615	1-:137159289-137180611
11445	Atp1a2	98660	1-:174153650-174228133
11446	Dock8	76088	19+:25248778-25276668
11447	Nr1h4	20186	10-:88917122-88969372
11448	Arl1	104303	10+:88194139-88206827
11449	Ppap2a	19012	13+:113591130-113657850
11450	1110039B18Rik	68796	5+:31174509-31179834
11451	Glis3	226075	19-:28358189-28391903
11452	Ctla2a	13024	13-:61036133-61037895
11453	Adc	242669	4-:128608484-128638804
11454	Cdkl1	71091	12-:70847650-70891697
11455	Olfml2a	241327	2+:38787497-38806934
11456	Las1l	76130	X-:93130650-93152259
11457	Slu7	193116	11+:43247242-43261461
11458	Olfr402	258703	11+:73968657-73969604
11459	Mas1	17171	17-:13034426-13035400
11460	F2rl3	14065	8+:75285801-75287406
11461	Diap2	54004	X+:126643787-126862936
11462	Gpam	14732	19-:55142136-55201706
11463	MMT00057138		13-:71761731-71762275
11464	Gnat3	242851	5+:17468364-17525485
11465	Eif3k	73830	7-:29756391-29766833
11466	MMT00075077		15+:84838519-84839627
11467	Ypel5	383295	17+:73193373-73198143
11468	Fpgs	14287	2-:32537827-32559656
11469	D16Ertd472e	67102	16-:78544256-78576902
11470	MMT00038467		10+:91721450-91722076
11471	Numb	18222	12-:85136388-85183293
11472	Ckmt2	76722	13-:91992992-92016471
11473	MMT00047279		16-:79914704-79914959

11474	Bsg	12215	10+:79167103-79174716
11475	H2-D1	14964	17+:35400836-35400949
11476	4921528I01Rik	70950	18-:22175840-22329942
11477	MMT00006495		12+:117389883-117390811
11478	Olfr124	259064	17+:37942091-37943039
11479	Tmed4	103694	11-:6170716-6174840
11480	Olfr1245	258784	2-:89414959-89415881
11481	Morn1	76866	4+:154460708-154519615
11482	4930412L05Rik	73941	4-:53217937-53230435
11483	Ephb4	13846	5+:137791657-137815537
11484	Tusc5	237858	11+:76493797-76507798
11485	l7Rn6	67669	7-:97067198-97089695
11486	Tacc3	21335	5+:34001309-34014844
11487	1200009F10Rik	67454	10+:90545815-90565352
11488	MMT00035330	100040259	9-:14649688-14650215
11489	Sgsh	27029	11-:119207592-119216798
11490	Gpr133	243277	5+:129615452-129621052
11491	OTTMUSG000000	327963	11+:73885158-73899836
11492	Ttll7	70892	3+:146593014-146642962
11493	Hcfc1	15161	X-:71189777-71211342
11494	Olfr365	258656	2+:37056762-37063648
11495	Rrp9	27966	9+:106379639-106387745
11496	BC049715	320135	6+:136776416-136788645
11497	MMT00046610		8+:13851614-13853065
11498	Opa3	403187	7+:19813288-19841189
11499	1810048J11Rik	67708	12+:73656109-73681159
11500	3300001P08Rik	67684	11-:94152388-94183276
11501	Gpr128	239853	16-:56724723-56778814
11502	Dgat2l4	245532	X-:97600345-97601035
11503	Rangap1	19387	15-:81534683-81560305
11504	Tpm3	59069	3+:89883653-89903532
11505	MMT00018714		17-:93467314-93467864
11506	Rfxank	19727	8-:72656386-72663012
11507	Adarb2	94191	13+:8202394-8756688
11508	1110003E01Rik	68552	5-:65841043-65884069
11509	Ndst1	15531	18-:60848898-60872889
11510	Plch2	269615	4-:154357224-154417264
11511	BC020002	252875	6+:8159228-8186271
11512	Ppil5	69706	12+:70269807-70279992
11513	Serpinf1	20317	11-:75223532-75236013
11514	Muc3	666339	
11515	EG544707	544707	10+:74814968-74815776
11516	Sfxn5	94282	6-:85165482-85283416
11517	MMT00001765		
11518	Tmem130	243339	5-:145504465-145522474
11519	Slc2a10	170441	2+:165329479-165345409
11520	Olfr1	258923	11-:73208579-73209522
11521	Dnaja1	15502	4+:40669585-40680933
11522	Igbp1b	50540	6-:138605610-138606965
11523	MMT00052418		7+:17736871-17748692
11524	MMT00080614		X+:102228106-102228582

11525	MMT00048506		10+:126330168-126330965
11526	Pak6	214230	2+:118489496-118522309
11527	MMT00042501		X-:65260183-65260462
11528	Ccdc49	67480	11-:97606928-97627870
11529	Ceacam12	67315	7+:18651277-18657161
11530	Glis1	230587	4+:107236545-107307665
11531	Tcrg-V3	21637	13+:19334863-19353109
11532	Plekhm3	241075	1-:64841339-64969056
11533	Mpzl3	319742	9+:44863301-44883292
11534	Ndufs4	17993	13-:115078858-115178251
11535	MMT00080036		2+:181729661-181735699
11536	Igj	16069	5-:88949540-88956809
11537	MMT00040737		2+:111840271-111841717
11538	Cntn4	269784	6+:106131769-106623967
11539	Clmn	94040	12-:106009966-106103286
11540	Ctps	51797	4-:120212470-120242869
11541	Cox11	69802	11+:90499483-90507520
11542	Taz	66826	X+:71528106-71535487
11543	Dusp14	56405	11-:83862118-83862715
11544	Fip1l1	66899	5+:74931548-74988447
11545	Ppcs	106564	4-:119091137-119094996
11546	Rdm1	66599	11+:101489262-101497395
11547	Nanog	71950	6+:122657609-122664649
11548	EG625219	625219	5-:22437147-22437894
11549	Depdc6	97998	15+:54943878-55084334
11550	MMT00057961		
11551	MMT00046383		13+:20840333-20840705
11552	Col4a5	12830	X+:137910004-138123735
11553	Polk	27015	13-:97250644-97312440
11554	V1rh10	171269	13-:22919063-22919983
11555	Cldnd1	224250	16+:58728060-58734352
11556	Smarca5	93762	8-:83224293-83263010
11557	Blnk	17060	19-:41003664-41068548
11558	Tbc1d14	100855	5-:36833257-36873150
11559	Fem1c	240263	18-:46661403-46685381
11560	MMT00001695		5-:76455924-76456824
11561	A830093I24Rik	207921	12-:4746694-4776038
11562	Lrrc4	192198	6-:28779513-28781716
11563	Prdm11	100042784	2-:92815406-92886107
11564	Paip2	67869	18+:35758320-35776839
11565	Serpina3c	16625	12-:105385116-105392082
11566	Cdca2	108912	14-:68294739-68333430
11567	Mrps31	57312	8+:23521891-23539948
11568	Lman1l	235416	9-:57454837-57468575
11569	Unc13d	70450	11-:115923409-115938974
11570	Plxnb3	140571	X+:71003884-71017841
11571	Bpnt1	23827	1+:187156079-187180664
11572	OTTMUSG00000(	381569	4-:143401400-143405979
11573	MMT00050792		10+:21844382-21845243
11574	Gne	50798	4-:44049989-44079790
11575	Wdr77	70465	3+:105762501-105770240

11576	Gm1381	384198	5+:92984096-93019689
11577	Usp27x	54651	X-:6950671-6954002
11578	MMT00050323		
11579	MMT00033506		7-:99842463-99910014
11580	MMT00054669		7+:92455950-92491185
11581	Cast	12380	13-:74830741-74946257
11582	MMT00044915		Y+:1520846-1585248
11583	Tbp	21374	17+:15640038-15653747
11584	Zfp592	233410	7+:88168156-88188210
11585	Bdp1	544971	13-:100787951-100873989
11586	MMT00070266		18+:10262204-10263318
11587	Syt14	329324	1-:194723408-194853211
11588	Ncapg2	76044	12+:117664318-117702003
11589	Tead3	21678	17-:28468616-28487091
11590	Cbx4	12418	11-:118941470-118947453
11591	Vil1	22349	1+:74455979-74482088
11592	C130030K03Rik	320644	10-:48813784-48814526
11593	Usf1	22278	1+:173341811-173348871
11594	Heca	380629	10-:17618539-17635838
11595	Zfp14	243906	7-:30821378-30872186
11596	Zfp120	104348	2-:149941955-149962414
11597	2700060E02Rik	68045	14-:20631121-20643035
11598	MMT00031533	628236	19-:33484689-33492127
11599	Coq10b	67876	1+:55109714-55129543
11600	Igk-V24	16113	6+:67883363-67967519
11601	Nmt2	18108	2+:3201559-3243641
11602	Adora2b	11541	11+:62062603-62092717
11603	EG633736	633736	X+:138847269-138847552
11604	Ptov1	84113	7-:52118438-52125158
11605	MMT00067009		4+:118643863-118660622
11606	Mast4	328329	13-:103523408-103643964
11607	Mib2	76580	4-:155028791-155042923
11608	Itgb4	192897	11+:115836020-115869725
11609	Ccnc	51813	4+:21654872-21677693
11610	Hey1	15213	3-:8663358-8666986
11611	S100a13	20196	3+:90318921-90328496
11612	Trp53bp1	27223	2-:121022428-121097105
11613	Egr3	13655	14+:70477251-70479964
11614	Sult1e1	20860	5-:88004994-88020619
11615	Gfra2	14586	14+:71290661-71378297
11616	Btf3l4	70533	4-:108486900-108506192
11617	C130039O16Rik	238317	12-:85500597-85559857
11618	Rpl23	65019	11-:97638859-97643687
11619	Grap	71520	11+:61466822-61486279
11620	Zfr	22763	15+:12047607-12114218
11621	Mixl1	27217	1-:182624750-182627143
11622	Nell1	338352	7+:58081695-58111798
11623	Iqcd	75732	5+:121039033-121057122
11624	MMT00001761		
11625	Hcrtr2	387285	9-:76077764-76171311
11626	Htatsf1	72459	X+:54306745-54320356

11627	MMT00076264	278676	14+:52423159-52425266
11628	Slc6a12	14411	6+:121296721-121315790
11629	Ovol3	381867	7-:31018458-31019522
11630	Mug-ps1	17835	6+:122086686-122198581
11631	Ccl21c	65956	4+:41774242-41775336
11632	MMT00001633		5-:18001747-18002965
11633	Ier3	15937	17+:35958657-35959855
11634	Olfr1223	258894	2-:88984244-88985178
11635	Atad2b	320817	12+:4964862-5051582
11636	V1rl1	171262	7+:11218935-11219832
11637	Ugcgl1	320011	1-:36197830-36268331
11638	MMT00016639		7+:12034325-12035153
11639	Rsbn1l	242860	5-:20413928-20457721
11640	Zxdb	668166	X+:91970834-91972629
11641	2-Sep	18000	1+:95375620-95406307
11642	Smyd1	12180	6-:71165171-71212247
11643	D2hgdh	98314	1+:95722861-95744847
11644	Kcnk9	223604	15-:72342841-72376728
11645	Prtn3	19152	10+:79342420-79345720
11646	4930519G04Rik	67593	5+:115303722-115333668
11647	B4galt2	53418	4-:117541802-117545209
11648	Lrfn2	70530	17+:49209187-49236912
11649	Hfe	15216	13-:23795884-23802655
11650	1700021K19Rik	224118	16-:32821821-32868379
11651	S1pr4	13611	10-:80961222-80962882
11652	Ddx56	52513	11-:6158572-6167741
11653	Mrpl1	94061	5+:96639175-96693434
11654	Scrt1	170729	15-:76347323-76348073
11655	Cd200r1	57781	16+:44766121-44794453
11656	Prkag2	108099	5-:24368575-24606248
11657	Olfr569	259092	7-:110035721-110036665
11658	Grik2	14806	10-:48820794-49503215
11659	Gm1679	381667	5+:110783159-110785108
11660	MMT00068731		15-:102806165-102807888
11661	Rtn1	104001	12-:73312739-73509688
11662	Mre11a	17535	9+:14589115-14638404
11663	Dusp5	240672	19+:53603648-53616916
11664	Efna4	13639	3-:89138096-89141867
11665	Hyal1	15586	9+:107479306-107482425
11666	Eng	13805	2+:32502114-32537635
11667	Rbpjl	19668	2+:164228693-164240948
11668	D6Wsu163e	28040	6+:126892327-126925635
11669	Trappc3	27096	4+:125939657-125952632
11670	Med11	66172	11+:70265454-70267223
11671	Postn	50706	3+:54165046-54194953
11672	E030018N11Rik	319622	7-:125633241-125634848
11673	Pex10	668173	4+:154441090-154446543
11674	MMT00023435		1+:48938556-48939472
11675	MMT00017829		10+:64997375-64998191
11676	Slc24a4	238384	12+:103369714-103505298
11677	5730507C01Rik	236366	12+:18411527-19105834

11678	Fbxo44	230903	4-:147526908-147532934
11679	Lamc1	226519	1-:155065534-155109829
11680	Chfr	231600	5+:110552712-110600986
11681	Pura	19290	18+:36440815-36447899
11682	Nutf2	68051	8+:108384560-108402929
11683	Chm	12662	X-:110155054-110299124
11684	Mif	17319	10-:75322098-75322985
11685	Cd83	12522	13+:43880571-43898499
11686	5830434P21Rik	227723	2+:32038031-32090160
11687	C530008M17Rik	320827	5+:77285355-77300340
11688	Hes3	15207	4-:151660997-151661974
11689	MMT00078109		7+:117442229-117446277
11690	Pex1	71382	5+:3596388-3637230
11691	Muted	17828	13-:38694575-38726978
11692	Eif3j	78655	2+:121854327-121877833
11693	Gpr161	240888	1+:167236268-167251981
11694	Pip4k2b	108083	11-:97580174-97605918
11695	MMT00081973	675985	8+:91330258-91331072
11696	MMT00051197		2-:128863875-128873912
11697	Rasl2-9	19428	7-:5076607-5077558
11698	MMT00067243		13-:12517372-12517899
11699	MMT00044175		18+:42360453-42360956
11700	Nhej1	75570	1-:75013928-75121778
11701	Oas1c	114643	5-:121250216-121258791
11702	Rag2	19374	2+:101464960-101471411
11703	MMT00024473		18+:81050660-81051464
11704	EG668525	668525	12+:20675479-20676524
11705	Polr3f	70408	2+:144353494-144365094
11706	Shisa3	330096	5+:67999383-68002713
11707	MMT00053032		18-:64240415-64241170
11708	MMT00078566		6-:13005596-13006527
11709	Asb2	65256	12-:104559355-104594211
11710	Hsd17b14	66065	7+:52814758-52822687
11711	Atp5e	67126	2-:174286575-174289590
11712	Neil1	72774	9-:56991062-56994841
11713	Mpdu1	24070	11-:69470205-69476079
11714	MMT00043933		3-:38457084-38457590
11715	Lars	107045	18-:42362003-42393473
11716	Slco6d1	70866	1+:100317699-100405958
11717	Wdhd1	218973	14-:47860617-47896272
11718	Nmb	68039	7-:88047127-88049922
11719	Clasp1	76707	1+:120286025-120506009
11720	Cdk10	234854	8+:125748655-125756683
11721	Usp37	319651	1-:74485061-74552396
11722	Ccdc104	216618	11-:29121546-29147281
11723	Dnase1	13419	16+:4037356-4040022
11724	Tuba3a	22144	6-:125228306-125236049
11725	Itln1	16429	1-:173448253-173465424
11726	MMT00065091		3+:135567308-135591948
11727	Olfr24	18322	9-:18559136-18559750
11728	2310043L02Rik	69664	11-:99441297-99442330

11729	Ncr1	17086	7+:4289336-4296362
11730	Tcea1	21399	1+:4847972-4886725
11731	Gpr141	353346	13-:19843305-19916097
11732	Rundc3a	51799	11+:102254989-102263867
11733	MMT00064839		6+:38234531-38235012
11734	MMT00024780	100009600	9-:20866835-20870592
11735	Thoc5	107829	11+:4795334-4828863
11736	EG667148	667148	12-:102102291-102102649
11737	Hgsnat	52120	8-:27054930-27056954
11738	Tmed2	56334	5+:124990837-124999252
11739	Ccnb1	268697	13+:101548688-101556418
11740	Cpm	70574	10+:117066553-117122359
11741	Slc30a10	226781	1+:187278726-187289068
11742	Ifitm2	80876	7-:148140740-148141860
11743	Prdx2	21672	8+:87494135-87498209
11744	Ctnnbl1	66642	2+:157563176-157716827
11745	Ece2	107522	16+:20629982-20645992
11746	MMT00002998	545600	4-:37086175-37086886
11747	4933434I06Rik	71233	2-:21102357-21126992
11748	MMT00044356		1-:36059977-36060748
11749	Rp1h	19888	1-:4333584-4399340
11750	MMT00034237		7-:74109913-74139054
11751	MMT00014760		13-:49938581-50126853
11752	MMT00061847		X+:99680782-99697479
11753	CU075549.1	620807	4+:60016418-60016983
11754	Ddx42	72047	11+:106102972-106110453
11755	Il4	16189	11-:53425969-53432167
11756	2810011L19Rik	69952	12+:106575064-106620320
11757	Ercc3	13872	18+:32399984-32429801
11758	S100a1	20193	3-:90315031-90318216
11759	3110079O15Rik	73234	1+:89366832-89372058
11760	MMT00068385	56716	17-:24612762-24615973
11761	Ankrd37	654824	8-:47082263-47085222
11762	Ggt5	23887	10+:75052140-75077930
11763	Seh1l	72124	18+:67934628-67952247
11764	Chrna2	110902	14+:66759796-66771783
11765	Gm884	380730	11-:103461614-103462212
11766	Plekhh2	213556	17+:84911187-85019148
11767	Srr	27364	11-:74722017-74732999
11768	Pcna	18538	2-:132075022-132078891
11769	MMT00020194		X-:50135871-50149863
11770	MMT00077046		14+:40819357-40819841
11771	Cebpe	110794	14-:55329376-55330958
11772	Ascl3	56787	7-:116870978-116875246
11773	Dhx35	71715	2+:158620555-158683949
11774	4933433C11Rik	74472	2-:25067725-25070153
11775	Phyh	16922	2+:4840076-4859770
11776	Ifi30	65972	8-:73286873-73290530
11777	MMT00008513		5-:44797035-44797838
11778	Mastl	67121	2-:22973650-22995496
11779	Irx2	16372	13+:72767251-72771641

11780	Taok1	216965	11-:77351102-77369095
11781	Erc2	238988	14+:29289261-29291715
11782	Cenpt	320394	8-:108368563-108375902
11783	Crim2	333088	6-:29437180-29439153
11784	Es1	13884	8-:95622913-95655170
11785	MMT00075578		15+:84696703-84697800
11786	Gzmg	14944	14-:56775517-56778402
11787	Rsf1	233532	7-:104802885-104803263
11788	MMT00014479		15-:59761367-59761815
11789	Ccdc90a	76137	13-:43633780-43655538
11790	Plekha5	109135	6+:140492673-140545628
11791	Duoxa2	66811	2+:122124635-122128621
11792	Neu2	23956	1+:89491124-89494401
11793	Wdr79	216853	11-:69375256-69392826
11794	OTTMUSG00000(	100038570	11+:116518498-116521429
11795	Gps2	56310	11+:69727551-69730092
11796	Ela3	67868	4-:136976924-136986433
11797	Sbk1	104175	7+:133416132-133438512
11798	A530082C11Rik	320541	4+:154993303-154994621
11799	EG636947	636947	6-:130788442-130820615
11800	Clip3	76686	7+:31077174-31092167
11801	Gtf2f2	68705	14-:76296735-76410735
11802	Armc4	74934	18-:7088232-7297938
11803	1500032D16Rik	78330	17+:31657095-31668272
11804	MMT00071318		16-:48399565-48412399
11805	Bat2	53761	17-:35286206-35299928
11806	Ptprj	19271	2-:90276208-90420627
11807	Fbxo31	76454	8-:124075915-124102555
11808	Pck1	18534	2+:172978573-172984752
11809	4930534B04Rik	75216	12-:92527833-92622820
11810	Tfb2m	15278	1-:181458186-181476349
11811	Lypla2	26394	4-:135524139-135528509
11812	Mrpl16	94063	19+:11844904-11849434
11813	MMT00009937		1-:48371105-48371743
11814	Pdlim5	56376	3-:141905620-142058582
11815	Nlrp1c	627984	11-:70969524-71074918
11816	Ccdc99	70385	11-:34622691-34647126
11817	Dpysl3	22240	18-:43483941-43552985
11818	MMT00048396		2-:147468585-147468916
11819	Mcee	73724	7+:71537531-71557000
11820	Abcg4	192663	9-:44081271-44096206
11821	Qsox2	227638	2-:26064408-26092885
11822	Elovl2	54326	13-:41277867-41315762
11823	Mmp7	17393	9+:7692089-7699584
11824	MMT00033503		7+:101345719-101345969
11825	MMT00024508		5+:101734596-101734896
11826	Rps24	20088	14+:25310970-25312715
11827	Ubn1	170644	16+:5050147-5086377
11828	Ggps1	14593	13-:14145945-14155556
11829	MMT00004382		4-:33288659-33289039
11830	Zbtb48	100090	4-:151393882-151401767

11831	AI747699	381236	19-:33749595-33805439
11832	Rnf11	29864	4-:109125503-109149588
11833	Abo	80908	2-:26698708-26704510
11834	Pja2	224938	17-:64632232-64660716
11835	Olfr338	258949	2+:36232297-36233216
11836	Csf1r	12978	18+:61265528-61290786
11837	Paqr6	68957	3+:88186020-88186764
11838	MMT00005019		14+:53627284-53627815
11839	Olfr25	18323	9+:38137422-38138113
11840	Chrdl1	83453	X-:139732681-139824691
11841	MMT00046311		13+:66737466-66737671
11842	Rarb	218772	14-:17263355-17408109
11843	Dusp22	105352	13+:30751964-30803092
11844	Krt33a	71888	11-:99872515-99877526
11845	Psme1	19186	14+:56197330-56200364
11846	Cxcr5	12145	9-:44319924-44334461
11847	Tmem45b	235135	9-:31233781-31271823
11848	MMT00064912		13+:87922528-87923396
11849	Olfr648	258746	7-:111327970-111328920
11850	Kdelc2	68304	9+:53192204-53209971
11851	Smchd1	74355	17-:71778801-71786166
11852	Igsf11	207683	16+:39007241-39027266
11853	EG214403	214403	1-:141471761-141677817
11854	Btbd1	83962	7-:88936960-88963150
11855	4930402F06Rik	74854	2-:35231096-35252696
11856	Centa1	231821	5-:139747830-139783826
11857	Arv1	68865	8+:127246038-127258023
11858	Rnf135	71956	11+:79997373-80013255
11859	Poln	272158	5-:34349838-34452083
11860	Hesx1	15209	14+:27813547-27815509
11861	Dyrk3	226419	1-:133025018-133034811
11862	Rce1	19671	19-:4622550-4625617
11863	Srbd1	78586	17-:86535709-86544568
11864	Dis3l	213550	9-:64154564-64156236
11865	EG383815	383815	3+:3791896-3792295
11866	Snx2	67804	18+:53336018-53380514
11867	Rcor3	214742	1-:193925269-193961809
11868	Krtcap3	69815	5+:31554088-31555570
11869	Pcsk5	18552	19-:17507405-17729390
11870	Apitd1	69928	4-:148502457-148511659
11871	4931417E11Rik	66740	6-:73418575-73419661
11872	Vamp5	53620	6-:72319026-72320326
11873	Mmp2	17390	8+:95351480-95377318
11874	Nucb1	18220	7-:52748477-52765219
11875	Pgbd5	209966	8-:126894391-126957980
11876	MMT00010256		11+:120966851-120967903
11877	Luzp2	233271	7+:62422569-62467257
11878	Olfr1248	258787	2-:89457423-89458347
11879	Cubn	65969	2-:13197961-13413550
11880	Svep1	64817	4-:58055668-58219468
11881	Mpl	17480	4-:118115019-118130076

11882	Prdm4	72843	10-:85354714-85379664
11883	Ptprn2	19276	12+:118392619-118515290
11884	EG433225	433225	19-:15034039-15035256
11885	2810402E24Rik	66465	14+:21429440-21460276
11886	Cep250	16328	2+:155817369-155824609
11887	Vps8	209018	16+:21434983-21442537
11888	Myadm	50918	7+:3290534-3299348
11889	Stag1	20842	9+:100543993-100857054
11890	Il22ra1	230828	4+:135288892-135306635
11891	Rtn2	20167	7+:19868015-19881508
11892	D430042O09Rik	233865	7+:132993891-133018305
11893	Cdc40	71713	10-:40551425-40602915
11894	Fstl4	320027	11+:52813698-53000515
11895	2810405J04Rik	72722	17-:75936430-75951183
11896	Kif6	319991	17+:49754462-49978532
11897	ENSMUSG00000(	100038642	19-:53519582-53522231
11898	Olfr186	258318	16-:59026801-59027731
11899	MMT00070220		9+:55797825-55875733
11900	MMT00005346		
11901	A730041O05Rik	320330	4+:16090526-16091339
11902	MMT00029171		X-:5274662-5279002
11903	4933437F05Rik	71275	12-:88561975-88579683
11904	Actr5	109275	2+:158450636-158465181
11905	Utp6	216987	11-:79749258-79775884
11906	Nfe2l3	18025	6+:51382668-51408767
11907	Tmeff2	56363	1+:50984403-51242552
11908	Taf3	209361	2-:9835764-9970220
11909	Sag	20215	1+:89700468-89741732
11910	MMT00016683		10+:76599937-76600411
11911	Wnt6	22420	1+:74818719-74831519
11912	Pcdhb22	93893	18+:37678134-37680801
11913	Stap2	106766	17-:56136503-56144991
11914	Adam1b	280667	5-:121950694-121970451
11915	Calm1	12313	12+:101437896-101445521
11916	Pard6b	58220	2+:167906784-167924706
11917	Fahd1	68636	17-:24985843-24987082
11918	Meis2	17536	2-:115890907-115891494
11919	Ammecr1	56068	X-:139289355-139401271
11920	D10Wsu52e	28088	10-:85401381-85420528
11921	Ddx21	56200	10-:62042995-62061938
11922	Cox19	68033	5-:139813467-139821171
11923	Pde3b	18576	7+:121559071-121681693
11924	MMT00037881		11+:4021929-4029658
11925	Epm2aip1	77781	9+:111174345-111181593
11926	Ampd2	109674	3-:107876981-107889568
11927	Ghrhr	14602	6+:55326285-55338523
11928	E330017A01Rik	224247	16-:58635278-58636990
11929	Slc22a7	108114	17-:46569133-46575380
11930	Igh-VJ558	16061	12-:116784206-116784642
11931	MMT00009560		1+:71901410-71901976
11932	Pkp1	18772	1-:137767974-137815601

11933	EG624918	624918	7+:29541187-29542586
11934	Rbms2	56516	10-:127570287-127613497
11935	Nxph2	18232	2+:23177008-23255941
11936	MMT00022429		8+:74047403-74048218
11937	Snx10	71982	6+:51473961-51540267
11938	Slc14a2	27411	18-:78379983-78793590
11939	V1rd14	81011	7+:23197249-23199685
11940	Chi3l1	12654	1+:136079041-136086612
11941	Iqch	78250	9-:63269262-63450299
11942	MMT00005752		Y_random-:7688830-8281973
11943	Tcf20	21411	15-:82639781-82685360
11944	Ctla4	12477	1+:60965957-60972674
11945	Pabpc4	230721	4+:122960606-122975336
11946	MMT00030953		18-:31979216-31980063
11947	MMT00018910		X+:151208994-151212575
11948	3322402L07Rik	74023	1+:193801253-193812163
11949	Cldn3	12739	5+:135462257-135463342
11950	Vsx1	114889	2-:150507280-150514738
11951	Denr	68184	5+:124357283-124378841
11952	Olfr710	258594	7-:114087582-114091826
11953	Gtf2b	229906	3+:142428210-142446566
11954	MMT00003007		7-:112239215-112241355
11955	Ccl6	20305	11-:83401393-83406492
11956	Bmper	73230	9+:23097840-23289646
11957	Elmod1	270162	9-:53759268-53822981
11958	MMT00028670		3-:111124244-111125637
11959	4631422O05Rik	78749	16+:57507661-57572911
11960	Tm4sf4	229302	3+:57229380-57245587
11961	Nol4	319211	18-:22853442-23198543
11962	Inoc1	68142	2-:119198781-119257680
11963	1110054O05Rik	66209	4-:59782524-59796739
11964	Plch1	269437	3-:63439483-63571549
11965	MMT00013041		5-:87805183-87807199
11966	Zfp280b	64453	10+:75495435-75502733
11967	Zmynd19	67187	2+:24805321-24814933
11968	Cenpm	66570	15-:82064512-82075177
11969	Gbas	14467	5+:130230973-130264126
11970	AA408296	215193	1-:194933482-194956388
11971	1700025D03Rik	67944	X-:134742703-134743300
11972	Rdh19	216453	10+:127287050-127297392
11973	Kcnv2	240595	19+:27397240-27408326
11974	1700023B02Rik	66935	2-:73125663-73150765
11975	Usmg5	66477	19-:47157957-47165123
11976	MMT00035978		
11977	Hhip	15245	8-:82496145-82581888
11978	She	214547	3+:89653289-89658634
11979	1700029F09Rik	75623	1-:44142819-44159313
11980	Otud1	71198	2+:19578345-19582207
11981	Sh3bp2	24055	5+:34868497-34906203
11982	MMT00035514		1-:91152714-91153562
11983	MMT00078789		14+:54173687-54180092

11984	N6amt2	68043	14-:58168434-58190406
11985	B430211C08Rik	627280	7-:15116000-15207836
11986	E030002O03Rik	244180	7-:111302277-111313343
11987	Dmkn	73712	7+:31548778-31566086
11988	MMT00041161	100042507	19-:39128203-39129471
11989	Tmprss11b	319875	5-:87089456-87105392
11990	Prkdc	19090	16+:15637980-15842342
11991	MMT00003201		17-:73539843-73540506
11992	Mrpl19	56284	6-:81911578-81915943
11993	Sfxn2	94279	19+:46647913-46669540
11994	MMT00036655		1+:35027157-35204261
11995	Cetn1	26369	18-:9618597-9619856
11996	MMT00020927		
11997	MMT00082790		
11998	4930403N07Rik	73936	12-:73222478-73286006
11999	Slc24a1	214111	9-:64770667-64799414
12000	Tmem16h	382014	8-:73999204-74005145
12001	Rras2	66922	7-:121190301-121261295
12002	Kif3c	16570	12+:3365555-3404064
12003	Ado	211488	10-:67010751-67013180
12004	Lgals6	16857	7+:29619148-29626651
12005	Arhgap22	239027	14+:34084969-34183121
12006	Sox9	20682	11+:112643595-112647449
12007	Olfr1106	258747	2-:86888454-86889391
12008	Dhdh	71755	7-:52728220-52744163
12009	Gykl1	14625	18+:52853332-52855313
12010	Foxg1	15228	12+:50483993-50487843
12011	Wdr61	66317	9-:54564960-54582308
12012	Cd247	12503	1+:167718853-167797565
12013	Gsdmc1	83492	15-:63607649-63636153
12014	Kifc2	16581	15+:76491085-76498624
12015	Esf1	66580	2-:139945616-139996299
12016	Dyrk1b	13549	7+:28964512-28971941
12017	Agl	77559	3-:116445489-116511046
12018	1700012C15Rik	76390	11+:73503781-73525936
12019	Rnf138	56515	18+:21159841-21184774
12020	Bglap2	12097	3-:88181657-88182620
12021	MMT00003645		14+:80347485-80348077
12022	MMT00048107		11-:105225403-105251015
12023	Slc36a2	246049	11-:54971891-54998545
12024	OTTMUSG00000(	667373	19-:34667377-34673920
12025	Sdccag3	68112	2-:26238882-26244803
12026	MMT00035413		14-:49731841-49733154
12027	Dnajb3	15504	1-:90101310-90102323
12028	Capn13	381122	17-:73709723-73748599
12029	Tcf19	106795	17-:35649679-35653699
12030	Dsc2	13506	18-:20190518-20217805
12031	Pcdh12	53601	18-:38428429-38444011
12032	Cdca7	66953	2+:72314275-72324947
12033	EG545507	545507	3+:14722415-14729015
12034	Pip4k2a	18718	2-:18765764-18919636

12035	Ncoa1	17977	12-:4249454-4483976
12036	Atp2b4	381290	1-:135608219-135649858
12037	Pfdn1	67199	18-:36563332-36614149
12038	Tnks1bp1	228140	2+:84892561-84913202
12039	Fignl1	60530	11-:11700835-11706200
12040	Commd10	69456	18+:47118522-47247650
12041	Lrrc55	241528	2-:85032067-85036796
12042	Nfat5	54446	8+:109816622-109903416
12043	Tdrd5	214575	1-:158206691-158214615
12044	Sycp1	20957	3-:102622421-102740222
12045	Metap2	56307	10-:93323977-93352436
12046	Impdh1	23917	6-:29151198-29162166
12047	MMT00073939		X-:157923523-157924269
12048	Pdzd3	170761	9-:44055390-44059543
12049	1700026D11Rik	70371	2+:132316449-132334010
12050	Zfp11	22648	5-:130162326-130177674
12051	Pik3c3	225326	18+:30432397-30507779
12052	Ntsr2	18217	12+:16660279-16667033
12053	2310005L22Rik	69471	4+:131864588-131866941
12054	2610109H07Rik	70433	4-:147476436-147484265
12055	1810008A18Rik	108707	10-:76949408-76978530
12056	Zic3	22773	X+:55283819-55289080
12057	Dok5	76829	2+:170557453-170704860
12058	Slc13a4	243755	6-:35218857-35257456
12059	2700049P18Rik	108900	1+:133424543-133435611
12060	Gfm2	320806	13+:97911881-97945000
12061	Fbxo15	50764	18+:85126711-85150866
12062	Dzip1l	72507	9+:99549973-99560194
12063	Capn13	381122	17-:73655803-73671379
12064	Mllt3	70122	4-:87657536-87679186
12065	Cmpk2	22169	12+:27154373-27162992
12066	MMT00040000		5+:87406182-87420763
12067	Rab17	19329	1-:92855460-92860877
12068	Plekha7	233765	7-:123278553-123286953
12069	4933438B17Rik	71247	5+:127473784-127568067
12070	Adat2	66757	10+:13272699-13282842
12071	Tbc1d16	207592	11-:119008881-119070374
12072	Rod1	230257	4-:59488132-59562146
12073	Olfr622	259087	7-:110787699-110788652
12074	MMT00013159		17+:17026222-17027374
12075	Tnfrsf8	21941	4-:144858878-144905050
12076	MMT00040312		6+:68520756-68522348
12077	MMT00059499		
12078	Olfr1195	258748	2-:88522962-88523887
12079	Spred2	114716	11+:19824084-19824883
12080	2410017P09Rik	69709	12+:4237478-4238583
12081	Pex11c	69129	8-:3458816-3467648
12082	Abcc5	27416	16-:20331378-20426408
12083	Dcst1	77772	3-:89154141-89158141
12084	Siva1	30954	12+:113883133-113887354
12085	EG214403	214403	1-:141594211-141890831

12086	Cd69	12515	6-:129217339-129225387
12087	Akap3	11642	6+:126803190-126824325
12088	5730416F02Rik	70550	X+:106066877-106197724
12089	Scn8a	20273	15+:100766755-100871119
12090	Nf2	18016	11-:4667667-4748998
12091	1810015C04Rik	66270	15+:25872433-25902092
12092	Hoxa11	15396	6-:52192105-52195766
12093	A230106M15Rik	231717	5+:122299036-122304641
12094	Carhsp1	52502	16-:8658679-8672238
12095	Gucy1a2	234889	9+:3759395-3894710
12096	Nupl1	71844	14-:60824449-60870318
12097	9430015G10Rik	230996	4+:155484097-155501370
12098	BC003266	80284	4+:126921084-126925052
12099	Prep	19072	10+:44787136-44878330
12100	MMT00011598		7+:4590137-4590970
12101	Rnf123	84585	9-:107953865-107981648
12102	Rnh1	107702	7-:148346377-148352667
12103	Mtr	238505	13-:12278802-12350368
12104	MMT00063115	231872	5-:144663573-144670687
12105	Cplx1	12889	5-:108948616-108978972
12106	Tcirg1	27060	19-:3896051-3907097
12107	Nck2	17974	1+:43566551-43626331
12108	MMT00009760		15-:70930152-70930455
12109	Smad4	17128	18-:73799654-73837885
12110	MMT00047508		1-:130000420-130025531
12111	AA474455	231151	5-:36816291-36826709
12112	Tmem53	68777	4+:116924593-116941185
12113	Disp2	214240	2+:118605475-118618561
12114	Homer2	26557	7-:88754229-88794602
12115	Hist1h2an	319170	13-:21878696-21879087
12116	BC057371	194237	4-:119140443-119165078
12117	1700024G13Rik	67085	14-:33189272-33201577
12118	Mtrr	210009	13-:68701154-68721039
12119	Dmd	13405	X+:80194241-82450380
12120	Rad51	19361	2+:118938600-118961459
12121	Ren1	19701	1+:135247268-135256894
12122	C130092O11Rik	232035	6+:61735140-61761038
12123	Slc7a11	26570	3-:50175835-50247502
12124	Olfr459	258569	6-:41721351-41722296
12125	MMT00004451		14-:63643966-63644689
12126	Znfx1	98999	2-:166861298-166882441
12127	0610010F05Rik	71675	11-:23473772-23533210
12128	Atp11b	76295	3+:35737674-35755193
12129	Cfi	12630	3+:129539656-129578248
12130	Cyp3a16	13114	5-:146197177-146230563
12131	MMT00037089	100046250	16-:19195091-19260802
12132	Svs5	20944	2+:164158500-164160129
12133	7-Sep	235072	9+:25094233-25116152
12134	Blmh	104184	11+:76759053-76800878
12135	5730403I07Rik	70487	9-:77214221-77247302
12136	MMT00070724		19-:8553607-8616005

12137	Slc39a11	69806	11-:113231040-113427092
12138	Svs6	20945	2+:164142482-164144187
12139	Cmtm4	97487	8-:106877575-106919596
12140	Mrps15	66407	4+:125724207-125732776
12141	Terf1	21749	1+:15795746-15833512
12142	Zc3hav1	78781	6-:38265671-38304603
12143	Thyn1	77862	9+:26807306-26814917
12144	Epha6	13840	16-:59653314-60605073
12145	BC057079	230393	4+:87770340-87800645
12146	Hddc2	69692	10+:31033212-31048008
12147	MMT00058675		12-:83596328-83597539
12148	Ly6k	76486	15-:74627301-74630403
12149	MMT00000708		17+:20186006-20195358
12150	Trim65	338364	11-:115987380-115992319
12151	MMT00082650		8-:83721076-83722253
12152	Trpc2	22064	7+:109224393-109244848
12153	Lrch1	380916	14-:75155738-75347592
12154	MMT00039105		8-:99951122-99953078
12155	Gmfb	63985	14-:47427825-47441885
12156	Setd4	224440	16-:93583705-93604048
12157	Gal3st4	330217	5-:138706146-138712604
12158	Tmub2	72053	11+:102146212-102149891
12159	Lpp	210126	16+:24608192-24981492
12160	Prdm13	230025	4-:21604414-21613109
12161	MMT00006470		6+:3158183-3158405
12162	4930524B15Rik	67592	11+:31865632-31879651
12163	Smad9	55994	3+:54586279-54601426
12164	Gtf2h1	14884	7+:54051635-54078659
12165	Ppp2r1b	73699	9+:50678287-50689343
12166	Prss28	114661	17+:25446349-25448617
12167	MMT00014394	622491	4+:69353011-69353572
12168	H2-Ke6	14979	17-:34162979-34165002
12169	MMT00013125		12-:72947250-72948340
12170	Arid3b	56380	9-:57638319-57682041
12171	MMT00076485	100043803	5-:136626149-136637581
12172	Adam9	11502	8-:26060104-26127260
12173	Acbd6	72482	1+:157405251-157534362
12174	2610027C15Rik	230752	4+:125823225-125827118
12175	Klf15	66277	6+:90416438-90424281
12176	Plcb3	18797	19-:7028203-7044199
12177	Tmem35	67564	X+:130829929-130840510
12178	Slc16a2	20502	X-:100892761-101017298
12179	6430706D22Rik	381280	1+:90153225-90155289
12180	Ank	11732	15+:27396728-27524054
12181	Bcl2l10	12049	9+:75195592-75199447
12182	Immt	76614	6+:71821526-71827382
12183	MMT00028068		2-:5077312-5122574
12184	Ncapd2	68298	6-:125118026-125141604
12185	Cyp2d10	13101	15-:82233337-82237552
12186	Syne2	319565	12+:77141340-77153844
12187	Hoxb13	15408	11+:96055756-96057542

12188	Chek2	50883	5+:111270231-111302765
12189	Prh1	19131	6+:132519859-132522417
12190	Krt18	16668	15+:101858655-101862452
12191	Zfp704	170753	3-:9427019-9610085
12192	2900092E17Rik	67278	7-:134158564-134160866
12193	Aco2	11429	15+:81452911-81745561
12194	ENSMUSG00000(	791344	10-:89207144-89208581
12195	Agbl3	76223	6+:34782464-34807932
12196	Birc7	329581	2+:180663867-180668038
12197	Anxa11	11744	14+:26661655-26706283
12198	Atp13a5	268878	16-:29232754-29321829
12199	MMT00074915		4-:8599925-8600927
12200	Ksr1	16706	11-:78828302-78959856
12201	Rab5b	19344	10-:128116576-128123967
12202	Etv2	14008	7-:31418636-31420871
12203	MMT00009475	100042638	1-:63411292-63441743
12204	Ankmy2	217473	12+:36883776-36914800
12205	Odz2	23964	11-:35881819-35886356
12206	Snx12	55988	X-:98407606-98417829
12207	MMT00039890		13+:3352061-3353235
12208	Pnpla7	241274	2+:24895025-24909577
12209	MMT00014030		3-:59309547-59313773
12210	Supt6h	20926	11-:78020525-78059204
12211	Sfrs17b	338351	X-:34152535-34164331
12212	Tox2	269389	2+:163028899-163101953
12213	Entpd5	12499	12-:85717626-85746419
12214	Olfr1271	258789	2-:90105668-90106585
12215	1810043G02Rik	67884	10+:77441394-77447545
12216	Tmem88	67020	11-:69210737-69211736
12217	Dpm1	13480	2-:168036028-168055853
12218	Slc26a5	80979	5-:21316966-21353147
12219	Cyb5b	66427	8+:109674578-109708000
12220	Pgam1	18648	19+:41986449-41993154
12221	Tbc1d23	67581	16-:57170014-57191340
12222	Tnfrsf11b	18383	15-:54083520-54109892
12223	MMT00043745		3-:68112746-68113103
12224	Il13ra2	16165	X-:143818020-143838375
12225	Tsr1	104662	11+:74711576-74722337
12226	MMT00020912		8+:73649085-73650468
12227	Glrx2	69367	1+:145586777-145593741
12228	Grpel1	17713	5+:36807831-36814345
12229	Sash1	70097	10-:8442990-8605656
12230	MMT00005802		18+:15571723-15573912
12231	Gnao1	14681	8+:96335214-96492726
12232	MMT00060943	100039903	
12233	Mcts1	68995	X+:35953834-35966699
12234	1700025F22Rik	69416	19-:11214153-11239883
12235	Serbp1	66870	6+:67216972-67235957
12236	OTTMUSG00000(	217122	11-:94617233-94622483
12237	Atp8b3	67331	10-:79982329-79998145
12238	Traf3	22031	12+:112458020-112505358

12239	A630077B13Rik	215900	10-:33845867-33847789
12240	MMT00009032		9-:72022327-72022787
12241	Eif2ak4	27103	2+:118214352-118300969
12242	Ube2e1	22194	14-:19115262-19163650
12243	Edar	13608	10-:58063536-58138444
12244	Srl	106393	16-:4493770-4523053
12245	Zfp322a	218100	13-:23446715-23461016
12246	Plac1l	225922	19-:11721769-11735046
12247	Son	20658	16+:91648124-91663002
12248	Tnp2	21959	16-:10788363-10788694
12249	Smu1	74255	4-:40684202-40704847
12250	P2ry14	140795	3-:58918542-58934543
12251	Tcta	102791	9-:108205025-108208336
12252	Gm101	226356	1-:121833818-121844695
12253	MMT00080172		2+:162602426-162631656
12254	BC010304	218236	13-:48974583-49063150
12255	Tas1r3	83771	4-:155233380-155237462
12256	Atp10d	231287	5+:72618907-72690004
12257	EG212225	212225	1-:46068745-46087172
12258	Mdn1	100019	4+:32855625-32862191
12259	MMT00080137		2-:155311398-155312165
12260	MMT00010989		3+:118133360-118176154
12261	Bcan	12032	3-:87791452-87804152
12262	Olfr397	258343	11+:73778111-73779059
12263	Atp6v0d2	242341	4-:19805063-19849754
12264	Strm	20904	12+:81291569-81295430
12265	Yipf1	230584	4+:106987035-107032427
12266	MMT00051604		6-:145264788-145284933
12267	Sec24b	99683	3-:129685683-129744367
12268	Eaf1	74427	14+:32307582-32323043
12269	MMT00014063		16+:15609116-15610074
12270	Slc7a6	330836	8+:108692772-108722610
12271	Entpd3	215446	9+:120448936-120476268
12272	Fshr	14309	17-:89384511-89599952
12273	Olfr572	259093	7+:110076143-110077098
12274	Erf	13875	7-:26029186-26035766
12275	MMT00014892		19+:40002553-40027309
12276	Hmga1	15361	17+:27693843-27700608
12277	V1ri10	171279	13+:23191017-23191913
12278	Plxna4	243743	6-:32094332-32094929
12279	Rpl22l1	68028	3+:28704432-28706337
12280	Cln5	211286	14+:103469512-103476847
12281	Tacc2	57752	7+:137836069-137908218
12282	Lce1b	68720	3-:92459571-92460848
12283	Fbxo39	628100	11+:72127953-72132920
12284	Tnfrsf4	22163	4+:155387803-155390697
12285	Setd5	72895	6+:113059406-113103418
12286	EG545208	545208	17+:47547200-47548727
12287	6430706D22Rik	381280	1-:90165525-90174152
12288	Olfr392	259008	11-:73627645-73628582
12289	Ext1	14042	15-:52900093-53177500

12290	4930542N07Rik	75185	X+:150916544-150919225
12291	Lim2	233187	7+:50685488-50691361
12292	Htatip	81601	19-:5602471-5609920
12293	E430029J22Rik	100033459	1+:175613580-175626363
12294	Hes5	15208	4+:154335104-154335783
12295	Guf1	231279	5+:69948149-69963042
12296	Rrad	56437	8-:107152350-107154813
12297	Upk1b	22268	16-:38773302-38788156
12298	Lad1	16763	1+:137715339-137729537
12299	Thbs1	21825	2+:117938378-117949995
12300	Zfp568	243905	7+:30769095-30784209
12301	Atpaf2	246782	11-:60214131-60230507
12302	Cbx1	12412	11+:96662121-96668074
12303	Lmo4	16911	3-:143851692-143865271
12304	Olfr109	258832	17+:37603152-37604097
12305	Ubl4	27643	X-:71612739-71613866
12306	Vsig1	78789	X+:137442158-137473497
12307	MMT00030713		2-:22616795-22621005
12308	MMT00005726		18-:12657194-12657622
12309	Gimap8	243374	6+:48597252-48600842
12310	Csn1s1	12990	5+:88096271-88111598
12311	Rpp40	208366	13-:35988581-35998161
12312	Ms4a6d	68774	19-:11661096-11679276
12313	Prl7d1	18814	13-:27800891-27808606
12314	Slc25a34	384071	4-:141176326-141179657
12315	Srebf1	20787	11-:60013100-60016311
12316	Mpa2l	100702	5-:105587936-105600749
12317	Tfdp2	211586	9+:96188017-96218581
12318	Olfr1441	258678	19+:12496800-12497757
12319	Mfap2	17150	4+:140566595-140571889
12320	Gstm7	68312	3-:107729253-107734635
12321	Igh-VJ558	16061	12-:115725304-115725833
12322	Ndufs5	595136	4-:123389953-123395404
12323	Rgag4	331474	X-:99263928-99265981
12324	Vwa3a	233813	7+:127883542-127911745
12325	Lsm7	66094	10-:80315578-80317954
12326	Arhgef3	71704	14+:28149241-28217089
12327	Ccl11	20292	11+:81871423-81876453
12328	MMT00039977		13-:12222142-12275641
12329	Sox6	20679	7-:122620184-123138600
12330	Aldh6a1	104776	12-:85771672-85791900
12331	Homer3	26558	8+:72806936-72818248
12332	Ankrd5	319196	2+:136357918-136387834
12333	Fcrl1	229499	3+:87180370-87189237
12334	Gcg	14526	2-:62312577-62321790
12335	Plcb4	18798	2+:135734080-135840679
12336	Timm13	30055	10-:80362660-80363714
12337	Khdrbs3	13992	15+:68758849-68923946
12338	Snrpf	69878	10-:93046055-93052403
12339	BC025546	234728	8+:112741888-112748197
12340	Pcsk6	18553	7+:73007022-73195272

12341	EG330731	330731	8-:32847179-32850202
12342	Neo1	18007	9-:58724347-58884115
12343	Atf5	107503	7-:52067627-52071942
12344	MMT00034062		4+:37976231-38331106
12345	MMT00061111		Y-:1893121-1903212
12346	Myoz2	59006	3-:122709125-122737867
12347	MMT00014037		13-:33668741-33688437
12348	Rps6ka6	67071	X-:108501802-108649953
12349	Tcstv1	54382	Un_random-:12669-38049
12350	Zfp445	235682	9-:122766592-122771140
12351	Dnm1l	74006	16-:16311997-16359075
12352	MMT00079007		
12353	Barx2	12023	9-:31653864-31720658
12354	Slc26a11	268512	11+:119238563-119242361
12355	AI507611	245884	6+:29232706-29240681
12356	MMT00061517		16-:91539282-91540305
12357	Wipi2	74781	5+:143105496-143145623
12358	A430083B19Rik	218739	14+:14503430-14514979
12359	Rpl22	19934	4+:151699970-151706585
12360	EG630825	630825	X-:53208692-53213180
12361	MMT00079310		7-:110988540-110988941
12362	Shoc2	56392	19+:54062170-54105765
12363	Ifitm6	213002	7-:148201597-148202803
12364	EG637021	637021	17+:18198896-18211184
12365	Tdrkh	72634	3+:94217184-94233380
12366	Nwd1	319555	8+:75233395-75235772
12367	MMT00072365		X+:141282222-141283172
12368	Prickle3	54630	X+:7234519-7248450
12369	Ubash3a	328795	17+:31346693-31379566
12370	Gm1123	382097	9-:98914451-98936121
12371	1700128F08Rik	76669	9+:8221888-8241987
12372	MMT00039397		
12373	Sirt2	64383	7+:29551785-29573677
12374	Snx8	231834	5-:140816913-140865223
12375	Gm941	381112	17+:80767521-80769754
12376	MMT00011143		12-:78724038-78725029
12377	MMT00050540		8-:49154859-49156054
12378	MMT00075531		15-:65026999-65044593
12379	Mon1b	270096	8+:116159730-116179433
12380	Rftn2	74013	1-:55227005-55283624
12381	MMT00067966		17+:54559458-54560157
12382	1810009J06Rik	73626	6+:40914787-40918330
12383	Ugt2a3	72094	5-:87753996-87766201
12384	MMT00056390		
12385	Mrpl33	66845	5+:31916344-31925014
12386	4930579G24Rik	75939	3+:79432979-79436739
12387	Olfr1156	258814	2-:87789426-87790388
12388	8430406I07Rik	74528	2+:144096711-144106196
12389	MMT00022576		
12390	Eps15l1	13859	8-:74864897-74945359
12391	Mrpl9	78523	3+:94247239-94252629

12392	MMT00003217		6+:42930688-42931172
12393	Mtap1b	17755	13-:100194395-100286495
12394	5430413K10Rik	433492	2+:154141922-154144629
12395	Pdzd7	212684	19-:45102104-45120048
12396	Zfp106	20402	2-:120358216-120365582
12397	Ccnt1	12455	15-:98373642-98398067
12398	Ifi44	99899	3-:151393888-151412813
12399	Dpm2	13481	2+:32426405-32428536
12400	Kifc5c	94118	10+:117031199-117032683
12401	MMT00011281		2+:156994509-156995015
12402	Th1l	57314	2+:174241304-174252998
12403	Slc7a1	11987	5-:149143657-149211398
12404	Col16a1	107581	4+:129774847-129776520
12405	Cyp4f14	64385	17-:33042015-33054023
12406	EG623818	623818	1+:138611805-138690085
12407	Vmn2r67	620672	7-:92284870-92301404
12408	Cryba2	12958	1-:74936517-74939709
12409	Mab21l2	23937	3-:86349973-86351947
12410	2410003K15Rik	75593	6+:49023841-49034716
12411	ENSMUSG00000(	100049172	11-:62563565-62564782
12412	BC004728	207818	15-:100451772-100467643
12413	Psmd3	22123	11+:98543867-98557291
12414	Rap1a	109905	3-:105531200-105604175
12415	MMT00052705		
12416	Rps18	20084	17-:34088944-34092586
12417	Mtap7d1	245877	4-:125909410-125917355
12418	Ifna9	15972	4-:88237717-88238289
12419	MMT00081393		2+:15049374-15050459
12420	Klhl21	242785	4+:151382632-151391786
12421	EG433643	433643	3-:112856806-112857181
12422	Eme1	268465	11-:94505833-94512337
12423	Cyp2g1	13108	7+:27593945-27606216
12424	Ccdc73	211936	2+:104832333-104858060
12425	Olfr1340	258301	4+:118398853-118399801
12426	Dnalc4	54152	15-:79591885-79604866
12427	MMT00052926		10-:24192657-24239774
12428	Hdac4	208727	1-:93825357-94045073
12429	Slitrk5	75409	14+:112078150-112082371
12430	Fst	14313	13-:115243554-115248794
12431	Krtap13	16699	16-:88750991-88751873
12432	Tshz2	228911	2+:169702244-169754676
12433	5033413D22Rik	75973	10-:41270447-41273222
12434	Frs2	327826	10-:116509618-116585557
12435	0610006I08Rik	66836	19+:8845485-8846965
12436	Serinc3	26943	2-:163450407-163470807
12437	Six6	20476	12+:74040880-74043584
12438	Crygc	12966	1-:65118174-65120079
12439	Bola2	66162	7+:133839489-133840857
12440	Sult5a1	57429	8-:125666747-125682155
12441	Rbm42	68035	7-:31438727-31449994
12442	MMT00067811		2-:112443630-112444866

12443	A130030D18Rik	228116	2-:82215536-82232359
12444	Mmp20	30800	9+:7628230-7674968
12445	A830080D01Rik	382252	X+:155991683-156031005
12446	Tnfrsf21	94185	17+:43153945-43224921
12447	MMT00005496		18-:25982074-25982882
12448	Pofut2	80294	10+:76722054-76731444
12449	Ube2l6	56791	2+:84639006-84649869
12450	Pum2	80913	12+:8681486-8757144
12451	Srd5a2	94224	17-:74367047-74397256
12452	AU017962	103341	10+:103606933-103607996
12453	Dnaja1	15502	4+:40670745-40671183
12454	Zscan4d	545913	7-:11650789-11751513
12455	Chrnd	11447	1+:89087211-89095679
12456	Arl6ip5	65106	6+:97160797-97183308
12457	MMT00072579		8-:114760259-114760970
12458	AW146299	381686	5-:145750554-145768921
12459	Olfr1130	258835	2+:87447546-87448491
12460	Pnpla2	66853	7+:148641105-148645993
12461	Cbx5	12419	15-:103029859-103045715
12462	Cox7c	12867	13-:86184425-86186351
12463	MMT00045655		
12464	MMT00047924	20947	7+:117365324-117424850
12465	Kcnj3	16519	2+:55289566-55447936
12466	MMT00079257		5+:88491788-88493396
12467	E330026B02Rik	245026	9-:105598041-105688605
12468	Cyp2d13	68444	15-:82467180-82472455
12469	Dock10	210293	1-:80497648-80630870
12470	Pabpc2	18459	18+:39933172-39935307
12471	Olfr1020	258573	2+:85689610-85690563
12472	Serpinb7	116872	1+:109319265-109349265
12473	Tmem66	67887	8+:35217691-35233897
12474	Trim40	195359	17-:37019889-37027069
12475	MMT00080458		7-:7047956-7050739
12476	Spry3	236576	X_random-:104590-110974
12477	3-Mar	320253	18-:56934808-56971749
12478	Rhot2	214952	17-:25981719-25998028
12479	Ifrd1	15982	12-:40929716-40949751
12480	Cdc42bpg	240505	19+:6314486-6314780
12481	Ddx23	74351	15-:98475814-98493328
12482	MMT00030762		2+:24039162-24040077
12483	5133401N09Rik	75731	13+:58258917-58265746
12484	Tcfap2e	332937	4-:126393245-126413353
12485	Zc3h18	76014	8+:124900686-124940684
12486	Aldh18a1	56454	19-:40624746-40662859
12487	Tbc1d10a	103724	11+:4086835-4115507
12488	MMT00014971		3-:157480150-157480789
12489	Wdr8	59002	4+:153516500-153530924
12490	Morf4l2	56397	X-:133267491-133270156
12491	Ptma	19231	1+:88423310-88427239
12492	Ybx1	22608	4-:118950838-118966995
12493	Ankfy1	11736	11+:72503507-72584723

12494	Thnsl1	208967	2+:21133243-21135307
12495	Casp1	12362	9+:5299288-5306766
12496	Ccl27	20301	4-:41716351-41717085
12497	Kir3dl2	245615	X-:132987795-132992336
12498	Abcg8	67470	17+:85082571-85098138
12499	Avpi1	69534	19-:42197770-42203483
12500	Parp3	235587	9-:106373349-106378511
12501	Zfand6	65098	7-:91764330-91782902
12502	Yy1	22632	12+:110031594-110054463
12503	3110043J09Rik	73167	15+:84550531-84602631
12504	Ptpn3	545622	4-:57205160-57283049
12505	MMT00013047		3-:110080052-110080403
12506	Pask	269224	1-:95206013-95234151
12507	Fcrls	80891	3-:87054690-87067515
12508	MMT00071217		8+:75039113-75040786
12509	Mtap6	17760	7+:106416531-106485511
12510	Rs1	20147	X+:157206093-157232677
12511	Thsd3	217738	12-:88620631-88628087
12512	Eef1e1	66143	13-:38737562-38750879
12513	Whsc2	24116	5-:34239777-34278971
12514	MMT00078654		2-:181687789-181687870
12515	N4bp2	333789	5+:66177371-66218212
12516	Pls3	102866	X-:73031002-73119877
12517	4930573I19Rik	104859	12+:112179662-112209879
12518	Ctsl	13039	13-:64465104-64471600
12519	Bid	12122	6-:120844113-120851770
12520	MMT00057426		X+:76031242-76040294
12521	Sergef	27414	7-:53698544-53895160
12522	MMT00056674		15+:24287282-24287933
12523	1110005A03Rik	74319	11+:116704983-116711050
12524	Dgkb	217480	12+:38810310-38954710
12525	Vmn2r54	666085	7-:13200640-13221490
12526	9-Mar	216438	10-:126492652-126497031
12527	MMT00054567	624112	14+:42092091-42102104
12528	Zfp30	22693	7+:30569808-30579559
12529	MMT00019851		3-:64143486-64159606
12530	1700120K04Rik	68232	7-:134747417-134747956
12531	Psg25	114868	7-:19106511-19150679
12532	Palb2	233826	7-:129250777-129268441
12533	Slc29a3	71279	10-:60177976-60215530
12534	Nol7	70078	13+:43493761-43498230
12535	Sfrs16	53609	7-:20169938-20188592
12536	Ak1	11636	2+:32485023-32490572
12537	MMT00047757		2-:128383137-128413904
12538	Mstn	17700	1+:53118506-53124923
12539	Galnt7	108150	8-:60004857-60131776
12540	Smarca1	93761	X-:45162876-45245719
12541	Stx16	228960	2+:173901923-173924361
12542	Cyp2a21-ps	330491	7+:27702397-27737314
12543	Ube3b	117146	5+:114837102-114871146
12544	Stxbp1	20910	2-:32643481-32702646

12545	Dennd2a	209773	6-:39472755-39473592
12546	Cd7	12516	11-:120898064-120900732
12547	Plekha7	233765	7-:123272067-123276290
12548	Olfr1238	258786	2-:89246287-89247234
12549	Cpsf6	432508	10-:116781727-116805049
12550	Sctr	319229	1+:121903471-121933015
12551	4930443G12Rik	74686	3+:108883384-108919604
12552	Gm967	381217	19-:24047245-24105301
12553	Kpnb1	16211	11-:97022926-97049066
12554	Keap1	50868	9-:21035076-21043380
12555	Zfp87	170763	13-:67616719-67774627
12556	Ass1	11898	2+:31325818-31376149
12557	Gmppb	331026	9+:107951622-107954460
12558	Mrpl23	19935	7+:149719066-149726647
12559	MMT00010105		13+:22818171-22993125
12560	Sulf1	240725	1+:12708583-12851270
12561	MMT00060321		2+:65115428-65116534
12562	MMT00057239		13+:13415628-13447211
12563	Col17a1	12821	19-:47720851-47766584
12564	MMT00036520		1-:20434340-20434650
12565	Grem2	23893	1-:176763915-176851950
12566	Shpk	74637	11+:73012961-73037061
12567	D430039N05Rik	241520	2+:83652793-83720606
12568	Lrrc37a	237954	11-:103312057-103357732
12569	Acat1	110446	9-:53389792-53418427
12570	Wsb1	78889	11-:79053804-79067894
12571	Cdkn2d	12581	9-:21092935-21095415
12572	Zfp90	22751	8+:108939300-108949628
12573	Wbp2nl	74716	15+:82129383-82145053
12574	Ubap2	68926	4-:41141346-41222147
12575	Cib3	234421	8-:74728232-74734310
12576	Kbtbd11	74901	8+:15027402-15029304
12577	MMT00021707		
12578	Ddx1	104721	12-:13226113-13255980
12579	Gm534	218993	14-:49489957-49501508
12580	Ppfibp2	19024	7+:114738779-114888398
12581	Mpp3	13384	11-:101860977-101888219
12582	Thada	240174	17-:84708964-84840434
12583	D630037F22Rik	544696	10-:55737227-55784363
12584	Glt28d2	320302	3-:85675574-85691311
12585	Olfr1508	57270	14-:53082750-53083682
12586	G3bp2	23881	5-:92483539-92512684
12587	Susd3	66329	13-:49326201-49343532
12588	Vps26a	30930	10-:61918024-61949500
12589	MMT00003619		14-:82103076-82103482
12590	Rasgrp1	19419	2-:117108232-117168453
12591	Cep70	68121	9+:99154671-99200361
12592	F13a1	74145	13-:36959056-36997927
12593	Palmd	114301	3-:116630085-116660845
12594	9130227C08Rik	219094	14+:56473200-56516324
12595	Atp8b2	54667	3-:89743405-89765063

12596	Olfr1208	258774	2-:88736826-88737752
12597	4930572J05Rik	223626	15+:74551643-74554670
12598	Olfr801	258282	10-:129106616-129107573
12599	Golt1b	66964	6+:142335761-142350643
12600	Ifi204	15951	1-:175677426-175692019
12601	Chrm5	213788	2-:112319328-112320926
12602	Filip1	70598	9-:79662863-79825653
12603	MMT00076813		8+:5527633-5528371
12604	Acr	11434	15+:89398792-89404858
12605	Nrxn1	18189	17-:90762100-90854392
12606	MMT00003662	546828	4+:11060360-11061255
12607	2900005J15Rik	67261	5+:24607275-24615927
12608	Meis1	17268	11-:18780432-18918669
12609	Tmem91	320208	7-:26454161-26460177
12610	9630031F12Rik	58227	5-:45943802-46030664
12611	MMT00068616		14+:33007082-33015133
12612	Rapgef4	56508	2+:71819343-72094433
12613	Uqcrc1	22273	9+:108838720-108851683
12614	V1ra4	113846	6+:89971881-89972813
12615	5530400B01Rik	71434	7+:149265392-149267338
12616	Asgr2	11890	11+:69906270-69919686
12617	Zfp46	22704	4+:135845707-135850238
12618	EG628004	628004	1+:55283516-55291868
12619	Igf2r	16004	17-:12875274-12963273
12620	MMT00008462		2-:134555858-134556983
12621	Mybphl	68753	3+:108167826-108182974
12622	EG629581	629581	X+:90883953-90884222
12623	Ccdc123	72140	7+:36182111-36223703
12624	Clptm1l	218335	13+:73741748-73758051
12625	AI597468	103266	10+:84565484-84583363
12626	Fut7	14347	2+:25278803-25281891
12627	MMT00061942		12+:85226718-85227928
12628	Akr1c13	27384	13+:4190466-4204842
12629	MMT00027682		11+:24101416-24117601
12630	EG667410	667410	5-:149965708-149967768
12631	Cyp51	13121	5-:4082950-4104449
12632	Ehmt1	77683	2-:24646291-24775104
12633	Klk4	56640	7+:51136541-51141174
12634	Olfr1372-ps1	257871	11-:51968385-51969779
12635	Spt2	20771	Un_random+:4834599-4838176
12636	MMT00000549	213043	1+:58340343-58415929
12637	Itpka	228550	2+:119568072-119576988
12638	Atp6v1b1	110935	6+:83693025-83708733
12639	Bcas3	192197	11+:85166685-85323138
12640	Qrich2	217341	11-:116304225-116320431
12641	Erg	13876	16-:95581812-95751972
12642	Tmc7	209760	7-:125693448-125704673
12643	Fate1	77905	X+:69218323-69234385
12644	Klf14	619665	6-:30907719-30992919
12645	MMT00039341	627312	2-:145335896-145336416
12646	MMT00052842		6+:41221401-41221920

12647	1110002N22Rik	68550	11-:79950176-79955628
12648	Nt5dc1	319638	10-:34023415-34138358
12649	Itgad	381924	7+:135317501-135349015
12650	Klra19	93971	6-:129963051-129976978
12651	Tsc22d4	78829	5+:138203603-138209680
12652	Tcte2	21646	17-:13849457-13898486
12653	C86187	97402	7+:54223111-54231224
12654	4930485B16Rik	380654	10-:92532577-92544642
12655	Tmem180	75146	19+:46431425-46449741
12656	Coq5	52064	5+:115729710-115746981
12657	Phf19	74016	2-:34749274-34769491
12658	MMT00081819		1+:14540512-14541476
12659	Agk	69923	6+:40275481-40346760
12660	MMT00029272		2+:85703569-85704519
12661	4932438A13Rik	229227	3+:36893696-36951565
12662	Suhw4	235469	9+:72136950-72199379
12663	Mdm1	17245	10+:117578979-117585194
12664	C130079G13Rik	229333	3+:59732755-59740649
12665	MMT00041311		6+:109075509-109076528
12666	D8Ertd82e	244418	8+:37162899-37165921
12667	Slc39a2	214922	14+:52513474-52515481
12668	Creb1	12912	1+:64646232-64648449
12669	Mras	17532	9+:99285835-99287184
12670	Spata5l1	214616	2+:122456362-122458440
12671	Cul7	66515	17+:46799000-46801309
12672	Nphs2	170484	1+:158240855-158256564
12673	Cd40	21939	2+:164881199-164897149
12674	Lyzl6	69444	11-:103492136-103500195
12675	Rspo3	72780	10-:29219292-29227524
12676	Nrxn3	18191	12+:90425465-90587024
12677	Palld	72333	8-:64355709-64381460
12678	MMT00007486		14-:124097499-124201881
12679	Cyp4a10	13117	4+:115196847-115206259
12680	Bptf	207165	11-:106948023-106961102
12681	Eif2c2	239528	15-:72936136-72976998
12682	Olfr1176	258767	2+:88179723-88180669
12683	Mgat4a	269181	1-:37542695-37592977
12684	Trim42	78911	9-:97249984-97270377
12685	BC088983	382010	8-:59010317-59029659
12686	MMT00002622		18-:3858153-4009246
12687	Zc3h8	57432	2-:128752005-128769755
12688	Olfr922	258777	9+:38623089-38624024
12689	Pgm3	109785	9-:86448005-86465435
12690	Jak3	16453	8+:74202225-74214455
12691	MMT00021912		X+:32607392-32630212
12692	Ttn	22138	2-:76688051-76688437
12693	Zfp161	22666	17+:69734781-69738342
12694	Riok2	67045	17+:17511539-17530935
12695	Tfdp1	21781	8+:13339673-13377702
12696	RP23-392P11.3	545481	2+:158356890-158369144
12697	MMT00054375		10-:116409791-116410285

12698	Mtf1	17764	4+:124479842-124521993
12699	Vmn2r24	243628	6+:123729039-123754402
12700	Nfia	18027	4+:97444589-97779083
12701	Doc2b	13447	11-:75583459-75609551
12702	Gsdmc2	331063	15-:63655906-63662422
12703	Pcsk1	18548	13+:75227434-75269946
12704	Camk1	52163	6-:113284118-113293916
12705	Maob	109731	X-:16286407-16394489
12706	Plaa	18786	4-:94235310-94269945
12707	Dmrt2	226049	19+:25748220-25753485
12708	Fbxl21	213311	13+:56628219-56633830
12709	Ghitm	66092	14-:37934003-37948452
12710	Snrpb2	20639	2+:142888788-142898585
12711	Nap1l4	17955	7-:150699483-150734995
12712	MMT00059415		5-:138830114-138832821
12713	MMT00007860		8-:40730424-40804227
12714	MMT00019666		X+:44740560-44990843
12715	Clcn5	12728	X-:6760914-6765817
12716	5830417I10Rik	76022	3+:88694731-88714020
12717	1110038F14Rik	117171	15+:76779018-76781159
12718	Dhrsx	236082	4_random+:109716-130125
12719	Apbh	57426	7-:32075538-32076835
12720	Rbm34	52202	8-:129472922-129494969
12721	Tmem126b	68472	7-:97616791-97624508
12722	EG624866	624866	3+:65469877-65587947
12723	Fbxl7	448987	15-:26472631-26482808
12724	D3Ertd751e	73852	3+:41548605-41560074
12725	Bak1	12018	17-:27156756-27165572
12726	Gpi1	14751	7-:34987176-34996825
12727	Dpf2	19708	19-:5897376-5912823
12728	Hax1	23897	3-:89799378-89802608
12729	Cnih4	98417	1+:183074405-183096656
12730	9130019O22Rik	78921	7-:134526789-134537146
12731	Zfp366	238803	13+:99998287-100014043
12732	Hsd17b1	15485	11+:100939724-100941819
12733	Igfbp3	16009	11-:7108383-7113834
12734	Zfpl1	81909	19-:6080764-6084891
12735	MMT00062371	236260	12+:17940360-18340295
12736	Stat3	20848	11-:100750831-100772675
12737	Map4k2	26412	19+:6341249-6353526
12738	EG619597	619597	1+:95872496-95876993
12739	Insl5	23919	4-:102690478-102699333
12740	2310021P13Rik	268721	14+:21527070-21542843
12741	Slit3	20564	11+:34985074-35048710
12742	Pola1	18968	X-:90550105-90877494
12743	Ncan	13004	8-:72618862-72644705
12744	1110034B05Rik	68736	1-:57445082-57463565
12745	Spna2	20740	2+:29821362-29886968
12746	Tbca	21371	13+:95558897-95612852
12747	MMT00045901	100048110	16-:44802949-44805624
12748	Bat2d	226562	1-:164650674-164670647

12749	Gng3	14704	19-:8912297-8913701
12750	Cntd1	68107	11+:101140516-101150015
12751	Sorl1	20660	9-:41772804-41932196
12752	MMT00064206		16-:67593029-67593470
12753	MMT00043344		7+:49744170-49761147
12754	E130304F04Rik	245269	13_random+:108410-110864
12755	Irx4	50916	13+:73398246-73407041
12756	Ablim2	231148	5+:36100556-36227618
12757	Klhl12	240756	1+:136352114-136386009
12758	Pcgf3	69587	5+:108890319-108933828
12759	F8a	14070	X+:70473356-70475128
12760	Esd	13885	14+:75132115-75149653
12761	Tsc22d3	14605	X-:137074075-137077710
12762	Zfp639	67778	3+:32413323-32419660
12763	MMT00075731		17-:34856780-34859197
12764	Mxi1	17859	19+:53404133-53447774
12765	4921511H03Rik	70920	5+:7304820-7305582
12766	EG545047	545047	14-:52333319-52335815
12767	Slurp1	57277	15-:74557075-74558456
12768	Nxt2	237082	X+:138662487-138673352
12769	Tmem177	66343	1-:121804482-121809752
12770	Nola2	52530	11+:51433274-51437216
12771	Tdh	58865	14-:64111295-64127593
12772	Axl	26362	7-:26543681-26573497
12773	Klhl1	93688	14-:96505393-96918180
12774	Pgm1	66681	5+:64484179-64519592
12775	Vstm2b	58188	7+:48154647-48185335
12776	MMT00061492	100045495	7-:11379597-11381473
12777	Rapgef2	76089	3-:78866441-78976997
12778	1700024P16Rik	242594	4+:104668302-104685936
12779	Zfyve26	211978	12-:80383788-80387314
12780	Olfr630	259102	7-:110902663-110905864
12781	Prss1	114228	6+:41408941-41413732
12782	MMT00056302		12-:111557461-111558341
12783	MMT00015517	433743	4-:107432629-107433070
12784	Nudt6	229228	3-:37303911-37318495
12785	Jmjd2b	193796	17+:56491051-56542292
12786	Plec1	18810	15-:76001402-76073843
12787	AI451557	102084	8+:97043130-97046788
12788	Trim69	70928	2+:121986435-122004762
12789	MMT00046128		2+:68624241-68669336
12790	Fhl1	14199	X+:53984990-54046518
12791	MMT00003726		7+:109929991-109930942
12792	Eef1g	67160	19+:9041683-9052670
12793	MMT00071809		5+:5082278-5082919
12794	MMT00082139	100048814	4+:145454073-145886627
12795	Slc20a2	20516	8+:23616945-23680089
12796	BC048943	217874	12-:113217323-113219266
12797	Brdt	114642	5+:107760177-107814713
12798	Dct	13190	14-:118412028-118451407
12799	Spon2	100689	5-:33556578-33560820

12800	Vav2	22325	2-:27119154-27282345
12801	2610301F02Rik	545428	2-:76883781-77008667
12802	Arhgef4	226970	1+:34802162-34869582
12803	Ranbp3	71810	17+:56799593-56851183
12804	Sugt1	67955	14+:79987472-80030597
12805	Bmp7	12162	2-:172695190-172765673
12806	Socs6	54607	18-:88835464-88927889
12807	Faim	23873	9+:98892541-98902122
12808	Fpr-rs3	14290	17-:20760809-20761841
12809	4933432B09Rik	75729	15-:79442696-79489227
12810	Mier1	71148	4+:102787070-102837215
12811	Olfr908	258872	9+:38323618-38324549
12812	MMT00075804		1-:5266188-5266713
12813	MMT00041243		6-:92514611-92606372
12814	Hars	15115	18-:36926524-36942839
12815	Wfikkn2	278507	11-:94098912-94103863
12816	Zfp93	22755	7+:25055443-25061842
12817	Mrpl39	27393	16-:84718527-84735465
12818	MMT00048374		2+:146098882-146132125
12819	Zfyve9	230597	4-:108310917-108453369
12820	Slc6a16	381884	7+:52514523-52528733
12821	Rnf157	217340	11-:116197675-116274350
12822	4930560E09Rik	67737	17+:80615806-80617275
12823	Rab3il1	74760	19+:10092752-10109572
12824	Dbt	13171	3+:116216058-116252310
12825	Gal3st3	545276	19+:5302585-5307893
12826	Zfp354c	30944	11-:50628066-50639960
12827	Stmn4	56471	14+:66973188-66980510
12828	MMT00047250		X-:23819283-23842045
12829	MMT00018827	223286	15-:4938821-5013751
12830	Cmah	12763	13+:24508768-24560510
12831	Tm6sf2	107770	8+:72596874-72603962
12832	4930556L07Rik	75283	2-:151573315-151575713
12833	Drp2	13497	X+:130939319-130987938
12834	Fabp7	12140	10+:57504745-57508162
12835	Ckmt1	12716	2+:121184376-121189472
12836	EG629820	629820	12+:40949630-40955775
12837	Olfr800	258541	10+:129096863-129097799
12838	Tec	21682	5-:73146978-73259722
12839	Fh1	14194	1-:177531209-177555720
12840	MMT00009117		2-:148244666-148245041
12841	Defcr3	13237	8+:22427086-22767199
12842	Gphn	268566	12+:79710182-79785754
12843	Zfp644	52397	5-:107045761-107067654
12844	Slc1a7	242607	4+:107640938-107686133
12845	F11	109821	8-:46326523-46347385
12846	MMT00016174		9-:5554270-5554398
12847	Sar1b	66397	11+:51577208-51605423
12848	MMT00006400		6-:68784815-68830668
12849	Sftpd	20390	14-:41985502-41998452
12850	Galnt13	271786	2+:54912875-54965942

12851	Apod	11815	16-:31297205-31314535
12852	EG432879	432879	14+:79851673-79853360
12853	Phc3	241915	3-:30805916-30860805
12854	Ndufa13	67184	8-:72418068-72425544
12855	2310022A10Rik	66367	7+:28338289-28367115
12856	Clstn3	232370	6-:124380784-124414744
12857	MMT00054072		
12858	Lepre1	56401	4+:118905550-118920972
12859	Fbxw5	30839	2+:25356337-25360987
12860	Tmem156	243025	5-:65466801-65482794
12861	Rhcg	56315	7-:86738254-86762534
12862	Trem2	83433	17+:48485813-48491596
12863	Fgg	99571	3+:82811817-82818969
12864	Myh10	77579	11+:68505609-68630129
12865	MMT00044296		17+:18960085-18992146
12866	4921517L17Rik	70873	2+:156138183-156201370
12867	MMT00021210	667832	11-:54931618-54932378
12868	Taf7	24074	18-:37801675-37803801
12869	0610011F06Rik	68347	17+:26012069-26014109
12870	Skiv2l	108077	17-:34976351-34987100
12871	Scn7a	20272	2-:66512479-66622975
12872	Rg9mtd3	69934	4+:45310031-45328353
12873	Ndufb11	104130	X-:20192452-20194383
12874	Rhox6	19202	X+:35180231-35183033
12875	Map6d1	208158	16-:20235320-20241386
12876	Htra2	64704	6-:83001277-83004449
12877	Ecd	70601	14-:21139115-21167310
12878	5730601F06Rik	77519	9-:20310419-20325927
12879	Ermp1	226090	19-:29684692-29698148
12880	Fgfr4	14186	13+:55254235-55270098
12881	Lgals8	56048	13-:12533091-12553707
12882	Tmem134	66990	19+:4125986-4132285
12883	Saps1	243819	7-:4582459-4610589
12884	Zfp692	103836	11+:58120611-58128127
12885	Ccl24	56221	5-:136046664-136048842
12886	Acp6	66659	3+:96963222-96980412
12887	1810018F18Rik	69166	19+:58773406-58775813
12888	AI931714	102182	8+:80073279-80086415
12889	A630052C17Rik	320757	11-:29071941-29073541
12890	B230339M05Rik	228850	2+:158246579-158323544
12891	Fat2	245827	11-:55065089-55125759
12892	1700019L03Rik	227736	2-:32632656-32639948
12893	Zdhhc7	102193	8-:122604994-122611981
12894	Tor2a	30933	2+:32612763-32645176
12895	Rpe	66646	1+:66747466-66766379
12896	Olfr415	258709	1+:176321201-176322122
12897	Tsx	22127	X+:100609820-100619919
12898	BC038167	270150	9+:44048776-44055390
12899	Slc39a7	14977	17-:34165217-34168503
12900	Zfp54	22712	17+:21560224-21572118
12901	Fbxl11	225876	19-:4316154-4396645

12902	Pip5k1a	18720	3-:94863522-94910642
12903	AU019823	270156	9-:50415387-50425293
12904	MMT00040704	676923	19+:40265187-40271489
12905	Nfil3	18030	13-:53062848-53064235
12906	Nts	67405	10-:101944807-101953047
12907	Zkscan1	74570	5+:138547900-138549048
12908	Jdp2	81703	12+:86940967-86980827
12909	Olfr604	259073	7-:110531575-110532514
12910	1110028A07Rik	68680	14+:56194503-56195789
12911	2210415F13Rik	70163	11+:58192527-58204040
12912	AI597479	98404	1+:43155554-43172789
12913	MMT00046078		2-:55628810-55629028
12914	9430091E24Rik	434350	8-:113661469-113669413
12915	Slc7a5	20539	8-:124405051-124431532
12916	Fkbp1a	14225	2+:151368356-151387426
12917	Nubp1	26425	16+:10412067-10423623
12918	Gfap	14580	11-:102748660-102758431
12919	Nup210l	77595	3+:89908054-90014380
12920	Mlstd2	67420	7+:120657374-120711896
12921	MMT00075833		12-:93206779-93207726
12922	Lilrb4	14728	10+:51210780-51216415
12923	MMT00016981		16+:10045036-10046094
12924	Zcchc7	319885	4+:44938232-44945079
12925	Farp1	223254	14+:121606471-121682968
12926	Lrmp	16970	6+:145064182-145123448
12927	Sod3	20657	5+:52759179-52759982
12928	Abca7	27403	10+:79460359-79478315
12929	6330578E17Rik	76178	1-:37473936-37486883
12930	Pthr1	19228	9-:110624758-110644804
12931	Tpt1	22070	14+:76245137-76248104
12932	Mpv17l	93734	16+:13940787-13949450
12933	MMT00048320		3+:134598828-134626488
12934	Rbp7	63954	4-:148823797-148829077
12935	Zfp454	237758	11-:50686218-50701033
12936	Pdlim1	54132	19-:40296729-40346023
12937	Uhrf2	109113	19+:30105092-30168212
12938	Fhod3	225288	18+:25056137-25292001
12939	Fzd6	14368	15+:38838799-38868310
12940	H47	109815	7+:73224580-73234291
12941	Elovl5	68801	9+:77765262-77832323
12942	Btbd11	74007	10+:84849617-84851223
12943	Slc25a27	74011	17-:43778848-43803964
12944	Olfr631	258961	7+:111063575-111078425
12945	Spata2L	78779	8-:125756154-125760133
12946	Hoxd4	15436	2+:74560034-74567214
12947	Ferd3l	114712	12+:34613354-34613860
12948	MMT00009927		15-:77200601-77200694
12949	Atg4d	235040	9+:21069725-21075303
12950	Sgsm2	97761	11-:74664116-74673401
12951	Pcdha6	12937	18+:37127284-37347311
12952	Socs3	12702	11+:117829542-117830707

12953	Spin2	278240	X+:150266822-150268784
12954	Gli2	14633	1-:120730637-120786979
12955	Atp5o	28080	16-:91925467-91931870
12956	Wipf1	215280	2-:73272088-73367214
12957	Ccdc101	75565	7+:133792915-133816292
12958	Itpr2	16439	6-:146369939-146450400
12959	Slc6a1	232333	6+:114251043-114267516
12960	Arrdc4	66412	7-:75886496-75894089
12961	MMT00054077		
12962	Caly	68566	7-:147255785-147268447
12963	Erp27	69187	6-:136855907-136870701
12964	Dync1li1	235661	9+:114597948-114632893
12965	Pmm1	29858	15-:81781539-81791297
12966	Slc39a1	30791	3+:90052156-90056994
12967	Plod1	18822	4-:147284170-147310813
12968	Fbxw9	68628	8+:87583985-87591208
12969	Xaf1	327959	11+:72115134-72122426
12970	MMT00077927		12-:41610141-41611250
12971	Dip2c	208440	13+:9549429-9666591
12972	MMT00004439		17-:54493986-54494360
12973	MMT00014886		X-:7418772-7452419
12974	Slc1a6	20513	10+:78243246-78277510
12975	MMT00013656		4-:100240287-100240653
12976	MMT00066041		2+:59972094-59973359
12977	B4galt3	57370	1+:173200518-173207026
12978	Efcab4a	213573	7+:148646996-148652511
12979	OTTMUSG00000(	433490	2-:152418925-152422161
12980	Prl5a1	28078	13+:28234406-28243299
12981	Nek6	59126	2+:38367216-38442085
12982	Cars	27267	7-:150743610-150785961
12983	MMT00062297		10+:95939064-95940012
12984	Cyb5	109672	18+:85020805-85049255
12985	B230112C05Rik	320557	13+:97861771-97901149
12986	Cox4i2	84682	2+:152579908-152590773
12987	Krt86	16679	15+:101303900-101310414
12988	Zfp433	73610	10+:81341710-81343322
12989	1190002H23Rik	66214	14-:79688557-79701442
12990	Dgke	56077	11-:88902047-88921699
12991	MMT00065030		10-:77233378-77234070
12992	1700027A23Rik	76420	3-:105799874-105804417
12993	Map3k2	26405	18+:32322855-32388162
12994	Notch1	18128	2-:26314670-26359157
12995	Tmed9	67511	13+:55694503-55699049
12996	Hormad2	75828	11-:4245816-4341108
12997	Csgalnact2	78752	6-:118058206-118089158
12998	Slc6a19	74338	13-:73818440-73838227
12999	Igfbp6	16012	15+:101974792-101979942
13000	9130404D14Rik	227737	2+:32731743-32780766
13001	EG668525	668525	12+:20670547-20672246
13002	MMT00005328		6+:12807600-12808015
13003	Pdcd5	56330	7-:36427155-36432390

13004	Arhgef10	234094	8+:14940208-15001079
13005	Dkk2	56811	3+:131748871-131842621
13006	Akr1b7	11997	6+:34362389-34373142
13007	4632412N22Rik	320159	17+:72066589-72079008
13008	Adam34	252866	8-:44710646-44761093
13009	MMT00017557	545339	9+:42771931-42774152
13010	Cyp4b1	13120	4-:115215989-115217663
13011	Mis12	67139	11+:70833112-70840636
13012	Sac3d1	66406	19-:6116003-6118518
13013	Cxcl11	56066	5-:92788572-92792303
13014	4733401H21Rik	70831	11+:99769233-99770204
13015	D14Ertd500e	219072	14-:55160622-55173173
13016	Actn3	11474	19-:4861222-4877884
13017	Rbbp6	19647	7+:130112906-130146073
13018	AW544981	213956	15+:80502298-80530852
13019	Als2cr2	227154	1+:59030463-59052558
13020	Diablo	66593	5-:123961919-123974137
13021	Rad50	19360	11-:53463078-53520799
13022	MMT00002248		X+:10797732-10799101
13023	Adamts1	11504	16-:85795647-85802904
13024	Lpp	210126	16+:24991439-25044246
13025	Irf2	16363	8+:47825144-47932810
13026	Rrn3	106298	16+:13780786-13818206
13027	Ppp2r5c	26931	12-:111714401-111716823
13028	Mesp1	17292	7-:86937127-86938654
13029	4930563M21Rik	75258	9-:55810395-55828739
13030	Pcdhb10	93881	18+:37571526-37573918
13031	Olfr339	258951	2+:36276919-36277848
13032	Gpihbp1	68453	15+:75427070-75428769
13033	MMT00013795		
13034	4933437I04Rik	71246	2+:163886550-163888021
13035	Spata9	75571	13+:76105186-76136416
13036	Zdhhc13	243983	7+:56044371-56082806
13037	Serpinb3b	383548	1-:109050519-109057693
13038	Hoxa13	15398	6-:52208851-52210874
13039	Vps11	71732	9-:44155830-44169731
13040	Mylk	107589	16+:34994949-35001449
13041	Kpna1	16646	16+:36000272-36034095
13042	Aldh5a1	214579	13-:25003382-25029473
13043	Agtrl1	23796	2+:84976789-84977923
13044	5730596K20Rik	109168	19+:7569078-7608690
13045	A330021E22Rik	207686	5-:5580985-5664239
13046	Cd80	12519	16+:38459011-38487012
13047	Stra13	20892	11-:120572256-120575046
13048	Angptl6	70726	9-:20678252-20684154
13049	Sh3tc1	231147	5-:36039831-36066801
13050	4930578C19Rik	75905	X-:17995920-18038495
13051	Rab3b	69908	4+:108562912-108613418
13052	Tnfrsf19	29820	14-:61582711-61656787
13053	MMT00004328		14+:70353264-70353775
13054	Mcm4	17217	16-:15624263-15637399

13055	Med21	108098	6+:146591100-146599120
13056	AW121567	270028	8-:9768259-9770658
13057	Aspn	66695	13+:49639857-49662926
13058	Gpx3	14778	11+:54716451-54723878
13059	Centd1	212285	5-:62993686-63157321
13060	Ccdc100	225523	18-:53882736-53903984
13061	Eml5	319670	12-:100028822-100097115
13062	MMT00016210		11+:85998819-85999238
13063	4931408A02Rik	70967	16+:90826963-90905354
13064	Zbbx	213234	3-:74940335-74955453
13065	Adamts9	101401	6-:92746982-92893486
13066	Spock1	20745	13-:57527763-58009683
13067	MMT00074323		9-:63290972-63291889
13068	Yap1	22601	9-:7932001-8004586
13069	MMT00063010		19+:37308111-37308986
13070	Slc22a23	73102	13-:34274822-34436490
13071	MMT00010913		13-:14056735-14058127
13072	BC022687	217887	12+:114047527-114054463
13073	MMT00046824		5+:10068845-10070679
13074	MMT00014847		13+:51391129-51434428
13075	Mrps2	118451	2+:28323594-28325635
13076	Pias4	59004	10-:80616717-80630668
13077	Cyp2c70	226105	19-:40227852-40261761
13078	Eps8l2	98845	7+:148524900-148548914
13079	Lrp2	14725	2-:69277826-69424086
13080	Olfr1395	258877	11+:48961760-48962713
13081	6-Sep	56526	X-:34453033-34529440
13082	Egln3	112407	12-:55279968-55304835
13083	Myo7b	17922	18-:32118889-32196615
13084	AU042671	269700	5+:121752645-121756115
13085	Nr0b1	11614	X+:83437125-83441285
13086	Ccdc79	320022	8-:106971480-106996847
13087	Cxadr	13052	16+:78302000-78359992
13088	Tspan3	56434	9-:55983694-56008671
13089	F5	14067	1+:166082088-166149666
13090	Rnf128	66889	X+:136145158-136206853
13091	Ccdc54	69339	16-:50589970-50591202
13092	MMT00011921		1+:119637961-120097249
13093	Rassf2	215653	2-:131822119-131855618
13094	Cnn3	71994	3+:121129458-121161123
13095	4921528O07Rik	78258	1-:164954480-165006500
13096	Otor	57329	2+:142904218-142907436
13097	D330045A20Rik	102871	X+:136015034-136088063
13098	Kcnk1	16525	8+:128519069-128554583
13099	Otp	18420	13+:95645581-95653629
13100	Herpud2	80517	9-:24912576-24956075
13101	3110032G18Rik	73121	5+:125483805-125492916
13102	Prkaa1	105787	15+:5110586-5128977
13103	Erlin1	226144	19-:44109437-44144239
13104	Ccdc70	67929	8+:23080246-23084489
13105	Gtf2f1	98053	17-:57142825-57150711

13106	Fbxo41	330369	6-:85422496-85425232
13107	MMT00009003	546543	12+:89459023-89461049
13108	Olfr749	56858	14-:51355895-51356869
13109	Rab33a	19337	X+:45872646-45883409
13110	Lyg1	69541	1-:38003575-38014605
13111	Nalcn	338370	14-:123769162-123999211
13112	Ate1	11907	7-:137537655-137663038
13113	Man2a2	140481	7-:87496182-87516071
13114	Aff3	16764	1-:38250403-38267492
13115	B230120H23Rik	65964	2+:72136243-72280665
13116	Actn2	11472	13-:12361698-12432767
13117	Tmprss5	80893	9+:48910883-48925690
13118	Sfrp2	20319	3+:83570242-83578237
13119	MMT00016763		12+:14936606-14937394
13120	Lactb	80907	9-:66803199-66823291
13121	MMT00081159		1+:4601331-4602573
13122	Alx4	11695	2+:93482590-93518093
13123	Chd1	12648	17+:15842253-15907328
13124	Arfgap3	66251	15-:83130169-83180593
13125	4930534B04Rik	75216	12-:92236930-92322170
13126	Rai1	19377	11+:59953585-60005527
13127	Col5a1	12831	2+:27742204-27893044
13128	MMT00007627		
13129	Bmp8a	12163	4-:122989889-123020046
13130	RP23-166L2.1	242627	4-:113613241-113615457
13131	Pscd1	19157	11-:118027331-118055009
13132	MMT00070380		14+:44864398-44866804
13133	Tmeff1	230157	4+:48598238-48676000
13134	Lipg	16891	18-:75100521-75120899
13135	Ugt2a1	94215	5-:87889425-87915771
13136	Itgb1bp3	69564	10-:80661939-80664801
13137	2610301G19Rik	219158	14-:70537983-70540903
13138	Olfr1030	258581	2+:85823998-85824953
13139	Mid1	17318	X+:166266090-166404099
13140	MMT00005814		
13141	MMT00071569		15-:3695893-3696875
13142	Wdr81	192652	11-:75254454-75259071
13143	Zmym6	100177	4+:126754625-126806890
13144	Calml3	70405	13-:3802140-3803562
13145	Dapl1	76747	2+:59322711-59343030
13146	Pomgnt1	68273	4+:115823199-115832446
13147	1700023I07Rik	76705	X+:22038757-22039484
13148	Xab2	67439	8-:3610089-3621296
13149	Ifi203	15950	1-:175850535-175872523
13150	4921528I07Rik	77049	9-:114185780-114226148
13151	Asb14	142687	14+:27716428-27728444
13152	MMT00053509		5-:106226341-106226732
13153	4930453N24Rik	67609	16-:64766770-64770689
13154	Mtap1a	17754	2+:121115336-121136104
13155	Serpina1f	68348	12-:104927846-104932928
13156	Ifit3	15959	19+:34658043-34663217

13157	Mmp16	17389	4+:17780813-18043542
13158	Pde1b	18574	15+:103333728-103360483
13159	Map4k4	26921	1+:39958074-40081770
13160	Frem3	333315	8+:83135126-83219247
13161	EG634346	634346	1+:99243005-99339300
13162	Pcdh17	219228	14+:84845631-84934510
13163	2700045P11Rik	72555	16+:12244699-12268195
13164	Il4ra	16190	7+:132695800-132721481
13165	5730437N04Rik	70544	17-:5433024-5440256
13166	Stim2	116873	5+:54389914-54511415
13167	Idi1	319554	13+:8885105-8891640
13168	Col8a1	12837	16-:57627025-57632751
13169	Clptm1	56457	7-:20218597-20250365
13170	B4galt5	56336	2-:167126560-167174683
13171	Cacng7	81904	7+:3336684-3366536
13172	Epb4.1l3	13823	17+:69506149-69639325
13173	MMT00065775		5-:151810398-151811930
13174	MMT00032085	628236	19-:33540369-33543377
13175	4930432O21Rik	74670	17+:22099517-22131327
13176	Gpr85	64450	6-:13785791-13786903
13177	Cops8	108679	1+:92500083-92509915
13178	Slc46a2	30936	4-:59918833-59927900
13179	Plxna2	18845	1+:196446095-196640656
13180	Nox4	50490	7+:94395307-94545509
13181	Lcp2	16822	11+:33947168-33991134
13182	Tmem184b	223693	15-:79191114-79209144
13183	Rg9mtd2	108943	3+:137806869-137817721
13184	MMT00017268		8+:32114328-32114626
13185	MMT00053665		12-:11672561-11673857
13186	3110082I17Rik	73212	5-:139835693-139936467
13187	Slc27a5	26459	7-:13573702-13583499
13188	Sumf2	67902	5+:130322885-130339299
13189	Dnajb4	67035	3-:151847982-151873047
13190	Igsf6	80719	7-:128207581-128218044
13191	Cyp4f17	208285	17+:32643457-32665838
13192	Impg1	63859	9-:80160893-80359018
13193	Sohlh1	227631	2-:25698522-25702372
13194	4922502D21Rik	381816	6-:129276672-129281775
13195	Mtif2	76784	11+:29426464-29445255
13196	Obsl1	98733	1-:75471145-75544102
13197	Mett5d1	76894	2-:108932456-109118447
13198	Adcy3	104111	12+:4134325-4212786
13199	Pold3	67967	7-:107230619-107270051
13200	5730449L18Rik	66637	1-:154217882-154233752
13201	Rbl2	19651	8+:93594056-93646925
13202	Sass6	72776	3+:116297796-116332052
13203	Ppp1r14a	68458	7+:30074340-30078410
13204	Vmn2r42	22310	7+:9927006-9944059
13205	EG626215	626215	12+:79298255-79299732
13206	Qprt	67375	7-:134251284-134265543
13207	Sptlc2	20773	12-:88650588-88729151

13208	Tbx20	57246	9-:24545052-24578289
13209	Mcm3ap	54387	10+:75931682-75978602
13210	Nudt1	17766	5+:140807875-140814089
13211	Sbno2	216161	10-:79519743-79603581
13212	Tspan9	109246	6-:127911412-127984615
13213	MMT00048024	100048144	9+:50023455-50023839
13214	Slc12a8	171286	16+:33518431-33663487
13215	EG626858	626858	10-:85173019-85195563
13216	Ccdc106	232821	7+:5008798-5012384
13217	Rnase1	19752	14-:51764854-51766442
13218	C330043M08Rik	330323	6+:55153468-55213858
13219	Trhde	237553	10-:113836079-114238420
13220	EG329521	329521	2-:132040704-132041033
13221	Srd5a3	57357	5+:76569296-76584495
13222	OTTMUSG00000(	544922	13+:21570753-21576605
13223	BC022651	330460	7-:4667145-4676683
13224	Cp	12870	3+:19857178-19892590
13225	Grhl2	252973	15+:37162790-37263402
13226	Ttc32	75516	12+:9036238-9042986
13227	Krtap15	26560	16+:88829253-88830087
13228	Zfp830	66983	11+:82577856-82579429
13229	Abcd1	11666	X+:70961935-70983626
13230	MMT00000808		7+:35168698-35169696
13231	MMT00080853		11+:98046648-98047461
13232	Olfr177	258998	16-:58872044-58872974
13233	Oaz2	18247	9+:65524399-65537263
13234	MMT00050170		1+:119663968-119795744
13235	Park7	57320	4-:150271250-150283959
13236	Pak1ip1	68083	13+:41096628-41108380
13237	Fzd7	14369	1+:59539439-59541701
13238	Nr1h3	22259	2-:91024267-91035273
13239	Wiz	22404	17-:32490999-32525589
13240	Ing1	26356	8+:11556909-11562121
13241	Gm879	380702	11-:66316152-66339296
13242	Sv2c	75209	13-:96731118-96902546
13243	A230046K03Rik	319277	10+:83006651-83059227
13244	MMT00005261		6-:17660584-17672345
13245	Gpr177	68151	3+:159502702-159597865
13246	Klc4	74764	17-:46767579-46782093
13247	Cspg5	29873	9+:110148101-110164724
13248	4833423E24Rik	228151	2-:85323748-85358923
13249	Tchhl1	71325	3+:93272675-93275902
13250	Cst13	69294	2+:148645855-148656145
13251	EG622268	622268	18+:15169754-15170200
13252	Wnt9b	22412	11-:103592053-103611098
13253	Mtvr2	17826	19-:5728087-5731713
13254	MMT00031174	676175	6-:70407469-70408030
13255	Samd7	75953	3+:30645285-30656383
13256	Sh2d1b1	26904	1+:172207506-172214810
13257	Nadk	192185	4+:154936512-154965098
13258	Tbc1d19	67249	5+:54200906-54294771

13259	Rgag1	209540	X+:139536971-139538840
13260	2210011C24Rik	70134	8-:86534900-86536070
13261	Aldh7a1	110695	18-:56685389-56732524
13262	Zfp410	52708	12+:85658071-85684673
13263	Chchd5	66170	2+:128955528-128959845
13264	Camk1g	215303	1-:195172548-195196424
13265	MMT00068882		14+:41370482-41371580
13266	Man1c1	230815	4-:134117734-134259384
13267	EG628822	628822	17-:53726607-53737988
13268	1600015I10Rik	69761	6+:48882536-48883679
13269	MMT00039476		11-:29501785-29502542
13270	Spag1	26942	15+:36109122-36165365
13271	Idh2	269951	7-:87239735-87260194
13272	Rcan3	53902	4-:134972304-134981349
13273	MMT00033789		12+:105503304-105506842
13274	Qk	19317	17-:10403051-10512719
13275	V1ra3	113845	6-:89985927-89986835
13276	Slc13a1	55961	6-:24038294-24118092
13277	9530015I07Rik	619310	9+:21971843-22005468
13278	0610025J13Rik	78687	4+:97988088-97998482
13279	Ifitm1	68713	7+:148154112-148155711
13280	MMT00038895		8-:77798373-77799290
13281	Tmcc2	68875	1-:134252893-134287760
13282	2310005N03Rik	66359	1+:180249289-180252824
13283	Zdhhc2	70546	8+:41509193-41595619
13284	Olfr128	383243	17+:38060512-38084543
13285	Cd276	102657	9-:58372105-58388663
13286	Slc23a1	20522	18-:35779059-35786791
13287	Dnajb14	70604	3+:137531123-137571895
13288	AA407270	102371	9-:71352152-71440143
13289	Dusp10	63953	1+:185858333-185898756
13290	MMT00047906		17+:78027607-78068313
13291	Ccdc94	72886	17+:56098596-56107704
13292	Sntb2	20650	8+:109459651-109536882
13293	Gsta2	14858	9-:78178848-78194952
13294	MMT00047713		2-:118419817-118420927
13295	EG625054	625054	17-:79250129-79251571
13296	Aim1	11630	10-:43670440-43724696
13297	Emb	13723	13+:118009552-118062905
13298	Ankfn1	382543	11-:89327359-89430937
13299	Areg	11839	5+:91568813-91575772
13300	Pdpn	14726	4-:142857348-142889313
13301	Hey2	15214	10-:30552165-30562589
13302	Bche	12038	3-:73443777-73506030
13303	4933402E13Rik	74437	X+:59543538-59544839
13304	Gadd45b	17873	10+:80392835-80394949
13305	Mknk1	17346	4+:115511840-115550969
13306	Cables2	252966	2-:179993249-179999181
13307	Hsd3b4	15495	3-:98245429-98314448
13308	Kit	16590	5+:75971048-76052744
13309	Tgtp	21822	11-:48871734-48877545

13310	Nol8	70930	13+:49748444-49774383
13311	Prl3c1	27372	13+:27291192-27295616
13312	Syne1	64009	10+:5277561-5324786
13313	MMT00058564		X-:151938548-151939467
13314	Tnfrsf25	85030	4+:151490187-151493947
13315	Slc24a3	94249	2+:145442334-145466095
13316	MMT00038617		13-:84478921-84479766
13317	Pi4kb	107650	3+:94778620-94811099
13318	Adamtsl2	77794	2+:26937901-26964495
13319	Gsx1	14842	5+:148000271-148002520
13320	Olfr156	29846	4-:43833007-43834310
13321	MMT00003602		14-:86615858-86616158
13322	Slc36a4	234967	9+:15527957-15543232
13323	MMT00011678		6-:72023056-72024126
13324	Tmem184a	231832	5-:140280915-140283666
13325	BC043301	210104	7+:50827958-50847420
13326	Trim33	94093	3+:103083801-103159487
13327	Vmn2r95	328759	17+:18576804-18589286
13328	Ly6h	23934	15-:75395507-75397244
13329	Osbp2	74309	11-:3604079-3763870
13330	Lamp2	16784	X-:35758240-35809556
13331	MMT00058856		3+:45081884-45101549
13332	Hsbp1	68196	8+:121868437-121872827
13333	MMT00048365		X-:87049908-87051157
13334	Rabggta	56187	14-:56334713-56341013
13335	Rnf34	80751	5+:123300196-123318954
13336	MMT00046340		11-:3544556-3549969
13337	Pard3b	72823	1+:62056877-62391572
13338	Mcpt1	17224	14+:56636800-56639228
13339	D630042F21Rik	330428	6+:129462572-129468713
13340	BC056923	227358	1+:93263057-93268147
13341	Tnip2	231130	5-:34838744-34856628
13342	Eapp	66266	12-:55774454-55796806
13343	V1rd6	81013	7-:5959352-5962612
13344	2310002L09Rik	71886	4-:73585274-73596752
13345	Mmrn2	105450	14+:35188648-35217468
13346	MMT00052558	171266	5-:3000139-3001058
13347	Abi3	66610	11-:95693706-95703474
13348	Ank3	11735	10+:68996913-69487702
13349	MMT00014600		8-:107199431-107231509
13350	Adcy5	224129	16+:35156680-35305173
13351	Zrsr1	22183	11+:22872036-22874908
13352	Usp38	74841	8-:83504624-83511579
13353	Flrt3	71436	2-:140484230-140497206
13354	MMT00077747		X-:154083345-154089083
13355	Foxs1	14239	2-:152757642-152761001
13356	Aldh3b2	621603	19+:3964465-3981653
13357	Rpp14	67053	14+:8912880-8924348
13358	Cyb5r3	109754	15-:82983925-83002608
13359	BC043098	214469	1-:34869854-34899808
13360	2810006K23Rik	72650	5+:124778101-124791091

13361	C1qtnf2	69183	11+:43287773-43299577
13362	Ddt	13202	10-:75233977-75236119
13363	Pcmt1	18537	10-:7350034-7383048
13364	Nrf1	18181	6+:29998052-30101995
13365	Pdc	20028	1+:152166546-152181035
13366	Olfr1110	258765	2-:86975537-86976476
13367	MMT00060916	624083	5-:138160983-138163193
13368	Jrk	16469	15-:74536060-74538296
13369	1110058L19Rik	68002	1-:24002784-24012479
13370	MMT00051799		12-:116358116-116532715
13371	Peli2	93834	14+:48740614-48876497
13372	Slc11a1	18173	1+:74421802-74432626
13373	Cacna2d3	12294	14-:29718128-30535050
13374	Wipi1	52639	11-:109434841-109472695
13375	Terf2	21750	8-:109593833-109620324
13376	Alg2	56737	4-:47482742-47487176
13377	Nod1	107607	6-:54873942-54899755
13378	0610007P08Rik	76251	13+:63916639-64001605
13379	Xpnpep1	170750	19-:53065879-53113086
13380	2900026A02Rik	243219	5-:113519079-113592128
13381	Dnm3	103967	1-:163920359-164161902
13382	Bco2	170752	9-:50341256-50358807
13383	Cybb	13058	X-:9015341-9046376
13384	Fmo3	14262	1-:164883930-164914622
13385	Stard8	236920	X+:96256066-96268538
13386	Crisp4	78081	1-:18105284-18135985
13387	Olfr1093	258363	2+:86625888-86626855
13388	Klra18	93970	6-:129993762-130017276
13389	Six6os1	75801	12-:73982095-74018752
13390	Ddx31	227674	2+:28695846-28761087
13391	Ovch2	244199	7-:114925483-114932246
13392	Pramel7	347712	2-:87329241-87332575
13393	OTTMUSG000000	433424	2+:44966657-44968822
13394	MMT00054980		13+:8127312-8130100
13395	Ube2m	22192	7-:13620587-13623327
13396	MMT00077496		7+:94012953-94015158
13397	Mlycd	56690	8+:121934085-121934987
13398	Ccdc98	70681	5-:101243228-101244302
13399	Slc16a3	80879	11+:120816560-120820084
13400	Haao	107766	17-:84230495-84246089
13401	Prap1	22264	7+:147279301-147283082
13402	MMT00042033		
13403	Wac	225131	18+:7868830-7927515
13404	MMT00082585		1+:24078764-24079635
13405	Rabep2	70314	7+:133572953-133589414
13406	Atp1b2	11932	11-:69414703-69419019
13407	2310014G06Rik	654795	14-:56515986-56519059
13408	MMT00079369		14+:52689116-52689675
13409	Stk38	106504	17-:29107831-29144882
13410	Zfp192	93681	13-:21618291-21618880
13411	Gpr137c	70713	14+:45840065-45898926

13412	Fhdc1	229474	3+:84247942-84248638
13413	Cnih2	12794	19-:5092873-5098418
13414	Mrgprh	80978	17+:13068899-13070708
13415	Gucy1b3	54195	3-:81836848-81878607
13416	Tlr6	21899	5-:65344219-65346821
13417	1700025G04Rik	69399	1-:153739394-153937379
13418	Lgi4	243914	7+:31844953-31855761
13419	Stxbp6	217517	12-:45956208-46175340
13420	MMT00065419		13+:88687340-88687792
13421	Ahctf1	226747	1-:181658339-181670214
13422	Usp3	235441	9-:66366146-66440896
13423	ENSMUSG00000(	791337	15-:54943897-54945279
13424	MMT00039710		6-:67904198-67904684
13425	Aasdh	231326	5-:77304474-77331009
13426	BC052040	399568	2+:115407505-115602766
13427	MMT00017298		8+:36865261-36878007
13428	Gpr20	239530	15-:73525891-73544267
13429	1700063I17Rik	68223	7+:138478055-138480229
13430	MMT00014656		15+:101334252-101439438
13431	MMT00038058		13+:83971918-83973422
13432	Nr2e3	23958	9-:59790957-59797748
13433	Tubd1	56427	11+:86358759-86380701
13434	Bcl2l11	12125	2+:127954368-127984064
13435	Cdk6	12571	5+:3344311-3522225
13436	Dsg1a	13510	18+:20469403-20478833
13437	Zbtb33	56805	X+:35543017-35547717
13438	Btg3	12228	16-:78360106-78373669
13439	MMT00061352		3+:30873552-30873981
13440	Igh-VX24	195176	12-:114826474-114826943
13441	Lipa	16889	19-:34567318-34599337
13442	4930500O05Rik	242409	4+:43682158-43704242
13443	MMT00053568		5-:96802525-96930214
13444	Lrrcc1	71710	3+:14533786-14572657
13445	Stk32b	64293	5-:37838066-38108392
13446	Elof1	66126	9-:21917433-21921616
13447	Optn	71648	2-:4967021-4975762
13448	Brunol4	108013	18-:25645293-25696260
13449	Tmem103	72341	9+:110207699-110224605
13450	Slc26a9	320718	1+:133640596-133666373
13451	Tbcb	66411	7-:31009158-31016797
13452	D1Ertd471e	27877	1+:168199437-168238064
13453	Vmn2r18	632671	5-:152364236-152389509
13454	Maged2	80884	X-:147241252-147248825
13455	Cenpe	229841	3+:134875581-134936484
13456	Coq6	217707	12+:85702944-85717057
13457	Hexa	15211	9+:59387379-59412914
13458	BC050210	381337	1-:36620931-36665376
13459	Rgs4	19736	1-:171673799-171677654
13460	Arl6ip4	65105	5+:124566116-124568204
13461	MMT00003593		14+:8633670-8635295
13462	Fbxo3	57443	2+:103868016-103895190

13463	Mcrs1	51812	15-:99073350-99078996
13464	Mc4r	17202	18-:67018697-67092999
13465	1700008P02Rik	69347	3-:6615422-6620445
13466	MMT00046952		2-:87006843-87007477
13467	Ndufb7	66916	8+:86094726-86095522
13468	Zfp518b	100515	5-:39060538-39062220
13469	Ccrl1	252837	9-:104000473-104029263
13470	Actr3	74117	1-:127289488-127332305
13471	Cln8	26889	8+:14889045-14897938
13472	B230219D22Rik	78521	13+:55794446-55804860
13473	Nhsl1	215819	10+:18127480-18232316
13474	Myct1	68632	10-:4740984-4752760
13475	Morn2	378462	17+:80689549-80696815
13476	Rin3	217835	12+:103564004-103629065
13477	RP23-248K2.4	210535	11+:46337752-46349117
13478	Olfr1125	259030	2+:87274645-87275600
13479	4632433K11Rik	77043	7+:4985679-4988370
13480	Zdhhc21	68268	4-:82450855-82505446
13481	Gpnmb	93695	6+:48986632-49006779
13482	Nxph4	104080	10-:126962525-126971615
13483	MMT00017256		11+:4463600-4464526
13484	Olfr1048	259016	2-:86076029-86076990
13485	MMT00022214		6+:149310022-149312645
13486	Muc5b	74180	7+:148876260-149058989
13487	A230051G13Rik	216792	11-:58970557-58977231
13488	Art4	109978	6-:136796971-136806121
13489	D230025D16Rik	234678	8+:107749204-107776949
13490	Vps35	65114	8-:87784292-87823415
13491	Plk2	20620	13+:111185267-111191055
13492	Arrdc1	215705	2-:24780872-24790723
13493	Ring1	19763	17-:34158154-34160391
13494	Nrcam	319504	12+:45633555-45701150
13495	Abcb10	56199	8-:126476360-126506752
13496	H1fnt	70069	15-:98086413-98087697
13497	Pax8	18510	2-:24276080-24331076
13498	Aqp9	64008	9-:70960010-71010893
13499	St7l	229681	3+:104667477-104729823
13500	OTTMUSG00000(	100038464	3-:96361207-96361818
13501	Prkx	19108	X-:75006337-75041320
13502	Tmem198	319998	1+:75479664-75482279
13503	ENSMUSG00000(	100038757	16-:89041165-89041697
13504	Lair1	52855	7-:3960607-4014790
13505	1700018P08Rik	73330	8+:119013641-119014823
13506	Gm1815	211895	8-:42742948-42920825
13507	Spats1	71020	17-:45585885-45611952
13508	Ormdl1	227102	1+:53353973-53367088
13509	Gm1323	383710	2+:68406898-68408885
13510	MMT00078434	654470	5-:122691531-122714453
13511	Aytl1b	70902	5+:107860567-107864059
13512	Tmem144	70652	3-:79617600-79646601
13513	Trim25	217069	11+:88860769-88881607

13514	Apol9b	71898	15+:77559508-77566811
13515	Crabp2	12904	3+:87752609-87757294
13516	Fibcd1	98970	2-:31671857-31701302
13517	MMT00015234		3-:138240292-138241371
13518	Pole	18973	5+:110715406-110766474
13519	Sgcg	24053	14-:61840280-61877261
13520	MMT00029899		12+:53179996-53180611
13521	Ears2	67417	7-:129180728-129210558
13522	Slfn5	327978	11+:82769763-82776439
13523	Olfr1179	258919	2-:88242169-88243089
13524	EG434168	434168	7-:46701201-46701635
13525	Dgka	13139	10-:128157451-128174146
13526	Tnfrsf10b	21933	14+:70167338-70183142
13527	Ctss	13040	3+:95330765-95360316
13528	Capn12	60594	7+:29666674-29678605
13529	Abi3bp	320712	16+:56574500-56690240
13530	Gm93	225497	18+:50441305-50442675
13531	Sox30	214105	11+:45793955-45805613
13532	MMT00075762		17+:32059273-32088036
13533	Col27a1	373864	4+:62992026-62995172
13534	MMT00001967		14-:87841015-87841533
13535	Rgs18	64214	1-:146600436-146622558
13536	Vmn2r99	665376	17+:18831062-18844113
13537	Hbb-y	15135	7-:111000388-111001666
13538	Otc	18416	X+:9829558-9897379
13539	Tcfap2d	226896	1+:19098327-19156405
13540	6330409N04Rik	66674	14-:62152713-62175725
13541	Nphs1	54631	7+:31245234-31271734
13542	Gpn3	68080	5+:122822516-122832776
13543	Zfp617	170938	8+:74446762-74457899
13544	Parp8	52552	13-:117643725-117666399
13545	Tmie	20776	9-:110768550-110782587
13546	MMT00010242		14-:33080134-33080921
13547	Sync	68828	4+:128964928-128985630
13548	MMT00032336		7-:3935393-3935789
13549	Acad9	229211	3+:35964988-35990834
13550	Mbnl2	105559	14+:120724015-120830918
13551	Zfp623	78834	15+:75771484-75779830
13552	Slc39a9	328133	12+:81745496-81779341
13553	Smox	228608	2+:131317678-131350908
13554	Aqr	11834	2-:113926895-114001074
13555	Nlrc5	434341	8+:97027982-97030650
13556	Nlrc5	434341	8+:96999594-97021764
13557	Olfr1443	258693	19+:12754599-12755537
13558	Klk7	23993	7+:51066836-51071435
13559	Ctag2	70062	X+:62300815-62302191
13560	Nucb2	53322	7+:123647882-123684068
13561	Dexi	58239	16-:10530300-10542848
13562	Irak4	266632	15+:94374083-94387384
13563	9930023K05Rik	226245	19+:56536134-56561215
13564	Qrsl1	76563	10-:43593982-43621632

13565	Fcho2	218503	13-:99493362-99576327
13566	EG544888	544888	12+:104521239-104554926
13567	1200011I18Rik	67467	14-:76488238-76510602
13568	Cad	69719	5+:31357090-31380851
13569	1190003M12Rik	68888	6-:87333249-87338849
13570	Olfr171	258960	16-:19624250-19625194
13571	Samd8	67630	14+:22569752-22612510
13572	C85627	546161	9-:109579246-109591430
13573	MMT00075852		5+:67366914-67375378
13574	Pth2r	213527	1+:65357832-65435811
13575	Epo	13856	5-:137924313-137926973
13576	Zfp820	75424	17-:21956087-21958683
13577	Cd1d2	12480	3+:86790479-86793702
13578	Asph	65973	4-:9376231-9596231
13579	1700020D05Rik	75555	19-:5502867-5503751
13580	Adamts16	271127	13-:70877361-70980688
13581	Srxn1	76650	2+:151931470-151935536
13582	Tnnt3	21957	7+:149684747-149701908
13583	EG545306	545306	1-:16757094-16758874
13584	MMT00053640		10-:87733607-87734130
13585	Nckap1l	105855	15+:103284273-103329240
13586	Tsfm	66399	10-:126459511-126467860
13587	MMT00016619		7+:12197581-12199457
13588	MMT00042387		14-:105596999-105600644
13589	Ifnab	15974	4-:88336559-88337131
13590	Mcart6	67062	X-:133549792-133572842
13591	Xrcc2	57434	5-:25197818-25211609
13592	Tceal3	594844	X+:133200998-133202913
13593	4933402D24Rik	74426	1-:63801480-63815843
13594	Ppcdc	66812	9-:57260484-57287930
13595	Creb5	231991	6+:53554454-53644364
13596	Zfp37	22696	4-:61852030-61853452
13597	Rgs6	50779	12+:84086280-84259798
13598	Reg1	19692	6+:78375976-78378660
13599	Hbs1l	56422	10+:21027156-21088699
13600	1700023B02Rik	66935	2-:73122123-73143964
13601	Rnasel	24014	1+:155599113-155609750
13602	Actl7b	11471	4-:56752971-56754228
13603	Bahd1	228536	2+:118741623-118748308
13604	Slc16a13	69309	11-:70030206-70034540
13605	Osbpl10	74486	9+:114887916-115142876
13606	Bre	107976	5+:32000422-32387112
13607	Ifna1	15962	4+:88495990-88496559
13608	Lrrc6	54562	15-:66211551-66332403
13609	Olfr659	259052	7+:111819217-111820185
13610	Btrc	12234	19+:45438257-45603783
13611	Rnf113a2	66381	12+:85758149-85759527
13612	Ihpk3	271424	17-:27281241-27304659
13613	Rptn	20129	3+:93197620-93203364
13614	Zfp60	22718	7+:28521974-28535040
13615	Arl16	70317	11-:120326195-120328924

13616	Mical3	194401	6-:120884016-120992277
13617	Acin1	56215	14-:55261004-55305768
13618	MMT00032504		X+:11444390-11444829
13619	Bckdha	12039	7-:26414964-26443757
13620	Stard5	170460	7+:90780550-90789078
13621	Pkhd1l1	192190	15+:44393316-44399908
13622	MMT00058973		6-:55276137-55278295
13623	Chit1	71884	1+:136035217-136048117
13624	Ubc	22190	5-:125866427-125868631
13625	Apobec1	11810	6-:122527809-122541380
13626	RP23-395H4.4	13706	4-:141370877-141381918
13627	Flrt1	396184	19-:7169646-7171670
13628	Ehd2	259300	7-:16533580-16552887
13629	Psmd2	21762	16+:20651777-20663485
13630	Cdkn1a	12575	17+:29235151-29236507
13631	Gpbp1l1	77110	4+:116265626-116266309
13632	Spock2	94214	10+:59569002-59594377
13633	Peli3	240518	19-:4931858-4943065
13634	Alpk2	225638	18-:65508852-65528037
13635	Ttc25	74407	11+:100407015-100433276
13636	Hist3h2bb	382522	11-:58767550-58768014
13637	Lin9	72568	1+:182578425-182620819
13638	Il11ra1	16157	4+:41709230-41716346
13639	Cnga1	12788	5-:72994937-73035513
13640	Atp6v0a1	11975	11+:100870795-100925025
13641	Nfe2l2	18024	2-:75513570-75542604
13642	Pcdhb4	93875	18+:37467292-37469645
13643	Dlec1	320256	9+:119011683-119056811
13644	Acsl3	74205	1+:78654387-78704313
13645	Dek	110052	13-:47178631-47201197
13646	MMT00034706		2+:132487977-132488328
13647	Msx1	17701	5-:38211731-38215695
13648	Dap	223453	15+:31154187-31204090
13649	MMT00005723		1-:128946635-128947324
13650	Cntn1	12805	15+:91958624-92170542
13651	Osm	18413	11+:4138408-4140012
13652	Ednra	13617	8-:80186110-80248351
13653	MMT00021989		5+:30366014-30366675
13654	Stat1	20846	1+:52179434-52216163
13655	Foxk1	17425	5+:142877469-142936777
13656	Ly86	17084	13+:37437213-37510813
13657	MMT00065461	100040887	1+:100160277-100161054
13658	Dhx30	72831	9-:109986824-110018086
13659	Mapk15	332110	15+:75824257-75911156
13660	Atp5f1	11950	3-:105745617-105762193
13661	A430041B07Rik	328108	12+:66066963-66123559
13662	Magee2	272790	X-:102050811-102052382
13663	Sgip1	73094	4+:102432967-102643781
13664	AU017455	105590	14-:79612461-79614164
13665	Reg3g	19695	6-:78416261-78418868
13666	MMT00039363		

13667	Cbs	12411	17-:31749589-31774086
13668	Skap2	54353	6-:51809164-51962463
13669	5730410E15Rik	319613	15-:44577786-44583734
13670	Pa2g4	18813	10-:127995698-128003004
13671	MMT00014009		19+:15424103-15437161
13672	Dnm3	103967	1-:164212697-164408127
13673	G6pc3	68401	11+:102050930-102055398
13674	Ifngr2	15980	16+:91547328-91563402
13675	C530008M07Rik	208994	9-:76303579-76393590
13676	MMT00010979		2+:151273791-151273860
13677	Tinagl	94242	4-:129842962-129852366
13678	Vmn2r63	435975	7-:49867874-50183954
13679	Loxhd1	240411	18+:77620275-77623825
13680	Slc12a9	83704	5-:137756179-137773864
13681	C79407	217653	12-:66233724-66273586
13682	BC051142	407788	17+:34553408-34597665
13683	9030420J04Rik	71544	9-:8997098-9006605
13684	Abcd4	19300	12-:85943618-85958320
13685	Ppp1r9a	243725	6+:4853675-4856854
13686	Ttll7	70892	3+:146515368-146578758
13687	Mal	17153	2-:127458963-127481907
13688	Ppa1	67895	10+:61111367-61136911
13689	MMT00081177		1-:6370617-6373187
13690	Pdgfa	18590	5-:139452930-139470786
13691	Snx29	74478	16+:11347963-11403925
13692	MMT00082247		2-:170779646-170780383
13693	Copb2	50797	9+:98464062-98488802
13694	Exdl1	241624	2-:119364483-119373967
13695	Tlr3	142980	8-:46481719-46494053
13696	Dtl	76843	1-:193362745-193399395
13697	Arhgap26	71302	18+:39257282-39531587
13698	Pacsin1	23969	17+:27792626-27848051
13699	Fzr1	56371	10-:80829624-80841115
13700	Olfml2a	241327	2+:38813205-38819269
13701	Butr1	192194	11+:58731558-58740467
13702	Sgk1	20393	10+:21602237-21710209
13703	MMT00011278		2-:156172539-156173733
13704	MMT00048234		9-:66207972-66208407
13705	Osbpl1a	64291	18-:12913842-13021423
13706	Vcpip1	70675	1-:9712774-9738406
13707	MMT00003565		4-:25793463-25795602
13708	Aldoa	11674	7-:133938748-133942613
13709	Wdfy3	72145	5-:102390134-102410608
13710	MMT00013185		11-:95198261-95199922
13711	Chuk	12675	19-:44147825-44181932
13712	Lypd5	76942	7+:25136535-25139268
13713	MMT00005343		6+:10832434-10838917
13714	Igfals	16005	17+:25016893-25018692
13715	Snx33	235406	9-:56765001-56774851
13716	D5Ertd579e	320661	5-:36943139-37064482
13717	Slc6a6	21366	6+:91648994-91705130

13718	9130209A04Rik	77668	18+:47613734-47630522
13719	Ak5	229949	3-:152126502-152330823
13720	Serpina11	380780	12-:105218464-105228163
13721	Anapc7	56317	5+:122872489-122894460
13722	MMT00037048		
13723	MMT00042104		4-:34887475-34888656
13724	MMT00002755		7+:90560486-90562804
13725	Dnaic2	432611	11+:114594206-114618558
13726	Nlrc3	268857	16-:3948868-3954641
13727	Art2b	11872	7-:108727373-108729675
13728	Slc12a5	57138	2+:164793853-164822761
13729	Smap2	69780	4-:120640993-120642227
13730	Scin	20259	12-:40786359-40860770
13731	2210408F21Rik	73652	6+:31170357-31286857
13732	Tpm4	326618	8+:74659176-74677031
13733	Setd3	52690	12-:109344641-109405608
13734	Srp9	27058	1+:184054941-184061921
13735	Rab3d	19340	9-:21714927-21720376
13736	Rraga	68441	4+:86178332-86222763
13737	Igk-V34	16118	6+:68889570-68890084
13738	Nek5	330721	8-:23207928-23224331
13739	Olfr103	258830	17-:37473235-37474175
13740	MMT00022502		2-:155024351-155025012
13741	Hspa4l	18415	3+:40549540-40593502
13742	1700024P16Rik	242594	4+:104621278-104651691
13743	MMT00013696		4-:91773560-91773816
13744	Ptpdc1	218232	13-:48674578-48688681
13745	Slc35e4	103710	11-:3807022-3813971
13746	E330013P04Rik	107376	19-:60229174-60230571
13747	Entpd2	12496	2+:25251477-25256837
13748	MMT00017889		6-:133881935-133882427
13749	Parp9	80285	16+:35939125-35972689
13750	MMT00070601		14+:54136603-54139601
13751	Klk6	19144	7+:51080819-51087156
13752	Map3k8	26410	18-:4331324-4352951
13753	MMT00042249		3-:104221656-104222184
13754	Olfr173	259002	16-:58796676-58797640
13755	1110001D15Rik	68553	9-:105791410-105983039
13756	MMT00012630		14-:85157910-85158920
13757	Cdon	57810	9+:35259660-35311928
13758	Ubtd2	327900	11+:32355476-32418710
13759	Olfr683	259047	7-:112291865-112292823
13760	Lrp6	16974	6-:134400226-134516800
13761	1700021P22Rik	75538	7+:51751961-51756503
13762	Hspa1l	15482	17+:35109676-35116167
13763	Dcn	13179	10+:96942216-96980365
13764	Plcz1	114875	6-:139938242-139989952
13765	RP23-45P4.1	100043757	2+:174469034-174472159
13766	Slc7a9	30962	7+:36234197-36251017
13767	Kcnk15	241769	2+:163683917-163684609
13768	Mrps10	64657	17+:47505834-47515629

13769	Sap18	20220	14+:58417119-58420914
13770	D5Wsu178e	28042	5+:30559204-30592171
13771	Slc23a3	22626	1-:75122117-75130464
13772	Zfp319	79233	8-:97851729-97853473
13773	Plunc	18843	2+:153968620-153974952
13774	Gdf15	23886	8-:73153292-73155994
13775	MMT00051108		2+:129203160-129205119
13776	D12Ertd551e	52635	12+:117519723-117610694
13777	MMT00015864	668030	2-:175918911-176407018
13778	MMT00065405		15-:81673737-81674182
13779	5830404H04Rik	207781	16-:98078375-98144218
13780	Pilra	231805	5-:138264513-138276946
13781	Dnahc6	330355	6-:73141570-73155948
13782	AV249152	216560	11+:21472320-21798637
13783	MMT00003005		7-:112274996-112276973
13784	Arid1b	239985	17+:5242826-5345304
13785	MMT00032015		18+:36769557-36770424
13786	Tha1	71776	11-:117729264-117734788
13787	Kcnj9	16524	1-:174252217-174259413
13788	Ankrd12	106585	17-:66386137-66426429
13789	H2-Q8	15019	17+:35531070-35531975
13790	Olfr909	258873	9+:38331318-38332252
13791	Zfp330	30932	8-:85287519-85298025
13792	Slc25a35	71998	11+:68781632-68786017
13793	Wrb	71446	16+:96367025-96376382
13794	Higd1a	56295	9-:121758658-121766649
13795	MMT00010159		4+:26369707-26370215
13796	Lrch2	210297	X-:143907263-143988395
13797	MMT00046285		2+:83230285-83230752
13798	3110001I20Rik	70354	2-:125563267-125565690
13799	9430031J16Rik	241134	1+:81073777-81084068
13800	Lrp5	16973	19-:3584876-3686554
13801	Adh4	26876	3+:138078472-138093856
13802	MMT00007459		4-:92932323-92932982
13803	Plekhm2	69582	4-:141181649-141219835
13804	Gm281	238939	14-:14662151-14746954
13805	Eftud1	101592	7+:89797135-89926362
13806	Speer3	71026	5+:13791630-13796820
13807	B3galt4	54218	17-:34086856-34088411
13808	Svs7	56872	9-:35415171-35419431
13809	MMT00066585	57748	13-:94204888-94269387
13810	1110036O03Rik	66180	11-:100269770-100276207
13811	Fkbp7	14231	2-:76501103-76511127
13812	MMT00072366		4-:94705730-94706343
13813	Ktelc1	224143	16-:38525157-38550312
13814	Thy1	21838	9+:43638105-43698878
13815	Hoxd9	15438	2+:74536112-74537477
13816	MMT00015290		4-:147943224-147943838
13817	MMT00008817		Y+:627407-630311
13818	Lce1g	66195	3-:92554072-92556260
13819	Il1a	16175	2-:129126448-129130567

13820	Pwwp2a	70802	11+:43495903-43536107
13821	10-Mar	632687	11-:105262962-105315657
13822	Rpl41	67945	10-:127985334-127986030
13823	Olfr1043	258570	2-:86002160-86003104
13824	1700012B07Rik	69324	11-:109649602-109689341
13825	Myoz1	59011	14-:21468329-21475702
13826	Melk	17279	4+:44313851-44377166
13827	Olfr1361	258534	13-:21750236-21750969
13828	Armc2	213402	10-:41636625-41691006
13829	MMT00014696		19+:29047534-29048567
13830	MMT00030474		8-:18656608-18665326
13831	Rhobtb2	246710	14-:70212223-70213520
13832	MMT00077132		
13833	Trap1	68015	16-:4039976-4077810
13834	MMT00016791		10+:81630034-81630162
13835	Lama1	16772	17+:68046626-68171989
13836	Bsnd	140475	4-:106156064-106164845
13837	MMT00073750		1+:163751993-163753167
13838	Erbb2ip	59079	13-:104608872-104623709
13839	Gypc	71683	18-:32687977-32719517
13840	Isg20	57444	7+:86059232-86065047
13841	Mxd4	17122	5-:34519640-34530222
13842	Aurkaip1	66077	4+:155205754-155207166
13843	Rfesd	218341	13-:76139969-76145734
13844	Trib1	211770	15+:59480624-59487539
13845	Ccdc28a	215814	10-:17936732-17954784
13846	Zmym2	76007	14+:57506627-57579738
13847	Tmigd1	66601	11+:76718046-76730088
13848	MMT00005901	329358	2-:24999273-25000843
13849	Klhl20	226541	1-:163019164-163020155
13850	Tmem165	21982	5+:76612974-76638269
13851	Zfp275	27081	X+:70587973-70604301
13852	Rasd2	75141	8+:77737071-77746627
13853	Ccdc142	243510	6+:83052554-83057955
13854	Akap7	432442	10-:24890858-24919851
13855	Olfr618	259049	7+:110745831-110746787
13856	Mapk9	26420	11+:49667761-49696812
13857	MMT00010823		13-:21472651-21472999
13858	Myl1	17901	1-:66970867-66991718
13859	Lmo7	380928	14+:102129371-102318865
13860	Cpt1b	12895	15-:89246835-89255778
13861	Ccdc60	269693	5-:116584036-116586461
13862	Myh15	667772	16+:49065068-49197003
13863	Olfr1188	258921	2+:88399627-88400565
13864	Larp1	73158	11+:57822979-57875537
13865	Papd5	214627	8+:90767083-90783624
13866	Nkx3-1	18095	14+:69808748-69812715
13867	Sgcb	24051	5-:74026498-74038983
13868	Phlpp	98432	1+:107945669-108098892
13869	6330442E10Rik	268567	12-:80062782-80108326
13870	4921521F21Rik	70861	1-:65059262-65079321

13871	2300006N05Rik	69464	11-:99670391-99671206
13872	Cyb5r2	320635	7-:114893483-114900776
13873	Cdkn2aip	70925	8-:48795578-48799530
13874	Prkd1	18760	12-:51442707-51750085
13875	Hsd17b10	15108	X+:148436433-148438982
13876	C530008M17Rik	320827	5+:77239012-77271614
13877	Btnl9	237754	11-:48982337-48996595
13878	MMT00061131	331475	X+:99273641-99278721
13879	Anp32b	67628	4+:46463986-46485395
13880	MMT00057535	631010	17-:22172839-22177526
13881	Vmn2r100	627537	17+:19658845-19669024
13882	Mcm3	17215	1-:20792640-20810358
13883	EG627788	627788	19-:28837489-28838019
13884	Aven	74268	2+:112333120-112471227
13885	Clec7a	56644	6-:129411610-129422790
13886	Hnrpdl	50926	5-:100463746-100467948
13887	Rfx2	19725	17-:56915467-56970386
13888	MMT00063459	329633	3+:49158747-49171281
13889	Agpat2	67512	2-:26448579-26459748
13890	Olfr1263	258790	2+:89855088-89856007
13891	Aqp3	11828	4-:41039838-41045202
13892	Zfp516	329003	18+:83125094-83174661
13893	Slc6a2	20538	8+:95485146-95520980
13894	Ssr2	66256	3+:88383529-88392341
13895	MMT00072628		4-:23444393-23446309
13896	Btbd12	52864	16-:3990721-3994812
13897	Palld	72333	8-:64163528-64238883
13898	MMT00078764		7+:131277122-131798973
13899	Nup54	269113	5-:92844569-92864212
13900	4933417M04Rik	619288	1-:192986567-192988323
13901	EG667795	667795	1+:155152561-155153607
13902	Lcn10	332578	2+:25539022-25541598
13903	Olfr1389	259069	11+:49243979-49244913
13904	Tmc3	233424	7+:90739149-90758569
13905	Map3k1	26401	13-:112536637-112598524
13906	Hmgb4	69317	4-:127937456-127938137
13907	B4galnt4	330671	7+:148247208-148258291
13908	AU018091	245128	7-:3158177-3169106
13909	C030010B13Rik	77310	2-:173405746-173484955
13910	V1rb1	113852	6+:90057268-90058200
13911	Clec12a	232413	6+:129299799-129303642
13912	MMT00048812		7-:76750882-76751774
13913	Gm70	210762	12+:77518609-77540468
13914	Hdac5	15184	11-:102057062-102086153
13915	MMT00039793		4+:98795260-98796875
13916	MMT00063984		X+:58344006-58344903
13917	Rell2	225392	18+:38115281-38118409
13918	Elovl1	54325	4+:118100745-118105222
13919	Synpo	104027	18-:60753636-60764144
13920	MMT00049947		16+:3601980-3602364
13921	9930022N03Rik	245126	7-:3489961-3498596

13922	MMT00008875		Y+:1351339-1353880
13923	Nploc4	217365	11-:120241110-120298836
13924	Lcmt1	30949	7+:130561755-130573862
13925	Lmod2	93677	6+:24547811-24554654
13926	MMT00011018		3-:84716819-84733388
13927	MMT00007324		8-:78918991-78919632
13928	Cdc123	98828	2-:5715712-5765995
13929	Lamb3	16780	1+:195128219-195170070
13930	EG620205	620205	3-:133452331-133454469
13931	MMT00069367	675747	X+:92753651-92754108
13932	Il1f10	215274	2+:24148683-24149287
13933	Usp26	83563	X-:49107135-49150930
13934	Hunk	26559	16+:90284122-90488156
13935	Aldh8a1	237320	10+:21097127-21116390
13936	Rnf166	68718	8-:124990044-124999956
13937	A230107C01Rik	320225	7-:29970455-29975654
13938	Dcps	69305	9-:34931998-34983572
13939	Utp15	105372	13-:99017518-99032967
13940	1700014N06Rik	69389	X+:133461861-133465708
13941	6430706D22Rik	381280	1+:90124801-90127363
13942	Psg21	72242	7-:19237068-19242065
13943	Spata16	70862	3+:26536590-26826401
13944	Brp16	59053	15+:76199327-76201833
13945	Hivep1	110521	13+:42150871-42279880
13946	Slc35b2	73836	17+:45701036-45705072
13947	Crip2	68337	12+:114378757-114383715
13948	Igh-VJ558	16061	12-:115034345-115034796
13949	Btd	26363	14+:32454250-32481369
13950	Fabp4	11770	3-:10204346-10208554
13951	Fgf2	14173	3+:37247771-37303718
13952	Spdyb	74673	5-:143977994-143987561
13953	4930469G21Rik	74933	1-:163084688-163089666
13954	Chst1	76969	2+:92451929-92455407
13955	Dennd2d	72121	3+:106285489-106306662
13956	BC035044	232406	6-:128818327-128841142
13957	Chodl	246048	16+:78931369-78951971
13958	Auts2	319974	5-:131913218-131952896
13959	MMT00077196		19+:11839881-11840246
13960	A930038C07Rik	68169	6+:65646033-65656923
13961	2210404O07Rik	72273	10+:80855806-80858243
13962	Chd7	320790	4+:8678469-8793800
13963	Sel1l2	228684	2-:140055593-140111042
13964	Lpcat2	270084	8+:95433067-95442397
13965	MMT00061510		7+:11330623-11331328
13966	Aatk	11302	11-:119868636-119903062
13967	Wdr41	218460	13+:95746465-95793257
13968	Spin1	20729	13+:51196273-51244563
13969	MMT00021905		6-:99789901-99790336
13970	Tarbp2	21357	15+:102348889-102354105
13971	Enox2	209224	X-:46364625-46641393
13972	Hadhb	231086	5+:30500349-30510627

13973	Ubl4b	67591	3-:107356615-107357991
13974	Flna	192176	X-:71468802-71491856
13975	Slc27a6	225579	18+:58715893-58772523
13976	Bmi1	12151	2+:18603438-18605881
13977	MMT00049981		
13978	Sv2b	64176	7-:82262481-82364906
13979	Ociad2	433904	5-:73713436-73729893
13980	Thop1	50492	10+:80532877-80544964
13981	1700063D05Rik	78500	9+:41014239-41025639
13982	Tnfaip6	21930	2+:51893632-51912108
13983	Copz2	56358	11+:96711189-96722514
13984	Thap3	69876	4-:151356744-151363547
13985	Anapc4	52206	5+:53225320-53258285
13986	MMT00020948		
13987	Atp6v1d	73834	12-:79943975-79962446
13988	Cntnap2	66797	6+:47194400-47249311
13989	Foxp3	20371	X+:7156818-7172360
13990	MMT00040357		19+:39053872-39077776
13991	Carf	241066	1+:60155124-60207876
13992	MMT00045875	100042690	2+:90164353-90165283
13993	MMT00017885		12+:73334913-73360855
13994	Lrrtm4	243499	6+:79971636-79973159
13995	1700034I23Rik	73297	3-:40704120-40706465
13996	Utp18	217109	11-:93720584-93747013
13997	MMT00033247		X-:73232499-73232954
13998	MMT00024160		
13999	Fgf13	14168	X-:56316709-56387240
14000	Apaf1	11783	10-:90453346-90545443
14001	Dolpp1	57170	2+:30247935-30256047
14002	1700055M20Rik	73407	8+:97836854-97851153
14003	MMT00075867		7+:71152654-71165816
14004	Aspscr1	68938	11+:120534353-120570758
14005	MMT00009841		15-:75715075-75719200
14006	Dus4l	71916	12-:32324919-32339691
14007	Idh3g	15929	X-:71024303-71032236
14008	Fbxw15	382105	9-:109455044-109462442
14009	2310030G06Rik	66952	9-:50547803-50554632
14010	Snip1	76793	4+:124743867-124751373
14011	MMT00069899		7+:20048620-20055353
14012	2700023E23Rik	70036	5+:74489127-74490361
14013	MMT00017766		2-:22768812-22769369
14014	MMT00008376		4-:97187838-97190377
14015	Prss3	22073	6-:41323772-41327578
14016	Znrf3	407821	11-:5176336-5238771
14017	Ces5	234673	8+:107450183-107458567
14018	F830045P16Rik	228592	2-:129286051-129289467
14019	Fbf1	217335	11-:116003597-116029455
14020	Chchd3	66075	6-:32742226-33010152
14021	Gpr113	381628	5-:30519973-30524155
14022	Car9	230099	4+:43519878-43526597
14023	Prkg1	19091	19-:31660056-31738771

14024	C78339	97863	13+:46765117-46773430
14025	4930522H14Rik	67646	4-:109177979-109203902
14026	Clcnkb	56365	4-:140960274-140971896
14027	Rpo1-1	20016	17-:46380876-46384913
14028	3110050N22Rik	67306	3+:7503489-7553849
14029	2810022L02Rik	67198	1+:57831768-58003808
14030	Csrp2	13008	10+:110357231-110376550
14031	MMT00002421		
14032	Cog6	67542	3-:52785799-52821159
14033	MMT00077425		6+:66466376-66478451
14034	Tg	21819	15+:66502353-66682168
14035	Lrrc52	240899	1-:169375931-169398144
14036	4833427G06Rik	235345	9-:50889218-50910075
14037	Zp4	664793	13+:11618321-11618975
14038	1700074P13Rik	73481	6-:40870882-40890496
14039	E230008N13Rik	381522	4+:45903234-45962457
14040	Hcst	23900	7-:31202825-31204765
14041	Cldn1	12737	16-:26358962-26371867
14042	MMT00046983	279067	2-:90870934-90871279
14043	4930455C21Rik	76916	16-:38498432-38522709
14044	Vat1	26949	11-:101319784-101327531
14045	Rps4y2	66184	6+:148303177-148304118
14046	Ms4a7	109225	19-:11395526-11409186
14047	Odf2	18286	2+:29748566-29749089
14048	Ccny	67974	18-:9315943-9449981
14049	Fdft1	14137	14-:63765240-63796638
14050	Il12rb1	16161	8+:73332392-73345320
14051	Stim1	20866	7+:109416736-109585352
14052	EG226654	226654	1+:173349231-173350486
14053	Zfp418	232854	7+:7124083-7136262
14054	Oplah	75475	15-:76126864-76140171
14055	MMT00050438		4-:119006855-119008123
14056	Opa1	74143	16+:29586094-29589836
14057	MMT00080613	100041265	7+:133630887-133634249
14058	Dennd4b	229541	3+:90071357-90084595
14059	Cyp4f18	72054	8-:74512380-74533516
14060	MMT00015243		11+:16919211-16920191
14061	2900053A13Rik	554362	8+:3621549-3624226
14062	Pan3	72587	5+:148341504-148360063
14063	Syne1	64009	10+:5028587-5040787
14064	MMT00025629		12+:20283650-20284556
14065	D2Bwg1335e	52838	2-:26204911-26215079
14066	Rab2a	59021	4+:8462800-8533812
14067	Sfpi1	20375	2+:90936953-90955913
14068	B3gntl1	210004	11-:121478804-121534387
14069	MMT00045633		9-:24905761-24905940
14070	Oaz1	18245	10+:80289504-80291749
14071	Marveld3	73608	8-:112476810-112486025
14072	MMT00031664	635326	12+:116828633-116829405
14073	Ndufa4	17992	6-:11850025-11857452
14074	Lrrc2	74249	9+:110854048-110883467

14075	E030049G20Rik	210356	1-:127873502-127923947
14076	Ust	338362	10-:7927082-8110729
14077	Trpv5	194352	6-:41603129-41626221
14078	MMT00074838		16+:31925014-31925776
14079	Cryga	12964	1-:65147027-65149911
14080	2310007F21Rik	66939	9+:63450476-63489698
14081	Tmem29	382245	X-:146832316-146893678
14082	Fosl1	14283	19+:5447697-5455938
14083	Nup205	70699	6+:35127614-35197590
14084	D530033C11Rik	78581	15+:51709037-51714224
14085	Cdkl2	53886	5-:92435182-92472044
14086	2700097O09Rik	72658	12-:56146647-56181097
14087	Ugdh	22235	5-:65804462-65826959
14088	Kntc1	208628	5+:124199733-124271601
14089	Acsf2	264895	11-:94419621-94420289
14090	Pgls	66171	8-:74114795-74116156
14091	Gata5	14464	2-:180059837-180069378
14092	0610007P22Rik	68327	17+:25377143-25379733
14093	Tbpl2	227606	2-:23942585-23946671
14094	Srpk2	20817	5-:23013099-23122182
14095	Nav1	215690	1-:137337418-137481765
14096	MMT00000268		18+:56131887-56132960
14097	Hs3st1	15476	5-:40005174-40146714
14098	Txndc8	67402	4-:57996900-58021996
14099	Nfe2	18022	15-:103078643-103085868
14100	Ccnk	12454	12+:109417809-109440617
14101	Zfp69	381549	4-:120602747-120620174
14102	Evi2a	14017	11-:79340259-79341868
14103	EG245516	245516	X+:91226407-91231500
14104	Mkrn1-ps1	353327	5-:89267019-89268425
14105	Vcp	269523	4-:42992835-43013188
14106	Syk	20963	13-:52743318-52744151
14107	1190007I07Rik	544717	10-:82081432-82085948
14108	Polr3b	70428	10+:84085028-84189922
14109	Fads2	56473	19-:10138655-10175993
14110	Aspm	12316	1+:141351517-141353460
14111	4921533I20Rik	664619	18-:17392832-17394005
14112	F630110N24Rik	73822	10+:80820334-80826909
14113	Hmgb2l1	70823	8+:77517584-77553931
14114	Ccdc23	69216	4+:118868013-118873903
14115	MMT00048392		X-:55883578-55884361
14116	Sp1	20683	15+:102236758-102263109
14117	C1qb	12260	4-:136436062-136442092
14118	Fbxw7	50754	3+:84756132-84781545
14119	MMT00046758		15-:36049854-36050713
14120	Med29	67224	7-:29171383-29177709
14121	Npas1	18142	7-:17041070-17061037
14122	Sesn2	230784	4-:132048727-132066371
14123	Itga2	16398	13-:115626597-115722245
14124	Clrn1	229320	3-:58594041-58730626
14125	Rora	19883	9+:69043346-69098641

14126	Traf5	22033	1-:193821087-193916445
14127	MMT00009389	236598	
14128	Htr1d	15552	4+:135998385-135999492
14129	5430403G16Rik	77200	5-:110103562-110104226
14130	Aamp	227290	1-:74326420-74330543
14131	Ubr5	70790	15-:37897082-37908789
14132	Glo1	109801	17-:30729810-30749551
14133	6720456H20Rik	218989	14+:49066073-49132874
14134	Phf10	72057	17-:15082013-15093866
14135	Eepd1	67484	9+:25289187-25411692
14136	F7	14068	8+:13026045-13035808
14137	Ephx1	13849	1-:182919869-182947586
14138	Anks4b	72074	7+:127317389-127327228
14139	2410005O16Rik	66302	4+:19502340-19533424
14140	Ipo5	70572	14+:121316569-121347253
14141	EG633570	633570	X-:102920421-102920854
14142	MMT00021961		5-:30157973-30158961
14143	Prelid1	66494	13+:55423393-55426631
14144	Dbc1	56710	4-:68423044-68565988
14145	Sgsm3	105835	15+:80808234-80842500
14146	C230095G01Rik	319776	6-:116645307-116648410
14147	Acss2	60525	2+:155343828-155388234
14148	Fbxl3	50789	14-:103480265-103498088
14149	BC048562	434439	9+:108338810-108348412
14150	V1rc6	113863	6+:57208142-57209042
14151	Slc44a1	100434	4+:53494335-53635350
14152	Serhl	68607	15+:82930642-82949691
14153	5031408O05Rik	331491	X-:103430592-103434573
14154	Apof	103161	10+:127705052-127707207
14155	Strn3	94186	12-:52710631-52792874
14156	Med9	192191	11+:59761768-59774588
14157	Pcdh10	18526	3+:45183095-45186401
14158	Dld	13382	12-:32016515-32036318
14159	1190003K10Rik	68880	13-:64582553-64599101
14160	MMT00023287		17+:93730800-93731219
14161	Necap1	67602	6+:122824574-122838959
14162	Vamp3	22319	4-:150423056-150432003
14163	H2-M10.4	224753	17-:36534664-36598925
14164	Elf3	13710	1-:137150156-137155015
14165	MMT00065181		
14166	Cldn15	60363	5+:137443738-137451710
14167	Exo1	26909	1+:177813029-177838863
14168	MMT00060736		7-:109668988-109669502
14169	1700052N19Rik	73419	10-:5906571-5913921
14170	Gpr50	14765	X+:68916941-68921263
14171	Gypa	14934	8+:83017952-83033372
14172	Olfr479	257891	7+:115198626-115199480
14173	Pik3c2a	18704	7-:123483074-123561566
14174	Fcamr	64435	1+:132697478-132711317
14175	Col7a1	12836	9+:108855789-108886920
14176	1500011H22Rik	68948	5-:122814594-122822364

14177	MMT00059964		3+:94948207-94949601
14178	Hgfac	54426	5+:35384244-35391057
14179	MMT00017967		17-:13030800-13031001
14180	Xlr3c	22446	X-:70499879-70510729
14181	Pde5a	242202	3+:122432163-122558249
14182	MMT00058220		15+:40368854-40369848
14183	Gng11	66066	6+:3954226-3958443
14184	Olfr480	56861	7-:115209281-115210310
14185	Poldip3	73826	15-:82956398-82979739
14186	Prpsap1	67763	11-:116332159-116351674
14187	Zic1	22771	9-:91255338-91260383
14188	Ttc8	76260	12+:100158784-100221442
14189	MMT00033425	100042394	5-:66589169-66624981
14190	Ppil1	68816	17-:29387779-29400916
14191	Scn2b	72821	9+:44925959-44935296
14192	Trpv2	22368	11+:62388048-62413798
14193	Gmeb1	56809	4-:131780933-131807767
14194	Defcr23	497114	8+:22194759-22195599
14195	MMT00061107		X-:99919367-99921281
14196	Prtg	235472	9+:72657261-72755670
14197	Fhod3	225288	18+:24868109-24929048
14198	Clca2	80797	3-:144460402-144482448
14199	Epn1	13854	7+:5031841-5049779
14200	Kirrel	170643	3-:86892285-86945031
14201	Casp3	12367	8+:47702802-47724060
14202	Fnbp4	55935	2+:90585526-90621175
14203	Adh7	11529	3+:137880845-137894240
14204	Gm317	207209	17+:25307131-25308855
14205	Ppm1m	67905	9-:106097276-106100666
14206	C030046E11Rik	226089	19+:29641974-29680373
14207	V1rc3	113860	6+:57087346-57088246
14208	Ech1	51798	7+:29610356-29617258
14209	Btg3	12228	16-:78360106-78377030
14210	Utf1	22286	7+:147129754-147131009
14211	Hpcal4	170638	4+:122860440-122868200
14212	Mcf2	109904	X-:57309135-57400820
14213	Rcor2	104383	19+:7344253-7349714
14214	Clca4	229927	3-:144485705-144512266
14215	Rab5a	271457	17+:53618713-53647003
14216	Ttc14	67120	3+:33700135-33706953
14217	Gabra1	14394	11-:41946771-41995802
14218	Med31	67279	11-:72025227-72028534
14219	4930519F09Rik	114676	10-:28641765-28643170
14220	Mrps5	77721	2+:127413161-127429721
14221	Ccdc66	320234	14-:28303848-28306311
14222	MMT00076410		7+:115006493-115007624
14223	1110019N10Rik	68523	8-:107163738-107165628
14224	Hnrnpab	15384	11-:51414387-51420338
14225	Sva	20939	6+:41988392-41992797
14226	Rbm47	245945	5-:66410008-66489418
14227	Lrrc39	109245	3+:116265889-116284342

14228	MMT00061518		1+:30826524-30829177
14229	Smtnl1	68678	2-:84651333-84662809
14230	Snx4	69150	16+:33251541-33299647
14231	Baiap3	545192	17-:25380417-25393232
14232	Mapkapk5	17165	5-:121975059-121995901
14233	Fgfrl1	116701	5+:109123520-109135915
14234	MMT00012377		X-:94080844-94083212
14235	MMT00002524		18+:8958025-8968920
14236	Zfp518b	100515	5-:39062650-39065728
14237	Rac2	19354	15-:78391275-78403111
14238	4632404H22Rik	78755	X-:50596610-50622208
14239	Acpt	546967	7-:51508457-51512388
14240	Mettl7a1	70152	15+:100135219-100135777
14241	C130074G19Rik	226777	1-:186698121-186706870
14242	Prss29	114662	17+:25457227-25459451
14243	Gm600	239151	14+:66253887-66260859
14244	Nap1l1	53605	10+:110910297-110933175
14245	H2-Ke2	14976	17-:34075869-34077250
14246	Nalcn	338370	14-:123677122-123723114
14247	V1rc33	171206	6-:58165101-58166011
14248	Olfr679	259046	7+:112234231-112235177
14249	Lzts2	226154	19+:45095879-45101576
14250	MMT00041856		
14251	Efhd1	98363	1+:89160938-89207366
14252	MMT00044012		12+:45322729-45324190
14253	Olfr466	258816	13+:65253533-65254459
14254	Eefsec	65967	6-:88207864-88396489
14255	Rcsd1	226594	1-:167579076-167639891
14256	MMT00024953		1+:121054452-121054944
14257	Gm1673	381633	5+:34326164-34327657
14258	Ifi47	15953	11+:48900556-48910471
14259	Mon2	67074	10-:122431568-122513431
14260	Mlkl	74568	8-:113835699-113862078
14261	2310066E14Rik	75687	8+:108145081-108146094
14262	Tcfec	21426	6-:16783391-16848441
14263	Arrdc3	105171	13+:81022595-81035299
14264	MMT00063236		7-:61667527-61724407
14265	Hnrnpa3	229279	2+:75499520-75500768
14266	Sox13	20668	1-:135278879-135289795
14267	Naaladl2	635702	3-:23703256-23780422
14268	Rps25	75617	9+:44215755-44221097
14269	Scgb3a1	68662	11+:49477111-49478620
14270	Pigs	276846	11+:78141948-78156511
14271	Cox6a1	12861	5-:115795663-115798963
14272	Opn3	13603	1-:177592558-177622721
14273	Stk11	20869	10+:79578946-79593422
14274	Hadh	15107	3-:130936373-130974945
14275	MMT00070777		7+:13030699-13054267
14276	Cdc42	12540	4-:136875618-136913577
14277	Pklr	18770	3+:88940063-88949554
14278	Tpmt	22017	13+:47118979-47120361

14279	3930402G23Rik	74027	8-:10924425-10928522
14280	MMT00004656		6-:24996506-25149473
14281	5031414D18Rik	271221	14+:75431791-75452333
14282	Htr7	15566	19-:36034761-36131838
14283	Vmn2r75	546981	7-:93296553-93314958
14284	Mki67ip	67949	1+:120218453-120230164
14285	C1qbp	12261	11-:70791356-70796467
14286	Tram1	72265	1-:13554782-13580045
14287	EG241041	241041	1-:21278222-21283693
14288	Atmin	234776	8+:119467329-119484344
14289	MMT00073214		17-:56690445-56693869
14290	EG329055	329055	19-:33851911-33859360
14291	ORF19	68767	17+:66460941-66469790
14292	Htr5a	15563	5+:28168486-28177728
14293	Col9a1	12839	1+:24184546-24258839
14294	Cux1	13047	5-:136723928-136733999
14295	Cnpy2	56530	10+:127759514-127764242
14296	Slc35b1	110172	11+:95246235-95252966
14297	MMT00040516		6+:71633291-71633877
14298	Atp2a2	11938	5-:122903522-122951904
14299	V1rf1	171232	17+:21365789-21366777
14300	MMT00023851		1+:81793834-81794316
14301	Pdcd11	18572	19+:47165232-47207289
14302	Rnf213	629974	11+:119279387-119294942
14303	V1ri2	171253	13+:23167660-23168561
14304	Mrpl48	52443	7-:107697223-107699149
14305	MMT00024654		2-:149158807-149160401
14306	Gtf3c2	71752	5-:31459173-31482294
14307	Kcnj8	16523	6-:142513890-142519829
14308	Tbcd	108903	11+:121313303-121478675
14309	Cyp3a41b	100041375	5-:146319535-146325438
14310	MMT00073582		9+:63745916-63747639
14311	4933437N03Rik	66783	4+:63042391-63055050
14312	1110012J17Rik	68617	17-:66691784-66798086
14313	Tnk1	83813	11-:69665104-69670576
14314	2810405K02Rik	66469	4-:154270521-154273152
14315	Ccdc39	51938	3-:33711283-33743223
14316	MMT00012375		18-:54190446-54191079
14317	Ccdc58	381045	16+:36071587-36092204
14318	Vmn2r9	435864	5-:109271965-109281529
14319	MMT00076860		7+:150782189-150782899
14320	Paxip1	55982	5-:28068587-28118090
14321	Drd5	13492	5+:38710904-38712161
14322	Bat1a	53817	17+:35380184-35390443
14323	Scarb1	20778	5-:125757461-125821444
14324	2410022L05Rik	66423	8-:13884794-13890270
14325	1700029P11Rik	66346	15+:81810974-81811991
14326	Lztfl1	93730	9-:123608772-123626671
14327	MMT00033454		5-:67789474-67911179
14328	MMT00077647		X+:159915827-159916499
14329	MMT00046641		16+:65793994-65794401

14330	Stx11	74732	10-:12660918-12728746
14331	Dgat2l6	668257	X+:97736406-97741222
14332	Trpm6	225997	19+:18853009-18963531
14333	Zcchc17	619605	4-:129993147-130037207
14334	Fxyd3	17178	7-:31855556-31861697
14335	Olfr33	18332	7-:109861970-109862925
14336	Msh3	17686	13-:93019782-93036028
14337	Slc2a12	353169	10+:22364884-22412063
14338	Rtel1	269400	2+:181057505-181091322
14339	Olfr1477	258691	19+:13539382-13577780
14340	Tmem55a	72519	4+:14791406-14841249
14341	Ormdl3	66612	11-:98442607-98448559
14342	4930412F15Rik	242408	4-:43677419-43679483
14343	Hnrnpa0	77134	13-:58227829-58229945
14344	4921506M07Rik	70846	12+:58665108-58723099
14345	Nup37	69736	10+:87610968-87640991
14346	V1rj3	252909	7-:13669638-13670564
14347	Dact2	240025	17-:14332059-14340873
14348	Chrnb2	11444	3-:89560998-89568491
14349	1700018F24Rik	69396	5+:145803858-145806547
14350	Adamts2	216725	11+:50415981-50617553
14351	Polr2g	67710	19-:8867618-8873047
14352	MMT00048271		17-:9514957-9515571
14353	Slc35f2	72022	9+:53619546-53665927
14354	Aim1l	230806	4+:133643398-133648416
14355	Tle1	21885	4-:71779317-71861093
14356	Olfr244	404222	1-:176047222-176048178
14357	Ing4	28019	6+:124989865-124999282
14358	Wdr1	22388	5-:38918052-38952834
14359	Hist2h2bb	319189	3+:96073674-96074054
14360	Olfr1220	258902	2-:88937148-88938082
14361	Txndc12	66073	4+:108507294-108534722
14362	S100z	268686	13-:96247257-96248610
14363	Cxcl16	66102	11-:70268905-70272949
14364	Robo1	19876	16+:72204862-72742697
14365	Ctsj	26898	13-:61101638-61107272
14366	Carm1	59035	9+:21351429-21393904
14367	MMT00034267		6+:86234933-86237330
14368	9330128J19Rik	271144	13-:97189619-97199839
14369	MMT00057201		X+:132862837-132863488
14370	Ppm1e	320472	11-:87171934-87172405
14371	Proz	66901	8+:13060964-13076022
14372	Vmn2r26	56552	6+:123974775-124012052
14373	Cyp2c50	107141	19+:40164185-40188440
14374	Lect2	16841	13-:56643827-56649861
14375	D17H6S56E-5	110956	17-:35133667-35137272
14376	St8sia5	225742	18+:77424738-77494057
14377	Ggn	243897	7+:29955408-29958122
14378	5730409E04Rik	230757	4+:126286983-126289705
14379	Car12	76459	9+:66561328-66614653
14380	MMT00051866		2-:129203437-129205150

14381	Thsd7b	210417	1+:131999647-132091876
14382	MMT00053015		18+:64447981-64448486
14383	MMT00047926		17-:82591397-82668297
14384	Dnajc7	56354	11-:100444164-100480921
14385	2010001M09Rik	69816	18-:35806925-35809021
14386	Begain	380785	12-:110271194-110306377
14387	Ccdc88c	68339	12-:102207553-102267119
14388	Adam11	11488	11+:102622919-102640348
14389	Abcc2	12780	19+:43856893-43912441
14390	EG545216	545216	17+:58659787-58734264
14391	MMT00017335	100043120	19-:61332778-61333797
14392	Dcxr	67880	11-:120586714-120588594
14393	Ms4a12	381213	19-:11290536-11305038
14394	MMT00000133		7-:112152788-112153161
14395	MMT00007523		14-:91599022-91599684
14396	Hnrnpf	98758	6+:117856832-117874830
14397	Padi2	18600	4+:140462341-140508500
14398	Snn	20621	16+:11072324-11072677
14399	Csnk2a1	12995	2+:152083203-152104962
14400	Scd2	20250	19+:44368577-44381350
14401	Muc16	73732	9-:18440957-18465571
14402	Mrps27	218506	13+:100114741-100185517
14403	S100g	12309	X-:159399948-159402497
14404	Pars2	230577	4+:106323725-106327163
14405	1700015E13Rik	76925	1+:172222992-172242258
14406	Zc3h14	75553	12+:99985176-100026156
14407	Six3	20473	17+:86012946-86024533
14408	1100001H23Rik	66857	6-:136560591-136610337
14409	Olfr1378	258912	11+:50782521-50783469
14410	Irf2bp1	272359	7+:19589785-19591540
14411	MMT00013858		10-:41517069-41517472
14412	2500003M10Rik	66511	3-:90302877-90313372
14413	Kcnq1	16535	7+:150293352-150612947
14414	Twsg1	65960	17-:66272405-66300470
14415	MMT00011828		11-:82849878-82850424
14416	Serpinb11	66957	1+:109258890-109277052
14417	Grb2	14784	11-:115505459-115560622
14418	Vcan	13003	13-:89796661-89871139
14419	Slc24a3	94249	2+:144993646-145070825
14420	Cfd	11537	10+:79353656-79355399
14421	Shox2	20429	3-:66777192-66785693
14422	Ubash3a	328795	17+:31344784-31345178
14423	1300012G16Rik	71772	5-:120933908-120953606
14424	MMT00015366		17-:45300176-45327522
14425	4930579E17Rik	75847	12+:37108765-37310044
14426	Zc3h4	330474	7+:17002121-17020859
14427	Hoxa1	15394	6-:52105592-52108285
14428	Atad5	237877	11+:79946476-79947689
14429	Rasgrp2	19395	19+:6400566-6415210
14430	D130043K22Rik	210108	13+:24936981-24991666
14431	Piwil1	57749	5+:129242155-129261348

14432	MMT00059141		X+:151672067-151672543
14433	Adipor1	72674	1+:136311978-136329925
14434	Sec14l2	67815	11-:3997044-4018732
14435	Stch	110920	16-:75755479-75767035
14436	AI847670	330050	5+:20964975-20987943
14437	B230206F22Rik	78878	X-:100802880-100817844
14438	V1rc24	171197	6-:57875878-57876785
14439	Ubd	24108	17+:37330506-37332782
14440	MMT00015397		
14441	Ywhaz	22631	15-:36700525-36723272
14442	Ethe1	66071	7+:25378927-25393853
14443	C3	12266	17-:57343383-57387113
14444	Pdia4	12304	6-:47746360-47758344
14445	Arl6	56297	16-:59613153-59638879
14446	MMT00007789		8+:4777833-4778940
14447	MMT00000775	627004	7-:34352273-34391071
14448	MMT00010161		13+:22661169-22768681
14449	Il23r	209590	6-:67372925-67441849
14450	Alg5	66248	3+:54539460-54553717
14451	Speer3	71026	5+:13791631-13796820
14452	Slc5a5	114479	8-:73407400-73416623
14453	Inpp5d	16331	1+:89516953-89617076
14454	Sall3	20689	18-:81165207-81171368
14455	2700029M09Rik	72612	8+:63369207-63386377
14456	AW146242	232023	6-:57702258-57775119
14457	4921507P07Rik	70821	6-:50523304-50546589
14458	Tm7sf3	67623	6-:146550772-146582999
14459	Tollip	54473	7-:149070271-149088213
14460	EG638904	638904	6+:40736939-40807794
14461	Cts3	117066	13-:61665990-61671426
14462	Zfp385b	241494	2-:77248699-77254198
14463	MMT00051367		8+:83294589-83295567
14464	MMT00059232		12+:83644585-83645174
14465	Hrasls3	225845	19+:7632013-7660290
14466	MMT00057557		17-:22056771-22058139
14467	Crtap	56693	9-:114284255-114299788
14468	Tmtc2	278279	10-:104626034-104627073
14469	Ripply3	170765	16+:94550026-94558540
14470	Taf1a	21339	1_random+:362346-383535
14471	Pdgfrl	68797	8+:42011694-42076120
14472	Ahcy	269378	2-:154885427-154900133
14473	6330534C20Rik	434778	X+:50144385-50152668
14474	Wdr76	241627	2+:121332504-121382132
14475	Abcg1	11307	17+:31194685-31252717
14476	Akr1b8	14187	6+:34304163-34318454
14477	Stk35	67333	2+:129625234-129654216
14478	Mdh2	17448	5+:136254559-136266259
14479	Ada	11486	2-:163552550-163575897
14480	Shank2	210274	7+:151581366-151607038
14481	Csde1	229663	3+:102832743-102861048
14482	Shfm1	20422	6-:6508289-6528586

14483	Dgkb	217480	12+:39056579-39165321
14484	MMT00017000		2+:23090568-23091297
14485	Atp8b1	54670	18-:64690606-64765032
14486	Sox7	20680	14+:64562542-64569569
14487	Phf2	18676	13-:48898813-48966254
14488	Tshb	22094	3-:102581320-102586637
14489	MMT00045030		16+:22236564-22238122
14490	2510003E04Rik	72320	10-:62021220-62041817
14491	Pml	18854	9-:58066367-58097488
14492	Arhgap27	544817	11-:103192811-103193968
14493	6230409E13Rik	76132	4+:21858508-21921302
14494	Btnl2	547431	17+:34493166-34502406
14495	A630018P17Rik	245695	X-:162940250-162941329
14496	MMT00072975		
14497	Xirp2	241431	2+:67343559-67364099
14498	Layn	244864	9-:50864907-50885159
14499	2310065K24Rik	102122	8-:97098842-97125588
14500	Entpd4	67464	14+:69955241-69984744
14501	Ptk7	71461	17-:46701416-46766387
14502	MMT00033057		7-:3264574-3265223
14503	Sh2d1b2	545378	1+:172178263-172181783
14504	Prl2b1	66392	13-:27475215-27482715
14505	Slc7a12	140918	3+:14480698-14505807
14506	Slc15a3	65221	19+:10917033-10932393
14507	Ttc13	234875	8-:127195233-127212525
14508	Rpl21	19933	5+:147645771-147647527
14509	Smug1	71726	15-:102983737-102993761
14510	Olfr643	259081	7-:111207170-111208114
14511	Sostdc1	66042	12+:37040812-37045032
14512	Tubg1	103733	11+:100981444-100987731
14513	Arpc3	56378	5+:122846820-122855999
14514	Rem2	140743	14+:55095066-55099268
14515	Shmt2	108037	10-:126954372-126959442
14516	Ihh	16147	1-:74992098-74998080
14517	Clcn4-2	12727	7+:7251292-7252146
14518	A430033K04Rik	243308	5+:139064085-139089042
14519	Trex1	22040	9-:108960492-108961435
14520	Stmn1	16765	4+:134026580-134029316
14521	MMT00050820		5+:38285934-38288479
14522	MMT00019997		11-:20474675-20475163
14523	Defb3	27358	8+:19293363-19295338
14524	Rarres2	71660	6-:48519696-48522669
14525	Pitpnm1	18739	19+:4100621-4113961
14526	Pacsin3	80708	2+:91097064-91104826
14527	A230107C01Rik	320225	7-:29983358-29998696
14528	Slc4a7	218756	14+:15535519-15626571
14529	Rax	19434	18-:66094291-66098731
14530	1190017O12Rik	68936	16+:92301563-92313284
14531	Mmel1	27390	4+:154243744-154269639
14532	Fhl3	14201	4+:124377953-124385334
14533	Cutc	66388	19+:43827485-43843121

14534	Ubxd1	66530	17-:56207952-56214427
14535	Elk1	13712	X-:20512052-20527715
14536	Hltf	20585	3+:19957987-20016608
14537	EG546896	546896	6-:42488327-42510608
14538	Ier3ip1	66191	18+:77168779-77180344
14539	Rdh20	70061	10+:127335329-127348142
14540	Tmem140	68487	6+:34818514-34824009
14541	Gm806	328250	13+:50565045-50568494
14542	A730069N07Rik	244425	8-:38000613-38015496
14543	Cfl2	12632	12-:55959807-55963864
14544	Olig2	50913	16+:91226644-91227615
14545	Exoc5	105504	14-:49631818-49686344
14546	1810011H11Rik	69069	14+:33599146-33631153
14547	Otoa	246190	7+:128226951-128306603
14548	MMT00057967		X+:72699908-72704798
14549	Edf1	59022	2+:25413419-25417601
14550	Acot7	70025	4+:151552242-151645956
14551	MMT00047598		
14552	Shc2	216148	10-:79083800-79100595
14553	Bud31	231889	5+:145901243-145909085
14554	Dll4	54485	2+:119151564-119161392
14555	MMT00017897		19-:16565011-16565597
14556	Txndc14	66958	2-:84511475-84518204
14557	Pyroxd1	232491	6+:142294190-142311144
14558	Arhgef15	442801	11-:68756656-68770365
14559	Frrs1	20321	3+:116603514-116606667
14560	Slc16a11	216867	11+:70027410-70029937
14561	V1ri9	171259	13-:22310730-22311689
14562	Sf3a2	20222	10+:80261328-80267558
14563	Dna2	327762	10+:62409828-62436926
14564	Mrpl49	18120	19-:6053629-6057751
14565	Rnf207	433809	4-:151679011-151693013
14566	2410089E03Rik	73692	15+:8137270-8152292
14567	Cln6	76524	9+:62692376-62699809
14568	Gm1947	384929	X+:153857762-153868258
14569	Med22	20933	2-:26760786-26766162
14570	Erdr1	170942	
14571	MMT00079617		2-:146580250-146581487
14572	Epx	13861	11-:87677502-87689033
14573	4931406I20Rik	66743	4-:117162193-117169557
14574	Lipn	70166	19+:34141961-34159141
14575	Slc39a10	227059	1-:46863562-46892996
14576	Mcm8	66634	2+:132653672-132669431
14577	Cdc37l1	67072	19+:29064983-29092059
14578	6720475J19Rik	68157	19-:9910227-9911446
14579	2610015P09Rik	212153	16+:43898202-43963808
14580	Rnf32	56874	5+:29524545-29551830
14581	Casc5	76464	2+:118872767-118929850
14582	Mt1	17748	8+:96703210-96704092
14583	Prss22	70835	17-:24130501-24135050
14584	Vps24	66700	6+:71493892-71530212

14585	Bxdc1	67239	10-:39943302-39966808
14586	Paics	67054	5+:77380681-77396526
14587	Atcay	16467	10-:80667262-80693561
14588	EG245376	245376	X-:23534829-23536060
14589	Ccdc6	76551	10+:69559868-69652151
14590	Prl7c1	67505	13-:27866378-27868177
14591	Wasf2	242687	4+:132686491-132755669
14592	Thumpd2	72167	17-:81425670-81464410
14593	Tmem25	71687	9-:44601861-44607299
14594	MMT00024923		2-:94224134-94225281
14595	Tmem157	67698	1+:97210231-97231102
14596	Upf2	326622	2+:5877869-5977002
14597	Col19a1	12823	1-:24270832-24594250
14598	Ooep	67968	9-:78225213-78226395
14599	1700029M20Rik	73937	4+:135182732-135184400
14600	Pgf	18654	12-:86507592-86518230
14601	MMT00038159		5+:13080735-13082866
14602	Dnajb5	56323	4+:42962846-42970620
14603	Ly6e	17069	15+:74788206-74789761
14604	Setd1a	233904	7+:134924119-134926878
14605	MMT00026739		9-:18583781-18680448
14606	Il1rl1	17082	1+:40496606-40504988
14607	Dbndd1	72185	8-:126029587-126039252
14608	Chmp2a	68953	7-:13617367-13620093
14609	Cc2d1b	319965	4+:108292601-108306818
14610	Zfp60	22718	7+:28537494-28538719
14611	Ddx60	234311	8+:64479366-64516152
14612	MMT00035477		19+:11453226-11464852
14613	Olfr1097	258840	2-:86730384-86731330
14614	Gp6	243816	7-:4320315-4349320
14615	Zfhx2	239102	14-:55681442-55694710
14616	4933421E11Rik	321000	3+:106524843-106539492
14617	MMT00033859	26930	19+:55920381-55923221
14618	Ergic3	66366	2+:155833859-155844013
14619	4930403L05Rik	67395	X-:14687736-14688615
14620	Tbc1d8	54610	1-:39428343-39459755
14621	Six4	20474	12-:74200596-74214404
14622	MMT00009403	637477	3+:35831963-35836963
14623	4933416E14Rik	71180	X-:9993768-9994262
14624	Spopl	76857	2+:23364243-23365615
14625	Prss36	77613	7-:135080083-135088839
14626	Zbtb2	381990	10+:5971607-5979466
14627	Ern1	78943	11-:106258933-106349110
14628	MMT00019519		18+:35654872-35655659
14629	Nfkb2	18034	19+:46380107-46386578
14630	Ank2	109676	3-:126625997-126807787
14631	Stag3	50878	5+:138721778-138753620
14632	2510049J12Rik	70291	6-:115535135-115542593
14633	H2afz	51788	3+:137527562-137529884
14634	MMT00022705		4-:67963494-67964324
14635	MMT00053491		10+:27445607-27590264

14636	Parg	26430	14+:33015405-33110403
14637	Gnb2	14693	5-:137969732-137971979
14638	Srgap2	14270	1-:133185935-133422171
14639	Olfr691	259063	7-:112485260-112486228
14640	Tmem97	69071	11-:78355319-78364237
14641	MMT00000142		
14642	Mtap1s	270058	8+:73429889-73441427
14643	Ets2	23872	16+:95924113-95940752
14644	Herc2	15204	7+:63376906-63487166
14645	Mbd3l2	234988	9+:18247989-18249899
14646	V1ri6	171257	13+:22370230-22371179
14647	Cst3	13010	2-:148697479-148701137
14648	Cdkn1b	12576	6+:134870720-134874325
14649	5430405G05Rik	108832	2+:151527646-151533040
14650	2810433K01Rik	66468	18-:74356419-74367433
14651	Gapdhs	14447	7-:31514907-31524375
14652	Tceb2	67673	17-:23961706-23966076
14653	Napsa	16541	7+:51827814-51842216
14654	Tll2	24087	19-:41157242-41281325
14655	Tlk2	24086	11+:105040120-105143490
14656	Grip2	243547	6-:91713442-91738438
14657	Mefv	54483	16-:3707221-3718097
14658	Tex19.2	70956	11-:120977530-120979991
14659	MMT00043452		
14660	Gnai3	14679	3-:107910198-107949063
14661	MMT00033581		14+:17779242-17786712
14662	MMT00070823		7+:29319790-29320109
14663	1700029F12Rik	66479	13-:97791824-97800377
14664	A430093A21Rik	227195	1-:63004992-63104775
14665	Ifna5	15968	4+:88481360-88488324
14666	MMT00009515		1-:66015695-66135723
14667	Fggy	75578	4+:95224198-95467357
14668	Lrrc61	243371	6+:48504793-48519022
14669	Pftk2	271697	1+:59314659-59409292
14670	MMT00048137		4+:143261125-143261585
14671	Ccdc105	70976	10-:78209665-78215814
14672	EG627009	627009	4-:143962662-143967094
14673	4933407C03Rik	74440	8+:119873067-119983616
14674	Ppap2c	50784	10-:78990070-78996513
14675	Tlx3	27140	11-:33101279-33103459
14676	Mecr	26922	4+:131399385-131423682
14677	S100b	20203	10+:75719779-75722812
14678	Nsg1	18196	5-:38529910-38550244
14679	Prmt6	99890	3-:110052753-110054057
14680	MMT00005929		6-:69233745-69255130
14681	Alg12	223774	15-:88635675-88649710
14682	MMT00026181		6+:76071752-76072372
14683	Dennd1c	70785	17-:57204742-57217938
14684	Rassf3	192678	10-:120849618-120913306
14685	Foxd1	15229	13+:99124209-99126647
14686	Zdhhc5	228136	2-:84529108-84550781

14687	3300005D01Rik	78512	17+:5798652-5803239
14688	5730590G19Rik	77011	7+:86839357-86841649
14689	AB124611	382062	9+:21333206-21349219
14690	Atf4	11911	15+:80085930-80087934
14691	Nckap1	50884	2-:80340681-80421133
14692	C5ar1	12273	7-:16833386-16834440
14693	Nat12	70646	14+:49808299-49810548
14694	Ddx17	67040	15-:79357616-79376986
14695	1700025E21Rik	75647	6+:30462303-30469963
14696	Pou4f2	18997	8-:80958635-80959604
14697	Mtrf1	211253	14+:79797662-79823395
14698	Dnajb9	27362	12-:45306882-45311027
14699	V1ri3	171254	13+:22446214-22447116
14700	Tbc1d17	233204	7-:52096402-52104449
14701	MMT00068860		14-:83255634-83256154
14702	Krt71	56735	15-:101564380-101573528
14703	MMT00009557		15-:14551460-14551778
14704	MMT00046147		2+:69604238-69604849
14705	Slc43a1	72401	2+:84679551-84703750
14706	MMT00014463		4+:109302638-109303400
14707	Klk14	317653	7+:50947324-50982101
14708	Gm71	207965	12-:70678645-70684092
14709	Rab36	76877	10+:74499827-74515671
14710	Gipc1	67903	8+:86184891-86188166
14711	1700001F09Rik	71826	14-:44020916-44026344
14712	V1rh8	171251	13-:22683848-22684799
14713	Nme7	171567	1+:166238203-166367425
14714	Ccdc102a	234582	8-:97426769-97441331
14715	Poldip2	67811	11+:78325813-78335410
14716	MMT00052972		5-:107907545-107920096
14717	Srpk3	56504	X+:71019760-71024261
14718	Meox2	17286	12+:37835141-37906115
14719	Tbc1d2b	67016	9-:90117144-90165280
14720	Lcn9	77704	2+:25678668-25681057
14721	4930517E11Rik	74702	2-:123949371-123955692
14722	Scrib	105782	15-:75877615-75900160
14723	Cd14	12475	18-:36884722-36886177
14724	Gm467	232585	6-:13124192-13137260
14725	MMT00013262		2+:87366204-87366895
14726	Slc19a1	20509	10+:76496003-76513171
14727	Rab40c	224624	17-:26019059-26056647
14728	Fadd	14082	7-:151765681-151768296
14729	A530057A03Rik	215456	2+:127250882-127256690
14730	1700008O03Rik	69349	7-:51615207-51630401
14731	BC006965	217294	11-:112548958-112572655
14732	Mga	29808	2+:119728408-119790700
14733	Fhit	14198	14-:10382613-11994549
14734	Nrsn2	228777	2-:152194491-152202346
14735	1700008F21Rik	75453	8+:131591033-131707631
14736	Pigr	18703	1+:132723356-132748150
14737	Cldn12	64945	5-:5505112-5514915

14738	Ccl17	20295	8+:97334373-97335935
14739	V1ra7	113849	6+:90128709-90129638
14740	Mfap1b	100034361	2-:121286459-121299760
14741	Cep135	381644	5+:77017741-77024559
14742	RP23-292J1.1	545637	4-:73518124-73521297
14743	D11Wsu47e	276852	11+:113545750-113555960
14744	Cabp4	73660	19-:4135809-4139604
14745	MMT00000323		8+:106595048-106595632
14746	MMT00013045		14+:15862394-15862536
14747	Igf2bp1	140486	11-:95824453-95867077
14748	Crhr1	12921	11+:103994355-104036836
14749	Mnt	17428	11+:74644482-74656988
14750	Bves	23828	10+:45055577-45089512
14751	MMT00011983		7+:50408538-50519624
14752	MMT00052718	100041004	5-:143285576-143289203
14753	Slc25a19	67283	11-:115476296-115489453
14754	Atoh8	71093	6-:72156172-72185290
14755	Olfr452	258207	6+:42740039-42740992
14756	AI595406	237730	11-:35610769-35612387
14757	Hdx	245596	X+:108685773-108687484
14758	Rims4	241770	2-:163689183-163744419
14759	Olfr470	258417	7-:114988396-114989245
14760	Mylc2pl	59310	5+:137169015-137176964
14761	Kank2	235041	9-:21572520-21602947
14762	Ndufa1	54405	X-:34727595-34731158
14763	1110005A23Rik	66118	10+:128258974-128314673
14764	Ugt2b34	100727	5-:87318777-87335983
14765	Flad1	319945	3-:89207315-89215575
14766	Glcci1	170772	6-:8546256-8573973
14767	Dpy19l3	233115	7-:36501524-36507849
14768	1110020G09Rik	68646	15+:9001008-9040234
14769	Thoc1	225160	18+:9957586-9994627
14770	Cops7a	26894	6-:124909816-124912716
14771	Pus10	74467	11+:23565696-23631525
14772	MMT00077971		6+:117947159-117948101
14773	Anxa13	69787	15-:58173022-58220799
14774	Tbc1d22b	381085	17+:29686745-29741917
14775	Rpl24	68193	16+:55966659-55971504
14776	4930542D17Rik	75211	16+:50590614-50594005
14777	Psmb11	73902	14+:55244154-55245070
14778	Anln	68743	9-:22136459-22193615
14779	Dsg3	13512	18+:20668781-20699833
14780	D230014K01Rik	217364	11+:118338299-118349248
14781	Mars2	212679	1+:55294046-55295959
14782	Gmppa	69080	1+:75432558-75439748
14783	Tceb1	67923	1-:16632848-16646965
14784	MMT00024114		9+:79851871-79853061
14785	F3	14066	3+:121426598-121437502
14786	BC033932	434325	8-:74079656-74082824
14787	Gca	227960	2+:62502400-62531554
14788	Zfp184	193452	13+:22037227-22052472

14789	Cox8a	12868	19-:7289651-7292019
14790	MMT00009373		3+:7454626-7455521
14791	MMT00048381		2-:147106437-147106820
14792	Rarg	19411	15-:102066291-102087753
14793	1700003M02Rik	69329	4-:34635823-34663664
14794	Sprr2f	20760	3+:92169796-92170364
14795	Adamts3	330119	5-:90106691-90290730
14796	Ubr4	69116	4+:138953208-138957383
14797	Ecsit	26940	9-:21876689-21889826
14798	Reep2	225362	18+:35000311-35007108
14799	1700021K02Rik	65971	10+:60638009-60643427
14800	MMT00016856		10-:88133238-88134014
14801	MMT00053527		10+:29501350-29501617
14802	Fbxo16	50759	14+:65885537-65940338
14803	Ins1	16333	19+:52339113-52339438
14804	Naalad2	72560	9-:18127706-18151934
14805	St3gal6	54613	16-:58470654-58523425
14806	Ccdc45	320162	11+:106650567-106680173
14807	Sdk1	330222	5+:142661110-142688425
14808	Pitx3	18742	19-:46210175-46222815
14809	MMT00064476		5+:111334261-111334760
14810	1700010H22Rik	75500	5-:98982647-98995943
14811	Slc12a7	20499	13+:73901144-73954186
14812	Ppib	19035	9+:65908050-65914428
14813	1700113I22Rik	73635	11-:101283178-101283270
14814	MMT00013754		4+:86739237-86749704
14815	Lpin3	64899	2+:160706468-160731528
14816	Pcdhb9	93880	18+:37560713-37563093
14817	Tgfa	21802	6+:86145409-86225150
14818	Il17ra	16172	6+:120413251-120433051
14819	Rps6ka3	110651	X+:155693850-155802983
14820	Olfr1344	257882	7+:6367878-6382275
14821	Olfr1206	258896	2+:88704763-88705679
14822	Nrsn1	22360	13-:25343908-25361818
14823	Zfp583	213011	7-:6267877-6282910
14824	14-Sep	74211	9-:57100923-57110354
14825	Ube2h	22214	6-:30163017-30254274
14826	Fbxo5	67141	10+:4536590-4546998
14827	Pbx2	18515	17+:34729415-34734286
14828	Rab28	100972	5-:42016213-42099396
14829	1110007M04Rik	68493	4+:24825275-24829362
14830	Olfr421	258715	1+:176081648-176082594
14831	Olfr806	258546	10-:129175030-129175971
14832	Acaca	107476	11+:84160204-84215160
14833	Rnf40	233900	7+:134732297-134747124
14834	Upf3b	68134	X-:34631671-34650299
14835	Orc5l	26429	5-:21992307-22056136
14836	Hsd17b3	15487	13-:64159581-64190494
14837	MMT00049854	100043416	12-:22013714-22013834
14838	MMT00070887		2-:41841492-41844385
14839	Pdpk1	18607	17-:24215851-24278519

14840	Lonp2	66887	8+:89148028-89240531
14841	2010311D03Rik	109129	2-:50135402-50152197
14842	Gipr	381853	7-:19742279-19750373
14843	MMT00081492		14+:101743797-101744459
14844	Gpsn2	106529	8-:86095598-86118364
14845	Tmem188	382030	8+:90642675-90654298
14846	MMT00053748		9+:96437743-96438277
14847	MMT00003977		17-:54051087-54088615
14848	Pcsk1n	30052	X+:7497056-7500228
14849	MMT00001925		9-:10285082-10290948
14850	MMT00057793		19-:46748724-46749775
14851	MMT00034182		7+:80518417-80518637
14852	Otx1	18423	11-:21895568-21901191
14853	MMT00027428		9-:47808561-47942720
14854	MMT00017294		19+:61243042-61244660
14855	Xylt1	233781	7+:124692097-124811132
14856	Eif1b	68969	9+:120401758-120404445
14857	Kctd12	239217	14-:103379782-103381851
14858	Itpr3	16440	17+:27252928-27259167
14859	Hspa1a	193740	17-:35106944-35108870
14860	MMT00051231	100041002	2-:29700512-29701882
14861	Sema6a	20358	18-:47408540-47527926
14862	Dhrs7	66375	12-:73751342-73765766
14863	Prm3	19120	16-:10790677-10790982
14864	Cdk4	12567	10+:126501291-126504076
14865	Dpysl4	26757	7+:146271960-146286991
14866	Kctd2	70382	11+:115283286-115292592
14867	MMT00040108		17-:3130154-3130512
14868	Tmem159	233806	7+:127245939-127264500
14869	S100a8	20201	3+:90473006-90473956
14870	Capn2	12334	1-:184397390-184447655
14871	F630043A04Rik	219114	14-:58426812-58445213
14872	1810030O07Rik	69155	X-:12234986-12250699
14873	Npm1	18148	11-:33052510-33063056
14874	MMT00016788		12-:15871951-15872550
14875	Cenpf	108000	1-:191464971-191511968
14876	C330002I19Rik	77480	12+:25659792-25744405
14877	Ptx3	19288	3+:66023884-66029726
14878	MMT00036534		1-:22019412-22020696
14879	EG636544	636544	7-:88367247-88367760
14880	Ms4a4c	64380	19+:11482190-11501267
14881	Nkain4	58237	2-:180669517-180689361
14882	Kcnk6	52150	7-:30010058-30017508
14883	Itgal	16408	7+:134439906-134478650
14884	Mrps22	64655	9-:98489157-98502078
14885	2010111I01Rik	72061	13+:63341359-63341640
14886	Olfr411	258704	11-:74160151-74161084
14887	Gga3	260302	11-:115445567-115466186
14888	Abca8b	27404	11-:109795579-109857163
14889	Bpil1	66557	2+:153700808-153721006
14890	Naprt1	223646	15-:75721386-75725203

14891	MMT00078932		11-:104540610-104541570
14892	Armc3	70882	2+:19120926-19231858
14893	Wdr36	225348	18+:33012584-33025684
14894	G0s2	14373	1-:195098355-195099286
14895	Wdr85	67228	2+:24817964-24828991
14896	Fev	260298	1-:74928920-74931784
14897	Rgs7	24012	1-:176989347-177422521
14898	Orm1	18405	4+:63005599-63009196
14899	1700013B16Rik	72219	13-:59802934-59806456
14900	Hist1h1e	50709	13-:23713644-23714429
14901	MMT00053127		11-:35136708-35137044
14902	MMT00006351		16+:45936429-45939611
14903	1700012A03Rik	76382	6+:32000287-32008915
14904	Ripk4	72388	16-:97964691-97985316
14905	Slamf6	30925	1+:173847667-173873998
14906	Ugt1a10	394430	1+:89952056-89960769
14907	Sox12	20667	2+:152220638-152222104
14908	Psma2	19166	13+:14705533-14717854
14909	Jazf1	231986	6-:52995950-53031285
14910	Dlg5	71228	14-:24953178-25064672
14911	4930544G11Rik	67653	6+:65902602-65904008
14912	Gpr1	241070	1-:63229265-63260783
14913	Yipf4	67864	17+:74888855-74899313
14914	Rnf111	93836	9-:70273236-70351532
14915	MMT00001646		X-:10442126-10442855
14916	Adprhl2	100206	4-:125993288-125998948
14917	Gjb5	14622	4-:127032440-127035365
14918	Clp1	98985	2-:84563280-84567425
14919	Pex13	72129	11-:23548509-23565868
14920	Folr1	14275	7-:109006845-109012659
14921	Fbxl4	269514	4+:22284762-22361210
14922	C130092O11Rik	232035	6+:61520661-61668381
14923	MMT00071473		9+:76944860-76947307
14924	Hn1l	52009	17-:25079100-25097586
14925	Tagln2	21346	1+:174430178-174437506
14926	Olfr981	258283	9+:39829979-39830911
14927	Tcl1b1	27379	12+:106397916-106404833
14928	Eme2	193838	17-:25029092-25032086
14929	2810432D09Rik	69961	4-:41659066-41660535
14930	C4bp	12269	1-:132532505-132558209
14931	Jhdm1d	338523	6-:39091973-39157554
14932	Tmtc4	70551	14-:123318199-123382439
14933	MMT00053967		8+:22818344-22819193
14934	Dcx	13193	X-:140298045-140367637
14935	Ppfia1	233977	7-:151662663-151738438
14936	Camk4	12326	18+:33099011-33346580
14937	Acot9	56360	X+:151697138-151732050
14938	Sfrs10	20462	16-:22245879-22265975
14939	Adam29	244486	8-:58349924-58352214
14940	Apba1	319924	19+:24018488-24020911
14941	MMT00037660		7-:46529621-46531829

14942	Grk1	24013	8+:13405100-13416578
14943	Pnmal2	434128	7+:17531028-17532654
14944	Rock2	19878	12+:16901995-16984908
14945	Mmp3	17392	9+:7445821-7455972
14946	Txndc5	105245	13-:38592145-38620320
14947	Ern2	26918	7-:129313468-129329689
14948	Gbgt1	227671	2+:28353285-28360913
14949	Bcl7c	12055	7-:134848493-134852111
14950	Yeats4	64050	10-:116652199-116661529
14951	Dpep3	71854	8-:108497419-108503319
14952	Tex2	21763	11-:106363451-106441824
14953	Actc1	11464	2-:113873024-113878547
14954	Rab19	19331	6+:39330849-39340376
14955	Dpp7	83768	2-:25207940-25211847
14956	Tsga10	211484	1-:37817672-37912218
14957	Lgr5	14160	10-:114888695-115024549
14958	Ppp4r2	232314	6+:100783626-100819921
14959	Cacna2d1	12293	5+:15440605-15876867
14960	Olfr843	258560	9-:19052901-19053841
14961	Dstn	56431	2+:143741346-143769059
14962	Casd1	213819	6+:4579999-4593018
14963	4930568D16Rik	75859	2-:35209737-35223250
14964	MMT00027165	257663	2+:90240819-90241749
14965	Csnk2b	13001	17-:35253140-35258392
14966	Ddx51	69663	5+:111085348-111087216
14967	Ndufa10	67273	1-:94336297-94370335
14968	Srbd1	78586	17-:86384003-86529620
14969	EG245506	245506	X+:85975211-85977435
14970	V1rg3	171238	7+:12778313-12779239
14971	Wnt7b	22422	15-:85367419-85411880
14972	Syt3	20981	7+:51641340-51654520
14973	Shf	435684	2-:122174668-122194493
14974	Serpinb9f	20709	13+:33415924-33424148
14975	Nsbp1	50887	X-:106201382-106208630
14976	Slc26a7	208890	4-:14432343-14548904
14977	MMT00048238		1+:88370233-88384685
14978	Ash2l	23808	8-:26927347-26951241
14979	Ccnf	12449	17-:24360487-24388290
14980	Gfra1	14585	19-:58312651-58529849
14981	0610012H03Rik	74088	2+:105064500-105219948
14982	Dlg1	13383	16+:31664929-31872206
14983	MMT00069757		
14984	MMT00037514		10+:129557418-129558263
14985	Dspp	666279	5+:104599719-104609118
14986	4632412N22Rik	320159	17+:72034143-72038781
14987	MMT00027573		1-:161139261-161141196
14988	Pdzrn4	239618	15+:92227891-92232477
14989	Map3k5	26408	10+:19654288-19861527
14990	Serpina3b	271047	12+:105368671-105376986
14991	Gapdh	14433	6-:125112527-125115600
14992	Hist1h3b	319150	13+:23844248-23844657

14993	MMT00015245		
14994	1110059E24Rik	66206	19-:21671802-21727281
14995	MMT00042618		X-:87989990-88001685
14996	MMT00005425		7-:13676875-13679095
14997	Pi4k2a	84095	19+:42175119-42187606
14998	MMT00047701	241621	2+:116858774-116881746
14999	MMT00068456		5-:108574243-108575926
15000	Tbx18	76365	9-:87599499-87626020
15001	Tm9sf2	68059	14+:122506364-122558555
15002	Ncapg	54392	5+:46061159-46091364
15003	Zcchc10	67966	11+:53138180-53146822
15004	MMT00067307	433111	17+:45871454-45886306
15005	Loxl1	16949	9-:58136405-58148560
15006	MMT00038974		15-:68545365-68546190
15007	MMT00030946		6-:91270533-91271029
15008	Chrdl2	69121	7+:107155009-107183238
15009	Phf5a	68479	15-:81695748-81702337
15010	Kcnk4	16528	19-:7000180-7009005
15011	Olfr657	258309	7+:111784189-111785141
15012	MMT00030248		8-:86001064-86003137
15013	Nfkb1	18033	3-:135247657-135354295
15014	Mrp63	67840	14+:58445075-58447582
15015	G3bp1	27041	11+:55283253-55314387
15016	MMT00074608		8+:41940765-41941725
15017	Galnt13	271786	2+:54288900-54369092
15018	D10Ertd322e	67270	10-:94943439-94964561
15019	Slc39a5	72002	10-:127832989-127837573
15020	5830435N17Rik	76066	11-:96451685-96481017
15021	Ppargc1a	19017	5-:51848896-51945100
15022	Tmem14c	66154	13+:41111660-41117947
15023	Mcoln2	68279	3+:145812806-145858472
15024	2310061J03Rik	66391	16-:55973389-55974663
15025	Flii	14248	11-:60527650-60540723
15026	MMT00070733		7+:12556464-12557444
15027	Brd7	26992	8-:90856210-90886052
15028	MMT00009785		8+:102188070-102188756
15029	Nudt16l1	66911	16+:4939110-4941023
15030	Pdhb	68263	14-:8996872-9005548
15031	4930415O20Rik	73863	15+:98415681-98420011
15032	2310033P09Rik	67862	11+:59021869-59024234
15033	Olfr126	258892	17+:37987538-37988498
15034	Pdhx	27402	2-:102860919-102913667
15035	EG333669	333669	10+:75448664-75467655
15036	2410004B18Rik	66421	3+:145600995-145607237
15037	Olfr690	56860	7-:112477757-112478720
15038	Centb5	140500	4+:155266419-155281359
15039	Zmynd15	574428	11+:70274407-70279701
15040	B3galt2	26878	1+:145493257-145494526
15041	Mark1	226778	1-:186721481-186823358
15042	MMT00065766		7+:27523605-27524111
15043	MMT00014281	636687	8-:72070056-72109061

15044	4930483J18Rik	67638	15+:81021282-81023082
15045	Vmn2r105	627743	17-:20345193-20365002
15046	Arl6ip2	56298	17-:80249516-80252264
15047	MMT00028841		6+:67811145-67811719
15048	2310001H12Rik	69504	5+:110425559-110425653
15049	Rnf125	67664	18+:21119968-21142500
15050	MMT00003733		14+:77126081-77376503
15051	Nin	18080	12-:71119741-71212945
15052	Kifc3	16582	8-:97623733-97634324
15053	Tns4	217169	11-:98928682-98950614
15054	Dci	13177	17+:24563647-24576147
15055	Vgll4	232334	6-:114809371-114919976
15056	D930020B18Rik	216393	10+:121104461-121127004
15057	Atad2	70472	15-:57925597-57966638
15058	Foxp2	114142	6+:14851613-14899118
15059	Mogat1	68393	1+:78508465-78534738
15060	Ttc14	67120	3+:33707638-33709059
15061	MMT00030104		X+:111995166-111995268
15062	P2ry5	67168	14+:73637698-73639440
15063	MMT00059184		7-:32488798-32490780
15064	MMT00069658		16-:51362282-51364011
15065	Wdr17	244484	8-:55772693-55809701
15066	Tmcc1	330401	6-:115971187-115993823
15067	Tmbim4	68212	10+:119645881-119661953
15068	MMT00046711		9+:30901774-30902582
15069	MMT00014274		4-:84822378-84889932
15070	MMT00037369		16+:3463904-3466526
15071	Htr2b	15559	1-:87995846-88007492
15072	Commd9	76501	2+:101726418-101741796
15073	Lrrc45	217366	11+:120575273-120582812
15074	Usp18	24110	6+:121195974-121220934
15075	Ercc5	22592	1+:44204691-44238105
15076	Stil	20460	4+:114672804-114715500
15077	Spaca5	278203	X+:20645613-20654811
15078	Slc1a3	20512	15-:8585807-8660743
15079	Plcd3	72469	11-:102931617-102962920
15080	MMT00054557		3+:4211712-4213844
15081	Asah1	11886	8-:42426018-42459981
15082	Dsg1b	225256	18+:20381711-20630099
15083	Phb	18673	11+:95529369-95541163
15084	Ccl3	20302	11-:83461346-83462859
15085	MMT00070062		1-:82816242-82819425
15086	EG435977	435977	7-:54988387-54989356
15087	Slc25a17	20524	15-:81149525-81191181
15088	2810452K22Rik	67236	12-:112111374-112127309
15089	Pla2g7	27226	17+:43705660-43748996
15090	Psg18	26438	7-:18931193-18940224
15091	Gm1418	384514	6-:69494270-69494886
15092	Rhbdd2	215160	5-:136113004-136113366
15093	Kcnh8	211468	17+:52865087-53044544
15094	Pbx4	80720	8+:72356602-72396190

15095	0610005C13Rik	71661	7-:52823157-52830696
15096	Snx15	69024	19-:6119394-6124192
15097	Arl13b	68146	16-:62812971-62846868
15098	MMT00024304		15+:38821688-38823208
15099	Arhgap10	78514	8-:79774817-80041684
15100	Ganab	14376	19+:8972600-8991151
15101	Ythdf1	228994	2-:180639083-180655047
15102	Lrrc16b	268747	14+:56110072-56113258
15103	Olfr473	258771	7+:115077035-115077968
15104	MMT00059576		5+:26584224-26589882
15105	Crk	12928	11+:75492824-75518881
15106	Plekha7	233765	7-:123319223-123341912
15107	Cxcl14	57266	13-:56390004-56397912
15108	Fuca1	71665	4+:135476679-135495193
15109	Zfp189	230162	4+:49534096-49544409
15110	MMT00073562		
15111	Kctd13	233877	7+:134072385-134089123
15112	Uba6	231380	5-:86538311-86601781
15113	MMT00069471		9-:119802185-119803965
15114	V1re3	171226	17-:20913218-20914218
15115	9030420J04Rik	71544	9-:9115689-9155364
15116	MMT00034189		7+:79445302-79819163
15117	Rps6kc1	320119	1-:192596758-192735629
15118	Chchd8	68185	7+:107682991-107687858
15119	EG545921	545921	7-:16065517-16067128
15120	Pgap1	241062	1-:54568237-54614465
15121	Itln1	16429	1-:173448372-173461885
15122	MMT00063982		X-:57829621-57831971
15123	Nrtn	18188	17-:56890747-56896953
15124	Ncaph2	52683	15+:89186210-89202010
15125	Oxtr	18430	6-:112425758-112439935
15126	Megf8	269878	7+:26111398-26150937
15127	Cct6b	12467	11-:82532751-82577808
15128	St6gal2	240119	17+:55621264-55649644
15129	MMT00030044		2+:82783788-82880349
15130	Hnf1b	21410	11+:83664550-83718846
15131	Atf6	226641	1-:172637344-172797903
15132	MMT00007672		7-:110975180-110976389
15133	3110048E14Rik	73225	15+:84867633-84893258
15134	Nol9	74035	4+:151413444-151435603
15135	Col5a3	53867	9-:20574493-20619478
15136	Mkl2	239719	16+:13265169-13413348
15137	Olfr1403	258645	1+:175200307-175201240
15138	Rpl6	19988	5+:121656749-121658975
15139	Dpp8	74388	9+:64880527-64932307
15140	Slc47a1	67473	11-:61156903-61191566
15141	MMT00074858		2+:153870210-153871525
15142	Cnot4	53621	6-:34995982-35083704
15143	Gp9	54368	6+:87728129-87729756
15144	Kank4	242553	4-:98428093-98484229
15145	Olfr286	629524	15-:98057107-98058072

15146	Smc5	226026	19-:23283318-23293020
15147	Pabpc1	18458	15-:36525775-36538515
15148	Tmem87a	211499	2-:120181051-120229806
15149	Atp5d	66043	10+:79605109-79608556
15150	MMT00014640		3+:90562586-90565233
15151	Cdh11	12552	8-:105157384-105244409
15152	F730047E07Rik	212377	4+:24423642-24430557
15153	Foxm1	14235	6+:128313108-128324304
15154	Dhx29	218629	13+:113717662-113759639
15155	Rnasen	14000	15+:12819369-12820308
15156	Trim66	330627	7-:116598515-116629664
15157	Eno1	13806	4+:149611363-149622981
15158	Clk3	102414	9-:57598425-57612926
15159	Timd4	276891	11+:46652762-46657820
15160	Vezf1	22344	11+:87881863-87896043
15161	Ppp4r1	70351	17+:66169484-66191186
15162	Il18	16173	9+:50373472-50389942
15163	Gm347	241289	2+:28305888-28309370
15164	MMT00030321		
15165	Tmem129	68366	5-:33995867-34000546
15166	EG383080	383080	15-:81329513-81399475
15167	MMT00007970		10+:74283466-74283958
15168	Oxa1l	69089	14+:54980523-54989353
15169	Scp2	20280	4-:107716445-107791112
15170	Tspan17	74257	13+:54890821-54898129
15171	Nr4a3	18124	4+:48064119-48096223
15172	Cul1	26965	6+:47434858-47475631
15173	Tmem132b	208151	5+:126102830-126179120
15174	3110001D03Rik	66928	4-:74923261-74924191
15175	Enthd1	383075	15-:80364938-80395589
15176	MMT00051529		1-:46953374-47071736
15177	Bmpr2	12168	1+:59821480-59927703
15178	Elavl4	15572	4-:109878771-110024514
15179	Ralb	64143	1-:121368283-121380184
15180	Asgr1	11889	11+:69867916-69871388
15181	Enpp4	224794	17-:44233177-44242756
15182	Gpr112	236798	X+:54210449-54233531
15183	Slain2	75991	5+:73305612-73366085
15184	Capn6	12338	X-:140238172-140261838
15185	Zfp119	104349	17-:56004157-56018218
15186	D10Bwg1364e	216169	10-:80046399-80053049
15187	Nradd	67169	9-:110523640-110526957
15188	MMT00073131		9-:62189309-62192197
15189	BC005764	216152	10-:79327527-79337380
15190	MMT00046191		8+:61698114-61699740
15191	Ankrd13b	268445	11-:77283998-77303054
15192	Skap1	78473	11+:96325945-96442824
15193	RP23-9A5.1	623270	2-:110974270-110993137
15194	MMT00078831		7+:132218918-132219345
15195	Slc25a1	13358	16-:17925310-17928291
15196	BC053393	407814	11+:46390596-46402732

15197	Hmx3	15373	7+:138686696-138688439
15198	Pstk	214580	7+:138514652-138517852
15199	MMT00001490		7-:114131830-114133189
15200	Gk2	14626	5-:97884179-97886027
15201	MMT00038384		11+:14949178-14950192
15202	5330437I02Rik	319888	18+:65857922-65882277
15203	Prss3	22073	6-:41323812-41327596
15204	Doc2a	13446	7+:133991447-134005392
15205	MMT00030262		2+:14642750-14644166
15206	Wbscr18	66114	5+:135540071-135541235
15207	Cnp	12799	11+:100436288-100443039
15208	Tmem70	70397	1+:16655298-16668354
15209	Ppig	228005	2+:69560874-69589727
15210	Osr1	23967	12+:9585897-9587694
15211	Casc4	319996	2+:121692991-121761762
15212	Oaz3	53814	3-:94237311-94240538
15213	Allc	94041	12-:29238624-29267348
15214	D430042O09Rik	233865	7+:132896486-132914217
15215	Nkx1-2	20231	7-:139788354-139791298
15216	Ttc33	67515	15+:5135717-5168328
15217	Tgfbr2	21813	9-:115999091-116084310
15218	V1rc27	171200	6+:57354457-57355383
15219	0910001L09Rik	66096	5+:138696959-138700624
15220	Ddx6	13209	9+:44415217-44445189
15221	2810004N23Rik	66523	8-:127363256-127386891
15222	L3mbtl4	320858	17+:68805032-68979698
15223	MMT00004769		5+:21104556-21105366
15224	Cyp2a4	13086	7+:26685881-27100106
15225	Zrsr2	22184	X-:160373374-160396521
15226	Musk	18198	4+:58298983-58390288
15227	Ifngr1	15979	10+:19311791-19330035
15228	5730588L14Rik	329369	2+:26499141-26501462
15229	MMT00008641		12+:101703793-101726600
15230	Tgm3	21818	2+:129838084-129874297
15231	Fem1a	14154	17+:56396331-56398295
15232	Man1b1	227619	2+:25188301-25207730
15233	Camta2	216874	11-:70477275-70501433
15234	Gimap4	107526	6+:48634579-48642056
15235	Ankrd13d	68423	19-:4270178-4283104
15236	Slc2a3	20527	6-:122679949-122692408
15237	Sstr1	20605	12+:59313579-59314754
15238	Nrg1	211323	8-:33068983-33119720
15239	Ube2q2	109161	9+:54997211-55055335
15240	Tanc2	77097	11+:105620235-105786418
15241	EG636752	636752	6-:69349797-69367189
15242	Kcnk3	16527	5+:30890458-30925854
15243	Tbc1d21	74286	9-:58207520-58218262
15244	6430550H21Rik	245386	X-:35552160-35605635
15245	Olfr27	258826	9+:38951686-38952602
15246	Ccdc5	225745	18-:77996306-78006519
15247	Cstb	13014	10+:77888536-77890183

15248	Egr4	13656	6-:85461115-85463536
15249	Serpina4-ps1	321018	12+:105318854-105325131
15250	Hoxd3	15434	2+:74581984-74585663
15251	Olfr60	18361	7-:147530952-147531886
15252	MMT00019402		11+:50920177-50920984
15253	Tmem33	67878	5+:67651915-67677763
15254	2600009E05Rik	77006	2-:130479145-130490732
15255	Ccl8	20307	11+:81928684-81930297
15256	Jph2	59091	2-:163163509-163223686
15257	Zbtb32	58206	7-:31374700-31376922
15258	Coil	12812	11+:88835263-88852925
15259	Grin2d	14814	7-:53088150-53121937
15260	MMT00021250		11+:53972427-53973557
15261	Vmn2r109	627814	17-:20677482-20692206
15262	Timm8b	30057	9+:50412014-50413423
15263	Cyc1	66445	15+:76173952-76176364
15264	Flywch2	76917	17-:23913884-23923044
15265	Prph2	19133	17+:47047433-47061875
15266	Cnnm4	94220	1+:36528829-36563794
15267	Mdga2	320772	12-:67572175-67790514
15268	Syne1	64009	10+:5170624-5177854
15269	2310050C09Rik	66533	3-:92672281-92673205
15270	Serac1	321007	17-:6044063-6079728
15271	Dbf4	27214	5-:8397011-8422606
15272	MMT00057343		X-:79646064-79646380
15273	Relb	19698	7-:20191572-20214625
15274	Ccdc100	225523	18-:53841377-53879019
15275	Enox1	239188	14+:77907943-78121567
15276	Eif4e	13684	3+:138190241-138220156
15277	2810002N01Rik	68020	12+:112951481-112972402
15278	Epha5	13839	5-:84486816-84845932
15279	Clps	109791	17-:28695158-28697659
15280	Cxcl10	15945	5-:92775666-92777880
15281	V1rc22	171195	6-:58384931-58385839
15282	Olfr1505	258151	19+:13993625-13994461
15283	Snx3	54198	10+:42222061-42254774
15284	EG632778	632778	7-:26399640-26400910
15285	Vps52	224705	17-:34117258-34117692
15286	MMT00073158		4-:69615112-69616312
15287	St6galnac5	26938	3-:152484007-152645174
15288	Pet112l	229487	3+:85378048-85458085
15289	9130017N09Rik	78906	10+:79283765-79293195
15290	Ankzf1	52231	1+:75188766-75195955
15291	Prss36	77613	7-:135076145-135079593
15292	2010107G23Rik	69894	10-:61570959-61573761
15293	MMT00001423		16+:50111185-50112217
15294	Lrrc3	237387	10-:77363525-77365254
15295	Agbl5	231093	5+:31192538-31209337
15296	Mier3	218613	13+:112490993-112505883
15297	MMT00048291		7+:76354769-76355794
15298	1190003J15Rik	76974	7+:148020915-148023865

15299	Mrps21	66292	3-:95666655-95675404
15300	Olfr1214	258899	2-:88827423-88828357
15301	Dyrk2	69181	10-:118290436-118294066
15302	Lypla3	192654	8+:108674386-108687235
15303	Spcs3	76687	8-:55605785-55615329
15304	Kin	16588	2+:10002209-10014326
15305	Elf2	69257	3-:51059592-51129909
15306	Dpy30	66310	17-:74698820-74715742
15307	MMT00047381		7-:74927664-74928355
15308	Mcc	328949	18-:44589266-44693954
15309	Aldh3a2	11671	11-:61058357-61080614
15310	Mlxipl	58805	5+:135582785-135613755
15311	2810408A11Rik	70419	11-:69710967-69714489
15312	1700034H14Rik	67105	18-:85116685-85120916
15313	Olfr66	18367	7-:111029552-111034790
15314	Olfr202	258997	16-:59283399-59284321
15315	Gak	231580	5-:108997600-109058774
15316	MMT00077385		11+:19620238-19621473
15317	Lrrk1	233328	7-:73426513-73437307
15318	Aars2	224805	17+:45643802-45657790
15319	Tceb1	67923	1-:16633366-16647121
15320	MMT00017479		1+:7167814-7169828
15321	Pus7	78697	5-:23246467-23289513
15322	Rasl12	70784	9+:65246282-65260509
15323	Lysmd1	217779	3+:94938738-94942455
15324	Tep1	21745	14-:51443730-51490217
15325	MMT00007852		2-:127385145-127385244
15326	Tbrg4	21379	11-:6516427-6526026
15327	Olfr1018	258579	2+:85663129-85664063
15328	V1rb9	113857	6+:89696524-89696571
15329	Olfr1033	258571	2+:85881473-85882404
15330	Exosc1	66583	19-:41998473-42007772
15331	Pdk1	228026	2+:71711277-71738223
15332	Ube2g2	22213	10+:77085065-77108735
15333	Tmem190	78052	7+:4734547-4735936
15334	Rnaseh2c	68209	19+:5601884-5602932
15335	Rps17	20068	7-:88487620-88489897
15336	Dpp4	13482	2-:62169775-62249965
15337	Rgs22	626596	15-:35940439-36036876
15338	Nav3	260315	10-:109290000-109341078
15339	Ptges3	56351	10+:127496229-127513513
15340	Cdk5rap1	66971	2-:154161123-154198455
15341	MMT00043449		
15342	Ctnnbip1	67087	4+:148892432-148940545
15343	Rnf183	76072	4-:62088576-62095766
15344	MMT00063326		
15345	Tcstv3	236219	Un_random-:3053-3772
15346	Defb11	246081	8-:23015861-23016883
15347	Pkd1	18763	17+:24686894-24733428
15348	Eif2ak2	19106	17-:79253849-79281882
15349	Endog	13804	2+:30027043-30029589

15350	Exosc5	27998	7+:26444182-26453042
15351	AW822252	331578	X-:51016490-51019923
15352	Socs2	216233	10-:94875005-94877825
15353	Ednrb	13618	14-:104215877-104242896
15354	MMT00080064		9+:61699432-61699869
15355	Capn8	170725	1+:184495142-184562483
15356	Rfc2	19718	5+:135058640-135074197
15357	5133400J02Rik	71297	11-:50924082-51025560
15358	Eif5a2	208691	3+:28680929-28693600
15359	4930465K10Rik	67640	18+:77952948-77954196
15360	Tcfe3	209446	X+:7339826-7352326
15361	Mrm1	217038	11-:84626564-84633017
15362	Zfp35	22694	18+:24148179-24163230
15363	Abi3bp	320712	16+:56614801-56619786
15364	MMT00067596		5+:147294614-147296057
15365	Ext2	14043	2-:93535789-93662702
15366	Ces7	67935	8-:96038094-96058612
15367	Vegfc	22341	8+:55162979-55271803
15368	Rhbdl2	230726	4+:123487283-123506874
15369	MMT00004924		X-:93419675-93420126
15370	Gm249	216797	11-:59186927-59189132
15371	1700010C24Rik	70363	15-:71275519-71356271
15372	Gpr179	217143	11-:97210744-97212857
15373	Olfm3	229759	3+:114783974-114825775
15374	Chd2	244059	7+:80571532-80572744
15375	Rnd3	74194	2-:50985960-51004631
15376	MMT00024461		7+:4257555-4258556
15377	Fxr2	23879	11+:69446819-69466199
15378	Exdl2	97827	12+:81576980-81599106
15379	Cd2bp2	70233	7-:134337189-134339475
15380	AW209491	105351	13+:14728564-14730468
15381	Prl8a1	73244	13-:27665790-27674040
15382	Rhox5	18617	
15383	MMT00043941		9+:4977904-4979576
15384	Mat2a	232087	6-:72383693-72389492
15385	Wdr64	75820	1+:177656519-177674017
15386	Tex12	66654	9-:50364923-50369430
15387	Mgl1	17312	11+:69980275-69984335
15388	Gcap14	72972	14-:37711396-37781902
15389	Tdg	21665	10+:82092611-82113063
15390	Accn3	171209	5+:23919437-23923654
15391	Abca8a	217258	11-:109886949-109947951
15392	Adh6a	69117	3+:137985851-137994098
15393	Sec22a	317717	16-:35313498-35349083
15394	Olfr47	18346	6+:43185608-43186574
15395	Tmem63c	217733	12+:88367439-88430981
15396	MMT00005917		6+:6852812-6853431
15397	Inhbc	16325	10-:126794142-126807452
15398	Galnt13	271786	2+:54707012-54785980
15399	5830408C22Rik	74744	8+:26211632-26220445
15400	Adcy8	11514	15-:64530595-64753722

15401	MMT00075823		15+:57953105-57953476
15402	Zfp354b	27274	11-:50735794-50744435
15403	C330046G03Rik	252974	10+:77292211-77344030
15404	Ireb2	64602	9+:54711590-54760298
15405	MMT00015238	667659	11-:52291383-52293515
15406	Mdfi	17240	17-:47952278-47970168
15407	Kcnn3	140493	3+:89324342-89471326
15408	Mkln1	27418	6+:31348844-31459475
15409	Trim36	28105	18-:46326647-46372018
15410	Pvalb	19293	15-:78021874-78036815
15411	AI747699	381236	19-:33834392-33836240
15412	2210010C17Rik	70080	7-:20523404-20536094
15413	Brp44l	55951	17+:8476721-8490506
15414	Arhgap18	73910	10+:26492332-26638451
15415	Olfr484	258492	7-:115252577-115268775
15416	Alg10b	380959	15+:90054868-90058217
15417	5830433M19Rik	67770	4-:94166775-94223770
15418	Klhl23	277396	2+:69660004-69674707
15419	Olfr745	258296	14+:51261957-51262943
15420	Glod5	69824	X-:7581326-7595618
15421	Scgn	214189	13-:24045329-24083083
15422	Ubap1	67123	4+:41296085-41336798
15423	Tinag	26944	9-:76799591-76893530
15424	Atp6v1g2	66237	17+:35373708-35374743
15425	Adprh	11544	16-:38445769-38450521
15426	1700108M19Rik	71156	12-:36934932-36979987
15427	MMT00009375		10+:121542301-121607417
15428	MMT00071860		8-:73565243-73565470
15429	MMT00045518		Y+:1337257-1338233
15430	Nr4a1	15370	15+:101097292-101105223
15431	Ezh1	14055	11-:101052429-101089262
15432	Ppapdc2	74411	19+:29036401-29039368
15433	Myb	17863	10-:20844741-20880790
15434	MMT00039082		6+:102021453-102022163
15435	MMT00016377		12-:98802695-98803278
15436	3000002C10Rik	378954	9-:109732887-109734003
15437	Mt3	17751	8+:96676643-96678031
15438	Tctex1d1	67344	4+:102658986-102677173
15439	Cdc14b	218294	13-:64296665-64376096
15440	Edem1	192193	6+:108778553-108809951
15441	Zbtb24	268294	10+:41170925-41185377
15442	Scarb2	12492	5-:92873061-92934542
15443	6130401L20Rik	75740	3+:28981494-29052525
15444	Triml1	244448	8-:44215405-44226823
15445	Guca1a	14913	17-:47531592-47537385
15446	Cd320	54219	17+:33980115-33985372
15447	Eif4a3	192170	11-:119149677-119161357
15448	1700018B24Rik	66332	3-:48409653-48413023
15449	Mitd1	69028	1-:37935731-37947277
15450	Dio1	13370	4-:106974424-106979723
15451	Ftcd	14317	10+:76038430-76052304

15452	Adora3	11542	3+:105673820-105726968
15453	Dhrs7c	68460	11+:67611687-67629499
15454	MMT00010886		2+:111022637-111027656
15455	Lsm4	50783	8+:73197290-73202646
15456	Chi3l3	12655	3-:105950471-105970439
15457	Olfr430	258713	1+:175999485-176000382
15458	Eed	13626	7-:97103185-97129480
15459	Man2b2	17160	5-:37198159-37221866
15460	D130059P03Rik	320538	6+:38383932-38441932
15461	Galnt2	108148	8-:126868394-126869620
15462	Enox1	239188	14-:77556571-77558585
15463	Eftud2	20624	11-:102700114-102739028
15464	Aarsd1	69684	11-:101268155-101278747
15465	MMT00053549		10+:30908689-30929709
15466	Gm136	214568	4-:34690809-34693907
15467	Cfc1	12627	1+:34592492-34601156
15468	EG545136	545136	15-:99101450-99126240
15469	4931428F04Rik	74356	8-:107805387-107813764
15470	Trrap	100683	5+:145619865-145620645
15471	Mrps33	14548	6-:39751814-39761038
15472	Tmem209	72649	6-:30447161-30459750
15473	Foxf1a	15227	8+:123608373-123610778
15474	Wfdc15b	192201	2-:164040190-164047396
15475	Olfr846	258279	9-:19164859-19165797
15476	Fem1b	14155	9-:62643837-62659186
15477	Araf	11836	X+:20425694-20438130
15478	OTTMUSG000000	432552	11+:46218228-46221484
15479	Pde6g	18588	11-:120309344-120314743
15480	MMT00078167		7+:119796187-119797434
15481	1110067D22Rik	216551	11-:20723360-20731110
15482	Sdccag10	67285	13-:105421316-105601398
15483	Il17rb	50905	14-:30809385-30821995
15484	Lrrc42	77809	4-:106906120-106926138
15485	Zfp251	71591	15-:76681720-76765960
15486	Mlf2	30853	6+:124881493-124886156
15487	Cyp20a1	77951	1+:60400163-60444903
15488	E430029J22Rik	100033459	1+:175603809-175631841
15489	Tmem158	72309	9-:123168919-123217057
15490	Mtfr1	67472	3+:19088272-19119556
15491	Gdi2	14569	13+:3537514-3564409
15492	Efnb1	13641	X+:96331507-96343567
15493	Zbtb39	320080	10+:127176680-127180752
15494	Dync1li2	234663	8-:106941394-106966701
15495	1700034F02Rik	73324	11+:29447947-29478350
15496	Helz	78455	11+:107409239-107549129
15497	Ftl1	14325	7-:52713317-52715205
15498	Snrpe	20643	1+:135426046-135506785
15499	Dact1	59036	12+:72414878-72421079
15500	MMT00081411		2+:15318470-15319347
15501	Smad7	17131	18+:75527182-75555581
15502	1700018G05Rik	69388	X+:100123710-100124442

15503	MMT00044964		17+:65776811-65778500
15504	Mx2	17858	16+:97757689-97782506
15505	Rpl35	66489	2-:38857100-38860651
15506	Ovch2	244199	7-:114932603-114938468
15507	Shkbp1	192192	7-:28127155-28141038
15508	4632415L05Rik	70808	3+:19794911-19797219
15509	Gabra6	14399	11-:42119939-42134510
15510	4930417G10Rik	74855	12-:4722224-4745238
15511	Acin1	56215	14+:55299785-55300474
15512	Mitf	17342	6+:97941785-97968696
15513	Mcat	223722	15-:83382772-83386117
15514	Msh6	17688	17+:88374423-88390403
15515	Gpx2-ps1	14777	7+:107413059-107413756
15516	Ctcf	13018	8+:108160440-108206821
15517	Olfr1445	258694	19+:12958372-12959315
15518	MMT00013286		2-:87387043-87387352
15519	Eml3	225898	19+:9004183-9016062
15520	1500005I02Rik	72014	11-:114651726-114657212
15521	Mast3	546071	8-:73302016-73321860
15522	1810011O10Rik	69068	8-:25548087-25549418
15523	Krtap5-2	71623	7-:149360439-149361910
15524	Ccr3	12771	9+:123943361-123944441
15525	Emp2	13731	16-:10282727-10313973
15526	Pnliprp2	18947	19+:58834218-58852018
15527	Olfr1265	258340	2+:89877077-89878007
15528	EG629292	629292	18+:60585836-60586861
15529	MMT00011590		7-:110975180-110976389
15530	Syt5	53420	7-:4491697-4497913
15531	Prickle4	66119	17-:47823594-47825047
15532	Map3k13	71751	16+:21892041-21928120
15533	Olfml2b	320078	1+:172574646-172612919
15534	V1rc28	171201	6-:57793558-57794451
15535	Hoxa2	15399	6-:52112511-52114830
15536	Garnl4	380711	11-:74196864-74250638
15537	Ift88	21821	14+:58042962-58136773
15538	B130024G19Rik	434198	7+:77533734-77556033
15539	MMT00028325	100040304	X+:67653506-67655496
15540	C2	12263	17-:34999552-35018992
15541	Amph	218038	13+:19040268-19241712
15542	E230028L10Rik	242805	4+:155165743-155167291
15543	Efhb	211482	17-:53538213-53602379
15544	Ccdc71	72454	9+:108362907-108366865
15545	Grlf1	232906	7-:17082233-17150487
15546	Abca2	11305	2+:25284289-25303719
15547	MMT00044818		11+:3851670-3852908
15548	Cand1	71902	10-:118637085-118677093
15549	Ppp1r1c	75276	2+:79548330-79649583
15550	2010200O16Rik	67495	3-:108359344-108365297
15551	Psmd5	66998	2-:34707619-34726447
15552	AW061290	381110	17+:80014561-80092531
15553	4921536K21Rik	67430	11-:3786202-3795101

15554	Txndc13	52837	2-:134420219-134470214
15555	6030422M02Rik	240697	1+:9914137-9922262
15556	Tm7sf2	73166	19-:6062819-6067841
15557	MMT00080723		17-:15406076-15458846
15558	Pnp1	18950	14+:51567526-51571295
15559	A2m	232345	6+:121586281-121629237
15560	Lrrc25	211228	8+:73141459-73145384
15561	Msgn1	56184	12-:11215188-11215754
15562	Mrpl13	68537	15-:55365649-55388868
15563	EG225058	225058	17+:88530034-88530456
15564	Lix1	66643	17+:17539832-17594585
15565	2310001A20Rik	71881	2-:150408816-150434259
15566	Snn	20621	16+:11073786-11075062
15567	1600002H07Rik	72016	17-:24352002-24357795
15568	MMT00016706		4+:137981634-137981958
15569	BC003331	226499	1-:152209662-152237483
15570	MMT00017284		8+:36878364-36880464
15571	Grid2ip	170935	5+:144125586-144153476
15572	Dtx3l	209200	16-:35926599-35933486
15573	Aipl1	114230	11-:71842666-71850960
15574	MMT00017182	671641	8+:37088765-37089204
15575	Bckdhb	12040	9+:83818761-84017846
15576	Mboat5	14792	6+:124613145-124654196
15577	Glt8d4	232313	6+:100683177-100755070
15578	Lrrc3b	218763	14-:16190033-16269019
15579	MMT00073770		12+:57151519-57152064
15580	MMT00077001		17-:34881932-34883717
15581	Olfr601	258311	7-:110506762-110507706
15582	Klrk1	27007	6-:129562433-129572790
15583	Npm1	18148	11-:33052510-33063057
15584	MMT00027452		9-:49994212-49995150
15585	MMT00033125		8-:58452400-58453004
15586	Ppef1	237178	X-:157061437-157131547
15587	Actl6b	83766	5+:137994862-138010772
15588	Kndc1	76484	7+:147081085-147113170
15589	Hsdl2	72479	4+:59594561-59630935
15590	Sec62	69276	3+:30691796-30719053
15591	Tmem200b	623230	4+:131477071-131480188
15592	Spef2	320277	15-:9507945-9678585
15593	Rit2	19762	18-:31133968-31476722
15594	Gm1967	381411	2-:93695519-93698127
15595	Olfr1233	258974	2-:89179540-89180457
15596	Pcdhb6	93877	18+:37493681-37496000
15597	Cplx3	235415	9-:57449182-57454038
15598	Etfb	110826	7+:50699499-50713149
15599	MMT00019452	677457	7-:54810742-54811683
15600	EG382156	382156	9-:109286400-109289281
15601	Plekhm3	241075	1-:64984403-65003320
15602	Sctr	319229	1+:121940027-121960107
15603	MMT00013018		5-:87780494-87793844
15604	Arl4a	11861	12-:40762408-40764005

15605	Cyp2c39	13098	19+:39585360-39643021
15606	MMT00008386		2+:127515802-127516731
15607	Klf13	50794	7-:71036394-71083735
15608	Stxbp2	20911	8+:3631167-3642730
15609	Mreg	381269	1-:72207087-72238778
15610	1110051M20Rik	228356	2-:91117024-91284864
15611	MMT00008492		2-:139641830-139642262
15612	MMT00008662		3+:3939288-4003173
15613	1810037C20Rik	66294	X-:71630058-71638478
15614	Tpr	108989	1+:152239964-152297434
15615	Zfp800	627049	6-:28191311-28211926
15616	Pon2	330260	6-:5215227-5248308
15617	Atm	11920	9-:53247472-53342071
15618	Sel1l	20338	12-:93079898-93087581
15619	Spef1	70997	2-:130996769-131001439
15620	Cenpb	12616	2-:131003813-131005612
15621	4632428N05Rik	74048	10+:59809670-59835055
15622	MMT00032239		
15623	Tdrd6	210510	17-:43757374-43766867
15624	Crcp	12909	5+:130505175-130536651
15625	Capg	12332	6+:72494530-72512966
15626	MMT00035685		19-:11109903-11110324
15627	Nmral1	67824	16-:4711307-4719362
15628	MMT00036336		10+:124949566-124950227
15629	Asb7	117589	7-:73792673-73824176
15630	B020004C17Rik	432860	14+:57635528-57636378
15631	Lpin1	14245	12-:16542763-16596555
15632	Gsc	14836	12-:105709418-105711446
15633	Wnt8b	22423	19+:44567962-44587005
15634	C130069I09Rik	233026	7-:28769902-28770553
15635	Crkrs	69131	11+:98064616-98112360
15636	Hsp90ab1	15516	17+:45709314-45710136
15637	Vmn2r99	665376	17+:19515529-19531014
15638	MMT00048114		4-:142011581-142180961
15639	Clec2d	93694	6+:129130639-129136552
15640	Ints3	229543	3-:90195323-90206332
15641	5230400G24Rik	75734	1+:82721493-82748962
15642	Ugt2b38	100559	5-:87838971-87853213
15643	MMT00059954		
15644	Diras2	68203	13-:52603039-52603638
15645	Mrpl18	67681	17-:13104220-13108957
15646	Tatdn3	68972	1-:192869717-192886785
15647	MMT00032340		7+:3181089-3181960
15648	Eif4enif1	74203	11+:3102764-3144584
15649	0610012G03Rik	106264	16-:31947136-31948580
15650	Lrrc26	227618	2+:25145466-25146696
15651	Tm2d3	68634	7+:72838344-72846786
15652	Mmp28	118453	11-:83256052-83276360
15653	Gm73	218476	13+:97694472-97695098
15654	1700020A23Rik	75656	2+:130231236-130231808
15655	Bcl2l14	66813	6+:134348659-134388742

15656	Olfr1109	258762	2-:86932614-86933552
15657	Zfyve28	231125	5-:34537523-34540235
15658	Slfn8	276950	11-:82794837-82831217
15659	MMT00074093		16-:64917105-64918916
15660	9130204L05Rik	229550	3-:90892059-90894730
15661	Adm2	223780	15+:89153404-89155163
15662	Avil	11567	10+:126440440-126457619
15663	OTTMUSG000000	241593	2-:104554463-104554942
15664	Masp2	17175	4+:147976662-147978522
15665	Blcap	53619	2-:157382098-157392052
15666	Xrcc4	108138	13-:89988527-90229128
15667	Gpr6	140741	10-:40790299-40831465
15668	1810032O08Rik	66293	11+:116532973-116537110
15669	Klf17	75753	4-:117430440-117438237
15670	Pnmal1	71691	7+:17545105-17546860
15671	St6gal1	20440	16+:23290589-23358668
15672	Ahi1	52906	10+:20672345-20800226
15673	Krtap16-4	170654	16-:88885030-88885334
15674	Riok1	71340	13+:38129163-38153298
15675	MMT00077535	641192	5-:23698515-23715426
15676	Fads1	76267	19+:10257462-10271364
15677	Slc9a9	331004	9+:94839907-95130859
15678	MMT00016379		12+:99082690-99083258
15679	MMT00004444		17+:55387084-55387395
15680	2810030E01Rik	72668	2-:17967238-17969966
15681	MMT00068730		X+:100733627-100793674
15682	Rnasen	14000	15+:12808398-12814790
15683	EG234159	234159	8+:35390880-35391448
15684	Wdr38	76646	2+:38853836-38857102
15685	B3gnt5	108105	16+:19761146-19770714
15686	MMT00078205		4+:46340140-46340755
15687	Olfr1487	258629	19+:13693653-13694599
15688	Tmem71	213068	15-:66358570-66392665
15689	Dscaml1	114873	9+:45234710-45379307
15690	MMT00007845		2-:127271727-127272081
15691	A930034L06Rik	319317	2+:158201447-158209155
15692	MMT00018858		17+:41482995-41483775
15693	P2ry4	57385	X-:97788545-97789629
15694	2310039E09Rik	68016	4+:48676428-48686365
15695	Tead4	21679	6-:128220875-128250843
15696	Pycr2	69051	1+:182834439-182838218
15697	Tbc1d8b	245638	X+:136237690-136240027
15698	Them2	66834	13-:24909823-24923358
15699	Exosc3	66362	4-:45329174-45333487
15700	Crocc	230872	4-:140572559-140603044
15701	MMT00059157		X+:20723825-20724777
15702	MMT00047561	64164	1+:157888023-157899927
15703	Hoxa3	15400	6-:52118988-52141702
15704	MMT00038941		1-:24601056-24602334
15705	Mad1l1	17120	5-:140484643-140797504
15706	4930503L19Rik	269033	18-:70611866-70632412

15707	Trp53i13	216964	11-:77321605-77329249
15708	Retsat	67442	6+:72548592-72558357
15709	Tceal1	237052	X+:133242603-133244403
15710	MMT00050492		15+:98836486-98836800
15711	Anxa1	16952	19-:20447921-20465163
15712	5033413D22Rik	75973	10-:41300028-41307258
15713	Rbm3	19652	X-:7719491-7722807
15714	MMT00017591		17+:35472853-35473227
15715	Tnfrsf18	21936	4+:155400450-155402998
15716	8-Sep	20362	11+:53333397-53361925
15717	MMT00046853		1-:140888230-140897814
15718	Macrod2	72899	2+:142082252-142200047
15719	BC060267	212516	6-:115760754-115773729
15720	Trp53i11	277414	2+:93027756-93041908
15721	MMT00007615		
15722	Ccdc9	243846	7-:16859387-16872110
15723	Csf1	12977	3-:107543974-107563140
15724	Esco2	71988	14-:66437327-66452775
15725	MMT00034976		9-:50961271-50962543
15726	Sncaip	67847	18+:52997663-53067132
15727	MMT00047785		2+:132503885-132505211
15728	Ush2a	22283	1+:190377067-190402410
15729	MMT00052703		
15730	EG545124	545124	15+:82346706-82349153
15731	Mfhas1	52065	8+:36651426-36740304
15732	Usp31	76179	7-:128791261-128850416
15733	Speer4a	75657	5-:26361229-26366043
15734	Mkrn1	54484	6-:39347818-39370386
15735	EG629689	629689	10+:52248325-52249842
15736	Cox7b	66142	X+:103211088-103217296
15737	Smpdl3a	57319	10+:57514405-57531626
15738	2900093L17Rik	78508	4-:45247601-45256865
15739	MMT00079446	100045878	14+:53242466-53373563
15740	1810010H24Rik	69066	11+:106889123-106891752
15741	MMT00062445		15-:10490280-10491327
15742	2900062L11Rik	76976	X+:132528369-132529890
15743	Hoxc6	15425	15+:102839909-102842313
15744	Iqcf1	74267	9+:106402311-106404579
15745	MMT00077003		5-:143614122-143614803
15746	Calr	12317	8-:87365989-87370765
15747	Sfrs1	110809	11+:87860314-87863106
15748	MMT00032580		Y_random-:40879070-40880061
15749	Cd22	12483	7-:31650847-31665286
15750	MMT00015073		14+:54063225-54074528
15751	9230104L09Rik	77705	2-:148672452-148676673
15752	Cyp4a14	13119	4-:115158750-115168737
15753	MMT00014019		2-:142773461-142777220
15754	1700006E09Rik	75437	11+:101848364-101852828
15755	Nfam1	74039	15-:82840804-82847056
15756	MMT00043772		3+:65197258-65199105
15757	A830018L16Rik	320492	1+:11404296-11586400

15758	Stx4a	20909	7+:134985552-134992164
15759	Nkx2-3	18089	19+:43686989-43690380
15760	Trib3	228775	2-:152163160-152169768
15761	C6	12274	15+:4677211-4754044
15762	Rpl12	269261	2+:32817245-32819561
15763	Vmn2r-ps14	545700	4+:145054108-145057441
15764	Smpd2	20598	10-:41206981-41210150
15765	Bcr	110279	10+:74523693-74647794
15766	Bmp2	12156	2+:133378743-133387451
15767	Tmem174	68344	13-:99404933-99407312
15768	Suhw4	235469	9+:72208021-72211305
15769	Pkhd1l1	192190	15+:44413298-44432897
15770	B3gnt7	227327	1+:88200042-88203878
15771	F730014I05Rik	228866	2+:164704898-164716876
15772	C87977	97187	4-:143796666-143802901
15773	MMT00069895		5+:107899726-107920599
15774	Ppp1r16b	228852	2+:158492853-158587916
15775	Nat11	70999	19-:7299765-7315674
15776	Hectd2	226098	19+:36643860-36673436
15777	Lphn3	319387	5+:81941008-82226494
15778	MMT00061514		7+:11390635-11391334
15779	Pln	18821	10+:53057530-53064420
15780	Pde4d	238871	13+:110693294-110742244
15781	Cct4	12464	11+:22890592-22903336
15782	Eml1	68519	12+:109701736-109777821
15783	Fasl	14103	1-:163710963-163718540
15784	9430070O13Rik	77352	1-:158279031-158349477
15785	Isoc2a	664994	7+:4828653-4847001
15786	Apc2	23805	10+:79764582-79778770
15787	Wdr92	103784	11+:17111929-17133773
15788	E130016E03Rik	623474	4+:11506880-11511348
15789	Dnahc11	13411	12-:119116455-119437516
15790	Slc37a3	72144	6-:39284774-39327706
15791	Txn1	22166	4-:57956633-57969156
15792	Use1	67023	8+:73891124-73893628
15793	MMT00010957		13+:26286493-26287207
15794	Olfr589	259054	7-:110303307-110304259
15795	Col1a2	12843	6+:4455760-4490729
15796	4930549C01Rik	67663	4+:136166351-136169128
15797	Ppp2r5c	26931	12+:111685525-111821271
15798	MMT00013138		11+:94143255-94147299
15799	MMT00032080		8-:87377540-87378510
15800	MMT00023224	66449	16-:4616474-4624937
15801	Efna2	13637	10+:79642458-79652437
15802	Ppy	19064	11-:101961244-101962614
15803	Zmym4	67785	4-:126599461-126625725
15804	MMT00056548	245350	X+:12514073-12515660
15805	Ttyh3	78339	5-:141099410-141124902
15806	Dennd4c	329877	4+:86420141-86496504
15807	MMT00028673		14-:99764857-99765273
15808	Vps4b	20479	1-:108667734-108693253

15809	Zfp365	216049	10-:67349535-67375165
15810	Nat3	17962	8+:70071369-70072242
15811	Opn1mw	14539	X+:71372832-71396092
15812	Nptxr	73340	15-:79619835-79635122
15813	Vpreb3	22364	10+:75411102-75412207
15814	Alpk2	225638	18-:65425480-65466975
15815	MMT00080242		15-:26658038-26659479
15816	Fat1	14107	8+:46074281-46137609
15817	MMT00025100		5-:115547692-115548304
15818	Zcchc4	78796	5+:53174235-53215903
15819	MMT00055433	545798	5-:116490544-116533256
15820	Stard9	668880	2+:120490615-120519550
15821	Pcp4	18546	16+:96689373-96747394
15822	Snapc2	102209	8+:4253101-4256219
15823	Krtap5-4	50775	7+:149489499-149490170
15824	D19Ertd721e	225896	19+:8946048-8950145
15825	Fermt3	108101	19-:7073466-7093928
15826	Arl4d	80981	11+:101526874-101529143
15827	Rrm1	20133	7+:109590457-109617441
15828	1700060C16Rik	73425	6-:143541872-143599808
15829	Hlcs	110948	16-:94351294-94490230
15830	Sema5a	20356	15+:32347113-32626085
15831	Olfr1217	258903	2-:88863243-88864158
15832	Crybb2	12961	5-:113487286-113497786
15833	4932412H11Rik	242838	5+:3858628-3866664
15834	9030611O19Rik	104943	12+:31758858-31763850
15835	Sar1a	20224	10+:61143078-61156042
15836	Hpn	15451	7-:31883743-31900294
15837	Plg	18815	17+:12571511-12612250
15838	BC039210	234839	8-:125005617-125025879
15839	MMT00045078		16+:23033913-23050925
15840	H2-M9	14997	17-:36777371-36779589
15841	Ndufb6	230075	4-:40217686-40226440
15842	Med12l	329650	3+:59031604-59122331
15843	Tnn	329278	1-:162015165-162077971
15844	Klhdc9	68874	1-:173288536-173290949
15845	Zpld1	239852	16-:55226493-55283351
15846	1700088E04Rik	27660	15-:78965188-78971661
15847	Gm392	235858	8-:57910790-57912361
15848	Bst1	12182	5+:44210146-44234550
15849	Pacs2	217893	12+:114252871-114310783
15850	Zfp62	22720	11+:49017820-49031262
15851	Rbbp8	225182	18+:11851520-11901712
15852	MMT00023533	544896	12-:114899906-114900439
15853	D430036J16Rik	319405	9+:81525957-81538235
15854	EG382109	382109	9-:109626227-109635227
15855	Boll	75388	1-:55356913-55463474
15856	C330019G07Rik	215476	5-:33136130-33170464
15857	Itih5	209378	2+:10075245-10173892
15858	Snrpe	20643	1-:135500450-135506844
15859	MMT00057272		X+:135791423-135792688

15860	BC004004	80748	17+:29405895-29438470
15861	MMT00002080		3-:81359853-81360707
15862	Mrap	77037	16+:90738354-90750032
15863	Dph5	69740	3+:115591111-115632005
15864	Rps6ka2	20112	17+:7374635-7507662
15865	MMT00018841		9-:26766058-26770212
15866	Cdc45l	12544	16-:18780545-18812065
15867	Crhr2	12922	6-:55040080-55082966
15868	5430425J12Rik	71390	13+:73837271-73847358
15869	Amh	11705	10+:80267984-80270392
15870	Aqp5	11830	15+:99421212-99425252
15871	MMT00078036		13-:91023054-91038463
15872	4933424B01Rik	71177	6-:146498154-146526281
15873	Syne2	319565	12+:77128694-77129067
15874	Vmn2r14	231591	5-:109644721-109650439
15875	1700072E05Rik	73495	X+:83289537-83292652
15876	Eif4ebp2	13688	10-:60895244-60915417
15877	Olfr1491	258342	19+:13779318-13780276
15878	MMT00001587		2-:110258323-110259773
15879	Obp1b	18250	X-:75485801-75490729
15880	Olfr554	258322	7+:109788761-109789714
15881	Hcn4	330953	9+:58671318-58708569
15882	AA986860	212439	1+:132628605-132641198
15883	Tulp3	22158	6-:128271183-128305812
15884	Olfr395	259007	11-:73720053-73720992
15885	Adamts7	108153	9+:90066311-90094931
15886	Gm941	381112	17+:80734570-80748926
15887	Lce1m	66203	3-:92821731-92822982
15888	Cyp2d34	223706	15-:82446394-82451337
15889	Moxd2	194357	6-:40828793-40837493
15890	Fbxw2	30050	2-:34660864-34678701
15891	Dgat2l3	245533	X+:97767649-97773226
15892	Slc22a5	20520	11-:53678525-53705143
15893	Dock4	238130	12+:41362669-41571232
15894	Rif1	51869	2+:51932712-51977899
15895	MMT00080717		17-:15672028-15672655
15896	Pdgfrb	18596	18+:61205094-61244713
15897	D10Jhu81e	28295	10-:77624811-77632513
15898	Papd1	67440	18+:4375589-4396782
15899	MMT00058392		3-:113084762-113089679
15900	Rasal2	226525	1-:159070130-159216985
15901	Tpm3	59069	3+:89876658-89896047
15902	MMT00045851		18+:9252930-9254563
15903	Ntng1	80883	3-:109584022-109946143
15904	Ncl	17975	1-:88247194-88255798
15905	Gemin8	237221	X+:162608419-162627682
15906	0610010K14Rik	104457	11-:70048711-70051416
15907	Hyou1	12282	9+:44188602-44199131
15908	Pebp1	23980	5-:117732662-117737555
15909	Rpl9	20005	5-:65779605-65781682
15910	Adamtsl1	77739	4+:85699878-86020896

15911	Glra4	14657	X-:133292235-133310450
15912	Hcls1	15163	16+:36935100-36963298
15913	Ston2	108800	12-:92828718-92982320
15914	Dapk2	13143	9+:66006039-66120046
15915	Spata21	329972	4+:140658888-140660882
15916	Il1rap	16180	16+:26581853-26716652
15917	3100002J23Rik	75429	11-:58606297-58615467
15918	Fkbp11	66120	15-:98554802-98558629
15919	Fbxl6	30840	15-:76366160-76369165
15920	A630001G21Rik	319997	1-:87613658-87623200
15921	Naca	17938	10+:127472690-127485692
15922	Psd	73728	19-:46386574-46399419
15923	Olfr652	259050	7+:111712736-111713692
15924	Rbm38	56190	2+:172847429-172860230
15925	Rpl19	19921	11+:97889134-97891791
15926	Kctd20	66989	17+:29089444-29104898
15927	Ltb4r2	57260	14+:56380760-56381843
15928	5830411J07Rik	74742	2-:70659716-70661490
15929	Cdc16	69957	8+:13769354-13781930
15930	Slc38a6	625098	12+:74387766-74452883
15931	Ces3	104158	8-:95689971-95721678
15932	Comp	12845	8+:72897456-72905964
15933	Serp2	72661	14-:76932621-76956512
15934	Armcx3	71703	X+:131291137-131295995
15935	Pnpla3	116939	15+:83998266-84019944
15936	Mid2	23947	X+:137199494-137302251
15937	Adamts4	240913	1+:173180548-173190027
15938	Cst11	78240	2-:148594344-148597233
15939	Nagk	56174	6+:83745049-83752549
15940	Npr1	18160	3-:90255017-90269436
15941	MMT00024086		9+:79421614-79422433
15942	Lhfpl2	218454	13+:94942762-94962104
15943	Gpr89	67549	3-:96674988-96709221
15944	Tmem62	96957	2+:120802490-120833568
15945	1700018L24Rik	75528	8+:11840513-11855760
15946	Nol14	75416	5-:34981185-35002772
15947	BC057552	212123	8-:86618736-86625629
15948	Padi1	18599	4-:140368895-140401693
15949	MMT00060795		Y-:200804-202689
15950	Adpgk	72141	9+:59139414-59164003
15951	C330027C09Rik	224171	16+:48994297-49019814
15952	Bai3	210933	1-:25477402-25616710
15953	Acsf3	257633	8+:125309791-125341782
15954	MMT00027348		19+:5324132-5325229
15955	Inadl	12695	4+:98213424-98386293
15956	1700023D19Rik	75510	7+:51964112-51975214
15957	6330548G22Rik	76167	5+:124933187-124941132
15958	Rag1ap1	19729	3-:89072167-89074480
15959	Vmn2r101	627576	17+:19726430-19749281
15960	MMT00074791		19+:3205603-3206071
15961	MMT00067071		10-:29334060-29334382

15962	Olfr472	258770	7+:115046232-115047165
15963	Ush2a	22283	1+:190472154-190502567
15964	Pglyrp3	242100	3+:91826526-91835484
15965	Kif1a	16560	1-:94914855-94998399
15966	Mfsd8	72175	3-:40623038-40650667
15967	Atp2b2	11941	6-:113698287-113930577
15968	Pknox1	18771	17+:31701745-31742991
15969	Tnrc4	78784	3+:94282251-94296115
15970	Npal1	70701	5+:73039050-73059555
15971	Aifm2	71361	10+:61183389-61191059
15972	Pygo1	72135	9+:72773453-72794527
15973	MMT00025143	100045149	15+:62009747-62016306
15974	Usp9y	107868	Y-:639815-779046
15975	Cnnm1	83674	19+:43515700-43570238
15976	Nmu	56183	5-:76762520-76792802
15977	Phca	66190	7-:105364953-105458038
15978	Glrp1	14659	1-:90396446-90406641
15979	Rmi1	74386	13+:58503961-58511451
15980	Myh14	71960	7-:51861172-51920867
15981	Adamts18	208936	8-:116233373-116372813
15982	Syne2	319565	12+:77050326-77068739
15983	Cxx1c	72865	X+:50911208-50911601
15984	MMT00075074		16+:38483773-38484678
15985	Nova1	664883	12-:47798886-47814563
15986	Ahnak	66395	19+:9063770-9151410
15987	MMT00064591		
15988	Lbx1	16814	19-:45307781-45310226
15989	MMT00069487		X-:71380375-71381632
15990	Dnmbp	71972	19-:43921301-43977548
15991	Tmcc1	330401	6-:116083778-116143371
15992	Creb3l2	208647	6-:37282425-37314142
15993	Tcrg-V7	21641	13+:19269893-19270394
15994	9130213B05Rik	231440	5+:91946725-92053020
15995	Scg3	20255	9-:75491180-75531824
15996	Grtp1	66790	8-:13176869-13200535
15997	Tspan8	216350	10+:115254339-115286671
15998	MMT00049642		5-:148994412-148995992
15999	3110039M20Rik	67293	12+:50490873-50508335
16000	Nol10	217431	12+:17355256-17436900
16001	D330027H18Rik	101359	6-:29119237-29119830
16002	Mia2	338320	12+:60196786-60205545
16003	Rps6kb1	72508	11-:86313645-86358258
16004	Sfrs8	231769	5+:130007096-130077255
16005	MMT00048275		12-:115298032-115347680
16006	Anxa2	12306	9+:69312276-69339593
16007	EG622589	622589	10-:128113167-128113783
16008	MMT00081082		10+:57011200-57011671
16009	1110033F04Rik	68673	11-:99495442-99496398
16010	MMT00002955		13-:41330153-41330446
16011	Zbtb37	240869	1-:162950051-162962858
16012	AU017962	103341	10+:103607129-103607572

16013	Nt5c3	107569	6-:56832398-56873823
16014	Banf2	403171	2+:143858834-143899713
16015	Spt1	20770	Un_random-:2674949-2678403
16016	Nfkbid	243910	7+:31208415-31212771
16017	Ppp2r5b	225849	19-:6227763-6235843
16018	MMT00074939		18+:87401389-87403116
16019	2410004P03Rik	73667	12-:17011763-17018361
16020	Ddx52	78394	11+:83755635-83776576
16021	Phxr1	18686	2+:3349139-3349643
16022	Efna3	13638	3-:89117826-89120674
16023	A430060F13Rik	100039739	11-:94706143-94710813
16024	P2ry6	233571	7-:108086144-108112884
16025	Otop2	237987	11+:115168474-115185123
16026	Gsg1l	269994	7-:133024215-133164185
16027	Ppp2r2d	52432	7+:146027800-146074737
16028	Rtn4r	65079	16+:18127798-18152499
16029	Igh-VJ558	16061	12-:117158466-117158975
16030	Prpf3	70767	3-:95634539-95659711
16031	Stab2	192188	10-:86303954-86470687
16032	Cyp27a1	104086	1+:74778500-74784464
16033	MMT00023218		13-:13988251-14047236
16034	Dclk3	245038	9+:111370115-111391741
16035	Chd8	67772	14-:52842273-52857247
16036	Bin1	30948	18+:32536910-32595385
16037	MMT00011022		3-:84355935-84357187
16038	Sec31a	69162	5+:100844942-100929882
16039	Erbb4	13869	1-:68086615-68127369
16040	Slc22a1	20517	17-:12841741-12868559
16041	Cnot2	72068	10-:115922564-116018557
16042	Pold4	69745	19+:4231936-4233631
16043	Sult1c2	69083	17-:53969185-53985143
16044	Creb5	231991	6+:53237725-53397712
16045	Rasl10b	276952	11+:83225971-83232284
16046	Ppwd1	238831	13-:104995204-105019060
16047	Irf8	15900	8+:123260290-123280318
16048	Nsfl1c	386649	2+:151319921-151337038
16049	MMT00031860	60315	15+:102162140-102168280
16050	MMT00008777		3-:10753934-10754428
16051	1700001C02Rik	75434	5+:30768449-30786460
16052	Tsg101	22088	7-:54144745-54175263
16053	Hnf4a	15378	2+:163372996-163396138
16054	Prr15	78004	6+:54276898-54279644
16055	Slco4c1	227394	1-:98715153-98717906
16056	Tcf4	21413	18+:69504174-69843997
16057	Vps36	70160	8+:23303282-23329723
16058	MMT00019507		11+:50805294-50805723
16059	Ttn	22138	2-:76665539-76671192
16060	Gys2	232493	6-:142371133-142421467
16061	Ccdc131	216345	10+:114822012-114837953
16062	EG545741	545741	X+:117274705-117275324
16063	Ndufs5	595136	4-:123389953-123395491

16064	B020004J07Rik	545662	4-:101508145-101510744
16065	Ldhd	52815	8-:114150880-114154236
16066	Coq2	71883	5-:101083745-101103265
16067	Pcaf	18519	17+:53706654-53810192
16068	1700008P20Rik	69301	7-:25118100-25118955
16069	E330021D16Rik	243676	6-:136350373-136351908
16070	Tubgcp5	233276	7+:63061485-63086816
16071	Ccdc90b	66365	7+:99710039-99730801
16072	MMT00011155		8-:71997409-71999134
16073	Car11	12348	7+:52955375-52959979
16074	Prkcc	18752	7+:3303847-3330721
16075	Bloc1s2	73689	19-:44213748-44220933
16076	MMT00039676		X-:123218651-123219246
16077	Mocs2	17434	13+:115608493-115618932
16078	Defb15	246082	8-:23040284-23043182
16079	MMT00078587		6+:12515690-12516881
16080	Prkcdbp	109042	7-:112628596-112630726
16081	EG382450	382450	10+:10332800-10334057
16082	Abcg4	192663	9-:44089164-44089774
16083	Pla2g2c	18781	4+:138281661-138300488
16084	Tns3	319939	11-:8331653-8515854
16085	MMT00023382		19-:25870247-25871343
16086	D18Ertd653e	52662	18+:68181136-68266346
16087	Olfr640	258819	7-:111169886-111170830
16088	MMT00030639		2-:14733469-14745930
16089	4732474O15Rik	238455	12+:120684086-120704145
16090	Exoc1	69940	5+:76989345-76996203
16091	Art1	11870	7+:109250256-109262447
16092	Akt2	11652	7+:28376781-28423219
16093	4732473B16Rik	100342	4+:133036102-133043851
16094	MMT00082062		12-:3931470-3932059
16095	MMT00071048		10-:4554200-4554622
16096	Csmd3	239420	15-:48289041-48505172
16097	Dnmt3l	54427	10+:77512586-77526366
16098	Rps20	67427	4-:3758731-3762746
16099	D030070L09Rik	225280	18-:24263253-24280333
16100	Tnni3	21954	7-:4469970-4473917
16101	MMT00044631		5-:146251281-146251852
16102	Cd72	12517	4-:43460606-43467435
16103	Vip	22353	10-:4701475-4707323
16104	Ugt3a2	223337	15+:9265367-9300625
16105	Vasp	22323	7-:19842268-19868182
16106	Ccdc36	434438	9-:108319652-108323892
16107	Entpd6	12497	2+:150574803-150597410
16108	MMT00077778		18+:9544546-9545478
16109	MMT00067385		12+:89569567-89571682
16110	Adrb1	11554	19+:56796861-56798262
16111	EG627967	627967	7-:122070473-122071240
16112	V1ra1	22296	6+:90078417-90080980
16113	Zfp498	666311	5+:146041394-146052027
16114	Lyplal1	226791	1-:187911764-187941189

16115	Mup3	17842	4-:61744512-61748405
16116	Dnahc14	240960	1+:183682646-183685575
16117	Id4	15904	13+:48356798-48359405
16118	Cby3	76653	11+:50171254-50173205
16119	4930578N16Rik	75051	2-:69596089-69627586
16120	Utp11l	67205	4-:124356009-124370798
16121	E130303B06Rik	102124	8-:108227552-108232096
16122	Lsp1	16985	7+:149657747-149680504
16123	Sec11c	66286	18+:65960231-65977311
16124	Mdm4	17248	1-:134888182-134921966
16125	MMT00014025		19+:16099940-16100208
16126	E330034G19Rik	105418	14+:25115217-25129183
16127	Dffb	13368	4-:153339476-153349098
16128	Agbl1	244071	7+:83569540-83589879
16129	Trafd1	231712	5-:121821735-121835582
16130	Gosr2	56494	11-:103540550-103558972
16131	Ptpre	19267	7+:142729604-142875038
16132	Psat1	107272	19-:15979222-15999549
16133	Robo3	19649	9-:37223746-37237719
16134	Alg11	207958	8+:23171198-23178761
16135	Srd5a2l2	243078	5-:83729908-83784025
16136	MMT00046779		13+:66699587-66699622
16137	Xrcc5	22596	1+:72353994-72441526
16138	Il12a	16159	3+:68494565-68502447
16139	Vkorc1l1	69568	5+:130452856-130458763
16140	Xpo6	74204	7-:133245780-133317763
16141	Ccdc96	66717	5+:36827240-36829448
16142	Gpr165	76206	X+:93908777-93912823
16143	Cd209d	170779	8-:3871956-3878483
16144	Rybp	56353	6-:100182111-100237404
16145	Olfr1449	258300	19+:13009229-13010172
16146	Gucy1a3	60596	3-:81898547-81949774
16147	Tlr2	24088	3-:83640194-83645475
16148	Krt13	16663	11-:99978911-99982809
16149	MMT00025643		12+:20280792-20281220
16150	Snrp70	20637	7-:52631967-52647503
16151	MMT00042117		2+:133025307-133026029
16152	Ceacam10	26366	7+:25562279-25569670
16153	Syngr1	20972	15+:79921781-79943870
16154	Rgs20	58175	1-:4900325-5009507
16155	Dusp12	80915	1-:172809790-172811172
16156	MMT00028187		2+:160671261-160672201
16157	D9Ertd280e	272636	9-:99217063-99258936
16158	4930511J11Rik	74720	16-:8409368-8425229
16159	Igh-VJ558	16061	12-:116861366-116930533
16160	Top3b	21976	16+:16877946-16893077
16161	Lrch4	231798	5+:138070410-138081729
16162	Myo16	244281	8+:10272533-10562269
16163	Tmco1	68944	1+:169238391-169260921
16164	Fdps	110196	3-:88897510-88905794
16165	Taf5l	102162	8-:126520217-126545209

16166	Si	20431	10+:128143342-128157072
16167	Stbd1	52331	5+:93032065-93035612
16168	Crlf2	57914	5-:109983729-109987960
16169	Tigd5	105734	15+:75740689-75742148
16170	S100a11	20195	3+:93324418-93330208
16171	Sncb	104069	13-:54860221-54867801
16172	Bcl2l15	229672	3+:103636524-103656316
16173	Lba1	320429	9+:111250291-111268242
16174	Lba1	320429	9+:111275449-111296806
16175	Agpat5	52123	8+:18846268-18884375
16176	St3gal5	20454	6+:72047839-72104564
16177	C87499	381590	4-:88274718-88276043
16178	Myh11	17880	16-:14194992-14291456
16179	Gulo	268756	14-:66605623-66607112
16180	Rnaseh2a	69724	8-:87481621-87482124
16181	MMT00078660		1-:81738169-81739463
16182	Oasl1	231655	5+:115373414-115387920
16183	1110002B05Rik	104725	12-:55746379-55757485
16184	Arf6	11845	12+:70473193-70474803
16185	Olfr414	258756	1+:176360560-176361513
16186	Cfp	18636	X-:20502649-20508888
16187	6530403A03Rik	67797	13+:38296813-38318917
16188	Adamts16	271127	13-:70867359-70874752
16189	Olfr807	258931	10-:129191568-129192504
16190	Osbpl11	106326	16+:33196166-33243397
16191	Klc1	16593	12+:112997166-113033107
16192	MMT00013307		19-:13697166-13714196
16193	MMT00045006	635992	16+:19028324-19028745
16194	Alg9	102580	9+:50583434-50651631
16195	Unk	217331	11+:115891663-115922507
16196	Grin3b	170483	10+:79433459-79439935
16197	MMT00065977		3-:97994426-97995001
16198	Atp8b4	241633	2-:126148549-126306422
16199	Samhd1	56045	2-:156925131-156960881
16200	MMT00017071		16-:8619047-8621311
16201	MMT00072359		X+:142425310-142429203
16202	Ccnd1	12443	7-:152115836-152125735
16203	Tspan6	56496	X-:130426278-130432874
16204	Olfr586	259115	7-:110270343-110271296
16205	2810002O09Rik	72345	X-:92621905-92623938
16206	Kcna2	16490	3+:106907022-106908521
16207	Ica1	15893	6-:8580527-8708458
16208	Ppara	19013	15+:85566560-85632163
16209	MMT00031904		15+:95545588-95546623
16210	Metrnl	210029	11+:121563725-121577594
16211	Accn1	11418	11-:80693683-80966405
16212	Med19	381379	2+:84518581-84528371
16213	4922503N01Rik	230603	4+:109079722-109117868
16214	Ocln	18260	13-:101267721-101322673
16215	Serpinb9d	20726	13+:33286431-33330092
16216	MMT00068421		

16217	Csnk1a1	93687	18+:61715235-61747953
16218	Rpgrip1	77945	14+:52730596-52780605
16219	4932415G12Rik	67723	10-:94136449-94151316
16220	MMT00074659		1+:42160134-42161004
16221	Troap	78733	15+:98905391-98913838
16222	Adipor2	68465	6-:119303169-119367670
16223	Mag	17136	7-:31684201-31699851
16224	Cdc2l5	69562	13-:17809233-17896143
16225	Mak10	78689	13+:59686771-59736140
16226	MMT00033542		5+:73772040-73783378
16227	Rarres1	109222	3-:67282813-67319535
16228	ORF34	207375	X+:147778687-147905030
16229	Abcb11	27413	2-:69076345-69180644
16230	MMT00020946		
16231	Rnf4	19822	5+:34692532-34696082
16232	Dchs2	229459	3+:83074219-83160463
16233	1600027J07Rik	69794	8-:105867777-105871435
16234	Taf6	21343	5-:138619849-138625725
16235	MMT00011701		
16236	Dopey1	320615	9+:86380860-86449528
16237	Bat5	193742	17+:35226200-35239930
16238	Nmi	64685	2-:51804019-51828731
16239	Vmn2r42	22310	7+:9213439-9934715
16240	Eya1	14048	1-:14160723-14294651
16241	MMT00003591		4+:25346490-25346553
16242	MMT00053207		12+:86596449-86597574
16243	Kctd19	279499	8-:107910649-107913047
16244	1700020C07Rik	75642	2-:164652880-164654007
16245	4930588N13Rik	75860	5+:150242290-150273296
16246	4930473A06Rik	320226	4+:83301431-83309595
16247	4930596D02Rik	239036	14-:36622676-36624845
16248	Cdr2	12585	7-:128100555-128125798
16249	Lyz2	17105	10-:116715177-116719298
16250	MMT00069147	329858	4-:59342087-59440936
16251	G6pc	14377	11+:101229029-101239215
16252	Ubtd1	226122	19+:42056426-42109133
16253	MMT00044478		1-:144989922-144991247
16254	Lyrm5	67636	6+:145159675-145165456
16255	Reg3b	18489	6+:78320878-78323459
16256	Tmem132d	243274	5-:128344937-128751594
16257	Osbpl8	237542	10+:110601872-110734303
16258	Krt2	16681	15-:101641121-101648600
16259	Igf1r	16001	7+:75097744-75149237
16260	Nav2	78286	7+:56617467-56653525
16261	Olfr1337	258306	4-:118454242-118455188
16262	MMT00000771		
16263	Olfr283	259038	15-:98208609-98209539
16264	Zfp768	233890	7-:134486308-134488828
16265	Prkci	18759	3+:30894897-30949368
16266	Rnf214	235315	9-:45674647-45715556
16267	Zfp574	232976	7+:25857663-25867502

16268	Cdc34	216150	10+:79145127-79151680
16269	Ccdc50	67501	16+:27389148-27447107
16270	Lgals12	56072	19-:7671150-7681654
16271	Wdr68	71833	11+:105898190-105920634
16272	MMT00025784		2+:171926040-171926814
16273	Ltbp3	16998	19+:5742126-5758525
16274	Gm609	208166	16+:45411651-45412566
16275	Mlxip	208104	5+:123844938-123890907
16276	Tmem40	94346	6-:115679157-115708979
16277	Olfr518	258303	7-:116024117-116025118
16278	Ccdc148	227933	2-:58675840-58871016
16279	BC052055	328643	16+:20594639-20605449
16280	MMT00018773	677414	17+:40990985-40998259
16281	2600010E01Rik	72446	2-:101554442-101679123
16282	6130401L20Rik	75740	3+:29412809-29570549
16283	EG627427	627427	18+:37079953-37081860
16284	Rasl11a	68895	5+:147656630-147659301
16285	Oscar	232790	7-:3562366-3563434
16286	MMT00030638	100047024	6-:69808415-69913603
16287	Extl1	56219	4-:133912288-133927694
16288	Ctbs	74245	3+:146112763-146128796
16289	Myocd	214384	11-:64990068-65083443
16290	Myo5b	17919	18+:74762815-74930513
16291	Tbrg1	21376	9-:37455560-37464910
16292	Prpf18	67229	2-:4543266-4544429
16293	4933406J08Rik	74401	2+:122012096-122032126
16294	Chst7	60322	X+:19636695-19674644
16295	Nlrp14	76858	7+:114324671-114341616
16296	MMT00003690		4+:10846226-10874044
16297	Sprr2g	20761	3+:92178561-92178792
16298	X99384	27355	10-:60782409-60846056
16299	Creld2	76737	15+:88650075-88657111
16300	Gab3	210710	X-:72233890-72330246
16301	Pm20d2	242377	4-:33261701-33276355
16302	MMT00005255		14+:28023523-28024849
16303	Fgd4	224014	16-:16435798-16504311
16304	MMT00056871		X-:72003825-72004262
16305	Tub	22141	7+:116154494-116173915
16306	1700016K13Rik	74222	5-:130189265-130203812
16307	Lemd1	213409	1+:134122889-134153947
16308	4931406C07Rik	70984	9-:15110740-15111815
16309	Tmem186	66690	16-:8633823-8637794
16310	Sae1	56459	7-:16912402-16973134
16311	A830018L16Rik	320492	1+:11738033-11788647
16312	4930529M08Rik	78774	2+:145968748-145979653
16313	Fbxo30	71865	10+:11001167-11016896
16314	MMT00046842	229687	3-:105819839-105831837
16315	MMT00048401		2+:149182625-149183140
16316	Bclaf1	72567	10+:20042768-20064434
16317	Ugcgl2	66435	14-:119488386-119498563
16318	Ms4a15	545279	19-:11053698-11057785

16319	MMT00051350		4+:54866863-54867508
16320	Ivd	56357	2+:118687756-118706322
16321	P2rx4	18438	5+:123157633-123179049
16322	Lmod3	320502	6-:97188525-97202637
16323	Smpd3	58994	8-:108779158-108789819
16324	D2Wsu81e	227695	2-:30028966-30033965
16325	Psmc3ip	19183	11-:100952833-100956703
16326	MMT00070606	278676	14+:52445394-52446131
16327	Ubfd1	28018	7+:129210935-129222646
16328	Slc10a2	20494	8-:5085622-5105232
16329	0610010O12Rik	66060	18+:36508407-36562631
16330	2310043J07Rik	69665	5+:136533181-136540189
16331	Setd8	67956	5-:124911790-124912317
16332	Scd4	329065	19+:44408422-44419388
16333	Wdr64	75820	1+:177697168-177745862
16334	Parp2	11546	14+:51427613-51440875
16335	Otop1	21906	5+:38691379-38694614
16336	2310040A07Rik	69638	4-:41585176-41587319
16337	Grin2c	14813	11-:115110483-115128557
16338	Senp1	223870	15-:97872400-97917565
16339	Bach1	12013	16+:87699193-87730946
16340	Scrn2	217140	11+:96891307-96895272
16341	Cmtm2a	73381	8-:106804948-106817033
16342	Krt33a	71888	11-:99877242-99877304
16343	Dgkh	380921	14-:78968631-79125492
16344	Arpc5l	74192	2+:38863658-38871392
16345	Rasip1	69903	7+:52890342-52894461
16346	Phospho2	73373	2+:69627793-69635225
16347	Mgp	17313	6-:136820957-136824269
16348	Tubgcp3	259279	8-:12614274-12672101
16349	Crybb1	12960	5+:112684840-112698599
16350	Nadsyn1	78914	7-:150981498-151008746
16351	MMT00019132		X-:89076437-89108867
16352	4831426I19Rik	212073	12-:106201380-106236888
16353	EG666504	666504	15-:76363827-76364911
16354	Abhd11	68758	5+:135485168-135488038
16355	Rcl1	59028	19+:29175994-29217781
16356	9030607L17Rik	71564	10+:80165051-80168118
16357	AI480653	268880	16-:30957423-31050865
16358	MMT00020436		7-:123947395-123947978
16359	Fbxw11	103583	11+:32542599-32646788
16360	Cdk5r2	12570	1+:74901671-74902781
16361	Olfr703	258589	7+:113988126-113989084
16362	Lyrm4	380840	13-:36184665-36209213
16363	Adh5	11532	3+:138106115-138118215
16364	MMT00063014		15+:37350888-37352499
16365	Ube2k	53323	5+:65928569-65986379
16366	Glis3	226075	19-:28605362-28614898
16367	MMT00076058		8_random+:321154-339848
16368	Smarca4	20586	9+:21420706-21507813
16369	Rcan1	54720	16-:92393698-92466164

16370	Olfr115	257908	17-:37746545-37764061
16371	Ppp2cb	19053	8+:34710388-34729662
16372	MMT00077009		14-:37831938-37837696
16373	G6pc2	14378	2+:69058027-69065907
16374	4932431P20Rik	114675	7+:30309561-30322883
16375	MMT00080030	353287	8-:113582806-113586346
16376	Thsd1	56229	8+:23348971-23370357
16377	Tmem9	66241	1+:137914591-137931601
16378	Ccl8	20307	11+:81928693-81930297
16379	Tppp3	67971	8-:107991396-107995289
16380	Vasn	246154	16+:4639972-4650797
16381	Ccbp2	59289	9+:121815575-121819148
16382	Arpc1b	11867	5+:145882527-145889054
16383	Il20rb	213208	9-:100358138-100375368
16384	Slc25a42	73095	8-:72709239-72736167
16385	MMT00073590		17-:64050829-64051715
16386	Cdyl	12593	13+:35751748-35965492
16387	2010316F05Rik	67939	11-:29413054-29415033
16388	EG381818	381818	6-:132388168-132391755
16389	MMT00040811		
16390	Plb1	665270	5+:32535080-32619909
16391	Pde10a	23984	17+:9042849-9175265
16392	EG627648	627648	13-:58110636-58214949
16393	MMT00023354		1-:60579498-60579865
16394	MMT00004253		14-:74733337-74890909
16395	Adora2b	11541	11+:62062570-62079271
16396	Plcb2	18796	2-:118528360-118554174
16397	1700112E06Rik	76633	14+:23396948-23418491
16398	EG239502	239502	15-:63322517-63324102
16399	Izumo1	73456	7+:52877179-52882598
16400	Nup50	18141	15+:84757807-84770277
16401	4932415M13Rik	211496	17+:53863012-53942804
16402	Adad2	75773	8+:122136644-122140824
16403	Agk	69923	6+:40275493-40345860
16404	Trappc2	66226	X-:162878732-162891070
16405	Pafah1b1	18472	11-:74487456-74538172
16406	Mettl6	67011	14-:32292196-32308216
16407	Mrpl44	69163	1+:79774431-79778017
16408	Etv4	18612	11-:101631064-101646577
16409	Eif3c	56347	7-:133690425-133709880
16410	2010001E11Rik	72045	10-:39640187-39646731
16411	V1rc12	171185	6-:66628793-66629678
16412	Tank	21353	2+:61416641-61492168
16413	Pkd1l2	76645	8-:119533469-119568514
16414	Scrn1	69938	6-:54458967-54516406
16415	Sult1c1	20888	17-:54101178-54129967
16416	Commd2	52245	3-:57450281-57455629
16417	Pitx2	18741	3+:128916889-128922506
16418	Eef2k	13631	7+:128001933-128046903
16419	Maml2	270118	9+:13424433-13513976
16420	MMT00020928		

16421	Hrasls	27281	16+:29217697-29228320
16422	Kctd5	69259	17-:24184402-24210411
16423	Rreb1	68750	13+:37917834-38043871
16424	MMT00046595		9+:39847539-39848323
16425	Agxt2l1	71760	3+:130320492-130338666
16426	MMT00023627		17-:92584656-92585156
16427	Snrpb	20638	2-:129997382-130005095
16428	Nfya	18044	17-:48527259-48549046
16429	MMT00047901		5-:34824041-34825219
16430	Vac14	234729	8+:113160268-113244296
16431	Recql4	79456	15-:76534171-76540906
16432	Ttc18	76670	14-:21213419-21271449
16433	Asb3	65257	11+:30898325-31001241
16434	Fhad1	329977	4-:141452341-141478346
16435	MMT00071843		12+:32103596-32105117
16436	Gpr39	71111	1+:127736579-127769750
16437	Tkt	21881	14+:31362364-31386797
16438	Igfl3	232925	7+:18761837-18767674
16439	MMT00056625		5-:115503476-115503858
16440	Gprc5a	232431	6+:135015701-135034723
16441	Cant1	76025	11-:118271923-118280345
16442	Glb1l2	244757	9-:26572686-26614060
16443	Ccdc59	52713	10+:105278534-105284564
16444	Sox15	20670	11+:69468874-69470121
16445	Mkrn3	22652	7-:69562478-69565042
16446	Taf11	68776	17-:28038069-28046107
16447	Cat	12359	2-:103294160-103325277
16448	Vmn2r104	22313	17-:20166388-20178707
16449	BC004044	80752	5+:139269142-139286017
16450	EG545238	545238	18+:3447055-3473440
16451	2010007H12Rik	69871	5+:138220145-138221335
16452	Slc6a11	243616	6+:114081390-114197733
16453	Olfr1372-ps1	257871	
16454	2310038H17Rik	67099	1-:64653668-64663801
16455	Zkscan2	210162	7-:130623027-130643964
16456	Il33	77125	19+:29999626-30035205
16457	BC106175	724112	12-:8485906-8487109
16458	Olfr677	258355	7+:112204761-112205698
16459	2310002D06Rik	69522	12+:81608190-81618940
16460	MMT00045897		6-:131556442-131559000
16461	Dll1	13388	17-:15504319-15512217
16462	Dnase2b	56629	3-:146244880-146259576
16463	MMT00036516		
16464	Vmn2r66	233437	7-:92143318-92155743
16465	Ccdc130	67736	8-:86781693-86794259
16466	Syne1	64009	10+:5188781-5193442
16467	Card10	105844	15-:78605567-78633099
16468	MMT00079120		4+:138898053-138899246
16469	MMT00061447		3+:87358680-87359408
16470	Cyhr1	54151	15-:76476197-76489870
16471	Prim1	19075	10+:127452270-127467086

16472	1700015C15Rik	75532	13-:64532557-64533750
16473	Neto2	74513	8-:88160486-88224793
16474	Bhlhb2	20893	6+:108610690-108616768
16475	Samd5	320825	10-:9394302-9395483
16476	E130009J12Rik	381107	17-:65785602-65826120
16477	MMT00004032	677563	12-:116625091-116625339
16478	Chrna7	11441	7-:70243821-70357412
16479	Pcsk7	18554	9+:45715079-45737803
16480	Slitrk4	245446	X-:61522643-61530155
16481	Gatad2a	234366	8-:72432555-72440256
16482	Dgcr8	94223	16-:18283778-18284509
16483	AW551984	244810	9-:39395138-39411200
16484	Tet1	52463	10-:62274452-62285508
16485	Cuedc1	103841	11+:87983065-88006432
16486	Slc25a22	68267	7-:148615650-148623517
16487	Cggbp1	106143	16+:64851887-64859319
16488	A330017A19Rik	442840	17+:47025946-47026729
16489	Olfr90	258469	17-:37222176-37223108
16490	MMT00048286		13+:80300564-80301136
16491	6330500D04Rik	193385	13+:24706495-24823952
16492	Hk2	15277	6-:82675043-82724453
16493	Mphosph9	269702	5-:124765197-124766180
16494	2310007D09Rik	71878	2+:158593825-158612371
16495	Gap43	14432	16-:42248744-42340663
16496	Tdrd9	74691	12+:113301703-113307066
16497	MMT00063964		X-:58437015-58439320
16498	2410017P07Rik	103268	10-:41438651-41529419
16499	Pusl1	433813	4-:155263540-155266222
16500	4921539E11Rik	70941	4-:102903054-102963464
16501	Loh11cr2a	67776	9+:38525881-38550877
16502	Chsy1	269941	7+:73254829-73318650
16503	MMT00025844		
16504	Srpr	67398	9+:35018761-35024598
16505	MMT00062901		2-:23446098-23446782
16506	MMT00082773		
16507	A830039N20Rik	268723	14-:24111516-24112033
16508	Foxa1	15375	12-:58641620-58646949
16509	Baat	12012	4-:49502290-49516043
16510	Tspyl4	72480	10+:34017277-34021126
16511	MMT00017530		11-:49940599-49941622
16512	Ryr3	20192	2-:112471540-112797456
16513	EG629036	629036	17+:53493810-53494533
16514	Vps52	224705	17+:34092723-34103841
16515	Gramd4	223752	15+:85898242-85967744
16516	Dvl1	13542	4+:155221843-155232474
16517	D4Wsu53e	27981	4+:134479559-134483287
16518	4933426G20Rik	74460	15+:63746013-63757008
16519	Igf2bp3	140488	6-:49037340-49164470
16520	MMT00064994	238440	12-:116550353-116550843
16521	Adar	56417	3+:89534726-89555096
16522	Ccdc85a	216613	11-:28476390-28483542

16523	Rps13	68052	7-:123475025-123477647
16524	Arl5b	75869	2+:14977032-14998219
16525	Lbp	16803	2+:158132280-158158032
16526	Sftpa1	20387	14+:41945071-41947765
16527	B230208H17Rik	227624	2-:25439560-25463974
16528	Ddr2	18214	1-:171907776-172040690
16529	Nrn1l	234700	8+:108418265-108418917
16530	MMT00070246		18+:9629728-9630956
16531	6330403K07Rik	103712	11-:70845451-70846957
16532	2610016C23Rik	71804	10+:20067630-20081125
16533	MMT00027784		18-:30579549-30602631
16534	Nbea	26422	3-:55429115-55895045
16535	Kctd9	105440	14+:68334351-68360218
16536	L1td1	381591	4+:98393497-98403953
16537	A930021C24Rik	77803	13-:105635369-105653974
16538	Ccdc81	70884	7-:97014658-97052123
16539	Klhl20	226541	1-:163020550-163061582
16540	MMT00017193		8-:78235492-78404744
16541	1700092M07Rik	74307	19+:8815243-8815707
16542	Clstn1	65945	4+:148960746-149022008
16543	Nek7	59125	1-:140381505-140458357
16544	2700045P11Rik	72555	16+:11984382-11997273
16545	Cyld	74256	8+:91221032-91272771
16546	Ankrd45	73844	1+:163072816-163097969
16547	1500002O20Rik	71997	7+:25184648-25207796
16548	Atp6v1e1	11973	6-:120745720-120772632
16549	Wtap	60532	17-:13159665-13185978
16550	MMT00018745		17-:92771632-92772463
16551	C1ql2	226359	1+:122237160-122239151
16552	MMT00049458		2+:104660314-104662221
16553	Cilp2	68709	8-:72404266-72407356
16554	1700049K14Rik	73382	14+:64723177-64732590
16555	BC006662	223267	14-:123290095-123312357
16556	MMT00014805	100045978	13-:50398623-50439316
16557	Rbpms	19663	8-:34893115-34976877
16558	Arhgdig	14570	17-:26336127-26338127
16559	MMT00001400		16-:50664932-50666467
16560	Slc22a12	20521	19-:6536111-6542851
16561	Fbxo9	71538	9-:77929306-77956432
16562	MMT00005808		18+:7932849-7933510
16563	Pctk2	237459	10+:92671044-92729816
16564	Tead4	21679	6-:128174301-128202007
16565	Arsb	11881	13+:94541638-94711181
16566	Igfbpl1	75426	4-:45822348-45839687
16567	Rpl14	67115	9+:120480633-120483551
16568	2310005G13Rik	69457	16-:57038747-57071467
16569	Scn1a	20265	2-:66110948-66156196
16570	2410187C16Rik	76773	15+:57972960-57990207
16571	Pqlc1	66943	18+:80453045-80489453
16572	D14Ertd668e	219132	14-:59967567-59984283
16573	Cryge	12968	1-:65095198-65097687

16574	4732496O08Rik	242736	4+:143002329-143009598
16575	Aff3	16764	1-:38232835-38243852
16576	Ms4a2	14126	19-:11691682-11698133
16577	MMT00070754		11+:28856718-28857990
16578	Wdr26	226757	1-:183107823-183129065
16579	Rcan2	53901	17+:43938798-43973446
16580	Tnfaip2	21928	12+:112683172-112693228
16581	1600027N09Rik	73247	2+:180317314-180320995
16582	Mapk8ip2	60597	15+:89284560-89292420
16583	Kcnu1	16532	8+:26960127-27048245
16584	Nup35	69482	2+:80478987-80499263
16585	Syvn1	74126	19+:6047089-6053707
16586	Adamts8	30806	9+:30750145-30751186
16587	E130112L23Rik	268739	14+:52615734-52625331
16588	Cnbp	12785	6-:87793167-87801002
16589	2610019F03Rik	72148	8-:13952010-13974754
16590	C920005C14Rik	338368	15+:82175460-82179500
16591	MMT00051549		3-:6135078-6139030
16592	Psmf1	228769	2-:151543992-151566993
16593	Vangl1	229658	3-:101961374-102000904
16594	Rbx1	56438	15+:81296785-81305690
16595	Psmb1	19170	17-:15612923-15635244
16596	Ptrf	19285	11-:100818053-100819252
16597	Dido1	23856	2-:180357116-180436807
16598	E430018J23Rik	101604	7-:134534943-134536950
16599	4833424O15Rik	75769	3+:117278279-117390816
16600	C230055K05Rik	626391	5-:105243910-105246095
16601	MMT00019843		12+:107747069-107748042
16602	Itln1	16429	1-:173460625-173463595
16603	Nkx3-2	12020	5-:42152409-42155459
16604	Loh12cr1	67774	6+:134591042-134661186
16605	MMT00009297		9-:104071163-104072141
16606	4930579G22Rik	69034	5+:130384434-130387150
16607	Gpx2	14776	12-:77893325-77896493
16608	MMT00026286		17-:36896490-36898247
16609	Gucy2c	14917	6-:136645805-136730245
16610	Rem1	19700	2+:152452765-152460499
16611	MMT00082279		7-:35280943-35286422
16612	Dazl	13164	17-:50420375-50432834
16613	1110031I02Rik	66179	11-:121038906-121065962
16614	Zscan21	22697	5+:138558268-138575444
16615	Chdh	218865	14+:30822130-30854865
16616	Paox	212503	7+:147311581-147320233
16617	Sh2d4a	72281	8+:70805930-70871479
16618	Hps3	12807	3-:19896620-19935299
16619	Anks1	224650	17+:28111849-28199721
16620	Cpt2	12896	4-:107576587-107596139
16621	Btbd10	68815	7+:120495293-120512917
16622	Incenp	16319	19-:9946835-9973959
16623	RP23-357I14.1	381417	2+:122319954-122349283
16624	Psma8	73677	18+:14864657-14920807

16625	Mrps16	66242	14-:21210452-21212777
16626	Pip5kl1	227733	2+:32430313-32431411
16627	MMT00074583		8-:41833718-41875442
16628	MMT00052888		X-:39481500-39483061
16629	Sall2	50524	14-:52930848-52948437
16630	Armcx5	494468	X+:132278962-132281857
16631	Psmd12	66997	11+:107340841-107359350
16632	MMT00062595		X-:51185502-51187617
16633	D14Ertd436e	218978	14-:48161777-48188116
16634	Crim1	50766	17+:78600108-78734697
16635	Spry4	24066	18-:38749257-38761069
16636	Tmem189	407243	2-:167468725-167487033
16637	Vmn2r85	623734	10-:129855333-129866512
16638	Spred2	114716	11+:19824933-19921449
16639	MMT00078937		11-:105026089-105028054
16640	4930408G06Rik	68179	1-:59053964-59064601
16641	Vmn2r116	619697	17+:23522878-23534725
16642	Usp29	57775	7+:6683293-6919926
16643	Srrm1	51796	4-:134877947-134909051
16644	Tcrb-J	21580	6+:41012331-41013101
16645	Aqp7	11832	4-:40981226-40995061
16646	Rsbn1l	242860	5-:20401894-20402724
16647	D16H22S680E	27883	16-:18300919-18324536
16648	Sema3f	20350	9-:107584760-107607873
16649	Wasf1	83767	10+:40640081-40658375
16650	Cd300a	217303	11+:114751675-114759133
16651	Tnfrsf13b	57916	11+:60954336-60961143
16652	Rab4a	19341	8+:126329924-126359187
16653	Pdzd2	68070	15-:12518269-12528813
16654	Oas1f	243262	5+:121297400-121307634
16655	MMT00012959		3-:105846092-105846385
16656	Slc33a1	11416	3-:63747171-63768112
16657	Atpif1	11983	4-:132086470-132089513
16658	MMT00011796	632223	6+:136348473-136350157
16659	Lrp12	239393	15-:39702139-39711868
16660	Gpc6	23888	14+:117324651-118375766
16661	Tmprss4	214523	9-:44980809-45012158
16662	Gemin4	276919	11-:76024259-76027425
16663	Olfr818	258773	10-:129382161-129383116
16664	Car4	12351	11+:84771344-84779541
16665	Ppp1r3c	53412	19-:36806037-36811162
16666	Eif4g1	208643	16+:20683685-20692954
16667	2900010M23Rik	67267	17-:27259616-27270821
16668	Opn4	30044	14-:35405547-35413328
16669	Tjp2	21873	19-:24169088-24248478
16670	Csmd2	329942	4+:127736205-128241394
16671	MMT00081758		7-:3908044-3935099
16672	Tmem82	213989	4-:141170149-141173939
16673	Gm62	215919	10-:38540606-38541572
16674	BC024814	239706	16+:8470905-8490289
16675	Flrt2	399558	12+:96930444-97019311

16676	Aspa	11484	11-:73118942-73137989
16677	OTTMUSG00000(	238568	13+:33755913-33763453
16678	Olfr669	259045	7+:112087041-112087994
16679	Ctsk	13038	3+:95304756-95312843
16680	MMT00014809	100126778	17+:40590632-40591521
16681	Rpl38	67671	11+:114529910-114533645
16682	Lrdd	57913	7-:148624413-148629254
16683	Cst8	13012	2+:148624572-148631330
16684	9830001H06Rik	320706	2-:156853122-156904542
16685	Vwf	22371	6+:125502977-125636695
16686	Wdfy3	72145	5-:102261973-102263981
16687	Disc1	244667	8+:127648395-127785047
16688	Snapc3	77634	4+:83063415-83114013
16689	MMT00070066		1+:83154587-83156002
16690	Tjp3	27375	10-:80735972-80753979
16691	Mast2	17776	4-:115979367-116136453
16692	Bat4	81845	17+:35259896-35261751
16693	Dip2a	64451	10-:75725482-75776049
16694	Sepp1	20363	15+:3224665-3230504
16695	Hs3st6	328779	17+:24889935-24895628
16696	Magee1	107528	X+:102316531-102318706
16697	Abcg3	27405	5-:105364784-105411688
16698	Sumo1	22218	1-:59696287-59727607
16699	EG226601	226601	1-:168413874-168426148
16700	Rad21	19357	15-:51794149-51814218
16701	H2-M10.1	14985	17-:36460962-36463095
16702	2810422J05Rik	75620	8-:73037296-73046994
16703	MMT00018063		12-:58799488-58800089
16704	Ppif	105675	14+:26513655-26519950
16705	Plcb1	18795	2+:134612265-135298158
16706	MMT00026784		9-:36620616-36629038
16707	Ywhaz	22631	15-:36705259-36720745
16708	AI846148	68229	19-:7432171-7457364
16709	Srpk1	20815	17-:28726547-28759357
16710	Adam12	11489	7-:141075654-141416780
16711	MMT00062670		8+:74284115-74284388
16712	Pnrc1	108767	4-:33332211-33335615
16713	Ppp1r14d	72112	2-:119043858-119055588
16714	EG625603	625603	9-:22134708-22134944
16715	2610034M16Rik	69239	17-:59018231-59106888
16716	Asah2	54447	19-:32059137-32177630
16717	Stam	20844	2+:13995881-14069165
16718	Zbtb7c	207259	18+:76219111-76306096
16719	Slc43a3	58207	2+:84776820-84798086
16720	Mtmr7	54384	8-:41636453-41694370
16721	Stk3	56274	15-:34806361-35085437
16722	Gmeb2	229004	2-:180988043-181012827
16723	Tnfsf8	21949	4-:63494204-63522205
16724	Psmd11	69077	11+:80242115-80286367
16725	Sry	21674	Y-:1918381-1919568
16726	MMT00033971		1-:34483861-34489235

16727	Gm73	218476	13+:97636285-97663054
16728	Uhmk1	16589	1-:172130013-172145381
16729	Smpx	66106	X+:154137049-154190521
16730	Osbpl3	71720	6-:50246805-50332836
16731	V1rb3	113853	6-:90173402-90174334
16732	Atxn3	110616	12-:103160359-103196414
16733	Cd81	12520	7+:150238699-150253828
16734	Tbx4	21387	11+:85704358-85728451
16735	Gm239	237558	10-:116216611-116233192
16736	Olfr1126	258834	2+:87297323-87298268
16737	BC053393	407814	11+:46385110-46390997
16738	Pmch	110312	10+:87553681-87555118
16739	Tgfbrap1	73122	1-:43104063-43132797
16740	MMT00032254		
16741	MMT00079972		5+:26333464-26402521
16742	Bbc3	170770	7+:16894986-16903682
16743	Bad	12015	19+:7016350-7026383
16744	Evpl	14027	11-:116081873-116082600
16745	Ccdc37	243538	6-:90353731-90363869
16746	Bhmt2	64918	13-:94426214-94444234
16747	Acvr2a	11480	2+:48670221-48756173
16748	Zfp810	235050	9-:22081191-22112082
16749	Ivl	16447	3-:92375271-92376678
16750	Casp6	12368	3+:129608383-129616865
16751	4921510H08Rik	66716	10+:97155692-97157555
16752	Hapln4	330790	8+:72607355-72613678
16753	Hfe2	69585	3+:96331871-96333132
16754	Hsp90aa1	15519	12-:111929240-111933891
16755	MMT00000111		10+:24420098-24420906
16756	MMT00047531		1-:129179371-129198052
16757	Tmem146	106757	17+:56800664-56803877
16758	MMT00023865		4-:17936232-17936666
16759	Gabrq	57249	X+:70070774-70083883
16760	1600029D21Rik	76509	9+:50302629-50313238
16761	MMT00018149		17-:92926783-92927221
16762	Atp8a2	50769	14-:60266586-60705671
16763	C920021L13Rik	109149	3+:95675367-95691556
16764	Ear6	93719	14+:52473672-52474140
16765	Igfn1	226438	1-:137851146-137872643
16766	Sharpin	106025	15-:76177467-76181534
16767	MMT00038956		13+:64775836-64776091
16768	Hnmt	140483	2-:23858446-23904896
16769	Cox10	70383	11-:63777393-63789980
16770	Krtap16-1	170651	16-:88873909-88874514
16771	Abi2	329165	1+:60466574-60536543
16772	MMT00010554		1-:79919112-79920001
16773	Tmem164	209497	X+:139186571-139268960
16774	4932438A13Rik	229227	3+:36784278-36845428
16775	Kcna10	242151	3+:106996996-106998507
16776	1110032O16Rik	68742	7-:134029658-134066452
16777	MMT00053707		5+:125915438-125916193

16778	Pik3c2b	240752	1+:134983149-135002733
16779	MMT00018299		11+:111137342-111138308
16780	Rel	19696	11-:23641989-23670970
16781	Pramef12	77632	4-:143981576-143998367
16782	1110012N22Rik	68515	11-:120507345-120509473
16783	Vps37c	107305	19+:10718625-10789114
16784	D330012F22Rik	269952	7+:87377802-87393324
16785	MMT00053231		12+:86758797-86796669
16786	Rp1l1	271209	14+:64640747-64646886
16787	Samd8	67630	14-:22616441-22617103
16788	Imp3	102462	9+:56785306-56786204
16789	Lpar2	53978	8+:72346524-72352021
16790	EG382639	382639	12+:113917603-113918865
16791	Rimbp3	239731	16+:17209100-17213997
16792	Sh3yl1	24057	12+:31597051-31645021
16793	Arl11	219144	14+:61928686-61930618
16794	Chrnb1	11443	11-:69598122-69609382
16795	Olfr628	259101	7+:110878864-110881902
16796	Agt	11606	8-:127080487-127088483
16797	MMT00016048		13-:16730403-16731326
16798	Gpr37	14763	6-:25618107-25640729
16799	Obscn	380698	11-:58906797-58917052
16800	Sstr3	20607	15-:78369690-78370975
16801	Ms4a14	383435	19-:11375985-11388823
16802	Def8	23854	8+:125966904-125987167
16803	Etv1	14009	12+:39506895-39593572
16804	Hist1h4j	319159	13+:21826964-21827274
16805	Ddx39	68278	8+:86239105-86247246
16806	Havcr1	171283	11+:46564678-46592114
16807	Fos	14281	12+:86814989-86817407
16808	Elk3	13713	10-:92711629-92773782
16809	Rxrb	20182	17+:34168942-34174475
16810	Sh3pxd2b	268396	11+:32271517-32323578
16811	Rere	68703	4+:149780448-149996071
16812	1500002O10Rik	68986	2-:70327999-70401416
16813	MMT00032610		6+:114688964-114693809
16814	AU042651	328258	13+:56539761-56575599
16815	Tmem120b	330189	5+:123526333-123567455
16816	Btf3	218490	13-:99079852-99086922
16817	OTTMUSG000000	208820	4+:12834032-12908599
16818	Abhd8	64296	8-:73980606-73987545
16819	Lyve1	114332	7-:117995366-118006467
16820	Bambi-ps1	81913	2+:122292625-122293240
16821	Abcd2	26874	15-:90979314-91022230
16822	Gadl1	73748	9+:115850391-115983465
16823	Inhbb	16324	1-:121312041-121335320
16824	2310076L09Rik	66968	17-:56251024-56256970
16825	MMT00056634		1+:159526434-159526829
16826	4833418A01Rik	75763	2+:70893789-70937198
16827	Arhgdib	11857	6-:136872170-136890259
16828	MMT00004165		16+:85644498-85645182

16829	Igh-6	16019	12-:114923646-114924126
16830	MMT00014034		3-:59406405-59422757
16831	Ush2a	22283	1+:190551989-190557483
16832	Trim63	433766	4+:133871104-133879151
16833	Lrp11	237253	10+:7309658-7345273
16834	Hist2h4	97122	3-:96066858-96067256
16835	MMT00062293		10-:96231541-96232476
16836	Hip1r	29816	5+:124423657-124452855
16837	Crabp1	12903	9+:54612614-54620916
16838	Akap13	75547	7+:82600529-82895723
16839	Nedd4l	83814	18+:65080784-65157076
16840	Rpl38	67671	11+:114529837-114533645
16841	Syt16	238266	12+:75230355-75330411
16842	2310003L22Rik	69487	2+:139996765-140029368
16843	Cry1	12952	10-:84595800-84647534
16844	Ugt2b1	71773	5-:87345661-87355554
16845	Mamdc4	381352	2-:25418636-25425881
16846	EG638251	638251	7-:15050512-15077275
16847	Olfr176	258995	16-:58872040-58872974
16848	Spnb1	20741	12-:77698643-77750911
16849	Trmt1	212528	8+:87213176-87224127
16850	MMT00063598		11-:35606217-35606965
16851	Slc34a2	20531	5+:53440595-53462900
16852	Vash1	238328	12+:88029768-88036634
16853	4933407H18Rik	71101	5+:33721874-33757557
16854	Kif15	209737	9+:122860199-122927852
16855	Itk	16428	11-:46138654-46202995
16856	Hif1a	15251	12+:75009016-75047878
16857	Perp	64058	10+:18564876-18576876
16858	Tmem38a	74166	8+:75096001-75111181
16859	0610007L01Rik	71667	5+:130695710-130719559
16860	Cdc42bpa	226751	1+:181890557-182092722
16861	Nlrc4	268973	17-:74825652-74847558
16862	Peg12	27412	7-:69606756-69609396
16863	2410001C21Rik	66404	2+:172266058-172295401
16864	Tfb2m	15278	1-:181458186-181476348
16865	Olfr273	258821	4-:52868429-52869383
16866	Scara3	219151	14-:66538235-66557148
16867	Stab1	192187	14-:31952204-31981758
16868	EG667587	667587	19-:38777538-38778938
16869	Ndufa12	66414	10+:93661753-93683693
16870	Atic	108147	1+:71603735-71626203
16871	MMT00072624		4+:23412430-23412994
16872	Cntnap2	66797	6+:45870790-45965487
16873	Klrc2	16642	6-:129599554-129610707
16874	Fcer1g	14127	1-:173159708-173164417
16875	Wfdc3	71856	2-:164556685-164571555
16876	B930036G03Rik	408070	12-:109444084-109449832
16877	5930434B04Rik	381356	2+:26865097-26874805
16878	Zbed4	223773	15+:88610370-88614980
16879	Ltb4r1	16995	14+:56384867-56387326

16880	Depdc1b	218581	13+:109106560-109179775
16881	MMT00031504		14+:114879627-114879658
16882	Ogfrl1	70155	1-:23373275-23389912
16883	Rnf7	19823	9+:96371915-96379049
16884	Lrat	79235	3-:82699325-82707892
16885	MMT00040429		6-:69581633-69582172
16886	Dpy30	66310	17-:74698813-74715789
16887	Olfr1260	258983	2+:89817936-89818868
16888	H6pd	100198	4-:149353561-149377330
16889	Smarcc2	68094	10+:127897962-127927641
16890	H2-Eb2	381091	17+:34462624-34472830
16891	Pex2	19302	3-:5560511-5576128
16892	Syngr3	20974	17-:24822706-24826886
16893	Matr3	17184	18+:35713035-35714755
16894	Lin7c	22343	2+:109740309-109741086
16895	F2rl2	14064	13+:96466874-96472673
16896	MMT00002629		X-:140667303-140668026
16897	Vps28	66914	15-:76452517-76456457
16898	Invs	16348	4+:48292689-48444821
16899	Atp8b3	67331	10-:79998279-80001864
16900	4930404K13Rik	74845	14-:120183978-120215075
16901	Pias2	17344	18+:77392289-77394446
16902	MMT00045509		
16903	MMT00033771		4-:131471358-131475945
16904	Sphk2	56632	7-:52966095-52969035
16905	Gkn1	66283	6-:87295646-87300909
16906	EG433170	433170	18-:33543822-33544663
16907	MMT00061537		16-:91514119-91514816
16908	Muc13	17063	16+:33794122-33820013
16909	Cav3	12391	6+:112409684-112422864
16910	Ptprr	19279	10+:115455454-115711985
16911	Pou5f1	18999	17+:35642995-35647714
16912	Rplp2	67186	7+:148633553-148637248
16913	Pira3	18726	7-:3860216-3867087
16914	MMT00039379		
16915	MMT00019508		3+:93924626-93924985
16916	Lrrc19	100061	4-:94304832-94316837
16917	Gpr4	319197	7+:19807503-19808600
16918	Slc35a1	24060	4-:34611198-34634508
16919	Pea15a	18611	1-:174126858-174128546
16920	Acsl1	14081	8+:47578185-47619832
16921	Wee2	381759	6+:40393976-40416807
16922	Ube2d1	216080	10-:70717727-70747981
16923	A430048G15Rik	381426	2+:133257804-133279097
16924	Sfrp5	54612	19-:42273175-42276742
16925	2410016O06Rik	71952	12+:85291619-85293893
16926	Cnpy3	72029	17-:46872988-46889143
16927	Cyp3a57	622127	5+:146106148-146131974
16928	Sltm	66660	9+:70390559-70440037
16929	Slc39a3	106947	10-:80491285-80496657
16930	Hmgb1-rs17	628431	8+:34594926-34595502

16931	Actn4	60595	7-:29678274-29747299
16932	Sacm1l	83493	9+:123439004-123501716
16933	1700012H19Rik	76393	11-:112684278-112686439
16934	Kctd19	279499	8-:107919170-107932526
16935	Tmem16k	102566	9-:122085010-122260715
16936	Ccdc117	104479	11-:5428410-5442571
16937	Ubac2	68889	14+:122419329-122420255
16938	AI662250	106639	17-:56851595-56851941
16939	MMT00038813		X-:138840619-138840986
16940	St3gal3	20441	4-:117605601-117807021
16941	EG434339	434339	8+:96929038-96930156
16942	MMT00041689		13+:61824802-61825540
16943	Gimap7	231932	6+:48668657-48674633
16944	Zc3h3	223642	15-:75584861-75672338
16945	Col23a1	237759	11+:51330698-51371089
16946	Ivns1abp	117198	1+:153191645-153211388
16947	Wfs1	22393	5-:37357345-37380148
16948	Slco1a6	28254	6-:142034299-142134674
16949	Iigp1	60440	18+:60549448-60552281
16950	MMT00079465		7+:151977841-151978170
16951	R74862	97423	7-:150219494-150238995
16952	Pax1	18503	2+:147190795-147200782
16953	Pdlim7	67399	13-:55608134-55614795
16954	Sh3glb1	54673	3-:144351807-144383287
16955	MMT00068267		10-:68885784-68886498
16956	Slc24a5	317750	2+:124894507-124914510
16957	MMT00046108		2+:59234728-59245772
16958	Gpc1	14733	1+:94728262-94756772
16959	A230072C01Rik	320742	X+:20539239-20563849
16960	MMT00008703		3-:14627380-14635239
16961	Mup1	17840	4-:60510886-60514832
16962	4921525H12Rik	329738	3+:108568027-108578219
16963	Tceal5	331532	X-:132735489-132738394
16964	EG666111	666111	18-:30618618-30619231
16965	Gstt2	14872	10-:75294630-75297031
16966	Col4a4	12829	1-:82449128-82583391
16967	MMT00070933		13-:76339455-76372410
16968	Epha2	13836	4+:140857176-140885292
16969	Kcnc2	268345	10+:111892662-111900367
16970	1500005K14Rik	76566	11-:75834138-75841286
16971	MMT00013779		11-:98192553-98204015
16972	Nenf	66208	1-:193130675-193141997
16973	MMT00027196		17+:53335694-53338047
16974	Eif2b4	13667	5-:31489940-31495458
16975	BC030307	103220	10+:86168451-86175935
16976	Zhx3	320799	2-:160604959-160663062
16977	Lgals3	16854	14+:47993482-48005789
16978	5730536A07Rik	68250	9+:65974476-65986762
16979	Rft1	328370	14+:31467544-31503140
16980	Syne1	64009	10+:5056968-5075683
16981	Ms4a1	12482	19-:11325840-11419606

16982	4930448N21Rik	384817	8-:48498125-48531717
16983	Pigx	72084	16-:32084507-32087594
16984	Ctnnal1	54366	4-:56825189-56878067
16985	Tmem16f	105722	15+:95621327-95805904
16986	Gpr87	84111	3-:58982844-58984007
16987	Pex14	56273	4-:148334645-148473921
16988	Clec4f	51811	6-:83595017-83606026
16989	E030037K03Rik	319836	1-:175511802-175529394
16990	Sc4mol	66234	8-:67196941-67212375
16991	Gjb6	14623	14-:57742855-57743639
16992	Irf2bp2	270110	8-:129112197-129117090
16993	MMT00075165		5-:7399414-7400277
16994	Trove2	20822	1-:145602725-145624108
16995	Dppa3	73708	6+:122576441-122580289
16996	MMT00059495		
16997	Spp2	75396	1+:90303593-90323013
16998	Fbxl15	68431	19+:46402698-46404933
16999	MMT00018943		16-:11185350-11186019
17000	2410089E03Rik	73692	15+:8136082-8137065
17001	Hmga1	15361	17+:27696477-27700608
17002	Limd2	67803	11-:106019988-106020703
17003	V1re11	171264	7-:10859522-10860453
17004	MMT00016962	634201	3+:85983800-85984031
17005	MMT00015015		X+:132874649-132875206
17006	Igh-VJ558	16061	12-:116597327-116861676
17007	Rundc3b	242819	5-:8494783-8622612
17008	Ntn4	57764	10+:93104198-93208598
17009	Pclo	26875	5+:14514917-14795987
17010	Galns	50917	8-:125102309-125135308
17011	MMT00039362		13+:3004955-3008498
17012	2300002M23Rik	69542	17+:35704479-35705889
17013	Fhl4	14202	10-:84560470-84565233
17014	4930403N07Rik	73936	12-:73202056-73210204
17015	Rbm25	67039	12-:85009128-85009230
17016	Dnajc11	230935	4+:151307798-151356233
17017	Slmo1	225655	18+:67624553-67639422
17018	Galnt14	71685	17-:73843524-73924313
17019	1810006K21Rik	69038	19+:10278502-10281987
17020	Tox3	244579	8-:92771740-92871848
17021	6720468P15Rik	78073	19+:57583075-57587259
17022	MMT00055891		8-:67654962-67656225
17023	Slc6a9	14664	4+:117507940-117541909
17024	Fgf16	80903	X+:102959879-102969517
17025	MMT00072213		12-:116383674-116842641
17026	Lass3	545975	7+:73888530-73940825
17027	Iws1	73473	18+:32227405-32257328
17028	MMT00043846		3+:129370553-129370683
17029	Erc2	238988	14+:28689772-28954565
17030	Rpgrip1l	244585	8-:93745270-93837122
17031	Wdr52	212517	16+:44432072-44481900
17032	MMT00052549		13-:75661021-75661729

17033	Ccdc111	408022	8-:47671696-47695979
17034	MMT00020368		13-:36650437-36651076
17035	Glt8d3	223827	15-:93074967-93105512
17036	Tctn3	67590	19-:40679587-40686690
17037	Dctn1	13191	6+:83129847-83149964
17038	Ppp1r12b	329251	1-:136656985-136852522
17039	Kctd19	279499	8-:107906356-107909421
17040	Olfr513	258718	7+:115898371-115899301
17041	1700001J11Rik	72224	9+:39858207-39860507
17042	Epas1	13819	17+:87153439-87230934
17043	Tmem127	69470	2+:127074004-127086826
17044	Gpr55	227326	1-:87837450-87907557
17045	1810030N24Rik	66291	4-:34715920-34725586
17046	Fggy	75578	4+:95478798-95593636
17047	Mlh3	217716	12-:86575478-86607234
17048	Olfr1502	258793	19+:13936284-13937234
17049	Capsl	75568	15+:9365782-9395790
17050	Kmo	98256	1+:177562323-177590983
17051	Mrps36	66128	13-:101505982-101514590
17052	Golga5	27277	12+:103707344-103736115
17053	MMT00068622		4+:47466491-47468272
17054	Jph3	57340	8+:124254462-124314721
17055	Brpf3	268936	17+:28942899-28973689
17056	Pla2g2d	18782	4+:138331649-138338058
17057	MMT00028474		13+:49846022-49917765
17058	V1rh16	171274	13-:23323983-23324910
17059	Upp2	76654	2+:58558178-58588063
17060	MMT00078061		3-:116230233-116231625
17061	MMT00028106		18-:6028476-6030437
17062	2900010J23Rik	72931	2-:32134328-32143586
17063	Hoxc8	15426	15+:102820969-102823797
17064	Cd48	12506	1+:173612224-173635007
17065	MMT00006878		
17066	MMT00078909		1-:52560185-52561072
17067	1700057G04Rik	78459	9+:92245763-92252714
17068	Olfr661	258743	7+:111836530-111837488
17069	1500003O03Rik	56398	2+:119373531-119411035
17070	MMT00008656		3-:4890967-4891231
17071	Samd9l	209086	6-:3322521-3327259
17072	Zwint	52696	10+:72117660-72137709
17073	1810049H13Rik	66431	11-:120317917-120319377
17074	ENSMUSG00000(	668178	15+:100165360-100170129
17075	Creb3l3	208677	10-:80547070-80555390
17076	Elavl1	15568	8-:4289601-4311770
17077	MMT00071470		7+:24848728-24850023
17078	Syde2	214804	3+:145650962-145684308
17079	Ssh3	245857	19-:4263906-4269257
17080	Fosl2	14284	5+:32439076-32455560
17081	3830406C13Rik	218734	14+:13116756-13134318
17082	MMT00013155		17+:16984358-16985042
17083	Atp1b1	11931	1-:166368205-166387999

17084	Larp7	28036	3-:127239629-127256267
17085	Sct	20287	7-:148464228-148465005
17086	Olfr780	258281	10+:128758680-128759618
17087	Gulo	268756	14-:66609215-66628059
17088	Fntb	110606	12+:77938494-78021194
17089	Pilrb1	170741	5-:138293374-138299226
17090	Lass2	76893	3+:95119173-95127488
17091	Slc38a3	76257	9-:107553486-107569700
17092	Ttn	22138	2-:76542035-76818252
17093	Opa1	74143	16+:29579427-29652576
17094	Map3k11	26403	19+:5690246-5702343
17095	Slc25a21	217593	12-:57813080-57959100
17096	Csnk1g3	70425	18+:54055203-54115187
17097	MMT00006515		7+:32844338-33390838
17098	Qtrt1	60507	9+:21216346-21224631
17099	Ppp2r3c	59032	12-:56382494-56403976
17100	Scn4b	399548	9+:44957345-44959160
17101	MMT00060041		
17102	Dmrta2	242620	4+:109652467-109656289
17103	Tmem87b	72477	2+:128644074-128679996
17104	MMT00071673		1+:77062575-77063554
17105	Zfpm1	22761	8+:124806040-124861147
17106	Cep192	70799	18+:67988004-67989697
17107	Prdx1	18477	4+:116358216-116372118
17108	Olfr1280	258910	2+:111155637-111156554
17109	C430049B03Rik	72575	X-:50407775-50410447
17110	MMT00063698	751864	3-:15296552-15332303
17111	MMT00058505		12-:115717096-115717662
17112	Dok7	231134	5+:35429196-35430479
17113	1700010M22Rik	66328	2+:144649401-144650150
17114	Myh7	140781	14-:55589524-55613386
17115	2700099C18Rik	77022	17+:95151474-95174477
17116	Tceal6	66104	X-:131743227-131745226
17117	Acrbp	54137	6+:124999992-125013261
17118	Fxyd7	57780	7-:31827533-31836473
17119	Gpr124	78560	8+:28196312-28233890
17120	Wdr12	57750	1-:60134986-60155335
17121	Fcho1	74015	8-:74232551-74249565
17122	MMT00068814		X-:96157424-96162857
17123	Olfr1511	258268	14-:53009489-53010446
17124	Chrm2	243764	6+:35700951-36474608
17125	Nrxn1	18189	17-:90561268-90607840
17126	Oaz1	18245	10+:80289504-80291749
17127	Mcpt4	17227	14-:56645502-56681147
17128	Vmn2r88	669149	14+:52030747-52039201
17129	Dpy19l4	381510	4-:11194759-11204093
17130	Baiap2	108100	11+:119867419-119868093
17131	Nat14	269854	7+:4874492-4876602
17132	E330009J07Rik	243780	6-:40351374-40352945
17133	2700078E11Rik	78832	19-:60604949-60613395
17134	Tssk3	58864	4-:129166263-129167986

17135	Mum1	68114	10+:79697018-79706647
17136	Slc35a4	67843	18+:36839245-36843511
17137	Nudt4	71207	10-:95011728-95026698
17138	Hsf4	26386	8+:107793773-107799747
17139	Olfr921	258778	9+:38582841-38583783
17140	Cd6	12511	19-:10863836-10904498
17141	Il11	16156	7-:4724656-4729627
17142	Cox7a1	12865	7+:30970095-30970985
17143	2010002N04Rik	106878	18-:60633846-60661179
17144	B3gat2	280645	1+:23768590-23852279
17145	Itgam	16409	7+:135270348-135270694
17146	EG217600	217600	12-:58733564-58734002
17147	Ttll12	223723	15-:83406072-83425871
17148	Suclg2	20917	6-:95423816-95668769
17149	Pms2	18861	5+:144670838-144692313
17150	MMT00013160		
17151	9230105E10Rik	319236	7-:111488684-111493648
17152	Scfd2	212986	5-:74602057-74632609
17153	Crnn	381457	3+:92948708-92953730
17154	2610039C10Rik	66578	16-:90719557-90727618
17155	MMT00037407		2+:102014519-102015032
17156	MMT00067284		11-:74069532-74070133
17157	L3mbtl4	320858	17+:69113890-69127710
17158	Zfp113	56314	5-:138584327-138596924
17159	Gtpbp6	107999	5-:110532991-110536454
17160	Ccdc127	67433	13+:74487790-74495122
17161	Lhx3	16871	2-:26056466-26063769
17162	Cux2	13048	5-:122310316-122499860
17163	Fmo1	14261	1-:164759781-164796502
17164	Slc38a10	72055	11-:119989774-120012574
17165	Psmd6	66413	14-:14944701-14953451
17166	Nudt10	102954	X-:5749458-5750045
17167	ENSMUSG00000(	100038358	2-:6052947-6055037
17168	5730494M16Rik	66648	18-:25296717-25327376
17169	4930543E12Rik	75239	7-:120243681-120285537
17170	Nae1	234664	8-:107034928-107058508
17171	MMT00045847		16-:32326267-32326781
17172	Insrr	23920	3+:87600983-87620023
17173	MMT00054186		11+:12548078-12548279
17174	Dmrtc1a	70887	X-:100098560-100104026
17175	MMT00005925	100046973	6-:69725695-69765127
17176	Gnl2	230737	4+:124707281-124732609
17177	Accn2	11419	15+:99502214-99529694
17178	Ifnar2	15976	16+:91373143-91405610
17179	Stfa3	20863	16-:36450622-36455478
17180	Tmem55b	219024	14-:51546888-51550512
17181	Kbtbd10	228003	2+:69508181-69522295
17182	C230021P08Rik	380969	15-:99252465-99258598
17183	Kbtbd3	69149	9+:4309743-4331721
17184	Bsn	12217	9-:108005520-108092589
17185	MMT00007403		8-:19540452-19541169

17186	Reep5	13476	18-:34506647-34532992
17187	Kif20a	19348	18+:34784266-34792458
17188	EG547263	547263	16+:30115265-30139780
17189	Gm263	238333	12+:88541408-88549768
17190	Ndufa5	68202	6-:24468665-24477687
17191	Xylb	102448	9+:119266709-119302447
17192	MMT00028284		7-:48801044-48828948
17193	Npb	208990	11+:120469804-120470413
17194	Cmya5	76469	13-:93810671-93868385
17195	Magea3	17139	X-:151383085-151384048
17196	Fkrp	243853	7-:17394619-17402081
17197	Igtp	16145	11+:58013145-58021086
17198	Sohlh2	74434	3+:54985965-55013872
17199	Apol8	239552	15-:77579810-77585703
17200	Ttll10	330010	4-:155408941-155422086
17201	Tsnaxip1	72236	8+:108351608-108368814
17202	Olfr791	258932	10+:128963284-128964223
17203	Svs3a	64335	2+:164115018-164117225
17204	Cask	12361	X-:13098172-13423682
17205	Tyw3	209584	3-:154242071-154260059
17206	Lyzl4	69032	9-:121486967-121551214
17207	2700050L05Rik	214764	7+:140843845-140863858
17208	Ttn	22138	2-:76691907-76700231
17209	Col18a1	12822	10-:76515783-76629283
17210	MMT00058938		3-:47829508-47830619
17211	OTTMUSG00000(	194227	4+:143379270-143385635
17212	Slc9a2	226999	1+:40738473-40826116
17213	MMT00057554		17-:22321732-22323096
17214	Surf6	20935	2-:26746292-26758316
17215	Mvd	192156	8-:124957503-124967290
17216	Nuf2	66977	1-:171428442-171461547
17217	MMT00027866		1-:130416317-130427667
17218	Cldn18	56492	9-:99591217-99610434
17219	Asnsd1	70396	1-:53380009-53399034
17220	Abhd9	71932	17-:32321605-32326417
17221	Zfp264	116812	7+:6942005-6942686
17222	Usp21	30941	1-:173212332-173217100
17223	4933426M11Rik	217684	12+:81891226-81981791
17224	5430407P10Rik	227545	2-:6021090-6051192
17225	Impa2	114663	18+:67448876-67478495
17226	Utp14a	72554	X+:45610067-45635628
17227	MMT00005015		14+:56780091-56781642
17228	Scgb1a1	22287	19-:9158129-9162418
17229	Olfr68	18369	7-:110925909-110926857
17230	Cmtm2b	75502	8+:106846136-106854656
17231	Cyp2u1	71519	3-:130994047-131006121
17232	Nkx6-1	18096	5-:102088226-102093730
17233	Nmur1	14767	1-:88282900-88284716
17234	Stk33	117229	7-:116422722-116582594
17235	1110001J03Rik	66117	6+:38484867-38489449
17236	MMT00020437		10+:111191928-111205199

17237	Med23	70208	10+:24627094-24633055
17238	MMT00061024		1-:129901996-129903055
17239	Rpl6	19988	5+:121655423-121658974
17240	Gabarap	56486	11+:69805164-69808173
17241	MMT00051399	620963	18+:80209373-80213552
17242	Olah	99035	2-:3259261-3283841
17243	Bcl6b	12029	11-:70037629-70043223
17244	Myef2	17876	2-:124912302-124949398
17245	Fgf12	14167	16-:28161705-28189725
17246	Vmn2r47	100042891	7+:8246138-8617250
17247	A030007L17Rik	68252	6-:54935642-54942862
17248	Rap1gap	110351	4+:137237465-137285777
17249	Vmn2r78	637896	7+:94069358-94103540
17250	Uhrf1bp1	224648	17+:28024415-28027141
17251	Tulp4	68842	17+:6138743-6240621
17252	Vmn2r94	665227	17-:18380534-18394077
17253	RP23-157O10.7	56013	11-:97372982-97436440
17254	Cyp2f2	13107	7+:27904993-27918678
17255	Tm9sf1	74140	14-:56254802-56262643
17256	MMT00046675		3-:93495972-93496781
17257	Ube3a	22215	7+:66484581-66560302
17258	4930448H16Rik	74872	5+:143990225-143996427
17259	Vash1	238328	12+:88021195-88026459
17260	Agtr2	11609	X+:21061743-21065951
17261	MMT00050554		4-:5781664-5782072
17262	Spns3	77577	11-:72311657-72363803
17263	Ccnb3	209091	X-:6557377-6572729
17264	Fzd9	14371	5-:135725120-135726899
17265	MMT00006374	100044772	7+:37019362-37020487
17266	Prl2c3	18812	13-:12883088-12892345
17267	Agbl2	271813	2+:90623374-90653507
17268	MMT00049308		8+:29296474-29297498
17269	Spsb1	74646	4-:149272075-149283840
17270	MMT00018883	100046275	12-:116886148-116886601
17271	MMT00038682		
17272	Dppa4	73693	16+:48283880-48294351
17273	Psmc3	19182	2+:90894238-90899585
17274	Pcnp	76302	16-:56015618-56029801
17275	MMT00079760		X-:74535063-74535993
17276	Cyp4f13	170716	17-:33061632-33084306
17277	Lrrc8d	231549	5-:106133797-106188727
17278	Adrbk2	320129	5-:113342901-113444499
17279	Nupl2	231042	5+:23670820-23688789
17280	Atf2	11909	2-:73656979-73730606
17281	MMT00066360		5-:152238600-152264468
17282	Pdhb	68263	14-:8998850-9005446
17283	Adss	11566	1-:179694269-179726603
17284	D1Bwg0212e	52846	1+:39592633-39603720
17285	1700019E19Rik	76411	12+:87423512-87503406
17286	1700007B13Rik	71830	7-:126630100-126667003
17287	9030411M15Rik	667742	18-:63269177-63317019

17288	Snai3	30927	8-:124978752-124984514
17289	Ppm1f	68606	16+:16896559-16924502
17290	Elmod2	244548	8-:85840215-85856346
17291	Cct8	12469	16-:87484120-87496118
17292	Atp6v1b2	11966	8+:71612682-71637604
17293	Amn1	232566	6-:149106100-149137216
17294	Krtap5-5	114666	7+:149415323-149415834
17295	MMT00082577		2+:137221009-137221079
17296	Mtm1	17772	X+:68468256-68568440
17297	Comtd1	69156	14-:22666277-22668156
17298	Stk36	269209	1+:74648054-74681330
17299	Fzd8	14370	18+:9212917-9214974
17300	Olfr187	258319	16-:59035634-59036561
17301	Amdhd2	245847	17-:24299939-24300750
17302	Dhcr24	74754	4+:106233754-106259391
17303	C630007B19Rik	320265	6+:96063236-96081942
17304	Cdc23	52563	18-:34792365-34811389
17305	Itm2a	16431	X-:104592534-104598699
17306	Fgd5	232237	6+:91999909-92012898
17307	Gpr63	81006	4+:24934424-24935701
17308	Cpn1	93721	19-:44030799-44061010
17309	MMT00052961		9-:101350626-101351672
17310	MMT00068323		10-:47044924-47045517
17311	Ddx19b	234733	8-:113527085-113528460
17312	Sirpa	19261	2+:129419057-129456668
17313	Slc17a5	235504	9-:78384311-78426514
17314	Napa	108124	7+:16683991-16703324
17315	Nhsl1	215819	10+:18244416-18253687
17316	Nkx2-1	21869	12-:57634022-57636047
17317	Jmjd1a	104263	6-:71538962-71582732
17318	Igh	111507	12-:115202578-115203083
17319	Lsm1	67207	8+:26895781-26913010
17320	Olfr73	117004	2-:87874354-87875294
17321	Hepacam	72927	9+:37175187-37192680
17322	Zfp758	224598	17+:22498488-22514247
17323	Tubb6	67951	18+:67550372-67562402
17324	MMT00023460		1-:49060930-49061049
17325	4933400A11Rik	66747	X-:166214309-166217567
17326	Zranb3	226409	1-:129850761-129937582
17327	MMT00004644	626904	6-:25926812-25927081
17328	H2afv	77605	11-:6328105-6344429
17329	MMT00035807		6-:140005316-140006121
17330	Frmpd4	333605	X-:163911906-164048964
17331	Nsun4	72181	4-:115705446-115726481
17332	Smc3	13006	19+:53702562-53719636
17333	Slc25a10	27376	11+:120353185-120360479
17334	Spag4l	76407	2-:153681920-153693375
17335	Clcn1	12723	6+:42236765-42264176
17336	Cdadc1	71891	14-:60182784-60216793
17337	1810065E05Rik	69864	11+:58234612-58239525
17338	Mocos	68591	18+:24811986-24860074

17339	Acaa1b	235674	9-:119057161-119066124
17340	MMT00014772		19+:32490002-32498118
17341	Batf	53314	12+:87027669-87050036
17342	Dhx32	101437	7-:140912728-140968486
17343	Timm8a1	30058	X-:131071797-131076168
17344	Lonrf2	381338	1-:38853305-38868671
17345	4930519N13Rik	78177	2-:150761016-150805927
17346	Liph	239759	16-:21954350-21995556
17347	MMT00005275		18+:13530594-13531052
17348	Pf4	56744	5+:91201538-91202409
17349	Ralyl	76897	3+:13723415-13814618
17350	Tbl1xr1	81004	3+:22047863-22109461
17351	Rtkn2	170799	10+:67504148-67505262
17352	Cebpb	12608	2+:167514414-167515920
17353	Gtf3c6	67371	10-:39968534-39977470
17354	Tcn2	21452	11-:3817195-3831586
17355	Ramp2	54409	11+:101107744-101109559
17356	Rgnef	110596	13-:98669423-98976122
17357	1810059H22Rik	69820	5-:149765323-149766779
17358	MMT00012966		5+:85359500-85359944
17359	Tspan12	269831	6-:21721392-21808113
17360	Ppm1j	71887	3+:104584004-104588937
17361	Dhps	330817	8+:87595375-87603773
17362	Ankrd34a	545554	3+:96401404-96402805
17363	Aebp2	11569	6+:140572746-140626992
17364	Mef2c	17260	13+:83643193-83802948
17365	Isl2	104360	9+:55389013-55393986
17366	MMT00069569		7-:147073369-147074261
17367	MMT00064436		5+:110295640-110295832
17368	1700123M08Rik	76665	4+:11893297-11921427
17369	BC033915	70661	9+:45931319-46010968
17370	Cdh12	215654	15+:21409980-21516236
17371	Emx2	13797	19+:59533890-59538927
17372	MMT00017409		16+:4864247-4867691
17373	EG433016	433016	16+:36184292-36188194
17374	Uchl5	56207	1+:145624464-145653945
17375	Nlrp4c	83564	7+:6017316-6056750
17376	MMT00032526		15+:71624995-71626176
17377	Olfr1504	258627	19-:13952162-13972708
17378	3110062M04Rik	78412	6-:34824335-34825637
17379	MMT00053584	545867	6-:95982853-95983369
17380	Slc25a38	208638	9+:120019516-120033435
17381	Star	20845	8+:26918945-26925195
17382	MMT00001167		X+:155271464-155272250
17383	Pah	18478	10+:86984718-87046876
17384	Trim27	19720	13+:21272240-21284726
17385	Asah3	171168	17-:57094502-57121531
17386	Vps16	80743	2+:130266049-130269468
17387	Fastkd2	75619	1+:63777133-63799038
17388	MMT00067636		
17389	Pop4	66161	7-:39047838-39056367

17390	Nme6	54369	9+:109735704-109745132
17391	C530028O21Rik	319352	6+:124946835-124953116
17392	MMT00047040		10-:39472168-39472692
17393	MMT00063804		10-:35428509-35431626
17394	E130319B15Rik	209645	2+:4639202-4721960
17395	MMT00035275		15-:27988455-27988652
17396	Myoz3	170947	18-:60735943-60751442
17397	MMT00047810		2+:136091275-136225438
17398	MMT00045090		5-:117082489-117083618
17399	4933416C03Rik	619332	10-:115549544-115550675
17400	MMT00050193		1+:119502965-119523538
17401	Ptgfr	19220	3-:151463748-151500411
17402	Capza2	12343	6+:17587014-17616167
17403	N4bp1	80750	8-:89368220-89385212
17404	Ctsb	13030	14+:63741301-63764758
17405	Spata6	67946	4+:111392610-111501039
17406	4933428G09Rik	66768	5+:48763582-48779586
17407	Usp49	224836	17-:47820212-47820811
17408	Iqwd1	74106	1-:167259634-167390422
17409	A630055G03Rik	223970	16+:10835182-10840070
17410	1700021F07Rik	72221	2+:173348114-173353996
17411	Fert2	14158	17+:64287320-64488545
17412	Aif1	11629	17-:35307939-35309537
17413	MMT00043948		
17414	MMT00059213	672682	17+:35755624-35760052
17415	Sema5b	20357	16+:35541656-35663787
17416	Col28a1	213945	6-:7949606-7965044
17417	Pxn	19303	5+:115956760-116004666
17418	D1Ertd622e	52392	1-:99540485-99558542
17419	EG432555	432555	11-:48707836-48715515
17420	Vps13b	666173	15+:35301294-35382022
17421	MMT00002048		2-:73797668-73798769
17422	Dctd	320685	8+:49195522-49227013
17423	Gna13	14674	11+:109224159-109258960
17424	Akt1	11651	12-:113892031-113912401
17425	MMT00007116		12-:32579491-32579840
17426	Arid2	77044	15+:96117952-96235727
17427	V1rc16	171189	6-:57310461-57311372
17428	Hapln2	73940	3-:87825984-87831362
17429	Tpi1	21991	6-:124760733-124764430
17430	B4galt2	53418	4-:117545736-117554423
17431	Foxc2	14234	8+:123640482-123642790
17432	Pafah1b2	18475	9-:45776641-45792880
17433	4932432K03Rik	74385	14+:49686169-49707032
17434	Eda2r	245527	X-:94534165-94566595
17435	2410003L11Rik	69729	11+:97476237-97479813
17436	Taf8	63856	17-:47625234-47639236
17437	Il13ra1	16164	X+:33652171-33709238
17438	Gnas	14683	2+:174109831-174172241
17439	Igsf9	93842	1+:174414619-174428668
17440	Tex18	83559	10+:86376243-86377425

17441	Zfp335	329559	2-:164717001-164737525
17442	Zfp318	57908	17+:46523688-46557865
17443	Tas1r2	83770	4+:139209452-139226192
17444	Tuba1a	22142	15-:98762061-98781118
17445	Anp32a	11737	9+:62189290-62225632
17446	RP23-195K8.6	622404	4+:43506238-43508798
17447	Ctsf	56464	19+:4855128-4860912
17448	MMT00038194		5+:13811208-13814806
17449	Zfp296	63872	7+:20163164-20166004
17450	MMT00047013	619941	2-:91363426-91364026
17451	F12	58992	13-:55519350-55528141
17452	Tmco3	234076	8+:13287950-13321492
17453	Foxp1	108655	6-:98880084-99113012
17454	Klhl6	239743	16-:19946588-19983106
17455	Lpcat1	210992	13+:73626526-73652560
17456	Lgtn	16865	1+:133049768-133070072
17457	MMT00026898		19+:28107984-28108635
17458	Msr1	20288	8-:40690543-40718181
17459	Wnt9a	216795	11+:59120467-59144878
17460	Dpyd	99586	3+:118265022-118647253
17461	Ripk5	213452	1+:134313989-134363536
17462	Nat8l	269642	5+:34339739-34344192
17463	Rgl1	19731	1-:154365952-154472285
17464	MMT00024460		2+:53410828-53411919
17465	Ccin	442829	4+:43994444-43998231
17466	Fbxl18	231863	5-:143633488-143656876
17467	Kif13b	16554	14+:65271350-65425131
17468	Anubl1	67492	6+:116216546-116280316
17469	Sec14l3	380683	11+:3967951-3979089
17470	Zfp784	654801	7-:4985678-4990047
17471	Defb19	246700	2-:152401824-152406033
17472	Cd53	12508	3-:106562851-106592970
17473	4930500O09Rik	75811	4-:86090602-86204233
17474	MMT00055944		8+:67461856-67467919
17475	MMT00043153		13-:81225550-81226433
17476	Ube2d2	56550	18+:35931260-35966815
17477	Hook3	320191	8-:27141763-27229620
17478	Ptpn7	320139	1+:137029885-137039830
17479	E030049G20Rik	210356	1-:127999120-128020644
17480	Ppp1r2	66849	16-:31253317-31275310
17481	Defb21	403172	2+:152398478-152400679
17482	Hdlbp	110611	1-:95304344-95375381
17483	Rab8b	235442	9-:66691423-66767461
17484	Ercc6	319955	14+:33330142-33390247
17485	Ndufab1	70316	7-:129230349-129245360
17486	Cnn2	12798	10+:79451378-79458135
17487	Mcm7	17220	5-:138605816-138613090
17488	Gsdmc4	74548	15-:63722735-63734491
17489	Cd79a	12518	7+:25682531-25686876
17490	Mras	17532	9-:99288348-99312022
17491	4930435E12Rik	74663	16-:38824441-38828858

17492	MMT00078159		14+:54210578-54260976
17493	Rfx4	71137	10+:84218807-84369279
17494	Lyrm7	75530	11-:54640369-54674419
17495	Mmaa	109136	8-:81791806-81805547
17496	9030612M13Rik	208292	17-:32911296-32924882
17497	Ptges	64292	2-:30747782-30758782
17498	MMT00008723		13+:66699587-66699622
17499	Sirt6	50721	10-:81084534-81090395
17500	Atad1	67979	19-:32747312-32776024
17501	EG385499	385499	X-:91853446-91854598
17502	Gabra4	14397	5-:71961628-72049591
17503	Cnnm2	94219	19+:46836786-46953069
17504	Nars	70223	18-:64659301-64676200
17505	Igh-VJ558	16061	12-:115942513-115942803
17506	Isy1	57905	6-:87768688-87788717
17507	Olfr174	259004	16-:58823607-58824533
17508	MMT00023781		18-:16997814-16998436
17509	Gfi1	14581	5-:108145678-108153363
17510	Stk40	74178	4+:125806958-125818268
17511	MMT00000525		4+:146318109-146319184
17512	EG433216	433216	19+:6152665-6153711
17513	Rin1	225870	19+:5050807-5057070
17514	Thbd	21824	2-:148230206-148233839
17515	Itln1	16429	1-:173460625-173461346
17516	1520402A15Rik	68075	4-:115809333-115817223
17517	Kcnh1	16510	1+:194014775-194330925
17518	Rpl18	19899	7+:52973487-52976197
17519	MMT00061180		9-:39493613-39494527
17520	EG668457	668457	3+:95426160-95426733
17521	Foxn2	14236	17+:88840078-88886897
17522	Sufu	24069	19+:46471447-46560499
17523	Gdf5	14563	2-:155766762-155771103
17524	MMT00006341		1+:150823157-150826053
17525	Ctgf	14219	10+:24315312-24318486
17526	Olfr539	258963	7+:147853187-147854145
17527	2310033K02Rik	69553	2-:167805663-167819135
17528	Gmnn	57441	13-:24843714-24853724
17529	Tnfrsf13c	72049	15-:82037384-82043239
17530	MMT00041792		8-:38463277-38464078
17531	OTTMUSG00000(	668929	2-:151471140-151481075
17532	Tacr3	21338	3+:134492236-134595404
17533	Srd5a1	78925	13-:69712325-69751872
17534	E230019M04Rik	331537	X+:136605670-136636249
17535	MMT00008110		18-:88826941-88828108
17536	Chn2	69993	6+:54144018-54251801
17537	Piga	18700	X+:160857749-160861155
17538	2210012G02Rik	66526	4-:106806766-106807826
17539	Olfr1365	404335	13-:21646910-21647889
17540	Ywhaz	22631	15-:36701459-36723274
17541	Fgf22	67112	10+:79217863-79219705
17542	MMT00044805	240219	18-:38519096-38520141

17543	Olfr1023	258580	2+:85726958-85727892
17544	Col4a6	94216	X-:137600570-137908619
17545	Pdha2	18598	3-:140872967-140875319
17546	Wdsub1	72137	2-:59690422-59720642
17547	C730007P19Rik	20865	7+:14318888-14364987
17548	Pogz	229584	3+:94657868-94686244
17549	Exoc6b	75914	6-:84696893-84858283
17550	Exoc8	102058	8-:127418693-127421526
17551	Satl1	73809	X-:109497914-109520365
17552	Pdzd4	245469	X-:71039556-71069805
17553	Eif4ebp1	13685	8+:28370830-28385968
17554	N28178	230085	4+:42946491-42953377
17555	Tspan5	56224	3+:138405171-138567388
17556	Dcbld1	66686	10+:51953424-52040081
17557	Smchd1	74355	17-:71693835-71740775
17558	Pole3	59001	4-:62184966-62186048
17559	Olfr920	258783	9+:38563274-38564205
17560	Tagln3	56370	16-:45711492-45724477
17561	Slc39a13	68427	2-:90901946-90910475
17562	Rbed1	232089	6-:72515918-72544805
17563	Aldh1l1	107747	6+:90507191-90521989
17564	MMT00054039		8+:41023626-41025793
17565	Zfp185	22673	X+:70242372-70275500
17566	Olfr645	258247	7-:111232645-111233465
17567	Supt16h	114741	14-:52780093-52816876
17568	Rexo2	104444	9-:48276618-48288716
17569	1810058I24Rik	67705	6+:35202686-35211982
17570	2810433D01Rik	77132	11-:102481108-102486034
17571	MMT00002547		6-:15749586-15814504
17572	Krt80	74127	15-:101178753-101195009
17573	Tnfrsf1b	21938	4-:144805681-144836790
17574	Prdm16	70673	4-:153694400-153741421
17575	C330001K17Rik	77609	9-:21799256-21807078
17576	Meis3	17537	7+:16760778-16771848
17577	Med28	66999	5+:45911539-45916838
17578	Kif19a	286942	11+:114649223-114652050
17579	MMT00078068		19+:44925782-44926468
17580	Ccdc3	74186	2+:5058976-5151910
17581	Pisd	320951	5-:33078969-33128276
17582	Plcxd2	433022	16-:45964984-46009888
17583	Per2	18627	1-:93312560-93355873
17584	F11r	16456	1+:173367701-173394724
17585	Gramd1a	52857	7-:31915145-31936052
17586	Gata4	14463	14-:63818911-63864061
17587	Tcl1	21432	12-:106454996-106460923
17588	Bcl7b	12054	5+:135644361-135657715
17589	Madd	228355	2-:91017805-91023994
17590	MMT00047848		2-:140781633-140781973
17591	D930015E06Rik	229473	3-:83701577-83742090
17592	Man1a	17155	10-:53626819-53794951
17593	Gfer	11692	17-:24831131-24832881

17594	MMT00040814		5+:87426114-87427458
17595	MMT00048325		3+:132505358-132507882
17596	MMT00032256		
17597	Abcf1	224742	17-:36093584-36106750
17598	Olfr458	258436	6-:42410074-42411016
17599	D3Ertd254e	241944	3+:36049999-36065842
17600	Kcnq5	226922	1-:21391688-21503216
17601	Msx2	17702	13-:53563255-53568443
17602	Trim54	58522	5+:31419084-31439998
17603	4921509O07Rik	70917	13-:114236495-114274865
17604	C77370	245555	X-:101278529-101283015
17605	Usp14	59025	18-:9996096-10029926
17606	Tmem90a	627191	12-:86019666-86039563
17607	Orc6l	56452	8+:87823689-87831969
17608	Shd	20420	17+:56109905-56116040
17609	Ptpn9	56294	9+:56843305-56910609
17610	Zfp131	72465	13_random+:31981-57600
17611	Sec14l5	665119	16+:5165681-5184277
17612	Vhlh	22346	6+:113574005-113580264
17613	P2rx7	18439	5+:123094082-123131313
17614	MMT00071219		8-:74555571-74556774
17615	Exph5	320051	9+:53109837-53185607
17616	Flt1	14254	5-:148373771-148537336
17617	Atp5sl	66349	7+:26404452-26410569
17618	Zfp458	238690	13-:67357052-67585153
17619	Zfp551	619331	7-:13000499-13191900
17620	Macf1	11426	4-:123026843-123231609
17621	D930001I22Rik	228859	2-:163292042-163298441
17622	Foxo1	56458	3+:52072339-52151010
17623	Olfr715	258776	7-:114271958-114272905
17624	Ccdc65	105833	15+:98538643-98553733
17625	Olfr441	258649	6+:43065940-43066674
17626	Ryr1	20190	7-:29809011-29910146
17627	Shisa4	77552	1-:137267637-137271659
17628	Lrrc16a	68732	13-:24270073-24372642
17629	Saa2	20209	7+:54007202-54009682
17630	Fbxo40	207215	16-:36966159-36990301
17631	Pla2r1	18779	2-:60257095-60391318
17632	Lypd3	72434	7+:25421588-25426135
17633	Tmem79	71913	3-:88133641-88137561
17634	Dner	227325	1-:84472916-84582195
17635	Mmp10	17384	9+:7502360-7510238
17636	MMT00039631		6-:66791341-66874170
17637	Olfr915	258781	9-:38454174-38455107
17638	Adssl1	11565	12+:113858299-113879550
17639	Ell3	269344	2-:121264713-121268263
17640	Tomm70a	28185	16+:57121826-57153143
17641	Mapk7	23939	11-:61302314-61307705
17642	Srl	106393	16-:4482004-4492391
17643	6030426L16Rik	628850	12-:21678265-21840135
17644	Dse	212898	10-:33871196-33927092

17645	Atp5c1	11949	2-:9981126-9981889
17646	C4b	12268	17-:34865331-34880828
17647	MMT00074110		13+:22556164-22559725
17648	Scrg1	20284	8+:59934719-59956382
17649	Esx1	13984	X-:133649936-133654710
17650	Scgb1c1	338417	7+:148031470-148032667
17651	Commd1	17846	11-:22799763-22878996
17652	Cd36	12491	5-:17288303-17341535
17653	MMT00039948		13-:66650716-66651678
17654	Ppm1e	320472	11-:87044348-87062620
17655	MMT00075835		16-:38685246-38686069
17656	Hdhd3	72748	4-:62160087-62163234
17657	Lrfn1	80749	7+:29239514-29247143
17658	MMT00034425		1+:43944818-43945851
17659	MMT00017514		
17660	Nlrp12	378425	7-:3221915-3249524
17661	Birc5	11799	11+:117710564-117717046
17662	Ankrd52	237615	10+:127814197-127827681
17663	Arhgef5	54324	6+:43215712-43222874
17664	Jup	16480	11-:100231264-100259322
17665	2310056P07Rik	70186	16-:36043929-36071601
17666	Fstl1	14314	16+:37777159-37835832
17667	Phactr1	218194	13+:43230752-43233948
17668	6430527G18Rik	238330	12-:88221650-88225761
17669	2310007A19Rik	66353	3-:94268904-94277513
17670	MMT00021061		14-:72849453-72852690
17671	Klk1b1	16623	7+:51222156-51226642
17672	EG237412	237412	10+:81816993-81842187
17673	Nola1	68147	3-:129527837-129534074
17674	MMT00021031		14-:72825819-72831611
17675	Txndc10	67988	18+:90679547-90712787
17676	Amz1	231842	5+:141217237-141229240
17677	MMT00040539		6+:76234406-76368044
17678	4933427G23Rik	330053	5-:23337006-23337441
17679	Col3a1	12825	1+:45368474-45413525
17680	1700069L16Rik	73476	5-:114150351-114174783
17681	Bcl2	12043	1-:108439841-108609457
17682	Zfp386	56220	12+:117285986-117298232
17683	MMT00010505	654450	6-:130002975-130013208
17684	4930572J10Rik	75884	18-:43198204-43330863
17685	Eif6	16418	2-:155645571-155652660
17686	Fstl3	83554	10+:79240039-79245067
17687	Cdv3	321022	9-:103255432-103268001
17688	Znhit4	70020	6-:83071823-83075023
17689	4933405O20Rik	243996	7+:57698992-57855779
17690	Mtap7d3	320923	X-:54051158-54054369
17691	Kank3	80880	17+:33947467-33959859
17692	Smc6	67241	12+:11306211-11324901
17693	MMT00044427		X-:115253343-115253599
17694	Ppp1r14b	18938	19+:7049475-7051812
17695	Rec8	56739	14+:56237072-56244226

17696	MMT00065923		X-:88791878-88792718
17697	1300014I06Rik	66895	13-:34719709-34744550
17698	Wwox	80707	8+:116963600-117876605
17699	Ccdc43	52715	11-:102546001-102559039
17700	Crim2	333088	6-:29424269-29447828
17701	Spink6	433180	18+:44231047-44243264
17702	Ccdc40	207607	11+:119099177-119125828
17703	Large	16795	8-:75338497-75876455
17704	EG620480	620480	7-:91983050-91995915
17705	Rps7	20115	12-:29315965-29320790
17706	Kirrel3	67703	9+:34719150-34843889
17707	Rshl2b	100037282	17-:7109064-7152705
17708	Reg2	19693	6+:78355148-78358091
17709	Gnai1	14677	5-:17770951-17866446
17710	Calcb	116903	7+:121862150-121866737
17711	EG434609	434609	12-:117218706-117219395
17712	Nup62	18226	7+:52071789-52086174
17713	B4galt6	56386	18-:20844503-20904905
17714	Ckap5	75786	2+:91366948-91460819
17715	Ltbp2	16997	12-:86124162-86217482
17716	Rnf10	50849	5-:115691779-115722903
17717	Klhl15	236904	X+:91480675-91518865
17718	2010109I03Rik	67038	15-:74708771-74712134
17719	Gm1006	381399	2+:153768456-153789588
17720	Rap2b	74012	3+:61168522-61170953
17721	MMT00077153		3-:141846870-141847784
17722	MMT00014461		15-:59825986-59826492
17723	Hsh2d	209488	8+:74713565-74724678
17724	Sdf4	20318	4+:155367019-155384317
17725	Trim12	76681	7-:111452110-111455902
17726	Slc25a13	50799	6-:5991218-6167118
17727	MMT00039356		
17728	Cyp2b19	13090	7+:27542205-27557648
17729	Prim2	19076	1-:33510655-33726603
17730	MMT00058199		10+:20345591-20348727
17731	MMT00005424		11+:33072883-33073368
17732	Cyp2c65	72303	19+:39135519-39168436
17733	Cdx1	12590	18-:61179379-61195548
17734	Ghsr	208188	3+:27270718-27273843
17735	Supt5h	20924	7-:29099916-29123738
17736	Maml3	433586	3-:51493356-51501671
17737	MMT00020869		14-:73378022-73379845
17738	Acad8	66948	9-:26781719-26807128
17739	Zzef1	195018	11+:72609798-72637776
17740	Rpl36	54217	17+:56753036-56753657
17741	Bnip2	12175	9+:69837421-69856018
17742	Dscaml1	114873	9+:45469215-45561793
17743	Phb2	12034	6+:124662353-124666936
17744	2310028H24Rik	71901	4-:41466317-41516544
17745	MMT00035996		
17746	Prlr	19116	15+:10153740-10276776

17747	MMT00071421		7-:144359766-144359864
17748	Olfr878	258794	9+:37726228-37727172
17749	Btbd6	399566	12+:114214775-114217219
17750	Hs6st2	50786	X-:49033065-49034779
17751	Olfr1201	258897	2+:88634540-88635463
17752	Chac1	69065	2+:119177025-119180058
17753	RP23-25C1.8	625638	4-:137951914-137953615
17754	6720489N17Rik	211378	13-:62705589-62723192
17755	BC117090	100038854	16-:36321728-36367708
17756	Morc1	17450	16+:48431349-48631018
17757	Hnrnph2	56258	X+:131139443-131141593
17758	Epdr1	105298	13-:19684929-19711582
17759	EG433862	433862	5+:28186988-28187678
17760	Ccr6	12458	17+:8437944-8449985
17761	4930564B18Rik	75272	14+:76042063-76066945
17762	Tph1	21990	7-:53902500-53922718
17763	Selplg	20345	5-:114268558-114270259
17764	Ccdc128	73825	17+:88978069-88987705
17765	Uck1	22245	2-:32110041-32115653
17766	Gad1	14415	2+:70400352-70440069
17767	1700091H14Rik	73553	14-:42668556-42674084
17768	MMT00023753		14+:25104332-25105492
17769	Cyp19a1	13075	9-:54013743-54041243
17770	Ttbk1	106763	17-:46581974-46621167
17771	Htr6	15565	4-:138617324-138630704
17772	Flot2	14252	11+:77853805-77873933
17773	Rfng	19719	11-:120642760-120645516
17774	Ints1	68510	5-:140227236-140251601
17775	MMT00025230		1-:53453188-53622529
17776	BC043118	224273	16-:59491914-59556610
17777	Mtap7	17761	10+:19868819-20000511
17778	Sprr1a	20753	3-:92287876-92289796
17779	D6Wsu116e	28006	6+:116158058-116212688
17780	Slc38a5	209837	X+:7848556-7857299
17781	EG625796	625796	15+:31206235-31207592
17782	5730507A09Rik	70638	7-:71903397-71931683
17783	D630012G11Rik	320904	12-:74303610-74304276
17784	Lama5	16776	2-:179905969-179960558
17785	Grin2a	14811	16-:9577802-9612850
17786	Cntn2	21367	1-:134413575-134417857
17787	MMT00069688		8-:108109141-108110131
17788	Xrcc1	22594	7+:25332201-25358453
17789	Cnga4	233649	7+:112553402-112557251
17790	Olfr870	57251	9-:19975078-19976013
17791	Cdkl5	382253	X-:157246406-157341866
17792	Vmn2r79	621430	7+:94150477-94186337
17793	MMT00000967		6+:56896903-56897202
17794	2010203O07Rik	72429	4+:59030533-59036271
17795	Pglyrp3	242100	3+:91800353-91800990
17796	Rims2	116838	15+:39029877-39513486
17797	Rltpr	234695	8+:108214291-108222099

17798	Tmc2	192140	2+:130020929-130090181
17799	Nsmaf	18201	4-:6323372-6381408
17800	Lrtm1	319476	14+:29831386-29841486
17801	Syt12	171180	19-:4447688-4467940
17802	Gpd2	14571	2+:57090856-57220006
17803	1810049H19Rik	435889	6-:41261231-41264710
17804	Npffr2	104443	5+:89996840-90012489
17805	Slc39a12	277468	2+:14317698-14416581
17806	Gpsm3	106512	17+:34727160-34728698
17807	Pebp1	23980	5-:117732662-117737555
17808	Ncor2	20602	5-:125497532-125659423
17809	Tdrd9	74691	12+:113236017-113263949
17810	MMT00054025		13+:75940136-75940731
17811	D10Ertd761e	52706	10+:81685763-81686514
17812	Rnf6	74132	5-:147020779-147033013
17813	Vmn2r16	384220	5+:109759399-109793299
17814	Napb	17957	2-:148520513-148521861
17815	Xpnpep2	170745	X+:45462051-45490158
17816	3110003A17Rik	73112	10-:17731065-17743041
17817	Ccnd2	12444	6-:127080252-127101046
17818	Chchd4	72170	6-:91414270-91423417
17819	C030006K11Rik	223665	15-:76551895-76554249
17820	Cpd	12874	11-:76595490-76660498
17821	MMT00005082		9_random-:59600-76418
17822	MMT00043095		13-:19396516-19429512
17823	D2Ertd750e	51944	2+:118639738-118660375
17824	Tmem138	72982	19-:10645374-10651587
17825	Nudt18	213484	14+:70977709-70980333
17826	3300001G02Rik	78372	11+:32105414-32108984
17827	Klhl13	67455	X-:22796396-22942179
17828	Serpina3n	20716	12+:105644968-105652535
17829	Otx2	18424	14-:49277860-49282547
17830	Lrrc40	67144	3+:157711393-157730053
17831	Ndufa6	67130	15-:82180592-82184667
17832	MMT00043412		1-:93068006-93068484
17833	Nckipsd	80987	9+:108710746-108720698
17834	Hemgn	93966	4-:46406861-46417055
17835	BC037112	231128	5+:34753300-34829170
17836	Ermn	77767	2-:57898950-57905274
17837	Polr3gl	69870	3-:96381796-96398066
17838	Apoc3	11814	9-:46041306-46042796
17839	Tmem132e	270893	11+:82202400-82259828
17840	1110029L17Rik	68722	15-:72926496-72926919
17841	Cdipt	52858	7+:134119910-134124014
17842	Pcdh8	18530	14-:80166598-80171040
17843	Stxbp5	78808	10-:9479736-9620772
17844	Centb1	216859	11-:69694719-69709039
17845	Erc1	111173	6-:119525263-119798150
17846	Wbp11	60321	6-:136762177-136769784
17847	Tmem16g	404545	1+:95286871-95299337
17848	Il17b	56069	18+:61847588-61852190

17849	Tfip11	54723	5+:112755388-112767091
17850	Prg4	96875	1-:152296998-152313295
17851	Card11	108723	5-:141348946-141476537
17852	Cpeb2	231207	5+:43635824-43677428
17853	Olfr1353	259044	10+:78432395-78433323
17854	Rdh11	17252	12-:80275323-80293280
17855	Slc4a3	20536	1+:75542872-75555654
17856	Tmem1	216131	10-:77659026-77707334
17857	C1qtnf7	109323	5+:44000239-44007888
17858	MMT00053971		6-:87898427-87899445
17859	4922505G16Rik	629499	2-:157034281-157049031
17860	Zcchc6	214290	13-:59872912-59923471
17861	Psen1	19164	12+:85029512-85076148
17862	Fbxo21	231670	5+:118426741-118458695
17863	MMT00025642	100038927	8+:22274359-22274508
17864	D930015E06Rik	229473	3-:83744418-83844086
17865	Txlnb	378431	10+:17516085-17565469
17866	Zmat5	67178	11+:4604680-4637669
17867	Stard13	243362	5-:151840205-151992769
17868	Zfp316	54201	5-:144013804-144017885
17869	Foxn4	116810	5-:114705500-114713163
17870	Ret	19713	6-:118103872-118147151
17871	Scamp3	24045	3+:88981499-88986685
17872	Akap10	56697	11-:61686344-61728927
17873	Vrk3	101568	7+:52003998-52032882
17874	Atp6v1h	108664	1+:5073252-5152526
17875	Cntnap5a	636808	1+:117956698-118189112
17876	Tubb4	22153	17-:57220114-57226936
17877	Aptx	66408	4-:40633274-40650213
17878	Brap	72399	5+:122113363-122135606
17879	MMT00069486		14+:70702013-70702703
17880	Ncald	52589	15-:37296018-37389098
17881	Mtg1	212508	7+:147323528-147336505
17882	Ddx26b	236790	X+:53708022-53761019
17883	D730040F13Rik	242474	4-:56891192-56960298
17884	Lnx2	140887	5-:147828679-147888105
17885	Tasp1	75812	2-:139772892-139892541
17886	Foxl1	14241	8+:123651861-123652870
17887	Cryl1	68631	14-:57893871-58017320
17888	MMT00075624	70772	17+:27166212-27173320
17889	Bbox1	170442	2-:110105240-110145882
17890	2610021K21Rik	78767	12-:101092802-101122606
17891	Olfr619	259080	7+:110752169-110753131
17892	MMT00037578		19-:15746724-15747278
17893	Nr6a1	14536	2-:38582433-38781940
17894	Rps21	66481	2+:179992118-179993141
17895	Smcp	17235	3-:92387805-92392919
17896	Ttc9b	73032	7+:28438940-28441231
17897	Gm1019	368202	3-:85798002-85804466
17898	4930578I06Rik	67750	14-:64589917-64606630
17899	BC016423	105203	13-:3584209-3589209

17900	Ift81	12589	5-:123000510-123064527
17901	Krtap16-5	77918	16-:88877767-88878235
17902	Slc35d2	70484	13-:64197616-64230615
17903	MMT00073283	103172	10+:75398318-75400491
17904	Cds1	74596	5+:102194391-102252878
17905	Nog	18121	11-:89162635-89163334
17906	Mdn1	100019	4+:32809155-32810076
17907	Suz12	52615	11+:79806997-79847623
17908	Tbcel	272589	9-:42223640-42269464
17909	Mesdc1	80889	7-:91029558-91032829
17910	Ints2	70422	11-:86025607-86071061
17911	MMT00047239		6+:83981563-83982264
17912	Mpo	17523	11+:87607085-87617895
17913	Phtf2	68770	5-:20267481-20387897
17914	BC052484	328783	17+:25883718-25885274
17915	V1rh3	171246	13+:22486897-22487836
17916	Arl10	56795	13+:54676425-54682488
17917	Ctsq	104002	13-:61136397-61139548
17918	Nxn	18230	11-:76212201-76212622
17919	6330403A02Rik	381310	1-:182368446-182413633
17920	Nlgn2	216856	11-:69636624-69694564
17921	Lars2	102436	9+:123276052-123371785
17922	Reg3a	19694	6+:78330702-78333833
17923	Muc20	224116	16-:32786129-32797494
17924	Alox8	11688	11-:68997386-69011344
17925	F13b	14060	1+:141398303-141420333
17926	Nptx1	18164	11-:119400685-119409139
17927	MMT00042294	434130	7+:17579726-17581992
17928	Pex11a	18631	7-:86882149-86887911
17929	Atg4d	235040	9+:21076491-21078479
17930	Cidec	14311	6-:113374624-113385754
17931	Chrna1	11435	2-:73403855-73418367
17932	Gosr1	53334	11-:76543199-76577076
17933	Npsr1	319239	9+:24094240-24118633
17934	MMT00038301		18+:12447105-12447977
17935	Pde6c	110855	19+:38207435-38258227
17936	Adamts12	239337	15+:11001253-11266209
17937	MMT00069919		5+:139051966-139052547
17938	Kcnd2	16508	6+:21664128-21679813
17939	MMT00075513		15+:63463251-63592135
17940	Zfp706	68036	15-:36930383-36936956
17941	Ror2	26564	13-:53205589-53381246
17942	Msn	17698	X+:93337224-93362640
17943	D330001F17Rik	223658	15+:76220456-76283466
17944	Zfp558	72230	9-:18260018-18282710
17945	Cog1	16834	11+:113510859-113523714
17946	Tmprss11a	194597	5-:86840646-86860936
17947	MMT00008227		18-:88583938-88584464
17948	MMT00026476		5+:98582278-98583193
17949	Htr3a	15561	9-:48707318-48719141
17950	Heatr5b	320473	17-:79152198-79234705

17951	4833436C18Rik	329831	4-:43439883-43441361
17952	Ripk2	192656	4-:16050702-16090543
17953	Ros1	19886	10-:51765604-51914883
17954	Mboat7	77582	7-:3629861-3644537
17955	MMT00038380		11-:9637155-9637430
17956	Serpina1d	20703	12-:105001808-105011784
17957	1700086L19Rik	74284	12+:75386502-75397160
17958	Guca2b	14916	4-:119329221-119331550
17959	MMT00033462		1-:181855051-181855578
17960	Yod1	226418	1+:132612799-132616173
17961	Ube2a	22209	X+:34414352-34424210
17962	Grm5	108071	7+:95123644-95281094
17963	EG433319	433319	1+:72050829-72051390
17964	Asb11	68854	X+:160876032-160897032
17965	Alpl	11647	4-:137297653-137352238
17966	2310021H06Rik	67135	2+:153988343-153994179
17967	B3galnt2	97884	13+:14046915-14090446
17968	8230402K04Rik	268807	15-:58146130-58154887
17969	BC030336	233812	7+:127821304-127877771
17970	EG638532	638532	1+:134178781-134181385
17971	Nudt17	78373	3-:96509129-96512638
17972	BC023744	231668	5+:117769375-117801801
17973	MMT00065215		14-:14610252-14610864
17974	Tek	21687	4+:94405990-94541629
17975	MMT00079364		5+:8179591-8181202
17976	P2ry10	78826	X+:104207740-104335105
17977	T2	21331	17+:8589493-8615589
17978	MMT00009316		7+:4363924-4365042
17979	Krt42	68239	11-:100124223-100127239
17980	EG629065	629065	17-:53839593-53882749
17981	MMT00036029	268527	12-:16678430-16764047
17982	2310047O13Rik	66960	2-:12269220-12341038
17983	MMT00058803		17-:51623616-51623911
17984	Zcrb1	67197	15-:93216543-93228721
17985	Ntng2	171171	2-:29050340-29103560
17986	MMT00019829		3+:64038543-64038849
17987	Fgfr2	14183	7-:137307301-137409705
17988	Abcb9	56325	5-:124511866-124545800
17989	V1rf2	171233	17+:21398378-21399364
17990	MMT00014863		13+:51440364-51441017
17991	Fbxl20	72194	11-:97950015-98011706
17992	Gmpr	66355	13+:45602837-45641750
17993	MMT00013883		17+:72605761-72606658
17994	Zh2c2	252876	1+:99666776-99689546
17995	Hist1h4d	319156	13+:23673470-23673780
17996	Igfbp2	16008	1+:72871153-72898734
17997	Plp2	18824	X-:7245526-7248421
17998	Sqstm1	18412	11-:50013654-50024292
17999	A730017L22Rik	613258	2+:130706338-130707869
18000	EG628870	628870	10-:107199418-107329314
18001	Nrl	18185	14-:56137814-56143028

18002	4930533N22Rik	75205	7+:82193760-82220050
18003	Tff3	21786	17-:31262259-31266556
18004	Rps3	27050	7-:106627396-106632136
18005	Chchd1	66121	14+:21522290-21523639
18006	Wdr16	71860	11-:67738313-67779144
18007	Mgat5	107895	1+:129203338-129380281
18008	1700011H14Rik	67082	14-:49846033-49865070
18009	MMT00068893		15-:103416861-103418807
18010	Tex28	385380	X-:71397630-71406955
18011	MMT00021469		4+:74793507-74795000
18012	Rhobtb3	73296	13-:76008585-76081246
18013	Acot5	217698	12+:85410273-85416969
18014	Dicer1	192119	12-:105925952-105990141
18015	Rgsl1	240816	1-:155640884-155668703
18016	Prrg2	65116	7-:52308959-52317792
18017	6330514A18Rik	216166	10+:79826760-79828227
18018	Galnt11	231050	5+:24728720-24771735
18019	MMT00049952		19+:3138928-3143133
18020	D19Ertd737e	76539	19-:60274070-60302610
18021	Zdhhc22	238331	12-:88324330-88329626
18022	Adamts17	233332	7+:74060735-74114441
18023	Pstpip1	19200	9+:55937775-55976685
18024	Cdh26	381409	2+:178184681-178206504
18025	1810012P15Rik	66274	11+:78640112-78652973
18026	Oit3	18302	10-:58885712-58901663
18027	5730403B10Rik	66626	16-:4766827-4789916
18028	Diap1	13367	18-:38004480-38095065
18029	Mpdz	17475	4-:80925503-81088576
18030	Ndufb8	67264	19-:44624743-44629901
18031	Pde4d	238871	13+:110530594-110562889
18032	MMT00056397		7+:18815422-18816640
18033	Klhl17	231003	4-:155603439-155608890
18034	3110006E14Rik	76980	13-:69841710-69878948
18035	Pigl	327942	11+:62271778-62327405
18036	Tmem100	67888	11+:89891661-89897819
18037	Klf9	16601	19+:23216244-23240447
18038	Sec24c	218811	14+:21493558-21514075
18039	Ubxd4	217379	12-:4886159-4914070
18040	Slc25a36	192287	9-:96978176-97011452
18041	MMT00063084		2-:48665213-48665981
18042	Aph1c	68318	9-:66666729-66682478
18043	Fgd3	30938	13-:49358669-49404609
18044	Zfp568	243905	7+:30798847-30808665
18045	Klk10	69540	7+:51036448-51040779
18046	Ublcp1	79560	11-:44269738-44284002
18047	Efnb3	13643	11-:69369362-69373315
18048	MMT00008116		12+:73701105-73701841
18049	Ribc1	66611	X-:148439126-148450838
18050	MMT00003408	330962	9-:65260522-65270741
18051	Smad5	17129	13+:56824929-56837363
18052	Fbn2	14119	18-:58169911-58369459

18053	Psmc4	23996	7-:28826735-28835062
18054	Mrps36	66128	13-:101505915-101514543
18055	Aes	14797	10+:81022347-81028909
18056	4632434I11Rik	74041	7-:100006075-100022749
18057	MMT00033947		1+:34985793-34987484
18058	Plekho1	67220	3-:95792763-95799762
18059	Centb2	78618	16-:31092500-31201324
18060	Ftsj1	54632	X-:7817546-7829436
18061	MMT00002521		6+:18094868-18095708
18062	Otog	18419	7+:53496356-53566803
18063	Rnf146	68031	10-:29065981-29081846
18064	MMT00035925		15-:77238201-77238877
18065	Peo1	226153	19+:45081046-45087251
18066	Ccdc51	66658	9+:108991850-108995006
18067	Itgb1bp2	26549	X+:98644503-98649118
18068	Tbc1d13	70296	2+:29989387-30007533
18069	Gpt	76282	15+:76526956-76530115
18070	Ogg1	18294	6+:113277004-113284178
18071	Zfp354a	21408	11+:50873573-50884764
18072	1110012D08Rik	73827	10-:128237024-128239573
18073	Hipk3	15259	2-:104269921-104334401
18074	Rorb	225998	19-:19006097-19007207
18075	EG623133	623133	8+:4641452-4643725
18076	Cadm4	260299	7+:25267117-25288762
18077	Clip2	269713	5-:134967761-135011040
18078	MMT00024172		15+:38892881-38893698
18079	Olfr75-ps1	258186	11-:73201611-73202526
18080	Olfr975	258825	9-:39757421-39758354
18081	Zic4	22774	9+:91273036-91279236
18082	Cntn5	244682	9-:9660902-9833582
18083	Gadd45a	13197	6-:66985089-66987401
18084	Pak7	241656	2-:135908957-136000011
18085	Pwwp2b	101631	7+:146434391-146764239
18086	Olfr1022	258582	2+:85708750-85709696
18087	St6galnac3	20447	3-:152865473-153388097
18088	Alkbh6	233065	7+:31093719-31099325
18089	Phc1	13619	6-:122267749-122287015
18090	D10Bwg1379e	215821	10-:18308996-18384715
18091	Rap2c	72065	X-:48368146-48371312
18092	Erbb4	13869	1-:68245415-68442889
18093	Pfn4	382562	12+:4776099-4785611
18094	MMT00041834		3+:18317460-18434288
18095	P4ha3	320452	7+:107459296-107465620
18096	Slc39a6	106957	18-:24738382-24760073
18097	Apbb1	11785	7-:112706996-112722930
18098	Rdh14	105014	12+:10397587-10402370
18099	Nfx1	74164	4+:40918067-40971905
18100	Sdsl	257635	5-:120908211-120922819
18101	D030056L22Rik	225995	19+:18787674-18792918
18102	Mynn	80732	3+:30502424-30515751
18103	Spaca1	67652	4-:34115288-34137040

18104	Zfp687	78266	3-:94810527-94819140
18105	Vmn2r121	100038941	X-:121240948-121249519
18106	Med10	28077	13+:69948759-69954972
18107	Nek5	330721	8-:23204677-23207327
18108	Il31	76399	5-:123930167-123932596
18109	V1rc21	171194	6+:56906506-56907399
18110	MMT00056457		3-:37498187-37499111
18111	Renbp	19703	X-:71167460-71176148
18112	Ikzf5	67143	7-:138535151-138540367
18113	MMT00034400	100047043	2+:80677101-80678935
18114	Lmtk2	231876	5+:144861459-144949068
18115	Nup62cl	279706	X-:136542213-136562241
18116	Ahctf1	226747	1-:181719263-181723954
18117	MMT00043956		
18118	Cacybp	12301	1-:162133047-162143001
18119	Sin3a	20466	9+:56924182-56975959
18120	Tmem201	230917	4-:149089490-149102165
18121	MMT00028858		6-:68164212-68165247
18122	Snx21	101113	2+:164611514-164618275
18123	Plekhg6	213522	6-:125312124-125330520
18124	Ptprt	19281	2-:161353359-162486883
18125	MMT00040589		17+:33283596-33285270
18126	Dyrk2	69181	10-:118296607-118316068
18127	Mxra7	67622	11-:116664697-116689361
18128	Tpi1	21991	6-:124761898-124764176
18129	Fbxl5	242960	5-:44135856-44173280
18130	Klrd1	16643	6+:129543505-129548788
18131	Clcf1	56708	19+:4214391-4222615
18132	Rbms3	207181	9-:116868580-117019248
18133	Zfand5	22682	19+:21347181-21356516
18134	Hebp1	15199	6-:135087545-135118155
18135	Tmed3	66111	9-:89594042-89599829
18136	Tmprss11a	194597	5-:86873131-86874909
18137	Minpp1	17330	19+:32560258-32589843
18138	MMT00000385		8-:112274892-112276164
18139	8030498B09Rik	77547	9+:21194543-21195699
18140	Atg2b	76559	12-:106854348-106861352
18141	Camk1d	227541	2-:5219652-5366190
18142	Ppox	19044	1-:173207122-173211291
18143	Dnajc1	13418	2-:18138769-18314134
18144	Dmap1	66233	4-:117347340-117354542
18145	Rala	56044	13-:17974214-17988900
18146	MMT00067785		12-:7886611-7890676
18147	Nhlrc2	66866	19+:56623014-56673335
18148	Cables1	63955	18+:11997782-12104072
18149	Zfp13	22654	17-:23717737-23723219
18150	D10627	234358	8+:71752915-71753096
18151	Naip2	17948	13-:100914017-100967079
18152	Grhl3	230824	4-:135113082-135115079
18153	Serpinb5	20724	1+:108757779-108778684
18154	MMT00038850		17+:38151850-38151884

18155	Vmn2r28	665255	7-:5435612-5445417
18156	Olfr129	258324	17-:38191545-38192536
18157	Dgki	320127	6-:36796881-37099658
18158	MMT00060883		13+:62252373-62253404
18159	MMT00082708		7+:133319530-133320085
18160	Bace2	56175	16+:97578383-97658706
18161	Nbn	27354	4+:15885119-15919735
18162	BC049352	408059	9+:45004017-45056986
18163	Raly	19383	2+:154616892-154692977
18164	Gdf10	14560	14+:34736957-34748463
18165	5830482F20Rik	320435	7+:29573987-29583979
18166	Poll	56626	19-:45626767-45635033
18167	MMT00012252		6-:130433206-130889385
18168	Ptchd1	211612	X-:152008084-152057785
18169	Cbll1	104836	12-:32171765-32184405
18170	MMT00057405		X-:77306385-77307051
18171	MMT00007396		14-:95684454-95738711
18172	Nupr1	56312	7-:133766759-133768984
18173	Arpp19	59046	9+:74885723-74904611
18174	Stat2	20847	10+:127707631-127729905
18175	Clcn2	12724	16-:20709951-20716041
18176	Aqp11	66333	7-:104874888-104886758
18177	Cyp7b1	13123	3-:17972180-18143283
18178	Pitx1	18740	13-:55926416-55932786
18179	Dbp	13170	7+:52960601-52965521
18180	Gtrgeo22	110012	10+:79131717-79138877
18181	Epb4.1l2	13822	10+:25079684-25180699
18182	Nudt8	66387	19+:4000641-4002103
18183	MMT00016159		9+:5397319-5398630
18184	Klrb1b	80782	6-:128764397-128776309
18185	Ndufb2	68198	6+:39553034-39553295
18186	Parp14	547253	16-:35832965-35841606
18187	Prrc1	73137	18+:57514392-57549599
18188	Ezh2	14056	6-:47480279-47545019
18189	Camsap1l1	67886	1-:138164700-138221442
18190	Rcn2	26611	9+:55889666-55906648
18191	Ccdc21	70012	4-:133685777-133690052
18192	Tmtc3	237500	10-:99909564-99939409
18193	Sp8	320145	12+:120086001-120088891
18194	5730590G19Rik	77011	7+:86830662-86835889
18195	Mafk	17135	5+:140267500-140278606
18196	Camk2b	12323	11-:5869646-5965566
18197	Dnd1	213236	18-:36923320-36925867
18198	MMT00015146		7+:64281644-64282895
18199	Cdc42ep4	56699	11-:113588163-113612632
18200	Cd151	12476	7+:148653291-148657372
18201	MMT00060232		13+:62914411-62916315
18202	4933416E03Rik	71081	2+:159773073-159807024
18203	Ablim3	319713	18-:61959049-62031357
18204	Coro1c	23790	5-:114294078-114358686
18205	Olfr114	258284	17-:37726357-37727296

18206	Ralyl	76897	3+:13946396-14182281
18207	Dnali1	75563	4-:124733752-124743903
18208	Smoc2	64074	17+:14416541-14541771
18209	MMT00077977		7-:114070049-114070987
18210	Myom3	242702	4+:135318416-135320863
18211	Folr2	14276	7-:108988503-108992340
18212	Ttc3	22129	16+:94605518-94690828
18213	Tomm40	53333	7-:20286673-20300605
18214	Ankrd40	71452	11+:94189353-94196221
18215	Gpr146	80290	5+:139856649-139869777
18216	Atp5g3	228033	2-:73746502-73750461
18217	Mgat1	17308	11+:49059003-49076524
18218	Sepx1	27361	17+:24873611-24879723
18219	Ndufa11	69875	17+:56857188-56862114
18220	Odz1	23963	X-:40765589-40783405
18221	Ctrb1	66473	8-:114210420-114214910
18222	Mphosph9	269702	5-:124702224-124757055
18223	Cntn5	244682	9-:9970524-10145968
18224	AA414992	102402	13+:68320459-68321759
18225	Sfn	55948	4-:133156470-133157726
18226	Olfr1496	258991	19+:13855103-13856056
18227	Kcnq3	110862	15-:65826354-65863032
18228	Olfr978	259109	9+:39801396-39802330
18229	Syne2	319565	12+:77016175-77026468
18230	4930535B03Rik	75137	3-:95587890-95622786
18231	Kcnj4	16520	15-:79314870-79316207
18232	Chrna4	11438	2-:180759398-180773780
18233	Dapk3	13144	10+:80646749-80655743
18234	Hsf5	327992	11+:87449460-87471607
18235	Epsti1	108670	14+:78304070-78402457
18236	MMT00052782	626578	5-:105653679-105665181
18237	Foxp2	114142	6+:15146960-15388020
18238	MMT00011616		13+:23148584-23149485
18239	Golim4	73124	3-:75680112-75760779
18240	Scrt2	545474	2+:151917707-151919599
18241	MMT00022841		8-:71944329-71946072
18242	St3gal2	20444	8+:113481523-113494206
18243	Stk17b	98267	1-:53812949-53842014
18244	Rps27a	78294	11-:29445845-29447850
18245	Mup4	17843	4-:59969678-59973537
18246	Ndufs3	68349	2-:90734868-90742612
18247	Wdr69	71227	1+:83156336-83207143
18248	Scn2a1	110876	2+:65508552-65604465
18249	Atp5c1	11949	2-:9984912-10002059
18250	Mmp14	17387	14+:55050530-55060059
18251	Susd4	96935	1+:184695026-184825787
18252	Dip2b	239667	15+:99869173-100046097
18253	MMT00063327	100045681	12-:6501773-6505412
18254	MMT00078950		9+:17537011-17538656
18255	Nek11	208583	9-:105146593-105295351
18256	Sez6	20370	11+:77744540-77791563

18257	MMT00055244		19+:21630172-21632356
18258	Gm73	218476	13+:97556551-97601826
18259	Shbg	20415	11-:69428317-69431370
18260	Nid2	18074	14+:20570517-20631009
18261	Olfr1221	258904	2-:88951733-88952667
18262	Gabbr1	54393	17+:37187798-37209372
18263	MMT00061427		14-:98064978-98065596
18264	Tex14	83560	11+:87287905-87298367
18265	Herc4	67345	10+:62706691-62737181
18266	Olfr1184	258820	2+:88326890-88327824
18267	1810021J13Rik	66279	9+:37015919-37030765
18268	MMT00043750		3-:65310085-65319763
18269	Smc4	70099	3+:68808699-68835833
18270	Cecr2	330409	6+:120616437-120715401
18271	MMT00007507		14-:124797026-124797911
18272	4930524B17Rik	74725	6-:19154820-19269029
18273	Surf2	20931	2+:26771945-26775332
18274	Traf7	224619	17-:24665999-24669267
18275	D730039F16Rik	77996	2+:34729987-34747651
18276	Apoa2	11807	1+:173155219-173156501
18277	MMT00002597		18-:6370407-6371809
18278	Aspm	12316	1+:141377545-141390509
18279	Ogfr	72075	2+:180324163-180330230
18280	Dars	226414	1-:130260284-130313891
18281	MMT00077107		5+:105204789-105205824
18282	Tbcc	72726	17+:47027638-47028663
18283	5730593F17Rik	215512	11+:95224990-95243184
18284	Arl8a	68724	1+:137043403-137052846
18285	Mrpl11	66419	19+:4962314-4964943
18286	Slc41a3	71699	6+:90554978-90596403
18287	Ttc16	338348	2-:32617239-32627538
18288	Fli1	14247	9-:32231234-32349037
18289	Tap1	21354	17+:34324824-34333744
18290	Kif21b	16565	1+:138028025-138070735
18291	Crisp2	22024	17-:40901685-40931037
18292	Fras1	231470	5+:97123892-97136514
18293	Depdc5	277854	5+:33287621-33334320
18294	MMT00073933		12-:24398972-25403056
18295	Thsd4	207596	9-:60223754-60242353
18296	Pcgf5	76073	19+:36453556-36530694
18297	Psma7	26444	2-:179771080-179777107
18298	Slc25a37	67712	14-:69859907-69903113
18299	MMT00035424		8-:35370129-35370775
18300	Arg1	11846	10-:24635322-24647233
18301	MMT00075851		7+:70750826-70751432
18302	Fer1l3	226101	19-:37973528-38097135
18303	Mef2b	17259	8+:72663609-72691386
18304	Astl	215095	2+:127167381-127175919
18305	Etv5	104156	16-:22383086-22411820
18306	2410089E03Rik	73692	15+:8194376-8221162
18307	Tmem37	170706	1-:121964286-121970309

18308	Zfp672	319475	11-:58128568-58136859
18309	Obfc2b	69917	10-:127838779-127846855
18310	MMT00069164	100042074	18+:43632309-43636495
18311	Olfr1348	258915	7-:6453997-6454935
18312	Apon	28194	10+:127691272-127692692
18313	Prkce	18754	17+:86567929-87054029
18314	4631416L12Rik	622434	3+:62142697-62266141
18315	Rph3a	19894	5-:121391857-121459338
18316	Tgm6	241636	2+:129961485-129971027
18317	Arl13b	68146	16-:62793518-62811877
18318	MMT00037377		6+:65382327-65383995
18319	Pmf1	67037	3-:88198064-88214238
18320	Fancc	14088	13-:63413062-63533033
18321	Hydin	244653	8+:113029642-113049552
18322	Ubxd8	76577	13+:54723201-54765932
18323	6-Mar	223455	15-:31428328-31460235
18324	4930546H06Rik	75202	17+:17967848-17976035
18325	Cst10	58214	2+:149230984-149236012
18326	Rap1b	215449	10-:117250925-117261731
18327	Tmprss12	75002	15+:100111257-100123484
18328	Cyfip1	20430	7+:63097644-63185841
18329	MMT00069482		14-:71122952-71123216
18330	Hoxc10	209448	15+:102797234-102801507
18331	AF085738	192158	2-:174155841-174172225
18332	Gpr19	14760	6-:134819177-134837739
18333	Rassf8	71323	6+:145699084-145766363
18334	Bckdk	12041	7+:135048341-135052134
18335	Ddef2	211914	12+:21048788-21276274
18336	Lrpap1	16976	5-:35434153-35448374
18337	Esr2	13983	12-:77222061-77268471
18338	Akr1e1	56043	13-:4591776-4608382
18339	Sap30bp	57230	11+:115794980-115825962
18340	Slc25a31	73333	3+:40512790-40530151
18341	Msl31	17692	X-:165092048-165111830
18342	Hamp	84506	7-:31727389-31729036
18343	Smyd2	226830	1-:191704372-191746243
18344	Tsc22d4	78829	5+:138187484-138200975
18345	MMT00001228		18-:30654654-30655099
18346	Trib2	217410	12-:15800108-15822462
18347	2810451A06Rik	72778	15+:98929889-98935536
18348	Slc9a10	208169	16+:45555747-45599996
18349	Olfr851	258907	9+:19301193-19302132
18350	Tc2n	74413	12-:102884774-102947748
18351	Nfyc	18046	4-:120430782-120463086
18352	Olfr1094	258362	2+:86668910-86669901
18353	MMT00075622		15-:76356906-76357461
18354	Zfp650	68795	2+:69808193-69862648
18355	Sec63	140740	10+:42481351-42550208
18356	Prkrip1	66801	5-:136656954-136674824
18357	Plekha8	231999	6+:54544890-54591293
18358	Mdk	17242	2-:91770008-91772439

18359	Otop2	237987	11+:115190292-115193610
18360	Ntsr1	18216	2+:180234680-180279684
18361	MMT00000729		6-:42469515-42470312
18362	Srf	20807	17-:46683787-46693111
18363	Dao1	13142	5+:114459844-114474914
18364	Rgs17	56533	10+:4424202-4513768
18365	Bcl2l12	75736	7-:52246594-52252949
18366	Morc2a	74522	11+:3550382-3590425
18367	2310033E01Rik	641361	7-:25326674-25331025
18368	Mpped2	77015	2+:106533684-106707592
18369	Tmem136	235300	9-:42919287-42924633
18370	Col28a1	213945	6-:8025337-8025904
18371	Sap130	269003	18+:31793931-31882715
18372	Zfp593	68040	4-:133799221-133801443
18373	Dnajb1	81489	8+:86132091-86135801
18374	Lrtm2	211187	6-:119267072-119280613
18375	8430415E04Rik	74521	12+:104770773-104852042
18376	Pex11b	18632	3+:96439607-96448649
18377	Zfp423	94187	8-:90185710-90383212
18378	Igh	111507	12-:114954860-114955330
18379	MMT00001586		5-:23370623-23382264
18380	Tspan31	67125	10-:126504347-126507317
18381	Dupd1	435391	14-:22495804-22522298
18382	Dnpep	13437	1-:75305140-75313582
18383	Mrps17	66258	5+:130221402-130224566
18384	Klf6	23849	13+:5860791-5867031
18385	Spc25	66442	2-:69031952-69044214
18386	Anks3	72615	16-:4941442-4964277
18387	MMT00012948	100043129	3+:105893920-105905725
18388	Lnx1	16924	5-:74993204-75073817
18389	Rbms3	207181	9-:116486426-116732014
18390	2310057J16Rik	69697	8+:3602553-3609094
18391	EG546347	546347	X+:89735342-89736015
18392	Kbtbd2	210973	6-:56727518-56730303
18393	Spred1	114715	2+:116947460-117003684
18394	Cxcr3	12766	X-:98926874-98929486
18395	Clpp	53895	17+:57129731-57135610
18396	Laptm4a	17775	12+:8928218-8945533
18397	Ccng1	12450	11-:40564282-40568731
18398	Fkbp15	338355	4-:61965226-62021577
18399	Rilpl1	75695	5-:124943088-124981030
18400	Paip2b	232164	6-:83756057-83781658
18401	Crebbp	12914	16-:4083548-4213324
18402	MMT00015207		13+:54605157-54626161
18403	Itpr1	16438	6+:108163111-108501095
18404	Hdgfl1	15192	13-:26860658-26861988
18405	MMT00035749		14+:111178626-111179289
18406	Unc50	67387	1+:37487030-37495829
18407	Tsen54	76265	11+:115676023-115684402
18408	Styxl1	76571	5-:136223518-136254161
18409	Nicn1	66257	9+:108192782-108198021

18410	Socs5	56468	17+:87532973-87534582
18411	2610028H24Rik	76964	10+:75911870-75923917
18412	Usp7	252870	16-:8688818-8757165
18413	Trmt5	76357	12-:74381396-74387698
18414	Tprkb	69786	6+:85865722-85880277
18415	Cyp4f39	320997	17+:32605405-32629423
18416	Enpp7	238011	11+:118849530-118853652
18417	Kbtbd7	211255	14+:79826536-79830843
18418	Hspa1b	15511	17-:35093377-35096532
18419	Srprb	20818	9-:103092517-103104409
18420	Igh	111507	12-:115821756-116153281
18421	Fpr-rs4	14291	17+:18158696-18159668
18422	Tnfrsf11a	21934	1+:107677304-107741657
18423	Tmem43	74122	6+:91423744-91438452
18424	Fscn2	238021	11+:120223022-120229486
18425	Prickle2	243548	6-:92325941-92408652
18426	MMT00051243		10+:7205479-7207012
18427	Serpinb1a	66222	13-:32933994-32943054
18428	Sfrs2	20382	11-:116711220-116714396
18429	Hmha1	70719	10+:79479486-79494214
18430	Pxdn	69675	12+:30622998-30701175
18431	Tnxb	81877	17+:34807479-34856571
18432	4933430H15Rik	74485	3-:87541817-87552808
18433	Tmc5	74424	7+:125743745-125767332
18434	Furin	18550	7-:87534081-87547648
18435	EG665828	665828	8+:73455580-73456894
18436	MMT00010869		13-:15210646-15245917
18437	Trim10	19824	17+:37006536-37014765
18438	Map3k7ip2	68652	10-:7625937-7644660
18439	Cd209f	69142	8-:4102793-4105764
18440	Upk3a	22270	15+:84847657-84852962
18441	Fshb	14308	2-:106897438-106899807
18442	Zfp764	233893	7-:134547208-134550258
18443	B230380D07Rik	235461	9-:70450837-70504981
18444	MMT00058658		15-:3232775-3251191
18445	MMT00008642		3+:5595413-5611141
18446	Zyg11a	230590	4-:107859844-107877951
18447	Foxn3	71375	12-:100432319-100688325
18448	Klhl11	217194	11-:100323926-100334099
18449	MMT00015343		17+:42856677-42864986
18450	Kdr	16542	5-:76329300-76374400
18451	Csgalnact1	234356	8-:70881677-70985450
18452	Uts2r	217369	11+:121021584-121023287
18453	RP23-67E6.3	668066	2-:177171374-177173929
18454	Tor1aip1	208263	1-:157853912-157883425
18455	Mtnr1a	17773	8+:46154563-46173858
18456	Uts2d	224065	16-:27353408-27370324
18457	Gm9	194854	X-:34749917-34750981
18458	Pcyt1b	236899	X+:90920749-90991620
18459	Klhl22	224023	16+:17762900-17793473
18460	Hnrnpul	232989	7-:26507057-26539740

18461	Klra5	16636	6-:129849005-129863242
18462	Gm1060	381738	5+:30607918-30668992
18463	Clec1a	243653	6-:129377946-129402017
18464	Mc3r	17201	2+:172074359-172075331
18465	Prkacb	18749	3-:146395526-146475845
18466	Cacna2d4	319734	6+:119186546-119191279
18467	MMT00079660		X-:67922792-67922925
18468	Abca9	217262	11-:109962135-110029467
18469	Olfr906	258799	9+:38295615-38296551
18470	Stt3a	16430	9-:36539645-36575206
18471	Aph1b	208117	9-:66626650-66643239
18472	MMT00070953		18+:10236938-10237509
18473	Ptar1	72351	19+:23762207-23795501
18474	MMT00070175	671232	9+:122705181-122711247
18475	Mmp8	17394	9+:7558462-7568028
18476	Epm2a	13853	10+:11063201-11177521
18477	Gnat2	14686	3+:107896161-107904194
18478	F10	14058	8+:13037837-13055900
18479	Olfr1368	258527	13-:21233980-21234924
18480	Hs2st1	23908	3-:144096527-144232858
18481	E130306D19Rik	230098	4-:43509018-43512507
18482	Nup88	19069	11-:70754833-70783463
18483	Mthfd1	108156	12+:77356394-77420789
18484	MMT00033535	665506	6+:41496728-41498179
18485	Wdr31	71354	4-:62114767-62131906
18486	Lrfn4	225875	19-:4611785-4614504
18487	Serpina5	268591	12+:105339358-105344342
18488	Mapre2	212307	18+:23962504-24051278
18489	Olfr902	258798	9+:38256458-38257387
18490	MMT00020332		10-:105629435-105629826
18491	Hist1h1a	80838	13+:23855536-23856282
18492	Dhfr	13361	13+:93125158-93155635
18493	Oas1b	23961	5+:121262643-121274167
18494	Senp8	71599	9-:59584974-59585639
18495	C2cd3	277939	7+:107592825-107618665
18496	Mbd4	17193	6-:115792445-115803739
18497	1700065I17Rik	67343	18+:56748003-56754436
18498	Slc5a6	330064	5-:31338404-31351267
18499	Atp2c2	69047	8+:122223945-122281620
18500	MMT00074605	384806	8+:41878610-41882651
18501	1700012G19Rik	67078	17+:24607355-24608510
18502	MMT00069276	100044315	X-:103478397-103480435
18503	Serpinb1b	282663	13+:33176997-33186248
18504	0610040J01Rik	76261	5+:64203733-64290858
18505	MMT00071192		16+:48073089-48074594
18506	Otub2	68149	12+:104626914-104644553
18507	Bfsp2	107993	9-:103327250-103382703
18508	Atp4b	11945	8-:13386208-13396778
18509	MMT00038317		18+:53112103-53112887
18510	MMT00072943		9-:31851971-31852290
18511	Vmn2r72	244114	7-:92886295-92899182

18512	Sult3a1	57430	10+:33577526-33599322
18513	MMT00044275		10+:22511074-22512280
18514	4930412F15Rik	242408	4-:43672494-43673626
18515	Ddx28	71986	8-:108533521-108535333
18516	4932413F04Rik	319215	9+:103461717-103783600
18517	Klc2	16594	19-:5107747-5118244
18518	Cap1	12331	4-:122537303-122563107
18519	A4gnt	333424	9+:99512929-99521233
18520	MMT00005421		7-:13736393-13736930
18521	Prdm5	70779	6+:65729032-65886367
18522	MMT00046256		2+:80442889-80443161
18523	Lypd6	320343	2+:50021059-50046313
18524	2310003C23Rik	76425	2+:180444968-180453436
18525	Mpped1	223726	15+:83610509-83686877
18526	Dydc2	71200	14-:41862407-41882498
18527	EG632477	632477	14+:19875016-19935800
18528	Mark2	13728	19-:7351248-7415806
18529	MMT00006395		3+:93236987-93237855
18530	Robo4	74144	9+:37209631-37221607
18531	Akr1d1	208665	6+:37480172-37518392
18532	Olfr713	259036	7+:114179649-114180622
18533	Hmox1	15368	8+:77617520-77624484
18534	Ovol2	107586	2-:144130915-144156781
18535	Cntnap5a	636808	1+:118308661-118477250
18536	MMT00074528		
18537	Moxd1	59012	10+:23943387-24022450
18538	Cysltr2	70086	14+:73383447-73425982
18539	Vrk2	69922	11-:26371405-26493611
18540	Olfr1507	57269	14-:53109696-53110637
18541	Rab15	104886	12-:77898949-77923457
18542	Ankra2	68558	13+:99033196-99043867
18543	Add1	11518	5+:34916460-34974264
18544	Rbp3	19661	14+:34767207-34777401
18545	BC021891	234878	8+:128434353-128469215
18546	F930017I19Rik	791260	7-:109048302-109082380
18547	Sema6d	214968	2+:124479882-124493504
18548	1700012P22Rik	69364	4-:144008089-144028713
18549	Robo1	19876	16+:72904868-73046337
18550	MMT00008087		14-:122889747-122894211
18551	Skiv2l2	72198	13-:113657989-113717588
18552	Nr2c2ap	75692	8+:72654686-72657657
18553	Tekt4	71840	17+:25608531-25613538
18554	Lhx9	16876	1-:140724574-140744147
18555	Sdcbp2	228765	2+:151398367-151415733
18556	MMT00001956		6-:37636278-37637195
18557	Limch1	77569	5+:67249115-67448396
18558	Nol11	68979	11-:107027976-107050574
18559	Csf2ra	12982	19-:61300313-61304262
18560	Islr	26968	9-:58004082-58006312
18561	Ngly1	59007	14+:17081841-17144050
18562	Nup188	227699	2+:30152164-30199786

18563	MMT00004228		4+:39714645-39715454
18564	E4f1	13560	17-:24580737-24588464
18565	Barx1	12022	13+:48758663-48761888
18566	Pcdha12	192164	18+:37179883-37344909
18567	Ccr10	12777	11-:101034927-101036737
18568	MMT00068464		11+:10139499-10140252
18569	Thumpd1	233802	7-:126858609-126864279
18570	MMT00037828		13-:22255749-22257596
18571	Gm973	381260	1+:59689051-59693257
18572	Stx18	71116	5+:38430471-38528056
18573	Nphp3	74025	9+:103904657-103908025
18574	Parc	78309	17-:46637553-46683264
18575	Bzrap1	207777	11+:87574873-87598409
18576	Qars	97541	9+:108410383-108418278
18577	Btbd9	224671	17-:30357041-30551683
18578	Vps54	245944	11+:21139283-21221136
18579	Dr1	13486	5+:108697937-108707951
18580	Phactr4	100169	4-:131911844-131978325
18581	MMT00048298		12-:115434540-115492137
18582	Krtap6-2	16701	16-:89419570-89420284
18583	Rnf39	386454	17+:37079652-37084778
18584	Tex21	80384	12-:77299780-77346132
18585	MMT00020464		11+:60156743-60157666
18586	Tns1	21961	1-:73960484-73975087
18587	Krt35	53617	11-:99953511-99957539
18588	1700023A16Rik	69371	6+:146798622-146819929
18589	B3gat3	72727	19+:8995015-9001725
18590	Fgb	110135	3-:82845783-82853733
18591	MMT00034813		1-:155466728-155467858
18592	Txnl4a	27366	18+:80403580-80420264
18593	Rgs7bp	52882	13-:105741382-105844984
18594	1200002N14Rik	71712	10-:87785548-87819820
18595	Tmem109	68539	19-:10945153-10956233
18596	BC032203	210982	17-:46937614-46969265
18597	Ganc	76051	2+:120229936-120287432
18598	Git2	26431	5-:115180191-115223374
18599	Vmn2r70	670940	7-:92707214-92713870
18600	Guk1	14923	11-:58997611-59005274
18601	Oxct2a	64059	4-:122999118-123000859
18602	Wisp1	22402	15+:66722954-66754761
18603	MMT00039662		6+:67155612-67156075
18604	Cept1	99712	3-:106305902-106350649
18605	Lamp1	16783	8+:13159281-13175338
18606	A230074B11Rik	215413	1+:37117216-37120491
18607	Acat3	224530	17-:13117121-13133233
18608	Atn1	13498	6-:124692564-124699840
18609	Lrrc1	214345	9-:77278624-77392505
18610	Nt5c1b	70881	12+:10376868-10396939
18611	C1qtnf3	81799	15+:10882110-10909454
18612	Kcnn4	16534	7+:25155415-25169882
18613	Uqcrq	22272	11-:53241423-53244316

18614	Cep152	99100	2-:125389924-125444277
18615	MMT00033780		15+:5901688-5954532
18616	MMT00038680		
18617	BC022224	192970	11-:84633279-84642502
18618	Tex14	83560	11+:87299787-87369324
18619	2810406K13Rik	72184	7+:106614474-106622527
18620	Slmap	83997	14-:27234376-27262120
18621	MMT00076582		9+:32315996-32324542
18622	Pwp2	110816	10-:77633654-77639894
18623	Ccs	12460	19-:4825497-4839235
18624	Plagl1	22634	10+:12810714-12849691
18625	Cit	12704	5+:116295652-116458571
18626	Magoh	17149	4+:107552420-107560001
18627	MMT00059478		3+:60664692-60681509
18628	Disp1	68897	1_random-:48181-109669
18629	4933427E11Rik	66769	15+:74539590-74540802
18630	Epgn	71920	5+:91456542-91464236
18631	Gja1	14609	10+:56097152-56110221
18632	Asxl1	228790	2+:153217264-153228669
18633	Nbeal1	269198	1+:60310239-60317219
18634	Xrcc6	14375	15+:81846729-81870502
18635	Hps1	192236	19-:42829701-42853135
18636	Heg1	77446	16+:33684682-33767908
18637	Trim29	72169	9+:43118837-43144203
18638	Tubgcp6	328580	15-:88929660-88953247
18639	Cdk2	12566	10-:128135001-128142035
18640	Olfr168	258354	16-:19530073-19531011
18641	Nt5c1a	230718	4+:122878800-122893450
18642	Hdac11	232232	6+:91106808-91124676
18643	1700022A22Rik	71120	15+:46156103-46205445
18644	Shprh	268281	10+:10879308-10882025
18645	Xlr	22441	X-:51086958-51100861
18646	Abca3	27410	17+:24489086-24547530
18647	Celsr2	53883	3-:108195549-108218412
18648	Rnft1	76892	11+:86298200-86310049
18649	Ccne1	12447	7-:38883004-38892349
18650	Hiatl1	66631	13-:65166453-65174937
18651	Tulp2	56734	7+:52773313-52777750
18652	5830467P10Rik	241639	2-:132732289-132771615
18653	MMT00072901		19+:8798625-8800932
18654	Rae1	66679	2+:172825635-172841238
18655	Angpt2	11601	8-:18691330-18741488
18656	Myo10	17909	15+:25552680-25741979
18657	Arih1	23806	9-:59236358-59334932
18658	Gstk1	76263	6+:42195943-42200440
18659	Ephb6	13848	6+:41555790-41570502
18660	Pms2	18861	5+:144720548-144722129
18661	Scfd2	212986	5-:74712581-74793910
18662	Batf3	381319	1+:192921726-192932825
18663	4930408O21Rik	67983	7-:127802244-127813766
18664	Bcl2l11	12125	2-:127951667-127953562

18665	Hs6st1	50785	1+:36125253-36163290
18666	Hint3	66847	10-:30328165-30338266
18667	Baz1b	22385	5+:135663238-135720437
18668	Rab25	53868	3-:88345958-88352137
18669	Fxyd5	18301	7-:31817756-31827148
18670	MMT00045493		2+:55039890-55160219
18671	Stag2	20843	X+:39555056-39628566
18672	Oraov1	72284	7+:152101039-152107038
18673	Muc1	17829	3+:89032973-89037291
18674	Spaca4	69363	7-:52980477-52981177
18675	Ntan1	18203	16+:13819413-13835541
18676	5830428H23Rik	414758	19-:61194460-61194588
18677	Dlx1	13390	2+:71367501-71372037
18678	Olfr1413	259039	1+:94469749-94470720
18679	Cpne8	66871	15-:90317913-90509806
18680	EG633285	633285	3-:82667826-82670558
18681	Hexb	15212	13-:97946362-97968225
18682	3110082D06Rik	73235	17+:42453634-42514610
18683	Ctsa	19025	2+:164658483-164665082
18684	Cttn	13043	7-:151622773-151656629
18685	MMT00004912		12+:16214507-16245517
18686	Zfp143	20841	7+:117205269-117238741
18687	2310057M21Rik	68277	7-:138486231-138506185
18688	Leap2	259301	11-:53235554-53236684
18689	Fnbp1	14269	2-:30883401-30971832
18690	Defb7	246080	8+:19495096-19497775
18691	Ndfip1	65113	18+:38578718-38624857
18692	C630004H02Rik	217310	11-:115209021-115229032
18693	Klf4	16600	4-:55540140-55545338
18694	Pkn2	109333	3-:142454523-142545246
18695	MMT00013792	27278	5-:39097696-39267932
18696	MMT00042876		1+:180074024-180075515
18697	Afg3l1	114896	8+:126001820-126026077
18698	1700084J12Rik	73486	15-:33334728-33335731
18699	2700089E24Rik	381820	6+:132986504-133055808
18700	Trappc2l	59005	8+:125135545-125139488
18701	Il22ra2	237310	10+:19341833-19351589
18702	Mrc1	17533	2+:14151040-14253416
18703	Oog3	100012	4-:143747462-143750421
18704	Heatr3	234549	8+:90661417-90695925
18705	Emx1	13796	6+:85137574-85154455
18706	Lect1	16840	14-:80037500-80061977
18707	MMT00067242		5-:146383938-146384054
18708	Vax1	22326	19-:59240676-59244519
18709	Srp68	217337	11-:116106480-116124569
18710	MMT00034179		3-:121083174-121083658
18711	Usp10	22224	8+:122434751-122481457
18712	Tyw1	100929	5+:130732922-130817437
18713	3110037I16Rik	73172	4-:120593806-120597610
18714	Farsb	23874	1-:78414532-78485434
18715	MMT00032843		18+:9495618-9496481

18716	MMT00056724		4+:28280982-28281937
18717	Slc30a3	22784	5-:31390322-31395871
18718	Rab21	216344	10-:114726364-114752657
18719	AI464131	329828	4-:41444511-41445602
18720	BC002059	213811	17+:17088502-17111119
18721	MMT00030422		5-:43354611-43355981
18722	Polm	54125	11-:5727863-5737763
18723	Ccdc82	66396	9_random+:372094-378682
18724	Ramp1	51801	1+:93076615-93120256
18725	Ccdc60	269693	5-:116575599-116581182
18726	D630004A14Rik	382137	9+:50576341-50580773
18727	9130221D24Rik	77669	3-:132779456-132897916
18728	MMT00044404		5+:11891549-11928596
18729	Camta1	100072	4-:150448066-150538078
18730	1700019B03Rik	76406	8+:3475830-3487182
18731	Fgf17	14171	14-:71036525-71041579
18732	MMT00041012		10-:78091450-78092970
18733	Snx30	209131	4+:59818718-59913405
18734	EG622175	622175	13-:74715338-74754725
18735	Ddx18	66942	1-:123450420-123464479
18736	Tubb3	22152	8+:125935480-125945909
18737	Exoc1	69940	5+:76958337-76997197
18738	Hadha	97212	5-:30445964-30474563
18739	Hydin	244653	8+:113105859-113106705
18740	MMT00052300		1+:46111487-46115946
18741	Dnajc6	72685	4+:101180475-101315387
18742	Rnf217	268291	10-:31223556-31328350
18743	Ruvbl1	56505	6+:88415458-88447559
18744	Tbl1x	21372	X+:74756397-74908330
18745	4932422M17Rik	74366	5-:123584634-123591095
18746	Rhov	228543	2-:119094938-119096957
18747	MMT00049213		10+:126750204-126751202
18748	Chrna3	110834	9-:54860463-54874339
18749	Il2rb	16185	15-:78310980-78325452
18750	Thap6	381650	5+:92391409-92399608
18751	Cpa6	329093	1-:10314807-10474144
18752	Fgf7	14178	2+:125861450-125915295
18753	Olfr1189	258768	2+:88431962-88432881
18754	Rbm8a	60365	3+:96433855-96436833
18755	Klkb1	16621	8-:46355025-46393820
18756	Tspan14	52588	14-:41719733-41780096
18757	Vsnl1	26950	12-:11333017-11393782
18758	Cxx1a	66158	X-:50945667-50946859
18759	Mctp1	78771	13+:76780972-77170005
18760	Il9r	16199	11-:32088996-32100222
18761	Rcn1	19672	2-:105227089-105239396
18762	Herc5	67138	6+:57530984-57615108
18763	Osgin2	209212	4-:15924373-15929268
18764	Olfr1426	258805	19-:12162344-12163280
18765	MMT00058531		12+:34531211-34532392
18766	Slc39a11	69806	11-:113106168-113427068

18767	Kcnc4	99738	3-:107241221-107261816
18768	Elmo2	140579	2-:165115861-165141818
18769	Zfp518	72672	19+:40916757-40990731
18770	Rnf208	68846	2+:25098815-25099613
18771	Tia1	21841	6+:86354404-86380721
18772	BC048355	381101	17+:46633729-46636175
18773	MMT00014972		12-:89522856-89524816
18774	MMT00054062		2+:45665083-45666035
18775	Best2	212989	8-:87531103-87537490
18776	Crnkl1	66877	2-:145743217-145760434
18777	MMT00066855		5+:147386463-147387697
18778	Prickle1	106042	15-:93329532-93349841
18779	MMT00032058		1+:134573522-134590401
18780	Olfr649	259057	7-:111337781-111338719
18781	MMT00014109		19+:23741437-23741952
18782	Anks1b	77531	10+:90292584-90404257
18783	1700019M22Rik	69423	12-:97284830-97285365
18784	Sdad1	231452	5-:92720038-92726249
18785	Angptl4	57875	17-:33911845-33918451
18786	MMT00059421		8-:109968184-109968498
18787	Rps23	66475	13+:91063191-91064555
18788	Triap1	69076	5+:115791255-115793561
18789	MMT00071096		12-:50941709-50942809
18790	Car3	12350	3+:14863519-14871863
18791	Dgkd	227333	1+:89776941-89842261
18792	Hmbox1	219150	14-:65442162-65568750
18793	Tdrd5	214575	1-:158185428-158193719
18794	MMT00054053		
18795	Olfr1262	258976	2+:89842564-89843479
18796	MMT00044598		11-:116995912-116996419
18797	1700019G24Rik	72249	6-:5913899-5927361
18798	Ndufa4l2	407790	10+:126952019-126954975
18799	Olfr1009	258565	2+:85561563-85562506
18800	Nr2e1	21907	10-:42283036-42303432
18801	MMT00031280		1+:134922400-134923053
18802	Nit2	52633	16-:57156778-57167445
18803	Shank1	243961	7+:51568028-51612814
18804	Pllp	67801	8-:97199343-97220623
18805	Ibtk	108837	9-:85580967-85642790
18806	Hist3h2ba	78303	11+:58762412-58763032
18807	Rpl37a	19981	1+:72757876-72760190
18808	Pdcd6	18570	13-:74440968-74454711
18809	Tmc6	217353	11-:117627302-117641922
18810	Igh-VJ558	16061	12-:115895540-115896003
18811	4930519B02Rik	75122	10-:12873277-12897799
18812	Fer1l5	211430	1+:36441929-36479188
18813	Chmp2b	68942	16-:65539042-65563113
18814	Cst12	69362	2+:148615104-148619162
18815	Hist1h1d	14957	13+:23646958-23647624
18816	Hisppd2a	327655	2-:121136080-121176745
18817	Zc3h13	67302	14+:75684399-75727410

18818	Pde4a	18577	9+:21001484-21016054
18819	V1rb7	113855	6+:89664196-89665127
18820	Capn7	12339	14+:32149909-32184455
18821	Gltscr2	68077	7-:16523186-16531407
18822	Megf6	230971	4+:153544850-153649822
18823	Vmn2r74	546980	7-:93100378-93110018
18824	Nrd1	230598	4+:108673369-108733755
18825	4933400E14Rik	71007	7-:141457957-141541511
18826	4930427A07Rik	104732	12+:114394645-114401722
18827	Oprs1	18391	4-:41685369-41688186
18828	MMT00042894		1+:179809871-179878451
18829	Smarcd2	83796	11-:106124500-106128936
18830	MMT00013368		11+:56272895-56273576
18831	4921524J17Rik	66714	8-:87932657-87956730
18832	Mybph	53311	1+:136090033-136097808
18833	Rfwd2	26374	1+:161236699-161277747
18834	Slc8a3	110893	12-:82300432-82434167
18835	Dtwd1	69185	2+:125980276-125991012
18836	Vmn2r27	232367	6-:124141871-124180483
18837	Paox	212503	7+:147312287-147320051
18838	Rdh13	108841	7-:4379216-4397243
18839	Pcdha1	116731	18+:37089938-37347311
18840	MMT00044904		17+:63684571-63697277
18841	C130038G02Rik	77521	5+:149114777-149125612
18842	Phlda1	21664	10+:110943341-110945704
18843	Rnf139	75841	15+:58726901-58731687
18844	1810043H04Rik	208501	11+:119960247-119962017
18845	Gna11	14672	10-:80993349-81007728
18846	Ikbkg	16151	X+:71678143-71694190
18847	Armetl1	227526	2+:3430333-3441285
18848	Sema3b	20347	9-:107500685-107507733
18849	Macrod2	72899	2+:142217967-142218699
18850	MMT00069233		X-:103564204-103565634
18851	Apip	56369	2+:102913939-102932795
18852	MMT00001015		
18853	Acp1	11431	12-:31580740-31596414
18854	MMT00016808		12+:15734853-15735773
18855	MMT00074946		17+:88349234-88349854
18856	Hmgcl	15356	4+:135502359-135518528
18857	1110012L19Rik	68618	X+:67639087-67642591
18858	5830418K08Rik	319675	9-:15126680-15151480
18859	C230094A16Rik	237711	11-:29644136-29664233
18860	Traf6	22034	2+:101518743-101538095
18861	Apoc4	11425	7-:20263496-20266729
18862	1300003B13Rik	74149	17+:22591344-22592804
18863	Zfp668	244219	7-:135009508-135019160
18864	Rwdd2b	53858	16-:87433653-87440791
18865	Ptges3	56351	10+:127496229-127514049
18866	MMT00038112	670717	5-:13121449-13121766
18867	5930416I19Rik	72440	6-:128307017-128313009
18868	Mpeg1	17476	19+:12535310-12539774

18869	Cxcl2	20310	5+:91332924-91334964
18870	Hnf4g	30942	3+:3508101-3657373
18871	AI314976	106821	17+:48548706-48556593
18872	Zfp64	22722	2-:168750870-168780759
18873	AB041550	58229	6+:87680862-87684010
18874	Olfr777	258537	10-:128623667-128706035
18875	EG636756	636756	X-:37353960-37354374
18876	Klf16	118445	10-:80029879-80040039
18877	Trpa1	277328	1-:14871677-14900986
18878	Pde4dip	83679	3-:97493749-97494486
18879	Cacna2d4	319734	6+:119192795-119299757
18880	Nxf3	245610	X-:132609873-132615314
18881	Cops6	26893	5+:138602543-138605520
18882	Tbc1d2	381605	4-:46617271-46662964
18883	MMT00050191		1+:119574427-119624531
18884	Ell	13716	8+:73063600-73116723
18885	Rps11	27207	7-:52377754-52379689
18886	Slc18a1	110877	8-:71561611-71613089
18887	Foxn1	15218	11-:78171789-78200060
18888	Unc13c	208898	9-:73328396-73606484
18889	Shq1	72171	6-:100523070-100621144
18890	MMT00011144		11+:85763996-85764319
18891	Frmd3	242506	4+:73659348-73848117
18892	Cyp4f40	631304	17+:32799075-32813423
18893	Lace1	215951	10-:42032391-42198220
18894	MMT00030135		7-:58196157-58198524
18895	Gcs1	57377	6+:83065574-83068700
18896	Smap1	98366	1-:23852461-23916529
18897	EG625835	625835	16+:23179628-23180083
18898	Rcbtb1	71330	14+:59820317-59856101
18899	Ifnar1	15975	16+:91485493-91507682
18900	Btbd12	52864	16-:4000711-4003748
18901	Hpx	15458	7-:112740122-112748636
18902	A530023O14Rik	244183	7-:111505221-111511484
18903	Crem	12916	18-:3266353-3327517
18904	Fdx1	14148	9-:51751411-51771638
18905	Prmt2	15468	10-:75669973-75700520
18906	Zfand3	21769	17+:30272258-30347638
18907	Prl4a1	19110	13+:28108091-28115412
18908	Cldn10	58187	14+:119187129-119273746
18909	Rfx3	19726	19-:27842811-28057438
18910	Zfp39	22698	11-:58716243-58717720
18911	Selenbp2	20342	3+:94497494-94508328
18912	1700019G17Rik	75541	6-:85849044-85852527
18913	Olfr552	259106	7+:109752869-109753822
18914	Ntrk2	18212	13+:59087161-59230164
18915	Med4	67381	14+:73909854-73918355
18916	Slbp	20492	5-:33982711-33994955
18917	Olfr448	258270	6+:42846451-42847383
18918	MMT00079827		7-:111122547-111125663
18919	6030468B19Rik	77727	11+:117659068-117667642

18920	Cd2ap	12488	17-:42932787-42989477
18921	V1rh1	171245	13+:22533140-22534031
18922	Syce1	74075	7-:147963128-147973779
18923	BC025816	234796	8+:122386204-122400888
18924	Sema6b	20359	17-:56262507-56272288
18925	Kcne1	16509	16-:92348676-92359119
18926	MMT00070399		14+:44326413-44328553
18927	Prl3d3	215029	13+:27213563-27254459
18928	Ttc28	209683	5+:111529712-111716417
18929	Klhdc7a	242721	4-:139520300-139538871
18930	Ugt1a6b	394435	1+:89999813-90004375
18931	MMT00014022		6+:46994898-46996404
18932	Cebpg	12611	7-:35835402-35842310
18933	Nmbr	18101	10+:14480094-14490361
18934	MMT00015154		11-:120533745-120534553
18935	D230044M03Rik	76743	10-:14435432-14438023
18936	Zfp560	434377	9-:20151747-20206511
18937	Cass4	320664	2+:172219362-172253425
18938	MMT00074326	100047309	10-:95332035-95333441
18939	Matn2	17181	15+:34236439-34365993
18940	Olfr1086	258585	2-:86516557-86517488
18941	Ube3c	100763	5+:29896054-30002616
18942	Hs3st3b1	54710	11-:63702535-63735734
18943	Prr11	270906	11-:86903666-86922195
18944	Cyp2j13	230459	4-:95711924-95744221
18945	MMT00052502		13+:74670998-74672030
18946	D8Ertd82e	244418	8+:37166665-37210843
18947	Ctse	13034	1+:133558522-133572084
18948	Olfr1270	258987	2-:89989246-89990161
18949	Rg9mtd2	108943	3+:137806004-137821240
18950	MMT00005163		6+:20445549-20445977
18951	MMT00063372		5+:35896362-35898566
18952	Slfnl1	194219	4+:120207618-120209272
18953	Rab33b	19338	3+:51287937-51300116
18954	2310034C09Rik	117172	16+:88759091-88760034
18955	Chgb	12653	2+:132607407-132620812
18956	Tmco5	67356	2+:116704443-116718230
18957	4921505C17Rik	78757	15+:6658380-6727442
18958	MMT00024874		2-:19958403-19959035
18959	Olfr681	404318	7+:112270098-112270918
18960	Eif2c1	236511	4-:126020671-126210564
18961	Arsk	77041	13-:76198329-76236009
18962	OTTMUSG000000	666317	13+:27943546-27949657
18963	1700041C02Rik	73332	4-:119065258-119073651
18964	Letmd1	68614	15+:100299524-100309594
18965	Spink4	20731	4+:40867089-40878427
18966	Dgkg	110197	16-:22468535-22622365
18967	MMT00011087		1+:79954536-79957431
18968	Cst6	73720	19-:5347860-5349385
18969	1300010F03Rik	219189	14+:79249050-79602115
18970	Tnfsf11	21943	14-:78677258-78707841

18971	Dok7	231134	5+:35409211-35422923
18972	Nat5	67877	2+:145729004-145742052
18973	Camk2n2	73047	16-:20619291-20621293
18974	Cse1l	110750	2+:166731595-166771763
18975	MMT00005776		X+:127059113-127059948
18976	Gm684	270157	9-:51085521-51085926
18977	4933432I09Rik	66779	16-:18195758-18213957
18978	Dnahc2	327954	11-:69234312-69358058
18979	Cd33	12489	7-:50783364-50788471
18980	Il7	16196	3-:7573147-7613727
18981	MMT00007409		13-:80840857-80841345
18982	Ascl2	17173	7-:150153823-150154894
18983	Ifrd2	15983	9+:107490048-107495369
18984	Pias3	229615	3+:96500391-96509886
18985	MMT00008426		X+:105642186-105644093
18986	BC057893	272027	4-:46128379-46151605
18987	Treh	58866	9+:44481320-44494387
18988	MMT00012332		11-:6736286-6737306
18989	Tff2	21785	17-:31278154-31281187
18990	Hal	15109	10+:92951828-92979227
18991	Gpkow	209416	X+:7285053-7287396
18992	Eif1	20918	11+:100181381-100182673
18993	EG330513	330513	7-:46666256-46668516
18994	Orai1	109305	5+:123464733-123480474
18995	Capza3	12344	6+:139990101-139991115
18996	Ahctf1	226747	1-:181692705-181713175
18997	Qpct	70536	17+:79451502-79489715
18998	Oc90	18256	15-:65707613-65743959
18999	Gatad2b	229542	3+:90145575-90162042
19000	Sfmbt2	353282	2+:10301807-10512195
19001	Ndufs7	75406	10+:79712196-79719537
19002	Ociad1	68095	5+:73684093-73705179
19003	Tmed6	66269	8-:109585383-109589544
19004	Pdk4	27273	6-:5433350-5446278
19005	ENSMUSG00000(	621427	6+:39370566-39371291
19006	Rnf130	59044	11+:49838872-49918214
19007	Ocrl	320634	X+:45265629-45319040
19008	Krt27	16675	11-:99206879-99212408
19009	MMT00006729		8+:10755221-10755452
19010	Bhmt	12116	13-:94387430-94407456
19011	Rpl3l	66211	17+:24869629-24873088
19012	Ankrd54	223690	15-:78883523-78893289
19013	Rpl18	19899	7+:52973476-52975823
19014	Nsmce4a	67872	7-:137676032-137690918
19015	MMT00042937		12-:96152582-96154135
19016	EG625530	625530	7-:111881956-111925984
19017	1700017D01Rik	69369	19-:11171305-11205371
19018	1700048O14Rik	73433	19-:26116472-26120958
19019	Timeless	21853	10+:127683139-127683516
19020	V1rh20	171277	13-:22943469-22944364
19021	Rtkn	20166	6+:83087082-83102570

19022	Ccnb2	12442	9-:70255496-70269337
19023	Kynu	70789	2+:43410847-43538237
19024	Mylk3	213435	8-:87866555-87879466
19025	Slc37a4	14385	9+:44206437-44211046
19026	Isg15	53606	4-:155573563-155574905
19027	MMT00058026		17-:22101212-22101549
19028	MMT00058251		3+:22424533-22424932
19029	Tada2l	217031	11-:83892179-83943091
19030	MMT00011246		14-:59498087-59499113
19031	Mms19	72199	19-:42018691-42055620
19032	Tymp	72962	15-:89202361-89207468
19033	Mettl9	59052	7+:128178186-128220347
19034	Bag4	67384	8-:26877672-26895670
19035	Atad2b	320817	12+:4924147-4961462
19036	Ppp2r1a	51792	17+:21082469-21102434
19037	Slc11a2	18174	15-:100218332-100253414
19038	MMT00013556		2-:50377811-50378556
19039	Csmd3	239420	15-:47413270-47585464
19040	Cdk2ap1	13445	5-:124795446-124804565
19041	9830102E05Rik	277898	6-:137965905-137992210
19042	Bnip3l	12177	14-:67606060-67627701
19043	Mfsd10	68294	5-:34976297-34979775
19044	MMT00004517		6-:38495246-38499153
19045	MMT00027514		9-:59614519-59614995
19046	Efs	13644	14-:55535471-55544649
19047	Olfr910	258807	9+:38346481-38347412
19048	4933417A18Rik	66761	13+:35016277-35047744
19049	Prkag1	19082	15-:98643228-98661911
19050	MMT00072769	100044229	5+:51071382-51073931
19051	Podnl1	244550	8+:86650243-86656414
19052	Mkrn2	67027	6+:115551986-115568696
19053	Rbak	57782	5-:143933867-143938342
19054	1700003M02Rik	69329	4-:34666821-34676152
19055	F430201B04Rik	621239	17-:26080306-26085506
19056	Zfp7	223669	15+:76709688-76722822
19057	Cntnap2	66797	6+:46297080-46371463
19058	Rbm4b	66704	19+:4756542-4762750
19059	MMT00071954		12+:31703383-31704688
19060	Adamts13	279028	2+:26831164-26862327
19061	Dolk	227697	2-:30139946-30142274
19062	Shh	20423	5-:28784395-28793303
19063	Evl	14026	12+:109793166-109926634
19064	MMT00004867		1-:154796483-154797341
19065	Slc35f3	210027	8+:128822685-128918468
19066	MMT00012632		3-:130631968-130633683
19067	BC023882	231123	5-:34496549-34512073
19068	6030408C04Rik	217558	12+:52449348-52476614
19069	MMT00002942	633282	14-:52188567-52192792
19070	EG436059	436059	8+:107538350-107544310
19071	Actr10	56444	12+:72038893-72063269
19072	MMT00068804		X-:98903951-98904917

19073	Akap4	11643	X+:6644674-6655729
19074	Gigyf1	57330	5+:137960365-137966931
19075	Lmnb2	16907	10-:80364108-80380906
19076	1500016O10Rik	68952	7+:133967470-133973734
19077	Has2	15117	15-:56497183-56526053
19078	EG668139	668139	7-:113648313-113740829
19079	Map3k9	338372	12-:82823025-82874170
19080	Myc	17869	15+:61816940-61821800
19081	9130011E15Rik	71617	19-:45892462-46039675
19082	Alk	11682	17-:72218327-72953167
19083	MMT00006959		12+:119841149-119841393
19084	Mmab	77697	5-:114883022-114894034
19085	MMT00050224		12+:3171970-3201057
19086	MMT00029151		
19087	Wapal	218914	14+:35486838-35559772
19088	Rest	19712	5+:77694511-77713168
19089	Peci	23986	13-:35069617-35085985
19090	Slc2a1	20525	4+:118781372-118809932
19091	Micall2	231830	5-:140182648-140212267
19092	MMT00045362		11+:96806153-96814775
19093	Gm1079	381836	7-:4908683-4915993
19094	Fscn1	14086	5+:143722051-143734862
19095	MMT00006438		6-:69009641-69061200
19096	C87198	97836	12+:57692547-57695539
19097	Sema3d	108151	5+:12383551-12588943
19098	MMT00017494		17+:9605375-9615322
19099	Olfr196	258775	16-:59167037-59167967
19100	Prss33	353130	17-:23970832-23981007
19101	4921504E06Rik	70909	2-:19415325-19475541
19102	Uhrf1bp1l	75089	10+:89207779-89283216
19103	Gm889	380755	12-:40903324-40912012
19104	2310057N15Rik	69696	16-:88773425-88774451
19105	Unc5c	22253	3+:141128527-141497886
19106	Nsbp1	50887	X-:106199873-106208698
19107	Olfr1383	404337	11+:49337226-49367547
19108	Armet	74840	9-:106791091-106794246
19109	Nanog	71950	6+:122657733-122664643
19110	Gm114	228730	2+:146715326-146795830
19111	Diap3	56419	14-:87056131-87540921
19112	Upb1	103149	10+:74869777-74902938
19113	Ifna2	15965	4-:88329111-88329683
19114	MMT00045204		2+:75646357-75646957
19115	Rab32	67844	10-:10265034-10278016
19116	Entpd3	215446	9+:120430925-120431940
19117	Olfr1318	258022	2+:111996283-111997070
19118	Prp2	83380	6+:132545963-132550718
19119	Wnt7a	22421	6-:91315843-91361103
19120	1700073E17Rik	381827	6+:145339945-145340747
19121	Trim31	224762	17+:37035074-37047162
19122	Rad9b	231724	5-:122775516-122804204
19123	Bub1	12235	2-:127625935-127657783

19124	Pim3	223775	15+:88692623-88696155
19125	Muc15	269328	2+:110571805-110579680
19126	Rnf167	70510	11+:70460786-70465372
19127	Zfp553	233887	7+:134376956-134381368
19128	Hnrnpk	15387	13-:58493311-58503877
19129	Serf2	378702	2+:121275152-121276793
19130	Wasl	73178	6-:24563808-24615010
19131	Gad2	14417	2+:22477837-22546510
19132	Adam7	11500	14-:69115395-69151732
19133	MMT00050037		2-:104630327-104656881
19134	Fn3k	63828	11+:121296281-121311797
19135	Ankrd22	52024	19-:34197111-34240393
19136	Trim13	66597	14+:62218392-62224780
19137	Bfsp1	12075	2-:143652402-143688667
19138	Krtap12-1	16694	10+:77183330-77184001
19139	Olfr112	258096	17-:37700292-37701254
19140	EG238217	238217	12-:67384700-67385344
19141	Gorasp1	74498	9-:119834375-119846677
19142	Gpatch1	67471	7-:36061562-36103459
19143	Mrpl40	18100	16-:18872310-18876730
19144	Ndufa2	17991	18-:36902114-36904193
19145	Acsbg1	94180	9-:54452803-54509692
19146	N4bp2l1	100637	5-:151374223-151379566
19147	MMT00070588		5+:138960653-138973694
19148	Rnf144b	218215	13+:47289389-47341778
19149	Cd34	12490	1+:196765109-196787472
19150	Kir3dl1	245616	X+:133059487-133064672
19151	Dmp1	13406	5+:104631635-104643129
19152	Ccdc15	245902	9-:37084943-37123472
19153	Ptgs1	19224	2+:36086046-36107782
19154	3110001K24Rik	75698	14-:35050222-35123702
19155	MMT00008800		6+:29700745-29701155
19156	Dalrd3	67789	9+:108473982-108475102
19157	Rps19bp1	66538	15-:80091051-80094726
19158	Dvl3	13544	16+:20517058-20531719
19159	Epn3	71889	11-:94351996-94361257
19160	MMT00042532		X+:85633638-85634431
19161	Cav2	12390	6+:17231357-17239004
19162	D15Ertd621e	210998	15+:58261546-58289292
19163	Cadps2	320405	6-:23212842-23394045
19164	Arhgap15	76117	2+:43604369-43746048
19165	MMT00000241		18-:55997841-55998528
19166	Soat2	223920	15+:101981005-101993867
19167	MMT00035949		
19168	Sgk3	170755	1+:9788289-9888995
19169	Atp10a	11982	7+:65913702-66084790
19170	Ephb1	270190	9-:102096476-102134600
19171	Tmem16d	320091	10-:88434016-88551039
19172	Prkg2	19092	5-:99361027-99466370
19173	Mb	17189	15-:76845918-76881098
19174	Otud5	54644	X+:7419812-7451925

19175	Spag6	50525	16-:16753109-16829387
19176	2210404J11Rik	381062	17+:15123436-15201197
19177	Gm131	229697	3-:107014209-107024635
19178	Bhlhb5	59058	3+:17954710-17957509
19179	Rnf187	108660	11-:58745790-58752609
19180	Sparc	20692	11-:55208002-55233368
19181	Slc35a3	229782	3-:116376074-116415170
19182	Igsf2	630146	3-:100797454-100824655
19183	Mnat1	17420	12+:74224716-74373839
19184	Skap1	78473	11+:96563877-96620737
19185	MMT00016747		4-:15746220-15748520
19186	Pla2g4c	232889	7+:13910002-13941675
19187	Chrm3	12671	13-:9876474-9878244
19188	MMT00042824		13+:62107935-62108670
19189	Sp100	20684	1+:87546588-87598136
19190	MMT00077382		19-:3821822-3824584
19191	Ccng2	12452	5+:93696620-93705221
19192	Hist1h1c	50708	13+:23830675-23832235
19193	Tsku	244152	7-:105500568-105509799
19194	EG668450	668450	14+:70759826-70760251
19195	Reep6	70335	10+:79792914-79799185
19196	Nfe2l1	18023	11-:96678733-96689061
19197	H2-Ob	15002	17+:34375916-34390849
19198	D10Wsu102e	28109	10+:82822981-82831578
19199	Gla	11605	X-:131122894-131135494
19200	Hemk1	69536	9-:107230039-107240626
19201	2210018M11Rik	233545	7-:105736939-105805101
19202	1500031L02Rik	66994	16+:32099887-32108140
19203	Mettl10	72096	7-:140042268-140044329
19204	Prr13	66151	15+:102289605-102293214
19205	Zfp329	67230	7-:13391445-13404201
19206	EG665186	665186	4-:133746718-133752866
19207	B430119L13Rik	320974	2-:132704970-132706628
19208	Vps52	224705	17+:34098555-34099962
19209	Zdhhc17	320150	10-:110379919-110423100
19210	Jag2	16450	12-:114146811-114167706
19211	MMT00046586		12-:21584046-21586504
19212	Mak	17152	13-:41121683-41165828
19213	5730403M16Rik	232853	7-:7054737-7089433
19214	Snapc1	75627	12+:75065547-75085377
19215	Mettl2	52686	11+:104987789-105001706
19216	Bik	12124	15+:83357301-83375065
19217	Cyr61	16007	3-:145309941-145312945
19218	Mdh1	17449	11-:21457243-21471816
19219	MMT00015104		3+:138264462-138265091
19220	MMT00040501	54381	15+:33013002-33524259
19221	Lrrc28	67867	7-:74658297-74790126
19222	MMT00029634		6-:130888044-130889986
19223	Ercc8	71991	13+:108948931-108984705
19224	4930430F08Rik	68281	10-:100031472-100052028
19225	Lrrk1	233328	7-:73410310-73427227

19226	Nras	18176	3+:102862151-102870343
19227	MMT00058471		2-:166832485-166834547
19228	CU075549.1	620807	4+:60018738-60019967
19229	Sumf1	58911	6-:108057022-108135550
19230	1700106N22Rik	73582	17+:85489942-85602361
19231	Muc10	17830	5+:88746336-88757835
19232	Exoc6b	75914	6-:84568480-84575233
19233	Ankrd23	78321	1-:36587038-36604064
19234	Nrg4	83961	9-:55074420-55130425
19235	4921510J17Rik	70894	11+:104953531-104978851
19236	MMT00045089		5+:117508677-117510005
19237	Znrd1	66136	17-:37091423-37095246
19238	Ttc35	66736	15+:43308774-43359323
19239	Olfr469	258418	7-:114966077-114966981
19240	Ahnak2	382643	12-:114023926-114026210
19241	Ewsr1	14030	11-:4969903-4999080
19242	Ran	19384	5+:129526051-129529038
19243	Olfr557	258358	7+:109846753-109847721
19244	Impdh2	23918	9+:108462864-108467849
19245	MMT00013285		19-:13669561-13670541
19246	MMT00061306		11-:77499897-77501385
19247	Prelp	116847	1-:135808988-135817979
19248	Txndc9	98258	1-:38043905-38054098
19249	Olfr767	258315	10-:128516089-128517017
19250	Olfr483	258730	7+:115246824-115247770
19251	Opn1sw	12057	6-:29327942-29330415
19252	Tmsb4x	19241	X-:163645026-163647081
19253	Ddx5	13207	11-:106642861-106649790
19254	Klhdc6	74589	6-:88567429-88577439
19255	Wnt8a	20890	18+:34701966-34707927
19256	AW121567	270028	8-:9207257-9233105
19257	Omp	18378	7-:105293437-105293928
19258	Daf2	13137	1-:132285113-132319586
19259	Kng1	16644	16+:23058492-23081762
19260	Nubp2	26426	17-:25019563-25023273
19261	Olfr31	18330	14+:15160626-15161579
19262	Cd274	60533	19+:29446941-29462582
19263	Usp35	244144	7-:104459877-104470688
19264	Cd52	23833	4-:133649453-133650977
19265	EG546036	546036	8+:13986085-14003560
19266	1700018B08Rik	76405	8-:124054683-124065854
19267	Olfr341	258952	2-:36334708-36335648
19268	Tubb1	545486	2+:174276245-174283471
19269	Lrrc48	74665	11+:60166881-60207835
19270	Krit1	79264	5+:3806466-3840941
19271	MMT00063670		5+:90794340-90799635
19272	Ctsg	13035	14-:56718719-56721411
19273	MMT00073897		14+:43722358-43724478
19274	Afap1	70292	5+:36236138-36346561
19275	Farp2	227377	1+:95408701-95518358
19276	H28	15061	3-:151421702-151425855

19277	MMT00044927		17-:65411187-65415719
19278	1700031M16Rik	73302	15+:98228875-98246699
19279	MMT00012963		5+:84292273-84292738
19280	Il17rd	171463	14+:27919446-27920468
19281	MMT00054244		10-:56397990-56411502
19282	Tbx3	21386	5+:120121701-120134727
19283	MMT00022420		4+:32294138-32295062
19284	Fbxo8	50753	8+:59029923-59070855
19285	Col22a1	69700	15-:71773509-71784740
19286	1110012M11Rik	73711	8+:74066850-74071923
19287	Rock1	19877	18-:10067468-10181315
19288	Olfr15	18312	16+:3838974-3839912
19289	Pofut1	140484	2+:153067298-153091715
19290	MMT00008156		11+:115354337-115357881
19291	MMT00032606		6-:114118922-114119518
19292	Pold2	18972	11-:5772191-5778220
19293	Ppil4	67418	10+:7512725-7542933
19294	Zfp385a	29813	15-:103144318-103170515
19295	Mto1	68291	9+:78296026-78321946
19296	MMT00010896		17+:57881369-57882284
19297	MMT00005272		1-:113948872-113951950
19298	Unc93b1	54445	19+:3935193-3949337
19299	Ptprz1	19283	6+:22825721-22954668
19300	Pla2g4f	271844	2-:120125702-120139738
19301	Cnrip1	380686	11+:16951584-16979374
19302	MMT00009466		11+:107277919-107331988
19303	Olfr1122	259033	2+:87227863-87228844
19304	MMT00065828		11-:30471232-30474683
19305	Azin1	54375	15-:38419879-38431276
19306	AI428936	233066	7+:31099839-31104066
19307	Mall	228576	2-:127530941-127555668
19308	C330006A16Rik	109299	2-:25992332-25995776
19309	Slc9a8	77031	2+:167247208-167302499
19310	Cylc1	67407	X+:108316066-108318433
19311	Masp1	17174	16-:23449489-23520676
19312	Vmn2r68	620697	7-:92370140-92381900
19313	Tomm7	66169	5-:23345471-23349925
19314	MMT00004478		14+:61061201-61072586
19315	MMT00056434		2+:56932158-56932287
19316	Ruvbl2	20174	7-:52677327-52689665
19317	EG626952	626952	15-:78384613-78385361
19318	Plk1	18817	7+:129302951-129313353
19319	Papolb	56522	5-:143003709-143006030
19320	Cyp2j7	546837	4-:95866026-95903350
19321	1700029I15Rik	75641	2+:92223210-92223725
19322	Myo15b	217328	11+:115749673-115753902
19323	Bnip3	12176	7-:146082933-146101152
19324	Reps2	194590	X-:158855278-159081439
19325	MMT00015449		19-:43277530-43353488
19326	Usp48	170707	4+:137212948-137214444
19327	Phb	18673	11+:95528342-95541349

19328	Daam1	208846	12+:73037544-73091255
19329	Usp40	227334	1-:89841340-89845171
19330	Ascc1	69090	10+:59465566-59562739
19331	Dtna	13527	18+:23693663-23762496
19332	Bpnt1	23827	1+:187156079-187180664
19333	Ccdc13	434446	9-:121736351-121743065
19334	Gpr44	14764	19+:11014610-11015758
19335	MMT00045767		2-:79001143-79001754
19336	Ap4e1	108011	2+:126834457-126893220
19337	Prelid1	66494	13+:55423391-55426511
19338	Sipa1l2	244668	8-:127988049-128016232
19339	Gem	14579	4+:11631603-11641896
19340	Serpina3k	20714	12+:105578715-105582491
19341	Tnip3	414084	6+:65540388-65564787
19342	Cth	107869	3-:157557387-157588013
19343	Nhlrc1	105193	13-:47108943-47110148
19344	Hdgfrp3	29877	7-:89038684-89078908
19345	Ssh2	237860	11+:77234760-77270615
19346	MMT00047912		17-:81645256-81646083
19347	1700054M17Rik	73358	2+:118130648-118133897
19348	Serpinb8	20725	1+:109486591-109503900
19349	Psg28	114871	7-:19008239-19017214
19350	Siglec5	233186	7+:50606774-50614839
19351	Prkcsh	19089	9+:21807478-21818665
19352	Vapb	56491	2+:173563193-173603722
19353	Zfp287	170740	11-:62526788-62542595
19354	Clu	12759	14+:66587461-66600385
19355	MMT00065736		7+:26766835-26773474
19356	Herc1	235439	9+:66198373-66221864
19357	Adra1a	11549	14+:67256414-67346800
19358	Grip1	74053	10+:119256567-119512629
19359	Sycp1-ps1	20958	7+:19371648-19373610
19360	Aco1	11428	4+:40090432-40146043
19361	Fzd10	93897	5+:129107092-129108840
19362	Gper	76854	5+:139899615-139903743
19363	Olfr520	259066	7+:106883654-106884604
19364	Ubqln4	94232	3+:88357637-88373646
19365	Myst4	54169	14+:22319089-22491354
19366	Top3a	21975	11-:60553560-60590867
19367	MMT00026682		16-:23717209-23717968
19368	Rln1	19773	19-:29405665-29409100
19369	MMT00069664		16-:50994968-50995364
19370	Gpr34	23890	X+:13216320-13217487
19371	Fgfr1	14182	8+:26642752-26684392
19372	Gnai2	14678	9-:107516918-107537631
19373	MMT00018326		16-:16196394-16198862
19374	Dclre1c	227525	2+:3341421-3371361
19375	Inhba	16323	13+:16106307-16119044
19376	Pet2	18630	X-:86649192-86655018
19377	1700011E24Rik	75467	17-:87788910-87827085
19378	C1ql4	239659	15-:98914869-98918814

19379	Lrsam1	227738	2-:32779838-32817112
19380	Atrn	11990	2+:130732231-130853476
19381	Tmem16e	233246	7+:58802031-58849726
19382	Slc2a6	227659	2-:26876884-26883483
19383	Med15	94112	16-:17651314-17722916
19384	MMT00073448		15+:89215695-89219069
19385	Akr1c6	83702	13+:4433636-4456642
19386	Jazf1	231986	6-:52719256-52727707
19387	Nol12	97961	15+:78765377-78771294
19388	MMT00078130		7+:117665946-117670638
19389	Iqcb1	320299	16+:36829088-36872806
19390	Ckap2	80986	8-:23278632-23296264
19391	Apex2	77622	X-:147006050-147022692
19392	Slc25a16	73132	10+:62383409-62409239
19393	Synpr	72003	14+:14117372-14447895
19394	Decr1	67460	4-:15844387-15872520
19395	Xk	22439	X+:8850039-8888298
19396	Wnt3a	22416	11-:59061545-59104254
19397	Dis3l2	208718	1+:88641254-88946673
19398	MMT00062467		11+:5534089-5534752
19399	Camta1	100072	4-:150433634-150448171
19400	Slc30a8	239436	15+:52138004-52165210
19401	Ms4a10	69826	19-:11036784-11049155
19402	Ddx19a	13680	8-:113498913-113521701
19403	A830080D01Rik	382252	X+:155970635-155990259
19404	MMT00004384		14-:68206664-68244531
19405	MMT00004324		14-:72931776-72932478
19406	Ikzf1	22778	11+:11590368-11669801
19407	Zhx1	22770	15-:57878860-57907776
19408	MMT00042388	260347	7-:103059527-103061913
19409	Rgsl1	240816	1-:155672405-155688978
19410	Glb1	12091	9+:114310236-114383495
19411	Hnrnpl	15388	7+:29593556-29607285
19412	1700019D03Rik	67080	1-:52982028-53020104
19413	Nptx2	53324	5+:145306864-145317500
19414	MMT00065577		2-:162807992-162809075
19415	B3gnt2	53625	11-:22735955-22759513
19416	Lcat	16816	8-:108463452-108467282
19417	Pdzd11	72621	X-:97818246-97821246
19418	Igk-V19-14	667881	6-:70356439-70356998
19419	MMT00002063		19+:8050307-8052539
19420	Lcn8	78076	2+:25508637-25511734
19421	Mbd2	17191	18+:70727841-70785783
19422	Fkbp1b	14226	12-:4839993-4848345
19423	Rlf	109263	4-:120818488-120887580
19424	Mcoln1	94178	8+:3500518-3515229
19425	Nphp4	260305	4+:151852284-151937289
19426	Golga7	57437	8-:24351817-24367484
19427	Rhod	11854	19-:4425458-4439424
19428	Tbc1d22a	223754	15+:86069578-86328933
19429	Scx	20289	15+:76287866-76289550

19430	Gm626	268729	14+:34316092-34352802
19431	Rpn1	103963	6+:88034504-88053954
19432	MMT00068429		
19433	Mex3b	108797	7+:90017065-90018718
19434	MMT00071117	546100	8-:108148149-108161946
19435	Mtdh	67154	15+:34012479-34045065
19436	Dapp1	26377	3-:137594569-137644488
19437	MMT00010950		13-:26991141-26991886
19438	MMT00012923		5+:78110124-78223321
19439	MMT00019887		12-:13794702-13795415
19440	Anxa9	71790	3-:95100016-95111054
19441	Armc2	213402	10-:41719615-41731828
19442	Rad17	19356	13-:101387118-101420789
19443	Prkab1	19079	5-:116463596-116474460
19444	EG623519	623519	16+:16968701-16969021
19445	Dync1i2	13427	2+:71050072-71101349
19446	2810453I06Rik	67238	5+:144309640-144325212
19447	MMT00004670		14+:24775161-24827036
19448	Olfr476	258926	7+:115111152-115111845
19449	Nkx6-3	74561	8+:24263741-24269111
19450	Tspan1	66805	4-:115835200-115840143
19451	Armc7	276905	11+:115336999-115351783
19452	Gm97	225923	19+:11771551-11786346
19453	Cdk2ap2	52004	19+:4097350-4099017
19454	Lce1i	76585	3-:92581131-92582821
19455	Ppp1r10	52040	17+:36054139-36088876
19456	Olfr727	258316	14+:50746253-50747226
19457	MMT00000372		4+:122935285-122936145
19458	Trim55	381485	3+:19544506-19574506
19459	Frk	14302	10+:34203325-34328382
19460	1700024B05Rik	75518	14+:42522801-42582626
19461	Gipc3	209047	10-:80800627-80806002
19462	4933439C20Rik	66776	11+:3027555-3059995
19463	Nol4	319211	18-:23199643-23286824
19464	Klra2	16633	6-:131169661-131197296
19465	Hacl1	56794	14-:32421045-32454149
19466	Pptc7	320717	5+:122734371-122774286
19467	Tmem42	66079	9+:122930467-122932602
19468	Plxna1	18844	6-:89268850-89312590
19469	Zer1	227693	2-:29955832-29968950
19470	Gm239	237558	10-:116240454-116271700
19471	Olfr503	259143	7+:115686139-115688727
19472	Olfr558	259097	7+:109857774-109858728
19473	MMT00055742		7+:132690752-132692137
19474	Foxred1	235169	9-:35011784-35018585
19475	Ddhd1	114874	14-:46238932-46277686
19476	Ift140	106633	17+:25182047-25236431
19477	Map2k2	26396	10+:80568770-80586627
19478	Rnaseh2b	67153	14+:62950940-62991830
19479	Cap2	67252	13+:46597514-46744249
19480	0610010K06Rik	71678	1_random-:249703-270618

19481	Stx5a	56389	19+:8816924-8829557
19482	Efhc1	71877	1+:20941765-20980923
19483	Depdc2	109294	1+:11079742-11279401
19484	MMT00054524		1-:45576787-45577587
19485	Vipr2	22355	12+:117316265-117383841
19486	MMT00016573		6-:17885835-17886847
19487	Adh5	11532	3+:138106106-138118264
19488	Rnft2	269695	5-:118643549-118692992
19489	Cldn9	56863	17-:23819963-23820616
19490	MMT00078676		7+:35036056-35036510
19491	Slc9a9	331004	9+:94570335-94836923
19492	Cd96	84544	16-:46035769-46120222
19493	Icosl	50723	10+:77532203-77542270
19494	MMT00008749		3+:12631296-12631933
19495	Ptrh2	217057	11+:86497414-86505952
19496	Glyctk	235582	9-:106055291-106060449
19497	Dync2h1	110350	9-:7140888-7177020
19498	Zc3h7a	106205	16-:11136696-11176382
19499	A930001A20Rik	77127	3-:14973810-14974552
19500	Tcra-V8	21566	14+:53549382-53550052
19501	Gm711	279029	2+:26800916-26804847
19502	Fbxo24	71176	5-:138053733-138066308
19503	Ptpla	30963	2-:13948458-13977514
19504	Stab2	192188	10-:86465697-86470759
19505	Card14	170720	11+:119176121-119206501
19506	Col11a1	12814	3+:113733642-113923205
19507	Ints10	70885	8+:71320610-71353306
19508	Cuedc2	67116	19-:46405035-46413114
19509	MMT00006904		5-:130061333-130061450
19510	Mysm1	320713	4-:94613629-94619628
19511	Pcdhb21	93892	18+:37673473-37676017
19512	Gtf2ird2	114674	5+:134659907-134694013
19513	Ap2a1	11771	7-:52155752-52184836
19514	Smc1a	24061	X+:148450970-148496510
19515	Pcnx	54604	12+:83019675-83098107
19516	Ppfia4	68507	1-:136193370-136229100
19517	AI316807	102032	8-:23573087-23587279
19518	Arfip1	99889	3-:84300018-84351948
19519	BC011248	224823	17-:46804406-46811204
19520	Uck2	80914	1-:169156218-169215254
19521	Ighmbp2	20589	19-:3259075-3282906
19522	Tcf21	21412	10-:22537084-22539934
19523	Sptlc3	228677	2+:139319907-139463404
19524	Rhag	19743	17+:40948097-40977703
19525	Srp19	66384	18+:34490836-34496250
19526	MMT00030710		6+:70359159-70410058
19527	Tfam	21780	10-:70690686-70700784
19528	Zfp503	218820	14-:22803184-22807084
19529	V1ri5	171256	13+:23228353-23229249
19530	Adam24	13526	8+:41764711-41767480
19531	1700124P09Rik	76998	9-:109668565-109693151

19532	Med8	80509	4+:118081984-118087714
19533	Gpr62	436090	9-:106366291-106368225
19534	Pkp2	67451	16+:16213437-16272805
19535	MMT00041431		2+:16191092-16192193
19536	Csnk2a2	13000	8-:97971879-98012445
19537	Kcnk12	210741	17-:88145166-88415053
19538	Wdr18	216156	10+:79422896-79431081
19539	Ptpn14	19250	1+:191610521-191694186
19540	Zfp428	232969	7+:25292204-25300683
19541	Glyat	107146	19+:12708022-12726227
19542	Hspb7	29818	4+:140977642-140980114
19543	B3gnt3	72297	8-:74215796-74217798
19544	Cga	12640	4+:34841027-34854623
19545	Rps9	76846	7+:3655610-3658643
19546	Dennd2c	329727	3+:102935478-102953145
19547	MMT00022553		12+:14501275-14502094
19548	Mgst3	66447	1-:169302514-169323928
19549	C330007P06Rik	77644	X-:34388540-34414106
19550	Zfx	22764	X-:91320427-91368239
19551	Rab11fip2	74998	19-:59982599-60018903
19552	Nlgn1	192167	3-:25594122-26032688
19553	Chtf18	214901	17-:25855975-25864347
19554	6330549D23Rik	229613	3-:96411544-96412703
19555	Cthrc1	68588	15+:38908482-38918665
19556	Efna1	13636	3-:89076136-89083526
19557	Cyyr1	224405	16-:85457480-85550639
19558	Syt11	229521	3-:88551623-88576246
19559	Icmt	57295	4+:151671419-151681759
19560	Trmt6	66926	2-:132629941-132641791
19561	Farsa	66590	8+:87380883-87393157
19562	Hdgf	15191	3+:87710488-87719615
19563	Adam18	13524	8-:25712718-25785225
19564	Bcam	57278	7-:20341487-20355825
19565	EG385454	385454	X-:149546947-149548891
19566	Kcnj11	16514	7-:53353912-53355396
19567	MMT00053547		5-:98504396-98507325
19568	MMT00005188		6+:19735571-19739463
19569	Mta2	23942	19+:9016534-9026767
19570	Pomc	18976	12+:3958128-3960467
19571	Gabrg1	14405	5-:71144860-71233458
19572	MMT00048292	238427	12-:115595912-115630427
19573	Serpine1	18787	5-:137537377-137548129
19574	Gm1502	385120	6-:69217839-69218103
19575	Ranbp1	19385	16-:18239908-18248795
19576	Stox1	216021	10-:62128120-62187182
19577	Rpa3	68240	6-:8205938-8209113
19578	Aox1	11761	1+:58086847-58163254
19579	Sirt1	93759	10-:62781808-62801765
19580	Spns1	73658	7-:133513581-133520925
19581	Mapk8ip3	30957	17-:25034498-25073907
19582	Tacr1	21336	6+:82353599-82507224

19583	Hcrt	15171	11-:100623006-100624245
19584	Nav2	78286	7+:56675543-56708328
19585	Krtap5-2	71623	7-:149420493-149437392
19586	1700015G11Rik	75529	7-:59267050-59271089
19587	Avpr2	12000	X+:71137440-71139765
19588	Ddb1	13194	19+:10680114-10704306
19589	Slc16a9	66859	10+:69708023-69748699
19590	MMT00026015		4+:82839845-82840950
19591	Adrm1	56436	2+:179906377-179910988
19592	Sema4a	20351	3-:88239883-88265104
19593	4930555F03Rik	75359	8+:50456266-50587338
19594	Ren1	19701	1+:135247268-135256894
19595	MMT00056884		
19596	Rltpr	234695	8+:108210046-108212440
19597	MMT00001011		
19598	1110021J02Rik	68597	17-:29840853-29842916
19599	Mobkl2c	100465	4+:115503827-115506440
19600	MMT00065551		10-:73153004-73193363
19601	Mpp6	56524	6+:50060375-50148579
19602	Il5ra	16192	6-:106660374-106699031
19603	Grsf1	231413	5-:89088265-89104696
19604	MMT00033363		1+:67632141-67633055
19605	EG631906	631906	10-:62884149-62884487
19606	Egr2	13654	10+:67000626-67004935
19607	Birc3	11796	9-:7848702-7873024
19608	Syt9	60510	7+:114514504-114650132
19609	Cage1	71213	13-:38111033-38128996
19610	Igh-VX24	195176	12-:114849374-114849828
19611	Cog8	97484	8-:109572608-109580557
19612	MMT00028216		13+:68995359-68996138
19613	EG546387	546387	X-:146657131-146657879
19614	MMT00075215		17-:34906777-34908179
19615	Bai3	210933	1-:25280984-25285732
19616	Spag17	74362	3+:99910630-99946683
19617	MMT00013143		
19618	Snx6	72183	12-:55847343-55896649
19619	Zfp438	240186	18-:5210033-5334438
19620	Zfand2a	100494	5-:139947170-139960342
19621	Cldn5	12741	16+:18777067-18778352
19622	Bdh2	69772	3+:134944189-134967387
19623	Myl7	17898	11-:5796638-5798782
19624	Olfr378	259026	11-:73238539-73239483
19625	MMT00052369	100043120	19-:61308300-61308841
19626	2010305A19Rik	69893	4+:108000751-108011404
19627	Ilvbl	216136	10+:78037737-78047243
19628	Impad1	242291	4-:4689638-4720241
19629	Smarcd3	66993	5-:24098442-24107766
19630	MMT00054560		14-:42132197-42137638
19631	Nsmce1	67711	7-:132611167-132634957
19632	Eif2b3	108067	4+:116692043-116759911
19633	Gm694	277744	4-:140988587-140992031

19634	Slc34a3	142681	2-:25084412-25089757
19635	Mmp23	26561	4-:155024764-155027493
19636	Sprr1b	20754	3-:92240845-92242685
19637	Tbx21	57765	11-:96959387-96976589
19638	Samd3	268288	10+:25983587-25991977
19639	Galr3	14429	15+:78872361-78873901
19640	Tph2	216343	10-:114516768-114619955
19641	Rps14	20044	18+:60936056-60938151
19642	Hdac7	56233	15-:97624073-97661808
19643	Ttc29	73301	8+:80769997-80857681
19644	MMT00042763	65115	8+:106694787-106743021
19645	Il12rb2	16162	6-:67242011-67326131
19646	MMT00047746		X+:8231584-8232131
19647	Cort	12854	4-:148499301-148500771
19648	Nlrp4a	243880	7+:27229230-27261161
19649	Cpsf3	54451	12+:21292304-21320632
19650	MMT00081007		X-:142036241-142036860
19651	Thex1	67276	8-:36530292-36558587
19652	Fbxo32	67731	15-:58011633-58046429
19653	Obfc2b	69917	10-:127838733-127846888
19654	D930017K21Rik	214685	15-:81514750-81526429
19655	3110040N11Rik	67290	7-:88926879-88934317
19656	Sox17	20671	1-:4481793-4486444
19657	MMT00026309		9-:17902456-18060609
19658	Cldnd2	74276	7+:50696149-50698697
19659	MMT00005649		X+:98899617-98921323
19660	Gkap1	56278	13-:58334815-58357204
19661	Epb4.1l2	13822	10+:25187897-25243320
19662	Dlgap3	242667	4+:126870311-126914238
19663	Tnfrsf22	79202	7-:150824191-150835503
19664	Chrnb3	108043	8+:28479253-28508452
19665	MMT00034233		18-:59989185-60015084
19666	MMT00012505		3+:112394525-112395469
19667	Vps37d	194309	5-:135549669-135554069
19668	Zfp509	75079	5-:38590835-38607860
19669	Nub1	53312	5+:24191792-24216194
19670	Fbxl14	101358	6+:119429803-119432631
19671	Upp2	76654	2+:58607351-58643648
19672	Cldn19	242653	4+:118928004-118932461
19673	Zc3h12c	244871	9-:51923511-51952300
19674	MMT00030712	16098	6+:70468234-70468839
19675	Rer1	67830	4-:154448220-154460406
19676	V1ra6	113848	6-:89794590-89795531
19677	Nlrp2	232827	7-:5250235-5302638
19678	Scn11a	24046	9-:119662884-119734574
19679	Il17d	239114	14+:58144026-58162002
19680	Gm1420	546213	6-:69598810-69701304
19681	Fbxw16	320083	9-:109334832-109342213
19682	Elmo1	140580	13+:20277293-20697167
19683	Cox6c	12864	15-:35861737-35867917
19684	A030005K14Rik	77921	1-:83054860-83055950

19685	MMT00009602		11-:117179232-117202029
19686	Olfr1098	258842	2-:86762741-86763665
19687	Mbd3l1	73503	9+:18282838-18289736
19688	Fap	14089	2-:62339001-62412078
19689	1110032A13Rik	68731	18-:80388998-80397398
19690	Arf5	11844	6+:28373639-28376499
19691	Olfr1239	258972	2-:89257651-89258568
19692	rp9	55934	9-:22252780-22272767
19693	MMT00038173		5+:13879195-13880101
19694	Grb14	50915	2-:64750541-64860823
19695	C330024D12Rik	78444	7-:75381569-75409121
19696	Thoc4	21681	11-:120455829-120459679
19697	Nek1	18004	8+:63472015-63610140
19698	Sema3c	20348	5+:17080681-17233655
19699	Rnf180	71816	13-:105921527-106084900
19700	E030011K20Rik	208613	3-:27764987-27785416
19701	9930021J17Rik	229488	3-:85463981-85621216
19702	MMT00053066		3+:9392258-9392728
19703	Dync1i1	13426	6+:5675811-5978037
19704	Ccdc90b	66365	7+:99709701-99721160
19705	H2-T3	15043	17-:36323516-36327073
19706	Vrk1	22367	12+:107274754-107313246
19707	Timm44	21856	8-:4259737-4275895
19708	Ly6a	110454	15-:74825416-74828330
19709	1-Sep	54204	7-:134357963-134361967
19710	Olfr799	258929	10+:129084185-129085119
19711	Eps8	13860	6-:137426697-137597790
19712	3010026O09Rik	68067	11+:49988352-50013611
19713	BC048599	232717	6-:40845261-40847865
19714	Fgf14	14169	14-:124379567-124591743
19715	St3gal4	20443	9-:34855018-34863174
19716	Rpp30	54364	19+:36158226-36179106
19717	Ccdc27	381580	4-:153400474-153402237
19718	2610002D18Rik	69885	4+:134077384-134079839
19719	EG634650	634650	5-:105752643-105775478
19720	Cyp26c1	546726	19+:37760169-37767771
19721	Mfsd7	243197	5-:108871140-108877915
19722	Pvrl1	58235	9+:43612803-43614851
19723	Pfkfb1	18639	X+:147028925-147078423
19724	Lancl2	71835	6+:57652629-57688473
19725	Dcp2	70640	18+:44540156-44577605
19726	Erp29	67397	5-:121894764-121902388
19727	Ttr	22139	18+:20823913-20832818
19728	1110007L15Rik	67604	5+:139728328-139746004
19729	Wdr27	71682	17-:14966522-15028730
19730	Mamdc2	71738	19-:23377098-23522931
19731	Olfr275	258857	4+:52838270-52839228
19732	MMT00024414	73980	1+:54738570-54793660
19733	MMT00044118		17-:66576845-66577348
19734	Dchs1	233651	7-:112901503-112921713
19735	Zfp579	68490	7-:4944452-4947716

19736	MMT00014681		13+:41735892-41736716
19737	Enpp1	18605	10-:24361216-24431908
19738	Lgals3bp	19039	11-:118254065-118263245
19739	Cmpk1	66588	4-:114633244-114659751
19740	Gpr98	110789	13-:81234067-81581613
19741	Acad10	71985	5-:122048298-122110499
19742	Cdc2l1	12537	4+:154998991-155024041
19743	Atg16l1	77040	1+:89652672-89688997
19744	Acy3	71670	19+:3986572-3990003
19745	MMT00047549		1+:125101729-125184245
19746	Glra2	237213	X-:161567872-161765400
19747	Spr	20751	6-:85083679-85087812
19748	Cpa2	232680	6+:30491656-30514477
19749	Ric8	101489	7+:148043301-148049041
19750	Tceb3	27224	4-:135561323-135577548
19751	Kbtbd5	72330	9+:121686807-121692937
19752	MMT00038877		13+:62486476-62486778
19753	Nrg3	18183	14-:39183612-40286376
19754	C130039O16Rik	238317	12+:85520899-85534804
19755	Eef1g	67160	19+:9041684-9052965
19756	Pex16	18633	2+:92215654-92221373
19757	Fkbp5	14229	17-:28536866-28578001
19758	Snx1	56440	9-:65935945-65972693
19759	MMT00001545		13-:78512247-78518319
19760	Ovol1	18426	19-:5549761-5560584
19761	Senp5	320213	16-:31960388-31961948
19762	Pih1d1	68845	7+:52409767-52415434
19763	Acp2	11432	2+:91042983-91052296
19764	Nrm	106582	17+:35998262-36002344
19765	Serpina3a	74069	12+:105350933-105360106
19766	Slc26a4	23985	12-:32204684-32244834
19767	Etv6	14011	6+:133985957-134216777
19768	Eef1b2	55949	1+:63223738-63226226
19769	Pomp	66537	5+:148672247-148687354
19770	MMT00051974		13+:75337041-75337488
19771	Fbp1	14121	13-:62966113-62989596
19772	Gnmt	14711	17-:46862614-46866114
19773	EG668838	668838	7-:54076232-54076871
19774	Arhgap6	11856	X+:165234152-165234743
19775	Klhl31	244923	9+:77497810-77503818
19776	Vipr1	22354	9+:121551966-121578682
19777	Lrit1	239037	14+:37868020-37878123
19778	OTTMUSG00000(	624860	11+:58248017-58253559
19779	Itga8	241226	2-:12029555-12223706
19780	MMT00052614		12-:79356420-79356718
19781	Muc6	353328	7-:148820338-148838208
19782	Serpina1b	20701	12-:104952883-104971924
19783	Mybl2	17865	2+:162880422-162909405
19784	Polr2d	69241	18+:31948812-31956296
19785	Slc25a44	229517	3-:88214420-88229022
19786	Tpp2	22019	1+:43990851-44059816

19787	Tmem9b	56786	7-:116879349-116895777
19788	Btbd12	52864	16-:3979148-3989119
19789	Slc31a1	20529	4+:62021763-62052799
19790	Polg2	50776	11-:106629619-106640756
19791	E2f3	13557	13-:30001287-30077289
19792	Hook1	77963	4+:95633987-95689115
19793	Loxhd1	240411	18+:77669805-77680994
19794	Rgs2	19735	1-:145848226-145851263
19795	MMT00065928		16+:23514341-23515173
19796	MMT00006746		8-:10808730-10858119
19797	Coq9	67914	8+:97362295-97378793
19798	Tnk2	51789	16+:32644946-32683578
19799	9530008L14Rik	109254	13-:41859501-41942986
19800	4921524L21Rik	70901	18+:6619300-6638964
19801	Chst14	72136	2+:118752296-118754315
19802	E130309D02Rik	231868	5-:144062600-144077080
19803	BC003993	80744	2-:77733824-77784223
19804	Rela	19697	19+:5637774-5648130
19805	Pfkfb2	18640	1-:132594129-132612310
19806	4732429D16Rik	217305	11-:114843762-114851200
19807	Eea1	216238	10+:95436612-95508144
19808	Fetub	59083	16+:22920373-22939824
19809	Cpvl	71287	6-:53823272-53928668
19810	BC021395	225283	18-:24646450-24688620
19811	Cel	12613	2-:28411339-28418882
19812	Armcx2	67416	X-:131338685-131343535
19813	Defcr-rs1	13218	8-:22235560-22235729
19814	4930458L03Rik	78124	13+:65018862-65138977
19815	Tnr	21960	1+:161780177-161854606
19816	Olfr905	258800	9+:38280333-38281264
19817	Olfr38	258988	6+:42712052-42713005
19818	Cndp1	338403	18-:84782142-84819417
19819	Thy1	21838	9+:43854064-43855533
19820	Med30	69790	15+:52543999-52561986
19821	Vangl2	93840	1-:173934730-173957164
19822	MMT00004881		4-:105046460-105047615
19823	Gp5	14729	16-:30308238-30309940
19824	Cetn3	12626	13+:81922339-81935694
19825	Tssk2	22115	16+:17898806-17900116
19826	Nanp	67311	2-:150855432-150865091
19827	MMT00003831		19-:50614298-50615375
19828	Apoa1	11806	9+:46036792-46038547
19829	Zfp238	30928	1+:179375951-179380891
19830	Irf4	16364	13+:30841126-30858794
19831	Fgd6	13998	10+:93498743-93506365
19832	MMT00062321	634933	12+:18371645-18372838
19833	Ddah2	51793	17+:35196045-35199038
19834	Jarid1d	20592	Y+:234215-280253
19835	Iars2	381314	1-:187108096-187121466
19836	Prkrir	72981	7+:105851892-105866754
19837	Angpt4	11602	2+:151737067-151770389

19838	Rabl3	67657	16+:37539985-37572468
19839	EG269105	269105	1+:15057153-15057543
19840	Kcnip3	56461	2-:127282239-127347076
19841	Ccdc84	382073	9-:44218242-44226120
19842	B230315N10Rik	237411	10-:81687603-81704015
19843	Rbm45	241490	2+:76208113-76221822
19844	Ngf	18049	3+:102317877-102324928
19845	MMT00011958		10+:67038626-67049136
19846	Olfr62	18363	4+:118338123-118339070
19847	Vax2	24113	6+:83661257-83688298
19848	EG621322	621322	X+:131805391-131808752
19849	Dusp18	75219	11+:3795237-3799320
19850	MMT00036391		6+:140368645-140369175
19851	Gnb4	14696	3-:32483976-32501063
19852	1700007I06Rik	69306	11-:32422750-32427579
19853	Uqcr	66594	10-:79865743-79869510
19854	Kcnj14	211480	7-:53071837-53080117
19855	Btbd2	208198	10-:80105369-80108153
19856	Olfr1444	258697	19+:12936266-12937226
19857	Rab1b	76308	19-:5099206-5106996
19858	Lmbr1	56873	5-:29691715-29704732
19859	Fbxo46	243867	7+:19717039-19723603
19860	Tm9sf3	107358	19-:41288284-41338426
19861	Lrrc7	242274	3-:157747339-158115753
19862	Drap1	66556	19-:5422825-5424916
19863	Olfr65	18365	7+:111051970-111056050
19864	Olfr1406	258758	1-:175113630-175114563
19865	MMT00005074		14+:53562438-53695496
19866	Uso1	56041	5+:92566965-92631832
19867	Timm22	56322	11+:76220692-76228073
19868	Was	22376	X-:7658591-7667578
19869	MMT00038937		13-:64725573-64726139
19870	Pcdhb15	93886	18+:37633370-37635729
19871	MMT00065790		14-:15280277-15281554
19872	D430028G21Rik	228607	2+:131059873-131073758
19873	Olfr1242	258970	2-:89333548-89334467
19874	Prmt1	15469	7-:52232127-52241702
19875	Sos1	20662	17-:80796788-80879278
19876	6030443O07Rik	226151	19+:45005571-45058273
19877	Ccnb1	268697	13-:101553526-101556357
19878	Gm561	228715	2+:144419791-144421103
19879	Slc5a11	233836	7+:130358379-130416764
19880	MMT00046829		17+:25841993-25842766
19881	Tle3	21887	9+:61221478-61264783
19882	Prepl	213760	17-:85462817-85489561
19883	MMT00033230		7+:33576622-33577703
19884	BC067068	216292	10-:105200244-105260366
19885	AI427122	102502	9-:95653063-95745698
19886	MMT00015235		13+:66699587-66699622
19887	Hba-x	15126	11+:32176663-32178006
19888	Cadm3	94332	1-:175266130-175297770

19889	Ide	15925	19-:37344919-37405103
19890	Tmem119	231633	5-:114243738-114250361
19891	Azi1	12009	11-:119925749-119948132
19892	EG546164	546164	9-:115346777-115347723
19893	MMT00026027		12-:114411096-114411408
19894	4930488B01Rik	75036	7+:17548571-17549954
19895	Zfp612	234725	8+:112603662-112615273
19896	MMT00006823		
19897	Chsy3	78923	18+:59568537-59570928
19898	Vps13b	666173	15+:35501833-35572708
19899	Senp2	75826	16+:22009578-22047019
19900	Serinc5	218442	13+:93430972-93481907
19901	1700001P01Rik	72215	11-:97632793-97637195
19902	Spats2	72572	15+:98957275-99042896
19903	1700041C23Rik	67319	9+:57023079-57033910
19904	Ltb	16994	17+:35331466-35333247
19905	Ccnd3	12445	17+:47730496-47736635
19906	Tfpi	21788	2-:84280878-84314152
19907	Spata19	75469	9+:27204405-27209295
19908	Olfr676	259099	7+:112183713-112184665
19909	Sppl3	74585	5+:115461219-115548801
19910	St6galnac1	20445	11-:116626339-116636821
19911	MMT00045665		11+:22286427-22286910
19912	Inpp1	16329	1-:52846265-52874381
19913	1810030J14Rik	66289	1+:176260648-176263004
19914	Acaca	107476	11+:84008939-84159590
19915	Grp	225642	18+:66032453-66046244
19916	Mxd1	17119	6-:86600520-86619048
19917	Nek2	18005	1+:193645440-193656921
19918	MMT00061975	633269	4-:122272938-122282148
19919	Unc5b	107449	10-:60227343-60294329
19920	Dph2	67728	4-:117561248-117564636
19921	Stxbp5l	207227	16-:37115917-37385025
19922	MMT00048460		17+:86174726-86284916
19923	Capn3	12335	2+:120289608-120330234
19924	Ubiad1	71707	4-:147808605-147818860
19925	Arsa	11883	15-:89302907-89307203
19926	Tmem22	245020	9-:100452797-100475816
19927	Npy6r	18169	18+:44435167-44436283
19928	Olfr1123	258347	2+:87258206-87259177
19929	MMT00079667		13-:82517908-82518445
19930	Gapdh	14433	6-:125112004-125115332
19931	Lsamp	268890	16+:42134418-42175073
19932	MMT00075591		3+:68649921-68651817
19933	Pi16	74116	17+:29456186-29464894
19934	MMT00079286		
19935	Zdhhc11	71164	13+:74101296-74130437
19936	Egf	13645	3-:129380495-129458234
19937	2410018G20Rik	72083	16-:15848533-15863415
19938	4930547C10Rik	68274	4+:52609145-52625035
19939	Serpinb9b	20706	13+:33119282-33132425

19940	Eomes	13813	9+:118387329-118395238
19941	1700080E11Rik	73532	9-:105045673-105047412
19942	Mtmr1	53332	X+:68617964-68672371
19943	1700013N18Rik	73318	5+:108259271-108260361
19944	Adad1	21744	3+:36962592-37010433
19945	MMT00036313		9+:106267972-106270684
19946	Elf5	13711	2+:103264233-103291142
19947	Fcrla	98752	1-:172848025-172857671
19948	MMT00019088		1+:103282750-103283166
19949	Tubgcp2	74237	7-:147181894-147222165
19950	Psmg1	56088	16-:96201541-96212510
19951	Tmem63b	224807	17-:45797125-45823856
19952	Tlcd1	68385	11+:77992267-77994273
19953	4931429L15Rik	74361	9-:46111449-46124810
19954	Abcd3	19299	3-:121463149-121518036
19955	Cbx2	12416	11+:118884275-118890649
19956	Mycs	17870	X-:5044848-5046142
19957	MMT00013660		4+:98769562-98770251
19958	Rab38	72433	7+:95578840-95639983
19959	1700052H01Rik	73462	3+:41158066-41171669
19960	MMT00015578		5+:10558352-10559434
19961	E430025E21Rik	223593	15-:59163014-59201739
19962	Eno2	13807	6-:124710382-124719552
19963	Prl7c1	67505	13-:27865363-27870856
19964	Ndufa9	66108	6-:126771972-126799143
19965	MMT00003301		6+:42750840-42751439
19966	Thap4	67026	1-:95601968-95647652
19967	MMT00003872		6+:41085165-41085621
19968	EG546337	546337	X-:76100636-76103634
19969	Dlx2	13392	2-:71381465-71384798
19970	Mrpl36	94066	13+:73468456-73469625
19971	B930046C15Rik	544998	14+:3518337-3539523
19972	Copg2	54160	6-:30698411-30846697
19973	5730507C01Rik	236366	12+:18521298-18572254
19974	Pax6	18508	2+:105516098-105537498
19975	1200015N20Rik	71721	10+:70015790-70021477
19976	Cyp4a10	13117	4+:115192187-115193802
19977	Pgk1	18655	X+:103382589-103390117
19978	Rspo1	192199	4+:124663673-124686343
19979	Inha	16322	1+:75503822-75506736
19980	MMT00003169		6+:43072291-43073249
19981	MMT00032188		1+:165553844-165554840
19982	ENSMUSG00000(	100038711	17-:86897071-86904579
19983	Cox8a	12868	19-:7289692-7292025
19984	Elk4	13714	1+:133904452-133920238
19985	Gstcd	67553	3-:132644739-132747968
19986	MMT00026797		16+:47075750-47076054
19987	Tspan13	66109	12-:36741143-36769003
19988	Shroom2	110380	X-:149046924-149204442
19989	Pip5kl1	227733	2+:32433628-32439293
19990	4921515J06Rik	66715	3+:108743029-108763690

19991	MMT00032841		18-:9696469-9726641
19992	Trpm1	17364	7+:71330475-71369634
19993	Vps33b	233405	7+:87414579-87436466
19994	Depdc5	277854	5+:33206416-33298949
19995	Pwp1	103136	10+:85334609-85351841
19996	Ankrd27	245886	7+:36371271-36394113
19997	Ikbke	56489	1-:133151177-133173153
19998	Prl2a1	56635	13+:27893523-27900584
19999	Ift122	81896	6+:115724628-115876712
20000	MMT00017707		13-:101783039-101783970
20001	BC037034	231807	5-:138700887-138705060
20002	4932414N04Rik	75721	2+:68495472-68586585
20003	Nek10	238944	14+:15683273-15694202
20004	1190005F20Rik	98685	1+:153275792-153305294
20005	Dpy19l3	233115	7-:36514704-36539390
20006	Epha7	13841	4+:28740301-28893966
20007	Rhbdf1	13650	11-:32109586-32122198
20008	Anapc5	59008	5-:123237735-123271294
20009	Acot8	170789	2-:164618267-164630372
20010	Ccdc144b	241943	3-:35916595-35952455
20011	EG667441	667441	14+:53200268-53200931
20012	Gm336	240539	19-:11165060-11166907
20013	MMT00009595		14+:28527566-28529147
20014	Bbs12	241950	3+:37201776-37235295
20015	Il28ra	242700	4+:135242440-135259784
20016	MMT00051462		9-:83859587-83860671
20017	MMT00006294		17+:36294836-36296988
20018	Ficd	231630	5+:114186958-114189139
20019	Qdpr	110391	5-:45825813-45841419
20020	Clgn	12745	8+:85913776-85950729
20021	Igsf9b	235086	9+:27116988-27142318
20022	Mei1	74369	15+:81912904-81957244
20023	Myom3	242702	4+:135331459-135370572
20024	Lrrc32	434215	7+:105642731-105650338
20025	Crmp1	12933	5+:37637027-37682755
20026	Lrrc29	234684	8-:107836166-107850154
20027	Tcrb-J	21580	6+:41168089-41168564
20028	Ece2	107522	16+:20611673-20619080
20029	Mamld1	333639	X+:68354656-68408087
20030	Ncam1	17967	9-:49325611-49606834
20031	Loxl3	16950	6+:82984198-83001317
20032	Aaas	223921	15-:102169072-102186397
20033	MMT00018272		17-:25463496-25464477
20034	9030409G11Rik	71529	4-:141660706-141673118
20035	Zbtb46	72147	2-:181145254-181194131
20036	Olfr1342	258708	4-:118362108-118363055
20037	Extl3	54616	14-:65673184-65697025
20038	MMT00008174		11-:79184763-79185440
20039	Trpc4	22066	3+:53960025-54122389
20040	MMT00056557		2-:8962276-8978376
20041	Fat4	329628	3+:38785862-38908252

20042	Zfp281	226442	1+:138521522-138526627
20043	Grin1	14810	2-:25147767-25174499
20044	Hnrnpa2b1	53379	6-:51412761-51419771
20045	D4Wsu114e	28010	4-:147234900-147242571
20046	MMT00078491		11-:48564219-48567365
20047	Dysf	26903	6+:83958576-84161058
20048	ORF61	216157	10-:79439865-79446894
20049	2310044G17Rik	217732	12+:88288389-88306304
20050	Vps25	28084	11+:101115062-101119361
20051	V1rf5	171236	17-:21130692-21131650
20052	MMT00028786		16-:20797298-20797855
20053	Wnt10a	22409	1+:74838089-74850747
20054	Spcs1	69019	14-:31813013-31814549
20055	Ghrh	14601	2-:157155236-157159528
20056	BC050196	384619	7-:52448829-52451020
20057	BC048651	330277	6+:29270770-29273964
20058	Dnaic1	68922	4+:41516825-41585578
20059	2610528E23Rik	66497	16-:57302112-57606956
20060	MMT00045050		16-:22639990-22703314
20061	EG383436	383436	19+:12921355-12921695
20062	Ankrd50	99696	3-:38352961-38382091
20063	MMT00048272		17-:9513261-9514294
20064	Tpmt	22017	13-:47120546-47138572
20065	Chic1	12212	X+:100551841-100585381
20066	Psmd7	17463	8-:110104280-110112382
20067	Parp11	101187	6+:127403690-127441809
20068	2610204M08Rik	70435	12+:113838204-113853770
20069	EG621542	621542	X-:101577307-101577996
20070	Med13	327987	11-:86083911-86145535
20071	Ccdc95	233875	7-:133995704-134005876
20072	Zfp110	65020	7+:13420411-13435917
20073	Tcp11l1	320554	2-:104521912-104552250
20074	Eral1	57837	11-:77886878-77893847
20075	MMT00049252		16+:15451938-15453322
20076	Dbnl	13169	11+:5688532-5700964
20077	Ccdc115	69668	1-:34493514-34501514
20078	MMT00023360		7-:10045692-10045742
20079	MMT00019254		14-:58054555-58055353
20080	Eya4	14051	10-:22825868-23070592
20081	Slc19a3	80721	1-:83010371-83035023
20082	Slc38a7	234595	8-:98360690-98377410
20083	ENSMUSG00000(	69717	17-:36278702-36279740
20084	Zfp277	246196	12-:41041634-41172365
20085	Lpin2	64898	17+:71533317-71596419
20086	Clk2	12748	3+:88969004-88980517
20087	Cd44	12505	2-:102651301-102696126
20088	Rapgef1	107746	2+:29475306-29593850
20089	MMT00015975		
20090	Slc45a2	22293	15+:10930567-10958065
20091	Cox6b1	110323	7-:31401992-31411170
20092	Matr3	17184	18+:35731677-35732590

20093	Ablim1	226251	19-:57107760-57290289
20094	Olfr1133	258348	2-:87485337-87486278
20095	Eif4h	22384	5-:135095746-135115198
20096	Olfr444	258650	6+:42878371-42906429
20097	Glp2r	93896	11-:67522834-67584374
20098	MMT00046724		Y+:2086608-2097759
20099	MMT00019756		17+:55839839-55840883
20100	Guca1b	107477	17+:47522341-47529914
20101	Kcne2	246133	16+:92292633-92298374
20102	Caskin2	140721	11-:115660692-115673494
20103	Car14	23831	3-:95701723-95708562
20104	BC048679	210321	7-:88639165-88643170
20105	Dkkl1	50722	7-:52462894-52467150
20106	MMT00030605		1+:193916252-193923720
20107	Nphp1	53885	2-:127566468-127614590
20108	Gins4	109145	8-:24337310-24348111
20109	Aldh1a3	56847	7-:73536754-73572311
20110	Zfp640	386626	13+:65988506-66699883
20111	Pdxp	57028	15+:78744458-78749947
20112	Pla2g10	26565	16-:13715156-13730678
20113	Spata22	380709	11+:73153717-73159427
20114	Cndp2	66054	18-:84836861-84855015
20115	M6pr	17113	6+:122262252-122266467
20116	Ctnna3	216033	10+:63713445-63723713
20117	Syt2	20980	1+:136637413-136644853
20118	Tmem175	72392	5+:109058899-109075805
20119	Polr3h	78929	15-:81745459-81756639
20120	MMT00038870	100041430	13+:62375676-62376660
20121	Asb1	65247	1+:93437729-93451450
20122	Tmem86a	67893	7+:54306009-54310146
20123	Ing2	69260	8-:48753877-48760041
20124	1700021F05Rik	67851	10-:43244926-43260800
20125	Olfr568	259095	7+:110025635-110026575
20126	Aoc3	11754	11+:101191918-101200737
20127	Ipo8	320727	6-:148719206-148779959
20128	Frmd5	228564	2+:121632146-121632664
20129	Pdcd1lg2	58205	19+:29485449-29545642
20130	Igh-6	16019	12-:114507636-114663087
20131	Bpgm	12183	6+:34437329-34454474
20132	Cops2	12848	2-:125657337-125684721
20133	Aqp6	11831	15+:99431838-99434323
20134	Ppp1r14c	76142	10-:6881567-6980391
20135	Gramd3	107022	18+:56559990-56662493
20136	Col13a1	12817	10-:61301245-61441856
20137	MMT00022616		3-:86067157-86067494
20138	MMT00054007		11+:75241420-75242154
20139	Plxna4	243743	6-:32100442-32189537
20140	2010002M12Rik	112419	19-:34693304-34697228
20141	Rpl36a	19982	X+:131120224-131122528
20142	2410025L10Rik	381668	5-:110845042-110877894
20143	Abhd13	68904	8+:9977671-9988679

20144	Gk5	235533	9+:96019819-96080608
20145	Glrb	14658	3-:80648052-80717456
20146	Tm2d1	94043	4-:98024384-98062761
20147	Ybx1	22608	4-:118950590-118966411
20148	Pttg1	30939	11-:43233766-43239483
20149	Cabyr	71132	18+:12899857-12913645
20150	Ptprc	19264	1-:139959991-140071843
20151	MMT00052319		1-:17930905-17931239
20152	Tmc5	74424	7+:125770667-125818596
20153	Notch2	18129	3+:97817459-97951371
20154	Trdn	76757	10+:33165224-33194477
20155	Pnldc1	240023	17-:13092398-13101992
20156	Apoc2	11813	7-:20256933-20262111
20157	Ets1	23871	9+:32503878-32562090
20158	Slc38a1	105727	15-:96406706-96454601
20159	Ccm2	216527	11+:6446958-6496745
20160	Hist1h1t	107970	13+:23787679-23788409
20161	Capn5	12337	7-:105272039-105326717
20162	MMT00050725		8-:82634705-82636075
20163	Prr7	432763	13+:55573385-55574512
20164	MMT00004320		4-:34326761-34328211
20165	4933405L10Rik	71046	8+:108232184-108234143
20166	2510012J08Rik	70312	10+:80784016-80788988
20167	Zc3h12b	547176	X+:93094764-93123308
20168	Aard	239435	15+:51871563-51877266
20169	Ralgds	19730	2+:28368850-28408590
20170	MMT00069463		14-:77035238-77068783
20171	1700008G05Rik	75497	3-:10245919-10301183
20172	4930502E18Rik	75013	X+:51027999-51041617
20173	MMT00080451		X+:102677255-102678490
20174	Ctnna2	12386	6-:76831648-77929582
20175	Hoxb2	103889	11+:96214114-96215326
20176	Immt	76614	6+:71781303-71802065
20177	Tex101	56746	7-:25453031-25457061
20178	MMT00081394		6-:52296682-52296997
20179	EG238836	238836	13+:105708758-105709614
20180	Hexdc	238023	11+:121065751-121085469
20181	4930403C10Rik	73813	7+:52977550-52984859
20182	MMT00033340		2-:86165863-86168654
20183	MMT00011814		1+:16043390-16044000
20184	D030016E14Rik	320714	8-:48575482-48618808
20185	Rpl39l	68172	16+:10170320-10175004
20186	Rit1	19769	3+:88520838-88533586
20187	Gphn	268566	12-:79326104-79328751
20188	Cnr2	12802	4+:135451467-135476122
20189	AV026068	102265	8-:59793245-59799627
20190	MMT00019998		15+:71140369-71141202
20191	Clca5	229933	3-:144734164-144762122
20192	Wnt4	22417	4+:136833549-136852693
20193	Olfr771	258540	10-:128597084-128598038
20194	Pnldc1	240023	17-:13081596-13088463

20195	Dpm3	68563	3+:89063279-89071002
20196	Lemd3	380664	10-:120362821-120415310
20197	Igh	111507	12-:116152786-116153243
20198	1200014J11Rik	66874	11+:72861381-72893718
20199	MMT00005037		14-:55969009-56026745
20200	MMT00057474		X-:73625838-73784724
20201	Brd8	78656	18-:34759483-34784216
20202	Elf4	56501	X-:45766415-45777533
20203	Olfr1448	258696	19-:12993852-12994797
20204	MMT00048665		14-:12113447-12274276
20205	Kpna3	16648	14-:61986469-62058628
20206	Sfi1	78887	11-:3030954-3036977
20207	Lif	16878	11+:4166430-4174493
20208	Bloc1s1	14533	10-:128357052-128360561
20209	5730557B15Rik	67434	15-:31226418-31297320
20210	Copb1	70349	7-:121359075-121398133
20211	Pdzk1	59020	3+:96634048-96674848
20212	2300009A05Rik	69478	9-:63242240-63247060
20213	Psd3	234353	8-:70487856-70586173
20214	MMT00082316		14-:18931766-18932622
20215	MMT00044766		4-:144115810-144117020
20216	BC028528	229600	3-:95688882-95695893
20217	Rab3ip	216363	10-:116342836-116376532
20218	MMT00015455		13+:56291929-56349550
20219	MMT00076423		7-:115094858-115096142
20220	MMT00080766	664870	8-:3841083-3842610
20221	Ift57	73916	16+:49699388-49764223
20222	Eef1a2	13628	2-:180882397-180891488
20223	Sc5d	235293	9-:42062195-42072388
20224	Kctd18	51960	1-:58011946-58075802
20225	Kctd17	72844	15+:78259114-78269732
20226	EG623402	623402	19-:8663012-8663686
20227	Kras	16653	6-:145168331-145198729
20228	Gpr27	14761	6+:99642672-99643811
20229	Syne2	319565	12+:77084946-77090315
20230	Ppp1r14b	18938	19+:7050984-7051815
20231	1700071K01Rik	237880	11-:81386159-81386975
20232	Akt3	23797	1-:178955224-179178875
20233	Krt6b	16688	15-:101506960-101524679
20234	Tmtc2	278279	10-:104760607-104850843
20235	Ttc21a	74052	9+:119846764-119876912
20236	Rab43	69834	6-:87741493-87759751
20237	RP23-433P19.11	574418	2-:121276913-121282429
20238	H2-T9	15051	17-:36175353-36179405
20239	Olfr110	258325	17+:37635597-37636549
20240	Bcs1l	66821	1+:74634942-74638760
20241	Cts7	56092	13-:61453821-61459557
20242	Prg3	53856	2+:84828371-84834043
20243	Wfikkn1	215001	17-:26014634-26016988
20244	Grm1	14816	10-:10799636-10802155
20245	Arrb2	216869	11+:70246199-70254322

20246	Pdia5	72599	16-:35397388-35490961
20247	MMT00051075		
20248	BC051628	332713	2+:180954773-180957151
20249	2010107G12Rik	243753	6-:34897507-34929055
20250	Cog5	238123	12+:32339747-32622495
20251	St18	240690	1+:6720036-6848893
20252	MMT00048656		1+:3043866-3044096
20253	Casp2	12366	6+:42215037-42232495
20254	D12Ertd551e	52635	12-:117608805-117608949
20255	Elfn2	207393	15-:78502303-78504775
20256	Rps6ka4	56613	19-:6903574-6915091
20257	Actbl2	238880	13+:112045340-112046471
20258	Prrt1	260297	17+:34766630-34769203
20259	B830045N13Rik	215378	1+:148529688-148749599
20260	Mpz	17528	1+:173085712-173090174
20261	MMT00068974		8+:65018515-65019549
20262	MMT00068012		1+:38964789-38965562
20263	Pus3	67049	9+:35372366-35374723
20264	Ccdc44	70207	11+:105927339-105935303
20265	Rsph1	22092	17-:31391968-31414252
20266	Insig2	72999	1-:123200930-123224339
20267	Lgals1	16852	15+:78757154-78760625
20268	Lyar	17089	5+:38611790-38625536
20269	Hsd17b13	243168	5-:104392479-104406408
20270	Gcm2	107889	13-:41196796-41205552
20271	Igh-V3609N	16057	12-:116113670-116113960
20272	V1rd9	81010	7+:24443334-24445681
20273	4733401H18Rik	66706	9-:108468195-108469673
20274	Klf2	16598	8+:74842931-74845248
20275	Igfbp5	16011	1-:72904611-72921460
20276	Cdcp2	242603	4+:106775073-106779676
20277	Cul4a	99375	8+:13105742-13147775
20278	Clybl	69634	14+:122580947-122801263
20279	Apoa1bp	246703	3-:87860444-87862417
20280	MMT00049741		1+:191992549-191993513
20281	Naip1	17940	13-:101177720-101216466
20282	Gsdma1	57911	11+:98525675-98538188
20283	Atp6v1e2	74915	17-:87343446-87347213
20284	Olfr829	259070	9+:18661070-18662035
20285	Nsd1	18193	13+:55314563-55416241
20286	Btbd7	238386	12-:104022297-104076908
20287	Atp5g2	67942	15-:102493298-102501479
20288	MMT00004355		17-:23053924-23065128
20289	Helt	234219	8-:47377403-47379142
20290	Rabgap1l	29809	1-:162632496-162722916
20291	Rasa2	114713	9-:96442863-96531912
20292	1810048J11Rik	67708	12+:73637365-73643230
20293	MMT00017521		X-:103836719-103838010
20294	Myod1	17927	7+:53631851-53634464
20295	Thsd7a	330267	6-:12266760-12355224
20296	C030027H14Rik	77465	10+:121218048-121218499

20297	Unc13a	382018	8-:74153057-74195622
20298	Rfwd2	26374	1+:161162397-161219172
20299	Lca5l	385668	16-:96395339-96397874
20300	Plekhb1	27276	7-:107792410-107810879
20301	Vmn2r23	435916	6+:123652889-123692011
20302	Siglec15	620235	18-:78242609-78254007
20303	EG272350	272350	7+:5310762-5311470
20304	Ccrk	105278	13+:64533850-64540971
20305	MMT00018250		1+:186747013-186764454
20306	Pcdh11x	245578	X+:117478438-117515506
20307	Catsper4	329954	4-:133767884-133769733
20308	A230106N23Rik	329274	1-:157926028-157927174
20309	MMT00079689		15+:77532357-77533543
20310	Scye1	13722	3-:132323466-132346794
20311	Ptprd	19266	4-:75589848-75990144
20312	Htt	15194	5+:35104373-35252045
20313	Rpusd2	271842	2+:118860523-118865493
20314	Zfp212	232784	6+:47870693-47882626
20315	MMT00072353		8-:41796986-41798641
20316	Adamts14	237360	10-:60661309-60734005
20317	Pex5l	58869	3-:33030492-33042001
20318	MMT00009577		1+:75632119-75633749
20319	Syne2	319565	12+:77026619-77031717
20320	Klf3	16599	5+:65207973-65220415
20321	Ms4a6b	69774	19+:11593118-11604557
20322	Ube2e3	22193	2+:78710638-78760739
20323	Hist1h2ak	319169	13+:21813268-21813744
20324	Tppp	72948	13+:74158590-74169146
20325	4921522P10Rik	66719	8+:8661800-8664728
20326	Zfp715	69930	7-:50551566-50568631
20327	MMT00004978		X+:98714191-98715062
20328	Csl	71832	10-:99220338-99222292
20329	Fars2	69955	13+:36209752-36629461
20330	Foxa3	15377	7-:19598631-19608888
20331	Lsr	54135	7-:31742448-31758463
20332	Stau2	29819	1-:16218886-16221172
20333	Calca	12310	7-:121776811-121779808
20334	MMT00043907		15-:43048504-43049713
20335	Srgn	19073	10-:61957175-61970503
20336	MMT00037968	436100	6+:149514609-149514717
20337	St6galnac4	20448	2+:32442602-32455214
20338	Sav1	64010	12-:71066001-71087989
20339	OTTMUSG00000C	626316	4+:146064815-146094022
20340	Slfn14	237890	11-:83092652-83097665
20341	Srebf2	20788	15+:81977675-82035810
20342	Cerkl	228094	2-:79172726-79178778
20343	Ttc19	72795	11+:62094983-62128748
20344	Grb10	14783	11-:11833466-11870619
20345	AI842396	103844	11-:70501861-70513646
20346	Gna-rs1	14670	17+:36117622-36125811
20347	2310009B15Rik	69549	1-:140748563-140753409

20348	EG210853	210853	17-:22065153-22426864
20349	MMT00054511		9-:65888767-65890437
20350	Pigc	67292	1+:163899357-163902461
20351	Tfdp2	211586	9+:96096719-96174366
20352	Slitrk1	76965	14-:109310405-109312494
20353	Antxr1	69538	6-:87083847-87285436
20354	Crip3	114570	17+:46565890-46568719
20355	Zfyve28	231125	5-:34541416-34630852
20356	Mrps35	232536	6+:146991325-147019421
20357	Serpinb12	71869	1+:108831025-108853655
20358	Rwdd2a	69519	9+:86465659-86468503
20359	Spata3	70060	1+:87918527-87926533
20360	Olfr1085	258583	2-:86497673-86498613
20361	Mtmr14	97287	6+:113187854-113231377
20362	Sh3bp5l	79566	11+:58145048-58161228
20363	Dcun1d2	102323	8-:13255963-13288167
20364	Zfp422	67255	6-:116574935-116578939
20365	Grid1	14803	14+:35633351-36394083
20366	Olfr1167	258291	2-:87989223-87990174
20367	Rgs14	51791	13+:55471252-55486044
20368	Iqsec3	243621	6-:121326087-121423581
20369	OTTMUSG00000(	381531	4-:61789431-61811841
20370	Sccpdh	109232	1+:181606521-181617318
20371	Scnm1	69269	3-:94933639-94937935
20372	Heatr1	217995	13+:12524927-12531159
20373	Klhl14	225266	18-:21708881-21810869
20374	Zfp780b	338354	7-:28695189-28790337
20375	Gje1	118446	5-:138398440-138399216
20376	Dnajc10	66861	2+:80159310-80194200
20377	Grik1	14805	16-:87912660-88289860
20378	Cyp2t4	384724	7+:27940706-27943582
20379	MMT00002289		X-:12954992-12955666
20380	MMT00058516		11+:70171425-70174036
20381	Vtn	22370	11+:78312787-78315825
20382	Olfr1423	258675	19-:12110299-12111056
20383	MMT00072890		17+:34221635-34222767
20384	BC034069	100038347	7+:80885194-80921804
20385	Chd1l	68058	3-:97364670-97414113
20386	Sdk1	330222	5+:142411997-142648557
20387	Defb1	13214	8+:22887081-22905191
20388	Olr1	108078	6-:129435269-129457172
20389	Clcn5	12728	X-:6736673-6765840
20390	Cd9	12527	6-:125410283-125444711
20391	Oas1h	246729	5+:121311430-121323512
20392	Gja4	14612	4-:126989210-126990212
20393	1110018G07Rik	68497	12-:86259100-86284342
20394	EG668225	668225	15-:100882896-100884841
20395	Syt8	55925	7+:149622738-149626210
20396	Myt1l	17933	12+:30213430-30605597
20397	Sema3e	20349	5+:14025316-14254314
20398	Prh1	19131	6+:132521906-132521950

20399	Josd1	74158	15+:79504681-79505454
20400	Kif7	16576	7-:86842597-86859044
20401	Lztr1	66863	16+:17509067-17526423
20402	MMT00007367		16+:3391231-3391269
20403	MMT00024375		14+:38535407-38536343
20404	MMT00003113		19-:45471254-45472111
20405	Clip1	56430	5-:124029082-124062172
20406	Cacng2	12300	15-:77825392-77949710
20407	Praf2	54637	X+:7305696-7308189
20408	Muc15	269328	2+:110561649-110572080
20409	EG628904	628904	7-:52045568-52046165
20410	MMT00028069		9-:88169814-88176034
20411	Mpg	268395	11+:32126458-32132024
20412	MMT00011980		9-:113233368-113261490
20413	Wdr86	269633	5-:24218083-24218639
20414	MMT00017740		17-:35415087-35416147
20415	6-Mar	223455	15-:31385655-31425957
20416	Cnn1	12797	9+:21903711-21913169
20417	Trappc9	76510	15-:72420050-72587793
20418	Pvr	52118	7-:20490400-20504259
20419	Adra1b	11548	11-:43589362-43650305
20420	Krt33b	16671	11-:99884950-99891190
20421	Cdkn2c	12580	4-:109333751-109338267
20422	MMT00035096		12+:111065431-111066329
20423	8-Mar	71779	6+:116288399-116359556
20424	Map2k4	26398	11-:65503147-65601750
20425	Zbtb11	271377	16+:55973992-56008993
20426	Tex9	21778	9-:72308908-72358729
20427	Rps6ka1	20111	4-:133403208-133443699
20428	MMT00026261		6-:47736147-47738382
20429	Tmem202	73893	9-:59366461-59373259
20430	Trio	223435	15-:27660409-27849046
20431	Slc7a7	20540	14-:54989127-55027885
20432	Smc1b	140557	15-:84895118-84962387
20433	Cdca5	67849	19+:6085098-6091773
20434	Lrp3	435965	7-:35985917-35998208
20435	Cyp4x1	81906	4-:114781286-114806582
20436	Mctp1	78771	13+:76521982-76598910
20437	Gimap3	83408	6-:48714467-48720850
20438	Wac	225131	18-:7928068-7933174
20439	Frg1	14300	8-:42482818-42502428
20440	MMT00078132		3+:34082230-34082983
20441	Olfr1034	258216	2+:85886874-85887572
20442	Spag4	245865	2+:155890945-155895235
20443	Tnfaip1	21927	11-:78338807-78343734
20444	Ckap2l	70466	2-:129094653-129122947
20445	MMT00010910		1+:148267549-148268382
20446	Acadl	11363	1-:66877867-66909823
20447	Ltc4s	17001	11-:50049973-50051973
20448	Pom121	107939	5-:135852012-135870375
20449	MMT00062045		3+:98624862-98626457

20450	Tnrc18	231861	5-:143201452-143550438
20451	Agr2	23795	12+:36719511-36730667
20452	Kcna6	16494	6-:126687640-126690857
20453	Zfp235	56525	7+:24919218-24928256
20454	MMT00018457		2-:36929746-36938002
20455	Sympk	68188	7+:19637685-19639961
20456	Bcap31	27061	X-:70931518-70960225
20457	Gtf2a2	235459	9+:69860348-69870672
20458	Dync2h1	110350	9-:6928501-7137110
20459	MMT00030019		2+:82347278-82348437
20460	Emcn	59308	3+:137004106-137094032
20461	H2-Ab1	14961	17+:34400256-34405429
20462	Lrrtm2	107065	18-:35369584-35374573
20463	AA536717	105428	14+:21167380-21202714
20464	EG622645	622645	17+:69189667-69191715
20465	V1re6	171229	17+:20872063-20873088
20466	Zdhhc16	74168	19+:42008004-42018585
20467	BC026590	230234	4+:56815222-56819844
20468	Spa17	20686	9-:37410885-37421199
20469	MMT00040453		4-:99096893-99097952
20470	Scml4	268297	10+:42580672-42678541
20471	MMT00002448		18+:9909144-9910503
20472	A030009H04Rik	80515	11+:69154318-69156141
20473	Gpr137b-ps	664862	13-:12707324-12742647
20474	Gnb3	14695	6-:124784257-124790293
20475	Dbh	13166	2+:27021035-27038315
20476	Slc35e3	215436	10-:117170537-117171737
20477	Csn1s2a	12993	5+:88203591-88217822
20478	MMT00009155		5-:16014199-16014689
20479	Lipm	78753	19+:34175420-34197833
20480	V1ri8	252908	13-:22236389-22237394
20481	Tmem106b	71900	6+:13019855-13035064
20482	Vwa1	246228	4-:155143412-155148640
20483	Vgll3	73569	16+:65815877-65839984
20484	Ces1	12623	8-:95826294-95861093
20485	6430520M22Rik	319716	6+:145809702-145811100
20486	C1qtnf1	56745	11+:118295166-118311276
20487	MMT00061855		10-:26393342-26394792
20488	Bok	51800	1+:95582278-95592339
20489	MMT00032252		2+:90081278-90081580
20490	Tmem143	70209	7+:53152444-53172781
20491	1700125H20Rik	73634	11+:84983903-84994646
20492	Glra3	110304	8+:58419700-58604145
20493	Ankrd43	237761	11-:53291763-53301048
20494	Tmem115	56395	9+:107436275-107440987
20495	Tmem41b	233724	7-:117115707-117129774
20496	Mtpn	14489	6-:35461919-35489819
20497	Ubr2	224826	17-:47153518-47153986
20498	Porcn	53627	X-:7770977-7783627
20499	Wdr44	72404	X+:23270284-23383107
20500	2810441K11Rik	68642	19-:10624955-10630728

20501	Ccl1	20290	11-:81990167-81993314
20502	Mtus1	102103	8-:42076264-42127039
20503	Krtdap	64661	7+:31572915-31576114
20504	MMT00033407		X+:147122692-147124227
20505	Ttll9	74711	2+:152788241-152833076
20506	1700011F03Rik	74221	6-:70863080-70868918
20507	Sh2d6	71130	6-:72463636-72470621
20508	Hic2	58180	16+:17233674-17260457
20509	C130092O11Rik	232035	6+:61260806-61373073
20510	Prame	75829	X-:132147539-132162246
20511	Decr2	26378	17-:26218155-26227109
20512	MMT00005340	93684	3+:144233442-144260638
20513	Il1rapl2	60367	X+:134709065-134771192
20514	Rprm	67874	2-:53936647-53937952
20515	Dimt1	66254	13+:107737253-107749840
20516	Mafa	378435	15-:75577380-75577632
20517	AK129128	218343	13+:76236242-76327774
20518	Mtnr1b	244701	9-:15667110-15678904
20519	Col1a1	12842	11+:94797565-94814353
20520	EG546143	546143	9-:72827529-72833594
20521	BC057079	230393	4+:87823985-88056913
20522	MMT00054229		10+:56375999-56397773
20523	Rhox2h	622301	X-:35083503-35087786
20524	Cdc27	217232	11-:104381584-104411792
20525	Olfr281	258277	15+:98286742-98287677
20526	MMT00056393		7-:18222787-18232578
20527	Actr1b	226977	1-:36756048-36766770
20528	Etnk1	75320	6+:143115768-143152135
20529	Nf1	18015	11+:79197269-79395110
20530	EG640268	640268	9+:48228150-48240042
20531	MMT00015606		3-:69123941-69124507
20532	Rnf26	213211	9-:43919758-43921558
20533	Pon3	269823	6-:5171509-5206226
20534	Stk24	223255	14-:121686020-121778284
20535	Rbm4	19653	19-:4785153-4793860
20536	V1rd1	81017	7-:5753953-5757047
20537	Mmp13	17386	9+:7272544-7283330
20538	Nek10	238944	14+:15763763-15770301
20539	Klk1b5	16622	7+:51471828-51476082
20540	Rps6kl1	238323	12-:86476591-86492186
20541	Vapa	30960	17-:65929393-65962836
20542	H2-M1	224756	17-:36807185-36809142
20543	MMT00020117		8+:40402541-40541667
20544	4932438A13Rik	229227	3+:36762046-36776660
20545	Skp2	27401	15-:9042165-9070073
20546	Lrrc15	74488	16-:30272660-30283339
20547	Pde2a	207728	7+:108599820-108661330
20548	1700041C02Rik	73332	4-:118996446-119004224
20549	Itsn2	20403	12+:4620083-4720002
20550	Nrip1	268903	16-:76290984-76331013
20551	Fzd5	14367	1-:64781416-64783174

20552	Cdh9	12565	15+:16707815-16786045
20553	Echdc2	52430	4+:107838100-107851912
20554	Numa1	101706	7+:109149689-109163472
20555	Mlf2	30853	6+:124882097-124885436
20556	Chst11	58250	10+:82448242-82655099
20557	Snw1	66354	12-:88790859-88813229
20558	EG624180	624180	13+:62136547-62137653
20559	Nat13	72117	16+:44139996-44160092
20560	Mef2c	17260	13-:83803698-83806683
20561	MMT00002184		X-:8643144-8643462
20562	E130309F12Rik	272031	4+:49219251-49420669
20563	Mns1	17427	9+:72286341-72306381
20564	Olfr433	258712	1+:175972082-175973025
20565	MMT00065821		7-:27254261-27255255
20566	Tnni2	21953	7+:149628372-149630310
20567	Ggt6	71522	11+:72249027-72252065
20568	Luc7l2	192196	6+:38501518-38559444
20569	MMT00063637		7+:11817129-11822371
20570	Ap3m1	55946	14-:21854031-21871407
20571	Tmem45a	56277	16-:56806491-56885976
20572	Rdh5	19682	10-:128350648-128356250
20573	MMT00040001		3+:101037410-101038015
20574	Apool	68117	X+:109424971-109486297
20575	MMT00068533		5+:93850712-93852176
20576	1700066M21Rik	73467	1+:57434514-57442260
20577	Dner	227325	1-:84366414-84442194
20578	MMT00014841		13-:50587858-50588527
20579	Zkscan3	72739	13-:21479544-21499920
20580	Cdk9	107951	2-:32562959-32568249
20581	V1rc7	113864	6+:57183432-57184344
20582	MMT00026692		17+:53499221-53501373
20583	Cdh16	12556	8-:107138047-107148161
20584	Sirt4	75387	5-:115928022-115934307
20585	Snx7	76561	3-:117484577-117549625
20586	Afmid	71562	11+:117687237-117700337
20587	Abcc1	17250	16+:14361688-14473660
20588	Brd2	14312	17-:34248976-34256255
20589	Frmd8	67457	19-:5851285-5875230
20590	Actl7a	11470	4+:56756326-56757792
20591	Ilk	16202	7+:112885261-112891432
20592	Kcnc1	16502	7+:53652911-53683814
20593	Ptbp1	19205	10+:79317424-79327516
20594	Kdelr1	68137	7+:53128209-53139096
20595	Ero1l	50527	14-:45903995-45938202
20596	Fkbp10	14230	11+:100277010-100286139
20597	EG226604	226604	1-:168564870-168573610
20598	Ptgs2	19225	1+:151947229-151954943
20599	Gsto2	68214	19+:47940292-47960795
20600	Epha10	230735	4+:124559118-124592677
20601	Rpo1-4	20019	6+:71859156-71929352
20602	Prnpip1	140546	4+:117222989-117346901

20603	Hcn3	15168	3-:88950698-88964079
20604	Luc7l	66978	17+:26389903-26417664
20605	MMT00047084		14-:79578596-79584131
20606	MMT00042166		7-:26205542-26208082
20607	Cdc5l	71702	17-:45528832-45570687
20608	Zbtb26	320633	2-:37290539-37298688
20609	Aftph	216549	11-:20585095-20641584
20610	Ttll11	74410	2-:35606760-35835059
20611	Map2k3	26397	11+:60745558-60766297
20612	OTTMUSG00000C	545667	4-:108049951-108056261
20613	4930431B09Rik	74645	3-:100275528-100293078
20614	Gna15	14676	10-:80965426-80986741
20615	Olfr706	258350	7-:114029380-114030329
20616	9030404E10Rik	71528	16-:30021926-30039567
20617	MMT00077828		12+:53096148-53096573
20618	Ctnna1	12385	18+:35278565-35414427
20619	Mmp1a	83995	9+:7464186-7476416
20620	MMT00013477		1-:175398434-175417003
20621	MMT00001928		9+:15448768-15450324
20622	Cd47	16423	16+:49855836-49911192
20623	Vmn2r55	100042499	7-:13237054-13256130
20624	Utp15	105372	13+:99016769-99018856
20625	Pgam2	56012	11-:5701646-5703736
20626	Nfxl1	100978	5-:72904542-72915468
20627	5530400B04Rik	71405	2-:155654584-155660589
20628	Hoxd10	15430	2+:74530036-74532425
20629	Ctns	83429	11-:72997104-73012508
20630	Lsamp	268890	16+:41889024-41955476
20631	Grm1	14816	10-:10408553-10502593
20632	Cep78	208518	19-:16029841-16059370
20633	Thra	21833	11+:98614596-98630184
20634	Pias1	56469	9-:62729775-62828678
20635	Jmjd6	107817	11-:116698746-116704689
20636	MMT00055765		1+:135061034-135066666
20637	MMT00078257		6-:149298790-149302300
20638	Med12	59024	X+:98469599-98492792
20639	MMT00003860		6+:41231374-41232091
20640	4930432E11Rik	243900	7+:30359630-30362134
20641	Icam1	15894	9+:20820478-20832389
20642	V1rd7	81012	7+:24416996-24419385
20643	Rcvrn	19674	11+:67508827-67516835
20644	Lct	226413	1-:130181333-130224880
20645	Zfp575	101544	7-:25370456-25396609
20646	BC031781	208768	1+:182781281-182798242
20647	Zcchc18	66995	X+:133527716-133530917
20648	B430212C06Rik	338360	18-:67482672-67484375
20649	5830472M02Rik	76080	2+:163428082-163442847
20650	Naaladl1	381204	19+:6105797-6116009
20651	MMT00006027		5-:61212852-61213676
20652	Dlx4	13394	11-:95001764-95006795
20653	1700049L16Rik	108950	10-:71442840-71443382

20654	Maea	59003	5+:33678258-33715162
20655	MMT00045504		
20656	MMT00068363		13+:31101103-31105812
20657	MMT00059805		7+:78841754-78841979
20658	EG623757	623757	7+:149110287-149128751
20659	Cryba4	12959	5-:112675552-112681522
20660	Dmpk	13400	7+:19669343-19678450
20661	Tuft1	22156	3-:94416680-94462728
20662	MMT00066519		14-:56796839-56797941
20663	Depdc1a	76131	3+:159158415-159192917
20664	Rpl13a	22121	7-:52381292-52384102
20665	Caln1	140904	5+:130924670-131316044
20666	Mosc1	66112	1-:186610654-186635182
20667	MMT00054056		
20668	Rras	20130	7+:52273382-52277014
20669	EG216185	216185	10+:82417072-82417821
20670	Klhl25	207952	7+:82993277-83012331
20671	Clk4	12750	11+:51076699-51094932
20672	MMT00046375		7-:119001458-119002089
20673	MMT00007807		8+:40367602-40367914
20674	D17H6S53E	114585	17+:35264059-35265424
20675	MMT00070017		13+:9222766-9224294
20676	MMT00067648		11+:6346185-6346571
20677	MMT00062099		8-:59759790-59770682
20678	Cyp39a1	56050	17+:43804482-43887796
20679	Ifna12	242519	4-:88248655-88249212
20680	Xpo4	57258	14-:58201109-58283793
20681	EG667683	667683	6-:69388204-69388768
20682	Usp40	227334	1-:89846587-89905109
20683	Foxd4	14237	19-:24973454-24975799
20684	Twist1	22160	12+:34642676-34644691
20685	Cacna1f	54652	X+:7184229-7212319
20686	MMT00042548		7+:149281224-149351612
20687	Txnrd2	26462	16+:18426509-18479160
20688	Iyd	70337	10-:6791663-6806262
20689	Cnksr3	215748	10+:3134785-3227478
20690	Zfp276	57247	8+:125779841-125793635
20691	Armcx6	278097	X-:131283826-131285932
20692	Fryl	72313	5-:73411427-73647908
20693	Ssr1	107513	13+:38063269-38069327
20694	Tbc1d30	74694	10-:120702542-120748052
20695	Brd9	105246	13+:74075252-74098341
20696	Kif12	16552	4-:62826670-62833169
20697	Galnt5	241391	2+:57850800-57891637
20698	Lman2	66890	13-:55446204-55463965
20699	Senp7	66315	16+:56075578-56126652
20700	Acot2	171210	12+:85328851-85334030
20701	Rpl36al	66483	12-:70283729-70285027
20702	Mbtps1	56453	8-:122032055-122071464
20703	Itga11	319480	9+:62525751-62631795
20704	Atp2a3	53313	11+:72774812-72805437

20705	Supv3l1	338359	10-:61891966-61913355
20706	Exosc10	50912	4+:147932566-147956504
20707	Ranbp6	240614	19-:29884123-29887440
20708	Phf15	76901	11-:51630264-51662569
20709	Enpp6	320981	8+:48072324-48074096
20710	Prpsap2	212627	11-:61543158-61554506
20711	E430028B21Rik	211948	14-:27487724-27488939
20712	1700012B15Rik	74173	12+:3235510-3241409
20713	1700054N08Rik	73420	8-:126364729-126383388
20714	MMT00015505		5+:6674890-6749370
20715	Tmem179b	67706	19-:8847018-8848932
20716	Tmem151a	381199	19-:5081769-5085372
20717	Snf8	27681	11+:95896230-95908719
20718	Fmo4	226564	1-:164724013-164742680
20719	Sall1	58198	8-:91552278-91566326
20720	Ptk2b	19229	14-:66772098-66899831
20721	4432412L15Rik	74337	8+:86545367-86554191
20722	Olfr429	258717	1+:176019172-176020110
20723	Cdh1	12550	8+:109127267-109194146
20724	Mdn1	100019	4+:32822192-32825745
20725	Nudt5	53893	2+:5775859-5789410
20726	Ei24	13663	9-:36586743-36604653
20727	Mmp11	17385	10-:75385968-75395208
20728	MMT00055001		
20729	Ttc28	209683	5+:111717228-111717889
20730	Dusp26	66959	8+:32200120-32207520
20731	2010100O12Rik	67067	2+:155969953-155971529
20732	MMT00004153	100045669	15-:91856199-91858037
20733	H1f0	14958	15+:78858905-78860266
20734	Alg6	320438	4+:99382309-99413104
20735	Tnrc6b	213988	15+:80541488-80577903
20736	Itgb1bp1	16413	12-:21273280-21292080
20737	ORF28	246738	16-:91615975-91619238
20738	MMT00010890		13+:14611270-14688167
20739	Cabp5	29865	7+:13983543-13993318
20740	Ola1	67059	2-:72930859-73052504
20741	Mepe	94111	5+:104754394-104767575
20742	Birc2	11797	9-:7818226-7835255
20743	Trp53inp1	60599	4+:11083587-11101524
20744	Ipmk	69718	10+:70810582-70844783
20745	Sord	20322	2+:122060574-122091073
20746	2210015D19Rik	76508	11+:5662128-5684713
20747	Olfr1099	258764	2-:86798674-86799613
20748	OTTMUSG00000(	665229	X+:34996289-34998578
20749	Usp1	230484	4+:98590620-98602227
20750	Foxo4	54601	X+:98449866-98455033
20751	Jmjd2d	244694	9-:14267624-14269004
20752	4930420K17Rik	652925	5+:9100664-9120508
20753	C330006K01Rik	231855	5+:142939476-142950466
20754	Crisp1	11571	17-:40430708-40456122
20755	Mmp25	240047	17-:23767535-23781138

20756	Omd	27047	13+:49678115-49687978
20757	Suclg1	56451	6+:73198666-73226876
20758	Crtam	54698	9-:40780880-40812669
20759	Slc17a1	20504	13+:23962182-23987568
20760	Bnip3l	12177	14-:67606071-67627676
20761	Caml	12328	13+:55724429-55733758
20762	Fkbp9	27055	6+:56782069-56829351
20763	Tnfsf9	21950	17+:57244854-57246926
20764	Vit	74199	17+:78907512-79026729
20765	MMT00060789		13-:62771158-62772324
20766	V1ra2	22297	6-:89809361-89890501
20767	Dhx8	217207	11+:101618900-101628669
20768	Traf3ip3	215243	1-:195000787-195027689
20769	Ube2e1	22194	14-:19115262-19164313
20770	Mettl4	76781	17-:95125275-95147258
20771	Slc16a10	72472	10-:39754934-39862065
20772	Timd4	276891	11+:46629015-46631284
20773	Cttnbp2	30785	6-:18383982-18464823
20774	Atp6v1c2	68775	12-:17291526-17331518
20775	Cd244	18106	1+:173489382-173507477
20776	Nudcd2	52653	11+:40547169-40553546
20777	MMT00010185		7-:5078280-5080880
20778	Arf3	11842	15-:98570731-98593520
20779	Tmem194	210035	10+:127114145-127133995
20780	Zfp219	69890	14-:52625758-52634712
20781	Cyp11a1	13070	9+:57862823-57874830
20782	Kcnma1	16531	14-:24119243-24823152
20783	Olfr689	258745	7+:112462519-112463480
20784	Csad	246277	15-:102007428-102019474
20785	BC051070	229688	3-:106191609-106207274
20786	Bspry	192120	4+:62141100-62158332
20787	Cabp2	29866	19+:4083505-4087337
20788	Rbm43	71684	2-:51780103-51790407
20789	Il17rd	171463	14+:27905114-27914158
20790	Efna5	13640	17-:62956667-63230493
20791	MMT00014813	667370	19+:34682173-34687127
20792	MMT00032790		5-:42890113-42891653
20793	Figf	14205	X+:160811479-160840578
20794	MMT00003639		4+:11327468-11328470
20795	C330001K17Rik	77609	9-:21794313-21797525
20796	E130113E03Rik	319433	14+:63282502-63283949
20797	Dlc1	50768	8-:37632586-37676934
20798	Ccpg1	72278	9+:72833391-72864140
20799	EG619750	619750	3+:91828416-91828953
20800	MMT00007848		3+:75195607-75196036
20801	Mrps11	67994	7+:85928195-85937874
20802	Cdc42se2	72729	11-:54531687-54601187
20803	Tmem50a	71817	4-:134453760-134470915
20804	Eno1	13806	4+:149611363-149622984
20805	D730005E14Rik	109361	15-:79720842-79722749
20806	V1rg2	171237	7+:12341605-12342516

20807	MMT00017315		19+:61227777-61227862
20808	Bms1	213895	6-:118333397-118369433
20809	Nefh	380684	11-:4838760-4848116
20810	Mttp	17777	3-:137753849-137794288
20811	Dlst	78920	12+:86451788-86475795
20812	Olfr467	257919	7+:114958099-114959019
20813	1700049E17Rik	73415	14+:42386499-42394594
20814	Ptpn18	19253	1+:34516602-34530622
20815	MMT00047851		2+:143682053-143707243
20816	Foxi3	504196	6+:70906602-70911057
20817	EG667414	667414	1+:136005924-136006417
20818	Gnb5	14697	9+:75159201-75192456
20819	MMT00004365		11-:18225730-18227675
20820	Fbxo41	330369	6-:85425387-85434017
20821	Slco3a1	108116	7-:81429125-81699564
20822	Smurf1	75788	5-:145637396-145663456
20823	Cradd	12905	10-:94637379-94786731
20824	Cd68	12514	11-:69477873-69479564
20825	D10Ertd641e	52717	10-:59450656-59465858
20826	EG384486	384486	6-:124031714-124060668
20827	Sccpdh	109232	1+:181598358-181600826
20828	MMT00007878		8-:40805672-40806165
20829	Zfp385c	278304	11-:100490145-100499206
20830	MMT00057509		16+:53983940-53986320
20831	MMT00057242		X+:133424796-133425379
20832	Stard9	668880	2+:120527790-120541818
20833	MMT00022580		
20834	C80913	19777	7-:38745949-38767967
20835	Pgrmc1	53328	X+:34138269-34146067
20836	Park2	50873	17+:11033278-12254698
20837	Dlgap4	228836	2+:156547622-156588828
20838	MMT00058389		19+:34698453-34699140
20839	Nefm	18040	14-:68738095-68742870
20840	Rasa1	218397	13-:85355633-85396069
20841	Def6	23853	17+:28292811-28365554
20842	MMT00046069		6-:148647645-148648158
20843	Arf2	11841	11+:103828086-103845595
20844	MMT00051561		3-:6164627-6167555
20845	Gars	353172	6+:54987960-55029496
20846	EG667952	667952	14-:56039106-56088250
20847	Pdik1l	230809	4-:133832574-133843142
20848	Tceal7	68496	X+:132758579-132760638
20849	Wrn	22427	8-:34346162-34495969
20850	Rusc1	72296	3-:88887902-88897234
20851	Rpusd3	101122	6-:113365325-113369575
20852	Extl2	58193	3+:115711249-115731878
20853	Tatdn1	69694	15-:58721707-58765370
20854	Commd7	99311	2-:153444550-153458492
20855	Phldb2	208177	16-:45746356-45953710
20856	Mybl1	17864	1-:9659956-9690035
20857	Vmn2r10	22307	5-:109422430-109435490

20858	Duox1	99439	2+:122143654-122173706
20859	BC063263	434178	7-:49730359-49838309
20860	Gm1564	268491	11+:102536537-102542051
20861	Dcst1	77772	3-:89161394-89169177
20862	Olfr827	258297	10-:129647217-129648184
20863	Ccdc83	75338	7-:97384270-97413942
20864	MMT00062115		7-:149761435-149763609
20865	Appl1	72993	14+:27732688-27733297
20866	2900073C17Rik	73020	8+:49928797-49929962
20867	Cnih	12793	14-:47395256-47408206
20868	A230106M20Rik	210094	7-:50731258-50736029
20869	Nudcd3	209586	11-:6005714-6100413
20870	1700028K03Rik	76421	5+:107926739-107940230
20871	Pop1	67724	15+:34425077-34460392
20872	MMT00077363		19-:3316427-3317608
20873	Arfgef2	99371	2+:166631214-166721495
20874	Cyp2j6	13110	4-:96184443-96220319
20875	Olfr1335	435804	4-:118481460-118482443
20876	Zfp239	22685	6+:117820444-117822782
20877	Adal	75894	2+:120966164-120982404
20878	MMT00047858		17-:74731301-74731869
20879	Klk1b26	16618	7+:51268047-51272329
20880	Raf1	110157	6-:115568615-115626521
20881	1110059G10Rik	66202	9-:122856655-122860113
20882	MMT00013663		
20883	Gpr175	24100	6+:88855938-88862223
20884	MMT00017334		
20885	MMT00004673		6+:23687067-23688033
20886	Mylpf	17907	7+:134356373-134357776
20887	MMT00060807		7-:111840853-111841877
20888	MMT00076579		7+:73335446-73336263
20889	S1pr3	13610	13+:51504147-51515629
20890	Psmb4	19172	3-:94688014-94690862
20891	2810055F11Rik	67217	12-:73174414-73186300
20892	C1r	50909	6+:124462649-124473124
20893	Calb1	12307	4+:15808429-15832710
20894	Cyp4a12a	277753	4+:114971704-115005419
20895	MMT00009700		15+:82729583-82736246
20896	Rasa3	19414	8-:13567217-13677572
20897	Clca6	99663	3-:144615450-144638010
20898	Bcl2l2	12050	14+:55502261-55507069
20899	0610037L13Rik	74098	4+:107562503-107570407
20900	Epb4.1l4a	13824	18-:33956614-34165788
20901	MMT00043221		15-:74643886-74647626
20902	Itln1	16429	1-:173448372-173461345
20903	Rpia	19895	6-:70715694-70742149
20904	Urm1	68205	2+:29682903-29699582
20905	MMT00004364		4-:117401222-117402041
20906	Tpbpb	116913	13-:61002548-61006208
20907	Lims2	225341	18+:32091171-32118272
20908	Olfr583	258752	7+:110199813-110200772

20909	Rnf215	71673	11+:4035204-4041175
20910	MMT00014676		8+:107511030-107520108
20911	Narg1	74838	3+:51246688-51277946
20912	Chordc1	66917	9+:18096710-18118444
20913	Osgepl1	72085	1+:53370519-53382993
20914	Utx	22289	X+:17799871-17857060
20915	MMT00016910		X+:106684015-107106737
20916	MMT00023465		8+:71853024-71853615
20917	Gstm6	14867	3-:107742146-107746551
20918	Nxph3	104079	11-:95371166-95375585
20919	Ceacam18	72431	7+:50890075-50904280
20920	5730412P04Rik	70491	X+:132639138-132649958
20921	BC016579	212998	16-:45626960-45654231
20922	Mxd3	17121	13-:55426841-55431106
20923	Prkra	23992	2-:76467995-76486051
20924	MMT00030481	545291	19-:42863436-43065771
20925	2810410L24Rik	76377	11-:120048945-120097489
20926	Amotl1	75723	9-:14351788-14447975
20927	MMT00002557		18+:7765902-7806511
20928	MMT00006812		3+:64400827-64409738
20929	MMT00053572		10-:39115519-39164664
20930	Stc1	20855	14+:69647446-69656558
20931	Ythdf3	229096	3+:16083277-16105424
20932	2610207I05Rik	233789	7-:125274818-125387151
20933	Dclk2	70762	3-:86590080-86635709
20934	Ect2	13605	3-:26996148-27052758
20935	Gpr83	14608	9+:14664697-14673943
20936	Supt7l	72195	5-:31817406-31825634
20937	Olfr494	258732	7+:115511005-115511948
20938	Unkl	74154	17+:25326321-25368111
20939	Il13	16163	11-:53444825-53448204
20940	4930455B06Rik	223825	15+:4894947-4912199
20941	Zfp28	22690	7+:6238228-6349624
20942	MMT00006980		12-:119970961-119993457
20943	1700113H08Rik	76640	10+:86668719-86693331
20944	Tmem164	209497	X+:139115999-139117492
20945	Traf4	22032	11-:77972902-77979033
20946	Nufip2	68564	11+:77420825-77526241
20947	Neurod4	11923	10-:129705206-129708468
20948	Cstf2t	83410	19+:31157493-31161080
20949	Tmem195	319660	12+:37968337-38132624
20950	8030462N17Rik	212163	18-:77873879-77952725
20951	Pkhd1	241035	1-:20556838-20557670
20952	Zdhhc9	208884	X-:45525661-45561861
20953	Btaf1	107182	19+:37019701-37088545
20954	5830403L16Rik	240817	1-:155697254-155747360
20955	Dnaja3	83945	16+:4684114-4706678
20956	Pbx3	18516	2-:34027428-34227556
20957	Arhgef5	54324	6+:43223588-43239320
20958	4933403F05Rik	108654	18-:68425430-68459989
20959	Lrrc8d	231549	5+:106240744-106243323

20960	Abcc3	76408	11-:94204604-94254229
20961	Lig3	16882	11+:82594724-82615690
20962	Txndc4	76299	4-:48207238-48292430
20963	Ifna6	15969	4+:88473251-88474201
20964	MMT00038797		4+:12041130-12054397
20965	Usp28	235323	9+:48808455-48850621
20966	MMT00014378		4+:69514980-69515718
20967	Fabp2	14079	3+:122598295-122602423
20968	Ube2cbp	70348	9-:86200840-86358539
20969	Slc9a3r2	65962	17-:24776232-24787223
20970	Slco6b1	67854	1-:98802752-98885276
20971	Tsga13	116732	6-:30846980-30865573
20972	MMT00031355		2-:35346729-35421930
20973	Stx2	13852	5-:129490441-129514447
20974	Col4a3bp	68018	13+:97312700-97407505
20975	Actrt2	73353	4-:154040543-154041820
20976	MMT00057291		13+:13205616-13211075
20977	MMT00039950		15-:8479135-8479788
20978	MMT00069660		14+:54485596-54566510
20979	D10Jhu81e	28295	10-:77624617-77632516
20980	Taf12	66464	4+:131830327-131849240
20981	4933408B17Rik	271508	18-:34739227-34745985
20982	Pomt2	217734	12-:88447820-88480685
20983	Hlx	15284	1-:186551024-186556487
20984	MMT00036444		18-:47574120-47575031
20985	Fcer2a	14128	8-:3681736-3694174
20986	Olfr805	258548	10-:129159638-129160598
20987	Fez1	235180	9+:36640392-36686508
20988	MMT00010339		18-:48228593-48229474
20989	MMT00007956		12-:116532196-116532715
20990	3300002I08Rik	69277	2-:150136672-150188501
20991	2900093K20Rik	68037	2+:163244447-163246095
20992	Dedd	21945	1+:173268446-173271334
20993	Snx13	217463	12+:35731873-35829228
20994	Pcdhb17	93888	18+:37644812-37647943
20995	Tfrc	22042	16+:32613276-32630420
20996	Osmr	18414	15-:6763589-6824283
20997	MMT00028135	631323	11+:57997341-58002440
20998	Irx1	16371	13-:72095399-72100972
20999	1110049F12Rik	66193	4-:135531518-135542320
21000	Vmo1	327956	11-:70327044-70328104
21001	Slc9a7	236727	X-:19682826-19868913
21002	Olfr862	258555	9-:19687827-19688747
21003	Uckl1	68556	2-:181303852-181327780
21004	MMT00013593		7+:77635832-77636747
21005	Wdfy2	268752	14+:63456757-63576109
21006	Spry2	24064	14-:106291733-106296036
21007	Rbp2	19660	9+:98390974-98410012
21008	4933436I01Rik	66780	X-:65173038-65174625
21009	Msto1	229524	3-:88713565-88717822
21010	Dyrk1a	13548	16+:94833014-94916917

21011	Clasp2	76499	9+:113650590-113828431
21012	MMT00064569		
21013	Vnn3	26464	10+:23571277-23589637
21014	Usp54	78787	14-:21368139-21380894
21015	MMT00014048		19+:17615518-17636722
21016	AW146020	330361	6+:81873851-81882911
21017	Gstz1	14874	12-:88495797-88495928
21018	Eif3m	98221	2-:104839812-104857184
21019	Picalm	233489	7+:97279031-97357434
21020	Rgmb	68799	17-:15944108-15955445
21021	Npas2	18143	1+:39251056-39419031
21022	MMT00001569		5+:23532691-23558340
21023	5730559C18Rik	67313	1-:138120139-138124241
21024	Zkscan6	52712	11+:65620676-65642740
21025	1500005A01Rik	85308	14-:56200360-56204091
21026	Vstm2a	211739	11+:16157774-16184188
21027	Gabre	14404	X-:69502770-69520060
21028	Tmem209	72649	6-:30431229-30444838
21029	MMT00015031		4-:103872648-104067460
21030	Ccdc80	67896	16+:45093786-45128001
21031	Slc44a5	242259	3+:153906493-153921473
21032	2310016D23Rik	76443	1+:60835653-60849426
21033	Gzmk	14945	13-:113962082-113971105
21034	Cts8	56094	13-:61349241-61356713
21035	4933427E13Rik	71234	11+:25226104-25267139
21036	Rhoc	11853	3+:104591969-104597377
21037	Nufip1	27275	14+:76510739-76534893
21038	Exosc7	66446	9+:123022362-123045243
21039	Pcnxl2	270109	8-:128275430-128421981
21040	4930533K18Rik	75189	10+:70331388-70387215
21041	Cyp17a1	13074	19-:46741672-46747363
21042	Tbx6	21389	7+:133924996-133929061
21043	Gns	75612	10+:120802147-120834301
21044	Zfyve20	78287	6-:92136714-92164938
21045	Crb3	224912	17+:57201692-57205335
21046	4921517D21Rik	67722	9+:107830799-107834792
21047	Morf4l1	21761	9-:89986508-90009507
21048	Cdrt4	66338	11+:62764694-62806597
21049	1700129I04Rik	330577	7-:89783078-89794339
21050	MMT00072914		3-:124745178-124746241
21051	Coq4	227683	2+:29643785-29653262
21052	Marcksl1	17357	4+:129190887-129193218
21053	Ypel1	106369	16+:17070321-17086434
21054	EG620395	620395	16+:3730509-3731025
21055	Gltp	56356	5-:115120415-115140870
21056	Tmem92	544806	11-:94640567-94643988
21057	Pja1	18744	X-:96661454-96666011
21058	MMT00013752	100044420	4-:88245080-88245777
21059	Cabp7	192650	11-:4638823-4646730
21060	Ela1	109901	15-:100504853-100518353
21061	Mdh1b	76668	1-:63745401-63766155

21062	Rpl7l1	66229	17-:46912049-46919595
21063	MMT00007639		6+:48931099-48931889
21064	Mea1	17256	17+:46818085-46820052
21065	MMT00048282		3-:140402038-140604181
21066	Gfod1	328232	13-:43295693-43399787
21067	MMT00077109		5-:105170617-105181507
21068	Fbxo10	269529	4-:45048783-45097394
21069	Tet2	214133	3-:133148475-133150801
21070	Cnot8	69125	11+:57917730-57931348
21071	Il4i1	14204	7+:52091658-52096178
21072	MMT00015730		1-:29541368-29542780
21073	Zfp51	22709	17+:21589397-21602551
21074	Gata2	14461	6+:88143884-88157024
21075	Gtf2h5	66467	17+:6079816-6085478
21076	Abra	223513	15-:41697235-41701251
21077	6430537H07Rik	226265	19+:59006387-59045903
21078	MMT00067065		10-:29803972-29805141
21079	Tfpi2	21789	6-:3912595-3918354
21080	MMT00035581		13+:28163933-28180873
21081	Mapk13	26415	17+:28906317-28915649
21082	E330009J07Rik	243780	6-:40356363-40386124
21083	Serpinb9c	20707	13-:33241846-33249693
21084	Nkiras2	71966	11+:100484268-100488916
21085	MMT00021687		9-:114556710-114557164
21086	Stat5b	20851	11-:100644293-100711798
21087	Mepce	231803	5-:138223134-138226186
21088	MMT00055569		1-:96365291-96366482
21089	MMT00048642		1-:146069047-146070255
21090	Fbxo18	50755	2-:11664200-11699154
21091	Nup160	59015	2+:90517371-90576484
21092	Zfp750	319530	11-:121372290-121375361
21093	Lyrm2	108755	4+:32887245-32889227
21094	A730046J19Rik	319764	X+:140588263-140596777
21095	9130401M01Rik	75758	15-:57853830-57865772
21096	Olfr958	258327	9-:39357515-39358454
21097	1700055N04Rik	73458	19+:3958806-3964578
21098	Terf2ip	57321	8+:114535304-114544360
21099	Rapgef6	192786	11+:54251798-54508080
21100	MMT00065179		
21101	1110032A03Rik	68721	9-:50571831-50576209
21102	Cmtm7	102545	9-:114665954-114690947
21103	Onecut2	225631	18+:64500627-64546284
21104	Ppp2r2a	71978	14-:67689069-67691274
21105	Pex26	74043	6+:121133905-121146203
21106	Il1f5	54450	2+:24132918-24137595
21107	Qtrtd1	106248	16-:43863142-43881326
21108	Fndc3a	319448	14-:72937757-73109800
21109	Col8a2	329941	4+:125986859-125991185
21110	Emg1	14791	6-:124654404-124662145
21111	Tmem132c	208213	5+:128031872-128045272
21112	Pigb	55981	9-:72863502-72887506

21113	MMT00028787		16+:20820417-20821085
21114	Ints7	77065	1+:193399645-193447570
21115	MMT00082318		14-:19376517-19377691
21116	Lrrc10	237560	10+:116482410-116483820
21117	mCG_124640	624652	2+:38829917-38832192
21118	D17Wsu92e	224647	17+:27921322-27922077
21119	Apob	238055	12+:7984453-8023641
21120	Igk-C	16071	6+:70613261-70676963
21121	5730590G19Rik	77011	7+:86805118-86805888
21122	Fndc7	320181	3-:108683854-108692717
21123	1700012I11Rik	69334	15+:67058328-67208655
21124	Hoxb5	15413	11+:96164825-96167434
21125	Mtif3	76366	5-:147766504-147775350
21126	Dera	232449	6+:137703061-137805860
21127	Triobp	110253	15+:78813509-78836300
21128	Olfr76	258677	19-:12194211-12194923
21129	Odz4	23966	7+:103319753-104055760
21130	MMT00011706	66449	16-:4616465-4624946
21131	Mlstd1	330450	6+:148087056-148123728
21132	Pdss2	71365	10+:42941291-43160407
21133	Treml2	328833	17+:48441887-48447724
21134	Ppp2r2a	71978	14-:67634163-67657817
21135	Gm711	279029	2+:26806073-26809014
21136	Golga1	76899	2-:38871672-38921007
21137	Smad1	17125	8-:81863377-81896117
21138	Auts2	319974	5-:132503558-132526282
21139	Olfr461	258380	6-:40494034-40494976
21140	Prdx2	21672	8+:87493598-87498214
21141	Asb15	78910	6+:24478143-24520984
21142	Gm129	229599	3-:95682425-95686173
21143	Ypel3	66090	7+:133920488-133924023
21144	Lhfpl3	269629	5+:22608229-22608707
21145	Gm784	333564	X-:103555704-103642981
21146	Rhoa	11848	9+:108208825-108239251
21147	Bcl7a	77045	5+:123794514-123822855
21148	MMT00044214		14+:35329353-35329766
21149	A230065H16Rik	380787	12+:112645018-112650301
21150	Rbm17	76938	2-:11506506-11524832
21151	Nkap	67050	X+:34666806-34688277
21152	Gpx5	14780	13-:21378301-21384555
21153	Mr1	15064	1-:156975029-156993821
21154	MMT00009510		11-:30654557-30668836
21155	Clec4b1	69810	6+:123000023-123021562
21156	Bmper	73230	9+:23028002-23058915
21157	Efemp1	216616	11+:28767772-28826733
21158	Casp14	12365	10-:78174741-78180903
21159	Pik3c2g	18705	6+:139564832-139668705
21160	Irs2	384783	8-:10984687-11008770
21161	Sox2	20674	3+:34549337-34550296
21162	MMT00008728		3-:12384339-12662887
21163	Slc16a5	217316	11+:115323818-115335706

21164	Tmem104	320534	11-:115107814-115108338
21165	Hmbs	15288	9-:44144376-44150429
21166	A930029G22Rik	654806	17-:69766732-69788671
21167	Irf5	27056	6+:29476732-29487319
21168	Olfr498	258304	7+:115608839-115609831
21169	MMT00015992		
21170	D6Wsu176e	27999	6-:22257890-22306081
21171	1700016G05Rik	67690	6-:40464822-40469507
21172	Rgl3	71746	9-:21776249-21793856
21173	Rassf6	73246	5-:91032120-91069501
21174	Nhlh1	18071	1-:173982423-173987682
21175	1700025I17Rik	70372	13+:53489005-53550188
21176	Arl15	218639	13+:114712448-114757999
21177	Trpv6	64177	6-:41570623-41586297
21178	MMT00020279		10-:104307609-104311543
21179	Olfr288	545140	15-:98037844-98048622
21180	Arhgef1	16801	7+:25688074-25711608
21181	MMT00021170		5+:11192522-11193468
21182	Runx2	12393	17-:44744854-44951576
21183	Zfp142	77264	1-:74612387-74636663
21184	Svs1	243377	6+:48940928-48941731
21185	MMT00008840		Y+:992142-993278
21186	4930451G09Rik	74684	16+:4964379-4978176
21187	RP23-54E4.8	625752	13-:21388116-21388587
21188	Pnkp	59047	7+:52112773-52118020
21189	Arl5c	217151	11-:97850895-97857486
21190	Senp5	320213	16-:31963227-31990520
21191	Znrf1	170737	8+:114060843-114148194
21192	2310047A01Rik	71918	14-:26531130-26588357
21193	Lbh	77889	17+:73267644-73288909
21194	Zfp641	239652	15-:98118903-98124234
21195	Tbc1d7	67046	13-:43247112-43266728
21196	MMT00072944	622480	4+:129626030-129629104
21197	Smtn	29856	11-:3417525-3439242
21198	Mbl1	17194	14+:41964744-41972246
21199	Pcdh9	211712	14-:93414732-93429790
21200	Phex	18675	X-:153596617-153853210
21201	MMT00014061		15+:101469004-101470430
21202	Acly	104112	11-:100337671-100389186
21203	C330018D20Rik	77422	18-:57115697-57133141
21204	1700001L19Rik	69315	13+:68736312-68752165
21205	Pkmyt1	268930	17+:23863407-23873704
21206	Supt3h	109115	17+:45060126-45256238
21207	Pi15	94227	1+:17592075-17615527
21208	MMT00077807		X-:151585483-151585859
21209	Ebf4	228598	2+:130121763-130196587
21210	Plaur	18793	7+:25247528-25260489
21211	2010301N04Rik	72103	6-:87579740-87622058
21212	Klb	83379	5+:65739649-65775242
21213	Tdrd1	83561	19+:56916758-56941130
21214	Scd1	20249	19-:44472409-44481899

21215	Mep1b	17288	18+:21230844-21258681
21216	Whsc1	107823	5+:34163500-34240608
21217	2010309E21Rik	66488	6+:86315717-86320051
21218	Col9a2	12840	4+:120711936-120727903
21219	9930039A11Rik	320040	11+:68702109-68706452
21220	Myd116	17872	7-:52778289-52781583
21221	Otud6b	72201	4-:14739599-14753565
21222	Fig4	103199	10-:40907977-41023047
21223	Pira1	18722	7-:3788417-3867086
21224	MMT00039633		8+:88348200-88348461
21225	Zfp449	78619	X+:53601457-53616391
21226	Arid5b	71371	10-:67560371-67740935
21227	MMT00016115		2+:150817917-150818530
21228	A430107D22Rik	320484	17-:32527175-32540328
21229	Acss1	68738	2-:150443846-150493965
21230	Ppp2r5c	26931	12-:111717443-111723861
21231	Cnr1	12801	4+:34030588-34032011
21232	Nkiras1	69721	14+:19103798-19112954
21233	Rmi1	74386	13+:58502763-58503877
21234	Olfr665	258810	7+:112029222-112030172
21235	Ttn	22138	2-:76666542-76667387
21236	Glrx5	73046	12+:106270898-106279120
21237	Poli	26447	18-:70668337-70689975
21238	BC068110	383103	16-:10422689-10447372
21239	Ctdp1	67655	18-:80604703-80637598
21240	Dock11	75974	X+:33428865-33616556
21241	Fcrl1	229499	3+:87190339-87207889
21242	Zfp654	72020	16-:64765058-64794382
21243	BC019943	234138	8+:32261102-32270558
21244	Nfatc1	18018	18-:80845197-80904863
21245	Dock2	94176	11-:34501010-34597354
21246	Telo2	71718	17-:25236521-25252720
21247	Tmed5	73130	5-:108550665-108561610
21248	Slc25a3	18674	10-:90579536-90586712
21249	MMT00056913		X-:71739035-71740292
21250	Asb10	117590	5-:24038992-24046275
21251	Cpt1a	12894	19+:3323278-3385732
21252	Dnajc12	30045	10+:62849364-62871567
21253	MMT00068187		4-:105808498-105810353
21254	Bbs7	71492	3-:36474214-36512313
21255	Kif18a	228421	2+:109120894-109181901
21256	Tirap	117149	9-:34994369-35007459
21257	MMT00009771		7-:106395146-106397764
21258	2010003K15Rik	75606	10-:25247749-25256077
21259	5430416O09Rik	71406	4+:43742922-43744950
21260	Ropn1	76378	16+:34666781-34678566
21261	Pax2	18504	19+:44831883-44835478
21262	Myot	58916	18+:44493727-44515376
21263	Thrb	21834	14+:18814088-18866287
21264	Rn18s	19791	
21265	Zfp712	78251	13-:67140520-67162107

21266	Cercam	99151	2+:29725012-29738368
21267	3110056O03Rik	73218	10+:80318075-80331454
21268	MMT00025084		X-:38646510-38648239
21269	A1cf	69865	19+:31943260-32021998
21270	V1rg6	171241	7+:12465691-12466607
21271	Pax9	18511	12+:57796657-57812216
21272	Scnn1g	20278	7+:128878020-128911990
21273	St14	19143	9-:30896992-30939273
21274	BC005624	227707	2-:30828356-30837406
21275	MMT00041364		15+:92799335-92800701
21276	Saps3	52036	19-:3454930-3575738
21277	Eif5a	276770	11-:69730215-69734874
21278	MMT00072454		19-:8586378-8587816
21279	Stk38l	232533	6+:146706970-146725074
21280	Olfr555	259107	7+:109807336-109808284
21281	Zfp111	56707	7-:24981895-24988602
21282	Slc6a8	102857	X+:70918488-70927837
21283	Prl3b1	18776	13+:27334298-27341547
21284	Cep110	26920	2+:34982885-35010909
21285	Psg22	243862	7+:19304793-19312594
21286	Zmat2	66492	18+:36953581-36958313
21287	Cplx4	225644	18-:66115377-66129832
21288	Tcp11l2	216198	10+:84039691-84077096
21289	Dbi	13167	1-:122009975-122017592
21290	Ttl	69737	2+:128891710-128919489
21291	Slc39a4	72027	15-:76442814-76447322
21292	Trdmt1	13434	2-:13432724-13466278
21293	Fau	14109	19+:6057950-6059518
21294	Sphkap	77629	1-:83253016-83404686
21295	Snf1lk	17691	17-:31981201-31992723
21296	Rpl14	67115	9+:120480633-120483770
21297	Gcnt7	654821	2-:172276565-172280402
21298	Psg17	26437	7-:19399342-19406956
21299	Pcdh19	279653	X+:130077915-130118104
21300	Hdac10	170787	15-:88953735-88959624
21301	Slc26a2	13521	18-:61356507-61371136
21302	Scg5	20394	2-:113616521-113669226
21303	Kcnk2	16526	1-:191031815-191167711
21304	Prcp	72461	7+:100023781-100082255
21305	Acmsd	266645	1+:129625971-129664151
21306	Pvrl1	58235	9+:43552726-43612097
21307	Krcc1	57896	6+:71232273-71235312
21308	Lcorl	209707	5-:46113601-46247777
21309	Olfr768	258863	10-:128530091-128531028
21310	MMT00046273		
21311	Zscan4-ps1	545912	7-:11653844-11655064
21312	C430003P19Rik	109359	7+:140050920-140076792
21313	Rhou	69581	8+:126177828-126187782
21314	Mycbp2	105689	14-:103512626-103745734
21315	A430005L14Rik	97159	4+:153331368-153336031
21316	MMT00058095	385049	13+:13337651-13368062

21317	Paip1	218693	13+:120217728-120246096
21318	MMT00000812		7+:34420180-34482717
21319	Krt15	16665	11-:99995487-99995616
21320	Kcne1l	66240	X-:138739295-138740741
21321	Acoxl	74121	2+:127680363-127949609
21322	Uspl1	231915	5+:149996289-150027009
21323	MMT00074228		X-:76876748-76877213
21324	Ssna1	68475	2-:25126560-25127929
21325	Shb	230126	4-:45436150-45543625
21326	MMT00047910		5-:39792387-39793484
21327	Camk2d	108058	3+:126300357-126543283
21328	MMT00040005		X+:100684816-100685271
21329	Azi2	27215	9+:117949801-117972689
21330	Abcc12	244562	8-:89028485-89082223
21331	Slc10a6	75750	5-:104034729-104058422
21332	6330439K17Rik	241688	2-:144301825-144353135
21333	Cirbp	12696	10+:79630585-79634398
21334	Dio3	107585	12+:111517444-111518354
21335	Trp53rk	76367	2+:166619296-166621652
21336	Lck	16818	4-:129225593-129250851
21337	Ldlrap1	100017	4-:134301336-134323858
21338	Lcn3	16820	2+:25621088-25623619
21339	Glb1l3	70893	9-:26625612-26668241
21340	Armc1	74252	3-:19033754-19063079
21341	Zfp59	22717	7+:28631421-28641995
21342	Tmco2	69469	4-:120778262-120781797
21343	Cryz	12972	3+:154259825-154286147
21344	Iqub	214704	6-:24395447-24400852
21345	Hells	15201	19+:39005446-39042746
21346	Pcsk5	18552	19-:17749904-17911931
21347	Gjd2	14617	2-:113836765-113838870
21348	MMT00039685		X-:136210588-136211975
21349	MMT00066665		X-:57735009-57735926
21350	Lce1e	68694	3-:92511322-92512996
21351	Shisa5	66940	9+:108941140-108960236
21352	Dtnbp1	94245	13-:45017577-45097341
21353	Tbc1d5	72238	17-:50875407-51164532
21354	Xpa	22590	4-:46188113-46209183
21355	Cyb5d2	192986	11-:72596149-72609521
21356	Nkx2-6	18092	14+:69789789-69793597
21357	Cd3g	12502	9-:44777771-44788507
21358	Jarid2	16468	13+:44826206-45015476
21359	Rabl4	67042	15-:77989894-78004532
21360	Sox11	20666	12-:28024667-28027561
21361	9530082P21Rik	638247	17+:23886185-23887153
21362	Cxcr4	12767	1-:130485421-130488786
21363	Mrrf	67871	2+:35991907-36032794
21364	Ppm1h	319468	10+:122219238-122379594
21365	Igh	111507	12-:116067451-116089871
21366	2610304G08Rik	70470	2+:157854229-157903055
21367	1700057K13Rik	73435	1-:159029277-159038782

21368	Pccb	66904	9-:100882456-100935294
21369	Cd4	12504	6-:124814710-124838227
21370	MMT00081548	100043861	6-:128655853-128663633
21371	Spnb2	20742	11-:30007625-30098164
21372	Gm1661	381544	4-:116885939-116910446
21373	MMT00077926		14-:35007630-35009003
21374	Gal3st1	53897	11+:3896911-3899315
21375	MMT00043149		13+:81203605-81204605
21376	MMT00058413		3-:112133818-112134721
21377	MMT00055233		19+:20996875-21026557
21378	Havcr2	171285	11+:46268436-46294754
21379	Rnf121	75212	7-:109167649-109213654
21380	Ier5	15939	1-:156944656-156946766
21381	Rilpl2	80291	5-:124913275-124928244
21382	Aste1	66595	9+:105299250-105307791
21383	MMT00003576		4-:25449652-25469072
21384	Alg13	67574	X+:140752565-140759731
21385	BC094916	545384	1-:175450080-175466084
21386	Pcbp3	59093	10-:76224601-76424533
21387	Cnot7	18983	8-:41579081-41595524
21388	Gbe1	74185	16+:70314380-70569959
21389	Odf2	18286	2+:29745619-29778056
21390	Cnga2	12789	X+:69248605-69255573
21391	Lgi2	246316	5-:52929102-52957519
21392	Crx	12951	7-:16452576-16465257
21393	Rai2	24004	X+:158155100-158217789
21394	Cdh15	12555	8+:125371865-125391297
21395	Mast4	328329	13-:103847920-103961637
21396	4930451I11Rik	78118	7-:133973980-133974994
21397	Nt5dc3	103466	10+:86267525-86297227
21398	Tmco6	71983	18+:36894798-36902045
21399	MMT00055421	675238	10-:77315396-77325187
21400	Il1rapl1	331461	X-:84545923-84550289
21401	Ankrd11	77087	8-:125407222-125498693
21402	Pigv	230801	4-:133218168-133228533
21403	MMT00010875		18-:75340262-75342540
21404	Hoxa10	15395	6-:52181195-52184938
21405	Slc39a11	69806	11-:113111766-113167274
21406	Lrp2	14725	2-:69262392-69276531
21407	Slc25a46	67453	18-:31741243-31769559
21408	Svil	225115	18+:4920535-5119286
21409	Gldc	104174	19-:30172938-30249908
21410	Calr4	108802	4+:108908016-108927235
21411	Lenep	57275	3-:89205920-89206579
21412	Afap1l1	106877	18-:61889915-61946343
21413	4922501C03Rik	382090	9-:87087550-87150371
21414	BC017643	217370	11-:121083904-121090579
21415	Has1	15116	17-:17980292-17992152
21416	D030018L15Rik	402773	15+:95903628-95906402
21417	Psmb6	19175	11+:70338937-70341124
21418	Nr3c2	110784	8+:79609425-79766444

21419	Giyd2	75764	7-:133834191-133839153
21420	Smarcad1	13990	6+:64992523-65066042
21421	Usp34	17847	11+:23332245-23390534
21422	Ric8b	237422	10+:84380486-84420857
21423	Foxl2	26927	9+:98855961-98858959
21424	Supt3h	109115	17+:44914119-44923683
21425	Rab6	19346	7+:107756300-107788704
21426	4930590J08Rik	381798	6+:91866247-91900057
21427	Tc2n	74413	12-:103074865-103151474
21428	MMT00012296		13-:18213671-18214236
21429	Ptpra	19262	2+:130276275-130379759
21430	Pes1	64934	11+:3864017-3879218
21431	Xbp1	22433	11+:5420969-5425873
21432	Olfr531	258955	7-:147586029-147586943
21433	Ubxd6	108159	8-:34730660-34753816
21434	Nhs	195727	X-:158274746-158280359
21435	V1rc1	113858	6+:57063418-57064352
21436	Toe1	68276	4-:116476609-116480164
21437	Irg1	16365	14+:103446190-103455312
21438	Plekha4	69217	7+:52781698-52809598
21439	Il25	140806	14+:55551607-55554671
21440	Tmsb10	19240	6-:72907346-72908393
21441	MMT00041291		6+:100676497-100677620
21442	Rad51l1	19363	12+:80401535-80915338
21443	Lax1	240754	1-:135575633-135586594
21444	Pnpt1	71701	11+:29030744-29061595
21445	Kap	16483	6-:133799872-133803688
21446	Ccl12	20293	11+:81915379-81916793
21447	Adcyap1r1	11517	6+:55402288-55447164
21448	Paqr7	71904	4+:134052892-134064725
21449	Serpinb3a	20248	1-:108942592-108948916
21450	Col22a1	69700	15-:71628899-71837618
21451	Gja5	14613	3+:96854551-96855627
21452	Tgfb1i1	21804	7+:135391531-135397225
21453	Vmn2r108	627805	17-:20599846-20609335
21454	MMT00068006		1+:38295963-38302553
21455	MMT00002739		11+:100104573-100105006
21456	Artn	11876	4-:117599198-117600288
21457	MMT00036264		1+:62707381-62708331
21458	Nr1h5	381463	3-:102753354-102758810
21459	Hpse	15442	5-:101109419-101148700
21460	MMT00008970	631037	4-:115254589-115255749
21461	MMT00034426		17-:22016905-22018029
21462	Klhl28	66689	12-:66043428-66066521
21463	Gulp1	70676	1+:44608537-44845719
21464	Pop5	117109	5+:115688043-115690975
21465	Nxn	18230	11-:76072066-76212562
21466	Rimbp2	231760	5-:129266555-129325722
21467	Senp3	80886	11-:69486613-69495204
21468	Slc44a4	70129	17+:35051431-35067180
21469	Nsdhl	18194	X+:70171674-70202952

21470	Lrrc18	67580	14+:33804567-33827546
21471	Stmn2	20257	3+:8509526-8561604
21472	Olfr340	258953	2+:36308106-36309044
21473	BC048403	270802	10+:121177001-121188566
21474	Crispld1	83691	1+:17717502-17754829
21475	Olfr836	258557	9+:18925400-18926353
21476	Pds5b	100710	5+:151518974-151613277
21477	Tmem27	57394	X+:160528118-160556791
21478	Loxhd1	240411	18+:77580666-77613937
21479	Steap2	74051	5-:5671724-5677285
21480	Igf1r	16001	7+:75309871-75372722
21481	Bcl2a1c	12046	9+:114239273-114239658
21482	Plscr4	235527	9+:92367739-92385751
21483	Ippk	75678	13+:49516638-49558430
21484	Zc3hc1	232679	6-:30316384-30341015
21485	Pfdn2	18637	1+:173275839-173288236
21486	Vmn2r1	56544	3+:63885563-63909380
21487	1810063B07Rik	67509	14-:20896343-20902363
21488	MMT00080701		11+:98587506-98588329
21489	Slc38a11	320106	2-:65160723-65202086
21490	Gnaz	14687	10+:74430020-74478693
21491	MMT00017308		X-:166637383-166637413
21492	Dvl2	13543	11+:69814127-69823611
21493	EG385328	385328	X+:13788237-13788776
21494	Nr1d2	353187	14-:19038936-19071587
21495	Olfr656	259078	7+:111766170-111767158
21496	Ereg	13874	5+:91503647-91522672
21497	Pias2	17344	18+:77336331-77391865
21498	C920005C14Rik	338368	15+:82171624-82174508
21499	Slfn3	20557	11+:83004802-83038131
21500	MMT00018393		16+:68511115-68511943
21501	Stmn1	16765	4+:134026575-134029316
21502	Csk	12988	9-:57474721-57478818
21503	Srp72	66661	5+:77403733-77428931
21504	Dnajc17	69408	2-:118998242-119034510
21505	Acsbg2	328845	17-:56982526-57007795
21506	St7	64213	6+:17699232-17892965
21507	Glt8d2	74782	10-:82113176-82153397
21508	Echdc1	52665	10+:29033429-29065463
21509	Tekt3	71062	11+:62875158-62908462
21510	Tmem16c	228432	2-:110498084-110725202
21511	MMT00054100		
21512	Gas6	14456	8-:13465375-13494506
21513	Ccdc66	320234	14-:28295822-28299807
21514	Rfk	54391	19+:17468572-17475825
21515	MMT00013736	625574	4+:88863120-88863720
21516	Crkl	12929	16+:17452236-17483959
21517	Sil1	81500	18-:35426050-35658520
21518	Adamts17	233332	7+:74269810-74295255
21519	1700012B15Rik	74173	12+:3235767-3235829
21520	Hsd17b12	56348	2-:93873693-93998045

21521	Prdx4	53381	X-:151758463-151772997
21522	MMT00007903		8+:43008873-43012428
21523	Galntl1	108760	12+:81620020-81704879
21524	Ncoa4	27057	14+:32978989-32991030
21525	Txlna	109658	4-:129303322-129318269
21526	Mkl1	223701	15-:80842713-81020954
21527	Rnf144a	108089	12-:26995417-27100113
21528	Nup85	445007	11+:115431296-115445298
21529	Card6	239319	15-:5049913-5058447
21530	Ogdhl	239017	14+:33140164-33160039
21531	Ttc23	67009	7+:74792335-74871462
21532	Akap9	100986	5+:4070291-4080200
21533	BC057022	433940	5+:115017437-115062113
21534	Rnf121	75212	7-:109168378-109213654
21535	Bmp10	12154	6+:87379026-87384486
21536	MMT00011639		17-:37306653-37307591
21537	MMT00014241		3+:14098101-14099213
21538	Rg9mtd1	52575	16-:56033832-56037887
21539	Rbm11	224344	16+:75593188-75601499
21540	Coil	12812	11+:88833933-88834428
21541	Brf2	66653	8-:28233055-28239108
21542	Larp4	207214	15+:99803129-99846782
21543	BC064033	208164	6-:35263432-35276113
21544	MMT00004338		17-:49449519-49487258
21545	Pcdha9	192161	18+:37157533-37347311
21546	Orm2	18406	4+:63023520-63026911
21547	Il10	16153	1+:132916423-132921547
21548	MMT00052315		2+:24034090-24036471
21549	Rtp4	67775	16+:23610021-23613819
21550	Sgcd	24052	11-:46792765-47193024
21551	Bmf	171543	2-:118358294-118372743
21552	MMT00031646		12-:109679116-109679553
21553	EG628438	628438	18+:47271792-47272216
21554	EG627607	627607	12+:88081891-88095385
21555	Sgsm2	97761	11-:74678587-74710383
21556	4930579F01Rik	67741	3-:137827043-137849678
21557	Gabrr1	14408	4+:33219530-33250563
21558	Tgfb1	21803	7+:26472020-26490015
21559	Rnf219	72486	14-:104876753-104878430
21560	Apol7e	666348	15+:77542747-77548355
21561	BC050196	384619	7-:52444703-52445476
21562	Mocs3	69372	2+:168056119-168057516
21563	Arhgap9	216445	10+:126760787-126767000
21564	Cdh18	320865	15+:23366204-23404173
21565	Pde6h	78600	6+:136907781-136911793
21566	MMT00047754		X+:8536916-8560306
21567	5830417I10Rik	76022	3+:88650290-88667534
21568	Cdc73	214498	1-:145454629-145549814
21569	Rbx1	56438	15+:81296785-81305690
21570	Gpr116	224792	17+:43497366-43593662
21571	D030074E01Rik	75964	18-:20975727-21054592

21572	Ankrd13a	68420	5+:115254401-115255825
21573	U90926	57425	5-:92638920-92644434
21574	Acot11	329910	4-:106420109-106472362
21575	Cyp2c55	72082	19+:39081508-39117177
21576	Pigk	329777	3+:152377068-152448887
21577	6330408A02Rik	321008	7-:13844316-13864034
21578	P2ry12	70839	3-:59020281-59024181
21579	Lsm12	268490	11-:102023809-102046566
21580	MMT00027671	639930	12-:116170561-116175628
21581	Cbx3	12417	6+:51425235-51432603
21582	Setx	269254	2+:29000906-29003997
21583	Gstz1	14874	12+:88488724-88505671
21584	Pde7a	18583	3-:19125917-19164957
21585	Hipk1	15257	3-:103547595-103595102
21586	Palm	18483	10+:79256353-79282967
21587	MMT00002799		5+:69852287-69854009
21588	Snrpa	53607	7-:27972024-27981078
21589	Sdc2	15529	15+:32850477-32964476
21590	Rpl41	67945	10-:127985334-127986031
21591	Hectd3	76608	4+:116667964-116677875
21592	Wdr46	57315	17+:34077682-34086360
21593	8430427H17Rik	329540	2-:153296421-153355498
21594	Hbxip	68576	3+:107081774-107086997
21595	Trpv1	193034	11+:73051727-73074008
21596	P2rx5	94045	11+:72974026-72986187
21597	Yeats2	208146	16+:20150514-20230977
21598	Olfr353	258943	2-:36745430-36746366
21599	AI462493	107197	19-:8954509-8955427
21600	MMT00069173		5-:93800966-93804715
21601	MMT00052061		1-:45945745-45947227
21602	V1ra5	113847	6-:89819507-89820448
21603	Nfatc2	18019	2-:168305027-168415761
21604	Klhl24	75785	16+:20097641-20123303
21605	Uevld	54122	7-:54203014-54213842
21606	Prkcq	18761	2+:11094008-11222853
21607	Aldh1a1	11668	19+:20676457-20717944
21608	Tpcn2	233979	7-:152439963-152444960
21609	MMT00039978		X+:100232943-100233745
21610	Gpx1	14775	9+:108241606-108242747
21611	1700085A12Rik	78631	X+:36699441-36700135
21612	MMT00080634		5-:69423728-69424341
21613	MMT00033404		X-:148196545-148197026
21614	A230107C01Rik	320225	7-:29975745-29976724
21615	Dip2c	208440	13+:9492341-9536111
21616	Sh3pxd2a	14218	19-:47341402-47538899
21617	MMT00050850		18-:82885510-82895572
21618	MMT00041235		6+:90848276-90856207
21619	MMT00060699		7+:107093648-107118866
21620	Tmem166	232146	6+:81991666-82043092
21621	MMT00071794		17-:37977249-37978796
21622	Sprr2i	20763	3+:92212637-92212867

21623	Gpr98	110789	13-:81731551-81772338
21624	Ube2b	22210	11-:51799704-51813865
21625	MMT00008928		7+:69083122-69083724
21626	1700023L04Rik	76419	6+:29910992-29945290
21627	Fcgr3	14131	1-:172981325-172989466
21628	Slc35e1	270066	8-:75007664-75016500
21629	MMT00017186		8-:77929978-77999042
21630	Ahcyl2	74340	6+:29718497-29720618
21631	Diap2	54004	X+:126338548-126623312
21632	MMT00046455		9+:108062719-108063800
21633	Krtap9-1	16705	11+:99734753-99735313
21634	MMT00042049		
21635	Hs6st2	50786	X-:48742232-49033576
21636	1700127D06Rik	76999	7+:51200381-51201297
21637	Cpxm1	56264	2-:130216511-130223310
21638	MMT00011534		6-:83652125-83654013
21639	Hspa9	15526	18-:35097088-35113977
21640	Hspa14	50497	2-:3406125-3430018
21641	Gm1966	434223	7-:113742570-113747549
21642	E130309D14Rik	432582	11+:74433403-74452114
21643	Agtr1a	11607	13+:30428307-30474735
21644	MMT00024277		1+:90047612-90048456
21645	Slc22a6	18399	19+:8692604-8701011
21646	Dnajb7	57755	15-:81237517-81238703
21647	Rnf13	24017	3+:57540055-57637152
21648	MMT00038910		13-:63548239-63548693
21649	MMT00076721		10+:72664358-72666028
21650	Ap4b1	67489	3+:103613552-103625946
21651	Glipr1l2	67537	10+:111520409-111545154
21652	Olfr700	258593	7-:113949025-113949974
21653	MMT00081761		3+:74618728-74619289
21654	MMT00066017		15-:82482420-82484072
21655	Rab40b	217371	11-:121218116-121249453
21656	MMT00054267		11-:7083159-7086362
21657	Etaa1	68145	11-:17839655-17853878
21658	Oxt	18429	2+:130401945-130402667
21659	Hydin	244653	8+:113056575-113073687
21660	Il1rn	16181	2+:24192378-24205150
21661	Spag17	74362	3+:99879649-99885962
21662	A530088I07Rik	212167	5+:20692130-20797531
21663	Vta1	66201	10-:14375136-14425366
21664	Insig1	231070	5+:28397977-28405203
21665	Nat5	67877	2+:145729036-145742052
21666	Ncor1	20185	11-:62131042-62233285
21667	MMT00076649		18+:47030475-47030960
21668	Tst	22117	15-:78230053-78236277
21669	Zic5	65100	14-:122858381-122864892
21670	Ggt1	14598	10+:75036337-75048927
21671	MMT00006397		8-:37435651-37436718
21672	Atp2b1	67972	10+:98431322-98488773
21673	Rbm18	67889	2-:35973125-35992104

21674	Olfr875	258744	9+:37580245-37581176
21675	Mosc2	67247	1-:186637572-186669709
21676	Camkv	235604	9+:107838255-107852021
21677	EG330129	330129	5-:93819627-93822069
21678	Prcc	94315	3-:87662836-87689502
21679	Pnpla6	50767	8+:3515383-3544266
21680	Gpr3	14748	4-:132765280-132767772
21681	9130011J15Rik	66818	8-:75089788-75094960
21682	Med17	234959	9-:15064795-15084311
21683	Prdm10	382066	9+:31087890-31186128
21684	Cd82	12521	2-:93259268-93302568
21685	Gzma	14938	13-:113884032-113891187
21686	MMT00026800		16+:47212481-47213039
21687	Zbtb7b	22724	3-:89183210-89185179
21688	MMT00002470		7-:26613565-26613914
21689	Ankrd53	75305	6+:83712677-83718319
21690	Slc3a1	20532	17+:85427731-85463581
21691	Olfr432	258711	1+:175980505-175981443
21692	MMT00013924		9+:57331735-57332397
21693	BC029169	208659	11-:109534234-109583569
21694	OTTMUSG00000(	433804	4+:146630900-146958343
21695	Olfr493	258307	7-:115489550-115490493
21696	Ctnna3	216033	10+:63871938-64138664
21697	2610208M17Rik	108909	1_random+:259469-297849
21698	1700022C21Rik	76416	17+:37095536-37102567
21699	MMT00013188		17+:16918752-16919523
21700	Lingo2	242384	4-:35655408-35657227
21701	Adam23	23792	1+:63492705-63639542
21702	Fut2	14344	7-:52905674-52906716
21703	Tmem38b	52076	4+:53838933-53873461
21704	MMT00074789		10+:21923206-21974036
21705	Actb	11461	5-:143664794-143668401
21706	Fundc1	72018	X-:17133694-17149423
21707	Tesc	57816	5+:118477926-118511876
21708	AI413582	106672	17-:27699873-27702844
21709	Tex22	75671	12+:114312713-114327129
21710	Slc30a6	210148	17+:74801250-74822853
21711	Pkhd1	241035	1-:20047858-20094758
21712	Ttc5	219022	14-:51385085-51405190
21713	Adnp	11538	2-:168007547-168010033
21714	Olfr655	258817	7-:111744767-111745693
21715	Siah1a	20437	8-:89247906-89269833
21716	Tmem85	68032	2-:112203175-112208184
21717	EG628893	628893	X-:74962581-74963366
21718	Creb3l4	78284	3-:90041421-90047434
21719	Olfr633	258351	7+:111095081-111096018
21720	Clec4d	17474	6+:123212192-123224934
21721	Styk1	243659	6-:131249161-131262616
21722	MMT00054655		11-:12836541-12837709
21723	Homer1	26556	13+:94075012-94126407
21724	Pfdn4	109054	2+:170336931-170344571

21725	Sost	74499	11-:101825159-101828287
21726	Rsu1	20163	2-:12998688-13192851
21727	Khk	16548	5+:31224288-31233419
21728	MMT00024565		13+:28457685-28609291
21729	Hdc	15186	2-:126419402-126444379
21730	Cntnap3	238680	13-:64842121-64989111
21731	Agxt	11611	1+:95031821-95041984
21732	Prrg3	208748	X+:69211840-69212923
21733	Rhebl1	69159	15-:98708209-98711693
21734	Doc2g	60425	19+:4003386-4006854
21735	C130073F10Rik	242574	4-:101562629-101566382
21736	A430060F13Rik	100039739	11-:94721829-94723105
21737	Srst	20819	17-:83209639-83211256
21738	Abhd14a	68644	9-:106342389-106349967
21739	Epb4.1	269587	4-:131479017-131628013
21740	D19Ertd652e	70806	19-:47827457-47838077
21741	EG621605	621605	3+:142321915-142325661
21742	MMT00066036	433806	4-:147436378-147442601
21743	Ccno	218630	13+:113778105-113780986
21744	MMT00011997		9+:114027204-114061561
21745	MMT00039382		
21746	Mgat5b	268510	11+:116780305-116846969
21747	H2afx	15270	9+:44142793-44144154
21748	Ucn2	171530	9+:108888218-108889073
21749	Krt4	16682	15-:101748966-101755142
21750	Rab31	106572	17-:66001069-66122021
21751	Ptpn6	15170	6-:124670734-124683122
21752	MMT00013098		11+:93494227-93513235
21753	Pcdhb14	93885	18+:37607496-37609885
21754	Synj2	20975	17+:5975640-6044289
21755	Fgd6	13998	10+:93565986-93604749
21756	MMT00017467		X-:104540011-104550744
21757	Kcng3	225030	17-:83987075-84030694
21758	Dnajc9	108671	14-:21203861-21208111
21759	1700011L22Rik	67687	8-:81737375-81772469
21760	Crb2	241324	2+:37638719-37652673
21761	D14Ertd449e	66039	14-:26743655-26746694
21762	Lage3	66192	X-:71597500-71598957
21763	Cspg4	121021	9+:56712966-56746941
21764	Tgm5	74176	2-:120871846-120911558
21765	Arpp19	59046	9+:74885723-74904611
21766	Elavl2	15569	4-:90918936-91038745
21767	Gm1758	385737	16+:14502192-14507354
21768	Necab2	117148	8+:121970618-121996533
21769	Loxl2	94352	14+:70009000-70095642
21770	MMT00042324		17+:57152018-57158974
21771	1700008F21Rik	75453	8+:131599364-131702286
21772	MMT00002494		6+:34989351-34989622
21773	Pcdhb2	93873	18+:37454629-37457027
21774	MMT00031273		4-:111463186-111481072
21775	MMT00051649		14-:76329373-76331352

21776	Ppp4r1l	109229	9-:35024247-35075366
21777	Crp	12944	1+:174628188-174630055
21778	Tmem201	230917	4-:149102265-149112056
21779	Ncstn	59287	1-:173996698-174012830
21780	Ankrd35	213121	3+:96474259-96494956
21781	1700001G17Rik	67503	1+:33726668-33727549
21782	Spire2	234857	8+:125856730-125893421
21783	MMT00047957		5+:123516253-123516800
21784	Gm853	332942	4-:129887608-129897729
21785	MMT00043723		3-:69139122-69139666
21786	1700016D06Rik	76413	8-:11654923-11678750
21787	MMT00008246		16+:32405920-32408101
21788	MMT00036513		5+:109665987-109667178
21789	Fdxr	14149	11-:115129338-115138283
21790	Smarcal1	54380	1+:72629844-72679704
21791	MMT00042985		9-:96714255-96717996
21792	Ptp4a2	19244	4+:129516647-129525104
21793	Pla2g12a	66350	3+:129581636-129597872
21794	Pfkp	56421	13-:6580420-6647976
21795	0610009B22Rik	66050	11-:51498886-51502136
21796	Dhrs4	28200	14+:56097711-56106408
21797	Dst	13518	1+:34068669-34365495
21798	MMT00070546		16-:45426908-45427916
21799	Cops5	26754	1-:10014913-10027979
21800	EG626058	626058	1+:155260671-155261163
21801	Serpinb6a	20719	13-:34009796-34027847
21802	Nptn	20320	9+:58430215-58500678
21803	Slc15a2	57738	16-:36750266-36785048
21804	D4Ertd22e	213491	4-:140671054-140695673
21805	Plk3	12795	4-:116800998-116806407
21806	MMT00064565		13-:51300538-51301473
21807	MMT00045004		17+:71278397-71278884
21808	BC065085	219148	14+:64071092-64081667
21809	Chrna10	504186	7-:109260554-109264831
21810	Pms1	227099	1-:53245487-53338936
21811	Ak3l1	11639	4+:101092343-101139177
21812	4930485B16Rik	380654	10-:92498164-92501994
21813	Eif1	20918	11+:100181428-100182673
21814	Accn5	58170	3+:81800843-81825155
21815	Itga5	16402	15-:103175540-103197005
21816	Trappc6a	67091	7+:20094078-20101491
21817	MMT00028065		11-:57877313-57886415
21818	2010003K11Rik	69861	19-:4496785-4498582
21819	1110008J03Rik	100764	5-:121059071-121062578
21820	Aldoart2	79459	12+:56666225-56667883
21821	Olfr1032	258572	2+:85847934-85848865
21822	Psme4	103554	11+:30671775-30780396
21823	Atp6v0d1	11972	8-:108048369-108089825
21824	5830443L24Rik	76074	5-:105443175-105447782
21825	BC030396	239463	15+:57817457-57841594
21826	MMT00027189		15+:96019030-96019653

21827	MMT00010458	403088	6-:42537500-42547275
21828	Cyp2c66	69888	19+:39188395-39261561
21829	MMT00018097		12-:58619233-58620633
21830	MMT00062008		5+:57652508-57653255
21831	MMT00067591		14+:53741724-53775966
21832	Lrp8	16975	4+:107474865-107546772
21833	Acbd5	74159	2+:22923733-22970479
21834	Best3	382427	10+:116439575-116446097
21835	mCG_13386	100113398	10+:80065624-80070397
21836	Sec23ip	207352	7+:135888101-135927510
21837	BC052328	223433	15-:27586020-27611238
21838	Ccdc116	76872	16-:17139928-17144518
21839	Gprasp1	67298	X+:132336824-132338003
21840	Tmie	20776	9-:110769364-110770592
21841	RP23-157O10.7	56013	11-:97372211-97372655
21842	Abca12	74591	1-:71289663-71368425
21843	MMT00051870	195576	4-:145059253-145070153
21844	Ncoa7	211329	10-:30367339-30491624
21845	Gm648	270599	X-:53797243-53798334
21846	Olfr214	258754	6+:116506444-116507407
21847	MMT00063855		X+:114000424-114000790
21848	Mpnd	68047	17+:56148748-56156084
21849	Icam2	15896	11-:106238970-106243955
21850	Gtf2i	14886	5-:134713713-134790563
21851	Ldb1	16825	19-:46107092-46114111
21852	Clcnka	12733	4-:140940800-140954111
21853	5430432N15Rik	74490	7+:52895344-52901137
21854	Fkbp3	30795	12-:66163422-66174927
21855	Mtap2	17756	1+:66368669-66485762
21856	B430201A12Rik	329739	3-:108795445-108806338
21857	1700061G19Rik	78625	17+:57014896-57028321
21858	Neil3	234258	8-:54672220-54724350
21859	Fancg	60534	4-:43015817-43022982
21860	6230416C02Rik	545490	2+:174866483-175305255
21861	Vprbp	321006	9+:106724204-106784415
21862	MMT00041029		10+:77250577-77277719
21863	Dok2	13449	14+:71174187-71178300
21864	Zdhhc20	75965	14-:58455237-58509099
21865	1100001E04Rik	75404	X+:46823736-46850728
21866	Hhipl1	214305	12+:109544411-109566422
21867	4933412E14Rik	244885	9+:54386770-54388886
21868	Cpne5	240058	17-:29295941-29374733
21869	Olfr729	258275	14-:50767576-50768547
21870	Dut	110074	2+:125073460-125084234
21871	Spock3	72902	8+:65430028-65835648
21872	Stambpl1	76630	19+:34307131-34314816
21873	Hsd11b2	15484	8+:108042753-108047417
21874	Nppb	18158	4+:147360084-147361087
21875	Zw10	26951	9+:48863736-48886877
21876	Cilp2	68709	8-:72411384-72415924
21877	Slitrk6	239250	14-:111148972-111151495

21878	MMT00048839		16-:11047097-11048025
21879	Tmem34	234463	8-:80120015-80134486
21880	MMT00045458		2-:49182896-49183178
21881	MMT00038325		5-:107167134-107167784
21882	Map2k5	23938	9-:63011576-63225659
21883	Trmt11	73681	10-:30254030-30320498
21884	2310035K24Rik	69596	2+:131036159-131039247
21885	Trim38	214158	13+:23874440-23883363
21886	Foxj2	60611	6+:122770201-122793115
21887	Ankmy1	241158	1-:94767386-94796265
21888	Kti12	100087	4+:108520461-108522017
21889	Mgat3	17309	15+:80041403-80043020
21890	Slc22a13	102570	9-:119102109-119118204
21891	Nnt	18115	13-:120124552-120197723
21892	Rabgef1	56715	5+:130647739-130690205
21893	Iqck	434232	7+:125999288-126116769
21894	Zfp263	74120	16+:3744103-3750787
21895	Crygn	214301	5-:24256871-24260148
21896	Lce1c	73719	3+:92483168-92484840
21897	Akap8l	54194	17-:32458369-32487515
21898	MMT00050545		15+:90530472-90554430
21899	Ccdc47	67163	11-:106061779-106077617
21900	Dpep2	319446	8-:108508957-108514810
21901	BC035537	212326	8-:46424429-46444058
21902	Gemin7	69731	7-:20150297-20158692
21903	Nr1d1	217166	11-:98629245-98636136
21904	Svopl	320590	6-:37962290-37964868
21905	Gpr114	382045	8+:97461575-97466248
21906	Jph4	319984	14-:55726464-55734283
21907	Dynlrb1	67068	2+:155062313-155076009
21908	Susd2	71733	10-:75099453-75106722
21909	Neurl2	415115	2-:164656232-164658966
21910	P2rx2	231602	5-:110768832-110771974
21911	Ribc2	67747	15+:84962507-84974997
21912	OTTMUSG00000(	629754	2-:164475133-164477308
21913	Fzd1	14362	5-:4755652-4757580
21914	C030037F17Rik	77475	8-:14745327-14747430
21915	MMT00006856		13+:33810144-33811373
21916	Cdk4	12567	10+:126501291-126504076
21917	Taf9b	407786	X-:103403659-103415105
21918	Snx24	69226	18+:53405422-53550574
21919	MMT00078220		5+:3090867-3092385
21920	Fnip2	329679	3-:79263609-79371602
21921	Tpm2	22004	4-:43527583-43536260
21922	MMT00028085		2+:5520620-5664750
21923	Olfr585	259091	7+:110246256-110247212
21924	Ang2	11731	14-:51815160-51815598
21925	Tial1	21843	7-:135585815-135604803
21926	MMT00059984		6+:145214919-145215296
21927	Hoxb4	15412	11+:96180010-96181995
21928	Nudcd1	67429	15-:44207616-44259853

21929	Vldlr	22359	19+:27291921-27324109
21930	1700081D17Rik	73523	14+:70363241-70459706
21931	Nrxn3	18191	12+:90741461-90771454
21932	Clec9a	232414	6+:129358880-129372991
21933	Mylk3	213435	8-:87882813-87888992
21934	Id2	15902	12-:25780183-25780875
21935	Prok2	50501	6-:99661293-99676386
21936	Gpr158	241263	2+:21737192-21749320
21937	MMT00015325		17+:41238748-41238994
21938	Rab3a	19339	8+:73279754-73281986
21939	1110038D17Rik	68778	10-:74969031-74980068
21940	Fxyd2	11936	9+:45207300-45218360
21941	MMT00028347		1+:125653267-125654326
21942	Fech	14151	18-:64616203-64648720
21943	Plekhn1	231002	4-:155598014-155602649
21944	MMT00008092		3+:9053800-9073101
21945	Ccnb3	209091	X-:6584137-6587228
21946	MMT00009539		11+:28855319-28857605
21947	Cdca8	52276	4-:124595709-124614153
21948	MMT00011696		
21949	MMT00065773		19+:57659239-57659454
21950	Chd6	71389	2-:160772721-160885717
21951	Cypt6	66742	X+:16099997-16100809
21952	Tfb1m	224481	17-:3519258-3557713
21953	Colec12	140792	18+:9707674-9877990
21954	BC030476	239368	15+:34400867-34402678
21955	Atxn7l1	380753	12+:34000322-34053138
21956	Olfr474	258488	7+:115098507-115099089
21957	Olfr507	258738	7+:115765327-115766278
21958	Slc27a1	26457	8+:74092806-74110438
21959	Sh3gl1	20405	17-:56156173-56175999
21960	Acaca	107476	11+:84045175-84155449
21961	Olfr1012	258561	2-:85599597-85600531
21962	Gdf11	14561	10-:128321588-128328445
21963	Edaradd	171211	13-:12569967-12612705
21964	Vwce	71768	19+:10708720-10716232
21965	AY036118	170798	17-:39985039-39985774
21966	Pcp4l1	66425	1-:173103394-173126371
21967	Tyk2	54721	9-:20908518-20935654
21968	Zfp426	235028	9-:20273992-20297186
21969	Tk1	21877	11-:117676840-117687267
21970	Eif3f	66085	7+:116077929-116085201
21971	Epb4.2	13828	2-:120843953-120862491
21972	Pde6b	18587	5+:108817458-108860754
21973	Faf1	14084	4+:109349300-109634651
21974	Ifltd1	74071	6-:145345754-145383445
21975	MMT00008417		2+:130935301-130935523
21976	Siae	22619	9+:37421425-37455120
21977	MMT00013499		1-:175204593-175207882
21978	Txnl1	53382	18-:63823755-63851842
21979	Fxyd1	56188	7-:31836726-31840601

21980	Gdf6	242316	4+:9771537-9787431
21981	Ormdl2	66844	10-:128255550-128258662
21982	MMT00009230		7-:105132959-105133906
21983	C130022K22Rik	232236	6+:91828061-91849832
21984	Tshz1	110796	18-:84182418-84185648
21985	Lrp2bp	67620	8+:47084656-47112874
21986	Cstf1	67337	2+:172196502-172206585
21987	Caps2	353025	10+:111612254-111652737
21988	MMT00015654	675749	7-:3813573-3820818
21989	Zfp167	382118	9+:122795968-122805240
21990	MMT00030729		6-:70378917-70381216
21991	Olfr935	258741	9-:38802093-38803018
21992	Lap3	66988	5+:45884651-45903688
21993	D430015B01Rik	58909	6-:58885369-58953507
21994	Dbndd2	52840	2+:164312076-164316774
21995	Nktr	18087	9+:121628293-121665959
21996	Mme	17380	3+:63104056-63184253
21997	Phf1	21652	17+:27070276-27074834
21998	Nomo1	211548	7+:53289087-53339752
21999	ENSMUSG000000	100049171	14+:53666865-53667623
22000	Pim2	18715	X+:7455431-7460558
22001	Gtlf3b	24083	11-:60716562-60727291
22002	Dcst2	329702	3+:89177144-89177503
22003	Zfp326	54367	5+:106305620-106344831
22004	Gtpbp2	56055	17+:46298084-46306316
22005	MMT00009438		10-:129906384-129907689
22006	Rc3h1	381305	1+:162836496-162898226
22007	Nek9	217718	12-:86640462-86680281
22008	Rbm35a	207920	4-:11259082-11313583
22009	Tmem14a	75712	1+:21208742-21220245
22010	Ccdc41	77048	10+:94151398-94252790
22011	Aldh9a1	56752	1+:169280375-169297629
22012	Itgae	16407	11+:72904084-72960942
22013	Usp24	329908	4+:106019139-106031079
22014	Wbp1	22377	6-:83069038-83071417
22015	2410127E18Rik	76788	6+:30367033-30402332
22016	1110019J04Rik	68512	4-:45118083-45120985
22017	MMT00041197		6+:83791675-83791944
22018	Prima1	170952	12-:104435117-104480356
22019	Chrng	11449	1+:89102388-89108208
22020	Gramd2	546134	9+:59555448-59558424
22021	Csnk1d	104318	11-:120824737-120852633
22022	Otud7b	229603	3+:95908449-95959890
22023	Cd3d	12500	9+:44789968-44794425
22024	Lrrc58	320184	16+:37878372-37884355
22025	Ccl22	20299	8+:97269489-97275599
22026	Fgfbp1	14181	5-:44370433-44371187
22027	A4galt	239559	15-:83057155-83082149
22028	MMT00050637		10-:18022473-18022878
22029	Igsf3	78908	3+:101229197-101267402
22030	Mtmr6	219135	14+:60884107-60920502

22031	Vcam1	22329	3-:115813057-115832342
22032	Kirrel3	67703	9+:34293797-34318199
22033	MMT00018345		13-:74690636-74691860
22034	Appl1	72993	14-:27736760-27783697
22035	Olfr142	406186	2-:90092226-90093143
22036	MMT00055735		13+:99804221-99804658
22037	Oas1g	23960	5-:121326732-121337591
22038	Tnip1	57783	11-:54724290-54776414
22039	Npc2	67963	12-:86097356-86113848
22040	Rasal1	19415	5+:121099366-121129605
22041	Kirrel2	243911	7-:31234855-31241553
22042	Mgam	232714	6+:40594829-40719115
22043	Rdh8	235033	9+:20622947-20629987
22044	MMT00024365		14-:38725099-38725899
22045	Nbr1	17966	11+:101414171-101443258
22046	Cml4	68396	6-:85780375-85781829
22047	12-Sep	71089	16-:4988177-4997872
22048	MMT00018179		16-:11189233-11191343
22049	Wdr40b	245404	X-:42141592-42143006
22050	Vamp5	53620	6-:72318128-72318637
22051	Cno	117197	5-:37138879-37139897
22052	Necab3	56846	2-:154370140-154381390
22053	Pstpip2	19201	18+:78033456-78118143
22054	Fut1	14343	7+:52873993-52875126
22055	Il9	16198	13-:56580637-56583607
22056	Nov	18133	15+:54577526-54584058
22057	Lrrtm3	216028	10-:63550590-63561032
22058	Slc11a2	18174	15+:100222523-100225166
22059	Myo3a	667663	2+:22149129-22435377
22060	Ccbl1	70266	2-:30040642-30061619
22061	MMT00054756		8+:3105753-3105802
22062	B230110C06Rik	77981	14+:16270133-16284959
22063	BC051227	234384	8-:73282294-73284820
22064	MMT00075928		3-:35435737-35436751
22065	Hes2	15206	4+:151533777-151534658
22066	Cabp1	29867	5-:115618954-115636130
22067	Ap1s2	108012	X+:160346947-160371599
22068	Trabd	67976	15+:88906500-88917500
22069	Fkbpl	56299	17+:34782204-34783247
22070	MMT00067910		15-:103275811-103277130
22071	Hoxc4	15423	15+:102864825-102867027
22072	MMT00078067		7+:115162595-115163186
22073	Lrig3	320398	10+:125403204-125452414
22074	EG212753	212753	X-:98985390-98989445
22075	C1s	50908	6-:124480644-124483991
22076	Pgm2l1	70974	7+:107376115-107427381
22077	MMT00057550	677168	17-:46543911-46544526
22078	Zfp661	72180	2-:127401270-127403681
22079	Tmem81	74626	1+:134402806-134405214
22080	MMT00030613		X+:72153071-72163420
22081	Nme2	18103	11-:93811129-93817154

22082	Cyp4a30b	435802	4+:115126939-115143665
22083	4933413J09Rik	71106	14+:27182114-27197404
22084	MMT00070787		9+:122656463-122659550
22085	MMT00036089		6-:122741561-122743432
22086	Atp11a	50770	8+:12757223-12865173
22087	Slc30a7	66500	3-:115649581-115710132
22088	Samd4b	233033	7-:29184568-29221211
22089	Niban	63913	1+:153418669-153566166
22090	Plscr1	22038	9+:92157864-92166813
22091	Nt5c3l	68106	11-:100284062-100303122
22092	Plekhg4	102075	8+:107898800-107905956
22093	Zbbx	213234	3-:74841831-74916146
22094	Rnf149	67702	1-:39608137-39634253
22095	Mgst1	56615	6+:138096210-138104897
22096	Slc4a9	240215	18+:36687802-36700947
22097	Olfr61	18362	7+:147823601-147824537
22098	MMT00009248		7-:104740367-104740914
22099	Cnot8	69125	11-:57910274-57911351
22100	Lcn5	13863	2+:25513587-25517164
22101	Wdr52	212517	16+:44421234-44430170
22102	MMT00059646		16-:9105550-9106543
22103	Hvcn1	74096	5+:122660295-122692282
22104	Rnf123	84585	9-:107964083-107964976
22105	MMT00056818		13-:116318706-116319989
22106	Mmp15	17388	8+:97876763-97897770
22107	MMT00064159		3+:58455445-58456762
22108	Cdk5	12568	5-:23925046-23929357
22109	Jarid1b	75605	1+:136456749-136528564
22110	Olfr1232	258320	2-:89165401-89166335
22111	Ttn	22138	2-:76677461-76679353
22112	Xlr4a	434794	X-:70319678-70326134
22113	MMT00030381		8-:4529593-4538223
22114	Olfr477	258928	7+:115134120-115134813
22115	Ccl5	20304	11-:83339282-83343991
22116	Fbxo42	213499	4+:140723457-140756725
22117	Slc4a11	269356	2-:130509849-130523193
22118	Grhl3	230824	4-:135098574-135117270
22119	MMT00067609	547323	14+:53511585-53512490
22120	Ccna1	12427	3-:54849396-54858977
22121	MMT00015026		12-:89546482-89546909
22122	MMT00042954		
22123	MMT00046185		2+:44643947-44646204
22124	4921509C19Rik	381393	2-:151296280-151320279
22125	Gab1	14388	8-:83289721-83403525
22126	RP23-303F24.3	622459	11-:53596920-53672732
22127	Pafah1b3	18476	7-:26080067-26082974
22128	1810054D07Rik	69863	4-:82872323-82970146
22129	MMT00066623	100044382	X+:52766936-53097329
22130	Cks1b	54124	3-:89219394-89222213
22131	Slc35c2	228875	2-:165102055-165113327
22132	Ids	15931	X-:67599338-67618056

22133	V1rd17	404286	7+:24585865-24714375
22134	Ikzf4	22781	10-:128082775-128088395
22135	MMT00033583		14+:17779358-17779949
22136	Olfr701	66786	7+:113957657-113964223
22137	Nudt7	67528	8+:116657514-116676204
22138	Ang	11727	14+:51721078-51721516
22139	Stip1	20867	19-:7095197-7114433
22140	Crygf	12969	1+:65973095-65974878
22141	Rorb	225998	19-:19012131-19075607
22142	5830457O10Rik	214987	8-:109407764-109410858
22143	1700006A11Rik	71824	3-:124103908-124124391
22144	Arr3	170735	X+:97800894-97813831
22145	MMT00006164		11-:21582393-21584105
22146	Aifm2	71361	10+:61195069-61202035
22147	Ap1s1	11769	5-:137510863-137521930
22148	MMT00035976		
22149	MMT00059396		3-:57521848-57522211
22150	Fhdc1	229474	3-:84247398-84278704
22151	Plxnd1	67784	6-:115904826-115944675
22152	5830411G16Rik	78937	6+:56664918-56709117
22153	Pold1	18971	7-:51788159-51798806
22154	Agpat1	55979	17+:34742826-34749449
22155	EG435392	435392	14-:23377353-23377678
22156	MMT00059480		13+:15035566-15037025
22157	Mxra8	74761	4+:155213905-155218202
22158	Lrrc31	320352	3-:30577989-30588939
22159	Apba3	57267	10+:80730983-80735972
22160	Pmpcb	73078	5+:21242956-21262973
22161	Tmem132a	98170	19-:10932322-10944221
22162	MMT00008624		3+:7759565-7932337
22163	MMT00001291		3-:8615431-8616422
22164	Msrb3	320183	10-:120220946-120308216
22165	St8sia4	20452	1-:97484260-97564171
22166	Traf1	22029	2-:34798775-34817105
22167	MMT00036488		6+:140003599-140004324
22168	Uxs1	67883	1-:43806022-43884615
22169	Ctrl	109660	8-:108455902-108457762
22170	Chrnb4	108015	9-:54877911-54896302
22171	MMT00012636	100042265	9-:121758658-121766649
22172	Sgpp1	81535	12-:76816691-76836666
22173	Kcna3	16491	3+:106839340-106840925
22174	MMT00041338		6-:112343341-112343553
22175	Odf3	69287	7+:148033729-148036906
22176	Klra18	93970	6-:130013122-130017276
22177	Lepr	16847	4+:101390043-101464862
22178	Snx26	233071	7-:31308650-31318051
22179	Itga6	16403	2+:71625014-71694240
22180	Nlrp9a	233001	7+:27342304-27359163
22181	Zfp75	244713	9+:19448051-19454156
22182	Oprm1	18390	10-:3309339-3557732
22183	Tut1	70044	19+:9028328-9040697

22184	MMT00033856		11+:83614245-83615066
22185	4930422G04Rik	71643	3+:127286935-127320939
22186	Lpo	76113	11-:87619929-87639612
22187	MMT00042192		6+:127308950-127361702
22188	Top2a	21973	11-:98855077-98885429
22189	Tmem168	101118	6-:13531097-13558044
22190	MMT00042070		
22191	Dhrs9	241452	2+:69230966-69235830
22192	9030205A07Rik	217843	12+:104301314-104422208
22193	MMT00007348		4+:14097146-14098124
22194	Olfr39	258822	9+:20090144-20091079
22195	Mc1r	17199	8+:125931395-125932653
22196	Cyp3a41a	53973	5-:146529490-146531712
22197	Ankrd28	105522	14-:32511959-32643590
22198	Metap1	75624	3-:138121928-138152369
22199	Acsm5	272428	7+:126669776-126687306
22200	Adam32	353188	8-:25983043-26059280
22201	Pard3	93742	8+:129588144-130134729
22202	OTTMUSG000000	545539	3+:89196186-89202147
22203	Txndc11	106200	16-:11075006-11088024
22204	Slc6a20a	102680	9-:123545239-123587963
22205	Dnahc1	110084	14-:32121891-32137080
22206	V1rd2	81016	7+:5626205-5628197
22207	Edem3	66967	1+:153602437-153669458
22208	Me3	109264	7+:96781327-97000472
22209	Cdc42ep5	58804	7-:4102865-4116104
22210	1700123O20Rik	58248	14+:55305052-55309578
22211	Vgll1	170828	X+:54341282-54358623
22212	Pex12	103737	11-:83108577-83112017
22213	Adora1	11539	1-:136097952-136131999
22214	Msx3	17703	7-:147232155-147234985
22215	A630047E20Rik	271981	3+:132349787-132501573
22216	Sp5	64406	2+:70313222-70315753
22217	Flvcr2	217721	12+:87087488-87154535
22218	Rfc3	69263	5-:152445392-152453770
22219	Cyp11b1	110115	15-:74665322-74672036
22220	Ccdc22	54638	X-:7170935-7182463
22221	Dpysl5	65254	5+:31013936-31101341
22222	Gstt1	14871	10-:75246784-75261241
22223	Spg7	234847	8+:125589426-125621653
22224	Svs1	243377	6+:48936900-48940688
22225	Slc30a4	22785	2-:122506980-122528200
22226	Pm20d1	212933	1+:133701200-133714693
22227	Rab11a	53869	9-:64564416-64585551
22228	MMT00013109		
22229	Dnmt1	13433	9-:20711964-20757423
22230	Myh9	17886	15-:77591018-77672529
22231	Tnfsf4	22164	1+:163325588-163348337
22232	Krt19	16669	11-:100002128-100007228
22233	Asrgl1	66514	19-:9186020-9210100
22234	Tmem160	69094	7+:17038130-17040846

22235	Usp45	77593	4+:21694301-21761651
22236	Panx1	55991	9-:14810230-14849520
22237	Macrod2	72899	2+:140221189-140278828
22238	Sardh	192166	2-:27044902-27102332
22239	Ap3b1	11774	13+:95128985-95336270
22240	Spen	56381	4-:141024772-141078246
22241	Sfrs10	20462	16-:22246535-22265983
22242	Gopc	94221	10-:52059180-52101846
22243	Tmem16a	101772	7-:151774452-151864752
22244	Slc16a7	20503	10-:124664541-124765591
22245	Tmem161b	72745	13+:84361820-84435743
22246	Kcmf1	74287	6-:72791107-72811920
22247	Pigyl	66268	9+:21961309-21962792
22248	OTTMUSG00000(	329919	4+:112296613-112298701
22249	Wdr3	269470	3-:99942103-99952132
22250	Wdr45l	66840	11-:121188547-121215723
22251	Ankrd6	140577	4-:32892891-32947564
22252	Plekha2	83436	8-:26152997-26212226
22253	Tpsb2	17229	17+:25503494-25506039
22254	4933402N03Rik	233918	7-:138281225-138289809
22255	2900010M23Rik	67267	17-:27259609-27270836
22256	Arg2	11847	12+:80231810-80257282
22257	Lonrf3	74365	X+:33868401-33898750
22258	Ttk	22137	9+:83728347-83765991
22259	Sfrs12ip1	67288	13+:105607302-105628440
22260	Slc38a4	69354	15-:96827200-96861642
22261	4933428G20Rik	58996	11+:97351386-97363714
22262	Mthfd1l	270685	10-:6240002-6373416
22263	Gzmd	14941	14-:56748417-56751359
22264	Lpxn	107321	19+:12873082-12908330
22265	Sez6l	56747	5-:113005791-113019018
22266	Pyhin1	236312	1+:175560995-175578005
22267	Unc13c	208898	9-:73778403-73816781
22268	MMT00013001		5-:87591295-87645522
22269	Mgmt	17314	7+:144142956-144320072
22270	Olfr74	117005	2-:87813865-87814820
22271	Cyp2c29	13095	19+:39361600-39405189
22272	Vpreb1	22362	16-:16868495-16869343
22273	Zfp146	26465	7-:30946619-30954706
22274	Slc22a16	70840	10+:40290274-40323946
22275	Sst	20604	16-:23889666-23890930
22276	Fbxl21	213311	13+:56638184-56638826
22277	A030005L19Rik	77922	1+:82909899-82910705
22278	Ap4s1	11782	12+:52792023-52839918
22279	Krr1	52705	10+:111409810-111425943
22280	Clca3	23844	3-:144667501-144695723
22281	Lcor	212391	19+:41558007-41602650
22282	Rhob	11852	12-:8505848-8506438
22283	Erlin2	244373	8+:28134300-28149896
22284	Tcerg1l	70571	7-:145401374-145589241
22285	MMT00043109		X-:10274096-10274516

22286	Mmachc	67096	4-:116375039-116380926
22287	Mylc2b	67938	17-:71323302-71339856
22288	MMT00061717		
22289	Smn1	20595	13+:100894848-100907645
22290	Tubb2a	22151	13-:34166150-34169875
22291	V1re7	171230	17-:21026681-21027615
22292	Olfr45	18344	7+:147876805-147877739
22293	Gprc6a	210198	10-:51334671-51348358
22294	Ttc35	66736	15+:43308820-43359308
22295	Iqsec1	232227	6-:90609057-90666266
22296	1700030G06Rik	69507	5-:22199664-22200709
22297	Zfyve1	217695	12-:84887504-84916086
22298	Zfp259	22687	9+:46081248-46089685
22299	Heatr2	433956	5+:139646148-139662460
22300	Ddx41	72935	13-:55631778-55637873
22301	Cbr4	234309	8+:63966580-63982279
22302	MMT00041810		8-:38434879-38436192
22303	Cdt1	67177	8+:125091941-125097033
22304	Pigt	78928	2+:164323064-164333266
22305	Mcph1	244329	8+:18595272-18625674
22306	Myl4	17896	11+:104438765-104447067
22307	Sgtb	218544	13+:104899869-104931519
22308	2310015A10Rik	69548	12-:81224570-81233832
22309	Ptch1	19206	13-:63612841-63666828
22310	Asb5	76294	8+:55635683-55673189
22311	1700037H04Rik	67326	2-:130972060-130985756
22312	Itln1	16429	1-:173448372-173461884
22313	Prokr2	246313	2-:132197950-132207356
22314	E2f2	242705	4+:135728307-135751074
22315	Myo3b	329421	2+:69934353-70072535
22316	Kcnab2	16498	4-:151767143-151809990
22317	AI450540	226252	19+:57455727-57461427
22318	Cox8b	12869	7-:148084841-148086296
22319	Maff	17133	15+:79187427-79188285
22320	MMT00047284		
22321	Apol7a	75761	15-:77218645-77226747
22322	Rnf151	67504	17-:24852790-24854963
22323	MMT00051740		7-:17304466-17305259
22324	MMT00060741		7-:109796211-109796566
22325	Sts	20905	
22326	MMT00014155		14-:51511064-51512974
22327	Tyrobp	22177	7+:31198857-31202427
22328	MMT00040306		6+:68491651-68492524
22329	Galntl5	67909	5+:24687297-24726107
22330	Prox1	19130	1-:191946860-191986192
22331	Cck	12424	9-:121398944-121404780
22332	MMT00075553		15-:69892979-69894929
22333	Hnrnph3	432467	10-:62477414-62482275
22334	9430023L20Rik	68118	15+:101114731-101121365
22335	Ceacam2	26367	7-:26302889-26324876
22336	Hsd3b5	15496	3-:98422558-98434175

22337	Nlk	18099	11-:78381693-78510055
22338	Unc119	22248	11+:78157006-78162544
22339	Slc12a1	20495	2+:124979465-125021457
22340	Lnx2	140887	5+:147828219-147830167
22341	Hdhd2	76987	18+:77182868-77210141
22342	MMT00008396		5-:10241751-10245340
22343	Blm	12144	7-:87599977-87679983
22344	Hspd1	15510	1-:55140586-55143783
22345	Scamp2	24044	9+:57408760-57435602
22346	6330405D24Rik	70715	2-:119698480-119704139
22347	Gpr114	382045	8+:97457774-97459066
22348	MMT00061474		4+:143886695-143887769
22349	Cab39	12283	1+:87690359-87746617
22350	Indol1	209176	8-:25642925-25686805
22351	Fanca	14087	8-:125792201-125842476
22352	MMT00051519		18+:74400956-74401179
22353	A930041I02Rik	320271	2-:38930304-39046241
22354	2010301N04Rik	72103	6-:87657235-87672165
22355	Clec4e	56619	6-:123231806-123239889
22356	Gli3	14634	13+:15555812-15818806
22357	Chmp1b	67064	18+:67365076-67366653
22358	MMT00038532		
22359	Dhrs7b	216820	11+:60644188-60671647
22360	MMT00075564		15-:38173282-38173967
22361	Sephs2	20768	7-:134415396-134417560
22362	Laptm4b	114128	15+:34168078-34213134
22363	C530044N13Rik	223978	16-:11803808-11909496
22364	Cnbp2	75064	X+:100818852-100826524
22365	Jun	16476	4-:94715745-94718878
22366	MMT00005283		6+:16838966-16876948
22367	Gas8	104346	8+:126042733-126060549
22368	RP23-149D11.5	332923	4+:101613011-101615934
22369	Spinlw1	75526	2-:164413842-164419071
22370	1700058G18Rik	67342	10+:115855176-115858291
22371	1810063B05Rik	67892	8-:128948313-128999049
22372	MMT00030553	627908	X+:72478193-72481092
22373	Txnip	56338	3+:96361880-96365780
22374	BC087945	432940	15-:27535678-27560256
22375	MMT00053882		14+:95818909-95820406
22376	MMT00044511		4-:18771350-18773019
22377	Gm941	381112	17+:80770528-80787141
22378	Wdr60	217935	12-:117445928-117471145
22379	Gpx6	75512	13+:21404118-21411488
22380	MMT00014053	676914	3+:59757737-59776406
22381	Wdr37	207615	13-:8802244-8870266
22382	Tprg	71338	16+:25286903-25422427
22383	2200002K05Rik	69137	9+:15043486-15063837
22384	2410015M20Rik	224904	17-:56746380-56749250
22385	Olfr1437	258117	19-:12284792-12397072
22386	BC043118	224273	16-:59543001-59554049
22387	Tmc7	209760	7-:125680541-125693579

22388	2410018M08Rik	71970	5+:130371591-130379486
22389	AA409316	105732	15-:75831359-75843304
22390	Dhcr7	13360	7+:151016137-151034314
22391	Tmem16g	404545	1+:95281387-95282260
22392	Dhdds	67422	4-:133525089-133556786
22393	2310044H10Rik	69683	7-:51745332-51751882
22394	Sertad3	170742	7+:28258854-28261779
22395	Kcna1	16485	6-:126591689-126594831
22396	Fcrl5	329693	3+:87245926-87252359
22397	Lbxcor1	207667	9-:62992188-62994385
22398	Nav2	78286	7+:56708439-56862513
22399	Nell1	338352	7+:57230758-57431688
22400	2610036D13Rik	75425	2-:157807538-157854128
22401	Sgcz	244431	8-:38586316-39016111
22402	MMT00071327		8-:11683460-11684248
22403	Bank1	242248	3-:135717373-135763554
22404	Slc22a21	56517	11-:53764325-53793529
22405	6030422M02Rik	240697	1+:9901799-9905750
22406	Vnn1	22361	10+:23614665-23625145
22407	Rhbdf2	217344	11-:116459485-116485567
22408	Calr3	73316	8-:74948450-74967677
22409	Larp2	214048	3+:40766089-40781704
22410	MMT00010164		4+:133559277-133560800
22411	Reck	53614	4+:43888471-43957392
22412	Ptpn5	19259	7-:54333171-54389054
22413	MMT00061444		13-:61538488-61540814
22414	Myom2	17930	8+:15057652-15133540
22415	2310002L13Rik	75577	18-:70400083-70404239
22416	Wdr20a	69641	12+:111976153-112042448
22417	Mnx1	15285	5-:29799803-29805010
22418	Snph	241727	2-:151419015-151458277
22419	MMT00078129		14+:53627287-53627815
22420	Fgf23	64654	6+:127023047-127030496
22421	Cnot10	78893	9-:114494805-114549316
22422	Gpr172b	52710	15+:76369393-76371818
22423	MMT00037852		11-:3525481-3525851
22424	Gm693	277743	4+:140924197-140939561
22425	4632415K11Rik	74347	8-:122284011-122297339
22426	Zfp282	101095	6+:47827662-47856967
22427	MMT00049287		X-:62003662-62004338
22428	Csmd3	239420	15-:47678652-47836513
22429	Cblb	208650	16+:52033135-52204805
22430	Taar9	503558	10-:23821182-23829340
22431	1700019A02Rik	69397	1-:53215420-53244461
22432	4922505G16Rik	629499	2-:157083762-157105285
22433	Nsun3	106338	16-:62734954-62786600
22434	Olfr1255	258979	2+:89656484-89657415
22435	Tmem98	103743	11+:80623916-80635533
22436	Ksr2	333050	5+:118139568-118213328
22437	Ankrd29	225187	18-:12412886-12454498
22438	6330416G13Rik	230279	4+:63223489-63245850

22439	4921533L14Rik	68187	1-:24018521-24036655
22440	MMT00042948		8+:82229577-82230204
22441	Nlrp4f	97895	13-:65278422-65296696
22442	E130319B15Rik	209645	2+:4621283-4621716
22443	Tuba1c	22146	15+:98860808-98868535
22444	Vgll2	215031	10+:51742488-51745169
22445	Prkag3	241113	1-:74785498-74795530
22446	Ccr5	12774	9+:124039100-124040164
22447	Pcdhb11	93882	18+:37581272-37583664
22448	MMT00023461		3+:19834373-19834864
22449	Phf20	228829	2+:156022376-156134752
22450	Prpf19	28000	19+:10969753-10979806
22451	Olfr1461	258299	19+:13232689-13308799
22452	Tcfap2b	21419	1+:19199078-19224593
22453	Spag17	74362	3+:99892204-99896975
22454	Synj2bp	24071	12-:82601700-82633855
22455	Gprc5d	93746	6-:135056009-135068301
22456	Slc7a15	328059	12-:8536151-8546373
22457	6530401D17Rik	76219	X+:132567905-132569483
22458	Spata17	74717	1-:188872280-189039346
22459	Hnrnpa3	229279	2+:75497320-75500813
22460	4930430F21Rik	73784	15+:30968876-30973087
22461	Crkrs	69131	11+:98124798-98139806
22462	MMT00026054	100041835	11-:95934335-95936342
22463	Gorasp2	70231	2+:70499206-70529799
22464	AU042671	269700	5+:121765763-121768774
22465	Olfr790	258935	10+:128937941-128938909
22466	Pdyn	18610	2-:129513736-129515604
22467	Crhbp	12919	13-:96201332-96214728
22468	V1ri4	171255	13+:22566486-22567387
22469	Rtkn2	170799	10+:67489265-67498687
22470	Ppargc1b	170826	18-:61457790-61560085
22471	Ankrd42	73845	7-:99738615-99780172
22472	Lin28b	380669	10-:45096435-45100883
22473	Jsrp1	71912	10-:80271245-80276240
22474	Ubqln1	56085	13-:58279144-58316799
22475	Sh3bp1	20401	15+:78732786-78742473
22476	Tspan4	64540	7+:148661375-148679315
22477	A130034M23Rik	320759	16-:30312504-30388501
22478	Tanc1	66860	2+:59537404-59684269
22479	Ascc2	75452	11+:4537795-4583386
22480	MMT00056658		3-:32778162-32779534
22481	Fmnl3	22379	15-:99147651-99168153
22482	Vmn2r93	627132	17+:18440927-18462865
22483	Utp20	70683	10-:88209349-88289527
22484	MMT00043129		4-:106690539-106695105
22485	Unc119	22248	11-:78156957-78162597
22486	Sh3bgrl3	73723	4-:133683321-133684668
22487	Trub1	72133	19+:57527401-57563395
22488	Gzmc	14940	14-:56850277-56853493
22489	BC049730	232972	7+:25494288-25497626

22490	Lta4h	16993	10+:92916174-92947578
22491	Kif3b	16569	2+:153141950-153157314
22492	Ubb	22187	11+:62365007-62366714
22493	Slc35a2	22232	X+:7461369-7471531
22494	MMT00005811		4-:52948302-52949253
22495	Nek5	330721	8-:23184088-23193936
22496	Slfn1	20555	11+:82930346-82936161
22497	4921531P14Rik	70960	18-:83462161-83475854
22498	Krt5	110308	15-:101537502-101543322
22499	9030409G11Rik	71529	4-:141696730-141766123
22500	Dub1	13531	7+:112005528-112011179
22501	Rgs5	19737	1+:171624624-171625927
22502	Rilp	280408	11+:75323594-75326666
22503	Cnot6l	231464	5-:96505816-96591009
22504	Rab1	19324	11+:20101502-20125529
22505	Ogfod2	66627	5+:124562346-124565485
22506	Slc22a4	30805	11-:53796811-53841590
22507	Nap1l3	54561	X-:119508169-119510994
22508	Setd8	67956	5+:124910928-124912282
22509	MMT00038045		17-:74837565-74838004
22510	Hnrph1	59013	11+:50191270-50200027
22511	Shroom1	71774	11+:53270707-53281306
22512	MMT00005785		18+:8415120-8416047
22513	Ufd1l	22230	16+:18812404-18834853
22514	MMT00076032	667804	2+:74590836-74591356
22515	Ctf1	13019	7+:134856249-134861699
22516	Zscan2	22691	7+:88008053-88021401
22517	Ttc36	192653	9-:44607482-44611034
22518	Narfl	67563	17+:25910751-25919793
22519	1700054K19Rik	67336	6+:112160699-112162507
22520	Sec13	110379	6-:113678063-113690678
22521	Hsp90b1	22027	10-:86153585-86168189
22522	2010300C02Rik	72097	1-:37694830-37733634
22523	Ube2i	22196	17-:25398863-25406426
22524	Heatr2	433956	5+:139626587-139642157
22525	MMT00028383		7-:48922341-48923570
22526	8430410K20Rik	78100	9+:4383536-4386863
22527	EG668174	668174	3-:90718795-90720858
22528	Frmd5	228564	2-:121373822-121412207
22529	Fto	26383	8+:93837437-94192328
22530	MMT00050654		10-:14306897-14307345
22531	Gm484	243967	7+:52941550-52949922
22532	MMT00026824		13+:66630311-66677023
22533	1200003I07Rik	66869	8-:72229038-72240831
22534	Pik3r2	18709	8-:73292077-73300580
22535	4933401J01Rik	71042	1+:3063332-3064289
22536	Zfat	380993	15-:67915883-68056505
22537	MMT00049274		16+:15219036-15221648
22538	Atp6v1g3	338375	1+:140170315-140184834
22539	MMT00044599		4+:52049043-52254398
22540	Blzf1	66352	1-:166221519-166237349

22541	4632413I24Rik	74031	10+:7132928-7133737
22542	MMT00040348	434034	6-:68757165-68757702
22543	Psma4	26441	9+:54798680-54805629
22544	Cog7	233824	7-:129066681-129155747
22545	Uvrag	78610	7-:106036403-106289635
22546	Tnnt1	21955	7-:4456264-4467577
22547	Rhpn1	14787	15+:75534823-75544654
22548	Itgb8	320910	12-:120401589-120476658
22549	Ptdss2	27388	7+:148317300-148341418
22550	MMT00050817	100039518	8+:83457527-83458426
22551	Anapc11	66156	11+:120459787-120466988
22552	MMT00007799		3-:95218849-95219391
22553	Ms4a8a	64381	19-:11141961-11155518
22554	Tectb	21684	19+:55255374-55270802
22555	Ela2	50701	10+:79349079-79350843
22556	Slc26a8	224661	17-:28786612-28826932
22557	P4hb	18453	11-:120421355-120434500
22558	Cd209a	170786	8-:3744084-3748906
22559	MMT00031309		2+:34686022-34688171
22560	Gpr56	14766	8+:97501008-97538095
22561	Whdc1	434204	7+:88716165-88741478
22562	Sdha	66945	13-:74459703-74487690
22563	MMT00004885		4+:105466782-105467514
22564	Ccdc33	382077	9-:57925346-57950105
22565	Zgpat	229007	2+:181100140-181115489
22566	5330417C22Rik	229722	3-:108260986-108339665
22567	Pcdh15	11994	10+:73284630-74095409
22568	MMT00033197		12-:105427732-105430646
22569	Xpot	73192	10-:121024437-121063374
22570	Phtf1	18685	3+:103772180-103811411
22571	Hexim2	71059	11+:102991483-103000378
22572	5430411K18Rik	71423	18+:78144888-78231764
22573	Kif2c	73804	4-:116832742-116855189
22574	Baz2b	407823	2-:59737428-59963868
22575	St8sia6	241230	2-:13576566-13715147
22576	Tlr12	384059	4-:128292690-128295264
22577	Olfr574	258357	7+:110096980-110098020
22578	Bmp5	12160	9+:75623899-75746389
22579	Eif4a2	13682	16+:23107828-23114019
22580	MMT00081414		2+:15766872-15791436
22581	2310035C23Rik	227446	1+:107560445-107651761
22582	Gpr132	56696	12-:114090181-114099163
22583	Vmn2r89	22301	14+:52030599-52080294
22584	Gspt2	14853	X+:91881657-91883890
22585	MMT00004539		6-:37772702-37816091
22586	Olfr1394	258273	11+:48973517-48974456
22587	Ocm	18261	5-:144780801-144786575
22588	Hspb2	69253	9-:50559384-50560375
22589	Neto1	246317	18+:86565010-86670665
22590	Klk1b27	16619	7+:51307659-51312079
22591	Dok4	114255	8-:97387769-97400174

22592	MMT00055028		12+:100742958-100747794
22593	Spink3	20730	18-:43887737-43896848
22594	Ppig	228005	2+:69570299-69588277
22595	Mtmr15	330554	7-:71491644-71518242
22596	MMT00009596		2-:177099652-177100616
22597	Sytl1	269589	4-:132809081-132818981
22598	4930402H24Rik	228602	2-:130629128-130629599
22599	MMT00000084		3+:71663190-71663765
22600	2410018C20Rik	67873	8-:86775381-86781191
22601	Lig4	319583	8-:9971042-9973778
22602	V1rg8	171243	7-:12906556-12907477
22603	Il1f8	69677	2+:24008677-24016038
22604	Zc3h6	78751	2+:128793144-128819866
22605	MMT00030012		12+:114601948-114635798
22606	Cds2	110911	2+:132088977-132131122
22607	Bex1	19716	X-:132748511-132749977
22608	Ctnna3	216033	10+:64297325-64465713
22609	Gjd3	353155	11-:98843493-98844330
22610	Prdm9	213389	17-:15690199-15701304
22611	Tmem5	216395	10-:121516443-121517069
22612	1500010J02Rik	68964	11+:68840817-68849974
22613	Atp8a1	11980	5-:68013838-68238449
22614	MMT00005389		7-:12430599-12431453
22615	MMT00067023		6-:10122057-10122856
22616	A930001M12Rik	320500	4+:40420256-40421336
22617	MMT00037046		
22618	Ccl21c	65956	4+:41774242-41775336
22619	Noc3l	57753	19-:38380215-38893688
22620	Olfr1408	258759	1-:175060413-175061346
22621	Cklf	75458	8+:106774802-106787343
22622	Prl6a1	19111	13+:27404496-27411120
22623	Snupn	66069	9+:56798631-56831010
22624	Kcnj15	16516	16+:95517208-95518605
22625	Jarid1c	20591	X+:148668103-148707616
22626	Shprh	268281	10+:10884440-10935069
22627	Adck5	268822	15+:76406735-76426238
22628	EG626903	626903	5-:20621883-20635619
22629	Asb17	66772	3+:153507229-153516575
22630	MMT00034355		4+:24165577-24166314
22631	MMT00054932		11+:101337214-101338381
22632	Gpc4	14735	X-:49406098-49518429
22633	Ccdc124	234388	8-:73391902-73397281
22634	Rab27b	80718	18-:70143312-70213017
22635	Nrn1	68404	13-:36817495-36826349
22636	Trim2	80890	3-:83964367-84024783
22637	Angel2	52477	1+:192752398-192769672
22638	Astn2	56079	4-:65041836-65846485
22639	D17Wsu104e	28106	17-:56315976-56323320
22640	Bud13	215051	9+:46091104-46106864
22641	Kif1c	16562	11+:70514046-70545471
22642	Uts2	24111	4+:150371205-150375918

22643	Ece1	230857	4+:137469486-137519654
22644	Dennd3	105841	15+:73343175-73402652
22645	Zar1	317755	5-:72968354-72972376
22646	MMT00066171		5-:148679761-148680121
22647	Pkib	18768	10+:57351786-57456887
22648	Mpzl1	68481	1-:167522314-167564652
22649	MMT00042862	629761	2-:164488414-164499587
22650	Ccdc129	232016	6+:55925581-55928732
22651	Kif2a	16563	13-:107751339-107812194
22652	Olfr616	259103	7-:110712839-110713791
22653	Ric3	320360	7-:116181406-116226820
22654	Usp4	22258	9+:108250198-108294857
22655	Sorcs3	66673	19+:48678332-48879987
22656	Atp6ap1	54411	X+:71542517-71549477
22657	MMT00051642		9-:54671149-54673168
22658	BC011426	224893	17+:56031542-56038351
22659	Pex19	19298	1+:174056904-174064739
22660	Kif4	16571	X+:97821075-97922278
22661	Pilrb2	545812	5-:138307055-138313042
22662	H2-T24	15042	17-:36143599-36157436
22663	Olfr497	258733	7+:115566086-115567031
22664	9430020K01Rik	240185	18-:4681791-4682867
22665	Cgn	70737	3-:94571038-94590383
22666	R3hdm1	226412	1+:129999940-130134311
22667	Gcm1	14531	9+:77899730-77913242
22668	Rai14	75646	15-:10500395-10620182
22669	Ahctf1	226747	1-:181675029-181684203
22670	Clec4a1	269799	6+:122871991-122884638
22671	Wdr54	75659	6-:83102741-83106361
22672	Adam33	110751	2-:130876326-130882277
22673	Prpf38b	66921	3-:108705236-108714643
22674	4632411B12Rik	226976	1-:36392148-36426026
22675	Olfr133	258828	17+:38285534-38286471
22676	9030420J04Rik	71544	9-:9180015-9239026
22677	Prss2	22072	6+:41471786-41475078
22678	Daglb	231871	5+:144225464-144264470
22679	MMT00061143	100043315	15-:94927973-94979797
22680	Kcna4	16492	2+:107135079-107137043
22681	Gm88	224813	17+:46390464-46394264
22682	EG245305	245305	16+:98218417-98231335
22683	Vmn2r89	22301	14+:52030590-52080967
22684	Slco1a5	108096	6-:142182750-142271470
22685	Olfr370	258267	8+:86065044-86065985
22686	Acvr1	11477	2-:58299559-58419568
22687	Nr2f1	13865	13-:78328237-78338134
22688	4930432K21Rik	74666	8+:86672148-86696488
22689	2610028A01Rik	72400	14+:64479200-64538641
22690	A230050P20Rik	319278	9+:20673080-20678265
22691	Ercc6l	236930	X-:99338296-99339789
22692	Pik3c2b	240752	1+:134962876-134982189
22693	Trim68	101700	7-:109826100-109835764

22694	EG629219	629219	7-:14807826-14840218
22695	Tgoln2	22135	6-:72560950-72566742
22696	Ypel4	241525	2+:84574165-84578798
22697	Skil	20482	3+:30995754-31017908
22698	Olfr218	258880	1+:175133488-175134428
22699	Cntnap2	66797	6+:46120168-46184329
22700	Mrps18c	68735	5+:101227768-101233543
22701	EG545936	545936	7+:25623291-25625277
22702	Pdlim2	213019	14-:70564024-70577479
22703	Olfr599	258726	7+:110486569-110487515
22704	MMT00012998		4+:105161470-105184159
22705	Actr2	66713	11-:19962308-20012916
22706	D15Wsu75e	28075	15-:81824433-81846525
22707	Olfr666	259100	7-:112041183-112042140
22708	BC042782	230451	4+:94742350-94822970
22709	Proc	19123	18-:32282780-32295593
22710	MMT00075201		12+:36499659-36501226
22711	Gabra5	110886	7-:64663978-64746231
22712	Phf11	219131	14-:59895750-59916356
22713	Orm3	18407	4+:63017233-63020430
22714	Ptpn21	24000	12-:99914951-99972804
22715	Ap3m1	55946	14-:21855721-21871402
22716	Agr3	403205	12+:36652207-36676323
22717	EG620161	620161	6+:112926194-112926462
22718	1700093K21Rik	67358	11-:23416248-23419101
22719	Resp18	19711	1-:75268776-75274955
22720	Rpl8	26961	15+:76734528-76736374
22721	2200002J24Rik	69147	7+:31483969-31485558
22722	Ttc14	67120	3+:33700135-33706871
22723	MMT00064316		
22724	Adamts13	279028	2+:26864045-26864967
22725	4930504O13Rik	403200	11-:58259651-58266424
22726	Cdc42bpb	217866	12-:112531187-112615571
22727	Pdgfra	18595	5+:75548368-75594220
22728	Spg11	214585	2-:121879262-121890679
22729	MMT00014927		4+:135547508-135553800
22730	Glt8d1	76485	14+:31814891-31825171
22731	Mrpl50	28028	4-:49525381-49533953
22732	Cyb5b	66427	8+:109674576-109708000
22733	Alpk3	116904	7+:88202485-88250497
22734	Ap3s2	11778	7-:87025043-87065481
22735	MMT00007455		14-:109091411-109091860
22736	Il2rg	16186	X-:98460142-98463515
22737	MMT00035824		8+:52964916-52965501
22738	Atox1	11927	11-:55260143-55274633
22739	Olfr22-ps1	258209	11+:73768545-73769129
22740	Manbal	69161	2+:157204819-157222498
22741	Rbck1	24105	2-:152142071-152156838
22742	MMT00004923		14-:58731592-58731681
22743	Igh-2	238447	12-:114496927-116384147
22744	9-Sep	53860	11+:117193033-117223622

22745	Cntnap4	170571	8+:115093985-115406322
22746	Galnt9	231605	5+:110973424-111050392
22747	Gpc3	14734	X-:49625605-49967091
22748	Rbm13	67920	8-:32269939-32279233
22749	Tmem16d	320091	10-:88411776-88423519
22750	Mupcdh	72040	7-:148454983-148462673
22751	Nosip	66394	7+:52317862-52332786
22752	MMT00001763		4+:38610921-38611781
22753	Sidt2	214597	9-:45745944-45763804
22754	Bicd2	76895	13+:49437039-49478622
22755	Rpl32	19951	6-:115755576-115757915
22756	Gm438	329993	4-:144367105-144370548
22757	Slc44a2	68682	9+:21125187-21159471
22758	2810003C17Rik	108897	2+:31805822-31828961
22759	Btbd10	68815	7-:120459131-120513055
22760	Rlbp1l1	74438	4+:9198142-9377045
22761	Cep350	74081	1-:157787848-157820383
22762	Slc6a4	15567	11+:76812098-76845843
22763	Alcam	11658	16-:52249106-52310062
22764	MMT00049130		12+:60393050-60393923
22765	H2-M11	224754	17+:36684380-36685956
22766	MMT00003147		6-:105013478-105014830
22767	Nutf2	68051	8+:108384552-108403236
22768	Isl1	16392	13-:117089651-117099762
22769	Stx1a	20907	5+:135499441-135526969
22770	Slc27a2	26458	2+:126378890-126413671
22771	Dhx37	208144	5-:125894230-125895773
22772	Gm732	213450	X-:105141079-105143760
22773	Dkk4	234130	8+:23734514-23738016
22774	MMT00005320	625758	6+:131439062-131441511
22775	MMT00048918		3-:96258691-96259874
22776	Gsr	14782	8+:34764011-34807908
22777	Duox2	214593	2-:122106173-122123963
22778	Olfr64	18366	7-:111041325-111042985
22779	Pik3cg	30955	12-:32860500-32893335
22780	Gpr153	100129	4+:151653194-151659447
22781	Hibch	227095	1+:52901889-52977704
22782	Frrs1	20321	3+:116564609-116606559
22783	MMT00015001		2+:72954804-72955360
22784	Tex15	104271	8+:34681015-34696056
22785	MMT00078758		
22786	Mterfd3	74238	10-:84582176-84590769
22787	Olfr1230	258785	2-:89136508-89137425
22788	MMT00013067		11-:92567035-92654529
22789	2410025L10Rik	381668	5-:110790773-110825094
22790	Eif3j	78655	2+:121873198-121879365
22791	EG668831	668831	3-:128656700-128658013
22792	MMT00017271		8+:26350963-26537692
22793	Cbr2	12409	11-:120590822-120593309
22794	MMT00030143		1+:193301208-193301795
22795	Flt4	14257	11+:49423228-49465731

22796	MMT00048652		1+:3522003-3522786
22797	Prop1	19127	11-:50764307-50767134
22798	Pigw	70325	11-:84690525-84692003
22799	Olfr794	258375	10+:129007712-129008675
22800	Zbtb34	241311	2-:33173167-33267993
22801	1190005P17Rik	66225	10+:119664133-119668715
22802	Svs2	53878	2-:164062595-164063979
22803	Coq3	230027	4+:21806832-21837899
22804	Omt2a	18379	9-:78159809-78160890
22805	MMT00028156	619973	4-:57425173-57426990
22806	6330406I15Rik	70717	5+:150214382-150234277
22807	Mboat2	67216	12+:25516479-25644558
22808	Lrba	80877	3+:86028611-86586607
22809	Mrvi1	17540	7-:118014872-118089558
22810	MMT00038169		X+:119214523-119216090
22811	MMT00049679		3-:63856952-63857936
22812	Cybasc3	225912	19+:10651791-10664513
22813	MMT00052087		15+:73669856-73670337
22814	Cacna1s	12292	1+:137949360-138016105
22815	Gucy2g	73707	19-:55272586-55312588
22816	Arl4c	320982	1-:90597661-90619274
22817	1700027D21Rik	76573	10+:76025016-76032945
22818	Mdga1	74762	17-:29969000-29986717
22819	Fkbp2	14227	19-:7052362-7053627
22820	MMT00010881		18-:75330068-75330553
22821	Tmem135	72759	7-:96288714-96487270
22822	MMT00013433		19+:13866454-13867106
22823	Sh2d1a	20400	X+:39855846-39875258
22824	Hddc3	68695	7+:87488022-87490983
22825	Olfr1316	258737	2-:111970029-111970972
22826	Alox15	11687	11-:70158044-70165507
22827	Clec4a2	26888	6+:123073333-123092947
22828	Scyl1	78891	19-:5758427-5771401
22829	Sval2	84543	6+:41810337-41814320
22830	MMT00081088		10+:57106425-57107621
22831	Fut8	53618	12+:78432909-78576546
22832	Parp14	547253	16-:35860568-35871468
22833	Acat2	110460	17-:13135760-13153593
22834	Mtmr2	77116	9+:13553621-13610922
22835	Fbln1	14114	15+:85036440-85116084
22836	Grpr	14829	X-:159951835-159987571
22837	Acsl4	50790	X-:138755080-138824926
22838	2310003H01Rik	71885	11-:120230368-120240025
22839	Ehmt2	110147	17+:35035443-35050990
22840	MMT00042053		
22841	Cib1	23991	7-:87372251-87377446
22842	MMT00076318		15-:84980220-84990920
22843	Trim16	94092	11+:62633730-62664284
22844	Gabrg3	14407	7-:63979611-64642166
22845	Olfr564	258356	7+:109951993-109952986
22846	Sel1l	20338	12-:93044484-93071667

22847	Kcnh3	16512	15+:99055382-99073204
22848	H2afy3	67552	15-:62049097-62050958
22849	Gpn1	74254	5+:31797133-31813788
22850	Gabbr2	242425	4-:46680356-46888674
22851	Prhoxnb	231903	5-:148126390-148133920
22852	Nepn	66650	10+:52111413-52124410
22853	Olfr983	258824	9-:39899601-39900549
22854	Defb35	246084	8+:23048823-23051350
22855	Anapc2	99152	2+:25128025-25141434
22856	Pak2	224105	16-:31977031-32079410
22857	Fahd2a	68126	2-:127261951-127270277
22858	Fbxl17	50758	17-:63706093-63840237
22859	Rtf1	76246	2+:119500835-119561153
22860	Dtx2	74198	5+:136470746-136508734
22861	E430002G05Rik	210622	2+:102390172-102483195
22862	Kpna6	16650	4-:129325237-129338678
22863	Prkd2	101540	7+:17428328-17455812
22864	Gsx2	14843	5+:75471625-75473918
22865	MMT00044720		6-:131556410-131558977
22866	Lhx5	16873	5+:120881778-120891232
22867	Vps52	224705	17+:34102597-34103979
22868	Krt15	16665	11-:99993332-99997172
22869	Tceal8	66684	X-:132704872-132706725
22870	MMT00060201		7+:106221765-106239266
22871	Pgrmc2	70804	3-:40870260-40886895
22872	Dub2a	384701	7-:110398599-110401019
22873	Slc38a2	67760	15-:96517829-96530140
22874	Dnaja2	56445	8-:88061538-88079164
22875	Tmem106c	380967	15+:97794663-97800716
22876	Sash3	74131	X+:45499705-45514740
22877	MMT00056677		15+:23419645-23420281
22878	Nudt21	68219	8-:96543305-96560921
22879	1700049E22Rik	73395	6+:100477392-100483461
22880	Slc45a4	106068	15-:73410714-73436109
22881	1700045I19Rik	74264	X-:160198071-160199261
22882	MMT00011767	629750	11-:96656754-96657157
22883	Slc35f4	75288	14-:49918194-49942161
22884	Mapt	17762	11+:104092865-104189533
22885	Pank1	75735	19-:34885384-34952407
22886	6330527O06Rik	76161	2+:135883965-135895644
22887	1700020I14Rik	66602	2+:119420054-119427566
22888	Adrb3	11556	8-:28337270-28338916
22889	Foxj3	230700	4+:119213134-119300547
22890	D1Pas1	110957	1+:188791754-188793736
22891	MMT00041324		5+:138042787-138043419
22892	Olfr770	258862	10-:128569887-128570822
22893	Sip1	66603	12+:60114377-60129455
22894	MMT00016059		6-:28311913-28313176
22895	Gapvd1	66691	2-:34531704-34586167
22896	Chml	12663	1-:177616618-177618484
22897	MMT00053836		

22898	Cd300e	217306	11-:114914432-114995322
22899	Zfp609	214812	9-:65543016-65643302
22900	Olfr419	258710	1-:176180116-176181056
22901	7-Mar	57438	2+:60047992-60086441
22902	1700017G19Rik	72227	3-:40403805-40421835
22903	Mospd2	76763	X-:161374111-161418262
22904	Ift172	67661	5-:31555547-31556952
22905	MMT00020711	329639	3+:52543628-52579195
22906	Olfr606	259098	7+:110599852-110600810
22907	MMT00054594		6-:145264134-145265169
22908	Slc29a1	63959	17-:45722171-45729342
22909	Nup133	234865	8-:126423197-126473130
22910	Olfml3	99543	3-:103526144-103538153
22911	Cep350	74081	1-:157695219-157783011
22912	Stap1	56792	5+:86500862-86532832
22913	Zmynd10	114602	9+:107449787-107453366
22914	EG626280	626280	15+:76188886-76194156
22915	Abtb1	80283	6-:88785910-88791861
22916	Tal1	21349	4+:114732131-114744360
22917	G6pdx	14381	X-:71654824-71674205
22918	MMT00002192		12+:58815050-58838891
22919	Lig1	16881	7+:13862631-13896740
22920	Hoxb7	15415	11+:96145876-96151639
22921	2810038D20Rik	67218	14+:45202804-45208076
22922	2310010M24Rik	71897	2+:49643207-49804366
22923	Zfml	18139	6+:83864360-83936855
22924	Midn	59090	10+:79611034-79621112
22925	Parp16	214424	9+:65062928-65087021
22926	Ergic2	67456	6-:148128566-148160559
22927	1700001L05Rik	69291	15-:83187963-83197643
22928	Sdf2	20316	11+:78059504-78068981
22929	Rnf14	56736	18+:38456342-38477457
22930	Mro	71263	18+:74019046-74040987
22931	Tcf25	66855	8+:125897447-125928056
22932	Polh	80905	17-:46309020-46339579
22933	Tssc4	56844	7+:150255272-150256992
22934	4932425I24Rik	320214	16-:38297839-38341914
22935	Hmga2	15364	10-:119799147-119914024
22936	Rhobtb1	69288	10+:68675916-68752765
22937	Glrx3	30926	7+:144629332-144659865
22938	Lrit3	242235	3-:129491208-129503728
22939	C1galt1	94192	6+:7795263-7821454
22940	2310042E22Rik	66561	16-:21152741-21154017
22941	Hs6st3	50787	14+:119537453-119538340
22942	Bzrpl1	70026	17-:48587286-48590827
22943	Tpd52l1	21987	10-:31052185-31165727
22944	Tmem44	224090	16-:30513777-30550621
22945	Cpb2	56373	14+:75642147-75683358
22946	MMT00071077		10+:41023784-41077470
22947	Zfp358	140482	8+:3493170-3497208
22948	MMT00069572		14-:57455124-57455950

22949	Il1f6	54448	2+:24071366-24080115
22950	Lsm2	27756	17+:35119103-35122834
22951	Sstr4	20608	2+:148221206-148222363
22952	Dppa1	347708	11-:46421955-46442779
22953	Mvp	78388	7-:134130381-134158106
22954	Slc25a30	67554	14-:76161805-76186844
22955	4930430E16Rik	73873	11+:22908372-22921138
22956	1700034K16Rik	73314	4-:14592901-14723213
22957	1300018I05Rik	74157	17+:29797639-29839630
22958	Vwa2	240675	19+:56980879-56983873
22959	Zfp174	385674	16+:3847872-3854811
22960	Olfr714	259035	7+:114217343-114218297
22961	MMT00055950	665565	15+:59070014-59070558
22962	MMT00013609		4+:101523329-101548604
22963	Ctso	229445	3+:81736513-81756214
22964	C9	12279	15+:6395332-6448476
22965	Frmd7	385354	X-:48248146-48255286
22966	Epha4	13838	1-:77365846-77511663
22967	Egln2	112406	7-:27943677-27951732
22968	Jam3	83964	9-:26904969-26962965
22969	Axin2	12006	11+:108781797-108810797
22970	D17Ertd663e	52042	17-:13012718-13014031
22971	Zfp644	52397	5-:107119623-107125592
22972	9030617O03Rik	217830	12+:102024351-102110558
22973	MMT00067195		17+:17785784-17786290
22974	Olfr1377	258913	11+:50798204-50799128
22975	Med14	26896	X-:12321842-12338825
22976	Parvg	64099	15+:84155191-84172205
22977	MMT00025397		17-:80332850-80333959
22978	MMT00043176		5+:106644617-106645918
22979	Edem2	108687	2-:155527763-155555122
22980	Nat6	56441	9+:107483144-107486378
22981	Bcar3	29815	3+:122122744-122233098
22982	Mug2	17837	6+:121956815-122035983
22983	Ctdsp1	227292	1+:74438471-74443836
22984	Tnnt2	21956	1+:137732981-137748837
22985	EG665330	665330	9-:58993442-58994024
22986	Prkg1	19091	19-:31004875-31067652
22987	Eif2s2	67204	2-:154698172-154718511
22988	Hscb	100900	5-:111258092-111265448
22989	Lmcd1	30937	6+:112223811-112280418
22990	Rps2	16898	17+:24857057-24858853
22991	Rab11b	19326	17-:33883635-33897242
22992	A430107O13Rik	214642	6+:22038688-22187742
22993	Syt6	54524	3+:103379300-103434850
22994	Cpne3	70568	4-:19446401-19497257
22995	Drd2	13489	9+:49148731-49216282
22996	MMT00076852		14-:45341673-45344626
22997	Gbf1	107338	19+:46227026-46360999
22998	Ass1	11898	2+:31325821-31376149
22999	MMT00043885		4-:155283451-155284852

23000	Faim2	72393	15-:99330579-99358025
23001	Galm	319625	17+:80526793-80584441
23002	MMT00009646		15-:75797172-75798020
23003	EG209550	209550	12+:11462884-11469489
23004	Ccnd3-ps	12446	4-:138900309-138902193
23005	4930405A21Rik	74846	2-:156540273-156542803
23006	Try4	22074	6+:41252283-41255485
23007	2410015N17Rik	66422	7-:134400475-134404181
23008	Ctsz	64138	2-:174252995-174264503
23009	Amdhd2	245847	17-:24292798-24295670
23010	MMT00047055		7-:14547637-14551245
23011	MMT00012889		13+:15906733-15907217
23012	Spg20	229285	3+:54916061-54939876
23013	MMT00077564		18+:90749215-90749284
23014	MMT00077643		
23015	Zc3h12d	237256	10+:7559632-7587867
23016	Atg9b	213948	5-:23895599-23896441
23017	MMT00013118		4+:20318347-20319375
23018	Magi2	50791	5+:19413589-20208738
23019	ENSMUSG00000(	791318	18-:5574983-5583802
23020	Wdr34	71820	2-:29887066-29904398
23021	Sfxn3	94280	19+:45122306-45130870
23022	Kif3a	16568	11+:53380894-53414604
23023	Rassf10	78748	7+:120068482-120099234
23024	Tcra	21473	14+:54285603-54286191
23025	Foxi2	270004	7+:142602121-142603714
23026	Apom	55938	17-:35265942-35268697
23027	Glud1	14661	14+:35124008-35158089
23028	Erv3	71995	2-:131681151-131685483
23029	Zfp27	22689	7-:30629768-30728569
23030	Cyp26b1	232174	6-:84523974-84543912
23031	Gria4	14802	9-:4793809-4796207
23032	Mett10d	67493	11+:74584364-74632194
23033	MMT00072013		15-:54596155-54597015
23034	Slc25a29	214663	12-:110064086-110073959
23035	AI848100	226551	1-:163746983-163806743
23036	Stk19	54402	17-:34961223-34973821
23037	Thbs3	21827	3+:89019128-89030738
23038	Casp4	12363	9+:5308873-5336707
23039	Ift52	245866	2+:162843088-162872280
23040	Slc34a1	20505	13+:55501064-55515452
23041	Kdelc1	72050	1-:44163895-44175380
23042	Bloc1s3	232946	7-:20091652-20092950
23043	Olfr930	258269	9+:38737757-38738682
23044	Ctr9	22083	7+:118172534-118199850
23045	Oas1d	100535	5+:121364872-121371661
23046	Zdhhc4	72881	5-:144078171-144090900
23047	Xrn1	24127	9+:95855287-95953047
23048	2310002J15Rik	67859	2-:25094346-25095417
23049	4933403O08Rik	71030	X+:109352657-109357462
23050	BC065397	436230	X-:133337375-133340598

23051	MMT00046509		17+:33896950-33897011
23052	EG634331	634331	1+:99059343-99190012
23053	1700012B09Rik	69325	9-:14562643-14575474
23054	V1rb8	113856	6+:89926164-89927093
23055	Lmo1	109594	7-:116282084-116314027
23056	Ccdc135	330830	8+:97579794-97602039
23057	Rpl13	270106	8+:125626332-125629061
23058	Prph	19132	15+:98885637-98889100
23059	Fbxl20	72194	11-:97943868-97945481
23060	8430422H06Rik	74529	18+:14390811-14437050
23061	Pole2	18974	12-:70302763-70329160
23062	Tiprl	226591	1-:167145714-167167034
23063	Rbmx2	209003	X+:46048178-46063899
23064	Myh2	17882	11+:66984720-67011014
23065	Ppp3cc	19057	14-:70617705-70689256
23066	Gtf2a1	83602	12-:92798108-92828019
23067	Slc24a2	76376	4-:86636869-86722069
23068	Rnf126	70294	10-:79221284-79229669
23069	Tmem117	320709	15+:94709811-94925436
23070	BC024659	108934	13+:41345546-41368403
23071	Megf10	70417	18+:57349154-57453944
23072	MMT00048970		5+:4764813-4765730
23073	4833414E09Rik	71633	1-:170349770-170350960
23074	3021401N23Rik	70680	13-:108832313-108834513
23075	MMT00038489		10+:91947044-91947522
23076	Kcnc2	268345	10+:111708177-111709460
23077	Daam2	76441	17-:49597765-49638260
23078	Msrb2	76467	2+:19293272-19316602
23079	Spag17	74362	3+:99689353-99867396
23080	Dmrta1	242523	4+:89355063-89358966
23081	Golga2	99412	2+:32143875-32163432
23082	Gria3	53623	X+:38754687-39029736
23083	Olfr6	233670	7-:114099499-114100103
23084	Vsig4	278180	X-:93442546-93488775
23085	Olfr1336	258917	7+:6413221-6414165
23086	MMT00059956		
23087	Nusap1	108907	2+:119444452-119475512
23088	1700009N14Rik	75471	4+:39397828-39398478
23089	Slc1a2	20511	2+:102546594-102618263
23090	Anxa7	11750	14-:21274498-21299038
23091	Parvb	170736	15+:84062501-84143460
23092	Ifih1	71586	2-:62435396-62484223
23093	Mical1	171580	10+:41196182-41206834
23094	Sap30	60406	8-:59961523-59966654
23095	2410137M14Rik	76797	17-:37114645-37118182
23096	Wdr60	217935	12-:117494300-117501482
23097	Pip4k2c	117150	10-:126635144-126648613
23098	Wdr4	57773	17-:31632568-31649202
23099	Cyp2d12	380997	15+:82385503-82395648
23100	MMT00027568	544718	10+:89495957-89533914
23101	Pnliprp1	18946	19+:58803376-58818659

23102	Fbxo7	69754	10+:85484672-85511072
23103	4932431H17Rik	545527	3-:58420233-58425256
23104	Gp2	67133	7-:126586057-126602786
23105	MMT00047833		17+:71306438-71307258
23106	Rdh18	380674	10+:127261395-127278955
23107	MMT00049669		1+:183020913-183022059
23108	Map4k3	225028	17-:80979851-81127176
23109	A430088P11Rik	106059	15-:80526972-80528033
23110	Prg2	19074	2+:84821494-84823674
23111	Eml5	319670	12-:100101306-100139238
23112	Gys1	14936	7+:52690358-52711978
23113	Olfr617	258838	7+:110732537-110733492
23114	Fbxl8	50788	8+:107788637-107793225
23115	Mad2l1	56150	6+:66485472-66489946
23116	2810032G03Rik	72669	12+:5382675-5423111
23117	Slc9a1	20544	4+:132926143-132979611
23118	Ttrap	56196	13+:24923553-24934015
23119	Cps1	227231	1+:67169601-67277833
23120	Prrxl1	107751	14+:33412949-33442185
23121	Agps	228061	2+:75670230-75764098
23122	Fat3	270120	9-:15719442-15835864
23123	Acadsb	66885	7+:138554216-138589718
23124	4932411E22Rik	214604	11-:89253539-89280263
23125	Mrpl51	66493	6+:125142219-125144410
23126	1700013G24Rik	69380	4+:137009211-137011376
23127	Ard1b	97243	5-:97820659-97821316
23128	Fras1	231470	5+:97188474-97210999
23129	MMT00035335		9-:14612082-14612496
23130	Hps4	192232	5+:112773971-112807434
23131	Pla1a	85031	16-:38396204-38432968
23132	Mcm6	17219	1-:130228167-130256233
23133	EG434373	434373	9+:16834410-16835649
23134	Lce3c	94060	3+:92748407-92749649
23135	Btg4	56057	9+:50924222-50927728
23136	Usp48	170707	4+:137150144-137181206
23137	MMT00015670		4-:133052889-133053426
23138	Osbpl6	99031	2+:76386396-76434101
23139	Ggta1	14594	2-:35255733-35316848
23140	Galnt12	230145	4+:47104824-47135914
23141	Ebf2	13592	14+:67853597-68048604
23142	Sec14l1	74136	11-:117019581-117020576
23143	Acadm	11364	3-:153585322-153607390
23144	9430025M13Rik	233147	7+:46735274-46753703
23145	Syne1	64009	10+:5019058-5022437
23146	Nos3	18127	5+:23870648-23889814
23147	Sox21	223227	14-:118633346-118635070
23148	MMT00063766		8+:126377691-126378135
23149	Nln	75805	13-:104813136-104899682
23150	EG622657	622657	14+:34026276-34026984
23151	Sdk1	330222	5+:142058245-142089195
23152	Kif27	75050	13+:58374894-58397948

23153	Angptl1	72713	1+:158769072-158775746
23154	Rbm42	68035	7-:31426020-31435184
23155	Mycn	18109	12-:12943813-12947199
23156	MMT00013465	433597	3+:59547517-59556206
23157	MMT00001961		18+:6516319-6557797
23158	Snx11	74479	11+:96628105-96630217
23159	Myh8	17885	11+:67090934-67122132
23160	Cdh18	320865	15+:22865462-22899521
23161	Gclc	14629	9+:77602352-77642269
23162	Csda	56449	6-:131314875-131338449
23163	1700106N22Rik	73582	17+:85790911-85851667
23164	MMT00051283		14-:66579956-66581727
23165	Fxn	14297	19-:24335942-24355076
23166	MMT00018155		X+:142529876-142530933
23167	Slit3	20564	11+:35321724-35525533
23168	Tmub1	64295	5-:23951281-23953623
23169	Pla2g2e	26970	4+:138433856-138438727
23170	MMT00004222		4+:39770983-39771300
23171	Reg3d	30053	6-:78325867-78328857
23172	Atp13a4	224079	16-:29396130-29544951
23173	Cuzd1	16433	7-:138452068-138465806
23174	Psme2	19188	14-:56206322-56209726
23175	Acsm4	233801	7+:126838122-126854941
23176	Pgk1	18655	X+:103382567-103398999
23177	Il17a	16171	1+:20720998-20724534
23178	Odz3	23965	8-:49313044-49760044
23179	Olfr575	259118	7-:110103179-110104134
23180	Sult1d1	53315	5-:87984278-87997909
23181	Wdr17	244484	8-:55739231-55746250
23182	Ace3	217246	11+:105856044-105865922
23183	Nnmt	18113	9-:48399981-48413258
23184	9330182L06Rik	231014	5+:9380276-9479731
23185	Ndufs1	227197	1-:63190171-63223283
23186	4933436H12Rik	71244	7+:75562308-75571018
23187	MMT00044007		18-:53380936-53459300
23188	MMT00013081		3+:112822792-112826285
23189	Rbm28	68272	6-:29075020-29108200
23190	MMT00078777		18+:68067307-68068742
23191	3632451O06Rik	67419	14-:50301234-50403003
23192	EG665509	665509	18-:16433158-16649832
23193	Akap9	100986	5+:3928282-4069207
23194	Rab3c	67295	13-:110852024-111070226
23195	Mysm1	320713	4-:94617616-94645794
23196	MMT00005166		6-:20375053-20375498
23197	Sra1	24068	18-:36826900-36829947
23198	Moap1	64113	12-:103980440-103981498
23199	EG435927	435927	6-:141657043-141660656
23200	2610206C17Rik	72495	7+:91754703-91925059
23201	S1pr1	13609	3-:115413356-115417963
23202	Olfr559	259116	7-:109872045-109873002
23203	Drg2	13495	11+:60268118-60282207

23204	2900073G15Rik	67268	17-:71342995-71351562
23205	MMT00060665		7-:108722053-108722838
23206	Dgat1	13350	15-:76332607-76342240
23207	Rab7	19349	6-:87949116-87995214
23208	Hck	15162	2+:152934322-152977162
23209	Immp1l	66541	2+:105770904-105805487
23210	Tprgl	67808	4-:153531596-153534775
23211	Pscd3	19159	5+:144383421-144471109
23212	Polr2b	231329	5+:77739505-77778522
23213	Txndc3	73412	13-:19780805-19788919
23214	2610019A05Rik	72149	11-:106024649-106054862
23215	Mphosph6	68533	8-:120315544-120325829
23216	Acvr1c	269275	2-:58127024-58168434
23217	Acyp2	75572	11-:30532202-30549408
23218	Rttn	246102	18+:89215494-89265038
23219	Wfdc5	209232	2-:164003360-164008478
23220	Zranb2	53861	3+:157197284-157206402
23221	MMT00061047		1+:193231584-193232612
23222	MMT00008477		10-:30501081-30502184
23223	MMT00076151		19-:3730467-3731012
23224	C030014K22Rik	226610	1+:168931696-169009599
23225	MMT00072643		
23226	Suox	211389	10-:128106948-128110938
23227	Grin2a	14811	16-:9577802-9992626
23228	MMT00012254		5-:19011108-19011911
23229	Phf12	268448	11+:77796254-77844034
23230	1110014K08Rik	319370	11+:116294940-116300386
23231	1700023E05Rik	71868	5+:77445066-77490556
23232	Crisp3	11572	17-:40358727-40379215
23233	Npw	381073	17-:24794273-24795087
23234	Chd3	216848	11-:69156774-69162112
23235	Vwa2	240675	19+:56961367-56978013
23236	MMT00038991		6-:49493301-49716696
23237	Irs1	16367	1-:82283372-82287068
23238	Zfp407	240476	18-:84485197-84601826
23239	Aadacl1	320024	3+:27081922-27177269
23240	Gtpbp10	207704	5-:5537463-5559535
23241	Nkpd1	69547	7+:20107282-20110122
23242	Gngt2	14710	11+:95703983-95707043
23243	MMT00042878		1+:179716978-179717638
23244	MMT00006045	100046034	2-:157347388-157347807
23245	Nfib	18028	4-:81941984-82151195
23246	Cited2	17684	10+:17443052-17444594
23247	Ercc1	13870	7+:19930454-19941871
23248	Tesk2	230661	4+:116393581-116476707
23249	MMT00013167		
23250	Plcl2	224860	17+:50745613-50827811
23251	A930001N09Rik	77128	17+:26852619-26908350
23252	Snrpd1	20641	18+:10617864-10627968
23253	D030011O10Rik	320560	6-:148941534-149049831
23254	Wdtc1	230796	4-:132849494-132895173

23255	H13	14950	2+:152495215-152530753
23256	Olfr1026	258577	2+:85763426-85764349
23257	Igh-VJ558	16061	12-:115920833-116020075
23258	Rhox4c	434759	X+:34894882-34899631
23259	Dnajc1	13418	2-:18135381-18136521
23260	MMT00051542		X+:135893005-135893840
23261	Clic6	209195	16+:92498379-92541482
23262	C530043G21Rik	215015	1-:158611184-158649130
23263	Ppm2c	381511	4-:11886961-11893565
23264	Defb10	246085	8+:22969372-22972480
23265	Tmem67	329795	4-:11966263-12015084
23266	Cenpq	83815	17-:41060003-41071500
23267	Hsd17b11	114664	5-:104419413-104450797
23268	Patz1	56218	11+:3190303-3209324
23269	Rnf17	30054	14+:57090127-57143240
23270	Nutf2	68051	8+:108384552-108403237
23271	Isca1	69046	13-:59858080-59871130
23272	Orc1l	18392	4+:108252080-108287434
23273	Glg1	20340	8-:113681459-113783102
23274	1700054O13Rik	67334	X+:9424072-9424626
23275	Hoxa7	15404	6-:52165624-52168572
23276	Evi2b	216984	11-:79328524-79337196
23277	Suv420h2	232811	7+:4691789-4699089
23278	Cd46	17221	1-:196868147-196918439
23279	9-Sep	53860	11+:117060973-117152353
23280	Dscam	13508	16-:96814550-97391987
23281	1700126L10Rik	73603	2-:164295801-164311797
23282	MMT00063825		X+:111775882-111915446
23283	Mrps12	24030	7-:29524660-29526799
23284	MMT00071882		8-:4724307-4724808
23285	Srrd	70118	5-:112766411-112772045
23286	Trhr2	170732	8-:124880866-124884646
23287	Supt4h1	20922	11+:87551066-87557118
23288	Cramp1l	57354	17-:25148246-25150969
23289	Cyp46a1	13116	12+:109572590-109600443
23290	MMT00061154		X+:99333976-99334474
23291	Rexo1	66932	10-:80005017-80009547
23292	0710008K08Rik	69225	8+:11497601-11513284
23293	N6amt1	67768	16+:87354466-87368415
23294	MMT00035523		4+:90035421-90036411
23295	Snx11	74479	11-:96629190-96638834
23296	Ddx59	67997	1+:138311847-138336728
23297	Umod	22242	7-:126606486-126621826
23298	Pno1	66249	11-:17103204-17111556
23299	Ubox5	140629	2-:130417067-130455700
23300	C2cd3	277939	7+:107548176-107558960
23301	Scamp1	107767	13-:94971265-95055382
23302	MMT00025620		2-:94430818-94431258
23303	Olfr1302	258891	2+:111620469-111621415
23304	Ptf1a	19213	2+:19367289-19369119
23305	Trpm5	56843	7-:150257696-150275513

23306	Bag2	213539	1-:33802330-33814593
23307	Myst2	217127	11-:95135573-95171492
23308	Dock9	105445	14-:121941369-121980657
23309	Vmn2r103	627636	17+:19994491-20010795
23310	Ush2a	22283	1+:190085913-190276280
23311	2310010M20Rik	69576	16+:32271546-32274864
23312	Fsd1l	319636	4+:53644456-53714263
23313	Htr1b	15551	9-:81524999-81526159
23314	A630033E08Rik	240041	17-:22851106-22989733
23315	Olfr460	258381	6+:40521386-40522331
23316	Adam28	13522	14-:69224139-69273840
23317	MMT00018454		2-:36663308-36663936
23318	Cul2	71745	18+:3383028-3436372
23319	6230427J02Rik	68176	9-:107886552-107888211
23320	3110053B16Rik	382686	12-:20879559-20883033
23321	Npff	54615	15-:102354271-102355052
23322	Ard1	56292	X-:71162215-71167267
23323	Olfr904	258797	9+:38271627-38272559
23324	Prdx6	11758	1-:163171140-163181296
23325	Wdr70	545085	15-:7823057-8049208
23326	Il7r	16197	15-:9435915-9459544
23327	Brp44l	55951	17+:8476755-8490027
23328	Rora	19883	9+:68501608-69226833
23329	MMT00004368		14+:53047641-53048592
23330	Tmem110	69179	14+:31638799-31688307
23331	Csmd1	94109	8-:15895502-17535281
23332	Sos2	20663	12-:70684752-70759065
23333	Abhd7	384214	5+:107848786-107859048
23334	Olfr692	258352	7+:112516841-112517829
23335	MMT00030313		9-:3495833-3496959
23336	Gpr26	233919	7+:139158610-139176622
23337	Tcf7	21414	11-:52066738-52096516
23338	MMT00007161		X+:35094607-35097311
23339	Olfr871	258905	9+:20016794-20017729
23340	Arhgef16	230972	4-:153665059-153674138
23341	MMT00033853		11-:84021278-84021707
23342	Ndfip2	76273	14+:105657864-105708513
23343	MMT00022044		5-:42266142-42384482
23344	Nfkbiz	80859	16-:55811490-55838967
23345	MMT00076071		
23346	Mtch2	56428	2+:90687413-90705547
23347	Lingo4	320747	3+:94205753-94207534
23348	Calml4	75600	9+:62705893-62723722
23349	Ap1m2	11768	9-:21099901-21116751
23350	BC048546	232400	6-:128489837-128531614
23351	Zfp598	213753	17+:24806798-24818959
23352	MMT00001681		X+:14942325-14942614
23353	Slmo2	66390	2-:174290600-174298399
23354	Tshr	22095	12+:92639449-92777167
23355	Nedd1	17997	10-:92147490-92185105
23356	Gpr98	110789	13-:81644732-81723855

23357	Ccnyl1	227210	1+:64759637-64772202
23358	MMT00048666	381484	3-:15863318-15906322
23359	Dusp2	13537	2+:127161973-127162641
23360	MMT00066283		5-:52783909-52830585
23361	Bivm	246229	1+:44176854-44201592
23362	Aplp2	11804	9-:30957142-31019400
23363	Wibg	78428	10+:128185581-128203617
23364	Ina	226180	19+:47089170-47099863
23365	Rcor1	217864	12+:112278169-112354105
23366	ENSMUSG00000(	100038591	10+:126631989-126637805
23367	Tm2d2	69742	8+:26127751-26133731
23368	6330503K22Rik	101565	7+:125858887-125880534
23369	Oaz2	18247	9+:65524399-65537263
23370	Cpeb4	67579	11+:31820408-31832197
23371	Cfb	14962	17-:34993326-34999162
23372	Rbm5	83486	9-:107642825-107673314
23373	Vps13d	230895	4-:144753078-144784961
23374	EG231836	231836	5+:140938130-140945725
23375	MMT00055444		5-:100268536-100269009
23376	Dlk1	13386	12+:110691715-110698828
23377	Pdxk	216134	10-:77903349-77927724
23378	RP23-357I14.1	381417	2+:122347366-122350632
23379	MMT00043310		7+:50122593-50151331
23380	Ddhd2	72108	8-:26838129-26864886
23381	MMT00003885		6+:41080121-41080591
23382	MMT00079398		9-:98773480-98774346
23383	Setd7	73251	3-:51320071-51322248
23384	Grid2	14804	6+:63206850-64616272
23385	4833422F24Rik	74614	2-:165220949-165225894
23386	2310002J21Rik	66360	19-:54002646-54006722
23387	Psma1	26440	7-:121407968-121419616
23388	Olfr561	259096	7+:109923039-109923984
23389	Ero1lb	67475	13+:12658174-12703663
23390	MMT00057645		9-:84866668-84868520
23391	MMT00008521		8-:48712340-48713397
23392	3830417A13Rik	70696	X+:61426723-61431936
23393	MMT00034099		3-:120953050-120953655
23394	Psd4	215632	2+:24223102-24264686
23395	MMT00061495		4+:144381739-144382372
23396	Krtap16-10	71369	16-:88962808-88963044
23397	Ccnjl	380694	11+:43342856-43399806
23398	Adamts5	23794	16-:85896279-85900512
23399	Caskin1	268932	17+:24625644-24644160
23400	BC024997	215723	11+:68369687-68371746
23401	H2-Q2	15013	17+:35479254-35496927
23402	Ugt2b36	231396	5-:87494950-87521610
23403	Ndc80	67052	17-:71845444-71876197
23404	Phf23	78246	11+:69809278-69813418
23405	Rps9	76846	7+:3657387-3658473
23406	4922501K12Rik	105511	14+:33647206-33649971
23407	MMT00007320		8-:79386108-79386947

23408	Clip4	78785	17+:72130638-72213550
23409	Gm1157	382384	10-:79102639-79105438
23410	C1qa	12259	4-:136451831-136454675
23411	Mtx2	53375	2+:74663904-74714795
23412	EG625690	625690	10-:84256896-84257449
23413	Hmx2	15372	7+:138692286-138700100
23414	Tnfaip8l2	69769	3-:94943442-94946282
23415	Acp5	11433	9-:21931193-21935098
23416	ENSMUSG00000(	628709	13-:65498322-66077477
23417	Gtpbp1	14904	15+:79521438-79550569
23418	4933402J15Rik	74425	14+:74643272-74698619
23419	Tipin	66131	9+:64129413-64152599
23420	Ehbp1	216565	11-:21905829-22185594
23421	Ube2q1	70093	3+:89577613-89587915
23422	Aggf1	66549	13-:96120638-96145292
23423	Pla2g6	53357	15-:79117181-79148399
23424	AI450540	226252	19+:57435590-57447892
23425	Tob1	22057	11+:94074953-94076040
23426	Arvcf	11877	16+:18380875-18406840
23427	MMT00054636	654456	8+:19300676-19300738
23428	Ndufb4	68194	16-:37647687-37654454
23429	Adck4	76889	7+:28019007-28042964
23430	Aifm3	72168	16+:17489783-17507574
23431	Serpinb9e	20710	13+:33341490-33352713
23432	Rtn4rl2	269295	2-:84711487-84726849
23433	Ugcgl2	66435	14-:119385415-119405921
23434	ENSMUSG00000(	623548	17-:32213903-32237231
23435	Rgs11	50782	17+:26339917-26348263
23436	Slc6a7	240332	18-:61160075-61169259
23437	Efhd2	27984	4-:141414303-141430824
23438	C1galt1c1	59048	X-:35983961-35988230
23439	Tmem59	56374	4+:106851283-106873597
23440	EG243642	243642	6+:127176793-127184635
23441	Lama3	16774	18+:12492525-12741518
23442	MMT00047160		2+:174681781-174692362
23443	Olfr1002	258566	2-:85487519-85488476
23444	MMT00012529		3-:108252030-108254484
23445	Crlf1	12931	8+:73017056-73027980
23446	Acsl6	216739	11+:54117703-54175026
23447	Smyd5	232187	6+:85381982-85395772
23448	9430025M13Rik	233147	7+:46704890-46729636
23449	Prelid2	77619	18-:42092246-42110876
23450	D12Ertd647e	52668	12+:104672413-104678454
23451	MMT00059918		6+:141086547-141087128
23452	EG434123	434123	7-:14917286-14918908
23453	Dnajc3a	100037258	14+:119337211-119377404
23454	Znhit1	70103	5-:137458062-137462879
23455	Ptafr	19204	4+:132120002-132136239
23456	Tbxa2r	21390	10+:80791574-80797916
23457	Selenbp2	20342	3+:94497494-94508328
23458	Clic4	29876	4-:134769886-134828675

23459	Mbtd1	103537	11+:93773722-93806615
23460	Rragd	52187	4+:33070410-33109147
23461	Mcoln3	171166	3+:145784754-145803610
23462	Bag5	70369	12-:112947703-112951505
23463	Gimap1	16205	6+:48692180-48693793
23464	Srgap1	117600	10-:121222294-121368565
23465	Afp	11576	5+:90919765-90937882
23466	Pou2f1	18986	1-:167805606-167932738
23467	Amac1	56293	11-:69573410-69575346
23468	MMT00058901		3-:51206065-51207112
23469	MMT00020032	620790	2-:174994321-174995963
23470	Tmod1	21916	4+:46073837-46127676
23471	Ddx46	212880	13+:55754271-55780492
23472	Cts3	117066	13-:61589372-61669779
23473	4933401F05Rik	71037	14-:64694273-64704232
23474	MMT00011721		2+:83819001-83822053
23475	Nudt12	67993	17-:59140823-59152745
23476	Ppp1r9a	243725	6+:4995935-5110715
23477	Uba1	22201	X+:20235564-20259829
23478	Sacs	50720	14+:61821599-61833091
23479	Sox10	20665	15-:78985354-78995671
23480	Eml4	78798	17+:83750306-83879678
23481	Prkch	18755	12+:74686090-74878110
23482	Syt4	20983	18-:31598839-31606860
23483	Stxbp4	20913	11-:90341249-90499398
23484	Fbxo36	66153	1+:84836429-84897059
23485	Olfr716	258597	7+:114290831-114291775
23486	MMT00029075		5-:112649088-112650782
23487	Polb	18970	8-:23738737-23763886
23488	Ier5l	72500	2-:30328167-30329718
23489	Tbc1d15	66687	10-:114635514-114688481
23490	Fndc3b	72007	3-:27315076-27362768
23491	Dixdc1	330938	9-:50474092-50536043
23492	Krtcap2	66059	3+:89050359-89053644
23493	Tgs1	116940	4+:3502172-3542151
23494	Tubal3	238463	13+:3927334-3932930
23495	Cryab	12955	9+:50560862-50564738
23496	Zfp40	22700	17-:23311845-23315506
23497	Mchr1	207911	15+:81066203-81068541
23498	2010204N08Rik	69983	3-:72692485-72704389
23499	Edil3	13612	13+:89429023-89461489
23500	BC055004	381680	5+:138671109-138693280
23501	BC049762	193286	11-:51067149-51077146
23502	Pcdh11x	245578	X+:118019398-118024201
23503	Zim1	22776	7-:6628727-6649140
23504	MMT00072241		5-:64581272-64582250
23505	Bicd1	12121	6+:149357521-149506340
23506	Cts6	58518	13-:61296507-61304753
23507	MMT00010687		14+:59512322-59513004
23508	Pdgfd	71785	9+:6288469-6379261
23509	AI595366	432779	13-:74498122-74501407

23510	Spn	20737	7-:134278135-134280850
23511	Sec31b	240667	19-:44591800-44620339
23512	Olfr42	18341	13+:21221472-21225599
23513	Il18rap	16174	1+:40572206-40606142
23514	Hdac8	70315	X-:99479978-99700702
23515	Ptprm	19274	17-:67016882-67703727
23516	Mrpl28	68611	17+:26260476-26263556
23517	Pmm2	54128	16+:8637799-8657616
23518	Abhd4	105501	14+:54878971-54888839
23519	Cbfa2t2	12396	2+:154262338-154343468
23520	Wdr5	140858	2+:27013770-27392053
23521	St8sia3	20451	18+:64425058-64431875
23522	Adcy10	271639	1+:167415311-167506900
23523	MMT00039380		
23524	MMT00080757		17+:14550488-14551566
23525	Dctn4	67665	18+:60685821-60716143
23526	Fabp7	12140	10+:57502089-57502590
23527	MMT00021936		5-:30133239-30134698
23528	0610038L08Rik	74092	17-:29715751-29733251
23529	Golm1	105348	13-:59736355-59767042
23530	Drd4	13491	7+:148477936-148480759
23531	Foxb2	14240	19-:16946806-16948320
23532	Adra2b	11552	2+:127189315-127190661
23533	Ccnb1ip1	239083	14-:51408923-51413549
23534	MMT00040702		
23535	Yipf3	28064	17+:46385047-46388965
23536	Tapbpl	213233	6-:125174238-125181878
23537	Ndor1	78797	2-:25100339-25110929
23538	MMT00081764		1-:9772263-9784342
23539	Sntg1	71096	1-:8353556-8794024
23540	Olfr155	29845	4+:43867163-43868335
23541	MMT00002245		2-:110049431-110050374
23542	Olfr895	258875	9+:38076099-38077048
23543	Rufy3	52822	5+:89012560-89070631
23544	Atg4c	242557	4+:98884666-98925779
23545	Adamtsl3	269959	7+:89484204-89761006
23546	Zzef1	195018	11+:72680105-72683429
23547	Tmed7	66676	18-:46745311-46757190
23548	Crtac1	72832	19-:42358019-42506123
23549	Es22	13897	8-:95725117-95753440
23550	Ap2m1	11773	16+:20535576-20544125
23551	Foxe3	30923	4-:114597752-114598618
23552	Rasl11b	68939	5+:74591259-74595495
23553	Pcnp	76302	16-:56017305-56029779
23554	MMT00078678	235106	9-:28816809-29005328
23555	BC026585	226527	1+:159388863-159419213
23556	Olfr571	259089	7-:110057382-110058351
23557	Ppp1r12c	232807	7-:4433115-4453624
23558	MMT00030937		15+:18409242-18409510
23559	AI450886	103731	11+:99846715-99855465
23560	Pms2	18861	5+:144697851-144705510

23561	Sox14	20669	9-:99775382-99776103
23562	4933434E20Rik	99650	3+:89856863-89866369
23563	MMT00004841		11-:17873743-17874841
23564	Vmn2r107	22312	17+:20493166-20512558
23565	3300001A09Rik	75697	9+:67607243-67607944
23566	Snx29	74478	16+:11405740-11755565
23567	Tom1l1	71943	11-:90506634-90548908
23568	Ugt8a	22239	3-:125570111-125641444
23569	Atp5s	68055	12+:70825948-70845645
23570	Sh3kbp1	58194	X+:156202000-156411097
23571	2410166I05Rik	76824	4-:134082021-134091175
23572	4631426J05Rik	77590	7-:139428793-139508792
23573	Vcp	269523	4-:42992835-43013191
23574	5133400G04Rik	71242	18-:35816349-35821809
23575	Tmem173	72512	18-:35893374-35900172
23576	Fabp9	21884	3-:10193625-10197222
23577	E330001B16Rik	546160	9-:109554133-109557245
23578	Cyp2j5	13109	4-:96295837-96330742
23579	Gm98	225908	19-:10282759-10315241
23580	BC059842	230676	4-:118064321-118070324
23581	3110070M22Rik	67304	13-:120276067-120277190
23582	Slc25a37	67712	14+:69859904-69861378
23583	Vps45	22365	3-:95803794-95862378
23584	Adamts5	23794	16-:85862668-85900529
23585	Mgmt	17314	7+:144086235-144143086
23586	MMT00041086		9-:109716749-109718373
23587	D430041D05Rik	241589	2-:103988939-104145251
23588	Anks1b	77531	10+:90411276-90434735
23589	Akap11	219181	14-:78892075-78916264
23590	Ptgds	19215	2-:25322221-25325546
23591	Dlg4	13385	11+:69832365-69858270
23592	ENSMUSG000000	100038614	16+:84972891-84973054
23593	Eif4e3	66892	6-:99575130-99616769
23594	Sertad1	55942	7+:28272064-28275115
23595	Gm550	225852	19+:6095867-6098245
23596	Cx3cr1	13051	9-:119960371-119977400
23597	Pcdha3	192163	18+:37105860-37347311
23598	Ctbp1	13016	5-:33590372-33617618
23599	Ndrg3	29812	2-:156753082-156817761
23600	Dpp3	75221	19-:4907228-4927063
23601	Piga	18700	X+:160863416-160871846
23602	Sec14l4	103655	11+:3931784-3948010
23603	Ercc4	50505	16+:13109828-13148825
23604	Ephb3	13845	16+:21204825-21223375
23605	MMT00077586		18-:90491386-90492213
23606	Cmtm8	70031	9-:114698462-114753270
23607	MMT00040265		12+:71081867-71083890
23608	Tas2r108	57253	6+:40443590-40444484
23609	Trim44	80985	2-:102144500-102241004
23610	Prkaa2	108079	4-:104706216-104782403
23611	Wwp2	66894	8+:109960282-110081112

23612	Es2el	27886	16-:17901857-17911430
23613	Jak2	16452	19+:29326944-29386472
23614	G630016D24Rik	619301	14+:52632591-52640170
23615	MMT00005149		6-:23105933-23106684
23616	Fgfr1op2	67529	6+:146526456-146547712
23617	Nme1	18102	11-:93820339-93822103
23618	Edn2	13615	4+:119834037-119839966
23619	Tmc1	13409	19-:20859144-21028692
23620	Pycr1	209027	11-:120500743-120505009
23621	1700029H14Rik	66501	8-:13550732-13562405
23622	Arhgap17	70497	7-:130422784-130513398
23623	2010305C02Rik	380712	11+:75281573-75284400
23624	Patl2	67578	2-:121945843-122011925
23625	Pkd2	18764	5+:104888655-104932705
23626	MMT00003764		14+:75117517-75118640
23627	Lypla1	18777	1+:4797994-4835096
23628	Ccdc87	399599	19+:4839481-4842047
23629	4930552P12Rik	77594	19-:55715079-55776694
23630	Tcea2	21400	2+:181415014-181422756
23631	MMT00039126		8+:99688153-99688772
23632	4930579C15Rik	67753	4-:94573960-94595536
23633	Prkcbp1	228880	2-:165609654-165610861
23634	Zfp39	22698	11-:58703280-58706557
23635	Htr2a	15558	14+:75041739-75106205
23636	Bst2	69550	8-:74058160-74061335
23637	Ptprf	19268	4-:117882405-117950051
23638	Ddr1	12305	17-:35818512-35840932
23639	Tlx2	21909	6-:83018584-83020137
23640	Bdh1	71911	16+:31422384-31458980
23641	Zfy1	22767	Y-:61649-96389
23642	Lypd4	232973	7-:25649636-25654767
23643	Olfr482	258728	7-:115238112-115239082
23644	Pou3f3	18993	1+:42753990-42755483
23645	1700041C02Rik	73332	4-:119006184-119056366
23646	Hsp90ab1	15516	17-:45704723-45710209
23647	Ptprs	19280	17-:56551843-56615816
23648	Pigy	66459	6-:57639344-57642071
23649	BC049635	277773	4-:42880876-42884992
23650	Rtkn2	170799	10+:67442349-67468442
23651	MMT00027295		18-:24212752-24262836
23652	MMT00082110		12+:4226348-4228521
23653	Zfp407	240476	18-:84378134-84381307
23654	Sox5	20678	6-:143781348-144158000
23655	MMT00006451		6+:3227561-3227772
23656	MMT00054819		16+:18218851-18219215
23657	Entpd7	93685	19+:43764274-43804989
23658	Ncoa3	17979	2+:165860383-165895946
23659	EG633250	633250	13+:62006047-62212827
23660	Dpyd	99586	3+:118702087-119134638
23661	RP23-272C1.10	100041488	11-:99471108-99476097
23662	Serpinb2	18788	1+:109412049-109422175

23663	Dab2ip	69601	2+:35477679-35586512
23664	Btg1	12226	10+:96079538-96082262
23665	Ccdc125	76041	13+:101439671-101466426
23666	Dcpp1	13184	17+:24017962-24019819
23667	Cdh17	12557	4+:11685407-11745021
23668	Gse1	382034	8+:123012901-123105248
23669	1110033M05Rik	68675	13+:77847953-78145733
23670	Zscan10	332221	17+:23737757-23747988
23671	Clec2h	94071	6+:128612426-128626718
23672	EG545989	545989	7+:112523611-112526491
23673	Alg13	67574	X+:140780154-140808345
23674	Exoc3l2	74463	7+:20074404-20082109
23675	Gm266	212539	12-:112723378-112723981
23676	Ms4a13	73466	19-:11243908-11271238
23677	Aof2	99982	4-:136106455-136158254
23678	1700110M21Rik	76627	14-:101597037-101598618
23679	Ccl20	20297	1+:83113356-83115741
23680	5730419I09Rik	74741	6-:142959447-143048638
23681	Whrn	73750	4-:63075946-63156610
23682	Trim8	93679	19+:46576788-46590839
23683	Dnahc7c	100101919	1+:46706054-46864976
23684	AI987944	233168	7-:48628294-48648697
23685	Tbxas1	21391	6+:38868973-39034559
23686	Il17rc	171095	6+:113421500-113433132
23687	MMT00075954		3+:35762750-35763987
23688	Ube2e2	218793	14-:19406090-19726141
23689	Ptcd1	71799	5-:145908400-145927891
23690	Mug1	17836	6+:121788628-121839074
23691	Sh3d19	27059	3+:85844766-85932398
23692	Safb2	224902	17-:56702368-56723946
23693	A530016L24Rik	319942	12+:113733198-113736602
23694	Plcg1	18803	2+:160557517-160558603
23695	1700081L11Rik	76719	11-:104229987-104287786
23696	Galr2	14428	11+:116142799-116144973
23697	Rrp1b	72462	17+:32173045-32197674
23698	Trpc5	22067	X-:140816214-141122723

Table T3: Probes selected for single tissue analysis

Probe_ID	Gene_Symbol	Entrez_ID	Chromosomal_Location
10	NAN		
83	NAN		
98	NAN		
115	NAN		
126	0610012D14Rik	68352	7+:51720804-51723121
127	Clic1	114584	17+:35187187-35195664
129	Podn	242608	4-:107687402-107704561
131	Irx3	16373	8-:94322425-94325139
133	Olfr313	258529	11+:58630511-58631437
134	Peli1	67245	11+:21035471-21050325
137	Sorcs2	81840	5-:36359879-36740757
139	Mpst	246221	15+:78240468-78244435
141	Jag1	16449	2-:136908713-136942256
143	Gvin1	74558	7-:113043647-113047500
144	Krt12	268482	11-:99276979-99283570
145	Tmem121	69195	12+:114425891-114427759
146	Rlbp1l2	215890	10-:33232090-33344002
147	Uox	22262	3+:146260130-146294369
150	Rpp38	227522	2-:3246216-3249873
153	2010011I20Rik	67017	2+:172171136-172179397
160	MMT00078580		X-:147359162-147360639
165	Scn5a	20271	9-:119394347-119471790
171	MMT00025388		18-:85885324-85886049
173	Vps39	269338	2-:120143770-120178723
176	Sesn1	140742	10+:41530366-41628229
177	Appl2	216190	10-:83062780-83111409
180	Rbm39	170791	2-:155973371-156005876
183	Bmp4	12159	14-:47003534-47006863
184	Sorbs1	20411	19-:40369260-40588240
185	Ap2b1	71770	11+:83116275-83216514
187	Wdr62	233064	7-:31025159-31034896
189	MMT00059108		X+:47726478-47727129
191	Fbp2	14120	13-:62938243-62959676
192	Eif3d	55944	15-:77789430-77801190
193	C630028N24Rik	235386	9+:54768279-54795128
199	Gabrb3	14402	7+:64846561-65080216
202	Naif1	71254	2+:32307958-32312474
204	EG432649	432649	12+:36808292-36817625
205	Cnksr1	194231	4-:133783960-133794223
212	1700026J04Rik	71864	13-:35001832-35011798
213	Rgs16	19734	1+:155587478-155592599
214	Lrp1	16971	10-:126975244-127058182
217	Ubxd2	67812	1+:130140470-130175951
218	4930511H11Rik	78016	1+:54386983-54425580

219	MMT00058440		12-:65977816-65978615
221	1700009P17Rik	75472	1+:173051791-173057081
223	Trp53inp2	68728	2+:155207575-155215575
225	MMT00073704	635169	4-:127605246-127628693
227	B4galt1	14595	4-:40751636-40801009
230	MMT00000744		3+:65417429-65419248
232	Pcdh9	211712	14-:94268644-94290147
236	Bbs9	319845	9+:22280165-22483508
237	Cyp2c37	13096	19+:40066929-40086733
242	4732471D19Rik	319719	13+:54638439-54653391
245	Sqrdl	59010	2+:122610696-122635072
246	MMT00053742		13-:112672463-112673919
247	Clns1a	12729	7+:104845200-104867388
250	Tubb2a-ps2	627110	12-:11888133-11889704
252	C730027P07Rik	245050	9+:121874316-121887805
253	Slc5a8	216225	10+:88381107-88382303
255	Twf2	23999	9+:106105573-106117694
256	Zbtb7a	16969	10+:80600778-80611198
260	Serpinb3d	394252	1-:108974771-108980057
263	Akr1b3	11677	6-:34253935-34267421
264	Dcakd	68087	11-:102855362-102858862
265	Taf9	108143	13+:101420926-101436363
266	Nr2f2	11819	7-:77498327-77505249
268	Sys1	66460	2+:164286495-164291012
269	Tuba1b	22143	15-:98762050-98763635
272	Tgm1	21816	14-:56318872-56332312
274	Trit1	66966	4+:122693839-122732177
276	Olfr1019	259017	2-:85681013-85681946
280	Snai2	20583	16+:14706026-14709467
281	MMT00063621		X-:53531300-53535766
283	Gmcl1l	71847	X+:30732653-30734582
284	Osr2	107587	15+:35225866-35233059
286	MMT00070509	100037278	8+:74127597-74132047
288	Col6a2	12834	10-:76058506-76086059
290	B230118H07Rik	68170	2-:101402262-101469143
291	Sbp	20234	17+:24078962-24082567
294	Ehhadh	74147	16-:21761365-21763011
295	Pdlim1	54132	19-:40296730-40346025
299	MMT00061667		1-:53641301-53693021
300	Kcnk13	217826	12+:101204117-101300884
301	AU014645	433702	4+:46151508-46185276
305	Cma2	545055	14+:56569816-56592866
308	Meig1	104362	2-:3326318-3336313
309	Slc25a11	67863	11-:70457826-70460517
314	Samd12	320679	15-:53489903-53551239
316	4930471G03Rik	74966	18+:36265806-36269607

317	Ceacam13	69785	7+:18595246-18604569
318	9130208D14Rik	77700	7-:112972738-112980029
322	Abcb5	77706	12-:120106320-120204895
326	4931409K22Rik	231045	5-:24049250-24061227
327	H2-Ea	14968	17-:34479878-34481590
329	Lmod1	93689	1+:137221407-137263405
337	Rasgef1b	320292	5-:99646445-99681873
339	Hmgcs1	208715	13_random-:114708-127798
342	Nrbf2	641340	10-:66729439-66748031
343	1700123I01Rik	76663	19+:6184400-6224217
344	MMT00036566		1+:29794139-29881997
346	Olfr1484	258288	19+:13659795-13660741
352	4732415M23Rik	320869	8+:125736748-125745944
354	MMT00007996		6-:67583076-67584137
357	Ftsj3	56095	11-:106110457-106117112
359	Ciita	12265	16+:10488371-10527656
360	Igh-VJ558	16061	12-:115984439-115984952
361	Fign	60344	2-:63815896-63936045
363	Adora2a	11540	10+:74787665-74797535
364	0610040B09Rik	75395	11-:96152349-96154868
365	1700019N12Rik	67077	19-:6996915-6999870
366	Adcy6	11512	15-:98420421-98435257
371	MMT00058786		8-:109560987-109562129
372	Txndc1	72736	12+:71554101-71569008
375	MMT00077729		19+:23680345-23734472
379	Nmnat1	66454	4-:148842745-148848706
380	Fry	320365	5+:151062843-151300326
381	Rbmxrt	19656	8-:81029169-81032740
385	Cog3	338337	14-:76102158-76154245
387	Rgs10	67865	7-:135517138-135561686
391	4933416I08Rik	71159	X+:50989700-50994509
392	Sec22b	20333	3+:97705203-97725429
393	Ucp1	22227	8+:85814481-85821868
394	Ccdc85a	216613	11-:28288819-28334158
397	Ak7	78801	12+:106944326-107020654
398	BC025446	223631	15+:75047025-75053089
401	MMT00061194		4+:33051752-33052247
402	Timp3	21859	10+:85763281-85812250
403	Leprotl1	68192	8-:35200370-35209862
405	Emr4	52614	17+:55889297-55992735
407	Serpina6	12401	12-:104884842-104895281
409	Lxn	17035	3-:67261918-67267735
410	MMT00048420		17+:85248800-85273194
413	Acy1	109652	9-:106335326-106340567
415	Ypel2	77864	11+:86749926-86750408
416	Xlr5b	627081	X+:70397218-70401916

419	Foxk2	68837	11+:121121570-121171208
420	Ear11	93726	14-:51875113-51875581
423	Klhl5	71778	5+:65499025-65559424
425	MMT00064181		X-:53144982-53147353
426	Mobkl3	19070	1+:55188133-55210100
427	Smg6	103677	11+:74802858-74805687
433	Pla2g12b	69836	10+:58866410-58884481
434	Psmd1	70247	1+:87961212-88035865
437	Ndrg1	17988	15-:66760883-66801201
439	Ugt2b35	243085	5+:87429916-87437385
443	Xpo5	72322	17+:46339694-46380544
445	2610029I01Rik	77032	4-:21684530-21694363
446	MMT00020922		
448	Sh3glb2	227700	2-:30200330-30214751
449	1700019P21Rik	75546	1-:140772851-140773554
451	Mllt11	56772	3-:95023058-95032166
458	Olfr876	258883	9+:37611497-37612429
459	Tcfap2a	21418	13-:40812269-40829150
461	Zfp664	269704	5+:125343059-125369006
468	2610020C07Rik	69918	16+:11203489-11220817
470	MMT00076063		7-:14168976-14170985
473	MMT00063585		X-:53407721-53409696
478	Sfrs9	108014	5+:115777225-115783086
479	Best1	24115	19-:10059660-10067811
482	Rnf43	207742	11+:87477636-87549040
484	Atp4a	11944	7+:31497370-31510203
486	Josd2	66124	7+:51723425-51727021
487	Slc6a15	103098	10+:102830476-102882011
488	Igh-VJ558	16061	12-:116396590-116397077
491	MMT00030239		2+:11341818-11343062
494	Tspan7	21912	X+:10062293-10173728
495	Ddx10	77591	9-:52906745-53056196
496	Fgf1	14164	18-:38999700-39078312
499	2310057J16Rik	69697	8+:3587811-3602281
502	Iars	105148	13+:49777445-49829628
508	MMT00028550		2+:83985036-83985869
510	Neu1	18010	17+:35068318-35071775
511	Itpa	16434	2+:130493550-130507349
514	Abca6	76184	11-:110038137-110113042
523	1700040L02Rik	73287	10-:67893701-67996666
524	Hsd3b2	15493	3-:98515030-98528478
525	Lyl1	17095	8+:87225442-87228591
530	Mest	17294	6+:30688062-30698457
533	MMT00040762		2-:150222864-150225421
534	Plrg1	53317	3+:82860692-82876092
537	Dmbt1	12945	7+:138175590-138265140

538	Mpp2	50997	11-:101918332-101949816
540	Chd9	109151	8+:93456138-93507447
544	Il16	16170	7-:90791575-90803906
545	4930524O08Rik	74700	9+:89721353-89758866
546	Gltscr1	243842	7-:16556611-16584843
547	Mknk2	17347	10-:80128787-80134754
548	Gpr109a	80885	5-:124313585-124315505
550	Hars2	70791	18+:36942933-36952207
552	MMT00013481		5-:94124018-94126763
553	Plekhb2	226971	1+:34906843-34936413
556	Spint2	20733	7-:30041350-30066997
558	Crebl1	12915	17+:34784179-34791641
563	D4Bwg0951e	52829	4+:80557282-80600207
566	Ccdc132	73288	6+:3505430-3553313
568	A330049M08Rik	230822	4-:134925824-134954136
569	1700125D06Rik	68233	4+:129168442-129169450
572	MMT00014316		4-:84569446-84572305
574	Zfp57	22715	17+:37139982-37147580
575	Edn1	13614	13+:42397233-42403356
577	Rrp12	107094	19-:41937331-41970690
583	Taf15	70439	11+:83286586-83320246
584	Tmem207	224058	16-:26504592-26526809
585	Tas2r119	57254	15+:32107043-32108047
589	Epb4.1l4b	54357	4-:57074610-57156520
591	Slco6c1	74441	1-:98955615-99024881
602	9630041N07Rik	214779	11-:50645537-50652056
604	Ppp1r15b	108954	1+:135027863-135036364
605	Unc119b	106840	5-:115572577-115584936
607	Me1	17436	9-:86474982-86589830
609	MMT00060868		13-:63068834-63070096
611	Tac2	21334	10+:127161533-127168823
612	Ccr1	12768	9-:123878257-123879324
614	C8g	69379	2-:25354166-25357244
617	MMT00077405		15+:62812180-62813611
620	Prpf31	68988	7+:3581614-3592829
621	Cyp2b13	13089	7+:26846513-26881215
622	Serping1	12258	2-:84605543-84615561
623	Lmf1	76483	17+:25716148-25799668
624	4931406I20Rik	66743	4-:116944531-117048062
626	D19Ertd386e	52013	19+:42636995-42665857
627	Eif1a	13664	18+:46767353-46767788
630	C1s	50908	6-:124480362-124492299
633	Ap1b1	11764	11+:4886971-4942732
635	MMT00008587		2+:147493729-147494129
640	MMT00065211		14+:14734306-14735766
649	Pop7	74097	5-:137942666-137943657

652	Veph1	72789	3-:65860558-65940360
654	Rassf5	54354	1-:133073356-133141736
658	Vmn2r-ps88	209786	8+:3093456-3104954
659	Bace1	23821	9+:45646681-45670302
661	Siglec1	20612	2-:130894478-130912501
662	Mid1ip1	68041	X+:10294715-10296811
663	Muc19	239611	15+:91702790-91736981
665	Slc26a1	231583	5-:109098950-109110090
668	Olfr802	258934	10-:129118855-129119793
672	Calcrl	54598	2-:84170790-84217144
673	Tchh	99681	3+:93246251-93253000
676	Ms4a4d	66607	19+:11611290-11632952
677	Ace2	70008	X+:160578419-160626353
678	Ncaph	215387	2-:126929566-126959641
679	Lrpprc	72416	17-:85104590-85189855
680	Loxl4	67573	19-:42669824-42683021
681	Hcn1	15165	13+:118391506-118765609
683	Olfr1450	258368	19+:13028080-13029056
684	1700102P08Rik	112418	9+:108295164-108300094
688	MMT00020896		
689	2010004A03Rik	76527	8-:113264997-113329776
690	Lrrc9	78257	12+:73600566-73611545
692	Pelo	105083	13-:115878571-115881033
696	MMT00037949		16+:38927463-38927965
700	4930529C04Rik	619677	3+:90875583-90880395
701	Polr3g	67486	13-:81812841-81849966
703	Spryd4	66701	10-:127646969-127648855
707	Bdnf	12064	2+:109532875-109564409
708	Ophn1	94190	X-:95753590-96086120
709	Pparg	19016	6+:115372090-115440418
714	Igk-V8	384422	6+:68071041-68387930
715	Padi3	18601	4-:140341283-140366563
716	Dbpht1	20749	15+:64648686-64649468
719	Usp2	53376	9+:43897214-43901997
725	BC005561	243171	5+:104946438-104951890
726	Phkb	102093	8+:88366929-88420438
727	Ywhae	22627	11+:75546501-75579305
737	MMT00023716		
738	Rbp4	19662	19-:38191112-38199804
739	Ric8b	237422	10+:84432607-84479185
740	Txnrd3	232223	6+:89593988-89625522
749	Mex3c	240396	18+:73733247-73752224
755	Ppp1r13l	333654	7+:19946374-19963890
756	Pou1f1	18736	16+:65520846-65535866
760	Akr1c19	432720	13+:4227945-4247604
761	Amigo1	229715	3+:107989267-107994694

763	4933406E20Rik	74443	9-:108481213-108499902
768	Samd1	666704	8+:86522443-86524316
769	5430437P03Rik	68251	8+:73920759-73928517
771	Zbtb45	232879	7-:13591013-13593679
772	Hnrnpr	74326	4+:135866911-135896587
778	Galnt3	14425	2-:65921491-65962686
784	Actg1	11465	11-:120208395-120209809
786	Macrod1	107227	19+:7131532-7272547
790	MMT00026414	83701	5-:137737059-137748395
791	Il18r1	16182	1+:40522850-40557678
793	Morc3	338467	16+:93832534-93876317
794	Mrps14	64659	1+:162125378-162130567
797	Gpi1	14751	7-:34987176-35015281
801	Olfr295	258850	7+:93733786-93734714
803	Lhb	16866	7+:52676322-52677147
804	2210407C18Rik	78354	11-:58421710-58426972
805	Reep1	52250	6+:71657831-71760532
806	Ly96	17087	1+:16678536-16699686
808	Ctsc	13032	7+:95426644-95458806
812	En2	13799	5+:28493019-28498710
814	Gpr64	237175	X+:156828597-156935996
817	C130039O16Rik	238317	12-:85490239-85500005
820	3200002M19Rik	75430	7+:109044930-109048057
822	Ddah1	69219	3+:145421689-145557242
824	Esco1	77805	18-:10566600-10594053
826	Fezf1	73191	6-:23195050-23198148
827	Flcn	216805	11-:59605254-59623275
828	Flnc	68794	6+:29409404-29411881
830	Socs1	12703	16-:10783903-10785602
838	MMT00011949		19-:12219977-12241545
845	Myo5a	17918	9+:74919406-75067280
848	MMT00008599		8+:53859486-53860062
849	Pxmp4	59038	2-:154413323-154429398
853	Crct1	74175	3-:92818128-92819608
857	Sfrs12	218543	13-:104533165-104564597
860	Psmb10	19171	8-:108459663-108462005
863	Dnajc14	74330	10+:128242731-128254559
864	MMT00031865	329122	1+:37992371-38006685
865	Npc1	18145	18-:12348370-12394867
866	Zfp369	170936	13+:65380235-65398890
870	Rint1	72772	5+:23293529-23327653
872	MMT00004441		4+:76573197-76573933
873	Slc22a3	20519	17-:12612841-12700570
874	Aldh1a7	26358	19-:20767442-20802046
877	MMT00015473		19+:43618672-43619815
880	Atp6v0a2	21871	5+:125079736-125202515

884	Kif26b	269152	1+:180751613-180862984
885	2210010L05Rik	98682	1-:52714300-52766004
894	Ddit4	74747	10-:59412422-59414518
897	Mut	17850	17+:41073992-41098598
902	Slc30a1	22782	1+:193730738-193733694
904	MMT00003579		14-:88179628-88179937
905	Fyco1	17281	9-:123698627-123761010
908	Lypd2	68311	15-:74562676-74564745
910	Sned1	208777	1+:95192288-95194148
915	9130206N08Rik	103406	10+:80702678-80713479
917	Kal1	14038	11+:83522607-83524842
923	MMT00033681		14-:120043166-120048850
929	Dusp4	319520	8+:35870424-35885009
930	Cd55	13136	1-:132336790-132359261
931	Ccdc147	381229	19+:48011400-48129383
932	Chad	12643	11+:94426362-94430433
937	Pi4ka	224020	16-:17280441-17405919
938	Sdcbp	53378	4+:6292867-6322797
939	Daxx	13163	17+:34047373-34052463
940	Pramel3	83565	X+:131836850-131847175
942	Kcna5	16493	6-:126482570-126485455
949	Plekha6	240753	1+:135155585-135200013
952	1810008K16Rik	67366	5-:109206253-109224382
954	Adam25	23793	8+:41837561-41841529
958	Ppp2r5e	26932	12-:76550450-76694320
959	Cckar	12425	5-:54089726-54099341
961	Rwdd4a	192174	8+:48619008-48638304
962	Klhl10	66720	11+:100303237-100318336
963	Lrrfip2	71268	9+:111063805-111128172
966	Wif1	24117	10+:120471285-120537698
967	Matr3	17184	18+:35747218-35751870
969	MMT00054728		10+:78006018-78006876
972	Sri	109552	5+:8046077-8067854
981	1110015M06Rik	68511	12-:29209539-29215352
982	Ccl7	20306	11+:81859233-81861023
983	MMT00078030		13-:91400536-91402025
984	Derl3	70377	10+:75356142-75358685
985	Hsd3b6	15497	3-:98609427-98615031
988	Olfr491	258731	7+:115460409-115461342
989	Phyhd1	227696	2+:30121720-30137667
992	8430410A17Rik	232210	6+:87863943-87886618
996	Ppbp	57349	5+:91197543-91199086
1000	2900024O10Rik	72852	13+:81850045-81890207
1006	2600011E07Rik	72125	14+:60997188-60999841
1008	MMT00069490		X+:71732110-71732758
1009	Rps2	16898	17+:24857053-24858868

1014	Rtcd1	66368	3-:116191883-116211028
1017	Dync2li1	213575	17+:85025814-85054927
1019	Slc26a3	13487	12+:32123165-32158584
1020	Olfr195	259000	16+:59148677-59149602
1021	Rhpn2	52428	7+:36119332-36177300
1023	Ccdc64b	212733	17+:23798726-23805584
1026	Ftsj2	68017	5-:140803627-140807852
1027	Accs	329470	2-:93675574-93690099
1031	Uros	22276	7-:140878038-140900716
1036	Dusp19	68082	2+:80457201-80471351
1039	Zfp207	22680	11+:80196847-80209639
1040	Dnhd1	77505	7+:112826403-112870312
1041	3110049J23Rik	67307	10+:62487259-62521560
1050	MMT00044489		4-:59707420-59707804
1051	MMT00065456	100041868	1+:100272681-100356563
1052	Cog4	102339	8+:113370946-113406124
1055	Gm525	217071	11+:88934958-88954377
1058	Olfr868	258552	9+:19905204-19906133
1059	P2rx6	18440	16+:17562092-17571358
1061	Krt20	66809	11-:99289716-99299467
1065	Hmgcr	15357	13-:97418921-97440804
1068	1700129I15Rik	78478	X-:132253204-132255213
1073	Ppp1r7	66385	1+:95240221-95264195
1074	Acsm3	20216	7+:126904456-126928403
1076	Megf11	214058	9+:64345146-64554489
1079	Hr	15460	14+:70954578-70972201
1083	Ddx3y	26900	Y-:597157-623024
1084	Pros1	19128	16+:62854153-62929179
1085	Col5a2	12832	1-:45432930-45559759
1086	2610205E22Rik	66617	2+:30663460-30679260
1088	Lef1	16842	3+:130813250-130927276
1089	1700010D01Rik	76386	X-:92927928-92928600
1090	5730409G15Rik	70511	16-:5244523-5256050
1091	Acsl5	433256	19+:55328144-55371117
1092	MMT00024026	554292	15+:100183640-100214386
1093	MMT00012906		3-:78695787-78696346
1097	Dctn6	22428	8-:35153477-35171497
1099	Ap1m1	11767	8+:74764030-74781278
1101	Sec11a	56529	7-:88060264-88092436
1105	Crls1	66586	2+:132672401-132692502
1108	MMT00067207		14+:56868857-56870107
1109	Snai1	20613	2+:167363775-167368309
1112	Ccdc56	52469	11-:101139283-101140262
1114	Cdca7l	217946	12+:119082333-119117178
1115	Rbbp7	245688	X+:159206779-159216559
1120	Rbpms2	71973	9+:65477454-65508333

1122	Pax3	18505	1-:78099278-78193621
1126	Slc25a14	20523	X+:45976754-46015469
1127	Pomt2	217734	12-:88488120-88488849
1128	Amtn	71421	5+:88805132-88814941
1129	Gsta4	14860	9+:78039838-78057155
1130	Ly75	17076	2-:60130160-60221315
1134	Vmn2r19	232358	6+:123259676-123280222
1135	Akap12	83397	10-:5987069-6080163
1136	Kdelr3	105785	15+:79346897-79358168
1137	Mlxip	208104	5+:123893038-123903952
1140	Rmnd5a	68477	6-:71338631-71390133
1141	Oxr1	170719	15+:41621060-41692591
1144	MMT00060803		11+:32084994-32085702
1145	Arhgap6	11856	X+:165233904-165742367
1148	Pak1	18479	7+:104991479-105059842
1156	Rbj	217378	12+:4082556-4110603
1159	Tmem104	320534	11+:115048841-115108336
1162	Cpsf4	54188	5+:145928113-145942911
1163	Olfr52	18352	2-:86021307-86022266
1164	A930011O12Rik	268755	14+:65209109-65212802
1167	Atg2a	329015	19+:6241637-6262304
1170	Faim3	69169	1+:132762337-132777421
1177	EG545391	545391	1+:180034087-180086933
1180	Clec4a3	73149	6+:122902683-122919897
1183	MMT00009390		
1185	Atxn2	20239	5+:122161617-122264717
1187	MMT00075773		19+:24930589-24930857
1194	Per3	18628	4-:150378820-150418698
1200	Grm3	108069	5-:9485239-9589959
1201	Ptger3	19218	3+:157229863-157307720
1203	Enah	13800	1-:183835645-183949792
1214	Trip13	69716	13-:74049907-74075187
1219	EG665503	665503	13-:62629440-62630551
1223	Slc30a9	109108	5+:67698199-67747022
1227	4933421I07Rik	71162	7-:49700831-49703450
1230	MMT00057236	630663	13-:13413093-13415645
1232	Abhd1	57742	5+:31252444-31257456
1233	Sidt1	320007	16-:44240015-44333081
1235	Cpne9	211232	6+:113232298-113254819
1236	Ccdc113	244608	8+:98060363-98082788
1238	Habp2	226243	19+:56362427-56394578
1239	Cerkl	228094	2-:79208838-79269162
1241	Meox1	17285	11-:101738837-101755623
1243	Phf3	213109	1-:30859185-30930877
1244	Tmem150	232086	6+:72305486-72309756
1246	EG434121	434121	7-:14495025-14574937

1248	Usp24	329908	4+:106032818-106046647
1250	Nol6	230082	4-:41061460-41071321
1252	Tacc2	57752	7+:137765094-137815172
1253	Scel	64929	14+:103912558-104012389
1254	Lum	17022	10+:97028463-97035337
1255	Grhpr	76238	4+:44994282-45003567
1256	Baiap2l2	207495	15-:79088628-79115110
1257	Slc25a45	107375	19+:5877841-5885763
1258	Hba-a1	15122	11+:32183673-32184492
1263	Folh1	53320	7-:93867906-93924342
1270	1700029I01Rik	70005	4+:146506142-146554953
1271	2810403A07Rik	74200	3+:88489739-88516844
1272	1110008P14Rik	73737	2-:32234623-32237433
1276	Col4a1	12826	8-:11198421-11312621
1279	Ctps2	55936	X+:159340115-159469249
1280	Mesdc2	67943	7+:91040533-91047690
1281	Alkbh3	69113	2-:93820708-93850369
1284	Serpina3h	546546	12+:105452765-105492619
1286	Rad51c	114714	11-:87192888-87217923
1287	Acss3	380660	10-:106441719-106560639
1290	Zscan12	22758	13+:21455153-21461693
1293	Safb	224903	17+:56724245-56745715
1294	MMT00016640		7+:12194645-12196115
1296	5330401P04Rik	227094	1+:52694208-52708763
1297	Phox2a	11859	7+:108967003-108971238
1305	4930485B16Rik	380654	10-:92507829-92510823
1307	Tmem116	77462	5+:121913205-121945429
1310	Cabc1	67426	1-:182096745-182123597
1311	Mpp4	227157	1-:59177778-59215512
1312	MMT00051556		X-:137356725-137392567
1315	Tmem16j	71345	7-:148287124-148303661
1317	Fcgrt	14132	7-:52348433-52358462
1321	AW548124	106522	17+:83615139-83624400
1322	Cep290	216274	10+:100005050-100036288
1323	Orc2l	18393	1-:58520039-58561763
1324	Rfc5	72151	5-:117828846-117839045
1325	Pigu	228812	2-:155103980-155127101
1326	Tox	252838	4-:6615494-6917657
1327	Pitpnc1	71795	11-:107073419-107332013
1329	5730494N06Rik	70612	2-:132065229-132073518
1330	Fgf21	56636	7-:52869390-52870676
1336	MMT00069537		14-:61214340-61215729
1339	MMT00077775		19-:26308024-26629706
1340	Tap2	21355	17+:34342239-34353037
1342	Pik3ca	18706	3+:32335127-32361752
1344	Marcks	17118	10-:36854626-36858720

1351	Whsc1l1	234135	8+:26712042-26825166
1353	Plcg2	234779	8+:120022087-120159043
1354	Lsmd1	78304	11+:69209260-69210703
1355	MMT00047341		1-:28858362-28859394
1356	MMT00045517		Y-:1317414-1317910
1357	Pabpn1	54196	14+:55512992-55516891
1358	Pappa2	23850	1-:160644843-160887569
1359	4931406C07Rik	70984	9-:15087782-15106000
1361	2010001M06Rik	69812	5-:135487979-135489124
1362	Avpr1a	54140	10+:121885554-121890509
1363	BC021767	545551	3-:94464511-94474619
1364	Prl8a9	67310	13-:27650021-27656415
1368	Pctp	18559	11-:89845905-89864179
1371	1200009I06Rik	74190	12+:112659771-112669393
1374	Mdn1	100019	4+:32771613-32780612
1375	Strap	20901	6+:137683646-137699610
1376	Mdm2	17246	10-:117127265-117146840
1379	A930041C12Rik	78443	5-:108059270-108062871
1380	Sycp3	20962	10+:87922331-87935980
1383	Bhlhb9	70237	X+:132420530-132425617
1386	Ppid	67738	3+:79395353-79407570
1390	AU015836	385493	X+:91214066-91214923
1392	Gm1040	433864	5+:29761265-29778014
1393	Klhl2	77113	8-:67218481-67373628
1394	MMT00051501		17+:8005628-8006677
1396	Rassf7	66985	7+:148401731-148404555
1397	2900009I07Rik	68034	19-:24550939-24551933
1398	MMT00060205		14+:31215187-31215917
1401	Klk8	259277	7+:51052946-51059192
1405	MMT00078938		7+:110292103-110293045
1406	Clic3	69454	2+:25312359-25314298
1410	Bicc1	83675	10-:70387849-70622371
1411	Tada3l	101206	6-:113316656-113327514
1412	Phf17	269424	3+:41367382-41420773
1415	Timm9	30056	12-:72224282-72237621
1416	6720460F02Rik	109212	11+:71856011-71860873
1419	Olfr1121	258345	2+:87211690-87212635
1423	Spdya	70891	17+:71876198-71938872
1424	Dapk1	69635	13+:60703790-60864543
1427	Ahrr	11624	13-:74348565-74429757
1429	Strn4	97387	7+:17401704-17426284
1433	Deb1	26901	9+:121619503-121622037
1435	Ptges3	56351	10+:127505742-127514045
1436	Nfu1	56748	6+:86959897-86978409
1438	Dclre1b	140917	3-:103606679-103612107
1441	MMT00001620		X+:9588913-9590834

1443	Znhit2	29805	19+:6061196-6062470
1444	C1r	50909	6+:124469358-124473458
1445	Ssbp2	66970	13+:91600701-91840840
1446	Eno3	13808	11+:70470705-70476012
1447	Naaa	67111	5-:92687286-92707207
1448	Zwilch	68014	9-:63985482-64020715
1451	2010300C02Rik	72097	1-:37668520-37694874
1452	Gm1027	381538	4-:106353243-106382321
1453	Gfod2	70575	8-:108240012-108282507
1455	Smek1	68734	12-:102277619-102321144
1457	Efr3b	668212	12-:3967102-4038729
1458	Cpsf2	51786	12+:103214197-103241820
1462	Rpusd4	71989	9+:35075446-35083543
1464	Taf13	99730	3+:108374616-108384986
1466	Ubap2l	74383	3-:89803505-89856403
1470	Kcns3	238076	12-:11098027-11099502
1471	Igfbp7	29817	5-:77778270-77837070
1472	MMT00078790		1+:33157660-33159540
1473	MMT00007831		8+:40369078-40370255
1476	2610020H08Rik	434234	7+:126937638-126992452
1480	Plekhh3	217198	11-:101023995-101032616
1482	Nme5	75533	18-:34722376-34738340
1485	Plek	56193	11-:16871452-16908705
1487	MMT00019522		6+:147220566-147221162
1490	Mapk1	26413	16+:16983659-17039133
1493	MMT00047335		12-:10731050-10731395
1494	Cyp2c69	100043108	19-:39917150-39961217
1495	Zfp445	235682	9-:122758064-122771246
1496	MMT00061267		2-:84997574-84998454
1497	Dnaja4	58233	9+:54547190-54562571
1500	Leprel1	210530	16-:25959375-25997389
1501	Rnf145	74315	11+:44332196-44379023
1502	Prelid2	77619	18-:42035254-42072135
1503	Lingo1	235402	9-:56466864-56483435
1504	Xpo7	65246	14-:71064653-71166341
1505	Dusp9	75590	X+:70884779-70888449
1507	Tcof1	21453	18-:60974196-61008562
1508	Tlr4	21898	4+:66488844-66503828
1510	A530058N18Rik	320846	2+:113839131-113858021
1513	Gzmb	14939	14-:56877696-56881097
1515	Med20	56771	17+:47748579-47761199
1518	Amigo2	105827	15-:97075410-97076880
1520	MMT00069758		6+:4308509-4309482
1522	Nfkbib	18036	7-:29544062-29551512
1525	MMT00024951		1-:121138548-121139323
1530	Cideb	12684	14-:56372896-56377295

1531	Uqcrc2	67003	7+:127778720-127802784
1533	BC013712	230787	4-:132337754-132352268
1536	Hopx	74318	5-:77470227-77524282
1537	1700052K11Rik	73431	11-:105041137-105042747
1543	Acta2	11475	19-:34316006-34329805
1544	MMT00046046		17-:24029863-24030559
1547	Eid1	58521	2+:125498830-125500519
1549	Gmfg	63986	7+:29226451-29231560
1550	Chst12	59031	5+:140999493-141001191
1554	Rph3al	380714	11-:75646902-75667983
1561	Aurka	20878	2-:172181690-172196042
1562	Btg2	12227	1-:135971905-135975696
1563	Galp	232836	7+:6154402-6165516
1566	Kctd15	233107	7-:35424037-35437860
1568	Frmd4b	232288	6-:97236861-97567576
1569	Tmco4	77056	4+:138528806-138615079
1570	Olfr522	258954	7-:147347909-147348847
1572	Saa3	20210	7-:53967496-53970470
1579	Clca1	12722	3-:144392881-144423733
1580	Mrs2	380836	13-:25084440-25112248
1582	Srr	27364	11-:74721776-74722708
1585	Mif4gd	69674	11-:115469234-115474236
1587	MMT00044543		4+:52913468-52915778
1592	Amy1	11722	3-:113258871-113277680
1593	D3Ertd300e	56790	3+:54497026-54520758
1594	Mrpl24	67707	3+:87723465-87727586
1600	Csrp1	13007	1+:137635972-137647923
1602	Mina	67014	16+:59471632-59492113
1603	Lrwd1	71735	5-:136598936-136611871
1604	Ssbp4	76900	8-:73121389-73132172
1606	Srp14	20813	2-:118301586-118305357
1607	Memo1	76890	17-:74600045-74694323
1612	Clec1b	56760	6+:129347603-129355294
1613	Trim26	22670	17+:36974128-36996321
1614	4933402P03Rik	108803	11-:69630066-69631970
1615	Timm10	30059	2+:84667199-84670368
1618	Mfap1a	67532	2-:121319443-121332385
1619	Fbxw17	109082	13+:50513253-50527925
1620	Htra1	56213	7+:138079748-138129167
1624	Dzip3	224170	16-:48925921-48994254
1625	AW146154	101835	7+:48745783-48746625
1627	2310030N02Rik	76947	4-:10978197-11003334
1629	Gcsh	68133	8-:119505871-119517351
1631	Aldob	230163	4-:49549296-49562357
1635	8430427H17Rik	329540	2-:153233197-153262098
1637	MMT00071316		1-:71760373-71761572

1641	Hoxc11	109663	15+:102784937-102787130
1646	Slc15a4	100561	5-:128076036-128097762
1648	Gnpnat1	54342	14-:45996095-46008471
1649	Pcolce	18542	5-:138046337-138052587
1650	Ctdsp2	52468	10+:126432666-126437025
1653	Anxa5	11747	3-:36347847-36374620
1654	Dhx58	80861	11-:100564073-100565585
1655	Mgrn1	17237	16+:4886343-4938284
1656	Ahr	11622	12-:36182693-36219576
1660	Zfp33b	69623	5+:105012010-105013638
1661	Duoxa1	213696	2-:122129288-122139429
1663	Fkbp4	14228	6-:128380127-128388545
1667	Tbr1	21375	2+:61642669-61652168
1668	Lhx1	16869	11-:84332880-84339036
1672	Mlh1	17350	9-:111130758-111174066
1676	Tmem170	66817	8-:114388797-114400575
1677	Fabp1	14080	6+:71149881-71155016
1678	Tcf15	21407	2+:151969307-151974830
1679	MMT00009278		3+:95786885-95787446
1688	Camp	12796	9-:109749900-109751963
1690	Nlrp5	23968	7+:24189120-24226935
1693	Ufsp1	70240	5+:137735896-137736892
1696	V1re9	252904	7-:11165193-11166151
1697	Sf4	70616	8+:72566711-72596244
1700	Ccdc64	75665	5-:116098188-116181568
1702	Galc	14420	12-:99442005-99497617
1705	Gltpd1	79554	4-:155239391-155241412
1709	Mt2	17750	8+:96696346-96697461
1711	Grhl1	195733	12+:25265583-25302252
1712	Rnf2	19821	1-:153316535-153348027
1714	Ampd1	229665	3+:102877953-102903646
1716	Git1	216963	11+:77307063-77321269
1717	Ndufs2	226646	1-:173176219-173177245
1719	Trak2	70827	1-:58957293-59030314
1722	Iqgap1	29875	7-:87857671-87948180
1724	MMT00004378		X-:92406801-92407116
1728	Car6	12353	4-:149561126-149572579
1731	Rnf20	109331	4+:49644958-49670124
1732	Hspa4	15525	11-:53075072-53113683
1734	Arrdc2	70807	8-:73360045-73363618
1736	Elovl3	12686	19+:46206388-46210184
1737	Gtpbp4	69237	13-:8972368-8995242
1739	Lrrc17	74511	5+:21049376-21081718
1740	RP23-244H7.9	378700	2+:153745371-153758732
1744	Clec3b	21922	9+:123059966-123066545
1745	Gas7	14457	11+:67416606-67498461

1747	RP23-162H3.1	666581	4+:146753337-146753561
1748	Rbp1	19659	9+:98323379-98346967
1750	Fcer1a	14125	1-:175151414-175157345
1751	Xdh	22436	17-:74233251-74299493
1755	Ocel1	77090	8+:73895224-73897056
1758	Cadps2	320405	6-:23446750-23638963
1759	Rgs3	50780	4+:62220880-62320868
1760	Nek8	140859	11-:77979607-77990118
1761	2010110K16Rik	67899	9-:117973646-118059210
1762	EG620986	620986	1-:9792275-9792622
1764	Tifab	212937	13-:56275845-56280014
1766	Srpx2	68792	X+:130443234-130466352
1767	BC016495	225994	19+:18706505-18726505
1768	Fmo9	240894	1-:168593079-168611977
1769	Nr5a1	26423	2-:38548146-38570020
1770	BC054059	246747	2+:158328347-158333934
1771	Park2	50873	17+:11494485-11696865
1772	Stambp	70527	6-:83493200-83522430
1776	4921511C04Rik	70853	1+:37210690-37244460
1778	Gtf2e1	74197	16-:37509881-37539855
1783	Arcn1	213827	9-:44550229-44575876
1786	Tnfsf10	22035	3+:27216022-27235019
1787	MMT00004455		14-:63133622-63134211
1790	Hic1	15248	11-:74978070-74983021
1792	Zmat4	320158	8+:24858871-25125688
1793	D3Ucla1	28146	3-:58325899-58329676
1795	Rabgap1l	29809	1-:162237110-162402248
1797	Batf2	74481	19+:6140980-6172472
1798	Hoxc5	15424	15+:102844512-102845873
1800	Retn	57264	8+:3655788-3658093
1802	Ipo4	75751	14-:56244749-56254435
1804	Tgm2	21817	2-:157943535-157972042
1805	Tmem204	407831	17-:25194652-25218124
1806	Nola3	66181	2+:112102125-112103055
1813	Commd3	12238	2+:18594091-18597551
1816	Gjb2	14619	14-:57718907-57719586
1820	Mat2b	108645	11-:40492815-40506115
1827	Gnb1l	13972	16+:18498912-18564614
1828	Elmo3	234683	8+:107821623-107833891
1829	MMT00030026		1+:193816705-193817410
1834	Ermap	27028	4-:118848195-118862579
1839	Igf2	16002	7-:149836673-149847765
1840	Fpr1	14293	17-:18013596-18014689
1841	MMT00054498		2+:31841028-31842169
1849	MMT00063181		1-:151869353-151870959
1851	Tmem41a	66664	16-:21934399-21947617

1853	Stat5a	20850	11+:100720969-100746470
1855	Prss12	19142	3+:123149830-123209512
1856	MMT00000408		9-:108260488-108261433
1858	2810432L12Rik	67063	4-:49598286-49610670
1862	Vdac1	22333	11+:52174616-52202898
1863	MMT00052951		5-:109445281-109445937
1866	2010012O05Rik	66439	19+:46764403-46776385
1867	Bcas2	68183	3+:102975667-102982750
1868	Atp6v0e2	76252	6+:48487613-48491793
1870	EG627821	627821	7+:6224575-6233457
1874	Slc19a2	116914	1+:166179185-166195497
1876	Amdhd1	71761	10-:92986449-93002732
1877	Amica1	270152	9+:44896695-44916591
1881	Slc16a14	71781	1-:84908769-84931804
1885	Smr3a	20599	5+:88431573-88437558
1886	Tcte1	21645	17+:45670001-45679629
1887	Mapkapk3	102626	9-:107157262-107191727
1888	Olfr1396	258334	11-:48926279-48927259
1889	Dcun1d3	233805	7-:127000760-127039194
1890	Nanp	67311	2-:150855452-150865091
1892	A1bg	117586	15-:60749156-60752829
1893	MMT00072326	330914	9+:31995218-32068816
1894	Lrp4	228357	2+:91297664-91351916
1899	1110031B06Rik	67826	11-:59220652-59263458
1900	Hlf	217082	11-:90249097-90252154
1901	Tpp1	12751	7-:112894592-112900729
1902	Eml2	72205	7+:19761962-19791828
1906	Ssr3	67437	3-:65183578-65196475
1907	D230010M03Rik	241547	2+:91551082-91553022
1908	Rnf213	629974	11+:119308548-119311188
1910	Krtap5-2	71623	7+:149442728-149444277
1911	1300007F04Rik	67477	11+:77328621-77334117
1913	Polr2j	20022	5+:136592606-136598809
1918	Usp39	28035	6-:72268671-72295169
1920	Oas1e	231699	5-:121236330-121245513
1921	4930431L04Rik	270049	8-:60980148-61179845
1928	Triml2	622117	8+:44285339-44285697
1929	Leng8	232798	7+:4088637-4099773
1930	Ccdc109b	66815	3-:129617878-129672997
1932	Racgap1	26934	15-:99450955-99482031
1935	2810439F02Rik	72747	18+:12802308-12895558
1936	Cbwd1	226043	19-:24994920-25036048
1937	MMT00075644		3-:26945708-26946550
1940	MMT00002367		11+:41312177-41313958
1941	MMT00079618		6+:78998315-78998815
1947	Myo9b	17925	8+:73796632-73884612

1954	Bank1	242248	3-:135876793-135988939
1956	Usp50	75083	2-:126587157-126609180
1957	Mansc1	67729	6-:134559227-134582420
1958	Bai1	107831	15+:74359586-74419886
1960	Ubr2	224826	17-:47067196-47107076
1961	Mlf1	17349	3+:67178112-67203884
1963	MMT00039783		10+:34618039-34618914
1964	C730048C13Rik	319800	19-:8145456-8479572
1967	Brwd1	93871	16-:96214055-96304033
1968	Nudc	18221	4-:133088458-133101864
1972	Ly6g6e	70274	17+:35213711-35215749
1975	Dnahc9	237806	11-:65644827-65794849
1978	Ublcp1	79560	11-:44268078-44274332
1979	Tubgcp4	51885	2+:120996903-121024358
1982	EG668192	668192	10+:43541036-43542042
1984	Ubqln3	244178	7-:111289418-111291464
1985	Pepd	18624	7+:35697425-35829725
1986	Crebl2	232430	6+:134799120-134808700
1991	Ttc17	74569	2-:94140923-94246822
1995	Rab6b	270192	9+:103014278-103085542
1996	Gsdma3	450219	11+:98460132-98499247
1998	MMT00061243		9-:22284377-22284926
2000	Bola1	69168	3-:96000163-96001660
2002	Hapln3	67666	7-:86261914-86275906
2003	Ppapdc1	71910	8+:26830513-26835364
2005	Rheb	19744	5-:24309068-24348231
2011	Slc2a5	56485	4+:149494830-149517864
2012	Cetn4	207175	3-:37207549-37211368
2013	Il15ra	16169	2+:11627162-11655588
2016	Wdr78	242584	4-:102710674-102786829
2017	Dock2	94176	11-:34127668-34597325
2018	Atp7a	11977	X+:103265113-103320039
2019	Sf3a1	67465	11+:4060374-4080626
2021	Atf7ip	54343	6+:136467378-136555589
2023	MMT00072215		1+:77046869-77047310
2024	Tdgf1	21667	9-:110842106-110848571
2026	Nrg2	381149	18-:36177313-36228471
2027	Wnt5b	22419	6-:119383007-119398272
2028	Isca2	74316	12+:86114218-86116035
2031	Il6	16193	5+:30339700-30346508
2033	2010106E10Rik	67715	X+:109608883-109671942
2035	Slc44a3	213603	3-:121162446-121235249
2037	Ankrd16	320816	2+:11700151-11711955
2039	9930105H17Rik	100669	14+:41866897-41867955
2041	Cct7	12468	6+:85409107-85418368
2043	Arl2bp	107566	8+:97190654-97198320

2044	Hsd17b7	15490	1-:171882491-171899326
2049	Gsta3	14859	1+:21239829-21255300
2050	Lpl	16956	8+:71404452-71430830
2051	Ddx27	228889	2+:166840809-166891360
2054	Acaa1a	113868	9+:119250595-119259412
2056	Ttn	22138	2-:76705214-76709945
2057	Pawr	114774	10+:107769727-107851885
2058	Hk3	212032	13-:55106856-55122559
2062	EG668830	668830	17-:81337261-81338134
2065	Aph1a	226548	3+:95697915-95701385
2066	Olfr1256	258985	2-:89675179-89676100
2067	Myo1a	432516	10+:127142870-127157997
2074	Sfxn1	14057	13+:54180828-54201971
2075	Atp6v1g1	66290	4+:63205870-63211734
2079	Mthfs	107885	9+:89106027-89135063
2081	Dlat	235339	9-:50444459-50467622
2082	AU018778	234564	8-:95780140-95803610
2084	Pftk1	18647	5-:5373457-5380364
2086	Prpf8	192159	11+:75300591-75322885
2088	Ppan	235036	9+:20692700-20696622
2090	Rpo1-2	20017	2+:128926776-128952330
2093	Mbp	17196	18+:82644554-82755019
2095	Siglech	233274	7+:63023596-63027953
2096	MMT00072212	380822	12-:116446282-116746212
2097	C8a	230558	4-:104488290-104548988
2106	4931406H21Rik	77592	14-:26406032-26413423
2110	Gtf2ird1	57080	5-:134833538-134932560
2113	Rasd1	19416	11-:59776686-59778437
2115	Gpr12	14738	5-:147394327-147396408
2118	Kptn	70394	7+:16705256-16712863
2119	Lamc3	23928	2+:31742809-31802640
2121	Brms1l	52592	12+:56937603-56970720
2126	Dgat2	67800	7-:106302951-106331081
2127	Ldha	16828	7+:54102582-54110996
2128	4930447K03Rik	432745	13-:34176023-34205141
2130	Smcr7	237781	11+:60541924-60546442
2134	Pes1	64934	11+:3864039-3870699
2141	Chmp1a	234852	8-:125728163-125736638
2142	Phldb1	102693	9-:44494391-44535257
2145	MMT00075271		7+:13328413-13329074
2146	MMT00044046		18-:51277050-51414788
2148	Tas2r105	57252	6-:131636578-131637481
2150	Rragc	54170	4+:123594755-123614233
2157	0610007C21Rik	381629	5+:31350592-31356994
2162	2410042D21Rik	72425	2+:112219411-112253491
2166	Amd1	11702	10-:40007283-40021921

2167	Pif1	208084	9+:65435008-65443769
2174	MMT00053383		7+:24142108-24143830
2177	Htatip2	53415	7+:57014968-57028895
2178	Slc8a1	20541	17-:81784942-82137709
2179	Trim3	55992	7-:112759161-112782045
2185	Pef1	67898	4+:129784858-129805378
2187	Kif5b	16573	18-:6203086-6241342
2188	Prpf38a	230596	4-:108237313-108251934
2190	Heyl	56198	4+:122910798-122925962
2199	Aff1	17355	5+:104283521-104284335
2200	BC006779	229003	2-:180962318-180975622
2203	Cts3	117066	13-:61589372-61591397
2207	Ogn	18295	13+:49704513-49718684
2210	Chn1	108699	2-:73450944-73463797
2211	Ttpa	50500	4+:19935586-19957932
2214	Larp6	67557	9+:60560923-60586337
2215	Zfp71-rs1	235907	13-:67806245-67830019
2218	D15Mgi27	106073	15+:102110526-102112184
2222	Tagln	21345	9-:45738056-45743980
2223	Slc28a2	269346	2+:122267294-122286740
2224	Oasl2	23962	5+:115346944-115362235
2225	Ceacam11	66996	7+:18557472-18563898
2226	Amt	434437	9+:108199212-108204964
2227	Cenpa	12615	5+:30969274-30977197
2228	Nox1	237038	X-:130621685-130642380
2230	Slc35d1	242585	4-:102846085-102887314
2231	Piwil4	330890	9-:14506220-14539101
2232	Strn	268980	17-:79054572-79135827
2234	MMT00046581		16-:55940431-55940830
2235	Ndufv1	17995	19-:4007506-4012725
2237	Gpr110	77596	17+:43407277-43461686
2239	4930503E24Rik	75030	10+:123906127-123961543
2241	Trim7	94089	11+:48650972-48654917
2243	Gcsh	68133	8-:119505729-119517349
2244	Klre1	243655	6+:129528324-129535842
2245	Sh2d4b	328381	14-:41673671-41706556
2249	BB014433	434285	8-:15041449-15046069
2253	Lysmd1	217779	3+:94938016-94943440
2256	Serpina9	71907	12-:105234831-105251862
2259	Ddx3x	13205	X+:12858163-12869740
2260	Spink10	328971	18+:62810503-62821020
2262	Papss2	23972	19+:32693437-32740227
2263	Dpy19l2	320752	9-:24361496-24500734
2264	Alb	11657	5+:90889920-90905775
2266	Angptl2	26360	2+:33083714-33101821
2270	Pter	19212	2+:12845756-12923240

2273	Haghl	68977	17-:25919769-25922278
2274	Tnnc2	21925	2-:164602663-164605229
2276	Nelf	56876	2+:24909932-24918400
2278	Gtf3c1	233863	7-:132784466-132851279
2282	Cbx8	30951	11-:118899751-118902227
2283	MMT00029554		10+:81893449-81894588
2290	Il1rapl2	60367	X+:134106087-135381494
2291	MMT00041633		13-:61788625-61789350
2294	AU040829	216831	11-:64816675-64976461
2297	Syce2	71846	8+:87396009-87411294
2301	Pcyox1	66881	6-:86336965-86347100
2304	Jund	16478	8+:73222931-73224514
2305	2610002M06Rik	67028	X-:104982682-105011620
2306	Gtdc1	227835	2-:44419938-44490836
2308	A930005I04Rik	403174	5+:35260190-35266487
2309	Tet2	214133	3-:133128829-133134781
2310	Pdia3	14827	2+:121239695-121263715
2311	RP23-336F11.32	213765	2-:112090715-112095980
2314	Zswim6	67263	13-:108516172-108523916
2315	Abcb1a	18671	5+:8660091-8748519
2317	Ankar	319695	1-:72696779-72705377
2318	Igh-VJ558	16061	12-:117150531-117151034
2319	E2f1	13555	2-:154385373-154395588
2320	Spc24	67629	9-:21560437-21564747
2321	ENSMUSG000000742	677044	9-:62689305-62690180
2322	Tuba4a	22145	1-:75212130-75215799
2325	H2-K1	14972	17-:34133342-34137230
2326	zfp507	668501	7-:36560979-36662496
2328	Traf3ip1	74019	1+:93391254-93425949
2329	Wdr6	83669	9-:108474648-108480997
2330	Ccdc28b	66264	4-:129296518-129301144
2332	Nox3	224480	17-:3646475-3695583
2334	Gch1	14528	14-:47775327-47809064
2336	Lpar3	65086	3+:145883959-145948924
2338	Olfr620	258808	7-:110759924-110760865
2339	5730449L18Rik	66637	1-:154217868-154233812
2344	Ogt	108155	X+:98835624-98877668
2347	Zmynd17	74843	14-:21302091-21315905
2348	Irak1	16179	X-:71260321-71269254
2350	Ruvbl1	56505	6+:88415458-88447559
2351	1110008F13Rik	67388	2+:156688863-156699295
2356	Dnajc13	235567	9-:104053611-104140870
2360	6330407G11Rik	68050	4-:123412602-123427504
2362	Mkks	59030	2-:136699800-136716914
2365	BC031181	407819	18+:75165582-75169494
2369	MMT00026815		11-:121767077-121831952

2370	8430408G22Rik	213393	6+:116600716-116602811
2372	Stx12	100226	4-:132409978-132440316
2377	Mmd	67468	11+:90110836-90139891
2384	Micalcl	70877	7+:119517848-119556616
2385	Ctsd	13033	7-:149561821-149573775
2387	Dscc1	72107	15-:54907654-54922049
2388	Dot1l	208266	10+:80217707-80256188
2394	Tnc	21923	4-:63620821-63681760
2397	Rb1cc1	12421	1+:6220083-6264356
2399	MMT00060167		1-:173609465-173609784
2400	Gck	103988	11-:5801673-5815014
2403	Cfl1	12631	19+:5490505-5494032
2404	MMT00049822		1-:113701733-113704372
2407	Pgap1	241062	1-:54543313-54550250
2420	2010016I18Rik	69206	3-:106284902-106286465
2421	Itgb5	16419	16+:33829844-33949231
2424	Unc5cl	76589	17+:48594246-48608007
2428	Car1	12346	3-:14766229-14808322
2433	Armc10	67211	5+:21151801-21168410
2436	Fblim1	74202	4-:141132915-141151866
2437	Lrrc23	16977	6-:124719880-124729736
2439	Pitpnm2	19679	5-:124568699-124666427
2440	1500003O22Rik	101867	7-:112880722-112885014
2442	Rab2b	76338	14-:52881440-52899222
2443	5033411D12Rik	192136	13-:16949313-17786589
2447	Ubac1	98766	2-:25854067-25877231
2449	Mybpc2	233199	7-:51757087-51780009
2454	MMT00045890		3-:108042224-108042877
2456	Mrpl10	107732	11+:96902899-96910527
2457	1110032E23Rik	68659	3+:79689909-79747306
2461	Appbp2	66884	11-:85004860-85048327
2462	2610528J11Rik	66451	4+:118199886-118202822
2464	A130022J15Rik	101351	6-:97059922-97085264
2465	MMT00048312	632654	12-:115460995-115461531
2469	Coasy	71743	11+:100943919-100947931
2470	Grb7	14786	11+:98308204-98316686
2471	Ccdc134	76457	15+:81958392-81972630
2472	MMT00014056		14+:114879627-114879658
2473	4930504H06Rik	75040	12+:34079717-34086135
2475	Cyp4f13	170716	17-:33061738-33102711
2477	Cldn11	18417	3+:31048912-31063241
2479	Cenpo	52504	12-:4213481-4233937
2480	MMT00000773		
2483	Gmeb1	56809	4-:131907849-131909549
2485	Adck1	72113	12+:89598997-89700161
2486	Ephb1	270190	9-:101824459-101969363

2489	Npr3	18162	15-:11774933-11835394
2490	MMT00070880		17+:6806524-6812615
2493	Apoh	11818	11+:108256626-108275705
2500	Vmn2r65	100009609	7-:92091650-92096161
2501	Isx	71597	8+:77396613-77417405
2507	Oaf	102644	9-:43030009-43047993
2510	Olfr136	258803	17+:38472103-38473041
2511	Ltv1	353258	10-:12897947-12912946
2512	Gan	209239	8+:119682034-119729083
2520	Rgn	19733	X+:20126943-20139213
2521	Olfml3	99543	3-:103539321-103541871
2522	Olfr1442	258692	19+:12748696-12749633
2523	Pgs1	74451	11+:117848193-117885324
2527	1600012F09Rik	67008	13-:18080036-18085171
2529	Hmcn2	665700	2+:31235722-31316253
2533	Arhgef19	213649	4+:140803761-140813467
2534	4921517D22Rik	70900	13-:59790687-59795463
2538	Clec4g	75863	8-:3716071-3720645
2541	Ppa2	74776	3+:132973079-133041031
2542	Dck	13178	5+:89194268-89212264
2543	MMT00001648	83409	3-:88353828-88356819
2550	MMT00036681		15-:77234219-77236051
2554	Sphk1	20698	11+:116396053-116397982
2555	MMT00033243		X+:73160819-73161742
2557	Casq1	12372	1-:174140470-174149972
2558	Tmem132b	208151	5+:126229604-126269041
2562	Nhedc2	97086	3+:134981255-135005500
2565	Zbtb41	226470	1+:141319727-141344171
2570	Tmod4	50874	3+:94929452-94933130
2571	Rmnd1	66084	10+:5914151-5943319
2578	Csf2rb2	12984	15-:78112939-78136033
2580	Ucn3	83428	13-:3940403-3940896
2583	Runx1	12394	16-:92604349-92697573
2584	Serpind1	15160	16+:17336313-17343665
2595	Acbd4	67131	11+:102964825-102973513
2596	Vsig4	278180	X-:93464386-93464955
2598	Naglu	27419	11+:100931438-100938965
2599	Lsm14a	67070	7-:35129663-35174628
2601	Dnajc18	76594	18-:35834684-35862745
2603	Ipp	16351	4+:116183176-116193421
2605	N4bp2l2	381695	5-:151449573-151465088
2606	MMT00077133		
2607	Olfr1467	258686	19+:13439119-13440045
2608	Clcn2	12724	16-:20703040-20716187
2612	Sf3b2	319322	19-:5273934-5295425
2615	EG624862	624862	14+:44842695-44843903

2618	Map3k7ip3	66724	X+:82877615-82879647
2619	Zfp622	52521	15+:25914214-25927182
2625	MMT00077446		15-:73237740-73238725
2626	MMT00013951		4+:89940729-89940760
2627	Ccl12	20293	11+:81915379-81916793
2630	Il17re	57890	6+:113408827-113420368
2631	Aldh1l1	107747	6+:90500807-90550196
2632	Aph1a	226548	3+:95697918-95702211
2635	Tmem126a	66271	7-:97599221-97605718
2636	Tars	110960	15-:11313417-11329413
2638	Cyp3a41a	53973	5-:146505625-146531712
2639	Abhd2	54608	7+:86418161-86505287
2642	Neurod2	18013	11-:98187852-98190775
2643	Inhbe	16326	10-:126786457-126788828
2644	Srrm2	75956	17+:23955853-23996744
2646	Ugt2b37	112417	5-:87669517-87683829
2648	MMT00005098		6+:22683708-22684595
2651	Ttc7	225049	17+:87681989-87781713
2655	Adamdec1	58860	14-:69181444-69200129
2658	Fzd2	57265	11+:102465848-102467886
2659	Kcng2	240444	18-:80492127-80519384
2661	Eif4g2	13690	7-:118215446-118226486
2662	Sgpl1	20397	10-:60563471-60610346
2668	C3ar1	12267	6-:122797991-122806021
2669	Ier2	15936	8-:87185236-87186752
2670	Igsf1	209268	X-:47135720-47147957
2671	Sh3rf1	59009	8+:63703068-63872992
2674	1810005K13Rik	66255	18-:80426804-80433600
2675	Nfyc	18046	4-:120430782-120463086
2677	2310057J18Rik	67719	10-:28692170-28706109
2681	Hectd1	207304	12-:52844708-52847097
2693	Asprv1	67855	6+:86578720-86579694
2694	Siah2	20439	3-:58478877-58496025
2699	Hax1	23897	3-:89799378-89802574
2703	MMT00073763		1+:163676198-163677877
2705	Hrbl	231801	5-:138104053-138125921
2707	Defb39	360214	8-:19037369-19064810
2708	Sgk1	20393	10+:21714500-21719695
2713	Six1	20471	12-:74142817-74147687
2714	1700001K19Rik	66323	12-:111905926-111920822
2716	BC014805	236149	19-:7856474-7877157
2718	Scrn3	74616	2+:73150627-73173922
2719	St6galnac2	20446	11-:116538018-116555939
2721	Inmt	21743	6-:55120620-55124984
2724	Tpsab1	17230	17-:25480190-25482338
2725	Gm1614	381148	18-:35811820-35814853

2726	Chd9	109151	8+:93513353-93578409
2727	Stc2	20856	11-:31260080-31269908
2728	Exoc4	20336	6+:33199123-33922332
2729	Dcun1d4	100737	5+:73875636-73951753
2730	Bcor	71458	X-:11613868-11638772
2737	Mllt10	17354	2+:17986313-18132597
2741	3230401D17Rik	66680	2-:163231672-163245225
2743	Tcf3	21415	6-:72577163-72738950
2749	Naf1	234344	8+:69384156-69414459
2751	Lrriq1	74978	10-:102633054-102698956
2752	Fcgr1	14129	3-:96088140-96097886
2753	Myo6	17920	9+:80012911-80157198
2754	Zc3hav1l	209032	6-:38245057-38249315
2755	EG382421	382421	10+:110937798-110938837
2756	Pmvk	68603	3+:89263237-89272928
2757	EG435927	435927	6-:141684625-141693021
2764	Ffar2	233079	7-:31604049-31605344
2766	Elp4	77766	2-:105542599-105744652
2767	MMT00031206		6+:57138500-57139337
2769	AI606181	226118	19+:41668209-41670648
2770	Fbl	14113	7+:28954778-28964268
2771	Keg1	64697	19+:12770282-12794384
2775	Sec61a2	57743	2-:5792855-5816331
2776	Magel2	27385	7+:69524349-69526524
2778	Coro1a	12721	7-:133843394-133846687
2780	Fes	14159	7-:87522641-87532846
2781	Lamb2	16779	9+:108382233-108392792
2783	Pdzrn4	239618	15+:92427591-92511021
2788	Galnt10	171212	11+:57583010-57599560
2789	Sirt5	68346	13+:43460868-43490571
2793	MMT00005644	497266	
2794	Map3k6	53608	4+:132796732-132808843
2800	Dusp11	72102	6-:85897507-85900556
2801	H2-Bl	14963	17-:36216993-36221404
2802	Lin7c	22343	2+:109731034-109737862
2805	Rhog	56212	7-:109388183-109388758
2808	Spesp1	66712	9-:62118535-62129986
2811	MMT00062390		7+:144002509-144004197
2813	Mrpl55	67212	11+:59016018-59019504
2815	Eif2ak3	13666	6+:70794528-70855231
2818	Egr1	13653	18+:35020859-35024635
2819	Kcnk5	16529	14-:20960805-21000674
2823	Sipa1l2	244668	8-:127941972-128016496
2827	Raet1d	56554	10+:22082079-22093806
2829	Lrfn3	233067	7-:31140503-31145817
2830	4632417K18Rik	107373	19+:12648014-12664186

2832	2810021J22Rik	69944	11+:58680716-58693934
2834	Crim1	50766	17+:78743688-78775925
2835	Gpr120	107221	19+:38171568-38188749
2838	Stk25	59041	1-:95518484-95531962
2841	Kcne4	57814	1+:78813523-78816599
2845	Lce1d	69611	3-:92489420-92491132
2847	Pscd4	72318	15+:78449725-78452443
2848	Serpine2	20720	1-:79790897-79855211
2850	Slc16a6	104681	11-:109314053-109334523
2851	Inpp5e	64436	2-:26252027-26264701
2853	Glipr2	384009	4+:43970561-43991988
2855	D330050I23Rik	399603	15-:60654517-60655450
2858	Arhgap20	244867	9+:51593840-51661960
2862	MMT00068885		14-:79365696-79366375
2863	MMT00007006		12-:119996497-119999465
2866	Clk1	12747	1-:58468844-58480848
2867	MMT00001941		6-:39792201-39794334
2868	Hbp1	73389	12-:32611342-32635100
2869	Copa	12847	1+:174012669-174052460
2870	D5Ertd577e	320549	5+:95947713-95951732
2874	Nol3	78688	8+:107800401-107803949
2875	Wfdc2	67701	2+:164388215-164394006
2877	Pcyt2	68671	11-:120471401-120479198
2879	Ptprv	13924	1-:137005090-137023649
2880	Cd93	17064	2-:148262387-148269280
2881	Hlf	217082	11-:90197850-90207295
2883	MMT00048540		17-:90309276-90379134
2885	Hebp2	56016	10-:18260571-18265814
2889	Pqlc2	212555	4-:138854445-138866582
2895	2410076I21Rik	73673	9-:58500635-58589403
2900	6330416L07Rik	319615	17-:22475663-22498377
2901	Tmem132d	243274	5-:128263790-128276473
2902	D4Ertd196e	52174	4-:132821961-132833702
2904	Gzme	14942	14-:56736460-56739393
2907	MMT00010854		3+:100217482-100218699
2912	Wtip	101543	7-:34894583-34918078
2915	Nfs1	18041	2-:155949385-155969921
2917	Scly	50880	1+:93194962-93217639
2918	Mtmr10	233315	7+:71432585-71485693
2920	4922501C03Rik	382090	9+:87084408-87085910
2928	Igf1	16000	10+:87324073-87393619
2932	Slco1a1	28248	6-:141856373-141895315
2935	Slc28a1	434203	7+:88269753-88313948
2938	H2-T23	15040	17-:36166923-36169646
2945	MMT00005190		X-:131362702-131363503
2947	Bcl2a1b	12045	9+:89094066-89102664

2950	Micall1	27008	15+:78945434-78967328
2954	AI317395	215929	10-:39722405-39744978
2958	Grik4	110637	9-:42328494-42496180
2959	Rgs3	50780	4+:62350531-62363279
2962	AW146154	101835	7-:48722734-48754789
2963	Tmem64	100201	4+:15193310-15213871
2965	Fgf15	14170	7+:152082436-152086854
2967	Igh	111507	12-:116081854-116170993
2969	Ptpro	19277	6+:137359257-137411751
2970	MMT00022996		10+:18821380-18822122
2972	Zfp3	193043	11+:70577859-70586432
2973	4-Sep	18952	11+:87394632-87404042
2978	St6galnac1	20445	11-:116626339-116627586
2980	Slc37a2	56857	9-:37037898-37063215
2984	MMT00029895		13-:50710320-50711479
2989	Zfp36l1	12192	12-:81208994-81213931
2993	Gm1752	385658	16-:55843302-55895391
2994	Cldn7	53624	11+:69779428-69781218
2996	Pik3r1	18708	13-:102450519-102538126
2998	Lzic	69151	4+:148859401-148870773
2999	BC061194	381350	2+:18620696-18671374
3006	Alas2	11656	X+:146981966-147005166
3008	Nxt1	56488	2+:148498409-148501499
3009	Serpina1c	20702	12-:105133137-105137037
3012	Actl6a	56456	3+:32607647-32625880
3015	Arhgef6	73341	X-:54484663-54591865
3016	Bsdc1	100383	4+:129138915-129165740
3017	Fas	14102	19+:34365155-34402260
3021	Gdf9	14566	11+:53246879-53251402
3022	Als2cr12	108812	1-:58714959-58752806
3026	D10Ertd610e	52666	10-:126619581-126627091
3028	Uhrf1	18140	17+:56442848-56462908
3029	Ybx1	22608	4-:118950838-118966413
3031	Rab10	19325	12-:3249734-3309662
3036	Slamf9	98365	1+:174405535-174408538
3037	MMT00017312		17-:15712487-15714358
3038	Serpina12	68054	12-:105266978-105282653
3039	S100a11	20195	3+:93324410-93330219
3040	Bcl3	12051	7-:20393811-20408064
3041	2410004A20Rik	66991	9+:72950266-72952250
3043	Otop1	21906	5+:38668654-38685787
3045	Alx3	11694	3+:107397948-107408687
3046	6430573F11Rik	319582	8+:37521311-37576010
3052	9130404D08Rik	74549	8-:72542479-72566599
3054	Hnrnpa1	15382	15+:103070935-103075386
3055	Pde4c	110385	8+:73247962-73274318

3059	Tctex1d2	66061	16+:32419829-32429184
3061	Scarf2	224024	16+:17802608-17834411
3066	MMT00035562	545618	4-:44765021-44766010
3067	Ucp3	22229	7+:107627975-107633616
3072	MMT00002308		1-:13397087-13397792
3075	EG629203	629203	7-:14652905-14708339
3077	Ppfibp1	67533	6+:146837182-146980547
3078	9630033F20Rik	319801	6-:127037487-127059512
3079	Gstt3	103140	10-:75236865-75243787
3080	MMT00014480		15+:60575413-60578213
3086	Tubg2	103768	11+:101017226-101023100
3088	Ftl2	14337	4+:124787922-125182221
3089	Eif2s3y	26908	Y+:347054-365035
3093	Egflam	268780	15-:7156120-7348390
3096	Ugt1a6a	94284	1+:90031383-90115578
3097	Ppp1r3g	76487	13+:36035371-36061510
3101	MMT00080431		7-:7013153-7015975
3103	MMT00062748	625360	Un_random-:688-21009
3104	4933411G11Rik	330228	5+:143953957-143966747
3107	Hspb3	56534	13-:114453103-114453867
3111	Ddefl1	230837	4+:135762433-135801229
3116	Gm1574	380842	13+:46380746-46394099
3119	Mapk10	26414	5-:103341387-103640096
3127	Plcd1	18799	9-:118980647-119002614
3130	Mrpl20	66448	4+:155177726-155182938
3134	Usp11	236733	X+:20289265-20297663
3135	Gcdh	270076	8-:87410489-87417601
3136	Itfg1	71927	8-:88242438-88364671
3138	Espl1	105988	15+:102126691-102154789
3139	Gba2	230101	4-:43579792-43591409
3140	Ptpn20	19256	14+:34416291-34453940
3141	Tmf1	232286	6-:97105257-97129094
3143	Slc17a6	140919	7+:58877375-58924852
3144	Ube2j2	140499	4+:155323115-155331473
3145	Idi1	319554	13+:8885230-8891614
3147	Higd2a	67044	13+:54691591-54692493
3149	1190005I06Rik	68918	8-:123132502-123152417
3150	Syne2	319565	12+:77167499-77211887
3154	Mcm5	17218	8+:77633522-77651587
3156	Usp13	72607	3+:32716612-32834476
3159	Pld1	18805	3+:27837544-28032284
3162	Lep	16846	6+:29018951-29021181
3163	MMT00039919		13-:8028796-8030700
3164	Hsd11b1	15483	1-:195047886-195090214
3166	Vmn2r57	269902	7-:48655329-48683582
3167	Dym	69190	18+:75216372-75446618

3168	Camk2a	12322	18+:61123207-61144517
3170	Mcart1	230125	4-:45411210-45421633
3173	Glycam1	14663	15-:103393188-103395512
3174	Acyp1	66204	12-:86613355-86621385
3177	Setd8	67956	5+:124889948-124901388
3182	Stau1	20853	2-:166773772-166821799
3186	Kcnj16	16517	11+:110829345-110888155
3188	Plp1	18823	X+:133357289-133372867
3189	Ndst2	17423	14-:21542962-21549549
3190	Anxa4	11746	6-:86686840-86743550
3191	Lamc2	16782	1-:154969888-155033148
3194	Dnahc8	13417	17+:30807641-30814219
3195	Mypn	68802	10-:62578538-62656030
3196	Tat	234724	8+:112514356-112523700
3198	Fbxw4	30838	19-:45652746-45734683
3201	Ptcd2	68927	13-:100089605-100114651
3202	Boc	117606	16-:44485154-44520616
3204	Gm24	195236	13+:22073068-22076348
3206	Gpr160	71862	3+:30754883-30796089
3208	4930511I11Rik	67645	17+:28667805-28676052
3209	Lmna	16905	3-:88287127-88297234
3211	MMT00046813		1+:144651732-144653209
3213	Prdx6	11758	1-:163171140-163181296
3214	Dnmt3b	13436	2+:153475338-153513462
3219	Vps13a	271564	19-:16754499-16855407
3220	Ap2s1	232910	7+:17328567-17334361
3222	Zbtb10	229055	3+:9251357-9285327
3223	Ak2	11637	4+:128670545-128688054
3226	Tnfrsf17	21935	16+:11313901-11320161
3228	Saa1	20208	7-:53996074-53997892
3229	Tdo2	56720	3-:81762706-81779637
3230	Sh3bgrl	56726	X+:106290892-106356950
3235	BC029214	227622	2-:25310643-25316613
3236	Tcfap4	83383	16-:4544661-4559720
3239	Zfp294	78913	16-:87376923-87432780
3242	2700049A03Rik	76967	12+:72238207-72344284
3246	Prei4	74182	2-:132355135-132376184
3249	Pnpla8	67452	12+:45370172-45413274
3250	Olfm1	56177	2+:28061195-28085980
3253	Tbc1d20	67231	2+:152119604-152139732
3254	Grifin	77998	5-:141039077-141041023
3257	Map1lc3a	66734	2+:155102079-155103807
3259	Chrna6	11440	8-:28514848-28524254
3261	Rpap2	231571	5+:108026753-108090853
3265	Dntt	21673	19+:41103877-41134004
3274	Evx1	14028	6+:52263491-52268371

3277	MMT00038655		
3279	Slc46a1	52466	11+:78279176-78285445
3280	Tlr1	21897	5-:65316072-65323938
3282	Ndufs6	407785	13-:73457318-73465924
3283	Mmp9	17395	2+:164773756-164780405
3284	Plekha7	233765	7-:123298142-123301893
3286	Alox5ap	11690	5+:150076637-150099722
3288	Slc26a6	171429	9+:108757068-108815373
3291	Ptk2	14083	15-:73035535-73225154
3293	Nup153	218210	13-:46775254-46823638
3294	Prss23	76453	7-:96657618-96666054
3295	4930534B04Rik	75216	12-:92452004-92505454
3296	Olfr1143	258290	2+:87642547-87643490
3297	Trappc4	60409	9-:44211851-44215590
3298	Man2a1	17158	17+:64950988-65104450
3306	Rps6kb2	58988	19-:4156976-4163245
3307	Cd8b1	12526	6+:71272805-71287445
3308	Satb2	212712	1-:56853110-57028172
3309	Grpel2	17714	18-:61874372-61885977
3310	Tdrd5	214575	1-:158230724-158232736
3312	6430704M03Rik	230235	4-:56975738-57003009
3314	Wars2	70560	3+:98945006-99021178
3315	Serf2	378702	2+:121275152-121276793
3316	Fah	14085	7-:91733682-91754260
3317	Ak3	56248	19-:29095320-29123218
3318	2310003F16Rik	67693	2+:121282836-121284175
3322	Ofd1	237222	X-:162828874-162878489
3323	Idh3b	170718	2-:130105048-130110164
3325	Plekhj1	78670	10-:80258842-80261303
3326	Lysmd2	70082	9+:75473547-75485578
3328	Acsm2	233799	7+:126705245-126739690
3329	Car5a	12352	8-:124440045-124468804
3330	MMT00020226		10+:98907117-98908450
3332	Lrrc59	98238	11+:94491137-94506530
3337	Klra7	16638	6-:130168623-130181705
3339	Dip2a	64451	10-:75780611-75807996
3342	Ik	24010	18+:36904353-36917289
3344	Rad1	19355	15+:10415913-10423430
3345	EG620934	620934	7-:68694130-68694587
3349	MMT00043953		
3351	Rad23b	19359	4+:55363233-55402868
3353	Defb9	246079	8-:22992210-22995893
3354	Ropn1l	252967	15-:31370967-31383402
3356	Adhfe1	76187	1+:9538150-9566987
3357	Rev1	56210	1-:38109862-38165302
3359	Pp11r	19011	15-:97541449-97551581

3362	Fgf8	14179	19-:45811308-45817363
3363	MMT00033159		8-:58017542-58019101
3365	Tiam2	24001	17+:3397206-3519396
3372	Prok1	246691	3-:107038048-107041576
3376	Trip4	56404	9-:65681156-65732835
3377	Cs	12974	10+:127774857-127798355
3379	Cdo1	12583	18-:46873158-46888009
3380	MMT00067570		X-:17457996-17494616
3381	Ctdspl	69274	9+:118835589-118953116
3383	Cd99	673094	
3388	a	50518	2+:154839266-154876747
3389	MMT00013165		
3391	Mfap3	216760	11+:57332208-57344384
3393	Snhg8	66487	14-:31937673-31942116
3394	Als2cr13	72750	1+:60005896-60042189
3396	Ctage5	217615	12+:60230780-60291160
3401	BB146404	103511	10-:33811230-33816278
3402	A930018P22Rik	68243	2+:103962925-103964903
3405	Map3k3	26406	11+:105869516-106017968
3408	Cyp2c40	13099	19-:39841563-39887218
3410	Paqr9	75552	9+:95460377-95461504
3411	Rabl2a	68708	15-:89412967-89422296
3412	Olfr78	170639	7-:109890553-109891515
3414	MMT00059380		3+:57166891-57167200
3419	MMT00056938		X+:71252568-71252806
3421	2010106G01Rik	66552	2-:126719868-126758971
3426	Hap1	15114	11-:100208643-100217391
3428	Il1r2	16178	1+:40141612-40182068
3430	Rhof	23912	5-:123569439-123582428
3431	Lrfn5	238205	12+:62940414-62959327
3432	EG330963	330963	9-:65349991-65362552
3433	Naaladl2	635702	3-:24312271-24332132
3435	MMT00006420		X+:35533132-35536354
3445	Rnf25	57751	1-:74640326-74647951
3446	Eno1	13806	4+:149611363-149622981
3447	Fmr1	14265	X+:65931874-65971134
3448	Apobec3	80287	15+:79722855-79738344
3449	Hes6	55927	1-:93308080-93309767
3450	Itgb2l	16415	16-:96643897-96665206
3451	Spint1	20732	2+:119063153-119075247
3452	Ccrn4l	12457	3+:51028381-51055565
3454	MMT00030912	545291	19-:43368684-43462771
3455	Pou2af1	18985	9+:51021794-51048183
3456	MMT00063288		
3458	MMT00001194		4-:122952126-122952715
3459	Nags	217214	11+:102006900-102010384

3460	Ddb2	107986	2-:91051744-91077064
3462	Nmnat3	74080	9+:98197001-98311845
3463	MMT00029730		10-:53018944-53020003
3464	1700048O20Rik	69430	9+:121847232-121851112
3465	MMT00064177		X-:53098936-53099743
3468	MMT00009124		9+:71756669-71757359
3471	Stmn3	20262	2-:181041163-181048985
3475	2610305D13Rik	112422	4-:146986047-146989538
3480	Pnpo	103711	11-:96799141-96805260
3482	MMT00069653		14-:54943752-54944517
3485	Six3os1	100043902	17-:86011912-86018156
3491	Btn1a1	12231	13-:23548877-23557361
3493	Wbp4	22380	14-:79861555-79880917
3495	EG633752	633752	15+:68707192-68708287
3496	Slc41a2	338365	10-:82696146-82779298
3501	Raph1	77300	1-:60540030-60623608
3502	Gde1	56209	7-:125832516-125849233
3504	Anpep	16790	7-:86966690-86987170
3511	Rnf182	328234	13+:43763343-43816877
3512	6530401N04Rik	328092	12-:53089296-53107491
3513	Aloxe3	23801	11+:68939878-68962616
3523	Trim34	94094	7+:111393077-111485295
3524	Erc1	111173	6-:119520813-119521152
3526	Rccd1	269955	7-:87461398-87465354
3529	Cyp8b1	13124	9-:121823880-121825382
3531	Tslp	53603	18+:32975036-32979446
3532	S100a5	20199	3+:90412443-90415698
3535	S100a9	20202	3-:90496554-90499613
3537	F9	14071	X+:57252640-57283936
3542	Atp5j2	57423	5-:145944575-145952461
3545	Mtrf1l	108853	10+:4522321-4533475
3552	1110017I16Rik	68527	2+:4897203-4906790
3554	Npas3	27386	12+:54349663-55170060
3555	Sbds	66711	5-:130721604-130731385
3556	AI661453	224833	17+:47573587-47607092
3559	4933433P14Rik	66787	12+:106923037-106940153
3560	Usp5	22225	6-:124765036-124779465
3561	MMT00020644		4-:3646817-3648177
3562	MMT00011909		10+:66467767-66503648
3563	MMT00045357		14-:118580620-118581352
3564	Spcs2	66624	7-:106987418-106997571
3565	MMT00045908		5-:117442097-117452943
3569	ENSMUSG000000735	100038417	18-:62767196-62774658
3572	Ebi3	50498	17+:56092062-56096442
3577	Ptplad2	66775	4-:88059863-88084813
3578	A130090K04Rik	320495	10+:3366096-3455988

3579	Ncbp2	68092	16+:31948712-31957682
3584	Foxp1	108655	6-:99102052-99216516
3585	Hint1	15254	11+:54679941-54683999
3587	MMT00041940		3-:41779988-41781970
3594	Rasa4	54153	5+:136559867-136587725
3598	Rpo1-3	20018	5+:147889116-147890656
3599	MMT00035426		8+:35570238-35570761
3603	BC027231	212547	16+:44724416-44737241
3604	2610029G23Rik	67683	X+:102275133-102278823
3609	Plvap	84094	8-:74021665-74035630
3610	Rnf38	73469	4-:44139090-44200391
3612	Cd2	12481	3-:101079832-101091862
3614	2610030H06Rik	67048	X+:69061263-69070044
3616	Dnajc16	214063	4-:141319050-141344970
3617	Rasl10b	276952	11+:83232799-83234515
3620	Bet1	12068	6-:4027877-4036899
3622	Trim21	20821	7-:109707349-109713948
3624	Pygb	110078	2+:150612521-150657491
3626	Ralgps1	241308	2-:32989395-33140437
3627	MMT00048939		16-:18944350-18944684
3631	Vps37b	330192	5-:124454649-124482254
3632	Lctl	235435	9+:63964953-63985923
3635	Ndrg4	234593	8+:98226962-98238855
3640	D11Bwg0517e	52897	11-:118352177-118471514
3642	Spsb4	211949	9-:96843901-96896732
3643	Nell2	54003	15-:95050363-95358683
3647	Nucks1	98415	1+:133807070-133832744
3648	Odf2l	52184	3+:144781563-144816849
3649	Zfp395	380912	14+:66004224-66015602
3652	Adm	11535	7+:117771182-117773333
3654	St13	70356	15-:81195474-81230124
3655	0610009O20Rik	66839	18+:38409928-38415204
3657	Armcx1	78248	X+:131252549-131256443
3659	EG236451	236451	14-:59939802-59946936
3661	Dhx15	13204	5-:52541454-52581633
3665	Zfp61	22719	7-:25076064-25084609
3666	Tas1r1	110326	4-:151402022-151412677
3670	Dusp3	72349	11-:101835472-101846084
3671	1810033B17Rik	69189	8+:3665752-3668915
3673	Tro	56191	X-:147080247-147084030
3675	Pglyrp4	384997	3+:90532548-90544383
3676	Ucp2	22228	7+:107645261-107647959
3677	MMT00052835	633387	10+:38918264-38918972
3683	Oas2	246728	5-:121180342-121199797
3685	Apoc1	11812	7-:20274832-20277988
3686	Psmd9	67151	5+:123696248-123698970

3687	Bcl9	77578	3-:97007591-97009680
3689	Adh1	11522	3+:137940608-137953654
3695	1700007H22Rik	75484	16-:86139548-86150921
3696	Elovl6	170439	3+:129235356-129341406
3698	1600014C10Rik	72244	7+:38968834-38980115
3699	Dscr3	13185	16-:94719394-94748050
3700	Nrxn1	18189	17-:90436234-90458903
3701	Zfp68	24135	5-:139047347-139060955
3703	Ggcx	56316	6+:72364326-72380701
3704	H2-Q7	15018	17+:35576165-35580297
3705	5730590G19Rik	77011	7+:86814405-86820605
3706	2410002O22Rik	66975	13-:104933396-104968500
3707	Etos1	114660	7-:137914151-137916166
3708	Thrap3	230753	4-:125856027-125879856
3709	Mylip	218203	13+:45485109-45507382
3713	Acot6	217700	12+:85441455-85452294
3715	Mapk14	26416	17+:28828389-28885350
3717	Nlrp1c	627984	11-:70905760-71094536
3720	Zbtb44	235132	9+:30838107-30877921
3721	Me2	107029	18-:73929695-73975046
3725	OTTMUSG00000008	666048	4+:114252888-114283277
3727	Erdr1	170942	
3728	Sfxn4	94281	19-:60914648-60937267
3738	Cadm2	239857	16-:66663339-66815707
3743	Corin	53419	5-:72691264-72895447
3744	Rmnd1	66084	10+:5918863-5938724
3746	Osbpl5	79196	7-:150874667-150927868
3749	Aebp1	11568	11+:5761949-5772052
3753	Setdb1	84505	3-:95127447-95161078
3754	Dock6	319899	9-:21604303-21651716
3755	Ndn	17984	7+:69493207-69494811
3756	Chrne	11448	11-:70428385-70432696
3767	Pnma2	239157	14+:67530073-67536686
3768	Cyp2c44	226143	19-:44079510-44103732
3776	Btbd16	330660	7+:137923070-137967890
3777	Wdr91	101240	6-:34830279-34860843
3779	Ghdc	80860	11-:100627647-100632271
3780	Ryk	20187	9+:102737245-102810635
3781	Arpc2	76709	1+:74283073-74314782
3782	Rgs5	19737	1+:171585677-171622687
3783	MMT00082345		6-:39777684-39778771
3787	Myf6	17878	10-:106929915-106931785
3789	4833442J19Rik	320204	6+:149090114-149099694
3790	Mtss1	211401	15-:58774802-58913544
3794	Ywhab	54401	2+:163820933-163842538
3795	Psmc6	67089	14+:45951608-45969355

3796	D19Bwg1357e	52874	19-:27463189-27504341
3797	Nrip2	60345	6+:128349778-128358946
3799	Serpinh1	12406	7-:106493887-106501606
3800	Ltf	17002	9+:110921795-110944706
3801	Casp8ap2	26885	4+:32702487-32740235
3804	MMT00015534		5+:7179364-7180662
3806	MMT00063223		11-:102437760-102440602
3807	Vmn2r83	625029	10+:78940711-78943081
3808	2010209O12Rik	100910	5+:24093855-24098306
3811	MMT00052318		2+:23763958-23764363
3812	Zfyve26	211978	12-:80333330-80374663
3816	Gstm5	14866	3+:107698773-107701602
3818	Cep192	70799	18+:67994293-68044828
3823	Slc22a15	242126	3-:101663850-101728370
3825	Uaca	72565	9+:60642346-60728237
3832	Sdhb	67680	4+:140517319-140535015
3833	Atxn7l1	380753	12+:33832644-33936011
3834	Calb2	12308	8-:112666424-112692105
3836	2200001I15Rik	69134	14-:35165067-35168578
3837	Tradd	71609	8-:107782144-107788511
3838	Trim32	69807	4+:65266054-65277275
3841	MMT00014519		7-:49303224-49305709
3842	Abca12	74591	1-:71408315-71461470
3847	Tdrd7	100121	4+:46000333-46047629
3849	MMT00047578		1+:111171746-111732864
3850	Dach1	13134	14-:98188801-98568528
3856	Slc30a5	69048	13-:101572917-101584591
3857	1700001C19Rik	75462	17-:47550324-47574327
3858	Gcnt2	14538	13+:40982533-41054685
3859	Fpr2	14289	17+:18029707-18030762
3860	Mlx	21428	11+:100948632-100952829
3862	Gpd1	14555	15+:99548031-99554203
3865	Acta1	11459	8-:126415661-126418675
3867	Sln	66402	9+:53697970-53702367
3868	Ebpl	68177	14-:61959597-61979276
3869	Zadh2	225791	18+:84264344-84266906
3871	MMT00067361		12-:91309251-91382876
3874	Slitrk3	386750	3-:72852375-72860782
3875	MMT00077845		12-:45303417-45303845
3876	Zeb2	24136	2-:44843941-44965649
3877	Gsk3b	56637	16+:38090445-38240746
3880	Trf	22041	9-:103111206-103132616
3882	Itfg3	106581	17-:26349653-26381152
3885	Apol7c	108956	15-:77355282-77363746
3886	MMT00047275	546271	X+:23730763-23731986
3887	Tcrg-V5	21639	13+:19282236-19286398

3888	Ran	19384	5+:129526094-129528692
3889	Wasf3	245880	5+:147196581-147282701
3892	D0H6S2654E	108161	13+:34838320-34839491
3895	Art5	11875	7-:109245386-109251361
3901	F730047E07Rik	212377	4+:24448387-24530093
3904	Serinc1	56442	10-:57235583-57252255
3905	H2-Q1	15006	17+:35457506-35460984
3907	Dkk3	50781	7-:119260526-119302562
3908	Fundc2	67391	X+:72627760-72640322
3909	MMT00012984		5-:87389544-87390739
3910	Trpm7	58800	2-:126617301-126701947
3913	Mrpl15	27395	1-:4764014-4775808
3921	2300002D11Rik	69539	4-:133047015-133054050
3922	Clec11a	20256	7-:51559624-51562264
3923	D9Ertd402e	382117	9+:122714739-122744303
3927	MMT00021352		15-:99385921-99386665
3928	Polr3k	67005	2+:181599160-181604295
3929	Sfrs11	69207	3-:157673458-157699601
3938	Abt1	30946	13-:23513408-23515770
3943	Atoh1	11921	6+:64679316-64680372
3945	Cugbp2	14007	2-:6462915-6806015
3946	Klk1	16612	7+:51480848-51484938
3948	V1rb4	113854	6+:89843267-89844199
3952	Bcar1	12927	8-:114234650-114267647
3955	Neurod1	18012	2-:79292678-79296793
3956	Psat1	107272	19-:15980536-15999413
3957	Dennd1b	329260	1-:141030362-141031320
3960	MMT00007654		2+:117066618-117068004
3964	Colq	382864	14-:32337492-32390504
3965	Pwp2	110816	10-:77633654-77647874
3967	Tysnd1	71767	10+:61158261-61165516
3970	4931414P19Rik	74359	14-:55202500-55224741
3973	Dmtf1	23857	5-:9119662-9161724
3980	Gkap1	56278	13-:58334711-58365534
3983	4930542C12Rik	67648	9-:78242762-78243965
3985	Gjb3	14620	4-:127002480-127006405
3986	Ep400	75560	5-:111093125-111199611
3987	MMT00005386		11-:32780362-32780990
3988	4933427G17Rik	74466	7+:128126021-128158300
3989	Wrnip1	78903	13+:32894049-32914477
3991	Hoxb9	15417	11+:96132821-96137905
3998	4930528F23Rik	75178	17+:24955221-24976731
3999	Nr3c1	14815	18-:39573930-39646901
4001	Apoa4	11808	9+:46049236-46051372
4004	Thbs2	21826	17-:14804160-14831097
4005	Ccdc19	71870	1+:174451267-174476002

4008	Gnl3l	237107	X-:147419697-147451866
4009	Cotl1	72042	8-:122333124-122364440
4010	Alkbh2	231642	5-:114573944-114578157
4012	Ctsw	13041	19-:5465341-5468483
4014	Dock1	330662	7+:142039811-142067998
4022	Gdpd5	233552	7+:106535613-106610356
4029	Samsn1	67742	16-:75859504-75909469
4030	Prps1	19139	X+:136991211-137010677
4032	MMT00008594		13-:109010657-109011035
4033	Mgst2	211666	3+:51465110-51486596
4034	Slc6a13	14412	6+:121250313-121287735
4038	1200009O22Rik	66873	6-:53767162-53770477
4039	Klc3	232943	7-:19979789-19985205
4046	Phb	18673	11+:95529348-95541341
4047	Nbeal1	269198	1+:60238432-60292289
4048	BC002230	217827	12-:101363660-101397843
4049	MMT00053760		7-:135159956-135160734
4053	Pcdha4	12936	18+:37112342-37347311
4056	Tnfaip8l1	66443	17+:56301952-56312440
4057	Slc2a4	20528	11-:69756044-69761636
4059	MMT00048362		X+:87308393-87309159
4061	Apcs	20219	1-:174824092-174825172
4065	Igsf5	72058	16+:96583400-96625635
4066	Actg2	11468	6-:83462903-83486219
4070	Chst10	98388	1-:38920721-38954837
4072	Adcy2	210044	13-:68758930-69138158
4074	Ush2a	22283	1+:190686251-190768687
4076	Larp5	217980	13+:9093165-9173650
4079	Emilin3	280635	2-:160731240-160738052
4080	Atp2a1	11937	7-:133589373-133591457
4084	Asah3l	230379	4+:86520367-86566778
4085	Cd302	66205	2-:60090049-60122475
4086	Adamts8	30806	9+:30750146-30770441
4088	Pdcl2	79455	5-:76741291-76760158
4089	Socs6	54607	18-:89038345-89056679
4090	Orai3	269999	7+:134913355-134918664
4093	Akp5	11650	1-:88983623-88986338
4095	Trub2	227682	2-:29631630-29643170
4098	Il2ra	16184	2+:11564614-11611409
4100	Pla2g3	237625	11+:3388354-3393064
4105	Speer5-ps1	70365	10+:43891440-43939389
4106	MMT00041409		4-:73158398-73159655
4107	MMT00068618		6+:126721939-126724832
4108	Apobec2	11811	17-:48558555-48572044
4109	Abpb	233099	7-:34797102-34798961
4114	Parp12	243771	6-:39036411-39068158

4116	Ifi205	226695	1-:175664569-175962188
4119	Magea7	17143	X+:73592732-73592974
4120	Mrpl3	94062	9+:104955628-104979693
4122	Ehbp1l1	114601	19-:5708051-5726270
4124	1110037F02Rik	66185	4+:11413147-11542956
4126	Vti1a	53611	19+:55390850-55701007
4132	Hrc	15464	7+:52590770-52594340
4133	Cbr3	109857	16+:93683459-93691231
4137	Pof1b	69693	X-:109753196-109812262
4138	Cited4	56222	4+:120339205-120340423
4139	Sh2d5	230863	4+:137806379-137815136
4141	Pank4	269614	4+:154338254-154355046
4143	Zfp286	192651	11-:62593107-62597268
4146	Cml1	66116	6-:85860150-85865655
4152	Lrrc33	224109	16-:32142908-32165572
4158	4933400N17Rik	619289	1-:39722152-39727434
4163	5830415L20Rik	68152	5+:3543835-3561044
4165	Cux1	13047	5-:136748771-136849218
4166	Cblc	80794	7-:20366318-20382131
4171	1700023F02Rik	69433	10-:65583359-65584736
4173	MMT00008170		13-:47104637-47106562
4174	Unc13b	22249	4+:43071840-43277742
4181	Gzf1	74533	2+:148506915-148518686
4182	Foxd3	15221	4+:99322989-99325313
4185	Hmgb2	97165	8+:59991854-59993062
4186	Car7	12354	8+:107064683-107074245
4188	Sult1a1	20887	7-:133816384-133819871
4189	Epb4.1l1	13821	2+:156246836-156366015
4191	Fbln2	14115	6+:91162857-91222533
4192	Zranb1	360216	7+:140141290-140178050
4194	Dnttip2	99480	3+:121977323-121988194
4195	Csrp2bp	228714	2+:144194758-144233390
4196	Atg16l2	73683	7-:108438734-108450613
4198	Ghr	14600	15-:3267776-3533231
4199	B3galt5	93961	16+:96536775-96537701
4201	Tubb2c	227613	2-:25077681-25080161
4203	Fermt2	218952	14-:46078471-46087081
4205	Rgs1	50778	1-:146091799-146096165
4206	Nipsnap1	18082	11+:4774005-4794203
4214	MMT00014098		13+:34653871-34654238
4220	EG629065	629065	17-:53840190-53842391
4223	Dnahc7l	207778	14+:27516798-27704888
4229	Pth	19226	7-:120529091-120532086
4232	B020011L13Rik	547097	1+:119637961-120026240
4233	Brpf1	78783	6+:113257220-113274854
4235	Pfkfb3	170768	2-:11393452-11423652

4245	Ulk4	209012	9-:120873567-121186321
4246	Fbxw12	434440	9-:109125786-109127028
4247	Sfpq	71514	4+:126697954-126713103
4258	Rpl5	19983	5+:108329587-108345390
4261	MMT00014970		12+:89573046-89574124
4266	Fn1	14268	1-:71632090-71699746
4272	Hsd17b6	27400	10-:127428006-127437732
4275	EG331392	331392	X+:20938065-20940149
4276	Chmp4b	75608	2+:154482975-154520518
4282	RP23-100C5.8	241638	2-:130460502-130468535
4284	Irf1	16362	11+:53583579-53590825
4285	Dnajc19	67713	3-:33976206-33980243
4286	Ptp4a1	19243	1-:30998436-31006590
4289	MMT00057248		X+:16187190-16188720
4291	Impa1	55980	3-:10315185-10329005
4298	St3gal1	20442	15-:66937849-66945371
4301	AW049604	106014	15+:87455659-87589792
4302	Vmn2r120	224916	17-:57648206-57665382
4303	Phkg1	18682	5-:130339308-130354960
4305	Tmem51	214359	4-:141586907-141593849
4306	Cpeb1	12877	7-:88492329-88599562
4308	Nphp3	74025	9+:103917654-103918975
4310	B3gnt8	232984	7+:26412656-26414509
4311	MMT00033987		4-:40808262-40860214
4313	Ppap2b	67916	4+:104829964-104903745
4319	1810009O10Rik	69109	11-:78564413-78565164
4321	MMT00066182		5+:52972590-52975039
4325	Slc16a4	229699	3+:107094209-107115026
4327	Klf10	21847	15-:38224168-38230462
4328	Spag11	78128	8+:19141376-19159577
4332	Sf3b1	81898	1-:55042015-55084323
4333	Sumo3	20610	10+:77069014-77080121
4336	Rbbp9	26450	2-:144369557-144376514
4337	Myst3	244349	8+:23971985-24053489
4338	EG328280	328280	13+:67884730-67944975
4340	Sdk2	237979	11-:113641489-113731229
4344	1810073G14Rik	67726	11-:57296501-57332117
4345	MMT00074600		8-:41939180-41940429
4346	Ccdc46	76380	11+:108543937-108721925
4348	Nedd9	18003	13-:41406940-41582665
4349	Mrpl27	94064	11+:94515109-94521209
4351	Plxnb1	235611	9+:109002591-109022421
4352	Rtn4rl1	237847	11+:75078258-75081263
4355	Pard6a	56513	8+:108225582-108227366
4359	Tmem208	66320	8+:107850282-107852955
4363	2810002I04Rik	72350	12+:86629575-86638361

4365	Tapbp	21356	17+:34056632-34063651
4369	Acot4	171282	12+:85379460-85384746
4375	Cysltr2	70086	14-:73428934-73448879
4376	2310015A05Rik	72315	16+:17646642-17650830
4378	1700026D08Rik	75556	7-:90924128-90943336
4379	Glrx	93692	13+:75977354-75987416
4380	Smarcb1	20587	10-:75359519-75384333
4384	Gamt	14431	10-:79720943-79723757
4385	Sfrp4	20379	13+:19715051-19724690
4386	Sptlc1	268656	13-:53428120-53472729
4387	Frs3	107971	17+:47832155-47841235
4388	MMT00019672		X-:96276741-96277655
4391	Zfp467	68910	6-:48387584-48395606
4392	1810055G02Rik	72056	19+:3708332-3717881
4400	9030425E11Rik	71566	9+:40494129-40591188
4404	Commd5	66398	15+:76730370-76731727
4407	4930429B21Rik	67576	3+:32264696-32266358
4409	5430435G22Rik	226421	1+:133585301-133610044
4411	2310039H08Rik	67101	17+:46909760-46910344
4413	MMT00030347		5-:12354694-12355404
4414	5033406O09Rik	77675	12-:113180557-113182419
4415	B230399E16Rik	240479	18+:84889670-84906366
4417	Stom	13830	2-:35169510-35192529
4418	H2-Aa	14960	17-:34419804-34424733
4421	Synj1	104015	16-:90936337-91011554
4425	Olfr1346	258918	7+:6426822-6427763
4426	Iigp2	54396	11+:58032970-58036282
4428	Prl8a8	74188	13-:27598940-27605082
4429	MMT00042276		13+:7307045-7308207
4430	Ankrd36	76389	11+:5469687-5484284
4431	Tmem17	103765	11+:22412082-22419236
4433	Snrpa1	68981	7+:73205454-73219468
4443	Zfp790	233056	7+:30601137-30615958
4444	Ksr2	333050	5+:117955497-118005945
4446	Myo5c	208943	9+:75135637-75153255
4454	BC055107	268709	14-:9130066-9150355
4455	Ccdc76	229780	3-:116283647-116297970
4457	Cks2	66197	13+:51740714-51746023
4459	Crybb3	12962	5-:113504860-113509673
4462	Cyp1a1	13076	9+:57545419-57551629
4463	Ncoa5	228869	2-:164825857-164860279
4464	BC028471	414071	5+:150171612-150195061
4466	MMT00014327	233575	7+:109371587-109386263
4468	MMT00052724		
4469	Cyp3a13	13113	5-:138335222-138362823
4470	Slc5a8	216225	10+:88348781-88398828

4471	Sdccag8	76816	1+:178754869-178951013
4472	MMT00074121	100040305	12-:103213872-103215162
4473	EG628040	628040	X-:8903532-8905467
4474	EG627022	627022	6-:136434167-136435708
4480	Bahcc1	268515	11+:120135297-120151196
4481	Olfr1100	258587	2-:86817987-86818951
4484	MMT00014015		13+:61767510-61768501
4487	Gdpd2	71584	X+:97925339-97934238
4492	Suv420h1	225888	19+:3768145-3805463
4493	Ptgir	19222	7+:17491838-17496243
4495	Nid1	18073	13+:13529957-13604521
4499	Enpp2	18606	15-:54670455-54751557
4500	Usp53	99526	3-:122655472-122657473
4501	MMT00033071		
4504	1700113I22Rik	73635	11-:101280127-101286642
4506	Zc3h7b	20286	15+:81598389-81626700
4508	Ifit1	15957	19+:34715403-34723970
4510	Ttf1	22130	2+:28915796-28942613
4512	Esrra	26379	19-:6985467-6996256
4514	MMT00045906		5-:117799052-117799379
4516	Rsad2	58185	12-:27130172-27141293
4517	Itih3	16426	14-:31721761-31736728
4518	Gpr65	14744	12+:99506866-99514842
4523	Ptpru	19273	4-:131324372-131394193
4524	Usp12	22217	5-:147546398-147606583
4526	Satb1	20230	17-:51878793-51971958
4531	EG434175	434175	7-:49361153-49362629
4532	BC034090	207792	1-:157059602-157089407
4533	Itgb1	16412	8+:131208845-131256651
4534	Ndel1	83431	11-:68634950-68666555
4535	MMT00025220		7-:94846328-94847249
4536	BC013529	215751	10-:7487753-7500703
4537	Olfr44	18343	9-:39291891-39292653
4540	Prl8a2	13529	13+:27437557-27446073
4549	9930012K11Rik	268759	14-:70554213-70556326
4550	Stard10	56018	7+:108465632-108494825
4551	Cyp2e1	13106	7+:147949637-147960874
4553	Xpnpep3	321003	15+:81230579-81283678
4555	Cchcr1	240084	17+:35654120-35667950
4557	MMT00031263		12-:52234347-52235904
4558	Alg3	208624	16-:20605545-20610817
4560	1700011F14Rik	75645	19+:58586480-58627575
4561	Itgbl1	223272	14+:124059394-124373299
4565	MMT00019450		7+:54604843-54605630
4566	MMT00078134	547323	14+:53511660-53512863
4573	Nrxn1	18189	17-:91391454-91488944

4574	MMT00064324		18+:14583369-14650895
4576	Chchd7	66433	4+:3866034-3870674
4577	Ltbp4	108075	7-:28056959-28104789
4578	Pglyrp2	57757	17-:32550305-32557920
4579	Dpys	64705	15-:39614243-39689003
4580	Impact	16210	18+:13130760-13151457
4582	Armcx4	102910	X+:131221039-131231292
4583	Krtap14	23927	16-:88825535-88826375
4584	MMT00022168		13+:18041731-18042930
4586	Zfp618	72701	4+:62694927-62794905
4590	Arhgap21	71435	2-:20769558-20793395
4597	MMT00051225		10+:3820035-3820664
4601	Th	21823	7-:150078680-150085871
4602	Pdzrn3	55983	6-:101099608-101327891
4603	MMT00009680		14+:29538056-29538230
4605	Myo1f	17916	17+:33692734-33744703
4607	Acad11	102632	9+:103966014-104029975
4608	Lag3	16768	6-:124854378-124861723
4612	Hspa5	14828	2+:34627749-34631981
4617	Agpat9	231510	5+:101275149-101328115
4619	Htra3	78558	5-:35994690-36022214
4625	Cwc15	66070	9+:14305171-14314988
4626	2310046K01Rik	69698	2+:151822363-151834992
4628	MMT00040865		2+:150017591-150151534
4630	Akr1c20	116852	13-:4506404-4522579
4631	Itgb2	16414	10+:77004727-77028128
4633	Olfr125	258287	17+:37971945-37972906
4635	MMT00023332		15+:100127548-100128056
4638	Alkbh4	72041	5+:136612024-136617482
4642	Dact3	629378	7+:17460773-17472812
4643	Mbl2	17195	19+:30307446-30314171
4645	MMT00030322		
4648	1700007G11Rik	75784	5+:99127420-99231068
4650	RP23-438H3.2	69357	X+:90428631-90429280
4654	Myl9	98932	2+:156601152-156607393
4655	1810029B16Rik	66282	8-:69000244-69010404
4657	C230096C10Rik	230866	4+:138918044-138931841
4664	MMT00079290		13+:108138207-108138595
4665	Dynll1	56455	5-:115748543-115750933
4666	Exosc9	50911	3+:36451564-36464647
4669	H2-M10.5	224761	17+:36870567-36871698
4670	8430426H19Rik	71508	13-:62504184-62706778
4671	Itga7	16404	10+:128370873-128395338
4674	Cxcl12	20315	6+:117118595-117131384
4675	Sparcl1	13602	5-:104508131-104542705
4676	Cenph	26886	13-:101529640-101545854

4677	BC016423	105203	13-:3565281-3574438
4678	Kank1	107351	19+:25311462-25508987
4682	Sertad4	214791	1-:194670625-194683097
4683	E330013P04Rik	107376	19+:60220559-60236173
4684	MMT00006152	268859	16+:5885447-7410072
4686	BC021785	215928	10-:39666719-39706481
4687	Gtpbp8	66067	16-:44739098-44746472
4692	MMT00049231		1+:10858784-10859392
4694	MMT00046622		7-:113465907-113471636
4695	D330027H18Rik	101359	6-:29121509-29127222
4697	Cyp2a12	13085	7+:27814108-27821829
4701	MMT00062346	236260	12+:17888729-17945713
4702	Tmem39b	230770	4-:129353600-129374040
4707	Hsd3b5	15496	3-:98383764-98385715
4708	Traf3ip2	103213	10+:39359186-39374629
4709	Prune2	77754	19+:17030605-17129452
4715	Tmem120a	215210	5-:136173097-136220096
4717	Cd177	68891	7-:25539571-25545331
4719	Col2a1	12824	15-:97806472-97834924
4720	Ckm	12715	7+:19996442-20006932
4723	Art3	109979	5+:92760842-92843656
4729	Slc7a10	53896	7+:35971489-35985965
4731	Zmynd12	332934	4+:119095435-119126500
4732	Idh3a	67834	9+:54434296-54451596
4734	Ccbl2	229905	3+:142364039-142407498
4737	Olfr449	259067	6+:42787881-42788817
4738	Ppm1a	19042	12+:73884668-73894849
4741	MMT00070324	100041422	14+:44701632-44704860
4745	Lhfp	108927	3+:53035649-53065600
4746	1810007M14Rik	67367	16-:91015187-91034444
4748	Xylt2	217119	11-:94525166-94531854
4749	9230107M04Rik	77684	7-:14140214-14156419
4752	Pik3r4	75669	9+:105546566-105589978
4767	Ktn1	16709	14+:48283453-48356232
4768	Lsm10	116748	4+:125773995-125775823
4772	2810007J24Rik	76971	7-:14996067-15032119
4774	Ccdc112	240261	18-:46445266-46471548
4776	Dclk1	13175	3+:55046549-55340599
4778	Cyct	13067	2-:76192086-76198505
4780	Sfrs5	20384	12+:82047562-82051486
4784	Slc2a2	20526	3+:28596898-28627249
4785	ORF63	224419	16+:87553574-87595580
4787	Parn	74108	16-:13538056-13668261
4788	Pak3	18481	X+:140100287-140226353
4790	Nrg3	18183	14-:40278445-40340075
4792	Neurod6	11922	6-:55627816-55631063

4793	Bbs1	52028	19-:4888746-4906638
4794	Gsdmd	69146	15+:75692768-75697834
4796	Rcbtb2	105670	14+:73554161-73582489
4797	Fbxo25	66822	8+:13907805-13940521
4801	MMT00057721		18+:23375130-23376062
4802	5430433G21Rik	319909	2+:139504507-139584294
4805	Hspb1	15507	5+:136363928-136365307
4807	Fgl2	14190	5+:20878499-20882878
4810	Vsig8	240916	1+:174486202-174493847
4811	Gripap1	54645	X+:7367142-7397687
4812	Myom1	17929	17+:71368860-71476195
4813	Agpat6	102247	8-:24284988-24293313
4815	Upf1	19704	8-:72855428-72877178
4817	MMT00046203		
4818	Enpep	13809	3-:128972537-129035486
4819	MMT00082285		14+:19363856-19365450
4825	Nfkbil1	18038	17-:35357120-35372760
4826	Sypl2	17306	3-:108015183-108029517
4832	Nxnl2	75124	13+:51266393-51270556
4833	Synpo2	118449	3-:122815319-122817593
4836	Gtf2h3	209357	5+:125029100-125046849
4837	Ear2	13587	14+:44680551-44681022
4839	Cyp4a32	100040843	4+:115236254-115294212
4843	Oma1	67013	4+:102986450-103039033
4847	Sbpl	638345	17-:24090051-24091883
4848	Flt3	14255	5-:148142321-148211871
4849	Zxdc	80292	6+:90319527-90333086
4850	Fancd2	211651	6+:113481695-113547011
4851	Gpn2	100210	4+:133140294-133147646
4855	Csrnp3	77771	2+:65683869-65716875
4863	Atad4	217138	11+:96790637-96796959
4864	S100a16	67860	3+:90341297-90347070
4868	Mertk	17289	2+:128524732-128627924
4870	Olfr550	259108	7+:109727010-109727959
4879	Olfr784	258724	10+:128824690-128825650
4881	4930434E21Rik	381693	5+:150408863-150414465
4885	Fhad1	329977	4-:141484294-141507592
4886	Jarid1a	214899	6+:120314475-120369459
4896	4930405M20Rik	73942	13-:93219577-93254049
4897	Ciao1	26371	2-:127068300-127073496
4898	MMT00072808		14-:114708037-114710025
4899	Nr4a2	18227	2-:56960568-56967666
4900	5031426D15Rik	68144	2-:6845969-6849768
4901	MMT00073557	171504	7+:133728521-133732598
4904	Tinf2	28113	14-:56297933-56300645
4906	A830059I20Rik	58238	7+:100228428-100230228

4910	5530400C23Rik	232426	6+:133242234-133245807
4913	Zfp619	70227	7+:46753549-46822953
4915	Asb18	208372	1-:91849252-91911152
4918	Scara5	71145	14+:66285263-66383660
4920	Rbl1	19650	2-:156971623-157025312
4932	Cd70	21948	17-:57285420-57289200
4933	Clec14a	66864	12-:59366922-59369883
4934	Abca13	268379	11+:9235231-9528671
4936	MMT00031931		18-:80072327-80073065
4938	Mccc2	78038	13-:100718483-100785567
4939	1700023B02Rik	66935	2-:73121928-73150560
4949	Taok2	381921	7-:134009189-134024277
4950	Dnajb10	56812	1+:75233130-75242265
4955	Cxcl1	14825	5+:91320335-91322141
4958	Ndufa7	66416	17+:33961574-33975133
4959	Optn	71648	2-:4941686-4963566
4960	MMT00067115		17-:18048003-18049853
4965	Kl	16591	5+:151755273-151796386
4966	Zscan20	269585	4-:128262620-128281844
4967	Plekhm1	353047	11-:103241432-103274027
4971	Hist1h4i	319158	13-:22129747-22133192
4973	Grik4	110637	9-:42616574-42616709
4980	MMT00006445		6-:68966533-68993274
4982	4930449A18Rik	74672	3+:59642287-59650737
4983	Rabgef1	56715	5+:130647717-130690211
4988	2400001E08Rik	66508	7+:109054442-109060398
4993	Tsc22d1	21807	14-:76906439-76907570
4996	Echdc3	67856	2-:6109865-6134021
4997	Trim24	21848	6+:37820630-37916287
5002	Ifi202b	26388	1-:175892745-175912845
5003	2410081M15Rik	73680	4-:129030875-129055272
5010	Gnpda2	67980	5-:69964348-69982030
5011	A930016P21Rik	68073	15+:31531872-31547290
5013	Fancl	67030	11+:26287201-26371876
5014	MMT00017503		1+:7654291-7655057
5017	Plat	18791	8+:23868239-23893316
5018	Phf21a	192285	2+:92024494-92201050
5021	Gdap2	14547	3+:99966440-100010904
5023	Spast	50850	17+:74751284-74790540
5024	MMT00020852		10+:128052418-128053090
5027	Pde4b	18578	4+:101927607-102278428
5029	1110034G24Rik	73747	2+:132512673-132576943
5032	Gm872	380653	10-:92238409-92278681
5033	MMT00012520		X-:129422743-129423698
5034	EG621976	621976	13+:41701584-41730291
5036	C030003D03Rik	77220	10-:25712699-25798168

5043	Cxcl13	55985	5+:96385957-96390086
5044	Ifi35	70110	11+:101309794-101319776
5046	Pthlh	19227	6-:147200621-147212723
5048	Mgll	23945	6+:88674477-88778351
5051	Angpt1	11600	15-:42258288-42508313
5052	Lrrn3	16981	12-:42178781-42216157
5053	BC002199	211556	3-:127510184-127511782
5054	Zfp821	75871	8+:112230251-112248831
5060	MMT00066059		4+:149583796-149602550
5061	Myo9a	270163	9+:59598979-59773196
5063	MMT00046201		2-:78353035-78353496
5064	Tekt2	24084	4-:125999366-126002443
5068	Afap1l2	226250	19-:56986849-57082667
5071	Aff2	14266	X+:66613505-67121211
5072	MMT00052662		15+:48720103-48721563
5076	Tmem57	66146	4-:134358671-134408862
5082	Ibsp	15891	5+:104728315-104740484
5083	Gprc5b	64297	7-:126115043-126128159
5084	Gsn	227753	2+:35137961-35163414
5086	Ccnh	66671	13+:85329041-85352512
5092	Ydjc	69101	16+:17144711-17148938
5094	Yes1	22612	5+:32913605-32989429
5098	1700029J11Rik	76426	2+:172298044-172299791
5099	MMT00056385		
5100	Pebp1	23980	5-:117732662-117737555
5101	Gm101	226356	1-:121819919-121832748
5103	EG237361	237361	10+:60772577-60773022
5104	1700094C09Rik	78634	8+:12573048-12600738
5106	Usp38	74841	8-:83511469-83539565
5107	Gprc5c	70355	11+:114713368-114730409
5108	2200002D01Rik	72275	7-:30032521-30033492
5109	Cyp3a11	13112	5-:146666541-146691426
5113	Nxnl1	234404	8-:74084454-74090500
5114	Fbxo33	70611	12-:60301643-60320437
5118	Hmcn1	545370	1-:152409635-152707507
5120	Alkbh7	66400	17+:57136775-57138745
5123	Zfp191	59057	18-:24172660-24176588
5125	Fmod	14264	1+:135934119-135944736
5126	Fcnb	14134	2-:27931897-27940377
5127	Kif20b	240641	19+:34996848-35050241
5132	1600016N20Rik	72000	7-:148395760-148396896
5133	6430517E21Rik	240843	1-:160175400-160225908
5135	Pisd	320951	5+:33069248-33079766
5142	Eppk1	223650	15-:75949949-75950994
5145	Scn3b	235281	9+:40077125-40096767
5147	5730455P16Rik	70591	11-:80173994-80191506

5149	Sirt3	64384	7-:148049572-148068208
5152	Cdh10	320873	15+:18748679-18943559
5153	MMT00072251		4-:101719173-101722109
5156	Srm	20810	4+:147965655-147968701
5158	Rtn4	68585	11+:29618782-29642086
5159	MMT00020962		7-:80133985-80135027
5161	Tbc1d1	57915	5+:64726514-64736671
5169	MMT00037010		
5172	Sumo1	22218	1-:59696287-59727653
5173	Azgp1	12007	5+:138422787-138431458
5175	MMT00032221		
5180	E130304I02Rik	78547	7+:36587185-36623093
5181	4930481B07Rik	74937	3+:94819035-94820196
5182	Pcx	18563	19+:4594357-4621697
5183	St5	76954	7-:116667426-116760080
5185	Dus2l	66369	8+:108535394-108577739
5187	Speer4f	70935	5+:16981939-16986754
5188	Cma1	17228	14-:56560289-56563498
5200	Tm6sf1	107769	7+:89004016-89028952
5205	MMT00065634		10+:62137891-62165263
5208	Plod2	26432	9+:92437289-92503258
5211	Fubp3	320267	2+:31428259-31473015
5215	Col6a1	12833	10-:76171536-76188789
5216	Spnb5	640524	2-:119872115-119911478
5218	ENSMUSG00000046(	194612	17+:22825894-22884251
5220	MMT00037083		12+:55817773-55818252
5222	B630005N14Rik	101148	6-:13575937-13627833
5224	MMT00071383		10-:68154349-68154847
5225	Maf1	68877	15+:76182432-76184802
5227	2310008M10Rik	66357	3-:130399068-130412335
5228	Actr3b	242894	5+:25265902-25355764
5229	Katna1	23924	10+:7445819-7482948
5230	MMT00077995		6-:117423016-117423443
5232	Arhgef16	230972	4-:153652595-153662123
5233	Pcbd1	13180	10+:60552108-60557067
5234	Lhx8	16875	3-:153969261-153993364
5237	Cpa1	109697	6+:30592297-30595360
5238	Il12b	16160	11+:44213564-44227512
5242	Gata3	14462	2-:9779481-9799424
5244	MMT00022648		X+:75636043-75636562
5245	Cacna1h	58226	17-:25511294-25570113
5247	Irak2	108960	6+:113588493-113645003
5249	Olfr615	259084	7+:110708992-110709933
5250	Ifit2	15958	19+:34644804-34651013
5252	Mfsd3	69572	15+:76531894-76533998
5255	Zkscan16	100041581	4+:58959022-58970725

5256	Tnfrsf13c	72049	15-:82052173-82054764
5258	Mcts2	66405	2+:152512883-152513682
5259	Mat1a	11720	14+:41918669-41937699
5262	Mecp2	17257	X-:71272168-71330932
5263	Anapc13	69010	9+:102532119-102536468
5265	A3galt2	215493	4+:128438662-128444916
5270	Rnf8	58230	17+:29751806-29778603
5271	Npy	109648	6+:49773596-49779502
5272	Grk4	14772	5+:35003601-35097952
5273	Vmn2r89	22301	14+:52071636-52080967
5274	MMT00065196		2-:134587804-134589386
5283	B3galt1	26877	2+:67955999-67956980
5284	Npffr1	237362	10+:61076703-61089132
5285	Tmc8	217356	11+:117643432-117645506
5291	Chl1	12661	6+:103460996-103679821
5294	C730048C13Rik	319800	19-:8409849-8479572
5295	F2r	14062	13-:96371743-96388388
5296	Rnf181	66510	6-:72309714-72312387
5298	Sbno1	243272	5-:124842977-124875577
5303	Ndufv2	72900	17-:66428550-66450866
5306	Ywhag	22628	5-:136386681-136410446
5313	9630058J23Rik	226744	1+:181536593-181557605
5319	Hydin	244653	8+:113110933-113134145
5320	MMT00007643		8+:23517938-23518974
5322	Slpi	20568	2-:164179806-164198446
5325	C230097I24Rik	328918	18-:24122539-24130290
5328	MMT00020895		
5330	Cbfa2t3	12398	8-:125153890-125222877
5332	Pdlim4	30794	11-:53868433-53882470
5334	MMT00021505		13-:109098648-109100938
5336	Timeless	21853	10+:127675120-127689988
5339	4930488E11Rik	399591	X-:133491272-133511389
5340	MMT00048531		X+:144181262-144199155
5342	Eps8l3	99662	3+:107680154-107695817
5345	MMT00023326	57435	17-:56240016-56249226
5352	Pdcd2l	68079	7-:34969515-34981692
5355	Gpsm2	76123	3-:108482007-108525087
5357	MMT00021917		2-:174979623-174979793
5360	Cep76	225659	18-:67777455-67800854
5362	Nans	94181	4+:46502153-46515545
5364	Kif22	110033	7-:134171245-134185934
5365	C87436	232196	6+:86388681-86420379
5369	Rag1	19373	2-:101478438-101489658
5370	Crh	12918	3-:19593437-19595485
5372	Tor1a	30931	2-:30816147-30823404
5374	Chka	12660	19+:3851913-3894367

5375	Sec23b	27054	2+:144384956-144416109
5377	MMT00077700		X+:161986982-162118532
5380	Olfr1403	258645	1+:175200515-175201240
5382	Fbn1	14118	2-:125126331-125332121
5385	Tubb2b	73710	13-:34218876-34222223
5389	Abce1	24015	8-:82207543-82235514
5406	Cd207	246278	6-:83621677-83627793
5410	Cyp4f15	106648	17+:32822603-32840294
5413	Flot1	14251	17+:35960301-35969732
5414	Etnk2	214253	1+:135260359-135276200
5416	Jhdm1d	338523	6-:39086652-39088048
5417	Ssb	20823	2+:69699689-69709856
5420	Mbnl1	56758	3+:60333070-60433302
5422	4933422A05Rik	71133	9+:35090119-35093008
5425	Ung	22256	5+:114581208-114589330
5431	Fkbp6	94244	5-:135767574-135825861
5433	2410004L22Rik	76478	8-:73775023-73796275
5434	Inpp5b	16330	4+:124428773-124478680
5435	Lins2	72635	7+:73834840-73859630
5436	Prl7a1	19113	13-:27725247-27734343
5438	Slc25a15	18408	8-:23487758-23508932
5440	1810020G14Rik	66272	12-:82572811-82586069
5443	Nap1l2	17954	X-:100379514-100381979
5445	1700003E16Rik	71837	6+:83106456-83112936
5449	Got1	14718	19-:43574244-43598999
5450	Nrbp1	192292	5+:31543290-31553933
5452	Mogat2	233549	7-:106367595-106387078
5453	Odf3l1	382075	9-:56696466-56699583
5455	Kcnab3	16499	11+:69143825-69145645
5456	Rabgap1l	29809	1-:162153666-162187675
5463	Krt14	16664	11-:100064477-100068824
5465	Slamf8	74748	1-:174511890-174520620
5466	2310047D13Rik	116972	11+:76014676-76022918
5468	MMT00053630		10+:40268705-40269545
5472	Mppe1	225651	18-:67384702-67405479
5475	Akr1c18	105349	13-:4131872-4149877
5477	2210412D01Rik	70178	7-:91257869-91300403
5482	Cdsn	386463	17+:35689120-35693858
5483	Gpc5	103978	14+:115768753-115798511
5487	Palmd	114301	3-:116621185-116671667
5488	C920009B18Rik	606736	10+:22026526-22034002
5492	MMT00039914		13+:4247980-4249023
5494	Gpr101	245424	X-:54753277-54754965
5504	Itsn1	16443	16+:91782437-91912254
5505	Thap2	66816	10-:114808894-114821470
5508	Lrig2	269473	3-:104261050-104315822

5511	BC055004	381680	5+:138680464-138693315
5517	Stx17	67727	4+:48137801-48195989
5518	Jmjd1c	108829	10+:66590005-66719072
5522	Pink1	68943	4-:137869319-137882193
5523	Taok1	216965	11-:77343564-77344872
5524	Klhl7	52323	5+:23606429-23666603
5525	Prkca	18750	11-:107799561-108205242
5526	Ush1c	72088	7-:53450726-53493860
5527	Zfp398	272347	6+:47785665-47818255
5528	Rfx1	19724	8+:86590827-86619942
5530	MMT00027303		16+:21896562-21897207
5531	Ptk6	20459	2-:180929828-180937494
5536	5830415F09Rik	74753	4-:46389379-46402540
5539	Eif2s2	67204	2-:154697153-154718518
5541	Bcap29	12033	12-:32280260-32319245
5544	Camsap1	227634	2-:25782493-25838494
5548	Fgf6	14177	6+:126965603-126977447
5552	Rasgrp3	240168	17+:75895692-75927270
5553	MMT00013152		12-:72369920-72371344
5554	Emr1	13733	17+:57498112-57622948
5555	Fbln7	70370	2+:128703086-128722768
5556	Ccl4	20303	11+:83476088-83478182
5558	V1ra9	113851	6+:90219099-90220045
5563	Slco1a4	28250	6-:141753960-141804692
5566	2610002J02Rik	67513	4+:154623908-154630799
5567	Mapk8ip1	19099	2-:92224326-92241310
5569	Pdgfc	54635	3+:80841161-81017954
5572	Opalin	226115	19-:41134027-41151655
5574	Hsd3b7	101502	7+:134944182-134947312
5577	MMT00009628		11+:117736358-117825335
5578	Phactr1	218194	13+:43051984-43230728
5583	Igh	111507	12-:115008053-115008508
5586	OTTMUSG000000012	433719	4-:72512345-73084353
5587	Rmnd5b	66089	11-:51436993-51449499
5588	Snx20	71607	8-:91150726-91160027
5591	Igsf8	140559	1+:174242069-174249971
5594	Erbb3	13867	10-:128004583-128026677
5597	Ttll4	67534	1+:74725565-74744545
5598	Trp73	22062	4-:153430356-153516627
5601	4933417O08Rik	71146	19+:42322061-42343631
5603	BC020535	228788	2+:152891690-152907334
5609	Gtf2a2	235459	9+:69860390-69870268
5610	Syngap1	240057	17+:27081577-27107590
5611	Rapgef3	223864	15-:97575386-97598097
5613	Mrpl12	56282	11+:120345982-120350067
5614	Cope	59042	8+:72826683-72836889

5615	Sertad2	58172	11+:20443336-20549114
5619	Rap1gds1	229877	3-:138588876-138738034
5629	Als2	74018	1-:59219775-59294061
5632	Reck	53614	4+:43950178-43957675
5634	Creb1	12912	1+:64579467-64644851
5636	Trmu	72026	15+:85709756-85727820
5639	Gdpd3	68616	7+:133910686-133919157
5642	Crebzf	233490	7+:97592081-97593285
5644	Spsb2	14794	6+:124758958-124760632
5645	Tmem8	60455	17+:26250261-26260196
5646	Kifc1	16580	17+:27054159-27069227
5648	Zfp213	449521	17-:23693735-23701168
5652	Adam26a	13525	8-:44653631-44662020
5656	4933434I20Rik	67555	8+:85872369-85903301
5657	Arpc1b	11867	5+:145883424-145887638
5661	Lrrc46	69297	11-:96895911-96902658
5664	Fez2	225020	17-:78777222-78817426
5666	Vegfb	22340	19-:7056961-7062093
5669	Fndc1	68655	17-:7931447-7966670
5670	EG625253	625253	X+:153978628-153979376
5672	Galntl4	233733	7-:118615168-118759998
5674	MMT00073552		3-:121886794-121887878
5677	Cmas	12764	6+:142705292-142724227
5680	Lrg1	76905	17-:56259102-56261312
5681	Mbd1	17190	18+:74427957-74442339
5684	Ranbp10	74334	8-:108292209-108351095
5685	Gbx2	14472	1-:91824474-91827752
5686	Cys1	12879	12-:25350702-25366678
5687	Klf11	194655	12+:25336389-25347640
5689	Dsg2	13511	18+:20716573-20763024
5691	Csrp3	13009	7-:56085929-56103395
5692	2010005H15Rik	76770	16+:36253090-36257530
5693	Zfp809	235047	9+:22009228-22047796
5694	Sp7	170574	15-:102187040-102196764
5700	Tenc1	209039	15+:101933517-101946833
5706	Cyp1a2	13077	9-:57525035-57530336
5707	MMT00017539		4-:136165086-136165774
5715	ENSMUSG0000052(	433237	19+:29141789-29143990
5718	Tspan2	70747	3+:102538889-102575154
5721	Mlc1	170790	15-:88786314-88809437
5725	2-Mar	224703	17-:33828474-33855582
5727	Camk2g	12325	14-:21554097-21611844
5728	MMT00044077		18-:47853154-47895055
5729	2310016C08Rik	69573	6+:29222749-29225322
5730	Tasp1	75812	2-:139659214-139736380
5733	B4galnt2	14422	11-:95727335-95776185

5734	EG619653	619653	8-:128999567-129000755
5735	Nkapl	66707	13-:21558967-21560356
5737	Nkx2-2	18088	2-:147008887-147012095
5740	Efemp2	58859	19+:5475020-5481852
5742	Olfr782	257985	10+:128787620-128788563
5745	Cartpt	27220	13-:100668874-100670589
5747	Fbxl16	214931	17+:25953360-25956727
5748	Brsk2	75770	7+:149136256-149184917
5749	Ube2j2	140499	4+:155323115-155331473
5750	2610101N10Rik	67958	9-:95360816-95412304
5752	Dffa	13347	4+:148478359-148492047
5758	Tpst1	22021	5+:130577459-130611598
5760	Prl	19109	13+:27149444-27157051
5761	Ralgps2	78255	1-:158738960-158837895
5762	Trpc3	22065	3-:36519494-36570525
5764	Ltb4dh	67103	4-:58979360-58999973
5765	Ifitm2	80876	7-:148140737-148141860
5766	5033414D02Rik	67759	19-:29423166-29436361
5768	MMT00077590		18-:90480351-90481596
5773	1700129C05Rik	67932	14-:59751876-59761730
5776	Rpa1	68275	11-:75113850-75161806
5780	Col14a1	12818	15+:55139299-55352357
5783	4833413O15Rik	242286	4-:3989766-4004072
5786	Lta	16992	17-:35340107-35342128
5788	Smpdl3b	100340	4-:132288880-132313086
5791	Baiap2l1	66898	5-:145025397-145118981
5792	Tpd52l2	66314	2+:181231905-181250603
5795	MMT00057992		4+:41115654-41116684
5797	MMT00034010		4-:40663118-40664024
5801	MMT00042142		6-:127133424-127174210
5802	Mad2l2	71890	4+:147514598-147519805
5804	Thrsp	21835	7-:104565560-104566013
5806	Prrx2	20204	2+:30700800-30736771
5809	MMT00030928		X-:79067525-79068260
5811	Col4a2	12827	8+:11312890-11449286
5814	Nrbp2	223649	15-:75916024-75920443
5815	Ehf	13661	2-:103106667-103123917
5822	Car2	12349	3+:14886697-14900576
5827	6332401O19Rik	319832	6-:24901146-24906085
5829	2810455K09Rik	72805	12+:112088515-112107287
5831	Sh2b3	16923	5-:122267730-122282573
5833	Kcnk7	16530	19+:5704366-5707100
5837	Alg8	381903	7+:104520046-104540670
5839	Olfr1045	259019	2-:86037961-86038907
5840	Tbc1d9b	76795	11+:49944902-49986279
5841	Atg12	67526	18-:46892080-46901211

5842	Cldn6	54419	17+:23818030-23818689
5843	Anks1b	77531	10+:89973340-90143666
5845	Il18bp	16068	7-:109163590-109166669
5849	2310031A18Rik	69627	8-:127264157-127275506
5850	MMT00051577		14+:46587419-46612844
5853	Trim30	20128	7-:111557541-111613625
5854	Kif11	16551	19+:37450840-37496351
5856	Mfsd2	76574	4-:122623711-122638436
5858	Itch	16396	2+:154959300-155052587
5861	Anxa3	11745	5+:97222433-97274987
5864	C730036D15Rik	209186	4-:49392792-49396448
5866	EG432502	432502	10+:110562915-110563781
5869	MMT00058347		14+:108147699-108148978
5870	Limd1	29806	9+:123387823-123430634
5875	Cd86	12524	16-:36606620-36666079
5876	1600029O15Rik	442832	9+:58055194-58056247
5878	Iqgap2	544963	13-:96397129-96661872
5879	Lmnb1	16906	18+:56867526-56913076
5880	Ppme1	72590	7-:107475245-107503353
5881	Fzd4	14366	7+:96115428-96557763
5883	Olfr1087	258843	2-:86530190-86531130
5884	Gstm2	14863	3-:107784616-107789315
5889	Errfi1	74155	4+:150229199-150242988
5890	Zfx	22764	X-:91319970-91368239
5892	MMT00038457		13+:99383173-99384945
5899	Cpa3	12873	3-:20115521-20142076
5900	Igsf21	230868	4-:139582764-139802286
5902	4930563P21Rik	75275	2+:113125896-113137343
5905	Nos2	18126	11+:78734360-78773626
5908	MMT00027552		13-:79092343-79094053
5910	1110014J01Rik	74778	15-:82946278-82953181
5913	1700007E06Rik	75459	15-:89220593-89240649
5918	Ccl9	20308	11-:83388160-83392112
5919	B930041F14Rik	230991	4+:155042736-155069280
5920	Las1l	76130	X-:93147318-93152187
5921	MMT00026738		9-:72454194-72454753
5922	Hyal2	15587	9+:107471456-107475107
5923	Vav1	22324	17+:57418607-57467126
5925	Tmem59l	67937	8-:73007757-73011236
5929	Cpz	242939	5-:35844865-35868184
5930	Mst1r	19882	9+:107809219-107822712
5934	S100a10	20194	3+:93359085-93368565
5938	Pfn2	18645	3-:57645822-57651410
5941	Ttn	22138	2-:76673993-76674677
5944	Sp3	20687	2-:72774485-72819017
5946	MMT00048659		

5949	Zbp1	58203	2-:173032112-173044391
5953	MMT00008178		5-:28186044-28186980
5954	Lcn13	227627	2+:25555592-25558846
5959	Cdh3	12560	8+:109034779-109080047
5960	EG381065	381065	17+:20707334-20708162
5965	Pbld	68371	10+:62524345-62539576
5966	Cacnb4	12298	2-:52289380-52413917
5967	Nup85	445007	11+:115425773-115430038
5968	MMT00012419		4+:140640935-140641534
5973	MMT00070628		14-:52249951-52254309
5974	Arntl	11865	7+:120351092-120457638
5975	Car13	71934	3+:14641776-14661804
5976	Col6a3	12835	1-:92663435-92740506
5979	Lmo2	16909	2+:103810828-103821251
5980	Gabrb1	14400	5+:72091254-72528483
5982	Rab3gap1	226407	1+:129765370-129840443
5983	Pcolce2	76477	9+:95538046-95595968
5984	Parva	57342	7+:119571451-119732297
5989	2310042D19Rik	74183	4+:155590823-155595417
5990	Rhbg	58176	3-:88047046-88058606
5991	Tsc22d2	72033	3+:58219610-58265759
5994	Olfr609	259086	7-:110640431-110641390
5996	Tmod3	50875	9-:75345588-75407373
5998	Snrpd3	67332	10+:74982079-74998065
5999	RP23-275N21.2	329575	2-:177567215-177575136
6002	Med24	23989	11-:98565914-98590681
6005	Gucy2f	245650	X-:138523396-138631440
6011	Fbxl13	320118	5-:20989666-21029735
6012	Chid1	68038	7-:148681237-148725703
6016	A530057A03Rik	215456	2+:127259321-127261826
6017	MMT00018015	667716	11-:120388168-120393097
6019	Gdap10	14546	12+:33506736-33511764
6020	EG640530	640530	6+:121683047-121734773
6021	MMT00008784		14-:93001678-93003616
6023	Saa4	20211	7-:53984018-53987943
6024	Ddo	70503	10+:40349864-40369234
6026	MMT00058578		X-:151405749-151407971
6028	Alas1	11655	9-:106137717-106150212
6030	As3mt	57344	19+:46781962-46815562
6031	MMT00035612		19+:11566536-11586198
6035	Nkd1	93960	8+:91045333-91116220
6036	Pram1	378460	17+:33766045-33782650
6037	Ccnl2	56036	4+:155195181-155198405
6038	Acot1	26897	12+:85350454-85358617
6041	Penk1	18619	4-:4060684-4065921
6043	Cyp2ab1	224044	16-:20309922-20316132

6044	Sucnr1	84112	3+:59885790-59891481
6048	Psmd9	67151	5+:123678254-123698970
6049	Cpeb2	231207	5+:43626124-43628829
6059	Dsn1	66934	2-:156821000-156832811
6060	Tle6	114606	10-:81053649-81063645
6063	Kif21a	16564	15-:90764813-90880242
6065	Plce1	74055	19+:38695004-38858367
6066	Ppp1r1a	58200	15-:103361353-103368313
6067	Spam1	20690	6+:24745851-24751048
6070	D630003M21Rik	228846	2-:158021486-158046331
6072	C1qc	12262	4-:136445724-136448788
6073	Mthfd2	17768	6-:83255699-83267598
6077	Pik3cb	74769	9-:98938820-99022799
6078	Actrt1	73360	X+:43682183-43683522
6079	Tmem169	271711	1+:72330942-72348101
6085	MMT00007454		12-:64173516-64174101
6086	Fgl1	234199	8-:42276788-42300478
6088	Bambi	68010	18+:3507931-3512756
6091	Rbbp4	19646	4-:128986753-129012583
6092	Phlda3	27280	1+:137662709-137665710
6096	Acot3	171281	12+:85394401-85399975
6098	Ncf4	17972	15+:78075263-78093004
6101	Vmn2r71	233445	7+:92767046-92773055
6103	Cytl1	231162	5+:38126786-38131053
6105	Cryaa	12954	17+:31814883-31818667
6107	Ttc30a2	620631	2-:75814228-75816223
6109	Hsd17b2	15486	8+:120225884-120282774
6111	Irgc1	210145	7-:25216945-25220554
6112	Tmem183a	57439	1-:136242673-136258532
6115	Cd164	53599	10+:41239305-41250847
6116	Pcgf5	76073	19+:36534818-36535452
6117	Atp9a	11981	2-:168459964-168567462
6118	Slc1a4	55963	11-:20202209-20232587
6119	Ankrd24	70615	10+:81103683-81110352
6122	Tktl2	74419	8+:69035690-69037573
6125	Grm6	108072	11+:50664643-50665184
6126	MMT00067575		X-:17251959-17253344
6128	2210418O10Rik	76958	2-:175456017-175468166
6130	G430022H21Rik	210529	3-:123071212-123089017
6133	Agrp	11604	8-:108090655-108091389
6134	Hspb8	80888	5-:116858505-116872873
6135	Nrg1	211323	8-:32928500-33028827
6143	Pacsin2	23970	15-:83206547-83263249
6144	1700055N04Rik	73458	19+:3965901-3970438
6145	MMT00048203		3-:124291508-124292041
6147	Dirc2	224132	16-:35696991-35769403

6154	1700030G06Rik	69507	5-:22229852-22231992
6155	AB056442	171405	19-:7938880-8040517
6157	MMT00041452		19+:40356699-40357187
6158	Msh2	17685	17+:88071898-88123052
6163	Nudt13	67725	14+:21113943-21135981
6165	Hhex	15242	19+:37509359-37514294
6169	Nap1l5	58243	6-:58855226-58857059
6171	Abtb2	99382	2+:103518900-103558581
6174	MMT00000714		5-:15004031-15004577
6177	Thpo	21832	16-:20725239-20730599
6179	Cxcl5	20311	5+:91188403-91190650
6183	Atxn10	54138	15+:85166697-85293394
6184	MMT00000944		7+:114093791-114096155
6185	Icam4	78369	9+:20833897-20834938
6188	Myh7b	668940	2+:155437367-155460788
6189	MMT00020987		11+:79710708-79712255
6191	Ddost	13200	4+:137860650-137868147
6193	Lactb2	212442	1-:13615982-13650590
6196	Tpbpb	116913	13-:61002548-61006207
6197	Lss	16987	10+:75994371-76016671
6202	Hrk	12123	5+:118619772-118634966
6205	Ccl21b	18829	4+:42231058-42231890
6206	MMT00063701		7-:11692356-11693726
6209	Dsp	109620	13+:38243178-38290443
6211	Arhgap24	231532	5+:102910571-103326843
6213	Ccl2	20296	11+:81849075-81850728
6214	Mmd2	75104	5-:143039439-143084739
6216	Gm944	381126	18-:21286987-21458584
6218	Hnrnpc	15381	14-:52694473-52723657
6226	Vmn2r77	546983	7+:93949999-93960378
6231	9030622O22Rik	71570	2-:147770247-147855472
6233	Wnk2	75607	13-:49132968-49242559
6234	P4ha2	18452	11+:53915017-53945150
6236	MMT00024286		15+:39012214-39013022
6241	U2af1	108121	17-:31784159-31795641
6245	Syap1	67043	X-:159294783-159326363
6248	MMT00022090		3-:154939019-155056148
6249	Cep170	545389	1-:178663794-178737263
6250	Csnk2a1	12995	2+:152052631-152104985
6251	Ptger2	19217	14+:45608494-45622151
6258	MMT00052726		
6260	MMT00002928		17+:8453516-8455093
6261	2810026P18Rik	72655	9-:88415892-88418223
6262	Hprt1	15452	X+:50341313-50374836
6263	Slc7a4	224022	16-:17573053-17576299
6265	Trdn	76757	10+:32803315-33052271

6266	Gng10	14700	4+:59048069-59054285
6268	Slc45a3	212980	1+:133867185-133879540
6270	Cysltr1	58861	X-:103773094-103798935
6277	Prdm1	12142	10-:44156982-44178554
6282	Them4	75778	3+:94114087-94136460
6283	Ptpn22	19260	3+:103664214-103716169
6284	Cacnb2	12296	2+:14795279-14909057
6286	Rfc1	19687	5-:65653098-65726844
6293	Pggt1b	225467	18-:46397165-46440650
6295	Parl	381038	16-:20279889-20302458
6297	2310079F23Rik	78890	5-:35899986-35917709
6299	MMT00063871		5-:110211255-110276791
6300	RP24-87L14.2	100125272	2+:177757942-177761426
6304	Mrgprf	211577	7+:152486813-152495462
6305	MMT00012844		17-:87607995-87609589
6306	Dab2	13132	15+:6336823-6389597
6308	2410075B13Rik	223648	15-:75811102-75812323
6311	MMT00074658		7+:14066063-14066450
6313	Sim1	20464	10+:50615456-50708958
6320	1810055E12Rik	67894	19+:60887513-60912130
6322	Pknox2	208076	9-:36698573-36776010
6325	Cpsf3l	71957	4+:155243714-155262977
6329	Cyp2b10	13088	7+:26682694-26711474
6330	Dgkq	110524	5-:109075709-109089714
6333	Iqcg	69707	16-:33016660-33045787
6338	Setd3	52690	12-:109344649-109417473
6343	Slc20a1	20515	2+:129024535-129037344
6347	Rad18	58186	6-:112570798-112646588
6351	3321401G04Rik	77574	6-:42618000-42659527
6353	Spata13	219140	14+:61325636-61383386
6356	Dnahc1	110084	14-:32073559-32120346
6357	Ankrd9	74251	12-:112214633-112217206
6360	D17H6S56E-3	27762	17+:35153523-35163686
6362	Crispld2	78892	8+:122526690-122575693
6363	9130014G24Rik	215772	10-:10055507-10122805
6365	Rpl9	20005	5-:65779805-65782626
6367	Lhfp	108927	3+:52845468-52847620
6370	Aqp8	11833	7+:130605825-130611500
6371	Dedd2	67379	7-:25987856-26004946
6372	1110021L09Rik	76306	10+:23516786-23547773
6376	Nle1	217011	11-:82714272-82721864
6377	D18Ertd653e	52662	18+:68387669-68415207
6379	Asb9	69299	X+:160935834-160977684
6380	6330569M22Rik	229707	3-:107415448-107434612
6381	Dmxl2	235380	9-:54212966-54328272
6382	Col23a1	237759	11+:51371951-51396050

6383	Gbp2	14469	3+:142283626-142300970
6384	Rbm12	75710	2-:155920627-155929101
6386	Por	18984	5+:136165083-136211195
6387	MMT00042391		14+:105442696-105443405
6390	MMT00073452		10+:21009077-21010230
6392	Pdcl3	68833	1+:39044633-39054080
6393	3110043O21Rik	73205	4-:35138535-35173083
6394	Fbxo6	50762	4-:147519896-147525933
6396	Zfp105	22646	9+:122832215-122840137
6397	Slc2a9	117591	5-:38740559-38893391
6400	MMT00050153		1+:119637961-119883049
6401	MMT00053163	100041639	17-:15132750-15147126
6402	Npm3	18150	19-:45822225-45823640
6403	MMT00039607		14-:26661366-26662576
6404	Dbr1	83703	9+:99476235-99484485
6405	B3galnt1	26879	3-:69378854-69379848
6406	Sh3rf2	269016	18+:42213323-42313030
6410	Psd3	234353	8-:70212983-70214044
6413	Fbxo4	106052	15-:3928766-3929707
6417	Rasgef1c	74563	11+:49715351-49793724
6420	Mipep	70478	14+:61403394-61524315
6421	C230081A13Rik	244895	9-:56075514-56107846
6422	Grk5	14773	19+:60966242-61167953
6426	Mfsd4	213006	1-:133933357-133964488
6428	MMT00035510		5-:30422633-30424209
6429	Sim2	20465	16+:94085175-94348630
6432	2010109K11Rik	72123	12+:33064510-33065324
6436	4933401B06Rik	71011	X+:104737986-104739082
6437	Gnptg	214505	17-:25369169-25377082
6438	Vmn2r121	100038941	X-:121241111-121249525
6445	Mllt3	70122	4-:87419819-87452166
6449	1190007F08Rik	68859	4-:153397196-153399760
6456	Tyms	22171	5-:30387351-30400180
6458	Sycn	68416	7+:29325896-29327224
6461	Mtmr3	74302	11-:4380873-4494789
6462	Brip1	237911	11-:85874104-86014718
6464	Pcbp4	59092	9+:106356282-106366340
6465	Zfp474	66758	18+:52775568-52799484
6470	Arnt	11863	3+:95238458-95299492
6475	Sdc4	20971	2-:164249747-164268686
6476	Sntg2	268534	12-:30859858-31058095
6479	Tcerg1	56070	18+:42671176-42734498
6480	Lrfn5	238205	12+:62622416-62696244
6481	Atp1a1	11928	3-:101380147-101408580
6482	Nudt2	66401	4+:41412184-41427945
6484	A130010J15Rik	319266	1+:194999780-195002491

6485	Map3k7ip1	66513	15+:79963592-79992158
6486	OTTMUSG00000001	217066	11-:88826063-88826549
6487	Slc4a4	54403	5+:89316321-89664563
6488	Stat6	20852	10+:127080226-127097459
6489	MMT00008763		5-:121553095-121553514
6490	Il1r1	16177	1+:40281924-40373020
6491	Rpl5	19983	5+:108329620-108336243
6494	Evi5	14020	5-:108173814-108304126
6498	MMT00016809	100048067	1+:162965293-162968668
6500	MMT00069546		10-:78784301-78785112
6502	Col15a1	12819	4+:47221223-47325161
6503	Cep97	74201	16-:55905064-55934941
6504	Alad	17025	4-:62170201-62175226
6506	Scyl2	213326	10-:89102808-89149011
6510	Plcl2	224860	17+:50648932-50665916
6512	Ryr2	20191	13-:11646782-12198713
6513	Qpctl	67369	7-:19726212-19734592
6519	Phldb3	232970	7+:25396363-25414316
6520	Arnt2	11864	7-:91398290-91558424
6523	Hapln1	12950	13+:89679400-89748004
6525	Uchl5ip	73738	X-:70682834-70704360
6526	Thumpd3	14911	6+:112996326-113018171
6528	Dusp15	252864	2-:152770672-152777141
6529	Apeh	235606	9-:107987743-107996782
6531	EG629557	629557	9-:51810380-51811386
6532	Auh	11992	13-:52930492-53025034
6534	Cript	56724	17+:87424935-87435150
6539	Npy2r	18167	3-:82342304-82352006
6541	1810019J16Rik	69073	4+:133074949-133086704
6542	Epor	13857	9-:21763341-21767902
6544	AI790298	107239	19-:4164329-4175440
6545	Afm	280662	5+:90947988-90982571
6550	MMT00056589		1+:159526434-159526797
6551	Slc35b4	58246	6-:34105878-34127054
6554	MMT00003845	670007	10-:99760711-99859697
6555	Aadacl2	639634	3+:59814418-59829162
6564	Cebpd	12609	16+:15887105-15888559
6567	Trpm3	226025	19+:22767088-23062796
6569	Itih1	16424	14-:31742548-31756455
6570	Olfr148	258498	9+:39421153-39422085
6571	3000004C01Rik	70218	11-:102766891-102786403
6572	Foxc1	17300	13+:31898559-31902156
6573	Pecr	111175	1-:72305748-72330794
6576	Slfn2	20556	11+:82878613-82884178
6578	Pdcd10	56426	3-:75321043-75360677
6581	Cdc42ep1	104445	15+:78673076-78681332

6585	Tacc1	320165	8-:26279666-26367061
6587	Irf9	16391	14+:56223039-56228561
6588	Rbmx	19655	X-:54639519-54646158
6589	Mafb	16658	2-:160191441-160192798
6590	Pds5a	71521	5-:66014123-66039244
6592	Lgals2	107753	15-:78681291-78685959
6594	Zfyve27	319740	19+:42245081-42266214
6595	Oas1a	246730	5-:121346267-121357516
6597	Slc9a5	277973	8+:107873232-107893244
6599	1700020C11Rik	67900	11-:3991485-3995400
6601	Ttc30b	72421	2-:75774471-75776464
6605	Gpld1	14756	13+:25035080-25082624
6607	Reln	19699	5-:21390286-21850508
6608	Mtap7d3	320923	X-:54061020-54064726
6609	Tor1b	30934	2+:30808503-30812761
6612	MMT00077820		19+:27100470-27227226
6614	Cd300lg	52685	11+:101902823-101916930
6615	1810023F06Rik	217845	12-:104689107-104695433
6616	Prmt3	71974	7+:57033714-57113634
6621	Zfp521	225207	18-:13846723-14131047
6623	Ptbp2	56195	3-:119421914-119486176
6625	C1s	50908	6-:124487377-124492316
6628	MMT00004313		17+:48821671-48822052
6631	Tmem30a	69981	9-:79616750-79641079
6632	Dio1	13370	4-:106964078-106979748
6636	Ptchd2	242748	4-:147614641-147646513
6638	2610018G03Rik	70415	X+:48194612-48245025
6639	Fads3	60527	19+:10116095-10132739
6642	Aldh2	11669	5-:122017711-122043595
6644	Klhl9	242521	4-:88364198-88368747
6648	Cenpl	70454	1+:163001095-163016851
6650	MMT00050325		
6651	Gpx7	67305	4-:108073018-108079319
6652	MMT00004371		17-:50352073-50353549
6655	MMT00043469		1-:93385333-93386329
6657	Irf6	54139	1+:194979355-194998198
6659	Dpy19l4	381510	4-:11223082-11248777
6661	Hdac1	433759	4-:129193347-129219890
6665	AY026312	170939	16-:88932048-88932509
6670	Nat10	98956	2-:103561381-103601407
6673	Nasp	50927	4-:116274213-116300546
6675	Sfrs4	57317	4+:131453645-131457615
6678	Dmxl1	240283	18+:50120711-50124079
6680	Olfr393	259010	11-:73660686-73661625
6681	9130409I23Rik	619326	1+:182984887-182989945
6683	Cd59a	12509	2+:103936021-103955137

6685	Itgam	16409	7+:135206219-135261138
6686	Ldb3	24131	14-:35374453-35401860
6687	Ogfod1	270086	8+:96561094-96588935
6696	Zfp740	68744	15+:102034999-102046031
6697	Serpini1	20713	3+:75361494-75446276
6699	4930592I03Rik	75853	18-:83089332-83090009
6705	Sorbs2	234214	8+:46713537-46913253
6708	Serpini2	67931	3-:75046293-75074002
6709	MMT00003854		6-:41424683-41431715
6710	MMT00022451		8+:73611709-73613824
6714	Lima1	65970	15-:99608938-99650503
6716	Cd180	17079	13+:103483667-103497033
6718	Rnf168	70238	16+:32277585-32301493
6719	Cacng8	81905	7+:3394458-3415366
6726	Meis2	17536	2-:115688829-115890783
6728	Olfr75-ps1	258186	11-:73218945-73219881
6729	Hnrnpa1	15382	15+:103070924-103075386
6732	Olfr1038	259015	2+:85962081-85963037
6733	Mfng	17305	15-:78586312-78603875
6734	Ehd4	98878	2-:119915223-119980244
6735	Fus	233908	7+:135111067-135125545
6738	Fxr1	14359	3+:33919029-33968259
6746	E2f8	108961	7-:56121797-56136370
6749	Dnase1l3	13421	14-:8800262-8826662
6754	Trpv3	246788	11+:73081121-73110702
6755	Wdr22	320808	12-:81439378-81537574
6756	Sds	231691	5+:120926555-120933912
6759	Sqle	20775	15+:59146794-59162747
6760	2810428I15Rik	66462	8-:73028194-73030638
6761	MMT00030685		6-:69942472-70056173
6764	Cald1	109624	6+:34659484-34724091
6765	Ldhb	16832	6-:142438769-142456403
6766	Sspn	16651	6+:145882666-145913740
6767	Erc2	238988	14+:28435693-28466669
6768	Plekhf2	71801	4-:10915811-10934717
6769	Banp	53325	8+:124474479-124549837
6771	Insc	233752	7+:121912245-121993886
6774	Cilp	214425	9+:65113632-65128423
6777	B3galtl	381694	5+:150511894-150563361
6779	Gbp6	229900	3+:142193333-142210014
6784	AI182371	98870	2-:34937379-34957037
6788	Psen2	19165	1-:182157136-182176032
6789	Trp63	22061	16+:25802027-25892166
6795	Cdyl2	75796	8-:119092630-119095285
6797	Blvra	109778	2+:126906253-126922775
6798	Tcea3	21401	4+:135803866-135830820

6804	Cyp2d22	56448	15-:82201909-82206451
6805	Rrbp1	81910	2-:143811601-143836914
6806	Agrn	11603	4-:155539402-155571448
6808	EG433073	433073	17+:20798483-20798976
6809	Rorc	19885	3+:94176707-94202510
6812	MMT00040104		17+:3056713-3057111
6815	Sort1	20661	3+:108087057-108159607
6816	Slc46a3	71706	5-:148690029-148706391
6818	BC030183	230766	4-:129254056-129255782
6819	Cct2	12461	10-:116488054-116500821
6820	MMT00046996		7+:14889997-14894927
6822	Ldhc	16833	7+:54116624-54133360
6823	Yars	107271	4+:128867107-128896482
6824	Irf7	54123	7-:148449087-148452300
6825	4930528G09Rik	75172	5-:20798784-20930496
6828	Pigh	110417	12-:80183921-80190636
6830	Rsc1a1	69994	4-:141239767-141241514
6837	Arhgap25	232201	6-:87409383-87446106
6838	Nod2	257632	8+:91176763-91212304
6839	Mrpl46	67308	7-:85920228-85927927
6842	Amotl2	56332	9+:102624863-102635741
6845	Cyb561d1	72023	3-:107998702-108003354
6846	Olfr1402	258272	3-:97214139-97215102
6847	MMT00058311		19-:35126523-35126993
6852	MMT00007193		Y_random+:10231498-10250577
6855	Ash1l	192195	3+:88785599-88882514
6856	Tnrc6a	233833	7+:130323303-130337255
6857	Serinc2	230779	4-:129930739-129952831
6861	Krt8	16691	15-:101827141-101834714
6864	Sh3md4	237353	10+:58446668-58598735
6866	Gls	14660	1-:52220295-52290240
6870	Xlr4b	27083	X+:70459703-70467795
6873	BC023055	226123	19-:42149437-42160836
6878	MMT00041953	675689	11+:46375532-46375875
6880	Dmrtb1	56296	4-:107348899-107356822
6884	Hnrnpf	98758	6+:117856833-117875635
6888	Cxcl9	17329	5-:92752015-92757143
6899	Pla2g4e	329502	2-:119992149-120002680
6901	MMT00048285		9-:66653114-66654035
6905	Pkd1l3	244646	8+:112138563-112145913
6906	2610301B20Rik	67157	4+:10801714-10826398
6907	Usp50	75083	2-:126535090-126539157
6908	Mmp12	17381	9+:7347457-7358683
6910	Ly6c2	100093633	15-:74938587-74942237
6911	C130090K23Rik	231293	5+:73797488-73844515
6912	Crym	12971	7-:127329897-127345502

6914	Thrap3	230753	4-:125842644-125844308
6916	Wdr5b	69544	16+:36041360-36043058
6917	MMT00070118		7+:13188438-13189997
6918	Cog8	97484	8-:109570428-109572154
6921	Olfml1	244198	7+:114710990-114733856
6922	Tmem86b	68255	7-:4579635-4581784
6928	Klhl8	246293	5-:104291069-104340215
6931	Hrsp12	15473	15-:34413891-34424945
6932	Ckap4	216197	10-:83989048-83996368
6934	Panx3	208098	9-:37468658-37476776
6935	Arsj	271970	3+:126067769-126143293
6946	Nol5	55989	1+:59741942-59768352
6950	Zfp710	209225	7+:87225934-87235824
6956	Gria2	14800	3-:80492368-80606508
6959	Slc1a5	20514	7+:17366755-17383591
6962	Cyp1b1	13078	17-:80106293-80114381
6966	Mia3	338366	1_random-:300175-342929
6972	Trspap1	71787	4-:131867679-131885431
6974	Tmem65	74868	15-:58613824-58654683
6975	Wdr67	210544	15+:57743754-57801626
6976	Rbm35b	77411	8-:108653857-108692989
6985	Ntng1	80883	3-:109946353-110040056
6990	Nr1i2	18171	16-:38248436-38294910
6992	Olfr172	259003	16-:58760359-58761287
6994	Glul	14645	1+:155747097-155756824
6998	EG245651	245651	X-:138967983-138968278
7002	Lrrn1	16979	6+:107479778-107520204
7003	MMT00008726		11+:104692598-104892629
7010	Mlph	171531	1+:92811679-92847718
7011	1810022C23Rik	69123	13-:35038661-35055678
7012	Dennd4a	102442	9+:64686640-64767472
7020	Fbln5	23876	12-:102988149-103057279
7021	Slc16a8	57274	15-:79081443-79085187
7029	MMT00072012		12+:31569085-31578350
7030	RP23-232H13.5	100043387	2+:176503994-176871927
7033	Rfwd3	234736	8-:113796920-113824080
7035	4933425L06Rik	66763	13+:105872201-105911860
7039	Zmat3	22401	3-:32233719-32264542
7041	Sp140	434484	1+:87511062-87516733
7042	C730007P19Rik	20865	7+:14320095-14323700
7043	Cyp11b2	13072	15-:74681468-74686655
7044	Vbp1	22327	X+:72759691-72780249
7047	MMT00019310		12-:13759481-13760503
7049	Psmd14	59029	2+:61549825-61638432
7054	Trerf1	224829	17+:47450769-47496407
7057	Stk10	20868	11+:32433304-32524587

7058	Asns	27053	6-:7625165-7643106
7061	Spsb3	79043	17+:25024425-25029092
7066	Agtr1b	11608	3-:20214282-20302434
7069	Ube1l	74153	9+:107877852-107886389
7073	Arhgap4	171207	X-:71139697-71156568
7074	Dcbld2	73379	16+:58460843-58465940
7078	Olfr463	258408	11-:87706489-87707424
7083	MMT00044154		18+:44221143-44222334
7084	Spz1	79401	13-:93344784-93345921
7085	St8sia2	20450	7-:81084007-81158547
7087	Dio2	13371	12-:91962991-91976878
7088	Slc37a1	224674	17+:31432075-31487641
7089	AI646023	192734	10-:75012869-75022834
7093	Magt1	67075	X-:103165815-103207117
7094	Cyp2c54	404195	19-:40112424-40122137
7095	Timm8a1	30058	X-:131071796-131076168
7097	Dnajb6	23950	5+:30062564-30093628
7099	Kcnk10	72258	12-:99694928-99812922
7100	Tomm22	223696	15+:79501298-79504002
7101	Gimap6	231931	6-:48651579-48658212
7102	Hnrnpm	76936	17-:33783183-33822348
7105	Plac8	231507	5-:100982751-101001224
7108	Adamtsl1	77739	4+:86061429-86072048
7109	Bxdc2	67832	15-:10405936-10415579
7112	Apbb3	225372	18-:36830813-36839020
7116	Katnal1	231912	5-:149687626-149740223
7118	BC051019	57355	7-:116855695-116867392
7120	Dusp1	19252	17-:26642536-26645406
7122	Gigyf2	227331	1+:89329694-89347367
7124	Dnajb13	69387	7-:107650250-107661908
7126	MMT00002620		2+:73973812-73988889
7128	Gemin8	237221	X+:162614904-162627732
7131	1810037I17Rik	67704	3+:122627317-122628654
7132	Sp9	381373	2+:73109987-73112393
7133	Hook2	170833	8+:87514517-87527160
7134	Krba1	77827	6+:48345612-48366884
7135	Ezr	22350	17-:6942812-6987069
7136	Id3	15903	4+:135699736-135701301
7139	Pld3	18807	7-:28317036-28338131
7140	Mtbp	105837	15+:55389041-55457968
7141	MMT00061218	629364	12-:25176464-25178595
7143	Cxcr7	12778	1+:92100600-92112182
7145	2810021G02Rik	353208	2-:177802399-177813224
7149	9530077C05Rik	68283	9+:22215922-22249127
7151	MMT00007982		6-:67684189-67685598
7153	Glt6d1	71103	2-:25666520-25671357

7154	Fbxw13	211305	9-:109085520-109087904
7155	MMT00017420		14+:53303921-53304140
7156	Tmem1	216131	10-:77649474-77670789
7159	1700031F05Rik	73300	X-:100054526-100061695
7163	Ptplb	70757	16+:35022513-35106660
7165	Nt5m	103850	11+:59661574-59690034
7173	Trim59	66949	3-:68839215-68841208
7181	Nedd4	17999	9+:72525155-72595035
7185	Ostf1	20409	19-:18655244-18706299
7188	Atpbd4	66632	2-:114342155-114480619
7191	Fgfr1op	75296	17+:8361897-8389343
7192	Ttc21b	73668	2-:66022384-66090061
7199	Wee1	22390	7+:117265585-117285647
7206	Rgma	244058	7+:80520573-80564784
7209	Gpa33	59290	1+:168060590-168096640
7211	2410014A08Rik	109154	5-:115593029-115608170
7218	Cylc2	74914	4+:51229548-51242569
7220	1110018M03Rik	67606	2-:110201081-110203150
7224	Pafah2	100163	4+:133953385-133983323
7225	Tiam1	21844	16-:89787356-89961072
7228	OTTMUSG00000015	328483	14+:54048401-54050831
7229	H2-DMb1	14999	17+:34282252-34297169
7232	MMT00078948		7+:146960610-146960924
7234	MMT00005439		7-:12277020-12277559
7238	Pycard	66824	7-:135136171-135137239
7240	C730007P19Rik	20865	7+:14338705-14341327
7241	Ptpn11	19247	5-:121583799-121641283
7243	Slc4a10	94229	2+:61884650-62164782
7245	Ski	20481	4-:154531449-154596629
7251	MMT00018228		16-:16365094-16366304
7252	Nolc1	70769	19+:46150502-46157814
7253	Olfr593	258378	7+:110360375-110361358
7256	MMT00063357		13+:107223869-107266980
7258	Bmp2k	140780	5+:97426707-97520066
7261	Ttc7b	104718	12-:101538998-101758909
7263	Narg1l	66897	14-:79735440-79790484
7265	Dok1	13448	6-:82980951-82983430
7273	Rnf103	22644	6+:71443887-71460875
7274	Laptm4b	114128	15-:34199401-34199584
7278	1810030J14Rik	66289	1+:176260649-176263004
7279	Cldn13	57255	5-:135390571-135391199
7282	Ppic	19038	18-:53565994-53577661
7284	MMT00008674		5-:121378079-121382183
7285	Tbc1d4	210789	14-:101841584-101910783
7286	Noxo1	71893	17+:24833743-24837519
7289	L1cam	16728	X-:71100373-71115143

7292	Spna1	20739	1+:176102998-176178198
7300	Olfr560	259117	7-:109901496-109902441
7303	Tmem182	381339	1+:40862448-40913731
7307	Agmat	75986	4+:141302582-141315177
7308	Tmem52	69671	4+:154843221-154844966
7309	Mpp5	56217	12+:79850007-79939068
7311	Lsm6	78651	8-:81330466-81345036
7315	EG624219	624219	9+:21639938-21641788
7316	Ifi204	15951	1-:175677426-175843051
7318	Rfpl4	192658	7-:5061468-5068498
7320	MMT00051732		7+:148592427-148593223
7321	Rdbp	27632	17+:34987863-34993103
7322	D730048I06Rik	68171	9-:35595694-35597668
7328	Atp5b	11947	10+:127520382-127527328
7332	1700027L20Rik	69453	1+:89083238-89084979
7334	Lor	16939	3-:91884203-91887062
7335	Calm2	12314	17-:87833367-87842067
7336	Cd5l	11801	3+:87161896-87174994
7338	MMT00070692		19+:8346969-8349204
7339	Tdp1	104884	12+:101122734-101144344
7340	Ascc3l1	320632	2+:127034132-127067416
7341	Olfr1424	258676	19-:12133300-12149054
7342	Anp32e	66471	3+:95733504-95749494
7343	Mcm10	70024	2-:4911213-4933778
7347	1110032A04Rik	66183	3-:69623462-69663796
7349	MMT00065780		14+:15270505-15271223
7355	Procr	19124	2+:155577078-155581194
7360	Mfi2	30060	16+:31878895-31899103
7362	Rrm2	20135	12+:25393147-25398972
7367	Gchfr	320415	2+:118993513-118998125
7368	Adam8	11501	7-:147164839-147178387
7370	Spop	20747	11+:95275427-95354327
7371	MMT00063687		5-:110546730-110547947
7372	Fastk	66587	5-:23946857-23950188
7377	Suds3	71954	5-:117541690-117566836
7378	1700001J03Rik	69282	5-:146994023-146996876
7379	MMT00078052		19-:37778218-37797762
7382	RP23-34B24.1	230161	4-:49460165-49464007
7386	Pex6	224824	17+:46848411-46862489
7388	Amn	93835	12+:112509308-112514469
7389	Sfrs5	20384	12+:82047562-82051486
7391	Bhlhb8	17341	5+:144951154-144952534
7393	Igk-V28	16114	6+:70143469-70165974
7396	Lao1	100470	4+:118634571-118641489
7397	MMT00020969		3+:144390767-144392049
7398	Rbmxrt	19656	8-:81029168-81032738

7400	Cars2	71941	8-:11513978-11550744
7401	Pdzk1ip1	67182	4+:114761327-114766498
7404	Prpmp5	381832	6-:132261612-132314135
7405	Snx31	66696	15-:36434159-36485328
7406	Sult2a1	20859	7-:14417914-14422751
7407	Slc9a3r1	26941	11+:115024716-115042493
7408	1700081L11Rik	76719	11-:104194813-104218190
7409	MMT00014691		19-:27836215-27837137
7413	Olfr1101	258584	2-:86828400-86829331
7414	Clpx	270166	9+:65142115-65178449
7415	Isoc1	66307	18+:58819340-58837894
7416	6430548M08Rik	234797	8+:122638059-122687323
7418	Pkp3	56460	7+:148265676-148276408
7419	Arid5a	214855	1+:36364625-36377372
7424	Cdkn1c	12577	7-:150644615-150646883
7430	Gpr137	107173	19-:7012560-7016900
7431	Idh1	15926	1-:65205847-65221943
7436	1700063K16Rik	76610	16-:76819829-76842029
7439	Syt17	110058	7-:125525369-125587066
7444	Hspe1	15528	1+:55145175-55148039
7449	Creg1	433375	1+:167693910-167704160
7452	Abcc9	20928	6-:142537652-142650793
7455	MMT00011288		4+:151530158-151531670
7457	Mrpl22	216767	11+:57985176-57993080
7458	Cav1	12389	6+:17256449-17289453
7459	2610207O16Rik	66620	1-:42763909-42770328
7460	Krt6a	16687	15-:101520898-101524679
7464	MMT00059239		17-:3311765-3314181
7466	Insl6	27356	19-:29395844-29399808
7468	Hsd3b1	15492	3-:98656117-98662142
7469	Sez6l	56747	5-:112853642-112904607
7472	Klra1	16627	6-:130313935-130332546
7476	MMT00059569		13-:66497459-66891917
7479	Mcf2l	17207	8+:12873825-13020508
7480	4930506M07Rik	71653	19-:59047846-59150114
7481	Trh	22044	6-:92192055-92193882
7482	Tdg	21665	10+:82092625-82113018
7483	Cyp3a25	56388	5-:146788338-146821192
7492	Adfp	11520	4-:86302617-86315898
7493	Cngb3	30952	4+:19207996-19437768
7495	Ugt2b5	22238	5-:87553984-87569343
7496	Arsi	545260	18+:61071649-61078212
7498	MMT00040284		12+:71072008-71073929
7499	Nipa1	233280	7-:63233855-63274944
7500	MMT00011283	433546	2-:156615456-156616113
7503	Rab11fip1	75767	8-:28251273-28285121

7507	Ccdc136	232664	6+:29349588-29376952
7510	Gins3	78833	8+:98157458-98168959
7513	Nr5a2	26424	1-:138740852-138857025
7516	MMT00018931	100040020	X-:150499736-150504063
7517	T	20997	17+:8627287-8635361
7519	Gstm1	14862	3-:107815175-107820891
7520	MMT00070057		X+:65882566-65883524
7522	Rb1	19645	14-:73595309-73725547
7523	Daam1	208846	12+:72932055-73016780
7524	Aldh4a1	212647	4+:139178825-139204933
7525	Trim61	260296	8-:67536884-67542428
7527	Rnase10	75019	14+:51627649-51630431
7528	Cadm1	54725	9+:47596063-47659115
7537	Glce	93683	9-:61907817-61918647
7539	Eef2	13629	10+:80627326-80645252
7540	Olfr658	259051	7-:111792912-111793884
7543	Cct5	12465	15-:31520638-31531559
7544	Btbd14a	67991	2-:25915420-25978304
7545	C81431	98077	17-:34012613-34027561
7546	Ddhd1	114874	14-:46214211-46240515
7550	Rab11fip5	52055	6-:85284958-85324456
7551	Grik3	14807	4+:125300724-125385592
7556	A530050D06Rik	104816	12+:113345081-113365771
7558	Rbm10	236732	X+:20197121-20228014
7560	Cyp2r1	244209	7-:121693702-121706471
7572	Ppm1l	242083	3+:69301690-69359313
7577	Cldn8	54420	16-:88562602-88563280
7580	Slc22a20	381203	19-:5971398-5986140
7582	MMT00065786		14+:14754201-14780995
7586	5730469M10Rik	70564	14-:41807028-41827064
7589	4933404M02Rik	66748	2+:70346873-70378940
7590	Ankrd40	71452	11+:94199602-94203160
7596	Tifa	211550	3+:127494298-127501306
7600	Zfp382	233060	7+:30915692-30937948
7601	Kcne3	57442	7+:107329675-107333378
7602	Pcca	110821	14+:122933564-123289047
7603	Sod2	20656	17+:13200859-13208205
7609	Ctrc	76701	4-:141394566-141402200
7610	Scand1	19018	2-:156137593-156138262
7612	MMT00014216		6-:133707411-133713096
7618	Glis2	83396	16+:4608595-4614628
7619	Pik3ip1	216505	11+:3230382-3242971
7620	Foxo3	219038	14+:52538462-52540381
7624	Mum1l1	245631	X+:135769253-135771297
7637	MMT00056309	382035	8-:125144181-125146633
7641	Cyp26a1	13082	19+:37772343-37776026

7642	Igl-V1	16142	16-:19061844-19085553
7643	Mrps28	66230	3-:8802156-8923814
7644	MMT00033296		11+:76327564-76327948
7646	Sfrs18	66625	4+:21775071-21803672
7654	Ptpmt1	66461	2-:90750866-90758602
7656	Lonrf3	74365	X+:33916422-33917017
7657	Olfr731	258360	14-:50857579-50858558
7658	Lce1l	73730	3-:92653870-92655208
7659	Rab30	75985	7+:99968414-99985157
7661	Rac1	19353	5-:144266352-144288979
7662	Lyz1	17110	10-:116725639-116729894
7663	4930488L21Rik	75809	8-:96335590-96337455
7670	Ccdc57	71276	11-:120687844-120734986
7674	Spata7	104871	12+:99866372-99908029
7676	Jmjd2c	76804	4+:73897851-74051765
7678	Ltbr	17000	6-:125256593-125263872
7680	EG667232	667232	16-:36569040-36580930
7683	Man1a2	17156	3-:100370339-100489150
7686	Apoa5	66113	9+:46076690-46079492
7688	MMT00058344		13-:64423876-64424841
7690	4632417N05Rik	74032	8-:120185298-120195415
7691	Tnnc1	21924	14+:32021497-32024897
7693	B3galt6	117592	4-:155365330-155366731
7695	Cpsf1	94230	15-:76426238-76437984
7698	Klrb1a	17057	6-:128559634-128572952
7700	Iqce	74239	5-:141139461-141178210
7707	Mll3	231051	5-:24777617-24893859
7718	Pdia6	71853	12+:17273353-17291577
7721	MMT00000356		8-:110214319-110214744
7725	3230401D17Rik	66680	2-:163231672-163245224
7726	Cyp2d9	13105	15+:82282828-82287257
7727	MMT00065667		14+:121112299-121112967
7731	1500001M20Rik	68971	6-:114954374-114987893
7734	Cyp2b23	243881	7-:27450574-27471449
7735	Traip	22036	9+:107853299-107873859
7739	Grm8	14823	6-:27225611-28076195
7742	Spag9	70834	11+:93857582-93987393
7745	C330016O10Rik	212706	11-:51456591-51464288
7749	Mul1	68350	4+:137990605-137995818
7751	Sgk2	27219	2+:162813281-162839862
7752	Ugp2	216558	11-:21221798-21270480
7753	Gfm1	28030	3+:67234045-67278989
7755	B230317C12Rik	56279	2+:26483991-26492014
7756	Edc3	353190	9+:57587690-57597973
7761	Nudt19	110959	7-:36332211-36340852
7763	2010204K13Rik	68355	X-:6989231-7000074

7767	Bai3	210933	1-:25124323-25188246
7770	Galt	14430	4+:41702375-41705553
7775	Nif3l1	65102	1+:58501994-58518367
7779	Slit2	20563	5+:48666501-48697803
7781	Pip	18716	6+:41797552-41802063
7782	Gm114	228730	2+:146681598-146716820
7784	Msh4	55993	3-:153520112-153569102
7787	MMT00043009		9-:97127501-97129284
7788	1600021P15Rik	239796	16-:28826263-28929784
7791	Dtx4	207521	19-:12543625-12575707
7792	6720467C03Rik	68099	4-:12080869-12098989
7793	MMT00070312	668699	14+:103409421-103410015
7794	Hbs1l	56422	10+:21015939-21030325
7795	MMT00071427		7-:26204174-26204845
7797	Ddx47	67755	6+:134961637-134973789
7798	MMT00077178		19+:11511939-11513093
7800	Rgr	57811	14-:37850811-37862120
7802	Adcy4	104110	14-:56387929-56402856
7803	Ttyh1	57776	7+:4071178-4085578
7806	D1Ertd53e	213056	1-:58584099-58643136
7807	2410004N09Rik	69749	18+:33954572-33956736
7808	Bnc2	242509	4-:83921000-84037038
7810	Trim45	229644	3+:100726399-100735625
7813	Stac	20840	9-:111463937-111592720
7821	MMT00042003		7-:97959541-97960744
7824	MMT00001823	100044551	1-:82999078-83001428
7827	1700010A17Rik	75495	2+:35904997-35935236
7837	Pou3f1	18991	4+:124334895-124336413
7839	Asl	109900	5-:130487373-130500201
7842	Pdk2	18604	11-:94887572-94902629
7843	V1ri1	252907	13-:22279015-22279917
7844	Btc	12223	5-:91787835-91831845
7848	Casp12	12364	9+:5345510-5372647
7851	Fank1	66930	7+:140968564-141073215
7860	Bmp6	12161	13+:38437584-38591597
7861	Klhdc4	234825	8-:124320233-124353469
7862	Cpsf4l	52670	11-:113559485-113571331
7865	MMT00062659		15-:41861949-41864525
7873	Steap4	117167	5+:7960468-7982212
7875	Crat	12908	2-:30257505-30270980
7876	EG625534	625534	1+:152396446-152396808
7877	Obfc2a	109019	1-:51526333-51535251
7879	Ifitm3	66141	7-:148195488-148196643
7887	4930418G15Rik	69312	1-:9958707-9999201
7888	Pigz	239827	16+:31934062-31946136
7892	Ccr2	12772	9+:124016921-124023289

7894	MMT00029319	100039284	10+:61246511-61247569
7896	Polr3e	26939	7+:128061297-128089335
7897	MMT00067427	100041449	5+:146890910-146924395
7900	Myo18a	360013	11+:77590766-77679482
7901	Slc41a1	98396	1+:133727198-133745441
7907	Klk1b8	16624	7+:51206051-51210308
7908	MMT00082889		4-:127085611-127086063
7909	Oas3	246727	5-:121203108-121227668
7910	AK122209	382038	8+:126548955-126572397
7911	Pik3c2g	18705	6+:139792407-139917802
7913	MMT00065558		12+:9436283-9437290
7915	Cdx4	12592	X+:100516736-100525563
7916	MMT00016293		Y_random+:6427127-6429303
7918	Rtn4ip1	170728	10+:43621898-43666734
7922	Casp7	12369	19+:56471618-56516831
7929	Pole4	66979	6-:82596705-82602857
7934	MMT00002010		13-:3136659-3139392
7936	MMT00061970		1-:12591242-12591281
7938	Ppp2r2b	72930	18-:42804924-43219125
7940	Cyp2b9	13094	7+:26958427-26995679
7944	AI132487	104910	12+:110089344-110095023
7945	Gpc2	71951	5-:138714888-138721195
7950	Efha1	68514	14-:58535098-58618106
7953	MMT00066281		11+:45643053-45643532
7954	Ccdc12	72654	9+:110559006-110614098
7956	Pnoc	18155	14-:66020316-66044310
7957	MMT00074126		13+:22374396-22375819
7959	Tbx5	21388	5+:120246786-120333511
7962	Ajap1	230959	4-:152747328-152856813
7963	Rxrg	20183	1+:169528646-169569752
7965	Enpp5	83965	17+:44215817-44223508
7969	MMT00001293		3+:8327474-8330440
7973	Lgals9	16859	11-:78776896-78798333
7974	Arhgef7	54126	8+:11782561-11835213
7975	Lancl1	14768	1-:67047090-67085439
7976	Anapc5	59008	5-:123241616-123250569
7983	Syt13	80976	2+:92755331-92793824
7990	Nostrin	329416	2+:68973946-69027384
7991	Gpr115	78249	17-:42793837-42829236
8001	Efr3a	76740	15+:65618619-65704121
8005	Omg	18377	11-:79314857-79317521
8007	MMT00040041		8+:27408847-27464610
8009	Trat1	77647	16-:48733829-48750579
8016	Mrpl35	66223	6-:71765101-71773764
8020	Nope	56741	9+:64949341-64985749
8021	1600002K03Rik	69770	10+:79635687-79637864

8022	MMT00004959		X+:98257488-98258368
8031	Scoc	56367	8-:85958391-85966379
8034	Gas1	14451	13-:60275765-60278726
8035	Frzb	20378	2-:80252333-80287350
8036	Zfp160	224585	17+:21145937-21165823
8037	MMT00052151		1-:43055955-43077637
8039	H1foo	171506	6+:115894956-115900249
8043	4930405D01Rik	73795	16+:52849397-52979019
8045	Pgam5	72542	5-:110688179-110698898
8049	Pon1	18979	6-:5118104-5143824
8051	MMT00035998		
8053	Gal	14419	19-:3410095-3414112
8054	Smarca2	67155	19+:26822948-26852763
8055	Ptcd3	69956	6-:71830634-71839625
8056	EG433923	433923	5+:97693755-97694741
8058	Epb4.9	13829	14-:71003509-71018209
8067	Taok3	330177	5+:117583626-117723866
8071	D630002J15Rik	77522	6+:38059245-38065807
8072	Zcchc9	69085	13-:91936137-91945766
8073	Irak1bp1	65099	9+:82723629-82740627
8075	MMT00011946		5-:73867838-73868525
8079	Pts	19286	9-:50330120-50336746
8080	Lpar1	14745	4-:58447859-58566391
8081	Rhot1	59040	11+:80061973-80080313
8084	Psmd10	53380	X-:137483679-137491206
8085	MMT00057439		X-:136619365-136619999
8088	Negr1	320840	3+:156679145-156823707
8091	Slc10a1	20493	12-:82056478-82068971
8094	4930591A17Rik	68175	2+:179149640-179151585
8095	Bex2	12069	X-:132601104-132602649
8096	V1rh17	252912	13+:22179746-22180671
8099	MMT00052332		19+:58563727-58563925
8102	Hivep2	15273	10+:13847381-13870251
8106	MMT00065672		14-:121214347-121214954
8108	Cpne4	74020	9+:104775001-104936875
8109	6330545A04Rik	76166	4+:140769916-140775953
8110	Abhd7	384214	5+:107832606-107842646
8114	Kcnn1	84036	8-:73367056-73379484
8117	Ngp	18054	9+:110322352-110325514
8118	MMT00011146		12+:78920464-78921288
8120	5730410E15Rik	319613	15-:44503406-44560694
8122	Hexim1	192231	11+:102977638-102981036
8127	Hmg20b	15353	10-:80808796-80813155
8129	Gtse1	29870	15+:85690405-85707002
8136	Ikzf3	22780	11-:98327555-98380046
8141	Scnn1a	20276	6+:125271357-125294957

8147	1700029J07Rik	69479	8-:47038961-47060607
8148	Ppp1r9a	243725	6-:4942157-4961002
8149	Nav3	260315	10-:109121128-109207380
8150	Pnn	18949	12+:60167971-60174404
8154	Neb	17996	2-:51992182-52194317
8155	Mark3	17169	12+:112813181-112894430
8156	Ttll1	319953	15-:83314201-83341294
8157	MMT00005305		6-:15477154-15588274
8158	Smc5	226026	19-:23281403-23317224
8160	Akr1c12	622402	13-:4267422-4278606
8161	MMT00031928		16+:43312605-43313377
8162	Muc16	73732	9-:18337023-18371302
8164	MMT00017950		6-:142919365-142919929
8170	Snx22	382083	9-:65914914-65917545
8171	Spink8	78709	9+:109719158-109729137
8174	5330426P16Rik	68190	16-:50719411-50732853
8176	Stx6	58244	1+:157006022-157049145
8181	Thap1	73754	8+:27268694-27274622
8182	Tbx1	21380	16-:18581805-18587062
8184	Rbm26	74213	14-:105505998-105576497
8187	Gins1	69270	2+:150735322-150757012
8189	Cflar	12633	1+:58783206-58790070
8190	Rc3h2	319817	2-:37230577-37270266
8193	Pi4k2a	84095	19+:42164975-42196520
8194	MMT00077789		X-:153450772-153452073
8200	Dfna5h	54722	6-:50157402-50211768
8203	Rnf170	77733	8+:27234312-27253102
8209	MMT00072546		X-:12903091-12904107
8210	Hspa12a	73442	19-:58873309-58936058
8212	MMT00052607		3-:77889848-77891186
8217	Bhmt	12116	13-:94387430-94407456
8221	Stxbp3a	20912	3-:108596097-108643420
8222	Etfa	110842	9-:55302316-55359917
8223	Pdlim3	53318	8+:46970814-47004769
8226	Gli1	14632	10-:126766942-126778635
8231	Tcfcp2	21422	15-:100333227-100382378
8234	Tbpl1	237336	10-:22425181-22451133
8237	Caprin1	53872	2-:103605181-103637211
8241	MMT00047047		2-:99394281-99564304
8244	Vamp4	53330	1+:164504431-164525021
8252	Plcl1	227120	1+:55752583-55808444
8254	Bbs2	67378	8-:96591853-96622323
8255	Prkar1a	19084	11+:109510676-109530943
8256	Tjap1	74094	17-:46394824-46419958
8257	Aadac	67758	3+:59835709-59844079
8262	Syp	20977	X+:7215836-7230377

8266	Prosc	114863	8+:28153086-28163885
8268	Kctd7	212919	5+:130620833-130630984
8269	Ccdc11	74453	18+:74442775-74519638
8270	Clspn	269582	4+:126234236-126271157
8274	Zc3h11a	70579	1-:135518092-135550589
8278	Dlgap1	224997	17+:71158411-71168741
8279	Kif13a	16553	13-:46844458-47025087
8284	EG209380	209380	7-:113563185-113566144
8291	Lce1a2	73722	3-:92472534-92474237
8292	Mapkap1	227743	2+:34479448-34480469
8293	Mpi	110119	9-:57392066-57400559
8297	Dhx37	208144	5-:125896235-125977332
8300	MMT00043905		15-:74816454-74817477
8304	Fubp1	51886	3+:151873461-151895735
8313	Frap1	56717	4+:147822715-147931790
8314	Armc6	76813	8-:72744089-72758367
8317	E130311K13Rik	329659	3-:63724086-63733292
8319	MMT00062477		10-:14465758-14467192
8320	Nme3	79059	17+:25033450-25034448
8321	Olfr1120	259031	2+:87197602-87198547
8323	Cbx6	494448	15-:79654328-79656137
8325	Psme2	19188	14-:56206276-56209745
8327	4930503B20Rik	75015	3-:146312663-146314276
8328	Klhdc8a	213417	1+:134195637-134202152
8329	Pipox	19193	11-:77694116-77707283
8331	BC052688	432812	13+:61731372-61888643
8332	Tmem206	66950	1+:193149843-193174790
8334	MMT00082897		6-:73478635-73479239
8335	Cftr	12638	6+:18120686-18272767
8336	MMT00041615		5+:152083838-152084048
8337	Lgr4	107515	2+:109810843-109853629
8339	MMT00046482		14+:60759849-60760245
8340	Pde1a	18573	2-:79677036-79969269
8341	MMT00017270		6-:47926988-47927875
8342	Cwf19l1	72502	19-:44184514-44210366
8344	Cfh	12628	1-:141982455-142079899
8345	Elfn1	243312	5+:140447196-140449682
8346	Hiat1	15247	3-:116334086-116365468
8349	Rhoq	104215	17+:87362641-87399403
8355	Pms2	18861	5+:144737915-144746584
8356	Fbxl12	30843	9-:20442232-20447132
8357	Siva1	30954	12+:113883122-113887355
8359	1190002N15Rik	68861	9-:94418281-94438274
8361	MMT00025562		9+:18231928-18233469
8363	MMT00006564		19-:28936820-28938254
8366	Sema3a	20346	5+:13399847-13600084

8368	Rad51l3	19364	11-:82691886-82704107
8371	Hipk2	15258	6-:38647904-38825952
8372	Uap1	107652	1-:172072133-172105011
8388	Zfyve21	68520	12+:113052483-113066560
8392	Asf1b	66929	8+:86479592-86494093
8394	Dpy19l3	233115	7-:36473861-36497237
8396	MMT00040956		18+:10928840-10929732
8398	ND5	17721	M+:0-16297
8399	Herpud1	64209	8+:96910399-96919258
8400	Calm4	80796	13+:3837141-3837576
8401	Slain1	105439	14+:104049416-104104122
8403	Rps29	20090	12-:70258711-70260057
8404	Fa2h	338521	8-:113869045-113917720
8408	Nr1i3	12355	1+:173144166-173148975
8409	Mid1	17318	X+:166123181-166444500
8416	Cdc20	107995	4-:118105506-118109854
8421	Mbd5	109241	2+:49101451-49169245
8428	Sorbs3	20410	14-:70580275-70607444
8430	Pttg1ip	108705	10+:77044510-77061476
8432	Fgf18	14172	11-:33017438-33047400
8438	Mrpl48	52443	7-:107697222-107756816
8439	Slc25a33	70556	4-:149118583-149148292
8441	Tmem54	66260	4+:128782859-128788829
8443	Pola2	18969	19-:5940547-5964139
8444	Rdh16	19683	10+:127238209-127252895
8446	Cybrd1	73649	2+:70956108-70976909
8448	Sucla2	20916	14+:73952436-73995949
8449	Vash2	226841	1-:192771628-192803078
8456	Ftl2	14337	4+:125180618-125181709
8458	MMT00063985		X-:57979510-57980693
8464	Snapin	20615	3-:90292300-90294915
8465	BC023814	229503	3-:87727566-87734069
8467	Scfd1	76983	12+:52478565-52551094
8469	Sfrs6	67996	2+:162757263-162761082
8471	Tacstd1	17075	17+:88036047-88050467
8472	Txndc16	70561	14-:45754125-45839043
8473	Clint1	216705	11+:45665443-45724128
8477	MMT00002146	100041835	11-:95934335-95936342
8484	Cgref1	68567	5-:31235514-31247859
8486	Plod3	26433	5+:137463486-137472407
8488	Opcml	330908	9+:28620871-28727953
8494	MMT00028462		6+:67789786-67864069
8495	Lox	16948	18-:52675720-52689357
8499	C430048L16Rik	77604	4+:12016518-12073876
8500	Ddit3	13198	10+:126730766-126733340
8506	MMT00003599		4+:16173206-16177382

8507	1700054F22Rik	73398	4+:53006846-53008858
8508	Myl3	17897	9+:110666184-110672304
8510	Tbl2	27368	5+:135625593-135636005
8514	Arntl2	272322	6+:146754950-146782043
8515	Glcci1	170772	6+:8209449-8547480
8516	Plxnb2	140570	15-:88985978-88997478
8521	Ceacam1	26365	7-:26248486-26262569
8523	Ddit4l	73284	3+:137286635-137289477
8524	Cbr1	12408	16+:93608083-93610566
8525	2510006D16Rik	76799	4+:129277950-129285114
8529	MMT00070318		14+:44518941-44519459
8530	Snca	20617	6-:60681594-60779849
8533	Ptprcap	19265	19+:4154678-4156713
8534	Lhcgr	16867	17-:89140889-89191316
8541	Tubb5	22154	17-:35971751-35975076
8545	Spink12	78242	18+:44264061-44268195
8546	Vmn2r87	625131	10-:129908893-129934362
8547	Gbp4	17472	5-:105546592-105568563
8548	Tor1aip2	240832	1+:157906683-157912587
8554	Letm2	270035	8-:26688963-26708024
8555	H2-M3	14991	17+:37407206-37411431
8559	Rangrf	57785	11-:68785987-68787327
8563	Leng1	69757	7-:3612684-3617072
8564	Pld4	104759	12+:114001601-114007198
8568	Tnik	665113	3+:28308275-28569505
8574	Top1mt	72960	15-:75487464-75509199
8578	Wdr19	213081	5+:65590979-65651651
8582	Igsf10	242050	3-:59121809-59140509
8588	Notch4	18132	17+:34701274-34725441
8589	Sfrs7	225027	17-:80599425-80606625
8595	MMT00019109		X-:88531214-88531963
8597	Papss1	23971	3+:131227854-131306632
8600	Scn10a	20264	9-:119517584-119603478
8603	Slc6a3	13162	13+:73674271-73716118
8606	Cntnap5c	620292	17+:58153238-58445139
8610	Padi4	18602	4-:140301779-140330098
8617	2210412E05Rik	75690	7+:50718602-50719173
8618	EG433865	433865	5-:30120704-30121666
8620	MMT00048670		1+:146800131-146801383
8623	MMT00015399		17-:53606287-53606659
8624	Bnc1	12173	7-:89111546-89137182
8628	MMT00058602		X+:46064705-46065318
8632	Igfbp1	16006	11+:7097869-7102540
8634	MMT00047654	100041686	8-:73419313-73421275
8636	Cyp2j11	100066	4-:95961214-96015372
8637	Tnpo2	212999	8+:87561130-87579566

8638	Fkbp14	231997	6-:54527600-54543171
8639	Map1lc3b	67443	8+:124114424-124120668
8641	Slc7a2	11988	8+:41983791-42003073
8642	Serpinb9	20723	13+:33096539-33108356
8643	Mmp1b	83996	9-:7368240-7387993
8644	V1rg11	171267	7-:12947158-12948113
8648	Flnb	286940	14+:8650664-8784097
8649	MMT00072863	77652	17+:33437886-33441602
8652	Krt7	110310	15+:101242855-101258235
8653	AW124722	239570	15+:85662742-85687226
8654	Fbxl13	320118	5-:21119810-21120312
8658	Fmo2	55990	1-:164806576-164828517
8659	Psmb8	16913	17+:34335343-34338210
8660	Rragb	245670	X+:149574538-149604845
8662	Brca2	12190	5+:151325749-151372321
8664	MMT00014895		8+:125113580-125114085
8667	Plxna3	18846	X+:71574439-71589102
8670	MMT00051613		1+:46472135-46473775
8674	Gclm	14630	3+:121948863-121969397
8677	Zbtb8os	67106	4+:129013260-129031791
8679	Tiparp	99929	3+:65332331-65359440
8680	C1qtnf9	239126	14+:61391133-61399701
8683	3110002H16Rik	76482	18+:12327243-12349336
8684	Il15	16168	8-:84855524-84926444
8687	AB041803	232685	6-:31115524-31168433
8690	Cd84	12523	1+:173770734-173816561
8701	EG633640	633640	13+:120276876-120284312
8702	Mrpl30	107734	1+:37947397-37955178
8703	Gstp1	14870	19-:4035410-4037912
8705	Ahdc1	230793	4+:132621123-132633140
8706	Rab35	77407	5+:116082149-116097568
8707	AI747699	381236	19-:33657536-33664514
8708	Olfr992	258865	2-:85239758-85240688
8709	Trim58	216781	11+:58453969-58459628
8711	5430432M24Rik	73847	2-:151795135-151805865
8717	Litaf	56722	16-:10959366-10993182
8721	Tbc1d10c	108995	19-:4184355-4191049
8722	MMT00022745		X+:109579839-109580241
8724	Tgfb3	21809	12-:87397693-87419484
8727	Slc27a3	26568	3-:90189153-90193712
8728	4930438A08Rik	73988	11+:58099996-58107798
8730	MMT00008620		8+:54766414-55042434
8736	Rsbn1	229675	3+:103718307-103770783
8738	Reg4	67709	3+:98026078-98040671
8739	MMT00054598		7-:93206490-93211909
8740	Car5b	56078	X-:160417022-160465929

8742	Tmem39a	67846	16+:38562953-38591497
8743	Pkp4	227937	2+:58999877-59193260
8745	Mcm2	17216	6-:88833468-88848691
8747	Nme6	54369	9+:109735307-109745475
8748	EG625055	625055	3+:86668402-86669324
8749	Wdfy3	72145	5-:102364099-102386564
8750	1700010I14Rik	66931	17+:9181225-9201181
8752	Stk32c	57740	7-:146289537-146374372
8765	MMT00037007		
8766	Irs3	16369	5-:138084255-138086955
8767	Rgl2	19732	17+:34066910-34074340
8769	Cenpk	60411	13+:105019467-105039695
8770	Cpxm2	55987	7-:139235576-139346296
8772	MMT00042479		X-:65572813-65573103
8773	MMT00027093		14-:93593417-93594940
8776	Aox3	71724	1+:58169979-58257289
8777	1700028K03Rik	76421	5+:107963728-107977396
8782	ENSMUSG00000048	791319	3-:55181377-55195964
8783	Zfyve16	218441	13-:93257085-93300754
8784	Hgd	15233	16+:37580306-37632104
8785	Tbx15	21384	3+:99057793-99158182
8786	Rmnd1	66084	10+:5942553-5943313
8787	Sdf2l1	64136	16-:17130234-17132416
8789	MMT00059186		10+:101922990-101923547
8790	Galnt2	108148	8+:126755425-126867361
8791	Sectm1a	209588	11-:120928716-120942420
8792	Bop1	12181	15-:76283425-76307699
8794	1500011B03Rik	66236	5-:115258209-115263936
8795	EG629966	629966	11-:111474281-111542366
8797	Snrk	20623	9+:122041019-122078816
8800	Rnf213	629974	11+:119313824-119348731
8803	MMT00080648		10+:56217067-56217903
8807	Ankrd56	78088	5-:93470154-93473980
8808	RP23-136K12.4	19062	11+:75444489-75462373
8809	Tgfbr2	21813	9-:115996815-115998095
8815	Mx1	17857	16-:97668643-97684512
8816	Nfkbia	18035	12-:56590847-56593570
8817	Ptpn1	19246	2+:167757865-167802467
8822	Galntl4	233733	7-:118922826-118923004
8826	4933406C10Rik	74076	12-:33609822-33638650
8828	Slc30a2	230810	4+:133899201-133908442
8829	Kndc1	76484	7+:147113581-147127435
8836	Zmym5	219105	14-:57412164-57416618
8840	Tmem18	211986	12+:31269324-31276083
8843	Ell2	192657	13+:75899385-75909666
8844	2310037I24Rik	69612	15-:98349240-98364664

8847	Cpe	12876	8-:67071362-67076365
8849	Cachd1	320508	4+:100537582-100677150
8851	Pdcd4	18569	19+:53977958-54004340
8855	Psph	100678	5-:130271440-130285077
8856	Npepps	19155	11-:97068246-97141877
8857	Rab3gap2	98732	1+:187028120-187110624
8860	Prkab2	108097	3+:97462233-97477736
8863	Defcr5	13239	8+:22437117-22437959
8864	MMT00046672		7-:112561228-112562492
8867	Shcbp1	20419	8-:4735979-4779534
8870	Arid3a	13496	10+:79393377-79417448
8871	Gm443	242891	5+:25022106-25023795
8872	MMT00018546		12-:13442893-13444041
8881	Olfr462	258406	11-:87702461-87707424
8882	Adarb1	110532	10-:76757181-76822480
8883	Mtap4	17758	9+:109833963-109986450
8884	Plekho2	102595	9-:65402192-65420938
8887	Dpep1	13479	8+:125710141-125725124
8889	Dnahc6	330355	6-:72967598-73109292
8891	MMT00071061		13-:25673992-25675286
8893	Slc22a18	18400	7+:150659693-150685227
8896	Fasn	14104	11-:120668388-120685406
8897	BC062115	408062	10+:81523931-81614754
8900	Olfr307	258610	7-:93469534-93484904
8902	Aldh3b1	67689	19-:3913492-3923904
8904	Trerf1	224829	17+:47497941-47498909
8905	Trim67	330863	8+:127317710-127352161
8906	MMT00004929		14-:58668346-58668711
8907	Npr2	230103	4+:43644807-43664301
8909	Ryr1	20190	7-:29788362-29805289
8910	Ube4a	140630	9-:44754735-44773595
8911	Ces6	102022	8+:107257927-107265373
8915	Nob1	67619	8-:109936387-109948938
8919	BC026374	234677	8+:107655699-107674008
8920	Nol5a	67134	2+:130100160-130105048
8922	Il10ra	16154	9-:45061921-45077219
8925	MMT00026932		9+:53731827-53732372
8926	MMT00044569		4-:52373697-52375043
8931	Arf4	11843	14+:27457865-27475992
8932	C430004E15Rik	97031	2+:25118133-25125512
8933	Arpc5	67771	1+:154613782-154622705
8939	MMT00024195		15-:38505007-38506470
8945	E030030I06Rik	319887	10-:21868294-21868818
8949	Prkcd	18753	14-:31408554-31423498
8951	Cd97	26364	8-:86247268-86265068
8952	Marco	17167	1-:122371115-122401661

8957	Zfp511	69752	7+:147222272-147226499
8959	Cd209g	70192	8+:4134775-4137705
8962	Nisch	64652	14-:31984115-32004736
8964	Smad2	17126	18+:76422091-76464559
8965	Ahcy	269378	2-:154889942-154900128
8967	Acot12	74156	13+:91881125-91925753
8971	Slc18a2	214084	19+:59335641-59370495
8972	MMT00014869		19+:37944862-37945603
8980	Plekhf1	72287	7-:39005693-39012978
8981	Cacna1g	12291	11-:94269835-94335039
8986	1300010M03Rik	67998	11-:100957829-100981138
8987	F2	14061	2-:91465477-91476571
8988	1110007A13Rik	210711	7-:135841702-135883950
8991	Itm2c	64294	1+:87791139-87805263
8992	Trfr2	50765	5+:138011947-138028693
8995	Pxk	218699	14+:8930756-8997624
8996	Timp1	21857	X+:20448164-20451757
8999	Pspc1	66645	14-:57341285-57397153
9002	Col24a1	71355	3+:145171603-145214975
9005	Mapk1ip1l	218975	14+:47917964-47936462
9006	Paip1	218693	13+:120238761-120245886
9007	MMT00066154		
9010	8430419L09Rik	74525	6+:135148061-135184310
9011	Lrrk2	66725	15+:91610821-91646457
9012	MMT00017112		8-:74807637-74808055
9016	Itih2	16425	2-:10016217-10052290
9018	Usp15	14479	10-:122550299-122633979
9021	Cbln4	228942	2-:171861732-171867798
9022	Plek2	27260	12-:79989683-80007937
9026	Cep135	381644	5+:77030375-77075718
9028	Unc45a	101869	7-:87470180-87485094
9032	Jakmip2	76217	18-:43691062-43750330
9033	1810013L24Rik	69053	16+:8830190-8859008
9043	Nat2	17961	8+:70024922-70026226
9046	4921537P18Rik	70952	8+:28558141-28605422
9049	1810046J19Rik	103742	11-:98299038-98300284
9050	Msln	56047	17-:25885558-25891272
9052	A730037C10Rik	320604	3+:54943958-54960979
9053	Grm2	108068	9-:106546867-106556619
9054	Rdh7	54150	10-:127321086-127325755
9057	Spp1	20750	5+:104865210-104870062
9060	Prom1	19126	5-:44384861-44492920
9065	Reep4	72549	14+:70945050-70948740
9066	2210020M01Rik	66528	11+:115761258-115767575
9067	Tra2a	101214	6-:49193925-49214044
9068	Oip5	70645	2-:119435357-119444206

9074	MMT00003693		14+:77730240-77751187
9075	MMT00018252		1-:186434159-186453422
9077	Cnpy4	66455	5+:138628715-138635105
9078	Abpz	233090	7-:34522212-34524322
9081	EG229746	229746	3+:112105283-112106168
9082	Nrarp	67122	2+:25036542-25038845
9084	MMT00002596		6+:47314860-47354360
9087	Wdr63	242253	3-:145703485-145771002
9090	Pm20d1	212933	1+:133693979-133700704
9097	Endod1	71946	9-:14158449-14185414
9098	Rhox9	104384	X-:35252275-35254947
9100	Ms4a6c	73656	19+:11543888-11555816
9103	Igh-VJ558	16061	12-:115920819-116089829
9104	Cobra1	58202	2-:25055236-25066901
9107	Lrrc20	216011	10+:60938561-61043655
9109	BC089597	216454	10+:127303543-127314374
9111	BC038286	227298	1+:75139514-75144481
9116	M6prbp1	66905	17-:56418384-56429934
9120	Atp2c1	235574	9-:105313692-105372478
9121	Mtap7d3	320923	X-:54068701-54072116
9122	Sepw1	20364	7-:16502558-16507675
9123	Cdc42se1	57912	3+:95032669-95040331
9127	Mtfmt	69606	9+:65283588-65300209
9128	Nxph1	18231	6+:8859959-8937962
9132	Cyp7a1	13122	4-:6195359-6202718
9135	Mbd6	110962	10-:126719013-126720699
9137	Hunk	26559	16+:90386641-90499796
9140	EG623166	623166	9+:98743005-98744062
9141	MMT00028469		
9143	Akr1c14	105387	13+:4058836-4089663
9145	V1rh14	171273	13-:23205703-23206599
9146	Ergic1	67458	17+:26698503-26793874
9149	Ccdc15	245902	9-:37150020-37155974
9152	Indo	15930	8-:25694614-25707481
9154	Tfg	21787	16-:56690446-56717467
9156	Klra23	79410	6-:130253385-130256445
9157	Txnrd1	50493	10+:82321923-82360456
9158	Pde9a	18585	17+:31523223-31613101
9161	Mrpl17	27397	7-:112958097-112959581
9164	Uba5	66663	9-:103949844-103965449
9165	Nnat	18111	2+:157385858-157388239
9167	Vim	22352	2+:13495974-13504403
9172	Tmem131	56030	1-:36849038-36996032
9173	Arhgap15	76117	2+:43919268-44243139
9175	MMT00081793		1+:10795325-10796347
9181	1700060H10Rik	73390	10+:55826735-55836676

9190	Rbmxl2	76572	7+:114352930-114354436
9191	Lbxcor1	207667	9-:62986004-62987935
9192	MMT00042303		7-:17279167-17292589
9194	Dynlrb2	75465	8+:119031359-119039814
9195	Sub1	20024	15-:11911622-11925746
9200	Sdc1	20969	12+:8778201-8800491
9208	MMT00038995		6-:102886170-102887552
9212	Pde6d	18582	1-:88439590-88479035
9213	Osgin1	71839	8+:121961044-121970154
9217	Lrrc34	71827	3-:30523190-30546793
9218	Npy5r	18168	8-:69203865-69211993
9219	Mad2l1bp	66591	17-:46284337-46290485
9221	Ilf2	67781	3+:90280078-90291653
9224	MMT00013510	227885	2+:50765616-50767367
9226	Aim2	383619	1+:175382956-175395350
9230	1700027N10Rik	75564	17-:46266225-46281147
9232	4930430A15Rik	67575	2-:111033410-111069755
9233	Acss3	380660	10-:106374199-106425965
9234	Arf1	11840	11-:59025627-59041716
9235	Tnfrsf12a	27279	17-:23812413-23814411
9240	Olfr401	258701	11+:73934792-73935739
9244	Rabif	98710	1+:136391236-136404459
9245	Zc3h3	223642	15-:75587044-75609986
9251	P2ry1	18441	3+:60807332-60810924
9252	Slc22a2	20518	17+:12777054-12821353
9255	Emp3	13732	7-:53173433-53176774
9256	Psmd4	19185	3-:94836626-94846467
9258	P4ha1	18451	10+:58799791-58836050
9260	Cmtm6	67213	9+:114640320-114658461
9262	MMT00053633		3-:45578522-45580136
9263	Fbxo27	233040	7+:29383177-29484354
9270	MMT00072976		
9273	MMT00057004		16-:55325603-55325682
9275	Aass	30956	6-:23022501-23072733
9276	MMT00008480		8-:44814581-44815307
9278	Clec2i	93675	6+:128837631-128846864
9279	MMT00066808		10+:54844229-54844592
9281	Rnf122	68867	8+:32222313-32240138
9285	Mtcp1	17763	X-:72650203-72661882
9287	Trpm3	226025	19+:23066780-23067091
9290	Cd1d1	12479	3-:86799759-86803259
9298	MMT00010260		11-:121026773-121028707
9301	MMT00050551		3-:62974251-62977961
9304	AI449175	234362	8-:72134551-72149422
9305	Zfp292	30046	4-:34752099-34758805
9306	Spon1	233744	7+:120909653-121184873

9309	MMT00035904		10+:22063211-22065941
9310	Pdrg1	68559	2-:152834624-152841127
9312	Hyal5	74468	6+:24825942-24841959
9318	C79267	212632	4+:139130633-139172985
9320	Rnf31	268749	14+:56210683-56222511
9321	Vsx2	12677	12+:85910892-85936408
9322	Relt	320100	7-:107994359-108001637
9324	Egfl6	54156	X-:162960938-163023644
9326	Pgm5	226041	19-:24757505-24936279
9329	Serpina3m	20717	12+:105625401-105632468
9330	Tapt1	231225	5-:44566401-44617826
9335	Chac2	68044	11-:30876723-30886334
9338	Zfp313	81018	2+:167340879-167341664
9339	Tnmd	64103	X+:130385600-130400058
9340	Cdc42ep2	104252	19-:5917559-5924803
9343	Ildr1	106347	16+:36694123-36726827
9344	Tmem123	71929	9+:7764127-7794330
9350	Pcmtd1	319263	1+:7079126-7163706
9352	Hnrpll	72692	17-:80429682-80434896
9353	Emilin2	246707	17-:71601513-71660256
9354	Vps13b	666173	15+:35582009-35700296
9355	Reep1	52250	6-:71759350-71760705
9356	Emid2	140709	5-:137217634-137358977
9359	Dhx34	71723	7-:16782569-16807381
9360	Taf1c	21341	8-:122121877-122129083
9361	Hand1	15110	11-:57642214-57645649
9362	Gast	14459	11+:100197796-100198213
9363	Wisp2	22403	2+:163646634-163658880
9374	Thbs4	21828	13-:93521542-93564713
9376	Zscan18	232875	7-:13353442-13380337
9379	Ypel2	77864	11-:86752611-86807236
9380	Spata5	57815	3+:37319219-37478012
9385	Hmg20a	66867	9+:56266636-56344688
9392	4922505G16Rik	629499	2-:157054356-157064177
9393	Cd300a	217303	11+:114761052-114763230
9394	Cfhr1	50702	1-:141443640-141456799
9396	Grlf1	232906	7-:17232551-17233248
9401	Fcgr4	246256	1+:172949069-172959880
9402	1200016B10Rik	66875	1-:153214828-153275511
9405	Aldoc	11676	11+:78137559-78141551
9406	Calcoco1	67488	15-:102537209-102552500
9407	Cmah	12763	13-:24417640-24423491
9408	Hmgcs2	15360	3+:98084355-98114656
9409	11-Mar	211147	15+:26238841-26339328
9410	Zmym3	56364	X-:98599723-98616188
9411	Taf7l	74469	X-:130995161-131007506

9413	1190020J12Rik	233038	7-:29328621-29332280
9414	Olfr178	258999	16-:58889086-58890044
9417	Cd163	93671	6+:124254673-124280541
9418	MMT00034311		X-:48572007-48573190
9419	Acox2	93732	14-:9058025-9091450
9421	Serpinf2	18816	11-:75245237-75252977
9424	Cltc	67300	11-:86507857-86570993
9425	Gusb	110006	5-:130464891-130478698
9427	Nqo2	18105	13+:34059021-34077508
9436	Cntnap2	66797	6+:46799032-46858778
9438	Pctk3	18557	1-:134010237-134036261
9440	Zfp653	319601	9-:21859858-21875966
9446	MMT00017417		16+:4860962-4863120
9449	Sft2d2	108735	1-:167108821-167124443
9451	Gpr180	58245	14+:118536356-118562483
9454	2810426N06Rik	67607	7+:48854629-48906895
9459	Ccdc73	211936	2+:104747693-104791765
9460	Fer1l4	74562	2-:155844875-155877982
9463	Zdhhc23	332175	16-:43973580-43979241
9467	Aoah	27052	13+:20885981-21115571
9468	Gtf2a1l	71828	17+:89068044-89114488
9473	Gfra3	14587	18-:34849559-34879919
9474	Cops7b	26895	1+:88483719-88502837
9476	9130005N14Rik	68303	5+:65361352-65432382
9480	A430107O13Rik	214642	6+:21935927-22019450
9481	Ppl	19041	16-:5086823-5132514
9485	2700081O15Rik	108899	19+:7495744-7497596
9494	Cib2	56506	9-:54393158-54407886
9495	Srms	20811	2-:180940267-180947712
9504	Sfrp1	20377	8+:24522002-24558429
9505	Rps27	57294	3-:90016590-90017579
9507	Trip6	22051	5-:137751127-137755362
9510	Nt5c2	76952	19-:46963150-47036787
9512	MMT00081874		10+:110593271-110594471
9513	Tardbp	230908	4-:147990975-148000863
9514	Vps37a	52348	8+:41597154-41631914
9517	Med7	66213	11+:46250483-46255025
9519	Fstl5	213262	3+:76125975-76512555
9521	Ctnnb1	12387	9+:120842695-120868894
9522	Cidea	12683	18+:67503257-67527432
9524	Pum1	80912	4+:130219303-130337187
9528	Amelx	11704	X-:165617951-165625142
9529	Tmprss2	50528	16-:97787594-97832673
9531	MMT00043554	670496	11+:99676416-99680661
9532	MMT00050594		3+:135994449-135995957
9534	MMT00042032		6-:122515894-122517421

9535	MMT00069497		X+:38014636-38015105
9537	Sypl	19027	12+:33638832-33660587
9540	Csnk1e	27373	15-:79249596-79272432
9541	Npal3	74552	4-:135007790-135050455
9544	2410018C17Rik	74504	5-:33943000-33972275
9547	MMT00006479		12+:120849421-121115281
9552	Dnmt3a	13435	12+:3807090-3908996
9554	Hmgb2l1	70823	8+:77517580-77555876
9558	Akr7a5	110198	4+:138866669-138874334
9559	Zfpm2	22762	15+:40486817-40936131
9567	Cfl1	12631	19+:5490520-5493563
9569	Dda1	66498	8+:73993096-74000332
9570	Creld1	171508	6+:113433562-113443332
9574	Mphosph10	67973	7-:71521463-71532406
9575	MMT00011132		8+:71993967-71994296
9576	Polr1e	64424	4+:45031517-45045231
9577	Dusp8	18218	7-:149267402-149276175
9580	Tuba8	53857	6+:121160787-121176115
9582	MMT00041407		5+:138056838-138057318
9585	Neu3	50877	7-:106959950-106976927
9586	Mrpl53	68499	6+:83059101-83059926
9587	Bri3bp	76809	5+:125921966-125935506
9588	Ghrl	58991	6-:113666245-113669423
9594	Asb13	142688	13+:3633323-3651018
9597	Tnfsf13b	24099	8+:10006632-10035997
9601	Wt1	22431	2+:104966685-105013769
9610	Narf	67608	11+:121098591-121114836
9611	Ncf2	17970	1+:154655065-154683383
9616	AI451557	102084	8+:97047048-97051165
9620	Sdpr	20324	1+:51345969-51359803
9622	Rasgef1a	70727	6+:118016462-118041563
9627	Cbx6	494448	15-:79656536-79664667
9629	Spnb3	20743	19+:4718840-4752350
9630	5830417C01Rik	78825	1+:180117878-180179751
9634	Atp13a2	74772	4+:140542880-140563244
9635	Rabggtb	19352	3-:153570417-153575023
9644	Ankrd32	105377	13-:77243878-77274722
9650	Sh3gl3	20408	7+:89319745-89455919
9651	Cxxc5	67393	18+:36018186-36021341
9654	1810041L15Rik	72301	15-:84202330-84256540
9655	Sult2b1	54200	7-:52985353-53014892
9656	Cenpc1	12617	5-:86441060-86494475
9658	Abr	109934	11-:76230245-76384593
9671	AK129341	234915	9-:8076460-8134472
9672	Tmem101	76547	11-:102013862-102017675
9674	Pea15a	18611	1-:174128757-174130710

9676	Usp32	237898	11-:84797933-84953270
9678	AW555464	217882	12+:113962834-113984800
9680	Ahsa1	217737	12+:88607677-88614902
9683	Prdm8	77630	5+:98609919-98616470
9684	Phf21b	271305	15-:84617532-84686558
9685	Bcl9l	80288	9+:44316958-44318493
9686	Nek4	23955	14+:31764673-31800586
9689	4432416J03Rik	78252	9-:48126088-48148880
9696	Setd1b	208043	5+:123592965-123618638
9697	Mterf	545725	5-:3890581-3893927
9698	MMT00081969		6+:19069995-19070912
9700	Prodh	19125	16-:18071818-18089240
9701	Prkcz	18762	4-:154636185-154735352
9702	Stk39	53416	2-:68049808-68310013
9707	Golt1a	68338	1+:135206391-135218519
9710	Nrp2	18187	1+:62750054-62843547
9712	Cct3	12462	3+:88101056-88125688
9713	Ykt6	56418	11+:5855888-5866078
9715	OTTMUSG0000000045	100038514	4+:14865614-14880177
9717	Psmb9	16912	17-:34319043-34324275
9718	Fmo5	14263	3+:97432972-97456516
9721	Rrs1	59014	1+:9535522-9537532
9724	Oat	18242	7-:139749161-139761813
9725	Dok6	623279	18-:89590366-89768183
9729	Got1l1	76615	8-:28307928-28313023
9730	Olfr1501	258626	19-:13912715-13928068
9732	Iapp	15874	6+:142246947-142252337
9733	4921509E07Rik	70897	12+:79792521-79835500
9734	Wwp1	107568	4-:19536781-19605588
9736	Shisa2	219134	14+:60244681-60250495
9738	Cdk5rap2	214444	4-:69882772-69915753
9743	Gm189	235952	6-:69525779-69526494
9744	9530002B09Rik	77432	4+:122366609-122379627
9745	Gadd45g	23882	13+:51942063-51943841
9751	C1ql3	227580	2-:12923232-12933441
9755	MMT00066614	667386	5-:149774774-149781471
9756	1500015O10Rik	78896	1+:43787537-43799387
9757	Olfr502	258734	7-:115666517-115667462
9759	Cenpn	72155	8+:119445639-119465403
9762	Snx5	69178	2-:144076790-144096607
9763	Tuba3b	22147	6+:145564572-145569938
9766	MMT00050414		4+:119193594-119193965
9768	MMT00025372		18-:86410461-86412975
9770	Bcmo1	63857	8+:119619764-119657374
9773	MMT00068134		8+:107185898-107186994
9777	Cdh19	227485	1-:112785786-112826552

9779	Laptm5	16792	4+:130469208-130490946
9780	4930583H14Rik	67749	3-:51192337-51200431
9781	Prune	229589	3-:95057593-95085714
9782	Dnase2a	13423	8+:87432544-87435350
9787	Epb4.1l5	226352	1-:121491653-121545194
9790	Tspan10	208634	11+:120304006-120308263
9792	Miox	56727	15+:89164904-89167442
9798	MMT00072061		16+:97807935-97808963
9800	Phf7	71838	14-:32050881-32064404
9805	Tagap	72536	17+:8118884-8127761
9812	Atf1	11908	15+:100058499-100091674
9813	Cdh18	320865	15+:23103515-23191938
9814	Nbl1	17965	4-:138638206-138648780
9820	BC023105	207269	18+:60598948-60603504
9821	Pmaip1	58801	18+:66618257-66625212
9822	Celsr1	12614	15-:85729358-85864207
9825	MMT00007741		2+:121544265-121544901
9827	Pam	18484	1-:99717671-99992086
9829	Stoml1	69106	9+:58104435-58110325
9830	Rbks	71336	5-:31926808-32000001
9831	Nedd4l	83814	18+:65183547-65372961
9832	Brf1	72308	12-:114198255-114238612
9834	Gm1140	382217	X+:64948872-64959272
9835	Fastkd1	320720	2-:69528118-69550456
9836	Oprk1	18387	1+:5578572-5592945
9837	Gpm6b	14758	X+:162676903-162826919
9838	Hpgd	15446	8+:58773394-58799471
9839	Ift20	55978	11+:78345046-78354975
9841	MMT00050244		12+:3222416-3228151
9842	Qdpr	110391	5-:45825293-45841396
9843	Olfr642	258326	7-:111197922-111198866
9847	Ifi27	76933	12-:104680371-104681869
9849	4930445E18Rik	78115	15-:25225222-25262405
9852	Ilkap	67444	1-:93272408-93295311
9856	Tnf	21926	17-:35336337-35338941
9859	Cx3cl1	20312	8+:97296164-97306280
9860	MMT00060113		9+:38881607-38883427
9861	Serpinb6b	20708	13+:33057381-33070905
9862	AW112010	107350	19-:11122098-11125056
9867	Psrc1	56742	3+:108186760-108191080
9868	Sult4a1	29859	15-:83907820-83936057
9869	Gstt4	75886	10-:75277690-75285265
9870	Mgat2	217664	12+:70285199-70287227
9871	C1qdc2	67389	4+:155336426-155340738
9875	Cdk5rap3	80280	11-:96769100-96777795
9877	Cdc2a	12534	10-:68801052-68815664

9880	Leo1	235497	9+:75293231-75314236
9882	OTTMUSG00000005	626870	11+:8952696-8968736
9884	Hp	15439	8-:112099027-112102948
9885	4930401F20Rik	243822	7-:4704830-4709462
9886	Degs1	13244	1-:184206109-184212875
9887	MMT00062394		7+:143144480-143145309
9891	MMT00001343	74100	9-:111967762-112090230
9892	AU022252	230696	4-:118898347-118905280
9895	Nlgn1	192167	3-:25330763-25338999
9899	D13Wsu177e	28126	13-:54685551-54691435
9900	MMT00062271		14-:118774751-118775110
9903	Il22	50929	10+:117641997-117647100
9904	Hbegf	15200	18-:36664586-36675428
9906	Ninj2	29862	6+:120043367-120150356
9907	4932442K08Rik	67544	17+:15539487-15569002
9909	MMT00077883		3+:94257254-94258001
9910	EG666644	666644	1+:43327668-43359076
9912	Ttc27	74196	17+:75117088-75262909
9913	Tbc1d25	209815	X-:7731577-7753049
9918	Mki67	17345	7-:142881466-142907822
9919	Gabarapl1	57436	6+:129483464-129491184
9920	Syk	20963	13+:52678541-52744147
9921	Sec24d	69608	3+:122970384-123068560
9923	Tssk6	83984	8+:72426113-72427415
9925	Apoe	11816	7-:20281619-20283579
9926	Snx18	170625	13-:114382392-114408753
9927	Creb3l2	208647	6-:37305642-37392129
9928	Lgmn	19141	12-:103632308-103677907
9930	Phyhip	105653	14+:70857337-70868627
9931	Glt25d1	234407	8+:74134896-74148808
9934	MMT00002304		X-:13504412-13506637
9935	Prnd	26434	2+:131776597-131781865
9938	MMT00012610		19-:13176870-13177756
9939	Pcnt	18541	10-:75847607-75905657
9944	Cntfr	12804	4-:41604529-41644072
9946	Stam2	56324	2-:52550083-52601198
9951	Rpp21	67676	17-:36392617-36394791
9954	MMT00047280		X-:23931485-23931605
9955	Psmg2	107047	18+:67801252-67813816
9962	Krt23	94179	11-:99339287-99354424
9963	Rcn3	52377	7-:52338283-52347529
9968	Eif3s10	13669	19-:60842234-60851681
9970	MMT00082622		2+:40411263-40412198
9972	Aicda	11628	6+:122503835-122514197
9973	Tmem5	216395	10-:121517989-121534226
9976	Trnt1	70047	6+:106719130-106732466

9979	Myo1b	17912	1-:51806832-51964160
9980	MMT00008758		1+:98542150-98543132
9981	Snrpn	20646	7-:67128007-67133633
9982	MMT00002004		1+:12555611-12556670
9984	EG215895	215895	10-:33487976-33499645
9985	D16Ertd519e	52645	16+:70618318-70625069
9990	MMT00081303		5-:33773383-33774239
9994	Cd209b	69165	8-:3918483-3926757
9996	Rassf9	237504	10+:101974918-102008830
9997	Nr0b2	23957	4+:133109339-133112150
9998	Hfm1	330149	5-:107269612-107274495
9999	Zswim3	67538	2+:164630598-164647626
10002	Krt79	223917	15-:101759764-101770755
10003	B4galt7	218271	13+:55701471-55711314
10006	Ephx2	13850	14-:66703210-66743289
10007	MMT00033070		2-:97788230-97789852
10008	Etf1	225363	18-:35064135-35091584
10011	Brp44	70456	1+:167391338-167411345
10014	MMT00041851		
10017	Wdr73	71968	7-:88035610-88046123
10024	MMT00000014		17-:50366966-50367519
10025	A130082M07Rik	100038850	14+:54787766-54843877
10026	Ndufc2	68197	7+:104548601-104556304
10027	Mcm9	71567	10-:53256174-53283263
10028	Chchd6	66098	6-:89333149-89545631
10029	Trak1	67095	9+:121273118-121384034
10032	Sele	20339	1+:165978364-165987808
10035	MMT00041814		8+:38217794-38218856
10037	Usp51	434872	X+:149442688-149443965
10040	MMT00080694		4+:60449061-60453064
10042	MMT00028111		8-:100747156-100748701
10050	Olfr154	27216	2-:85503634-85504589
10052	6530404N21Rik	67795	19-:33212236-33277138
10053	MMT00002151		14+:67587971-67588594
10054	Fsip1	71313	2-:118030623-118082690
10055	Tpte	234129	8+:23438115-23476478
10056	Cmtm3	68119	8+:106864586-106871571
10057	Sult6b1	73671	17-:79284235-79306332
10058	Ahsg	11625	16+:22892114-22899511
10061	Nsd1	18193	13+:55417058-55419683
10065	9030625A04Rik	210808	14-:77424008-77430769
10072	Gipc2	54120	3-:151756806-151828864
10073	2010321M09Rik	69882	9+:64808690-64834780
10077	Cep63	28135	9-:102488914-102528545
10078	Tbc1d14	100855	5-:36835182-36928876
10080	Top2b	21974	14+:17198146-17263301

10081	Fth1	14319	19+:10057192-10059582
10084	Rbm7	67010	9-:48296813-48303410
10087	Bmp3	110075	5+:99284131-99309299
10088	Ddc	13195	11-:11714445-11780653
10092	Cdh7	241201	1+:111880220-112035766
10093	Olfr985	258854	9-:39934609-39935544
10094	MMT00022290		13-:65524617-65833379
10095	Ranbp2	19386	10+:57909727-57956714
10098	2610507B11Rik	72503	11+:78075293-78093420
10099	Smyd3	69726	1-:180887389-181448165
10100	Mterfd2	69821	1-:95197806-95201342
10102	Lman2l	214895	1-:36480029-36502105
10106	Bglap-rs1	12095	3-:88172543-88173478
10108	Ctsh	13036	9+:89949346-89970927
10109	Gbp3	55932	3+:142223571-142236171
10112	Kcns1	16538	2-:163990041-163996825
10115	Ptk2	14083	15-:73156178-73161446
10116	Mettl7b	71664	10-:128395332-128398044
10121	Esrrg	26381	1+:189821815-190035141
10127	Tpm1	22003	9-:66875702-66896975
10130	MMT00043239		5-:126505516-126506821
10131	Gdap1	14545	1+:17135465-17154094
10132	Ces2	234671	8+:107370985-107378367
10133	MMT00047183		7-:118530306-118530846
10134	Hivep3	16656	4+:119406376-119545933
10136	RP23-149D11.4	381535	4+:101581725-101582688
10139	Hdhd1a	67365	18-:50727309-50728324
10140	Cdc2l6	78334	10+:40020193-40203623
10142	5830405N20Rik	67596	10+:129784191-129799697
10143	Uchl1	22223	5+:67067526-67078472
10147	Itgax	16411	7+:135273081-135294170
10150	Oxct1	67041	15+:3976723-4103963
10156	Htr2c	15560	X+:143397188-143630220
10164	2310004N24Rik	69535	11+:116060194-116076631
10166	Rhbdd2	215160	5+:136108591-136120016
10173	Nck1	17973	9-:100395796-100446553
10176	Snrpg	68011	6+:86321537-86328896
10177	Osgep	66246	14-:51535358-51544503
10183	Tax1bp1	52440	6+:52663741-52716478
10189	Cage1	71213	13-:38097917-38108361
10190	Pcdh7	54216	5+:58109338-58120686
10195	Klf1	16596	8+:87425826-87429186
10199	MMT00050019	675135	1-:58918096-58933287
10203	Cyp4f16	70101	17+:32673573-32688741
10207	MMT00012324	328192	13+:16122413-16123441
10209	Plin	103968	7-:86866051-86877780

10210	Sgms1	208449	19-:32198262-32285470
10214	Sectm1b	58210	11-:120915844-120924852
10215	Nampt	59027	12+:33505339-33536472
10218	Vps41	218035	13+:18815054-18958681
10222	AF366264	231201	8-:13836581-13838059
10223	MMT00013190	236598	
10228	MMT00082629		8-:81010016-81010310
10229	Ripk1	19766	13+:34101477-34124881
10230	Yme1l1	27377	2+:23012055-23052877
10233	Abi3bp	320712	16+:56477962-56562602
10234	C230093N12Rik	98952	2+:32390814-32423648
10236	EG665033	665033	9-:105759273-105791705
10240	Cyp2w1	545817	5+:139828600-139833178
10245	Dus1l	68730	11-:120650515-120656813
10247	V1rc20	171193	6+:56952348-56953258
10248	Cwf19l2	244672	9+:3403830-3430474
10250	Pcsk9	100102	4-:106115335-106126971
10255	Muc16	73732	9-:18299897-18323792
10256	Gmds	218138	13-:31911458-32430413
10259	Kcnmb4	58802	10-:115854923-115910579
10260	Zfp36l2	12193	17-:84585332-84586477
10267	Setdb2	239122	14-:60020844-60059618
10273	Gpt2	108682	8+:88016483-88051465
10274	MMT00076184		9+:40295245-40295903
10275	Trim12	76681	7-:111452110-111463977
10276	Dcun1d1	114893	3-:35794141-35831888
10278	Dusp6	67603	10+:98725864-98730118
10279	Rnf186	66825	4+:138523118-138524274
10281	Slc15a1	56643	14-:121858842-121904475
10292	Cobl	12808	11-:12136680-12364750
10298	Olfr1015	258564	2+:85625669-85626607
10299	Rad52	19365	6+:119852731-119872840
10300	Igfbp4	16010	11+:98902810-98912892
10302	Rab18	19330	18+:6765258-6788718
10303	Cyp2a22	233005	7-:27716650-27724409
10308	Fuk	234730	8-:113406369-113413190
10311	Cpox	12892	16+:58670404-58680473
10318	Cdk8	264064	5+:147043070-147114448
10321	Gabra3	14396	X-:69679944-69901488
10327	Hint2	68917	4-:43667100-43669320
10328	Gpr182	11536	10-:127186677-127188749
10332	Gnrhr	14715	5-:86611036-86626895
10337	Zmym1	68310	4-:126724836-126726950
10340	Hc	15139	2-:34838850-34916916
10343	Malat1	72289	19-:5801054-5802672
10345	Camkk1	55984	11+:72832554-72855565

10351	Clcn4-2	12727	7-:7236790-7252042
10353	Matn4	17183	2-:164215162-164230037
10356	2810417H13Rik	68026	9+:65738235-65749410
10357	Tm4sf1	17112	3-:57091304-57098795
10359	Foxf2	14238	13+:31717647-31723271
10360	Nrxn2	18190	19+:6418943-6533208
10365	Aplp1	11803	7-:31220007-31230571
10366	4931403E03Rik	75718	4-:138124983-138179928
10367	Coq7	12850	7-:125668576-125676827
10370	MMT00017441	623205	11+:53014185-53014559
10373	Tmem141	51875	2-:25464153-25477523
10374	Trim15	69097	17-:36997637-37004155
10376	C920025E04Rik	667803	17-:36264613-36264812
10377	A430105I19Rik	214239	2-:118583232-118585443
10380	MMT00018157		X-:143033206-143035525
10382	Mtmr4	170749	11+:87405718-87429798
10384	Chic2	74277	5-:75402448-75440641
10387	4933427D06Rik	232217	6+:89046112-89058002
10393	Slc7a8	50934	14-:55341052-55400378
10394	MMT00028435		7-:48581594-48582685
10395	Abcg2	26357	6+:58605631-58642416
10396	MMT00072312		6+:39156261-39157552
10397	Gtf2e2	68153	8+:34846203-34887452
10402	Mia1	12587	7-:27964765-27966169
10406	Trip10	106628	17+:57388883-57402845
10407	Gpatch3	242691	4+:133138382-133140157
10408	Per1	18626	11+:68914143-68922861
10413	Olfr487	258042	7-:115355096-115394259
10415	Dcdc2a	195208	13+:25148072-25294336
10416	Eaf2	106389	16-:36793830-36828334
10421	MMT00016905		11+:53459332-53459823
10426	Ly6f	17071	15+:75098850-75102555
10434	Ppp2r5a	226849	1-:193175864-193180833
10435	Nab2	17937	10-:127097974-127103734
10437	MMT00062592		X-:51119052-51123209
10442	Pla2g4a	18783	1-:151676758-151808362
10443	Pvrl2	19294	7-:20302018-20334651
10444	1810020D17Rik	66273	7-:104698852-104727989
10447	Dynll2	68097	11-:87793034-87794338
10448	Rac3	170758	11+:120582914-120585283
10450	C330006K01Rik	231855	5+:142950827-142953758
10452	Grn	14824	11+:102294285-102297784
10455	MMT00015250		X+:150217937-150218848
10457	Olfr853	258908	9-:19341454-19342372
10462	MMT00070759		7-:12565016-12569261
10465	BC023892	212943	9-:85217149-85220900

10466	S1pr5	94226	9-:21047360-21050653
10467	MMT00074572	100038888	5+:143419271-143435261
10468	Tspan33	232670	6+:29644255-29668558
10470	Zmym4	67785	4-:126539069-126588529
10471	Igh-VJ558	16061	12-:116930076-116930533
10475	Ccna2	12428	3-:36463787-36470860
10482	Mcat	223722	15-:83377225-83379765
10483	Crot	74114	5-:8966047-8997144
10484	Dnajb11	67838	16+:22869210-22872439
10486	1300002K09Rik	74152	4+:45861818-45899880
10487	MMT00016719		3-:93517601-93518479
10489	Aldh1b1	72535	4+:45811998-45817473
10490	Peg3	18616	7-:6660217-6664362
10491	Acpl2	235534	9-:96722838-96789843
10492	1810046K07Rik	69809	9-:51097798-51137024
10497	Man2b1	17159	8+:87607228-87621415
10498	Ube2z	268470	11-:95908740-95926641
10499	Camkk2	207565	5-:123183212-123214303
10502	Axud1	215418	9-:119880282-119883533
10503	MMT00043055		10+:31358888-31360169
10504	Ripk3	56532	14-:56403833-56407623
10511	Tmem68	72098	4-:3476389-3501947
10519	MMT00061955		5-:66111856-66113448
10520	Xkr6	219149	14+:64413856-64438627
10521	Ttf2	74044	3-:100742784-100773576
10525	MMT00036668		X-:136980119-136980473
10526	MMT00036239		7-:61861136-61866491
10528	Pip5k1b	18719	19-:24369369-24518175
10531	Mlana	77836	19+:29772311-29782937
10532	MMT00057790		X-:114777483-114778383
10539	Unc45b	217012	11+:82725180-82756641
10541	Ap2a2	11772	7+:148748107-148817541
10542	Hao3	56185	3-:98678630-98697149
10544	Gdpd1	66569	11-:86847369-86887593
10545	Tmem147	69804	7-:31512734-31514502
10546	Prpf6	68879	2+:181336096-181390365
10550	Rchy1	68098	5-:92377867-92391790
10553	Dusp23	68440	1-:174560901-174563129
10554	ENSMUSG00000074l	100042314	9+:78137728-78153330
10558	Cyp2d40	71754	15-:82590264-82594613
10561	Eln	13717	5-:135179582-135223111
10568	Rage	26448	12-:112046008-112079149
10574	MMT00007972		6+:67741039-67741528
10576	Xrcc3	74335	12-:113041403-113052052
10582	Psmg3	66506	5-:140299547-140302781
10591	Pmp22	18858	11+:62945013-62973048

10593	Wdr53	68980	16+:32247617-32257166
10596	Nfrkb	235134	9+:31193719-31228921
10598	MMT00011231		X-:162920153-162921549
10606	Apbb1ip	54519	2+:22629960-22731172
10610	Nbeal1	269198	1+:60307878-60388635
10612	Slc24a6	170756	5+:120961200-120984028
10621	4833420G17Rik	67392	13+:120251565-120258567
10629	BC021614	225884	19-:4057486-4059294
10634	Kitl	17311	10+:99478457-99563045
10635	Itfg2	101142	6-:128359462-128374928
10636	Mal2	105853	15+:54403040-54434395
10638	Gpr97	54672	8+:97564185-97569143
10639	Tgfbr3	21814	5-:107535590-107718704
10641	Hpdl	242642	4-:116492508-116493928
10642	Ngef	53972	1-:89373412-89406921
10643	Gc	14473	5-:89846537-89886926
10650	Atf7	223922	15-:102367084-102455852
10651	2610044O15Rik	72139	17-:95214567-95233779
10656	2900092C05Rik	73090	7+:13097865-13141665
10657	Rnf12	19820	X-:101157466-101176587
10658	Macrod2	72899	2+:142035834-142060074
10659	ENSMUSG0000003 8 5	100038725	10-:52997850-53099484
10663	Sytl2	83671	7+:97497208-97558948
10667	H2-Eb1	14969	17+:34442886-34453144
10668	Kars	85305	8-:114517343-114535168
10671	Asz1	74068	6-:18021960-18059036
10672	BC031353	235493	9+:74800803-74880259
10673	MMT00071544		X+:117080065-117081181
10674	Antxr2	71914	5-:98313707-98459568
10675	Mobkl2a	208228	10-:80147456-80164582
10681	Dgkz	104418	2-:91772978-91803812
10684	Hmgb1	15289	5-:149860314-149861889
10686	MMT00001732		3-:11658663-11659505
10687	Itpkb	320404	1+:182262242-182352541
10689	Pitpnb	56305	5+:111759814-111815537
10700	Grwd1	101612	7-:53080584-53086157
10710	Smoc1	64075	12+:82127817-82287387
10711	1700108F19Rik	73272	14+:77077372-77086911
10712	Fchsd2	207278	7+:108257309-108432912
10713	Tnfaip8l3	244882	9-:53874882-53916174
10717	Ranbp9	56705	13-:43498750-43576342
10723	4930556N09Rik	75301	10+:96498493-96523693
10727	Klra3	16634	6-:130273303-130287644
10729	Fgd2	26382	17+:29497916-29516480
10732	Iars2	381314	1-:187126586-187153266
10733	Kctd6	71393	14+:9046659-9056081

10734	Limk1	16885	5-:135131922-135164271
10735	Cox6a2	12862	7-:135349127-135349866
10736	Cyba	13057	8-:124948675-124956827
10741	MMT00044006		2+:21989389-21990730
10745	Gale	74246	4+:135519666-135524096
10747	Abcf2	27407	5-:24070271-24083143
10751	Eif5	217869	12+:112776652-112782827
10752	Dnajc5b	66326	3+:19408625-19510857
10758	Gdf1	14559	8+:72839685-72855486
10759	Rbms1	56878	2-:60590796-60800976
10765	Retnla	57262	16+:48842903-48844574
10767	Plscr2	18828	9+:92170673-92192544
10769	Hnrnpd	11991	5-:100390025-100407572
10770	Sell	20343	1+:165992234-166010802
10772	Aacs	78894	5+:125956242-125997773
10774	Ehd3	57440	17+:74154211-74181428
10775	Slc16a1	20501	3+:104442606-104461160
10780	Fcgr2b	14130	1-:172890716-172906164
10782	4933403O08Rik	71030	X+:109276083-109284377
10785	Ctdspl2	329506	2+:121782188-121839391
10788	D430019H16Rik	268595	12+:106692172-106726651
10790	4930470H14Rik	75815	17-:4044656-4082095
10796	Spic	20728	10-:88138016-88145752
10797	Mageb2	17146	X+:88489482-88578695
10802	Kcnn2	140492	18+:45719807-45845536
10803	Wnt5a	22418	14+:29338328-29340631
10806	Rnf212	671564	5-:109158389-109178310
10808	Rab7l1	226422	1+:133763868-133769462
10812	9330101J02Rik	664857	7-:146845410-146847069
10815	Cpe	12876	8-:67071354-67171837
10816	Fcna	14133	2-:25480188-25483386
10817	Ankrd10	102334	8-:11611582-11635689
10820	Dsel	319901	1-:113755758-113759391
10821	Pkd2l2	53871	18+:34569076-34602443
10822	E330016A19Rik	214763	9-:78283247-78290696
10824	Vegfa	22339	17-:46155491-46168392
10827	MMT00063568		2-:87040084-87041485
10829	Chga	12652	12+:103793178-103803237
10830	Rgs21	624910	1-:146366821-146384126
10831	Dmn	233335	7-:74877489-74905060
10835	Kcnd2	16508	6+:21166298-21677340
10836	MMT00002878		6-:47753957-47754114
10838	Spdef	30051	17-:27851393-27865834
10842	Slc25a25	227731	2-:32270605-32279523
10843	Ccar1	67500	10-:62206677-62255110
10844	Erap1	80898	13+:74783616-74828267

10848	Gpbp1	73274	13-:112215891-112280324
10849	9330159F19Rik	212448	10+:28931498-28947150
10851	Tax1bp3	76281	11+:72990793-72995500
10852	Sgol1	72415	17-:53815384-53828637
10853	MMT00077065		14+:41262450-41262749
10862	Zfp703	353310	8+:28087804-28091935
10863	Tssc1	380752	12+:29436684-29552361
10865	Cd248	70445	19+:5068119-5070637
10869	Hao1	15112	2-:134323097-134380088
10871	EG434197	434197	7+:75420881-75503087
10876	Steap1	70358	5-:5736321-5749309
10878	Tsnax	53424	8+:127536896-127558089
10879	Slc9a6	236794	X+:53863029-53917399
10880	Klra8	16639	6-:130065236-130079897
10881	Uchl5ip	73738	X-:70682655-70704368
10883	Pols	210106	13-:69636823-69646016
10884	Add2	11519	6+:86032773-86068819
10885	Bri3	55950	5+:145016176-145025263
10886	Fndc3b	72007	3-:27360597-27541971
10888	Eya2	14049	2+:165455841-165597226
10889	Adamts15	235130	9-:30706738-30729822
10891	Atp6v1a	11964	16-:44087536-44116981
10894	Ngfrap1	12070	X+:132804878-132806494
10901	2810051F02Rik	72704	17+:12934239-13008423
10902	Orc3l	50793	4-:34518044-34562173
10908	Hipk4	233020	7+:28308535-28314817
10910	Cdc25a	12530	9+:109778493-109795283
10911	Mcl1	17210	3+:95462730-95467099
10916	Gm973	381260	1+:59663659-59686991
10919	EG624446	624446	3-:28663354-28664314
10920	Mdn1	100019	4+:32758490-32766544
10924	Actr6	67019	10-:89174717-89195040
10925	Cacnb1	12295	11-:97866239-97883952
10926	P2ry13	74191	3-:59011821-59014805
10928	Arid1a	93760	4-:133234924-133278954
10931	Pkn3	263803	2+:29933255-29946533
10932	Olfr578	259119	7-:110132736-110133676
10935	Chpt1	212862	10-:87951126-87966663
10938	Zbtb5	230119	4-:45005867-45025240
10940	Dcun1d1	114893	3-:35794141-35831888
10941	Pcdh18	73173	3-:49547422-49561184
10946	Ifitm7	74482	16-:13982891-13984714
10949	Smek2	104570	11+:29072995-29119129
10954	Ubp1	22221	9+:113840769-113886120
10955	Cdkn2a	12578	4-:88920540-88940469
10957	Pgc	109820	17+:47863790-47871431

10960	Rap2a	76108	14+:120877692-120878962
10961	Pgd	110208	4-:148524102-148540777
10964	Cldn23	71908	8-:36887782-36889533
10965	Ppp1cb	19046	5+:32761426-32796085
10972	Lrig1	16206	6-:94554527-94650139
10974	Vmn2r25	545874	6-:123775203-123803157
10978	MMT00078322		6-:93599341-93599899
10986	Pappa	18491	4+:64785865-65013191
10994	EG625253	625253	X-:153978644-153979688
10995	Ap4m1	11781	5+:138613407-138619910
10998	2310061C15Rik	66531	8-:119413417-119445354
11001	Pou6f2	218030	13-:18453979-18480108
11002	Smg5	229512	3+:88140232-88166259
11009	MMT00008885	330256	6-:3283189-3291900
11012	MMT00012665		19+:13449453-13450378
11013	Zfand2b	68818	1+:75165319-75168199
11014	Hibadh	58875	6-:52496224-52590294
11016	Pcp2	18545	8-:3623376-3625252
11017	Olfr926	258811	9+:38684762-38685688
11023	Lcn12	77701	2-:25346365-25348950
11024	MMT00049098		10-:126469961-126478497
11025	Cd244	18106	1+:173489499-173507478
11027	Prkar2b	19088	12-:32643343-32746105
11034	Kcnh2	16511	5-:23825406-23857422
11038	Atf7ip2	75329	16+:10192994-10242047
11040	4933425B07Rik	74458	14-:47222478-47257099
11041	Vmn2r92	627111	17+:18304214-18308349
11043	Hsph1	15505	5-:150419419-150438793
11050	2210010C04Rik	67373	6-:40981633-40985477
11051	A130042E20Rik	216119	10-:75930901-75931581
11054	Rnase4	58809	14+:51710846-51725817
11056	Surf4	20932	2-:26775558-26788947
11057	MMT00032844	353287	8-:113593397-113604831
11060	Gas2	14453	7+:59134519-59249828
11063	Phf8	320595	X+:147955721-148068407
11065	Mccc1	72039	3-:35858760-35899521
11071	Pcbp1	23983	6-:86474492-86475940
11074	Lpgat1	226856	1+:193542327-193608139
11075	Xrcc6bp1	68876	10-:126324304-126338404
11083	Kctd3	226823	1-:190794977-190831720
11084	AF529169	209743	9-:89485561-89517946
11086	Pnpla5	75772	15-:83942786-83953608
11087	Wnt11	22411	7+:105987354-106002261
11090	Lycat	225010	17+:73457353-73589860
11093	Zdhhc1	70796	8-:107996324-108020802
11094	Slc17a3	105355	13+:23931346-23950219

11095	Bag3	29810	7+:135667363-135689910
11096	Olfr654	258377	7+:111736268-111737293
11098	Casr	12374	16-:36493782-36562220
11099	Klhdc8b	78267	9-:108349964-108363912
11102	Gtf2h2	23894	13-:101238863-101260086
11104	MMT00014375		4+:69708524-69709268
11105	Atp11c	320940	X-:57476463-57668102
11108	Ltbp1	268977	17+:75404907-75790666
11109	AI931714	102182	8+:80087148-80105245
11112	Olfr131	258867	17-:38218978-38219921
11116	Olfr11	218066	13-:21730450-21748040
11119	Frmd4a	209630	2+:4352241-4535076
11120	Elf1	13709	14+:79915486-79982282
11121	Mtmr11	194126	3+:95965934-95975636
11125	Trim39	79263	17-:36395817-36408949
11128	Aars	234734	8+:113570866-113579468
11129	Ankhd1	108857	18+:36751100-36825562
11130	Slco1c1	58807	6+:141473070-141518237
11134	Tcf7l2	21416	19+:55816821-56006583
11137	0910001A06Rik	223601	15-:63762498-63829524
11145	Dctn3	53598	4-:41661981-41670164
11147	Gng12	14701	6+:66846416-66967923
11148	Htra4	330723	8-:26135400-26149395
11149	MMT00050488		17-:5110751-5111950
11153	1810063B05Rik	67892	8+:128946409-128950654
11154	MMT00037611		5-:92189250-92190710
11156	MMT00004568		12-:116293410-116293867
11161	Gpatch2	67769	1+:189039425-189058218
11162	Hkdc1	216019	10-:61845886-61885205
11163	1700011H22Rik	69314	4-:91811029-91813927
11164	Odf4	252868	11-:68735337-68740551
11166	Uqcc	56046	2-:155672642-155755767
11167	Dars2	226539	1-:162970732-163000787
11168	1700019H03Rik	228993	2+:180460043-180476982
11169	MMT00078034		11+:107052394-107052843
11170	Cpb1	76703	3-:20148982-20174638
11183	Pank3	211347	11+:35582985-35600014
11186	Prkg1	19091	19-:30638998-30639748
11193	Tsc22d1	21807	14+:76904337-76907569
11195	Fmn1	14260	2+:113167938-113550012
11196	Slco1b2	28253	6+:141578038-141635156
11198	Gbp1	14468	3+:142163517-142282131
11199	Sirt7	209011	11-:120479680-120486303
11201	Sunc1	194974	11-:8916056-8928120
11204	AI451617	209387	7-:111618529-111656339
11208	Hspb1	15507	5+:136363928-136365307

11211	Tctex1d4	242646	4+:116799431-116801332
11212	1300018I17Rik	72325	8-:125766256-125778010
11214	MMT00049207		1-:10544093-10545078
11218	Apc	11789	18+:34420676-34478258
11219	Mrps18a	68565	17+:46247952-46265855
11220	AU021092	239691	16-:5211736-5222387
11231	B2m	12010	2+:121973421-121978607
11232	Ch25h	12642	19-:34548272-34549625
11233	Tef	21685	15+:81641909-81654413
11234	Pygl	110095	12-:71291798-71328604
11235	Itga2b	16399	11-:102314610-102331058
11237	Mycbpap	104601	11-:94362661-94382759
11240	Ppm1d	53892	11+:85124974-85159806
11246	Apex1	11792	14+:51544695-51546813
11248	Glt1d1	319804	5+:128131052-128187392
11255	Srpx	51795	X-:9616033-9694713
11256	H2-DMa	14998	17+:34272615-34275480
11257	Trem3	58218	17+:48386943-48398207
11259	Ccdc69	52570	11-:54863232-54891630
11260	Frem1	329872	4-:82561985-82586681
11264	Zcwpw1	381678	5+:138237799-138263881
11265	Irgm	15944	11-:48678751-48684848
11267	Cd79b	15985	11-:106172655-106175984
11274	Srp54a	24067	12+:56181514-56214240
11275	Fanci	208836	7+:86537238-86595150
11281	Foxj1	15223	11-:116192016-116195998
11282	Tekt1	21689	11-:72158225-72175375
11283	Uroc1	243537	6+:90283299-90314540
11285	MMT00045951		14+:54580667-54581493
11291	1110018H23Rik	68509	17+:25259638-25262158
11292	Nr2f6	13864	8-:73898023-73905283
11301	Csrnp3	77771	2+:65787015-65861515
11303	Slc5a12	241612	2+:110437461-110481058
11305	Galntl2	78754	14+:32842030-32875382
11306	1700016G14Rik	193386	13+:24833101-24838460
11308	Cmbl	69574	15+:31498589-31519874
11311	Dusp21	73547	X+:17723074-17723642
11312	2210016H18Rik	70125	18+:11016862-11017825
11313	Vav3	57257	3+:109143930-109487231
11315	Dok3	27261	13-:55624595-55629899
11321	2310014L17Rik	381845	7+:13512767-13515332
11330	MMT00024873		2-:19887192-19888317
11332	4931432M23Rik	70993	8-:98082962-98099160
11333	Gyk	14933	X-:82948912-83021924
11338	Vamp8	22320	6-:72335372-72340613
11339	Eef1g	67160	19+:9041694-9052666

11340	Syn2	20965	6+:115084919-115225094
11345	Slc40a1	53945	1-:45964928-45982378
11348	Med20	56771	17-:47757558-47759119
11350	9030420J04Rik	71544	9-:9007965-9048974
11355	Csta	209294	16-:36122257-36131328
11356	Med27	68975	2+:29202355-29380308
11357	4930558O21Rik	68307	3+:30543428-30571286
11359	Clstn2	64085	9-:97345274-97933534
11361	Myl2	17906	5+:122550996-122556861
11369	Stx3	20908	19-:11849607-11893761
11370	Prm2	19119	16-:10791473-10792190
11371	Amhr2	110542	15+:102275850-102285061
11375	Derl2	116891	11-:70823427-70832744
11376	Slc7a11	26570	3+:50173828-50174687
11378	Ubl3	24109	5-:149316233-149364209
11379	Dnttip1	76233	2+:164571514-164593717
11382	Cdca3	14793	6+:124780121-124783313
11385	MMT00081444		12-:3521591-3523555
11388	Tdrd9	74691	12+:113278635-113290222
11391	2310016C16Rik	69590	13-:113832970-113836596
11392	AU021838	328099	12+:58331329-58598179
11393	A830007P12Rik	227612	2-:25048485-25052246
11395	Tcea1	21399	1+:4847983-4886725
11397	Zp2	22787	7-:127275892-127288804
11401	Ramp3	56089	11+:6558539-6577477
11403	2210016F16Rik	70153	13-:58482608-58486790
11404	Gnpat	14712	8+:127386966-127413803
11406	Ifi203	15950	1-:175787343-175805910
11409	Phf16	382207	X+:20039237-20094914
11411	Odf1	18285	15+:38148957-38156393
11412	Abcg5	27409	17-:85057574-85082263
11416	Ddr2	18214	1-:171903413-171905635
11419	Atrnl1	226255	19+:58116810-58207823
11421	MMT00034261		4+:150221175-150221376
11422	Pltp	18830	2-:164665017-164683113
11423	6720463M24Rik	77744	14+:99445437-99473757
11424	Iqcf3	68265	9-:106445718-106463956
11425	Evx2	14029	2-:74493672-74497476
11428	4932438A13Rik	229227	3+:36864442-36887237
11429	Lama2	16773	10-:26701086-27187181
11432	Dynll2	68097	11-:87794585-87801001
11435	Rnaset2a	100037283	17-:8321462-8340653
11436	Mapre1	13589	2+:153567092-153592996
11437	Gatm	67092	2-:122420611-122437013
11440	Cox8c	75483	12+:104137504-104138746
11444	Rnpep	215615	1-:137159289-137180611

11445	Atp1a2	98660	1-:174153650-174228133
11446	Dock8	76088	19+:25248778-25276668
11447	Nr1h4	20186	10-:88917122-88969372
11448	Arl1	104303	10+:88194139-88206827
11449	Ppap2a	19012	13+:113591130-113657850
11453	Adc	242669	4-:128608484-128638804
11459	Mas1	17171	17-:13034426-13035400
11462	Gpam	14732	19-:55142136-55201706
11463	MMT00057138		13-:71761731-71762275
11464	Gnat3	242851	5+:17468364-17525485
11465	Eif3k	73830	7-:29756391-29766833
11467	Ypel5	383295	17+:73193373-73198143
11472	Ckmt2	76722	13-:91992992-92016471
11475	H2-D1	14964	17+:35400836-35400949
11481	Morn1	76866	4+:154460708-154519615
11486	Tacc3	21335	5+:34001309-34014844
11500	3300001P08Rik	67684	11-:94152388-94183276
11503	Rangap1	19387	15-:81534683-81560305
11504	Tpm3	59069	3+:89883653-89903532
11506	Rfxank	19727	8-:72656386-72663012
11507	Adarb2	94191	13+:8202394-8756688
11513	Serpinf1	20317	11-:75223532-75236013
11514	Muc3	666339	
11515	EG544707	544707	10+:74814968-74815776
11516	Sfxn5	94282	6-:85165482-85283416
11519	Slc2a10	170441	2+:165329479-165345409
11521	Dnaja1	15502	4+:40669585-40680933
11522	Igbp1b	50540	6-:138605610-138606965
11525	MMT00048506		10+:126330168-126330965
11534	Ndufs4	17993	13-:115078858-115178251
11536	Igj	16069	5-:88949540-88956809
11540	Ctps	51797	4-:120212470-120242869
11542	Taz	66826	X+:71528106-71535487
11545	Ppcs	106564	4-:119091137-119094996
11549	Depdc6	97998	15+:54943878-55084334
11551	MMT00046383		13+:20840333-20840705
11557	Blnk	17060	19-:41003664-41068548
11560	MMT00001695		5-:76455924-76456824
11565	Serpina3c	16625	12-:105385116-105392082
11571	Bpnt1	23827	1+:187156079-187180664
11574	Gne	50798	4-:44049989-44079790
11576	Gm1381	384198	5+:92984096-93019689
11581	Cast	12380	13-:74830741-74946257
11585	Bdp1	544971	13-:100787951-100873989
11586	MMT00070266		18+:10262204-10263318
11587	Syt14	329324	1-:194723408-194853211

11588	Ncapg2	76044	12+:117664318-117702003
11590	Cbx4	12418	11-:118941470-118947453
11591	Vil1	22349	1+:74455979-74482088
11593	Usf1	22278	1+:173341811-173348871
11596	Zfp120	104348	2-:149941955-149962414
11598	MMT00031533	628236	19-:33484689-33492127
11599	Coq10b	67876	1+:55109714-55129543
11606	Mast4	328329	13-:103523408-103643964
11610	Hey1	15213	3-:8663358-8666986
11611	S100a13	20196	3+:90318921-90328496
11614	Sult1e1	20860	5-:88004994-88020619
11620	Zfr	22763	15+:12047607-12114218
11621	Mixl1	27217	1-:182624750-182627143
11627	MMT00076264	278676	14+:52423159-52425266
11628	Slc6a12	14411	6+:121296721-121315790
11630	Mug-ps1	17835	6+:122086686-122198581
11633	Ier3	15937	17+:35958657-35959855
11641	2-Sep	18000	1+:95375620-95406307
11645	Prtn3	19152	10+:79342420-79345720
11648	Lrfn2	70530	17+:49209187-49236912
11649	Hfe	15216	13-:23795884-23802655
11651	S1pr4	13611	10-:80961222-80962882
11652	Ddx56	52513	11-:6158572-6167741
11655	Cd200r1	57781	16+:44766121-44794453
11656	Prkag2	108099	5-:24368575-24606248
11662	Mre11a	17535	9+:14589115-14638404
11663	Dusp5	240672	19+:53603648-53616916
11665	Hyal1	15586	9+:107479306-107482425
11668	D6Wsu163e	28040	6+:126892327-126925635
11671	Postn	50706	3+:54165046-54194953
11677	5730507C01Rik	236366	12+:18411527-19105834
11682	Nutf2	68051	8+:108384560-108402929
11685	Cd83	12522	13+:43880571-43898499
11689	MMT00078109		7+:117442229-117446277
11695	MMT00081973	675985	8+:91330258-91331072
11697	Rasl2-9	19428	7-:5076607-5077558
11699	MMT00044175		18+:42360453-42360956
11701	Oas1c	114643	5-:121250216-121258791
11705	Polr3f	70408	2+:144353494-144365094
11709	Asb2	65256	12-:104559355-104594211
11713	Mpdu1	24070	11-:69470205-69476079
11716	Slco6d1	70866	1+:100317699-100405958
11717	Wdhd1	218973	14-:47860617-47896272
11718	Nmb	68039	7-:88047127-88049922
11722	Ccdc104	216618	11-:29121546-29147281
11724	Tuba3a	22144	6-:125228306-125236049

11725	Itln1	16429	1-:173448253-173465424
11727	Olfr24	18322	9-:18559136-18559750
11729	Ncr1	17086	7+:4289336-4296362
11735	Thoc5	107829	11+:4795334-4828863
11738	Tmed2	56334	5+:124990837-124999252
11740	Cpm	70574	10+:117066553-117122359
11741	Slc30a10	226781	1+:187278726-187289068
11743	Prdx2	21672	8+:87494135-87498209
11744	Ctnnbl1	66642	2+:157563176-157716827
11747	4933434I06Rik	71233	2-:21102357-21126992
11753	CU075549.1	620807	4+:60016418-60016983
11756	2810011L19Rik	69952	12+:106575064-106620320
11758	S100a1	20193	3-:90315031-90318216
11759	3110079O15Rik	73234	1+:89366832-89372058
11761	Ankrd37	654824	8-:47082263-47085222
11762	Ggt5	23887	10+:75052140-75077930
11764	Chrna2	110902	14+:66759796-66771783
11765	Gm884	380730	11-:103461614-103462212
11775	Phyh	16922	2+:4840076-4859770
11776	Ifi30	65972	8-:73286873-73290530
11777	MMT00008513		5-:44797035-44797838
11779	Irx2	16372	13+:72767251-72771641
11780	Taok1	216965	11-:77351102-77369095
11784	Es1	13884	8-:95622913-95655170
11785	MMT00075578		15+:84696703-84697800
11787	Rsf1	233532	7-:104802885-104803263
11789	Ccdc90a	76137	13-:43633780-43655538
11791	Duoxa2	66811	2+:122124635-122128621
11795	Gps2	56310	11+:69727551-69730092
11796	Ela3	67868	4-:136976924-136986433
11797	Sbk1	104175	7+:133416132-133438512
11798	A530082C11Rik	320541	4+:154993303-154994621
11800	Clip3	76686	7+:31077174-31092167
11802	Armc4	74934	18-:7088232-7297938
11803	1500032D16Rik	78330	17+:31657095-31668272
11805	Bat2	53761	17-:35286206-35299928
11807	Fbxo31	76454	8-:124075915-124102555
11808	Pck1	18534	2+:172978573-172984752
11809	4930534B04Rik	75216	12-:92527833-92622820
11812	Mrpl16	94063	19+:11844904-11849434
11814	Pdlim5	56376	3-:141905620-142058582
11816	Ccdc99	70385	11-:34622691-34647126
11817	Dpysl3	22240	18-:43483941-43552985
11818	MMT00048396		2-:147468585-147468916
11819	Mcee	73724	7+:71537531-71557000
11820	Abcg4	192663	9-:44081271-44096206

11822	Elovl2	54326	13-:41277867-41315762
11823	Mmp7	17393	9+:7692089-7699584
11825	MMT00024508		5+:101734596-101734896
11831	AI747699	381236	19-:33749595-33805439
11832	Rnf11	29864	4-:109125503-109149588
11834	Pja2	224938	17-:64632232-64660716
11836	Csf1r	12978	18+:61265528-61290786
11837	Paqr6	68957	3+:88186020-88186764
11840	Chrdl1	83453	X-:139732681-139824691
11843	Dusp22	105352	13+:30751964-30803092
11844	Krt33a	71888	11-:99872515-99877526
11845	Psme1	19186	14+:56197330-56200364
11846	Cxcr5	12145	9-:44319924-44334461
11847	Tmem45b	235135	9-:31233781-31271823
11849	Olfr648	258746	7-:111327970-111328920
11853	EG214403	214403	1-:141471761-141677817
11854	Btbd1	83962	7-:88936960-88963150
11855	4930402F06Rik	74854	2-:35231096-35252696
11857	Arv1	68865	8+:127246038-127258023
11859	Poln	272158	5-:34349838-34452083
11861	Dyrk3	226419	1-:133025018-133034811
11863	Srbd1	78586	17-:86535709-86544568
11864	Dis3l	213550	9-:64154564-64156236
11867	Rcor3	214742	1-:193925269-193961809
11869	Pcsk5	18552	19-:17507405-17729390
11870	Apitd1	69928	4-:148502457-148511659
11871	4931417E11Rik	66740	6-:73418575-73419661
11872	Vamp5	53620	6-:72319026-72320326
11873	Mmp2	17390	8+:95351480-95377318
11880	Svep1	64817	4-:58055668-58219468
11881	Mpl	17480	4-:118115019-118130076
11882	Prdm4	72843	10-:85354714-85379664
11886	Cep250	16328	2+:155817369-155824609
11888	Myadm	50918	7+:3290534-3299348
11891	Rtn2	20167	7+:19868015-19881508
11892	D430042O09Rik	233865	7+:132993891-133018305
11893	Cdc40	71713	10-:40551425-40602915
11895	2810405J04Rik	72722	17-:75936430-75951183
11896	Kif6	319991	17+:49754462-49978532
11899	MMT00070220		9+:55797825-55875733
11902	MMT00029171		X-:5274662-5279002
11903	4933437F05Rik	71275	12-:88561975-88579683
11906	Nfe2l3	18025	6+:51382668-51408767
11907	Tmeff2	56363	1+:50984403-51242552
11911	Wnt6	22420	1+:74818719-74831519
11915	Calm1	12313	12+:101437896-101445521

11917	Fahd1	68636	17-:24985843-24987082
11920	D10Wsu52e	28088	10-:85401381-85420528
11921	Ddx21	56200	10-:62042995-62061938
11922	Cox19	68033	5-:139813467-139821171
11923	Pde3b	18576	7+:121559071-121681693
11925	Epm2aip1	77781	9+:111174345-111181593
11926	Ampd2	109674	3-:107876981-107889568
11929	Slc22a7	108114	17-:46569133-46575380
11930	Igh-VJ558	16061	12-:116784206-116784642
11932	Pkp1	18772	1-:137767974-137815601
11937	Snx10	71982	6+:51473961-51540267
11940	Chi3l1	12654	1+:136079041-136086612
11946	MMT00030953		18-:31979216-31980063
11949	Cldn3	12739	5+:135462257-135463342
11951	Denr	68184	5+:124357283-124378841
11953	Gtf2b	229906	3+:142428210-142446566
11955	Ccl6	20305	11-:83401393-83406492
11956	Bmper	73230	9+:23097840-23289646
11957	Elmod1	270162	9-:53759268-53822981
11959	4631422O05Rik	78749	16+:57507661-57572911
11960	Tm4sf4	229302	3+:57229380-57245587
11961	Nol4	319211	18-:22853442-23198543
11965	MMT00013041		5-:87805183-87807199
11966	Zfp280b	64453	10+:75495435-75502733
11968	Cenpm	66570	15-:82064512-82075177
11970	AA408296	215193	1-:194933482-194956388
11971	1700025D03Rik	67944	X-:134742703-134743300
11974	1700023B02Rik	66935	2-:73125663-73150765
11975	Usmg5	66477	19-:47157957-47165123
11981	Sh3bp2	24055	5+:34868497-34906203
11982	MMT00035514		1-:91152714-91153562
11984	N6amt2	68043	14-:58168434-58190406
11987	Dmkn	73712	7+:31548778-31566086
11992	Mrpl19	56284	6-:81911578-81915943
12000	Tmem16h	382014	8-:73999204-74005145
12004	Lgals6	16857	7+:29619148-29626651
12010	Foxg1	15228	12+:50483993-50487843
12012	Cd247	12503	1+:167718853-167797565
12013	Gsdmc1	83492	15-:63607649-63636153
12015	Esf1	66580	2-:139945616-139996299
12018	1700012C15Rik	76390	11+:73503781-73525936
12020	Bglap2	12097	3-:88181657-88182620
12024	OTTMUSG00000016	667373	19-:34667377-34673920
12027	Dnajb3	15504	1-:90101310-90102323
12029	Tcf19	106795	17-:35649679-35653699
12032	Cdca7	66953	2+:72314275-72324947

12035	Ncoa1	17977	12-:4249454-4483976
12038	Tnks1bp1	228140	2+:84892561-84913202
12040	Commd10	69456	18+:47118522-47247650
12041	Lrrc55	241528	2-:85032067-85036796
12045	Metap2	56307	10-:93323977-93352436
12046	Impdh1	23917	6-:29151198-29162166
12053	2310005L22Rik	69471	4+:131864588-131866941
12055	1810008A18Rik	108707	10-:76949408-76978530
12056	Zic3	22773	X+:55283819-55289080
12059	2700049P18Rik	108900	1+:133424543-133435611
12062	Dzip1l	72507	9+:99549973-99560194
12063	Capn13	381122	17-:73655803-73671379
12066	MMT00040000		5+:87406182-87420763
12070	Adat2	66757	10+:13272699-13282842
12073	Olfr622	259087	7-:110787699-110788652
12084	Siva1	30954	12+:113883133-113887354
12085	EG214403	214403	1-:141594211-141890831
12088	5730416F02Rik	70550	X+:106066877-106197724
12091	1810015C04Rik	66270	15+:25872433-25902092
12092	Hoxa11	15396	6-:52192105-52195766
12097	9430015G10Rik	230996	4+:155484097-155501370
12104	MMT00063115	231872	5-:144663573-144670687
12109	Smad4	17128	18-:73799654-73837885
12111	AA474455	231151	5-:36816291-36826709
12112	Tmem53	68777	4+:116924593-116941185
12114	Homer2	26557	7-:88754229-88794602
12115	Hist1h2an	319170	13-:21878696-21879087
12117	1700024G13Rik	67085	14-:33189272-33201577
12118	Mtrr	210009	13-:68701154-68721039
12119	Dmd	13405	X+:80194241-82450380
12120	Rad51	19361	2+:118938600-118961459
12122	C130092O11Rik	232035	6+:61735140-61761038
12126	Znfx1	98999	2-:166861298-166882441
12127	0610010F05Rik	71675	11-:23473772-23533210
12129	Cfi	12630	3+:129539656-129578248
12130	Cyp3a16	13114	5-:146197177-146230563
12133	7-Sep	235072	9+:25094233-25116152
12139	Cmtm4	97487	8-:106877575-106919596
12140	Mrps15	66407	4+:125724207-125732776
12143	Thyn1	77862	9+:26807306-26814917
12146	Hddc2	69692	10+:31033212-31048008
12151	MMT00082650		8-:83721076-83722253
12155	Gmfb	63985	14-:47427825-47441885
12156	Setd4	224440	16-:93583705-93604048
12157	Gal3st4	330217	5-:138706146-138712604
12164	Gtf2h1	14884	7+:54051635-54078659

12165	Ppp2r1b	73699	9+:50678287-50689343
12168	H2-Ke6	14979	17-:34162979-34165002
12169	MMT00013125		12-:72947250-72948340
12172	Adam9	11502	8-:26060104-26127260
12173	Acbd6	72482	1+:157405251-157534362
12174	2610027C15Rik	230752	4+:125823225-125827118
12177	Tmem35	67564	X+:130829929-130840510
12178	Slc16a2	20502	X-:100892761-101017298
12180	Ank	11732	15+:27396728-27524054
12181	Bcl2l10	12049	9+:75195592-75199447
12182	Immt	76614	6+:71821526-71827382
12185	Cyp2d10	13101	15-:82233337-82237552
12188	Chek2	50883	5+:111270231-111302765
12190	Krt18	16668	15+:101858655-101862452
12191	Zfp704	170753	3-:9427019-9610085
12193	Aco2	11429	15+:81452911-81745561
12194	ENSMUSG0000005454	791344	10-:89207144-89208581
12198	Atp13a5	268878	16-:29232754-29321829
12203	MMT00009475	100042638	1-:63411292-63441743
12204	Ankmy2	217473	12+:36883776-36914800
12208	Pnpla7	241274	2+:24895025-24909577
12213	Entpd5	12499	12-:85717626-85746419
12214	Olfr1271	258789	2-:90105668-90106585
12219	Cyb5b	66427	8+:109674578-109708000
12220	Pgam1	18648	19+:41986449-41993154
12221	Tbc1d23	67581	16-:57170014-57191340
12225	Tsr1	104662	11+:74711576-74722337
12228	Grpel1	17713	5+:36807831-36814345
12229	Sash1	70097	10-:8442990-8605656
12230	MMT00005802		18+:15571723-15573912
12232	MMT00060943	100039903	
12235	Serbp1	66870	6+:67216972-67235957
12238	Traf3	22031	12+:112458020-112505358
12239	A630077B13Rik	215900	10-:33845867-33847789
12242	Ube2e1	22194	14-:19115262-19163650
12243	Edar	13608	10-:58063536-58138444
12245	Zfp322a	218100	13-:23446715-23461016
12248	Tnp2	21959	16-:10788363-10788694
12250	P2ry14	140795	3-:58918542-58934543
12256	Atp10d	231287	5+:72618907-72690004
12259	MMT00080137		2-:155311398-155312165
12261	Bcan	12032	3-:87791452-87804152
12263	Atp6v0d2	242341	4-:19805063-19849754
12270	Slc7a6	330836	8+:108692772-108722610
12272	Fshr	14309	17-:89384511-89599952
12276	Hmga1	15361	17+:27693843-27700608

12278	Plxna4	243743	6-:32094332-32094929
12280	Cln5	211286	14+:103469512-103476847
12281	Tacc2	57752	7+:137836069-137908218
12285	Setd5	72895	6+:113059406-113103418
12288	Olfr392	259008	11-:73627645-73628582
12289	Ext1	14042	15-:52900093-53177500
12290	4930542N07Rik	75185	X+:150916544-150919225
12291	Lim2	233187	7+:50685488-50691361
12296	Rrad	56437	8-:107152350-107154813
12297	Upk1b	22268	16-:38773302-38788156
12298	Lad1	16763	1+:137715339-137729537
12299	Thbs1	21825	2+:117938378-117949995
12302	Cbx1	12412	11+:96662121-96668074
12303	Lmo4	16911	3-:143851692-143865271
12312	Ms4a6d	68774	19-:11661096-11679276
12315	Srebf1	20787	11-:60013100-60016311
12316	Mpa2l	100702	5-:105587936-105600749
12319	Mfap2	17150	4+:140566595-140571889
12327	Ccl11	20292	11+:81871423-81876453
12329	Sox6	20679	7-:122620184-123138600
12330	Aldh6a1	104776	12-:85771672-85791900
12333	Fcrl1	229499	3+:87180370-87189237
12335	Plcb4	18798	2+:135734080-135840679
12336	Timm13	30055	10-:80362660-80363714
12341	EG330731	330731	8-:32847179-32850202
12343	Atf5	107503	7-:52067627-52071942
12344	MMT00034062		4+:37976231-38331106
12346	Myoz2	59006	3-:122709125-122737867
12347	MMT00014037		13-:33668741-33688437
12352	MMT00079007		
12355	AI507611	245884	6+:29232706-29240681
12358	A430083B19Rik	218739	14+:14503430-14514979
12360	EG630825	630825	X-:53208692-53213180
12363	Ifitm6	213002	7-:148201597-148202803
12367	MMT00072365		X+:141282222-141283172
12371	1700128F08Rik	76669	9+:8221888-8241987
12372	MMT00039397		
12376	MMT00011143		12-:78724038-78725029
12377	MMT00050540		8-:49154859-49156054
12380	Rftn2	74013	1-:55227005-55283624
12383	Ugt2a3	72094	5-:87753996-87766201
12384	MMT00056390		
12385	Mrpl33	66845	5+:31916344-31925014
12386	4930579G24Rik	75939	3+:79432979-79436739
12387	Olfr1156	258814	2-:87789426-87790388
12388	8430406I07Rik	74528	2+:144096711-144106196

12391	Mrpl9	78523	3+:94247239-94252629
12398	Ifi44	99899	3-:151393888-151412813
12399	Dpm2	13481	2+:32426405-32428536
12401	MMT00011281		2+:156994509-156995015
12402	Th1l	57314	2+:174241304-174252998
12404	Col16a1	107581	4+:129774847-129776520
12405	Cyp4f14	64385	17-:33042015-33054023
12412	BC004728	207818	15-:100451772-100467643
12414	Rap1a	109905	3-:105531200-105604175
12415	MMT00052705		
12417	Mtap7d1	245877	4-:125909410-125917355
12419	MMT00081393		2+:15049374-15050459
12420	Klhl21	242785	4+:151382632-151391786
12422	Eme1	268465	11-:94505833-94512337
12423	Cyp2g1	13108	7+:27593945-27606216
12425	Olfr1340	258301	4+:118398853-118399801
12430	Fst	14313	13-:115243554-115248794
12433	5033413D22Rik	75973	10-:41270447-41273222
12434	Frs2	327826	10-:116509618-116585557
12436	Serinc3	26943	2-:163450407-163470807
12437	Six6	20476	12+:74040880-74043584
12440	Sult5a1	57429	8-:125666747-125682155
12445	A830080D01Rik	382252	X+:155991683-156031005
12451	Srd5a2	94224	17-:74367047-74397256
12453	Dnaja1	15502	4+:40670745-40671183
12454	Zscan4d	545913	7-:11650789-11751513
12456	Arl6ip5	65106	6+:97160797-97183308
12457	MMT00072579		8-:114760259-114760970
12460	Pnpla2	66853	7+:148641105-148645993
12464	MMT00047924	20947	7+:117365324-117424850
12466	MMT00079257		5+:88491788-88493396
12468	Cyp2d13	68444	15-:82467180-82472455
12469	Dock10	210293	1-:80497648-80630870
12471	Olfr1020	258573	2+:85689610-85690563
12477	3-Mar	320253	18-:56934808-56971749
12478	Rhot2	214952	17-:25981719-25998028
12479	Ifrd1	15982	12-:40929716-40949751
12480	Cdc42bpg	240505	19+:6314486-6314780
12482	MMT00030762		2+:24039162-24040077
12483	5133401N09Rik	75731	13+:58258917-58265746
12484	Tcfap2e	332937	4-:126393245-126413353
12485	Zc3h18	76014	8+:124900686-124940684
12486	Aldh18a1	56454	19-:40624746-40662859
12487	Tbc1d10a	103724	11+:4086835-4115507
12489	Wdr8	59002	4+:153516500-153530924
12491	Ptma	19231	1+:88423310-88427239

12492	Ybx1	22608	4-:118950838-118966995
12493	Ankfy1	11736	11+:72503507-72584723
12494	Thnsl1	208967	2+:21133243-21135307
12495	Casp1	12362	9+:5299288-5306766
12497	Kir3dl2	245615	X-:132987795-132992336
12498	Abcg8	67470	17+:85082571-85098138
12499	Avpi1	69534	19-:42197770-42203483
12501	Zfand6	65098	7-:91764330-91782902
12502	Yy1	22632	12+:110031594-110054463
12507	Fcrls	80891	3-:87054690-87067515
12510	Rs1	20147	X+:157206093-157232677
12512	Eef1e1	66143	13-:38737562-38750879
12513	Whsc2	24116	5-:34239777-34278971
12514	MMT00078654		2-:181687789-181687870
12515	N4bp2	333789	5+:66177371-66218212
12516	Pls3	102866	X-:73031002-73119877
12517	4930573I19Rik	104859	12+:112179662-112209879
12518	Ctsl	13039	13-:64465104-64471600
12519	Bid	12122	6-:120844113-120851770
12520	MMT00057426		X+:76031242-76040294
12534	Nol7	70078	13+:43493761-43498230
12540	Smarca1	93761	X-:45162876-45245719
12541	Stx16	228960	2+:173901923-173924361
12546	Cd7	12516	11-:120898064-120900732
12552	Gm967	381217	19-:24047245-24105301
12553	Kpnb1	16211	11-:97022926-97049066
12556	Ass1	11898	2+:31325818-31376149
12560	Sulf1	240725	1+:12708583-12851270
12563	Col17a1	12821	19-:47720851-47766584
12564	MMT00036520		1-:20434340-20434650
12565	Grem2	23893	1-:176763915-176851950
12568	Lrrc37a	237954	11-:103312057-103357732
12570	Wsb1	78889	11-:79053804-79067894
12571	Cdkn2d	12581	9-:21092935-21095415
12572	Zfp90	22751	8+:108939300-108949628
12575	Cib3	234421	8-:74728232-74734310
12578	Ddx1	104721	12-:13226113-13255980
12581	Mpp3	13384	11-:101860977-101888219
12586	G3bp2	23881	5-:92483539-92512684
12587	Susd3	66329	13-:49326201-49343532
12592	F13a1	74145	13-:36959056-36997927
12594	9130227C08Rik	219094	14+:56473200-56516324
12598	Olfr801	258282	10-:129106616-129107573
12599	Golt1b	66964	6+:142335761-142350643
12600	Ifi204	15951	1-:175677426-175692019
12602	Filip1	70598	9-:79662863-79825653

12604	Acr	11434	15+:89398792-89404858
12611	MMT00068616		14+:33007082-33015133
12612	Rapgef4	56508	2+:71819343-72094433
12617	Zfp46	22704	4+:135845707-135850238
12619	Igf2r	16004	17-:12875274-12963273
12623	Ccdc123	72140	7+:36182111-36223703
12624	Clptm1l	218335	13+:73741748-73758051
12625	AI597468	103266	10+:84565484-84583363
12626	Fut7	14347	2+:25278803-25281891
12631	Cyp51	13121	5-:4082950-4104449
12633	Klk4	56640	7+:51136541-51141174
12634	Olfr1372-ps1	257871	11-:51968385-51969779
12636	MMT00000549	213043	1+:58340343-58415929
12637	Itpka	228550	2+:119568072-119576988
12645	MMT00039341	627312	2-:145335896-145336416
12648	Nt5dc1	319638	10-:34023415-34138358
12649	Itgad	381924	7+:135317501-135349015
12651	Tsc22d4	78829	5+:138203603-138209680
12654	4930485B16Rik	380654	10-:92532577-92544642
12655	Tmem180	75146	19+:46431425-46449741
12656	Coq5	52064	5+:115729710-115746981
12659	Agk	69923	6+:40275481-40346760
12660	MMT00029272		2+:85703569-85704519
12661	4932438A13Rik	229227	3+:36893696-36951565
12667	Slc39a2	214922	14+:52513474-52515481
12668	Creb1	12912	1+:64646232-64648449
12672	Nphs2	170484	1+:158240855-158256564
12674	Lyzl6	69444	11-:103492136-103500195
12676	Nrxn3	18191	12+:90425465-90587024
12679	Cyp4a10	13117	4+:115196847-115206259
12681	Eif2c2	239528	15-:72936136-72976998
12687	Zc3h8	57432	2-:128752005-128769755
12691	MMT00021912		X+:32607392-32630212
12692	Ttn	22138	2-:76688051-76688437
12694	Riok2	67045	17+:17511539-17530935
12696	RP23-392P11.3	545481	2+:158356890-158369144
12700	Nfia	18027	4+:97444589-97779083
12703	Pcsk1	18548	13+:75227434-75269946
12705	Maob	109731	X-:16286407-16394489
12707	Dmrt2	226049	19+:25748220-25753485
12709	Ghitm	66092	14-:37934003-37948452
12711	Nap1l4	17955	7-:150699483-150734995
12712	MMT00059415		5-:138830114-138832821
12716	5830417I10Rik	76022	3+:88694731-88714020
12718	Dhrsx	236082	4_random+:109716-130125
12722	EG624866	624866	3+:65469877-65587947

12725	Bak1	12018	17-:27156756-27165572
12728	Hax1	23897	3-:89799378-89802608
12730	9130019O22Rik	78921	7-:134526789-134537146
12732	Hsd17b1	15485	11+:100939724-100941819
12736	Stat3	20848	11-:100750831-100772675
12739	Insl5	23919	4-:102690478-102699333
12742	Pola1	18968	X-:90550105-90877494
12744	1110034B05Rik	68736	1-:57445082-57463565
12745	Spna2	20740	2+:29821362-29886968
12748	Bat2d	226562	1-:164650674-164670647
12751	Sorl1	20660	9-:41772804-41932196
12753	MMT00043344		7+:49744170-49761147
12754	E130304F04Rik	245269	13_random+:108410-110864
12761	Tsc22d3	14605	X-:137074075-137077710
12766	EG545047	545047	14-:52333319-52335815
12768	Nxt2	237082	X+:138662487-138673352
12769	Tmem177	66343	1-:121804482-121809752
12770	Nola2	52530	11+:51433274-51437216
12772	Axl	26362	7-:26543681-26573497
12773	Klhl1	93688	14-:96505393-96918180
12775	Vstm2b	58188	7+:48154647-48185335
12776	MMT00061492	100045495	7-:11379597-11381473
12778	1700024P16Rik	242594	4+:104668302-104685936
12780	Olfr630	259102	7-:110902663-110905864
12781	Prss1	114228	6+:41408941-41413732
12784	Nudt6	229228	3-:37303911-37318495
12788	Trim69	70928	2+:121986435-122004762
12790	Fhl1	14199	X+:53984990-54046518
12794	MMT00082139	100048814	4+:145454073-145886627
12795	Slc20a2	20516	8+:23616945-23680089
12797	Brdt	114642	5+:107760177-107814713
12798	Dct	13190	14-:118412028-118451407
12799	Spon2	100689	5-:33556578-33560820
12801	2610301F02Rik	545428	2-:76883781-77008667
12803	Ranbp3	71810	17+:56799593-56851183
12805	Bmp7	12162	2-:172695190-172765673
12807	Faim	23873	9+:98892541-98902122
12813	MMT00041243		6-:92514611-92606372
12823	Rab3il1	74760	19+:10092752-10109572
12824	Dbt	13171	3+:116216058-116252310
12825	Gal3st3	545276	19+:5302585-5307893
12826	Zfp354c	30944	11-:50628066-50639960
12827	Stmn4	56471	14+:66973188-66980510
12828	MMT00047250		X-:23819283-23842045
12829	MMT00018827	223286	15-:4938821-5013751
12833	Drp2	13497	X+:130939319-130987938

12834	Fabp7	12140	10+:57504745-57508162
12836	EG629820	629820	12+:40949630-40955775
12837	Olfr800	258541	10+:129096863-129097799
12838	Tec	21682	5-:73146978-73259722
12839	Fh1	14194	1-:177531209-177555720
12845	F11	109821	8-:46326523-46347385
12847	Sar1b	66397	11+:51577208-51605423
12851	Apod	11815	16-:31297205-31314535
12853	Phc3	241915	3-:30805916-30860805
12854	Ndufa13	67184	8-:72418068-72425544
12856	Clstn3	232370	6-:124380784-124414744
12858	Lepre1	56401	4+:118905550-118920972
12860	Tmem156	243025	5-:65466801-65482794
12861	Rhcg	56315	7-:86738254-86762534
12862	Trem2	83433	17+:48485813-48491596
12863	Fgg	99571	3+:82811817-82818969
12864	Myh10	77579	11+:68505609-68630129
12866	4921517L17Rik	70873	2+:156138183-156201370
12867	MMT00021210	667832	11-:54931618-54932378
12869	0610011F06Rik	68347	17+:26012069-26014109
12871	Scn7a	20272	2-:66512479-66622975
12872	Rg9mtd3	69934	4+:45310031-45328353
12879	Ermp1	226090	19-:29684692-29698148
12881	Lgals8	56048	13-:12533091-12553707
12885	Ccl24	56221	5-:136046664-136048842
12886	Acp6	66659	3+:96963222-96980412
12888	AI931714	102182	8+:80073279-80086415
12892	1700019L03Rik	227736	2-:32632656-32639948
12904	MMT00040704	676923	19+:40265187-40271489
12905	Nfil3	18030	13-:53062848-53064235
12906	Nts	67405	10-:101944807-101953047
12907	Zkscan1	74570	5+:138547900-138549048
12908	Jdp2	81703	12+:86940967-86980827
12909	Olfr604	259073	7-:110531575-110532514
12910	1110028A07Rik	68680	14+:56194503-56195789
12911	2210415F13Rik	70163	11+:58192527-58204040
12913	MMT00046078		2-:55628810-55629028
12915	Slc7a5	20539	8-:124405051-124431532
12916	Fkbp1a	14225	2+:151368356-151387426
12917	Nubp1	26425	16+:10412067-10423623
12919	Nup210l	77595	3+:89908054-90014380
12922	Lilrb4	14728	10+:51210780-51216415
12925	Farp1	223254	14+:121606471-121682968
12926	Lrmp	16970	6+:145064182-145123448
12929	6330578E17Rik	76178	1-:37473936-37486883
12930	Pthr1	19228	9-:110624758-110644804

12934	Rbp7	63954	4-:148823797-148829077
12936	Pdlim1	54132	19-:40296729-40346023
12937	Uhrf2	109113	19+:30105092-30168212
12938	Fhod3	225288	18+:25056137-25292001
12941	Elovl5	68801	9+:77765262-77832323
12943	Slc25a27	74011	17-:43778848-43803964
12948	MMT00009927		15-:77200601-77200694
12951	Pcdha6	12937	18+:37127284-37347311
12953	Spin2	278240	X+:150266822-150268784
12954	Gli2	14633	1-:120730637-120786979
12955	Atp5o	28080	16-:91925467-91931870
12958	Itpr2	16439	6-:146369939-146450400
12959	Slc6a1	232333	6+:114251043-114267516
12960	Arrdc4	66412	7-:75886496-75894089
12961	MMT00054077		
12965	Pmm1	29858	15-:81781539-81791297
12966	Slc39a1	30791	3+:90052156-90056994
12968	Fbxw9	68628	8+:87583985-87591208
12969	Xaf1	327959	11+:72115134-72122426
12974	Slc1a6	20513	10+:78243246-78277510
12975	MMT00013656		4-:100240287-100240653
12976	MMT00066041		2+:59972094-59973359
12981	Nek6	59126	2+:38367216-38442085
12982	Cars	27267	7-:150743610-150785961
12984	Cyb5	109672	18+:85020805-85049255
12986	Cox4i2	84682	2+:152579908-152590773
12989	1190002H23Rik	66214	14-:79688557-79701442
12994	Notch1	18128	2-:26314670-26359157
12995	Tmed9	67511	13+:55694503-55699049
12997	Csgalnact2	78752	6-:118058206-118089158
12999	Igfbp6	16012	15+:101974792-101979942
13000	9130404D14Rik	227737	2+:32731743-32780766
13004	Arhgef10	234094	8+:14940208-15001079
13005	Dkk2	56811	3+:131748871-131842621
13006	Akr1b7	11997	6+:34362389-34373142
13007	4632412N22Rik	320159	17+:72066589-72079008
13009	MMT00017557	545339	9+:42771931-42774152
13010	Cyp4b1	13120	4-:115215989-115217663
13015	D14Ertd500e	219072	14-:55160622-55173173
13016	Actn3	11474	19-:4861222-4877884
13018	AW544981	213956	15+:80502298-80530852
13019	Als2cr2	227154	1+:59030463-59052558
13023	Adamts1	11504	16-:85795647-85802904
13025	Irf2	16363	8+:47825144-47932810
13027	Ppp2r5c	26931	12-:111714401-111716823
13028	Mesp1	17292	7-:86937127-86938654

13032	Gpihbp1	68453	15+:75427070-75428769
13033	MMT00013795		
13034	4933437I04Rik	71246	2+:163886550-163888021
13037	Serpinb3b	383548	1-:109050519-109057693
13040	Mylk	107589	16+:34994949-35001449
13044	5730596K20Rik	109168	19+:7569078-7608690
13045	A330021E22Rik	207686	5-:5580985-5664239
13049	Sh3tc1	231147	5-:36039831-36066801
13052	Tnfrsf19	29820	14-:61582711-61656787
13054	Mcm4	17217	16-:15624263-15637399
13057	Aspn	66695	13+:49639857-49662926
13058	Gpx3	14778	11+:54716451-54723878
13059	Centd1	212285	5-:62993686-63157321
13067	MMT00074323		9-:63290972-63291889
13068	Yap1	22601	9-:7932001-8004586
13072	BC022687	217887	12+:114047527-114054463
13075	Mrps2	118451	2+:28323594-28325635
13077	Cyp2c70	226105	19-:40227852-40261761
13078	Eps8l2	98845	7+:148524900-148548914
13083	Myo7b	17922	18-:32118889-32196615
13086	Ccdc79	320022	8-:106971480-106996847
13087	Cxadr	13052	16+:78302000-78359992
13088	Tspan3	56434	9-:55983694-56008671
13090	Rnf128	66889	X+:136145158-136206853
13094	Cnn3	71994	3+:121129458-121161123
13096	Otor	57329	2+:142904218-142907436
13098	Kcnk1	16525	8+:128519069-128554583
13102	Prkaa1	105787	15+:5110586-5128977
13103	Erlin1	226144	19-:44109437-44144239
13104	Ccdc70	67929	8+:23080246-23084489
13113	Man2a2	140481	7-:87496182-87516071
13115	B230120H23Rik	65964	2+:72136243-72280665
13116	Actn2	11472	13-:12361698-12432767
13117	Tmprss5	80893	9+:48910883-48925690
13118	Sfrp2	20319	3+:83570242-83578237
13120	Lactb	80907	9-:66803199-66823291
13124	Arfgap3	66251	15-:83130169-83180593
13127	Col5a1	12831	2+:27742204-27893044
13129	Bmp8a	12163	4-:122989889-123020046
13131	Pscd1	19157	11-:118027331-118055009
13132	MMT00070380		14+:44864398-44866804
13133	Tmeff1	230157	4+:48598238-48676000
13134	Lipg	16891	18-:75100521-75120899
13136	Itgb1bp3	69564	10-:80661939-80664801
13137	2610301G19Rik	219158	14-:70537983-70540903
13138	Olfr1030	258581	2+:85823998-85824953

13139	Mid1	17318	X+:166266090-166404099
13140	MMT00005814		
13141	MMT00071569		15-:3695893-3696875
13142	Wdr81	192652	11-:75254454-75259071
13143	Zmym6	100177	4+:126754625-126806890
13144	Calml3	70405	13-:3802140-3803562
13145	Dapl1	76747	2+:59322711-59343030
13149	Ifi203	15950	1-:175850535-175872523
13150	4921528I07Rik	77049	9-:114185780-114226148
13151	Asb14	142687	14+:27716428-27728444
13156	Ifit3	15959	19+:34658043-34663217
13159	Map4k4	26921	1+:39958074-40081770
13160	Frem3	333315	8+:83135126-83219247
13161	EG634346	634346	1+:99243005-99339300
13162	Pcdh17	219228	14+:84845631-84934510
13164	Il4ra	16190	7+:132695800-132721481
13167	Idi1	319554	13+:8885105-8891640
13168	Col8a1	12837	16-:57627025-57632751
13172	Epb4.1l3	13823	17+:69506149-69639325
13174	MMT00032085	628236	19-:33540369-33543377
13175	4930432O21Rik	74670	17+:22099517-22131327
13179	Plxna2	18845	1+:196446095-196640656
13180	Nox4	50490	7+:94395307-94545509
13181	Lcp2	16822	11+:33947168-33991134
13183	Rg9mtd2	108943	3+:137806869-137817721
13184	MMT00017268		8+:32114328-32114626
13186	3110082I17Rik	73212	5-:139835693-139936467
13188	Sumf2	67902	5+:130322885-130339299
13189	Dnajb4	67035	3-:151847982-151873047
13190	Igsf6	80719	7-:128207581-128218044
13192	Impg1	63859	9-:80160893-80359018
13195	Mtif2	76784	11+:29426464-29445255
13200	5730449L18Rik	66637	1-:154217882-154233752
13201	Rbl2	19651	8+:93594056-93646925
13202	Sass6	72776	3+:116297796-116332052
13205	EG626215	626215	12+:79298255-79299732
13206	Qprt	67375	7-:134251284-134265543
13207	Sptlc2	20773	12-:88650588-88729151
13209	Mcm3ap	54387	10+:75931682-75978602
13210	Nudt1	17766	5+:140807875-140814089
13211	Sbno2	216161	10-:79519743-79603581
13214	Slc12a8	171286	16+:33518431-33663487
13216	Ccdc106	232821	7+:5008798-5012384
13217	Rnase1	19752	14-:51764854-51766442
13218	C330043M08Rik	330323	6+:55153468-55213858
13219	Trhde	237553	10-:113836079-114238420

13221	Srd5a3	57357	5+:76569296-76584495
13223	BC022651	330460	7-:4667145-4676683
13224	Cp	12870	3+:19857178-19892590
13225	Grhl2	252973	15+:37162790-37263402
13231	MMT00080853		11+:98046648-98047461
13233	Oaz2	18247	9+:65524399-65537263
13236	Pak1ip1	68083	13+:41096628-41108380
13238	Nr1h3	22259	2-:91024267-91035273
13240	Ing1	26356	8+:11556909-11562121
13245	Gpr177	68151	3+:159502702-159597865
13246	Klc4	74764	17-:46767579-46782093
13248	4833423E24Rik	228151	2-:85323748-85358923
13251	EG622268	622268	18+:15169754-15170200
13253	Mtvr2	17826	19-:5728087-5731713
13257	Nadk	192185	4+:154936512-154965098
13261	Aldh7a1	110695	18-:56685389-56732524
13263	Chchd5	66170	2+:128955528-128959845
13266	Man1c1	230815	4-:134117734-134259384
13268	1600015I10Rik	69761	6+:48882536-48883679
13269	MMT00039476		11-:29501785-29502542
13271	Idh2	269951	7-:87239735-87260194
13272	Rcan3	53902	4-:134972304-134981349
13273	MMT00033789		12+:105503304-105506842
13274	Qk	19317	17-:10403051-10512719
13277	9530015I07Rik	619310	9+:21971843-22005468
13279	Ifitm1	68713	7+:148154112-148155711
13281	Tmcc2	68875	1-:134252893-134287760
13282	2310005N03Rik	66359	1+:180249289-180252824
13285	Cd276	102657	9-:58372105-58388663
13286	Slc23a1	20522	18-:35779059-35786791
13287	Dnajb14	70604	3+:137531123-137571895
13288	AA407270	102371	9-:71352152-71440143
13289	Dusp10	63953	1+:185858333-185898756
13293	Gsta2	14858	9-:78178848-78194952
13296	Aim1	11630	10-:43670440-43724696
13297	Emb	13723	13+:118009552-118062905
13299	Areg	11839	5+:91568813-91575772
13302	Bche	12038	3-:73443777-73506030
13304	Gadd45b	17873	10+:80392835-80394949
13306	Cables2	252966	2-:179993249-179999181
13308	Kit	16590	5+:75971048-76052744
13309	Tgtp	21822	11-:48871734-48877545
13311	Prl3c1	27372	13+:27291192-27295616
13318	Adamtsl2	77794	2+:26937901-26964495
13324	Tmem184a	231832	5-:140280915-140283666
13325	BC043301	210104	7+:50827958-50847420

13327	Vmn2r95	328759	17+:18576804-18589286
13328	Ly6h	23934	15-:75395507-75397244
13329	Osbp2	74309	11-:3604079-3763870
13333	MMT00048365		X-:87049908-87051157
13337	Pard3b	72823	1+:62056877-62391572
13339	D630042F21Rik	330428	6+:129462572-129468713
13341	Tnip2	231130	5-:34838744-34856628
13345	Mmrn2	105450	14+:35188648-35217468
13347	Abi3	66610	11-:95693706-95703474
13348	Ank3	11735	10+:68996913-69487702
13349	MMT00014600		8-:107199431-107231509
13351	Zrsr1	22183	11+:22872036-22874908
13355	Foxs1	14239	2-:152757642-152761001
13356	Aldh3b2	621603	19+:3964465-3981653
13357	Rpp14	67053	14+:8912880-8924348
13362	Ddt	13202	10-:75233977-75236119
13367	MMT00060916	624083	5-:138160983-138163193
13371	Peli2	93834	14+:48740614-48876497
13372	Slc11a1	18173	1+:74421802-74432626
13374	Wipi1	52639	11-:109434841-109472695
13376	Alg2	56737	4-:47482742-47487176
13377	Nod1	107607	6-:54873942-54899755
13381	Dnm3	103967	1-:163920359-164161902
13382	Bco2	170752	9-:50341256-50358807
13383	Cybb	13058	X-:9015341-9046376
13384	Fmo3	14262	1-:164883930-164914622
13387	Olfr1093	258363	2+:86625888-86626855
13388	Klra18	93970	6-:129993762-130017276
13389	Six6os1	75801	12-:73982095-74018752
13390	Ddx31	227674	2+:28695846-28761087
13393	OTTMUSG00000012:	433424	2+:44966657-44968822
13394	MMT00054980		13+:8127312-8130100
13395	Ube2m	22192	7-:13620587-13623327
13404	MMT00082585		1+:24078764-24079635
13405	Rabep2	70314	7+:133572953-133589414
13408	MMT00079369		14+:52689116-52689675
13413	Cnih2	12794	19-:5092873-5098418
13417	1700025G04Rik	69399	1-:153739394-153937379
13418	Lgi4	243914	7+:31844953-31855761
13422	Usp3	235441	9-:66366146-66440896
13423	ENSMUSG00000053	791337	15-:54943897-54945279
13424	MMT00039710		6-:67904198-67904684
13428	Gpr20	239530	15-:73525891-73544267
13431	MMT00038058		13+:83971918-83973422
13438	Btg3	12228	16-:78360106-78373669
13445	Stk32b	64293	5-:37838066-38108392

13449	Tmem103	72341	9+:110207699-110224605
13451	Tbcb	66411	7-:31009158-31016797
13455	Cenpe	229841	3+:134875581-134936484
13457	Hexa	15211	9+:59387379-59412914
13459	Rgs4	19736	1-:171673799-171677654
13464	Mc4r	17202	18-:67018697-67092999
13465	1700008P02Rik	69347	3-:6615422-6620445
13469	Ccrl1	252837	9-:104000473-104029263
13470	Actr3	74117	1-:127289488-127332305
13471	Cln8	26889	8+:14889045-14897938
13472	B230219D22Rik	78521	13+:55794446-55804860
13475	Morn2	378462	17+:80689549-80696815
13478	Olfr1125	259030	2+:87274645-87275600
13481	Gpnmb	93695	6+:48986632-49006779
13487	A230051G13Rik	216792	11-:58970557-58977231
13488	Art4	109978	6-:136796971-136806121
13489	D230025D16Rik	234678	8+:107749204-107776949
13490	Vps35	65114	8-:87784292-87823415
13491	Plk2	20620	13+:111185267-111191055
13493	Ring1	19763	17-:34158154-34160391
13494	Nrcam	319504	12+:45633555-45701150
13495	Abcb10	56199	8-:126476360-126506752
13496	H1fnt	70069	15-:98086413-98087697
13499	St7l	229681	3+:104667477-104729823
13501	Prkx	19108	X-:75006337-75041320
13504	Lair1	52855	7-:3960607-4014790
13505	1700018P08Rik	73330	8+:119013641-119014823
13507	Spats1	71020	17-:45585885-45611952
13511	Aytl1b	70902	5+:107860567-107864059
13512	Tmem144	70652	3-:79617600-79646601
13513	Trim25	217069	11+:88860769-88881607
13514	Apol9b	71898	15+:77559508-77566811
13515	Crabp2	12904	3+:87752609-87757294
13516	Fibcd1	98970	2-:31671857-31701302
13521	Ears2	67417	7-:129180728-129210558
13522	Slfn5	327978	11+:82769763-82776439
13523	Olfr1179	258919	2-:88242169-88243089
13527	Ctss	13040	3+:95330765-95360316
13528	Capn12	60594	7+:29666674-29678605
13529	Abi3bp	320712	16+:56574500-56690240
13533	Col27a1	373864	4+:62992026-62995172
13534	MMT00001967		14-:87841015-87841533
13535	Rgs18	64214	1-:146600436-146622558
13543	Zfp617	170938	8+:74446762-74457899
13544	Parp8	52552	13-:117643725-117666399
13545	Tmie	20776	9-:110768550-110782587

13550	Mbnl2	105559	14+:120724015-120830918
13551	Zfp623	78834	15+:75771484-75779830
13554	Aqr	11834	2-:113926895-114001074
13558	Klk7	23993	7+:51066836-51071435
13559	Ctag2	70062	X+:62300815-62302191
13560	Nucb2	53322	7+:123647882-123684068
13561	Dexi	58239	16-:10530300-10542848
13563	9930023K05Rik	226245	19+:56536134-56561215
13564	Qrsl1	76563	10-:43593982-43621632
13565	Fcho2	218503	13-:99493362-99576327
13568	Cad	69719	5+:31357090-31380851
13569	1190003M12Rik	68888	6-:87333249-87338849
13570	Olfr171	258960	16-:19624250-19625194
13571	Samd8	67630	14+:22569752-22612510
13573	MMT00075852		5+:67366914-67375378
13576	Zfp820	75424	17-:21956087-21958683
13578	Asph	65973	4-:9376231-9596231
13579	1700020D05Rik	75555	19-:5502867-5503751
13581	Srxn1	76650	2+:151931470-151935536
13582	Tnnt3	21957	7+:149684747-149701908
13585	Nckap1l	105855	15+:103284273-103329240
13588	MMT00042387		14-:105596999-105600644
13590	Mcart6	67062	X-:133549792-133572842
13591	Xrcc2	57434	5-:25197818-25211609
13592	Tceal3	594844	X+:133200998-133202913
13598	Reg1	19692	6+:78375976-78378660
13608	Lrrc6	54562	15-:66211551-66332403
13613	Rptn	20129	3+:93197620-93203364
13614	Zfp60	22718	7+:28521974-28535040
13615	Arl16	70317	11-:120326195-120328924
13617	Acin1	56215	14-:55261004-55305768
13624	Ubc	22190	5-:125866427-125868631
13625	Apobec1	11810	6-:122527809-122541380
13626	RP23-395H4.4	13706	4-:141370877-141381918
13630	Cdkn1a	12575	17+:29235151-29236507
13637	Lin9	72568	1+:182578425-182620819
13639	Cnga1	12788	5-:72994937-73035513
13640	Atp6v0a1	11975	11+:100870795-100925025
13643	Dlec1	320256	9+:119011683-119056811
13644	Acsl3	74205	1+:78654387-78704313
13646	MMT00034706		2+:132487977-132488328
13648	Dap	223453	15+:31154187-31204090
13650	Cntn1	12805	15+:91958624-92170542
13654	Stat1	20846	1+:52179434-52216163
13656	Ly86	17084	13+:37437213-37510813
13657	MMT00065461	100040887	1+:100160277-100161054

13661	A430041B07Rik	328108	12+:66066963-66123559
13663	Sgip1	73094	4+:102432967-102643781
13664	AU017455	105590	14-:79612461-79614164
13665	Reg3g	19695	6-:78416261-78418868
13666	MMT00039363		
13667	Cbs	12411	17-:31749589-31774086
13676	MMT00010979		2+:151273791-151273860
13677	Tinagl	94242	4-:129842962-129852366
13682	BC051142	407788	17+:34553408-34597665
13684	Abcd4	19300	12-:85943618-85958320
13687	Mal	17153	2-:127458963-127481907
13688	Ppa1	67895	10+:61111367-61136911
13692	MMT00082247		2-:170779646-170780383
13693	Copb2	50797	9+:98464062-98488802
13695	Tlr3	142980	8-:46481719-46494053
13700	Olfml2a	241327	2+:38813205-38819269
13703	MMT00011278		2-:156172539-156173733
13704	MMT00048234		9-:66207972-66208407
13705	Osbpl1a	64291	18-:12913842-13021423
13708	Aldoa	11674	7-:133938748-133942613
13711	Chuk	12675	19-:44147825-44181932
13712	Lypd5	76942	7+:25136535-25139268
13714	Igfals	16005	17+:25016893-25018692
13716	D5Ertd579e	320661	5-:36943139-37064482
13717	Slc6a6	21366	6+:91648994-91705130
13718	9130209A04Rik	77668	18+:47613734-47630522
13720	Serpina11	380780	12-:105218464-105228163
13724	MMT00002755		7+:90560486-90562804
13731	2210408F21Rik	73652	6+:31170357-31286857
13732	Tpm4	326618	8+:74659176-74677031
13733	Setd3	52690	12-:109344641-109405608
13734	Srp9	27058	1+:184054941-184061921
13737	Igk-V34	16118	6+:68889570-68890084
13738	Nek5	330721	8-:23207928-23224331
13740	MMT00022502		2-:155024351-155025012
13741	Hspa4l	18415	3+:40549540-40593502
13742	1700024P16Rik	242594	4+:104621278-104651691
13746	E330013P04Rik	107376	19-:60229174-60230571
13747	Entpd2	12496	2+:25251477-25256837
13749	Parp9	80285	16+:35939125-35972689
13751	Klk6	19144	7+:51080819-51087156
13753	MMT00042249		3-:104221656-104222184
13758	Ubtd2	327900	11+:32355476-32418710
13763	Dcn	13179	10+:96942216-96980365
13766	Slc7a9	30962	7+:36234197-36251017
13767	Kcnk15	241769	2+:163683917-163684609

13768	Mrps10	64657	17+:47505834-47515629
13769	Sap18	20220	14+:58417119-58420914
13770	D5Wsu178e	28042	5+:30559204-30592171
13773	Plunc	18843	2+:153968620-153974952
13774	Gdf15	23886	8-:73153292-73155994
13776	D12Ertd551e	52635	12+:117519723-117610694
13777	MMT00015864	668030	2-:175918911-176407018
13780	Pilra	231805	5-:138264513-138276946
13785	MMT00032015		18+:36769557-36770424
13789	H2-Q8	15019	17+:35531070-35531975
13790	Olfr909	258873	9+:38331318-38332252
13792	Slc25a35	71998	11+:68781632-68786017
13793	Wrb	71446	16+:96367025-96376382
13794	Higd1a	56295	9-:121758658-121766649
13797	MMT00046285		2+:83230285-83230752
13798	3110001I20Rik	70354	2-:125563267-125565690
13799	9430031J16Rik	241134	1+:81073777-81084068
13800	Lrp5	16973	19-:3584876-3686554
13801	Adh4	26876	3+:138078472-138093856
13810	1110036O03Rik	66180	11-:100269770-100276207
13811	Fkbp7	14231	2-:76501103-76511127
13813	Ktelc1	224143	16-:38525157-38550312
13816	MMT00015290		4-:147943224-147943838
13823	Olfr1043	258570	2-:86002160-86003104
13825	Myoz1	59011	14-:21468329-21475702
13826	Melk	17279	4+:44313851-44377166
13835	Lama1	16772	17+:68046626-68171989
13838	Erbb2ip	59079	13-:104608872-104623709
13839	Gypc	71683	18-:32687977-32719517
13842	Aurkaip1	66077	4+:155205754-155207166
13844	Trib1	211770	15+:59480624-59487539
13845	Ccdc28a	215814	10-:17936732-17954784
13847	Tmigd1	66601	11+:76718046-76730088
13850	Tmem165	21982	5+:76612974-76638269
13858	Myl1	17901	1-:66970867-66991718
13860	Cpt1b	12895	15-:89246835-89255778
13862	Myh15	667772	16+:49065068-49197003
13864	Larp1	73158	11+:57822979-57875537
13865	Papd5	214627	8+:90767083-90783624
13867	Sgcb	24051	5-:74026498-74038983
13870	4921521F21Rik	70861	1-:65059262-65079321
13874	Prkd1	18760	12-:51442707-51750085
13875	Hsd17b10	15108	X+:148436433-148438982
13876	C530008M17Rik	320827	5+:77239012-77271614
13877	Btnl9	237754	11-:48982337-48996595
13881	Vmn2r100	627537	17+:19658845-19669024

13883	EG627788	627788	19-:28837489-28838019
13884	Aven	74268	2+:112333120-112471227
13885	Clec7a	56644	6-:129411610-129422790
13886	Hnrpdl	50926	5-:100463746-100467948
13888	MMT00063459	329633	3+:49158747-49171281
13889	Agpat2	67512	2-:26448579-26459748
13890	Olfr1263	258790	2+:89855088-89856007
13893	Slc6a2	20538	8+:95485146-95520980
13895	MMT00072628		4-:23444393-23446309
13897	Palld	72333	8-:64163528-64238883
13900	4933417M04Rik	619288	1-:192986567-192988323
13901	EG667795	667795	1+:155152561-155153607
13906	Hmgb4	69317	4-:127937456-127938137
13907	B4galnt4	330671	7+:148247208-148258291
13913	Gm70	210762	12+:77518609-77540468
13914	Hdac5	15184	11-:102057062-102086153
13915	MMT00039793		4+:98795260-98796875
13919	Synpo	104027	18-:60753636-60764144
13922	MMT00008875		Y+:1351339-1353880
13923	Nploc4	217365	11-:120241110-120298836
13929	Lamb3	16780	1+:195128219-195170070
13930	EG620205	620205	3-:133452331-133454469
13934	Hunk	26559	16+:90284122-90488156
13935	Aldh8a1	237320	10+:21097127-21116390
13937	A230107C01Rik	320225	7-:29970455-29975654
13942	Psg21	72242	7-:19237068-19242065
13946	Slc35b2	73836	17+:45701036-45705072
13947	Crip2	68337	12+:114378757-114383715
13950	Fabp4	11770	3-:10204346-10208554
13953	4930469G21Rik	74933	1-:163084688-163089666
13954	Chst1	76969	2+:92451929-92455407
13955	Dennd2d	72121	3+:106285489-106306662
13956	BC035044	232406	6-:128818327-128841142
13960	A930038C07Rik	68169	6+:65646033-65656923
13962	Chd7	320790	4+:8678469-8793800
13964	Lpcat2	270084	8+:95433067-95442397
13965	MMT00061510		7+:11330623-11331328
13966	Aatk	11302	11-:119868636-119903062
13972	Hadhb	231086	5+:30500349-30510627
13975	Slc27a6	225579	18+:58715893-58772523
13976	Bmi1	12151	2+:18603438-18605881
13977	MMT00049981		
13979	Ociad2	433904	5-:73713436-73729893
13982	Tnfaip6	21930	2+:51893632-51912108
13983	Copz2	56358	11+:96711189-96722514
13984	Thap3	69876	4-:151356744-151363547

13988	Cntnap2	66797	6+:47194400-47249311
13989	Foxp3	20371	X+:7156818-7172360
13990	MMT00040357		19+:39053872-39077776
13998	MMT00024160		
14002	1700055M20Rik	73407	8+:97836854-97851153
14006	Dus4l	71916	12-:32324919-32339691
14009	2310030G06Rik	66952	9-:50547803-50554632
14013	MMT00017766		2-:22768812-22769369
14014	MMT00008376		4-:97187838-97190377
14015	Prss3	22073	6-:41323772-41327578
14017	Ces5	234673	8+:107450183-107458567
14018	F830045P16Rik	228592	2-:129286051-129289467
14019	Fbf1	217335	11-:116003597-116029455
14022	Car9	230099	4+:43519878-43526597
14025	4930522H14Rik	67646	4-:109177979-109203902
14027	Rpo1-1	20016	17-:46380876-46384913
14029	2810022L02Rik	67198	1+:57831768-58003808
14030	Csrp2	13008	10+:110357231-110376550
14032	Cog6	67542	3-:52785799-52821159
14035	Lrrc52	240899	1-:169375931-169398144
14041	Cldn1	12737	16-:26358962-26371867
14042	MMT00046983	279067	2-:90870934-90871279
14043	4930455C21Rik	76916	16-:38498432-38522709
14045	Rps4y2	66184	6+:148303177-148304118
14046	Ms4a7	109225	19-:11395526-11409186
14048	Ccny	67974	18-:9315943-9449981
14049	Fdft1	14137	14-:63765240-63796638
14050	Il12rb1	16161	8+:73332392-73345320
14052	EG226654	226654	1+:173349231-173350486
14055	MMT00050438		4-:119006855-119008123
14056	Opa1	74143	16+:29586094-29589836
14059	Cyp4f18	72054	8-:74512380-74533516
14062	Pan3	72587	5+:148341504-148360063
14071	Marveld3	73608	8-:112476810-112486025
14074	Lrrc2	74249	9+:110854048-110883467
14075	E030049G20Rik	210356	1-:127873502-127923947
14077	Trpv5	194352	6-:41603129-41626221
14082	Fosl1	14283	19+:5447697-5455938
14084	D530033C11Rik	78581	15+:51709037-51714224
14085	Cdkl2	53886	5-:92435182-92472044
14087	Ugdh	22235	5-:65804462-65826959
14093	Tbpl2	227606	2-:23942585-23946671
14094	Srpk2	20817	5-:23013099-23122182
14095	Nav1	215690	1-:137337418-137481765
14099	Nfe2	18022	15-:103078643-103085868
14100	Ccnk	12454	12+:109417809-109440617

14101	Zfp69	381549	4-:120602747-120620174
14102	Evi2a	14017	11-:79340259-79341868
14108	Polr3b	70428	10+:84085028-84189922
14110	Aspm	12316	1+:141351517-141353460
14114	Ccdc23	69216	4+:118868013-118873903
14115	MMT00048392		X-:55883578-55884361
14116	Sp1	20683	15+:102236758-102263109
14117	C1qb	12260	4-:136436062-136442092
14127	MMT00009389	236598	
14129	5430403G16Rik	77200	5-:110103562-110104226
14132	Glo1	109801	17-:30729810-30749551
14134	Phf10	72057	17-:15082013-15093866
14135	Eepd1	67484	9+:25289187-25411692
14136	F7	14068	8+:13026045-13035808
14138	Anks4b	72074	7+:127317389-127327228
14139	2410005O16Rik	66302	4+:19502340-19533424
14140	Ipo5	70572	14+:121316569-121347253
14142	MMT00021961		5-:30157973-30158961
14145	Sgsm3	105835	15+:80808234-80842500
14147	Acss2	60525	2+:155343828-155388234
14148	Fbxl3	50789	14-:103480265-103498088
14149	BC048562	434439	9+:108338810-108348412
14151	Slc44a1	100434	4+:53494335-53635350
14154	Apof	103161	10+:127705052-127707207
14156	Med9	192191	11+:59761768-59774588
14167	Exo1	26909	1+:177813029-177838863
14169	1700052N19Rik	73419	10-:5906571-5913921
14172	Olfr479	257891	7+:115198626-115199480
14176	1500011H22Rik	68948	5-:122814594-122822364
14180	Xlr3c	22446	X-:70499879-70510729
14183	Gng11	66066	6+:3954226-3958443
14185	Poldip3	73826	15-:82956398-82979739
14187	Zic1	22771	9-:91255338-91260383
14188	Ttc8	76260	12+:100158784-100221442
14190	Ppil1	68816	17-:29387779-29400916
14191	Scn2b	72821	9+:44925959-44935296
14192	Trpv2	22368	11+:62388048-62413798
14200	Kirrel	170643	3-:86892285-86945031
14202	Fnbp4	55935	2+:90585526-90621175
14204	Gm317	207209	17+:25307131-25308855
14208	Ech1	51798	7+:29610356-29617258
14209	Btg3	12228	16-:78360106-78377030
14213	Rcor2	104383	19+:7344253-7349714
14215	Rab5a	271457	17+:53618713-53647003
14222	MMT00076410		7+:115006493-115007624
14225	Sva	20939	6+:41988392-41992797

14226	Rbm47	245945	5-:66410008-66489418
14227	Lrrc39	109245	3+:116265889-116284342
14229	Smtnl1	68678	2-:84651333-84662809
14233	Fgfrl1	116701	5+:109123520-109135915
14235	MMT00002524		18+:8958025-8968920
14237	Rac2	19354	15-:78391275-78403111
14241	C130074G19Rik	226777	1-:186698121-186706870
14242	Prss29	114662	17+:25457227-25459451
14244	Nap1l1	53605	10+:110910297-110933175
14245	H2-Ke2	14976	17-:34075869-34077250
14249	Lzts2	226154	19+:45095879-45101576
14251	Efhd1	98363	1+:89160938-89207366
14253	Olfr466	258816	13+:65253533-65254459
14256	MMT00024953		1+:121054452-121054944
14258	Ifi47	15953	11+:48900556-48910471
14260	Mlkl	74568	8-:113835699-113862078
14261	2310066E14Rik	75687	8+:108145081-108146094
14262	Tcfec	21426	6-:16783391-16848441
14263	Arrdc3	105171	13+:81022595-81035299
14265	Hnrnpa3	229279	2+:75499520-75500768
14266	Sox13	20668	1-:135278879-135289795
14267	Naaladl2	635702	3-:23703256-23780422
14271	Cox6a1	12861	5-:115795663-115798963
14279	3930402G23Rik	74027	8-:10924425-10928522
14280	MMT00004656		6-:24996506-25149473
14281	5031414D18Rik	271221	14+:75431791-75452333
14285	C1qbp	12261	11-:70791356-70796467
14288	Atmin	234776	8+:119467329-119484344
14293	Col9a1	12839	1+:24184546-24258839
14296	Slc35b1	110172	11+:95246235-95252966
14297	MMT00040516		6+:71633291-71633877
14298	Atp2a2	11938	5-:122903522-122951904
14303	V1ri2	171253	13+:23167660-23168561
14304	Mrpl48	52443	7-:107697223-107699149
14305	MMT00024654		2-:149158807-149160401
14307	Kcnj8	16523	6-:142513890-142519829
14309	Cyp3a41b	100041375	5-:146319535-146325438
14310	MMT00073582		9+:63745916-63747639
14312	1110012J17Rik	68617	17-:66691784-66798086
14313	Tnk1	83813	11-:69665104-69670576
14314	2810405K02Rik	66469	4-:154270521-154273152
14316	MMT00012375		18-:54190446-54191079
14322	Bat1a	53817	17+:35380184-35390443
14323	Scarb1	20778	5-:125757461-125821444
14331	Dgat2l6	668257	X+:97736406-97741222
14333	Zcchc17	619605	4-:129993147-130037207

14334	Fxyd3	17178	7-:31855556-31861697
14335	Olfr33	18332	7-:109861970-109862925
14342	4930412F15Rik	242408	4-:43677419-43679483
14343	Hnrnpa0	77134	13-:58227829-58229945
14344	4921506M07Rik	70846	12+:58665108-58723099
14347	Dact2	240025	17-:14332059-14340873
14350	Adamts2	216725	11+:50415981-50617553
14353	Slc35f2	72022	9+:53619546-53665927
14355	Tle1	21885	4-:71779317-71861093
14357	Ing4	28019	6+:124989865-124999282
14358	Wdr1	22388	5-:38918052-38952834
14359	Hist2h2bb	319189	3+:96073674-96074054
14361	Txndc12	66073	4+:108507294-108534722
14362	S100z	268686	13-:96247257-96248610
14363	Cxcl16	66102	11-:70268905-70272949
14364	Robo1	19876	16+:72204862-72742697
14368	9330128J19Rik	271144	13-:97189619-97199839
14369	MMT00057201		X+:132862837-132863488
14373	Cyp2c50	107141	19+:40164185-40188440
14375	D17H6S56E-5	110956	17-:35133667-35137272
14379	Car12	76459	9+:66561328-66614653
14380	MMT00051866		2-:129203437-129205150
14384	Dnajc7	56354	11-:100444164-100480921
14385	2010001M09Rik	69816	18-:35806925-35809021
14387	Ccdc88c	68339	12-:102207553-102267119
14390	EG545216	545216	17+:58659787-58734264
14391	MMT00017335	100043120	19-:61332778-61333797
14395	MMT00007523		14-:91599022-91599684
14397	Padi2	18600	4+:140462341-140508500
14398	Snn	20621	16+:11072324-11072677
14399	Csnk2a1	12995	2+:152083203-152104962
14400	Scd2	20250	19+:44368577-44381350
14403	S100g	12309	X-:159399948-159402497
14405	1700015E13Rik	76925	1+:172222992-172242258
14406	Zc3h14	75553	12+:99985176-100026156
14407	Six3	20473	17+:86012946-86024533
14408	1100001H23Rik	66857	6-:136560591-136610337
14414	Twsg1	65960	17-:66272405-66300470
14416	Serpinb11	66957	1+:109258890-109277052
14418	Vcan	13003	13-:89796661-89871139
14420	Cfd	11537	10+:79353656-79355399
14421	Shox2	20429	3-:66777192-66785693
14424	MMT00015366		17-:45300176-45327522
14427	Hoxa1	15394	6-:52105592-52108285
14428	Atad5	237877	11+:79946476-79947689
14431	Piwil1	57749	5+:129242155-129261348

14434	Sec14l2	67815	11-:3997044-4018732
14435	Stch	110920	16-:75755479-75767035
14438	V1rc24	171197	6-:57875878-57876785
14439	Ubd	24108	17+:37330506-37332782
14440	MMT00015397		
14442	Ethe1	66071	7+:25378927-25393853
14443	C3	12266	17-:57343383-57387113
14444	Pdia4	12304	6-:47746360-47758344
14445	Arl6	56297	16-:59613153-59638879
14447	MMT00000775	627004	7-:34352273-34391071
14449	Il23r	209590	6-:67372925-67441849
14453	Inpp5d	16331	1+:89516953-89617076
14454	Sall3	20689	18-:81165207-81171368
14455	2700029M09Rik	72612	8+:63369207-63386377
14457	4921507P07Rik	70821	6-:50523304-50546589
14460	EG638904	638904	6+:40736939-40807794
14462	Zfp385b	241494	2-:77248699-77254198
14463	MMT00051367		8+:83294589-83295567
14464	MMT00059232		12+:83644585-83645174
14466	MMT00057557		17-:22056771-22058139
14468	Tmtc2	278279	10-:104626034-104627073
14469	Ripply3	170765	16+:94550026-94558540
14470	Taf1a	21339	1_random+:362346-383535
14471	Pdgfrl	68797	8+:42011694-42076120
14472	Ahcy	269378	2-:154885427-154900133
14473	6330534C20Rik	434778	X+:50144385-50152668
14475	Abcg1	11307	17+:31194685-31252717
14476	Akr1b8	14187	6+:34304163-34318454
14479	Ada	11486	2-:163552550-163575897
14481	Csde1	229663	3+:102832743-102861048
14486	Sox7	20680	14+:64562542-64569569
14488	Tshb	22094	3-:102581320-102586637
14495	A630018P17Rik	245695	X-:162940250-162941329
14496	MMT00072975		
14497	Xirp2	241431	2+:67343559-67364099
14498	Layn	244864	9-:50864907-50885159
14503	Sh2d1b2	545378	1+:172178263-172181783
14506	Slc15a3	65221	19+:10917033-10932393
14507	Ttc13	234875	8-:127195233-127212525
14510	Olfr643	259081	7-:111207170-111208114
14511	Sostdc1	66042	12+:37040812-37045032
14512	Tubg1	103733	11+:100981444-100987731
14513	Arpc3	56378	5+:122846820-122855999
14517	Clcn4-2	12727	7+:7251292-7252146
14519	Trex1	22040	9-:108960492-108961435
14520	Stmn1	16765	4+:134026580-134029316

14524	Rarres2	71660	6-:48519696-48522669
14526	Pacsin3	80708	2+:91097064-91104826
14529	Rax	19434	18-:66094291-66098731
14531	Mmel1	27390	4+:154243744-154269639
14540	Tmem140	68487	6+:34818514-34824009
14543	Cfl2	12632	12-:55959807-55963864
14546	1810011H11Rik	69069	14+:33599146-33631153
14550	Acot7	70025	4+:151552242-151645956
14554	Dll4	54485	2+:119151564-119161392
14556	Txndc14	66958	2-:84511475-84518204
14557	Pyroxd1	232491	6+:142294190-142311144
14558	Arhgef15	442801	11-:68756656-68770365
14559	Frrs1	20321	3+:116603514-116606667
14560	Slc16a11	216867	11+:70027410-70029937
14563	Dna2	327762	10+:62409828-62436926
14564	Mrpl49	18120	19-:6053629-6057751
14565	Rnf207	433809	4-:151679011-151693013
14570	Erdr1	170942	
14571	MMT00079617		2-:146580250-146581487
14575	Slc39a10	227059	1-:46863562-46892996
14577	Cdc37l1	67072	19+:29064983-29092059
14578	6720475J19Rik	68157	19-:9910227-9911446
14579	2610015P09Rik	212153	16+:43898202-43963808
14581	Casc5	76464	2+:118872767-118929850
14582	Mt1	17748	8+:96703210-96704092
14584	Vps24	66700	6+:71493892-71530212
14585	Bxdc1	67239	10-:39943302-39966808
14593	Tmem25	71687	9-:44601861-44607299
14597	Col19a1	12823	1-:24270832-24594250
14600	Pgf	18654	12-:86507592-86518230
14601	MMT00038159		5+:13080735-13082866
14603	Ly6e	17069	15+:74788206-74789761
14607	Dbndd1	72185	8-:126029587-126039252
14611	Ddx60	234311	8+:64479366-64516152
14616	4933421E11Rik	321000	3+:106524843-106539492
14617	MMT00033859	26930	19+:55920381-55923221
14619	4930403L05Rik	67395	X-:14687736-14688615
14620	Tbc1d8	54610	1-:39428343-39459755
14621	Six4	20474	12-:74200596-74214404
14626	Zbtb2	381990	10+:5971607-5979466
14628	MMT00019519		18+:35654872-35655659
14629	Nfkb2	18034	19+:46380107-46386578
14631	Stag3	50878	5+:138721778-138753620
14636	Parg	26430	14+:33015405-33110403
14640	Tmem97	69071	11-:78355319-78364237
14641	MMT00000142		

14643	Ets2	23872	16+:95924113-95940752
14645	Mbd3l2	234988	9+:18247989-18249899
14647	Cst3	13010	2-:148697479-148701137
14648	Cdkn1b	12576	6+:134870720-134874325
14649	5430405G05Rik	108832	2+:151527646-151533040
14650	2810433K01Rik	66468	18-:74356419-74367433
14656	Grip2	243547	6-:91713442-91738438
14657	Mefv	54483	16-:3707221-3718097
14662	MMT00070823		7+:29319790-29320109
14664	A430093A21Rik	227195	1-:63004992-63104775
14667	Fggy	75578	4+:95224198-95467357
14669	Pftk2	271697	1+:59314659-59409292
14676	Mecr	26922	4+:131399385-131423682
14677	S100b	20203	10+:75719779-75722812
14681	Alg12	223774	15-:88635675-88649710
14683	Dennd1c	70785	17-:57204742-57217938
14684	Rassf3	192678	10-:120849618-120913306
14685	Foxd1	15229	13+:99124209-99126647
14690	Atf4	11911	15+:80085930-80087934
14691	Nckap1	50884	2-:80340681-80421133
14695	1700025E21Rik	75647	6+:30462303-30469963
14697	Mtrf1	211253	14+:79797662-79823395
14698	Dnajb9	27362	12-:45306882-45311027
14699	V1ri3	171254	13+:22446214-22447116
14702	Krt71	56735	15-:101564380-101573528
14705	Slc43a1	72401	2+:84679551-84703750
14706	MMT00014463		4+:109302638-109303400
14709	Rab36	76877	10+:74499827-74515671
14714	Ccdc102a	234582	8-:97426769-97441331
14717	Srpk3	56504	X+:71019760-71024261
14718	Meox2	17286	12+:37835141-37906115
14720	Lcn9	77704	2+:25678668-25681057
14725	MMT00013262		2+:87366204-87366895
14726	Slc19a1	20509	10+:76496003-76513171
14731	BC006965	217294	11-:112548958-112572655
14733	Fhit	14198	14-:10382613-11994549
14734	Nrsn2	228777	2-:152194491-152202346
14736	Pigr	18703	1+:132723356-132748150
14737	Cldn12	64945	5-:5505112-5514915
14740	Mfap1b	100034361	2-:121286459-121299760
14750	Bves	23828	10+:45055577-45089512
14752	MMT00052718	100041004	5-:143285576-143289203
14753	Slc25a19	67283	11-:115476296-115489453
14754	Atoh8	71093	6-:72156172-72185290
14757	Hdx	245596	X+:108685773-108687484
14760	Mylc2pl	59310	5+:137169015-137176964

14761	Kank2	235041	9-:21572520-21602947
14764	Ugt2b34	100727	5-:87318777-87335983
14767	Dpy19l3	233115	7-:36501524-36507849
14768	1110020G09Rik	68646	15+:9001008-9040234
14770	Cops7a	26894	6-:124909816-124912716
14773	Anxa13	69787	15-:58173022-58220799
14778	Anln	68743	9-:22136459-22193615
14779	Dsg3	13512	18+:20668781-20699833
14781	Mars2	212679	1+:55294046-55295959
14785	F3	14066	3+:121426598-121437502
14793	1700003M02Rik	69329	4-:34635823-34663664
14794	Sprr2f	20760	3+:92169796-92170364
14797	Ecsit	26940	9-:21876689-21889826
14798	Reep2	225362	18+:35000311-35007108
14802	Fbxo16	50759	14+:65885537-65940338
14804	Naalad2	72560	9-:18127706-18151934
14806	Ccdc45	320162	11+:106650567-106680173
14810	1700010H22Rik	75500	5-:98982647-98995943
14811	Slc12a7	20499	13+:73901144-73954186
14815	Lpin3	64899	2+:160706468-160731528
14816	Pcdhb9	93880	18+:37560713-37563093
14818	Il17ra	16172	6+:120413251-120433051
14822	Nrsn1	22360	13-:25343908-25361818
14825	Ube2h	22214	6-:30163017-30254274
14829	1110007M04Rik	68493	4+:24825275-24829362
14830	Olfr421	258715	1+:176081648-176082594
14831	Olfr806	258546	10-:129175030-129175971
14832	Acaca	107476	11+:84160204-84215160
14838	MMT00070887		2-:41841492-41844385
14842	Gipr	381853	7-:19742279-19750373
14843	MMT00081492		14+:101743797-101744459
14845	Tmem188	382030	8+:90642675-90654298
14847	MMT00003977		17-:54051087-54088615
14850	MMT00057793		19-:46748724-46749775
14854	MMT00017294		19+:61243042-61244660
14858	Itpr3	16440	17+:27252928-27259167
14859	Hspa1a	193740	17-:35106944-35108870
14860	MMT00051231	100041002	2-:29700512-29701882
14862	Dhrs7	66375	12-:73751342-73765766
14864	Cdk4	12567	10+:126501291-126504076
14866	Kctd2	70382	11+:115283286-115292592
14867	MMT00040108		17-:3130154-3130512
14868	Tmem159	233806	7+:127245939-127264500
14869	S100a8	20201	3+:90473006-90473956
14870	Capn2	12334	1-:184397390-184447655
14877	Ptx3	19288	3+:66023884-66029726

14878	MMT00036534		1-:22019412-22020696
14879	EG636544	636544	7-:88367247-88367760
14880	Ms4a4c	64380	19+:11482190-11501267
14883	Itgal	16408	7+:134439906-134478650
14884	Mrps22	64655	9-:98489157-98502078
14888	Abca8b	27404	11-:109795579-109857163
14891	MMT00078932		11-:104540610-104541570
14892	Armc3	70882	2+:19120926-19231858
14894	G0s2	14373	1-:195098355-195099286
14898	Orm1	18405	4+:63005599-63009196
14905	Slamf6	30925	1+:173847667-173873998
14909	Jazf1	231986	6-:52995950-53031285
14911	4930544G11Rik	67653	6+:65902602-65904008
14912	Gpr1	241070	1-:63229265-63260783
14913	Yipf4	67864	17+:74888855-74899313
14914	Rnf111	93836	9-:70273236-70351532
14915	MMT00001646		X-:10442126-10442855
14917	Gjb5	14622	4-:127032440-127035365
14919	Pex13	72129	11-:23548509-23565868
14920	Folr1	14275	7-:109006845-109012659
14924	Hn1l	52009	17-:25079100-25097586
14928	Eme2	193838	17-:25029092-25032086
14929	2810432D09Rik	69961	4-:41659066-41660535
14930	C4bp	12269	1-:132532505-132558209
14934	Dcx	13193	X-:140298045-140367637
14935	Ppfia1	233977	7-:151662663-151738438
14938	Sfrs10	20462	16-:22245879-22265975
14945	Mmp3	17392	9+:7445821-7455972
14946	Txndc5	105245	13-:38592145-38620320
14951	Dpep3	71854	8-:108497419-108503319
14952	Tex2	21763	11-:106363451-106441824
14953	Actc1	11464	2-:113873024-113878547
14954	Rab19	19331	6+:39330849-39340376
14957	Lgr5	14160	10-:114888695-115024549
14959	Cacna2d1	12293	5+:15440605-15876867
14962	Casd1	213819	6+:4579999-4593018
14963	4930568D16Rik	75859	2-:35209737-35223250
14965	Csnk2b	13001	17-:35253140-35258392
14966	Ddx51	69663	5+:111085348-111087216
14969	EG245506	245506	X+:85975211-85977435
14973	Shf	435684	2-:122174668-122194493
14974	Serpinb9f	20709	13+:33415924-33424148
14985	Dspp	666279	5+:104599719-104609118
14989	Map3k5	26408	10+:19654288-19861527
14993	MMT00015245		
14995	MMT00042618		X-:87989990-88001685

15000	Tbx18	76365	9-:87599499-87626020
15002	Ncapg	54392	5+:46061159-46091364
15004	MMT00067307	433111	17+:45871454-45886306
15005	Loxl1	16949	9-:58136405-58148560
15007	MMT00030946		6-:91270533-91271029
15008	Chrdl2	69121	7+:107155009-107183238
15013	Nfkb1	18033	3-:135247657-135354295
15014	Mrp63	67840	14+:58445075-58447582
15015	G3bp1	27041	11+:55283253-55314387
15016	MMT00074608		8+:41940765-41941725
15017	Galnt13	271786	2+:54288900-54369092
15021	Ppargc1a	19017	5-:51848896-51945100
15022	Tmem14c	66154	13+:41111660-41117947
15024	2310061J03Rik	66391	16-:55973389-55974663
15027	Brd7	26992	8-:90856210-90886052
15028	MMT00009785		8+:102188070-102188756
15029	Nudt16l1	66911	16+:4939110-4941023
15031	4930415O20Rik	73863	15+:98415681-98420011
15033	Olfr126	258892	17+:37987538-37988498
15036	2410004B18Rik	66421	3+:145600995-145607237
15039	Zmynd15	574428	11+:70274407-70279701
15046	Arl6ip2	56298	17-:80249516-80252264
15047	MMT00028841		6+:67811145-67811719
15049	Rnf125	67664	18+:21119968-21142500
15051	Nin	18080	12-:71119741-71212945
15052	Kifc3	16582	8-:97623733-97634324
15056	D930020B18Rik	216393	10+:121104461-121127004
15057	Atad2	70472	15-:57925597-57966638
15059	Mogat1	68393	1+:78508465-78534738
15062	P2ry5	67168	14+:73637698-73639440
15066	Tmcc1	330401	6-:115971187-115993823
15069	MMT00014274		4-:84822378-84889932
15072	Commd9	76501	2+:101726418-101741796
15074	Usp18	24110	6+:121195974-121220934
15078	Slc1a3	20512	15-:8585807-8660743
15079	Plcd3	72469	11-:102931617-102962920
15080	MMT00054557		3+:4211712-4213844
15084	Ccl3	20302	11-:83461346-83462859
15086	EG435977	435977	7-:54988387-54989356
15088	2810452K22Rik	67236	12-:112111374-112127309
15089	Pla2g7	27226	17+:43705660-43748996
15094	Pbx4	80720	8+:72356602-72396190
15095	0610005C13Rik	71661	7-:52823157-52830696
15097	Arl13b	68146	16-:62812971-62846868
15103	Olfr473	258771	7+:115077035-115077968
15107	Cxcl14	57266	13-:56390004-56397912

15108	Fuca1	71665	4+:135476679-135495193
15115	9030420J04Rik	71544	9-:9115689-9155364
15119	EG545921	545921	7-:16065517-16067128
15121	Itln1	16429	1-:173448372-173461885
15122	MMT00063982		X-:57829621-57831971
15125	Oxtr	18430	6-:112425758-112439935
15126	Megf8	269878	7+:26111398-26150937
15127	Cct6b	12467	11-:82532751-82577808
15129	MMT00030044		2+:82783788-82880349
15131	Atf6	226641	1-:172637344-172797903
15132	MMT00007672		7-:110975180-110976389
15135	Col5a3	53867	9-:20574493-20619478
15137	Olfr1403	258645	1+:175200307-175201240
15140	Slc47a1	67473	11-:61156903-61191566
15151	Cdh11	12552	8-:105157384-105244409
15152	F730047E07Rik	212377	4+:24423642-24430557
15153	Foxm1	14235	6+:128313108-128324304
15154	Dhx29	218629	13+:113717662-113759639
15157	Eno1	13806	4+:149611363-149622981
15159	Timd4	276891	11+:46652762-46657820
15160	Vezf1	22344	11+:87881863-87896043
15161	Ppp4r1	70351	17+:66169484-66191186
15162	Il18	16173	9+:50373472-50389942
15164	MMT00030321		
15167	MMT00007970		10+:74283466-74283958
15169	Scp2	20280	4-:107716445-107791112
15170	Tspan17	74257	13+:54890821-54898129
15172	Cul1	26965	6+:47434858-47475631
15174	3110001D03Rik	66928	4-:74923261-74924191
15177	Bmpr2	12168	1+:59821480-59927703
15180	Asgr1	11889	11+:69867916-69871388
15181	Enpp4	224794	17-:44233177-44242756
15187	Nradd	67169	9-:110523640-110526957
15189	BC005764	216152	10-:79327527-79337380
15194	MMT00078831		7+:132218918-132219345
15195	Slc25a1	13358	16-:17925310-17928291
15200	Gk2	14626	5-:97884179-97886027
15203	Prss3	22073	6-:41323812-41327596
15205	MMT00030262		2+:14642750-14644166
15208	Tmem70	70397	1+:16655298-16668354
15210	Osr1	23967	12+:9585897-9587694
15211	Casc4	319996	2+:121692991-121761762
15212	Oaz3	53814	3-:94237311-94240538
15218	V1rc27	171200	6+:57354457-57355383
15219	0910001L09Rik	66096	5+:138696959-138700624
15223	MMT00004769		5+:21104556-21105366

15224	Cyp2a4	13086	7+:26685881-27100106
15225	Zrsr2	22184	X-:160373374-160396521
15227	Ifngr1	15979	10+:19311791-19330035
15229	MMT00008641		12+:101703793-101726600
15235	Ankrd13d	68423	19-:4270178-4283104
15239	Ube2q2	109161	9+:54997211-55055335
15241	EG636752	636752	6-:69349797-69367189
15247	Cstb	13014	10+:77888536-77890183
15248	Egr4	13656	6-:85461115-85463536
15249	Serpina4-ps1	321018	12+:105318854-105325131
15250	Hoxd3	15434	2+:74581984-74585663
15252	MMT00019402		11+:50920177-50920984
15255	Ccl8	20307	11+:81928684-81930297
15257	Zbtb32	58206	7-:31374700-31376922
15258	Coil	12812	11+:88835263-88852925
15260	MMT00021250		11+:53972427-53973557
15261	Vmn2r109	627814	17-:20677482-20692206
15264	Flywch2	76917	17-:23913884-23923044
15266	Cnnm4	94220	1+:36528829-36563794
15267	Mdga2	320772	12-:67572175-67790514
15271	Dbf4	27214	5-:8397011-8422606
15273	Relb	19698	7-:20191572-20214625
15276	Eif4e	13684	3+:138190241-138220156
15277	2810002N01Rik	68020	12+:112951481-112972402
15279	Clps	109791	17-:28695158-28697659
15280	Cxcl10	15945	5-:92775666-92777880
15281	V1rc22	171195	6-:58384931-58385839
15284	EG632778	632778	7-:26399640-26400910
15286	MMT00073158		4-:69615112-69616312
15287	St6galnac5	26938	3-:152484007-152645174
15288	Pet112l	229487	3+:85378048-85458085
15292	2010107G23Rik	69894	10-:61570959-61573761
15293	MMT00001423		16+:50111185-50112217
15295	Agbl5	231093	5+:31192538-31209337
15296	Mier3	218613	13+:112490993-112505883
15297	MMT00048291		7+:76354769-76355794
15298	1190003J15Rik	76974	7+:148020915-148023865
15299	Mrps21	66292	3-:95666655-95675404
15301	Dyrk2	69181	10-:118290436-118294066
15303	Spcs3	76687	8-:55605785-55615329
15304	Kin	16588	2+:10002209-10014326
15306	Dpy30	66310	17-:74698820-74715742
15308	Mcc	328949	18-:44589266-44693954
15309	Aldh3a2	11671	11-:61058357-61080614
15310	Mlxipl	58805	5+:135582785-135613755
15314	Olfr202	258997	16-:59283399-59284321

15316	MMT00077385		11+:19620238-19621473
15317	Lrrk1	233328	7-:73426513-73437307
15318	Aars2	224805	17+:45643802-45657790
15320	MMT00017479		1+:7167814-7169828
15321	Pus7	78697	5-:23246467-23289513
15322	Rasl12	70784	9+:65246282-65260509
15324	Tep1	21745	14-:51443730-51490217
15325	MMT00007852		2-:127385145-127385244
15327	Olfr1018	258579	2+:85663129-85664063
15329	Olfr1033	258571	2+:85881473-85882404
15334	Rnaseh2c	68209	19+:5601884-5602932
15336	Dpp4	13482	2-:62169775-62249965
15339	Ptges3	56351	10+:127496229-127513513
15340	Cdk5rap1	66971	2-:154161123-154198455
15345	Tcstv3	236219	Un_random-:3053-3772
15348	Eif2ak2	19106	17-:79253849-79281882
15352	Socs2	216233	10-:94875005-94877825
15353	Ednrb	13618	14-:104215877-104242896
15360	Tcfe3	209446	X+:7339826-7352326
15361	Mrm1	217038	11-:84626564-84633017
15365	Ext2	14043	2-:93535789-93662702
15366	Ces7	67935	8-:96038094-96058612
15367	Vegfc	22341	8+:55162979-55271803
15375	Rnd3	74194	2-:50985960-51004631
15378	Exdl2	97827	12+:81576980-81599106
15380	AW209491	105351	13+:14728564-14730468
15384	Mat2a	232087	6-:72383693-72389492
15386	Tex12	66654	9-:50364923-50369430
15387	Mgl1	17312	11+:69980275-69984335
15388	Gcap14	72972	14-:37711396-37781902
15393	Sec22a	317717	16-:35313498-35349083
15396	MMT00005917		6+:6852812-6853431
15400	Adcy8	11514	15-:64530595-64753722
15403	C330046G03Rik	252974	10+:77292211-77344030
15404	Ireb2	64602	9+:54711590-54760298
15406	Mdfi	17240	17-:47952278-47970168
15407	Kcnn3	140493	3+:89324342-89471326
15408	Mkln1	27418	6+:31348844-31459475
15413	Brp44l	55951	17+:8476721-8490506
15414	Arhgap18	73910	10+:26492332-26638451
15417	5830433M19Rik	67770	4-:94166775-94223770
15423	Tinag	26944	9-:76799591-76893530
15424	Atp6v1g2	66237	17+:35373708-35374743
15425	Adprh	11544	16-:38445769-38450521
15426	1700108M19Rik	71156	12-:36934932-36979987
15427	MMT00009375		10+:121542301-121607417

15433	Myb	17863	10-:20844741-20880790
15436	3000002C10Rik	378954	9-:109732887-109734003
15438	Tctex1d1	67344	4+:102658986-102677173
15445	Guca1a	14913	17-:47531592-47537385
15446	Cd320	54219	17+:33980115-33985372
15447	Eif4a3	192170	11-:119149677-119161357
15449	Mitd1	69028	1-:37935731-37947277
15451	Ftcd	14317	10+:76038430-76052304
15452	Adora3	11542	3+:105673820-105726968
15454	MMT00010886		2+:111022637-111027656
15455	Lsm4	50783	8+:73197290-73202646
15456	Chi3l3	12655	3-:105950471-105970439
15457	Olfr430	258713	1+:175999485-176000382
15460	D130059P03Rik	320538	6+:38383932-38441932
15461	Galnt2	108148	8-:126868394-126869620
15466	Gm136	214568	4-:34690809-34693907
15467	Cfc1	12627	1+:34592492-34601156
15474	Wfdc15b	192201	2-:164040190-164047396
15476	Fem1b	14155	9-:62643837-62659186
15479	Pde6g	18588	11-:120309344-120314743
15480	MMT00078167		7+:119796187-119797434
15481	1110067D22Rik	216551	11-:20723360-20731110
15483	Il17rb	50905	14-:30809385-30821995
15484	Lrrc42	77809	4-:106906120-106926138
15485	Zfp251	71591	15-:76681720-76765960
15489	Tmem158	72309	9-:123168919-123217057
15492	Efnb1	13641	X+:96331507-96343567
15497	Ftl1	14325	7-:52713317-52715205
15499	Dact1	59036	12+:72414878-72421079
15500	MMT00081411		2+:15318470-15319347
15501	Smad7	17131	18+:75527182-75555581
15504	Mx2	17858	16+:97757689-97782506
15508	4632415L05Rik	70808	3+:19794911-19797219
15509	Gabra6	14399	11-:42119939-42134510
15512	Mitf	17342	6+:97941785-97968696
15521	Mast3	546071	8-:73302016-73321860
15522	1810011O10Rik	69068	8-:25548087-25549418
15523	Krtap5-2	71623	7-:149360439-149361910
15526	Pnliprp2	18947	19+:58834218-58852018
15529	MMT00011590		7-:110975180-110976389
15531	Prickle4	66119	17-:47823594-47825047
15533	Olfml2b	320078	1+:172574646-172612919
15536	Garnl4	380711	11-:74196864-74250638
15538	B130024G19Rik	434198	7+:77533734-77556033
15539	MMT00028325	100040304	X+:67653506-67655496
15540	C2	12263	17-:34999552-35018992

15544	Ccdc71	72454	9+:108362907-108366865
15550	2010200O16Rik	67495	3-:108359344-108365297
15557	MMT00080723		17-:15406076-15458846
15558	Pnp1	18950	14+:51567526-51571295
15559	A2m	232345	6+:121586281-121629237
15560	Lrrc25	211228	8+:73141459-73145384
15565	2310001A20Rik	71881	2-:150408816-150434259
15566	Snn	20621	16+:11073786-11075062
15567	1600002H07Rik	72016	17-:24352002-24357795
15569	BC003331	226499	1-:152209662-152237483
15570	MMT00017284		8+:36878364-36880464
15572	Dtx3l	209200	16-:35926599-35933486
15573	Aipl1	114230	11-:71842666-71850960
15575	Bckdhb	12040	9+:83818761-84017846
15576	Mboat5	14792	6+:124613145-124654196
15577	Glt8d4	232313	6+:100683177-100755070
15578	Lrrc3b	218763	14-:16190033-16269019
15580	MMT00077001		17-:34881932-34883717
15581	Olfr601	258311	7-:110506762-110507706
15582	Klrk1	27007	6-:129562433-129572790
15585	MMT00033125		8-:58452400-58453004
15587	Actl6b	83766	5+:137994862-138010772
15588	Kndc1	76484	7+:147081085-147113170
15593	Rit2	19762	18-:31133968-31476722
15594	Gm1967	381411	2-:93695519-93698127
15597	Cplx3	235415	9-:57449182-57454038
15601	Plekhm3	241075	1-:64984403-65003320
15602	Sctr	319229	1+:121940027-121960107
15604	Arl4a	11861	12-:40762408-40764005
15605	Cyp2c39	13098	19+:39585360-39643021
15606	MMT00008386		2+:127515802-127516731
15607	Klf13	50794	7-:71036394-71083735
15609	Mreg	381269	1-:72207087-72238778
15614	Tpr	108989	1+:152239964-152297434
15615	Zfp800	627049	6-:28191311-28211926
15616	Pon2	330260	6-:5215227-5248308
15617	Atm	11920	9-:53247472-53342071
15618	Sel1l	20338	12-:93079898-93087581
15620	Cenpb	12616	2-:131003813-131005612
15624	Crcp	12909	5+:130505175-130536651
15625	Capg	12332	6+:72494530-72512966
15626	MMT00035685		19-:11109903-11110324
15629	Asb7	117589	7-:73792673-73824176
15631	Lpin1	14245	12-:16542763-16596555
15632	Gsc	14836	12-:105709418-105711446
15635	Crkrs	69131	11+:98064616-98112360

15641	5230400G24Rik	75734	1+:82721493-82748962
15642	Ugt2b38	100559	5-:87838971-87853213
15645	Mrpl18	67681	17-:13104220-13108957
15657	Zfyve28	231125	5-:34537523-34540235
15658	Slfn8	276950	11-:82794837-82831217
15659	MMT00074093		16-:64917105-64918916
15664	Masp2	17175	4+:147976662-147978522
15665	Blcap	53619	2-:157382098-157392052
15668	1810032O08Rik	66293	11+:116532973-116537110
15670	Pnmal1	71691	7+:17545105-17546860
15672	Ahi1	52906	10+:20672345-20800226
15674	Riok1	71340	13+:38129163-38153298
15675	MMT00077535	641192	5-:23698515-23715426
15676	Fads1	76267	19+:10257462-10271364
15677	Slc9a9	331004	9+:94839907-95130859
15678	MMT00016379		12+:99082690-99083258
15680	2810030E01Rik	72668	2-:17967238-17969966
15688	Tmem71	213068	15-:66358570-66392665
15695	Tead4	21679	6-:128220875-128250843
15696	Pycr2	69051	1+:182834439-182838218
15697	Tbc1d8b	245638	X+:136237690-136240027
15698	Them2	66834	13-:24909823-24923358
15700	Crocc	230872	4-:140572559-140603044
15702	MMT00047561	64164	1+:157888023-157899927
15704	MMT00038941		1-:24601056-24602334
15706	4930503L19Rik	269033	18-:70611866-70632412
15708	Retsat	67442	6+:72548592-72558357
15709	Tceal1	237052	X+:133242603-133244403
15711	Anxa1	16952	19-:20447921-20465163
15713	Rbm3	19652	X-:7719491-7722807
15715	Tnfrsf18	21936	4+:155400450-155402998
15718	Macrod2	72899	2+:142082252-142200047
15719	BC060267	212516	6-:115760754-115773729
15720	Trp53i11	277414	2+:93027756-93041908
15721	MMT00007615		
15722	Ccdc9	243846	7-:16859387-16872110
15723	Csf1	12977	3-:107543974-107563140
15729	MMT00052703		
15731	Mfhas1	52065	8+:36651426-36740304
15738	2900093L17Rik	78508	4-:45247601-45256865
15740	1810010H24Rik	69066	11+:106889123-106891752
15742	2900062L11Rik	76976	X+:132528369-132529890
15743	Hoxc6	15425	15+:102839909-102842313
15744	Iqcf1	74267	9+:106402311-106404579
15745	MMT00077003		5-:143614122-143614803
15746	Calr	12317	8-:87365989-87370765

15747	Sfrs1	110809	11+:87860314-87863106
15748	MMT00032580		Y_random-:40879070-40880061
15749	Cd22	12483	7-:31650847-31665286
15752	Cyp4a14	13119	4-:115158750-115168737
15756	MMT00043772		3+:65197258-65199105
15758	Stx4a	20909	7+:134985552-134992164
15760	Trib3	228775	2-:152163160-152169768
15763	Vmn2r-ps14	545700	4+:145054108-145057441
15766	Bmp2	12156	2+:133378743-133387451
15767	Tmem174	68344	13-:99404933-99407312
15768	Suhw4	235469	9+:72208021-72211305
15769	Pkhd1l1	192190	15+:44413298-44432897
15773	MMT00069895		5+:107899726-107920599
15775	Nat11	70999	19-:7299765-7315674
15776	Hectd2	226098	19+:36643860-36673436
15778	MMT00061514		7+:11390635-11391334
15780	Pde4d	238871	13+:110693294-110742244
15787	Wdr92	103784	11+:17111929-17133773
15789	Dnahc11	13411	12-:119116455-119437516
15792	Use1	67023	8+:73891124-73893628
15794	Olfr589	259054	7-:110303307-110304259
15795	Col1a2	12843	6+:4455760-4490729
15800	MMT00023224	66449	16-:4616474-4624937
15801	Efna2	13637	10+:79642458-79652437
15806	Dennd4c	329877	4+:86420141-86496504
15809	Zfp365	216049	10-:67349535-67375165
15813	Vpreb3	22364	10+:75411102-75412207
15815	MMT00080242		15-:26658038-26659479
15819	MMT00055433	545798	5-:116490544-116533256
15820	Stard9	668880	2+:120490615-120519550
15821	Pcp4	18546	16+:96689373-96747394
15824	D19Ertd721e	225896	19+:8946048-8950145
15825	Fermt3	108101	19-:7073466-7093928
15826	Arl4d	80981	11+:101526874-101529143
15827	Rrm1	20133	7+:109590457-109617441
15830	Sema5a	20356	15+:32347113-32626085
15832	Crybb2	12961	5-:113487286-113497786
15834	9030611O19Rik	104943	12+:31758858-31763850
15835	Sar1a	20224	10+:61143078-61156042
15836	Hpn	15451	7-:31883743-31900294
15837	Plg	18815	17+:12571511-12612250
15839	MMT00045078		16+:23033913-23050925
15841	Ndufb6	230075	4-:40217686-40226440
15842	Med12l	329650	3+:59031604-59122331
15843	Tnn	329278	1-:162015165-162077971
15844	Klhdc9	68874	1-:173288536-173290949

15847	Gm392	235858	8-:57910790-57912361
15849	Pacs2	217893	12+:114252871-114310783
15853	D430036J16Rik	319405	9+:81525957-81538235
15859	MMT00057272		X+:135791423-135792688
15860	BC004004	80748	17+:29405895-29438470
15862	Mrap	77037	16+:90738354-90750032
15864	Rps6ka2	20112	17+:7374635-7507662
15870	Aqp5	11830	15+:99421212-99425252
15872	4933424B01Rik	71177	6-:146498154-146526281
15875	1700072E05Rik	73495	X+:83289537-83292652
15876	Eif4ebp2	13688	10-:60895244-60915417
15881	Hcn4	330953	9+:58671318-58708569
15882	AA986860	212439	1+:132628605-132641198
15883	Tulp3	22158	6-:128271183-128305812
15884	Olfr395	259007	11-:73720053-73720992
15885	Adamts7	108153	9+:90066311-90094931
15887	Lce1m	66203	3-:92821731-92822982
15890	Fbxw2	30050	2-:34660864-34678701
15892	Slc22a5	20520	11-:53678525-53705143
15895	MMT00080717		17-:15672028-15672655
15896	Pdgfrb	18596	18+:61205094-61244713
15897	D10Jhu81e	28295	10-:77624811-77632513
15899	MMT00058392		3-:113084762-113089679
15901	Tpm3	59069	3+:89876658-89896047
15904	Ncl	17975	1-:88247194-88255798
15907	Hyou1	12282	9+:44188602-44199131
15908	Pebp1	23980	5-:117732662-117737555
15911	Glra4	14657	X-:133292235-133310450
15914	Dapk2	13143	9+:66006039-66120046
15915	Spata21	329972	4+:140658888-140660882
15916	Il1rap	16180	16+:26581853-26716652
15917	3100002J23Rik	75429	11-:58606297-58615467
15918	Fkbp11	66120	15-:98554802-98558629
15920	A630001G21Rik	319997	1-:87613658-87623200
15922	Psd	73728	19-:46386574-46399419
15924	Rbm38	56190	2+:172847429-172860230
15926	Kctd20	66989	17+:29089444-29104898
15931	Ces3	104158	8-:95689971-95721678
15934	Armcx3	71703	X+:131291137-131295995
15935	Pnpla3	116939	15+:83998266-84019944
15937	Adamts4	240913	1+:173180548-173190027
15938	Cst11	78240	2-:148594344-148597233
15941	MMT00024086		9+:79421614-79422433
15945	1700018L24Rik	75528	8+:11840513-11855760
15951	C330027C09Rik	224171	16+:48994297-49019814
15952	Bai3	210933	1-:25477402-25616710

15953	Acsf3	257633	8+:125309791-125341782
15955	Inadl	12695	4+:98213424-98386293
15956	1700023D19Rik	75510	7+:51964112-51975214
15964	Pglyrp3	242100	3+:91826526-91835484
15967	Atp2b2	11941	6-:113698287-113930577
15969	Tnrc4	78784	3+:94282251-94296115
15970	Npal1	70701	5+:73039050-73059555
15971	Aifm2	71361	10+:61183389-61191059
15973	MMT00025143	100045149	15+:62009747-62016306
15975	Cnnm1	83674	19+:43515700-43570238
15977	Phca	66190	7-:105364953-105458038
15979	Rmi1	74386	13+:58503961-58511451
15982	Syne2	319565	12+:77050326-77068739
15984	MMT00075074		16+:38483773-38484678
15985	Nova1	664883	12-:47798886-47814563
15986	Ahnak	66395	19+:9063770-9151410
15989	MMT00069487		X-:71380375-71381632
15990	Dnmbp	71972	19-:43921301-43977548
15994	9130213B05Rik	231440	5+:91946725-92053020
15995	Scg3	20255	9-:75491180-75531824
15996	Grtp1	66790	8-:13176869-13200535
15997	Tspan8	216350	10+:115254339-115286671
15999	3110039M20Rik	67293	12+:50490873-50508335
16000	Nol10	217431	12+:17355256-17436900
16001	D330027H18Rik	101359	6-:29119237-29119830
16002	Mia2	338320	12+:60196786-60205545
16003	Rps6kb1	72508	11-:86313645-86358258
16006	Anxa2	12306	9+:69312276-69339593
16013	Nt5c3	107569	6-:56832398-56873823
16018	MMT00074939		18+:87401389-87403116
16024	P2ry6	233571	7-:108086144-108112884
16027	Ppp2r2d	52432	7+:146027800-146074737
16028	Rtn4r	65079	16+:18127798-18152499
16029	Igh-VJ558	16061	12-:117158466-117158975
16030	Prpf3	70767	3-:95634539-95659711
16031	Stab2	192188	10-:86303954-86470687
16032	Cyp27a1	104086	1+:74778500-74784464
16034	Dclk3	245038	9+:111370115-111391741
16035	Chd8	67772	14-:52842273-52857247
16037	MMT00011022		3-:84355935-84357187
16040	Slc22a1	20517	17-:12841741-12868559
16043	Sult1c2	69083	17-:53969185-53985143
16046	Ppwd1	238831	13-:104995204-105019060
16047	Irf8	15900	8+:123260290-123280318
16048	Nsfl1c	386649	2+:151319921-151337038
16051	1700001C02Rik	75434	5+:30768449-30786460

16052	Tsg101	22088	7-:54144745-54175263
16055	Slco4c1	227394	1-:98715153-98717906
16060	Gys2	232493	6-:142371133-142421467
16062	EG545741	545741	X+:117274705-117275324
16066	Coq2	71883	5-:101083745-101103265
16067	Pcaf	18519	17+:53706654-53810192
16068	1700008P20Rik	69301	7-:25118100-25118955
16072	MMT00011155		8-:71997409-71999134
16073	Car11	12348	7+:52955375-52959979
16074	Prkcc	18752	7+:3303847-3330721
16076	MMT00039676		X-:123218651-123219246
16077	Mocs2	17434	13+:115608493-115618932
16078	Defb15	246082	8-:23040284-23043182
16079	MMT00078587		6+:12515690-12516881
16080	Prkcdbp	109042	7-:112628596-112630726
16082	Abcg4	192663	9-:44089164-44089774
16083	Pla2g2c	18781	4+:138281661-138300488
16089	4732474O15Rik	238455	12+:120684086-120704145
16093	4732473B16Rik	100342	4+:133036102-133043851
16094	MMT00082062		12-:3931470-3932059
16096	Csmd3	239420	15-:48289041-48505172
16097	Dnmt3l	54427	10+:77512586-77526366
16098	Rps20	67427	4-:3758731-3762746
16100	Tnni3	21954	7-:4469970-4473917
16102	Cd72	12517	4-:43460606-43467435
16104	Ugt3a2	223337	15+:9265367-9300625
16107	Entpd6	12497	2+:150574803-150597410
16112	V1ra1	22296	6+:90078417-90080980
16114	Lyplal1	226791	1-:187911764-187941189
16115	Mup3	17842	4-:61744512-61748405
16117	Id4	15904	13+:48356798-48359405
16118	Cby3	76653	11+:50171254-50173205
16123	Sec11c	66286	18+:65960231-65977311
16128	Agbl1	244071	7+:83569540-83589879
16129	Trafd1	231712	5-:121821735-121835582
16130	Gosr2	56494	11-:103540550-103558972
16131	Ptpre	19267	7+:142729604-142875038
16136	MMT00046779		13+:66699587-66699622
16137	Xrcc5	22596	1+:72353994-72441526
16139	Vkorc1l1	69568	5+:130452856-130458763
16141	Ccdc96	66717	5+:36827240-36829448
16143	Cd209d	170779	8-:3871956-3878483
16145	Olfr1449	258300	19+:13009229-13010172
16146	Gucy1a3	60596	3-:81898547-81949774
16147	Tlr2	24088	3-:83640194-83645475
16152	Ceacam10	26366	7+:25562279-25569670

16153	Syngr1	20972	15+:79921781-79943870
16155	Dusp12	80915	1-:172809790-172811172
16157	D9Ertd280e	272636	9-:99217063-99258936
16159	Igh-VJ558	16061	12-:116861366-116930533
16163	Tmco1	68944	1+:169238391-169260921
16164	Fdps	110196	3-:88897510-88905794
16167	Stbd1	52331	5+:93032065-93035612
16168	Crlf2	57914	5-:109983729-109987960
16170	S100a11	20195	3+:93324418-93330208
16176	St3gal5	20454	6+:72047839-72104564
16178	Myh11	17880	16-:14194992-14291456
16179	Gulo	268756	14-:66605623-66607112
16181	MMT00078660		1-:81738169-81739463
16182	Oasl1	231655	5+:115373414-115387920
16186	Cfp	18636	X-:20502649-20508888
16187	6530403A03Rik	67797	13+:38296813-38318917
16190	Osbpl11	106326	16+:33196166-33243397
16191	Klc1	16593	12+:112997166-113033107
16192	MMT00013307		19-:13697166-13714196
16197	MMT00065977		3-:97994426-97995001
16198	Atp8b4	241633	2-:126148549-126306422
16199	Samhd1	56045	2-:156925131-156960881
16201	MMT00072359		X+:142425310-142429203
16202	Ccnd1	12443	7-:152115836-152125735
16203	Tspan6	56496	X-:130426278-130432874
16204	Olfr586	259115	7-:110270343-110271296
16205	2810002O09Rik	72345	X-:92621905-92623938
16208	Ppara	19013	15+:85566560-85632163
16210	Metrnl	210029	11+:121563725-121577594
16212	Med19	381379	2+:84518581-84528371
16214	Ocln	18260	13-:101267721-101322673
16218	Rpgrip1	77945	14+:52730596-52780605
16219	4932415G12Rik	67723	10-:94136449-94151316
16220	MMT00074659		1+:42160134-42161004
16222	Adipor2	68465	6-:119303169-119367670
16224	Cdc2l5	69562	13-:17809233-17896143
16225	Mak10	78689	13+:59686771-59736140
16226	MMT00033542		5+:73772040-73783378
16227	Rarres1	109222	3-:67282813-67319535
16229	Abcb11	27413	2-:69076345-69180644
16230	MMT00020946		
16231	Rnf4	19822	5+:34692532-34696082
16233	1600027J07Rik	69794	8-:105867777-105871435
16238	Nmi	64685	2-:51804019-51828731
16241	MMT00003591		4+:25346490-25346553
16242	MMT00053207		12+:86596449-86597574

16248	Cdr2	12585	7-:128100555-128125798
16250	MMT00069147	329858	4-:59342087-59440936
16251	G6pc	14377	11+:101229029-101239215
16252	Ubtd1	226122	19+:42056426-42109133
16253	MMT00044478		1-:144989922-144991247
16255	Reg3b	18489	6+:78320878-78323459
16256	Tmem132d	243274	5-:128344937-128751594
16257	Osbpl8	237542	10+:110601872-110734303
16265	Prkci	18759	3+:30894897-30949368
16270	Lgals12	56072	19-:7671150-7681654
16283	EG627427	627427	18+:37079953-37081860
16284	Rasl11a	68895	5+:147656630-147659301
16287	Extl1	56219	4-:133912288-133927694
16289	Myocd	214384	11-:64990068-65083443
16292	Prpf18	67229	2-:4543266-4544429
16294	Chst7	60322	X+:19636695-19674644
16295	Nlrp14	76858	7+:114324671-114341616
16299	Creld2	76737	15+:88650075-88657111
16300	Gab3	210710	X-:72233890-72330246
16307	Lemd1	213409	1+:134122889-134153947
16310	Sae1	56459	7-:16912402-16973134
16315	MMT00048401		2+:149182625-149183140
16318	Ms4a15	545279	19-:11053698-11057785
16322	Lmod3	320502	6-:97188525-97202637
16323	Smpd3	58994	8-:108779158-108789819
16327	Ubfd1	28018	7+:129210935-129222646
16328	Slc10a2	20494	8-:5085622-5105232
16330	2310043J07Rik	69665	5+:136533181-136540189
16331	Setd8	67956	5-:124911790-124912317
16333	Wdr64	75820	1+:177697168-177745862
16334	Parp2	11546	14+:51427613-51440875
16335	Otop1	21906	5+:38691379-38694614
16336	2310040A07Rik	69638	4-:41585176-41587319
16337	Grin2c	14813	11-:115110483-115128557
16338	Senp1	223870	15-:97872400-97917565
16339	Bach1	12013	16+:87699193-87730946
16340	Scrn2	217140	11+:96891307-96895272
16343	Dgkh	380921	14-:78968631-79125492
16344	Arpc5l	74192	2+:38863658-38871392
16346	Phospho2	73373	2+:69627793-69635225
16347	Mgp	17313	6-:136820957-136824269
16348	Tubgcp3	259279	8-:12614274-12672101
16355	Rcl1	59028	19+:29175994-29217781
16356	9030607L17Rik	71564	10+:80165051-80168118
16358	MMT00020436		7-:123947395-123947978
16361	Olfr703	258589	7+:113988126-113989084

16363	Adh5	11532	3+:138106115-138118215
16366	Glis3	226075	19-:28605362-28614898
16367	MMT00076058		8_random+:321154-339848
16370	Olfr115	257908	17-:37746545-37764061
16371	Ppp2cb	19053	8+:34710388-34729662
16372	MMT00077009		14-:37831938-37837696
16378	Ccl8	20307	11+:81928693-81930297
16379	Tppp3	67971	8-:107991396-107995289
16380	Vasn	246154	16+:4639972-4650797
16382	Arpc1b	11867	5+:145882527-145889054
16383	Il20rb	213208	9-:100358138-100375368
16385	MMT00073590		17-:64050829-64051715
16387	2010316F05Rik	67939	11-:29413054-29415033
16388	EG381818	381818	6-:132388168-132391755
16389	MMT00040811		
16390	Plb1	665270	5+:32535080-32619909
16392	EG627648	627648	13-:58110636-58214949
16393	MMT00023354		1-:60579498-60579865
16397	1700112E06Rik	76633	14+:23396948-23418491
16399	Izumo1	73456	7+:52877179-52882598
16401	4932415M13Rik	211496	17+:53863012-53942804
16404	Trappc2	66226	X-:162878732-162891070
16406	Mettl6	67011	14-:32292196-32308216
16411	V1rc12	171185	6-:66628793-66629678
16412	Tank	21353	2+:61416641-61492168
16416	Commd2	52245	3-:57450281-57455629
16417	Pitx2	18741	3+:128916889-128922506
16419	Maml2	270118	9+:13424433-13513976
16420	MMT00020928		
16421	Hrasls	27281	16+:29217697-29228320
16422	Kctd5	69259	17-:24184402-24210411
16424	MMT00046595		9+:39847539-39848323
16425	Agxt2l1	71760	3+:130320492-130338666
16426	MMT00023627		17-:92584656-92585156
16430	Vac14	234729	8+:113160268-113244296
16434	Fhad1	329977	4-:141452341-141478346
16435	MMT00071843		12+:32103596-32105117
16436	Gpr39	71111	1+:127736579-127769750
16437	Tkt	21881	14+:31362364-31386797
16438	Igfl3	232925	7+:18761837-18767674
16440	Gprc5a	232431	6+:135015701-135034723
16443	Ccdc59	52713	10+:105278534-105284564
16447	Cat	12359	2-:103294160-103325277
16448	Vmn2r104	22313	17-:20166388-20178707
16449	BC004044	80752	5+:139269142-139286017
16451	2010007H12Rik	69871	5+:138220145-138221335

16453	Olfr1372-ps1	257871	
16454	2310038H17Rik	67099	1-:64653668-64663801
16456	Il33	77125	19+:29999626-30035205
16457	BC106175	724112	12-:8485906-8487109
16460	MMT00045897		6-:131556442-131559000
16461	Dll1	13388	17-:15504319-15512217
16463	MMT00036516		
16465	Ccdc130	67736	8-:86781693-86794259
16467	Card10	105844	15-:78605567-78633099
16472	1700015C15Rik	75532	13-:64532557-64533750
16474	Bhlhb2	20893	6+:108610690-108616768
16479	Pcsk7	18554	9+:45715079-45737803
16485	Cuedc1	103841	11+:87983065-88006432
16486	Slc25a22	68267	7-:148615650-148623517
16490	MMT00048286		13+:80300564-80301136
16491	6330500D04Rik	193385	13+:24706495-24823952
16492	Hk2	15277	6-:82675043-82724453
16493	Mphosph9	269702	5-:124765197-124766180
16494	2310007D09Rik	71878	2+:158593825-158612371
16495	Gap43	14432	16-:42248744-42340663
16497	MMT00063964		X-:58437015-58439320
16501	Loh11cr2a	67776	9+:38525881-38550877
16502	Chsy1	269941	7+:73254829-73318650
16503	MMT00025844		
16507	A830039N20Rik	268723	14-:24111516-24112033
16508	Foxa1	15375	12-:58641620-58646949
16510	Tspyl4	72480	10+:34017277-34021126
16514	Vps52	224705	17+:34092723-34103841
16515	Gramd4	223752	15+:85898242-85967744
16517	D4Wsu53e	27981	4+:134479559-134483287
16518	4933426G20Rik	74460	15+:63746013-63757008
16519	Igf2bp3	140488	6-:49037340-49164470
16520	MMT00064994	238440	12-:116550353-116550843
16521	Adar	56417	3+:89534726-89555096
16522	Ccdc85a	216613	11-:28476390-28483542
16525	Lbp	16803	2+:158132280-158158032
16526	Sftpa1	20387	14+:41945071-41947765
16528	Ddr2	18214	1-:171907776-172040690
16531	6330403K07Rik	103712	11-:70845451-70846957
16534	Nbea	26422	3-:55429115-55895045
16535	Kctd9	105440	14+:68334351-68360218
16539	Klhl20	226541	1-:163020550-163061582
16540	MMT00017193		8-:78235492-78404744
16547	1500002O20Rik	71997	7+:25184648-25207796
16548	Atp6v1e1	11973	6-:120745720-120772632
16549	Wtap	60532	17-:13159665-13185978

16553	Cilp2	68709	8-:72404266-72407356
16557	Rbpms	19663	8-:34893115-34976877
16559	MMT00001400		16-:50664932-50666467
16562	MMT00005808		18+:7932849-7933510
16564	Tead4	21679	6-:128174301-128202007
16566	Igfbpl1	75426	4-:45822348-45839687
16567	Rpl14	67115	9+:120480633-120483551
16569	Scn1a	20265	2-:66110948-66156196
16571	Pqlc1	66943	18+:80453045-80489453
16572	D14Ertd668e	219132	14-:59967567-59984283
16573	Cryge	12968	1-:65095198-65097687
16576	Ms4a2	14126	19-:11691682-11698133
16579	Rcan2	53901	17+:43938798-43973446
16580	Tnfaip2	21928	12+:112683172-112693228
16584	Nup35	69482	2+:80478987-80499263
16585	Syvn1	74126	19+:6047089-6053707
16586	Adamts8	30806	9+:30750145-30751186
16588	Cnbp	12785	6-:87793167-87801002
16591	MMT00051549		3-:6135078-6139030
16592	Psmf1	228769	2-:151543992-151566993
16598	E430018J23Rik	101604	7-:134534943-134536950
16606	4930579G22Rik	69034	5+:130384434-130387150
16607	Gpx2	14776	12-:77893325-77896493
16608	MMT00026286		17-:36896490-36898247
16609	Gucy2c	14917	6-:136645805-136730245
16613	1110031I02Rik	66179	11-:121038906-121065962
16614	Zscan21	22697	5+:138558268-138575444
16615	Chdh	218865	14+:30822130-30854865
16616	Paox	212503	7+:147311581-147320233
16617	Sh2d4a	72281	8+:70805930-70871479
16618	Hps3	12807	3-:19896620-19935299
16619	Anks1	224650	17+:28111849-28199721
16624	Psma8	73677	18+:14864657-14920807
16627	MMT00074583		8-:41833718-41875442
16629	Sall2	50524	14-:52930848-52948437
16630	Armcx5	494468	X+:132278962-132281857
16632	MMT00062595		X-:51185502-51187617
16636	Tmem189	407243	2-:167468725-167487033
16637	Vmn2r85	623734	10-:129855333-129866512
16639	MMT00078937		11-:105026089-105028054
16640	4930408G06Rik	68179	1-:59053964-59064601
16644	Tcrb-J	21580	6+:41012331-41013101
16647	D16H22S680E	27883	16-:18300919-18324536
16650	Cd300a	217303	11+:114751675-114759133
16654	Oas1f	243262	5+:121297400-121307634
16656	Slc33a1	11416	3-:63747171-63768112

16657	Atpif1	11983	4-:132086470-132089513
16658	MMT00011796	632223	6+:136348473-136350157
16660	Gpc6	23888	14+:117324651-118375766
16661	Tmprss4	214523	9-:44980809-45012158
16664	Car4	12351	11+:84771344-84779541
16669	Tjp2	21873	19-:24169088-24248478
16670	Csmd2	329942	4+:127736205-128241394
16671	MMT00081758		7-:3908044-3935099
16672	Tmem82	213989	4-:141170149-141173939
16675	Flrt2	399558	12+:96930444-97019311
16676	Aspa	11484	11-:73118942-73137989
16678	Olfr669	259045	7+:112087041-112087994
16679	Ctsk	13038	3+:95304756-95312843
16683	Cst8	13012	2+:148624572-148631330
16685	Vwf	22371	6+:125502977-125636695
16690	Tjp3	27375	10-:80735972-80753979
16692	Bat4	81845	17+:35259896-35261751
16693	Dip2a	64451	10-:75725482-75776049
16694	Sepp1	20363	15+:3224665-3230504
16695	Hs3st6	328779	17+:24889935-24895628
16696	Magee1	107528	X+:102316531-102318706
16697	Abcg3	27405	5-:105364784-105411688
16698	Sumo1	22218	1-:59696287-59727607
16701	H2-M10.1	14985	17-:36460962-36463095
16702	2810422J05Rik	75620	8-:73037296-73046994
16704	Ppif	105675	14+:26513655-26519950
16706	MMT00026784		9-:36620616-36629038
16712	Pnrc1	108767	4-:33332211-33335615
16715	2610034M16Rik	69239	17-:59018231-59106888
16718	Zbtb7c	207259	18+:76219111-76306096
16719	Slc43a3	58207	2+:84776820-84798086
16720	Mtmr7	54384	8-:41636453-41694370
16721	Stk3	56274	15-:34806361-35085437
16723	Tnfsf8	21949	4-:63494204-63522205
16729	Smpx	66106	X+:154137049-154190521
16730	Osbpl3	71720	6-:50246805-50332836
16738	Pmch	110312	10+:87553681-87555118
16742	Bbc3	170770	7+:16894986-16903682
16743	Bad	12015	19+:7016350-7026383
16747	Acvr2a	11480	2+:48670221-48756173
16748	Zfp810	235050	9-:22081191-22112082
16749	Ivl	16447	3-:92375271-92376678
16750	Casp6	12368	3+:129608383-129616865
16753	Hfe2	69585	3+:96331871-96333132
16754	Hsp90aa1	15519	12-:111929240-111933891
16755	MMT00000111		10+:24420098-24420906

16756	MMT00047531		1-:129179371-129198052
16757	Tmem146	106757	17+:56800664-56803877
16760	1600029D21Rik	76509	9+:50302629-50313238
16761	MMT00018149		17-:92926783-92927221
16762	Atp8a2	50769	14-:60266586-60705671
16768	Hnmt	140483	2-:23858446-23904896
16770	Krtap16-1	170651	16-:88873909-88874514
16772	MMT00010554		1-:79919112-79920001
16774	4932438A13Rik	229227	3+:36784278-36845428
16775	Kcna10	242151	3+:106996996-106998507
16776	1110032O16Rik	68742	7-:134029658-134066452
16777	MMT00053707		5+:125915438-125916193
16781	Pramef12	77632	4-:143981576-143998367
16782	1110012N22Rik	68515	11-:120507345-120509473
16784	D330012F22Rik	269952	7+:87377802-87393324
16788	Imp3	102462	9+:56785306-56786204
16792	Sh3yl1	24057	12+:31597051-31645021
16793	Arl11	219144	14+:61928686-61930618
16794	Chrnb1	11443	11-:69598122-69609382
16796	Agt	11606	8-:127080487-127088483
16802	Def8	23854	8+:125966904-125987167
16809	Rxrb	20182	17+:34168942-34174475
16811	Rere	68703	4+:149780448-149996071
16814	AU042651	328258	13+:56539761-56575599
16819	Lyve1	114332	7-:117995366-118006467
16820	Bambi-ps1	81913	2+:122292625-122293240
16821	Abcd2	26874	15-:90979314-91022230
16823	Inhbb	16324	1-:121312041-121335320
16824	2310076L09Rik	66968	17-:56251024-56256970
16825	MMT00056634		1+:159526434-159526829
16827	Arhgdib	11857	6-:136872170-136890259
16829	Igh-6	16019	12-:114923646-114924126
16830	MMT00014034		3-:59406405-59422757
16832	Trim63	433766	4+:133871104-133879151
16835	MMT00062293		10-:96231541-96232476
16838	Akap13	75547	7+:82600529-82895723
16842	2310003L22Rik	69487	2+:139996765-140029368
16843	Cry1	12952	10-:84595800-84647534
16844	Ugt2b1	71773	5-:87345661-87355554
16849	Trmt1	212528	8+:87213176-87224127
16851	Slc34a2	20531	5+:53440595-53462900
16852	Vash1	238328	12+:88029768-88036634
16854	Kif15	209737	9+:122860199-122927852
16855	Itk	16428	11-:46138654-46202995
16856	Hif1a	15251	12+:75009016-75047878
16857	Perp	64058	10+:18564876-18576876

16858	Tmem38a	74166	8+:75096001-75111181
16859	0610007L01Rik	71667	5+:130695710-130719559
16860	Cdc42bpa	226751	1+:181890557-182092722
16861	Nlrc4	268973	17-:74825652-74847558
16863	2410001C21Rik	66404	2+:172266058-172295401
16864	Tfb2m	15278	1-:181458186-181476348
16865	Olfr273	258821	4-:52868429-52869383
16866	Scara3	219151	14-:66538235-66557148
16871	MMT00072624		4+:23412430-23412994
16874	Fcer1g	14127	1-:173159708-173164417
16878	Zbed4	223773	15+:88610370-88614980
16879	Ltb4r1	16995	14+:56384867-56387326
16880	Depdc1b	218581	13+:109106560-109179775
16881	MMT00031504		14+:114879627-114879658
16882	Ogfrl1	70155	1-:23373275-23389912
16884	Lrat	79235	3-:82699325-82707892
16886	Dpy30	66310	17-:74698813-74715789
16888	H6pd	100198	4-:149353561-149377330
16890	H2-Eb2	381091	17+:34462624-34472830
16891	Pex2	19302	3-:5560511-5576128
16894	Lin7c	22343	2+:109740309-109741086
16898	Invs	16348	4+:48292689-48444821
16904	Sphk2	56632	7-:52966095-52969035
16909	Cav3	12391	6+:112409684-112422864
16910	Ptprr	19279	10+:115455454-115711985
16912	Rplp2	67186	7+:148633553-148637248
16913	Pira3	18726	7-:3860216-3867087
16915	MMT00019508		3+:93924626-93924985
16916	Lrrc19	100061	4-:94304832-94316837
16919	Pea15a	18611	1-:174126858-174128546
16920	Acsl1	14081	8+:47578185-47619832
16927	Cyp3a57	622127	5+:146106148-146131974
16930	Hmgb1-rs17	628431	8+:34594926-34595502
16931	Actn4	60595	7-:29678274-29747299
16932	Sacm1l	83493	9+:123439004-123501716
16933	1700012H19Rik	76393	11-:112684278-112686439
16940	St3gal3	20441	4-:117605601-117807021
16941	EG434339	434339	8+:96929038-96930156
16942	MMT00041689		13+:61824802-61825540
16946	Ivns1abp	117198	1+:153191645-153211388
16947	Wfs1	22393	5-:37357345-37380148
16949	Iigp1	60440	18+:60549448-60552281
16950	MMT00079465		7+:151977841-151978170
16951	R74862	97423	7-:150219494-150238995
16952	Pax1	18503	2+:147190795-147200782
16954	Sh3glb1	54673	3-:144351807-144383287

16955	MMT00068267		10-:68885784-68886498
16956	Slc24a5	317750	2+:124894507-124914510
16957	MMT00046108		2+:59234728-59245772
16958	Gpc1	14733	1+:94728262-94756772
16959	A230072C01Rik	320742	X+:20539239-20563849
16961	Mup1	17840	4-:60510886-60514832
16963	Tceal5	331532	X-:132735489-132738394
16965	Gstt2	14872	10-:75294630-75297031
16966	Col4a4	12829	1-:82449128-82583391
16968	Epha2	13836	4+:140857176-140885292
16972	Nenf	66208	1-:193130675-193141997
16973	MMT00027196		17+:53335694-53338047
16974	Eif2b4	13667	5-:31489940-31495458
16976	Zhx3	320799	2-:160604959-160663062
16983	Pigx	72084	16-:32084507-32087594
16988	Clec4f	51811	6-:83595017-83606026
16990	Sc4mol	66234	8-:67196941-67212375
16991	Gjb6	14623	14-:57742855-57743639
16992	Irf2bp2	270110	8-:129112197-129117090
16994	Trove2	20822	1-:145602725-145624108
16995	Dppa3	73708	6+:122576441-122580289
16996	MMT00059495		
16999	MMT00018943		16-:11185350-11186019
17000	2410089E03Rik	73692	15+:8136082-8137065
17001	Hmga1	15361	17+:27696477-27700608
17004	MMT00016962	634201	3+:85983800-85984031
17005	MMT00015015		X+:132874649-132875206
17006	Igh-VJ558	16061	12-:116597327-116861676
17007	Rundc3b	242819	5-:8494783-8622612
17008	Ntn4	57764	10+:93104198-93208598
17010	Galns	50917	8-:125102309-125135308
17016	Dnajc11	230935	4+:151307798-151356233
17020	Tox3	244579	8-:92771740-92871848
17022	MMT00055891		8-:67654962-67656225
17031	Wdr52	212517	16+:44432072-44481900
17036	Tctn3	67590	19-:40679587-40686690
17043	Tmem127	69470	2+:127074004-127086826
17044	Gpr55	227326	1-:87837450-87907557
17046	Fggy	75578	4+:95478798-95593636
17049	Capsl	75568	15+:9365782-9395790
17053	MMT00068622		4+:47466491-47468272
17054	Jph3	57340	8+:124254462-124314721
17058	V1rh16	171274	13-:23323983-23324910
17059	Upp2	76654	2+:58558178-58588063
17062	2900010J23Rik	72931	2-:32134328-32143586
17063	Hoxc8	15426	15+:102820969-102823797

17064	Cd48	12506	1+:173612224-173635007
17067	1700057G04Rik	78459	9+:92245763-92252714
17068	Olfr661	258743	7+:111836530-111837488
17071	Samd9l	209086	6-:3322521-3327259
17073	1810049H13Rik	66431	11-:120317917-120319377
17078	Syde2	214804	3+:145650962-145684308
17081	3830406C13Rik	218734	14+:13116756-13134318
17082	MMT00013155		17+:16984358-16985042
17083	Atp1b1	11931	1-:166368205-166387999
17086	Olfr780	258281	10+:128758680-128759618
17087	Gulo	268756	14-:66609215-66628059
17089	Pilrb1	170741	5-:138293374-138299226
17090	Lass2	76893	3+:95119173-95127488
17091	Slc38a3	76257	9-:107553486-107569700
17092	Ttn	22138	2-:76542035-76818252
17093	Opa1	74143	16+:29579427-29652576
17098	Qtrt1	60507	9+:21216346-21224631
17099	Ppp2r3c	59032	12-:56382494-56403976
17101	MMT00060041		
17106	Cep192	70799	18+:67988004-67989697
17109	C430049B03Rik	72575	X-:50407775-50410447
17112	Dok7	231134	5+:35429196-35430479
17114	Myh7	140781	14-:55589524-55613386
17115	2700099C18Rik	77022	17+:95151474-95174477
17116	Tceal6	66104	X-:131743227-131745226
17119	Gpr124	78560	8+:28196312-28233890
17122	MMT00068814		X-:96157424-96162857
17123	Olfr1511	258268	14-:53009489-53010446
17125	Nrxn1	18189	17-:90561268-90607840
17128	Vmn2r88	669149	14+:52030747-52039201
17130	Baiap2	108100	11+:119867419-119868093
17135	Mum1	68114	10+:79697018-79706647
17136	Slc35a4	67843	18+:36839245-36843511
17137	Nudt4	71207	10-:95011728-95026698
17143	2010002N04Rik	106878	18-:60633846-60661179
17144	B3gat2	280645	1+:23768590-23852279
17146	EG217600	217600	12-:58733564-58734002
17147	Ttll12	223723	15-:83406072-83425871
17149	Pms2	18861	5+:144670838-144692313
17150	MMT00013160		
17152	Scfd2	212986	5-:74602057-74632609
17157	L3mbtl4	320858	17+:69113890-69127710
17158	Zfp113	56314	5-:138584327-138596924
17159	Gtpbp6	107999	5-:110532991-110536454
17160	Ccdc127	67433	13+:74487790-74495122
17162	Cux2	13048	5-:122310316-122499860

17163	Fmo1	14261	1-:164759781-164796502
17164	Slc38a10	72055	11-:119989774-120012574
17169	4930543E12Rik	75239	7-:120243681-120285537
17171	MMT00045847		16-:32326267-32326781
17172	Insrr	23920	3+:87600983-87620023
17174	Dmrtc1a	70887	X-:100098560-100104026
17177	Accn2	11419	15+:99502214-99529694
17178	Ifnar2	15976	16+:91373143-91405610
17179	Stfa3	20863	16-:36450622-36455478
17181	Kbtbd10	228003	2+:69508181-69522295
17182	C230021P08Rik	380969	15-:99252465-99258598
17188	EG547263	547263	16+:30115265-30139780
17189	Gm263	238333	12+:88541408-88549768
17191	Xylb	102448	9+:119266709-119302447
17193	Npb	208990	11+:120469804-120470413
17194	Cmya5	76469	13-:93810671-93868385
17195	Magea3	17139	X-:151383085-151384048
17196	Fkrp	243853	7-:17394619-17402081
17197	Igtp	16145	11+:58013145-58021086
17201	Tsnaxip1	72236	8+:108351608-108368814
17202	Olfr791	258932	10+:128963284-128964223
17203	Svs3a	64335	2+:164115018-164117225
17207	2700050L05Rik	214764	7+:140843845-140863858
17212	Slc9a2	226999	1+:40738473-40826116
17213	MMT00057554		17-:22321732-22323096
17214	Surf6	20935	2-:26746292-26758316
17215	Mvd	192156	8-:124957503-124967290
17216	Nuf2	66977	1-:171428442-171461547
17219	Asnsd1	70396	1-:53380009-53399034
17221	Zfp264	116812	7+:6942005-6942686
17223	4933426M11Rik	217684	12+:81891226-81981791
17224	5430407P10Rik	227545	2-:6021090-6051192
17225	Impa2	114663	18+:67448876-67478495
17226	Utp14a	72554	X+:45610067-45635628
17233	Nmur1	14767	1-:88282900-88284716
17234	Stk33	117229	7-:116422722-116582594
17239	Rpl6	19988	5+:121655423-121658974
17243	Bcl6b	12029	11-:70037629-70043223
17244	Myef2	17876	2-:124912302-124949398
17247	A030007L17Rik	68252	6-:54935642-54942862
17248	Rap1gap	110351	4+:137237465-137285777
17252	Vmn2r94	665227	17-:18380534-18394077
17254	Cyp2f2	13107	7+:27904993-27918678
17257	Ube3a	22215	7+:66484581-66560302
17263	Ccnb3	209091	X-:6557377-6572729
17267	Agbl2	271813	2+:90623374-90653507

17269	Spsb1	74646	4-:149272075-149283840
17273	Psmc3	19182	2+:90894238-90899585
17280	Atf2	11909	2-:73656979-73730606
17282	Pdhb	68263	14-:8998850-9005446
17284	D1Bwg0212e	52846	1+:39592633-39603720
17285	1700019E19Rik	76411	12+:87423512-87503406
17287	9030411M15Rik	667742	18-:63269177-63317019
17291	Cct8	12469	16-:87484120-87496118
17292	Atp6v1b2	11966	8+:71612682-71637604
17295	MMT00082577		2+:137221009-137221079
17296	Mtm1	17772	X+:68468256-68568440
17297	Comtd1	69156	14-:22666277-22668156
17302	Dhcr24	74754	4+:106233754-106259391
17305	Itm2a	16431	X-:104592534-104598699
17312	Sirpa	19261	2+:129419057-129456668
17314	Napa	108124	7+:16683991-16703324
17316	Nkx2-1	21869	12-:57634022-57636047
17317	Jmjd1a	104263	6-:71538962-71582732
17321	Hepacam	72927	9+:37175187-37192680
17322	Zfp758	224598	17+:22498488-22514247
17323	Tubb6	67951	18+:67550372-67562402
17324	MMT00023460		1-:49060930-49061049
17325	4933400A11Rik	66747	X-:166214309-166217567
17327	MMT00004644	626904	6-:25926812-25927081
17328	H2afv	77605	11-:6328105-6344429
17329	MMT00035807		6-:140005316-140006121
17332	Smc3	13006	19+:53702562-53719636
17333	Slc25a10	27376	11+:120353185-120360479
17337	1810065E05Rik	69864	11+:58234612-58239525
17338	Mocos	68591	18+:24811986-24860074
17339	Acaa1b	235674	9-:119057161-119066124
17342	Dhx32	101437	7-:140912728-140968486
17343	Timm8a1	30058	X-:131071797-131076168
17344	Lonrf2	381338	1-:38853305-38868671
17348	Pf4	56744	5+:91201538-91202409
17351	Rtkn2	170799	10+:67504148-67505262
17352	Cebpb	12608	2+:167514414-167515920
17355	Ramp2	54409	11+:101107744-101109559
17356	Rgnef	110596	13-:98669423-98976122
17363	Aebp2	11569	6+:140572746-140626992
17364	Mef2c	17260	13+:83643193-83802948
17365	Isl2	104360	9+:55389013-55393986
17366	MMT00069569		7-:147073369-147074261
17368	1700123M08Rik	76665	4+:11893297-11921427
17370	Cdh12	215654	15+:21409980-21516236
17371	Emx2	13797	19+:59533890-59538927

17373	EG433016	433016	16+:36184292-36188194
17376	MMT00032526		15+:71624995-71626176
17381	Star	20845	8+:26918945-26925195
17383	Pah	18478	10+:86984718-87046876
17385	Asah3	171168	17-:57094502-57121531
17386	Vps16	80743	2+:130266049-130269468
17389	Pop4	66161	7-:39047838-39056367
17391	C530028O21Rik	319352	6+:124946835-124953116
17393	MMT00063804		10-:35428509-35431626
17396	Myoz3	170947	18-:60735943-60751442
17397	MMT00047810		2+:136091275-136225438
17398	MMT00045090		5-:117082489-117083618
17399	4933416C03Rik	619332	10-:115549544-115550675
17400	MMT00050193		1+:119502965-119523538
17401	Ptgfr	19220	3-:151463748-151500411
17403	N4bp1	80750	8-:89368220-89385212
17404	Ctsb	13030	14+:63741301-63764758
17406	4933428G09Rik	66768	5+:48763582-48779586
17408	Iqwd1	74106	1-:167259634-167390422
17412	Aif1	11629	17-:35307939-35309537
17413	MMT00043948		
17416	Col28a1	213945	6-:7949606-7965044
17417	Pxn	19303	5+:115956760-116004666
17418	D1Ertd622e	52392	1-:99540485-99558542
17419	EG432555	432555	11-:48707836-48715515
17420	Vps13b	666173	15+:35301294-35382022
17421	MMT00002048		2-:73797668-73798769
17422	Dctd	320685	8+:49195522-49227013
17423	Gna13	14674	11+:109224159-109258960
17429	Tpi1	21991	6-:124760733-124764430
17431	Foxc2	14234	8+:123640482-123642790
17436	Taf8	63856	17-:47625234-47639236
17440	Tex18	83559	10+:86376243-86377425
17444	Tuba1a	22142	15-:98762061-98781118
17447	Ctsf	56464	19+:4855128-4860912
17449	Zfp296	63872	7+:20163164-20166004
17453	Foxp1	108655	6-:98880084-99113012
17456	Lgtn	16865	1+:133049768-133070072
17458	Msr1	20288	8-:40690543-40718181
17461	Ripk5	213452	1+:134313989-134363536
17463	Rgl1	19731	1-:154365952-154472285
17468	Anubl1	67492	6+:116216546-116280316
17472	Cd53	12508	3-:106562851-106592970
17481	Defb21	403172	2+:152398478-152400679
17485	Ndufab1	70316	7-:129230349-129245360
17486	Cnn2	12798	10+:79451378-79458135

17492	MMT00078159		14+:54210578-54260976
17493	Rfx4	71137	10+:84218807-84369279
17497	Ptges	64292	2-:30747782-30758782
17498	MMT00008723		13+:66699587-66699622
17503	Cnnm2	94219	19+:46836786-46953069
17511	MMT00000525		4+:146318109-146319184
17512	EG433216	433216	19+:6152665-6153711
17513	Rin1	225870	19+:5050807-5057070
17514	Thbd	21824	2-:148230206-148233839
17515	Itln1	16429	1-:173460625-173461346
17520	EG668457	668457	3+:95426160-95426733
17523	Gdf5	14563	2-:155766762-155771103
17524	MMT00006341		1+:150823157-150826053
17525	Ctgf	14219	10+:24315312-24318486
17527	2310033K02Rik	69553	2-:167805663-167819135
17528	Gmnn	57441	13-:24843714-24853724
17529	Tnfrsf13c	72049	15-:82037384-82043239
17530	MMT00041792		8-:38463277-38464078
17533	Srd5a1	78925	13-:69712325-69751872
17535	MMT00008110		18-:88826941-88828108
17536	Chn2	69993	6+:54144018-54251801
17537	Piga	18700	X+:160857749-160861155
17540	Ywhaz	22631	15-:36701459-36723274
17542	MMT00044805	240219	18-:38519096-38520141
17543	Olfr1023	258580	2+:85726958-85727892
17547	C730007P19Rik	20865	7+:14318888-14364987
17551	Satl1	73809	X-:109497914-109520365
17553	Eif4ebp1	13685	8+:28370830-28385968
17558	Pole3	59001	4-:62184966-62186048
17559	Olfr920	258783	9+:38563274-38564205
17562	Rbed1	232089	6-:72515918-72544805
17563	Aldh1l1	107747	6+:90507191-90521989
17566	Olfr645	258247	7-:111232645-111233465
17568	Rexo2	104444	9-:48276618-48288716
17569	1810058I24Rik	67705	6+:35202686-35211982
17572	Krt80	74127	15-:101178753-101195009
17574	Prdm16	70673	4-:153694400-153741421
17575	C330001K17Rik	77609	9-:21799256-21807078
17577	Med28	66999	5+:45911539-45916838
17578	Kif19a	286942	11+:114649223-114652050
17579	MMT00078068		19+:44925782-44926468
17581	Pisd	320951	5-:33078969-33128276
17583	Per2	18627	1-:93312560-93355873
17584	F11r	16456	1+:173367701-173394724
17585	Gramd1a	52857	7-:31915145-31936052
17589	Madd	228355	2-:91017805-91023994

17590	MMT00047848		2-:140781633-140781973
17591	D930015E06Rik	229473	3-:83701577-83742090
17593	Gfer	11692	17-:24831131-24832881
17594	MMT00040814		5+:87426114-87427458
17595	MMT00048325		3+:132505358-132507882
17596	MMT00032256		
17600	Kcnq5	226922	1-:21391688-21503216
17603	4921509O07Rik	70917	13-:114236495-114274865
17606	Tmem90a	627191	12-:86019666-86039563
17609	Ptpn9	56294	9+:56843305-56910609
17612	Vhlh	22346	6+:113574005-113580264
17613	P2rx7	18439	5+:123094082-123131313
17614	MMT00071219		8-:74555571-74556774
17615	Exph5	320051	9+:53109837-53185607
17616	Flt1	14254	5-:148373771-148537336
17617	Atp5sl	66349	7+:26404452-26410569
17618	Zfp458	238690	13-:67357052-67585153
17619	Zfp551	619331	7-:13000499-13191900
17620	Macf1	11426	4-:123026843-123231609
17621	D930001I22Rik	228859	2-:163292042-163298441
17622	Foxo1	56458	3+:52072339-52151010
17623	Olfr715	258776	7-:114271958-114272905
17626	Ryr1	20190	7-:29809011-29910146
17628	Lrrc16a	68732	13-:24270073-24372642
17629	Saa2	20209	7+:54007202-54009682
17631	Pla2r1	18779	2-:60257095-60391318
17632	Lypd3	72434	7+:25421588-25426135
17635	Mmp10	17384	9+:7502360-7510238
17638	Adssl1	11565	12+:113858299-113879550
17641	Mapk7	23939	11-:61302314-61307705
17643	6030426L16Rik	628850	12-:21678265-21840135
17644	Dse	212898	10-:33871196-33927092
17645	Atp5c1	11949	2-:9981126-9981889
17646	C4b	12268	17-:34865331-34880828
17648	Scrg1	20284	8+:59934719-59956382
17650	Scgb1c1	338417	7+:148031470-148032667
17651	Commd1	17846	11-:22799763-22878996
17652	Cd36	12491	5-:17288303-17341535
17653	MMT00039948		13-:66650716-66651678
17656	Hdhd3	72748	4-:62160087-62163234
17659	MMT00017514		
17660	Nlrp12	378425	7-:3221915-3249524
17661	Birc5	11799	11+:117710564-117717046
17664	Jup	16480	11-:100231264-100259322
17666	Fstl1	14314	16+:37777159-37835832
17667	Phactr1	218194	13+:43230752-43233948

17668	6430527G18Rik	238330	12-:88221650-88225761
17669	2310007A19Rik	66353	3-:94268904-94277513
17672	EG237412	237412	10+:81816993-81842187
17673	Nola1	68147	3-:129527837-129534074
17676	Amz1	231842	5+:141217237-141229240
17679	Col3a1	12825	1+:45368474-45413525
17680	1700069L16Rik	73476	5-:114150351-114174783
17682	Zfp386	56220	12+:117285986-117298232
17683	MMT00010505	654450	6-:130002975-130013208
17687	Cdv3	321022	9-:103255432-103268001
17689	4933405O20Rik	243996	7+:57698992-57855779
17690	Mtap7d3	320923	X-:54051158-54054369
17691	Kank3	80880	17+:33947467-33959859
17692	Smc6	67241	12+:11306211-11324901
17693	MMT00044427		X-:115253343-115253599
17694	Ppp1r14b	18938	19+:7049475-7051812
17695	Rec8	56739	14+:56237072-56244226
17696	MMT00065923		X-:88791878-88792718
17697	1300014I06Rik	66895	13-:34719709-34744550
17701	Spink6	433180	18+:44231047-44243264
17702	Ccdc40	207607	11+:119099177-119125828
17703	Large	16795	8-:75338497-75876455
17707	Rshl2b	100037282	17-:7109064-7152705
17708	Reg2	19693	6+:78355148-78358091
17712	Nup62	18226	7+:52071789-52086174
17715	Ltbp2	16997	12-:86124162-86217482
17716	Rnf10	50849	5-:115691779-115722903
17717	Klhl15	236904	X+:91480675-91518865
17720	Rap2b	74012	3+:61168522-61170953
17721	MMT00077153		3-:141846870-141847784
17725	Trim12	76681	7-:111452110-111455902
17727	MMT00039356		
17729	Prim2	19076	1-:33510655-33726603
17730	MMT00058199		10+:20345591-20348727
17731	MMT00005424		11+:33072883-33073368
17735	Supt5h	20924	7-:29099916-29123738
17743	Phb2	12034	6+:124662353-124666936
17746	Prlr	19116	15+:10153740-10276776
17747	MMT00071421		7-:144359766-144359864
17748	Olfr878	258794	9+:37726228-37727172
17750	Hs6st2	50786	X-:49033065-49034779
17751	Olfr1201	258897	2+:88634540-88635463
17752	Chac1	69065	2+:119177025-119180058
17754	6720489N17Rik	211378	13-:62705589-62723192
17756	Morc1	17450	16+:48431349-48631018
17757	Hnrnph2	56258	X+:131139443-131141593

17759	EG433862	433862	5+:28186988-28187678
17760	Ccr6	12458	17+:8437944-8449985
17775	MMT00025230		1-:53453188-53622529
17776	BC043118	224273	16-:59491914-59556610
17777	Mtap7	17761	10+:19868819-20000511
17780	Slc38a5	209837	X+:7848556-7857299
17781	EG625796	625796	15+:31206235-31207592
17783	D630012G11Rik	320904	12-:74303610-74304276
17787	MMT00069688		8-:108109141-108110131
17788	Xrcc1	22594	7+:25332201-25358453
17789	Cnga4	233649	7+:112553402-112557251
17790	Olfr870	57251	9-:19975078-19976013
17792	Vmn2r79	621430	7+:94150477-94186337
17793	MMT00000967		6+:56896903-56897202
17794	2010203O07Rik	72429	4+:59030533-59036271
17800	Lrtm1	319476	14+:29831386-29841486
17801	Syt12	171180	19-:4447688-4467940
17802	Gpd2	14571	2+:57090856-57220006
17803	1810049H19Rik	435889	6-:41261231-41264710
17804	Npffr2	104443	5+:89996840-90012489
17805	Slc39a12	277468	2+:14317698-14416581
17806	Gpsm3	106512	17+:34727160-34728698
17807	Pebp1	23980	5-:117732662-117737555
17808	Ncor2	20602	5-:125497532-125659423
17810	MMT00054025		13+:75940136-75940731
17811	D10Ertd761e	52706	10+:81685763-81686514
17814	Napb	17957	2-:148520513-148521861
17816	3110003A17Rik	73112	10-:17731065-17743041
17818	Chchd4	72170	6-:91414270-91423417
17819	C030006K11Rik	223665	15-:76551895-76554249
17820	Cpd	12874	11-:76595490-76660498
17822	MMT00043095		13-:19396516-19429512
17823	D2Ertd750e	51944	2+:118639738-118660375
17826	3300001G02Rik	78372	11+:32105414-32108984
17827	Klhl13	67455	X-:22796396-22942179
17828	Serpina3n	20716	12+:105644968-105652535
17831	Ndufa6	67130	15-:82180592-82184667
17832	MMT00043412		1-:93068006-93068484
17834	Hemgn	93966	4-:46406861-46417055
17837	Polr3gl	69870	3-:96381796-96398066
17838	Apoc3	11814	9-:46041306-46042796
17840	1110029L17Rik	68722	15-:72926496-72926919
17842	Pcdh8	18530	14-:80166598-80171040
17847	Tmem16g	404545	1+:95286871-95299337
17850	Prg4	96875	1-:152296998-152313295
17852	Cpeb2	231207	5+:43635824-43677428

17854	Rdh11	17252	12-:80275323-80293280
17857	C1qtnf7	109323	5+:44000239-44007888
17862	Fbxo21	231670	5+:118426741-118458695
17863	MMT00025642	100038927	8+:22274359-22274508
17865	Txlnb	378431	10+:17516085-17565469
17872	Akap10	56697	11-:61686344-61728927
17874	Atp6v1h	108664	1+:5073252-5152526
17875	Cntnap5a	636808	1+:117956698-118189112
17876	Tubb4	22153	17-:57220114-57226936
17878	Brap	72399	5+:122113363-122135606
17879	MMT00069486		14+:70702013-70702703
17880	Ncald	52589	15-:37296018-37389098
17882	Ddx26b	236790	X+:53708022-53761019
17883	D730040F13Rik	242474	4-:56891192-56960298
17884	Lnx2	140887	5-:147828679-147888105
17887	Cryl1	68631	14-:57893871-58017320
17888	MMT00075624	70772	17+:27166212-27173320
17890	2610021K21Rik	78767	12-:101092802-101122606
17891	Olfr619	259080	7+:110752169-110753131
17892	MMT00037578		19-:15746724-15747278
17895	Smcp	17235	3-:92387805-92392919
17897	Gm1019	368202	3-:85798002-85804466
17899	BC016423	105203	13-:3584209-3589209
17900	Ift81	12589	5-:123000510-123064527
17901	Krtap16-5	77918	16-:88877767-88878235
17903	MMT00073283	103172	10+:75398318-75400491
17904	Cds1	74596	5+:102194391-102252878
17909	Mesdc1	80889	7-:91029558-91032829
17910	Ints2	70422	11-:86025607-86071061
17911	MMT00047239		6+:83981563-83982264
17912	Mpo	17523	11+:87607085-87617895
17915	V1rh3	171246	13+:22486897-22487836
17920	Nlgn2	216856	11-:69636624-69694564
17922	Reg3a	19694	6+:78330702-78333833
17925	F13b	14060	1+:141398303-141420333
17926	Nptx1	18164	11-:119400685-119409139
17927	MMT00042294	434130	7+:17579726-17581992
17928	Pex11a	18631	7-:86882149-86887911
17929	Atg4d	235040	9+:21076491-21078479
17930	Cidec	14311	6-:113374624-113385754
17933	Npsr1	319239	9+:24094240-24118633
17934	MMT00038301		18+:12447105-12447977
17935	Pde6c	110855	19+:38207435-38258227
17937	MMT00069919		5+:139051966-139052547
17938	Kcnd2	16508	6+:21664128-21679813
17940	Zfp706	68036	15-:36930383-36936956

17941	Ror2	26564	13-:53205589-53381246
17944	Zfp558	72230	9-:18260018-18282710
17946	Tmprss11a	194597	5-:86840646-86860936
17947	MMT00008227		18-:88583938-88584464
17951	4833436C18Rik	329831	4-:43439883-43441361
17952	Ripk2	192656	4-:16050702-16090543
17955	MMT00038380		11-:9637155-9637430
17956	Serpina1d	20703	12-:105001808-105011784
17957	1700086L19Rik	74284	12+:75386502-75397160
17960	Yod1	226418	1+:132612799-132616173
17964	Asb11	68854	X+:160876032-160897032
17969	BC030336	233812	7+:127821304-127877771
17975	MMT00079364		5+:8179591-8181202
17981	MMT00036029	268527	12-:16678430-16764047
17987	Fgfr2	14183	7-:137307301-137409705
17991	Fbxl20	72194	11-:97950015-98011706
17992	Gmpr	66355	13+:45602837-45641750
17994	Zh2c2	252876	1+:99666776-99689546
17995	Hist1h4d	319156	13+:23673470-23673780
17996	Igfbp2	16008	1+:72871153-72898734
17999	A730017L22Rik	613258	2+:130706338-130707869
18001	Nrl	18185	14-:56137814-56143028
18003	Tff3	21786	17-:31262259-31266556
18006	Wdr16	71860	11-:67738313-67779144
18008	1700011H14Rik	67082	14-:49846033-49865070
18010	Tex28	385380	X-:71397630-71406955
18018	Galnt11	231050	5+:24728720-24771735
18019	MMT00049952		19+:3138928-3143133
18020	D19Ertd737e	76539	19-:60274070-60302610
18022	Adamts17	233332	7+:74060735-74114441
18027	5730403B10Rik	66626	16-:4766827-4789916
18033	Klhl17	231003	4-:155603439-155608890
18034	3110006E14Rik	76980	13-:69841710-69878948
18036	Tmem100	67888	11+:89891661-89897819
18037	Klf9	16601	19+:23216244-23240447
18038	Sec24c	218811	14+:21493558-21514075
18042	Aph1c	68318	9-:66666729-66682478
18045	Klk10	69540	7+:51036448-51040779
18046	Ublcp1	79560	11-:44269738-44284002
18049	Ribc1	66611	X-:148439126-148450838
18050	MMT00003408	330962	9-:65260522-65270741
18053	Psmc4	23996	7-:28826735-28835062
18059	Centb2	78618	16-:31092500-31201324
18061	MMT00002521		6+:18094868-18095708
18062	Otog	18419	7+:53496356-53566803
18063	Rnf146	68031	10-:29065981-29081846

18064	MMT00035925		15-:77238201-77238877
18065	Peo1	226153	19+:45081046-45087251
18069	Gpt	76282	15+:76526956-76530115
18070	Ogg1	18294	6+:113277004-113284178
18073	Hipk3	15259	2-:104269921-104334401
18078	MMT00024172		15+:38892881-38893698
18083	Gadd45a	13197	6-:66985089-66987401
18086	Olfr1022	258582	2+:85708750-85709696
18088	Alkbh6	233065	7+:31093719-31099325
18090	D10Bwg1379e	215821	10-:18308996-18384715
18096	Slc39a6	106957	18-:24738382-24760073
18099	Nfx1	74164	4+:40918067-40971905
18100	Sdsl	257635	5-:120908211-120922819
18103	Spaca1	67652	4-:34115288-34137040
18105	Vmn2r121	100038941	X-:121240948-121249519
18107	Nek5	330721	8-:23204677-23207327
18108	Il31	76399	5-:123930167-123932596
18109	V1rc21	171194	6+:56906506-56907399
18112	Ikzf5	67143	7-:138535151-138540367
18113	MMT00034400	100047043	2+:80677101-80678935
18114	Lmtk2	231876	5+:144861459-144949068
18116	Ahctf1	226747	1-:181719263-181723954
18118	Cacybp	12301	1-:162133047-162143001
18120	Tmem201	230917	4-:149089490-149102165
18122	Snx21	101113	2+:164611514-164618275
18124	Ptprt	19281	2-:161353359-162486883
18126	Dyrk2	69181	10-:118296607-118316068
18127	Mxra7	67622	11-:116664697-116689361
18128	Tpi1	21991	6-:124761898-124764176
18130	Klrd1	16643	6+:129543505-129548788
18132	Rbms3	207181	9-:116868580-117019248
18135	Tmed3	66111	9-:89594042-89599829
18136	Tmprss11a	194597	5-:86873131-86874909
18138	MMT00000385		8-:112274892-112276164
18140	Atg2b	76559	12-:106854348-106861352
18143	Dnajc1	13418	2-:18138769-18314134
18144	Dmap1	66233	4-:117347340-117354542
18147	Nhlrc2	66866	19+:56623014-56673335
18148	Cables1	63955	18+:11997782-12104072
18151	Naip2	17948	13-:100914017-100967079
18153	Serpinb5	20724	1+:108757779-108778684
18159	MMT00082708		7+:133319530-133320085
18160	Bace2	56175	16+:97578383-97658706
18162	BC049352	408059	9+:45004017-45056986
18163	Raly	19383	2+:154616892-154692977
18164	Gdf10	14560	14+:34736957-34748463

18165	5830482F20Rik	320435	7+:29573987-29583979
18167	MMT00012252		6-:130433206-130889385
18168	Ptchd1	211612	X-:152008084-152057785
18169	Cbll1	104836	12-:32171765-32184405
18171	MMT00007396		14-:95684454-95738711
18172	Nupr1	56312	7-:133766759-133768984
18173	Arpp19	59046	9+:74885723-74904611
18174	Stat2	20847	10+:127707631-127729905
18176	Aqp11	66333	7-:104874888-104886758
18177	Cyp7b1	13123	3-:17972180-18143283
18178	Pitx1	18740	13-:55926416-55932786
18179	Dbp	13170	7+:52960601-52965521
18181	Epb4.1l2	13822	10+:25079684-25180699
18183	MMT00016159		9+:5397319-5398630
18184	Klrb1b	80782	6-:128764397-128776309
18185	Ndufb2	68198	6+:39553034-39553295
18186	Parp14	547253	16-:35832965-35841606
18187	Prrc1	73137	18+:57514392-57549599
18190	Rcn2	26611	9+:55889666-55906648
18195	Mafk	17135	5+:140267500-140278606
18196	Camk2b	12323	11-:5869646-5965566
18201	MMT00060232		13+:62914411-62916315
18203	Ablim3	319713	18-:61959049-62031357
18204	Coro1c	23790	5-:114294078-114358686
18205	Olfr114	258284	17-:37726357-37727296
18207	Dnali1	75563	4-:124733752-124743903
18208	Smoc2	64074	17+:14416541-14541771
18209	MMT00077977		7-:114070049-114070987
18211	Folr2	14276	7-:108988503-108992340
18212	Ttc3	22129	16+:94605518-94690828
18213	Tomm40	53333	7-:20286673-20300605
18214	Ankrd40	71452	11+:94189353-94196221
18215	Gpr146	80290	5+:139856649-139869777
18218	Sepx1	27361	17+:24873611-24879723
18220	Odz1	23963	X-:40765589-40783405
18221	Ctrb1	66473	8-:114210420-114214910
18235	Epsti1	108670	14+:78304070-78402457
18239	Golim4	73124	3-:75680112-75760779
18240	Scrt2	545474	2+:151917707-151919599
18243	Stk17b	98267	1-:53812949-53842014
18245	Mup4	17843	4-:59969678-59973537
18247	Wdr69	71227	1+:83156336-83207143
18248	Scn2a1	110876	2+:65508552-65604465
18249	Atp5c1	11949	2-:9984912-10002059
18250	Mmp14	17387	14+:55050530-55060059
18251	Susd4	96935	1+:184695026-184825787

18258	Gm73	218476	13+:97556551-97601826
18260	Nid2	18074	14+:20570517-20631009
18262	Gabbr1	54393	17+:37187798-37209372
18268	MMT00043750		3-:65310085-65319763
18269	Smc4	70099	3+:68808699-68835833
18271	MMT00007507		14-:124797026-124797911
18275	D730039F16Rik	77996	2+:34729987-34747651
18276	Apoa2	11807	1+:173155219-173156501
18278	Aspm	12316	1+:141377545-141390509
18279	Ogfr	72075	2+:180324163-180330230
18280	Dars	226414	1-:130260284-130313891
18281	MMT00077107		5+:105204789-105205824
18282	Tbcc	72726	17+:47027638-47028663
18283	5730593F17Rik	215512	11+:95224990-95243184
18286	Slc41a3	71699	6+:90554978-90596403
18289	Tap1	21354	17+:34324824-34333744
18295	Thsd4	207596	9-:60223754-60242353
18296	Pcgf5	76073	19+:36453556-36530694
18297	Psma7	26444	2-:179771080-179777107
18298	Slc25a37	67712	14-:69859907-69903113
18299	MMT00035424		8-:35370129-35370775
18300	Arg1	11846	10-:24635322-24647233
18305	Etv5	104156	16-:22383086-22411820
18308	Zfp672	319475	11-:58128568-58136859
18312	Apon	28194	10+:127691272-127692692
18314	4631416L12Rik	622434	3+:62142697-62266141
18317	Arl13b	68146	16-:62793518-62811877
18318	MMT00037377		6+:65382327-65383995
18321	Hydin	244653	8+:113029642-113049552
18324	4930546H06Rik	75202	17+:17967848-17976035
18325	Cst10	58214	2+:149230984-149236012
18327	Tmprss12	75002	15+:100111257-100123484
18330	Hoxc10	209448	15+:102797234-102801507
18332	Gpr19	14760	6-:134819177-134837739
18333	Rassf8	71323	6+:145699084-145766363
18336	Lrpap1	16976	5-:35434153-35448374
18338	Akr1e1	56043	13-:4591776-4608382
18342	Hamp	84506	7-:31727389-31729036
18346	Trib2	217410	12-:15800108-15822462
18347	2810451A06Rik	72778	15+:98929889-98935536
18350	Tc2n	74413	12-:102884774-102947748
18351	Nfyc	18046	4-:120430782-120463086
18352	Olfr1094	258362	2+:86668910-86669901
18359	Otop2	237987	11+:115190292-115193610
18365	Bcl2l12	75736	7-:52246594-52252949
18368	Mpped2	77015	2+:106533684-106707592

18369	Tmem136	235300	9-:42919287-42924633
18371	Sap130	269003	18+:31793931-31882715
18373	Dnajb1	81489	8+:86132091-86135801
18375	8430415E04Rik	74521	12+:104770773-104852042
18376	Pex11b	18632	3+:96439607-96448649
18384	Klf6	23849	13+:5860791-5867031
18388	Lnx1	16924	5-:74993204-75073817
18391	EG546347	546347	X+:89735342-89736015
18392	Kbtbd2	210973	6-:56727518-56730303
18394	Cxcr3	12766	X-:98926874-98929486
18397	Ccng1	12450	11-:40564282-40568731
18398	Fkbp15	338355	4-:61965226-62021577
18401	Crebbp	12914	16-:4083548-4213324
18403	Itpr1	16438	6+:108163111-108501095
18405	MMT00035749		14+:111178626-111179289
18409	Nicn1	66257	9+:108192782-108198021
18416	Enpp7	238011	11+:118849530-118853652
18417	Kbtbd7	211255	14+:79826536-79830843
18418	Hspa1b	15511	17-:35093377-35096532
18419	Srprb	20818	9-:103092517-103104409
18420	Igh	111507	12-:115821756-116153281
18425	Prickle2	243548	6-:92325941-92408652
18427	Serpinb1a	66222	13-:32933994-32943054
18428	Sfrs2	20382	11-:116711220-116714396
18429	Hmha1	70719	10+:79479486-79494214
18432	4933430H15Rik	74485	3-:87541817-87552808
18438	Map3k7ip2	68652	10-:7625937-7644660
18439	Cd209f	69142	8-:4102793-4105764
18440	Upk3a	22270	15+:84847657-84852962
18442	Zfp764	233893	7-:134547208-134550258
18443	B230380D07Rik	235461	9-:70450837-70504981
18444	MMT00058658		15-:3232775-3251191
18448	Klhl11	217194	11-:100323926-100334099
18450	Kdr	16542	5-:76329300-76374400
18451	Csgalnact1	234356	8-:70881677-70985450
18453	RP23-67E6.3	668066	2-:177171374-177173929
18454	Tor1aip1	208263	1-:157853912-157883425
18455	Mtnr1a	17773	8+:46154563-46173858
18461	Klra5	16636	6-:129849005-129863242
18462	Gm1060	381738	5+:30607918-30668992
18471	Aph1b	208117	9-:66626650-66643239
18474	MMT00070175	671232	9+:122705181-122711247
18478	F10	14058	8+:13037837-13055900
18479	Olfr1368	258527	13-:21233980-21234924
18482	Nup88	19069	11-:70754833-70783463
18483	Mthfd1	108156	12+:77356394-77420789

18485	Wdr31	71354	4-:62114767-62131906
18486	Lrfn4	225875	19-:4611785-4614504
18487	Serpina5	268591	12+:105339358-105344342
18490	MMT00020332		10-:105629435-105629826
18492	Dhfr	13361	13+:93125158-93155635
18495	C2cd3	277939	7+:107592825-107618665
18496	Mbd4	17193	6-:115792445-115803739
18498	Slc5a6	330064	5-:31338404-31351267
18500	MMT00074605	384806	8+:41878610-41882651
18501	1700012G19Rik	67078	17+:24607355-24608510
18504	0610040J01Rik	76261	5+:64203733-64290858
18505	MMT00071192		16+:48073089-48074594
18506	Otub2	68149	12+:104626914-104644553
18507	Bfsp2	107993	9-:103327250-103382703
18509	MMT00038317		18+:53112103-53112887
18512	Sult3a1	57430	10+:33577526-33599322
18513	MMT00044275		10+:22511074-22512280
18514	4930412F15Rik	242408	4-:43672494-43673626
18515	Ddx28	71986	8-:108533521-108535333
18516	4932413F04Rik	319215	9+:103461717-103783600
18517	Klc2	16594	19-:5107747-5118244
18518	Cap1	12331	4-:122537303-122563107
18520	MMT00005421		7-:13736393-13736930
18525	Mpped1	223726	15+:83610509-83686877
18526	Dydc2	71200	14-:41862407-41882498
18531	Akr1d1	208665	6+:37480172-37518392
18533	Hmox1	15368	8+:77617520-77624484
18534	Ovol2	107586	2-:144130915-144156781
18537	Moxd1	59012	10+:23943387-24022450
18541	Rab15	104886	12-:77898949-77923457
18542	Ankra2	68558	13+:99033196-99043867
18548	1700012P22Rik	69364	4-:144008089-144028713
18549	Robo1	19876	16+:72904868-73046337
18552	Nr2c2ap	75692	8+:72654686-72657657
18553	Tekt4	71840	17+:25608531-25613538
18554	Lhx9	16876	1-:140724574-140744147
18555	Sdcbp2	228765	2+:151398367-151415733
18556	MMT00001956		6-:37636278-37637195
18557	Limch1	77569	5+:67249115-67448396
18558	Nol11	68979	11-:107027976-107050574
18559	Csf2ra	12982	19-:61300313-61304262
18560	Islr	26968	9-:58004082-58006312
18561	Ngly1	59007	14+:17081841-17144050
18562	Nup188	227699	2+:30152164-30199786
18572	Stx18	71116	5+:38430471-38528056
18574	Parc	78309	17-:46637553-46683264

18577	Btbd9	224671	17-:30357041-30551683
18580	Phactr4	100169	4-:131911844-131978325
18581	MMT00048298		12-:115434540-115492137
18582	Krtap6-2	16701	16-:89419570-89420284
18586	Tns1	21961	1-:73960484-73975087
18590	Fgb	110135	3-:82845783-82853733
18591	MMT00034813		1-:155466728-155467858
18592	Txnl4a	27366	18+:80403580-80420264
18593	Rgs7bp	52882	13-:105741382-105844984
18594	1200002N14Rik	71712	10-:87785548-87819820
18597	Ganc	76051	2+:120229936-120287432
18602	Wisp1	22402	15+:66722954-66754761
18606	A230074B11Rik	215413	1+:37117216-37120491
18607	Acat3	224530	17-:13117121-13133233
18608	Atn1	13498	6-:124692564-124699840
18609	Lrrc1	214345	9-:77278624-77392505
18611	C1qtnf3	81799	15+:10882110-10909454
18618	Tex14	83560	11+:87299787-87369324
18619	2810406K13Rik	72184	7+:106614474-106622527
18620	Slmap	83997	14-:27234376-27262120
18622	Pwp2	110816	10-:77633654-77639894
18624	Plagl1	22634	10+:12810714-12849691
18625	Cit	12704	5+:116295652-116458571
18627	MMT00059478		3+:60664692-60681509
18631	Gja1	14609	10+:56097152-56110221
18632	Asxl1	228790	2+:153217264-153228669
18633	Nbeal1	269198	1+:60310239-60317219
18635	Hps1	192236	19-:42829701-42853135
18642	Hdac11	232232	6+:91106808-91124676
18645	Xlr	22441	X-:51086958-51100861
18648	Rnft1	76892	11+:86298200-86310049
18649	Ccne1	12447	7-:38883004-38892349
18650	Hiatl1	66631	13-:65166453-65174937
18651	Tulp2	56734	7+:52773313-52777750
18653	MMT00072901		19+:8798625-8800932
18655	Angpt2	11601	8-:18691330-18741488
18658	Gstk1	76263	6+:42195943-42200440
18662	Batf3	381319	1+:192921726-192932825
18665	Hs6st1	50785	1+:36125253-36163290
18666	Hint3	66847	10-:30328165-30338266
18669	Fxyd5	18301	7-:31817756-31827148
18671	Stag2	20843	X+:39555056-39628566
18672	Oraov1	72284	7+:152101039-152107038
18673	Muc1	17829	3+:89032973-89037291
18679	Cpne8	66871	15-:90317913-90509806
18680	EG633285	633285	3-:82667826-82670558

18681	Hexb	15212	13-:97946362-97968225
18683	Ctsa	19025	2+:164658483-164665082
18684	Cttn	13043	7-:151622773-151656629
18686	Zfp143	20841	7+:117205269-117238741
18688	Leap2	259301	11-:53235554-53236684
18693	Klf4	16600	4-:55540140-55545338
18694	Pkn2	109333	3-:142454523-142545246
18696	MMT00042876		1+:180074024-180075515
18697	Afg3l1	114896	8+:126001820-126026077
18699	2700089E24Rik	381820	6+:132986504-133055808
18700	Trappc2l	59005	8+:125135545-125139488
18701	Il22ra2	237310	10+:19341833-19351589
18702	Mrc1	17533	2+:14151040-14253416
18703	Oog3	100012	4-:143747462-143750421
18706	Lect1	16840	14-:80037500-80061977
18709	Srp68	217337	11-:116106480-116124569
18711	Usp10	22224	8+:122434751-122481457
18713	3110037I16Rik	73172	4-:120593806-120597610
18714	Farsb	23874	1-:78414532-78485434
18715	MMT00032843		18+:9495618-9496481
18720	BC002059	213811	17+:17088502-17111119
18724	Ramp1	51801	1+:93076615-93120256
18726	D630004A14Rik	382137	9+:50576341-50580773
18727	9130221D24Rik	77669	3-:132779456-132897916
18728	MMT00044404		5+:11891549-11928596
18731	Fgf17	14171	14-:71036525-71041579
18732	MMT00041012		10-:78091450-78092970
18734	EG622175	622175	13-:74715338-74754725
18737	Exoc1	69940	5+:76958337-76997197
18738	Hadha	97212	5-:30445964-30474563
18740	MMT00052300		1+:46111487-46115946
18742	Rnf217	268291	10-:31223556-31328350
18743	Ruvbl1	56505	6+:88415458-88447559
18746	Rhov	228543	2-:119094938-119096957
18749	Il2rb	16185	15-:78310980-78325452
18750	Thap6	381650	5+:92391409-92399608
18751	Cpa6	329093	1-:10314807-10474144
18752	Fgf7	14178	2+:125861450-125915295
18754	Rbm8a	60365	3+:96433855-96436833
18756	Tspan14	52588	14-:41719733-41780096
18757	Vsnl1	26950	12-:11333017-11393782
18762	Herc5	67138	6+:57530984-57615108
18764	Olfr1426	258805	19-:12162344-12163280
18769	Zfp518	72672	19+:40916757-40990731
18772	BC048355	381101	17+:46633729-46636175
18773	MMT00014972		12-:89522856-89524816

18774	MMT00054062		2+:45665083-45666035
18775	Best2	212989	8-:87531103-87537490
18776	Crnkl1	66877	2-:145743217-145760434
18778	Prickle1	106042	15-:93329532-93349841
18781	MMT00014109		19+:23741437-23741952
18782	Anks1b	77531	10+:90292584-90404257
18783	1700019M22Rik	69423	12-:97284830-97285365
18784	Sdad1	231452	5-:92720038-92726249
18785	Angptl4	57875	17-:33911845-33918451
18789	MMT00071096		12-:50941709-50942809
18790	Car3	12350	3+:14863519-14871863
18797	1700019G24Rik	72249	6-:5913899-5927361
18798	Ndufa4l2	407790	10+:126952019-126954975
18802	Nit2	52633	16-:57156778-57167445
18804	Pllp	67801	8-:97199343-97220623
18806	Hist3h2ba	78303	11+:58762412-58763032
18827	Oprs1	18391	4-:41685369-41688186
18832	Mybph	53311	1+:136090033-136097808
18835	Dtwd1	69185	2+:125980276-125991012
18836	Vmn2r27	232367	6-:124141871-124180483
18839	Pcdha1	116731	18+:37089938-37347311
18841	C130038G02Rik	77521	5+:149114777-149125612
18842	Phlda1	21664	10+:110943341-110945704
18846	Ikbkg	16151	X+:71678143-71694190
18847	Armetl1	227526	2+:3430333-3441285
18848	Sema3b	20347	9-:107500685-107507733
18849	Macrod2	72899	2+:142217967-142218699
18850	MMT00069233		X-:103564204-103565634
18851	Apip	56369	2+:102913939-102932795
18852	MMT00001015		
18856	Hmgcl	15356	4+:135502359-135518528
18859	C230094A16Rik	237711	11-:29644136-29664233
18862	1300003B13Rik	74149	17+:22591344-22592804
18865	Ptges3	56351	10+:127496229-127514049
18866	MMT00038112	670717	5-:13121449-13121766
18868	Mpeg1	17476	19+:12535310-12539774
18869	Cxcl2	20310	5+:91332924-91334964
18871	AI314976	106821	17+:48548706-48556593
18873	AB041550	58229	6+:87680862-87684010
18874	Olfr777	258537	10-:128623667-128706035
18875	EG636756	636756	X-:37353960-37354374
18876	Klf16	118445	10-:80029879-80040039
18878	Pde4dip	83679	3-:97493749-97494486
18881	Cops6	26893	5+:138602543-138605520
18882	Tbc1d2	381605	4-:46617271-46662964
18884	Ell	13716	8+:73063600-73116723

18886	Slc18a1	110877	8-:71561611-71613089
18889	Shq1	72171	6-:100523070-100621144
18892	Cyp4f40	631304	17+:32799075-32813423
18893	Lace1	215951	10-:42032391-42198220
18894	MMT00030135		7-:58196157-58198524
18901	Hpx	15458	7-:112740122-112748636
18903	Crem	12916	18-:3266353-3327517
18905	Prmt2	15468	10-:75669973-75700520
18906	Zfand3	21769	17+:30272258-30347638
18908	Cldn10	58187	14+:119187129-119273746
18911	Selenbp2	20342	3+:94497494-94508328
18922	Syce1	74075	7-:147963128-147973779
18923	BC025816	234796	8+:122386204-122400888
18925	Kcne1	16509	16-:92348676-92359119
18926	MMT00070399		14+:44326413-44328553
18927	Prl3d3	215029	13+:27213563-27254459
18928	Ttc28	209683	5+:111529712-111716417
18931	MMT00014022		6+:46994898-46996404
18932	Cebpg	12611	7-:35835402-35842310
18934	MMT00015154		11-:120533745-120534553
18935	D230044M03Rik	76743	10-:14435432-14438023
18936	Zfp560	434377	9-:20151747-20206511
18937	Cass4	320664	2+:172219362-172253425
18938	MMT00074326	100047309	10-:95332035-95333441
18939	Matn2	17181	15+:34236439-34365993
18940	Olfr1086	258585	2-:86516557-86517488
18942	Hs3st3b1	54710	11-:63702535-63735734
18943	Prr11	270906	11-:86903666-86922195
18944	Cyp2j13	230459	4-:95711924-95744221
18947	Ctse	13034	1+:133558522-133572084
18948	Olfr1270	258987	2-:89989246-89990161
18955	Chgb	12653	2+:132607407-132620812
18963	1700041C02Rik	73332	4-:119065258-119073651
18966	Dgkg	110197	16-:22468535-22622365
18970	Tnfsf11	21943	14-:78677258-78707841
18971	Dok7	231134	5+:35409211-35422923
18972	Nat5	67877	2+:145729004-145742052
18974	Cse1l	110750	2+:166731595-166771763
18978	Dnahc2	327954	11-:69234312-69358058
18980	Il7	16196	3-:7573147-7613727
18983	Ifrd2	15983	9+:107490048-107495369
18984	Pias3	229615	3+:96500391-96509886
18987	Treh	58866	9+:44481320-44494387
18988	MMT00012332		11-:6736286-6737306
18989	Tff2	21785	17-:31278154-31281187
18990	Hal	15109	10+:92951828-92979227

18996	Ahctf1	226747	1-:181692705-181713175
18997	Qpct	70536	17+:79451502-79489715
19000	Sfmbt2	353282	2+:10301807-10512195
19002	Ociad1	68095	5+:73684093-73705179
19004	Pdk4	27273	6-:5433350-5446278
19010	Bhmt	12116	13-:94387430-94407456
19011	Rpl3l	66211	17+:24869629-24873088
19020	V1rh20	171277	13-:22943469-22944364
19022	Ccnb2	12442	9-:70255496-70269337
19023	Kynu	70789	2+:43410847-43538237
19025	Slc37a4	14385	9+:44206437-44211046
19026	Isg15	53606	4-:155573563-155574905
19027	MMT00058026		17-:22101212-22101549
19030	MMT00011246		14-:59498087-59499113
19031	Mms19	72199	19-:42018691-42055620
19032	Tymp	72962	15-:89202361-89207468
19034	Bag4	67384	8-:26877672-26895670
19036	Ppp2r1a	51792	17+:21082469-21102434
19037	Slc11a2	18174	15-:100218332-100253414
19038	MMT00013556		2-:50377811-50378556
19040	Cdk2ap1	13445	5-:124795446-124804565
19041	9830102E05Rik	277898	6-:137965905-137992210
19044	MMT00004517		6-:38495246-38499153
19045	MMT00027514		9-:59614519-59614995
19048	4933417A18Rik	66761	13+:35016277-35047744
19051	Podnl1	244550	8+:86650243-86656414
19052	Mkrn2	67027	6+:115551986-115568696
19056	Zfp7	223669	15+:76709688-76722822
19057	Cntnap2	66797	6+:46297080-46371463
19060	Adamts13	279028	2+:26831164-26862327
19063	Evl	14026	12+:109793166-109926634
19064	MMT00004867		1-:154796483-154797341
19066	MMT00012632		3-:130631968-130633683
19067	BC023882	231123	5-:34496549-34512073
19070	EG436059	436059	8+:107538350-107544310
19071	Actr10	56444	12+:72038893-72063269
19075	Lmnb2	16907	10-:80364108-80380906
19077	Has2	15117	15-:56497183-56526053
19078	EG668139	668139	7-:113648313-113740829
19080	Myc	17869	15+:61816940-61821800
19084	Mmab	77697	5-:114883022-114894034
19086	MMT00029151		
19087	Wapal	218914	14+:35486838-35559772
19089	Peci	23986	13-:35069617-35085985
19092	MMT00045362		11+:96806153-96814775
19094	Fscn1	14086	5+:143722051-143734862

19095	MMT00006438		6-:69009641-69061200
19097	Sema3d	108151	5+:12383551-12588943
19098	MMT00017494		17+:9605375-9615322
19099	Olfr196	258775	16-:59167037-59167967
19101	4921504E06Rik	70909	2-:19415325-19475541
19102	Uhrf1bp1l	75089	10+:89207779-89283216
19106	Nsbp1	50887	X-:106199873-106208698
19108	Armet	74840	9-:106791091-106794246
19110	Gm114	228730	2+:146715326-146795830
19112	Upb1	103149	10+:74869777-74902938
19115	Rab32	67844	10-:10265034-10278016
19116	Entpd3	215446	9+:120430925-120431940
19117	Olfr1318	258022	2+:111996283-111997070
19124	Pim3	223775	15+:88692623-88696155
19125	Muc15	269328	2+:110571805-110579680
19129	Serf2	378702	2+:121275152-121276793
19131	Gad2	14417	2+:22477837-22546510
19132	Adam7	11500	14-:69115395-69151732
19134	Fn3k	63828	11+:121296281-121311797
19135	Ankrd22	52024	19-:34197111-34240393
19136	Trim13	66597	14+:62218392-62224780
19139	Olfr112	258096	17-:37700292-37701254
19141	Gorasp1	74498	9-:119834375-119846677
19142	Gpatch1	67471	7-:36061562-36103459
19143	Mrpl40	18100	16-:18872310-18876730
19145	Acsbg1	94180	9-:54452803-54509692
19146	N4bp2l1	100637	5-:151374223-151379566
19148	Rnf144b	218215	13+:47289389-47341778
19151	Dmp1	13406	5+:104631635-104643129
19153	Ptgs1	19224	2+:36086046-36107782
19154	3110001K24Rik	75698	14-:35050222-35123702
19157	Rps19bp1	66538	15-:80091051-80094726
19159	Epn3	71889	11-:94351996-94361257
19160	MMT00042532		X+:85633638-85634431
19161	Cav2	12390	6+:17231357-17239004
19163	Cadps2	320405	6-:23212842-23394045
19164	Arhgap15	76117	2+:43604369-43746048
19169	Atp10a	11982	7+:65913702-66084790
19171	Tmem16d	320091	10-:88434016-88551039
19172	Prkg2	19092	5-:99361027-99466370
19173	Mb	17189	15-:76845918-76881098
19175	Spag6	50525	16-:16753109-16829387
19176	2210404J11Rik	381062	17+:15123436-15201197
19178	Bhlhb5	59058	3+:17954710-17957509
19182	Igsf2	630146	3-:100797454-100824655
19184	Skap1	78473	11+:96563877-96620737

19186	Pla2g4c	232889	7+:13910002-13941675
19187	Chrm3	12671	13-:9876474-9878244
19188	MMT00042824		13+:62107935-62108670
19189	Sp100	20684	1+:87546588-87598136
19190	MMT00077382		19-:3821822-3824584
19191	Ccng2	12452	5+:93696620-93705221
19192	Hist1h1c	50708	13+:23830675-23832235
19194	EG668450	668450	14+:70759826-70760251
19195	Reep6	70335	10+:79792914-79799185
19196	Nfe2l1	18023	11-:96678733-96689061
19199	Gla	11605	X-:131122894-131135494
19200	Hemk1	69536	9-:107230039-107240626
19201	2210018M11Rik	233545	7-:105736939-105805101
19202	1500031L02Rik	66994	16+:32099887-32108140
19204	Prr13	66151	15+:102289605-102293214
19205	Zfp329	67230	7-:13391445-13404201
19211	MMT00046586		12-:21584046-21586504
19214	Snapc1	75627	12+:75065547-75085377
19216	Bik	12124	15+:83357301-83375065
19217	Cyr61	16007	3-:145309941-145312945
19218	Mdh1	17449	11-:21457243-21471816
19219	MMT00015104		3+:138264462-138265091
19220	MMT00040501	54381	15+:33013002-33524259
19222	MMT00029634		6-:130888044-130889986
19223	Ercc8	71991	13+:108948931-108984705
19226	Nras	18176	3+:102862151-102870343
19227	MMT00058471		2-:166832485-166834547
19228	CU075549.1	620807	4+:60018738-60019967
19230	1700106N22Rik	73582	17+:85489942-85602361
19233	Ankrd23	78321	1-:36587038-36604064
19234	Nrg4	83961	9-:55074420-55130425
19235	4921510J17Rik	70894	11+:104953531-104978851
19236	MMT00045089		5+:117508677-117510005
19238	Ttc35	66736	15+:43308774-43359323
19242	Ran	19384	5+:129526051-129529038
19243	Olfr557	258358	7+:109846753-109847721
19244	Impdh2	23918	9+:108462864-108467849
19247	Prelp	116847	1-:135808988-135817979
19249	Olfr767	258315	10-:128516089-128517017
19250	Olfr483	258730	7+:115246824-115247770
19252	Tmsb4x	19241	X-:163645026-163647081
19259	Kng1	16644	16+:23058492-23081762
19262	Cd274	60533	19+:29446941-29462582
19265	EG546036	546036	8+:13986085-14003560
19267	Olfr341	258952	2-:36334708-36335648
19269	Lrrc48	74665	11+:60166881-60207835

19270	Krit1	79264	5+:3806466-3840941
19271	MMT00063670		5+:90794340-90799635
19273	MMT00073897		14+:43722358-43724478
19275	Farp2	227377	1+:95408701-95518358
19276	H28	15061	3-:151421702-151425855
19277	MMT00044927		17-:65411187-65415719
19278	1700031M16Rik	73302	15+:98228875-98246699
19282	Tbx3	21386	5+:120121701-120134727
19284	Fbxo8	50753	8+:59029923-59070855
19286	1110012M11Rik	73711	8+:74066850-74071923
19287	Rock1	19877	18-:10067468-10181315
19289	Pofut1	140484	2+:153067298-153091715
19291	MMT00032606		6-:114118922-114119518
19298	Unc93b1	54445	19+:3935193-3949337
19299	Ptprz1	19283	6+:22825721-22954668
19305	Azin1	54375	15-:38419879-38431276
19306	AI428936	233066	7+:31099839-31104066
19308	C330006A16Rik	109299	2-:25992332-25995776
19309	Slc9a8	77031	2+:167247208-167302499
19311	Masp1	17174	16-:23449489-23520676
19317	EG626952	626952	15-:78384613-78385361
19318	Plk1	18817	7+:129302951-129313353
19320	Cyp2j7	546837	4-:95866026-95903350
19322	Myo15b	217328	11+:115749673-115753902
19323	Bnip3	12176	7-:146082933-146101152
19326	Usp48	170707	4+:137212948-137214444
19332	Bpnt1	23827	1+:187156079-187180664
19333	Ccdc13	434446	9-:121736351-121743065
19334	Gpr44	14764	19+:11014610-11015758
19339	Gem	14579	4+:11631603-11641896
19340	Serpina3k	20714	12+:105578715-105582491
19342	Cth	107869	3-:157557387-157588013
19344	Hdgfrp3	29877	7-:89038684-89078908
19345	Ssh2	237860	11+:77234760-77270615
19347	1700054M17Rik	73358	2+:118130648-118133897
19349	Psg28	114871	7-:19008239-19017214
19350	Siglec5	233186	7+:50606774-50614839
19351	Prkcsh	19089	9+:21807478-21818665
19354	Clu	12759	14+:66587461-66600385
19356	Herc1	235439	9+:66198373-66221864
19358	Grip1	74053	10+:119256567-119512629
19359	Sycp1-ps1	20958	7+:19371648-19373610
19360	Aco1	11428	4+:40090432-40146043
19362	Gper	76854	5+:139899615-139903743
19372	Gnai2	14678	9-:107516918-107537631
19373	MMT00018326		16-:16196394-16198862

19374	Dclre1c	227525	2+:3341421-3371361
19375	Inhba	16323	13+:16106307-16119044
19382	Slc2a6	227659	2-:26876884-26883483
19385	Akr1c6	83702	13+:4433636-4456642
19389	Iqcb1	320299	16+:36829088-36872806
19391	Apex2	77622	X-:147006050-147022692
19392	Slc25a16	73132	10+:62383409-62409239
19393	Synpr	72003	14+:14117372-14447895
19394	Decr1	67460	4-:15844387-15872520
19399	Camta1	100072	4-:150433634-150448171
19401	Ms4a10	69826	19-:11036784-11049155
19402	Ddx19a	13680	8-:113498913-113521701
19406	Ikzf1	22778	11+:11590368-11669801
19407	Zhx1	22770	15-:57878860-57907776
19408	MMT00042388	260347	7-:103059527-103061913
19410	Glb1	12091	9+:114310236-114383495
19414	MMT00065577		2-:162807992-162809075
19415	B3gnt2	53625	11-:22735955-22759513
19419	MMT00002063		19+:8050307-8052539
19420	Lcn8	78076	2+:25508637-25511734
19421	Mbd2	17191	18+:70727841-70785783
19422	Fkbp1b	14226	12-:4839993-4848345
19423	Rlf	109263	4-:120818488-120887580
19424	Mcoln1	94178	8+:3500518-3515229
19426	Golga7	57437	8-:24351817-24367484
19429	Scx	20289	15+:76287866-76289550
19432	MMT00068429		
19433	Mex3b	108797	7+:90017065-90018718
19436	Dapp1	26377	3-:137594569-137644488
19440	Anxa9	71790	3-:95100016-95111054
19443	Prkab1	19079	5-:116463596-116474460
19444	EG623519	623519	16+:16968701-16969021
19447	MMT00004670		14+:24775161-24827036
19451	Armc7	276905	11+:115336999-115351783
19452	Gm97	225923	19+:11771551-11786346
19453	Cdk2ap2	52004	19+:4097350-4099017
19456	Olfr727	258316	14+:50746253-50747226
19459	Frk	14302	10+:34203325-34328382
19462	4933439C20Rik	66776	11+:3027555-3059995
19465	Hacl1	56794	14-:32421045-32454149
19466	Pptc7	320717	5+:122734371-122774286
19478	Rnaseh2b	67153	14+:62950940-62991830
19482	Efhc1	71877	1+:20941765-20980923
19483	Depdc2	109294	1+:11079742-11279401
19487	Adh5	11532	3+:138106106-138118264
19490	MMT00078676		7+:35036056-35036510

19493	Icosl	50723	10+:77532203-77542270
19496	Glyctk	235582	9-:106055291-106060449
19497	Dync2h1	110350	9-:7140888-7177020
19502	Fbxo24	71176	5-:138053733-138066308
19511	Pcdhb21	93892	18+:37673473-37676017
19516	Ppfia4	68507	1-:136193370-136229100
19519	BC011248	224823	17-:46804406-46811204
19520	Uck2	80914	1-:169156218-169215254
19522	Tcf21	21412	10-:22537084-22539934
19524	Rhag	19743	17+:40948097-40977703
19528	Zfp503	218820	14-:22803184-22807084
19532	Med8	80509	4+:118081984-118087714
19533	Gpr62	436090	9-:106366291-106368225
19535	MMT00041431		2+:16191092-16192193
19540	Zfp428	232969	7+:25292204-25300683
19541	Glyat	107146	19+:12708022-12726227
19542	Hspb7	29818	4+:140977642-140980114
19543	B3gnt3	72297	8-:74215796-74217798
19544	Cga	12640	4+:34841027-34854623
19545	Rps9	76846	7+:3655610-3658643
19547	MMT00022553		12+:14501275-14502094
19548	Mgst3	66447	1-:169302514-169323928
19550	Zfx	22764	X-:91320427-91368239
19551	Rab11fip2	74998	19-:59982599-60018903
19552	Nlgn1	192167	3-:25594122-26032688
19553	Chtf18	214901	17-:25855975-25864347
19554	6330549D23Rik	229613	3-:96411544-96412703
19555	Cthrc1	68588	15+:38908482-38918665
19556	Efna1	13636	3-:89076136-89083526
19559	Icmt	57295	4+:151671419-151681759
19560	Trmt6	66926	2-:132629941-132641791
19563	Adam18	13524	8-:25712718-25785225
19564	Bcam	57278	7-:20341487-20355825
19565	EG385454	385454	X-:149546947-149548891
19567	MMT00053547		5-:98504396-98507325
19570	Pomc	18976	12+:3958128-3960467
19573	Serpine1	18787	5-:137537377-137548129
19574	Gm1502	385120	6-:69217839-69218103
19577	Rpa3	68240	6-:8205938-8209113
19578	Aox1	11761	1+:58086847-58163254
19579	Sirt1	93759	10-:62781808-62801765
19583	Hcrt	15171	11-:100623006-100624245
19584	Nav2	78286	7+:56675543-56708328
19587	Avpr2	12000	X+:71137440-71139765
19589	Slc16a9	66859	10+:69708023-69748699
19590	MMT00026015		4+:82839845-82840950

19595	MMT00056884		
19597	MMT00001011		
19602	Il5ra	16192	6-:106660374-106699031
19606	Egr2	13654	10+:67000626-67004935
19607	Birc3	11796	9-:7848702-7873024
19610	Igh-VX24	195176	12-:114849374-114849828
19611	Cog8	97484	8-:109572608-109580557
19612	MMT00028216		13+:68995359-68996138
19613	EG546387	546387	X-:146657131-146657879
19617	MMT00013143		
19618	Snx6	72183	12-:55847343-55896649
19619	Zfp438	240186	18-:5210033-5334438
19620	Zfand2a	100494	5-:139947170-139960342
19621	Cldn5	12741	16+:18777067-18778352
19622	Bdh2	69772	3+:134944189-134967387
19623	Myl7	17898	11-:5796638-5798782
19624	Olfr378	259026	11-:73238539-73239483
19629	Smarcd3	66993	5-:24098442-24107766
19631	Nsmce1	67711	7-:132611167-132634957
19633	Gm694	277744	4-:140988587-140992031
19638	Samd3	268288	10+:25983587-25991977
19640	Tph2	216343	10-:114516768-114619955
19641	Rps14	20044	18+:60936056-60938151
19644	MMT00042763	65115	8+:106694787-106743021
19645	Il12rb2	16162	6-:67242011-67326131
19647	Cort	12854	4-:148499301-148500771
19651	Thex1	67276	8-:36530292-36558587
19655	3110040N11Rik	67290	7-:88926879-88934317
19658	Cldnd2	74276	7+:50696149-50698697
19659	MMT00005649		X+:98899617-98921323
19660	Gkap1	56278	13-:58334815-58357204
19661	Epb4.1l2	13822	10+:25187897-25243320
19663	Tnfrsf22	79202	7-:150824191-150835503
19665	MMT00034233		18-:59989185-60015084
19666	MMT00012505		3+:112394525-112395469
19668	Zfp509	75079	5-:38590835-38607860
19670	Fbxl14	101358	6+:119429803-119432631
19671	Upp2	76654	2+:58607351-58643648
19672	Cldn19	242653	4+:118928004-118932461
19674	MMT00030712	16098	6+:70468234-70468839
19675	Rer1	67830	4-:154448220-154460406
19678	Scn11a	24046	9-:119662884-119734574
19680	Gm1420	546213	6-:69598810-69701304
19685	MMT00009602		11-:117179232-117202029
19688	Fap	14089	2-:62339001-62412078
19689	1110032A13Rik	68731	18-:80388998-80397398

19690	Arf5	11844	6+:28373639-28376499
19694	Grb14	50915	2-:64750541-64860823
19696	Thoc4	21681	11-:120455829-120459679
19697	Nek1	18004	8+:63472015-63610140
19698	Sema3c	20348	5+:17080681-17233655
19699	Rnf180	71816	13-:105921527-106084900
19700	E030011K20Rik	208613	3-:27764987-27785416
19701	9930021J17Rik	229488	3-:85463981-85621216
19705	H2-T3	15043	17-:36323516-36327073
19708	Ly6a	110454	15-:74825416-74828330
19709	1-Sep	54204	7-:134357963-134361967
19713	BC048599	232717	6-:40845261-40847865
19716	Rpp30	54364	19+:36158226-36179106
19717	Ccdc27	381580	4-:153400474-153402237
19719	EG634650	634650	5-:105752643-105775478
19720	Cyp26c1	546726	19+:37760169-37767771
19722	Pvrl1	58235	9+:43612803-43614851
19723	Pfkfb1	18639	X+:147028925-147078423
19724	Lancl2	71835	6+:57652629-57688473
19725	Dcp2	70640	18+:44540156-44577605
19726	Erp29	67397	5-:121894764-121902388
19727	Ttr	22139	18+:20823913-20832818
19728	1110007L15Rik	67604	5+:139728328-139746004
19730	Mamdc2	71738	19-:23377098-23522931
19731	Olfr275	258857	4+:52838270-52839228
19732	MMT00024414	73980	1+:54738570-54793660
19734	Dchs1	233651	7-:112901503-112921713
19737	Enpp1	18605	10-:24361216-24431908
19738	Lgals3bp	19039	11-:118254065-118263245
19739	Cmpk1	66588	4-:114633244-114659751
19740	Gpr98	110789	13-:81234067-81581613
19742	Cdc2l1	12537	4+:154998991-155024041
19743	Atg16l1	77040	1+:89652672-89688997
19744	Acy3	71670	19+:3986572-3990003
19747	Spr	20751	6-:85083679-85087812
19748	Cpa2	232680	6+:30491656-30514477
19751	Kbtbd5	72330	9+:121686807-121692937
19757	Fkbp5	14229	17-:28536866-28578001
19758	Snx1	56440	9-:65935945-65972693
19762	Pih1d1	68845	7+:52409767-52415434
19763	Acp2	11432	2+:91042983-91052296
19766	Slc26a4	23985	12-:32204684-32244834
19767	Etv6	14011	6+:133985957-134216777
19771	Fbp1	14121	13-:62966113-62989596
19772	Gnmt	14711	17-:46862614-46866114
19773	EG668838	668838	7-:54076232-54076871

19775	Klhl31	244923	9+:77497810-77503818
19777	Lrit1	239037	14+:37868020-37878123
19780	MMT00052614		12-:79356420-79356718
19790	Polg2	50776	11-:106629619-106640756
19791	E2f3	13557	13-:30001287-30077289
19792	Hook1	77963	4+:95633987-95689115
19794	Rgs2	19735	1-:145848226-145851263
19797	Coq9	67914	8+:97362295-97378793
19798	Tnk2	51789	16+:32644946-32683578
19799	9530008L14Rik	109254	13-:41859501-41942986
19803	BC003993	80744	2-:77733824-77784223
19806	4732429D16Rik	217305	11-:114843762-114851200
19808	Fetub	59083	16+:22920373-22939824
19809	Cpvl	71287	6-:53823272-53928668
19811	Cel	12613	2-:28411339-28418882
19812	Armcx2	67416	X-:131338685-131343535
19814	4930458L03Rik	78124	13+:65018862-65138977
19822	MMT00004881		4-:105046460-105047615
19823	Gp5	14729	16-:30308238-30309940
19824	Cetn3	12626	13+:81922339-81935694
19826	Nanp	67311	2-:150855432-150865091
19827	MMT00003831		19-:50614298-50615375
19828	Apoa1	11806	9+:46036792-46038547
19830	Irf4	16364	13+:30841126-30858794
19831	Fgd6	13998	10+:93498743-93506365
19833	Ddah2	51793	17+:35196045-35199038
19834	Jarid1d	20592	Y+:234215-280253
19835	Iars2	381314	1-:187108096-187121466
19839	EG269105	269105	1+:15057153-15057543
19840	Kcnip3	56461	2-:127282239-127347076
19842	B230315N10Rik	237411	10-:81687603-81704015
19843	Rbm45	241490	2+:76208113-76221822
19844	Ngf	18049	3+:102317877-102324928
19850	MMT00036391		6+:140368645-140369175
19851	Gnb4	14696	3-:32483976-32501063
19854	Kcnj14	211480	7-:53071837-53080117
19857	Rab1b	76308	19-:5099206-5106996
19861	Lrrc7	242274	3-:157747339-158115753
19866	Uso1	56041	5+:92566965-92631832
19868	Was	22376	X-:7658591-7667578
19870	Pcdhb15	93886	18+:37633370-37635729
19871	MMT00065790		14-:15280277-15281554
19874	Prmt1	15469	7-:52232127-52241702
19876	6030443O07Rik	226151	19+:45005571-45058273
19885	AI427122	102502	9-:95653063-95745698
19890	Tmem119	231633	5-:114243738-114250361

19891	Azi1	12009	11-:119925749-119948132
19892	EG546164	546164	9-:115346777-115347723
19897	Chsy3	78923	18+:59568537-59570928
19900	Serinc5	218442	13+:93430972-93481907
19904	Ltb	16994	17+:35331466-35333247
19906	Tfpi	21788	2-:84280878-84314152
19907	Spata19	75469	9+:27204405-27209295
19908	Olfr676	259099	7+:112183713-112184665
19910	St6galnac1	20445	11-:116626339-116636821
19912	Inpp1	16329	1-:52846265-52874381
19913	1810030J14Rik	66289	1+:176260648-176263004
19914	Acaca	107476	11+:84008939-84159590
19917	Nek2	18005	1+:193645440-193656921
19919	Unc5b	107449	10-:60227343-60294329
19920	Dph2	67728	4-:117561248-117564636
19921	Stxbp5l	207227	16-:37115917-37385025
19923	Capn3	12335	2+:120289608-120330234
19924	Ubiad1	71707	4-:147808605-147818860
19925	Arsa	11883	15-:89302907-89307203
19929	MMT00079667		13-:82517908-82518445
19932	MMT00075591		3+:68649921-68651817
19933	Pi16	74116	17+:29456186-29464894
19934	MMT00079286		
19935	Zdhhc11	71164	13+:74101296-74130437
19936	Egf	13645	3-:129380495-129458234
19939	Serpinb9b	20706	13+:33119282-33132425
19940	Eomes	13813	9+:118387329-118395238
19941	1700080E11Rik	73532	9-:105045673-105047412
19946	Elf5	13711	2+:103264233-103291142
19947	Fcrla	98752	1-:172848025-172857671
19949	Tubgcp2	74237	7-:147181894-147222165
19951	Tmem63b	224807	17-:45797125-45823856
19952	Tlcd1	68385	11+:77992267-77994273
19954	Abcd3	19299	3-:121463149-121518036
19955	Cbx2	12416	11+:118884275-118890649
19958	Rab38	72433	7+:95578840-95639983
19960	MMT00015578		5+:10558352-10559434
19964	Ndufa9	66108	6-:126771972-126799143
19966	Thap4	67026	1-:95601968-95647652
19970	Mrpl36	94066	13+:73468456-73469625
19971	B930046C15Rik	544998	14+:3518337-3539523
19972	Copg2	54160	6-:30698411-30846697
19974	Pax6	18508	2+:105516098-105537498
19975	1200015N20Rik	71721	10+:70015790-70021477
19976	Cyp4a10	13117	4+:115192187-115193802
19978	Rspo1	192199	4+:124663673-124686343

19979	Inha	16322	1+:75503822-75506736
19982	ENSMUSG00000071(	100038711	17-:86897071-86904579
19985	Gstcd	67553	3-:132644739-132747968
19986	MMT00026797		16+:47075750-47076054
19987	Tspan13	66109	12-:36741143-36769003
19990	4921515J06Rik	66715	3+:108743029-108763690
19995	Pwp1	103136	10+:85334609-85351841
19997	Ikbke	56489	1-:133151177-133173153
19999	Ift122	81896	6+:115724628-115876712
20003	Nek10	238944	14+:15683273-15694202
20004	1190005F20Rik	98685	1+:153275792-153305294
20005	Dpy19l3	233115	7-:36514704-36539390
20012	Gm336	240539	19-:11165060-11166907
20017	MMT00006294		17+:36294836-36296988
20019	Qdpr	110391	5-:45825813-45841419
20022	Mei1	74369	15+:81912904-81957244
20024	Lrrc32	434215	7+:105642731-105650338
20035	Zbtb46	72147	2-:181145254-181194131
20036	Olfr1342	258708	4-:118362108-118363055
20037	Extl3	54616	14-:65673184-65697025
20038	MMT00008174		11-:79184763-79185440
20039	Trpc4	22066	3+:53960025-54122389
20045	D4Wsu114e	28010	4-:147234900-147242571
20047	Dysf	26903	6+:83958576-84161058
20049	2310044G17Rik	217732	12+:88288389-88306304
20053	Wnt10a	22409	1+:74838089-74850747
20055	Ghrh	14601	2-:157155236-157159528
20058	Dnaic1	68922	4+:41516825-41585578
20061	EG383436	383436	19+:12921355-12921695
20068	2610204M08Rik	70435	12+:113838204-113853770
20076	Dbnl	13169	11+:5688532-5700964
20077	Ccdc115	69668	1-:34493514-34501514
20078	MMT00023360		7-:10045692-10045742
20079	MMT00019254		14-:58054555-58055353
20083	ENSMUSG0000007З4	69717	17-:36278702-36279740
20085	Lpin2	64898	17+:71533317-71596419
20086	Clk2	12748	3+:88969004-88980517
20087	Cd44	12505	2-:102651301-102696126
20088	Rapgef1	107746	2+:29475306-29593850
20089	MMT00015975		
20090	Slc45a2	22293	15+:10930567-10958065
20092	Matr3	17184	18+:35731677-35732590
20093	Ablim1	226251	19-:57107760-57290289
20096	Olfr444	258650	6+:42878371-42906429
20098	MMT00046724		Y+:2086608-2097759
20099	MMT00019756		17+:55839839-55840883

20100	Guca1b	107477	17+:47522341-47529914
20102	Caskin2	140721	11-:115660692-115673494
20103	Car14	23831	3-:95701723-95708562
20104	BC048679	210321	7-:88639165-88643170
20106	MMT00030605		1+:193916252-193923720
20113	Spata22	380709	11+:73153717-73159427
20118	Tmem175	72392	5+:109058899-109075805
20119	Polr3h	78929	15-:81745459-81756639
20122	Tmem86a	67893	7+:54306009-54310146
20124	1700021F05Rik	67851	10-:43244926-43260800
20126	Aoc3	11754	11+:101191918-101200737
20127	Ipo8	320727	6-:148719206-148779959
20132	Cops2	12848	2-:125657337-125684721
20134	Ppp1r14c	76142	10-:6881567-6980391
20135	Gramd3	107022	18+:56559990-56662493
20136	Col13a1	12817	10-:61301245-61441856
20143	Abhd13	68904	8+:9977671-9988679
20145	Glrb	14658	3-:80648052-80717456
20147	Ybx1	22608	4-:118950590-118966411
20148	Pttg1	30939	11-:43233766-43239483
20149	Cabyr	71132	18+:12899857-12913645
20150	Ptprc	19264	1-:139959991-140071843
20151	MMT00052319		1-:17930905-17931239
20152	Tmc5	74424	7+:125770667-125818596
20154	Trdn	76757	10+:33165224-33194477
20156	Apoc2	11813	7-:20256933-20262111
20157	Ets1	23871	9+:32503878-32562090
20158	Slc38a1	105727	15-:96406706-96454601
20162	MMT00050725		8-:82634705-82636075
20163	Prr7	432763	13+:55573385-55574512
20168	Aard	239435	15+:51871563-51877266
20169	Ralgds	19730	2+:28368850-28408590
20171	1700008G05Rik	75497	3-:10245919-10301183
20172	4930502E18Rik	75013	X+:51027999-51041617
20174	Ctnna2	12386	6-:76831648-77929582
20175	Hoxb2	103889	11+:96214114-96215326
20179	EG238836	238836	13+:105708758-105709614
20181	4930403C10Rik	73813	7+:52977550-52984859
20183	MMT00011814		1+:16043390-16044000
20185	Rpl39l	68172	16+:10170320-10175004
20187	Gphn	268566	12-:79326104-79328751
20190	MMT00019998		15+:71140369-71141202
20191	Clca5	229933	3-:144734164-144762122
20193	Olfr771	258540	10-:128597084-128598038
20194	Pnldc1	240023	17-:13081596-13088463
20197	Igh	111507	12-:116152786-116153243

20209	5730557B15Rik	67434	15-:31226418-31297320
20210	Copb1	70349	7-:121359075-121398133
20211	Pdzk1	59020	3+:96634048-96674848
20213	Psd3	234353	8-:70487856-70586173
20215	MMT00044766		4-:144115810-144117020
20216	BC028528	229600	3-:95688882-95695893
20217	Rab3ip	216363	10-:116342836-116376532
20219	MMT00076423		7-:115094858-115096142
20220	MMT00080766	664870	8-:3841083-3842610
20221	Ift57	73916	16+:49699388-49764223
20223	Sc5d	235293	9-:42062195-42072388
20226	EG623402	623402	19-:8663012-8663686
20227	Kras	16653	6-:145168331-145198729
20230	Ppp1r14b	18938	19+:7050984-7051815
20233	Krt6b	16688	15-:101506960-101524679
20237	RP23-433P19.11	574418	2-:121276913-121282429
20238	H2-T9	15051	17-:36175353-36179405
20241	Cts7	56092	13-:61453821-61459557
20243	Wfikkn1	215001	17-:26014634-26016988
20246	Pdia5	72599	16-:35397388-35490961
20254	D12Ertd551e	52635	12-:117608805-117608949
20255	Elfn2	207393	15-:78502303-78504775
20259	B830045N13Rik	215378	1+:148529688-148749599
20265	Rsph1	22092	17-:31391968-31414252
20266	Insig2	72999	1-:123200930-123224339
20267	Lgals1	16852	15+:78757154-78760625
20269	Hsd17b13	243168	5-:104392479-104406408
20273	4733401H18Rik	66706	9-:108468195-108469673
20274	Klf2	16598	8+:74842931-74845248
20275	Igfbp5	16011	1-:72904611-72921460
20279	Apoa1bp	246703	3-:87860444-87862417
20281	Naip1	17940	13-:101177720-101216466
20283	Atp6v1e2	74915	17-:87343446-87347213
20285	Nsd1	18193	13+:55314563-55416241
20291	Rasa2	114713	9-:96442863-96531912
20292	1810048J11Rik	67708	12+:73637365-73643230
20293	MMT00017521		X-:103836719-103838010
20296	C030027H14Rik	77465	10+:121218048-121218499
20300	Plekhb1	27276	7-:107792410-107810879
20303	EG272350	272350	7+:5310762-5311470
20304	Ccrk	105278	13+:64533850-64540971
20309	MMT00079689		15+:77532357-77533543
20312	Htt	15194	5+:35104373-35252045
20313	Rpusd2	271842	2+:118860523-118865493
20317	Pex5l	58869	3-:33030492-33042001
20319	Syne2	319565	12+:77026619-77031717

20321	Ms4a6b	69774	19+:11593118-11604557
20324	Tppp	72948	13+:74158590-74169146
20325	4921522P10Rik	66719	8+:8661800-8664728
20328	Csl	71832	10-:99220338-99222292
20330	Foxa3	15377	7-:19598631-19608888
20332	Stau2	29819	1-:16218886-16221172
20333	Calca	12310	7-:121776811-121779808
20335	Srgn	19073	10-:61957175-61970503
20337	St6galnac4	20448	2+:32442602-32455214
20339	OTTMUSG00000010	626316	4+:146064815-146094022
20340	Slfn14	237890	11-:83092652-83097665
20341	Srebf2	20788	15+:81977675-82035810
20343	Ttc19	72795	11+:62094983-62128748
20345	AI842396	103844	11-:70501861-70513646
20346	Gna-rs1	14670	17+:36117622-36125811
20348	EG210853	210853	17-:22065153-22426864
20349	MMT00054511		9-:65888767-65890437
20350	Pigc	67292	1+:163899357-163902461
20353	Antxr1	69538	6-:87083847-87285436
20355	Zfyve28	231125	5-:34541416-34630852
20356	Mrps35	232536	6+:146991325-147019421
20361	Mtmr14	97287	6+:113187854-113231377
20362	Sh3bp5l	79566	11+:58145048-58161228
20363	Dcun1d2	102323	8-:13255963-13288167
20364	Zfp422	67255	6-:116574935-116578939
20370	Sccpdh	109232	1+:181606521-181617318
20372	Heatr1	217995	13+:12524927-12531159
20373	Klhl14	225266	18-:21708881-21810869
20374	Zfp780b	338354	7-:28695189-28790337
20380	MMT00058516		11+:70171425-70174036
20381	Vtn	22370	11+:78312787-78315825
20384	BC034069	100038347	7+:80885194-80921804
20385	Chd1l	68058	3-:97364670-97414113
20387	Defb1	13214	8+:22887081-22905191
20388	Olr1	108078	6-:129435269-129457172
20390	Cd9	12527	6-:125410283-125444711
20391	Oas1h	246729	5+:121311430-121323512
20393	1110018G07Rik	68497	12-:86259100-86284342
20394	EG668225	668225	15-:100882896-100884841
20397	Sema3e	20349	5+:14025316-14254314
20398	Prh1	19131	6+:132521906-132521950
20400	Kif7	16576	7-:86842597-86859044
20402	MMT00007367		16+:3391231-3391269
20403	MMT00024375		14+:38535407-38536343
20404	MMT00003113		19-:45471254-45472111
20405	Clip1	56430	5-:124029082-124062172

20406	Cacng2	12300	15-:77825392-77949710
20410	MMT00028069		9-:88169814-88176034
20413	Wdr86	269633	5-:24218083-24218639
20416	Cnn1	12797	9+:21903711-21913169
20417	Trappc9	76510	15-:72420050-72587793
20419	Adra1b	11548	11-:43589362-43650305
20421	Cdkn2c	12580	4-:109333751-109338267
20423	8-Mar	71779	6+:116288399-116359556
20425	Zbtb11	271377	16+:55973992-56008993
20428	MMT00026261		6-:47736147-47738382
20429	Tmem202	73893	9-:59366461-59373259
20430	Trio	223435	15-:27660409-27849046
20431	Slc7a7	20540	14-:54989127-55027885
20432	Smc1b	140557	15-:84895118-84962387
20433	Cdca5	67849	19+:6085098-6091773
20436	Mctp1	78771	13+:76521982-76598910
20441	Olfr1034	258216	2+:85886874-85887572
20444	Ckap2l	70466	2-:129094653-129122947
20449	MMT00062045		3+:98624862-98626457
20451	Agr2	23795	12+:36719511-36730667
20454	MMT00018457		2-:36929746-36938002
20457	Gtf2a2	235459	9+:69860348-69870672
20458	Dync2h1	110350	9-:6928501-7137110
20459	MMT00030019		2+:82347278-82348437
20460	Emcn	59308	3+:137004106-137094032
20461	H2-Ab1	14961	17+:34400256-34405429
20462	Lrrtm2	107065	18-:35369584-35374573
20468	Spa17	20686	9-:37410885-37421199
20470	Scml4	268297	10+:42580672-42678541
20473	Gpr137b-ps	664862	13-:12707324-12742647
20474	Gnb3	14695	6-:124784257-124790293
20481	Tmem106b	71900	6+:13019855-13035064
20482	Vwa1	246228	4-:155143412-155148640
20484	Ces1	12623	8-:95826294-95861093
20485	6430520M22Rik	319716	6+:145809702-145811100
20486	C1qtnf1	56745	11+:118295166-118311276
20488	Bok	51800	1+:95582278-95592339
20490	Tmem143	70209	7+:53152444-53172781
20491	1700125H20Rik	73634	11+:84983903-84994646
20492	Glra3	110304	8+:58419700-58604145
20496	Mtpn	14489	6-:35461919-35489819
20498	Porcn	53627	X-:7770977-7783627
20499	Wdr44	72404	X+:23270284-23383107
20500	2810441K11Rik	68642	19-:10624955-10630728
20503	Krtdap	64661	7+:31572915-31576114
20505	Ttll9	74711	2+:152788241-152833076

20506	1700011F03Rik	74221	6-:70863080-70868918
20507	Sh2d6	71130	6-:72463636-72470621
20510	Prame	75829	X-:132147539-132162246
20511	Decr2	26378	17-:26218155-26227109
20513	Il1rapl2	60367	X+:134709065-134771192
20514	Rprm	67874	2-:53936647-53937952
20515	Dimt1	66254	13+:107737253-107749840
20519	Col1a1	12842	11+:94797565-94814353
20520	EG546143	546143	9-:72827529-72833594
20521	BC057079	230393	4+:87823985-88056913
20522	MMT00054229		10+:56375999-56397773
20524	Cdc27	217232	11-:104381584-104411792
20528	Etnk1	75320	6+:143115768-143152135
20530	EG640268	640268	9+:48228150-48240042
20531	MMT00015606		3-:69123941-69124507
20532	Rnf26	213211	9-:43919758-43921558
20534	Stk24	223255	14-:121686020-121778284
20535	Rbm4	19653	19-:4785153-4793860
20537	Mmp13	17386	9+:7272544-7283330
20539	Klk1b5	16622	7+:51471828-51476082
20541	Vapa	30960	17-:65929393-65962836
20544	4932438A13Rik	229227	3+:36762046-36776660
20548	1700041C02Rik	73332	4-:118996446-119004224
20552	Cdh9	12565	15+:16707815-16786045
20553	Echdc2	52430	4+:107838100-107851912
20559	Nat13	72117	16+:44139996-44160092
20563	Mns1	17427	9+:72286341-72306381
20565	MMT00065821		7-:27254261-27255255
20566	Tnni2	21953	7+:149628372-149630310
20567	Ggt6	71522	11+:72249027-72252065
20570	Ap3m1	55946	14-:21854031-21871407
20571	Tmem45a	56277	16-:56806491-56885976
20572	Rdh5	19682	10-:128350648-128356250
20573	MMT00040001		3+:101037410-101038015
20574	Apool	68117	X+:109424971-109486297
20579	Zkscan3	72739	13-:21479544-21499920
20581	V1rc7	113864	6+:57183432-57184344
20583	Cdh16	12556	8-:107138047-107148161
20585	Snx7	76561	3-:117484577-117549625
20587	Abcc1	17250	16+:14361688-14473660
20588	Brd2	14312	17-:34248976-34256255
20589	Frmd8	67457	19-:5851285-5875230
20592	Kcnc1	16502	7+:53652911-53683814
20594	Kdelr1	68137	7+:53128209-53139096
20601	Rpo1-4	20019	6+:71859156-71929352
20606	MMT00042166		7-:26205542-26208082

20610	Ttll11	74410	2-:35606760-35835059
20611	Map2k3	26397	11+:60745558-60766297
20613	4930431B09Rik	74645	3-:100275528-100293078
20622	Cd47	16423	16+:49855836-49911192
20624	Utp15	105372	13+:99016769-99018856
20625	Pgam2	56012	11-:5701646-5703736
20628	Hoxd10	15430	2+:74530036-74532425
20632	Cep78	208518	19-:16029841-16059370
20635	Jmjd6	107817	11-:116698746-116704689
20636	MMT00055765		1+:135061034-135066666
20642	V1rd7	81012	7+:24416996-24419385
20643	Rcvrn	19674	11+:67508827-67516835
20644	Lct	226413	1-:130181333-130224880
20648	B430212C06Rik	338360	18-:67482672-67484375
20649	5830472M02Rik	76080	2+:163428082-163442847
20650	Naaladl1	381204	19+:6105797-6116009
20652	Dlx4	13394	11-:95001764-95006795
20653	1700049L16Rik	108950	10-:71442840-71443382
20654	Maea	59003	5+:33678258-33715162
20657	MMT00059805		7+:78841754-78841979
20661	Tuft1	22156	3-:94416680-94462728
20663	Depdc1a	76131	3+:159158415-159192917
20664	Rpl13a	22121	7-:52381292-52384102
20666	Mosc1	66112	1-:186610654-186635182
20667	MMT00054056		
20668	Rras	20130	7+:52273382-52277014
20670	Klhl25	207952	7+:82993277-83012331
20674	D17H6S53E	114585	17+:35264059-35265424
20676	MMT00067648		11+:6346185-6346571
20678	Cyp39a1	56050	17+:43804482-43887796
20680	Xpo4	57258	14-:58201109-58283793
20681	EG667683	667683	6-:69388204-69388768
20682	Usp40	227334	1-:89846587-89905109
20683	Foxd4	14237	19-:24973454-24975799
20684	Twist1	22160	12+:34642676-34644691
20688	Iyd	70337	10-:6791663-6806262
20689	Cnksr3	215748	10+:3134785-3227478
20690	Zfp276	57247	8+:125779841-125793635
20697	Galnt5	241391	2+:57850800-57891637
20703	Itga11	319480	9+:62525751-62631795
20705	Supv3l1	338359	10-:61891966-61913355
20714	MMT00015505		5+:6674890-6749370
20715	Tmem179b	67706	19-:8847018-8848932
20717	Snf8	27681	11+:95896230-95908719
20719	Sall1	58198	8-:91552278-91566326
20720	Ptk2b	19229	14-:66772098-66899831

20721	4432412L15Rik	74337	8+:86545367-86554191
20723	Cdh1	12550	8+:109127267-109194146
20725	Nudt5	53893	2+:5775859-5789410
20726	Ei24	13663	9-:36586743-36604653
20727	Mmp11	17385	10-:75385968-75395208
20728	MMT00055001		
20729	Ttc28	209683	5+:111717228-111717889
20730	Dusp26	66959	8+:32200120-32207520
20735	Tnrc6b	213988	15+:80541488-80577903
20736	Itgb1bp1	16413	12-:21273280-21292080
20740	Ola1	67059	2-:72930859-73052504
20742	Birc2	11797	9-:7818226-7835255
20743	Trp53inp1	60599	4+:11083587-11101524
20744	Ipmk	69718	10+:70810582-70844783
20745	Sord	20322	2+:122060574-122091073
20746	2210015D19Rik	76508	11+:5662128-5684713
20749	Usp1	230484	4+:98590620-98602227
20750	Foxo4	54601	X+:98449866-98455033
20754	Crisp1	11571	17-:40430708-40456122
20756	Omd	27047	13+:49678115-49687978
20757	Suclg1	56451	6+:73198666-73226876
20759	Slc17a1	20504	13+:23962182-23987568
20764	Vit	74199	17+:78907512-79026729
20765	MMT00060789		13-:62771158-62772324
20769	Ube2e1	22194	14-:19115262-19164313
20771	Slc16a10	72472	10-:39754934-39862065
20774	Atp6v1c2	68775	12-:17291526-17331518
20775	Cd244	18106	1+:173489382-173507477
20777	MMT00010185		7-:5078280-5080880
20784	Csad	246277	15-:102007428-102019474
20786	Bspry	192120	4+:62141100-62158332
20790	Efna5	13640	17-:62956667-63230493
20791	MMT00014813	667370	19+:34682173-34687127
20792	MMT00032790		5-:42890113-42891653
20796	E130113E03Rik	319433	14+:63282502-63283949
20798	Ccpg1	72278	9+:72833391-72864140
20800	MMT00007848		3+:75195607-75196036
20801	Mrps11	67994	7+:85928195-85937874
20802	Cdc42se2	72729	11-:54531687-54601187
20803	Tmem50a	71817	4-:134453760-134470915
20804	Eno1	13806	4+:149611363-149622984
20808	Bms1	213895	6-:118333397-118369433
20810	Mttp	17777	3-:137753849-137794288
20811	Dlst	78920	12+:86451788-86475795
20813	1700049E17Rik	73415	14+:42386499-42394594
20814	Ptpn18	19253	1+:34516602-34530622

20822	Smurf1	75788	5-:145637396-145663456
20828	MMT00007878		8-:40805672-40806165
20829	Zfp385c	278304	11-:100490145-100499206
20830	MMT00057509		16+:53983940-53986320
20831	MMT00057242		X+:133424796-133425379
20833	MMT00022580		
20835	Pgrmc1	53328	X+:34138269-34146067
20837	Dlgap4	228836	2+:156547622-156588828
20839	Nefm	18040	14-:68738095-68742870
20840	Rasa1	218397	13-:85355633-85396069
20842	MMT00046069		6-:148647645-148648158
20847	Pdik1l	230809	4-:133832574-133843142
20852	Extl2	58193	3+:115711249-115731878
20853	Tatdn1	69694	15-:58721707-58765370
20854	Commd7	99311	2-:153444550-153458492
20855	Phldb2	208177	16-:45746356-45953710
20858	Duox1	99439	2+:122143654-122173706
20860	Gm1564	268491	11+:102536537-102542051
20861	Dcst1	77772	3-:89161394-89169177
20862	Olfr827	258297	10-:129647217-129648184
20865	Appl1	72993	14+:27732688-27733297
20867	Cnih	12793	14-:47395256-47408206
20870	1700028K03Rik	76421	5+:107926739-107940230
20871	Pop1	67724	15+:34425077-34460392
20880	Raf1	110157	6-:115568615-115626521
20885	MMT00004673		6+:23687067-23688033
20886	Mylpf	17907	7+:134356373-134357776
20887	MMT00060807		7-:111840853-111841877
20890	Psmb4	19172	3-:94688014-94690862
20892	C1r	50909	6+:124462649-124473124
20894	Cyp4a12a	277753	4+:114971704-115005419
20896	Rasa3	19414	8-:13567217-13677572
20897	Clca6	99663	3-:144615450-144638010
20900	Epb4.1l4a	13824	18-:33956614-34165788
20902	Itln1	16429	1-:173448372-173461345
20903	Rpia	19895	6-:70715694-70742149
20906	Tpbpb	116913	13-:61002548-61006208
20907	Lims2	225341	18+:32091171-32118272
20908	Olfr583	258752	7+:110199813-110200772
20911	Narg1	74838	3+:51246688-51277946
20912	Chordc1	66917	9+:18096710-18118444
20913	Osgepl1	72085	1+:53370519-53382993
20914	Utx	22289	X+:17799871-17857060
20917	Gstm6	14867	3-:107742146-107746551
20918	Nxph3	104079	11-:95371166-95375585
20919	Ceacam18	72431	7+:50890075-50904280

20925	2810410L24Rik	76377	11-:120048945-120097489
20935	Gpr83	14608	9+:14664697-14673943
20939	Il13	16163	11-:53444825-53448204
20941	Zfp28	22690	7+:6238228-6349624
20942	MMT00006980		12-:119970961-119993457
20943	1700113H08Rik	76640	10+:86668719-86693331
20944	Tmem164	209497	X+:139115999-139117492
20945	Traf4	22032	11-:77972902-77979033
20946	Nufip2	68564	11+:77420825-77526241
20952	Zdhhc9	208884	X-:45525661-45561861
20954	5830403L16Rik	240817	1-:155697254-155747360
20957	Arhgef5	54324	6+:43223588-43239320
20958	4933403F05Rik	108654	18-:68425430-68459989
20960	Abcc3	76408	11-:94204604-94254229
20961	Lig3	16882	11+:82594724-82615690
20963	Ifna6	15969	4+:88473251-88474201
20965	Usp28	235323	9+:48808455-48850621
20966	MMT00014378		4+:69514980-69515718
20967	Fabp2	14079	3+:122598295-122602423
20970	Slco6b1	67854	1-:98802752-98885276
20971	Tsga13	116732	6-:30846980-30865573
20972	MMT00031355		2-:35346729-35421930
20973	Stx2	13852	5-:129490441-129514447
20979	D10Jhu81e	28295	10-:77624617-77632516
20980	Taf12	66464	4+:131830327-131849240
20983	Hlx	15284	1-:186551024-186556487
20984	MMT00036444		18-:47574120-47575031
20986	Olfr805	258548	10-:129159638-129160598
20988	MMT00010339		18-:48228593-48229474
20991	2900093K20Rik	68037	2+:163244447-163246095
20994	Pcdhb17	93888	18+:37644812-37647943
20997	MMT00028135	631323	11+:57997341-58002440
20999	1110049F12Rik	66193	4-:135531518-135542320
21000	Vmo1	327956	11-:70327044-70328104
21003	Uckl1	68556	2-:181303852-181327780
21008	4933436I01Rik	66780	X-:65173038-65174625
21012	MMT00064569		
21013	Vnn3	26464	10+:23571277-23589637
21014	Usp54	78787	14-:21368139-21380894
21015	MMT00014048		19+:17615518-17636722
21017	Gstz1	14874	12-:88495797-88495928
21018	Eif3m	98221	2-:104839812-104857184
21019	Picalm	233489	7+:97279031-97357434
21020	Rgmb	68799	17-:15944108-15955445
21030	Ccdc80	67896	16+:45093786-45128001
21039	Pcnxl2	270109	8-:128275430-128421981

21041	Cyp17a1	13074	19-:46741672-46747363
21045	Crb3	224912	17+:57201692-57205335
21048	Cdrt4	66338	11+:62764694-62806597
21049	1700129I04Rik	330577	7-:89783078-89794339
21052	Marcksl1	17357	4+:129190887-129193218
21053	Ypel1	106369	16+:17070321-17086434
21055	Gltp	56356	5-:115120415-115140870
21060	Ela1	109901	15-:100504853-100518353
21062	Rpl7l1	66229	17-:46912049-46919595
21067	MMT00077109		5-:105170617-105181507
21069	Tet2	214133	3-:133148475-133150801
21070	Cnot8	69125	11+:57917730-57931348
21071	Il4i1	14204	7+:52091658-52096178
21075	Gtf2h5	66467	17+:6079816-6085478
21076	Abra	223513	15-:41697235-41701251
21078	MMT00067065		10-:29803972-29805141
21079	Tfpi2	21789	6-:3912595-3918354
21080	MMT00035581		13+:28163933-28180873
21083	Serpinb9c	20707	13-:33241846-33249693
21084	Nkiras2	71966	11+:100484268-100488916
21089	MMT00048642		1-:146069047-146070255
21091	Nup160	59015	2+:90517371-90576484
21092	Zfp750	319530	11-:121372290-121375361
21095	9130401M01Rik	75758	15-:57853830-57865772
21098	Terf2ip	57321	8+:114535304-114544360
21099	Rapgef6	192786	11+:54251798-54508080
21100	MMT00065179		
21101	1110032A03Rik	68721	9-:50571831-50576209
21102	Cmtm7	102545	9-:114665954-114690947
21105	Pex26	74043	6+:121133905-121146203
21106	Il1f5	54450	2+:24132918-24137595
21108	Fndc3a	319448	14-:72937757-73109800
21109	Col8a2	329941	4+:125986859-125991185
21110	Emg1	14791	6-:124654404-124662145
21111	Tmem132c	208213	5+:128031872-128045272
21115	MMT00082318		14-:19376517-19377691
21119	Apob	238055	12+:7984453-8023641
21120	Igk-C	16071	6+:70613261-70676963
21122	Fndc7	320181	3-:108683854-108692717
21125	Mtif3	76366	5-:147766504-147775350
21126	Dera	232449	6+:137703061-137805860
21127	Triobp	110253	15+:78813509-78836300
21130	MMT00011706	66449	16-:4616465-4624946
21134	Ppp2r2a	71978	14-:67634163-67657817
21135	Gm711	279029	2+:26806073-26809014
21138	Auts2	319974	5-:132503558-132526282

21142	Gm129	229599	3-:95682425-95686173
21143	Ypel3	66090	7+:133920488-133924023
21149	A230065H16Rik	380787	12+:112645018-112650301
21151	Nkap	67050	X+:34666806-34688277
21153	Mr1	15064	1-:156975029-156993821
21157	Efemp1	216616	11+:28767772-28826733
21159	Pik3c2g	18705	6+:139564832-139668705
21160	Irs2	384783	8-:10984687-11008770
21162	MMT00008728		3-:12384339-12662887
21163	Slc16a5	217316	11+:115323818-115335706
21165	Hmbs	15288	9-:44144376-44150429
21167	Irf5	27056	6+:29476732-29487319
21168	Olfr498	258304	7+:115608839-115609831
21171	1700016G05Rik	67690	6-:40464822-40469507
21173	Rassf6	73246	5-:91032120-91069501
21175	1700025I17Rik	70372	13+:53489005-53550188
21182	Runx2	12393	17-:44744854-44951576
21187	RP23-54E4.8	625752	13-:21388116-21388587
21188	Pnkp	59047	7+:52112773-52118020
21189	Arl5c	217151	11-:97850895-97857486
21191	Znrf1	170737	8+:114060843-114148194
21192	2310047A01Rik	71918	14-:26531130-26588357
21195	Tbc1d7	67046	13-:43247112-43266728
21199	Pcdh9	211712	14-:93414732-93429790
21202	Acly	104112	11-:100337671-100389186
21203	C330018D20Rik	77422	18-:57115697-57133141
21205	Pkmyt1	268930	17+:23863407-23873704
21207	Pi15	94227	1+:17592075-17615527
21209	Ebf4	228598	2+:130121763-130196587
21211	2010301N04Rik	72103	6-:87579740-87622058
21212	Klb	83379	5+:65739649-65775242
21214	Scd1	20249	19-:44472409-44481899
21218	Col9a2	12840	4+:120711936-120727903
21223	Pira1	18722	7-:3788417-3867086
21225	Zfp449	78619	X+:53601457-53616391
21228	A430107D22Rik	320484	17-:32527175-32540328
21229	Acss1	68738	2-:150443846-150493965
21232	Nkiras1	69721	14+:19103798-19112954
21234	Olfr665	258810	7+:112029222-112030172
21236	Glrx5	73046	12+:106270898-106279120
21237	Poli	26447	18-:70668337-70689975
21239	Ctdp1	67655	18-:80604703-80637598
21240	Dock11	75974	X+:33428865-33616556
21242	Zfp654	72020	16-:64765058-64794382
21244	Nfatc1	18018	18-:80845197-80904863
21245	Dock2	94176	11-:34501010-34597354

21246	Telo2	71718	17-:25236521-25252720
21247	Tmed5	73130	5-:108550665-108561610
21249	MMT00056913		X-:71739035-71740292
21251	Cpt1a	12894	19+:3323278-3385732
21253	MMT00068187		4-:105808498-105810353
21254	Bbs7	71492	3-:36474214-36512313
21255	Kif18a	228421	2+:109120894-109181901
21256	Tirap	117149	9-:34994369-35007459
21257	MMT00009771		7-:106395146-106397764
21258	2010003K15Rik	75606	10-:25247749-25256077
21259	5430416O09Rik	71406	4+:43742922-43744950
21260	Ropn1	76378	16+:34666781-34678566
21261	Pax2	18504	19+:44831883-44835478
21262	Myot	58916	18+:44493727-44515376
21265	Zfp712	78251	13-:67140520-67162107
21266	Cercam	99151	2+:29725012-29738368
21270	V1rg6	171241	7+:12465691-12466607
21271	Pax9	18511	12+:57796657-57812216
21272	Scnn1g	20278	7+:128878020-128911990
21273	St14	19143	9-:30896992-30939273
21278	MMT00072454		19-:8586378-8587816
21287	Cplx4	225644	18-:66115377-66129832
21288	Tcp11l2	216198	10+:84039691-84077096
21291	Slc39a4	72027	15-:76442814-76447322
21295	Snf1lk	17691	17-:31981201-31992723
21296	Rpl14	67115	9+:120480633-120483770
21298	Psg17	26437	7-:19399342-19406956
21300	Hdac10	170787	15-:88953735-88959624
21303	Kcnk2	16526	1-:191031815-191167711
21306	Pvrl1	58235	9+:43552726-43612097
21307	Krcc1	57896	6+:71232273-71235312
21313	Rhou	69581	8+:126177828-126187782
21314	Mycbp2	105689	14-:103512626-103745734
21315	A430005L14Rik	97159	4+:153331368-153336031
21318	MMT00000812		7+:34420180-34482717
21320	Kcne1l	66240	X-:138739295-138740741
21322	Uspl1	231915	5+:149996289-150027009
21323	MMT00074228		X-:76876748-76877213
21326	MMT00047910		5-:39792387-39793484
21329	Azi2	27215	9+:117949801-117972689
21331	Slc10a6	75750	5-:104034729-104058422
21333	Cirbp	12696	10+:79630585-79634398
21335	Trp53rk	76367	2+:166619296-166621652
21340	Armc1	74252	3-:19033754-19063079
21348	MMT00039685		X-:136210588-136211975
21351	Shisa5	66940	9+:108941140-108960236

21352	Dtnbp1	94245	13-:45017577-45097341
21354	Xpa	22590	4-:46188113-46209183
21356	Nkx2-6	18092	14+:69789789-69793597
21357	Cd3g	12502	9-:44777771-44788507
21360	Sox11	20666	12-:28024667-28027561
21364	Ppm1h	319468	10+:122219238-122379594
21369	Cd4	12504	6-:124814710-124838227
21371	Spnb2	20742	11-:30007625-30098164
21375	MMT00043149		13+:81203605-81204605
21376	MMT00058413		3-:112133818-112134721
21378	Havcr2	171285	11+:46268436-46294754
21380	Ier5	15939	1-:156944656-156946766
21381	Rilpl2	80291	5-:124913275-124928244
21387	Cnot7	18983	8-:41579081-41595524
21388	Gbe1	74185	16+:70314380-70569959
21389	Odf2	18286	2+:29745619-29778056
21390	Cnga2	12789	X+:69248605-69255573
21391	Lgi2	246316	5-:52929102-52957519
21394	Cdh15	12555	8+:125371865-125391297
21398	Tmco6	71983	18+:36894798-36902045
21400	Il1rapl1	331461	X-:84545923-84550289
21404	Hoxa10	15395	6-:52181195-52184938
21406	Lrp2	14725	2-:69262392-69276531
21408	Svil	225115	18+:4920535-5119286
21409	Gldc	104174	19-:30172938-30249908
21412	Afap1l1	106877	18-:61889915-61946343
21414	BC017643	217370	11-:121083904-121090579
21415	Has1	15116	17-:17980292-17992152
21421	Usp34	17847	11+:23332245-23390534
21424	Supt3h	109115	17+:44914119-44923683
21425	Rab6	19346	7+:107756300-107788704
21427	Tc2n	74413	12-:103074865-103151474
21428	MMT00012296		13-:18213671-18214236
21431	Xbp1	22433	11+:5420969-5425873
21433	Ubxd6	108159	8-:34730660-34753816
21438	Plekha4	69217	7+:52781698-52809598
21439	Il25	140806	14+:55551607-55554671
21440	Tmsb10	19240	6-:72907346-72908393
21441	MMT00041291		6+:100676497-100677620
21442	Rad51l1	19363	12+:80401535-80915338
21444	Pnpt1	71701	11+:29030744-29061595
21446	Ccl12	20293	11+:81915379-81916793
21448	Paqr7	71904	4+:134052892-134064725
21449	Serpinb3a	20248	1-:108942592-108948916
21450	Col22a1	69700	15-:71628899-71837618
21451	Gja5	14613	3+:96854551-96855627

21453	Vmn2r108	627805	17-:20599846-20609335
21454	MMT00068006		1+:38295963-38302553
21457	MMT00036264		1+:62707381-62708331
21459	Hpse	15442	5-:101109419-101148700
21461	MMT00034426		17-:22016905-22018029
21465	Nxn	18230	11-:76072066-76212562
21466	Rimbp2	231760	5-:129266555-129325722
21471	Stmn2	20257	3+:8509526-8561604
21477	Tmem27	57394	X+:160528118-160556791
21478	Loxhd1	240411	18+:77580666-77613937
21479	Steap2	74051	5-:5671724-5677285
21481	Bcl2a1c	12046	9+:114239273-114239658
21483	Ippk	75678	13+:49516638-49558430
21491	MMT00017308		X-:166637383-166637413
21497	Pias2	17344	18+:77336331-77391865
21500	MMT00018393		16+:68511115-68511943
21501	Stmn1	16765	4+:134026575-134029316
21505	Acsbg2	328845	17-:56982526-57007795
21506	St7	64213	6+:17699232-17892965
21508	Echdc1	52665	10+:29033429-29065463
21509	Tekt3	71062	11+:62875158-62908462
21511	MMT00054100		
21512	Gas6	14456	8-:13465375-13494506
21517	Sil1	81500	18-:35426050-35658520
21518	Adamts17	233332	7+:74269810-74295255
21519	1700012B15Rik	74173	12+:3235767-3235829
21524	Ncoa4	27057	14+:32978989-32991030
21526	Mkl1	223701	15-:80842713-81020954
21528	Nup85	445007	11+:115431296-115445298
21531	Ttc23	67009	7+:74792335-74871462
21536	MMT00011639		17-:37306653-37307591
21538	Rg9mtd1	52575	16-:56033832-56037887
21539	Rbm11	224344	16+:75593188-75601499
21540	Coil	12812	11+:88833933-88834428
21541	Brf2	66653	8-:28233055-28239108
21542	Larp4	207214	15+:99803129-99846782
21545	Pcdha9	192161	18+:37157533-37347311
21547	Il10	16153	1+:132916423-132921547
21548	MMT00052315		2+:24034090-24036471
21549	Rtp4	67775	16+:23610021-23613819
21550	Sgcd	24052	11-:46792765-47193024
21551	Bmf	171543	2-:118358294-118372743
21552	MMT00031646		12-:109679116-109679553
21553	EG628438	628438	18+:47271792-47272216
21554	EG627607	627607	12+:88081891-88095385
21556	4930579F01Rik	67741	3-:137827043-137849678

21560	Apol7e	666348	15+:77542747-77548355
21563	Arhgap9	216445	10+:126760787-126767000
21564	Cdh18	320865	15+:23366204-23404173
21565	Pde6h	78600	6+:136907781-136911793
21568	Cdc73	214498	1-:145454629-145549814
21571	D030074E01Rik	75964	18-:20975727-21054592
21574	Acot11	329910	4-:106420109-106472362
21575	Cyp2c55	72082	19+:39081508-39117177
21579	Lsm12	268490	11-:102023809-102046566
21581	Cbx3	12417	6+:51425235-51432603
21582	Setx	269254	2+:29000906-29003997
21583	Gstz1	14874	12+:88488724-88505671
21585	Hipk1	15257	3-:103547595-103595102
21587	MMT00002799		5+:69852287-69854009
21588	Snrpa	53607	7-:27972024-27981078
21591	Hectd3	76608	4+:116667964-116677875
21592	Wdr46	57315	17+:34077682-34086360
21594	Hbxip	68576	3+:107081774-107086997
21595	Trpv1	193034	11+:73051727-73074008
21596	P2rx5	94045	11+:72974026-72986187
21602	V1ra5	113847	6-:89819507-89820448
21604	Klhl24	75785	16+:20097641-20123303
21605	Uevld	54122	7-:54203014-54213842
21607	Aldh1a1	11668	19+:20676457-20717944
21608	Tpcn2	233979	7-:152439963-152444960
21609	MMT00039978		X+:100232943-100233745
21611	1700085A12Rik	78631	X+:36699441-36700135
21617	MMT00050850		18-:82885510-82895572
21618	MMT00041235		6+:90848276-90856207
21619	MMT00060699		7+:107093648-107118866
21620	Tmem166	232146	6+:81991666-82043092
21621	MMT00071794		17-:37977249-37978796
21623	Gpr98	110789	13-:81731551-81772338
21627	Fcgr3	14131	1-:172981325-172989466
21630	Ahcyl2	74340	6+:29718497-29720618
21632	MMT00046455		9+:108062719-108063800
21634	MMT00042049		
21636	1700127D06Rik	76999	7+:51200381-51201297
21637	Cpxm1	56264	2-:130216511-130223310
21639	Hspa9	15526	18-:35097088-35113977
21640	Hspa14	50497	2-:3406125-3430018
21643	Agtr1a	11607	13+:30428307-30474735
21645	Slc22a6	18399	19+:8692604-8701011
21649	MMT00076721		10+:72664358-72666028
21650	Ap4b1	67489	3+:103613552-103625946
21651	Glipr1l2	67537	10+:111520409-111545154

21652	Olfr700	258593	7-:113949025-113949974
21655	Rab40b	217371	11-:121218116-121249453
21656	MMT00054267		11-:7083159-7086362
21657	Etaa1	68145	11-:17839655-17853878
21658	Oxt	18429	2+:130401945-130402667
21660	Il1rn	16181	2+:24192378-24205150
21662	A530088I07Rik	212167	5+:20692130-20797531
21664	Insig1	231070	5+:28397977-28405203
21665	Nat5	67877	2+:145729036-145742052
21666	Ncor1	20185	11-:62131042-62233285
21667	MMT00076649		18+:47030475-47030960
21669	Zic5	65100	14-:122858381-122864892
21670	Ggt1	14598	10+:75036337-75048927
21675	Mosc2	67247	1-:186637572-186669709
21681	9130011J15Rik	66818	8-:75089788-75094960
21682	Med17	234959	9-:15064795-15084311
21683	Prdm10	382066	9+:31087890-31186128
21684	Cd82	12521	2-:93259268-93302568
21685	Gzma	14938	13-:113884032-113891187
21686	MMT00026800		16+:47212481-47213039
21687	Zbtb7b	22724	3-:89183210-89185179
21692	MMT00013924		9+:57331735-57332397
21693	BC029169	208659	11-:109534234-109583569
21703	Tmem38b	52076	4+:53838933-53873461
21707	Tesc	57816	5+:118477926-118511876
21710	Slc30a6	210148	17+:74801250-74822853
21712	Ttc5	219022	14-:51385085-51405190
21713	Adnp	11538	2-:168007547-168010033
21715	Siah1a	20437	8-:89247906-89269833
21716	Tmem85	68032	2-:112203175-112208184
21717	EG628893	628893	X-:74962581-74963366
21720	Clec4d	17474	6+:123212192-123224934
21721	Styk1	243659	6-:131249161-131262616
21722	MMT00054655		11-:12836541-12837709
21723	Homer1	26556	13+:94075012-94126407
21727	Khk	16548	5+:31224288-31233419
21729	Hdc	15186	2-:126419402-126444379
21733	Rhebl1	69159	15-:98708209-98711693
21735	C130073F10Rik	242574	4-:101562629-101566382
21737	Srst	20819	17-:83209639-83211256
21739	Epb4.1	269587	4-:131479017-131628013
21743	Ccno	218630	13+:113778105-113780986
21745	MMT00039382		
21747	H2afx	15270	9+:44142793-44144154
21750	Rab31	106572	17-:66001069-66122021
21751	Ptpn6	15170	6-:124670734-124683122

21753	Pcdhb14	93885	18+:37607496-37609885
21754	Synj2	20975	17+:5975640-6044289
21761	D14Ertd449e	66039	14-:26743655-26746694
21762	Lage3	66192	X-:71597500-71598957
21764	Tgm5	74176	2-:120871846-120911558
21765	Arpp19	59046	9+:74885723-74904611
21767	Gm1758	385737	16+:14502192-14507354
21769	Loxl2	94352	14+:70009000-70095642
21772	MMT00002494		6+:34989351-34989622
21773	Pcdhb2	93873	18+:37454629-37457027
21774	MMT00031273		4-:111463186-111481072
21775	MMT00051649		14-:76329373-76331352
21780	Ankrd35	213121	3+:96474259-96494956
21785	MMT00043723		3-:69139122-69139666
21788	MMT00036513		5+:109665987-109667178
21790	Smarcal1	54380	1+:72629844-72679704
21791	MMT00042985		9-:96714255-96717996
21793	Pla2g12a	66350	3+:129581636-129597872
21794	Pfkp	56421	13-:6580420-6647976
21795	0610009B22Rik	66050	11-:51498886-51502136
21797	Dst	13518	1+:34068669-34365495
21799	Cops5	26754	1-:10014913-10027979
21800	EG626058	626058	1+:155260671-155261163
21801	Serpinb6a	20719	13-:34009796-34027847
21803	Slc15a2	57738	16-:36750266-36785048
21805	Plk3	12795	4-:116800998-116806407
21806	MMT00064565		13-:51300538-51301473
21811	Ak3l1	11639	4+:101092343-101139177
21814	Accn5	58170	3+:81800843-81825155
21817	MMT00028065		11-:57877313-57886415
21818	2010003K11Rik	69861	19-:4496785-4498582
21821	Olfr1032	258572	2+:85847934-85848865
21823	Atp6v0d1	11972	8-:108048369-108089825
21824	5830443L24Rik	76074	5-:105443175-105447782
21827	MMT00010458	403088	6-:42537500-42547275
21837	BC052328	223433	15-:27586020-27611238
21839	Gprasp1	67298	X+:132336824-132338003
21840	Tmie	20776	9-:110769364-110770592
21841	RP23-157O10.7	56013	11-:97372211-97372655
21844	Ncoa7	211329	10-:30367339-30491624
21845	Gm648	270599	X-:53797243-53798334
21847	MMT00063855		X+:114000424-114000790
21849	Icam2	15896	11-:106238970-106243955
21853	5430432N15Rik	74490	7+:52895344-52901137
21854	Fkbp3	30795	12-:66163422-66174927
21858	Neil3	234258	8-:54672220-54724350

21859	Fancg	60534	4-:43015817-43022982
21863	Dok2	13449	14+:71174187-71178300
21866	Hhipl1	214305	12+:109544411-109566422
21871	Spock3	72902	8+:65430028-65835648
21872	Stambpl1	76630	19+:34307131-34314816
21874	Nppb	18158	4+:147360084-147361087
21877	Slitrk6	239250	14-:111148972-111151495
21881	MMT00038325		5-:107167134-107167784
21888	Kti12	100087	4+:108520461-108522017
21889	Mgat3	17309	15+:80041403-80043020
21891	Nnt	18115	13-:120124552-120197723
21896	Lce1c	73719	3+:92483168-92484840
21898	MMT00050545		15+:90530472-90554430
21901	BC035537	212326	8-:46424429-46444058
21903	Nr1d1	217166	11-:98629245-98636136
21907	Dynlrb1	67068	2+:155062313-155076009
21909	Neurl2	415115	2-:164656232-164658966
21912	OTTMUSG00000001(	629754	2-:164475133-164477308
21914	C030037F17Rik	77475	8-:14745327-14747430
21915	MMT00006856		13+:33810144-33811373
21916	Cdk4	12567	10+:126501291-126504076
21917	Taf9b	407786	X-:103403659-103415105
21918	Snx24	69226	18+:53405422-53550574
21919	MMT00078220		5+:3090867-3092385
21920	Fnip2	329679	3-:79263609-79371602
21922	MMT00028085		2+:5520620-5664750
21923	Olfr585	259091	7+:110246256-110247212
21924	Ang2	11731	14-:51815160-51815598
21926	MMT00059984		6+:145214919-145215296
21929	Vldlr	22359	19+:27291921-27324109
21931	Nrxn3	18191	12+:90741461-90771454
21934	Id2	15902	12-:25780183-25780875
21935	Prok2	50501	6-:99661293-99676386
21937	MMT00015325		17+:41238748-41238994
21941	MMT00028347		1+:125653267-125654326
21946	MMT00009539		11+:28855319-28857605
21947	Cdca8	52276	4-:124595709-124614153
21951	Cypt6	66742	X+:16099997-16100809
21952	Tfb1m	224481	17-:3519258-3557713
21953	Colec12	140792	18+:9707674-9877990
21955	Atxn7l1	380753	12+:34000322-34053138
21960	Acaca	107476	11+:84045175-84155449
21963	Edaradd	171211	13-:12569967-12612705
21964	Vwce	71768	19+:10708720-10716232
21965	AY036118	170798	17-:39985039-39985774
21966	Pcp4l1	66425	1-:173103394-173126371

21967	Tyk2	54721	9-:20908518-20935654
21969	Tk1	21877	11-:117676840-117687267
21970	Eif3f	66085	7+:116077929-116085201
21971	Epb4.2	13828	2-:120843953-120862491
21973	Faf1	14084	4+:109349300-109634651
21974	Ifltd1	74071	6-:145345754-145383445
21975	MMT00008417		2+:130935301-130935523
21978	Txnl1	53382	18-:63823755-63851842
21981	Ormdl2	66844	10-:128255550-128258662
21983	C130022K22Rik	232236	6+:91828061-91849832
21986	Cstf1	67337	2+:172196502-172206585
21988	MMT00015654	675749	7-:3813573-3820818
21989	Zfp167	382118	9+:122795968-122805240
21991	Olfr935	258741	9-:38802093-38803018
21992	Lap3	66988	5+:45884651-45903688
21993	D430015B01Rik	58909	6-:58885369-58953507
21995	Nktr	18087	9+:121628293-121665959
21996	Mme	17380	3+:63104056-63184253
21997	Phf1	21652	17+:27070276-27074834
21998	Nomo1	211548	7+:53289087-53339752
22001	Gtlf3b	24083	11-:60716562-60727291
22004	Gtpbp2	56055	17+:46298084-46306316
22008	Rbm35a	207920	4-:11259082-11313583
22009	Tmem14a	75712	1+:21208742-21220245
22010	Ccdc41	77048	10+:94151398-94252790
22012	Itgae	16407	11+:72904084-72960942
22015	2410127E18Rik	76788	6+:30367033-30402332
22019	Chrng	11449	1+:89102388-89108208
22021	Csnk1d	104318	11-:120824737-120852633
22024	Lrrc58	320184	16+:37878372-37884355
22026	Fgfbp1	14181	5-:44370433-44371187
22030	Mtmr6	219135	14+:60884107-60920502
22031	Vcam1	22329	3-:115813057-115832342
22032	Kirrel3	67703	9+:34293797-34318199
22034	Appl1	72993	14-:27736760-27783697
22035	Olfr142	406186	2-:90092226-90093143
22037	Oas1g	23960	5-:121326732-121337591
22038	Tnip1	57783	11-:54724290-54776414
22039	Npc2	67963	12-:86097356-86113848
22042	Mgam	232714	6+:40594829-40719115
22043	Rdh8	235033	9+:20622947-20629987
22046	Cml4	68396	6-:85780375-85781829
22050	Vamp5	53620	6-:72318128-72318637
22062	B230110C06Rik	77981	14+:16270133-16284959
22073	Lrig3	320398	10+:125403204-125452414
22075	C1s	50908	6-:124480644-124483991

22076	Pgm2l1	70974	7+:107376115-107427381
22077	MMT00057550	677168	17-:46543911-46544526
22079	Tmem81	74626	1+:134402806-134405214
22080	MMT00030613		X+:72153071-72163420
22083	4933413J09Rik	71106	14+:27182114-27197404
22084	MMT00070787		9+:122656463-122659550
22085	MMT00036089		6-:122741561-122743432
22087	Slc30a7	66500	3-:115649581-115710132
22089	Niban	63913	1+:153418669-153566166
22090	Plscr1	22038	9+:92157864-92166813
22091	Nt5c3l	68106	11-:100284062-100303122
22092	Plekhg4	102075	8+:107898800-107905956
22093	Zbbx	213234	3-:74841831-74916146
22094	Rnf149	67702	1-:39608137-39634253
22095	Mgst1	56615	6+:138096210-138104897
22098	MMT00009248		7-:104740367-104740914
22099	Cnot8	69125	11-:57910274-57911351
22100	Lcn5	13863	2+:25513587-25517164
22103	Hvcn1	74096	5+:122660295-122692282
22104	Rnf123	84585	9-:107964083-107964976
22106	Mmp15	17388	8+:97876763-97897770
22111	Ttn	22138	2-:76677461-76679353
22112	Xlr4a	434794	X-:70319678-70326134
22115	Ccl5	20304	11-:83339282-83343991
22116	Fbxo42	213499	4+:140723457-140756725
22117	Slc4a11	269356	2-:130509849-130523193
22120	Ccna1	12427	3-:54849396-54858977
22122	MMT00042954		
22125	Gab1	14388	8-:83289721-83403525
22126	RP23-303F24.3	622459	11-:53596920-53672732
22130	Cks1b	54124	3-:89219394-89222213
22135	MMT00033583		14+:17779358-17779949
22136	Olfr701	66786	7+:113957657-113964223
22137	Nudt7	67528	8+:116657514-116676204
22138	Ang	11727	14+:51721078-51721516
22139	Stip1	20867	19-:7095197-7114433
22141	Rorb	225998	19-:19012131-19075607
22142	5830457O10Rik	214987	8-:109407764-109410858
22146	Aifm2	71361	10+:61195069-61202035
22147	Ap1s1	11769	5-:137510863-137521930
22148	MMT00035976		
22149	MMT00059396		3-:57521848-57522211
22150	Fhdc1	229474	3-:84247398-84278704
22151	Plxnd1	67784	6-:115904826-115944675
22152	5830411G16Rik	78937	6+:56664918-56709117
22157	Mxra8	74761	4+:155213905-155218202

22161	Tmem132a	98170	19-:10932322-10944221
22164	Msrb3	320183	10-:120220946-120308216
22165	St8sia4	20452	1-:97484260-97564171
22166	Traf1	22029	2-:34798775-34817105
22169	Ctrl	109660	8-:108455902-108457762
22171	MMT00012636	100042265	9-:121758658-121766649
22180	Nlrp9a	233001	7+:27342304-27359163
22184	MMT00033856		11+:83614245-83615066
22188	Top2a	21973	11-:98855077-98885429
22191	Dhrs9	241452	2+:69230966-69235830
22192	9030205A07Rik	217843	12+:104301314-104422208
22193	MMT00007348		4+:14097146-14098124
22194	Olfr39	258822	9+:20090144-20091079
22196	Cyp3a41a	53973	5-:146529490-146531712
22197	Ankrd28	105522	14-:32511959-32643590
22199	Acsm5	272428	7+:126669776-126687306
22201	Pard3	93742	8+:129588144-130134729
22202	OTTMUSG00000021	545539	3+:89196186-89202147
22203	Txndc11	106200	16-:11075006-11088024
22204	Slc6a20a	102680	9-:123545239-123587963
22208	Me3	109264	7+:96781327-97000472
22213	Adora1	11539	1-:136097952-136131999
22215	A630047E20Rik	271981	3+:132349787-132501573
22216	Sp5	64406	2+:70313222-70315753
22217	Flvcr2	217721	12+:87087488-87154535
22218	Rfc3	69263	5-:152445392-152453770
22219	Cyp11b1	110115	15-:74665322-74672036
22222	Gstt1	14871	10-:75246784-75261241
22224	Svs1	243377	6+:48936900-48940688
22226	Pm20d1	212933	1+:133701200-133714693
22228	MMT00013109		
22229	Dnmt1	13433	9-:20711964-20757423
22230	Myh9	17886	15-:77591018-77672529
22232	Krt19	16669	11-:100002128-100007228
22238	Sardh	192166	2-:27044902-27102332
22239	Ap3b1	11774	13+:95128985-95336270
22241	Sfrs10	20462	16-:22246535-22265983
22244	Slc16a7	20503	10-:124664541-124765591
22247	Pigyl	66268	9+:21961309-21962792
22250	Wdr45l	66840	11-:121188547-121215723
22255	2900010M23Rik	67267	17-:27259609-27270836
22256	Arg2	11847	12+:80231810-80257282
22257	Lonrf3	74365	X+:33868401-33898750
22258	Ttk	22137	9+:83728347-83765991
22260	Slc38a4	69354	15-:96827200-96861642
22263	Gzmd	14941	14-:56748417-56751359

22271	Cyp2c29	13095	19+:39361600-39405189
22272	Vpreb1	22362	16-:16868495-16869343
22273	Zfp146	26465	7-:30946619-30954706
22279	Krr1	52705	10+:111409810-111425943
22284	Tcerg1l	70571	7-:145401374-145589241
22286	Mmachc	67096	4-:116375039-116380926
22288	MMT00061717		
22289	Smn1	20595	13+:100894848-100907645
22290	Tubb2a	22151	13-:34166150-34169875
22291	V1re7	171230	17-:21026681-21027615
22293	Gprc6a	210198	10-:51334671-51348358
22294	Ttc35	66736	15+:43308820-43359308
22297	Zfyve1	217695	12-:84887504-84916086
22298	Zfp259	22687	9+:46081248-46089685
22302	MMT00041810		8-:38434879-38436192
22303	Cdt1	67177	8+:125091941-125097033
22304	Pigt	78928	2+:164323064-164333266
22305	Mcph1	244329	8+:18595272-18625674
22306	Myl4	17896	11+:104438765-104447067
22307	Sgtb	218544	13+:104899869-104931519
22310	Asb5	76294	8+:55635683-55673189
22312	Itln1	16429	1-:173448372-173461884
22314	E2f2	242705	4+:135728307-135751074
22315	Myo3b	329421	2+:69934353-70072535
22318	Cox8b	12869	7-:148084841-148086296
22320	MMT00047284		
22321	Apol7a	75761	15-:77218645-77226747
22322	Rnf151	67504	17-:24852790-24854963
22323	MMT00051740		7-:17304466-17305259
22324	MMT00060741		7-:109796211-109796566
22327	Tyrobp	22177	7+:31198857-31202427
22329	Galntl5	67909	5+:24687297-24726107
22333	Hnrnph3	432467	10-:62477414-62482275
22334	9430023L20Rik	68118	15+:101114731-101121365
22336	Hsd3b5	15496	3-:98422558-98434175
22337	Nlk	18099	11-:78381693-78510055
22338	Unc119	22248	11+:78157006-78162544
22339	Slc12a1	20495	2+:124979465-125021457
22340	Lnx2	140887	5+:147828219-147830167
22342	MMT00008396		5-:10241751-10245340
22343	Blm	12144	7-:87599977-87679983
22349	Cab39	12283	1+:87690359-87746617
22351	Fanca	14087	8-:125792201-125842476
22354	2010301N04Rik	72103	6-:87657235-87672165
22355	Clec4e	56619	6-:123231806-123239889
22358	MMT00038532		

22361	Sephs2	20768	7-:134415396-134417560
22362	Laptm4b	114128	15+:34168078-34213134
22364	Cnbp2	75064	X+:100818852-100826524
22365	Jun	16476	4-:94715745-94718878
22366	MMT00005283		6+:16838966-16876948
22370	1700058G18Rik	67342	10+:115855176-115858291
22371	1810063B05Rik	67892	8-:128948313-128999049
22372	MMT00030553	627908	X+:72478193-72481092
22373	Txnip	56338	3+:96361880-96365780
22375	MMT00053882		14+:95818909-95820406
22383	2200002K05Rik	69137	9+:15043486-15063837
22385	Olfr1437	258117	19-:12284792-12397072
22388	2410018M08Rik	71970	5+:130371591-130379486
22390	Dhcr7	13360	7+:151016137-151034314
22395	Kcna1	16485	6-:126591689-126594831
22396	Fcrl5	329693	3+:87245926-87252359
22398	Nav2	78286	7+:56708439-56862513
22402	MMT00071327		8-:11683460-11684248
22403	Bank1	242248	3-:135717373-135763554
22406	Vnn1	22361	10+:23614665-23625145
22407	Rhbdf2	217344	11-:116459485-116485567
22408	Calr3	73316	8-:74948450-74967677
22409	Larp2	214048	3+:40766089-40781704
22411	Reck	53614	4+:43888471-43957392
22412	Ptpn5	19259	7-:54333171-54389054
22427	MMT00049287		X-:62003662-62004338
22430	Taar9	503558	10-:23821182-23829340
22434	Olfr1255	258979	2+:89656484-89657415
22435	Tmem98	103743	11+:80623916-80635533
22439	4921533L14Rik	68187	1-:24018521-24036655
22440	MMT00042948		8+:82229577-82230204
22443	Tuba1c	22146	15+:98860808-98868535
22445	Prkag3	241113	1-:74785498-74795530
22446	Ccr5	12774	9+:124039100-124040164
22448	MMT00023461		3+:19834373-19834864
22450	Prpf19	28000	19+:10969753-10979806
22451	Olfr1461	258299	19+:13232689-13308799
22452	Tcfap2b	21419	1+:19199078-19224593
22453	Spag17	74362	3+:99892204-99896975
22454	Synj2bp	24071	12-:82601700-82633855
22455	Gprc5d	93746	6-:135056009-135068301
22457	6530401D17Rik	76219	X+:132567905-132569483
22458	Spata17	74717	1-:188872280-189039346
22460	4930430F21Rik	73784	15+:30968876-30973087
22462	MMT00026054	100041835	11-:95934335-95936342
22463	Gorasp2	70231	2+:70499206-70529799

22465	Olfr790	258935	10+:128937941-128938909
22470	Ppargc1b	170826	18-:61457790-61560085
22474	Ubqln1	56085	13-:58279144-58316799
22475	Sh3bp1	20401	15+:78732786-78742473
22476	Tspan4	64540	7+:148661375-148679315
22478	Tanc1	66860	2+:59537404-59684269
22479	Ascc2	75452	11+:4537795-4583386
22482	Vmn2r93	627132	17+:18440927-18462865
22486	Sh3bgrl3	73723	4-:133683321-133684668
22487	Trub1	72133	19+:57527401-57563395
22492	Ubb	22187	11+:62365007-62366714
22493	Slc35a2	22232	X+:7461369-7471531
22494	MMT00005811		4-:52948302-52949253
22495	Nek5	330721	8-:23184088-23193936
22496	Slfn1	20555	11+:82930346-82936161
22499	9030409G11Rik	71529	4-:141696730-141766123
22503	Cnot6l	231464	5-:96505816-96591009
22506	Slc22a4	30805	11-:53796811-53841590
22507	Nap1l3	54561	X-:119508169-119510994
22508	Setd8	67956	5+:124910928-124912282
22514	MMT00076032	667804	2+:74590836-74591356
22519	1700054K19Rik	67336	6+:112160699-112162507
22520	Sec13	110379	6-:113678063-113690678
22521	Hsp90b1	22027	10-:86153585-86168189
22522	2010300C02Rik	72097	1-:37694830-37733634
22528	Frmd5	228564	2-:121373822-121412207
22531	Gm484	243967	7+:52941550-52949922
22534	Pik3r2	18709	8-:73292077-73300580
22539	MMT00044599		4+:52049043-52254398
22541	4632413I24Rik	74031	10+:7132928-7133737
22546	Tnnt1	21955	7-:4456264-4467577
22551	Anapc11	66156	11+:120459787-120466988
22553	Ms4a8a	64381	19-:11141961-11155518
22555	Ela2	50701	10+:79349079-79350843
22556	Slc26a8	224661	17-:28786612-28826932
22557	P4hb	18453	11-:120421355-120434500
22559	MMT00031309		2+:34686022-34688171
22560	Gpr56	14766	8+:97501008-97538095
22561	Whdc1	434204	7+:88716165-88741478
22562	Sdha	66945	13-:74459703-74487690
22563	MMT00004885		4+:105466782-105467514
22568	MMT00033197		12-:105427732-105430646
22570	Phtf1	18685	3+:103772180-103811411
22573	Kif2c	73804	4-:116832742-116855189
22575	St8sia6	241230	2-:13576566-13715147
22578	Bmp5	12160	9+:75623899-75746389

22579	Eif4a2	13682	16+:23107828-23114019
22580	MMT00081414		2+:15766872-15791436
22581	2310035C23Rik	227446	1+:107560445-107651761
22583	Vmn2r89	22301	14+:52030599-52080294
22584	Gspt2	14853	X+:91881657-91883890
22592	MMT00055028		12+:100742958-100747794
22593	Spink3	20730	18-:43887737-43896848
22598	4930402H24Rik	228602	2-:130629128-130629599
22600	2410018C20Rik	67873	8-:86775381-86781191
22604	Zc3h6	78751	2+:128793144-128819866
22607	Bex1	19716	X-:132748511-132749977
22611	Tmem5	216395	10-:121516443-121517069
22612	1500010J02Rik	68964	11+:68840817-68849974
22618	Ccl21c	65956	4+:41774242-41775336
22621	Cklf	75458	8+:106774802-106787343
22622	Prl6a1	19111	13+:27404496-27411120
22624	Kcnj15	16516	16+:95517208-95518605
22627	Adck5	268822	15+:76406735-76426238
22628	EG626903	626903	5-:20621883-20635619
22629	Asb17	66772	3+:153507229-153516575
22630	MMT00034355		4+:24165577-24166314
22631	MMT00054932		11+:101337214-101338381
22635	Nrn1	68404	13-:36817495-36826349
22636	Trim2	80890	3-:83964367-84024783
22640	Bud13	215051	9+:46091104-46106864
22641	Kif1c	16562	11+:70514046-70545471
22642	Uts2	24111	4+:150371205-150375918
22643	Ece1	230857	4+:137469486-137519654
22645	Zar1	317755	5-:72968354-72972376
22647	Pkib	18768	10+:57351786-57456887
22648	Mpzl1	68481	1-:167522314-167564652
22650	Ccdc129	232016	6+:55925581-55928732
22653	Ric3	320360	7-:116181406-116226820
22655	Sorcs3	66673	19+:48678332-48879987
22656	Atp6ap1	54411	X+:71542517-71549477
22660	Kif4	16571	X+:97821075-97922278
22662	H2-T24	15042	17-:36143599-36157436
22668	Rai14	75646	15-:10500395-10620182
22669	Ahctf1	226747	1-:181675029-181684203
22670	Clec4a1	269799	6+:122871991-122884638
22672	Adam33	110751	2-:130876326-130882277
22673	Prpf38b	66921	3-:108705236-108714643
22676	9030420J04Rik	71544	9-:9180015-9239026
22677	Prss2	22072	6+:41471786-41475078
22681	Gm88	224813	17+:46390464-46394264
22684	Slco1a5	108096	6-:142182750-142271470

22688	4930432K21Rik	74666	8+:86672148-86696488
22689	2610028A01Rik	72400	14+:64479200-64538641
22691	Ercc6l	236930	X-:99338296-99339789
22693	Trim68	101700	7-:109826100-109835764
22694	EG629219	629219	7-:14807826-14840218
22695	Tgoln2	22135	6-:72560950-72566742
22696	Ypel4	241525	2+:84574165-84578798
22697	Skil	20482	3+:30995754-31017908
22698	Olfr218	258880	1+:175133488-175134428
22703	Olfr599	258726	7+:110486569-110487515
22705	Actr2	66713	11-:19962308-20012916
22709	Proc	19123	18-:32282780-32295593
22710	MMT00075201		12+:36499659-36501226
22712	Phf11	219131	14-:59895750-59916356
22715	Ap3m1	55946	14-:21855721-21871402
22716	Agr3	403205	12+:36652207-36676323
22717	EG620161	620161	6+:112926194-112926462
22718	1700093K21Rik	67358	11-:23416248-23419101
22722	Ttc14	67120	3+:33700135-33706871
22724	Adamts13	279028	2+:26864045-26864967
22727	Pdgfra	18595	5+:75548368-75594220
22730	Glt8d1	76485	14+:31814891-31825171
22732	Cyb5b	66427	8+:109674576-109708000
22733	Alpk3	116904	7+:88202485-88250497
22734	Ap3s2	11778	7-:87025043-87065481
22735	MMT00007455		14-:109091411-109091860
22740	Manbal	69161	2+:157204819-157222498
22743	Igh-2	238447	12-:114496927-116384147
22744	9-Sep	53860	11+:117193033-117223622
22747	Gpc3	14734	X-:49625605-49967091
22750	Mupcdh	72040	7-:148454983-148462673
22751	Nosip	66394	7+:52317862-52332786
22752	MMT00001763		4+:38610921-38611781
22753	Sidt2	214597	9-:45745944-45763804
22758	2810003C17Rik	108897	2+:31805822-31828961
22759	Btbd10	68815	7-:120459131-120513055
22760	Rlbp1l1	74438	4+:9198142-9377045
22761	Cep350	74081	1-:157787848-157820383
22762	Slc6a4	15567	11+:76812098-76845843
22763	Alcam	11658	16-:52249106-52310062
22764	MMT00049130		12+:60393050-60393923
22767	Nutf2	68051	8+:108384552-108403236
22770	Slc27a2	26458	2+:126378890-126413671
22771	Dhx37	208144	5-:125894230-125895773
22772	Gm732	213450	X-:105141079-105143760
22776	Gsr	14782	8+:34764011-34807908

22777	Duox2	214593	2-:122106173-122123963
22779	Pik3cg	30955	12-:32860500-32893335
22781	Hibch	227095	1+:52901889-52977704
22782	Frrs1	20321	3+:116564609-116606559
22785	MMT00078758		
22786	Mterfd3	74238	10-:84582176-84590769
22788	MMT00013067		11-:92567035-92654529
22793	Cbr2	12409	11-:120590822-120593309
22802	Svs2	53878	2-:164062595-164063979
22804	Omt2a	18379	9-:78159809-78160890
22806	6330406I15Rik	70717	5+:150214382-150234277
22807	Mboat2	67216	12+:25516479-25644558
22809	Mrvi1	17540	7-:118014872-118089558
22810	MMT00038169		X+:119214523-119216090
22814	Cacna1s	12292	1+:137949360-138016105
22816	Arl4c	320982	1-:90597661-90619274
22821	Tmem135	72759	7-:96288714-96487270
22822	MMT00013433		19+:13866454-13867106
22823	Sh2d1a	20400	X+:39855846-39875258
22825	Olfr1316	258737	2-:111970029-111970972
22826	Alox15	11687	11-:70158044-70165507
22827	Clec4a2	26888	6+:123073333-123092947
22829	Sval2	84543	6+:41810337-41814320
22830	MMT00081088		10+:57106425-57107621
22833	Acat2	110460	17-:13135760-13153593
22835	Fbln1	14114	15+:85036440-85116084
22836	Grpr	14829	X-:159951835-159987571
22841	Cib1	23991	7-:87372251-87377446
22852	Nepn	66650	10+:52111413-52124410
22854	Defb35	246084	8+:23048823-23051350
22856	Pak2	224105	16-:31977031-32079410
22857	Fahd2a	68126	2-:127261951-127270277
22858	Fbxl17	50758	17-:63706093-63840237
22860	Dtx2	74198	5+:136470746-136508734
22861	E430002G05Rik	210622	2+:102390172-102483195
22863	Prkd2	101540	7+:17428328-17455812
22867	Vps52	224705	17+:34102597-34103979
22868	Krt15	16665	11-:99993332-99997172
22869	Tceal8	66684	X-:132704872-132706725
22871	Pgrmc2	70804	3-:40870260-40886895
22873	Slc38a2	67760	15-:96517829-96530140
22876	Sash3	74131	X+:45499705-45514740
22880	Slc45a4	106068	15-:73410714-73436109
22885	Pank1	75735	19-:34885384-34952407
22889	Foxj3	230700	4+:119213134-119300547
22890	D1Pas1	110957	1+:188791754-188793736

22894	MMT00016059		6-:28311913-28313176
22900	Olfr419	258710	1-:176180116-176181056
22901	7-Mar	57438	2+:60047992-60086441
22903	Mospd2	76763	X-:161374111-161418262
22907	MMT00054594		6-:145264134-145265169
22909	Nup133	234865	8-:126423197-126473130
22912	Stap1	56792	5+:86500862-86532832
22914	EG626280	626280	15+:76188886-76194156
22918	MMT00002192		12+:58815050-58838891
22919	Lig1	16881	7+:13862631-13896740
22921	2810038D20Rik	67218	14+:45202804-45208076
22922	2310010M24Rik	71897	2+:49643207-49804366
22923	Zfml	18139	6+:83864360-83936855
22924	Midn	59090	10+:79611034-79621112
22925	Parp16	214424	9+:65062928-65087021
22926	Ergic2	67456	6-:148128566-148160559
22927	1700001L05Rik	69291	15-:83187963-83197643
22930	Mro	71263	18+:74019046-74040987
22933	Tssc4	56844	7+:150255272-150256992
22934	4932425I24Rik	320214	16-:38297839-38341914
22936	Rhobtb1	69288	10+:68675916-68752765
22939	C1galt1	94192	6+:7795263-7821454
22943	Tpd52l1	21987	10-:31052185-31165727
22944	Tmem44	224090	16-:30513777-30550621
22945	Cpb2	56373	14+:75642147-75683358
22946	MMT00071077		10+:41023784-41077470
22947	Zfp358	140482	8+:3493170-3497208
22948	MMT00069572		14-:57455124-57455950
22952	Dppa1	347708	11-:46421955-46442779
22953	Mvp	78388	7-:134130381-134158106
22954	Slc25a30	67554	14-:76161805-76186844
22955	4930430E16Rik	73873	11+:22908372-22921138
22957	1300018I05Rik	74157	17+:29797639-29839630
22959	Zfp174	385674	16+:3847872-3854811
22960	Olfr714	259035	7+:114217343-114218297
22961	MMT00055950	665565	15+:59070014-59070558
22962	MMT00013609		4+:101523329-101548604
22963	Ctso	229445	3+:81736513-81756214
22964	C9	12279	15+:6395332-6448476
22966	Epha4	13838	1-:77365846-77511663
22969	Axin2	12006	11+:108781797-108810797
22970	D17Ertd663e	52042	17-:13012718-13014031
22972	9030617O03Rik	217830	12+:102024351-102110558
22973	MMT00067195		17+:17785784-17786290
22978	MMT00043176		5+:106644617-106645918
22981	Bcar3	29815	3+:122122744-122233098

22982	Mug2	17837	6+:121956815-122035983
22984	Tnnt2	21956	1+:137732981-137748837
22987	Eif2s2	67204	2-:154698172-154718511
22988	Hscb	100900	5-:111258092-111265448
22989	Lmcd1	30937	6+:112223811-112280418
22990	Rps2	16898	17+:24857057-24858853
22992	A430107O13Rik	214642	6+:22038688-22187742
22994	Cpne3	70568	4-:19446401-19497257
22995	Drd2	13489	9+:49148731-49216282
22996	MMT00076852		14-:45341673-45344626
22998	Ass1	11898	2+:31325821-31376149
23000	Faim2	72393	15-:99330579-99358025
23001	Galm	319625	17+:80526793-80584441
23003	EG209550	209550	12+:11462884-11469489
23005	4930405A21Rik	74846	2-:156540273-156542803
23006	Try4	22074	6+:41252283-41255485
23007	2410015N17Rik	66422	7-:134400475-134404181
23008	Ctsz	64138	2-:174252995-174264503
23010	MMT00047055		7-:14547637-14551245
23011	MMT00012889		13+:15906733-15907217
23012	Spg20	229285	3+:54916061-54939876
23014	MMT00077643		
23019	ENSMUSG00000063(	791318	18-:5574983-5583802
23022	Kif3a	16568	11+:53380894-53414604
23025	Foxi2	270004	7+:142602121-142603714
23026	Apom	55938	17-:35265942-35268697
23029	Zfp27	22689	7-:30629768-30728569
23032	Mett10d	67493	11+:74584364-74632194
23034	Slc25a29	214663	12-:110064086-110073959
23037	Thbs3	21827	3+:89019128-89030738
23038	Casp4	12363	9+:5308873-5336707
23044	Ctr9	22083	7+:118172534-118199850
23049	4933403O08Rik	71030	X+:109352657-109357462
23052	EG634331	634331	1+:99059343-99190012
23053	1700012B09Rik	69325	9-:14562643-14575474
23055	Lmo1	109594	7-:116282084-116314027
23056	Ccdc135	330830	8+:97579794-97602039
23059	Fbxl20	72194	11-:97943868-97945481
23060	8430422H06Rik	74529	18+:14390811-14437050
23061	Pole2	18974	12-:70302763-70329160
23064	Myh2	17882	11+:66984720-67011014
23066	Gtf2a1	83602	12-:92798108-92828019
23070	BC024659	108934	13+:41345546-41368403
23073	4833414E09Rik	71633	1-:170349770-170350960
23076	Kcnc2	268345	10+:111708177-111709460
23077	Daam2	76441	17-:49597765-49638260

23079	Spag17	74362	3+:99689353-99867396
23082	Gria3	53623	X+:38754687-39029736
23084	Vsig4	278180	X-:93442546-93488775
23086	MMT00059956		
23087	Nusap1	108907	2+:119444452-119475512
23088	1700009N14Rik	75471	4+:39397828-39398478
23089	Slc1a2	20511	2+:102546594-102618263
23092	Ifih1	71586	2-:62435396-62484223
23094	Sap30	60406	8-:59961523-59966654
23097	Pip4k2c	117150	10-:126635144-126648613
23101	Pnliprp1	18946	19+:58803376-58818659
23102	Fbxo7	69754	10+:85484672-85511072
23104	Gp2	67133	7-:126586057-126602786
23108	Map4k3	225028	17-:80979851-81127176
23109	A430088P11Rik	106059	15-:80526972-80528033
23111	Eml5	319670	12-:100101306-100139238
23112	Gys1	14936	7+:52690358-52711978
23115	Mad2l1	56150	6+:66485472-66489946
23116	2810032G03Rik	72669	12+:5382675-5423111
23118	Ttrap	56196	13+:24923553-24934015
23119	Cps1	227231	1+:67169601-67277833
23130	Hps4	192232	5+:112773971-112807434
23131	Pla1a	85031	16-:38396204-38432968
23132	Mcm6	17219	1-:130228167-130256233
23138	Osbpl6	99031	2+:76386396-76434101
23141	Ebf2	13592	14+:67853597-68048604
23149	Nln	75805	13-:104813136-104899682
23150	EG622657	622657	14+:34026276-34026984
23152	Kif27	75050	13+:58374894-58397948
23156	MMT00013465	433597	3+:59547517-59556206
23157	MMT00001961		18+:6516319-6557797
23159	Myh8	17885	11+:67090934-67122132
23161	Gclc	14629	9+:77602352-77642269
23162	Csda	56449	6-:131314875-131338449
23169	Pla2g2e	26970	4+:138433856-138438727
23172	Atp13a4	224079	16-:29396130-29544951
23173	Cuzd1	16433	7-:138452068-138465806
23174	Psme2	19188	14-:56206322-56209726
23175	Acsm4	233801	7+:126838122-126854941
23179	Olfr575	259118	7-:110103179-110104134
23180	Sult1d1	53315	5-:87984278-87997909
23182	Ace3	217246	11+:105856044-105865922
23183	Nnmt	18113	9-:48399981-48413258
23185	Ndufs1	227197	1-:63190171-63223283
23188	MMT00013081		3+:112822792-112826285
23189	Rbm28	68272	6-:29075020-29108200

23191	3632451O06Rik	67419	14-:50301234-50403003
23194	Rab3c	67295	13-:110852024-111070226
23201	S1pr1	13609	3-:115413356-115417963
23202	Olfr559	259116	7-:109872045-109873002
23207	Rab7	19349	6-:87949116-87995214
23208	Hck	15162	2+:152934322-152977162
23210	Tprgl	67808	4-:153531596-153534775
23211	Pscd3	19159	5+:144383421-144471109
23213	Txndc3	73412	13-:19780805-19788919
23214	2610019A05Rik	72149	11-:106024649-106054862
23215	Mphosph6	68533	8-:120315544-120325829
23217	Acyp2	75572	11-:30532202-30549408
23223	MMT00076151		19-:3730467-3731012
23224	C030014K22Rik	226610	1+:168931696-169009599
23226	Suox	211389	10-:128106948-128110938
23227	Grin2a	14811	16-:9577802-9992626
23229	Phf12	268448	11+:77796254-77844034
23232	Crisp3	11572	17-:40358727-40379215
23233	Npw	381073	17-:24794273-24795087
23240	Gtpbp10	207704	5-:5537463-5559535
23242	Gngt2	14710	11+:95703983-95707043
23245	Nfib	18028	4-:81941984-82151195
23246	Cited2	17684	10+:17443052-17444594
23247	Ercc1	13870	7+:19930454-19941871
23248	Tesk2	230661	4+:116393581-116476707
23250	Plcl2	224860	17+:50745613-50827811
23252	Snrpd1	20641	18+:10617864-10627968
23253	D030011O10Rik	320560	6-:148941534-149049831
23254	Wdtc1	230796	4-:132849494-132895173
23259	Dnajc1	13418	2-:18135381-18136521
23261	Clic6	209195	16+:92498379-92541482
23262	C530043G21Rik	215015	1-:158611184-158649130
23265	Tmem67	329795	4-:11966263-12015084
23267	Hsd17b11	114664	5-:104419413-104450797
23272	Orc1l	18392	4+:108252080-108287434
23274	1700054O13Rik	67334	X+:9424072-9424626
23276	Evi2b	216984	11-:79328524-79337196
23280	Dscam	13508	16-:96814550-97391987
23281	1700126L10Rik	73603	2-:164295801-164311797
23283	Mrps12	24030	7-:29524660-29526799
23287	Supt4h1	20922	11+:87551066-87557118
23288	Cramp1l	57354	17-:25148246-25150969
23290	MMT00061154		X+:99333976-99334474
23291	Rexo1	66932	10-:80005017-80009547
23295	Snx11	74479	11-:96629190-96638834
23301	Scamp1	107767	13-:94971265-95055382

23303	Olfr1302	258891	2+:111620469-111621415
23306	Bag2	213539	1-:33802330-33814593
23307	Myst2	217127	11-:95135573-95171492
23308	Dock9	105445	14-:121941369-121980657
23313	Htr1b	15551	9-:81524999-81526159
23318	Cul2	71745	18+:3383028-3436372
23324	Prdx6	11758	1-:163171140-163181296
23326	Il7r	16197	15-:9435915-9459544
23327	Brp44l	55951	17+:8476755-8490027
23328	Rora	19883	9+:68501608-69226833
23331	Csmd1	94109	8-:15895502-17535281
23332	Sos2	20663	12-:70684752-70759065
23334	Olfr692	258352	7+:112516841-112517829
23335	MMT00030313		9-:3495833-3496959
23339	Olfr871	258905	9+:20016794-20017729
23342	Ndfip2	76273	14+:105657864-105708513
23344	Nfkbiz	80859	16-:55811490-55838967
23345	MMT00076071		
23348	Calml4	75600	9+:62705893-62723722
23350	BC048546	232400	6-:128489837-128531614
23351	Zfp598	213753	17+:24806798-24818959
23352	MMT00001681		X+:14942325-14942614
23353	Slmo2	66390	2-:174290600-174298399
23354	Tshr	22095	12+:92639449-92777167
23355	Nedd1	17997	10-:92147490-92185105
23356	Gpr98	110789	13-:81644732-81723855
23360	MMT00066283		5-:52783909-52830585
23361	Bivm	246229	1+:44176854-44201592
23367	Tm2d2	69742	8+:26127751-26133731
23368	6330503K22Rik	101565	7+:125858887-125880534
23369	Oaz2	18247	9+:65524399-65537263
23370	Cpeb4	67579	11+:31820408-31832197
23371	Cfb	14962	17-:34993326-34999162
23372	Rbm5	83486	9-:107642825-107673314
23373	Vps13d	230895	4-:144753078-144784961
23375	MMT00055444		5-:100268536-100269009
23377	Pdxk	216134	10-:77903349-77927724
23383	Setd7	73251	3-:51320071-51322248
23388	Olfr561	259096	7+:109923039-109923984
23391	MMT00008521		8-:48712340-48713397
23394	Psd4	215632	2+:24223102-24264686
23395	MMT00061495		4+:144381739-144382372
23397	Ccnjl	380694	11+:43342856-43399806
23398	Adamts5	23794	16-:85896279-85900512
23400	BC024997	215723	11+:68369687-68371746
23401	H2-Q2	15013	17+:35479254-35496927

23403	Ndc80	67052	17-:71845444-71876197
23410	C1qa	12259	4-:136451831-136454675
23412	EG625690	625690	10-:84256896-84257449
23414	Tnfaip8l2	69769	3-:94943442-94946282
23415	Acp5	11433	9-:21931193-21935098
23416	ENSMUSG000000713	628709	13-:65498322-66077477
23419	Tipin	66131	9+:64129413-64152599
23425	Tob1	22057	11+:94074953-94076040
23427	MMT00054636	654456	8+:19300676-19300738
23430	Aifm3	72168	16+:17489783-17507574
23437	Efhd2	27984	4-:141414303-141430824
23438	C1galt1c1	59048	X-:35983961-35988230
23440	EG243642	243642	6+:127176793-127184635
23445	Crlf1	12931	8+:73017056-73027980
23446	Acsl6	216739	11+:54117703-54175026
23449	Prelid2	77619	18-:42092246-42110876
23450	D12Ertd647e	52668	12+:104672413-104678454
23453	Dnajc3a	100037258	14+:119337211-119377404
23454	Znhit1	70103	5-:137458062-137462879
23455	Ptafr	19204	4+:132120002-132136239
23457	Selenbp2	20342	3+:94497494-94508328
23458	Clic4	29876	4-:134769886-134828675
23460	Rragd	52187	4+:33070410-33109147
23462	Bag5	70369	12-:112947703-112951505
23463	Gimap1	16205	6+:48692180-48693793
23465	Afp	11576	5+:90919765-90937882
23466	Pou2f1	18986	1-:167805606-167932738
23467	Amac1	56293	11-:69573410-69575346
23470	Tmod1	21916	4+:46073837-46127676
23471	Ddx46	212880	13+:55754271-55780492
23472	Cts3	117066	13-:61589372-61669779
23474	MMT00011721		2+:83819001-83822053
23475	Nudt12	67993	17-:59140823-59152745
23476	Ppp1r9a	243725	6+:4995935-5110715
23478	Sacs	50720	14+:61821599-61833091
23482	Syt4	20983	18-:31598839-31606860
23485	Olfr716	258597	7+:114290831-114291775
23487	Polb	18970	8-:23738737-23763886
23489	Tbc1d15	66687	10-:114635514-114688481
23490	Fndc3b	72007	3-:27315076-27362768
23493	Tgs1	116940	4+:3502172-3542151
23495	Cryab	12955	9+:50560862-50564738
23496	Zfp40	22700	17-:23311845-23315506
23498	2010204N08Rik	69983	3-:72692485-72704389
23499	Edil3	13612	13+:89429023-89461489
23500	BC055004	381680	5+:138671109-138693280

23508	Pdgfd	71785	9+:6288469-6379261
23522	Adcy10	271639	1+:167415311-167506900
23523	MMT00039380		
23527	MMT00021936		5-:30133239-30134698
23528	0610038L08Rik	74092	17-:29715751-29733251
23530	Drd4	13491	7+:148477936-148480759
23531	Foxb2	14240	19-:16946806-16948320
23533	Ccnb1ip1	239083	14-:51408923-51413549
23541	MMT00002245		2-:110049431-110050374
23543	Rufy3	52822	5+:89012560-89070631
23544	Atg4c	242557	4+:98884666-98925779
23546	Zzef1	195018	11+:72680105-72683429
23547	Tmed7	66676	18-:46745311-46757190
23548	Crtac1	72832	19-:42358019-42506123
23549	Es22	13897	8-:95725117-95753440
23550	Ap2m1	11773	16+:20535576-20544125
23552	Rasl11b	68939	5+:74591259-74595495
23553	Pcnp	76302	16-:56017305-56029779
23555	BC026585	226527	1+:159388863-159419213
23557	Ppp1r12c	232807	7-:4433115-4453624
23558	MMT00030937		15+:18409242-18409510
23561	Sox14	20669	9-:99775382-99776103
23563	MMT00004841		11-:17873743-17874841
23567	Tom1l1	71943	11-:90506634-90548908
23569	Atp5s	68055	12+:70825948-70845645
23572	4631426J05Rik	77590	7-:139428793-139508792
23576	Fabp9	21884	3-:10193625-10197222
23578	Cyp2j5	13109	4-:96295837-96330742
23579	Gm98	225908	19-:10282759-10315241
23580	BC059842	230676	4-:118064321-118070324
23584	Adamts5	23794	16-:85862668-85900529
23588	Anks1b	77531	10+:90411276-90434735
23590	Ptgds	19215	2-:25322221-25325546
23591	Dlg4	13385	11+:69832365-69858270
23595	Gm550	225852	19+:6095867-6098245
23597	Pcdha3	192163	18+:37105860-37347311
23599	Ndrg3	29812	2-:156753082-156817761
23602	Sec14l4	103655	11+:3931784-3948010
23604	Ephb3	13845	16+:21204825-21223375
23605	MMT00077586		18-:90491386-90492213
23606	Cmtm8	70031	9-:114698462-114753270
23607	MMT00040265		12+:71081867-71083890
23609	Trim44	80985	2-:102144500-102241004
23610	Prkaa2	108079	4-:104706216-104782403
23613	Jak2	16452	19+:29326944-29386472
23614	G630016D24Rik	619301	14+:52632591-52640170

23615	MMT00005149		6-:23105933-23106684
23617	Nme1	18102	11-:93820339-93822103
23623	2010305C02Rik	380712	11+:75281573-75284400
23625	Pkd2	18764	5+:104888655-104932705
23626	MMT00003764		14+:75117517-75118640
23631	MMT00039126		8+:99688153-99688772
23636	Bst2	69550	8-:74058160-74061335
23638	Ddr1	12305	17-:35818512-35840932
23640	Bdh1	71911	16+:31422384-31458980
23643	Olfr482	258728	7-:115238112-115239082
23645	1700041C02Rik	73332	4-:119006184-119056366
23646	Hsp90ab1	15516	17-:45704723-45710209
23648	Pigy	66459	6-:57639344-57642071
23649	BC049635	277773	4-:42880876-42884992
23651	MMT00027295		18-:24212752-24262836
23653	Zfp407	240476	18-:84378134-84381307
23659	EG633250	633250	13+:62006047-62212827
23660	Dpyd	99586	3+:118702087-119134638
23662	Serpinb2	18788	1+:109412049-109422175
23663	Dab2ip	69601	2+:35477679-35586512
23664	Btg1	12226	10+:96079538-96082262
23668	Gse1	382034	8+:123012901-123105248
23679	Ccl20	20297	1+:83113356-83115741
23680	5730419I09Rik	74741	6-:142959447-143048638
23681	Whrn	73750	4-:63075946-63156610
23682	Trim8	93679	19+:46576788-46590839
23683	Dnahc7c	100101919	1+:46706054-46864976
23685	Tbxas1	21391	6+:38868973-39034559
23690	Mug1	17836	6+:121788628-121839074
23691	Sh3d19	27059	3+:85844766-85932398
23693	A530016L24Rik	319942	12+:113733198-113736602
23695	1700081L11Rik	76719	11-:104229987-104287786
23697	Rrp1b	72462	17+:32173045-32197674

Table T4: Adipose Modules

Module	Locus Link	Gene Symbol
1	73205	3110043O21Rik
1	14062	F2r
1	29815	Bcar3
1	53320	Folh1
1	373864	Col27a1
1	100121	Tdrd7
1	105440	Kctd9
1	20409	Ostf1
1	226090	Ermp1
1	108159	Ubxd6
1	75939	4930579G24Rik
1	68815	Btbd10
1	233280	Nipa1
1	107227	Macrod1
1	50797	Copb2
1	14587	Gfra3
1	18750	Prkca
1	66070	Cwc15
1	69568	Vkorc1l1
1	232664	Ccdc136
1	232943	Klc3
1	353258	Ltv1
1	11732	Ank
1	102323	Dcun1d2
1	225579	Slc27a6
1	50490	Nox4
1	57340	Jph3
1	16193	Il6
1	76365	Tbx18
1	71778	Klhl5
1	108900	2700049P18Rik
1	66251	Arfgap3
1	14199	Fhl1
1	53886	Cdkl2
1	14238	Foxf2
1	20292	Ccl11
1	27404	Abca8b
1	328381	Sh2d4b
1	225659	Cep76
1	67072	Cdc37l1
1	24015	Abce1
1	71927	Itfg1
1	73373	Phospho2
1	21787	Tfg
1	67096	Mmachc
1	226823	Kctd3
1	13612	Edil3
1	67776	Loh11cr2a
1	544707	EG544707

1	74238	Mterfd3
1	74185	Gbe1
1	56444	Actr10
1	23924	Katna1
1	214048	Larp2
1	216558	Ugp2
1	11484	Aspa
1	17965	Nbl1
1	225888	Suv420h1
1	75572	Acyp2
1	59029	Psmd14
1	67059	Ola1
1	67795	6530404N21Rik
1	268822	Adck5
1	211347	Pank3
1	235293	Sc5d
1	54170	Rragc
1	56298	Arl6ip2
1	66971	Cdk5rap1
1	237553	Trhde
1	231532	Arhgap24
1	320727	Ipo8
1	69085	Zcchc9
1	19704	Upf1
1	668066	RP23-67E6.3
1	329575	RP23-275N21.2
1	100043387	RP23-232H13.5
1	213811	BC002059
1	218397	Rasa1
1	235907	Zfp71-rs1
1	14479	Usp15
1	83962	Btbd1
1	76958	2210418O10Rik
1	14670	Gna-rs1
1	16423	Cd47
1	54342	Gnpnat1
1	320717	Pptc7
1	217980	Larp5
1	68477	Rmnd5a
1	226255	Atrnl1
1	68310	Zmym1
1	22680	Zfp207
1	13680	Ddx19a
1	28019	Ing4
1	30795	Fkbp3
1	109652	Acy1
1	235036	Ppan
1	100041835	MMT00026054
1	100041835	MMT00002146
1	20623	Snrk
1	676923	MMT00040704

1	21848	Trim24
1	16600	Klf4
1	229699	Slc16a4
1	237412	EG237412
1	72899	Macrod2
1	72103	2010301N04Rik
1	71703	Armcx3
1	58861	Cysltr1
1	67229	Prpf18
1	66706	4733401H18Rik
1	13709	Elf1
1	54369	Nme6
1	21898	Tlr4
1	11502	Adam9
1	66205	Cd302
1	216616	Efemp1
1	140792	Colec12
1	56878	Rbms1
1	77864	Ypel2
1	68845	Pih1d1
1	225896	D19Ertd721e
1	66587	Fastk
1	73112	3110003A17Rik
1	233067	Lrfn3
1	319565	Syne2
1	207259	Zbtb7c
1	57815	Spata5
1	66141	Ifitm3
1	236366	5730507C01Rik
1	101604	E430018J23Rik
1	12915	Crebl1
1	108153	Adamts7
1	NaN	MMT00008170
1	14309	Fshr
1	98710	Rabif
1	16562	Kif1c
1	226646	Ndufs2
1	NaN	MMT00053630
1	104418	Dgkz
1	12567	Cdk4
1	12567	Cdk4
1	50798	Gne
1	NaN	MMT00014519
1	14411	Slc6a12
1	97387	Strn4
1	380969	C230021P08Rik
1	110637	Grik4
1	53761	Bat2
1	67832	Bxdc2
1	212647	Aldh4a1
1	101869	Unc45a

1	67873	2410018C20Rik
1	72748	Hdhd3
1	107999	Gtpbp6
1	66421	2410004B18Rik
1	NaN	MMT00004881
1	22194	Ube2e1
1	22194	Ube2e1
1	NaN	MMT00013262
1	27058	Srp9
1	NaN	MMT00058199
1	NaN	MMT00078937
1	NaN	MMT00076721
1	106957	Slc39a6
1	NaN	MMT00028111
1	19656	Rbmxrt
1	19656	Rbmxrt
1	56278	Gkap1
1	109082	Fbxw17
1	29809	Rabgap1l
1	67489	Ap4b1
1	233765	Plekha7
1	50883	Chek2
1	140484	Pofut1
1	212989	Best2
1	107747	Aldh1l1
1	231134	Dok7
1	20714	Serpina3k
1	22346	Vhlh
1	NaN	MMT00019109
1	NaN	MMT00080648
1	NaN	MMT00063687
1	57274	Slc16a8
1	74116	Pi16
1	NaN	MMT00026797
1	52713	Ccdc59
1	17748	Mt1
1	245269	E130304F04Rik
1	70369	Bag5
1	56715	Rabgef1
1	63913	Niban
1	241915	Phc3
1	30806	Adamts8
1	724112	BC106175
1	67416	Armcx2
1	107515	Lgr4
1	69178	Snx5
1	67168	P2ry5
1	NaN	MMT00061970
1	102084	AI451557
1	19663	Rbpms
1	100041868	MMT00065456

1	106821	AI314976
1	14029	Evx2
1	12325	Camk2g
1	71514	Sfpq
1	68010	Bambi
1	15904	Id4
1	12589	Ift81
1	56364	Zmym3
1	57814	Kcne4
1	68979	Nol11
1	69185	Dtwd1
1	11920	Atm
1	103784	Wdr92
1	30931	Tor1a
1	NaN	MMT00082622
1	69109	1810009O10Rik
1	NaN	MMT00051866
1	66624	Spcs2
1	56794	Hacl1
1	20520	Slc22a5
1	15357	Hmgcr
1	78833	Gins3
1	12144	Blm
1	50493	Txnrd1
1	27083	Xlr4b
1	67213	Cmtm6
1	74741	5730419I09Rik
1	102910	Armcx4
1	18087	Nktr
1	219140	Spata13
1	329540	8430427H17Rik
1	66416	Ndufa7
1	NaN	MMT00000385
1	66632	Atpbd4
1	52592	Brms1l
1	93885	Pcdhb14
1	320742	A230072C01Rik
1	NaN	MMT00040265
1	320253	3-Mar
1	319613	5730410E15Rik
1	329650	Med12l
1	75796	Cdyl2
1	22057	Tob1
1	66860	Tanc1
1	246293	Klhl8
1	12369	Casp7
1	19108	Prkx
1	15278	Tfb2m
1	27377	Yme1l1
1	14815	Nr3c1
1	71910	Ppapdc1

1	NaN	MMT00002755
1	NaN	MMT00007396
1	12457	Ccrn4l
1	74868	Tmem65
1	11426	Macf1
1	74044	Ttf2
1	NaN	MMT00008417
1	21763	Tex2
1	54375	Azin1
1	16653	Kras
1	67092	Gatm
1	23983	Pcbp1
1	264064	Cdk8
1	76804	Jmjd2c
1	18949	Pnn
1	NaN	MMT00040762
1	55980	Impa1
1	258268	Olfr1511
1	109791	Clps
1	18049	Ngf
1	26557	Homer2
1	17847	Usp34
1	12763	Cmah
1	330863	Trim67
1	18554	Pcsk7
1	217684	4933426M11Rik
1	22221	Ubp1
1	54419	Cldn6
1	224058	Tmem207
1	74966	4930471G03Rik
1	16177	Il1r1
1	51797	Ctps
1	76687	Spcs3
1	NaN	MMT00033243
1	434439	BC048562
1	58170	Accn5
1	70767	Prpf3
1	18004	Nek1
1	218543	Sfrs12
1	14629	Gclc
1	228790	Asxl1
1	59005	Trappc2l
1	66077	Aurkaip1
1	99586	Dpyd
1	56196	Ttrap
1	223435	Trio
1	78618	Centb2
1	97827	Exdl2
1	67533	Ppfibp1
1	17118	Marcks
1	14758	Gpm6b

1	72007	Fndc3b
1	17076	Ly75
1	NaN	MMT00045897
1	100047309	MMT00074326
1	53378	Sdcbp
1	19330	Rab18
1	50753	Fbxo8
1	226414	Dars
1	19027	Sypl
1	22770	Zhx1
1	NaN	MMT00078831
1	229445	Ctso
1	237082	Nxt2
1	74838	Narg1
1	235072	7-Sep
1	382030	Tmem188
1	109108	Slc30a9
1	214627	Papd5
1	70238	Rnf168
1	12807	Hps3
1	19822	Rnf4
1	231287	Atp10d
1	30960	Vapa
1	13178	Dck
1	102442	Dennd4a
1	233490	Crebzf
1	67581	Tbc1d23
1	234353	Psd3
1	19877	Rock1
1	14007	Cugbp2
1	53599	Cd164
1	75964	D030074E01Rik
1	70568	Cpne3
1	104215	Rhoq
1	93888	Pcdhb17
1	72123	2010109K11Rik
1	11639	Ak3l1
1	19053	Ppp2cb
1	258593	Olfr700
1	269704	Zfp664
1	50875	Tmod3
1	628040	EG628040
1	57437	Golga7
1	118451	Mrps2
1	52392	D1Ertd622e
1	17126	Smad2
1	59079	Erbb2ip
1	NaN	MMT00006420
1	77781	Epm2aip1
1	68187	4921533L14Rik
1	11538	Adnp

1	16452	Jak2
1	319901	Dsel
1	17961	Nat2
1	226026	Smc5
1	12675	Chuk
1	19821	Rnf2
1	66687	Tbc1d15
1	12421	Rb1cc1
1	22764	Zfx
1	76892	Rnft1
1	52635	D12Ertd551e
1	213109	Phf3
1	56726	Sh3bgrl
1	627049	Zfp800
1	24109	Ubl3
1	22632	Yy1
1	17912	Myo1b
1	56258	Hnrnph2
1	229227	4932438A13Rik
1	216705	Clint1
1	12387	Ctnnb1
1	98758	Hnrnpf
1	210711	1110007A13Rik
1	54607	Socs6
1	98415	Nucks1
1	52705	Krr1
1	56297	Arl6
1	627908	MMT00030553
1	68151	Gpr177
1	71835	Lancl2
1	227835	Gtdc1
1	225651	Mppe1
1	93760	Arid1a
1	109161	Ube2q2
1	231050	Galnt11
1	217615	Ctage5
1	217732	2310044G17Rik
1	74362	Spag17
1	NaN	MMT00065780
1	330962	MMT00003408
1	75710	Rbm12
1	258589	Olfr703
1	77134	Hnrnpa0
1	11846	Arg1
1	226151	6030443O07Rik
1	71492	Bbs7
1	74018	Als2
1	268288	Samd3
1	71773	Ugt2b1
1	108857	Ankhd1
1	217893	Pacs2

1	22073	Prss3
1	NaN	MMT00057004
1	630663	MMT00057236
1	228777	Nrsn2
1	52897	D11Bwg0517e
1	74666	4930432K21Rik
1	77531	Anks1b
1	13706	RP23-395H4.4
1	242819	Rundc3b
1	378700	RP23-244H7.9
1	629219	EG629219
1	17318	Mid1
1	217356	Tmc8
1	210356	E030049G20Rik
1	78634	1700094C09Rik
1	243853	Fkrp
1	NaN	MMT00047341
1	237759	Col23a1
1	NaN	MMT00073763
1	20501	Slc16a1
1	11843	Arf4
1	19353	Rac1
1	68303	9130005N14Rik
1	12778	Cxcr7
1	19876	Robo1
1	16948	Lox
1	21982	Tmem165
1	69608	Sec24d
1	56193	Plek
1	13032	Ctsc
1	70472	Atad2
1	13185	Dscr3
1	14700	Gng10
1	17973	Nck1
1	56456	Actl6a
1	20947	MMT00047924
1	12123	Hrk
1	NaN	MMT00059239
1	NaN	MMT00082889
1	16363	Irf2
1	56030	Tmem131
1	215814	Ccdc28a
1	320184	Lrrc58
1	68511	1110015M06Rik
1	19286	Pts
1	74552	Npal3
1	226413	Lct
1	116732	Tsga13
1	19652	Rbm3
1	NaN	MMT00076410
1	63872	Zfp296

1	80794	Cblc
1	17175	Masp2
1	NaN	MMT00076058
1	213989	Tmem82
1	67726	1810073G14Rik
1	68183	Bcas2
1	75627	Snapc1
1	16328	Cep250
1	78767	2610021K21Rik
1	216274	Cep290
1	NaN	MMT00070118
1	20346	Sema3a
1	69053	1810013L24Rik
1	20482	Skil
1	107753	Lgals2
1	12904	Crabp2
1	104910	AI132487
1	14776	Gpx2
1	75678	Ippk
1	407788	BC051142
1	19144	Klk6
1	21685	Tef
1	381073	Npw
1	73476	1700069L16Rik
1	NaN	MMT00081088
1	380787	A230065H16Rik
1	171277	V1rh20
1	NaN	MMT00048291
1	170947	Myoz3
1	NaN	MMT00033542
1	243547	Grip2
1	66877	Crnkl1
1	224813	Gm88
1	72242	Psg21
1	226123	BC023055
1	68891	Cd177
1	332937	Tcfap2e
1	19281	Ptprt
1	29818	Hspb7
1	74043	Pex26
1	NaN	MMT00015104
1	93961	B3galt5
1	209497	Tmem164
1	30051	Spdef
1	69864	1810065E05Rik
1	NaN	MMT00010979
1	381373	Sp9
1	66473	Ctrb1
1	56554	Raet1d
1	109660	Ctrl
1	16413	Itgb1bp1

1	193034	Trpv1
1	227526	Armetl1
1	71544	9030420J04Rik
1	11434	Acr
1	545474	Scrt2
1	215493	A3galt2
1	18752	Prkcc
1	71130	Sh2d6
1	233066	AI428936
1	68709	Cilp2
1	12794	Cnih2
1	12416	Cbx2
1	18260	Ocln
1	432516	Myo1a
1	20450	St8sia2
1	14764	Gpr44
1	15171	Hcrt
1	228598	Ebf4
1	74562	Fer1l4
1	NaN	MMT00012844
1	11778	Ap3s2
1	218210	Nup153
1	237362	Npffr1
1	55938	Apom
1	225852	Gm550
1	78369	Icam4
1	68416	Sycn
1	192188	Stab2
1	22364	Vpreb3
1	83813	Tnk1
1	83396	Glis2
1	12193	Zfp36l2
1	272636	D9Ertd280e
1	108899	2700081O15Rik
1	234421	Cib3
1	69169	Faim3
1	209786	Vmn2r-ps88
1	64451	Dip2a
1	27386	Npas3
1	434440	Fbxw12
1	18545	Pcp2
1	68311	Lypd2
1	51811	Clec4f
1	58214	Cst10
1	258408	Olfr463
1	19820	Rnf12
1	75301	4930556N09Rik
1	546036	EG546036
1	223726	Mpped1
1	56640	Klk4
1	11806	Apoa1

1	239530	Gpr20
1	NaN	MMT00033159
1	NaN	MMT00049098
1	12613	Cel
1	14240	Foxb2
1	240057	Syngap1
1	17202	Mc4r
1	211232	Cpne9
1	102075	Plekhg4
1	12504	Cd4
1	NaN	MMT00005098
1	16728	L1cam
1	258918	Olfr1346
1	72507	Dzip1l
1	241612	Slc5a12
1	671564	Rnf212
1	NaN	MMT00011231
1	NaN	MMT00013041
1	208372	Asb18
1	NaN	MMT00013481
1	627648	EG627648
1	59083	Fetub
1	78829	Tsc22d4
1	280635	Emilin3
1	171543	Bmf
1	12300	Cacng2
1	18479	Pak1
1	NaN	MMT00015975
1	NaN	MMT00033987
1	56296	Dmrtb1
1	208266	Dot1l
1	20841	Zfp143
1	27220	Cartpt
1	233038	1190020J12Rik
1	20410	Sorbs3
1	74563	Rasgef1c
1	30791	Slc39a1
1	20401	Sh3bp1
1	208104	Mlxip
1	214239	A430105I19Rik
1	70024	Mcm10
1	118449	Synpo2
1	545279	Ms4a15
1	19303	Pxn
1	18429	Oxt
1	76942	Lypd5
1	381148	Gm1614
1	231691	Sds
1	12854	Cort
1	13095	Cyp2c29
1	66932	Rexo1

1	635169	MMT00073704
1	26362	Axl
1	21786	Tff3
1	20977	Syp
1	56632	Sphk2
1	60507	Qtrt1
1	73728	Psd
1	58988	Rps6kb2
1	21341	Taf1c
1	68423	Ankrd13d
1	211651	Fancd2
1	104884	Tdp1
1	18548	Pcsk1
1	113854	V1rb4
1	243842	Gltscr1
1	57255	Cldn13
1	NaN	MMT00042954
1	108068	Grm2
1	NaN	MMT00048659
1	545481	RP23-392P11.3
1	19876	Robo1
1	94191	Adarb2
1	18439	P2rx7
1	29859	Sult4a1
1	NaN	MMT00031206
1	230863	Sh2d5
1	12962	Crybb3
1	13041	Ctsw
1	242939	Cpz
1	219149	Xkr6
1	NaN	MMT00017417
1	NaN	MMT00048670
1	68957	Paqr6
1	320404	Itpkb
1	98952	C230093N12Rik
1	NaN	MMT00078052
1	317755	Zar1
1	24046	Scn11a
1	67772	Chd8
1	22072	Prss2
1	20776	Tmie
1	22073	Prss3
1	73532	1700080E11Rik
1	631304	Cyp4f40
1	NaN	MMT00065821
1	NaN	MMT00027552
1	NaN	MMT00009628
1	207495	Baiap2l2
1	230235	6430704M03Rik
1	16596	Klf1
1	258715	Olfr421

1	330908	Opcml
1	382083	Snx22
1	246788	Trpv3
1	78304	Lsmd1
1	71781	Slc16a14
1	57776	Ttyh1
1	434437	Amt
1	17341	Bhlhb8
1	258701	Olfr401
1	213262	Fstl5
1	330428	D630042F21Rik
1	258573	Olfr1020
1	NaN	MMT00008620
1	74937	4930481B07Rik
1	NaN	MMT00006438
1	636752	EG636752
1	72948	Tppp
1	230809	Pdik1l
1	108934	BC024659
1	16973	Lrp5
1	101359	D330027H18Rik
1	333050	Ksr2
1	217734	Pomt2
1	11604	Agrp
1	66768	4933428G09Rik
1	71159	4933416I08Rik
1	11898	Ass1
1	11898	Ass1
1	258752	Olfr583
1	195018	Zzef1
1	404195	Cyp2c54
1	13385	Dlg4
1	545817	Cyp2w1
1	76574	Mfsd2
1	16891	Lipg
1	NaN	YGL234W_RC-551
1	235858	Gm392
1	171253	V1ri2
1	245688	Rbbp7
1	14134	Fcnb
1	75690	2210412E05Rik
1	14402	Gabrb3
1	214058	Megf11
1	56212	Rhog
1	114230	Aipl1
1	26992	Brd7
1	268756	Gulo
1	78928	Pigt
1	21416	Tcf7l2
1	NaN	MMT00015397
1	236598	MMT00013190

1	227545	5430407P10Rik
1	NaN	MMT00055001
1	236598	MMT00009389
1	NaN	MMT00079286
1	227195	A430093A21Rik
1	170798	AY036118
1	12168	Bmpr2
1	66314	Tpd52l2
1	75985	Rab30
1	71828	Gtf2a1l
1	245424	Gpr101
1	330836	Slc7a6
1	382137	D630004A14Rik
1	59030	Mkks
1	66975	2410002O22Rik
1	71761	Amdhd1
1	67736	Ccdc130
1	107869	Cth
1	NaN	MMT00057242
1	68184	Denr
1	72759	Tmem135
1	64085	Clstn2
1	623205	MMT00017441
1	99730	Taf13
1	76561	Snx7
1	19212	Pter
1	67290	3110040N11Rik
1	52076	Tmem38b
1	67468	Mmd
1	64213	St7
1	77647	Trat1
1	12450	Ccng1
1	55946	Ap3m1
1	66736	Ttc35
1	13664	Eif1a
1	NaN	MMT00024873
1	66143	Eef1e1
1	69721	Nkiras1
1	67834	Idh3a
1	18760	Prkd1
1	13809	Enpep
1	11964	Atp6v1a
1	11789	Apc
1	18041	Nfs1
1	74252	Armc1
1	NaN	MMT00006294
1	68050	6330407G11Rik
1	56305	Pitpnb
1	64652	Nisch
1	108664	Atp6v1h
1	66397	Sar1b

1	53382	Txnl1
1	19744	Rheb
1	624446	EG624446
1	19299	Abcd3
1	319263	Pcmtd1
1	NaN	MMT00011132
1	71982	Snx10
1	433862	EG433862
1	71968	Wdr73
1	67526	Atg12
1	214663	Slc25a29
1	22160	Twist1
1	234356	Csgalnact1
1	73910	Arhgap18
1	237542	Osbpl8
1	20452	St8sia4
1	117167	Steap4
1	72033	Tsc22d2
1	16168	Il15
1	94192	C1galt1
1	100125272	RP24-87L14.2
1	NaN	MMT00062592
1	22441	Xlr
1	72993	Appl1
1	72085	Osgepl1
1	15982	Ifrd1
1	66626	5730403B10Rik
1	210789	Tbc1d4
1	74147	Ehhadh
1	67993	Nudt12
1	633285	EG633285
1	67455	Klhl13
1	17022	Lum
1	20128	Trim30
1	209387	AI451617
1	74558	Gvin1
1	21824	Thbd
1	69181	Dyrk2
1	68564	Nufip2
1	73173	Pcdh18
1	15394	Hoxa1
1	71458	Bcor
1	18221	Nudc
1	13666	Eif2ak3
1	241230	St8sia6
1	108151	Sema3d
1	65960	Twsg1
1	74213	Rbm26
1	18783	Pla2g4a
1	214791	Sertad4
1	207304	Hectd1

1	76740	Efr3a
1	218441	Zfyve16
1	252876	Zh2c2
1	NaN	MMT00058658
1	228421	Kif18a
1	330256	MMT00008885
1	16643	Klrd1
1	71586	Ifih1
1	20583	Snai2
1	18788	Serpinb2
1	12173	Bnc1
1	22418	Wnt5a
1	66864	Clec14a
1	432812	BC052688
1	545370	Hmcn1
1	70823	Hmgb2l1
1	76117	Arhgap15
1	66871	Cpne8
1	19159	Pscd3
1	11671	Aldh3a2
1	271970	Arsj
1	NaN	MMT00025372
1	207214	Larp4
1	20382	Sfrs2
1	100201	Tmem64
1	626316	OTTMUSG00000010173
1	238831	Ppwd1
1	622175	EG622175
1	246229	Bivm
1	67606	1110018M03Rik
1	68833	Pdcl3
1	66897	Narg1l
1	212880	Ddx46
1	234865	Nup133
1	75698	3110001K24Rik
1	69934	Rg9mtd3
1	66935	1700023B02Rik
1	NaN	MMT00033070
1	12368	Casp6
1	12747	Clk1
1	70237	Bhlhb9
1	67367	1810007M14Rik
1	60599	Trp53inp1
1	67245	Peli1
1	69226	Snx24
1	12628	Cfh
1	214403	EG214403
1	140742	Sesn1
1	104015	Synj1
1	11796	Birc3
1	208177	Phldb2

1	338337	Cog3
1	11819	Nr2f2
1	55935	Fnbp4
1	104263	Jmjd1a
1	320712	Abi3bp
1	20680	Sox7
1	360216	Zranb1
1	215748	Cnksr3
1	270166	Clpx
1	108853	Mtrf1l
1	13006	Smc3
1	69048	Slc30a5
1	NaN	MMT00018157
1	219105	Zmym5
1	53328	Pgrmc1
1	98221	Eif3m
1	105522	Ankrd28
1	101565	6330503K22Rik
1	116914	Slc19a2
1	NaN	MMT00014691
1	74521	8430415E04Rik
1	NaN	MMT00065196
1	230484	Usp1
1	NaN	MMT00001293
1	16709	Ktn1
1	67028	2610002M06Rik
1	494468	Armcx5
1	235050	Zfp810
1	381062	2210404J11Rik
1	67311	Nanp
1	67311	Nanp
1	27362	Dnajb9
1	75430	3200002M19Rik
1	214804	Syde2
1	74670	4930432O21Rik
1	22644	Rnf103
1	230157	Tmeff1
1	66724	Map3k7ip3
1	59007	Ngly1
1	73942	4930405M20Rik
1	18139	Zfml
1	66875	1200016B10Rik
1	224598	Zfp758
1	105559	Mbnl2
1	56442	Serinc1
1	269424	Phf17
1	75424	Zfp820
1	237411	B230315N10Rik
1	64602	Ireb2
1	218503	Fcho2
1	22327	Vbp1

1	101214	Tra2a
1	66580	Esf1
1	271457	Rab5a
1	544971	Bdp1
1	240283	Dmxl1
1	214498	Cdc73
1	110920	Stch
1	77805	Esco1
1	73288	Ccdc132
1	67089	Psmc6
1	67500	Ccar1
1	70349	Copb1
1	67300	Cltc
1	70611	Fbxo33
1	382038	AK122209
1	80907	Lactb
1	219228	Pcdh17
1	66131	Tipin
1	170791	Rbm39
1	NaN	MMT00041792
1	22359	Vldlr
1	63985	Gmfb
1	84112	Sucnr1
1	210293	Dock10
1	64214	Rgs18
1	70536	Qpct
1	12364	Casp12
1	212898	Dse
1	66695	Aspn
1	NaN	MMT00046622
1	378462	Morn2
1	50500	Ttpa
1	17184	Matr3
1	226144	Erlin1
1	68146	Arl13b
1	20349	Sema3e
1	54561	Nap1l3
1	72902	Spock3
1	19084	Prkar1a
1	224794	Enpp4
1	80859	Nfkbiz
1	17158	Man2a1
1	76421	1700028K03Rik
1	56036	Ccnl2
1	21847	Klf10
1	70974	Pgm2l1
1	170772	Glcci1
1	216198	Tcp11l2
1	545389	Cep170
1	70099	Smc4
1	18080	Nin

1	70834	Spag9
1	629065	EG629065
1	232286	Tmf1
1	17311	Kitl
1	329877	Dennd4c
1	319448	Fndc3a
1	11416	Slc33a1
1	NaN	MMT00026286
1	320940	Atp11c
1	329506	Ctdspl2
1	58800	Trpm7
1	50789	Fbxl3
1	225028	Map4k3
1	78521	B230219D22Rik
1	22612	Yes1
1	69981	Tmem30a
1	170719	Oxr1
1	20663	Sos2
1	109151	Chd9
1	70155	Ogfrl1
1	227446	2310035C23Rik
1	321000	4933421E11Rik
1	67760	Slc38a2
1	74326	Hnrnpr
1	231051	Mll3
1	NaN	MMT00062595
1	11908	Atf1
1	328108	A430041B07Rik
1	22343	Lin7c
1	211255	Kbtbd7
1	56195	Ptbp2
1	271377	Zbtb11
1	52184	Odf2l
1	408062	BC062115
1	72421	Ttc30b
1	66625	Sfrs18
1	108829	Jmjd1c
1	19386	Ranbp2
1	22764	Zfx
1	NaN	MMT00038995
1	13204	Dhx15
1	52323	Klhl7
1	67010	Rbm7
1	12033	Bcap29
1	269198	Nbeal1
1	22215	Ube3a
1	57438	7-Mar
1	NaN	MMT00034426
1	319615	6330416L07Rik
1	224585	Zfp160
1	27215	Azi2

1	68145	Etaa1
1	67019	Actr6
1	93759	Sirt1
1	235469	Suhw4
1	231464	Cnot6l
1	235380	Dmxl2
1	109333	Pkn2
1	74182	Prei4
1	14453	Gas2
1	68734	Smek1
1	20687	Sp3
1	22762	Zfpm2
1	12452	Ccng2
1	53330	Vamp4
1	67043	Syap1
1	72999	Insig2
1	68861	1190002N15Rik
1	641340	Nrbf2
1	192657	Ell2
1	14600	Ghr
1	239122	Setdb2
1	NaN	MMT00071421
1	NaN	MMT00050193
1	210853	EG210853
1	74149	1300003B13Rik
1	56758	Mbnl1
1	NaN	MMT00014015
1	74114	Crot
1	76900	Ssbp4
1	NaN	MMT00029171
1	319638	Nt5dc1
1	66870	Serbp1
1	26611	Rcn2
1	240396	Mex3c
1	67864	Yipf4
1	210104	BC043301
1	216965	Taok1
1	21974	Top2b
1	NaN	MMT00082345
1	NaN	MMT00054557
1	70078	Nol7
1	75734	5230400G24Rik
1	17156	Man1a2
1	109905	Rap1a
1	68944	Tmco1
1	73130	Tmed5
1	213056	D1Ertd53e
1	20912	Stxbp3a
1	98267	Stk17b
1	15247	Hiat1
1	26885	Casp8ap2

1	66552	2010106G01Rik
1	668838	EG668838
1	13690	Eif4g2
1	545700	Vmn2r-ps14
1	104348	Zfp120
1	108645	Mat2b
1	NaN	MMT00045089
1	NaN	MMT00046586
1	67456	Ergic2
1	217869	Eif5
1	NaN	MMT00052614
1	52575	Rg9mtd1
1	110750	Cse1l
1	20462	Sfrs10
1	77591	Ddx10
1	NaN	MMT00060167
1	66213	Med7
1	56426	Pdcd10
1	NaN	MMT00011278
1	71900	Tmem106b
1	66631	Hiatl1
1	72098	Tmem68
1	68044	Chac2
1	70047	Trnt1
1	19302	Pex2
1	20462	Sfrs10
1	NaN	MMT00063585
1	68736	1110034B05Rik
1	14228	Fkbp4
1	68055	Atp5s
1	20843	Stag2
1	22782	Slc30a1
1	210106	Pols
1	226747	Ahctf1
1	68646	1110020G09Rik
1	72692	Hnrpll
1	93871	Brwd1
1	NaN	MMT00039793
1	107769	Tm6sf1
1	50766	Crim1
1	11856	Arhgap6
1	72404	Wdr44
1	213819	Casd1
1	19645	Rb1
1	56367	Scoc
1	73124	Golim4
1	70827	Trak2
1	76137	Ccdc90a
1	54635	Pdgfc
1	15273	Hivep2
1	15251	Hif1a

1	78923	Chsy3
1	74197	Gtf2e1
1	50850	Spast
1	242521	Klhl9
1	19046	Ppp1cb
1	67427	Rps20
1	142980	Tlr3
1	22235	Ugdh
1	17286	Meox2
1	211986	Tmem18
1	72139	2610044O15Rik
1	64451	Dip2a
1	269033	4930503L19Rik
1	76763	Mospd2
1	66713	Actr2
1	71721	1200015N20Rik
1	227059	Slc39a10
1	78248	Armcx1
1	11977	Atp7a
1	12617	Cenpc1
1	70799	Cep192
1	19650	Rbl1
1	17918	Myo5a
1	382252	A830080D01Rik
1	13405	Dmd
1	320267	Fubp3
1	17997	Nedd1
1	67770	5830433M19Rik
1	105203	BC016423
1	59015	Nup160
1	79264	Krit1
1	78752	Csgalnact2
1	338467	Morc3
1	233812	BC030336
1	17772	Mtm1
1	235574	Atp2c1
1	72425	2410042D21Rik
1	11622	Ahr
1	19091	Prkg1
1	NaN	MMT00024195
1	16438	Itpr1
1	14020	Evi5
1	77113	Klhl2
1	72508	Rps6kb1
1	21788	Tfpi
1	22289	Utx
1	16396	Itch
1	237898	Usp32
1	93836	Rnf111
1	73754	Thap1
1	19317	Qk

1	269016	Sh3rf2
1	26894	Cops7a
1	NaN	MMT00038159
1	70354	3110001I20Rik
1	67771	Arpc5
1	319601	Zfp653
1	20823	Ssb
1	77505	Dnhd1
1	216618	Ccdc104
1	56041	Uso1
1	207704	Gtpbp10
1	72568	Lin9
1	70612	5730494N06Rik
1	NaN	MMT00058516
1	NaN	MMT00056913
1	NaN	MMT00041235
1	NaN	MMT00048285
1	434339	EG434339
1	21353	Tank
1	NaN	MMT00002799
1	66588	Cmpk1
1	72129	Pex13
1	223601	0910001A06Rik
1	12632	Cfl2
1	19070	Mobkl3
1	66979	Pole4
1	71675	0610010F05Rik
1	59032	Ppp2r3c
1	15452	Hprt1
1	66092	Ghitm
1	NaN	MMT00051501
1	66725	Lrrk2
1	14265	Fmr1
1	55946	Ap3m1
1	29864	Rnf11
1	212285	Centd1
1	12633	Cflar
1	56705	Ranbp9
1	23950	Dnajb6
1	20280	Scp2
1	624862	EG624862
1	12176	Bnip3
1	NaN	MMT00020969
1	66840	Wdr45l
1	65972	Ifi30
1	67241	Smc6
1	216965	Taok1
1	83493	Sacm1l
1	12038	Bche
1	140483	Hnmt
1	67452	Pnpla8

1	215751	BC013529
1	NaN	MMT00005424
1	66884	Appbp2
1	229906	Gtf2b
1	229681	St7l
1	235461	B230380D07Rik
1	67204	Eif2s2
1	69482	Nup35
1	67005	Polr3k
1	65114	Vps35
1	59048	C1galt1c1
1	12301	Cacybp
1	57896	Krcc1
1	NaN	MMT00077382
1	67035	Dnajb4
1	14911	Thumpd3
1	216395	Tmem5
1	12912	Creb1
1	12068	Bet1
1	68117	Apool
1	381101	BC048355
1	NaN	MMT00040284
1	214505	Gnptg
1	66822	Fbxo25
1	67759	5033414D02Rik
1	76044	Ncapg2
1	74386	Rmi1
1	75296	Fgfr1op
1	67008	1600012F09Rik
1	98732	Rab3gap2
1	51886	Fubp1
1	110876	Scn2a1
1	224171	C330027C09Rik
1	211556	BC002199
1	13645	Egf
1	11938	Atp2a2
1	107767	Scamp1
1	26965	Cul1
1	64291	Osbpl1a
1	210544	Wdr67
1	72400	2610028A01Rik
1	71929	Tmem123
1	75974	Dock11
1	15446	Hpgd
1	73389	Hbp1
1	74533	Gzf1
1	114774	Pawr
1	14190	Fgl2
1	218442	Serinc5
1	NaN	MMT00010886
1	104776	Aldh6a1

1	106326	Osbpl11
1	14066	F3
1	109241	Mbd5
1	18115	Nnt
1	216805	Flcn
1	20437	Siah1a
1	229055	Zbtb10
1	80898	Erap1
1	67723	4932415G12Rik
1	380921	Dgkh
1	218203	Mylip
1	59042	Cope
1	67211	Armc10
1	NaN	MMT00051649
1	26422	Nbea
1	78903	Wrnip1
1	72309	Tmem158
1	73390	1700060H10Rik
1	22021	Tpst1
1	259279	Tubgcp3
1	70598	Filip1
1	78334	Cdc2l6
1	225207	Zfp521
1	28146	D3Ucla1
1	13637	Efna2
1	67437	Ssr3
1	12952	Cry1
1	72843	Prdm4
1	12812	Coil
1	17869	Myc
1	76936	Hnrnpm
1	76187	Adhfe1
1	20503	Slc16a7
1	NaN	MMT00044927
1	69535	2310004N24Rik
1	14084	Faf1
1	108079	Prkaa2
1	19687	Rfc1
1	NaN	MMT00041409
1	75770	Brsk2
1	56210	Rev1
1	11797	Birc2
1	66866	Nhlrc2
1	16588	Kin
1	217664	Mgat2
1	224938	Pja2
1	20272	Scn7a
1	225027	Sfrs7
1	NaN	MMT00017521
1	170938	Zfp617
1	114893	Dcun1d1

1	114893	Dcun1d1
1	56351	Ptges3
1	56351	Ptges3
1	67877	Nat5
1	12226	Btg1
1	109019	Obfc2a
1	18295	Ogn
1	18242	Oat
1	NaN	MMT00021961
1	NaN	MMT00007831
1	NaN	MMT00060741
1	56615	Mgst1
1	116940	Tgs1
1	105387	Akr1c14
1	226695	Ifi205
1	243967	Gm484
1	270118	Maml2
1	218693	Paip1
1	15951	Ifi204
1	74277	Chic2
1	102632	Acad11
1	19217	Ptger2
1	53605	Nap1l1
1	65099	Irak1bp1
1	105171	Arrdc3
1	20393	Sgk1
1	13388	Dll1
1	109113	Uhrf2
1	67198	2810022L02Rik
1	232288	Frmd4b
1	83453	Chrdl1
1	68743	Anln
1	NaN	MMT00044046
1	18073	Nid1
1	12380	Cast
1	12468	Cct7
1	13518	Dst
1	170625	Snx18
1	70747	Tspan2
1	240725	Sulf1
1	76453	Prss23
1	11745	Anxa3
1	624910	Rgs21
1	71720	Osbpl3
1	72672	Zfp518
1	218914	Wapal
1	77604	C430048L16Rik
1	71801	Plekhf2
1	219135	Mtmr6
1	50884	Nckap1
1	78913	Zfp294

1	101148	B630005N14Rik
1	67958	2610101N10Rik
1	241070	Gpr1
1	21814	Tgfbr3
1	67134	Nol5a
1	20742	Spnb2
1	116904	Alpk3
1	208117	Aph1b
1	18046	Nfyc
1	18046	Nfyc
1	13717	Eln
1	56324	Stam2
1	381580	Ccdc27
1	NaN	MMT00029554
1	74094	Tjap1
1	22040	Trex1
1	667804	MMT00076032
1	56748	Nfu1
1	20133	Rrm1
1	26360	Angptl2
1	286942	Kif19a
1	19172	Psmb4
1	83766	Actl6b
1	14601	Ghrh
1	108927	Lhfp
1	68533	Mphosph6
1	54204	1-Sep
1	NaN	MMT00006341
1	226922	Kcnq5
1	72590	Ppme1
1	240899	Lrrc52
1	76389	Ankrd36
1	319996	Casc4
1	50908	C1s
1	235033	Rdh8
1	20669	Sox14
1	258979	Olfr1255
1	259301	Leap2
1	11548	Adra1b
1	213211	Rnf26
1	13838	Epha4
1	NaN	MMT00027295
1	665270	Plb1
1	18504	Pax2
1	330830	Ccdc135
1	20182	Rxrb
1	241769	Kcnk15
1	66237	Atp6v1g2
1	224705	Vps52
1	11546	Parp2
1	68977	Haghl

1	79043	Spsb3
1	23989	Med24
1	55927	Hes6
1	12909	Crcp
1	56844	Tssc4
1	216551	1110067D22Rik
1	73132	Slc25a16
1	67045	Riok2
1	70025	Acot7
1	28113	Tinf2
1	76073	Pcgf5
1	223922	Atf7
1	22700	Zfp40
1	238317	C130039O16Rik
1	NaN	MMT00023461
1	75812	Tasp1
1	22218	Sumo1
1	22218	Sumo1
1	NaN	MMT00048325
1	56220	Zfp386
1	69207	Sfrs11
1	381644	Cep135
1	68730	Dus1l
1	338366	Mia3
1	18805	Pld1
1	54598	Calcrl
1	16210	Impact
1	12552	Cdh11
1	635702	Naaladl2
1	12192	Zfp36l1
1	235682	Zfp445
1	68818	Zfand2b
1	22259	Nr1h3
1	67138	Herc5
1	80285	Parp9
1	17472	Gbp4
1	234311	Ddx60
1	233056	Zfp790
1	NaN	MMT00047910
1	320951	Pisd
1	327956	Vmo1
1	263803	Pkn3
1	76686	Clip3
1	75646	Rai14
1	622459	RP23-303F24.3
1	170765	Ripply3
1	633752	EG633752
1	56724	Cript
1	321003	Xpnpep3
1	19359	Rad23b
1	56085	Ubqln1

1	52665	Echdc1
1	55951	Brp44l
1	NaN	MMT00081492
1	107734	Mrpl30
1	54364	Rpp30
1	NaN	MMT00002367
1	NaN	MMT00042824
1	23873	Faim
1	NaN	MMT00037377
1	66637	5730449L18Rik
1	66637	5730449L18Rik
1	NaN	MMT00009124
1	66917	Chordc1
1	NaN	MMT00052151
1	NaN	MMT00081393
1	24067	Srp54a
1	20916	Sucla2
1	225363	Etf1
1	17876	Myef2
1	12848	Cops2
1	12785	Cnbp
1	211253	Mtrf1
1	NaN	MMT00050725
1	546071	Mast3
1	245050	C730027P07Rik
1	12411	Cbs
1	75563	Dnali1
1	16426	Itih3
1	23920	Insrr
1	328779	Hs3st6
1	18781	Pla2g2c
1	108832	5430405G05Rik
1	57749	Piwil1
1	16326	Inhbe
1	268291	Rnf217
1	27214	Dbf4
1	70385	Ccdc99
1	67630	Samd8
1	70645	Oip5
1	216760	Mfap3
1	71276	Ccdc57
1	116972	2310047D13Rik
1	229599	Gm129
1	69216	Ccdc23
1	14164	Fgf1
1	52513	Ddx56
1	106068	Slc45a4
1	74315	Rnf145
1	74012	Rap2b
1	66591	Mad2l1bp
1	71974	Prmt3

1	214642	A430107O13Rik
1	11504	Adamts1
1	80292	Zxdc
1	69028	Mitd1
1	107528	Magee1
1	NaN	MMT00072251
1	268591	Serpina5
1	621976	EG621976
1	NaN	MMT00052662
1	224170	Dzip3
1	207209	Gm317
1	209294	Csta
1	NaN	MMT00014109
1	69239	2610034M16Rik
1	NaN	MMT00004670
1	246727	Oas3
2	22697	Zscan21
2	73916	Ift57
2	14412	Slc6a13
2	70103	Znhit1
2	NaN	MMT00077995
2	58246	Slc35b4
2	67898	Pef1
2	66989	Kctd20
2	66229	Rpl7l1
2	76308	Rab1b
2	71767	Tysnd1
2	18247	Oaz2
2	18247	Oaz2
2	NaN	MMT00065790
2	74754	Dhcr24
2	71207	Nudt4
2	67830	Rer1
2	20018	Rpo1-3
2	19662	Rbp4
2	640524	Spnb5
2	77697	Mmab
2	636544	EG636544
2	NaN	MMT00058026
2	NaN	MMT00057790
2	NaN	MMT00007454
2	NaN	MMT00081411
2	NaN	MMT00050438
2	NaN	MMT00015325
2	NaN	MMT00043176
2	NaN	MMT00054594
2	NaN	MMT00038058
2	NaN	MMT00030262
2	78688	Nol3
2	194231	Cnksr1
2	11792	Apex1

2	NaN	MMT00049287
2	104175	Sbk1
2	11428	Aco1
2	NaN	MMT00073452
2	NaN	MMT00059380
2	622117	Triml2
2	NaN	MMT00057439
2	NaN	MMT00062394
2	NaN	MMT00018149
2	NaN	MMT00071096
2	NaN	MMT00062477
2	NaN	MMT00038325
2	636756	EG636756
2	NaN	MMT00030239
2	NaN	MMT00067207
2	NaN	MMT00072546
2	NaN	MMT00002048
2	NaN	MMT00074600
2	NaN	MMT00079617
2	NaN	MMT00013125
2	NaN	MMT00075578
2	NaN	MMT00002004
2	NaN	MMT00004841
2	NaN	MMT00024286
2	629557	EG629557
2	NaN	MMT00019672
2	668192	EG668192
2	NaN	MMT00065577
2	NaN	MMT00002521
2	NaN	MMT00009375
2	NaN	MMT00075271
2	NaN	MMT00026015
2	NaN	MMT00001423
2	NaN	MMT00044275
2	NaN	MMT00053707
2	NaN	MMT00057721
2	NaN	MMT00010854
2	NaN	MMT00020962
2	378954	3000002C10Rik
2	NaN	MMT00011246
2	NaN	MMT00019310
2	NaN	MMT00054062
2	NaN	MMT00057793
2	13806	Eno1
2	13806	Eno1
2	100047043	MMT00034400
2	NaN	MMT00012632
2	100040887	MMT00065461
2	NaN	MMT00027303
2	NaN	MMT00022502
2	622268	EG622268

2	94064	Mrpl27
2	52469	Ccdc56
2	66958	Txndc14
2	14867	Gstm6
2	23827	Bpnt1
2	23827	Bpnt1
2	107732	Mrpl10
2	21428	Mlx
2	NaN	MMT00020987
2	28030	Gfm1
2	98766	Ubac1
2	226849	Ppp2r5a
2	11655	Alas1
2	NaN	MMT00077003
2	NaN	MMT00029730
2	434198	B130024G19Rik
2	56506	Cib2
2	22249	Unc13b
2	68744	Zfp740
2	NaN	MMT00037083
2	15194	Htt
2	20187	Ryk
2	102626	Mapkapk3
2	114601	Ehbp1l1
2	18938	Ppp1r14b
2	18938	Ppp1r14b
2	209683	Ttc28
2	71452	Ankrd40
2	56046	Uqcc
2	17826	Mtvr2
2	70784	Rasl12
2	21665	Tdg
2	100910	2010209O12Rik
2	382014	Tmem16h
2	66039	D14Ertd449e
2	66443	Tnfaip8l1
2	17388	Mmp15
2	52588	Tspan14
2	66498	Dda1
2	11773	Ap2m1
2	16907	Lmnb2
2	17216	Mcm2
2	17768	Mthfd2
2	67963	Npc2
2	72549	Reep4
2	13423	Dnase2a
2	70231	Gorasp2
2	NaN	MMT00023332
2	20321	Frrs1
2	109225	Ms4a7
2	57317	Sfrs4

2	11758	Prdx6
2	100678	Psph
2	69456	Commd10
2	21386	Tbx3
2	68144	5031426D15Rik
2	209039	Tenc1
2	16625	Serpina3c
2	72747	2810439F02Rik
2	NaN	MMT00051367
2	13806	Eno1
2	NaN	MMT00012520
2	NaN	MMT00066041
2	407819	BC031181
2	NaN	MMT00002245
2	19261	Sirpa
2	52009	Hn1l
2	111173	Erc1
2	67486	Polr3g
2	67956	Setd8
2	101437	Dhx32
2	228889	Ddx27
2	30843	Fbxl12
2	26447	Poli
2	319801	9630033F20Rik
2	20333	Sec22b
2	NaN	MMT00033583
2	226777	C130074G19Rik
2	NaN	MMT00022996
2	21991	Tpi1
2	26570	Slc7a11
2	NaN	MMT00007996
2	NaN	MMT00041810
2	NaN	MMT00003764
2	NaN	MMT00058347
2	NaN	MMT00055891
2	22142	Tuba1a
2	22147	Tuba3b
2	NaN	MMT00079618
2	NaN	MMT00007982
2	22144	Tuba3a
2	53860	9-Sep
2	NaN	MMT00050488
2	94219	Cnnm2
2	15216	Hfe
2	75590	Dusp9
2	76281	Tax1bp3
2	NaN	MMT00060868
2	16905	Lmna
2	16976	Lrpap1
2	NaN	MMT00056938
2	NaN	MMT00046455

2	233532	Rsf1
2	58245	Gpr180
2	16151	Ikbkg
2	26433	Plod3
2	320299	Iqcb1
2	16419	Itgb5
2	110006	Gusb
2	56401	Lepre1
2	12826	Col4a1
2	14489	Mtpn
2	71254	Naif1
2	20224	Sar1a
2	16061	Igh-VJ558
2	70110	Ifi35
2	NaN	MMT00078068
2	17984	Ndn
2	68556	Uckl1
2	235441	Usp3
2	12467	Cct6b
2	18606	Enpp2
2	18823	Plp1
2	22758	Zscan12
2	12833	Col6a1
2	57432	Zc3h8
2	NaN	MMT00072901
2	16005	Igfals
2	22248	Unc119
2	666048	OTTMUSG00000008561
2	217946	Cdca7l
2	71966	Nkiras2
2	211548	Nomo1
2	67727	Stx17
2	20646	Snrpn
2	235435	Lctl
2	22129	Ttc3
2	18405	Orm1
2	68033	Cox19
2	22276	Uros
2	63955	Cables1
2	NaN	MMT00058311
2	19384	Ran
2	NaN	MMT00014274
2	NaN	MMT00048420
2	NaN	MMT00046285
2	NaN	MMT00064324
2	52874	D19Bwg1357e
2	72722	2810405J04Rik
2	68051	Nutf2
2	68051	Nutf2
2	320904	D630012G11Rik
2	68981	Snrpa1

2	NaN	MMT00008728
2	NaN	MMT00077405
2	11973	Atp6v1e1
2	68032	Tmem85
2	100040305	MMT00074121
2	66073	Txndc12
2	NaN	MMT00065558
2	432649	EG432649
2	11603	Agrn
2	67973	Mphosph10
2	20717	Serpina3m
2	19231	Ptma
2	216134	Pdxk
2	15381	Hnrnpc
2	12159	Bmp4
2	13829	Epb4.9
2	16768	Lag3
2	66112	Mosc1
2	381694	B3galtl
2	21906	Otop1
2	73458	1700055N04Rik
2	66223	Mrpl35
2	20446	St6galnac2
2	54380	Smarcal1
2	235134	Nfrkb
2	381903	Alg8
2	12741	Cldn5
2	319322	Sf3b2
2	53424	Tsnax
2	21366	Slc6a6
2	22214	Ube2h
2	56338	Txnip
2	17112	Tm4sf1
2	22433	Xbp1
2	56048	Lgals8
2	69068	1810011O10Rik
2	81489	Dnajb1
2	58185	Rsad2
2	94094	Trim34
2	209200	Dtx3l
2	15242	Hhex
2	54354	Rassf5
2	78651	Lsm6
2	71951	Gpc2
2	72895	Setd5
2	17183	Matn4
2	68037	2900093K20Rik
2	319719	4732471D19Rik
2	53627	Porcn
2	226407	Rab3gap1
2	16553	Kif13a

2	494448	Cbx6
2	67017	2010011I20Rik
2	224807	Tmem63b
2	76665	1700123M08Rik
2	242083	Ppm1l
2	16348	Invs
2	68591	Mocos
2	54411	Atp6ap1
2	56294	Ptpn9
2	24070	Mpdu1
2	68137	Kdelr1
2	76952	Nt5c2
2	20751	Spr
2	81898	Sf3b1
2	18038	Nfkbil1
2	57751	Rnf25
2	26406	Map3k3
2	63856	Taf8
2	11536	Gpr182
2	227331	Gigyf2
2	72205	Eml2
2	11883	Arsa
2	78670	Plekhj1
2	107817	Jmjd6
2	100210	Gpn2
2	23991	Cib1
2	108014	Sfrs9
2	27054	Sec23b
2	21355	Tap2
2	14972	H2-K1
2	14264	Fmod
2	15006	H2-Q1
2	15936	Ier2
2	NaN	MMT00058471
2	27883	D16H22S680E
2	52004	Cdk2ap2
2	13663	Ei24
2	30937	Lmcd1
2	74626	Tmem81
2	229615	Pias3
2	244668	Sipa1l2
2	16205	Gimap1
2	101206	Tada3l
2	20587	Smarcb1
2	71810	Ranbp3
2	209446	Tcfe3
2	330192	Vps37b
2	74479	Snx11
2	26895	Cops7b
2	59090	Midn
2	109674	Ampd2

2	192232	Hps4
2	98999	Znfx1
2	20778	Scarb1
2	217198	Plekhh3
2	26397	Map2k3
2	66711	Sbds
2	15270	H2afx
2	71667	0610007L01Rik
2	NaN	MMT00020852
2	73873	4930430E16Rik
2	228730	Gm114
2	20509	Slc19a1
2	211401	Mtss1
2	19983	Rpl5
2	627312	MMT00039341
2	623519	EG623519
2	NaN	MMT00052332
2	NaN	MMT00074658
2	NaN	MMT00067648
2	NaN	MMT00040104
2	11972	Atp6v0d1
2	56358	Copz2
2	27047	Omd
2	NaN	MMT00063568
2	NaN	MMT00043055
2	NaN	MMT00033197
2	381820	2700089E24Rik
2	71452	Ankrd40
2	16404	Itga7
2	11966	Atp6v1b2
2	80748	BC004004
2	51792	Ppp2r1a
2	16010	Igfbp4
2	13830	Stom
2	14430	Galt
2	17347	Mknk2
2	22135	Tgoln2
2	107272	Psat1
2	245638	Tbc1d8b
2	279067	MMT00046983
2	383436	EG383436
2	NaN	MMT00041940
2	NaN	MMT00066808
2	227885	MMT00013510
2	NaN	MMT00079667
2	NaN	MMT00012419
2	NaN	MMT00077883
2	NaN	MMT00069569
2	NaN	MMT00016379
2	380730	Gm884
2	NaN	MMT00003113

2	NaN	MMT00042985
2	NaN	MMT00077845
2	NaN	MMT00070318
2	16898	Rps2
2	NaN	MMT00069490
2	NaN	MMT00015290
2	NaN	MMT00049231
2	NaN	MMT00011946
2	NaN	MMT00018943
2	NaN	MMT00040516
2	14791	Emg1
2	17975	Ncl
2	28088	D10Wsu52e
2	140887	Lnx2
2	55989	Nol5
2	20661	Sort1
2	56209	Gde1
2	NaN	MMT00034813
2	237860	Ssh2
2	66510	Rnf181
2	14547	Gdap2
2	68879	Prpf6
2	14043	Ext2
2	NaN	MMT00077001
2	74106	Iqwd1
2	101113	Snx21
2	66394	Nosip
2	97159	A430005L14Rik
2	13481	Dpm2
2	29810	Bag3
2	215615	Rnpep
2	NaN	MMT00007348
2	72726	Tbcc
2	234734	Aars
2	19727	Rfxank
2	78330	1500032D16Rik
2	97484	Cog8
2	NaN	MMT00016059
2	22192	Ube2m
2	58244	Stx6
2	11544	Adprh
2	59008	Anapc5
2	57342	Parva
2	50794	Klf13
2	NaN	MMT00062390
2	NaN	MMT00044006
2	NaN	MMT00017193
2	NaN	MMT00053882
2	71795	Pitpnc1
2	226527	BC026585
2	NaN	MMT00014378

2	NaN	MMT00033789
2	17896	Myl4
2	13033	Ctsd
2	14225	Fkbp1a
2	171508	Creld1
2	109154	2410014A08Rik
2	19732	Rgl2
2	69051	Pycr2
2	217737	Ahsa1
2	67160	Eef1g
2	75751	Ipo4
2	54219	Cd320
2	NaN	MMT00034355
2	22153	Tubb4
2	NaN	MMT00015534
2	22590	Xpa
2	14976	H2-Ke2
2	27681	Snf8
2	13445	Cdk2ap1
2	NaN	MMT00038941
2	75788	Smurf1
2	192159	Prpf8
2	16865	Lgtn
2	317750	Slc24a5
2	18541	Pcnt
2	NaN	MMT00025562
2	NaN	MMT00018546
2	15013	H2-Q2
2	14964	H2-D1
2	107829	Thoc5
2	140499	Ube2j2
2	56488	Nxt1
2	628893	EG628893
2	NaN	MMT00067575
2	18632	Pex11b
2	57436	Gabarapl1
2	16412	Itgb1
2	56722	Litaf
2	18582	Pde6d
2	14042	Ext1
2	14824	Grn
2	12227	Btg2
2	14773	Grk5
2	20317	Serpinf1
2	50917	Galns
2	56356	Gltp
2	NaN	MMT00063701
2	67389	C1qdc2
2	NaN	MMT00009278
2	14337	Ftl2
2	11867	Arpc1b

2	59069	Tpm3
2	13433	Dnmt1
2	66811	Duoxa2
2	11981	Atp9a
2	228869	Ncoa5
2	67130	Ndufa6
2	57912	Cdc42se1
2	226971	Plekhb2
2	225994	BC016495
2	215418	Axud1
2	NaN	MMT00041452
2	117592	B3galt6
2	67619	Nob1
2	78834	Zfp623
2	107047	Psmg2
2	NaN	MMT00045090
2	56055	Gtpbp2
2	67513	2610002J02Rik
2	15184	Hdac5
2	105245	Txndc5
2	233833	Tnrc6a
2	80743	Vps16
2	58202	Cobra1
2	22278	Usf1
2	70101	Cyp4f16
2	109900	Asl
2	192236	Hps1
2	12616	Cenpb
2	223775	Pim3
2	21354	Tap1
2	58239	Dexi
2	NaN	MMT00070057
2	14359	Fxr1
2	140499	Ube2j2
2	18010	Neu1
2	73711	1110012M11Rik
2	12496	Entpd2
2	72325	1300018I17Rik
2	19089	Prkcsh
2	66911	Nudt16l1
2	224824	Pex6
2	72075	Ogfr
2	14114	Fbln1
2	71983	Tmco6
2	18230	Nxn
2	100040304	MMT00028325
2	56717	Frap1
2	234344	Naf1
2	76719	1700081L11Rik
2	23980	Pebp1
2	23980	Pebp1

2	27059	Sh3d19
2	381990	Zbtb2
2	75669	Pik3r4
2	70439	Taf15
2	80860	Ghdc
2	NaN	MMT00069758
2	56459	Sae1
2	107065	Lrrtm2
2	68031	Rnf146
2	117591	Slc2a9
2	103968	Plin
2	80888	Hspb8
2	245651	EG245651
2	21672	Prdx2
2	69572	Mfsd3
2	224823	BC011248
2	12892	Cpox
2	20848	Stat3
2	23871	Ets1
2	24010	Ik
2	105827	Amigo2
2	224703	2-Mar
2	18028	Nfib
2	66170	Chchd5
2	NaN	MMT00030953
2	67655	Ctdp1
2	118445	Klf16
2	214895	Lman2l
2	97212	Hadha
2	18969	Pola2
2	108705	Pttg1ip
2	93679	Trim8
2	18799	Plcd1
2	625253	EG625253
2	12927	Bcar1
2	22370	Vtn
2	66246	Osgep
2	17761	Mtap7
2	67011	Mettl6
2	11443	Chrnb1
2	13836	Epha2
2	12161	Bmp6
2	66471	Anp32e
2	100037258	Dnajc3a
2	66286	Sec11c
2	20773	Sptlc2
2	67099	2310038H17Rik
2	268465	Eme1
2	15212	Hexb
2	259003	Olfr172
2	380780	Serpina11

2	233020	Hipk4
2	668940	Myh7b
2	84682	Cox4i2
2	27007	Klrk1
2	17480	Mpl
2	384997	Pglyrp4
2	268515	Bahcc1
2	217365	Nploc4
2	11727	Ang
2	75820	Wdr64
2	83702	Akr1c6
2	381204	Naaladl1
2	54710	Hs3st3b1
2	236260	MMT00062346
2	208098	Panx3
2	13108	Cyp2g1
2	13086	Cyp2a4
2	15110	Hand1
2	15496	Hsd3b5
2	50708	Hist1h1c
2	230866	C230096C10Rik
2	67683	2610029G23Rik
2	260315	Nav3
2	NaN	MMT00048401
2	70227	Zfp619
2	74020	Cpne4
2	NaN	MMT00001620
2	14782	Gsr
2	633640	EG633640
2	12660	Chka
2	217344	Rhbdf2
2	83431	Ndel1
2	59028	Rcl1
2	110809	Sfrs1
2	237361	EG237361
2	12914	Crebbp
2	NaN	MMT00017112
2	NaN	MMT00078676
2	NaN	MMT00034706
2	66834	Them2
2	15254	Hint1
2	72729	Cdc42se2
2	19737	Rgs5
2	73251	Setd7
2	67554	Slc25a30
2	64436	Inpp5e
2	445007	Nup85
2	NaN	MMT00008599
2	69181	Dyrk2
2	56332	Amotl2
2	433759	Hdac1

2	219022	Ttc5
2	20499	Slc12a7
2	20439	Siah2
2	12748	Clk2
2	12304	Pdia4
2	21807	Tsc22d1
2	574418	RP23-433P19.11
2	433424	OTTMUSG00000012358
2	107449	Unc5b
2	76527	2010004A03Rik
2	53872	Caprin1
2	12350	Car3
2	78309	Parc
2	11991	Hnrnpd
2	110119	Mpi
2	70615	Ankrd24
2	69923	Agk
2	68514	Efha1
2	216119	A130042E20Rik
2	13498	Atn1
2	246221	Mpst
2	72454	Ccdc71
2	381921	Taok2
2	226548	Aph1a
2	14674	Gna13
2	78783	Brpf1
2	16885	Limk1
2	54645	Gripap1
2	223774	Alg12
2	230770	Tmem39b
2	105450	Mmrn2
2	286940	Flnb
2	67876	Coq10b
2	76108	Rap2a
2	170728	Rtn4ip1
2	18088	Nkx2-2
2	217194	Klhl11
2	68352	0610012D14Rik
2	71839	Osgin1
2	13117	Cyp4a10
2	NaN	MMT00057138
2	73603	1700126L10Rik
2	227377	Farp2
2	227743	Mapkap1
2	NaN	MMT00081793
2	272428	Acsm5
2	106059	A430088P11Rik
2	54150	Rdh7
2	210009	Mtrr
2	238455	4732474O15Rik
2	73730	Lce1l

2	NaN	MMT00057554
2	NaN	MMT00013155
2	114128	Laptm4b
2	277753	Cyp4a12a
2	NaN	MMT00035685
2	68034	2900009I07Rik
2	12279	C9
2	12007	Azgp1
2	71754	Cyp2d40
2	13088	Cyp2b10
2	433809	Rnf207
2	56070	Tcerg1
2	208990	Npb
2	207278	Fchsd2
2	71978	Ppp2r2a
2	57321	Terf2ip
2	NaN	MMT00051549
2	210973	Kbtbd2
2	11746	Anxa4
2	74117	Actr3
2	NaN	MMT00030928
2	242894	Actr3b
2	NaN	MMT00054980
2	NaN	MMT00053760
2	56434	Tspan3
2	23971	Papss1
2	70572	Ipo5
2	629364	MMT00061218
2	NaN	MMT00017294
2	NaN	MMT00003599
2	68092	Ncbp2
2	72183	Snx6
2	NaN	MMT00041689
2	665503	EG665503
2	114863	Prosc
2	65098	Zfand6
2	11867	Arpc1b
2	NaN	MMT00024172
2	NaN	MMT00075773
2	NaN	MMT00082708
2	238836	EG238836
2	232906	Grlf1
2	74365	Lonrf3
2	NaN	MMT00078030
2	80912	Pum1
2	59003	Maea
2	77592	4931406H21Rik
2	319266	A130010J15Rik
2	102334	Ankrd10
2	226562	Bat2d
2	67444	Ilkap

2	22631	Ywhaz
2	NaN	MMT00062045
2	11840	Arf1
2	330260	Pon2
2	56334	Tmed2
2	NaN	MMT00036534
2	NaN	MMT00001681
2	NaN	MMT00043239
2	21681	Thoc4
2	NaN	MMT00077153
2	68922	Dnaic1
2	26879	B3galnt1
2	NaN	MMT00054728
2	NaN	MMT00077065
2	73469	Rnf38
2	67255	Zfp422
2	27057	Ncoa4
2	232717	BC048599
2	228960	Stx16
2	66818	9130011J15Rik
2	225115	Svil
2	114874	Ddhd1
2	66500	Slc30a7
2	15926	Idh1
2	20522	Slc23a1
2	76302	Pcnp
2	192285	Phf21a
2	140482	Zfp358
2	55950	Bri3
2	NaN	MMT00052318
2	NaN	MMT00063804
2	11702	Amd1
2	67488	Calcoco1
2	18843	Plunc
2	26889	Cln8
2	16909	Lmo2
2	18033	Nfkb1
2	20723	Serpinb9
2	60406	Sap30
2	13197	Gadd45a
2	223752	Gramd4
2	28295	D10Jhu81e
2	28295	D10Jhu81e
2	12040	Bckdhb
2	68980	Wdr53
2	NaN	MMT00034311
2	NaN	MMT00054655
2	NaN	MMT00060113
2	NaN	MMT00014216
2	NaN	MMT00078034
2	NaN	MMT00043149

2	11465	Actg1
2	54401	Ywhab
2	22388	Wdr1
2	71853	Pdia6
2	213827	Arcn1
2	11669	Aldh2
2	232798	Leng8
2	226751	Cdc42bpa
2	22095	Tshr
2	71846	Syce2
2	13629	Eef2
2	668457	EG668457
2	22121	Rpl13a
2	620161	EG620161
2	20090	Rps29
2	258741	Olfr935
2	67023	Use1
2	77605	H2afv
2	NaN	MMT00039978
2	12389	Cav1
2	57294	Rps27
2	233893	Zfp764
2	18176	Nras
2	56013	RP23-157O10.7
2	30805	Slc22a4
2	NaN	MMT00052300
2	100087	Kti12
2	53893	Nudt5
2	110391	Qdpr
2	433923	EG433923
2	NaN	MMT00027514
2	623402	EG623402
2	30056	Timm9
2	231086	Hadhb
2	667795	EG667795
2	68904	Abhd13
2	22044	Trh
2	66290	Atp6v1g1
2	321022	Cdv3
2	12238	Commd3
2	235459	Gtf2a2
2	66684	Tceal8
2	217066	OTTMUSG00000001305
2	NaN	MMT00078790
2	19042	Ppm1a
2	69663	Ddx51
2	NaN	MMT00042876
2	19300	Abcd4
2	69151	Lzic
2	NaN	MMT00071427
2	268656	Sptlc1

2	213043	MMT00000549
2	12729	Clns1a
2	104662	Tsr1
2	80509	Med8
2	68275	Rpa1
2	192136	5033411D12Rik
2	58875	Hibadh
2	109731	Maob
2	20846	Stat1
2	NaN	MMT00014375
2	217410	Trib2
2	244198	Olfml1
2	17250	Abcc1
2	207181	Rbms3
2	226049	Dmrt2
2	66664	Tmem41a
2	NaN	MMT00002063
2	75146	Tmem180
2	212679	Mars2
2	NaN	MMT00017766
2	442832	1600029O15Rik
2	NaN	MMT00015606
2	NaN	MMT00076151
2	NaN	MMT00012906
2	625690	EG625690
2	668699	MMT00070312
2	NaN	MMT00043723
2	NaN	MMT00059232
2	NaN	MMT00069919
2	NaN	MMT00002494
2	NaN	MMT00079369
2	269941	Chsy1
2	28018	Ubfd1
2	11774	Ap3b1
2	53625	B3gnt2
2	63859	Impg1
2	51875	Tmem141
2	16651	Sspn
2	72669	2810032G03Rik
2	108960	Irak2
2	66881	Pcyox1
2	83814	Nedd4l
2	229877	Rap1gds1
2	70351	Ppp4r1
2	20620	Plk2
2	68036	Zfp706
2	22031	Traf3
2	327762	Dna2
2	65964	B230120H23Rik
2	219072	D14Ertd500e
2	NaN	MMT00042166

2	69918	2610020C07Rik
2	320846	A530058N18Rik
2	22687	Zfp259
2	17721	ND5
2	14718	Got1
2	791337	ENSMUSG00000053749
2	16582	Kifc3
2	20519	Slc22a3
2	73724	Mcee
2	17387	Mmp14
2	109934	Abr
2	71994	Cnn3
2	17955	Nap1l4
2	66439	2010012O05Rik
2	70247	Psmd1
2	66405	Mcts2
2	56369	Apip
2	NaN	MMT00014098
2	17993	Ndufs4
2	64655	Mrps22
2	664857	9330101J02Rik
2	66067	Gtpbp8
2	66736	Ttc35
2	26754	Cops5
2	57439	Tmem183a
2	67681	Mrpl18
2	66272	1810020G14Rik
2	67713	Dnajc19
2	66050	0610009B22Rik
2	76983	Scfd1
2	75778	Them4
2	58172	Sertad2
2	22627	Ywhae
2	67458	Ergic1
2	18100	Mrpl40
2	108143	Taf9
2	218629	Dhx29
2	NaN	MMT00023354
2	19651	Rbl2
2	18189	Nrxn1
2	66292	Mrps21
2	66449	MMT00011706
2	66449	MMT00023224
2	70511	5730409G15Rik
2	59046	Arpp19
2	59046	Arpp19
2	23790	Coro1c
2	15528	Hspe1
2	628438	EG628438
2	66359	2310005N03Rik
2	66477	Usmg5

2	69010	Anapc13
2	12390	Cav2
2	69574	Cmbl
2	106840	Unc119b
2	252974	C330046G03Rik
2	240411	Loxhd1
2	74201	Cep97
2	20513	Slc1a6
2	12615	Cenpa
2	12527	Cd9
2	66098	Chchd6
2	227682	Trub2
2	29858	Pmm1
2	235606	Apeh
2	15288	Hmbs
2	68971	1500001M20Rik
2	66487	Snhg8
2	108148	Galnt2
2	219158	2610301G19Rik
2	78581	D530033C11Rik
2	NaN	MMT00068616
2	664870	MMT00080766
2	230161	RP23-34B24.1
2	75497	1700008G05Rik
2	68721	1110032A03Rik
2	104384	Rhox9
2	70314	Rabep2
2	68631	Cryl1
2	104303	Arl1
2	NaN	MMT00073582
2	68877	Maf1
2	67426	Cabc1
2	225372	Apbb3
2	69754	Fbxo7
2	14132	Fcgrt
2	16362	Irf1
2	78267	Klhdc8b
2	13641	Efnb1
2	228775	Trib3
2	258734	Olfr502
2	320541	A530082C11Rik
2	383619	Aim2
2	67980	Gnpda2
2	58203	Zbp1
2	19106	Eif2ak2
2	71176	Fbxo24
2	NaN	MMT00038457
2	21928	Tnfaip2
2	99382	Abtb2
2	20541	Slc8a1
2	68917	Hint2

2	18753	Prkcd
2	234593	Ndrg4
2	76123	Gpsm2
2	56277	Tmem45a
2	18974	Pole2
2	226250	Afap1l2
2	21873	Tjp2
2	103889	Hoxb2
2	71111	Gpr39
2	66401	Nudt2
2	67184	Ndufa13
2	57423	Atp5j2
2	216767	Mrpl22
2	13823	Epb4.1l3
2	57295	Icmt
2	70564	5730469M10Rik
2	20909	Stx4a
2	67186	Rplp2
2	224860	Plcl2
2	69961	2810432D09Rik
2	55944	Eif3d
2	103841	Cuedc1
2	55983	Pdzrn3
2	29876	Clic4
2	12034	Phb2
2	20928	Abcc9
2	240219	MMT00044805
2	NaN	MMT00077385
2	11736	Ankfy1
2	67812	Ubxd2
2	68190	5330426P16Rik
2	19224	Ptgs1
2	23970	Pacsin2
2	224829	Trerf1
2	16173	Il18
2	19363	Rad51l1
2	13448	Dok1
2	21664	Phlda1
2	52065	Mfhas1
2	228769	Psmf1
2	54188	Cpsf4
2	212503	Paox
2	67388	1110008F13Rik
2	15356	Hmgcl
2	613258	A730017L22Rik
2	102247	Agpat6
2	53619	Blcap
2	67477	1300007F04Rik
2	66350	Pla2g12a
2	627022	EG627022
2	22428	Dctn6

2	NaN	MMT00001956
2	381038	Parl
2	433237	ENSMUSG00000052976
2	11539	Adora1
2	51795	Srpx
2	329941	Col8a2
2	20256	Clec11a
2	240672	Dusp5
2	23999	Twf2
2	67860	S100a16
2	140559	Igsf8
2	68087	Dcakd
2	64136	Sdf2l1
2	12317	Calr
2	16172	Il17ra
2	105785	Kdelr3
2	15507	Hspb1
2	22240	Dpysl3
2	15211	Hexa
2	384009	Glipr2
2	66111	Tmed3
2	14998	H2-DMa
2	17940	Naip1
2	69638	2310040A07Rik
2	224109	Lrrc33
2	14961	H2-Ab1
2	12798	Cnn2
2	13057	Cyba
2	18807	Pld3
2	56745	C1qtnf1
2	14159	Fes
2	14999	H2-DMb1
2	18034	Nfkb2
2	50793	Orc3l
2	68197	Ndufc2
2	19352	Rabggtb
2	227737	9130404D14Rik
2	27984	Efhd2
2	216161	Sbno2
2	66151	Prr13
2	76267	Fads1
2	240916	Vsig8
2	68636	Fahd1
2	15109	Hal
2	232201	Arhgap25
2	217303	Cd300a
2	216445	Arhgap9
2	16414	Itgb2
2	17916	Myo1f
2	74776	Ppa2
2	15900	Irf8

2	12837	Col8a1
2	12818	Col14a1
2	74761	Mxra8
2	106572	Rab31
2	20540	Slc7a7
2	71607	Snx20
2	17948	Naip2
2	18173	Slc11a1
2	71653	4930506M07Rik
2	17110	Lyz1
2	14645	Glul
2	70097	Sash1
2	68659	1110032E23Rik
2	13058	Cybb
2	26364	Cd97
2	72416	Lrpprc
2	69692	Hddc2
2	11747	Anxa5
2	109552	Sri
2	53415	Htatip2
2	225020	Fez2
2	26921	Map4k4
2	71660	Rarres2
2	102644	Oaf
2	67971	Tppp3
2	20740	Spna2
2	56430	Clip1
2	16970	Lrmp
2	14528	Gch1
2	NaN	MMT00074583
2	105787	Prkaa1
2	NaN	MMT00058392
2	11722	Amy1
2	12443	Ccnd1
2	17977	Ncoa1
2	26934	Racgap1
2	236930	Ercc6l
2	12575	Cdkn1a
2	12305	Ddr1
2	383548	Serpinb3b
2	320840	Negr1
2	18604	Pdk2
2	12408	Cbr1
2	66116	Cml1
2	14979	H2-Ke6
2	NaN	MMT00008758
2	71878	2310007D09Rik
2	18005	Nek2
2	16765	Stmn1
2	16765	Stmn1
2	18140	Uhrf1

2	66929	Asf1b
2	17218	Mcm5
2	18817	Plk1
2	619653	EG619653
2	105988	Espl1
2	12704	Cit
2	73847	5430432M24Rik
2	16922	Phyh
2	338365	Slc41a2
2	100637	N4bp2l1
2	56072	Lgals12
2	224829	Trerf1
2	77048	Ccdc41
2	66355	Gmpr
2	64294	Itm2c
2	16881	Lig1
2	50934	Slc7a8
2	22403	Wisp2
2	67863	Slc25a11
2	29805	Znhit2
2	14792	Mboat5
2	74126	Syvn1
2	13169	Dbnl
2	27632	Rdbp
2	56513	Pard6a
2	666704	Samd1
2	110253	Triobp
2	231855	C330006K01Rik
2	14337	Ftl2
2	52635	D12Ertd551e
2	29819	Stau2
2	74764	Klc4
2	66597	Trim13
2	71701	Pnpt1
2	668030	MMT00015864
2	99929	Tiparp
2	18845	Plxna2
2	117150	Pip4k2c
2	57278	Bcam
2	268490	Lsm12
2	18400	Slc22a18
2	66124	Josd2
2	20971	Sdc4
2	78697	Pus7
2	77125	Il33
2	74198	Dtx2
2	110816	Pwp2
2	110816	Pwp2
2	217995	Heatr1
2	20324	Sdpr
2	76813	Armc6

2	101612	Grwd1
2	19228	Pthr1
2	NaN	MMT00005802
2	20810	Srm
2	98970	Fibcd1
2	22083	Ctr9
2	20867	Stip1
2	72962	Tymp
2	53817	Bat1a
2	NaN	MMT00074939
2	59047	Pnkp
2	76799	2510006D16Rik
2	NaN	MMT00030605
2	70394	Kptn
2	107173	Gpr137
2	20818	Srprb
2	71713	Cdc40
2	72057	Phf10
2	68558	Ankra2
2	67230	Zfp329
2	66970	Ssbp2
2	268566	Gphn
2	69807	Trim32
2	69562	Cdc2l5
2	76485	Glt8d1
2	15353	Hmg20b
2	77733	Rnf170
2	99480	Dnttip2
2	107569	Nt5c3
2	12727	Clcn4-2
2	66707	Nkapl
2	216197	Ckap4
2	74778	1110014J01Rik
2	233863	Gtf3c1
2	673094	Cd99
2	360013	Myo18a
2	73826	Poldip3
2	52174	D4Ertd196e
2	12626	Cetn3
2	70422	Ints2
2	67457	Frmd8
2	104252	Cdc42ep2
2	74157	1300018I05Rik
2	54126	Arhgef7
2	12721	Coro1a
2	24071	Synj2bp
2	20901	Strap
2	228140	Tnks1bp1
2	71177	4933424B01Rik
2	NaN	MMT00063181
2	69697	2310057J16Rik

2	NaN	MMT00048396
2	22130	Ttf1
2	78890	2310079F23Rik
2	56215	Acin1
2	240186	Zfp438
2	237422	Ric8b
2	NaN	MMT00055028
2	56771	Med20
2	78255	Ralgps2
2	72084	Pigx
2	NaN	MMT00072579
2	69994	Rsc1a1
2	13007	Csrp1
2	71787	Trspap1
2	21388	Tbx5
2	71340	Riok1
2	433865	EG433865
2	NaN	MMT00021936
2	13043	Cttn
2	23917	Impdh1
2	54721	Tyk2
2	210148	Slc30a6
2	211770	Trib1
2	193838	Eme2
2	320118	Fbxl13
2	68170	B230118H07Rik
2	80981	Arl4d
2	67332	Snrpd3
2	NaN	MMT00021505
2	67392	4833420G17Rik
2	194655	Klf11
2	170756	Slc24a6
2	11630	Aim1
2	22324	Vav1
2	20776	Tmie
2	15018	H2-Q7
2	69717	ENSMUSG00000073403
2	57265	Fzd2
2	66071	Ethe1
2	71093	Atoh8
2	170942	Erdr1
2	73274	Gpbp1
2	67738	Ppid
2	NaN	MMT00008178
2	15519	Hsp90aa1
2	18415	Hspa4l
2	NaN	MMT00071061
2	620986	EG620986
2	68240	Rpa3
2	105148	Iars
2	52633	Nit2

2	218335	Clptm1l
2	66645	Pspc1
2	18968	Pola1
2	NaN	MMT00022168
2	67048	2610030H06Rik
2	72612	2700029M09Rik
2	353208	2810021G02Rik
2	66671	Ccnh
2	67894	1810055E12Rik
2	77574	3321401G04Rik
2	NaN	MMT00067115
2	NaN	MMT00074659
2	68099	6720467C03Rik
2	66857	1100001H23Rik
2	16548	Khk
2	12334	Capn2
2	110962	Mbd6
2	66993	Smarcd3
2	77031	Slc9a8
2	226154	Lzts2
2	14870	Gstp1
2	319899	Dock6
2	14254	Flt1
2	70435	2610204M08Rik
2	268470	Ube2z
2	239691	AU021092
2	328280	EG328280
2	14595	B4galt1
2	12764	Cmas
2	67151	Psmd9
2	NaN	MMT00011281
2	24116	Whsc2
2	17184	Matr3
2	218811	Sec24c
2	234730	Fuk
2	16449	Jag1
2	27041	G3bp1
2	67169	Nradd
2	NaN	MMT00081444
2	66385	Ppp1r7
2	24055	Sh3bp2
2	78560	Gpr124
2	16906	Lmnb1
2	12018	Bak1
2	432763	Prr7
2	65106	Arl6ip5
2	69544	Wdr5b
2	77809	Lrrc42
2	NaN	MMT00068622
2	15469	Prmt1
2	20595	Smn1

2	20641	Snrpd1
2	50927	Nasp
2	107568	Wwp1
2	23857	Dmtf1
2	13667	Eif2b4
2	78889	Wsb1
2	26450	Rbbp9
2	619677	4930529C04Rik
2	233908	Fus
2	76022	5830417I10Rik
2	67755	Ddx47
2	74194	Rnd3
2	66156	Anapc11
2	67706	Tmem179b
2	14823	Grm8
2	170758	Rac3
2	16923	Sh2b3
2	19679	Pitpnm2
2	18036	Nfkbib
2	93892	Pcdhb21
2	76510	Trappc9
2	15258	Hipk2
2	67157	2610301B20Rik
2	107503	Atf5
2	55992	Trim3
2	66404	2410001C21Rik
2	625253	EG625253
2	69752	Zfp511
2	216963	Git1
2	102339	Cog4
2	20024	Sub1
2	56310	Gps2
2	66680	3230401D17Rik
2	66680	3230401D17Rik
2	78038	Mccc2
2	100226	Stx12
2	378460	Pram1
2	57344	As3mt
2	211286	Cln5
2	76663	1700123I01Rik
2	212862	Chpt1
2	100044551	MMT00001823
2	320661	D5Ertd579e
2	98932	Myl9
2	75452	Ascc2
2	15944	Irgm
2	17957	Napb
2	16409	Itgam
2	14696	Gnb4
2	12751	Tpp1
2	103967	Dnm3

2	NaN	MMT00052315
2	76539	D19Ertd737e
2	68652	Map3k7ip2
2	68146	Arl13b
2	433375	Creg1
2	227334	Usp40
2	78523	Mrpl9
2	30946	Abt1
2	12877	Cpeb1
2	69288	Rhobtb1
2	23881	G3bp2
2	229227	4932438A13Rik
2	16542	Kdr
2	NaN	MMT00077789
2	66307	Isoc1
2	NaN	MMT00002151
2	71723	Dhx34
2	15490	Hsd17b7
2	NaN	MMT00038380
2	NaN	MMT00077590
2	56351	Ptges3
2	13171	Dbt
2	16573	Kif5b
2	67075	Magt1
2	56217	Mpp5
2	18706	Pik3ca
2	NaN	MMT00004929
2	NaN	MMT00004959
2	546271	MMT00047275
2	105351	AW209491
2	70428	Polr3b
2	29875	Iqgap1
2	13618	Ednrb
2	211329	Ncoa7
2	24136	Zeb2
2	26416	Mapk14
2	192185	Nadk
2	93742	Pard3
2	70796	Zdhhc1
2	213988	Tnrc6b
2	12847	Copa
2	65246	Xpo7
2	NaN	MMT00065667
2	18986	Pou2f1
2	77578	Bcl9
2	16179	Irak1
2	107566	Arl2bp
2	NaN	MMT00030937
2	NaN	MMT00054025
2	NaN	MMT00032606
2	666581	RP23-162H3.1

2	NaN	MMT00001646
2	NaN	MMT00017950
2	NaN	MMT00053547
2	NaN	MMT00069653
2	NaN	MMT00044489
2	76482	3110002H16Rik
2	54409	Ramp2
2	66642	Ctnnbl1
2	17128	Smad4
2	NaN	MMT00082897
2	66407	Mrps15
2	NaN	MMT00050551
2	NaN	MMT00001695
2	224044	Cyp2ab1
2	53611	Vti1a
2	233064	Wdr62
2	216792	A230051G13Rik
2	66905	M6prbp1
2	109299	C330006A16Rik
2	19165	Psen2
2	74173	1700012B15Rik
2	67151	Psmd9
2	NaN	MMT00022553
2	231931	Gimap6
2	66066	Gng11
2	107239	AI790298
2	100669	9930105H17Rik
2	68157	6720475J19Rik
2	116701	Fgfrl1
2	382421	EG382421
2	NaN	MMT00078167
2	NaN	MMT00044154
2	NaN	MMT00008110
2	66999	Med28
2	85305	Kars
2	NaN	MMT00036264
2	16898	Rps2
2	625055	EG625055
2	546164	EG546164
2	NaN	MMT00065672
2	NaN	MMT00069688
2	243642	EG243642
2	NaN	MMT00066059
2	677044	ENSMUSG00000074250
2	NaN	MMT00000408
2	NaN	MMT00043750
2	NaN	MMT00005808
2	NaN	MMT00016905
2	NaN	MMT00060803
2	NaN	MMT00039476
2	NaN	MMT00082247

2	NaN	MMT00002524
2	NaN	MMT00045078
2	73830	Eif3k
2	NaN	MMT00017268
2	NaN	MMT00000356
2	625752	RP23-54E4.8
2	NaN	MMT00019254
2	NaN	MMT00004656
2	NaN	MMT00036668
2	NaN	MMT00061243
2	NaN	MMT00046046
2	NaN	MMT00026738
2	NaN	MMT00017270
2	NaN	MMT00017503
2	NaN	MMT00005190
2	55978	Ift20
2	20022	Polr2j
2	68565	Mrps18a
2	NaN	MMT00043412
2	NaN	MMT00073158
2	NaN	MMT00025220
2	68017	Ftsj2
2	54196	Pabpn1
2	NaN	MMT00078932
2	66293	1810032O08Rik
2	NaN	MMT00075644
2	NaN	MMT00015578
2	NaN	MMT00015399
2	NaN	MMT00080853
2	675985	MMT00081973
2	NaN	MMT00044478
3	11814	Apoc3
3	71943	Tom1l1
3	54384	Mtmr7
3	194974	Sunc1
3	626870	OTTMUSG00000005148
3	20539	Slc7a5
3	69719	Cad
3	13924	Ptprv
3	99031	Osbpl6
3	76866	Morn1
3	27276	Plekhb1
3	54357	Epb4.1l4b
3	11735	Ank3
3	17153	Mal
3	55987	Cpxm2
3	56461	Kcnip3
3	13384	Mpp3
3	232232	Hdac11
3	100039284	MMT00029319
3	21401	Tcea3

3	18552	Pcsk5
3	27260	Plek2
3	226245	9930023K05Rik
3	381149	Nrg2
3	66809	Krt20
3	13052	Cxadr
3	12349	Car2
3	11931	Atp1b1
3	14933	Gyk
3	68588	Cthrc1
3	213539	Bag2
3	54725	Cadm1
3	12296	Cacnb2
3	212980	Slc45a3
3	70887	Dmrtc1a
3	68283	9530077C05Rik
3	71877	Efhc1
3	57738	Slc15a2
3	434778	6330534C20Rik
3	66952	2310030G06Rik
3	12638	Cftr
3	12695	Inadl
3	64058	Perp
3	59058	Bhlhb5
3	381126	Gm944
3	77411	Rbm35b
3	107586	Ovol2
3	54326	Elovl2
3	94224	Srd5a2
3	78925	Srd5a1
3	17777	Mttp
3	208213	Tmem132c
3	224997	Dlgap1
3	20698	Sphk1
3	14385	Slc37a4
3	26944	Tinag
3	68509	1110018H23Rik
3	18082	Nipsnap1
3	100072	Camta1
3	268723	A830039N20Rik
3	226999	Slc9a2
3	17349	Mlf1
3	52187	Rragd
3	73284	Ddit4l
3	69121	Chrdl2
3	21956	Tnnt2
3	68854	Asb11
3	69354	Slc38a4
3	51801	Ramp1
3	234671	Ces2
3	71911	Bdh1

3	20377	Sfrp1
3	75104	Mmd2
3	26559	Hunk
3	268527	MMT00036029
3	380654	4930485B16Rik
3	12684	Cideb
3	14866	Gstm5
3	14609	Gja1
3	226419	Dyrk3
3	11815	Apod
3	15186	Hdc
3	12162	Bmp7
3	75600	Calml4
3	18719	Pip5k1b
3	19215	Ptgds
3	230779	Serinc2
3	230459	Cyp2j13
3	16763	Lad1
3	16525	Kcnk1
3	106648	Cyp4f15
3	53624	Cldn7
3	56089	Ramp3
3	224344	Rbm11
3	332934	Zmynd12
3	224912	Crb3
3	110902	Chrna2
3	68567	Cgref1
3	22092	Rsph1
3	30956	Aass
3	66889	Rnf128
3	66717	Ccdc96
3	19126	Prom1
3	244653	Hydin
3	215723	BC024997
3	224079	Atp13a4
3	338417	Scgb1c1
3	218630	Ccno
3	103712	6330403K07Rik
3	380653	Gm872
3	73608	Marveld3
3	16372	Irx2
3	14425	Galnt3
3	104362	Meig1
3	67344	Tctex1d1
3	56222	Cited4
3	20732	Spint1
3	57394	Tmem27
3	75434	1700001C02Rik
3	72185	Dbndd1
3	72236	Tsnaxip1
3	27375	Tjp3

3	18741	Pitx2
3	94180	Acsbg1
3	217328	Myo15b
3	12643	Chad
3	102502	AI427122
3	11847	Arg2
3	67085	1700024G13Rik
3	54562	Lrrc6
3	54120	Gipc2
3	208613	E030011K20Rik
3	71860	Wdr16
3	244608	Ccdc113
3	117229	Stk33
3	19193	Pipox
3	207920	Rbm35a
3	72022	Slc35f2
3	381738	Gm1060
3	69329	1700003M02Rik
3	22349	Vil1
3	192120	Bspry
3	68233	1700125D06Rik
3	327954	Dnahc2
3	207742	Rnf43
3	69073	1810019J16Rik
3	53419	Corin
3	20517	Slc22a1
3	71233	4933434I06Rik
3	19123	Proc
3	75533	Nme5
3	74665	Lrrc48
3	230822	A330049M08Rik
3	15451	Hpn
3	192734	AI646023
3	381260	Gm973
3	14786	Grb7
3	75472	1700009P17Rik
3	11542	Adora3
3	75429	3100002J23Rik
3	237320	Aldh8a1
3	24057	Sh3yl1
3	74053	Grip1
3	71062	Tekt3
3	68344	Tmem174
3	75426	Igfbpl1
3	76261	0610040J01Rik
3	403205	Agr3
3	71227	Wdr69
3	74711	Ttll9
3	12614	Celsr1
3	66104	Tceal6
3	207686	A330021E22Rik

3	14725	Lrp2
3	14121	Fbp1
3	70882	Armc3
3	73332	1700041C02Rik
3	330721	Nek5
3	19716	Bex1
3	226356	Gm101
3	77996	D730039F16Rik
3	13661	Ehf
3	18705	Pik3c2g
3	26458	Slc27a2
3	75607	Wnk2
3	353287	MMT00032844
3	17075	Tacstd1
3	17427	Mns1
3	104601	Mycbpap
3	14120	Fbp2
3	13105	Cyp2d9
3	218739	A430083B19Rik
3	102680	Slc6a20a
3	20464	Sim1
3	13370	Dio1
3	18171	Nr1i2
3	75568	Capsl
3	107146	Glyat
3	75495	1700010A17Rik
3	330355	Dnahc6
3	66930	Fank1
3	170790	Mlc1
3	320214	4932425I24Rik
3	77522	D630002J15Rik
3	76509	1600029D21Rik
3	21645	Tcte1
3	75556	1700026D08Rik
3	320159	4632412N22Rik
3	12069	Bex2
3	66825	Rnf186
3	106347	Ildr1
3	72088	Ush1c
3	632778	EG632778
3	71200	Dydc2
3	225266	Klhl14
3	71870	Ccdc19
3	58210	Sectm1b
3	75465	Dynlrb2
3	78801	Ak7
3	74934	Armc4
3	50525	Spag6
3	230163	Aldob
3	70405	Calml3
3	217138	Atad4

3	17393	Mmp7
3	70337	Iyd
3	67082	1700011H14Rik
3	59020	Pdzk1
3	15223	Foxj1
3	14598	Ggt1
3	12556	Cdh16
3	16433	Cuzd1
3	75040	4930504H06Rik
3	229302	Tm4sf4
3	30962	Slc7a9
3	69453	1700027L20Rik
3	12587	Mia1
3	72040	Mupcdh
3	69325	1700012B09Rik
3	69083	Sult1c2
3	72058	Igsf5
3	16006	Igfbp1
3	13511	Dsg2
3	212517	Wdr52
3	11514	Adcy8
3	210762	Gm70
3	78088	Ankrd56
3	330671	B4galnt4
3	381693	4930434E21Rik
3	16517	Kcnj16
3	66758	Zfp474
3	237806	Dnahc9
3	75784	1700007G11Rik
3	13487	Slc26a3
3	67758	Aadac
3	76589	Unc5cl
3	16591	Kl
3	93835	Amn
3	102448	Xylb
3	68396	Cml4
3	242253	Wdr63
3	75172	4930528G09Rik
3	13884	Es1
3	71313	Fsip1
3	171531	Mlph
3	67701	Wfdc2
3	12550	Cdh1
3	242646	Tctex1d4
3	319530	Zfp750
3	69787	Anxa13
3	71827	Lrrc34
3	78016	4930511H11Rik
3	71345	Tmem16j
3	22062	Trp73
3	110351	Rap1gap

3	226243	Habp2
3	22262	Uox
3	50528	Tmprss2
3	66451	2610528J11Rik
3	70853	4921511C04Rik
3	64406	Sp5
3	76484	Kndc1
3	17863	Myb
3	109620	Dsp
3	207778	Dnahc7l
3	19733	Rgn
3	72249	1700019G24Rik
3	13491	Drd4
3	231832	Tmem184a
3	76974	1190003J15Rik
3	66620	2610207O16Rik
3	96935	Susd4
3	81840	Sorcs2
3	103655	Sec14l4
3	17922	Myo7b
3	67182	Pdzk1ip1
3	19683	Rdh16
3	26448	Rage
3	14620	Gjb3
3	57816	Tesc
3	14275	Folr1
3	19143	St14
3	72315	2310015A05Rik
3	433904	Ociad2
3	20686	Spa17
3	216867	Slc16a11
3	20276	Scnn1a
3	12739	Cldn3
3	209012	Ulk4
3	16977	Lrrc23
3	170833	Hook2
3	52589	Ncald
3	16772	Lama1
3	226352	Epb4.1l5
3	73750	Whrn
3	228846	D630003M21Rik
3	240753	Plekha6
3	208943	Myo5c
3	58233	Dnaja4
3	52331	Stbd1
3	212733	Ccdc64b
3	58802	Kcnmb4
3	72930	Ppp2r2b
3	58187	Cldn10
3	54139	Irf6
3	26897	Acot1

3	209195	Clic6
3	66748	4933404M02Rik
3	18861	Pms2
3	432720	Akr1c19
3	73287	1700040L02Rik
3	230959	Ajap1
3	71687	Tmem25
3	71661	0610005C13Rik
3	13867	Erbb3
3	16323	Inhba
3	58226	Cacna1h
3	594844	Tceal3
3	269152	Kif26b
3	107587	Osr2
3	12070	Ngfrap1
3	230972	Arhgef16
3	232670	Tspan33
3	74424	Tmc5
3	12630	Cfi
3	654824	Ankrd37
3	242584	Wdr78
3	69312	4930418G15Rik
3	320752	Dpy19l2
3	140919	Slc17a6
3	26414	Mapk10
3	212516	BC060267
3	57355	BC051019
3	71354	Wdr31
3	209837	Slc38a5
3	319832	6332401O19Rik
3	70358	Steap1
3	270192	Rab6b
3	12560	Cdh3
3	50759	Fbxo16
3	53315	Sult1d1
3	93732	Acox2
3	331532	Tceal5
3	56185	Hao3
3	215772	9130014G24Rik
3	213649	Arhgef19
3	27281	Hrasls
3	73332	1700041C02Rik
3	72074	Anks4b
3	64103	Tnmd
3	14422	B4galnt2
3	16869	Lhx1
3	216019	Hkdc1
3	114662	Prss29
3	140709	Emid2
3	20531	Slc34a2
3	78286	Nav2

3	69894	2010107G23Rik
3	73712	Dmkn
3	72027	Slc39a4
3	16668	Krt18
3	NaN	MMT00053383
3	16691	Krt8
3	80720	Pbx4
3	434872	Usp51
3	69297	Lrrc46
3	11522	Adh1
3	12454	Ccnk
3	20733	Spint2
3	15114	Hap1
3	213208	Il20rb
3	230872	Crocc
3	53972	Ngef
3	56460	Pkp3
3	83674	Cnnm1
3	13800	Enah
3	224022	Slc7a4
3	110084	Dnahc1
3	18600	Padi2
3	98845	Eps8l2
3	235611	Plxnb1
3	207175	Cetn4
3	545551	BC021767
3	12817	Col13a1
3	18546	Pcp4
3	80883	Ntng1
3	67815	Sec14l2
3	18654	Pgf
3	232714	Mgam
3	211949	Spsb4
3	69219	Ddah1
3	21689	Tekt1
3	74596	Cds1
3	72778	2810451A06Rik
3	195208	Dcdc2a
3	74284	1700086L19Rik
3	278240	Spin2
3	71584	Gdpd2
3	217887	BC022687
3	16782	Lamc2
3	75767	Rab11fip1
3	69097	Trim15
4	72121	Dennd2d
4	17171	Mas1
4	NaN	MMT00031273
4	277744	Gm694
4	21419	Tcfap2b
4	258737	Olfr1316

4	347708	Dppa1
4	NaN	MMT00081414
4	244653	Hydin
4	76484	Kndc1
4	77619	Prelid2
4	18012	Neurod1
4	NaN	MMT00008227
4	NaN	MMT00039783
4	14171	Fgf17
4	193740	Hspa1a
4	12631	Cfl1
4	NaN	MMT00058578
4	NaN	MMT00003579
4	19273	Ptpru
4	12372	Casq1
4	29877	Hdgfrp3
4	245631	Mum1l1
4	16351	Ipp
4	22715	Zfp57
4	258985	Olfr1256
4	224661	Slc26a8
4	NaN	MMT00013609
4	23963	Odz1
4	258805	Olfr1426
4	NaN	MMT00046595
4	258540	Olfr771
4	70846	4921506M07Rik
4	NaN	MMT00055444
4	103978	Gpc5
4	80976	Syt13
4	212933	Pm20d1
4	140488	Igf2bp3
4	NaN	MMT00081874
4	NaN	MMT00015366
4	23985	Slc26a4
4	78508	2900093L17Rik
4	NaN	MMT00050594
4	11628	Aicda
4	258816	Olfr466
4	NaN	MMT00072975
4	381236	AI747699
4	218066	Olfr11
4	319800	C730048C13Rik
4	70789	Kynu
4	76654	Upp2
4	215446	Entpd3
4	666279	Dspp
4	241134	9430031J16Rik
4	228564	Frmd5
4	NaN	MMT00071794
4	619332	4933416C03Rik

4	NaN	MMT00054229
4	NaN	MMT00063855
4	NaN	MMT00023460
4	14183	Fgfr2
4	56293	Amac1
4	15043	H2-T3
4	NaN	MMT00059956
4	76858	Nlrp14
4	108989	Tpr
4	19076	Prim2
4	235040	Atg4d
4	26893	Cops6
4	NaN	MMT00056390
4	619326	9130409I23Rik
4	100855	Tbc1d14
4	15424	Hoxc5
4	NaN	MMT00048506
4	74591	Abca12
4	NaN	MMT00043469
4	12945	Dmbt1
4	14179	Fgf8
4	NaN	MMT00070692
4	NaN	MMT00047250
4	56278	Gkap1
4	69993	Chn2
4	NaN	MMT00003854
4	26413	Mapk1
4	246079	Defb9
4	NaN	MMT00007615
4	NaN	MMT00081758
4	78115	4930445E18Rik
4	258731	Olfr491
4	117066	Cts3
4	243085	Ugt2b35
4	73360	Actrt1
4	258686	Olfr1467
4	258498	Olfr148
4	83703	Dbr1
4	240539	Gm336
4	380842	Gm1574
4	627004	MMT00000775
4	15112	Hao1
4	638345	Sbpl
4	329872	Frem1
4	237353	Sh3md4
4	117066	Cts3
4	329324	Syt14
4	12737	Cldn1
4	235281	Scn3b
4	320502	Lmod3
4	109820	Pgc

4	21823	Th
4	67622	Mxra7
4	12295	Cacnb1
4	14177	Fgf6
4	17380	Mme
4	67645	4930511I11Rik
4	67931	Serpini2
4	22296	V1ra1
4	106369	Ypel1
4	20621	Snn
4	NaN	MMT00048540
4	16530	Kcnk7
4	14563	Gdf5
4	53606	Isg15
4	677168	MMT00057550
4	667370	MMT00014813
4	50769	Atp8a2
4	108803	4933402P03Rik
4	237877	Atad5
4	72103	2010301N04Rik
4	67358	1700093K21Rik
4	217316	Slc16a5
4	16447	Ivl
4	54393	Gabbr1
4	NaN	MMT00011639
4	70365	Speer5-ps1
4	NaN	MMT00017420
4	381818	EG381818
4	NaN	MMT00028069
4	74068	Asz1
4	NaN	MMT00077133
4	21418	Tcfap2a
4	NaN	MMT00076852
4	258776	Olfr715
4	70571	Tcerg1l
4	626058	EG626058
4	73332	1700041C02Rik
4	20819	Srst
4	105278	Ccrk
4	76757	Trdn
4	117149	Tirap
4	69769	Tnfaip8l2
4	213765	RP23-336F11.32
4	11839	Areg
4	68732	Lrrc16a
4	192663	Abcg4
4	73330	1700018P08Rik
4	NaN	MMT00079364
4	74362	Spag17
4	215654	Cdh12
4	NaN	MMT00030135

4	75532	1700015C15Rik
4	434130	MMT00042294
4	NaN	MMT00011949
4	18150	Npm3
4	53902	Rcan3
4	NaN	MMT00004371
4	14811	Grin2a
4	71011	4933401B06Rik
4	20339	Sele
4	NaN	MMT00015505
4	258610	Olfr307
4	11731	Ang2
4	16192	Il5ra
4	67653	4930544G11Rik
4	NaN	MMT00000111
4	320923	Mtap7d3
4	22634	Plagl1
4	628850	6030426L16Rik
4	94220	Cnnm4
4	17143	Magea7
4	NaN	MMT00064177
4	67781	Ilf2
4	100041004	MMT00052718
4	231045	4931409K22Rik
4	NaN	MMT00015015
4	NaN	MMT00026784
4	75547	Akap13
4	NaN	MMT00030946
4	223642	Zc3h3
4	19724	Rfx1
4	328949	Mcc
4	64934	Pes1
4	18441	P2ry1
4	NaN	MMT00021912
4	75645	1700011F14Rik
4	241528	Lrrc55
4	14459	Gast
4	66323	1700001K19Rik
4	11519	Add2
4	57315	Wdr46
4	NaN	MMT00079465
4	66343	Tmem177
4	NaN	MMT00063357
4	56735	Krt71
4	620205	EG620205
4	NaN	MMT00049822
4	60367	Il1rapl2
4	230753	Thrap3
4	83428	Ucn3
4	268686	S100z
4	269338	Vps39

4	382217	Gm1140
4	320982	Arl4c
4	16181	Il1rn
4	107476	Acaca
4	331392	EG331392
4	26356	Ing1
4	12417	Cbx3
4	28194	Apon
4	NaN	MMT00072359
4	353047	Plekhm1
4	320869	4732415M23Rik
4	235497	Leo1
4	76508	2210015D19Rik
4	NaN	MMT00000014
4	67050	Nkap
4	11448	Chrne
4	16415	Itgb2l
4	11720	Mat1a
4	52250	Reep1
4	22036	Traip
4	74914	Cylc2
4	170741	Pilrb1
4	NaN	MMT00054498
4	71162	4933421I07Rik
4	26877	B3galt1
4	NaN	MMT00052951
4	73407	1700055M20Rik
4	77998	Grifin
4	75510	1700023D19Rik
4	235582	Glyctk
4	83965	Enpp5
4	18559	Pctp
4	269902	Vmn2r57
4	16162	Il12rb2
4	110956	D17H6S56E-5
4	240832	Tor1aip2
4	NaN	MMT00057992
4	16538	Kcns1
4	18191	Nrxn3
4	329977	Fhad1
4	107831	Bai1
4	72097	2010300C02Rik
4	72016	1600002H07Rik
4	56697	Akap10
4	66700	Vps24
4	NaN	MMT00009771
4	97423	R74862
4	28042	D5Wsu178e
4	15426	Hoxc8
4	235041	Kank2
4	NaN	MMT00046383

4	17842	Mup3
4	20445	St6galnac1
4	NaN	MMT00077009
4	76426	1700029J11Rik
4	NaN	MMT00020895
4	320051	Exph5
4	73358	1700054M17Rik
4	NaN	MMT00010260
4	244329	Mcph1
4	320181	Fndc7
4	232925	Igfl3
4	72258	Kcnk10
4	320808	Wdr22
4	22420	Wnt6
4	231760	Rimbp2
4	57257	Vav3
4	74071	Ifltd1
4	81012	V1rd7
4	NaN	MMT00072454
4	231201	AF366264
4	NaN	MMT00004313
4	230895	Vps13d
4	NaN	MMT00031355
4	244646	Pkd1l3
4	21949	Tnfsf8
4	107476	Acaca
4	381535	RP23-149D11.4
4	NaN	MMT00030044
4	74031	4632413I24Rik
4	219094	9130227C08Rik
4	53620	Vamp5
4	100009609	Vmn2r65
4	20666	Sox11
4	NaN	MMT00007643
4	72333	Palld
4	18040	Nefm
4	14417	Gad2
4	73795	4930405D01Rik
4	270004	Foxi2
4	56092	Cts7
4	236904	Klhl15
4	59310	Mylc2pl
4	NaN	MMT00008587
4	72821	Scn2b
4	59041	Stk25
4	83602	Gtf2a1
4	74092	0610038L08Rik
4	434446	Ccdc13
4	NaN	MMT00016293
4	14178	Fgf7
4	22390	Wee1

4	17086	Ncr1
4	100737	Dcun1d4
4	100383	Bsdc1
4	26430	Parg
4	270110	Irf2bp2
4	65973	Asph
4	21453	Tcof1
4	75500	1700010H22Rik
4	243548	Prickle2
4	215001	Wfikkn1
4	71745	Cul2
4	328845	Acsbg2
4	214444	Cdk5rap2
4	330177	Taok3
4	20429	Shox2
4	94176	Dock2
5	21926	Tnf
5	80891	Fcrls
5	24088	Tlr2
5	52855	Lair1
5	14744	Gpr65
5	12045	Bcl2a1b
5	21897	Tlr1
5	20302	Ccl3
5	20303	Ccl4
5	16408	Itgal
5	16153	Il10
5	17167	Marco
5	50498	Ebi3
5	12481	Cd2
5	17474	Clec4d
5	14058	F10
5	20208	Saa1
5	20210	Saa3
5	12267	C3ar1
5	381319	Batf3
5	12517	Cd72
5	11629	Aif1
5	246256	Fcgr4
5	108670	Epsti1
5	236451	EG236451
5	231655	Oasl1
5	14129	Fcgr1
5	64380	Ms4a4c
5	15945	Cxcl10
5	17079	Cd180
5	217151	Arl5c
5	12766	Cxcr3
5	239827	Pigz
5	215900	A630077B13Rik
5	242248	Bank1

5	12140	Fabp7
5	21426	Tcfec
5	217370	BC017643
5	276891	Timd4
5	211577	Mrgprf
5	276905	Armc7
5	17937	Nab2
5	71946	Endod1
5	67689	Aldh3b1
5	13845	Ephb3
5	212937	Tifab
5	14347	Fut7
5	13030	Ctsb
5	210029	Metrnl
5	74127	Krt80
5	99151	Cercam
5	27419	Naglu
5	68337	Crip2
5	17210	Mcl1
5	14038	Kal1
5	11857	Arhgdib
5	27261	Dok3
5	70785	Dennd1c
5	104759	Pld4
5	17095	Lyl1
5	19253	Ptpn18
5	106512	Gpsm3
5	70719	Hmha1
5	102657	Cd276
5	19013	Ppara
5	56454	Aldh18a1
5	230766	BC030183
5	12051	Bcl3
5	14131	Fcgr3
5	12982	Csf2ra
5	230787	BC013712
5	213002	Ifitm6
5	17064	Cd93
5	17758	Mtap4
5	14710	Gngt2
5	56198	Heyl
5	71817	Tmem50a
5	17449	Mdh1
5	102595	Plekho2
5	19025	Ctsa
5	15361	Hmga1
5	15361	Hmga1
5	19698	Relb
5	223701	Mkl1
5	69612	2310037I24Rik
5	NaN	MMT00009927

5	217082	Hlf
5	19240	Tmsb10
5	64138	Ctsz
5	21391	Tbxas1
5	12259	C1qa
5	12978	Csf1r
5	52055	Rab11fip5
5	54445	Unc93b1
5	14276	Folr2
5	12260	C1qb
5	12262	C1qc
5	18636	Cfp
5	56489	Ikbke
5	16949	Loxl1
5	216725	Adamts2
5	21827	Thbs3
5	107581	Col16a1
5	21857	Timp1
5	18542	Pcolce
5	20204	Prrx2
5	56532	Ripk3
5	56742	Psrc1
5	85031	Pla1a
5	214425	Cilp
5	76905	Lrg1
5	18611	Pea15a
5	13555	E2f1
5	407790	Ndufa4l2
5	378702	Serf2
5	378702	Serf2
5	11772	Ap2a2
5	12831	Col5a1
5	14457	Gas7
5	14314	Fstl1
5	14118	Fbn1
5	68792	Srpx2
5	18301	Fxyd5
5	13449	Dok2
5	13732	Emp3
5	246707	Emilin2
5	227659	Slc2a6
5	72042	Cotl1
5	73723	Sh3bgrl3
5	234779	Plcg2
5	16331	Inpp5d
5	20963	Syk
5	232536	Mrps35
5	15483	Hsd11b1
5	242341	Atp6v0d2
5	14068	F7
5	14960	H2-Aa

5	231842	Amz1
5	100038514	OTTMUSG00000004551
5	12332	Capg
5	217303	Cd300a
5	98878	Ehd4
5	22029	Traf1
5	56857	Slc37a2
5	14962	Cfb
5	68318	Aph1c
5	20379	Sfrp4
5	171207	Arhgap4
5	15162	Hck
5	19354	Rac2
5	15170	Ptpn6
5	22376	Was
5	212032	Hk3
5	65221	Slc15a3
5	17084	Ly86
5	98365	Slamf9
5	73149	Clec4a3
5	13040	Ctss
5	11810	Apobec1
5	80719	Igsf6
5	226421	5430435G22Rik
5	67865	Rgs10
5	74131	Sash3
5	27056	Irf5
5	105855	Nckap1l
5	108101	Fermt3
5	17972	Ncf4
5	233571	P2ry6
5	14127	Fcer1g
5	269799	Clec4a1
5	22177	Tyrobp
5	74748	Slamf8
5	NaN	MMT00042387
5	66824	Pycard
5	231805	Pilra
5	211228	Lrrc25
5	56744	Pf4
5	17476	Mpeg1
5	14017	Evi2a
5	223433	BC052328
5	216984	Evi2b
5	19277	Ptpro
5	16822	Lcp2
5	67742	Samsn1
5	14115	Fbln2
5	12832	Col5a2
5	19141	Lgmn
5	54519	Apbb1ip

5	17025	Alad
5	230815	Man1c1
5	76088	Dock8
5	56213	Htra1
5	11690	Alox5ap
5	107373	4632417K18Rik
5	20305	Ccl6
5	19017	Ppargc1a
5	19038	Ppic
5	12523	Cd84
5	226422	Rab7l1
5	66815	Ccdc109b
5	217826	Kcnk13
5	69573	2310016C08Rik
5	20868	Stk10
5	22411	Wnt11
5	19222	Ptgir
5	233186	Siglec5
5	50778	Rgs1
5	16197	Il7r
5	18484	Pam
5	76477	Pcolce2
5	381924	Itgad
5	16411	Itgax
5	11501	Adam8
5	17381	Mmp12
5	171285	Havcr2
5	24108	Ubd
5	54483	Mefv
5	19267	Ptpre
5	667386	MMT00066614
5	14049	Eya2
5	54132	Pdlim1
5	54132	Pdlim1
5	72599	Pdia5
5	229600	BC028528
5	63986	Gmfg
5	74145	F13a1
5	NaN	MMT00082285
5	14137	Fdft1
5	330064	Slc5a6
5	14283	Fosl1
5	20310	Cxcl2
5	14825	Cxcl1
5	625360	MMT00062748
5	NaN	MMT00053742
5	17970	Ncf2
5	NaN	MMT00001732
5	22751	Zfp90
5	56045	Samhd1
5	331004	Slc9a9

5	17312	Mgl1
5	69146	Gsdmd
5	66775	Ptplad2
5	210530	Leprel1
5	68119	Cmtm3
5	12268	C4b
5	20719	Serpinb6a
5	56485	Slc2a5
5	231440	9130213B05Rik
5	140795	P2ry14
5	224530	Acat3
5	19073	Srgn
5	19128	Pros1
5	330723	Htra4
5	55984	Camkk1
5	233752	Insc
5	320078	Olfml2b
5	171286	Slc12a8
5	98363	Efhd1
5	70717	6330406I15Rik
5	68655	Fndc1
5	242050	Igsf10
5	58809	Rnase4
5	67305	Gpx7
5	14089	Fap
5	72560	Naalad2
5	14268	Fn1
5	70757	Ptplb
5	NaN	MMT00077446
5	67247	Mosc2
5	83961	Nrg4
5	69101	Ydjc
5	11861	Arl4a
5	231510	Agpat9
5	213956	AW544981
5	76408	Abcc3
5	108682	Gpt2
5	57914	Crlf2
5	20743	Spnb3
5	228859	D930001I22Rik
5	70556	Slc25a33
5	214855	Arid5a
5	70445	Cd248
5	26568	Slc27a3
5	52377	Rcn3
5	58859	Efemp2
5	14245	Lpin1
5	13036	Ctsh
5	381680	BC055004
5	30794	Pdlim4
5	14456	Gas6

5	104681	Slc16a6
5	12977	Csf1
5	23801	Aloxe3
5	237336	Tbpl1
5	22340	Vegfb
5	240641	Kif20b
5	69716	Trip13
5	30939	Pttg1
5	66949	Trim59
5	57441	Gmnn
5	16571	Kif4
5	218581	Depdc1b
5	26886	Cenph
5	101835	AW146154
5	72107	Dscc1
5	NaN	MMT00004673
5	73804	Kif2c
5	77022	2700099C18Rik
5	70466	Ckap2l
5	56150	Mad2l1
5	68026	2810417H13Rik
5	68774	Ms4a6d
5	20308	Ccl9
5	18726	Pira3
5	675749	MMT00015654
5	19288	Ptx3
5	13654	Egr2
5	83433	Trem2
5	237052	Tceal1
5	67951	Tubb6
5	22151	Tubb2a
5	67103	Ltb4dh
5	66357	2310008M10Rik
5	12409	Cbr2
5	108961	E2f8
5	72155	Cenpn
5	215387	Ncaph
5	66197	Cks2
5	107029	Me2
5	106795	Tcf19
5	12842	Col1a1
5	319170	Hist1h2an
5	20135	Rrm2
5	20878	Aurka
5	109212	6720460F02Rik
5	52276	Cdca8
5	21877	Tk1
5	269582	Clspn
5	12835	Col6a3
5	29870	Gtse1
5	208084	Pif1

5	229841	Cenpe
5	22137	Ttk
5	54392	Ncapg
5	20419	Shcbp1
5	12316	Aspm
5	16551	Kif11
5	434175	EG434175
5	12534	Cdc2a
5	72415	Sgol1
5	76464	Casc5
5	67052	Ndc80
5	66570	Cenpm
5	208836	Fanci
5	26909	Exo1
5	17279	Melk
5	67849	Cdca5
5	11799	Birc5
5	110033	Kif22
5	209737	Kif15
5	21335	Tacc3
5	107995	Cdc20
5	51944	D2Ertd750e
5	14793	Cdca3
5	12442	Ccnb2
5	12428	Ccna2
5	21973	Top2a
5	66977	Nuf2
5	108907	Nusap1
5	234258	Neil3
5	17345	Mki67
5	66468	2810433K01Rik
5	70218	3000004C01Rik
5	68014	Zwilch
5	19361	Rad51
5	17219	Mcm6
5	67177	Cdt1
5	68039	Nmb
5	13003	Vcan
5	80889	Mesdc1
5	21853	Timeless
5	15160	Serpind1
5	67652	Spaca1
5	545913	Zscan4d
5	100045495	MMT00061492
5	12064	Bdnf
5	72832	Crtac1
5	242594	1700024P16Rik
5	66934	Dsn1
5	66873	1200009O22Rik
5	320595	Phf8
5	55963	Slc1a4

5	69627	2310031A18Rik
5	14733	Gpc1
5	67666	Hapln3
5	16324	Inhbb
5	13048	Cux2
5	226153	Peo1
5	226105	Cyp2c70
5	68058	Chd1l
5	79059	Nme3
5	68777	Tmem53
5	68350	Mul1
5	75620	2810422J05Rik
5	213603	Slc44a3
5	170826	Ppargc1b
5	232210	8430410A17Rik
5	12652	Chga
5	67384	Bag4
5	20768	Sephs2
5	66431	1810049H13Rik
5	63828	Fn3k
5	16803	Lbp
5	67856	Echdc3
5	69206	2010016I18Rik
5	381411	Gm1967
5	234564	AU018778
5	171282	Acot4
5	104158	Ces3
5	68918	1190005I06Rik
5	18984	Por
5	69123	1810022C23Rik
5	68126	Fahd2a
5	12310	Calca
5	64945	Cldn12
5	74769	Pik3cb
5	70503	Ddo
5	18979	Pon1
5	14261	Fmo1
5	18639	Pfkfb1
5	16169	Il15ra
5	14546	Gdap10
5	103844	AI842396
5	329470	Accs
5	66391	2310061J03Rik
5	27053	Asns
5	23986	Peci
5	227696	Phyhd1
5	68465	Adipor2
5	76454	Fbxo31
5	170441	Slc2a10
5	13106	Cyp2e1
5	20216	Acsm3

5	59010	Sqrdl
5	14057	Sfxn1
5	66945	Sdha
5	229905	Ccbl2
5	71986	Ddx28
5	227095	Hibch
5	71699	Slc41a3
5	15200	Hbegf
5	60527	Fads3
5	270086	Ogfod1
5	320405	Cadps2
5	19349	Rab7
5	81703	Jdp2
5	66185	1110037F02Rik
5	20716	Serpina3n
5	13163	Daxx
5	217843	9030205A07Rik
5	71904	Paqr7
5	245877	Mtap7d1
5	67784	Plxnd1
5	11975	Atp6v0a1
5	15460	Hr
5	68728	Trp53inp2
5	104445	Cdc42ep1
5	107022	Gramd3
5	23893	Grem2
5	50776	Polg2
5	14778	Gpx3
5	319468	Ppm1h
5	13180	Pcbd1
5	20822	Trove2
5	18611	Pea15a
5	114664	Hsd17b11
5	26464	Vnn3
5	66966	Trit1
5	434234	2610020H08Rik
5	13850	Ephx2
5	545725	Mterf
5	100163	Pafah2
5	69742	Tm2d2
5	12827	Col4a2
5	13014	Cstb
5	68038	Chid1
5	78372	3300001G02Rik
5	66787	4933433P14Rik
5	72433	Rab38
5	52430	Echdc2
5	17035	Lxn
5	76630	Stambpl1
5	73246	Rassf6
5	109901	Ela1

5	20348	Sema3c
5	13039	Ctsl
5	17294	Mest
5	228550	Itpka
5	109042	Prkcdbp
5	59126	Nek6
5	20306	Ccl7
5	20296	Ccl2
5	214779	9630041N07Rik
5	338360	B430212C06Rik
5	223917	Krt79
5	12406	Serpinh1
5	98660	Atp1a2
5	211480	Kcnj14
5	20516	Slc20a2
5	76238	Grhpr
5	58176	Rhbg
5	53857	Tuba8
5	103172	MMT00073283
5	230119	Zbtb5
5	223453	Dap
5	233445	Vmn2r71
5	630825	EG630825
5	98170	Tmem132a
5	NaN	MMT00063223
5	73379	Dcbld2
5	80287	Apobec3
5	12834	Col6a2
5	238328	Vash1
5	231125	Zfyve28
5	20167	Rtn2
5	57440	Ehd3
5	380711	Garnl4
5	213696	Duoxa1
5	11576	Afp
5	19736	Rgs4
5	20518	Slc22a2
5	223337	Ugt3a2
5	320405	Cadps2
5	74257	Tspan17
5	16846	Lep
5	12505	Cd44
5	71854	Dpep3
5	116847	Prelp
5	226610	C030014K22Rik
5	77794	Adamtsl2
5	67369	Qpctl
5	232680	Cpa2
5	16485	Kcna1
5	70008	Ace2
5	52639	Wipi1

5	19011	Pp11r
5	22352	Vim
5	75712	Tmem14a
5	171241	V1rg6
5	57266	Cxcl14
5	326618	Tpm4
5	20255	Scg3
5	57262	Retnla
5	170779	Cd209d
5	20203	S100b
5	16780	Lamb3
5	171506	H1foo
5	66240	Kcne1l
5	76257	Slc38a3
5	93960	Nkd1
5	50918	Myadm
5	12727	Clcn4-2
5	13107	Cyp2f2
5	NaN	MMT00068187
5	74205	Acsl3
5	80708	Pacsin3
5	67844	Rab32
5	20321	Frrs1
5	71997	1500002O20Rik
5	70873	4921517L17Rik
5	69379	C8g
5	NaN	MMT00072312
5	381339	Tmem182
5	18074	Nid2
5	71683	Gypc
5	50497	Hspa14
5	66790	Grtp1
5	68272	Rbm28
5	231876	Lmtk2
5	64385	Cyp4f14
5	320292	Rasgef1b
5	235135	Tmem45b
5	13822	Epb4.1l2
5	75564	1700027N10Rik
5	24051	Sgcb
5	24052	Sgcd
5	192656	Ripk2
5	218035	Vps41
5	94089	Trim7
5	68347	0610011F06Rik
5	214952	Rhot2
5	72168	Aifm3
5	69479	1700029J07Rik
5	12793	Cnih
5	77032	2610029I01Rik
5	12509	Cd59a

5	380753	Atxn7l1
5	70772	MMT00075624
5	217473	Ankmy2
5	17257	Mecp2
5	270035	Letm2
5	223649	Nrbp2
5	NaN	MMT00050414
5	102462	Imp3
5	12585	Cdr2
5	12306	Anxa2
5	20702	Serpina1c
5	20703	Serpina1d
5	71103	Glt6d1
5	13244	Degs1
5	319189	Hist2h2bb
5	12819	Col15a1
5	110379	Sec13
5	66663	Uba5
5	104943	9030611O19Rik
5	620934	EG620934
5	244209	Cyp2r1
5	208715	Hmgcs1
5	14872	Gstt2
5	78124	4930458L03Rik
5	239796	1600021P15Rik
5	407243	Tmem189
5	84095	Pi4k2a
5	234875	Ttc13
5	209683	Ttc28
5	71838	Phf7
5	16795	Large
5	110075	Bmp3
5	14366	Fzd4
5	23794	Adamts5
5	18162	Npr3
5	104718	Ttc7b
5	75805	Nln
5	NaN	MMT00035807
5	226856	Lpgat1
5	77739	Adamtsl1
5	212377	F730047E07Rik
5	69882	2010321M09Rik
5	21339	Taf1a
5	22719	Zfp61
5	66867	Hmg20a
5	72117	Nat13
5	69876	Thap3
5	66085	Eif3f
5	52468	Ctdsp2
5	12313	Calm1
5	21652	Phf1

5	69870	Polr3gl
5	67544	4932442K08Rik
5	52685	Cd300lg
5	16857	Lgals6
5	269587	Epb4.1
5	16952	Anxa1
5	77827	Krba1
5	14559	Gdf1
5	67705	1810058I24Rik
5	12479	Cd1d1
5	671232	MMT00070175
5	13008	Csrp2
5	20502	Slc16a2
5	93689	Lmod1
5	57429	Sult5a1
5	67068	Dynlrb1
5	20195	S100a11
5	20195	S100a11
5	71770	Ap2b1
5	319953	Ttll1
5	68742	1110032O16Rik
5	72056	1810055G02Rik
5	104871	Spata7
6	16701	Krtap6-2
6	75731	5133401N09Rik
6	66467	Gtf2h5
6	227154	Als2cr2
6	100494	Zfand2a
6	242585	Slc35d1
6	12677	Vsx2
6	22171	Tyms
6	67769	Gpatch2
6	75758	9130401M01Rik
6	66928	3110001D03Rik
6	94066	Mrpl36
6	20220	Sap18
6	53380	Psmd10
6	17846	Commd1
6	626952	EG626952
6	66192	Lage3
6	13589	Mapre1
6	104721	Ddx1
6	66844	Ormdl2
6	107094	Rrp12
6	64424	Polr1e
6	68252	A030007L17Rik
6	23894	Gtf2h2
6	66720	Klhl10
6	170752	Bco2
6	12314	Calm2
6	66988	Lap3

6	19428	Rasl2-9
6	50887	Nsbp1
6	216131	Tmem1
6	28040	D6Wsu163e
6	67239	Bxdc1
6	18393	Orc2l
6	NaN	MMT00041633
6	625796	EG625796
6	66461	Ptpmt1
6	NaN	MMT00030026
6	622657	EG622657
6	54124	Cks1b
6	65102	Nif3l1
6	110960	Tars
6	74011	Slc25a27
6	20813	Srp14
6	103765	Tmem17
6	230810	Slc30a2
6	30952	Cngb3
6	13047	Cux1
6	71564	9030607L17Rik
6	12916	Crem
6	12309	S100g
6	52882	Rgs7bp
6	NaN	MMT00008376
6	70377	Derl3
6	113868	Acaa1a
6	NaN	MMT00001400
6	NaN	MMT00014895
6	26940	Ecsit
6	52552	Parp8
6	72480	Tspyl4
6	14579	Gem
6	71970	2410018M08Rik
6	16828	Ldha
6	NaN	MMT00001941
6	11674	Aldoa
6	407785	Ndufs6
6	69804	Tmem147
6	73738	Uchl5ip
6	73738	Uchl5ip
6	NaN	MMT00068885
6	81799	C1qtnf3
6	56741	Nope
6	71336	Rbks
6	13001	Csnk2b
6	67044	Higd2a
6	73272	1700108F19Rik
6	56421	Pfkp
6	27397	Mrpl17
6	19218	Ptger3

6	111175	Pecr
6	67014	Mina
6	81910	Rrbp1
6	NaN	MMT00011288
6	108124	Napa
6	12181	Bop1
6	114128	Laptm4b
6	56282	Mrpl12
6	NaN	MMT00018228
6	624219	EG624219
6	70335	Reep6
6	107376	E330013P04Rik
6	74192	Arpc5l
6	66181	Nola3
6	18105	Nqo2
6	77862	Thyn1
6	26425	Nubp1
6	67851	1700021F05Rik
6	NaN	MMT00035424
6	NaN	MMT00013924
6	30059	Timm10
6	66273	1810020D17Rik
6	66448	Mrpl20
6	380712	2010305C02Rik
6	100042265	MMT00012636
6	56295	Higd1a
6	67956	Setd8
6	75772	Pnpla5
6	72899	Macrod2
6	110196	Fdps
6	NaN	MMT00063985
6	56737	Alg2
6	66422	2410015N17Rik
6	NaN	MMT00021352
6	66538	Rps19bp1
6	67212	Mrpl55
6	226251	Ablim1
6	12462	Cct3
6	NaN	MMT00044543
6	19384	Ran
6	NaN	MMT00073590
6	20656	Sod2
6	64657	Mrps10
6	68082	Dusp19
6	319695	Ankar
6	70984	4931406C07Rik
6	15526	Hspa9
6	26901	Deb1
6	NaN	MMT00070823
6	NaN	MMT00054932
6	670717	MMT00038112

6	NaN	MMT00024508
6	NaN	MMT00079290
6	17434	Mocs2
6	109245	Lrrc39
6	69270	Gins1
6	NaN	MMT00002928
6	106564	Ppcs
6	13684	Eif4e
6	67557	Larp6
6	232493	Gys2
6	231070	Insig1
6	72900	Ndufv2
6	212442	Lactb2
6	NaN	MMT00070887
6	30058	Timm8a1
6	30058	Timm8a1
6	22088	Tsg101
6	66271	Tmem126a
6	108812	Als2cr12
6	67899	2010110K16Rik
6	70397	Tmem70
6	12759	Clu
6	20962	Sycp3
6	29817	Igfbp7
6	231207	Cpeb2
6	20660	Sorl1
6	271639	Adcy10
6	328258	AU042651
6	66042	Sostdc1
6	22333	Vdac1
6	72330	Kbtbd5
6	27226	Pla2g7
6	71998	Slc25a35
6	338359	Supv3l1
6	110095	Pygl
6	76614	Immt
6	215951	Lace1
6	107476	Acaca
6	271144	9330128J19Rik
6	NaN	MMT00010554
6	NaN	MMT00071316
6	NaN	MMT00041407
6	67895	Ppa1
6	56451	Suclg1
6	56078	Car5b
6	14194	Fh1
6	72039	Mccc1
6	14859	Gsta3
6	69487	2310003L22Rik
6	66847	Hint3
6	21682	Tec

6	270084	Lpcat2
6	17060	Blnk
6	58805	Mlxipl
6	67900	1700020C11Rik
6	20787	Srebf1
6	66659	Acp6
6	257633	Acsf3
6	13202	Ddt
6	67308	Mrpl46
6	67914	Coq9
6	11429	Aco2
6	NaN	MMT00035904
6	170718	Idh3b
6	20528	Slc2a4
6	14860	Gsta4
6	58200	Ppp1r1a
6	320415	Gchfr
6	20249	Scd1
6	68393	Mogat1
6	20850	Stat5a
6	214742	Rcor3
6	228602	4930402H24Rik
6	433702	AU014645
6	67078	1700012G19Rik
6	NaN	MMT00063982
6	110391	Qdpr
6	94062	Mrpl3
6	NaN	MMT00037611
6	67003	Uqcrc2
6	72170	Chchd4
6	18631	Pex11a
6	17713	Grpel1
6	102093	Phkb
6	70316	Ndufab1
6	14226	Fkbp1b
6	18120	Mrpl49
6	66462	2810428I15Rik
6	380752	Tssc1
6	NaN	MMT00013152
6	19247	Ptpn11
6	66427	Cyb5b
6	66427	Cyb5b
6	230075	Ndufb6
6	319554	Idi1
6	66390	Slmo2
6	76890	Memo1
6	18858	Pmp22
6	54722	Dfna5h
6	215928	BC021785
6	76633	1700112E06Rik
6	231123	BC023882

6	73046	Glrx5
6	14874	Gstz1
6	67460	Decr1
6	12049	Bcl2l10
6	NaN	MMT00015473
6	NaN	MMT00058786
6	60409	Trappc4
6	74316	Isca2
6	NaN	MMT00057426
6	621603	Aldh3b2
6	12840	Col9a2
6	272158	Poln
6	107747	Aldh1l1
6	77754	Prune2
6	12497	Entpd6
6	18648	Pgam1
6	13190	Dct
6	71934	Car13
6	56199	Abcb10
6	110821	Pcca
6	66333	Aqp11
6	170439	Elovl6
6	NaN	MMT00074126
6	17436	Me1
6	16407	Itgae
6	235339	Dlat
6	21835	Thrsp
6	68263	Pdhb
6	67528	Nudt7
6	52706	D10Ertd761e
6	NaN	MMT00060232
6	70456	Brp44
6	60525	Acss2
6	55951	Brp44l
6	NaN	MMT00011146
6	NaN	MMT00080137
6	105675	Ppif
6	71743	Coasy
6	14571	Gpd2
6	109232	Sccpdh
6	71116	Stx18
6	67292	Pigc
6	71832	Csl
6	12974	Cs
6	69113	Alkbh3
6	12580	Cdkn2c
6	78920	Dlst
6	26379	Esrra
6	380654	4930485B16Rik
6	104112	Acly
6	110460	Acat2

6	67512	Agpat2
6	68385	Tlcd1
6	12850	Coq7
6	66375	Dhrs7
6	64292	Ptges
6	68603	Pmvk
6	67892	1810063B05Rik
6	381338	Lonrf2
6	NaN	MMT00042032
6	14555	Gpd1
6	77037	Mrap
6	20856	Stc2
6	68499	Mrpl53
6	66230	Mrps28
6	11677	Akr1b3
6	28080	Atp5o
6	NaN	MMT00027093
6	18673	Phb
6	69861	2010003K11Rik
6	13358	Slc25a1
6	277973	Slc9a5
6	14104	Fasn
6	110208	Pgd
6	27376	Slc25a10
6	16178	Il1r2
6	433719	OTTMUSG00000001246
6	NaN	MMT00067065
6	21881	Tkt
6	13143	Dapk2
6	66234	Sc4mol
6	104086	Cyp27a1
6	20775	Sqle
6	13121	Cyp51
6	13371	Dio2
6	57742	Abhd1
6	68671	Pcyt2
6	56279	B230317C12Rik
6	NaN	MMT00011909
6	104816	A530050D06Rik
6	110157	Raf1
6	108156	Mthfd1
6	211666	Mgst2
6	29812	Ndrg3
6	NaN	MMT00036089
6	68927	Ptcd2
6	12261	C1qbp
6	NaN	MMT00001967
6	269105	EG269105
6	66459	Pigy
6	11949	Atp5c1
6	11949	Atp5c1

6	18563	Pcx
6	67680	Sdhb
6	17995	Ndufv1
6	270076	Gcdh
6	78894	Aacs
6	57264	Retn
6	12894	Cpt1a
6	70804	Pgrmc2
6	71973	Rbpms2
6	319942	A530016L24Rik
6	27280	Phlda3
6	11781	Ap4m1
6	230082	Nol6
6	116939	Pnpla3
6	11758	Prdx6
6	11813	Apoc2
6	67131	Acbd4
6	71648	Optn
6	16516	Kcnj15
6	11921	Atoh1
6	NaN	MMT00027196
6	NaN	MMT00000744
6	627427	EG627427
6	66214	1190002H23Rik
6	99543	Olfml3
6	72701	Zfp618
6	70370	Fbln7
6	214359	Tmem51
6	83675	Bicc1
6	50524	Sall2
6	194126	Mtmr11
6	381217	Gm967
6	319565	Syne2
6	78892	Crispld2
6	675135	MMT00050019
6	17988	Ndrg1
6	59009	Sh3rf1
6	50785	Hs6st1
6	494448	Cbx6
6	74443	4933406E20Rik
6	629378	Dact3
6	19325	Rab10
6	216856	Nlgn2
6	235130	Adamts15
6	17150	Mfap2
6	69217	Plekha4
6	16399	Itga2b
6	17390	Mmp2
6	246228	Vwa1
6	23887	Ggt5
6	22371	Vwf

6	227612	A830007P12Rik
6	234796	BC025816
6	319433	E130113E03Rik
6	22628	Ywhag
6	13822	Epb4.1l2
6	232313	Glt8d4
6	12499	Entpd5
6	11692	Gfer
6	13132	Dab2
6	105837	Mtbp
6	67729	Mansc1
6	223272	Itgbl1
6	14767	Nmur1
6	15257	Hipk1
6	66734	Map1lc3a
6	225884	BC021614
6	NaN	MMT00042142
6	21356	Tapbp
6	NaN	MMT00071569
6	58801	Pmaip1
6	67702	Rnf149
6	13685	Eif4ebp1
6	108148	Galnt2
6	14936	Gys1
6	NaN	MMT00036391
6	12908	Crat
6	97165	Hmgb2
6	17217	Mcm4
6	64899	Lpin3
6	66994	1500031L02Rik
6	75552	Paqr9
6	22431	Wt1
6	58243	Nap1l5
6	269473	Lrig2
6	73671	Sult6b1
6	230103	Npr2
6	17281	Fyco1
6	67471	Gpatch1
6	74302	Mtmr3
6	20679	Sox6
6	74616	Scrn3
6	56314	Zfp113
6	15259	Hipk3
6	76809	Bri3bp
6	56809	Gmeb1
6	23794	Adamts5
6	72308	Brf1
6	69125	Cnot8
6	59002	Wdr8
6	16434	Itpa
6	79554	Gltpd1

6	242642	Hpdl
6	93692	Glrx
6	171504	MMT00073557
6	235047	Zfp809
6	75785	Klhl24
6	56404	Trip4
6	212555	Pqlc2
6	50783	Lsm4
6	15360	Hmgcs2
6	242785	Klhl21
6	212153	2610015P09Rik
6	79263	Trim39
6	320225	A230107C01Rik
6	56177	Olfm1
6	18113	Nnmt
6	78787	Usp54
6	13435	Dnmt3a
6	625029	Vmn2r83
6	226075	Glis3
6	67378	Bbs2
6	NaN	MMT00031263
6	54427	Dnmt3l
6	18391	Oprs1
6	22145	Tuba4a
6	66469	2810405K02Rik
6	107986	Ddb2
6	21743	Inmt
6	71664	Mettl7b
6	67267	2900010M23Rik
6	27361	Sepx1
6	21991	Tpi1
6	12282	Hyou1
6	18595	Pdgfra
6	192191	Med9
6	67046	Tbc1d7
6	233549	Mogat2
6	67800	Dgat2
6	76282	Gpt
6	70153	2210016F16Rik
6	71146	4933417O08Rik
6	13360	Dhcr7
6	382075	Odf3l1
6	103733	Tubg1
6	98238	Lrrc59
6	67283	Slc25a19
6	192166	Sardh
6	12931	Crlf1
6	226122	Ubtd1
6	11769	Ap1s1
6	66264	Ccdc28b
6	27981	D4Wsu53e

6	73635	1700113I22Rik
6	69071	Tmem97
6	218271	B4galt7
6	192292	Nrbp1
6	104444	Rexo2
6	619605	Zcchc17
6	14431	Gamt
6	22183	Zrsr1
6	627110	Tubb2a-ps2
6	320204	4833442J19Rik
6	108735	Sft2d2
6	433216	EG433216
6	66508	2400001E08Rik
6	69928	Apitd1
6	18478	Pah
6	69291	1700001L05Rik
6	74359	4931414P19Rik
6	68097	Dynll2
6	52906	Ahi1
6	14187	Akr1b8
6	103742	1810046J19Rik
6	66411	Tbcb
6	17766	Nudt1
6	19387	Rangap1
6	53945	Slc40a1
6	105445	Dock9
6	13592	Ebf2
6	23996	Psmc4
6	73737	1110008P14Rik
6	67994	Mrps11
6	22154	Tubb5
6	NaN	MMT00051732
6	NaN	MMT00038317
6	57785	Rangrf
6	NaN	MMT00031928
6	21826	Thbs2
6	52466	Slc46a1
6	68043	N6amt2
6	66320	Tmem208
6	52530	Nola2
6	668830	EG668830
6	208624	Alg3
6	68073	A930016P21Rik
6	103850	Nt5m
6	76501	Commd9
6	NaN	MMT00033856
6	171429	Slc26a6
6	78754	Galntl2
6	56418	Ykt6
6	18102	Nme1
6	216505	Pik3ip1

6	75471	1700009N14Rik
6	74268	Aven
6	67974	Ccny
6	18025	Nfe2l3
6	68118	9430023L20Rik
6	20384	Sfrs5
6	20384	Sfrs5
6	237222	Ofd1
6	67785	Zmym4
6	30055	Timm13
6	23897	Hax1
6	23897	Hax1
6	231872	MMT00063115
6	19182	Psmc3
6	236794	Slc9a6
6	67843	Slc35a4
6	NaN	MMT00066182
6	53333	Tomm40
6	22225	Usp5
6	69507	1700030G06Rik
6	66096	0910001L09Rik
6	14083	Ptk2
6	13078	Cyp1b1
6	22329	Vcam1
6	56363	Tmeff2
6	18779	Pla2r1
6	11607	Agtr1a
6	13482	Dpp4
6	14660	Gls
6	17536	Meis2
6	74199	Vit
6	73172	3110037I16Rik
6	76967	2700049A03Rik
6	71779	8-Mar
6	229487	Pet112l
6	74246	Gale
6	234729	Vac14
6	192156	Mvd
6	16987	Lss
6	108155	Ogt
6	20356	Sema5a
6	257635	Sdsl
6	12447	Ccne1
6	76380	Ccdc46
6	171180	Syt12
6	117606	Boc
6	268977	Ltbp1
6	14451	Gas1
6	71145	Scara5
6	385658	Gm1752
6	108954	Ppp1r15b

6	627191	Tmem90a
6	72655	2810026P18Rik
6	18976	Pomc
6	20474	Six4
6	328099	AU021838
6	320923	Mtap7d3
6	75089	Uhrf1bp1l
6	13682	Eif4a2
6	NaN	MMT00032526
6	52348	Vps37a
6	619331	Zfp551
6	142688	Asb13
6	17763	Mtcp1
6	106581	Itfg3
6	24135	Zfp68
6	78689	Mak10
6	320162	Ccdc45
6	74570	Zkscan1
6	73668	Ttc21b
6	17344	Pias2
6	117198	Ivns1abp
6	236790	Ddx26b
6	216190	Appl2
6	56494	Gosr2
6	217378	Rbj
6	75692	Nr2c2ap
6	106389	Eaf2
6	26357	Abcg2
6	69770	1600002K03Rik
6	218820	Zfp503
6	70891	Spdya
6	192231	Hexim1
6	320816	Ankrd16
6	215512	5730593F17Rik
6	217700	Acot6
6	74200	2810403A07Rik
6	238333	Gm263
6	66531	2310061C15Rik
6	16852	Lgals1
6	53867	Col5a3
6	22232	Slc35a2
6	71724	Aox3
6	403088	MMT00010458
6	12876	Cpe
6	12876	Cpe
6	16431	Itm2a
6	234214	Sorbs2
6	50877	Neu3
6	223669	Zfp7
6	74051	Steap2
6	68859	1190007F08Rik

6	74646	Spsb1
6	16424	Itih1
6	629966	EG629966
6	237221	Gemin8
6	69454	Clic3
6	217721	Flvcr2
6	21961	Tns1
6	14251	Flot1
6	71268	Lrrfip2
6	56248	Ak3
6	NaN	MMT00024953
6	NaN	MMT00045847
6	19355	Rad1
6	NaN	MMT00067195
6	208144	Dhx37
6	100900	Hscb
6	100034361	Mfap1b
6	67155	Smarca2
6	12263	C2
6	24099	Tnfsf13b
6	20257	Stmn2
6	234683	Elmo3
6	64817	Svep1
6	214931	Fbxl16
6	20315	Cxcl12
6	13614	Edn1
6	69069	1810011H11Rik
6	237504	Rassf9
6	16568	Kif3a
6	232236	C130022K22Rik
6	52013	D19Ertd386e
6	67443	Map1lc3b
6	319229	Sctr
6	64075	Smoc1
6	26938	St6galnac5
6	20250	Scd2
6	22361	Vnn1
6	241274	Pnpla7
6	68487	Tmem140
6	66528	2210020M01Rik
6	69034	4930579G22Rik
6	224440	Setd4
6	227937	Pkp4
6	74164	Nfx1
6	231670	Fbxo21
6	81018	Zfp313
6	69726	Smyd3
6	212439	AA986860
6	228765	Sdcbp2
7	77864	Ypel2
7	78586	Srbd1

7	67998	1300010M03Rik
7	11637	Ak2
7	12465	Cct5
7	105835	Sgsm3
7	73692	2410089E03Rik
7	NaN	MMT00071219
7	18027	Nfia
7	17342	Mitf
7	232087	Mat2a
7	11600	Angpt1
7	18519	Pcaf
7	230753	Thrap3
7	74340	Ahcyl2
7	66816	Thap2
7	213550	Dis3l
7	233328	Lrrk1
7	235534	Acpl2
7	17237	Mgrn1
7	170707	Usp48
7	72289	Malat1
7	104859	4930573I19Rik
7	77300	Raph1
7	19185	Psmd4
7	NaN	MMT00062659
7	NaN	MMT00035426
7	21745	Tep1
7	12772	Ccr2
7	224903	Safb
7	230793	Ahdc1
7	81896	Ift122
7	208777	Sned1
7	72275	2200002D01Rik
7	72318	Pscd4
7	79455	Pdcl2
7	227120	Plcl1
7	210808	9030625A04Rik
7	68628	Fbxw9
7	67956	Setd8
7	67902	Sumf2
7	18585	Pde9a
7	19188	Psme2
7	19188	Psme2
7	415115	Neurl2
7	233865	D430042O09Rik
7	72278	Ccpg1
7	72023	Cyb561d1
7	16154	Il10ra
7	54153	Rasa4
7	17423	Ndst2
7	65970	Lima1
7	242248	Bank1

7	NaN	MMT00001961
7	226541	Klhl20
7	234135	Whsc1l1
7	320538	D130059P03Rik
7	72345	2810002O09Rik
7	12821	Col17a1
7	101835	AW146154
7	209239	Gan
7	16792	Laptm5
7	73656	Ms4a6c
7	NaN	MMT00065211
7	12581	Cdkn2d
7	74237	Tubgcp2
7	240084	Cchcr1
7	71718	Telo2
7	56876	Nelf
7	217038	Mrm1
7	234582	Ccdc102a
7	69106	Stoml1
7	69470	Tmem127
7	13852	Stx2
7	59031	Chst12
7	69161	Manbal
7	79196	Osbpl5
7	330731	EG330731
7	76681	Trim12
7	74645	4930431B09Rik
7	12576	Cdkn1b
7	399558	Flrt2
7	66236	1500011B03Rik
7	320923	Mtap7d3
7	76260	Ttc8
7	109979	Art3
7	234852	Chmp1a
7	67579	Cpeb4
7	18000	2-Sep
7	72776	Sass6
7	70415	2610018G03Rik
7	333789	N4bp2
7	12293	Cacna2d1
7	70604	Dnajb14
7	319817	Rc3h2
7	104836	Cbll1
7	212943	BC023892
7	104570	Smek2
7	13205	Ddx3x
7	218100	Zfp322a
7	12283	Cab39
7	71521	Pds5a
7	11767	Ap1m1
7	546387	EG546387

7	21399	Tcea1
7	105377	Ankrd32
7	NaN	MMT00000525
7	68152	5830415L20Rik
7	67263	Zswim6
7	224105	Pak2
7	109624	Cald1
7	NaN	MMT00002304
7	52846	D1Bwg0212e
7	26874	Abcd2
7	NaN	MMT00039126
7	218734	3830406C13Rik
7	269198	Nbeal1
7	NaN	MMT00077178
7	382117	D9Ertd402e
7	19883	Rora
7	18573	Pde1a
7	72739	Zkscan3
7	171212	Galnt10
7	20512	Slc1a3
7	231225	Tapt1
7	NaN	MMT00077107
7	381314	Iars2
7	NaN	MMT00000967
7	20965	Syn2
7	208449	Sgms1
7	72823	Pard3b
7	330914	MMT00072326
7	319565	Syne2
7	17714	Grpel2
7	12995	Csnk2a1
7	12995	Csnk2a1
7	18128	Notch1
7	NaN	MMT00047239
7	30934	Tor1b
7	66839	0610009O20Rik
7	78825	5830417C01Rik
7	229900	Gbp6
7	80750	N4bp1
7	100045149	MMT00025143
7	18099	Nlk
7	23888	Gpc6
7	50758	Fbxl17
7	231452	Sdad1
7	20239	Atxn2
7	18018	Nfatc1
7	216613	Ccdc85a
7	108797	Mex3b
7	231912	Katnal1
7	26408	Map3k5
7	68339	Ccdc88c

7	60596	Gucy1a3
7	54403	Slc4a4
7	12577	Cdkn1c
7	55990	Fmo2
7	226041	Pgm5
7	53623	Gria3
7	226747	Ahctf1
7	NaN	MMT00020644
7	12151	Bmi1
7	74143	Opa1
7	67532	Mfap1a
7	NaN	MMT00036239
7	109115	Supt3h
7	NaN	MMT00014022
7	12530	Cdc25a
7	23955	Nek4
7	NaN	MMT00080717
7	NaN	MMT00033071
7	214133	Tet2
7	NaN	MMT00042003
7	30046	Zfp292
7	12912	Creb1
7	17999	Nedd4
7	269198	Nbeal1
7	56376	Pdlim5
7	71544	9030420J04Rik
7	71544	9030420J04Rik
7	320712	Abi3bp
7	328329	Mast4
7	18145	Npc1
7	545428	2610301F02Rik
7	21871	Atp6v0a2
7	66454	Nmnat1
7	NaN	MMT00042479
7	NaN	MMT00030019
7	26444	Psma7
7	223870	Senp1
7	229227	4932438A13Rik
7	12013	Bach1
7	229663	Csde1
7	12874	Cpd
7	17246	Mdm2
7	77590	4631426J05Rik
7	13688	Eif4ebp2
7	14630	Gclm
7	218699	Pxk
7	76014	Zc3h18
7	NaN	MMT00075201
7	NaN	MMT00044175
7	NaN	MMT00047183
7	NaN	MMT00007970

7	NaN	MMT00012375
7	52822	Rufy3
7	13669	Eif3s10
7	70769	Nolc1
7	NaN	MMT00019519
7	NaN	MMT00048203
7	319887	E030030I06Rik
7	NaN	MMT00044766
7	628431	Hmgb1-rs17
7	15289	Hmgb1
7	NaN	MMT00046069
7	66257	Nicn1
7	14874	Gstz1
7	217830	9030617O03Rik
7	NaN	MMT00041615
7	327978	Slfn5
7	19766	Ripk1
7	244895	C230081A13Rik
7	13998	Fgd6
7	56637	Gsk3b
7	20481	Ski
7	20852	Stat6
7	NaN	MMT00032015
7	NaN	MMT00013656
7	51786	Cpsf2
7	50911	Exosc9
7	56095	Ftsj3
7	NaN	MMT00007878
7	99526	Usp53
7	320165	Tacc1
7	76719	1700081L11Rik
7	208647	Creb3l2
7	229279	Hnrnpa3
7	NaN	MMT00017539
7	13418	Dnajc1
7	69237	Gtpbp4
7	218544	Sgtb
7	72972	Gcap14
7	NaN	MMT00036444
7	432502	EG432502
7	NaN	MMT00060205
7	NaN	MMT00034010
7	243272	Sbno1
7	192195	Ash1l
7	100038725	ENSMUSG00000038594
7	21885	Tle1
7	385454	EG385454
7	NaN	MMT00050540
7	59057	Zfp191
7	233575	MMT00014327
7	NaN	MMT00047335

7	NaN	MMT00046996
7	114713	Rasa2
7	17300	Foxc1
7	NaN	MMT00000944
7	NaN	MMT00011143
7	NaN	MMT00040956
7	11909	Atf2
7	208846	Daam1
7	101240	Wdr91
7	93873	Pcdhb2
7	78937	5830411G16Rik
7	108655	Foxp1
7	227525	Dclre1c
7	434484	Sp140
7	98682	2210010L05Rik
7	240261	Ccdc112
7	218194	Phactr1
7	228355	Madd
7	234797	6430548M08Rik
7	NaN	MMT00047848
7	338355	Fkbp15
7	233977	Ppfia1
7	53416	Stk39
7	22608	Ybx1
7	22608	Ybx1
7	107589	Mylk
7	NaN	MMT00057557
7	17260	Mef2c
7	56200	Ddx21
7	67664	Rnf125
7	67797	6530403A03Rik
7	66935	1700023B02Rik
7	214899	Jarid1a
7	216613	Ccdc85a
7	271564	Vps13a
7	102182	AI931714
7	381650	Thap6
7	244672	Cwf19l2
7	NaN	MMT00061267
7	74528	8430406I07Rik
7	19155	Npepps
7	215160	Rhbdd2
7	NaN	MMT00048392
7	19988	Rpl6
7	NaN	MMT00058413
7	67916	Ppap2b
7	14313	Fst
7	240168	Rasgrp3
7	NaN	MMT00038301
7	74081	Cep350
7	68177	Ebpl

7	94282	Sfxn5
7	59001	Pole3
8	258315	Olfr767
8	77609	C330001K17Rik
8	NaN	MMT00013143
8	258096	Olfr112
8	353282	Sfmbt2
8	NaN	MMT00042049
8	272350	EG272350
8	52024	Ankrd22
8	73137	Prrc1
8	258730	Olfr483
8	270906	Prr11
8	75064	Cnbp2
8	110957	D1Pas1
8	233001	Nlrp9a
8	258935	Olfr790
8	NaN	MMT00077586
8	NaN	MMT00005149
8	100012	Oog3
8	74176	Tgm5
8	NaN	MMT00013109
8	20707	Serpinb9c
8	76999	1700127D06Rik
8	13640	Efna5
8	NaN	MMT00065179
8	258710	Olfr419
8	15551	Htr1b
8	230796	Wdtc1
8	NaN	MMT00028085
8	110789	Gpr98
8	NaN	MMT00061717
8	22301	Vmn2r89
8	211712	Pcdh9
8	14613	Gja5
8	654456	MMT00054636
8	NaN	MMT00047055
8	382118	Zfp167
8	12160	Bmp5
8	NaN	MMT00064569
8	16502	Kcnc1
8	78600	Pde6h
8	18158	Nppb
8	NaN	MMT00039382
8	627805	Vmn2r108
8	258022	Olfr1318
8	320664	Cass4
8	16688	Krt6b
8	546213	Gm1420
8	258358	Olfr557
8	71287	Cpvl

8	74998	Rab11fip2
8	258585	Olfr1086
8	72230	Zfp558
8	NaN	MMT00019756
8	319974	Auts2
8	15969	Ifna6
8	109263	Rlf
8	NaN	MMT00054100
8	NaN	MMT00046108
8	258247	Olfr645
8	NaN	MMT00036516
8	NaN	MMT00013160
8	18185	Nrl
8	279028	Adamts13
8	209550	EG209550
8	22074	Try4
8	NaN	MMT00038169
8	668225	EG668225
8	208884	Zdhhc9
8	66761	4933417A18Rik
8	73582	1700106N22Rik
8	633250	EG633250
8	20400	Sh2d1a
8	258880	Olfr218
8	278304	Zfp385c
8	74529	8430422H06Rik
8	NaN	MMT00009248
8	329693	Fcrl5
8	20495	Slc12a1
8	243725	Ppp1r9a
8	634331	EG634331
8	329679	Fnip2
8	234959	Med17
8	66780	4933436I01Rik
8	110789	Gpr98
8	50720	Sacs
8	217246	Ace3
8	240476	Zfp407
8	NaN	MMT00066283
8	72472	Slc16a10
8	271981	A630047E20Rik
8	66289	1810030J14Rik
8	NaN	MMT00078220
8	17540	Mrvi1
8	218476	Gm73
8	626904	MMT00004644
8	170651	Krtap16-1
8	258932	Olfr791
8	258783	Olfr920
8	258300	Olfr1449
8	14126	Ms4a2

8	17384	Mmp10
8	194597	Tmprss11a
8	12640	Cga
8	NaN	MMT00015154
8	NaN	MMT00070399
8	18193	Nsd1
8	666348	Apol7e
8	20983	Syt4
8	17773	Mtnr1a
8	258316	Olfr727
8	194597	Tmprss11a
8	116913	Tpbpb
8	93886	Pcdhb15
8	99663	Clca6
8	436059	EG436059
8	NaN	MMT00043095
8	192167	Nlgn1
8	17450	Morc1
8	NaN	MMT00008396
8	20730	Spink3
8	258891	Olfr1302
8	14829	Grpr
8	406186	Olfr142
8	239250	Slitrk6
8	210198	Gprc6a
8	NaN	MMT00012889
8	79202	Tnfrsf22
8	18508	Pax6
8	NaN	MMT00035581
8	78473	Skap1
8	78251	Zfp712
8	NaN	MMT00050850
8	NaN	MMT00000812
8	20278	Scnn1g
8	242574	C130073F10Rik
8	NaN	MMT00060699
8	73784	4930430F21Rik
8	93746	Gprc5d
8	76846	Rps9
8	384806	MMT00074605
8	74915	Atp6v1e2
8	110350	Dync2h1
8	72082	Cyp2c55
8	18511	Pax9
8	140806	Il25
8	242274	Lrrc7
8	380709	Spata22
8	242557	Atg4c
8	320183	Msrb3
8	78751	Zc3h6
8	67295	Rab3c

8	NaN	MMT00013081
8	233332	Adamts17
8	114871	Psg28
8	73980	MMT00024414
8	111507	Igh
8	76399	Il31
8	277898	9830102E05Rik
8	108950	1700049L16Rik
8	18419	Otog
8	320091	Tmem16d
8	77669	9130221D24Rik
8	68106	Nt5c3l
8	246729	Oas1h
8	NaN	MMT00006856
8	234353	Psd3
8	22293	Slc45a2
8	277773	BC049635
8	270599	Gm648
8	171211	Edaradd
8	233801	Acsm4
8	65100	Zic5
8	NaN	MMT00018393
8	217232	Cdc27
8	26465	Zfp146
8	67728	Dph2
8	16509	Kcne1
8	NaN	MMT00022580
8	110855	Pde6c
8	64335	Svs3a
8	665227	Vmn2r94
8	215029	Prl3d3
8	320865	Cdh18
8	16098	MMT00030712
8	243377	Svs1
8	56373	Cpb2
8	84543	Sval2
8	13828	Epb4.2
8	69125	Cnot8
8	382066	Prdm10
8	67336	1700054K19Rik
8	259118	Olfr575
8	319670	Eml5
8	71263	Mro
8	NaN	MMT00026800
8	NaN	MMT00034233
8	258650	Olfr444
8	19131	Prh1
8	NaN	MMT00028216
8	258726	Olfr599
8	16882	Lig3
8	67741	4930579F01Rik

8	627607	EG627607
8	207227	Stxbp5l
8	75320	Etnk1
8	640268	EG640268
8	75079	Zfp509
8	109254	9530008L14Rik
8	68799	Rgmb
8	75606	2010003K15Rik
8	19674	Rcvrn
8	258708	Olfr1342
8	NaN	MMT00031646
8	213234	Zbbx
8	67434	5730557B15Rik
8	NaN	MMT00035925
8	654450	MMT00010505
8	16576	Kif7
8	NaN	MMT00046724
8	171230	V1re7
8	NaN	MMT00041291
8	67120	Ttc14
8	213450	Gm732
8	268345	Kcnc2
8	18453	P4hb
8	NaN	MMT00078758
8	NaN	MMT00018457
8	17837	Mug2
8	216395	Tmem5
8	110350	Dync2h1
8	NaN	MMT00008521
8	329795	Tmem67
8	13109	Cyp2j5
8	NaN	MMT00005811
8	NaN	MMT00042948
8	NaN	MMT00030613
8	433597	MMT00013465
8	57354	Cramp1l
8	NaN	MMT00061154
8	258905	Olfr871
8	67909	Galntl5
8	233115	Dpy19l3
8	18700	Piga
8	57251	Olfr870
8	77918	Krtap16-5
8	57258	Xpo4
8	338354	Zfp780b
8	12000	Avpr2
8	171246	V1rh3
8	258362	Olfr1094
8	NaN	MMT00017494
8	70909	4921504E06Rik
8	71106	4933413J09Rik

8	14608	Gpr83
8	75829	Prame
8	NaN	MMT00036513
8	15496	Hsd3b5
8	20504	Slc17a1
8	66719	4921522P10Rik
8	78286	Nav2
8	NaN	MMT00054267
8	54122	Uevld
8	54601	Foxo4
8	113864	V1rc7
8	12393	Runx2
8	208518	Cep78
8	58869	Pex5l
8	110751	Adam33
8	NaN	MMT00030313
8	14237	Foxd4
8	12812	Coil
8	258548	Olfr805
8	54450	Il1f5
8	269254	Setx
8	66742	Cypt6
8	67854	Slco6b1
8	12565	Cdh9
8	20706	Serpinb9b
8	110304	Glra3
8	NaN	MMT00007848
8	NaN	MMT00019998
8	NaN	MMT00010339
8	NaN	MMT00005283
8	385674	Zfp174
8	258572	Olfr1032
8	258352	Olfr692
8	445007	Nup85
8	259035	Olfr714
8	626280	EG626280
8	94109	Csmd1
8	15458	Hpx
8	NaN	MMT00082318
8	225644	Cplx4
8	258297	Olfr827
8	258304	Olfr498
8	NaN	MMT00009539
8	258810	Olfr665
8	66673	Sorcs3
8	NaN	MMT00054056
8	331461	Il1rapl1
8	NaN	MMT00038532
8	22689	Zfp27
8	NaN	MMT00048642
8	329421	Myo3b

8	19111	Prl6a1
8	66786	Olfr701
8	74365	Lonrf3
8	18946	Pnliprp1
8	20511	Slc1a2
8	268491	Gm1564
8	73415	1700049E17Rik
8	73412	Txndc3
8	NaN	MMT00013067
8	15042	H2-T24
8	69694	Tatdn1
8	627132	Vmn2r93
8	320484	A430107D22Rik
8	78619	Zfp449
8	108096	Slco1a5
8	75050	Kif27
8	NaN	MMT00039380
8	78631	1700085A12Rik
8	NaN	MMT00060807
8	NaN	MMT00026261
8	271842	Rpusd2
8	78771	Mctp1
8	14729	Gp5
8	241391	Galnt5
8	111507	Igh
8	258299	Olfr1461
8	NaN	MMT00017308
8	74413	Tc2n
8	74369	Mei1
8	NaN	MMT00069233
8	76757	Trdn
8	404545	Tmem16g
8	213409	Lemd1
8	NaN	MMT00039356
8	71156	1700108M19Rik
8	NaN	MMT00029151
8	258216	Olfr1034
8	170770	Bbc3
8	NaN	MMT00020436
8	207596	Thsd4
8	NaN	MMT00029634
8	545741	EG545741
8	338320	Mia2
8	NaN	MMT00023360
8	260347	MMT00042388
8	67218	2810038D20Rik
8	67504	Rnf151
8	11449	Chrng
8	432555	EG432555
8	73809	Satl1
8	98752	Fcrla

8	67537	Glipr1l2
8	NaN	MMT00039685
8	238944	Nek10
8	NaN	MMT00035749
8	216049	Zfp365
8	19069	Nup88
8	71164	Zdhhc11
8	259054	Olfr589
8	237310	Il22ra2
8	52042	D17Ertd663e
8	241452	Dhrs9
8	114585	D17H6S53E
8	385737	Gm1758
8	77981	B230110C06Rik
8	239420	Csmd3
8	385120	Gm1502
9	NaN	MMT00019450
9	13085	Cyp2a12
9	14942	Gzme
9	27385	Magel2
9	668212	Efr3b
9	12425	Cckar
9	57752	Tacc2
9	72852	2900024O10Rik
9	258803	Olfr136
9	100043108	Cyp2c69
9	18343	Olfr44
9	14420	Galc
9	434121	EG434121
9	66712	Spesp1
9	450219	Gsdma3
9	230596	Prpf38a
9	242126	Slc22a15
9	12401	Serpina6
9	78478	1700129I15Rik
9	320679	Samd12
9	15497	Hsd3b6
9	258368	Olfr1450
9	15165	Hcn1
9	NaN	MMT00016640
9	211712	Pcdh9
9	12686	Elovl3
9	78354	2210407C18Rik
9	320534	Tmem104
9	50518	a
9	268780	Egflam
9	94176	Dock2
9	67715	2010106E10Rik
9	380822	MMT00072212
9	258290	Olfr1143
9	259015	Olfr1038

9	16061	Igh-VJ558
9	320007	Sidt1
9	114714	Rad51c
9	627081	Xlr5b
9	258934	Olfr802
9	170639	Olfr78
9	330660	Btbd16
9	547097	B020011L13Rik
9	20459	Ptk6
9	72789	Veph1
9	109151	Chd9
9	17230	Tpsab1
9	77771	Csrnp3
9	240479	B230399E16Rik
9	399603	D330050I23Rik
9	71623	Krtap5-2
9	75216	4930534B04Rik
9	232426	5530400C23Rik
9	NaN	MMT00052724
9	13099	Cyp2c40
9	241062	Pgap1
9	108655	Foxp1
9	15493	Hsd3b2
9	NaN	MMT00038655
9	109331	Rnf20
9	241547	D230010M03Rik
9	NaN	MMT00051225
9	330228	4933411G11Rik
9	12322	Camk2a
9	20445	St6galnac1
9	16184	Il2ra
9	NaN	MMT00037010
9	19226	Pth
9	259019	Olfr1045
9	258186	Olfr75-ps1
9	381832	Prpmp5
9	77531	Anks1b
9	70900	4921517D22Rik
9	66996	Ceacam11
9	241638	RP23-100C5.8
9	237625	Pla2g3
9	237038	Nox1
9	76816	Sdccag8
9	NaN	MMT00012984
9	75956	Srrm2
9	NaN	MMT00004378
9	217082	Hlf
9	320495	A130090K04Rik
9	632654	MMT00048312
9	224480	Nox3
9	258808	Olfr620

9	57252	Tas2r105
9	170716	Cyp4f13
9	20271	Scn5a
9	NaN	MMT00078580
9	103406	9130206N08Rik
9	NaN	MMT00000773
9	108069	Grm3
9	258883	Olfr876
9	259000	Olfr195
9	NaN	MMT00020896
9	112417	Ugt2b37
9	NaN	MMT00013165
9	NaN	MMT00080431
9	232910	Ap2s1
9	NaN	MMT00002308
9	NaN	MMT00067570
9	230558	C8a
9	NaN	MMT00025388
9	16061	Igh-VJ558
9	268980	Strn
9	215890	Rlbp1l2
9	21816	Tgm1
9	20749	Dbpht1
9	NaN	MMT00005305
9	72301	1810041L15Rik
9	NaN	MMT00050244
9	NaN	MMT00002596
9	76217	Jakmip2
9	225010	Lycat
9	98870	AI182371
9	75083	Usp50
9	18387	Oprk1
9	234736	Rfwd3
9	56747	Sez6l
9	20563	Slit2
9	16687	Krt6a
9	227627	Lcn13
9	13525	Adam26a
9	66763	4933425L06Rik
9	77220	C030003D03Rik
9	73747	1110034G24Rik
9	NaN	MMT00048531
9	233799	Acsm2
9	NaN	MMT00063288
9	NaN	MMT00047578
9	69168	Bola1
9	382090	4922501C03Rik
9	212712	Satb2
9	258587	Olfr1100
9	109663	Hoxc11
9	27028	Ermap

9	270163	Myo9a
9	241308	Ralgps1
9	20219	Apcs
9	228094	Cerkl
9	18440	P2rx6
9	252904	V1re9
9	74700	4930524O08Rik
9	75030	4930503E24Rik
9	258345	Olfr1121
9	74978	Lrriq1
9	16061	Igh-VJ558
9	381350	BC061194
9	23850	Pappa2
9	103677	Smg6
9	14738	Gpr12
9	21375	Tbr1
9	23928	Lamc3
9	67719	2310057J18Rik
9	68527	1110017I16Rik
9	NaN	MMT00020922
9	77700	9130208D14Rik
9	18352	Olfr52
9	78257	Lrrc9
9	NaN	MMT00077775
9	394252	Serpinb3d
9	259017	Olfr1019
9	NaN	MMT00045517
9	360214	Defb39
9	11657	Alb
9	12352	Car5a
9	75484	1700007H22Rik
9	NaN	MMT00063621
9	385493	AU015836
9	21667	Tdgf1
9	14125	Fcer1a
9	83565	Pramel3
9	67366	1810008K16Rik
9	NaN	MMT00014316
9	240894	Fmo9
9	73191	Fezf1
9	13089	Cyp2b13
9	14619	Gjb2
9	NaN	MMT00037949
9	232358	Vmn2r19
9	78443	A930041C12Rik
9	216225	Slc5a8
9	235386	C630028N24Rik
9	13417	Dnahc8
9	21639	Tcrg-V5
9	77011	5730590G19Rik
9	435927	EG435927

9	NaN	MMT00020226
9	545291	MMT00030912
9	239157	Pnma2
9	381538	Gm1027
9	23793	Adam25
9	232836	Galp
9	20599	Smr3a
9	71421	Amtn
9	268482	Krt12
9	74441	Slco6c1
9	20234	Sbp
9	384422	Igk-V8
9	258529	Olfr313
9	NaN	MMT00028550
9	NaN	MMT00059108
9	381229	Ccdc147
9	66617	2610205E22Rik
9	268755	A930011O12Rik
9	414071	BC028471
9	245650	Gucy2f
9	100041581	Zkscan16
9	28250	Slco1a4
9	15891	Ibsp
9	258843	Olfr1087
9	69693	Pof1b
9	237107	Gnl3l
9	NaN	MMT00007654
9	NaN	MMT00046813
9	19256	Ptpn20
9	627984	Nlrp1c
9	NaN	MMT00042391
9	114660	Etos1
9	640530	EG640530
9	23927	Krtap14
9	258287	Olfr125
9	229780	Ccdc76
9	NaN	MMT00022090
9	13239	Defcr5
9	20690	Spam1
9	113851	V1ra9
9	620631	Ttc30a2
9	18183	Nrg3
9	635702	Naaladl2
9	NaN	MMT00026815
9	11922	Neurod6
9	52028	Bbs1
9	246691	Prok1
9	224916	Vmn2r120
9	70086	Cysltr2
9	71508	8430426H19Rik
9	319800	C730048C13Rik

9	545391	EG545391
9	77706	Abcb5
9	380836	Mrs2
9	51885	Tubgcp4
9	226470	Zbtb41
9	55993	Msh4
9	52428	Rhpn2
9	252838	Tox
9	20397	Sgpl1
10	74100	MMT00001343
10	74183	2310042D19Rik
10	216739	Acsl6
10	12715	Ckm
10	17901	Myl1
10	74166	Tmem38a
10	244923	Klhl31
10	11474	Actn3
10	13808	Eno3
10	21955	Tnnt1
10	11565	Adssl1
10	12555	Cdh15
10	68680	1110028A07Rik
10	22138	Ttn
10	21953	Tnni2
10	53311	Mybph
10	11937	Atp2a1
10	268859	MMT00006152
10	21925	Tnnc2
10	56012	Pgam2
10	140781	Myh7
10	68802	Mypn
10	11472	Actn2
10	229665	Ampd1
10	22138	Ttn
10	76469	Cmya5
10	233199	Mybpc2
10	59011	Myoz1
10	11811	Apobec2
10	22003	Tpm1
10	59006	Myoz2
10	66106	Smpx
10	17189	Mb
10	22138	Ttn
10	21957	Tnnt3
10	17907	Mylpf
10	68678	Smtnl1
10	12292	Cacna1s
10	12862	Cox6a2
10	22138	Ttn
10	17885	Myh8
10	24131	Ldb3

10	15464	Hrc
10	228003	Kbtbd10
10	69585	Hfe2
10	12391	Cav3
10	76722	Ckmt2
10	66211	Rpl3l
10	17306	Sypl2
10	74843	Zmynd17
10	20190	Ryr1
10	56534	Hspb3
10	21384	Tbx15
10	13009	Csrp3
10	21924	Tnnc1
10	17882	Myh2
10	17996	Neb
10	22138	Ttn
10	59069	Tpm3
10	329502	Pla2g4e
10	17878	Myf6
10	241431	Xirp2
10	76294	Asb5
10	378431	Txlnb
10	50874	Tmod4
10	17897	Myl3
10	21828	Thbs4
10	68515	1110012N22Rik
10	69564	Itgb1bp3
10	11459	Acta1
10	223513	Abra
10	11875	Art5
10	56504	Srpk3
10	12335	Capn3
10	83679	Pde4dip
10	17906	Myl2
10	69700	Col22a1
10	53318	Pdlim3
11	75469	Spata19
11	77963	Hook1
11	76378	Ropn1
11	70894	4921510J17Rik
11	67342	1700058G18Rik
11	66338	Cdrt4
11	76653	Cby3
11	13524	Adam18
11	75483	Cox8c
11	69314	1700011H22Rik
11	18285	Odf1
11	320604	A730037C10Rik
11	69317	Hmgb4
11	434285	BB014433
11	242408	4930412F15Rik

11	67929	Ccdc70
11	69357	RP23-438H3.2
11	21884	Fabp9
11	77945	Rpgrip1
11	14028	Evx1
11	17002	Ltf
11	12231	Btn1a1
11	109648	Npy
11	228151	4833423E24Rik
11	210321	BC048679
11	269328	Muc15
11	56315	Rhcg
11	13608	Edar
11	69134	2200001I15Rik
11	66255	1810005K13Rik
11	547323	MMT00078134
11	11500	Adam7
11	77704	Lcn9
11	78709	Spink8
11	328971	Spink10
11	11997	Akr1b7
11	78128	Spag11
11	77015	Mpped2
11	67874	Rprm
11	18991	Pou3f1
11	381845	2310014L17Rik
11	68243	A930018P22Rik
11	14800	Gria2
11	13012	Cst8
11	14858	Gsta2
11	70163	2210415F13Rik
11	77684	9230107M04Rik
11	68171	D730048I06Rik
11	17057	Klrb1a
11	78242	Spink12
11	12097	Bglap2
11	231293	C130090K23Rik
11	14473	Gc
11	100038927	MMT00025642
11	16939	Lor
11	246082	Defb15
11	15874	Iapp
11	77701	Lcn12
11	26366	Ceacam10
11	246084	Defb35
11	16665	Krt15
11	240817	5830403L16Rik
11	11572	Crisp3
11	15930	Indo
11	12095	Bglap-rs1
11	12269	C4bp

11	11571	Crisp1
11	403172	Defb21
11	629754	OTTMUSG00000001088
11	78240	Cst11
11	78076	Lcn8
11	67935	Ces7
11	66183	1110032A04Rik
11	22270	Upk3a
11	72434	Lypd3
11	15375	Foxa1
11	20760	Sprr2f
11	14623	Gjb6
11	16622	Klk1b5
11	104174	Gldc
11	13214	Defb1
11	20724	Serpinb5
11	83984	Tssk6
11	211147	11-Mar
11	68265	Iqcf3
11	70069	H1fnt
11	219038	Foxo3
11	69423	1700019M22Rik
11	73302	1700031M16Rik
11	19119	Prm2
11	53814	Oaz3
11	16833	Ldhc
11	13074	Cyp17a1
11	17235	Smcp
11	75528	1700018L24Rik
11	100038417	ENSMUSG00000073551
11	110326	Tas1r1
11	66696	Snx31
11	73722	Lce1a2
11	69301	1700008P20Rik
12	214922	Slc39a2
12	226539	Dars2
12	107141	Cyp2c50
12	13101	Cyp2d10
12	258960	Olfr171
12	NaN	MMT00003591
12	100041375	Cyp3a41b
12	70902	Aytl1b
12	71760	Agxt2l1
12	171195	V1rc22
12	245880	Wasf3
12	100019	Mdn1
12	108114	Slc22a7
12	380714	Rph3al
12	629499	4922505G16Rik
12	97487	Cmtm4
12	330401	Tmcc1

12	230857	Ece1
12	74549	9130404D08Rik
12	74055	Plce1
12	27400	Hsd17b6
12	19125	Prodh
12	83997	Slmap
12	18628	Per3
12	72502	Cwf19l1
12	100727	Ugt2b34
12	18815	Plg
12	279028	Adamts13
12	320360	Ric3
12	19116	Prlr
12	NaN	MMT00074228
12	72297	B3gnt3
12	18705	Pik3c2g
12	58198	Sall1
12	209091	Ccnb3
12	368202	Gm1019
12	56219	Extl1
12	218973	Wdhd1
12	12654	Chi3l1
12	257871	Olfr1372-ps1
12	NaN	MMT00011155
12	NaN	MMT00052319
12	NaN	MMT00031931
12	NaN	MMT00066154
12	NaN	MMT00007367
12	21906	Otop1
12	27052	Aoah
12	22773	Zic3
12	24001	Tiam2
12	78547	E130304I02Rik
12	76770	2010005H15Rik
12	235952	Gm189
12	14807	Grik3
12	238205	Lrfn5
12	NaN	MMT00072061
12	16875	Lhx8
12	64297	Gprc5b
12	258692	Olfr1442
12	74175	Crct1
12	258954	Olfr522
12	192174	Rwdd4a
12	NaN	MMT00039919
12	258272	Olfr1402
12	NaN	MMT00077643
12	18798	Plcb4
12	68054	Serpina12
12	320873	Cdh10
12	242286	4833413O15Rik

12	15485	Hsd17b1
12	29862	Ninj2
12	259010	Olfr393
12	68507	Ppfia4
12	NaN	MMT00007523
12	258645	Olfr1403
12	100042314	ENSMUSG00000074179
12	NaN	MMT00064181
12	26423	Nr5a1
12	74143	Opa1
12	NaN	MMT00006980
12	72503	2610507B11Rik
12	12142	Prdm1
12	21948	Cd70
12	23872	Ets2
12	68837	Foxk2
12	NaN	MMT00058440
12	208151	Tmem132b
12	224024	Scarf2
12	56191	Tro
12	100040843	Cyp4a32
12	234199	Fgl1
12	207667	Lbxcor1
12	242748	Ptchd2
12	19752	Rnase1
12	NaN	MMT00068134
12	67608	Narf
12	109697	Cpa1
12	NaN	MMT00077820
12	72007	Fndc3b
12	16866	Lhb
12	NaN	MMT00043344
12	71768	Vwce
12	229613	6330549D23Rik
12	NaN	MMT00014972
12	72668	2810030E01Rik
12	636808	Cntnap5a
12	218613	Mier3
12	84506	Hamp
12	110135	Fgb
12	113847	V1ra5
12	20863	Stfa3
12	58218	Trem3
12	100102	Pcsk9
12	258811	Olfr926
12	21679	Tead4
12	245615	Kir3dl2
12	NaN	MMT00078109
12	72607	Usp13
12	20211	Saa4
12	13096	Cyp2c37

12	258987	Olfr1270
12	619301	G630016D24Rik
12	382035	MMT00056309
12	319239	Npsr1
12	14079	Fabp2
12	67133	Gp2
12	13406	Dmp1
12	435889	1810049H19Rik
12	52250	Reep1
12	NaN	MMT00005917
13	108078	Olr1
13	13733	Emr1
13	14728	Lilrb4
13	14255	Flt3
13	212167	A530088I07Rik
13	74076	4933406C10Rik
13	234407	Glt25d1
13	232685	AB041803
13	76709	Arpc2
13	20932	Surf4
13	21844	Tiam1
13	75665	Ccdc64
13	327900	Ubtd2
13	60322	Chst7
13	17285	Meox1
13	66650	Nepn
13	63953	Dusp10
13	14468	Gbp1
13	12363	Casp4
13	14991	H2-M3
13	19139	Prps1
13	71712	1200002N14Rik
13	19227	Pthlh
13	21789	Tfpi2
13	74760	Rab3il1
13	52614	Emr4
13	664862	Gpr137b-ps
13	73341	Arhgef6
13	59036	Dact1
13	105689	Mycbp2
13	234094	Arhgef10
13	77569	Limch1
13	224273	BC043118
13	18003	Nedd9
13	209630	Frmd4a
13	544963	Iqgap2
13	18768	Pkib
13	14130	Fcgr2b
13	66190	Phca
13	21930	Tnfaip6
13	15117	Has2

13	72575	C430049B03Rik
13	18791	Plat
13	21813	Tgfbr2
13	108927	Lhfp
13	20319	Sfrp2
13	69538	Antxr1
13	20363	Sepp1
13	22224	Usp10
13	71738	Mamdc2
13	16773	Lama2
13	50706	Postn
13	16004	Igf2r
13	83671	Sytl2
13	11624	Ahrr
13	56792	Stap1
13	56644	Clec7a
13	12522	Cd83
13	19264	Ptprc
13	57781	Cd200r1
13	12508	Cd53
13	17087	Ly96
13	12506	Cd48
13	26888	Clec4a2
13	12524	Cd86
13	17533	Mrc1
13	15939	Ier5
13	77579	Myh10
13	227157	Mpp4
13	53603	Tslp
13	76184	Abca6
14	12362	Casp1
14	216363	Rab3ip
14	53376	Usp2
14	56018	Stard10
14	215929	AI317395
14	218865	Chdh
14	50905	Il17rb
14	66433	Chchd7
14	319582	6430573F11Rik
14	66611	Ribc1
14	211323	Nrg1
14	69299	Asb9
14	213575	Dync2li1
14	232875	Zscan18
14	170753	Zfp704
14	NaN	MMT00078660
14	22038	Plscr1
14	18828	Plscr2
14	80890	Trim2
14	74019	Traf3ip1
14	18645	Pfn2

14	234915	AK129341
14	105439	Slain1
14	67492	Anubl1
14	328192	MMT00012324
14	232146	Tmem166
14	93761	Smarca1
14	66569	Gdpd1
14	69698	2310046K01Rik
14	233733	Galntl4
14	110197	Dgkg
14	226654	EG226654
14	20347	Sema3b
14	17240	Mdfi
14	14632	Gli1
14	217335	Fbf1
14	232970	Phldb3
14	211389	Suox
14	380694	Ccnjl
14	24117	Wif1
14	110385	Pde4c
14	50781	Dkk3
14	233335	Dmn
14	665033	EG665033
14	66601	Tmigd1
14	71213	Cage1
14	12228	Btg3
14	12228	Btg3
14	14853	Gspt2
14	99681	Tchh
14	237175	Gpr64
14	71893	Noxo1
14	14772	Grk4
14	67298	Gprasp1
14	213081	Wdr19
14	19659	Rbp1
14	19331	Rab19
14	192651	Zfp286
14	69772	Bdh2
14	192167	Nlgn1
14	20230	Satb1
14	209268	Igsf1
14	98396	Slc41a1
14	16440	Itpr3
14	100041639	MMT00053163
14	14766	Gpr56
14	216831	AU040829
14	17357	Marcksl1
14	NaN	MMT00039914
14	217882	AW555464
14	54324	Arhgef5
14	209588	Sectm1a

14	99662	Eps8l3
14	69364	1700012P22Rik
14	192201	Wfdc15b
14	71837	1700003E16Rik
14	71840	Tekt4
14	15417	Hoxb9
14	74453	Ccdc11
14	66353	2310007A19Rik
14	13195	Ddc
14	56739	Rec8
14	171281	Acot3
14	69836	Pla2g12b
14	384198	Gm1381
15	231507	Plac8
15	74153	Ube1l
15	17071	Ly6f
15	110454	Ly6a
15	NaN	MMT00008674
15	631323	MMT00028135
15	56417	Adar
15	327959	Xaf1
15	17069	Ly6e
15	16859	Lgals9
15	23960	Oas1g
15	69550	Bst2
15	229003	BC006779
15	16912	Psmb9
15	16913	Psmb8
15	217069	Trim25
15	80861	Dhx58
15	NaN	MMT00030347
15	547253	Parp14
15	23962	Oasl2
15	246728	Oas2
15	16145	Igtp
15	15953	Ifi47
15	243771	Parp12
15	54123	Irf7
15	24110	Usp18
15	17858	Mx2
15	15957	Ifit1
15	15959	Ifit3
15	67775	Rtp4
15	60440	Iigp1
15	21822	Tgtp
15	54396	Iigp2
15	99899	Ifi44
15	19039	Lgals3bp
15	246730	Oas1a
15	NaN	MMT00036681
15	15958	Ifit2

15	76074	5830443L24Rik
15	629974	Rnf213
15	219132	D14Ertd668e
15	219131	Phf11
15	55932	Gbp3
15	14938	Gzma
15	14963	H2-Bl
15	16068	Il18bp
16	15233	Hgd
16	66740	4931417E11Rik
16	NaN	MMT00044569
16	319804	Glt1d1
16	74468	Hyal5
16	68355	2010204K13Rik
16	NaN	MMT00077977
16	NaN	MMT00039710
16	330721	Nek5
16	259030	Olfr1125
16	20129	Rptn
16	70897	4921509E07Rik
16	75886	Gstt4
16	69347	1700008P02Rik
16	619288	4933417M04Rik
16	NaN	MMT00034062
16	74458	4933425B07Rik
16	258042	Olfr487
16	NaN	MMT00045951
16	68307	4930558O21Rik
16	258713	Olfr430
16	258579	Olfr1018
16	258571	Olfr1033
16	NaN	MMT00029272
16	67944	1700025D03Rik
16	629820	EG629820
16	258546	Olfr806
16	258541	Olfr800
16	NaN	MMT00008723
16	NaN	MMT00046779
16	14985	H2-M10.1
16	17139	Magea3
16	77668	9130209A04Rik
16	634346	EG634346
16	66654	Tex12
16	NaN	MMT00048365
16	74933	4930469G21Rik
16	258282	Olfr801
16	109821	F11
16	623279	Dok6
16	233060	Zfp382
16	16061	Igh-VJ558
16	NaN	MMT00007193

16	16867	Lhcgr
16	12374	Casr
16	NaN	MMT00004568
16	67892	1810063B05Rik
16	74156	Acot12
16	242851	Gnat3
16	320772	Mdga2
16	74249	Lrrc2
16	11676	Aldoc
16	15560	Htr2c
16	73090	2900092C05Rik
16	75647	1700025E21Rik
16	12788	Cnga1
16	105355	Slc17a3
16	230099	Car9
16	545798	MMT00055433
16	71275	4933437F05Rik
16	16061	Igh-VJ558
16	NaN	MMT00070220
16	70866	Slco6d1
16	57764	Ntn4
16	16061	Igh-VJ558
16	258789	Olfr1271
16	668257	Dgat2l6
16	627814	Vmn2r109
16	NaN	MMT00040811
16	102022	Ces6
16	243537	Uroc1
16	258377	Olfr654
16	70821	4921507P07Rik
16	NaN	MMT00002620
16	100038941	Vmn2r121
16	NaN	MMT00058344
16	226025	Trpm3
16	70952	4921537P18Rik
16	74672	4930449A18Rik
16	NaN	MMT00008763
16	116852	Akr1c20
16	258724	Olfr784
16	NaN	MMT00022745
16	239857	Cadm2
16	NaN	MMT00067361
16	209032	Zc3hav1l
16	546983	Vmn2r77
16	67648	4930542C12Rik
16	209186	C730036D15Rik
16	216454	BC089597
16	67937	Tmem59l
16	NaN	MMT00047047
16	19885	Rorc
16	NaN	MMT00059186

16	NaN	MMT00008480
16	58991	Ghrl
16	NaN	MMT00069497
16	NaN	MMT00014869
16	74469	Taf7l
16	NaN	MMT00059569
16	259117	Olfr560
17	72795	Ttc19
17	14732	Gpam
17	68876	Xrcc6bp1
17	241113	Prkag3
17	15442	Hpse
17	99439	Duox1
17	68149	Otub2
17	330460	BC022651
17	74080	Nmnat3
17	69387	Dnajb13
17	103161	Apof
17	19171	Psmb10
17	74481	Batf2
17	71898	Apol9b
17	20555	Slfn1
17	16601	Klf9
17	67030	Fancl
17	79560	Ublcp1
17	225791	Zadh2
17	52440	Tax1bp1
17	66302	2410005O16Rik
17	16478	Jund
17	11532	Adh5
17	11532	Adh5
17	102182	AI931714
17	15502	Dnaja1
17	71030	4933403O08Rik
17	68493	1110007M04Rik
17	19243	Ptp4a1
17	140570	Plxnb2
17	59040	Rhot1
17	17850	Mut
17	227197	Ndufs1
17	667832	MMT00021210
17	76947	2310030N02Rik
17	23939	Mapk7
17	14678	Gnai2
17	17925	Myo9b
17	50926	Hnrpdl
17	16779	Lamb2
17	232807	Ppp1r12c
17	20130	Rras
17	80876	Ifitm2
17	16206	Lrig1

17	76219	6530401D17Rik
17	60344	Fign
17	12631	Cfl1
17	76976	2900062L11Rik
17	18576	Pde3b
17	12015	Bad
17	19646	Rbbp4
17	71393	Kctd6
17	19246	Ptpn1
17	110842	Etfa
17	59027	Nampt
17	545260	Arsi
17	70616	Sf4
17	235323	Usp28
17	56437	Rrad
17	76478	2410004L22Rik
17	219144	Arl11
17	20556	Slfn2
17	12265	Ciita
17	320435	5830482F20Rik
17	59014	Rrs1
17	71991	Ercc8
17	72184	2810406K13Rik
17	NaN	MMT00030762
17	668450	EG668450
17	235132	Zbtb44
17	72554	Utp14a
17	633387	MMT00052835
17	60532	Wtap
17	108943	Rg9mtd2
17	54381	MMT00040501
17	381695	N4bp2l2
18	104110	Adcy4
18	140721	Caskin2
18	381379	Med19
18	20448	St6galnac4
18	13870	Ercc1
18	94242	Tinagl
18	104027	Synpo
18	434215	Lrrc32
18	22368	Trpv2
18	242691	Gpatch3
18	11512	Adcy6
18	217127	Myst2
18	105844	Card10
18	22344	Vezf1
18	76854	Gper
18	50780	Rgs3
18	228788	BC020535
18	15248	Hic1
18	84094	Plvap

18	75687	2310066E14Rik
18	15284	Hlx
18	192652	Wdr81
18	57783	Tnip1
18	107375	Slc25a45
18	69553	2310033K02Rik
18	74239	Iqce
18	214597	Sidt2
18	106628	Trip10
18	232879	Zbtb45
18	209011	Sirt7
18	59092	Pcbp4
18	66513	Map3k7ip1
18	66940	Shisa5
18	15896	Icam2
18	74772	Atp13a2
18	78388	Mvp
18	103724	Tbc1d10a
18	51800	Bok
18	230837	Ddefl1
18	269633	Wdr86
18	70192	Cd209g
18	102545	Cmtm7
18	16170	Il16
18	12984	Csf2rb2
18	12703	Socs1
18	15976	Ifnar2
18	26377	Dapp1
18	17873	Gadd45b
18	11307	Abcg1
18	230908	Tardbp
18	100198	H6pd
18	208659	BC029169
18	12091	Glb1
18	14026	Evl
18	17159	Man2b1
18	23876	Fbln5
18	219151	Scara3
18	12258	Serping1
18	210622	E430002G05Rik
18	15277	Hk2
18	21825	Thbs1
18	268534	Sntg2
18	11486	Ada
18	73683	Atg16l2
18	14133	Fcna
18	231633	Tmem119
18	16012	Igfbp6
18	68794	Flnc
18	16190	Il4ra
18	66102	Cxcl16

18	70727	Rasgef1a
18	19124	Procr
18	56175	Bace2
18	109264	Me3
18	17228	Cma1
18	17395	Mmp9
18	69534	Avpi1
18	16330	Inpp5b
18	22158	Tulp3
18	67604	1110007L15Rik
18	13636	Efna1
18	72322	Xpo5
18	72635	Lins2
18	18226	Nup62
18	103213	Traf3ip2
18	67393	Cxxc5
18	217166	Nr1d1
18	217827	BC002230
18	229473	D930015E06Rik
18	20969	Sdc1
18	68964	1500010J02Rik
18	20811	Srms
18	101540	Prkd2
18	12351	Car4
18	NaN	MMT00057509
18	19099	Mapk8ip1
18	101543	Wtip
18	16369	Irs3
19	NaN	MMT00079689
19	NaN	MMT00069487
19	NaN	MMT00043009
19	15382	Hnrnpa1
19	15382	Hnrnpa1
19	67115	Rpl14
19	68011	Snrpg
19	73212	3110082I17Rik
19	69263	Rfc3
19	69430	1700048O20Rik
19	20821	Trim21
19	16443	Itsn1
19	208043	Setd1b
19	12469	Cct8
19	NaN	MMT00053207
19	NaN	MMT00082629
19	NaN	MMT00059984
19	NaN	MMT00050545
19	57890	Il17re
19	11844	Arf5
19	21422	Tcfcp2
19	67041	Oxct1
19	NaN	MMT00046581

19	NaN	MMT00034261
19	NaN	MMT00020332
19	67704	1810037I17Rik
19	30954	Siva1
19	NaN	MMT00045890
19	30954	Siva1
19	67711	Nsmce1
19	NaN	MMT00048286
19	NaN	MMT00069486
19	NaN	MMT00011814
19	NaN	MMT00036520
19	NaN	MMT00042303
19	NaN	MMT00045906
19	NaN	MMT00048939
19	NaN	MMT00047531
19	NaN	MMT00007455
19	14388	Gab1
19	NaN	MMT00040108
19	NaN	MMT00024951
19	NaN	MMT00059805
19	NaN	MMT00056634
19	NaN	MMT00004769
19	NaN	MMT00056589
19	NaN	MMT00071327
19	NaN	MMT00051740
19	229746	EG229746
19	NaN	MMT00007741
19	20044	Rps14
19	NaN	MMT00048362
19	100041686	MMT00047654
20	28000	Prpf19
20	72145	Wdfy3
20	242608	Podn
20	12521	Cd82
20	77407	Rab35
20	14312	Brd2
20	22601	Yap1
20	30050	Fbxw2
20	68095	Ociad1
20	16994	Ltb
20	269003	Sap130
20	77652	MMT00072863
20	68585	Rtn4
20	75608	Chmp4b
20	74747	Ddit4
20	214987	5830457O10Rik
20	19383	Raly
20	108654	4933403F05Rik
20	72401	Slc43a1
20	22032	Traf4
20	68097	Dynll2

20	233979	Tpcn2
20	319156	Hist1h4d
20	14113	Fbl
20	66395	Ahnak
20	225467	Pggt1b
20	67564	Tmem35
20	238330	6430527G18Rik
20	433864	Gm1040
20	94178	Mcoln1
20	NaN	MMT00015250
20	99311	Commd7
20	68988	Prpf31
20	66089	Rmnd5b
20	15368	Hmox1
20	66146	Tmem57
20	67511	Tmed9
20	114584	Clic1
20	56440	Snx1
20	20610	Sumo3

Table T5: Hypothalamus Modules

Module	Locus Link	Gene Symbol
1	66994	1500031L02Rik
1	381148	Gm1614
1	77622	Apex2
1	22433	Xbp1
1	13435	Dnmt3a
1	67014	Mina
1	233649	Cnga4
1	66369	Dus2l
1	208715	Hmgcs1
1	216190	Appl2
1	66477	Usmg5
1	94226	S1pr5
1	13497	Drp2
1	16780	Lamb3
1	26423	Nr5a1
1	18508	Pax6
1	20935	Surf6
1	12684	Cideb
1	27373	Csnk1e
1	15976	Ifnar2
1	100226	Stx12
1	16428	Itk
1	20311	Cxcl5
1	64705	Dpys
1	56046	Uqcc
1	15122	Hba-a1
1	18092	Nkx2-6
1	208659	BC029169
1	17937	Nab2
1	16971	Lrp1
1	83669	Wdr6
1	72993	Appl1
1	18377	Omg
1	19092	Prkg2
1	27281	Hrasls
1	74108	Parn
1	666704	Samd1
1	58243	Nap1l5
1	27981	D4Wsu53e
1	628438	EG628438
1	252864	Dusp15
1	14860	Gsta4
1	71268	Lrrfip2
1	18546	Pcp4
1	381678	Zcwpw1
1	231872	MMT00063115
1	17713	Grpel1
1	59036	Dact1
1	85305	Kars

1	22608	Ybx1
1	216198	Tcp11l2
1	320267	Fubp3
1	407788	BC051142
1	235339	Dlat
1	68876	Xrcc6bp1
1	210106	Pols
1	80292	Zxdc
1	13653	Egr1
1	NaN	MMT00062394
1	66407	Mrps15
1	226250	Afap1l2
1	11432	Acp2
1	68038	Chid1
1	69071	Tmem97
1	23983	Pcbp1
1	76338	Rab2b
1	68975	Med27
1	22608	Ybx1
1	18027	Nfia
1	194612	ENSMUSG00000046088
1	338417	Scgb1c1
1	217779	Lysmd1
1	140492	Kcnn2
1	20813	Srp14
1	18417	Cldn11
1	72399	Brap
1	235493	BC031353
1	NaN	MMT00065821
1	70163	2210415F13Rik
1	19692	Reg1
1	258857	Olfr275
1	58235	Pvrl1
1	78286	Nav2
1	258822	Olfr39
1	22073	Prss3
1	69399	1700025G04Rik
1	239037	Lrit1
1	NaN	MMT00041452
1	NaN	MMT00071843
1	16061	Igh-VJ558
1	243262	Oas1f
1	101604	E430018J23Rik
1	226049	Dmrt2
1	17189	Mb
1	54721	Tyk2
1	100048814	MMT00082139
1	68678	Smtnl1
1	67772	Chd8
1	18405	Orm1
1	319716	6430520M22Rik

1	16523	Kcnj8
1	72607	Usp13
1	17857	Mx1
1	20541	Slc8a1
1	52513	Ddx56
1	330836	Slc7a6
1	269378	Ahcy
1	18430	Oxtr
1	75731	5133401N09Rik
1	217370	BC017643
1	668940	Myh7b
1	56089	Ramp3
1	76574	Mfsd2
1	28248	Slco1a1
1	74754	Dhcr24
1	224105	Pak2
1	224481	Tfb1m
1	15257	Hipk1
1	93873	Pcdhb2
1	68888	1190003M12Rik
1	239691	AU021092
1	227696	Phyhd1
1	11863	Arnt
1	20249	Scd1
1	12447	Ccne1
1	99031	Osbpl6
1	76539	D19Ertd737e
1	212937	Tifab
1	74549	9130404D08Rik
1	56496	Tspan6
1	NaN	MMT00077385
1	77411	Rbm35b
1	214642	A430107O13Rik
1	27762	D17H6S56E-3
1	11472	Actn2
1	50911	Exosc9
1	22065	Trpc3
1	56448	Cyp2d22
1	20962	Sycp3
1	84094	Plvap
1	53857	Tuba8
1	20305	Ccl6
1	73732	Muc16
1	67758	Aadac
1	58206	Zbtb32
1	233575	MMT00014327
1	15985	Cd79b
1	20017	Rpo1-2
1	22092	Rsph1
1	11781	Ap4m1
1	52662	D18Ertd653e

1	217700	Acot6
1	330355	Dnahc6
1	71870	Ccdc19
1	70853	4921511C04Rik
1	66748	4933404M02Rik
1	18861	Pms2
1	50498	Ebi3
1	68039	Nmb
1	18817	Plk1
1	17829	Muc1
1	105844	Card10
1	77505	Dnhd1
1	66185	1110037F02Rik
1	54188	Cpsf4
1	110208	Pgd
1	72199	Mms19
1	57357	Srd5a3
1	64385	Cyp4f14
1	74166	Tmem38a
1	230996	9430015G10Rik
1	68396	Cml4
1	56809	Gmeb1
1	106068	Slc45a4
1	73112	3110003A17Rik
1	66959	Dusp26
1	98267	Stk17b
1	54598	Calcrl
1	117167	Steap4
1	108151	Sema3d
1	214403	EG214403
1	NaN	MMT00078660
1	22038	Plscr1
1	212898	Dse
1	20379	Sfrp4
1	70717	6330406I15Rik
1	16952	Anxa1
1	54124	Cks1b
1	14231	Fkbp7
1	18828	Plscr2
1	54381	MMT00040501
1	68151	Gpr177
1	17112	Tm4sf1
1	71586	Ifih1
1	55932	Gbp3
1	15944	Irgm
1	109857	Cbr3
1	622657	EG622657
1	18791	Plat
1	29817	Igfbp7
1	16600	Klf4
1	68797	Pdgfrl

1	12010	B2m
1	18155	Pnoc
1	22329	Vcam1
1	13733	Emr1
1	NaN	MMT00062592
1	22441	Xlr
1	14137	Fdft1
1	NaN	MMT00082285
1	76137	Ccdc90a
1	22044	Trh
1	80859	Nfkbiz
1	NaN	MMT00050551
1	110902	Chrna2
1	217069	Trim25
1	13489	Drd2
1	407243	Tmem189
1	19730	Ralgds
1	74838	Narg1
1	23980	Pebp1
1	67023	Use1
1	75104	Mmd2
1	217827	BC002230
1	231583	Slc26a1
1	218543	Sfrs12
1	239827	Pigz
1	76408	Abcc3
1	74490	5430432N15Rik
1	72049	Tnfrsf13c
1	213956	AW544981
1	242785	Klhl21
1	23939	Mapk7
1	51798	Ech1
1	14866	Gstm5
1	233833	Tnrc6a
1	170439	Elovl6
1	69721	Nkiras1
1	234796	BC025816
1	93880	Pcdhb9
1	56715	Rabgef1
1	52592	Brms1l
1	21407	Tcf15
1	109161	Ube2q2
1	27215	Azi2
1	72508	Rps6kb1
1	74453	Ccdc11
1	67994	Mrps11
1	53378	Sdcbp
1	232807	Ppp1r12c
1	NaN	MMT00048325
1	71997	1500002O20Rik
1	50908	C1s

1	NaN	MMT00050540
1	NaN	MMT00042003
1	67211	Armc10
1	381695	N4bp2l2
1	66552	2010106G01Rik
1	11474	Actn3
1	17294	Mest
1	380969	C230021P08Rik
1	12009	Azi1
1	66314	Tpd52l2
1	30055	Timm13
1	214901	Chtf18
1	NaN	MMT00004670
1	71564	9030607L17Rik
1	227659	Slc2a6
1	NaN	MMT00014691
1	11772	Ap2a2
1	21786	Tff3
1	100041835	MMT00026054
1	100041835	MMT00002146
1	257635	Sdsl
1	215210	Tmem120a
1	83679	Pde4dip
1	20540	Slc7a7
1	381038	Parl
1	11861	Arl4a
1	234875	Ttc13
1	72795	Ttc19
1	52829	D4Bwg0951e
1	317750	Slc24a5
1	67457	Frmd8
1	20523	Slc25a14
1	60525	Acss2
1	233445	Vmn2r71
1	52174	D4Ertd196e
1	433375	Creg1
1	665565	MMT00055950
1	NaN	MMT00077009
1	215418	Axud1
1	109168	5730596K20Rik
1	79059	Nme3
1	19365	Rad52
1	14432	Gap43
1	11758	Prdx6
1	66845	Mrpl33
1	NaN	MMT00053207
1	56016	Hebp2
1	66179	1110031I02Rik
1	100434	Slc44a1
1	76813	Armc6
1	72325	1300018I17Rik

1	NaN	MMT00045078
1	20924	Supt5h
1	28080	Atp5o
1	73830	Eif3k
1	23918	Impdh2
1	215051	Bud13
1	102448	Xylb
1	19325	Rab10
1	11544	Adprh
1	NaN	MMT00035807
1	101142	Itfg2
1	75471	1700009N14Rik
1	71968	Wdr73
1	232430	Crebl2
1	11982	Atp10a
1	110816	Pwp2
1	110816	Pwp2
1	14104	Fasn
1	NaN	MMT00021936
1	246703	Apoa1bp
1	14419	Gal
1	214663	Slc25a29
1	66170	Chchd5
1	16061	Igh-VJ558
1	22177	Tyrobp
1	11972	Atp6v0d1
1	12759	Clu
1	13014	Cstb
1	19012	Ppap2a
1	67655	Ctdp1
1	14085	Fah
1	17925	Myo9b
1	223649	Nrbp2
1	15504	Dnajb3
1	69131	Crkrs
1	50874	Tmod4
1	27261	Dok3
1	63872	Zfp296
1	228790	Asxl1
1	399591	4930488E11Rik
1	19354	Rac2
1	108100	Baiap2
1	213006	Mfsd4
1	20186	Nr1h4
1	12123	Hrk
1	57264	Retn
1	15473	Hrsp12
1	20555	Slfn1
1	66538	Rps19bp1
1	12877	Cpeb1
1	12292	Cacna1s

1	17826	Mtvr2
1	192232	Hps4
1	69719	Cad
1	68183	Bcas2
1	71893	Noxo1
1	20778	Scarb1
1	214048	Larp2
1	72016	1600002H07Rik
1	71720	Osbpl3
1	320415	Gchfr
1	140919	Slc17a6
1	68477	Rmnd5a
1	12283	Cab39
1	20437	Siah1a
1	19876	Robo1
1	68877	Maf1
1	NaN	MMT00041792
1	74277	Chic2
1	67092	Gatm
1	100121	Tdrd7
1	15528	Hspe1
1	271377	Zbtb11
1	54561	Nap1l3
1	209630	Frmd4a
1	243743	Plxna4
1	320365	Fry
1	226747	Ahctf1
1	114642	Brdt
1	212555	Pqlc2
1	20185	Ncor1
1	17035	Lxn
1	18654	Pgf
1	415115	Neurl2
1	66431	1810049H13Rik
1	209012	Ulk4
1	76866	Morn1
1	18132	Notch4
1	66853	Pnpla2
1	333654	Ppp1r13l
1	54409	Ramp2
1	20130	Rras
1	27361	Sepx1
1	70877	Micalcl
1	83701	MMT00026414
1	270076	Gcdh
1	245651	EG245651
1	215951	Lace1
1	627110	Tubb2a-ps2
1	67186	Rplp2
1	NaN	MMT00082629
1	69010	Anapc13

1	80914	Uck2
1	208076	Pknox2
1	627312	MMT00039341
1	79196	Osbpl5
1	232210	8430410A17Rik
1	237361	EG237361
1	98766	Ubac1
1	19079	Prkab1
1	14609	Gja1
1	29820	Tnfrsf19
1	268656	Sptlc1
1	66333	Aqp11
1	26932	Ppp2r5e
1	116748	Lsm10
1	66229	Rpl7l1
1	67711	Nsmce1
1	103844	AI842396
1	111175	Pecr
1	64424	Polr1e
1	192185	Nadk
1	101543	Wtip
1	243547	Grip2
1	14979	H2-Ke6
1	171429	Slc26a6
1	233977	Ppfia1
1	233107	Kctd15
1	12450	Ccng1
1	50776	Polg2
1	NaN	MMT00072901
1	66928	3110001D03Rik
1	269955	Rccd1
1	66090	Ypel3
1	12497	Entpd6
1	105670	Rcbtb2
1	72635	Lins2
1	21665	Tdg
1	64945	Cldn12
1	18221	Nudc
1	319475	Zfp672
1	71667	0610007L01Rik
1	56461	Kcnip3
1	60527	Fads3
1	216963	Git1
1	107476	Acaca
1	NaN	MMT00057509
1	227622	BC029214
1	234407	Glt25d1
1	238317	C130039O16Rik
1	11767	Ap1m1
1	72033	Tsc22d2
1	24109	Ubl3

1	104718	Ttc7b
1	232333	Slc6a1
1	19353	Rac1
1	74315	Rnf145
1	19084	Prkar1a
1	67255	Zfp422
1	76976	2900062L11Rik
1	16924	Lnx1
1	NaN	MMT00017312
1	633752	EG633752
1	211556	BC002199
1	59014	Rrs1
1	108645	Mat2b
1	19822	Rnf4
1	619677	4930529C04Rik
1	53424	Tsnax
1	71989	Rpusd4
1	22214	Ube2h
1	20239	Atxn2
1	12261	C1qbp
1	21912	Tspan7
1	71941	Cars2
1	66089	Rmnd5b
1	71393	Kctd6
1	21787	Tfg
1	13244	Degs1
1	26444	Psma7
1	NaN	MMT00074658
1	623519	EG623519
1	NaN	MMT00047531
1	NaN	MMT00045906
1	NaN	MMT00048939
1	NaN	MMT00007455
1	NaN	MMT00082897
1	68011	Snrpg
1	69894	2010107G23Rik
1	252966	Cables2
1	259279	Tubgcp3
1	NaN	MMT00034706
1	NaN	MMT00078676
1	NaN	MMT00017112
1	19988	Rpl6
1	NaN	MMT00058413
1	442832	1600029O15Rik
1	NaN	MMT00015606
1	NaN	MMT00050545
1	NaN	MMT00059984
1	100087	Kti12
1	23980	Pebp1
1	23980	Pebp1
1	57752	Tacc2

1	54387	Mcm3ap
1	109934	Abr
1	72322	Xpo5
1	67160	Eef1g
1	67808	Tprgl
1	231510	Agpat9
1	15212	Hexb
1	23881	G3bp2
1	231050	Galnt11
1	56041	Uso1
1	319613	5730410E15Rik
1	223648	2410075B13Rik
1	74257	Tspan17
1	20019	Rpo1-4
1	19349	Rab7
1	209815	Tbc1d25
1	22340	Vegfb
1	53627	Porcn
1	227700	Sh3glb2
1	107831	Bai1
1	212986	Scfd2
1	212919	Kctd7
1	16423	Cd47
1	50762	Fbxo6
1	171207	Arhgap4
1	104759	Pld4
1	14751	Gpi1
1	67876	Coq10b
1	56310	Gps2
1	545389	Cep170
1	19155	Npepps
1	20602	Ncor2
1	67963	Npc2
1	54196	Pabpn1
1	11931	Atp1b1
1	75452	Ascc2
1	76954	St5
1	72088	Ush1c
1	12349	Car2
1	NaN	MMT00071316
1	270162	Elmod1
1	234779	Plcg2
1	14792	Mboat5
1	13449	Dok2
1	66320	Tmem208
1	27385	Magel2
1	71911	Bdh1
1	12348	Car11
1	70785	Dennd1c
1	19227	Pthlh
1	NaN	MMT00038317

1	57785	Rangrf
1	NaN	MMT00031928
1	14385	Slc37a4
1	71801	Plekhf2
1	66422	2410015N17Rik
1	67864	Yipf4
1	66096	0910001L09Rik
1	56705	Ranbp9
1	56351	Ptges3
1	18391	Oprs1
1	27362	Dnajb9
1	67468	Mmd
1	12181	Bop1
1	72123	2010109K11Rik
1	11502	Adam9
1	12894	Cpt1a
1	16590	Kit
1	227613	Tubb2c
1	72185	Dbndd1
1	110351	Rap1gap
1	232232	Hdac11
1	53598	Dctn3
1	76238	Grhpr
1	63828	Fn3k
1	17237	Mgrn1
1	66469	2810405K02Rik
1	230103	Npr2
1	621603	Aldh3b2
1	67666	Hapln3
1	66949	Trim59
1	75788	Smurf1
1	66642	Ctnnbl1
1	67337	Cstf1
1	68988	Prpf31
1	69870	Polr3gl
1	233274	Siglech
1	71336	Rbks
1	58246	Slc35b4
1	72278	Ccpg1
1	67122	Nrarp
1	68738	Acss1
1	16363	Irf2
1	13663	Ei24
1	20775	Sqle
1	78903	Wrnip1
1	73390	1700060H10Rik
1	231876	Lmtk2
1	109660	Ctrl
1	NaN	MMT00001400
1	12227	Btg2
1	67134	Nol5a

1	170718	Idh3b
1	76088	Dock8
1	14632	Gli1
1	213539	Bag2
1	77462	Tmem116
1	76737	Creld2
1	12879	Cys1
1	22240	Dpysl3
1	545339	MMT00017557
1	70902	Aytl1b
1	107885	Mthfs
1	14068	F7
1	15288	Hmbs
1	319953	Ttll1
1	232089	Rbed1
1	320078	Olfml2b
1	24030	Mrps12
1	228714	Csrp2bp
1	NaN	MMT00028462
1	69165	Cd209b
1	140484	Pofut1
1	20219	Apcs
1	76252	Atp6v0e2
1	67115	Rpl14
1	171405	AB056442
1	13821	Epb4.1l1
1	NaN	MMT00077995
1	16638	Klra7
1	230161	RP23-34B24.1
1	NaN	MMT00014895
1	18952	4-Sep
1	13162	Slc6a3
1	24117	Wif1
1	104174	Gldc
1	233065	Alkbh6
1	19683	Rdh16
1	17349	Mlf1
1	22138	Ttn
1	15377	Foxa3
1	12613	Cel
1	51811	Clec4f
1	110095	Pygl
1	74199	Vit
1	52685	Cd300lg
1	71276	Ccdc57
1	170765	Ripply3
1	268930	Pkmyt1
1	NaN	MMT00025372
1	21877	Tk1
1	16763	Lad1
1	20716	Serpina3n

1	17984	Ndn
1	12069	Bex2
1	117592	B3galt6
1	70082	Lysmd2
1	56488	Nxt1
1	16179	Irak1
1	66664	Tmem41a
1	14131	Fcgr3
1	116891	Derl2
1	14718	Got1
1	69471	2310005L22Rik
1	18102	Nme1
1	228869	Ncoa5
1	236732	Rbm10
1	64384	Sirt3
1	140499	Ube2j2
1	66506	Psmg3
1	383436	EG383436
1	22144	Tuba3a
1	22147	Tuba3b
1	NaN	MMT00007982
1	NaN	MMT00079618
1	226407	Rab3gap1
1	279067	MMT00046983
1	NaN	MMT00073590
1	19384	Ran
1	19317	Qk
1	378460	Pram1
1	21689	Tekt1
1	75429	3100002J23Rik
1	11514	Adcy8
1	70556	Slc25a33
1	232236	C130022K22Rik
1	109778	Blvra
1	13198	Ddit3
1	75710	Rbm12
1	211255	Kbtbd7
1	13388	Dll1
1	17118	Marcks
1	NaN	MMT00044046
1	NaN	MMT00002367
1	64451	Dip2a
1	227580	C1ql3
1	72480	Tspyl4
1	70611	Fbxo33
1	110391	Qdpr
1	52706	D10Ertd761e
1	56248	Ak3
1	218442	Serinc5
1	245884	AI507611
1	67283	Slc25a19

1	NaN	MMT00040001
1	NaN	MMT00042532
1	66404	2410001C21Rik
1	102339	Cog4
1	66989	Kctd20
1	14043	Ext2
1	20556	Slfn2
1	98878	Ehd4
1	71207	Nudt4
1	13433	Dnmt1
1	17131	Smad7
1	68041	Mid1ip1
1	55963	Slc1a4
1	213002	Ifitm6
1	226413	Lct
1	99151	Cercam
1	240396	Mex3c
1	67603	Dusp6
1	NaN	MMT00042618
1	30960	Vapa
1	29864	Rnf11
1	NaN	MMT00082345
1	628040	EG628040
1	57437	Golga7
1	15904	Id4
1	52392	D1Ertd622e
1	69568	Vkorc1l1
1	NaN	MMT00046482
1	19231	Ptma
1	18033	Nfkb1
1	50789	Fbxl3
1	69151	Lzic
1	16526	Kcnk2
1	68559	Pdrg1
1	11732	Ank
1	51792	Ppp2r1a
1	270035	Letm2
1	19645	Rb1
1	12861	Cox6a1
1	63985	Gmfb
1	19126	Prom1
1	60409	Trappc4
1	353208	2810021G02Rik
1	217473	Ankmy2
1	223917	Krt79
1	20717	Serpina3m
1	70231	Gorasp2
1	21823	Th
1	223254	Farp1
1	116972	2310047D13Rik
1	403174	A930005I04Rik

1	67704	1810037I17Rik
1	320118	Fbxl13
1	110084	Dnahc1
1	NaN	MMT00054932
1	NaN	MMT00070823
1	17763	Mtcp1
1	84505	Setdb1
1	12291	Cacna1g
1	11426	Macf1
1	67236	2810452K22Rik
1	12461	Cct2
1	53380	Psmd10
1	77781	Epm2aip1
1	54364	Rpp30
1	66310	Dpy30
1	66310	Dpy30
1	NaN	MMT00046285
1	94064	Mrpl27
1	622268	EG622268
1	NaN	MMT00058311
1	NaN	MMT00051556
1	NaN	MMT00033125
1	NaN	MMT00076184
1	NaN	MMT00001194
1	12238	Commd3
1	433546	MMT00011283
1	20220	Sap18
1	12850	Coq7
1	26458	Slc27a2
1	NaN	MMT00064324
1	56418	Ykt6
1	NaN	MMT00048362
1	67416	Armcx2
1	68981	Snrpa1
1	NaN	MMT00011146
1	19384	Ran
1	56434	Tspan3
1	269704	Zfp664
1	NaN	MMT00014274
1	57912	Cdc42se1
1	223752	Gramd4
1	71584	Gdpd2
1	56506	Cib2
1	56459	Sae1
1	NaN	MMT00059396
1	73738	Uchl5ip
1	73738	Uchl5ip
1	68099	6720467C03Rik
1	NaN	MMT00005424
1	NaN	MMT00077405
1	NaN	MMT00011281

1	28030	Gfm1
1	670717	MMT00038112
1	NaN	MMT00024508
1	NaN	MMT00079290
1	67877	Nat5
1	20853	Stau1
1	12632	Cfl2
1	22762	Zfpm2
1	NaN	MMT00060232
1	76178	6330578E17Rik
1	228942	Cbln4
1	13195	Ddc
1	626870	OTTMUSG00000005148
1	19217	Ptger2
1	68904	Abhd13
1	NaN	MMT00063585
1	20462	Sfrs10
1	70536	Qpct
1	319448	Fndc3a
1	77300	Raph1
1	208624	Alg3
1	56030	Tmem131
1	100044551	MMT00001823
1	21982	Tmem165
1	102334	Ankrd10
1	14700	Gng10
1	19983	Rpl5
1	81703	Jdp2
1	18604	Pdk2
1	NaN	MMT00046586
1	216705	Clint1
1	100737	Dcun1d4
1	66290	Atp6v1g1
1	NaN	MMT00051501
1	27058	Srp9
1	NaN	MMT00013262
1	22632	Yy1
1	109905	Rap1a
1	68044	Chac2
1	15452	Hprt1
1	18768	Pkib
1	72902	Spock3
1	71790	Anxa9
1	12876	Cpe
1	12876	Cpe
1	70238	Rnf168
1	13664	Eif1a
1	NaN	MMT00039793
1	235459	Gtf2a2
1	22782	Slc30a1
1	69109	1810009O10Rik

1	80907	Lactb
1	NaN	MMT00015399
1	NaN	MMT00024951
1	NaN	MMT00056938
1	NaN	MMT00046455
1	75572	Acyp2
1	NaN	MMT00068187
1	69961	2810432D09Rik
1	69178	Snx5
1	17060	Blnk
1	13589	Mapre1
1	80288	Bcl9l
1	NaN	MMT00063223
1	NaN	MMT00078948
1	66282	1810029B16Rik
1	54401	Ywhab
1	58203	Zbp1
1	NaN	MMT00078790
1	11758	Prdx6
1	23790	Coro1c
1	381379	Med19
1	14066	F3
1	17156	Man1a2
1	72007	Fndc3b
1	72421	Ttc30b
1	66357	2310008M10Rik
1	237052	Tceal1
1	69608	Sec24d
1	103768	Tubg2
1	18115	Nnt
1	57439	Tmem183a
1	319695	Ankar
1	320873	Cdh10
1	12565	Cdh9
1	105171	Arrdc3
1	67378	Bbs2
1	20333	Sec22b
1	52466	Slc46a1
1	210293	Dock10
1	12617	Cenpc1
1	20823	Ssb
1	20620	Plk2
1	74252	Armc1
1	52076	Tmem38b
1	22770	Zhx1
1	76804	Jmjd2c
1	338467	Morc3
1	70799	Cep192
1	18000	2-Sep
1	319263	Pcmtd1
1	219105	Zmym5

1	238447	Igh-2
1	76510	Trappc9
1	94229	Slc4a10
1	73728	Psd
1	20912	Stxbp3a
1	NaN	MMT00038995
1	56442	Serinc1
1	77032	2610029I01Rik
1	NaN	MMT00041407
1	78913	Zfp294
1	77591	Ddx10
1	229227	4932438A13Rik
1	117198	Ivns1abp
1	77048	Ccdc41
1	78248	Armcx1
1	54375	Azin1
1	99730	Taf13
1	12794	Cnih2
1	29805	Znhit2
1	70472	Atad2
1	170753	Zfp704
1	223435	Trio
1	26422	Nbea
1	76483	Lmf1
1	230157	Tmeff1
1	22194	Ube2e1
1	22194	Ube2e1
1	NaN	MMT00004881
1	NaN	MMT00052614
1	217869	Eif5
1	NaN	MMT00081393
1	67019	Actr6
1	23950	Dnajb6
1	13690	Eif4g2
1	676923	MMT00040704
1	70078	Nol7
1	224585	Zfp160
1	23971	Papss1
1	106073	D15Mgi27
1	140482	Zfp358
1	19302	Pex2
1	59032	Ppp2r3c
1	67003	Uqcrc2
1	19744	Rheb
1	620986	EG620986
1	NaN	MMT00011278
1	56426	Pdcd10
1	NaN	MMT00027514
1	30795	Fkbp3
1	70747	Tspan2
1	NaN	MMT00063982

1	14823	Grm8
1	67958	2610101N10Rik
1	235072	7-Sep
1	NaN	MMT00063687
1	NaN	MMT00080648
1	70834	Spag9
1	71982	Snx10
1	59079	Erbb2ip
1	70047	Trnt1
1	210973	Kbtbd2
1	NaN	MMT00007741
1	NaN	MMT00077590
1	67939	2010316F05Rik
1	NaN	MMT00018157
1	68010	Bambi
1	225896	D19Ertd721e
1	226122	Ubtd1
1	66725	Lrrk2
1	192657	Ell2
1	56195	Ptbp2
1	54607	Socs6
1	69048	Slc30a5
1	68352	0610012D14Rik
1	18049	Ngf
1	57896	Krcc1
1	329877	Dennd4c
1	13039	Ctsl
1	16653	Kras
1	224022	Slc7a4
1	15982	Ifrd1
1	234915	AK129341
1	18080	Nin
1	226747	Ahctf1
1	213819	Casd1
1	72139	2610044O15Rik
1	NaN	MMT00020969
1	624862	EG624862
1	12176	Bnip3
1	107769	Tm6sf1
1	17954	Nap1l2
1	19352	Rabggtb
1	75812	Tasp1
1	329650	Med12l
1	14658	Glrb
1	NaN	MMT00003599
1	245688	Rbbp7
1	NaN	MMT00060167
1	264064	Cdk8
1	624446	EG624446
1	17973	Nck1
1	18949	Pnn

1	22612	Yes1
1	NaN	MMT00038159
1	72999	Insig2
1	320940	Atp11c
1	20280	Scp2
1	69028	Mitd1
1	72183	Snx6
1	16452	Jak2
1	79264	Krit1
1	12301	Cacybp
1	21353	Tank
1	109019	Obfc2a
1	321000	4933421E11Rik
1	382252	A830080D01Rik
1	237542	Osbpl8
1	78521	B230219D22Rik
1	72098	Tmem68
1	17184	Matr3
1	67089	Psmc6
1	68845	Pih1d1
1	74020	Cpne4
1	242521	Klhl9
1	76073	Pcgf5
1	110789	Gpr98
1	226646	Ndufs2
1	12007	Azgp1
1	54608	Abhd2
1	272428	Acsm5
1	68338	Golt1a
1	13108	Cyp2g1
1	226143	Cyp2c44
1	208098	Panx3
1	72330	Kbtbd5
1	26557	Homer2
1	330460	BC022651
1	71839	Osgin1
1	109791	Clps
1	71754	Cyp2d40
1	13117	Cyp4a10
1	12279	C9
1	69085	Zcchc9
1	624219	EG624219
1	80287	Apobec3
1	13012	Cst8
1	170728	Rtn4ip1
1	68034	2900009I07Rik
1	78825	5830417C01Rik
1	NaN	MMT00057138
1	107589	Mylk
1	433424	OTTMUSG00000012358
1	84682	Cox4i2

1	71846	Syce2
1	17995	Ndufv1
1	17922	Myo7b
1	118449	Synpo2
1	54419	Cldn6
1	NaN	MMT00081793
1	227743	Mapkap1
1	102595	Plekho2
1	258999	Olfr178
1	545370	Hmcn1
1	65956	Ccl21c
1	258593	Olfr700
1	19650	Rbl1
1	NaN	MMT00014463
1	56358	Copz2
1	13611	S1pr4
1	109225	Ms4a7
1	74043	Pex26
1	57890	Il17re
1	NaN	MMT00012844
1	108114	Slc22a7
1	22224	Usp10
1	629378	Dact3
1	26940	Ecsit
1	211548	Nomo1
1	56876	Nelf
1	435684	Shf
1	66077	Aurkaip1
1	67437	Ssr3
1	668450	EG668450
1	66397	Sar1b
1	NaN	MMT00051866
1	57783	Tnip1
1	235606	Apeh
1	50753	Fbxo8
1	192652	Wdr81
1	67843	Slc35a4
1	NaN	MMT00066182
1	544963	Iqgap2
1	14062	F2r
1	242050	Igsf10
1	76501	Commd9
1	78240	Cst11
1	68617	1110012J17Rik
1	230661	Tesk2
1	231093	Agbl5
1	18588	Pde6g
1	73730	Lce1l
1	70382	Kctd2
1	76187	Adhfe1
1	59007	Ngly1

1	100177	Zmym6
1	23821	Bace1
1	58245	Gpr180
1	227522	Rpp38
1	16669	Krt19
1	66817	Tmem170
1	12833	Col6a1
1	74100	MMT00001343
1	66531	2310061C15Rik
1	68603	Pmvk
1	233812	BC030336
1	93885	Pcdhb14
1	20439	Siah2
1	69379	C8g
1	71838	Phf7
1	110006	Gusb
1	230101	Gba2
1	108705	Pttg1ip
1	NaN	MMT00011231
1	233744	Spon1
1	223665	C030006K11Rik
1	73652	2210408F21Rik
1	76890	Memo1
1	NaN	MMT00051740
1	NaN	MMT00071327
1	NaN	MMT00067648
1	66945	Sdha
1	78523	Mrpl9
1	NaN	MMT00042876
1	58202	Cobra1
1	18541	Pcnt
1	22217	Usp12
1	NaN	MMT00007006
1	NaN	MMT00030019
1	76273	Ndfip2
1	69536	Hemk1
1	NaN	MMT00002799
1	66588	Cmpk1
1	26611	Rcn2
1	53328	Pgrmc1
1	53382	Txnl1
1	100678	Psph
1	319322	Sf3b2
1	NaN	MMT00068885
1	77569	Limch1
1	70478	Mipep
1	19182	Psmc3
1	54380	Smarcal1
1	20224	Sar1a
1	52250	Reep1
1	226971	Plekhb2

1	230075	Ndufb6
1	NaN	MMT00020987
1	18719	Pip5k1b
1	627022	EG627022
1	NaN	MMT00056589
1	NaN	MMT00059805
1	NaN	MMT00004769
1	NaN	MMT00056634
1	667832	MMT00021210
1	17846	Commd1
1	75734	5230400G24Rik
1	74776	Ppa2
1	67443	Map1lc3b
1	68240	Rpa3
1	NaN	MMT00020332
1	NaN	MMT00034261
1	NaN	MMT00044478
1	20005	Rpl9
1	NaN	MMT00046581
1	NaN	MMT00048392
1	NaN	MMT00012906
1	625690	EG625690
1	NaN	MMT00059232
1	NaN	MMT00069919
1	NaN	MMT00002494
1	NaN	MMT00043723
1	NaN	MMT00017766
1	668699	MMT00070312
1	NaN	MMT00079369
1	17449	Mdh1
1	NaN	MMT00040108
1	NaN	MMT00048286
1	NaN	MMT00011814
1	NaN	MMT00036520
1	NaN	MMT00042303
1	NaN	MMT00069486
1	67157	2610301B20Rik
1	229746	EG229746
1	57344	As3mt
1	22021	Tpst1
1	20810	Srm
1	73723	Sh3bgrl3
1	NaN	MMT00001732
1	259086	Olfr609
1	225362	Reep2
1	73737	1110008P14Rik
1	20462	Sfrs10
1	64602	Ireb2
1	NaN	MMT00006294
1	68050	6330407G11Rik
1	12552	Cdh11

1	269424	Phf17
1	NaN	MMT00046996
1	76892	Rnft1
1	59048	C1galt1c1
1	66631	Hiatl1
1	68861	1190002N15Rik
1	68187	4921533L14Rik
1	93759	Sirt1
1	66684	Tceal8
1	NaN	MMT00045897
1	22235	Ugdh
1	240283	Dmxl1
1	225028	Map4k3
1	12675	Chuk
1	NaN	MMT00029171
1	12626	Cetn3
1	52184	Odf2l
1	68146	Arl13b
1	110842	Etfa
1	12785	Cnbp
1	74117	Actr3
1	210933	Bai3
1	66092	Ghitm
1	66385	Ppp1r7
1	83962	Btbd1
1	98221	Eif3m
1	229906	Gtf2b
1	56307	Metap2
1	66213	Med7
1	321022	Cdv3
1	53605	Nap1l1
1	15247	Hiat1
1	23989	Med24
1	70572	Ipo5
1	26922	Mecr
1	78618	Centb2
1	16979	Lrrn1
1	98758	Hnrnpf
1	71458	Bcor
1	NaN	MMT00041243
1	57321	Terf2ip
1	NaN	MMT00038380
1	65246	Xpo7
1	67028	2610002M06Rik
1	69981	Tmem30a
1	14265	Fmr1
1	217664	Mgat2
1	70155	Ogfrl1
1	69535	2310004N24Rik
1	29858	Pmm1
1	217038	Mrm1

1	12265	Ciita
1	22225	Usp5
1	59042	Cope
1	103733	Tubg1
1	79043	Spsb3
1	26425	Nubp1
1	NaN	MMT00028111
1	19656	Rbmxrt
1	19656	Rbmxrt
1	227835	Gtdc1
1	NaN	MMT00010554
1	69923	Agk
1	227197	Ndufs1
1	108664	Atp6v1h
1	56278	Gkap1
1	56278	Gkap1
1	209446	Tcfe3
1	22221	Ubp1
1	66204	Acyp1
1	NaN	MMT00081492
1	68777	Tmem53
1	654824	Ankrd37
1	99480	Dnttip2
1	24067	Srp54a
1	56771	Med20
1	76687	Spcs3
1	71767	Tysnd1
1	71966	Nkiras2
1	53317	Plrg1
1	12469	Cct8
1	67973	Mphosph10
1	217980	Larp5
1	214931	Fbxl16
1	83486	Rbm5
1	69804	Tmem147
1	70823	Hmgb2l1
1	56210	Rev1
1	56200	Ddx21
1	338337	Cog3
1	74741	5730419I09Rik
1	NaN	MMT00052332
1	210529	G430022H21Rik
1	50497	Hspa14
1	14862	Gstm1
1	74213	Rbm26
1	59290	Gpa33
1	50926	Hnrpdl
1	213811	BC002059
1	NaN	MMT00076058
1	66625	Sfrs18
1	67759	5033414D02Rik

1	67832	Bxdc2
1	67492	Anubl1
1	494468	Armcx5
1	NaN	MMT00026286
1	68184	Denr
1	234865	Nup133
1	68083	Pak1ip1
1	21848	Trim24
1	14017	Evi2a
1	107652	Uap1
1	13072	Cyp11b2
1	18830	Pltp
1	101565	6330503K22Rik
1	12978	Csf1r
1	216618	Ccdc104
1	140483	Hnmt
1	19139	Prps1
1	14545	Gdap1
1	94062	Mrpl3
1	15525	Hspa4
1	320720	Fastkd1
1	19025	Ctsa
1	66711	Sbds
1	NaN	MMT00037611
1	83433	Trem2
1	19682	Rdh5
1	98845	Eps8l2
1	225341	Lims2
1	55978	Ift20
1	11843	Arf4
1	66706	4733401H18Rik
1	76936	Hnrnpm
1	12064	Bdnf
1	18706	Pik3ca
1	68722	1110029L17Rik
1	NaN	MMT00023332
1	59010	Sqrdl
1	27280	Phlda3
1	26568	Slc27a3
1	66349	Atp5sl
1	229715	Amigo1
1	71687	Tmem25
1	13384	Mpp3
1	66098	Chchd6
1	NaN	MMT00038457
1	11692	Gfer
1	234825	Klhdc4
1	66268	Pigyl
1	66449	MMT00023224
1	66449	MMT00011706
1	71994	Cnn3

1	58809	Rnase4
1	13032	Ctsc
1	68588	Cthrc1
1	27226	Pla2g7
1	22223	Uchl1
1	93886	Pcdhb15
1	11676	Aldoc
1	66401	Nudt2
1	71853	Pdia6
1	NaN	MMT00008784
1	NaN	MMT00051613
1	641192	MMT00077535
1	68742	1110032O16Rik
1	20909	Stx4a
1	NaN	MMT00078932
1	NaN	MMT00047810
1	NaN	MMT00046201
1	15356	Hmgcl
1	80743	Vps16
1	407785	Ndufs6
1	108156	Mthfd1
1	68642	2810441K11Rik
1	56191	Tro
1	27681	Snf8
1	19300	Abcd4
1	59046	Arpp19
1	59046	Arpp19
1	11655	Alas1
1	26416	Mapk14
1	72726	Tbcc
1	67177	Cdt1
1	NaN	MMT00076151
1	20018	Rpo1-3
1	72832	Crtac1
1	14756	Gpld1
1	229487	Pet112l
1	50769	Atp8a2
1	69137	2200002K05Rik
1	107829	Thoc5
1	67046	Tbc1d7
1	27267	Cars
1	66293	1810032O08Rik
1	53817	Bat1a
1	NaN	MMT00035426
1	14057	Sfxn1
1	NaN	MMT00058026
1	15211	Hexa
1	66111	Tmed3
1	67367	1810007M14Rik
1	235469	Suhw4
1	218503	Fcho2

1	17344	Pias2
1	245050	C730027P07Rik
1	14800	Gria2
1	97165	Hmgb2
1	69507	1700030G06Rik
1	74443	4933406E20Rik
1	70349	Copb1
1	69226	Snx24
1	140742	Sesn1
1	71837	1700003E16Rik
1	269878	Megf8
1	14670	Gna-rs1
1	217335	Fbf1
1	213109	Phf3
1	11702	Amd1
1	NaN	MMT00063804
1	66680	3230401D17Rik
1	66680	3230401D17Rik
1	231712	Trafd1
1	76073	Pcgf5
1	NaN	MMT00008417
1	53872	Caprin1
1	56369	Apip
1	66050	0610009B22Rik
1	69482	Nup35
1	52575	Rg9mtd1
1	544971	Bdp1
1	19165	Psen2
1	545700	Vmn2r-ps14
1	211253	Mtrf1
1	23924	Katna1
1	19386	Ranbp2
1	73668	Ttc21b
1	16588	Kin
1	76123	Gpsm2
1	76044	Ncapg2
1	239122	Setdb2
1	109151	Chd9
1	13405	Dmd
1	107227	Macrod1
1	107767	Scamp1
1	228730	Gm114
1	56532	Ripk3
1	68815	Btbd10
1	68337	Crip2
1	66940	Shisa5
1	207214	Larp4
1	93871	Brwd1
1	74533	Gzf1
1	192166	Sardh
1	330192	Vps37b

1	381644	Cep135
1	17918	Myo5a
1	26433	Plod3
1	72930	Ppp2r2b
1	20747	Spop
1	235293	Sc5d
1	19361	Rad51
1	15587	Hyal2
1	67239	Bxdc1
1	13667	Eif2b4
1	17217	Mcm4
1	80752	BC004044
1	98970	Fibcd1
1	385454	EG385454
1	13197	Gadd45a
1	225791	Zadh2
1	319601	Zfp653
1	107173	Gpr137
1	229512	Smg5
1	71723	Dhx34
1	16399	Itga2b
1	17912	Myo1b
1	67533	Ppfibp1
1	71929	Tmem123
1	20501	Slc16a1
1	192663	Abcg4
1	232879	Zbtb45
1	74562	Fer1l4
1	69573	2310016C08Rik
1	67379	Dedd2
1	230868	Igsf21
1	66156	Anapc11
1	71146	4933417O08Rik
1	69065	Chac1
1	20262	Stmn3
1	55992	Trim3
1	74097	Pop7
1	98238	Lrrc59
1	67723	4932415G12Rik
1	66411	Tbcb
1	18100	Mrpl40
1	67334	1700054O13Rik
1	195733	Grhl1
1	59092	Pcbp4
1	12778	Cxcr7
1	407790	Ndufa4l2
1	74498	Gorasp1
1	18040	Nefm
1	54138	Atxn10
1	67784	Plxnd1
1	27377	Yme1l1

1	66687	Tbc1d15
1	50997	Mpp2
1	13637	Efna2
1	17975	Ncl
1	72759	Tmem135
1	59027	Nampt
1	NaN	MMT00069487
1	NaN	MMT00079689
1	NaN	MMT00054557
1	NaN	MMT00078030
1	13121	Cyp51
1	19046	Ppp1cb
1	NaN	MMT00050725
1	12314	Calm2
1	NaN	MMT00019522
1	NaN	MMT00009680
1	NaN	MMT00052151
1	226026	Smc5
1	319901	Dsel
1	75964	D030074E01Rik
1	52665	Echdc1
1	14733	Gpc1
1	216558	Ugp2
1	53330	Vamp4
1	83493	Sacm1l
1	629364	MMT00061218
1	226414	Dars
1	17126	Smad2
1	625796	EG625796
1	67204	Eif2s2
1	353310	Zfp703
1	56297	Arl6
1	667795	EG667795
1	104721	Ddx1
1	26965	Cul1
1	NaN	MMT00017521
1	NaN	MMT00073158
1	66355	Gmpr
1	59031	Chst12
1	53607	Snrpa
1	NaN	MMT00014970
1	12033	Bcap29
1	13612	Edil3
1	NaN	MMT00048420
1	14791	Emg1
1	240025	Dact2
1	269033	4930503L19Rik
1	320184	Lrrc58
1	100047309	MMT00074326
1	19762	Rit2
1	232906	Grlf1

1	NaN	MMT00024172
1	109241	Mbd5
1	NaN	MMT00001956
1	NaN	MMT00025562
1	269105	EG269105
1	NaN	MMT00036391
1	67488	Calcoco1
1	30056	Timm9
1	55951	Brp44l
1	68263	Pdhb
1	NaN	MMT00080137
1	NaN	MMT00040104
1	241308	Ralgps1
1	NaN	MMT00011132
1	18970	Polb
1	56055	Gtpbp2
1	NaN	MMT00046046
1	NaN	MMT00061243
1	NaN	MMT00004656
1	NaN	MMT00036668
1	56209	Gde1
1	68879	Prpf6
1	67513	2610002J02Rik
1	66637	5730449L18Rik
1	66637	5730449L18Rik
1	675689	MMT00041953
1	56724	Cript
1	107047	Psmg2
1	NaN	MMT00077382
1	675985	MMT00081973
1	NaN	MMT00080853
1	56294	Ptpn9
1	18950	Pnp1
1	NaN	MMT00015578
1	NaN	MMT00075644
1	101437	Dhx32
1	67619	Nob1
1	21672	Prdx2
1	16976	Lrpap1
1	210148	Slc30a6
1	53619	Blcap
1	214895	Lman2l
1	58521	Eid1
1	19679	Pitpnm2
1	434339	EG434339
1	11416	Slc33a1
1	104662	Tsr1
1	24070	Mpdu1
1	668838	EG668838
1	23873	Faim
1	NaN	MMT00074659

1	67676	Rpp21
1	76561	Snx7
1	72129	Pex13
1	433862	EG433862
1	16169	Il15ra
1	11532	Adh5
1	78309	Parc
1	64293	Stk32b
1	319468	Ppm1h
1	78372	3300001G02Rik
1	232969	Zfp428
1	94181	Nans
1	56615	Mgst1
1	14600	Ghr
1	75974	Dock11
1	70575	Gfod2
1	20196	S100a13
1	11484	Aspa
1	216274	Cep290
1	68117	Apool
1	74552	Npal3
1	11806	Apoa1
1	78124	4930458L03Rik
1	65972	Ifi30
1	20971	Sdc4
1	16516	Kcnj15
1	17318	Mid1
1	20293	Ccl12
1	74481	Batf2
1	11921	Atoh1
1	18611	Pea15a
1	55944	Eif3d
1	15496	Hsd3b5
1	12768	Ccr1
1	NaN	MMT00049098
1	636752	EG636752
1	NaN	MMT00006438
1	83702	Akr1c6
1	242594	1700024P16Rik
1	624866	EG624866
1	80985	Trim44
1	27007	Klrk1
1	75820	Wdr64
1	75497	1700008G05Rik
1	11727	Ang
1	277753	Cyp4a12a
1	15109	Hal
1	69123	1810022C23Rik
1	74568	Mlkl
1	11500	Adam7
1	11846	Arg1

1	329575	RP23-275N21.2
1	227377	Farp2
1	73603	1700126L10Rik
1	243822	4930401F20Rik
1	217365	Nploc4
1	28250	Slco1a4
1	330962	MMT00003408
1	73132	Slc25a16
1	15273	Hivep2
1	29815	Bcar3
1	244579	Tox3
1	71721	1200015N20Rik
1	241201	Cdh7
1	59029	Psmd14
1	67005	Polr3k
1	66979	Pole4
1	93888	Pcdhb17
1	74239	Iqce
1	68646	1110020G09Rik
1	78752	Csgalnact2
1	74670	4930432O21Rik
1	NaN	MMT00071421
1	20617	Snca
1	214627	Papd5
1	13178	Dck
1	73130	Tmed5
1	22289	Utx
1	231051	Mll3
1	69190	Dym
1	219135	Mtmr6
1	12387	Ctnnb1
1	107999	Gtpbp6
1	69876	Thap3
1	18036	Nfkbib
1	68867	Rnf122
1	NaN	MMT00044154
1	NaN	MMT00008110
1	NaN	MMT00078167
1	382421	EG382421
1	100038514	OTTMUSG00000004551
1	237553	Trhde
1	140570	Plxnb2
1	19039	Lgals3bp
1	269799	Clec4a1
1	NaN	MMT00058786
1	211389	Suox
1	19294	Pvrl2
1	80708	Pacsin3
1	73893	Tmem202
1	24057	Sh3yl1
1	52004	Cdk2ap2

1	12764	Cmas
1	230779	Serinc2
1	225870	Rin1
1	57816	Tesc
1	17306	Sypl2
1	228550	Itpka
1	68487	Tmem140
1	231147	Sh3tc1
1	15896	Icam2
1	231633	Tmem119
1	227545	5430407P10Rik
1	12259	C1qa
1	15353	Hmg20b
1	69159	Rhebl1
1	NaN	MMT00049207
1	14976	H2-Ke2
1	11966	Atp6v1b2
1	66958	Txndc14
1	12729	Clns1a
1	102462	Imp3
1	20464	Sim1
1	70385	Ccdc99
1	14587	Gfra3
1	18606	Enpp2
1	16939	Lor
1	20349	Sema3e
1	229699	Slc16a4
1	66183	1110032A04Rik
1	20112	Rps6ka2
1	NaN	MMT00047910
1	67184	Ndufa13
1	223701	Mkl1
1	20024	Sub1
1	277468	Slc39a12
1	12848	Cops2
1	13360	Dhcr7
1	77604	C430048L16Rik
1	432812	BC052688
1	103098	Slc6a15
1	22446	Xlr3c
1	75458	Cklf
1	66736	Ttc35
1	74386	Rmi1
1	17219	Mcm6
1	71860	Wdr16
1	18709	Pik3r2
1	21961	Tns1
1	217140	Scrn2
1	16331	Inpp5d
1	104445	Cdc42ep1
1	67393	Cxxc5

1	67860	S100a16
1	19735	Rgs2
1	102693	Phldb1
1	12421	Rb1cc1
1	67241	Smc6
1	24015	Abce1
1	67456	Ergic2
1	14479	Usp15
1	64213	St7
1	67198	2810022L02Rik
1	106581	Itfg3
1	68979	Nol11
1	215378	B830045N13Rik
1	66240	Kcne1l
1	71904	Paqr7
1	73847	5430432M24Rik
1	23894	Gtf2h2
1	11546	Parp2
1	59002	Wdr8
1	214855	Arid5a
1	66970	Ssbp2
1	223864	Rapgef3
1	22344	Vezf1
1	NaN	MMT00057242
1	28040	D6Wsu163e
1	66151	Prr13
1	67063	2810432L12Rik
1	71743	Coasy
1	52521	Zfp622
1	67397	Erp29
1	64652	Nisch
1	227298	BC038286
1	66085	Eif3f
1	11769	Ap1s1
1	667804	MMT00076032
1	70024	Mcm10
1	13088	Cyp2b10
1	236790	Ddx26b
1	17086	Ncr1
1	235047	Zfp809
1	16414	Itgb2
1	18559	Pctp
1	223433	BC052328
1	217066	OTTMUSG00000001305
1	56554	Raet1d
1	NaN	MMT00050414
1	12908	Crat
1	545279	Ms4a15
1	58170	Accn5
1	NaN	MMT00053630
1	66471	Anp32e

1	338523	Jhdm1d
1	26885	Casp8ap2
1	110960	Tars
1	105203	BC016423
1	103172	MMT00073283
1	19018	Scand1
1	319740	Zfyve27
1	110198	Akr7a5
1	18557	Pctk3
1	20167	Rtn2
1	22764	Zfx
1	30951	Cbx8
1	237412	EG237412
1	11468	Actg2
1	108161	D0H6S2654E
1	116701	Fgfrl1
1	65221	Slc15a3
1	13555	E2f1
1	18984	Por
1	104871	Spata7
1	18004	Nek1
1	71703	Armcx3
1	237175	Gpr64
1	73288	Ccdc132
1	66724	Map3k7ip3
1	20713	Serpini1
1	12015	Bad
1	216965	Taok1
1	67131	Acbd4
1	212326	BC035537
1	110821	Pcca
1	67168	P2ry5
1	74318	Hopx
1	619605	Zcchc17
1	50875	Tmod3
1	228543	Rhov
1	13809	Enpep
1	20743	Spnb3
1	277973	Slc9a5
1	12368	Casp6
1	15361	Hmga1
1	15361	Hmga1
1	14086	Fscn1
1	382014	Tmem16h
1	16511	Kcnh2
1	52055	Rab11fip5
1	76454	Fbxo31
1	632223	MMT00011796
1	380711	Garnl4
1	73046	Glrx5
1	67851	1700021F05Rik

1	100042265	MMT00012636
1	56295	Higd1a
1	67044	Higd2a
1	12862	Cox6a2
1	15165	Hcn1
1	67392	4833420G17Rik
1	271970	Arsj
1	19256	Ptpn20
1	68149	Otub2
1	98415	Nucks1
1	63955	Cables1
1	219134	Shisa2
1	20922	Supt4h1
1	68927	Ptcd2
1	NaN	MMT00006420
1	12192	Zfp36l1
1	14712	Gnpat
1	28019	Ing4
1	77134	Hnrnpa0
1	218203	Mylip
1	80912	Pum1
1	17766	Nudt1
1	212439	AA986860
1	NaN	MMT00001620
1	108075	Ltbp4
1	107986	Ddb2
1	68275	Rpa1
1	100342	4732473B16Rik
1	226744	9630058J23Rik
1	26921	Map4k4
1	NaN	MMT00077153
1	NaN	MMT00065667
1	244668	Sipa1l2
1	68507	Ppfia4
1	22142	Tuba1a
1	NaN	MMT00060868
1	12499	Entpd5
1	NaN	MMT00055891
1	NaN	MMT00008758
1	321018	Serpina4-ps1
1	12443	Ccnd1
1	27368	Tbl2
1	18627	Per2
1	11650	Akp5
1	69274	Ctdspl
1	NaN	MMT00021352
1	232223	Txnrd3
1	234199	Fgl1
1	76411	1700019E19Rik
1	NaN	MMT00052300
1	71768	Vwce

1	16205	Gimap1
1	56085	Ubqln1
1	NaN	MMT00050193
1	20520	Slc22a5
1	26424	Nr5a2
1	12611	Cebpg
1	15902	Id2
1	14555	Gpd1
1	66673	Sorcs3
1	224703	2-Mar
1	67956	Setd8
1	22138	Ttn
1	228357	Lrp4
1	223723	Ttll12
1	71529	9030409G11Rik
1	NaN	MMT00042479
1	381204	Naaladl1
1	435977	EG435977
1	NaN	MMT00064565
1	54710	Hs3st3b1
1	53901	Rcan2
1	53311	Mybph
1	20972	Syngr1
1	107993	Bfsp2
1	NaN	MMT00033856
1	12808	Cobl
1	56221	Ccl24
1	102632	Acad11
1	67115	Rpl14
1	11812	Apoc1
1	76947	2310030N02Rik
1	65099	Irak1bp1
1	70821	4921507P07Rik
1	66302	2410005O16Rik
1	268470	Ube2z
1	108767	Pnrc1
1	12398	Cbfa2t3
1	72341	Tmem103
1	68556	Uckl1
1	69871	2010007H12Rik
1	18991	Pou3f1
1	66999	Med28
1	67528	Nudt7
1	18167	Npy2r
1	17216	Mcm2
1	280645	B3gat2
1	675135	MMT00050019
1	11608	Agtr1b
1	58988	Rps6kb2
1	NaN	MMT00034311
1	NaN	MMT00013041

1	66950	Tmem206
1	NaN	MMT00037578
1	243659	Styk1
1	378462	Morn2
1	432745	4930447K03Rik
1	19119	Prm2
1	15446	Hpgd
1	207175	Cetn4
1	71881	2310001A20Rik
1	12296	Cacnb2
1	15930	Indo
1	544998	B930046C15Rik
1	54326	Elovl2
1	78321	Ankrd23
1	56196	Ttrap
1	101148	B630005N14Rik
1	14601	Ghrh
1	14275	Folr1
1	17957	Napb
1	242748	Ptchd2
1	380694	Ccnjl
1	12827	Col4a2
1	22158	Tulp3
1	70240	Ufsp1
1	21924	Tnnc1
1	269016	Sh3rf2
1	641340	Nrbf2
1	72404	Wdr44
1	67245	Peli1
1	12452	Ccng2
1	17311	Kitl
1	16508	Kcnd2
1	225651	Mppe1
1	52323	Klhl7
1	73173	Pcdh18
1	76219	6530401D17Rik
1	235682	Zfp445
1	19070	Mobkl3
1	66271	Tmem126a
1	108900	2700049P18Rik
1	68736	1110034B05Rik
1	19283	Ptprz1
1	170719	Oxr1
1	72140	Ccdc123
1	75871	Zfp821
1	70435	2610204M08Rik
1	214597	Sidt2
1	64540	Tspan4
1	378702	Serf2
1	378702	Serf2
1	17153	Mal

1	74563	Rasgef1c
1	380787	A230065H16Rik
1	52690	Setd3
1	93692	Glrx
1	14660	Gls
1	330149	Hfm1
1	66061	Tctex1d2
1	16210	Impact
1	24083	Gtlf3b
1	70591	5730455P16Rik
1	NaN	MMT00033070
1	66935	1700023B02Rik
1	71030	4933403O08Rik
1	57432	Zc3h8
1	217615	Ctage5
1	14758	Gpm6b
1	213056	D1Ertd53e
1	330731	EG330731
1	227059	Slc39a10
1	433702	AU014645
1	56048	Lgals8
1	229681	St7l
1	226144	Erlin1
1	56404	Trip4
1	76784	Mtif2
1	74096	Hvcn1
1	13057	Cyba
1	56222	Cited4
1	80915	Dusp12
1	22590	Xpa
1	NaN	MMT00011155
1	67815	Sec14l2
1	56282	Mrpl12
1	84036	Kcnn1
1	19171	Psmb10
1	68567	Cgref1
1	69101	Ydjc
1	66193	1110049F12Rik
1	72899	Macrod2
1	170938	Zfp617
1	67053	Rpp14
1	67095	Trak1
1	84095	Pi4k2a
1	20788	Srebf2
1	105835	Sgsm3
1	77794	Adamtsl2
1	67830	Rer1
1	26903	Dysf
1	18087	Nktr
1	231670	Fbxo21
1	107094	Rrp12

1	108707	1810008A18Rik
1	217119	Xylt2
1	22027	Hsp90b1
1	14251	Flot1
1	55927	Hes6
1	231842	Amz1
1	81500	Sil1
1	22670	Trim26
1	171212	Galnt10
1	80280	Cdk5rap3
1	66513	Map3k7ip1
1	242642	Hpdl
1	14228	Fkbp4
1	105837	Mtbp
1	240186	Zfp438
1	231123	BC023882
1	75430	3200002M19Rik
1	74772	Atp13a2
1	20587	Smarcb1
1	70616	Sf4
1	80720	Pbx4
1	231134	Dok7
1	269585	Zscan20
1	268759	9930012K11Rik
1	140917	Dclre1b
1	18387	Oprk1
1	224860	Plcl2
1	71522	Ggt6
1	NaN	MMT00078937
1	108670	Epsti1
1	257633	Acsf3
1	382038	AK122209
1	23996	Psmc4
1	12824	Col2a1
1	13001	Csnk2b
1	73750	Whrn
1	233489	Picalm
1	24071	Synj2bp
1	74019	Traf3ip1
1	12313	Calm1
1	NaN	MMT00011022
1	68628	Fbxw9
1	66968	2310076L09Rik
1	74451	Pgs1
1	71713	Cdc40
1	70530	Lrfn2
1	380752	Tssc1
1	13972	Gnb1l
1	140559	Igsf8
1	104886	Rab15
1	67544	4932442K08Rik

1	71918	2310047A01Rik
1	110637	Grik4
1	74334	Ranbp10
1	68744	Zfp740
1	16907	Lmnb2
1	13645	Egf
1	64929	Scel
1	319433	E130113E03Rik
1	211949	Spsb4
1	68743	Anln
1	75563	Dnali1
1	240641	Kif20b
1	108989	Tpr
1	76131	Depdc1a
1	66473	Ctrb1
1	16391	Irf9
1	20822	Trove2
1	22601	Yap1
1	56193	Plek
1	106369	Ypel1
1	NaN	MMT00057557
1	51795	Srpx
1	70527	Stambp
1	19240	Tmsb10
1	68731	1110032A13Rik
1	229055	Zbtb10
1	93892	Pcdhb21
1	NaN	MMT00001646
1	NaN	MMT00053547
1	NaN	MMT00069653
1	666581	RP23-162H3.1
1	231532	Arhgap24
1	68092	Ncbp2
1	11735	Ank3
1	20901	Strap
1	217695	Zfyve1
1	NaN	MMT00044927
1	66353	2310007A19Rik
1	70772	MMT00075624
1	231003	Klhl17
1	14347	Fut7
1	329910	Acot11
1	241274	Pnpla7
1	240753	Plekha6
1	103712	6330403K07Rik
1	66425	Pcp4l1
1	76508	2210015D19Rik
1	29812	Ndrg3
1	12354	Car7
1	224440	Setd4
1	56504	Srpk3

1	545539	OTTMUSG00000021274
1	246277	Csad
1	17993	Ndufs4
1	68980	Wdr53
1	75607	Wnk2
1	11773	Ap2m1
1	68251	5430437P03Rik
1	328381	Sh2d4b
1	29806	Limd1
1	52432	Ppp2r2d
1	66943	Pqlc1
1	320661	D5Ertd579e
1	72654	Ccdc12
1	209011	Sirt7
1	76457	Ccdc134
1	NaN	MMT00039978
1	19267	Ptpre
1	20382	Sfrs2
1	18569	Pdcd4
1	664862	Gpr137b-ps
1	52857	Gramd1a
1	68801	Elovl5
1	97287	Mtmr14
1	50493	Txnrd1
1	74237	Tubgcp2
1	80750	N4bp1
1	235497	Leo1
1	19243	Ptp4a1
1	381629	0610007C21Rik
1	76740	Efr3a
1	103967	Dnm3
1	68671	Pcyt2
1	103742	1810046J19Rik
1	54153	Rasa4
1	18189	Nrxn1
1	11677	Akr1b3
1	67797	6530403A03Rik
1	207304	Hectd1
1	104570	Smek2
1	66671	Ccnh
1	75712	Tmem14a
1	66254	Dimt1
1	72748	Hdhd3
1	69156	Comtd1
1	18823	Plp1
1	80290	Gpr146
1	17423	Ndst2
1	627427	EG627427
1	NaN	MMT00027196
1	NaN	MMT00000744
1	13358	Slc25a1

1	69539	2300002D11Rik
1	213417	Klhdc8a
1	51886	Fubp1
1	94230	Cpsf1
1	50783	Lsm4
1	74764	Klc4
1	103850	Nt5m
1	18573	Pde1a
1	12282	Hyou1
1	67914	Coq9
1	18030	Nfil3
1	104318	Csnk1d
1	213499	Fbxo42
1	72308	Brf1
1	237221	Gemin8
1	69674	Mif4gd
1	100072	Camta1
1	101502	Hsd3b7
1	114606	Tle6
1	140709	Emid2
1	64138	Ctsz
1	246293	Klhl8
1	14194	Fh1
1	78834	Zfp623
1	230125	Mcart1
1	21807	Tsc22d1
1	110460	Acat2
1	228769	Psmf1
1	56198	Heyl
1	83383	Tcfap4
1	13030	Ctsb
1	227682	Trub2
1	27273	Pdk4
1	68971	1500001M20Rik
1	22341	Vegfc
1	269587	Epb4.1
1	12159	Bmp4
1	27276	Plekhb1
1	72113	Adck1
1	56421	Pfkp
1	19188	Psme2
1	20660	Sorl1
1	NaN	MMT00007996
1	20840	Stac
1	NaN	MMT00001941
1	11674	Aldoa
1	13143	Dapk2
1	16178	Il1r2
1	79554	Gltpd1
1	14226	Fkbp1b
1	13823	Epb4.1l3

1	57317	Sfrs4
1	26570	Slc7a11
1	229707	6330569M22Rik
1	16593	Klc1
1	258692	Olfr1442
1	67826	1110031B06Rik
1	103765	Tmem17
1	73724	Mcee
1	75939	4930579G24Rik
1	12032	Bcan
1	11816	Apoe
1	225372	Apbb3
1	76877	Rab36
1	NaN	MMT00062477
1	232664	Ccdc136
1	67898	Pef1
1	NaN	MMT00032015
1	22215	Ube3a
1	13800	Enah
1	19159	Pscd3
1	16329	Inpp1
1	30058	Timm8a1
1	30058	Timm8a1
1	19141	Lgmn
1	69726	Smyd3
1	73608	Marveld3
1	105278	Ccrk
1	228355	Madd
1	110157	Raf1
1	NaN	MMT00027093
1	16828	Ldha
1	106522	AW548124
1	69742	Tm2d2
1	77031	Slc9a8
1	214952	Rhot2
1	20257	Stmn2
1	20356	Sema5a
1	74316	Isca2
1	380753	Atxn7l1
1	104444	Rexo2
1	235036	Ppan
1	66398	Commd5
1	19185	Psmd4
1	216131	Tmem1
1	19414	Rasa3
1	72155	Cenpn
1	240023	Pnldc1
1	227674	Ddx31
1	72315	2310015A05Rik
1	66236	1500011B03Rik
1	13680	Ddx19a

1	17207	Mcf2l
1	NaN	MMT00018228
1	11803	Aplp1
1	101358	Fbxl14
1	23801	Aloxe3
1	21334	Tac2
1	104836	Cbll1
1	72149	2610019A05Rik
1	20740	Spna2
1	108954	Ppp1r15b
1	218693	Paip1
1	66826	Taz
1	72960	Top1mt
1	20255	Scg3
1	69066	1810010H24Rik
1	68865	Arv1
1	109264	Me3
1	23897	Hax1
1	69635	Dapk1
1	66911	Nudt16l1
1	19655	Rbmx
1	68631	Cryl1
1	19698	Relb
1	78920	Dlst
1	18519	Pcaf
1	NaN	MMT00000525
1	NaN	MMT00047239
1	12226	Btg1
1	66866	Nhlrc2
1	26930	MMT00033859
1	67863	Slc25a11
1	234678	D230025D16Rik
1	55936	Ctps2
1	17191	Mbd2
1	217328	Myo15b
1	52906	Ahi1
1	71957	Cpsf3l
1	26448	Rage
1	75495	1700010A17Rik
1	11983	Atpif1
1	77862	Thyn1
1	27390	Mmel1
1	70122	Mllt3
1	71354	Wdr31
1	18286	Odf2
1	230163	Aldob
1	381693	4930434E21Rik
1	NaN	MMT00058471
1	213753	Zfp598
1	67484	Eepd1
1	625029	Vmn2r83

1	228602	4930402H24Rik
1	NaN	MMT00040762
1	223774	Alg12
1	212516	BC060267
1	20610	Sumo3
1	17898	Myl7
1	24136	Zeb2
1	12070	Ngfrap1
1	11603	Agrn
1	68816	Ppil1
1	70377	Derl3
1	208990	Npb
1	26901	Deb1
1	228889	Ddx27
1	56471	Stmn4
1	21422	Tcfcp2
1	93760	Arid1a
1	15516	Hsp90ab1
1	239528	Eif2c2
1	100040305	MMT00074121
1	100038888	MMT00074572
1	56494	Gosr2
1	66811	Duoxa2
1	60345	Nrip2
1	20044	Rps14
1	66462	2810428I15Rik
1	68565	Mrps18a
1	16525	Kcnk1
1	269999	Orai3
1	331004	Slc9a9
1	68404	Nrn1
1	13048	Cux2
1	16832	Ldhb
1	108897	2810003C17Rik
1	192120	Bspry
1	110596	Rgnef
1	269356	Slc4a11
1	319565	Syne2
1	442801	Arhgef15
1	170833	Hook2
1	NaN	MMT00053383
1	16691	Krt8
1	114893	Dcun1d1
1	75472	1700009P17Rik
1	20732	Spint1
1	20276	Scnn1a
1	68198	Ndufb2
1	217995	Heatr1
1	69770	1600002K03Rik
1	226153	Peo1
1	22278	Usf1

1	72704	2810051F02Rik
1	17202	Mc4r
1	14402	Gabrb3
1	12468	Cct7
1	171508	Creld1
1	16905	Lmna
1	16881	Lig1
1	229615	Pias3
1	80880	Kank3
1	380921	Dgkh
1	12847	Copa
1	26414	Mapk10
1	13498	Atn1
1	21916	Tmod1
1	74013	Rftn2
1	69470	Tmem127
1	71974	Prmt3
1	NaN	MMT00058347
1	70873	4921517L17Rik
1	74246	Gale
1	56812	Dnajb10
1	15184	Hdac5
1	12683	Cidea
1	69663	Ddx51
1	67727	Stx17
1	14083	Ptk2
1	19108	Prkx
1	13169	Dbnl
1	94180	Acsbg1
1	66116	Cml1
1	231125	Zfyve28
1	245038	Dclk3
1	NaN	MMT00023354
1	66867	Hmg20a
1	20720	Serpine2
1	103724	Tbc1d10a
1	52666	D10Ertd610e
1	594844	Tceal3
1	110119	Mpi
1	66104	Tceal6
1	53892	Ppm1d
1	107022	Gramd3
1	78255	Ralgps2
1	20187	Ryk
1	233733	Galntl4
1	230959	Ajap1
1	319352	C530028O21Rik
1	14768	Lancl1
1	319189	Hist2h2bb
1	58805	Mlxipl
1	11814	Apoc3

1	13010	Cst3
1	108124	Napa
1	229877	Rap1gds1
1	100038850	A130082M07Rik
1	66815	Ccdc109b
1	68252	A030007L17Rik
1	226849	Ppp2r5a
1	20515	Slc20a1
1	12040	Bckdhb
1	27220	Cartpt
1	109154	2410014A08Rik
1	210029	Metrnl
1	12977	Csf1
1	215615	Rnpep
1	71665	Fuca1
1	71660	Rarres2
1	102910	Armcx4
1	69161	Manbal
1	66870	Serbp1
1	67770	5830433M19Rik
1	18168	Npy5r
1	106326	Osbpl11
1	20687	Sp3
1	72003	Synpr
1	232146	Tmem166
1	170772	Glcci1
1	66881	Pcyox1
1	16328	Cep250
1	19663	Rbpms
1	22608	Ybx1
1	210762	Gm70
1	227095	Hibch
1	103784	Wdr92
1	107869	Cth
1	50880	Scly
1	12323	Camk2b
1	13629	Eef2
1	30805	Slc22a4
1	20272	Scn7a
1	67035	Dnajb4
1	101214	Tra2a
1	235461	B230380D07Rik
1	14933	Gyk
1	NaN	MMT00054980
1	408062	BC062115
1	13204	Dhx15
1	105559	Mbnl2
1	108829	Jmjd1c
1	73341	Arhgef6
1	225363	Etf1
1	109333	Pkn2

1	252876	Zh2c2
1	14172	Fgf18
1	272322	Arntl2
1	NaN	MMT00025220
1	12751	Tpp1
1	70405	Calml3
1	268709	BC055107
1	72349	Dusp3
1	15983	Ifrd2
1	11669	Aldh2
1	70615	Ankrd24
1	76108	Rap2a
1	21652	Phf1
1	16765	Stmn1
1	16765	Stmn1
1	57266	Cxcl14
1	81898	Sf3b1
1	233865	D430042O09Rik
1	107528	Magee1
1	237979	Sdk2
1	28295	D10Jhu81e
1	28295	D10Jhu81e
1	170758	Rac3
1	18038	Nfkbil1
1	246221	Mpst
1	109299	C330006A16Rik
1	494448	Cbx6
1	83409	MMT00001648
1	18187	Nrp2
1	226562	Bat2d
1	268859	MMT00006152
1	14571	Gpd2
1	226654	EG226654
1	231440	9130213B05Rik
1	18631	Pex11a
1	12295	Cacnb1
1	106200	Txndc11
1	71795	Pitpnc1
1	NaN	MMT00015534
1	22153	Tubb4
1	NaN	MMT00002524
1	NaN	MMT00034355
1	107734	Mrpl30
1	110391	Qdpr
1	11973	Atp6v1e1
1	68576	Hbxip
1	636544	EG636544
1	72722	2810405J04Rik
1	NaN	MMT00014216
1	NaN	MMT00060113
1	19286	Pts

1	NaN	MMT00077446
1	623205	MMT00017441
1	NaN	MMT00021961
1	114893	Dcun1d1
1	NaN	MMT00041633
1	14776	Gpx2
1	NaN	MMT00009124
1	26879	B3galnt1
1	11975	Atp6v0a1
1	192734	AI646023
1	68350	Mul1
1	64085	Clstn2
1	NaN	MMT00017294
1	238836	EG238836
1	NaN	MMT00082708
1	NaN	MMT00075773
1	66143	Eef1e1
1	11908	Atf1
1	16594	Klc2
1	16885	Limk1
1	65098	Zfand6
1	22333	Vdac1
1	20513	Slc1a6
1	66119	Prickle4
1	67458	Ergic1
1	71492	Bbs7
1	211286	Cln5
1	28088	D10Wsu52e
1	NaN	MMT00050438
1	66659	Acp6
1	11949	Atp5c1
1	NaN	MMT00042824
1	NaN	MMT00015325
1	19053	Ppp2cb
1	NaN	MMT00028347
1	NaN	MMT00037377
1	53893	Nudt5
1	628709	ENSMUSG00000071302
1	72085	Osgepl1
1	56717	Frap1
1	329015	Atg2a
1	234797	6430548M08Rik
1	16981	Lrrn3
1	14766	Gpr56
1	NaN	MMT00069758
1	14629	Gclc
1	19299	Abcd3
1	13723	Emb
1	276952	Rasl10b
1	50785	Hs6st1
1	16478	Jund

1	244058	Rgma
1	81840	Sorcs2
1	17772	Mtm1
1	30956	Aass
1	74326	Hnrnpr
1	67010	Rbm7
1	218734	3830406C13Rik
1	76763	Mospd2
1	67043	Syap1
1	74521	8430415E04Rik
1	235380	Dmxl2
1	56258	Hnrnph2
1	12747	Clk1
1	NaN	MMT00076721
1	627908	MMT00030553
1	22359	Vldlr
1	67834	Idh3a
1	52705	Krr1
1	13641	Efnb1
1	94352	Loxl2
1	30806	Adamts8
1	13797	Emx2
1	276905	Armc7
1	629966	EG629966
1	72333	Palld
1	16480	Jup
1	171543	Bmf
1	15442	Hpse
1	13706	RP23-395H4.4
1	NaN	MMT00011721
1	109979	Art3
1	74147	Ehhadh
1	208144	Dhx37
1	28010	D4Wsu114e
1	16562	Kif1c
1	72927	Hepacam
1	20293	Ccl12
1	69219	Ddah1
1	231287	Atp10d
1	13709	Elf1
1	19027	Sypl
1	17961	Nat2
1	20135	Rrm2
1	142980	Tlr3
1	12631	Cfl1
1	11671	Aldh3a2
1	11538	Adnp
1	330260	Pon2
1	623402	EG623402
1	71978	Ppp2r2a
1	214804	Syde2

1	56213	Htra1
1	271144	9330128J19Rik
1	NaN	MMT00014109
1	238455	4732474O15Rik
1	12412	Cbx1
1	20393	Sgk1
1	107503	Atf5
1	72056	1810055G02Rik
1	102323	Dcun1d2
1	620292	Cntnap5c
1	13824	Epb4.1l4a
1	98732	Rab3gap2
1	17357	Marcksl1
1	71760	Agxt2l1
1	20686	Spa17
1	217431	Nol10
1	67680	Sdhb
1	226255	Atrnl1
1	79566	Sh3bp5l
1	66626	5730403B10Rik
1	NaN	MMT00035904
1	226251	Ablim1
1	14813	Grin2c
1	230866	C230096C10Rik
1	217882	AW555464
1	105522	Ankrd28
1	72184	2810406K13Rik
1	17305	Mfng
1	19387	Rangap1
1	234852	Chmp1a
1	93834	Peli2
1	213649	Arhgef19
1	11302	Aatk
1	224024	Scarf2
1	56513	Pard6a
1	12580	Cdkn2c
1	381549	Zfp69
1	56012	Pgam2
1	11810	Apobec1
1	399558	Flrt2
1	100037283	Rnaset2a
1	16161	Il12rb1
1	21943	Tnfsf11
1	225908	Gm98
1	13867	Erbb3
1	19125	Prodh
1	246278	Cd207
1	66260	Tmem54
1	20482	Skil
1	72230	Zfp558
1	68859	1190007F08Rik

1	20210	Saa3
1	77522	D630002J15Rik
1	67077	1700019N12Rik
1	14939	Gzmb
1	56212	Rhog
1	217946	Cdca7l
1	242891	Gm443
1	20728	Spic
1	208144	Dhx37
1	NaN	MMT00035510
1	11808	Apoa4
1	13106	Cyp2e1
1	NaN	MMT00030262
1	13202	Ddt
1	67489	Ap4b1
1	332934	Zmynd12
1	18113	Nnmt
1	22259	Nr1h3
1	14595	B4galt1
1	17240	Mdfi
1	13448	Dok1
1	12051	Bcl3
1	333315	Frem3
1	433759	Hdac1
1	99543	Olfml3
1	11867	Arpc1b
1	83675	Bicc1
1	80876	Ifitm2
1	67512	Agpat2
1	234683	Elmo3
1	17972	Ncf4
1	230787	BC013712
1	56264	Cpxm1
1	277414	Trp53i11
1	216867	Slc16a11
1	117606	Boc
1	17965	Nbl1
1	16440	Itpr3
1	20509	Slc19a1
1	54324	Arhgef5
1	67951	Tubb6
1	16949	Loxl1
1	209837	Slc38a5
1	12516	Cd7
1	68792	Srpx2
1	380780	Serpina11
1	53608	Map3k6
1	207792	BC034090
1	16411	Itgax
1	14710	Gngt2
1	240084	Cchcr1

1	20847	Stat2
1	22409	Wnt10a
1	100689	Spon2
1	22051	Trip6
1	17150	Mfap2
1	72168	Aifm3
1	212032	Hk3
1	330064	Slc5a6
1	70314	Rabep2
1	13036	Ctsh
1	14794	Spsb2
1	17095	Lyl1
1	210622	E430002G05Rik
1	68591	Mocos
1	68318	Aph1c
1	16852	Lgals1
1	232970	Phldb3
1	100038347	BC034069
1	67305	Gpx7
1	230752	2610027C15Rik
1	110253	Triobp
1	98932	Myl9
1	58859	Efemp2
1	11857	Arhgdib
1	16000	Igf1
1	16923	Sh2b3
1	66066	Gng11
1	101540	Prkd2
1	234582	Ccdc102a
1	23887	Ggt5
1	18034	Nfkb2
1	70719	Hmha1
1	51793	Ddah2
1	14337	Ftl2
1	NaN	MMT00066281
1	14325	Ftl1
1	66905	M6prbp1
1	27419	Naglu
1	19253	Ptpn18
1	106512	Gpsm3
1	76257	Slc38a3
1	54123	Irf7
1	231655	Oasl1
1	76854	Gper
1	105450	Mmrn2
1	20342	Selenbp2
1	20342	Selenbp2
1	140859	Nek8
1	52430	Echdc2
1	100163	Pafah2
1	76263	Gstk1

1	14127	Fcer1g
1	76893	Lass2
1	56772	Mllt11
1	73649	Cybrd1
1	67900	1700020C11Rik
1	70456	Brp44
1	192161	Pcdha9
1	12936	Pcdha4
1	116731	Pcdha1
1	192163	Pcdha3
1	12937	Pcdha6
1	12260	C1qb
1	12931	Crlf1
1	52250	Reep1
1	105853	Mal2
1	68031	Rnf146
1	17064	Cd93
1	71973	Rbpms2
1	15936	Ier2
1	74131	Sash3
1	30794	Pdlim4
1	545260	Arsi
1	217344	Rhbdf2
1	140887	Lnx2
1	235441	Usp3
1	30937	Lmcd1
1	98365	Slamf9
1	233064	Wdr62
1	56489	Ikbke
1	286940	Flnb
1	13436	Dnmt3b
1	216739	Acsl6
1	78923	Chsy3
1	50723	Icosl
1	235041	Kank2
1	55951	Brp44l
1	625253	EG625253
1	12298	Cacnb4
1	59030	Mkks
1	104110	Adcy4
1	192188	Stab2
1	20411	Sorbs1
1	NaN	MMT00043009
1	18214	Ddr2
1	106014	AW049604
2	17341	Bhlhb8
2	78928	Pigt
2	21416	Tcf7l2
2	66402	Sln
2	75216	4930534B04Rik
2	382083	Snx22

2	76166	6330545A04Rik
2	80794	Cblc
2	18816	Serpinf2
2	68891	Cd177
2	NaN	MMT00005305
2	269902	Vmn2r57
2	NaN	r60_T1A12
2	218030	Pou6f2
2	18440	P2rx6
2	64297	Gprc5b
2	50883	Chek2
2	NaN	MMT00054100
2	NaN	MMT00068429
2	320398	Lrig3
2	78829	Tsc22d4
2	78076	Lcn8
2	15425	Hoxc6
2	20450	St8sia2
2	12193	Zfp36l2
2	78369	Icam4
2	433809	Rnf207
2	11434	Acr
2	18046	Nfyc
2	18046	Nfyc
2	15497	Hsd3b6
2	228094	Cerkl
2	217734	Pomt2
2	232910	Ap2s1
2	72074	Anks4b
2	72040	Mupcdh
2	76377	2810410L24Rik
2	99439	Duox1
2	13095	Cyp2c29
2	70069	H1fnt
2	78767	2610021K21Rik
2	227231	Cps1
2	14240	Foxb2
2	NaN	MMT00014037
2	286942	Kif19a
2	113854	V1rb4
2	71761	Amdhd1
2	208104	Mlxip
2	74966	4930471G03Rik
2	242939	Cpz
2	19876	Robo1
2	NaN	MMT00035685
2	434484	Sp140
2	74362	Spag17
2	71159	4933416I08Rik
2	108927	Lhfp
2	NaN	MMT00039607

2	76389	Ankrd36
2	229474	Fhdc1
2	240899	Lrrc52
2	219149	Xkr6
2	213989	Tmem82
2	272636	D9Ertd280e
2	NaN	MMT00074939
2	330863	Trim67
2	14936	Gys1
2	108153	Adamts7
2	258979	Olfr1255
2	193034	Trpv1
2	665270	Plb1
2	104252	Cdc42ep2
2	258811	Olfr926
2	NaN	MMT00052724
2	545874	Vmn2r25
2	15233	Hgd
2	73284	Ddit4l
2	319229	Sctr
2	233020	Hipk4
2	12156	Bmp2
2	17380	Mme
2	216445	Arhgap9
2	258589	Olfr703
2	54122	Uevld
2	213234	Zbbx
2	319554	Idi1
2	16364	Irf4
2	NaN	MMT00033197
2	16429	Itln1
2	NaN	MMT00073452
2	NaN	MMT00072579
2	NaN	MMT00009278
2	105787	Prkaa1
2	NaN	MMT00071569
2	209091	Ccnb3
2	77595	Nup210l
2	252837	Ccrl1
2	20190	Ryr1
2	NaN	MMT00039919
2	241547	D230010M03Rik
2	209225	Zfp710
2	320632	Ascc3l1
2	272347	Zfp398
2	56790	D3Ertd300e
2	68709	Cilp2
2	NaN	MMT00014519
2	258552	Olfr868
2	258701	Olfr401
2	72507	Dzip1l

2	432516	Myo1a
2	18545	Pcp2
2	18505	Pax3
2	69785	Ceacam13
2	207495	Baiap2l2
2	546271	MMT00047275
2	217214	Nags
2	NaN	YGL234W_RC-551
2	22346	Vhlh
2	108934	BC024659
2	53761	Bat2
2	100040304	MMT00028325
2	18985	Pou2af1
2	114662	Prss29
2	100855	Tbc1d14
2	79401	Spz1
2	NaN	MMT00017417
2	242286	4833413O15Rik
2	NaN	MMT00008170
2	NaN	MMT00032221
2	107376	E330013P04Rik
2	268465	Eme1
2	NaN	MMT00039397
2	23917	Impdh1
2	83997	Slmap
2	74276	Cldnd2
2	546036	EG546036
2	213988	Tnrc6b
2	73532	1700080E11Rik
2	58994	Smpd3
2	235858	Gm392
2	20669	Sox14
2	241769	Kcnk15
2	74092	0610038L08Rik
2	242653	Cldn19
2	57255	Cldn13
2	30051	Spdef
2	14773	Grk5
2	434439	BC048562
2	216781	Trim58
2	404195	Cyp2c54
2	244349	Myst3
2	18799	Plcd1
2	NaN	MMT00045847
2	98710	Rabif
2	NaN	MMT00000356
2	259081	Olfr643
2	210356	E030049G20Rik
2	67608	Narf
2	NaN	MMT00016640
2	207209	Gm317

2	16508	Kcnd2
2	23872	Ets2
2	110809	Sfrs1
2	237954	Lrrc37a
2	235952	Gm189
2	193385	6330500D04Rik
2	320685	Dctd
2	223726	Mpped1
2	14764	Gpr44
2	207393	Elfn2
2	319565	Syne2
2	628893	EG628893
2	58214	Cst10
2	227326	Gpr55
2	72729	Cdc42se2
2	20865	C730007P19Rik
2	223337	Ugt3a2
2	12839	Col9a1
2	NaN	MMT00015245
2	21354	Tap1
2	14312	Brd2
2	258960	Olfr171
2	NaN	MMT00047280
2	30944	Zfp354c
2	NaN	MMT00065977
2	258908	Olfr853
2	16196	Il7
2	94224	Srd5a2
2	114230	Aipl1
2	NaN	MMT00007396
2	408059	BC049352
2	NaN	MMT00006341
2	NaN	MMT00033542
2	18861	Pms2
2	241075	Plekhm3
2	18191	Nrxn3
2	67554	Slc25a30
2	217305	4732429D16Rik
2	54219	Cd320
2	70103	Znhit1
2	50917	Galns
2	58235	Pvrl1
2	22364	Vpreb3
2	71093	Atoh8
2	66257	Nicn1
2	NaN	MMT00043344
2	94191	Adarb2
2	671564	Rnf212
2	54126	Arhgef7
2	434437	Amt
2	68243	A930018P22Rik

2	245638	Tbc1d8b
2	332175	Zdhhc23
2	258952	Olfr341
2	26377	Dapp1
2	195018	Zzef1
2	16162	Il12rb2
2	76610	1700063K16Rik
2	56324	Stam2
2	15586	Hyal1
2	66816	Thap2
2	328234	Rnf182
2	19089	Prkcsh
2	56643	Slc15a1
2	14388	Gab1
2	50786	Hs6st2
2	207259	Zbtb7c
2	109294	Depdc2
2	NaN	MMT00015975
2	NaN	MMT00046108
2	72027	Slc39a4
2	20465	Sim2
2	56305	Pitpnb
2	68265	Iqcf3
2	68585	Rtn4
2	231699	Oas1e
2	75560	Ep400
2	320549	D5Ertd577e
2	17057	Klrb1a
2	75690	2210412E05Rik
2	13370	Dio1
2	75301	4930556N09Rik
2	NaN	MMT00077700
2	14179	Fgf8
2	21817	Tgm2
2	434203	Slc28a1
2	105785	Kdelr3
2	22229	Ucp3
2	380660	Acss3
2	68680	1110028A07Rik
2	16728	L1cam
2	12583	Cdo1
2	NaN	MMT00030026
2	108899	2700081O15Rik
2	74335	Xrcc3
2	18023	Nfe2l1
2	13043	Cttn
2	19152	Prtn3
2	258645	Olfr1403
2	319670	Eml5
2	106059	A430088P11Rik
2	NaN	MMT00005283

2	105727	Slc38a1
2	NaN	MMT00019402
2	72948	Tppp
2	113847	V1ra5
2	171241	V1rg6
2	NaN	MMT00014972
2	619301	G630016D24Rik
2	NaN	MMT00031309
2	19281	Ptprt
2	109697	Cpa1
2	238988	Erc2
2	76669	1700128F08Rik
2	21388	Tbx5
2	110058	Syt17
2	NaN	MMT00005098
2	319845	Bbs9
2	66797	Cntnap2
2	67373	2210010C04Rik
2	69864	1810065E05Rik
2	71452	Ankrd40
2	NaN	MMT00029554
2	NaN	YAL043C_RC-451
2	22040	Trex1
2	50701	Ela2
2	26556	Homer1
2	11548	Adra1b
2	NaN	MMT00033987
2	258743	Olfr661
2	14824	Grn
2	105827	Amigo2
2	NaN	MMT00031206
2	258645	Olfr1403
2	170574	Sp7
2	72007	Fndc3b
2	78304	Lsmd1
2	NaN	MMT00009628
2	NaN	MMT00043953
2	17354	Mllt10
2	231207	Cpeb2
2	381373	Sp9
2	215493	A3galt2
2	333050	Ksr2
2	52897	D11Bwg0517e
2	233765	Plekha7
2	12300	Cacng2
2	545474	Scrt2
2	12895	Cpt1b
2	54420	Cldn8
2	17167	Marco
2	19303	Pxn
2	14998	H2-DMa

2	11443	Chrnb1
2	83813	Tnk1
2	12350	Car3
2	214305	Hhipl1
2	13385	Dlg4
2	330830	Ccdc135
2	21959	Tnp2
2	14309	Fshr
2	71544	9030420J04Rik
2	19820	Rnf12
2	98952	C230093N12Rik
2	79455	Pdcl2
2	83396	Glis2
2	66237	Atp6v1g2
2	12142	Prdm1
2	11944	Atp4a
2	16449	Jag1
2	52428	Rhpn2
2	71781	Slc16a14
2	NaN	MMT00058440
2	258288	Olfr1484
2	NaN	MMT00048659
2	140499	Ube2j2
2	673094	Cd99
2	258954	Olfr522
2	252904	V1re9
2	280635	Emilin3
2	74094	Tjap1
2	93961	B3galt5
2	NaN	MMT00016293
2	26362	Axl
2	171267	V1rg11
2	50873	Park2
2	67645	4930511I11Rik
2	16529	Kcnk5
2	64453	Zfp280b
2	12915	Crebl1
2	NaN	MMT00027552
2	16596	Klf1
2	12351	Car4
2	116904	Alpk3
2	258790	Olfr1263
2	209497	Tmem164
2	18439	P2rx7
2	15567	Slc6a4
2	68957	Paqr6
2	214239	A430105I19Rik
2	320404	Itpkb
2	67576	4930429B21Rik
2	NaN	MMT00015104
2	258752	Olfr583

2	13838	Epha4
2	NaN	MMT00027295
2	NaN	MMT00078109
2	242594	1700024P16Rik
2	245615	Kir3dl2
2	14029	Evx2
2	NaN	MMT00014480
2	259108	Olfr550
2	29809	Rabgap1l
2	258918	Olfr1346
2	259003	Olfr172
2	76788	2410127E18Rik
2	631304	Cyp4f40
2	53878	Svs2
2	259091	Olfr585
2	56734	Tulp2
2	74221	1700011F03Rik
2	22403	Wisp2
2	545817	Cyp2w1
2	22390	Wee1
2	107141	Cyp2c50
2	329858	MMT00069147
3	103511	BB146404
3	12818	Col14a1
3	20296	Ccl2
3	208777	Sned1
3	11536	Gpr182
3	18596	Pdgfrb
3	15507	Hspb1
3	15507	Hspb1
3	21857	Timp1
3	21788	Tfpi
3	11745	Anxa3
3	13078	Cyp1b1
3	11668	Aldh1a1
3	624910	Rgs21
3	22361	Vnn1
3	16591	Kl
3	21859	Timp3
3	11746	Anxa4
3	17022	Lum
3	16948	Lox
3	216616	Efemp1
3	216350	Tspan8
3	208177	Phldb2
3	11754	Aoc3
3	12550	Cdh1
3	22160	Twist1
3	76453	Prss23
3	69068	1810011O10Rik
3	56744	Pf4

3	64074	Smoc2
3	13482	Dpp4
3	17533	Mrc1
3	76933	Ifi27
3	15903	Id3
3	29876	Clic4
3	13132	Dab2
3	11815	Apod
3	59012	Moxd1
3	22370	Vtn
3	19128	Pros1
3	26357	Abcg2
3	65970	Lima1
3	21898	Tlr4
3	13136	Cd55
3	18783	Pla2g4a
3	73656	Ms4a6c
3	73671	Sult6b1
3	209086	Samd9l
3	57814	Kcne4
3	21682	Tec
3	20194	S100a10
3	68303	9130005N14Rik
3	19264	Ptprc
3	22402	Wisp1
3	330256	MMT00008885
3	106628	Trip10
3	107350	AW112010
3	64075	Smoc1
3	65256	Asb2
3	15170	Ptpn6
3	14960	H2-Aa
3	12798	Cnn2
3	12724	Clcn2
3	57349	Ppbp
3	207742	Rnf43
3	16612	Klk1
3	11937	Atp2a1
3	14190	Fgl2
3	20514	Slc1a5
3	71345	Tmem16j
3	19218	Ptger3
3	12797	Cnn1
3	328779	Hs3st6
3	85031	Pla1a
3	71566	9030425E11Rik
3	69540	Klk10
3	17873	Gadd45b
3	18858	Pmp22
3	12263	C2
3	69291	1700001L05Rik

3	64292	Ptges
3	12835	Col6a3
3	16011	Igfbp5
3	227753	Gsn
3	16362	Irf1
3	67103	Ltb4dh
3	13179	Dcn
3	320712	Abi3bp
3	227612	A830007P12Rik
3	20347	Sema3b
3	71724	Aox3
3	102371	AA407270
3	208836	Fanci
3	19662	Rbp4
3	20306	Ccl7
3	14181	Fgfbp1
3	27047	Omd
3	56312	Nupr1
3	12642	Ch25h
3	11694	Alx3
3	241327	Olfml2a
3	11889	Asgr1
3	14114	Fbln1
3	71683	Gypc
3	14219	Ctgf
3	21824	Thbd
3	96875	Prg4
3	22268	Upk1b
3	16425	Itih2
3	20887	Sult1a1
3	17300	Foxc1
3	21814	Tgfbr3
3	11535	Adm
3	78896	1500015O10Rik
3	242509	Bnc2
3	104086	Cyp27a1
3	78892	Crispld2
3	74511	Lrrc17
3	12817	Col13a1
3	75750	Slc10a6
3	103149	Upb1
3	67082	1700011H14Rik
3	12825	Col3a1
3	14187	Akr1b8
3	23876	Fbln5
3	17312	Mgl1
3	12560	Cdh3
3	14130	Fcgr2b
3	231162	Cytl1
3	16008	Igfbp2
3	434198	B130024G19Rik

3	66898	Baiap2l1
3	380712	2010305C02Rik
3	109323	C1qtnf7
3	67856	Echdc3
3	21826	Thbs2
3	19331	Rab19
3	64058	Perp
3	71914	Antxr2
3	22411	Wnt11
3	18214	Ddr2
3	72565	Uaca
3	13094	Cyp2b9
3	71934	Car13
3	77037	Mrap
3	15160	Serpind1
3	93689	Lmod1
3	17313	Mgp
3	76365	Tbx18
3	103161	Apof
3	20518	Slc22a2
3	18399	Slc22a6
3	14412	Slc6a13
3	16012	Igfbp6
3	22420	Wnt6
3	12904	Crabp2
3	20204	Prrx2
3	16002	Igf2
3	14234	Foxc2
3	14268	Fn1
3	14049	Eya2
3	18400	Slc22a18
3	67473	Slc47a1
3	226999	Slc9a2
3	21384	Tbx15
3	223272	Itgbl1
3	18295	Ogn
3	102680	Slc6a20a
3	67606	1110018M03Rik
3	19038	Ppic
3	12831	Col5a1
3	104816	A530050D06Rik
3	NaN	MMT00019254
3	64817	Svep1
3	67182	Pdzk1ip1
3	76905	Lrg1
3	53620	Vamp5
3	21922	Clec3b
3	68713	Ifitm1
3	211577	Mrgprf
3	12258	Serping1
3	14411	Slc6a12

3	13732	Emp3
3	244550	Podnl1
3	20698	Sphk1
3	NaN	MMT00077820
3	76747	Dapl1
3	19215	Ptgds
3	269633	Wdr86
3	70355	Gprc5c
3	23967	Osr1
3	69553	2310033K02Rik
3	243967	Gm484
3	14264	Fmod
3	70370	Fbln7
3	23920	Insrr
3	216725	Adamts2
3	12843	Col1a2
3	74127	Krt80
3	68794	Flnc
3	19659	Rbp1
3	18542	Pcolce
3	11601	Angpt2
3	12870	Cp
3	69774	Ms4a6b
3	12364	Casp12
3	330723	Htra4
3	16773	Lama2
3	209039	Tenc1
3	16790	Anpep
3	11568	Aebp1
3	26968	Islr
3	21401	Tcea3
3	93671	Cd163
3	68774	Ms4a6d
3	20684	Sp100
3	11761	Aox1
3	246728	Oas2
3	14744	Gpr65
3	27356	Insl6
3	15959	Ifit3
3	629974	Rnf213
3	15953	Ifi47
3	72575	C430049B03Rik
3	NaN	MMT00072312
3	20324	Sdpr
3	66824	Pycard
3	12984	Csf2rb2
3	63986	Gmfg
3	74145	F13a1
3	21822	Tgtp
3	547253	Parp14
3	66775	Ptplad2

3	56644	Clec7a
3	19073	Srgn
3	66857	1100001H23Rik
3	15951	Ifi204
3	15945	Cxcl10
3	226421	5430435G22Rik
3	226041	Pgm5
3	16408	Itgal
3	19220	Ptgfr
3	268977	Ltbp1
3	17355	Aff1
3	18605	Enpp1
3	69590	2310016C16Rik
3	52377	Rcn3
3	20499	Slc12a7
3	219151	Scara3
3	229488	9930021J17Rik
3	12842	Col1a1
3	232313	Glt8d4
3	319480	Itga11
3	12834	Col6a2
3	68311	Lypd2
3	74761	Mxra8
3	21928	Tnfaip2
3	20409	Ostf1
3	67247	Mosc2
3	246154	Vasn
3	14238	Foxf2
3	17886	Myh9
3	69698	2310046K01Rik
3	14560	Gdf10
3	329941	Col8a2
3	76281	Tax1bp3
3	114774	Pawr
3	225115	Svil
3	67760	Slc38a2
3	16431	Itm2a
3	14469	Gbp2
3	17476	Mpeg1
4	NaN	MMT00037010
4	224661	Slc26a8
4	NaN	MMT00030322
4	640530	EG640530
4	26450	Rbbp9
4	13124	Cyp8b1
4	108655	Foxp1
4	NaN	MMT00050325
4	22301	Vmn2r89
4	69357	RP23-438H3.2
4	66289	1810030J14Rik
4	627984	Nlrp1c

4	111507	Igh
4	629065	EG629065
4	27395	Mrpl15
4	268780	Egflam
4	229665	Ampd1
4	107221	Gpr120
4	20271	Scn5a
4	NaN	MMT00036566
4	75863	Clec4g
4	71623	Krtap5-2
4	109331	Rnf20
4	238076	Kcns3
4	17878	Myf6
4	67555	4933434I20Rik
4	77220	C030003D03Rik
4	16184	Il2ra
4	19226	Pth
4	12322	Camk2a
4	621976	EG621976
4	547097	B020011L13Rik
4	227627	Lcn13
4	114714	Rad51c
4	237038	Nox1
4	72318	Pscd4
4	13525	Adam26a
4	21897	Tlr1
4	NaN	MMT00050153
4	241638	RP23-100C5.8
4	16846	Lep
4	237911	Brip1
4	12267	C3ar1
4	77771	Csrnp3
4	105349	Akr1c18
4	18343	Olfr44
4	234736	Rfwd3
4	NaN	MMT00048531
4	66996	Ceacam11
4	20445	St6galnac1
4	NaN	MMT00080431
4	NaN	MMT00012984
4	22035	Tnfsf10
4	68054	Serpina12
4	76816	Sdccag8
4	NaN	MMT00071383
4	57915	Tbc1d1
4	232426	5530400C23Rik
4	NaN	MMT00051225
4	259019	Olfr1045
4	399603	D330050I23Rik
4	17230	Tpsab1
4	75216	4930534B04Rik

4	445007	Nup85
4	77531	Anks1b
4	268980	Strn
4	NaN	MMT00029895
4	258186	Olfr75-ps1
4	11867	Arpc1b
4	75459	1700007E06Rik
4	NaN	MMT00070628
4	20459	Ptk6
4	243725	Ppp1r9a
4	259015	Olfr1038
4	382864	Colq
4	258290	Olfr1143
4	66255	1810005K13Rik
4	67477	1300007F04Rik
4	236149	BC014805
4	100040020	MMT00018931
4	12873	Cpa3
4	53973	Cyp3a41a
4	14942	Gzme
4	94094	Trim34
4	629203	EG629203
4	72151	Rfc5
4	15493	Hsd3b2
4	27364	Srr
4	214058	Megf11
4	22138	Ttn
4	52614	Emr4
4	57254	Tas2r119
4	NaN	MMT00076063
4	107587	Osr2
4	117586	A1bg
4	330662	Dock1
4	242248	Bank1
4	NaN	MMT00046813
4	NaN	MMT00013165
4	NaN	MMT00007654
4	234564	AU018778
4	12352	Car5a
4	NaN	MMT00059108
4	71421	Amtn
4	NaN	MMT00020896
4	259017	Olfr1019
4	NaN	MMT00077775
4	22227	Ucp1
4	240894	Fmo9
4	NaN	MMT00014316
4	21816	Tgm1
4	NaN	MMT00002308
4	381538	Gm1027
4	114660	Etos1

4	233799	Acsm2
4	NaN	MMT00020226
4	13587	Ear2
4	16182	Il18r1
4	21345	Tagln
4	21667	Tdgf1
4	101835	AW146154
4	235132	Zbtb44
4	NaN	MMT00004378
4	72789	Veph1
4	20234	Sbp
4	74441	Slco6c1
4	NaN	MMT00063621
4	20749	Dbpht1
4	384422	Igk-V8
4	258808	Olfr620
4	12945	Dmbt1
4	NaN	MMT00078580
4	102093	Phkb
4	NaN	MMT00054498
4	109151	Chd9
4	76386	1700010D01Rik
4	71162	4933421I07Rik
4	258934	Olfr802
4	54354	Rassf5
4	NaN	MMT00008599
4	NaN	MMT00054728
4	12401	Serpina6
4	56636	Fgf21
4	627081	Xlr5b
4	16061	Igh-VJ558
4	330228	4933411G11Rik
4	13099	Cyp2c40
4	70900	4921517D22Rik
4	217082	Hlf
4	213603	Slc44a3
4	14028	Evx1
4	NaN	MMT00038655
4	111507	Igh
4	170639	Olfr78
4	380822	MMT00072212
4	67715	2010106E10Rik
4	NaN	MMT00025388
4	170716	Cyp4f13
4	385493	AU015836
4	57252	Tas2r105
4	259000	Olfr195
4	224480	Nox3
4	112417	Ugt2b37
4	17395	Mmp9
4	16061	Igh-VJ558

4	12962	Crybb3
4	22419	Wnt5b
4	432649	EG432649
4	69611	Lce1d
4	80861	Dhx58
4	625360	MMT00062748
4	239618	Pdzrn4
4	278180	Vsig4
4	NaN	MMT00000773
4	100669	9930105H17Rik
4	20397	Sgpl1
4	241062	Pgap1
4	632654	MMT00048312
4	50780	Rgs3
4	20199	S100a5
4	103406	9130206N08Rik
4	243171	BC005561
4	258883	Olfr876
4	94176	Dock2
4	NaN	MMT00039783
4	21418	Tcfap2a
4	78128	Spag11
4	320495	A130090K04Rik
4	58185	Rsad2
4	14528	Gch1
4	258843	Olfr1087
4	229780	Ccdc76
4	12353	Car6
4	64697	Keg1
4	77011	5730590G19Rik
4	NaN	MMT00078938
4	NaN	MMT00072808
4	NaN	MMT00067115
4	73747	1110034G24Rik
4	75552	Paqr9
4	330660	Btbd16
4	319158	Hist1h4i
4	26877	B3galt1
4	67932	1700129C05Rik
4	NaN	MMT00003854
4	381065	EG381065
4	233490	Crebzf
4	NaN	MMT00020852
4	69454	Clic3
4	100040843	Cyp4a32
4	259277	Klk8
4	666048	OTTMUSG00000008561
4	630663	MMT00057236
4	73360	Actrt1
4	83703	Dbr1
4	70365	Speer5-ps1

4	665503	EG665503
4	252838	Tox
4	NaN	MMT00072251
4	NaN	MMT00062390
4	20504	Slc17a1
4	NaN	MMT00012296
4	71361	Aifm2
4	NaN	MMT00023461
4	73332	1700041C02Rik
4	213450	Gm732
4	73634	1700125H20Rik
4	99663	Clca6
4	21973	Top2a
4	NaN	MMT00060741
4	20297	Ccl20
4	67052	Ndc80
4	22072	Prss2
4	271639	Adcy10
4	66338	Cdrt4
4	NaN	MMT00067195
4	108907	Nusap1
4	NaN	MMT00030613
4	330577	1700129I04Rik
4	74413	Tc2n
4	631323	MMT00028135
4	241452	Dhrs9
4	11449	Chrng
4	258299	Olfr1461
4	NaN	MMT00005811
4	21419	Tcfap2b
4	NaN	MMT00082318
4	258216	Olfr1034
4	241391	Galnt5
4	15430	Hoxd10
4	NaN	MMT00031273
4	26437	Psg17
4	52635	D12Ertd551e
4	66786	Olfr701
4	74365	Lonrf3
4	433597	MMT00013465
4	NaN	MMT00001961
4	54601	Foxo4
4	NaN	MMT00061154
4	72462	Rrp1b
4	258728	Olfr482
4	NaN	MMT00054267
4	23794	Adamts5
4	11571	Crisp1
4	19111	Prl6a1
4	258905	Olfr871
4	12046	Bcl2a1c

4	78631	1700085A12Rik
4	67218	2810038D20Rik
4	56356	Gltp
4	NaN	MMT00060807
4	67909	Galntl5
4	12774	Ccr5
4	17836	Mug1
4	230676	BC059842
4	13109	Cyp2j5
4	75050	Kif27
[truncated: 502,571 more chars]
